# Plant-mPLoc: A Top-Down Strategy to Augment the Power for Predicting Plant Protein Subcellular Localization

**DOI:** 10.1371/journal.pone.0011335

**Published:** 2010-06-28

**Authors:** Kuo-Chen Chou, Hong-Bin Shen

**Affiliations:** 1 Institute of Image Processing & Pattern Recognition, Shanghai Jiaotong University, Shanghai, China; 2 Gordon Life Science Institute, San Diego, California, United States of America; University of Melbourne, Australia

## Abstract

One of the fundamental goals in proteomics and cell biology is to identify the functions of proteins in various cellular organelles and pathways. Information of subcellular locations of proteins can provide useful insights for revealing their functions and understanding how they interact with each other in cellular network systems. Most of the existing methods in predicting plant protein subcellular localization can only cover three or four location sites, and none of them can be used to deal with multiplex plant proteins that can simultaneously exist at two, or move between, two or more different location sites. Actually, such multiplex proteins might have special biological functions worthy of particular notice. The present study was devoted to improve the existing plant protein subcellular location predictors from the aforementioned two aspects. A new predictor called “Plant-mPLoc” is developed by integrating the gene ontology information, functional domain information, and sequential evolutionary information through three different modes of pseudo amino acid composition. It can be used to identify plant proteins among the following 12 location sites: (1) cell membrane, (2) cell wall, (3) chloroplast, (4) cytoplasm, (5) endoplasmic reticulum, (6) extracellular, (7) Golgi apparatus, (8) mitochondrion, (9) nucleus, (10) peroxisome, (11) plastid, and (12) vacuole. Compared with the existing methods for predicting plant protein subcellular localization, the new predictor is much more powerful and flexible. Particularly, it also has the capacity to deal with multiple-location proteins, which is beyond the reach of any existing predictors specialized for identifying plant protein subcellular localization. As a user-friendly web-server, Plant-mPLoc is freely accessible at http://www.csbio.sjtu.edu.cn/bioinf/plant-multi/. Moreover, for the convenience of the vast majority of experimental scientists, a step-by-step guide is provided on how to use the web-server to get the desired results. It is anticipated that the Plant-mPLoc predictor as presented in this paper will become a very useful tool in plant science as well as all the relevant areas.

## Introduction

Information of the subcellular localization of proteins is important because it can **(1)** indicate how and under what kind of cellular environments they interact with each other and with other molecules, **(2)** provide useful clues for revealing their functions, and **(3)** help understand the intricate pathways that regulate biological processes at the cellular level [1], [2]. Although this kind of information can be acquired by conducting various biochemical experiments, it is both time consuming and expensive to determine the subcellular localization of uncharacterized proteins one by one with experiments alone. With the avalanche of protein sequences generated in the Post-Genomic Age, it is highly desired to develop computational methods that can be used to identify the subcellular location site(s) of a newly found protein based on its sequence information alone.

During the past 17 years or so, numerous efforts have been made in this regard (see, e.g., [3], [4], [5], [6], [7], [8], [9], [10] as well as a long list of references cited in two comprehensive review articles [11], [12]). However, relatively much fewer predictors were developed specialized for predicting the subcellular localization of plant proteins. To the best of our knowledge, of the aforementioned methods only the one called “TargetP” [6] and the one called “Predotar” [8] are specialized for plant proteins. Ever since the two predictors were proposed, they have been widely used for studying various plant protein systems and related areas. However, TargetP and Predotar can discriminate plant proteins among only three or four location sites. For instance, TargetP [6] only covers the following sites: **(1)** mitochondria, **(2)** chloroplast, **(3)** secretory pathway, and **(4)** other. And Predotar [8] only covers the following sites: **(1)** endoplasmic reticulum, **(2)** mitochondrion, **(3)** plastid, and **(4)** other. After removing the ambiguous location of “other”, TargetP or Predotar actually covers only three subcellular location sites. If a user tried to use TargetP and Predotar to predict a query protein located outside the aforementioned sites, such as cell wall, peroxisome, Golgi apparatus, or vacuole, the two predictors would either fail to work or generate meaningless outcomes.

To improve the situation, the predictor called “Plant-PLoc” [13] was developed to extend the coverage scope for plant proteins from the three locations covered by TargetP or Predotar to the following eleven: **(1)** cell wall, **(2)** chloroplast, **(3)** cytoplasm, **(4)** endoplasmic reticulum, **(5)** extracellular, **(6)** mitochondrion, **(7)** nucleus, (**8**) peroxisome, (**9**) plasma membrane, (**10**) plastid, and (**11**) vacuole. The Plant-PLoc predictor was established by integrating the “higher-level” GO (gene ontology) [14] approach and PseAAC (pseudo amino acid composition) [15] approach. GO is a controlled vocabulary used to describe the biology of a gene product in any organism [16], [17]. The GO database was established based on the molecular function, biological process and cellular component [14], and hence proteins formulated in the GO database space would be clustered in a way much better reflecting their subcellular locations, as elucidated in [18]. For those proteins that cannot be meaningfully defined in the GO space, the PseAAC descriptor [15] would play a better complementary role than the classical AAC (amino acid composition) descriptor.

However, the existing Plant-PLoc [13] predictor has the following problems. **(1)** The accession number of a query protein is required as an input in order to utilize the advantage of GO approach. Many proteins, such as synthetic or hypothetical proteins, and newly discovered sequences without being deposited into databanks yet, do not have accession numbers, and hence cannot be treated with the GO approach. **(2)** Even with the accession numbers available, many proteins can still not be meaningfully formulated in a GO space because the current GO database is far from complete yet. **(3)** Although the PseAAC approach, a complementary approach to the GO approach in Plant-PLoc [13], can take into account some partial sequence order effects, the original PseAAC [15] did not contain the functional domain and sequential evolution informations, which have been proved to play an important role in enhancing the prediction quality of other protein attributes (see, e.g., [19], [20]). **(4)** Plant-PLoc [13] cannot be used to deal with multiplex proteins that may simultaneously exist at, or move between, two or more different subcellular locations. Proteins with multiple locations or dynamic feature of this kind are particularly interesting because they may have some very special biological functions intriguing to investigators in both basic research and drug discovery [2], [21]. Particularly, as pointed out by Millar et al. [22], recent evidence indicates that an increasing number of proteins have multiple locations in the cell.

The present study was initiated in an attempt to develop a new and more powerful predictor for predicting plant protein subcellular localization by addressing the above four problems.

## Materials and Methods

Protein sequences were collected from the Swiss-Prot database at http://www.ebi.ac.uk/swissprot/. The detailed procedures are basically the same as those elaborated in [13]; the only differences are as follows. **(1)** To get the updated benchmark dataset, instead of version 49.3 of the Swiss-Prot database, the version 55.3 released on 29-Apr-2008 was adopted. **(2)** In order to make the new predictor also able to deal with proteins having two or more location sites, the multiplex proteins are no longer excluded in this study. Actually, according to a statistical analysis on the current database, about 8% of plant proteins were found located in more than one location.

**Figure 1:**
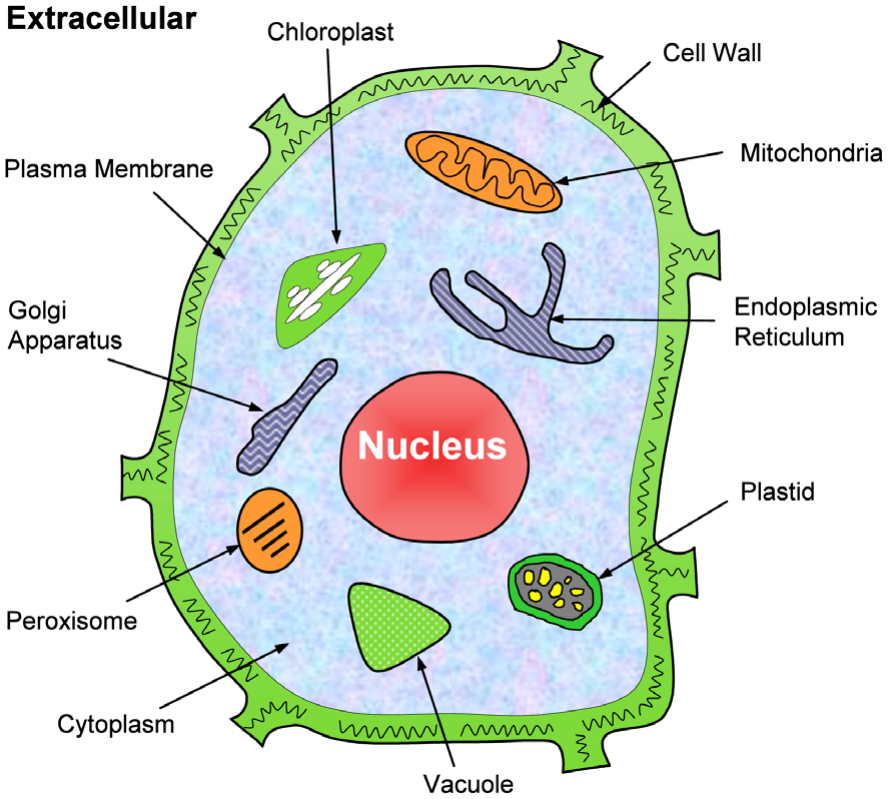
Schematic illustration to show the 12 subcellular locations of plant proteins. The 12 location sites are: (1) cell membrane, (2) cell wall, (3) chloroplast, (4) cytoplasm, (5) endoplasmic reticulum, (6) extracellular, (7) Golgi apparatus, (8) mitochondrion, (9) nucleus, (10) peroxisome, (11) plastid, and (12) vacuole.

After strictly following the aforementioned procedures, we finally obtained a benchmark dataset 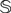 containing 978 different protein sequences, which are distributed among 12 subcellular locations (**Fig. 1**); i.e.,
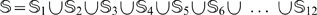
(1)where 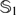 represents the subset for the subcellular location of cell membrane, 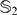 for cell wall, 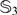 for chloroplast, and so forth; while 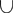 represents the symbol for “union” in the set theory. A breakdown of the 978 plant proteins in the benchmark dataset 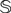 according to their 12 location sites is given in **Table 1**. To avoid redundancy and homology bias, none of the proteins in 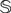 has 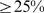 pairwise sequence identity to any other in a same subset. The corresponding accession numbers and protein sequences are given in **Table S1**.

**Table 1:** Breakdown of the plant protein benchmark dataset 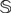 derived from Swiss-Prot database (release 55.3) according to the procedures described in the Materials section.

Subset	Subcellular location^a^	Number of proteins
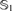	Cell membrane	56
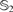	Cell wall	32
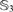	Chloroplast	286
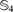	Cytoplasm	182
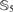	Endoplasmic reticulum	42
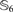	Extracellular	22
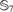	Golgi apparatus	21
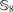	Mitochondrion	150
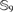	Nucleus	152
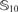	Peroxisome	21
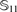	Plastid	39
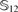	Vacuole	52
Total number of locative proteins 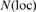		1,055^b^
Total number of different proteins 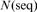		978^c^

None of proteins included here has 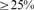 sequence identity to any other in a same subcellular location.

a The benchmark dataset 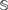 here covers 12 plant subcellular locations and the “Golgi apparatus” is newly added in comparison with the dataset in [13] that covered 11 location sites.

b See Eqs.2–3 for the definition about the number of locative proteins, and its relation with the number of different proteins.

c Of the 978 different proteins, 904 have one subcellular location, 71 have two locations, 3 have three locations, and none have four or more locations.

Since some proteins in 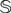 may occur in two or more locations, it is instructive to introduce the concept of “locative protein” [23], as briefed as follows. A protein coexisting at two different location sites will be counted as 2 locative proteins even though the two are with completely the same sequence; if coexisting at three sites, 3 locative proteins; and so forth. Thus, it follows
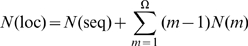
(2)where 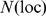 is the number of total locative proteins, 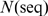 the number of total different protein sequences, 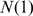 the number of proteins with one location, 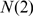 the number of proteins with two locations, and so forth; while 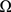 is the number of total subcellular location sites concerned (for the current case, 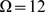 as shown in **Fig. 1**).

For the current 978 different protein sequences, 904 occur in one subcellular location, 71 in two locations, 3 in three locations, and none in four or more locations. Substituting these data into Eq.2, we have
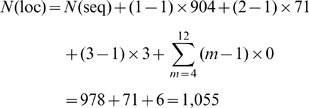
(3)which is fully consistent with the figures in **Table 1** and the data in **Table S1**.

To develop a powerful method for predicting protein subcellular localization, it is very important to formulate the sample of a protein in terms of the core features that are intrinsically correlated with its localization in a cell. To realize this, the strategy by integrating the GO representation and PseAAC representation was adopted in the original Plant-PLoc [13]. In this study, the essence of such a strategy will be still kept. However, in order to overcome the four shortcomings as mentioned in Introduction for Plant-PLoc [13], a completely different combination approach has been developed, as described below.

### 1. Gene Ontology Descriptor

The gene ontology (GO) representation for a protein sample in the original Plant-PLoc [13] was derived through its accession number from the GO database [16]. Therefore, in using Plant-PLoc to conduct prediction, the accession number of a query protein would be indispensable as a part of input. To avoid such a requirement, the following different procedures are proposed to derive the GO representation.

#### Step 1

Use BLAST [24] to search the homologous proteins of the query protein 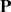 from the Swiss-Prot database (version 55.3), with the BLAST parameter of expect value 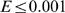.

#### Step 2

Those proteins that have 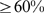 pairwise sequence identity with the query protein 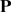 are collected into a set, 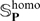, called the “homology set” of 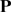. All the elements in 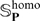 can be deemed as the *representative proteins* of 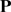. Because these representative proteins were retrieved from the Swiss-Prot database, they must each have their own accession numbers.

#### Step 3

Search each of these accession numbers collected in Step 2 against the GO database at http://www.ebi.ac.uk/GOA/ to find the corresponding GO numbers [16].

#### Step 4

The current GO database (version 70.0 released 10 March 2008) contains 60,020 GO numbers, thus the query protein 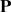 can be formulated through its representative proteins in 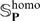 by the following equation
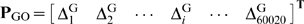
(4)where 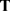 is the transposing operator, and
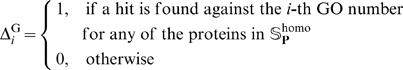
(5)


Through the above steps, we can use Eq.4 derived from the representative proteins in 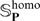 to investigate the query protein 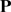. The rationale of such a practice is based on the fact that homology proteins generally share similar attributes, such as folding patterns [25] and biological functions [26], [27], [28]. Thus, the accession number is no longer needed for the input of the query protein even when using the high-level GO approach to predict its subcellular localization as required in the old Plant-PLoc [13].

The above homology-based GO extraction method is particularly useful for studying those proteins which do not have UniProt accession numbers. However, it would still fail to work under any of the following situations: **(1)** the query protein does not have significant homology to any protein in the Swiss-Prot database, i.e., 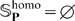 meaning the homology set is an empty one; **(2)** its representative proteins do not contain any useful information for statistical prediction based on a given training dataset.

Therefore, it is necessary to consider the following representations for those proteins that fail to be meaningfully defined in the GO space.

### 2. Functional Domain Descriptor

The functional domain (FunD) is the core of a protein. Therefore, in determining the 3-D (dimensional) structure of a protein by experiments (see, e.g., [29], [30]) or by computational modeling (see, e.g., [28], [31]), the first priority was always focused on its FunD. Using FunD to formulate protein samples was originally proposed in [32], [33] based on the 2005 FunDs in the SBASE-A database [34]. Since then, a series of new protein FunD databases were established, such as COG [35], KOG [35], SMART [36], Pfam [37], and CDD [38]. Of these databases, CDD contains the domains imported from COG, Pfam, and SMART, and hence is relatively much more complete [38] and will be adopted in this study. The version 2.11 of CDD contains 17,402 characteristic domains. Thus, using each of these domains as a base vector, a given protein sample can be defined as a vector in the 17402-D (dimensional) FunD space according to the following procedures:

#### Step 1

Use RPS-BLAST (Reverse PSI-BLAST) program [24] to conduct sequence alignment of the sequence of the query protein 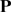 with each of the 17,402 domain sequences in the CDD database.

#### Step 2

If the significance threshold value (expect value) is 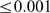 for the 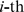 domain meaning that a “hit” is found, then the 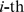 component of the protein 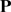 in the 17402-D space is assigned 1; otherwise, 0.

#### Step 3

The protein sample 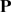 in the FunD space can thus be formulated as
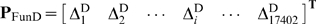
(6)where 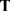 has the same meaning as in Eq.4, and
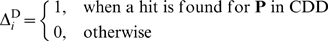
(7)


### 3. SeqEvo (Sequential Evolution) Descriptor

Biology is a natural science with historic dimension. All biological species have developed continuously starting out from a very limited number of ancestral species. The evolution in protein sequences involves changes of single residues, insertions and deletions of several residues [39], gene doubling, and gene fusion. In the course of time such changes accumulate, so that many similarities between initial and resultant amino acid sequences are eliminated, but the corresponding proteins may still share many common attributes, such as belonging to a same subcellular location and possessing basically the same function. To incorporate this kind of evolutionary effects, let us use the “Position-Specific Scoring Matrix” or “PSSM” [24] to express the protein sample 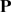, as formulated by
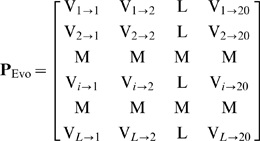
(8)where 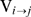 represents the score of the amino acid residue in the 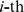 position of the protein sequence being changed to amino acid type 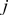 during the evolutionary process, and 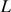 the sequence length of protein 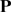. Here, the numerical codes 1, 2, …, 20 are used to denote the 20 native amino acid types according to the alphabetical order of their single character codes. The 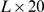 scores in Eq.8 were generated by using PSI-BLAST [24] to search the Swiss-Prot database (version 55.3 released on 29-Apr-2008) through three iterations with 0.001 as the 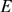-value cutoff for multiple sequence alignment against the sequence of the protein 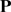, followed by a standard conversion given below:
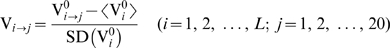
(9)where 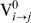 represent the original scores directly created by PSI-BLAST [24] that are generally shown as positive or negative integers (the positive score means that the corresponding mutation occurs more frequently than expected by chance, while the negative means just the opposite); the symbol 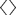 means taking the average of the quantity therein over 20 native amino acids, and 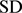 means the corresponding standard deviation. The converted values obtained by Eq.9 will have a zero mean value over the 20 amino acids and will remain unchanged if going through the same conversion procedure again. However, according to the descriptor of Eq.8, proteins with different lengths will correspond to row-different matrices causing difficulty for developing a predictor able to uniformly cover proteins of any length. To make the descriptor become a size-uniform matrix, one possible avenue is to represent a protein sample 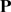 by
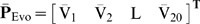
(10)where
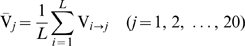
(11)where 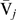 represents the average score of the amino acid residues in the protein 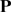 being changed to amino acid type 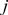 during the evolutionary process. However, if 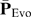 of Eq.10 was used to represent the protein 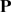, all the sequence-order information during the evolutionary process would be missed. To avoid complete loss of the sequence-order information, the concept of the pseudo amino acid composition (PseAAC) as originally proposed in [15] was utilized; i.e., instead of Eq.10, let us use the pseudo position-specific scoring matrix as given by
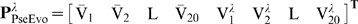
(12)to represent the protein 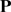, where
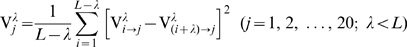
(13)meaning that 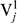 is the correlation factor by coupling the most contiguous position-specific scoring matrix scores along the protein chain for the amino acid type 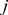; 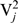 that by coupling the second-most contiguous position-specific scoring matrix scores; and so forth. Note that, as mentioned in the Material section of [13], the length of the shortest protein sequence in the benchmark dataset is 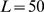, and hence the value allowed for 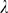 in Eq.13 must be smaller than 50. When 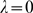, 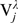 becomes a naught element and Eq.12 is degenerated to Eq.10.

It is instructive to point out that the above three protein descriptors, i.e., 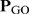 of Eq.4, 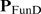 of Eq.6, and 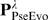 of Eq.12, can be actually deemed as three different kinds of PseAAC as well [40]. This is because, according to its original definition, the PseAAC is actually a set of discrete numbers [15] as long as it is different from the classical amino acid composition and it is derived from a protein sequence that is able to harbor some sort of sequence order or pattern information. The concept of PseAAC has also been widely used to deal with many other protein-related problems and sequence-related systems (see, e.g., [41], [42], [43], [44], [45], [46], [47], [48], [49], [50], [51], [52], [53], [54], [55], [56]).

### 4. Prediction Engine and Process

The prediction engine used in this study is the ensemble classifier 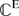 [12] formed by fusing many basic individual classifiers operated according to the OET-KNN (Optimized Evidence-Theoretic K Nearest Neighbor) rule [57], [58]. OET-KNN is a very powerful classifier as demonstrated in identifying membrane protein types [58]. For reader's convenience, a brief introduction about OET-KNN is given below.

Let us consider a problem of classifying 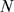 plant protein entities into 12 categories (subcellular location sites). The problem can be formulated as
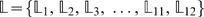
(14)The available information is assumed to consist in a training dataset
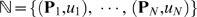
(15)where the 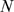 plant proteins 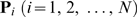 and their corresponding location labels 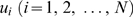 take the values in 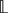 of Eq.14. According to the KNN (*K*-Nearest Neighbors) rule [59], an unclassified protein 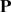 is assigned to the class (or location) represented by the majority of its *K* nearest neighbors of 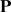. Owing to its good performance and simple-to-use feature, the KNN rule, also named as “voting KNN rule”, is quite popular in pattern recognition community.

The ET-KNN (Evidence Theoretic *K*-nearest Neighbors) rule is a pattern classification method based on the Dempster-Shafer theory of belief functions [57]. In the classification process, each neighbor of a protein to be classified is considered as a piece of evidence supporting certain hypotheses concerning the class (or location) membership of that protein. Based on this kind of evidence, the basic belief masses are assigned to each subset concerned. Such masses are obtained for each of the *K* nearest neighbors of the protein under consideration and aggregated using the Dempster's rule of combination [60]. A decision is made by assigning the query protein to the class (or location) with the maximum credibility.

Suppose 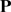 is a query protein to be classified, and 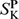 is the set of its *K*-nearest neighbors in the training dataset 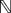 of Eq.15. Thus, for any 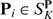, the knowledge that 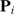 belongs to class (or location) 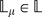 can be considered as a piece of evidence that increases our belief that 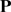 also belongs to 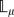. According to the basic belief assignment mapping theory [60], this piece of evidence can be formulated by
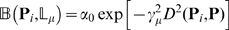
(16)where 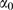 is a fixed parameter, 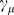 is a parameter associated with class (or location) 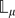, and 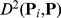 is the square distance between 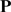 and 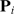. In this study, when the proteins are represented by the GO descriptor mode (cf. Eq.4) or the FunD mode (cf. Eq.6), then 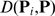 is defined as 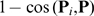, i.e.
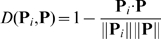
(17)where 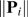 and 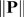 are the moduluses of 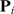 and **P**, respectively. When the proteins are represented by the PseEvo model (cf. Eq.12), then 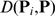 is defined as the Euclidean distance between 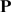 and 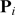.

In the ET-KNN rule, it was not addressed how to optimally select the parameters. In 1998 an optimization procedure to determine the optimal or near-optimal parameter values of 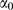 and 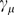 was proposed from the data by minimizing an error function [61]. It was observed that the OET-KNN rule obtained thru such an optimization treatment would lead to a substantial improvement in classification accuracy.

The belief function of 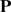 belonging to class (or location) 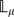 is a combination of its *K*-Nearest Neighbors, and can be formulated as
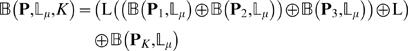
(18)where 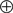 is called the orthogonal sum, which is commutative and associative. According to Dempster's rule [60], the belief function of Eq.18 can be expressed as
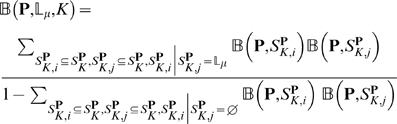
(19)where 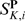 is the *i*-th possible subset of 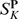, and 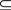, 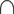, and 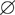 are the symbols in set theory, representing “contained in”, “intersection”, and the empty set, respectively.

A decision is made by assigning the query protein 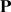 to the 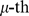 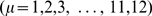 class (or location) with which the belief function of Eq.19 has the maximum value; i.e.,
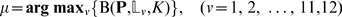
(20)where 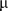 is the argument of 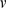 that maximizes the belief function 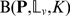. If there are two and more arguments leading to a same maximum value for 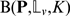, the query protein will be randomly assigned to one of the subcellular locations associated with these arguments although this kind of tie case rarely happens.

The power of the ensemble classifier 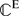 is also reflected by the fact that a statistical predictor established by fusing many basic individual predictors will significantly improve its performance as demonstrated by the recent studies on protein folding rate predictions [62], [63]. For the detailed procedures of how to fuse many individual OET-KNN classifiers to form the ensemble classifier 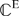, see Eqs.30–35 in [12]. For the procedures of how to make 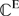 able to deal with both single-location and multiple-location proteins, see Eqs.36–48 of [12].

The prediction is processed according to the following order.

**(1)** If the query protein can be expressed as a meaningful or productive descriptor in the GO database via its representative proteins in 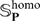, then 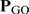 of Eq.4 should be input into the prediction engine for identifying its subcellular location site(s). And the output will be determined by fusing many basic OET-KNN predictors [12] with different numbers of 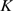 (cf. Eq.18–20), the parameter of the nearest neighbor rule [57].

**(2)** If the query protein does not have significant homology to any protein in the Swiss-Prot database, i.e., 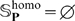, or its representative proteins in 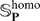 do not contain any useful GO information, then both the FunD representation 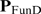 of Eq.6 and the pseudo position-specific scoring matrix representation 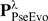 of Eq.12 should be input into the prediction engine. The output will be determined by fusing many basic OET-KNN predictors [12] with different numbers of 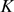 (cf. Eq.20) and 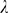 (cf. Eq.13).

The whole process can be formulated as
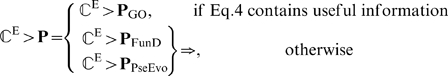
(21)where 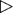 represents the identification operator, and 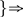 means fusing the results generated from its left side.

**Figure 2:**
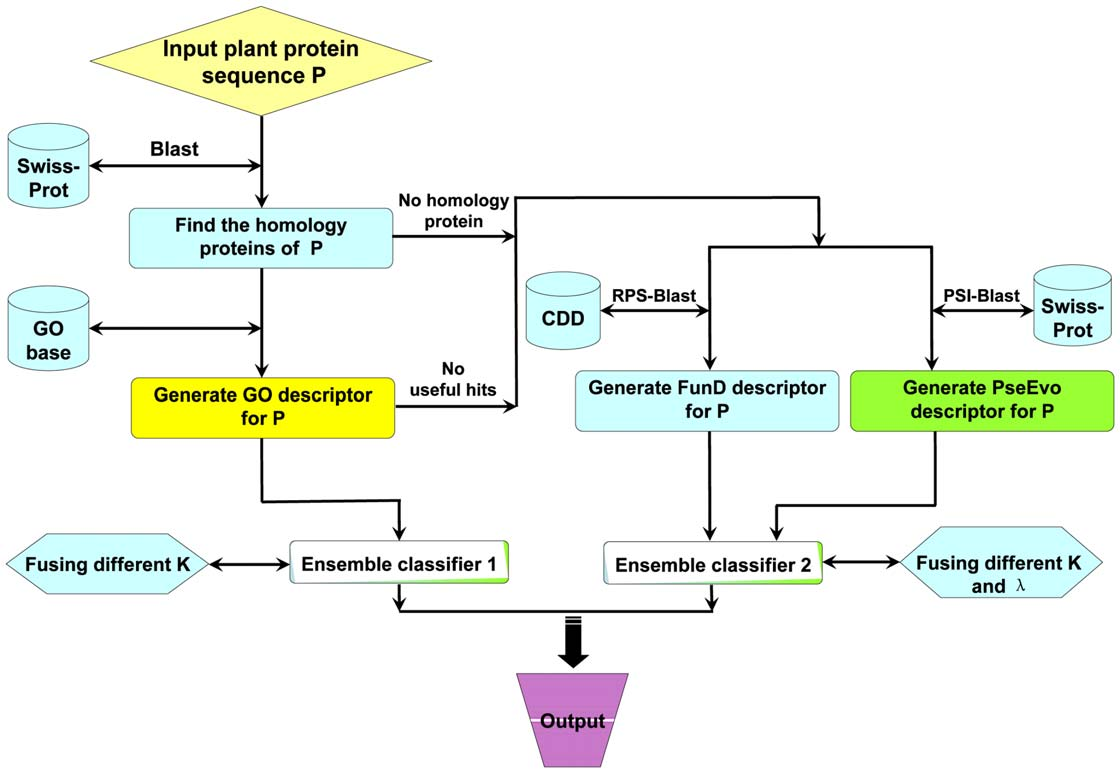
A flowchart to show the prediction process of Plant-mPLoc.

The entire ensemble classifier thus established is called “**Plant-mPLoc**”, where “m” stands for the first character of “multiple”, meaning that Plant-mPLoc is able to deal with proteins having both single and multiple subcellular locations. To provide an intuitive picture, a flowchart is given in **Fig. 2** to illustrate the prediction process of Plant-mPLoc.

### Protocol Guide

For the convenience of experimental scientists, a user-friendly web-server for Plant-mPLoc was established. Here let us provide a step-by-step guide on how to use the web-server to get the desired results.

#### Step 1

**Figure 3:**
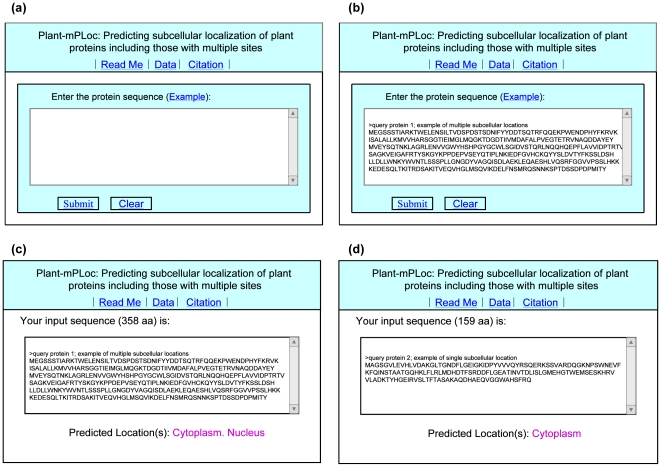
Semi-screenshot to show the prediction steps. (**a**) the top page of the Plant-mPLoc web server at http://www.csbio.sjtu.edu.cn/bioinf/plant-multi/, (**b**) the input of a query protein in FASTA format, (**c**) the output predicted by Plant-mPLoc for the query protein 1 in the Example window, and (**d**) the output for the query protein 2 in the Example window.

Open the web server at http://www.csbio.sjtu.edu.cn/bioinf/plant-multi/ and you will see the top page of the predictor on your computer screen, as shown in **Fig. 3a**. Click on the Read Me button to see a brief introduction about Plant-mPLoc predictor and the caveat in using it.

#### Step 2

Either type or copy and paste the query protein sequence into the input box at the center of **Fig. 3a**. The input sequence should be in the FASTA format. A sequence in FASTA format consists of a single-line description, followed by lines of sequence data. The first character of the description line is a greater-than symbol (“>”) in the first column. All lines should be shorter than 80 characters. Examples to show the input sequences format can be seen by clicking on the Example button right above the input box. For more information about FASTA format, visit http://en.wikipedia.org/wiki/Fasta_format.

#### Step 3

Click on the Submit button to see the predicted result. For example, if you use the sequence of query protein 1 in the Example window, the input screen should look like the illustration in **Fig. 3b**; after clicking the Submit button, you will see “Cytoplasm. Nucleus” shown on the predicted result window (**Fig. 3c**), meaning that the protein is a multiplex one, which can simultaneously occur in “cytoplasm” organelle and “nucleus” organelle, fully consistent with experimental observations. However, if using the sequence of query protein 2 in the Example window as an input, you will see “Cytoplasm” shown on the predicted result window (**Fig. 3d**), meaning that the protein is a single-location one occurring in “cytoplasm” compartment only, also consistent with experimental observations. It takes less than 15 seconds for a protein sequence of 300 amino acids before the predicted result appears on your computer screen. Generally speaking, the longer the sequence is, the more time it is needed.

#### Step 4

Click on the Citation button to find the relevant papers that document the detailed development and algorithm of Plant-mPLoc.

#### Step 5

Click on the Data button to download the benchmark datasets used to train and test the Plant-mPLoc predictor.

#### Caveat

To obtain the predicted result with the expected success rate, the entire sequence of the query protein rather than its fragment should be used as an input. A sequence with less than 50 amino acid residues is generally deemed as a fragment

## Results and Discussion

In statistical prediction, the following three methods are often used to examine the quality of a predictor: independent dataset test, subsampling test, and jackknife test [64]. Since independent dataset can be treated as a special case of sub-sampling test, one benchmark dataset is sufficient to serve all the three kinds of cross-validation. However, as elucidated in [18] and demonstrated by Eq.50 of [12], among the three cross-validation methods, the jackknife test is deemed the most objective that can always yield a unique result for a given benchmark dataset and hence has been increasingly and widely adopted to examine the power of various predictors (see, e.g., [42], [46], [51], [53], [55], [65], [66], [67], [68], [69]). Particularly for a benchmark dataset in which none of proteins included has 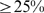 pairwise sequence identity to any other in a same subset (subcellular location), such as the one used in the current study (cf. **Table S1**), it would be highly unlikely to get an over-estimated success rate by the jackknife test. Quite the contrary, the success rate derived by the jackknife test on such kind of stringent dataset would actually be under-estimated in comparison with the success rates of practical applications in most cases, as will be seen later.

For the details of how to calculate the overall success rate for a statistical system with both single-location and multiple-location proteins, see Eqs.43–48 and Fig. 4 of [12], where the details of how to count the false positives (over-predictions) and false negatives (under-predictions) were also elaborated.

Let us first compare the current predictor Plant-mPLoc with the old Plant-PLoc [13]. Listed in **Table 2** are the results obtained with Plant-PLoc [13] and Plant-mPLoc, respectively, on the benchmark dataset (cf. **Table S1**) by the jackknife cross-validation test. During the testing process, only the sequences of proteins in **Table S1** but not their accession numbers were used as inputs in order to make the comparison between the two predictors under exactly the same condition. As we can see from **Table 2**, for such a stringent benchmark dataset, the overall success rate achieved by the new predictor is 63.7%, which is more than 25% higher than that by Plant-PLoc [13].

**Table 2:** A comparison of the jackknife success rates by Plant-PLoc [13] and the current Plant-mPLoc on the benchmark dataset (cf. **Table S1**) that covers 12 location sites of plant proteins in which none of the proteins included has 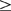
25% pairwise sequence identity to any other in a same location.

Subcellular location	Success rate^a^	
	Plant-PLoc	Plant-mPLoc
Cell membrane	15/56 = 26.8%	24/56 = 42.9%
Cell wall	7/32 = 21.9%	8/32 = 25.0%
Chloroplast	184/286 = 64.3%	248/286 = 86.7%
Cytoplasm	51/182 = 28.0%	72/182 = 39.6%
Endoplasmic reticulum	1/42 = 2.4%	17/42 = 40.5%
Extracellular	4/22 = 18.2%	3/22 = 13.6%
Golgi apparatus	6/21 = 28.6%	6/21 = 28.6%
Mitochondrion	26/150 = 17.3%	114/150 = 76.0%
Nucleus	92/152 = 60.5%	136/152 = 89.5%
Peroxisome	2/21 = 9.5%	14/21 = 66.7%
Plastid	9/39 = 23.1%	4/39 = 10.3%
Vacuole	4/52 = 7.7%	26/52 = 50.0%
Total	401/1055 = 38.0%	672/1055 = 63.7%

a Note that in order to make the comparison under exactly the same condition, only the sequences of proteins in the **Table S1** but not their accession numbers were used as inputs during the prediction.

Now, let us compare the current predictor with TargetP [6] and Predotar [8], two popular predictors widely used for predicting the subcellular locations of plant proteins. As mentioned in Introduction, the two predictors only cover three or four location sites. Therefore, it can be easily conceived that they would yield even much lower success rates when tested by the current benchmark dataset that covers twelve location sites.

Actually, even if tested by a benchmark dataset within the scope that can be covered by TargetP [6] or Predotar [8], the success rate by the current Plant-mPLoc predictor is also much higher than those by the two predictors, as demonstrated below.

Let us compare Plant-mPLoc with TargetP [6] first. The TargetP predictor also has a web-server at http://www.cbs.dtu.dk/services/TargetP/, with a built-in training dataset covering the following four items: “mitochondria”, “chloroplast”, “secretory pathway”, and “other”. Since the “secretory pathway” is not a final destination of subcellular location as annotated in Swiss-Prot databank, and hence was removed from the comparison. Also, the location of “other” is not a clear site for comparison, and should be removed as well. Thus, in order to compare TargetP with the new predictor Plant-mPLoc, let us construct an independent testing dataset by randomly picking testing proteins according to the following criteria: (i) they must belong to plant proteins, as clearly annotated in Swiss-Prot database; (ii) they must neither occur in the training dataset of TargetP nor occur in the training dataset of Plant-mPLoc in order to avoid the memory bias; (iii) their experimentally observed subcellular locations are known as clearly annotated in Swiss-Prot database, and also these locations must be within the scope covered by TargetP as a compromise for rationally utilizing its web-server. By following the above procedures, we obtained a degenerate independent testing dataset consisting of 1,775 plant proteins, of which 1,500 belong to chloroplast and 275 belong to mitochondrion. The accession numbers and sequences of these 1,775 proteins are given in **Table S2**.

The predicted results by TargetP [6] and the current Plant-mPLoc for each of the 1,775 independent testing proteins are listed in **Table S3**, where for facilitating comparison, the corresponding experimental results are also given. By examining **Table S3**, we can see the following. **(1)** Many proteins whose subcellular locations were misidentified by TargetP have been corrected by Plant-mPLoc. **(2)** Many proteins, which were identified by TargetP as belonging to the location of “other”, have been identified as “chloroplast” or “mitochondrion”, fully consistent with experimental observations. **(3)** There are quite a few proteins whose subcellular locations were incorrectly predicted by Plant-mPLoc, or the results yielded by Plant-mPLoc contain some false positives. Even though, the overall success rate by Plant-mPLoc on the 1,755 independent proteins is over 86%, which is at least more than 40% higher than that by TargetP [6].

Now, let us compare Plant-mPLoc with Predotar [8]. The web-server of Predotar is at: http://urgi.versailles.inra.fr/predotar/predotar.html, with a built-in training dataset covering the following four items: “endoplasmic reticulum”, “mitochondrion”, “plastid”, and “other”. Since the term “other” is not a clear description for subcellular location, and was removed from comparison. Thus, by following the aforementioned similar criteria as in constructing the independent dataset for comparing TargetP with Plant-mPLoc, we also constructed a degenerate independent dataset to compare Predotar [8] with Plant-mPLoc. The dataset consists of 381 plant proteins, of which 48 belong to endoplasmic reticulum, 253 belong to mitochondrion, and 70 belong to plastid. The accession numbers and sequences of these 381 proteins are given in **Table S4**. The predicted results by Predotar [8] and the current Plant-mPLoc for the 381 independent testing proteins and their corresponding experimental results are listed in **Table S5**, from which we can see the following. **(1)** Many proteins whose subcellular locations were correctly identified by Plant-mPLoc were unable to identify by Predotar [8] although all these location sites are within its coverage scope. **(2)** Many proteins whose subcellular locations were misidentified by Predotar [8] have been corrected by Plant-mPLoc. **(3)** Although Plant-mPLoc also had quite a few incorrect and false positive predicted results, its overall success rate for the 381 independent proteins could still be over 70%, which is at least more than 30% higher than that by Predotar [8].

Furthermore, it is interesting to see from **Table S3** and **Table S5** that some proteins with multiple locations have been correctly predicted by Plant-mPLoc. For example, according to the annotations of Swiss-Prot databank, the proteins with codes Q5YLB5, Q9FV51, and Q9LJL3 can coexist in both “chloroplast” and “mitochondrion” while the protein with code Q42560 can coexist in both “cytoplasm” and “mitochondrion”, and the predicted results by Plant-mPLoc are exactly so. This is beyond the reach of TargetP [6] and Predotar [8].

From the above three comparisons, we can now make the following points more clear.

The more stringent a benchmark dataset is in excluding homologous and high similarity sequences, or the more subcellular location sites it covers, the more difficult for a predictor to achieve a high overall success rate, as can be easily understood by considering the following cases. For a benchmark dataset only covering three subcellular locations each containing same number of proteins, the overall success rate by random assignments would generally be 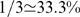; while for a benchmark dataset covering 12 subcellular locations, the overall success rate by random assignments would be only 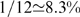. This means that the former is more than four times the latter.

Also, a predictor tested by jackknife cross-validation is very difficult to yield a high success rate when performed on a stringent benchmark dataset in which none of proteins included has 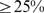 pairwise sequence identity to any other in a same subset (subcellular location). That is why the overall success rate achieved by Plant-mPLoc was only 63.7% when tested by the jackknife cross-validation on the benchmark dataset of **Table S1** but was over 86% and 70% when tested by the independent datasets of **Table S2** and **Table S4**, respectively. However, regardless of using what test methods or test datasets, one thing is crystal clear, i.e., the overall success rates achieved by the current Plant-mPLoc are significantly higher than those by its counterparts.

Meanwhile, it has also become understandable why the success rates as originally reported for TargetP [6] and Predotar [8] were over-estimated. This is because the benchmark datasets adopted by the two predictors only cover less than one-third of the location sites that are covered by the current Pant-mPLoc. Besides, the benchmark datasets used by TargetP and Predotar to estimate their success rates contain many homologous sequences. For the benchmark dataset used by Predotar [8], the cutoff threshold was set at 80%, meaning that only those sequences which have 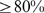 pairwise sequence identity to any other in a same subset were excluded [8]; while for the benchmark dataset used in TargetP [6], even no such a cutoff percentage was indicated. Compared with the current benchmark dataset (cf. **Table S1**) in which none of proteins included has 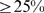 pairwise sequence identity to any other in a same subset, the benchmark datasets adopted in Predotar and TargetP are much less stringent and hence cannot avoid homologous bias and over estimation.

Plant-mPLoc was evolved from Plant-PLoc [13] through a top-down approach improvement. The new predictor distinguishes itself from the old one by the following remarkable features. **(1)** The ability of prediction is extended to cover both single-location and multiple-location proteins. **(2)** The input of accession number for using the higher-level GO approach [18] to perform the prediction is no longer needed; this is particularly useful when dealing with protein sequences without accession numbers available. **(3)** For those plant proteins without useful GO information to conduct the higher-level prediction, a sophisticated combination approach by fusing the FunD information and SeqEvo information is developed to replace the simple PseAAC approach [15].

It is instructive to point out that in a broader sense the protein descriptors, 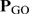, 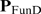, and 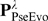 as introduced in the current study, are actually three different forms of PseAAC [40]. Accordingly, it is essentially through the concept of PseAAC [15] that the GO information, FunD information, and SeqEvo information have been effectively incorporated into the predictor Plant-mPLoc. Plant-mPLoc is available as a web-server at http://www.csbio.sjtu.edu.cn/bioinf/plant-multi/.

Finally, let us consider the following hypothetical case: a single amino acid mutation in the signal part of a protein sequence might lead it to a completely different subcellular location site. Can Plant-mPLoc be used to deal with such a subtle case? Like all existing predictors in this area, Plant-mPLoc is a statistical predictor. As a statistical predictor, it would generally not be so sensitive to reflect the change of only one amino acid. Nevertheless, since Plant-mPLoc is an ensemble classifier formed by fusing many basic individual classifiers as well as by incorporating functional domain and evolution informations, it would be relatively more competent in dealing with the cases of mutated sequences than those predictors based on single classifier alone. Of course, it remains a challenging problem how to incorporate into a statistical predictor with the subtle effect of a single amino acid mutation at the signal peptide of a protein.

## Supporting Information

Table S1 This benchmark dataset S for Plant-mPLoc includes 1,055 plant protein sequences (978 different proteins), classified into 12 plant subcellular locations. Among the 978 different proteins, 904 belong to one subcellular location, 71 to two locations, and 3 to three locations. Both the accession numbers and sequences are given. None of the proteins has ≥25% sequence identity to any other in the same subset (subcellular location). See the text of the paper for further explanation.
(0.78 MB PDF)

Table S2 The degenerate testing dataset used for comparing the performance between TargetP (Emanuelsson, et al. J. of Mol. Biol. 2000, 300: 1005–1016) and Plant-mPLoc of this paper. The dataset contains 1,775 plant proteins classified into 2 subcellular locations: (1) chloroplast, and (2) mitochondrion. To avoid bias, none of the proteins included here occurs in the training dataset of TargetP, nor in the training dataset of Plant-mPLoc. See the text of the paper for further explanation.
(0.91 MB PDF)

Table S3 List of the results predicted by TargetP (Emanuelsson et al. J. Mol. Biol. 2000, 300: 1005–1016) and Plant-mPLoc on the 1,775 independent proteins in the Table S2, and their experimental subcellular locations as annotated in Swiss-Prot databank (version 55.3 released on 29-Apr-2008). Note for TargetP outputs, “C” means “Chloroplast”, “M” means “Mitochondrion”, “S” means “Secretory pathway”, and “_” means “Any other location”.
(0.41 MB PDF)

Table S4 The degenerate testing dataset used for comparing the performance between Predotar (Small et al., Proteomics 2004, 4: 1581–1590) and Plant-mPLoc of this paper. The dataset contains 381 plant proteins classified into 3 subcellular locations: (1) endoplasmic reticulum, (2) mitochondrion, and (3) plastid. To avoid bias, none of the proteins included here occurs in the training dataset of TargetP, nor in the training dataset of Plant-mPLoc. See the text of the paper for further explanation.
(0.25 MB PDF)

Table S5 List of the results predicted by Predotar (Small et al., Proteomics 2004, 4:1581–90) and Plant-mPLoc on the 381 independent proteins in the Table S4, and their experimental subcellular locations as annotated in Swiss-Prot databank (version 55.3 released on 29-Apr-2008). Note for the Predotar output, “ER” means “Endoplasmic reticulum”.
(0.16 MB PDF)
