# Supplementary material for: Plant-mPLoc: A Top-Down Strategy to Augment the Power for Predicting Plant Protein Subcellular Localization
Source: PLoS One. 2010 Jun 28;5(6):e11335. doi: 10.1371/journal.pone.0011335 (PMC2893129; doi:10.1371/journal.pone.0011335)
Supplement: Table S1 — This benchmark dataset S for Plant-mPLoc includes 1,055 plant protein sequences (978 different proteins), classified into 12 plant subcellular locations. Among the 978 different proteins, 904 belong to one subcellular location, 71 to two locations, and 3 to three locations. Both the accession numbers and sequences are given. None of the proteins has ≥25% sequence identity to any other in the same subset (subcellular location). See the text of the paper for further explanation. (0.78 MB PDF) [file pone.0011335.s001.pdf]

Table S1. This benchmark dataset $\mathbb{S}$ for **Plant-mPLoc** includes 1,055 plant protein sequences (978 different proteins), classified into 12 plant subcellular locations. Among the 978 different proteins, 904 belong to one subcellular location, 71 to two locations, and 3 to three locations. Both the accession numbers and sequences are given. None of the proteins has ≥25% sequence identity to any other in the same subset (subcellular location). See the text of the paper for further explanation.

---

(1) $\mathbb{S}_1$: 56 cell membrane proteins

>P25848
MSATKKTYSSTTSAKSKHSVRVAQTTADAALEAVYEMSGDSGDSFDYSKSVGQSAESVPA
GAVTAYLQRMQREGLIQNFGCMVAVEEPNFCVIAYSENASEFLDLIPQAVPSMGEMDVLG
IGTDIRTLFTPSSSAALEKAAATQDISLLNPITVHCRRSGKPLYAIAHRIDIGIVIDFEA
VKMIDVPVSAAAGALQSHKLAARAITRLQALPGGDIELLCDTIVEEVRELTGYDRVMAFK
FHEDEHGEVVAEIRRMDLEPYMGLHYPATDIPQASRFLLMKNRVRLIADCYASPVKLIQD
PDIRQPVSLAGSTLRAPHGCHAQYMGNMGSIASLVMAVIINDNEEYSRGAIQRGRKLWGL
VVCQHTSPRTVPFPLRSVCEFLMQVFGMQLNLHVELAAQLREKHILRTQTLLCDMLLRDA
PIGIVSQTPNIMDLVKCDGAALYYGKRVWLLGTTPTENQIKEIADWLLEHHNDSTGLSTD
SLADANYPGAHLLGDAVCGMAAAKITAKDFLFWFRSHTATEVKWGGAKHDPDEKDDGRKM
HPRSSFKAFLEVVNKRSPPWEDVEMDAIHSLQLILRGSFRDIADSDTKTMIHARLNDLKL
QGVEERNALANEMSRVLETAAAPILAVDSRGMINAWNAKIAQVTGLPVEEAMHCSLTKDL
VLDESVVVVERLLSLALQGEEEQNVEIKLKTFGTQTTERAVILIVNACCSRDASDFVVGV
FFVGQDVTEQRMFMDRFTRIQGGEKTTVQDPHPLMRPSFDGDEFGRTFKRNSALGGLKDH
ATGSVERLDLYLRRAEECMEVMETIPSPKFNNKQCQYLAGKLKAVLQSASLFLRISHHEH
HELGASIDMGRHVEIFKLLLALAKEIESFIQGCCKDEWIKAAMTLTNVSEYVSSMGFNLE
LCKIAFCKSCAASGSLTLDQIEVICKDEAEVVKRNASIDVDTLFAKVIYDLTEKTLSSDQ
NDLAIYLLQRLKRAKPILPSFSSRPSWWNFYDDWSFSEKFFQWIQITGSLGSGSSATVEK
AVWLGTPVAKKTFYGRNNEDFKREVEILAELCHPNITSMFCSPLYRRKCSIIMELMDGDL
LALMQRRLDRNEDHDSPPFSILEVVDIILQTSEGMNYLHEKGIIHRDLKSMNILVKSVKV
TKSEIGYVHVKVADFGLSKTKDSSTRYSNQTWNRGTNRWMAPEVINLGYESTEGEISFDG
KVPKYPLKSDVYSFGMVCYEVLTGDVPFPEEKNPNNVKRMVLEGVRPDLPAHCPIELKAL
ITDCWNQDPLKRPSFAVICQKLKYLKYLLMTGFSSYQDSYPSTEEPS
>P93788
MAELEAKKVEIVDPAPPAPGPVEAPKEVVADEKAIVAPALPPPAEEKEKPDDSKALVVVE
TKAPEPADEKKEGSIDRDAVLARVATEKRVSLIKAWEESEKSKAENKAQKKVSAIGAWEN
SKKANLEAELKKMEEQLEKKKAEYTEKMKNKIALLHKEAEEKRAMIEAKRGEDLLKAEEL
AAKYRATGTAPKKILGIF
>Q9LDC0
MDESLEHQTQTHDQESEIVTEGSAVVHSEPSQEGNVPPKVDSEAEVLDEKVSKQIIKEGH
GSKPSKYSTCFLHYRAWTKNSQHKFEDTWHEQQPIELVLGKEKKELAGLAIGVASMKSGE
RALVHVGWELAYGKEGNFSFPNVPPMADLLYEVEVIGFDETKEGKARSDMTVEERIGAAD
RRKMDGNSLFKEEKLEEAMQQYEMAIAYMGDDFMFQLYGKYQDMALAVKNPCHLNIAACL
IKLKRYDEAIGHCNIVLTEEEKNPKALFRRGKAKAELGQMDSARDDFRKAQKYAPDDKAI
RRELRALAEQEKALYQKQKEMYKGIFKGKDEGGAKSKSLFWLIVLWQWFVSLFSRIFRRH

RVKAD
>Q8LCS8
MAEVKDQLEIKFRLNDGSDIGPKLFPDATTVATLKETVVAQWPRDKENGPKTVKDVKLIS
AGRILENNKTVGDCRSPVGNFSGAVTTMHVIIQHQVTEKEKKKKKPKGDLKQNKCVCLCF
GARC
>Q9C7F7
MKGLHLHLVLVTMTIVASIAAAAPAAPGGALADECNQDFQKVTLCLDFATGKATIPSKKC
CDAVEDIKERDPKCLCFVIQQAKTGGQALKDLGVQEDKLIQLPTSCQLHNASITNCPKLL
GISPSSPDAAVFTNNATTTPVAPAGKSPATPATSTDKGGSASAKDGHAVVALAVALMAVS
FVLTLPRHVTLGM
>Q9FJ62
MINMRDNPTMHVLQASKFLFLALILIQLLSTQLFAQRSKSPWQTLTGDAPLVIARGGFSG
LLPDSSLDAYSFVSQTSVPGAVLWCDVQLTKDAIGLCFPDVKMMNASNIQDVYPKRKTSY
LLNGVPTQDWFTIDFNFKDLTKVILKQGILSRSAAFDGNSYGISTVKDISTQLKPEGFWL
NVQHDAFYAQHNLSMSSFLLSISKTVIIDYLSSPEVNFFRNIGRRFGRNGPKFVFRFLEK
DDVEVSTNQTYGSLAGNLTFLKTFASGVLVPKSYIWPIESQYLLPRTSFVQDAHKAGLEV
YASGFGNDFDLAYNYSFDPLAEYLSFMDNGDFSVDGLLSDFPLTASSAVDCFSHLGSNAS
SQVDFLVISKNGASGDYPGCTDLAYTKAIKDGADVIDCSLQMSSDGIPFCLSSINLGEST
NVVQSPFRNRSTTVPEIGSLPGIYSFSLAWSEIQTLRPAIENPYSREFTMFRNPRERSSG
KFVSLSDFLNLAKNSSSLTGVLISVENATYLREKQGLDAVKAVLDTLTEAGYSNKTTTTR
VMIQSTNSSVLIDFKKQSRYETVYKVEETIRDILDTAIEDIKKFADAVVISKKSVFPTSE
SFTTGQTKLVERLQKFQLPVYVEVFRNEFVSQPWDFFADATVEINSHVTGAGINGTITEF
PLTAARYKRNSCLTRKDVPPYMIPVQPAGLLTIVSPASLPPAEAPSPVFTDADVTEPPLP
PVSARAPTTTPGPQSTGEKSPNGQTRVALSLLLSAFATVFASLLLL
>Q9SD53
MADKTDIISSSSDKASPPPPSAFRNYLSSGSKEPVLLLESAGKESCCIFRVPESFVALNP
KAYKPKVVSIGPYHYGEKHLQMIQQHKPRLLQLFLDEAKKKDVEENVLVKAVVDLEDKIR
KSYSEELKTGHDLMFMMVLDGCFILMVFLIMSGNIELSEDPIFSIPWLLSSIQSDLLLLE
NQVPFFVLQTLYVGSKIGVSSDLNRIAFHFFKNPIDKEGSYWEKHRNYKAKHLLDLIRET
FLPNTSESDKASSPHVQVQLHEGKSGNVPSVDSKAVPLILSAKRLRLQGIKFRLRRSKED
SILNVRLKKNKLQIPQLRFDGFISSFFLNCVAFEQFYTDSSNEITTYIVFMGCLLNNEED
VTFLRNDKLIIENHFGSNNEVSEFFKTISKDVVFEVDTSYLNNVFKGVNEYTKKWYNGLW
AGFRHTHFESPWTFLSSCAVLFVILLTMLQSTVAILSYLNDKKGNGNAAPPPLGLP
>Q5MFV8
MIGKVVVSVASILLIVGVAIGVVAYINKNGDANLSPQMKAVRGICEATSDKASCVKTLEP
VKSDDPNKLIKAFMLATRDAITQSSNFTGKTEGNLGSGISPNNKAVLDYCKKVFMYALED
LSTIVEEMGEDLNQIGSKIDQLKQWLTGVYNYQTDCLDDIEEDDLRKTIGEGIASSKILT
SNAIDIFHTVVSAMAKLNLKVEDFKNMTGGIFAPSDKGAAPVNKGTPPVADDSPVADPDG
PARRLLEDIDETGIPTWVSGADRKLMTKAGRGSNDGGARIRATFVVAKDGSGQFKTVQQA
VNACPEKNPGRCIIHIKAGIYREQVIIPKKKNNIFMFGDGARKTVISYNRSVKLSPGTTT
SLSGTVQVESEGFMAKWIGFKNTAGPMGHQAVAIRVNGDRAVIFNCRFDGYQDTLYVNNG
RQFYRNIVVSGTVDFIFGKSATVIQNSLIVVRKGNKGQFNTVTADGNEKGLAMKIGIVLQ
NCRIVPDKKLAAERLIVESYLGRPWKKFSTTVIINSEIGDVIRPEGWKIWDGESFHKSCR
YVEYNNRGPGAITNRRVNWVKIARSAAEVNDFTVANWLGPINWIQEANVPVTLGL

>O65493
MAFSAPSLSKFSLLVAISASALLCCAFARDFSIVGYTPEHLTNTDKLLELFESWMSEHSK
AYKSVEEKVHRFEVFRENLMHIDQRNNEINSYWLGLNEFADLTHEEFKGRYLGLAKPQFS
RKRQPSANFRYRDITDLPKSVDWRKKGAVAPVKDQGQCGSCWAFSTVAAVEGINQITTGN
LSSLSEQELIDCDTTFNSGCNGGLMDYAFQYIISTGGLHKEDDYPYLMEEGICQEQKEDV
ERVTISGYEDVPENDDESLVKALAHQPVSVAIEASGRDFQFYKGGVFNGKCGTDLDHGVA
AVGYGSSKGSDYVIVKNSWGPRWGEKGFIRMKRNTGKPEGLCGINKMASYPTKTK
>P46011
MSMQQETSHMTAAPQTNGHQIFPEIDMSAGDSSSIVRATVVQASTVFYDTPATLDKAERL
LSEAAENGSQLVVFPEAFIGGYPRGSTFELAIGSRTAKGRDDFRKYHASAIDVPGPEVER
LALMAKKYKVYLVMGVIEREGYTLYCTVLFFDSQGLFLGKHRKLMPTALERCIWGFGDGS
TIPVFDTPIGKIGAAICWENRMPSLRTAMYAKGIEIYCAPTADSRETWLASMTHIALEGG
CFVLSANQFCRRKDYPSPPEYMFSGSEESLTPDSVVCAGGSSIISPLGIVLAGPNYRGEA
LITADLDLGDIARAKFDFDVVGHYSRPEVFSLNIREHPRKAVSFKTSKVMEDESV
>P54774
MSQQGESSDPKSGKKDFSTAILERKKSPNRLVVDEAVNDDNSVVTMHPQTMEKLQLFRGD
TILIKGKKRKDTICIALADENCEEPKIRMNKVVRSNLRVRLGDVVSVHQCPDVKYGKRVH
ILPIDDTIEGVTGNLFDAFLKPYFLEAYRPVRKGDLFLVRGGMRSVEFKVVETDPGEYCV
VAPDTEIFCEGEPLKREDEERLDEVGYDDVGGVRKQMAQIRELVELPLRHPQLFKSIGVK
PPKGILLYGPPGSGKTLIARAVANETGAFFFCINGPEIMSKLAGESESNLRKAFEEAEKN
APSIIFIDEIDSIAPKREKTHGEVERRIVSQLLTLMDGLKSRAHVIVIGATNRPNSIDPA
LRRFGRFDREIDIGVPDEVGRLEVLRIHTKNMKLSDDVDLERIAKDTHGYVGADLAALCT
EAALQCIREKMDVIDLEDETIDAEVLNSMAVTNEHFQTALGTSNPSALRETVVEVPNVSW
EDIGGLENVKRELQETVQYPVEHPEKFEKFGMSPSKGVLFYGPPGCGKTLLAKAIANECQ
ANFISVKGPELLTMWFGESEANVREIFDKARQSAPCVLFFDELDSIATQRGSSVGDAGGA
ADRVLNQLLTEMDGMSAKKTVFIIGATNRPDIIDPALLRPGRLDQLIYIPLPDEDSRHQI
FKACLRKSPIAKNVDLRALARHTQGFSGADITEICQRACKYAIRENIEKDIERERKSREN
PEAMDEDTVDDEVAEIKAAHFEESMKFARRSVSDADIRKYQAFAQTLQQSRGFGSEFRFP
ESGDRTTTGSDPFAASAGGADEDDLYS
>P93339
MSVTELKERHMAATQTVNDLREKLKQKRLQLLDTDVSGYARSQGKTPVTFGPTDLVCCRI
LQGHTGKVYSLDWTPEKNRIVSASQDGRLIVWNALTSQKTHAIKLPCAWVMTCAFSPSGH
SVACGGLDSVCSIFNLNSPIDKDGNHPVSRMLSGHKGYVSSCQYVPDEDTHLITSSGDQT
CVLWDITTGLRTSVFGGEFQFGHTADVQSVSISSSNPRLFVSGSCDTTARLWDTRVASRA
QRTFYCHEGDVNTVKFFPDGNRFGTGSEDGTCRLFDIRTGHQLQVYYQPHGDGDIPHVTS
MAFSISGRLLFVRYSNGDCYVWDTLLAKVVLNLGAVQNSHEGXISCLGLSADGXXLCTGS
WDTNLKIWAFGGHRSVI
>Q8GUI4
MTRRAEFEMGLFVILQSMFLISLCSSQKPEEFLPEISPDTSPQPFLPFIAPSPMVPYINS
TMPKLSGLCSLNFSASESLIQTTSHNCWTVFAPLLANVMCCPQLDATLTIILGKASKETG
LLALNRTQSKHCLSDLEQILVGKGASGQLNKICSIHSSNLTSSSCPVINVDEFESTVDTA
KLLLACEKIDPVKECCEEACQNAILDAATNISLKASETLTDNSDRINDCKNVVNRWLATK
LDPSRVKETLRGLANCKINRVCPLVFPHMKHIGGNCSNELSNQTGCCRAMESYVSHLQKQ
TLITNLQALDCATSLGTKLQKLNITKNIFSVCHISLKDFSLQVGNQESGCLLPSLPSDAI

FDKDTGISFTCDLNDNIPAPWPSSSLSSASTCKKPVRIPALPAAASSQPRLHDEGVTRLV
IFVLSMLLVMLLS
>P54211
MADIKEGVEEGSVKVDMIKEPLTQGDTGVDEVDFAKITLDDAFKYLNCNKHGLSSAEAAA
RLQQHGPNKLPDSSRNPVLVFLGYMWNPLAWAMEAAAIISIALLDVADFVLIVGLLLINA
IISFYEESNADKAIKALTAALAPKAMVVRDGAIVTIDAVNLVPGDVILIRLGNIVPADVK
LLEEEGADEGEQEAPMQIDQAALTGESLPAKKFTGDVAFSGSSIKQGERHAVVYATGVNT
FFGRAAALISGTNNVSNLQTVMNKMSAICIVTILLWVVVELAVQFGHYSHECVGGREGCP
TLLNMLVVLVGGIPIAMPTVLSVTLALGAYKLAREGAIVTRMSAVEEMAGMDVLCSDKTG
TLTLNKLSIDKSMVVPVGNMGVDEIMRMGALSANTVTEEPIDMVLWESYPDRETIKRDYK
HTKYFPFNPNDKITIATCLEIATGRVFRVLKGSPQVVLAKAWNAAELDATVNQKMVEFAN
RGFRALGLAMADGDGKDGTKWEMLALLPLFDPPRHDTKETIEHCQNQGIQVKMITGDHLL
IGKETAKMLGMGTEMFPSEVMIKARNGDASQLHGYKNFVEMVETCNGFAQVFPEHKFEIV
KILQDSNHVVGMTGDGVNDAPALKKADVGVAVADATDAARGAADIVLTEPGLSTIVTAVI
GARKIFQRMTTYSKYTIAMTFRICFTFGLITVIYDWYFPTILIVIMAVFNDGAMIALSKD
RVVASKTPNSWNITNIFIMGMVYGLYLTLSTWALYQTATKTTFFEDKTPLHSLNDQYSVL
QPWCEDEVRAKLGQTIDPYASLCESNSYAKQFDECEGYQKGSGVQVEDVPTLHAQCVTEQ
RYLRGAMTRSLIYTQVSISGQALVFVVRTAGYSLMERAGTSTYLAFFFAQVGATLFGIFG
LGGFEKPRHQLEDCQFCDYSFHEPVDWFDSGIVPESGTESDFTASVIGCGGYVIVAWIWS
AIWYVLLDPIKWILFWILNEEGFRDTMSWRESTKRSLDRRSKDDIGDKEFTGPSGMVPAN
YSNPLGRASMSKPVSAVLDRKSASLVAINRNSMTVSQDPNRALNIGRRSMIGRPSGPVGR
TSMPLGRISRTSNTLSTGSKDGQIGRGSKPLNSSSAEIKPDKYDFASTIRE
>O81223
MGCSVSKKKKKNAMRPPGYEDPELLASVTPFTVEEVEALYELFKKLSSSIIDDGLIHKEE
FQLALFRNRNRRNLFADRIFDVFDVKRNGVIEFGEFVRSLGVFHPSAPVHEKVKFAFKLY
DLRQTGFIEREELKEMVVALLHESELVLSEDMIEVMVDKAFVQADRKNDGKIDIDEWKDF
VSLNPSLIKNMTLPYLKDINRTFPSFVSSCEEEEMELQNVSS
>Q8GZ17
MDSAPNFIPRLLLLSLLIVSIPLTSSQSDANTTNPSPSPPSDSDLCNGVFVSYTHTKGSK
IPPNDTANQPYRFESVITVLNHGRDELKSWRVFVKFAHREILVSASNAVLSDGSSLPVSV
ENGTVFAGYPSSDLKSAIQTAGDVTQMQARVELVGTQFGVAPPNVPLPKNITLATDGWKC
PKATQKGTNVLQVCCIPDPDYDNREIIDNEFLPRKDGDLTIMYDVVRSYSSNYMAQVTME
NHNPLGRLDNWKLSFDWMRDEFIYTMKGAYPSIVDSSDCVDGPQAKHYQDLDFSNVLSCA
RRPTVIDLPPTKYNDSTFGLIPFCCRNGTILPRSMDPSKSSSVFQMQVYKMPPDLNISAL
SPPQNWRINGTLNPDYKCGPPVRVSPSQFVDPSGLPSNRTAFASWQVVCNITQPKDASPR
CCVSFSAYFNDSIVPCKTCACGCSSNKAARACSATAPSLLLPQQALLVPFENRTELTVAW
AYLKQRPVPNPMPCGDNCGVSINWHLATDYRGGWTARVTVFNWGETDFVDWFTAVQMKNA
APGFEKAYSFNASTIGINGKNNTIFMEGLPGLNYLVAERDGENPLKNPRIPGKQQSVMSF
TKKLTPGINVPGGDGFPSKVFFNGEECSLPTILPMRSSQHRKHISVFLLALPVLALLILR
A
>Q9FMF5
MWESESDGGVGVGGGGGREYGDGVLSSNKHGGVKTDGFELRGQSWFVATDIPSDLLVKIG
DMNFHLHKYPLLSRSGKMNRLIYESRDPDPTILILDDLPGGPEAFELASKFCYGVPVDLT
ATNISGLRCAAEYLEMTEDLEEGNLIFKTEAFLSYVVLSSWRDSILVLKSCEKLSPWAEN

LQIVRRCSESIAWKACSNPKGIRWAYTGKAPSPSTTNFAGSSPRWNESKDSSFYCSPSRN
TNSQPVPPDWWFEDVSILRIDHFVRVITAIKVKGMRFELLGAVIMHYAGKWLPGLIKEGG
VAIAPAMSSAIGGGLGLGGDEMSISCGSNSSGGSSGPDWKGGLHMVLSAGKTNGHQDSVA
CLAGLGISPKDQRMIVESLISIIPPQKDSVTCSFLLRLLRAANMLKVAPALITELEKRVG
MQFEQATLQDLLIPGYNNKGETMYDVDLVQRLLEHFLVQEQTEGSSPSRMSPSPSQSMYA
DIPRGNNNNGGGGGGNNQNAKMRVARLVDSYLTEVARDRNLPLTKFQVLAEALPESARTC
DDGLYRAIDSYLKAHPTLSEHERKRLCRVMDCQKLSMDACMHAAQNERLPLRVVVQVLFS
EQVKISNALANTSLKESTTLGEAMGTYQPMIPNRKTLIEATPQSFQARWAAAKKDINTLK
FELETVKTKYVELQNEMEVMQRQFEKTGKVKNTPSSSAWTSGWKKLSKLTKMSGQESHDI
SSGGEQAGVDHPPPRKPRRWRNSIS
>Q6R3K9
MENERVEREQSQFQEDEFIDSRKPPPWRKQITVRAIVASLLIGIVYSVICLKLNLTTGLV
PNLNISSALLAFVFLKSWTKVLQKAGIATTPFTRQENTIAQTCAVACYSISLAGGFASYL
LGLNRRTYEETGVNTEGNNPRGIKEPGVGWMTSFLFVTSFIGLVVLVPLRKVMIIDYKLT
YPSGTATAVLINGFHTSKGDKTAKKQIRGFIKSFGLSFFWAFFGWFYSGGEKCGFSQFPT
FGLQALDKTFYFDFSMTYVGAGMICSHLVNLSLLFGAILSWGIMWPLIARLKGEWFPATL
KDNSMQGLNGYKVFICIALILGDGLYNFVKILFFTGRSFHSRLSKTNSISTLVEVPEDST
KESDNLKRENEVFVRESIPLWMACVGYLFFSLVSIIAIPLMFPQLKWYFVLVAYLLAPSL
SFCNAYGAGLTDMNMAYNYGKAALFVMAALAGKNDGVVAGMVACGLIKSIVSVSADLMHD
FKTGHLTQTSPRSMLVAQAIGTAIGCVVAPLTFFLFYKAFDVGNQNGEYKAPYAMIYRNM
AIIGVQGPSALPKHCLELCYGFFAFAVAANLARDLLPDKPGKWIPLPMAMAVPFLVGGSF
AIDMCIGSLVVYVWKKVNRKKADVMVPAVASGLICGDGLWILPSSLLALAKVRPPICMNF
TAAH
>Q9LVM5
MAMEIGEDEWKVCCGSSEFAKQMSTSGPLTSQEAIYTARDIWFNQVNVTDWLEAFSAHPQ
IGNTPSPSINSDFARRSVSEQSTAFATTSASALQELAEWNVLYKKKFGFIFIICASGRTH
AEMLHALKERYENRPIVELEIAAMEQMKITELRMAKLFSDKAKVISETDSSSSPVSTKPQ
DRLRIIGGHLNVAAEAKAPKRSRPPITTHVLDVSRGAPAAGVEVHLEVWSGTTGPSFVHG
GGGVWSSVGTSATDRDGRSGPLMDLVDALNPGTYRISFDTAKYSPGCFFPYVSIVFQVTE
SQKWEHFHVPLLLAPFSFSTYRGS
>Q9XFM6
MALELWQTLKEAIHAYTGLSPVVFFTALALAFAIYQVISGWFASPFDDVNRHQRARSLAQ
EEEPPIPQPVQVGEITEEELKQYDGSDPQKPLLMAIKHQIYDVTQSRMFYGPGGPYALFA
GKDASRALAKMSFEEKDLTWDVSGLGPFELDALQDWEYKFMSKYAKVGTVKVAGSEPETA
SVSEPTENVEQDAHVTTTPGKTVVDKSDDAPAETVLKKEE
>Q9T076
MTFLKMKSLSFFFTILLSLSTLFTISNARKFNVGGSGAWVTNPPENYESWSGKNRFLVHD
TLYFSYAKGADSVLEVNKADYDACNTKNPIKRVDDGDSEISLDRYGPFYFISGNEDNCKK
GQKLNVVVISARIPSTAQSPHAAAPGSSTPGSMTPPGGAHSPKSSSPVSPTTSPPGSTTP
PGGAHSPKSSSAVSPATSPPGSMAPKSGSPVSPTTSPPAPPKSTSPVSPSSAPMTSPPAP
MAPKSSSTIPPSSAPMTSPPGSMAPKSSSPVSNSPTVSPSLAPGGSTSSSPSDSPSGSAM
GPSGDGPSAAGDISTPAGAPGQKKSSANGMTVMSITTVLSLVLTIFLSA
>O50016
MIRFILLQNRQGKTRLAKYYVPLEDSEKHKVEYEVHRLVVNRDPKFTNFVEFRTHKVIYR

RYAGLFFSICVDITDNELAYLECIHLFVEILDHFFSNVCELDLVFNFHKVYRYLILDEFI
LAGELQETSKRQ
>Q8VYI9
MATHSSFTATTPLFLIVLLSLSSVSVLGASHHHATAPAPSVDCSTLILNMADCLSFVSSG
GTVAKPEGTCCSGLKTVLKADSQCLCEAFKSSASLGVTLNITKASTLPAACKLHAPSIAT
CGLSVAPSTAPGLAPGVAAAGPETAGFLAPNPSSGNDGSSLIPTSFTTVLSAVLFVLFFS
SA
>Q6NPN4
MKNPEKPLLLFLILASSLASMATAKSTIEPCSSKDTCNSLLGYTLYTDLKVTEVASLFQV
DPVSMLLSNSIDISYPDVENHVLPAKLFLKIPITCSCVDGIRKSLSTHYKTRTSDTLGSI
ADSVYGGLVSPEQIQVANSETDLSVLDVGTKLVIPLPCACFNGTDESLPALYLSYVVRGI
DTMAGIAKRFSTSVTDLTNVNAMGAPDINPGDILAVPLLACSSNFPKYATDYGLIIPNGS
YALTAGHCVQCSCVLGSRSMYCEPASISVSCSSMRCRNSNFMLGNITSQQSSSGCKLTTC
SYNGFASGTILTTLSMSLQPRCPGPQQLAPLIAPPDNVPKELMYLPSPSPSPSPEFDDIA
GGGSSIAAVPAASPGGATVSSSNSIPGNPANGPGGSISIASCPLSYYSFIALLIPIGSCF
FVF
>Q38890
MYGRDPWGGPLEINTADSATDDDRSRNLNDLDRAALSRPLDETQQSWLLGPTEQKKKKYV
DLGCIIVSRKIFVWTVGTLVAAALLAGFITLIVKTVPRHHPKTPPPDNYTIALHKALKFF
NAQKSGKLPKHNNVSWRGNSGLQDGKGETGSFYKDLVGGYYDAGDAIKFNFPMAYAMTML
SWSVIEYSAKYEAAGELTHVKELIKWGTDYFLKTFNSTADSIDDLVSQVGSGNTDDGNTD
PNDHYCWMRPEDMDYKRPVTTCNGGCSDLAAEMAAALASASIVFKDNKEYSKKLVHGAKV
VYQFGRTRRGRYSAGTAESSKFYNSSMYWDEFIWGGAWMYYATGNVTYLNLITQPTMAKH
AGAFWGGPYYGVFSWDNKLAGAQLLLSRLRLFLSPGYPYEEILRTFHNQTSIVMCSYLPI
FNKFNRTNGGLIELNHGAPQPLQYSVNAAFLATLYSDYLDAADTPGWYCGPNFYSTSVLR
DFARSQIDYILGKNPRKMSYVVGFGTKYPRHVHHRGASIPKNKVKYNCKGGWKWRDSKKP
NPNTIEGAMVAGPDKRDGYRDVRMNYNYTEPTLAGNAGLVAALVALSGEEEATGKIDKNT
IFSAVPPLFPTPPPPPAPWKP
>Q8VYM2
MAEQQLGVLKALDVAKTQLYHFTAIVIAGMGFFTDAYDLFCVSLVTKLLGRIYYFNPESA
KPGSLPPHVAAAVNGVALCGTLSGQLFFGWLGDKLGRKKVYGLTLVMMILCSVASGLSFG
HEAKGVMTTLCFFRFWLGFGIGGDYPLSATIMSEYANKKTRGAFIAAVFAMQGVGILAGG
FVALAVSSIFDKKFPAPTYAVNRALSTPPQVDYIWRIIVMFGALPAALTYYWRMKMPETA
RYTALVAKNIKQATADMSKVLQTDIELEERVEDDVKDPKQNYGLFSKEFLRRHGLHLLGT
TSTWFLLDIAFYSQNLFQKDIFSAIGWIPKAATMNATHEVFRIARAQTLIALCSTVPGYW
FTVAFIDTIGRFKIQLNGFFMMTVFMFAIAFPYNHWIKPENRIGFVVMYSLTFFFANFGP
NATTFIVPAEIFPARLRSTCHGISAAAGKAGAIVGAFGFLYAAQSQDKAKVDAGYPPGIG
VKNSLIMLGVLNFIGMLFTFLVPEPKGKSLEELSGEAEVSHDEK
>Q9FHD4
METTKKLFALLCLFVTMNQAISVSDPDDMETFCMKSSRNTTSNTTYNKNLNTLLSTLSNQ
SSFANYYNLTTGLASDTVHGMFLCTGDVNRTTCNACVKNATIEIAKNCTNHREAIIYNVD
CMVRYSDKFFLTTLETNPSYWWSSNDLIPKSFGKFGQRLSDKMGEVIVRSSLLSSSFTPY
YLMDTTRFDNLYDLESIVQCTPDLDPRNCTTCLKLALQELTECCGNQVWAFIYTPNCMVS
FDTYNSSLPPLPPPSRSGSFSHRGNNKLLGGMVLAVSVSVFAFLSLV

>Q9LW07
MKKKTWFLNFSLFFLQIFTSSNALDVTQFGAVGDGVTDDSQAFLKAWEAVCSGTGDGQFV
VPAGMTFMLQPLKFQGSCKSTPVFVQMLGKLVAPSKGNWKGDKDQWILFTDIEGLVIEGD
GEINGQGSSWWEHKGSRPTALKFRSCNNLRLSGLTHLDSPMAHIHISECNYVTISSLRIN
APESSPNTDGIDVGASSNVVIQDCIIATGDDCIAINSGTSNIHISGIDCGPGHGISIGSL
GKDGETATVENVCVQNCNFRGTMNGARIKTWQGGSGYARMITFNGITLDNVENPIIIDQF
YNGGDSDNAKDRKSSAVEVSKVVFSNFIGTSKSEYGVDFRCSERVPCTEIFLRDMKIETA
SSGSGQVAQGQCLNVRGASTIAVPGLECLELSTDMFSSAQLLEQTCMSAQSVQPRTTTQP
MQDPIWVFQSRGKQLRVYNIAILVSFISLVTYILAR
>Q8GUQ5
MKAHKTVFNQHPLSLNKLFFVLLLIFFLPPASPAASVNGLYKDSQQLLSFKAALPPTPTL
LQNWLSSTGPCSFTGVSCKNSRVSSIDLSNTFLSVDFSLVTSYLLPLSNLESLVLKNANL
SGSLTSAAKSQCGVTLDSIDLAENTISGPISDISSFGVCSNLKSLNLSKNFLDPPGKEML
KAATFSLQVLDLSYNNISGFNLFPWVSSMGFVELEFFSLKGNKLAGSIPELDFKNLSYLD
LSANNFSTVFPSFKDCSNLQHLDLSSNKFYGDIGSSLSSCGKLSFLNLTNNQFVGLVPKL
PSESLQYLYLRGNDFQGVYPNQLADLCKTVVELDLSYNNFSGMVPESLGECSSLELVDIS
YNNFSGKLPVDTLSKLSNIKTMVLSFNKFVGGLPDSFSNLLKLETLDMSSNNLTGVIPSG
ICKDPMNNLKVLYLQNNLFKGPIPDSLSNCSQLVSLDLSFNYLTGSIPSSLGSLSKLKDL
ILWLNQLSGEIPQELMYLQALENLILDFNDLTGPIPASLSNCTKLNWISLSNNQLSGEIP
ASLGRLSNLAILKLGNNSISGNIPAELGNCQSLIWLDLNTNFLNGSIPPPLFKQSGNIAV
ALLTGKRYVYIKNDGSKECHGAGNLLEFGGIRQEQLDRISTRHPCNFTRVYRGITQPTFN
HNGSMIFLDLSYNKLEGSIPKELGAMYYLSILNLGHNDLSGMIPQQLGGLKNVAILDLSY
NRFNGTIPNSLTSLTLLGEIDLSNNNLSGMIPESAPFDTFPDYRFANNSLCGYPLPIPCS
SGPKSDANQHQKSHRRQASLAGSVAMGLLFSLFCIFGLIIVAIETKKRRRKKEAALEAYM
DGHSHSATANSAWKFTSAREALSINLAAFEKPLRKLTFADLLEATNGFHNDSLVGSGGFG
DVYKAQLKDGSVVAIKKLIHVSGQGDREFTAEMETIGKIKHRNLVPLLGYCKVGEERLLV
YEYMKYGSLEDVLHDRKKIGIKLNWPARRKIAIGAARGLAFLHHNCIPHIIHRDMKSSNV
LLDENLEARVSDFGMARLMSAMDTHLSVSTLAGTPGYVPPEYYQSFRCSTKGDVYSYGVV
LLELLTGKQPTDSADFGDNNLVGWVKLHAKGKITDVFDRELLKEDASIEIELLQHLKVAC
ACLDDRHWKRPTMIQVMAMFKEIQAGSGMDSTSTIGADDVNFSGVEGGIEMGINGSIKEG
NELSKHL
>Q94BT2
MASSSSSLLILAVACFVSLISPAISQQACKSQNLNSAGPFDSCEDLPVLNSYLHYTYNSS
NSSLSVAFVATPSQANGGWVAWAINPTGTKMAGSQAFLAYRSGGGAAPVVKTYNISSYSS
LVEGKLAFDFWNLRAESLSGGRIAIFTTVKVPAGADSVNQVWQIGGNVTNGRPGVHPFGP
DNLGSHRVLSFTEDAAPGSAPSPGSAPAPGTSGSTTPGTAAGGPGNAGSLTRNVNFGVNL
GILVLLGSIFIF
>Q84TI7
MDRVVAKIAKIRSQLTKLRSLFFLYFIYFLFFSFLGFLALKITKPRTTSRPHDFDLFFTS
VSAITVSSMSTVDMEVFSNTQLIFLTILMFLGGEIFTSFLNLYVSYFTKFVFPHNKIRHI
LGSYNSDSSIEDRCDVETVTDYREGLIKIDERASKCLYSVVLSYHLVTNLVGSVLLLVYV
NFVKTARDVLSSKEISPLTFSVFTTVSTFANCGFVPTNENMIIFRKNSGLIWLLIPQVLM
GNTLFPCFLVLLIWGLYKITKRDEYGYILKNHNKMGYSHLLSVRLCVLLGVTVLGFLIIQ
LLFFCAFEWTSESLEGMSSYEKLVGSLFQVVNSRHTGETIVDLSTLSPAILVLFILMMYL

PPYTLFMPLTEQKTIEKEGGDDDSENGKKVKKSGLIVSQLSFLTICIFLISITERQNLQR
DPINFNVLNITLEVISAYGNVGFTTGYSCERRVDISDGGCKDASYGFAGRWSPMGKFVLI
IVMFYGRFKQFTAKSGRAWILYPSSS
>Q948U0
MRGLPGHERRWTSDTVSSGKDLSGESSPGTDSGNISGFASEEFVEVILDLQDDDTIILRS
VEPATVINIDASDPATGVGIGGVSIETPASLTSTSGTRSPTMRRSTSNKLRQFSQELKAE
AVAKAKHFSQELKAELRRFSWSHGHASRTFSPASFFQNAVVGTGNGVDSALAARALRRQR
AQLDRTRSSAHKALRGLKFISNNKTNGWNEVENNFAKLAKDGYLYRSDFAQCIGMKDSKE
FALELFDALSRRRRLKVDKISKEELYEYWSQITDQSFDSRLQIFFDMVDKNEDGRIGEEE
VKEIIMLSASANKLSRLKEQAEEYAALIMEELDPERLGYIELWQLETLLLQKDTYLNYSQ
ALSYTSQALSQNLQGLRKRSPIRRMSTKLVYSLQENWKRIWVLVLWILIMIGLFLWKFYL
YKQKSAFQVMGYCLLTAKGAAETLKFNMALILLPVCRNTITFLRSTKLSCFVPFDDNINF
HKTVAAAIVTGIILHAGNHLVCDFPKLIHANNTNYQKYLVNDFGPSQPQYIDLVKGVEGV
TGIIMVILMAIAFTLATRWFRRSLIKFPKPFDRLTGFNAFWYSHHLLIIVYIVLIIHGTF
LYLVHNWYSKTTWMYLAVPVLLYAGERTLRFFRSGLYTVRLLKVAIYPGNVLTLQMSKPP
QFRYKSGQYMFVQCPAVSPFEWHPFSITSAPGDDYLSIHIRQLGDWTQELKRVFSEACEQ
PEAGKSGLLRADENTKTSLPKLLIDGPYGAPAQDYRKYDVLLLVGLGIGATPFISILKDL
LKNIVTMEEQADLVSDFSGNSDMSAATSEQPALNKISPKKRKSTLKTTNAYFYWVTREQG
SFDWFKGVMNEVAELDQRGVIEMHNYLTSVYEEGDARSALITMVQALNHAKNGVDIVSGT
SVRTHFARPNWRKVFSKTLTKHANARIGVFYCGAPILAKELSKLCKEFNQKGTTKFEFHK
EHF
>Q9SVG4
MLTTPPRTFVSVPFFFFFLLFLSLPLSSFSQSNSVYNSFLKCFSDKTKSPQSQITDNVFS
QTNPAFSSVLRAYIRNARFNTSSTLKPTIIITPRSESHVSAAVTCSKTLNFLLKIRSGGH
DYDGLSYISDKPFFILDMSNIRDVSVDIASNSAWISAGATLGEVYYRIWEKSRVHGFPAG
VCPTVGVGGHLSGGGYGNMVRKFGLSVDYVEDAKIVDVNGRVLDRKAMGEDLFWAITGGG
GGSYGVVLGYKVKLVPVPSVVTVFRVEQYMDSGAVDMVHKWQSVGPKTDPNLFMRMLIQP
VTRKKVKTVRASVVALFLGRADEVVALLSKEFPELGLKKENCSEMTWFQSALWWDNRLNA
TQVDPKVFLDRNLDTSSFGKRKSDYVATAIPKKGIESLFKKMIELGKIGLVFNPYGGKMA
EVAVNAKPFPHRNKLFKIQYSVNWKENSAEIEKGYLNQAKVLYSFMTGFVSKNPRSSYFN
YRDVDIGVNDHGANSYKEGEVYGRKYFGENFDRLVKIKTAVDPGNFFRNEQSIPTLKNEK
GMLLPEPGKARRWSRVGGATVVATVVLHVF
>P83326
MAFSYCSSSLFVSLLLVILFISPLSQRPSVKAENHLISEICPKTRNPSLCLQALESDPRS
ASKDLKGLGQFSIDIAQASAKQTSKIIASLTNQATDPKLKGRYETCSENYADAIDSLGQA
KQFLTSGDYNSLNIYASAAFDGAGTCEDSFEGPPNIPTQLHQADLKLEDLCDIVLVISNL
LPGSK
>Q9SV84
MAPPEAEVGAVMVMAPPTPGTPGTPGGPLITGMRVDSMSFDHRKPTPRCKCLPVMGSTWG
QHDTCFTDFPSPDVSLTRKLGAEFVGTFILIFTATAGPIVNQKYDGAETLIGNAACAGLA
VMIIILSTGHISGAHLNPSLTIAFAALRHFPWAHVPAYIAAQVSASICASFALKGVFHPF
MSGGVTIPSVSLGQAFALEFIITFILLFVVTAVATDTRAVGELAGIAVGATVMLNILVAG
PSTGGSMNPVRTLGPAVASGNYRSLWVYLVAPTLGAISGAAVYTGVKLNDSVTDPPRPVR
SFRR

>Q3E7D0
MSSSDPKPGPKPGPWPPTPESAAMPPSSWAKKTGFRPKFSGETTATDSSSGQLSLPVRAK
QQETQPDLEAGQTRLRPPPPVSAAVTNGETDKDKKEKPPPPPPGSVAVPVKDQPVKRRRD
SDGVVGRSNGPDGANGSGDPVRRPGRIEETVEVLPQSMDDDLVARNLHMKYGLRDTPGLV
PIGFYGLQHYLSMLGSLILVPLVIVPAMGGSHEEVANVVSTVLFVSGITTLLHTSFGSRL
PLIQGPSFVFLAPALAIINSPEFQGLNGNNNFKHIMRELQGAIIIGSAFQAVLGYSGLMS
LILRLVNPVVVAPTVAAVGLSFYSYGFPLVGKCLEIGVVQILLVIIFALYLRKISVLSHR
IFLIYAVPLSLAITWAAAFLLTETGAYTYKGCDPNVPVSNVVSTHCRKYMTRMKYCRVDT
SHALSSAPWFRFPYPLQWGVPLFNWKMAFVMCVVSVIASVDSVGSYHASSLLVASRPPTR
GVVSRAIGLEGFTSVLAGLWGTGTGSTTLTENVHTIAVTKMGSRRVVELGACVLVIFSLV
GKVGGFLASIPQVMVASLLCFMWAMFTALGLSNLRYSEAGSSRNIIIVGLSLFFSLSVPA
YFQQYGISPNSNLSVPSYYQPYIVSSHGPFKSQYKGMNYVMNTLLSMSMVIAFIMAVILD
NTVPGSKQERGVYVWSDSETATREPALAKDYELPFRVGRFFRWVKWVGI
>Q9XIE2
MDYNPNLPPLGGGGVSMRRSISRSVSRASRNIEDIFSSGSRRTQSVNDDEEALKWAAIEK
LPTYSRLRTTLMNAVVEDDVYGNQLMSKEVDVTKLDGEDRQKFIDMVFKVAEQDNERILT
KLRNRIDRVGIKLPTVEVRYEHLTIKADCYTGNRSLPTLLNVVRNMGESALGMIGIQFAK
KAQLTILKDISGVIKPGRMTLLLGPPSSGKTTLLLALAGKLDKSLQVSGDITYNGYQLDE
FVPRKTSAYISQNDLHVGIMTVKETLDFSARCQGVGTRYDLLNELARREKDAGIFPEADV
DLFMKASAAQGVKNSLVTDYTLKILGLDICKDTIVGDDMMRGISGGQKKRVTTGEMIVGP
TKTLFMDEISTGLDSSTTFQIVKCLQQIVHLNEATVLMSLLQPAPETFDLFDDIILVSEG
QIVYQGPRDNILEFFESFGFKCPERKGTADFLQEVTSKKDQEQYWVNPNRPYHYIPVSEF
ASRYKSFHVGTKMSNELAVPFDKSRGHKAALVFDKYSVSKRELLKSCWDKEWLLMQRNAF
FYVFKTVQIVIIAAITSTLFLRTEMNTRNEGDANLYIGALLFGMIINMFNGFAEMAMMVS
RLPVFYKQRDLLFYPSWTFSLPTFLLGIPSSILESTAWMVVTYYSIGFAPDASRFFKQFL
LVFLIQQMAASLFRLIASVCRTMMIANTGGALTLLLVFLLGGFLLPKGKIPDWWGWAYWV
SPLTYAFNGLVVNEMFAPRWMNKMASSNSTIKLGTMVLNTWDVYHQKNWYWISVGALLCF
TALFNILFTLALTYLNPLGKKAGLLPEEENEDADQGKDPMRRSLSTADGNRRGEVAMGRM
SRDSAAEASGGAGNKKGMVLPFTPLAMSFDDVKYFVDMPGEMRDQGVTETRLQLLKGVTG
AFRPGVLTALMGVSGAGKTTLMDVLAGRKTGGYIEGDVRISGFPKVQETFARISGYCEQT
DIHSPQVTVRESLIFSAFLRLPKEVGKDEKMMFVDQVMELVELDSLRDSIVGLPGVTGLS
TEQRKRLTIAVELVANPSIIFMDEPTSGLDARAAAIVMRAVRNTVDTGRTVVCTIHQPSI
DIFEAFDELMLMKRGGQVIYAGPLGQNSHKVVEYFESFPGVSKIPEKYNPATWMLEASSL
AAELKLSVDFAELYNQSALHQRNKALVKELSVPPAGASDLYFATQFSQNTWGQFKSCLWK
QWWTYWRSPDYNLVRFIFTLATSLLIGTVFWQIGGNRSNAGDLTMVIGALYAAIIFVGIN
NCSTVQPMVAVERTVFYRERAAGMYSAMPYAISQVTCELPYVLIQTVYYSLIVYAMVGFE
WKAEKFFWFVFVSYFSFLYWTYYGMMTVSLTPNQQVASIFASAFYGIFNLFSGFFIPRPK
IPKWWIWYYWICPVAWTVYGLIVSQYGDVETRIQVLGGAPDLTVKQYIEDHYGFQSDFMG
PVAAVLIAFTVFFAFIFAFCIRTLNFQTR
>Q94CD8
MLLPRWFAEALLLLLSILACSNAAFIGVNIGTDLTNMPPPSDIVTLLKSQQITHVRLYDA
NSHMLKAFANTSIEVMVGVTNEEILKIGRFPSAAAAWVNKNVAAYIPSTNITAIAVGSEV
LTTIPHVAPILASALNNIHKALVASNLNFKVKVSSPMSMDIMPKPFPPSTSTFSPSWNTT
VYQLLQFLKNTGSFFMLNAYPYYGYTTANGIFPLDYALFKQLSPVKQIVDPNTLLHYNSM

FDAMVDAAYYSMEALNFSKIPVVVTETGWPSSGGSDEAAATVANAETFNTNLIKRVLNNS
GPPSQPDIPINTYIYELYNEDKRSGPVSERNWGILFPNGTSVYPLSLSGGSSSAALNGSS
MFCVAKADADDDKLVDGLNWACGQGRANCAAIQPGQPCYLPNDVKSHASFAFNDYYQKMK
SAGGTCDFDGTAITTTRDPSYRTCAYTGSLNANATNGNFPPDALGPASPLGGNANARIIF
SYHLPILAPLALTLLQLLLQHDRLL
>Q9LKW9
MTTVIDATMAYRFLEEATDSSSSSSSSKLESSPVDAVLFVGMSLVLGIASRHLLRGTRVP
YTVALLVIGIALGSLEYGAKHNLGKIGHGIRIWNEIDPELLLAVFLPALLFESSFSMEVH
QIKRCLGQMVLLAVPGVLISTACLGSLVKVTFPYEWDWKTSLLLGGLLSATDPVAVVALL
KELGASKKLSTIIEGESLMNDGTAIVVFQLFLKMAMGQNSDWSSIIKFLLKVALGAVGIG
LAFGIASVIWLKFIFNDTVIEITLTIAVSYFAYYTAQEWAGASGVLTVMTLGMFYAAFAR
TAFKGDSQKSLHHFWEMVAYIANTLIFILSGVVIAEGILDSDKIAYQGNSWRFLFLLYVY
IQLSRVVVVGVLYPLLCRFGYGLDWKESIILVWSGLRGAVALALSLSVKQSSGNSHISKE
TGTLFLFFTGGIVFLTLIVNGSTTQFVLRLLRMDILPAPKKRILEYTKYEMLNKALRAFQ
DLGDDEELGPADWPTVESYISSLKGSEGELVHHPHNGSKIGSLDPKSLKDIRMRFLNGVQ
ATYWEMLDEGRISEVTANILMQSVDEALDQVSTTLCDWRGLKPHVNFPNYYNFLHSKVVP
RKLVTYFAVERLESACYISAAFLRAHTIARQQLYDFLGESNIGSIVINESEKEGEEAKKF
LEKVRSSFPQVLRVVKTKQVTYSVLNHLLGYIENLEKVGLLEEKEIAHLHDAVQTGLKKL
LRNPPIVKLPKLSDMITSHPLSVALPPAFCEPLKHSKKEPMKLRGVTLYKEGSKPTGVWL
IFDGIVKWKSKILSNNHSLHPTFSHGSTLGLYEVLTGKPYLCDLITDSMVLCFFIDSEKI
LSLQSDSTIDDFLWQESALVLLKLLRPQIFESVAMQELRALVSTESSKLTTYVTGESIEI
DCNSIGLLLEGFVKPVGIKEELISSPAALSPSNGNQSFHNSSEASGIMRVSFSQQATQYI
VETRARAIIFNIGAFGADRTLHRRPSSLTPPRSSSSDQLQRSFRKEHRGLMSWPENIYAK
QQQEINKTTLSLSERAMQLSIFGSMVNVYRRSVSFGGIYNNKLQDNLLYKKLPLNPAQGL
VSAKSESSIVTKKQLETRKHACQLPLKGESSTRQNTMVESSDEEDEDEGIVVRIDSPSKI
VFRNDL
>Q94F08
MAKTNQAITICSLLLLLLLSETTSHLLCSDSKTPVNNNETLQFCDSYKERSCCNSKDDLQ
LQNRFNSMNISDSNCSSLLKSILCSKCDEFSGQLFGDDDSSLVPILCNSTSQDLCSKLWD
SCQNISIVSSPFSPTLLGGATSPSTSSNSSTLTDLWKSQTEFCTAFGGPSQTNNNKTKCF
NGEPVNRDTSDDDEDDVKTPKGICLEKIGTGSYLNMVAHPDGSNRAFFSNQPGKIWLGTI
PDQDSGKPMEIDESTPFVDITDQVSFDTQFGMMGMAFHPKFAENGRFFASFNCDKVKSPG
CSGRCACNSDVNCDPSKLPKDDGTTPCRYQTVVSEYTANGTSSSPSTAKIGKASEVRRIF
TMGLPYSSSHGGQILFGPDGYLYLMTGDGGGVSDTHNFAQNKKSLLGKILRLDVDVMPSV
SEISKLGLWGNYSIPKNNPFQGNENEQPEIWALGLRNPWRCSFDSERPDYFLCADVGKDT
YEEVDIITMGGNYGWRTYEGPYVFSPLSPFGENVSSDSNLTFPILGYNHSEVNKHEGSAS
IIGGYFYRSNTDPCSYGTYLYADLYANAMWAAIESPEDSGNFTDSLIPFSCSKDSPMKCT
AAPGGASSGPALGYIYSFGQDNNKDIHLLTSSGVYRIVRPSRCNLACSKENTTASAGKQN
PAGSAPPQPLPSSARKLCFSVFLLLSLLMMFLTLLD
>Q93XX5
MSACINGLCRAVTVSLLLLLLSFSFSSACSNGNCQLLDSCSSATDCVSGLYCGDCPAVGR
SKPVCTRGQATSPTSIINGLPFNKYTWLMTHNAFSNANAPLLPGVERITFYNQEDTITNQ
LQNGVRGLMLDMYDFNNDIWLCHSLRGQCFNFTAFQPAINILREVEAFLSQNPTEIVTII
IEDYVHRPKGLSTLFANAGLDKYWFPVSKMPRKGEDWPTVTDMVQENHRLLVFTSVAAKE

DEEGVAYQWRYMVENESGDPGVKRGSCPNRKESQPLNSKSSSLFLMNYFPTYPVEKDACK
EHSAPLAEMVGTCLKSGGNRMPNFLAVNFYMRSDGGGVFEILDRMNGPVLCGCETLSACQ
PGAAYGSCKNVTVQTRTPSMDSTAGSNSGGSYSGSVQFSRSLASVAQLNNIVVFCFSLLP
LLIFLL
>O23492
MVEGGIAKADKTEFTECWRTTWKTPYIMRLALSAGIGGLLFGYDTGVISGALLFIKEDFD
EVDKKTWLQSTIVSMAVAGAIVGAAVGGWINDKFGRRMSILIADVLFLIGAIVMAFAPAP
WVIIVGRIFVGFGVGMASMTSPLYISEASPARIRGALVSTNGLLITGGQFFSYLINLAFV
HTPGTWRWMLGVAGVPAIVQFVLMLSLPESPRWLYRKDRIAESRAILERIYPADEVEAEM
EALKLSVEAEKADEAIIGDSFSAKLKGAFGNPVVRRGLAAGITVQVAQQFVGINTVMYYS
PSIVQFAGYASNKTAMALSLITSGLNALGSIVSMMFVDRYGRRKLMIISMFGIIACLIIL
ATVFSQAAIHAPKIDAFESRTFAPNATCSAYAPLAAENAPPSRWNCMKCLRSECGFCASG
VQPYAPGACVVLSDDMKATCSSRGRTFFKDGCPSKFGFLAIVFLGLYIVVYAPGMGTVPW
IVNSEIYPLRYRGLGGGIAAVSNWVSNLIVSESFLSLTHALGSSGTFLLFAGFSTIGLFF
IWLLVPETKGLQFEEVEKLLEVGFKPSLLRRREKKGKEVDAA
>Q06611
MEGKEEDVRVGANKFPERQPIGTSAQSDKDYKEPPPAPLFEPGELASWSFWRAGIAEFIA
TFLFLYITVLTVMGVKRSPNMCASVGIQGIAWAFGGMIFALVYCTAGISGGHINPAVTFG
LFLARKLSLTRAVYYIVMQCLGAICGAGVVKGFQPKQYQALGGGANTIAHGYTKGSGLGA
EIIGTFVLVYTVFSATDAKRNARDSHVPILAPLPIGFAVFLVHLATIPITGTGINPARSL
GAAIIFNKDNAWDDHWVFWVGPFIGAALAALYHVIVIRAIPFKSRS
>Q9FM65
MAKKMSSLIIIFNILLLLTTQTHAHNVTRLLANHPSFSSFSHFLTQTHLADEINRRRTIT
VCAVDNAAMSALTSKGYTLSTLKNILSLHVLLDYFGTKKLHQIRDGSALAATLFQATGAA
PGTSGFVNITDLRGGKVGFGPDGGDLSSFFVKSIEEVPYNISIIQISRVLPSETAAAPTP
APAEMNLTGIMSAHGCKVFAETLLTNPGASKTYQESLEGGMTVFCPGDDAMKGFLPKYKN
LTAPKKEAFLDFLAVPTYYSMAMLKSNNGPMNTLATDGANKFELTVQNDGEKVTLKTRIN
TVKIVDTLIDEQPLAIYATDKVLLPKELFKASAVEAPAPAPAPEDGDVADSPKAAKGKAK
GKKKKAAPSPDNDPFGDSDSPAEGPDGEADDATADDAGAVRIIGGAKAGLVVSLLCLFAS
SWLL
>Q9LQU4
MEAQHLHAKPHAEGEWSTGFCDCFSDCKNCCITFWCPCITFGQVAEIVDRGSTSCGTAGA
LYALIAVVTGCACIYSCFYRGKMRAQYNIKGDDCTDCLKHFCCELCSLTQQYRELKHRGY
DMSLGWAGNVERQQNQGGVAMGAPVFQGGMTR
>Q944C1
MEGKKEMGSYKFCLIFTRKFRMTESGPVEDVRDLFEKYTEGDAHMSPEQLQKLMTEEGGE
GETSLEEAERIVDEVLRRKHHIAKFTRRNLTLDDFNYYLFSTDLNPPIADQVHQNMDAPL
SHYFIFTGHNSYLTGNQLSSNCSELPIADALRRGVRVVELDLWPRGTDDVCVKHGRTLTK
EVKLGKCLESIKANAFAISKYPVIITLEDHLTPKLQFKVAKMITQTFGDMLYYHDSQGCQ
EFPSPEELKEKILISTKPPKEYLEANDTKEKDNGEKGKDSDEDVWGKEPEDLISTQSDLD
KVTSSVNDLNQDDEERGSCESDTSCQLQAPEYKRLIAIHAGKPKGGLRMALKVDPNKIRR
LSLSEQLLEKAVASYGADVIRFTQKNFLRIYPKGTRFNSSNYKPQIGWMSGAQMIAFNMQ
GYGRALWLMEGMFRANGGCGYVKKPDFLMDASPNGQDFYPKDNSSPKKTLKVKVCMGDGW
LLDFKKTHFDSYSPPDFFVRVGIAGAPVDEVMEKTKIEYDTWTPIWNKEFTFPLAVPELA

LLRVEVHEHDVNEKDDFGGQTCLPVSEIRQGIRAVPLFNRKGVKYSSTRLLMRFEFV
>Q9FHN6
MAATDFFFAFVFSFALIFGFSFAGDPYVSYDFTLSYITASPLGVPQQVIAVNGKFPGPVI
NATTNYNVHVNVLNHLDEPLLLTWPGVQMRRNSWQDGVLGTNCPIPPNWNFTYDFQLKDQ
IGSYFYSPSLNFQRASGGFGALIINNRDLVPIPFTEPDGEIIFIIGDWYTQNHTALRRIL
DSGKELGMPDGVLINGKGPFKYNSSVPDGIEHETVNVDPGKTYRIRVHNVGISTSLNFRI
QNHKLLLIETEGRYTSQMNFTDFDVHVGQSYSFLVTMDQNATSDYYIVASARFVNETVWQ
RVTGVGILHYSNSKGPASGPLPVSATDVNHPWSAMNQPRAIKQNTSASGARPNPQGSFHY
GQINITRTYILRSLPPTKINGKLRATLNGISFVNPSTPMRLADDHKVKGDYMLDFPDRPL
DEKLPRLSSSIINATYKGFIQVIFQNNDTKIQSFHIDGYAFYVVAMDFGIWSEDRNSSYN
NWDAVARSTVEVYPGAWTAVLISLDNVGVWNIRVENLDRWYLGQETYMRIINPEENGSTE
MDPPENVMYCGALQAMQKEQHHSSATKSMTNGQLILIFSMMMVLLSSFSSFC
>P59833
MAISKLHLLFLLSVFLSLHPLVLSDTADEEDVLLTGINSYRASLNLTTLIHNHNAECLAD
EIADQFKNQPCTNTTGSASVPGTTPGFPNLPNLLSKCRLNPTVTRDGAILPACVPNLDPS
LVLTNFTMSQYSKDLNDSKFTGIGIGSDDNWIVVVLTTSTPEGSYSPASNSGAFAFGVNG
LVSSSLMFLLFCFFMF
>Q9LF79
MTSLLKSSPGRRRGGDVESGKSEHADSDSDTFYIPSKNASIERLQQWRKAALVLNASRRF
RYTLDLKKEQETREMRQKIRSHAHALLAANRFMDMGRESGVEKTTGPATPAGDFGITPEQ
LVIMSKDHNSGALEQYGGTQGLANLLKTNPEKGISGDDDDLLKRKTIYGSNTYPRKKGKG
FLRFLWDACHDLTLIILMVAAVASLALGIKTEGIKEGWYDGGSIAFAVILVIVVTAVSDY
KQSLQFQNLNDEKRNIHLEVLRGGRRVEISIYDIVVGDVIPLNIGNQVPADGVLISGHSL
ALDESSMTGESKIVNKDANKDPFLMSGCKVADGNGSMLVTGVGVNTEWGLLMASISEDNG
EETPLQVRLNGVATFIGSIGLAVAAAVLVILLTRYFTGHTKDNNGGPQFVKGKTKVGHVI
DDVVKVLTVAVTIVVVAVPEGLPLAVTLTLAYSMRKMMADKALVRRLSACETMGSATTIC
SDKTGTLTLNQMTVVESYAGGKKTDTEQLPATITSLVVEGISQNTTGSIFVPEGGGDLEY
SGSPTEKAILGWGVKLGMNFETARSQSSILHAFPFNSEKKRGGVAVKTADGEVHVHWKGA
SEIVLASCRSYIDEDGNVAPMTDDKASFFKNGINDMAGRTLRCVALAFRTYEAEKVPTGE
ELSKWVLPEDDLILLAIVGIKDPCRPGVKDSVVLCQNAGVKVRMVTGDNVQTARAIALEC
GILSSDADLSEPTLIEGKSFREMTDAERDKISDKISVMGRSSPNDKLLLVQSLRRQGHVV
AVTGDGTNDAPALHEADIGLAMGIAGTEVAKESSDIIILDDNFASVVKVVRWGRSVYANI
QKFIQFQLTVNVAALVINVVAAISSGDVPLTAVQLLWVNLIMDTLGALALATEPPTDHLM
GRPPVGRKEPLITNIMWRNLLIQAIYQVSVLLTLNFRGISILGLEHEVHEHATRVKNTII
FNAFVLCQAFNEFNARKPDEKNIFKGVIKNRLFMGIIVITLVLQVIIVEFLGKFASTTKL
NWKQWLICVGIGVISWPLALVGKFIPVPAAPISNKLKVLKFWGKKKNSSGEGSL
>Q9SUC9
MKKSLTLLILLLCSLLFSTVLSNLLVEPVQPNTVPAFPVETQAQSCRLDLSNELFGGVNE
ACGRNLDRSRCCPVLAAWLFAAHARSALQLPAPAPTPESSDPDEPMKPDDSQKCVNTLQS
ALLTKQIKIPQPNSSCDAILCFCGIRLHQISSLSCPAAFNVSSGFKNATPTAAVKNLEKE
CRNSSYSGCTRCLGALQKLKVRGGNKKTTTERGTKMMSKDCQLMGLTWLLARNKTAYIPT
VSAVLRAIMYSPHPPHLNKCSPDQENMPLAVDSLQFQKSFSSSSHLFGVLPFLPLVLCIF
LFLL
>O80725

MASESGLNGDPNILEEVSETKRDKEEEEEVKKTEKKDEEHEKTKTVPFYKLFAFADSFDF
LLMILGTLGSIGNGLGFPLMTLLFGDLIDAFGENQTNTTDKVSKVALKFVWLGIGTFAAA
FLQLSGWMISGERQAARIRSLYLKTILRQDIAFFDIDTNTGEVVGRMSGDTVLIQDAMGE
KVGKAIQLLATFVGGFVIAFVRGWLLTLVMLSSIPLLVMAGALLAIVIAKTASRGQTAYA
KAATVVEQTIGSIRTVASFTGEKQAISNYNKHLVTAYKAGVIEGGSTGLGLGTLFLVVFC
SYALAVWYGGKLILDKGYTGGQVLNIIIAVLTGSMSLGQTSPCLSAFAAGQAAAYKMFET
IERRPNIDSYSTNGKVLDDIKGDIELKDVYFTYPARPDEQIFRGFSLFISSGTTVALVGQ
SGSGKSTVVSLIERFYDPQAGDVLIDGINLKEFQLKWIRSKIGLVSQEPVLFTASIKDNI
AYGKEDATTEEIKAAAELANASKFVDKLPQGLDTMVGEHGTQLSGGQKQRIAVARAILKD
PRILLLDEATSALDAESERVVQEALDRIMVNRTTVVVAHRLSTVRNADMIAVIHQGKIVE
KGSHTELLKDPEGAYSQLIRLQEEKKSDENAAEEQKMSSIESFKQSSLRKSSLGRSLSKG
GSSRGNSSRHSFNMFGFPAGIDGNVVQDQEEDDTTQPKTEPKKVSIFRIAALNKPEIPVL
ILGSISAAANGVILPIFGILISSVIKAFFQPPKKLKEDTSFWAIIFMVLGFASIIAYPAQ
TFFFAIAGCKLVQRIRSMCFEKVVHMEVGWFDEPENSSGTIGARLSADAATIRGLVGDSL
AQTVQNLSSILAGLIIAFLACWQLAFVVLAMLPLIALNGFLYMKFMKGFSADAKKMYGEA
SQVANDAVGSIRTVASFCAEDKVMNMYSKKCEGPMKNGIRQGIVSGIGFGFSFFVLFSSY
AASFYVGARLVDDGKTTFDSVFRVFFALTMAAMAISQSSSLSPDSSKADVAAASIFAIMD
RESKIDPSVESGRVLDNVKGDIELRHVSFKYPARPDVQIFQDLCLSIRAGKTVALVGESG
SGKSTVIALLQRFYDPDSGEITLDGVEIKSLRLKWLRQQTGLVSQEPILFNETIRANIAY
GKGGDASESEIVSSAELSNAHGFISGLQQGYDTMVGERGIQLSGGQKQRVAIARAIVKDP
KVLLLDEATSALDAESERVVQDALDRVMVNRTTIVVAHRLSTIKNADVIAVVKNGVIVEK
GKHDTLINIKDGVYASLVQLHLTAAS
>Q96247
MSEGVEAIVANDNGTDQVNGNRTGKDNEEHDGSTGSNLSNFLWHGGSVWDAWFSCASNQV
AQVLLTLPYSFSQLGMLSGIVLQIFYGLLGSWTAYLISVLYVEYRARKEKEGKSFKNHVI
QWFEVLDGLLGSYWKALGLAFNCTFLLFGSVIQLIACASNIYYINDHLDKRTWTYIFGAC
CATTVFIPSFHNYRIWSFLGLGMTTYTAWYLAIASIIHGQAEGVKHSGPTKLVLYFTGAT
NILYTFGGHAVTVEIMHAMWKPQKFKYIYLMATLYVFTLTIPSAAAVYWAFGDALLDHSN
AFSLMPKNAWRDAAVILMLIHQFITFGFACTPLYFVWEKVIGMHDTKSICLRALARLPVV
IPIWFLAIIFPPFFGPINSAVGALLVSFTVYIIPSLAHMLTYRSASARQNAAEKPPFFMPS
WTAMYVLNAFVVVWVLIVGFGFGGWASVTNFVRQVDTFGLFAKCYQCKPAAAAAHAPVSA
LHHRL
>Q9LX20
MVSRSAFLLFCVLFLATEETLASLFSSRLIHRFSDEGRASIKTPSSSDSLPNKQSLEYYR
LLAESDFRRQRMNLGAKVQSLVPSEGSKTISSGNDFGWLHYTWIDIGTPSVSFLVALDTG
SNLLWIPCNCVQCAPLTSTYYSSLATKDLNEYNPSSSSTSKVFLCSHKLCDSASDCESPK
EQCPYTVNYLSGNTSSSGLLVEDILHLTYNTNNRLMNGSSSVKARVVIGCGKKQSGDYLD
GVAPDGLMGLGPAEISVPSFLSKAGLMRNSFSLCFDEEDSGRIYFGDMGPSIQQSTPFLQ
LDNNKYSGYIVGVEACCIGNSCLKQTSFTTFIDSGQSFTYLPEEIYRKVALEIDRHINAT
SKNFEGVSWEYCYESSAEPKVPAIKLKFSHNNTFVIHKPLFVFQQSQGLVQFCLPISPSG
QEGIGSIGQNYMRGYRMVFDRENMKLGWSPSKCQEDKIEPPQASPGSTSSPNPLPTDEQQ
SRGGHAVSPAIAGKTPSKTPSSSSSYSFSSIMRLFNSLLLLHWLASLM
>Q8L940
MEGTGVVAVYGNGAITEAKKSPFSVKVGLAQMLRGGVIMDVVNAEQARIAEEAGACAVMA

LERVPADIRAQGGVARMSDPQMIKEIKQAVTIPVMAKARIGHFVEAQILEAIGIDYIDES
EVLTLADEDHHINKHNFRIPFVCGCRNLGEALRRIREGAAMIRTKGEAGTGNIIEAVRHV
RSVNGDIRVLRNMDDDEVFTFAKKLAAPYDLVMQTKQLGRLPVVQFAAGGVATPADAALM
MQLGCDGVFVGSGIFKSGDPARRARAIVQAVTHYSDPEMLVEVSCGLGEAMVGINLNDEK
VERFANRSE

>Q7DM58

MWLLSSSPWLSELSCSYSAVVEHTSSVPVPIQWLRFVLLSPCPQRALFSAVDFIFLLCFA
LHKLFSSPSSSSEINGHAEIRKPLIGIRGRTPTRTTAWFKTTVAVTVLLSFCSVVLCVLA
FTGKRRTQRPWNLIDPLFWLIHAVTHLVIAVLVLHQKRFAALNHPLSLRIYWISSFVLTS
LFAVTGIFHFLSDAATSLRAEDVASFFSFPLTAFLLIASVRGITGLVTAETNSPTKPSDA
VSVEKSDNVSLYASASVFSKTFWLWMNPLLSKGYKSPLTLEQVPTLSPEHKAERLALLFE
SSWPKPSENSSHPIRTTLLRCFWKEILFTAILAIVRLGVMYVGPVLIQSFVDFTSGKRSS
PWQGYYLVLILLVAKFVEVLTTHQFNFDSQKLGMLIRSTLITALYKKGLKLTGSARQNHG
VGQIVNYMAVDAQQLSDMMLQLHAIWLMPLQVTVALVLLYGSLGASVITAVIGLTGVFVF
ILLGTQRNNGYQFSLMGNRDSRMKATNEMLNYMRVIKFQAWENHFNKRILKFRDMEFGWL
SKFLYSIAGNIIVLWSTPVLISALTFATALALGVKLDAGTVFTTTTIFKILQEPIRTFPQ
SMISLSQAMISLGRLDSYMMSKELSEDAVERALGCDGNTAVEVRDGSFSWDDEDNEPALS
DINFKVKKGELTAIVGTVGSGKSSLLASVLGEMHRISGQVRVCGSTGYVAQTSWIENGTV
QDNILFGLPMVREKYNKVLNVCSLEKDLQMMEFGDKTEIGERGINLSGGQKQRIQLARAV
YQECDVYLLDDVFSAVDAHTGSDIFKKCVRGALKGKTVLLVTHQVDFLHNVDCILVMRDG
KIVESGKYDELVSSGLDFGELVAAHETSMELVEAGADSAAVATSPRTPTSPHASSPRTSM
ESPHLSDLNDEHIKSFLGSHIVEDGSKLIKEEERETGQVSLGVYKQYCTEAYGWWGIVLV
LFFSLTWQGSLMASDYWLAYETSAKNAISFDASVFILGYVIIALVSIVLVSIRSYYVTHL
GLKTAQIFFRQILNSILHAPMSFFDTTPSGRILSRASTDQTNVDILIPFMLGLVVSMYTT
LLSIFIVTCQYAWPTAFFVIPLGWLNIWYRNYYLASSRELTRMDSITKAPIIHHFSESIA
GVMTIRSFRKQELFRQENVKRVNDNLRMDFHNNGSNEWLGFRLELVGSWVLCISALFMVL
LPSNVIRPENVGLSLSYGLSLNSVLFFAIYMSCFVENKMVSVERIKQFTDIPSESEWERK
ETLPPSNWPFHGNVHLEDLKVRYRPNTPLVLKGITLDIKGGEKVGVVGRTGSGKSTLIQV
LFRLVEPSGGKIIIDGIDISTLGLHDLRSRFGIIPQEPVLFEGTVRSNIDPTEQYSDEEI
WKSLERCQLKDVVATKPEKLDSLVVDNGENWSVGQRQLLCLGRVMLKRSRLLFLDEATAS
VDSQTDAVIQKIIREDFASCTIISIAHRIPTVMDGDRVLVIDAGKAKEFDSPARLLERPS
LFAALVQEYALRSAGI

>P31414

MVAPALLPELWTEILVPICAVIGIAFSLFQWYVVSRVKLTSDLGASSSGGANNGKNGYGD
YLIEEEEGVNDQSVVAKCAEIQTAISEGATSFLFTEYKYVGVFMIFFAAVIFVFLGSVEG
FSTDNKPCTYDTTRTCKPALATAAFSTIAFVLGAVTSVLSGFLGMKIATYANARTTLEAR
KGVGKAFIVAFRSGAVMGFLLAASGLLVLYITINVFKIYYGDDWEGLFEAITGYGLGGSS
MALFGRVGGGIYTKAADVGADLVGKIERNIPEDDPRNPAVIADNVGDNVGDIAGMGSDLF
GSYAEASCAALVVASISSFGINHDFTAMCYPLLISSMGILVCLITTLFATDFFEIKLVKE
IEPALKNQLIISTVIMTVGIAIVSWVGLPTSFTIFNFGTQKVVKNWQLFLCVCVGLWAGL
IIGFVTEYYTSNAYSPVQDVADSCRTGAATNVIFGLALGYKSVIIPIFAIAISIFVSFSF
AAMYGVAVAALGMLSTIATGLAIDAYGPISDNAGGIAEMAGMSHRIRERTDALDAAGNTT
AAIGKGFAIGSAALVSLALFGAFVSRAGIHTVDVLTPKVIIGLLVGAMLPYWFSAMTMKS
VGSAALKMVEEVRRQFNTIPGLMEGTAKPDYATCVKISTDASIKEMIPPGCLVMLTPLIV

GFFFGVETLSGVLAGSLVSGVQIAISASNTGGAWDNAKKYIEAGVSEHAKSLGPKGSEPH
KAAVIGDTIGDPLKDTSGPSLNILIKLMAVESLVFAPFFATHGGILFKYF

(2) $\mathbb{S}_2$: 32 cell wall proteins

>Q42589
MAGVMKLACLLLACMIVAGPITSNAALSCGSVNSNLAACIGYVLQGGVIPPACCSGVKNL
NSIAKTTPDRQQACNCIQGAARALGSGLNAGRAAGIPKACGVNIPYKISTSTNCKTVR
>P26792
MGVTIRNRNYDHGSLPFLQSLLAILLVTTTTLHINGVEAFHEIHYNLQSVGAENVKQVHR
TGYHFQPKQNWINDPNGPMYYKGVYHLFYQYNPKGAVWGNIVWAHSVSTDLINWTPLEPA
IFPSKPFDKYGCRSGSATILPGNKPVILYTGIVEGPPKNVQVQNYAIPANLSDPYLRKWI
KPDNNPLVVANNGENATAFRDPTTAWLDKSGHWKMLVGSKRNRRGIAYLYRSKDFIKWTK
AKHPIHSQANTGMWECPDFFPVSLKGLNGLDTSVTGESVKHVLKVSLDLTRYEYYTVGTY
LTDKDRYIPDNTSVDGWAGLRYDYGNFYASKTFFDPSKNRRILWGWANESDSTAHDVAKG
WAGIQLIPRTLWLDPSGKQLMQWPIEELETLRGSKVKFSRKQDLSKGILVEVKGITAAQA
DVEVTFSFKSLAKREPFDPKWLEYDAEKICSLKGSTVQGGVGPFGLLTLASEKLEEYTPV
FFRVFKAQNTHKVLMCSDATRSSLKEGLYRPSFAGFVDVDLATDKKISLRSLIDNSVVES
FGAKGKTCISSRVYPTLAVYENAHLYVFNNGSETITVENLDAWSMKKPLRMN
>Q5MFV8
MIGKVVVSVASILLIVGVAIGVVAYINKNGDANLSPQMKAVRGICEATSDKASCVKTLEP
VKSDDPNKLIKAFMLATRDAITQSSNFTGKTEGNLGSGISPNNKAVLDYCKKVFMYALED
LSTIVEEMGEDLNQIGSKIDQLKQWLTGVYNYQTDCLDDIEEDDLRKTIGEGIASSKILT
SNAIDIFHTVVSAMAKLNLKVEDFKNMTGGIFAPSDKGAAPVNKGTPPVADDSPVADPDG
PARRLLEDIDETGIPTWVSGADRKLMTKAGRGSNDGGARIRATFVVAKDGSGQFKTVQQA
VNACPEKNPGRCIIHIKAGIYREQVIIPKKKNNIFMFGDGARKTVISYNRSVKLSPGTTT
SLSGTVQVESEGFMAKWIGFKNTAGPMGHQAVAIRVNGDRAVIFNCRFDGYQDTLYVNNG
RQFYRNIVVSGTVDFIFGKSATVIQNSLIVVRKGNKGQFNTVTADGNEKGLAMKIGIVLQ
NCRIVPDKKLAAERLIVESYLGRPWKKFSTTVIINSEIGDVIRPEGWKIWDGESFHKSCR
YVEYNNRGPGAITNRRVNWVKIARSAAEVNDFTVANWLGPINWIQEANVPVTLGL
>Q03464
MKMMVVVVVMMLSWLILKPPSTWAINTITFDVGNATINKYATFMKSIHNQAKDPTLKCYG
IPMLPNTNLTPKYLLVTLQDSSLKTITLMLKRNNLYVMGYADTYNGKCRYHIFKDISNTT
ERNDVMTTLCPNPSSRVGKNINYDSSYPALEKKVGRPRSQVQLGIQILNSGIGKIYGVDS
FTEKTEAEFLLVAIQMVSEAARFKYIENQVKTNFNRAFYPNAKVLNLEESWGKISTAIHN
AKNGALTSPLELKNANGSKWIVLRVDDIEPDVGLLKYVNGTCQATYQSAMFPHL
>Q9S7Y7
MASSSSSLAFSLSLLLALILCFSPTQSYKTIGKGYRLVSIEESPDGGFIGYLQVKQKNKI
YGSDITTLRLFVKHETDSRLRVHITDAKQQRWEVPYNLLPREQPPQVGKVIGKSRKSPIT
VQEISGSELIFSYTTDPFTFAVKRRSNHETLFNTTSSLVFKDQYLEISTSLPKEASLYGL
GENSQANGIKLVPNEPYTLYTEDVSAINLNTDLYGSHPMYMDLRNVGGKAYAHAVLLLNS
NGMDVFYRGDSLTYKVIGGVFDFYFIAGPSPLNVVDQYTQLIGRPAPMPYWSLGFHQCRW
GYHNLSVVEDVVDNYKKAKIPLDVIWNDDDHMDGHKDFTLNPVAYPRAKLLAFLDKIHKI
GMKYIVINDPGIGVNASYGTFQRAMAADVFIKYEGKPFLAQVWPGPVYFPDFLNPKTVSW
WGDEIKRFHDLVPIDGLWIDMNEVSNFCSGLCTIPEGKQCPSGEGPGWVCCLDCKNITKT

RWDDPPYKINATGVVAPVGFKTIATSATHYNGVREYDAHSIYGFSETIATHKGLLNVQGK
RPFILSRSTFVGSGQYAAHWTGDNQGTWQSLQVSISTMLNFGIFGVPMVGSDICGFYPQP
TEELCNRWIEVGAFYPFSRDHANYYSPRQELYQWDTVADSARNALGMRYKILPFLYTLNY
EAHMTGAPIARPLFFSFPEYTECYGNSRQFLLGSSFMISPVLEQGKTEVEALFPPGSWYH
MFDMTQAVVSKNGKRVTLPAPLNFVNVHLYQNTILPTQQGGLISKDARTTPFSLVIAFPA
GASEGYATGKLYLDEDELPEMKLGNGQSTYVDFYASVGNGTMKMWSQVKEGKFALSKGWV
IEKVSVLGLRGAGQVSEIQINGSPMTKKIEVSSKEHTYVIGLEDEEENKSVMVEVRGLEM
LVGKDFNMSWKMGIN
>P15290
MGYSKTLVAGLFAMLLLAPAVLATDPDPLQDFCVADLDGKAVSVNGHTCKPMSEAGDDFL
FSSKLAKAGNTSTPNGSAVTELDVAEWPGTNTLGVSMNRVDFAPGGTNPPHIHPRATEIG
IVMKGELLVGILGSLDSGNKLYSRVVRAGETFLIPRGLMHFQFNVGKTEASMVVSFNSQN
PGIVFVPLTLFGSNPPIPTPVLTKALRVEARVVELLKSKFAAGF
>Q9C9H5
MTSLFFFVLLFSSLLISNGDANPNYKEALSKSLLFFQGQRSGPLPRGQQISWRASSGLSD
GSAAHVDLTGGYYDAGDNVKFNLPMAFTTTMLSWSALEYGKRMGPELENARVNIRWATDY
LLKCARATPGKLYVGVGDPNVDHKCWERPEDMDTPRTVYSVSASNPGSDVAAETAAALAA
ASMVFRKVDSKYSRLLLATAKDVMQFAIQYQGAYSDSLSSSVCPFYCSYSGYKDELMWGA
SWLLRATNNPYYANFIKSLGGGDQPDIFSWDNKYAGAYVLLSRRALLNKDSNFEQYKQAA
ENFICKILPDSPSSSTQYTQGGLMYKLPQSNLQYVTSITFLLTTYAKYMKATKHTFNCGS
SVIVPNALISLSKRQVDYILGDNPIKMSYMVGFSSNFPKRIHHRASSLPSHALRSQSLGC
NGGFQSFYTQNPNPNILTGAIVGGPNQNDGYPDQRDDYSHAEPATYINAAFVGPLAYFAA
GRST
>O04300
MASLPKPTPLLKDELDIVIPTIRNLDFLEMWRPFFEQYHLIIVQDGDPSKVIKVPEGFDY
ELYNRNDINRILGPKASCISFKDSACRCFGYMVSKKKYIYTIDDDCFVAKDPTGHEINAL
EQHIKNLLSPSTPFFFNTLYDPYREGTDFVRGYPFSLREGVPTAVSHGLWLNIPDYDAPT
QLVKPHERNTRFVDAVLTIPKGSLFPMCGMNLAFNRELIGPAMYFGLMGDGQPIGRYDDM
WAGWCIKVICDHLGYGVKTGLPYIWHSKASNPFVNLKKEYKGIFWQEEIIPFFQAATLSK
DCTSVQKCYIELSKQVKEKLGTIDPYFIKLADAMVTWVEAWDEINNNKSEETTSTKASEV
AATK
>Q05538
MRLSEFTTLFLLFSVLLLSASAEQCGSQAGGALCASGLCCSKFGWCGNTNEYCGPGNCQS
QCPGGPGPSGDLGGVISNSMFDQMLNHRNDNACQGKNNFYSYNAFVTAAGSFPGFGTTGD
ITARKREIAAFLAQTSHETTGGWPTAPDGPYAWGYCFLREQGSPGDYCTPSSQWPCAPGR
KYFGRGPIQISHNYNYGPCGRAIGVDLLNNPDLVATDPVISFKSAIWFWMTPQSPKPSCH
DVITGRWQPSGADQAANRVPGFGVITNIINGGLECGHGSDSRVQDRIGFYRRYCGILGVS
PGENLDCGNQRSFGNGLLVDIM
>Q9SYM4
MPGNKYNCSSSHIPLSRTERLLRDRELREKRKSNRARNPNDVAGSSENSENDLRLEGDSS
RQYVEQYLEGAAAAMAHDDACERQEVRPYNRQRLLVVANRLPVSAVRRGEDSWSLEISAG
GLVSALLGVKEFEARWIGWAGVNVPDEVGQKALSKALAEKRCIPVFLDEEIVHQYYNGYC
NNILWPLFHYLGLPQEDRLATTRSFQSQFAAYKKANQMFADVVNEHYEEGDVVWCHDYHL
MFLPKCLKEYNSKMKVGWFLHTPFPSSEIHRTLPSRSELLRSVLAADLVGFHTYDYARHF

VSACTRILGLEGTPEGVEDQGRLTRVAAFPIGIDSDRFIRALEVPEVIQHMKELKERFAG
RKVMLGVDRLDMIKGIPQKILAFEKFLEENANWRDKVVLLQIAVPTRTDVPEYQKLTSQV
HEIVGRINGRFGTLTAVPIHHLDRSLDFHALCALYAVTDVALVTSLRDGMNLVSYEFVAC
QEAKKGVLILSEFAGAAQSLGAGAILVNPWNITEVAASIGQALNMTAEEREKRHRHNFHH
VKTHTAQEWAETFVSELNDTVIEAQLRISKVPPELPQHDAIQRYSKSNNRLLILGFNATL
TEPVDNQGRRGDQIKEMDLNLHPELKGPLKALCSDPSTTIVVLSGSSRSVLDKNFGEYDM
WLAAENGMFLRLTNGEWMTTMPEHLNMEWVDSVKHVFKYFTERTPRSHFETRDTSLIWNY
KYADIEFGRLQARDLLQHLWTGPISNASVDVVQGSRSVEVRAVGVTKGAAIDRILGEIVH
SKSMTTPIDYVLCIGHFLGKDEDVYTFFEPELPSDMPAIARSRPSSDSGAKSSSGDRRPP
SKSTHNNNKSGSKSSSSSNSNNNNKSSQRSLQSERKSGSNHSLGNSRRPSPEKISWNVLD
LKGENYFSCAVGRTRTNARYLLGSPDDVVCFLEKLADTTSSP
>Q9M069
MALSISIYFLLIFLSHFPSSHAEPFIGVNYGQVADNLPPPSETVKLLQSTSIQKVRLYGA
DPAIIKALAGTGVGIVIGAANGDVPSLASDPNAATQWINSNVLPFYPASKIMLITVGNEI
LMSNDPNLVNQLLPAMQNVQKALEAVSLGGKIKVSTVNSMTVLGSSDPPSSGSFAAGYQT
GLKGILQFLSDTGSPFAINPYPFFAYQSDPRPETLAFCLFEPNAGRVDSKTGIKYTNMFD
AQVDAVHSALKSMGFEKVEIVVAETGWASRGDANEVGASVDNAKAYNGNLIAHLRSMVGT
PLMPGKPVDTYIFALYDENLKPGPSSERAFGLFKTDLSMVYDVGLAKSSSSSQVRY
>Q9FS16
MASLVATLLVLTISLTFVSQSTANYFYSSPPPPVKHYTPPVKHYSPPPVYHSPPPPKKHY
EYKSPPPPVKHYSPPPVYHSPPPPKKHYVYKSPPPPVKHYSPPPVYHSPPPPKKHYVYKS
PPPPVKHYSPPPVYHSPPPPKKHYVYKSPPPPVKHYSPPPVYHSPPPPKKHYVYKSPPPP
VKHYSPPPVYHSPPPPKKHYVYKSPPPPVKHYSPPPVYHSPPPPKKHYVYKSPPPPVKHY
SPPPVYHSPPPPKKHYVYKSPPPPVKHYSPPPVYHSPPPPKKHYVYKSPPPPVKHYSPPP
VYHSPPPPKKHYVYKSPPPPVKHYSPPPVYHSPPPPKKHYVYKSPPPPVKHYSPPPVYHS
PPPPKKHYVYKSPPPPVKHYSPPPVYHSPPPPKEKYVYKSPPPPPVHHYSPPHHPYLYKS
PPPPYHY
>P13983
MKLSTLFALVLLLQSTAILSLVAAEATTQYGGYLPPPVTSQPPPSSIGLSPPSAPTTTPP
SRGHVPSPRHAPPRHAYPPPSHGHLPPSVGGPPPHRGHLPPSRGFNPPPSPVISPSHPPP
SYGAPPPSHGPGHLPSHGQRPPSPSHGHAPPSGGHTPPRGQHPPSHRRPSPPSRHGHPPP
PTYAQPPPTPIYSPSPQVQPPPTYSPPPPTHVQPTPSPPSRGHQPQPPTHRHAPPTHRHA
PPTHQPSPLRHLPPSPRRQPQPPTYSPPPPAYAQSPQPSPTYSPPPPTYSPPPPSPIYSP
PPPAYSPSPPPTPTPTFSPPPPAYSPPPTYSPPPPTYLPLPSSPIYSPPPPVYSPPPPPS
YSPPPPTYLPPPPPSSPPPPSFSPPPPTYEQSPPPPPAYSPPLPAPPTYSPPPPTYSPPP
PTYAQPPPLPPTYSPPPPAYSPPPPPTYSPPPPTYSPPPPAYAQPPPPPPTYSPPPPAYS
PPPPSPIYSPPPPQVQPLPPTFSPPPPRRIHLPPPPHRQPRPPTPTYGQPPSPPTFSPPP
PRQIHSPPPPHWQPRTPTPTYGQPPSPPTFSAPPPRQIHSPPPPHRQPRPPTPTYGQPPS
PPTTYSPPSPPPYGLLLSTP
>O65351
MSSSFLSSTAFFLLLCLGFCHVSSSSSDQGTYIVHMAKSQMPSSFDLHSNWYDSSLRSIS
DSAELLYTYENAIHGFSTRLTQEEADSLMTQPGVISVLPEHRYELHTTRTPLFLGLDEHT
ADLFPEAGSYSDVVVGVLDTGVWPESKSYSDEGFGPIPSSWKGGCEAGTNFTASLCNRKL
IGARFFARGYESTMGPIDESKESRSPRDDDGHGTHTSSTAAGSVVEGASLLGYASGTARG

MAPRARVAVYKVCWLGGCFSSDILAAIDKAIADNVNVLSMSLGGGMSDYYRDGVAIGAFA
AMERGILVSCSAGNAGPSSSSLSNVAPWITTVGAGTLDRDFPALAILGNGKNFTGVSLFK
GEALPDKLLPFIYAGNASNATNGNLCMTGTLIPEKVKGKIVMCDRGINARVQKGDVVKAA
GGVGMILANTAANGEELVADAHLLPATTVGEKAGDIIRHYVTTDPNPTASISILGTVVGV
KPSPVVAAFSSRGPNSITPNILKPDLIAPGVNILAAWTGAAGPTGLASDSRRVEFNIISG
TSMSCPHVSGLAALLKSVHPEWSPAAIRSALMTTAYKTYKDGKPLLDIATGKPSTPFDHG
AGHVSPTTATNPGLIYDLTTEDYLGFLCALNYTSPQIRSVSRRNYTCDPSKSYSVADLNY
PSFAVNVDGVGAYKYTRTVTSVGGAGTYSVKVTSETTGVKISVEPAVLNFKEANEKKSYT
VTFTVDSSKPSGSNSFGSIEWSDGKHVVGSPVAISWT
>P05117
MVIQRNSILLLIIIFASSISTCRSNVIDDNLFKQVYDNILEQEFAHDFQAYLSYLSKNIE
SNNNIDKVDKNGIKVINVLSFGAKGDGKTYDNIAFEQAWNEACSSRTPVQFVVPKNKNYL
LKQITFSGPCRSSISVKIFGSLEASSKISDYKDRRLWIAFDSVQNLVVGGGGTINGNGQV
WWPSSCKINKSLPCRDAPTALTFWNCKNLKVNNLKSKNAQQIHIKFESCTNVVASNLMIN
ASAKSPNTDGVHVSNTQYIQISDTIIGTGDDCISIVSGSQNVQATNITCGPGHGISIGSL
GSGNSEAYVSNVTVNEAKIIGAENGVRIKTWQGGSGQASNIKFLNVEMQDVKYPIIIDQN
YCDRVEPCIQQFSAVQVKNVVYENIKGTSATKVAIKFDCSTNFPCEGIIMENINLVGESG
KPSEATCKNVHFNNAEHVTPHCTSLEISEDEALLYNY
>P23137
MGSKAFLFLGLCLAFFFLISSEVVAGELAETSNPMKLDGENGVDVDGRGGYNDVGGDGYY
GGGRGRGGGGYKRRGCRYGCCRKGYNGCKRCCSYAGEAMDKVTEAQPHN
>Q40161
MHTKIHLPPCILLLLLLFSLPSFNVVVGGDGESGNPFTPKGYLIRYWKKQISNDLPKPWFL
LNKASPLNAAQYATYTKLVADQNALTTQLHTFCSSANLMCAPDLSPSLEKHSGDIHFATY
SDKNFTNYGTNEPGIGVNTFKNYSEGENIPVNSFRRYGRGSPRDNKFDNYASDGNVIDQS
FNSYSTSTAGGSGKFTNYAANANDPNLHFTSYSDQGTGGVQKFTIYSQEANAGDQYFKSY
GKNGNGANGEFVSYGNDTNVIGSTFTNYGQTANGGDQKFTSYGFNGNVPENHFTNYGAGG
NGPSETFNSYRDQSNVGDDTFTTYVKDANGGEANFTNYGQSFNEGTDVFTTYGKGGNDPH
INFKTYGVNNTFKDYVKDTATFSNYHNKTSQVLASLMEVNGGKKVNNRWVEPGKFFREKM
LKSGTIMPMPDIKDKMPKRSFLPRVIASKLPFSTSKIAELKKIFHAGDESQVEKMIGDAL
SECERAPSAGETKRCVNSAEDMIDFATSVLGRNVVVRTTEDTKGSNGNIMIGSVKGINGG
KVTKSVSCHQTLYPYLLYYCHSVPKVRVYEADILDPNSKVKINHGVAICHVDTSSWGPSH
GAFVALGSGPGKIEVCHWIFENDMTWAIAD
>P31178
MSLATRRFGAAAALLVAACVLCTAPAWAQNETTGTGMVKTKSAFRWIRPPPARPPPFRRP
PPAQTPYVHKVEYTELQILCPQTIDSVTGYPMDDPRCNVPRATVAAGEEALTIRNEFELL
NGDVLNVTLEEVDTPENPSRRRLLSIIREEQRTGRVLLATSAELPTPTFRLKSLKSILKG
SQKEIYAGKPIDLRTIVYIMDFSSCKLSGWSAPATLTPEKVTSDMLRGASAPTNNLANYY
GACSYEKTLFNPDNFLVLGPVPVPCIGGVTPPPRPPRPPRPPPRAGSTISSLSRRNDTYD
DWWDLSKYCTASEQQAWERAAEAYAQAIVAQDPNSATGKKLQGILQWRERRRNIYILPPG
VKCSWSGYADVTCTSATCSAYVRGYSDTNAMQVIMHEAMHNYGLEHAGRGTLEYGDATDV
MGDFNKAGKGLLCPNAPNMYRIGWAKPINEPGVAPFQNATGAWGNLTAANFTTDPWIRGL
VIPAQGTRDDNMIVVNVGAQSTRDGAMKATGAQAYYFSYRIKNTTAGGYDSGLTLDFHKK
VLVHAYNGIQSERVFGFKSNLLDWGPNFQSRSNTWTSPFLAYNNGLGGGVRLVVQSTSDT

QAVVDICRISENGKELSCDDGIDNDCDGLQDNEDPDCQ
>P93164
MPNDSVLSLFFFVTLFTCLLSATSHDDHIFLPSQLHDDDSVSCTATDPSLNYKPVIGILT
HPGDGASGRLSNATGVSYIAASYVKFVESGGARVIPLIYNESPENLNKKLDLVNGVLFTG
GWAVSGPYLDTLGNIFKKALERNDAGDHFPVIAFNLGGNLVIRIVSEQTDILEPFTASSL
PSSLVLWNEANAKGSLFQRFPSDLLTQLKTDCLVLHNHRYAISPRKLQYNTKLSDFFEIL
ATSGDRDGKTFVSTARGRKYPVTVNLWQPEKNAFEWATSLKAPHTEDAIRVTQSTANFFI
SEARKSTNTPDAQKVRDSLIYNYKPTFGGTAGKGYDQVYLFE
>P80022
MASNSAFSLFLILLIITQCLSVLNAAKDFDFFYFVQQWPGSYCDTKQSCCYPTTGKPAAD
FGIHGLWPNNNDGTYPSNCDPNSPYDQSQISDLISSMQQNWPTLACPSGSGSTFWSHEWE
KHGTCAESVLTNQHAYFKKALDLKNQIDLLSILQGADIHPDGESYDLVNIRNAIKSAIGY
TPWIQCNVDQSGNSQLYQVYICVDGSGSSLIECPIFPGGKCGTSIEFPTF
>Q946Y7
MSSAQDPFYIVKEEIQDSIDKLQSTFHKWERISPDMGDQAHVAKELVATCGSIEWQVDEL
EKAITVAAKDPSWYGIDEAELEKRRRWTSNARTQVRNVKSGVLAGKVSSGAGHASEVRRE
LMRMPNSGEASRYDQYGGRDDDGFVQSESDRQMLLIKQQDEELDELSKSVQRIGGVGLTI
HDELVAQERIIDELDTEMDSTKNRLEFVQKKVGMVMKKAGAKGQMMMICFLLVLFIILFV
LVFLT
>P93349
MGVKGLLFSIVLINLSLLGLCGYPRKPVDVPFWKNYEPSWASHHIKYLSGGSTVDLVLDR
SSGAGFQSKKSYLFGHFSMKLKLVGGDSAGVVTAFYLSSNNAEHDEIDFEFLGNRTGQPY
ILQTNVFTGGKGDREQRIYLWFDPTKGYHSYSVLWNTFQIVIFVDDVPIRAFKNSKDLGV
KFPFNQPMKIYSSLWDADDWATRGGLEKTDWSNAPFTASYTSFHVDGCEAATPQEVQVCN
TKGMRWWDQKAFQDLDALQYRRLRWVRQKYTIYNYCTDRKRYPTLPPECTKDRDI
>Q9FL76
MELLKRKLYAKILMMVMVIWIAPMTNGHDHASHVPGGRPGAHPSHGAHPAHGAHPSHGAH
PSHGAHPSHGAHPSHGALPSHGGQVPHSGWGHGRATFYGDINGGETQQGACGYGDLHKQG
YGLETAALSTALFNNGSRCGACYEIMCEHAPQWCLPGSIKITATNFCPPDFTKPNDNWCN
PPQKHFDLSQPMFLKIAKYKAGVVPVKFRRVPCAKIGGVKFEIKGNPHFLMILPYNVGGA
GAVRAMQIKGTRTQWIAMKKNWGQIWSTGVVLTGQCLSFRLTTSDGVMKEFIDVTPPDWK
CNGQSFDGKVNF
>P35334
MTQFNIPVTMSSSLSIILVILVSLRTALSELCNPQDKQALLQIKKDLGNPTTLSSWLPTT
DCCNRTWLGVLCDTDTQTYRVNNLDLSGHNLPKPYPIPSSLANLPYLNFLYIGGINNLVG
PIPPAIAKLTQLHYLYITHTNVSGAIPDFLSQIKTLVTLDFSYNALSGTLPPSISSLPNL
GGITFDGNRISGAIPDSYGSFSKLFTAMTISRNRLTGKIPPTFANLNLAFVDLSRNMLEG
DASVLFGSDKNTKKIHLAKNSLAFDLGKVGLSKNLNGLDLRNNRIYGTLPQGLTQLKFLQ
SLNVSFNNLCGEIPQGGNLKRFDVSSYANNKCLCGSPLPSCT
>Q9SU40
MDLFKILLLVFFVNISFCFAADPYSFYNFEVSYITASPLGVPQQVIAINGKFPGPTINVT
TNENLVVNVRNKLDEGLLLHWNGIQQRRVSWQDGVLGTNCPIPPKWNWTYEFQVKDQIGS
FFYFPSLHFQRASGGFGSFVVNPRAIIPVPFSTPDGDITVTIGDWYIRNHTALRKALDDG
KDLGMPDGVLINGKGPYRYNDTLVADGIDFETITVHPGKTYRLRVSNVGISTSLNFRIQG

HNLVLAESEGSYTVQQNYTSLDIHVGQSYSFLVTMDQNASSDYYIVASARVVNETIWRRV
TGVGILKYTNSKGKAKGQLPPGPQDEFDKTFSMNQARSIRWNVSASGARPNPQGSFKYGS
INVTDVYVLRNMPPVTISGKRRTTLNGISFKNPSTPIRLADKLKVKDVYKLDFPKRPLTG
PAKVATSIINGTYRGFMEVVLQNNDTKMQSYHMSGYAFFVVGMDYGEWTENSRGTYNKWD
GIARSTIQVYPGAWSAILISLDNPGAWNLRTENLDSWYLGQETYVRVVNPDENNKTEFGH
PDNVLYCGALSKLQKPQKVSSSASKSIGFTSLSMVVMALVMMMMLQH
>Q8GXV7
MKLTYQFFIFWFFLPFFAISGDDDYKNLIFKGCANQKSPDPTGVFSQNLKNLFTSLVSQS
SQSSFASVTSGTDNTTAVIGVFQCRGDLQNAQCYDCVSKIPKLVSKLCGGGRDDGNVVAA
RVHLAGCYIRYESSGFRQTSGTEMLFRVCGKKDSNDPGFVGKRETAFGMAENGVKTGSSG
GGGGGGGFYAGQYESVYVLGQCEGSLGNSDCGECVKDGFEKAKSECGESNSGQVYLQKCF
VSYSYYSHGVPNIEPLSGGEKRQHTERTIALAVGGVFVLGFVIVCLLVLRSAMKKKSNKY
DAY
>P29060
MIKYSFLLTALVLFLRALKLEAGDIVIYWGQNGNEGSLADTCATNNYAIVNIAFLVVFGN
GQNPVLNLAGHCDPNAGACTGLSNDIRACQNQGIKVMLSLGGGAGSYFLSSADDARNVAN
YLWNNYLGGQSNTRPLGDAVLDGIDFDIEGGTTQHWDELAKTLSQFSQQRKVYLTAAPQC
PFPDTWLNGALSTGLFDYVWVQFYNNPPCQYSGGSADNLKNYWNQWNAIQAGKIFLGLPA
AQGAAGSGFIPSDVLVSQVLPLINGSPKYGGVMLWSKFYDNGYSSAIKANV
>P14918
MCPAFSIFFNSRRYSLTPPTYTPSPKPPTPKPTPPTYTPSPKPPASKPPTPKPTPPTYTP
SPKPPTPKPTPPTYTPSPKPPATKPPTPKPTPPTYTPSPKPPTPKPTPPTYTPSPKPPAT
KPPTPKPTPPTYTPSPKPPTPKPTPPTYTPSPKPPTPKPTPPTYTPSPKPPTHPTPKPTP
PTYTPSPKPPTPKPTPPTYTPSPKPPTPKPTPPTYTPSPKPPATKPPTPKPTPPTYTPTP
KPPATKPPTYTPTPPVSHTPSPPPPYY
>P43643
MGKEKFHINIVVIGHVDSGKSTTTGHLIYKLGGIDKRVIERFEKEAAEMNKRSFKYAWVL
DKLKAERERGITIDIALWKFETTKYYCTVIDAPGHRDFIKNMITGTSQADCAVLIIDSTT
GGFEAGISKDGQTREHALLAFTLGVKQMICCCNKMDATTPKYSKARYDEIVKEVSSYLKK
VGYNPDKIPFVPISGFEGDNMIERSTNLDWYKGPTLLEALDQINDAKRPSDKPLRLPLQD
VYKIGGIGTVPVGRVETGVLKPGMVVTFGPTGLTTEVKSVEMHHEALQEALPGDNVGFNV
KNVAVKDLKRGFVASNSKDDPAKGAASFTSQVIIMNHPGQIGNGYAPVLDCHTSHIAVKF
AEILTKIDRRSGKEIEKEPKFLKNGDAGMVKMIPTKPMVVETFSEYPPLGRFAVRDMRQT
VAVGVIKNVDKKDPTGAKVTKAAQKKK
>P20075
MASRKDERAAKEERAQAAAELAAKELRDVNQDRERGIKVVEHKEEVSGGPGVIGSILKSV
QGTLGQAKEVVVGKAHDTAEVSRENTDYAYDKGREGGDVAAQKAEEAKEKAKMAKDTTMG
KAGEYKDYTAQKAEEAKEKAAQKAEETKEKAGEYKNYTAQKAGEAKDTTLGKAGEYKDYA
AQKAAEAKDTTAQKAAEAKEKTGEYKDYAAQKAAEAKVLAAQKAAEAKDTTGKDGEYKDY
AAQKAAEAKDATMQKTGEYKDYAAQKTAETKDATMEKAKEYKEYAAQKAAEAKDATMQKT
GEYKDYSAQKAAETKDATMEKTKEYKDYTAQKAAETKDATMEKAKEAKDTTVQKTGEYKD
YAAEKAKEGKDVTVEKAKEGKDTTVGKMTELKDSAADAARKAMDMFLGKKEEVKGKAGET
AEAAKEKYEDTEFAARKKMEELKLQEEGVKDEAKQRAEADRETAGDRGSAAKGTIFGAMG
SVKDAIVGKLTMPSDVVKDKQQQEAVIKVDETRPGAVAEALKAADQMHGQAFNDVGKMGD

EEVIVERKETRQGKM
>Q9M1G9
MGPSAHLISALGVIIMATMVAAYEPETYASPPPLYSSPLPEVEYKTPPLPYVDSSPPPTY
TPAPEVEYKSPPPPYVYSSPPPPTYSPSPKVDYKSPPPPYVYSSPPPPYYSPSPKVDYKS
PPPPYVYNSPPPPYYSPSPKVDYKSPPPPYVYSSPPPPYYSPSPKVEYKSPPPPYVYSSP
PPPYYSPSPKVDYKSPPPPYVYSSPPPPYYSPSPKVEYKSPPPPYVYSSPPPPYYSPSPK
VDYKSPPPPYVYSSPPPPYYSPSPKVDYKSPPPPYVYSSPPPPYYSPSPKVDYKSPPPPY
VYSSPPPPYYSPSPKVDYKSPPPPYVYSSPPPPYYSPSPKVDYKSPPPPYVYSSPPPPTY
SPSPKVDYKSPPPPYVYSSPPPPYYSPSPKVEYKSPPPPYVYSSPPPPTYSPSPKVYYKS
PPPPYVYSSPPPPYYSPSPKVYYKSPPPPYVYSSPPPPYYSPSPKVYYKSPPPPYVYSSP
PPPYYSPSPKVYYKSPPPPYVYSSPPPPYYSPSPKVYYKSPPPPYVYSSPPPPYYSPSPK
VHYKSPPPPYVYSSPPPPYYSPSPKVHYKSPPPPYVYNSPPPPYYSPSPKVYYKSPPPPY
VYSSPPPPYYSPSPKVYYKSPPPPYVYSSPPPPYYSPSPKVYYKSPPPPYYSPSPKVYYK
SPPHPHVCVCPPPPPCYSPSPKVVYKSPPPPYVYNSPPPPYYSPSPKVYYKSPPPPSYYS
PSPKVEYKSPPPPSYSPSPKTEY
>P54609
MSTPAESSDSKSKKDFSTAILERKKSPNRLVVDEAINDDNSVVSLHPATMEKLQLFRGDT
ILIKGKKRKDTVCIALADETCEEPKIRMNKVVRSNLRVRLGDVISVHQCPDVKYGKRVHI
LPVDDTVEGVTGNLFDAYLKPYFLEAYRPVRKGDLFLVRGGMRSVEFKVIETDPAEYCVV
APDTEIFCEGEPVKREDEERLDDVGYDDVGGVRKQMAQIRELVELPLRHPQLFKSIGVKP
PKGILLYGPPGSGKTLIARAVANETGAFFFCINGPEIMSKLAGESESNLRKAFEEAEKNA
PSIIFIDEIDSIAPKREKTNGEVERRIVSQLLTLMDGLKSRAHVIVMGATNRPNSIDPAL
RRFGRFDREIDIGVPDEIGRLEVLRIHTKNMKLAEDVDLERISKDTHGYVGADLAALCTE
AALQCIREKMDVIDLEDDSIDAEILNSMAVTNEHFHTALGNSNPSALRETVVEVPNVSWN
DIGGLENVKRELQETVQYPVEHPEKFEKFGMSPSKGVLFYGPPGCGKTLLAKAIANECQA
NFISVKGPELLTMWFGESEANVREIFDKARQSAPCVLFFDELDSIATQRGGGSGGDGGGA
ADRVLNQLLTEMDGMNAKKTVFIIGATNRPDIIDSALLRPGRLDQLIYIPLPDEDSRLNI
FKAALRKSPIAKDVDIGALAKYTQGFSGADITEICQRACKYAIRENIEKDIEKEKRRSEN
PEAMEEDGVDEVSEIKAAHFEESMKYARRSVSDADIRKYQAFAQTLQQSRGFGSEFRFEN
SAGSGATTGVADPFATSAAAAGDDDDLYN

(3) $\mathbb{S}_3$: 286 chloroplast proteins

>Q85AT7
MNTYAIIDTGGEQLRVEPGRFYDMRHFTLLNPSILDSNTKVLIYRVLMIHHESNIALGDS
WLEDATIKGRVLHSHFKDKITIYKMRSKKKMRRKLGYRLNLARFVVDSICFDGKEFYK
>Q944I4
MAVAISGSSLISSTLQHYNNRIYIRDYPIPCHSSNPICNSFNFKRRSFSPSSPKFNDHVV
NPSSSYLSSKLSPIRTHSSFAACGCSWIQDNSMVHDYATTTNGTSKRCSALPTTNTVDVS
SVSDLFEFICSGPLVNKIGITPQRVGQSIDKWLLYGSQLCRLFQLNELKLTIPQKARLYH
YYIPVFIWCEDQIALHNSKFKDGDDVPPLVIGFSAPQGCGKTTLVFALDYLFKTTKKKSA
TISVDDFYLTAEGQAELRKKNPGNALLEYRGNAGSHDLKLSVETLEALSKLTKEGLKMKV
PRYNKSAYSGRGDRADSSTWPEVEGPLSVILFEGWMLGFKPLPADVVKAVDPQLEVVNKN
LEAYYDAWDKYIDAWVVIKIQDPSYVYRWRLQAEIAMRQDGQAGMSDEEVNDFVSRYLPA
YKAYLPTLYAEGPSGSDPDRVLAIDIDEERNPILAN

>Q39129
MAEESRVPSSVSVTVAHDLLLAGHRYLDVRTPEEFSQGHACGAINVPYMNRGASGMSKNP
DFLEQVSSHFGQSDNIIVGCQSGGRSIKATTDLLHAGFTGVKDIVGGYSAWAKNGLPTKA

>Q8L6J3
MSSTKTPISLTIKLNQFTDKPTGLDINRYHNSPIMWRNIIKQLSSRTPQKLLFSSKNRTY
SFLGFGQDSVFKDNTKFRSLIPISCSNIVMGFQNLGEYLPGDEFLSRPLLKNQVNSNDFC
CRKSYASVAEAVAVSSTDAEEDVSVVDEVQELLTELKKEEKKQFAFRRRKQRMLTSGMGH
RKYQTLKRRQVKVETEAWEQAAKEYKELLFDMCEQKLAPNLPYVKSLFLGWFEPLRDKIA
EEQELCSQGKSKAAYAKYLYQLPADMMAVITMHKLMGLLMTGGDHGTARVVQAALVIGDA
IEQEVRIHNFLEKTKKQKAEKDKQKEDGEHVTQEQEKLRKKVTNLMKKQKLRAVGQIVRR
QDDSKPWGQDAKAKVGSRLIELLLQTAYIQPPANQLAVDPPDIRPAFLHSVRTVAKETKS
ASRRYGIIQCDELVFKGLERTARHMVIPYMPMLVPPVKWTGYDKGGHLYLPSYVMRTHGA
RQQREAVKRASRNQLQPVFEALDTLGSTKWRINKRVLSVIDRIWAGGGRLADLVDRDDAP
LPEEPDTEDEALRTKWRWKVKSVKKENRERHSQRCDIELKLAVARKMKDEEGFFYPHNVD
FRGRAYPMHPHLNHLGSDICRGVLVFAEGRPLGESGLRWLKIHLANLFAGGVEKLSLEGR
IAFTENHMDDIFDSADKPLEGRRWWLNAEDPFQCLAVCINLSEAVRSSSPETSISHIPVH
QDGSCNGLQHYAALGRDELGAAAVNLVAGEKPADVYSGIAARVLDIMKRDAQRDPAEFPD
AVRARALVNQVDRKLVKQTVMTSVYGVTYIGARDQIKRRLKERGAIADDSELFGAACYAA
KVTLTALGEMFEAARSIMTWLGECAKIIASENEPVRWTTPLGLPVVQPYRKIGRHLIKTS
LQILTLQQETEKVMVKRQRTAFPPNFIHSLDGSHMMMTAVACRRAGLNFAGVHDSYWTHA
CDVDKLNRILREKFVELYETPILEKLLESFQTSYPTLLFPPLPERGDFDLRDVLESPYFF
N
>Q8L785
MAILHFSLPLIVSFLRPHASPRFFLLPRSLSQSPFLSRRRFHRTSAVSSAAVHHQSYRNP
DDDVTRAVSVPTFQQAIQRLQEYWASVGCAVMQPSNTEVGAGTMNPCTFLRVLGPEPWNV
AYVEPSIRPDDSRYGENPNRLQRHTQFQVILKPDPGNSQQLFINSLSALGIDVTAHDIRF
VEDNWESPVLGAWGLGWEIWMDGMEITQFTYFQQAGSLPLSPVSVEITYGLERIIMLLQE
VDHFKKILYADGITYGELFLENEKEMSSYYLEHASVDRLQKHFDYFDEEARSLLALGLPI
PAYDQLLKTSHAFNILDARGFIGVTERARYFGRMRSLARQCAQLWLATRESLGHPLGVAS
EPVPPVCHRAALEKVAEKVSEDPRSFIIEIGTEEMPPQDVINASEQLRVLVLELLENQRL
RHGAVKAFGTPRRLVVLVDAMSSKQLEEEVEVRGPPASKAFDDEGNPTKAAEGFSRRYGV
PLEKLYRKVSGKTEYVHARVTEPARLALEVLSEDLPGILAKISFPKSMRWNSSVMFSRPI
RWVMALHGDLVVPFSFAGISSGNVSCGLRNTASASLLVQNAESYEDTMRNSGINIEIEER
KKIILEKSNALAKSVSGRLVVPQNLLNEVANLVEAPVPLIGKFKESFLELPEELLTIVMQ
KHQKYFSIIDESGQLLPYFIAVANGAINEDVVKKGNEAVLRARYEDAKFFYEVDTRKRFS
EFRDQLQGILFHEKLGTMLDKMNRLKKMVSKLCLALKIDEDLLPVVEDAASLAMSDLATA
VVTEFTALSGIMARHYALRDGYSEQIAEALLEITLPRFSGDVIPKTDAGMVLAIGDRLDS
LVGLFAAGCQPSSTNDPFGLRRISYGLVQILVEKDKNVNFKRVLELAASVQPTKVEANTV
EDVYQFVTRRLEQLLVDNGVSPEVVRSVLAERGNNPCLAARTAYKTEKLSKGEMFPKIVE
AYSRPTRIVRGKDVGVGVEVDENAFETPQERTLWSTYTSIKDRIHTGIEIEDFTEISMQL
VEPLEDFFNNVFVMVEEERVRKNRLALLNNIANLPKGVIDLSFLPGF
>O20120
MVTLSDLESNASSSVTKNEVNTSSLVNGNGVLIITENVNKEHSVENQLASSKTEEQTLKI

SKKSNLNPAQKSSTFGLENTSPVIRPTSVFTKSTVLSTENNLVSFEVDFMNFFSALNFFL
ESLVYFVVNDFSS

>Q85A69

MSILVYEVSKSLGNLKVLDRVSLYVRKVSLVALLGPSGSGKSSLLRIIAGLDSPDYGSVW
LHGTDMTNTSTQYRHMAFVFQHYALFKNMTVYENISFGLRLRGFSYQKIRNKVNDLLDCL
RISDIVSEYPGKLSGGQKQRVALARSLAIKSDFLLLDEPFGALDGELRRHLSKWLKRYLK
DNGITTIMVTHDQKEAISMADEIVVLKQGRFLQQGRSKNLYDEPIDYFVGIFSGSFIEFP
QLEESLDAPLGSSSSSSSSTKKSMEKDFTPFIPDLIWSQIFTNQSIHHYHFFLRPHELYL
ESQIDLKAIPVQIKKIIYKRTFVQLDLSITPSSWNITIPIGYQAFRKLNIQSFVQKLYIK
PRNQVYLRAYPKKKNIISKQI

>P11673

MCLLYTHFYHLILNTRRVDSSFFVFSFHLYFLNDNKGMSPLCLSLVGSGLHLSVMHLLFH
VFLFLFDSFLRP

>P27684

MALLSPLLSLSSVPPITSIAVSSSSFPIKLQNVSVALLPSFGQRLVAHGPVIAQKRGTVV
AMVSAAAEETAGEDGDQSKVEEANISVQNLPLESKLQLKLEQKIKMKMAKKIRLRRTGLC
AKESLRKRGAWPPSKMKKLKNV

>P19593

MKRIKLKYRKLKRQQFIKVEKFNIGIDMVECHRWLENKQESIACAKREGNIVLVEKLAQE
IVNSSFGRAVAVQTVASSKGSRSPGLSRESFKTNKNYVAMMATLEQITSNPHKYKATPLS
RIYIPKRDGSARPLSIPSYTDRCLQALYKLAIEPMAEEVADLSSYGFRPMRNVSWAVGRV
LNGLNNPLANYQYVVEIDIKGCVDNINHQFISQVTPFIPKKILWAWLKCGYIERNSNTLQ
PTTTGVPQGGIISPLIMNLTLDGLEFHIYKKIQKSSSQSKGNTYCRYADDMVILTTTEET
ALIALPAVKEFLAVRGLEVKLAKTTIKNIINDRNGFEFLSFRFRKVYRRNRKRLTSQVGI
PISAIKNFRKNIKAISKTRKSLDTINDEINAVFRDCGYYYRFAHTSGYVFSSLGYWLWKQ
FYKHCYKRTKDKFDKANHTKINEIVLSSYFKPIGSGLSTKPFVIDKKGKPHSLFSIRSIE
YCPPTYTDSARNAFIFEDSVTLTKVNMRSKSSWKRVILEKWGPCCGLCRKNLEINSIPYE
LHHILPKRFGGKDTPNNMVLLCKSPCHQLVSSSIQKADVSEIQNYISLGILEIPMDYLEN
LKTSQSSY

>Q8VY26

MASLITTKAMMSHHHVLSSTRITTLYSDNSIGDQQIKTKPQVPHRLFARRIFGVTRAVIN
SAAPSPLPEKEKVEGERRCHVAWTSVQQENWEGELTVQGKIPTWLNGTYLRNGPGLWNIG
DHDFRHLFDGYSTLVKLQFDGGRIFAAHRLLESDAYKAAKKHNRLCYREFSETPKSVIIN
KNPFSGIGEIVRLFSGESLTDNANTGVIKLGDGRVMCLTETQKGSILVDHETLETIGKFE
YDDVLSDHMIQSAHPIVTETEMWTLIPDLVKPGYRVVRMEAGSNKREVVGRVRCRSGSWG
PGWVHSFAVTENYVVIPEMPLRYSVKNLLRAEPTPLYKFEWCPQDGAFIHVMSKLTGEVV
ASVEVPAYVTFHFINAYEEDKNGDGKATVIIADCCEHNADTRILDMLRLDTLRSSHGHDV
LPDARIGRFRIPLDGSKYGKLETAVEAEKHGRAMDMCSINPLYLGQKYRYVYACGAQRPC
NFPNALSKVDIVEKKVKNWHEHGMIPSEPFFVPRPGATHEDDGVVISIVSEENGGSFAIL
LDGSSFEEIARAKFPYGLPYGLHGCWIPKD

>P36211

MAATTLSIATTIRSSSFSSGLASAHHFPSRPLSIEFPFSFGVSSSSTLSHRAIYLHPISA
VKTPKKIKKIGSEISSLTLEESRILVDYVQDKFGVSILFSAPAAAALPPPLDNGGATASV
ERQTTFDVVINDVPRGNRIAVITAIRAMTSLSLSESKELIEGFPKKFKEGVTKDEAEEDK

TQLEEAGAKVSIV
>P07505
MAAHTILASAPSHTTFSLISPFSSTPTNALSSSLQSSSFNGLSFKLSPTTQSLSLSTSAA
SKPLTIVAATKKAVAVLKGTSNVEGVVTLTQEDDGPTTVNVRISGLAPGKHGFHLHEFGD
TTNGCMSTGPHFNPDKKTHGAPEDEVRHAGDLGNIVANTDGVAEATIVDNQIPLTGPNSV
VGRALVVHELEDDLGKGGHELSPTTGNAGGRLACGVVGLTPV
>Q07510
MATSYGFFSPSVPSSLNNKISPSLGINGSGFCSHLGISKRVFCSSIEASEKHAAAGVSSS
ESRVSRLVNRGCKLVGCGSAVPKLQISNDDLSKFVETSDEWIATRTGIRQRHVLSGKDSL
VDLAAEAARNALQMANVNPDDIDLILMCTSTPEDLFGSAPQVQRALGCSRTPLSYDITAA
CSGFMLGLVSAACHVRGGGFKNVLVIGADALSRFVDWTDRGTCILFGDAAGAVVVQACDS
EEDGMFAFDLHSDGGGGRHLNASLLNDETDAAIGNNGAVTGFPPKRPSYSCINMNGKEVF
RFAVRCVPQSIEAALQKAGLTSSNIDWLLLHQANQRIIDAVATRLEVPSERVLSNLANYG
NTSAASIPLALDEAVRSGKVKPGNIIATSGFGAGLTWGSSIIRWG
>Q9MTM6
MNLQAHLIVNGSNLSGLLAIGTEKPHFVFSIYRENRKESNKREGKFFYISVLRYYTYNAR
KGWGQCKKRRKSFGPGMKSNGIQG
>Q7XJM2
MSLPIPPKFLPPLKSPPIHHHQTPPPLAPPRAAISISIPDTGLGRTGTILDESTSSAFRD
YQSLFVSQRSETIEPVVIKPIEGSIPVNFPSGTYYLAGPGLFTDDHGSTVHPLDGHGYLR
AFHIDGNKRKATFTAKYVKTEAKKEEHDPVTDTWRFTHRGPFSVLKGGKRFGNTKVMKNV
ANTSVLKWAGRLLCLWEGGEPYEIESGSLDTVGRFNVENNGCESCDDDDSSDRDLSGHDI
WDTAADLLKPILQGVFKMPPKRFLSHYKVDGRRKRLLTVTCNAEDMLLPRSNFTFCEYDS
EFKLIQTKEFKIDDHMMIHDWAFTDTHYILFANRVKLNPIGSIAAMCGMSPMVSALSLNP
SNESSPIYILPRFSDKYSRGGRDWRVPVEVSSQLWLIHSGNAYETREDNGDLKIQIQASA
CSYRWFDFQKMFGYDWQSNKLDPSVMNLNRGDDKLLPHLVKVSMTLDSTGNCNSCDVEPL
NGWNKPSDFPVINSSWSGKKNKYMYSAASSGTRSELPHFPFDMVVKFDLDSNLVRTWSTG
ARRFVGEPMFVPKNSVEEGEEEDDGYIVVVEYAVSVERCYLVILDAKKIGESDAVVSRLE
VPRNLTFPMGFHGLWASD
>Q9MTM7
MLWEIRYTLRLFMERLRIIRSYLGQATPCPLTSLLYRFFLRFCFEKCYLCFIHSFHIMDH
EFKMVGLTLPFQNLKQFSENLSAPLLTLQSIPFSEYSKKIQQFSFIRTYFLIDLILSMNA
GIPIQ
>Q9MTM9
MGFDSVASGLTGLEPAASALTGRCSDQLNYNPREIKKYTYSYNCILTISI
>P82411
MSVSAIFGARVVTIPSVLRTSSVDGRTVKLQPSTGGSCGGGVITIECSSRPQKKGTAHHM
KTRPKKTARWDIKRGPAVYPPLPPLPAEWTIVSSAVDEADSSSSTTSSSAEIAQSA
>Q06SI4
MGQKIHPYGYRVGITQPHSARWFANTYQYPQYVFEDFLLRQSLFKNLPVENLPRKRTEDK
SETKLVQIKIERLIRNTIKIKLYVTSPENASELFEHEALTKSPKKDFGTTNSNKRNLQTN
LESKKDRNTLRLNIQKRLLNKKLLQLQILKLQNMQSKLVTMKNLLDGSQNTNKKVFSSSV
FVQKNETETIVNNYPFYLNNIAINYSIGKTLIEGIKDCLNMNLDGKKKVKYLFYFLQLKK
QLSKTLLYQLDVYQKVEKQKLVNFTGLNKSEEQISAFQNTINLRLFKLKALSLKWTNHSR

HLTNVLVSIQSKLTLELETLVSKLDQNKTNLNQKCATLINILKFKYMLRKNNFNKLELFV
LNQKIEEIKLKWFQSLTIAGTVQAETDSQSDFYSSKKILLELNMISCEKQKMLTLLTKFK
FSLNFYNTHLEKTKRIILAGLFLLSKFGWKKEIFNGLNTFLSQSVLIIKKLETHLGKNYK
ILQERIIVKINHMTILESTIKQNLNLLQILKIAQDHNTNRDKIKVSQLYKNLFKVFYLTS
LSSTPAFHETLTNKTFLYDLVSESLDKYALLISQKVINLNSSLICYSEEKTTQIPVYNDK
FLNILTREYLSNLIRLFKNDSINYYKSKALYQFSLALLNQRYKLNKIIEMKIKNLLLQIK
PEEKTKNTFLVSIRDLNISLKSNTLFIKQLETWLTLLKKKLTENTATNRYLLQARIHKIN
CYLKKIIPVKITENNLVSTIEKNFIFVNYNILKQKLLAELLQMQQTIILAKKEEWEKEES
LSFPPSTSFFTKTNISIGNLNQTLQILQNRILDLQGRFNQENQQKTAGKTVNTDLSPILQ
VENKTNVSKTLQLKTTLQQLLKTNYENSFIVLQTRRQVYNSIINRLGGHRKFGKSRAGEK
ENIWKNPYETAEYLDFKLKKVNNSTDTNSLTTLLETIKQKIYERKPIKLMYTRKQKFLAK
KTIKKKLKHFLVLMALKNNYTNLSSLKNIYDVKTFAKLCENLTNLQTMPKVSVIELVKVH
QPKQYAVCLANFVVENLEKRFSFRSTMKRASEQAMSTANVKGIKIQISGRLNGAEIARTE
WLRDGVVPLQTLRANIDYSYKTAKTIYGILGVKVWLFKDTPANMNS
>P82412
MLSMAVQPNINAIAKPSIYQSPKLSLKPFKTPAFANPKPFFSSPSFSQLKKKNNWSLFVA
PETISDVAIMGNEVDIDDDLLVNKEKLKVLVKPMDKPRLVLKFIWMEKNIGLALDQTIPG
HGTVPLSPYYFWPRKDAWEELKVLLENKPWISQKQMIILLNQATDIINLWQQSGGNLAS
>Q96255
MAATTNSFLVGSNNTQIPALKPKSSSQSFLHLSKPNTVNFVSKTKPVAVRCVASTTQVQD
GVRSGSVGSQERVFNFAAGPATLPENVLLKAQADLYNWRGSGMSVMEMSHRGKEFLSIIQ
KAESDLRQLLEIPQEYSVLFLQGGATTQFAALPLNLCKSDDTVDFVVTGSWGDKAVKEAK
KYCKTNVIWSGKSEKYTKVPSFEELEQTPDAKYLHICANETIHGVEFKDYPVPKNGFLVA
DMSSNFCSKPVDVSKFGVIYGGAQKNVGPSGVTIVIIRKDLIGNAQDITPVMLDYKIHDE
NSSLYNTPPCFGIYMCGLVFEDLLEQGGLKEVEKKNQRKADLLYNAIEESNGFFRCPVEK
SVRSLMNVPFTLEKSELEAEFIKEAAKEKMVQLKGHRSVGGMRASIYNAMPLAGVEKLVA
FMKDFQAKHA
>P46416
MGSGCSSLSYSSSSTCNATVFSISSSPSSSSSLKLNPSSFLFQNPKTLRNQSPLRCGRSF
KMESQKPIFDLEKLDDEFVQKLVYDALVWSSLHGLVVGDKSYQKSGNVPGVGLMHAPIAL
LPTAFPEAYWKQACNVTPLFNELIDRVSLDGKFLQDSLSRTKKVDVFTSRLLDIHSKMLE
RNKKEDIRLGLHRFDYMLDEETNSLLQIEMNTISCSFPGLSRLVSQLHQSLLRSYGDQIG
IDSERVPINTSTIQFADALAKAWLEYSNPRAVVMVIVQPEERNMYDQHLLSSILREKHNI
VVIRKTLAEVEKEGSVQEDETLIVGGQAVAVVYFRSGYTPNDHPSESEWNARLLIEESSA
VKCPSIAYHLTGSKKIQQELAKPGVLERFLDNKEDIAKLRKCFAGLWSLDDSEIVKQAIE
KPGLFVMKPQREGGGNNIYGDDVRENLLRLQKEGEEGNAAYILMQRIFPKVSNMFLVREG
VYHKHQAISELGVYGAYLRSKDEVIVNEQSGYLMRTKIASSDEGGVAAGFGVLDSIYLI
>P82413
MASKVLPQALLVIPSNHSLQCPPLKKQLGFPIDSNRRFSLSSNCRSNLMVSRASSNLFSS
NFSSIFSFPARNSFVVRSEAEDSSDAPAESVAVVAEEELPVESEAEAEERPPRQQRVKLG
DIMGILNKKAVHAAEELRPVPGIRTGDIVQIRLEVPENKRRLSVYKGIVISRQNAGIHTT
IRIRRIIAGVGVEIVFPLYSPNIKEIKVVSHRKVRKARLYYLRDKLPRLSTFK
>Q9CAF5
MAGVNLQLRHAYSIAQFVPTVSSPPPLPTQRVRLGTSPSRVLLCNLRANSAAAPILRTTR

RSVIVSASSVSSAVDSDSLVEDRDDVGRIPLLEVRDLRAVIAESRQEILKGVNLVVYEGE
VHAVMGKNGSGKSTFSKVLVGHPDYEVTGGSIVFKGQNLLDMEPEDRSLAGLFMSFQSPV
EIPGVSNMDFLNMAFNARKRKLGQPELDPIQFYSHLVSKLEVVNMKTDFLNRNVNEGFSG
GERKRNEILQLAVLGAELAILDEIDSGLDVDALQDVAKAVNGLLTPKNSVLMITHYQRLL
DYIKPTLIHIMENGRIIKTGDNSLAKLLEKEGYKAISG

>Q3BAI2

MQVVRIFTDMSISPSLSPRQCPDRYAFRAGRNLPDKEFRYLRTVLVTAAVHRGFGRRLPC
HQVTNFLDLPALGRRQPPYMVLRLCGDLCFW

>Q9CAF6

MTPVLCHSTASIPNPNSLMSLSSTLRLSSSLLRRSFFRFPLTDPLCRLRRTEPSATRFFS
SRTPRSGKFVVGAGKRGDEQVKEESGANNGGLVVSGDESRIVPFELHKEATESYMSYALS
VLLGRALPDVRDGLKPVHRRILFAMHELGMSSKKPYKKCARVVGEVLGKFHPHGDTAVYD
SLVRMAQSFSLRCPLIQGHGNFGSIDADPPAAMRYTECRLDPLAEAVLLSDLDQDTVDFV
ANFDNSQKEPAVLPARLPALLLNGASGIAVGMATNIPPHNLGELVDVLCALIHNPEATLQ
ELLEYMPAPDFPTGGIIMGNLGVLDAYRTGRGRVVVRGKAEVELLDPKTKRNAVIITEIP
YQTNKATLVQKIAELVENKTLEGISDIRDESDRNGMRVVIELKRGGDPALVLNNLYRHTA
LQSSFSCNMVGICDGEPKLMGLKELLQAFIDFRCSVVERRARFKLSHAQQRKHIIEGIVV
GLDNVDEVIELITKASSHSSATAALQSEYGLSEKQAEAILEITLRRLTALERKKFTDESS
SLTEQITKLEQLLSTRTNILKLIEQEAIELKDRFSSPRRSMLEDSDSGDLEDIDVIPNEE
MLMAVSEKGYVKRMKADTFNLQHRGTIGKSVGKLRVDDAMSDFLVCHAHDHVLFFSDRGI
VYSTRAYKIPECSRNAAGTPLVQILSMSEGERVTSIVPVSEFAEDRYLLMLTVNGCIKKV
SLKLFSGIRSTGIIAIQLNSGDELKWVRCCSSDDLVAMASQNGMVALSTCDGVRTLSRNT
KGVTAMRLKNEDKIASMDIIPASLRKDMEEKSEDASLVKQSTGPWLLFVCENGYGKRVPL
SSFRRSRLNRVGLSGYKFAEDDRLAAVFVVGYSLAEDGESDEQVVLVSQSGTVNRIKVRD
ISIQSRRARGVILMRLDHAGKIQSASLISAADEEETEGTLSNEAVEAVSL

>P22178

MISLNSSFLERSSVTGGSRTQSQSLRLSARRPVVTSMLNSNSLPERNVSVSVDSAVRDVN
APVAVEVDRSVGEKPFAAVGGGVEDMYGEDTATEDHYITPWSVSVASGYSLLRDPHHNKG
LAFTEKERDAHFLRGLLPPVVVNHDLQVKKMMHNIRQYQVPLQRYQAMMDLQQRNERLFY
KLLIENVEELLPIVYTPTVGEACQKYGSIFENSQGLFISLKDKGRILEILKNWPHKKIQV
IVVTDGERILGLGDLGCQGMGIPVGKLALYTALGGVRPSACLPITIDVGTNNEKLLNDDE
FYIGLKQKRAAGQEYAELMNEFMSAVKQNYGENLLIQFEDFANHNAFDLLEKYRTTHLVF
NDDIQGTASVVLGGLISALKLVGGSLADQKFLFLGAGEAGTGIAELIALEISKQTNIPLE
ESRKKVWLVDSKGLIVRSRLDSLQHFKKPWAHDHEPVNEFLDAIKTIRPTVLIGSSGTGQ
TFTKEVVETMSSLNEKPIILALSNPTSQSECTAEQAYTWSEGRAIFASGSPFKPVEYNGK
LYVSGQANNAYIFPGFGLGLIISGAIRVHDDMLLAASEAPAEQVTQEHFDKGLIFPPFTS
IRKISAHIAAKVAAKAYELGLASRLPQPENLVAYAESCMYSPKYRIYR

>P12629

MATMACASSLTFPSAQTQKSFFGTNVKQTPVLSFPRPTVAAAVAVSARKSTSASTKCTEE
WRQLKEAVKKEFAIPHVPLDQRWMFTLEEATGPDIWNTTWYPKSADHVPTDKKWYVVDAT
DLILGRMASTIAIHIRGKNLASYTPSVDMGAFVIVVNADKVAVSGKKRTQKLYRRHSGRP
GGLKEETFDQLQKRIPERIIEHAVRGMLPKGRLGRYLFNHLKVYKGAEHPHQAQQPIDLP
LRDKRIRVEK

>O20130

MKKEFLHFLAFLITFVFCVKTQQVVKILRSRTMIDFGLEFQKAQIEMHNNPEDRVRFVDA
VNLCLPASDTFFQNPDRENKFSENYSIKLIDTTPHVSEIVLQTFVKEILSLSNINTEDTL
GKETLGKETYRLSNLLKKSKFGMESHHRIQNNSEGPNTFENGLGISTPKHGIAHFADAIQ
RFLDHKGNSSSNTRAAGARVEAIREALNDRDAINKSEEARKAREEVFIPSEPSKPSIASK
RSSASKSTKS
>Q9LER7
MALLCFNSLPSLSSLSSSSSSRLLQSPSFASPVLSLKPNAVESKNRVSLSAYSLNSSHGR
IVVKAAASGVDGAEPESKEEPKTVVAAVPVDKLPLESKEAKEKLLLELRLKMKLAKKIRL
RRKRLVRKRKMRKKGRWPPSKMKKNKNV
>O20133
MRAKSIAYVWSTATQSLTPEQCEAMIKLREPLNVVFDQGMRNVQLILKKQDHFDELMTTY
ETCQTAEKNFHETTFLPAKANLDTFVGSQKARKEAANHFKKIKSQSIEYTKEANQAFTAA
MKYDQNHIVPAKNALQKIETCIINEVEDKLEEILNPSSITEKTLKGKTQRQKTNAALINR
IKRRNTAFNNHDNNIINSFHGPSSFEQISIYYNLPIDRSFQKMVQKQLEKIKVLPN
>Q9MTN0
MEPEISTDSCRVKKNMKSKKPAVPISSLSNLNIRIVDIVMILWARSTYFLFFFLLISHF
>P52334
MMRTVQLRTLRPCIRAQQQPVRAPTSVAAATATTPAPTKKCPFSLFAKLGGREAVEAAVD
KFYNKVVADPTVSVFFSKTDMKVQRSKQFAFLAYALGGAAEWKGKDMRTAHKDLVPHLTD
VHFQAVVRHLSDTLAELGVTPGDIADAMAVVASTKTEVLNMPRQQGAESNR
>Q9MTN3
MGSAKWDSRALSFYDTKKPGKPHTIKQHLHRRKGERGIRTLDNSLLRTMPVFKTGAINHS
AISPRDNFYYIFIPPNRTWPYQLIPSLSIDRIPTGYLSIVNLSRYTVQINA
>Q9MTN4
MRELRIPTRKTNPIHNDVPENTTFLLLDQPSGEANTTGTLISKSDEVAINAKTANWKAIV
MLLILQATNKRIIPF
>Q40545
MSQALNFFVSSSSRSPATFTISRPSVFPSTGSLRLLVKKSLRTLVVEASSAAASDLDEPQ
SSPVLVSENGSGGVLSSATQEYGRNAAPGTDSSSIEVDTVTEAELKENGFRSTRRTKLIC
TIGPATCGFEQLERLAEGGMNVARINMCHGTREWHRMVIERLRRLNEEKGFAVAIMMDTE
GSEIHMGDLGGASSAKAEDGEIWNFTVRSFDPPLPERTVTVNYDGFAEDVKVGDELLVDG
GMVRFEVIEKIGPDVKCLCTDPGLLLPRANLTFWRDGKLVRERNAMLPTISSKDWLDIDF
GIAEGVDFIAVSFVKSAEVIKHLKSYIQARARDSDISVIAKIESIDSLKNLEEIIQASDG
AMVARGDLGAQIPLEQVPSEQQKIVQICRQLNRPVIVASQLLESMIEYPIPTRAEVADVS
EAVRQRGDALMLSGESAMGQFPEKALTVLRSVSLRIERMWREQKRHEVIELPSIASSFSD
SISEEICNSAAKMANNLEVDALFVYTKNGHMASLLSRCRPDCPIFAFTTTTSVRRRLNLQ
WGLMPFRLSFSDDMESNLNKTFSLLKARGMIKSGDLIIAVSDMLQSIQVMNVP
>Q9MTN9
MFVGPTILLSSDLDKQGYQISNKVRLLIPRRSASSSMPPIGTHGAGLTCNT
>Q85FG9
MSIRLEDIDPVVVSMGPHHPSMHGVLRLVVALQGENVVDCELILGYLHRGMEKIAENRTT
SQYLPYVTRWDYLATMFTEAVTVNAPEKLANIQIPERASYIRVIMLELSRIASHLLWLGP
FLADIGAQTPFFYIFREREMIYDLFEAATGMRMMHNYFRIGGVAADLPYGWIDKCLDFCQ
YCLPKIYEYERLVTKNPIFLKRVQGVGFINRQEAINWGLSGPSLRASGVQWDLRKIDHYE

CYDKLDWQIQWQGEGDSLARYLVRIDEMKESINILRQALKLLPGGPYENLEGRRLVQKKD
KIDWNGFNYQFVGKKSSPTLKLPKQEHYVRVEAPKGELGIFLIGDDSVFPWRLKIRPPGF
INLQIVPQLIRGMKLADIVTILGSIDIIMGEVDR
>Q06SJ3
MSRYLGPRLRITRRLGHLSGLTRKKPAFKPLNPVNPFGPRKIIPPGEHGRNKSFKKKPYE
SCEYDYLIRLKLKQRLRFHYGLTERQLVRYVQQAKKIKGSTGRVLLRLLEMRLDNIVFRL
HMAPTIKAARQLISHGHILINKKKVTIPSYQCEPKDIITVAPKIISMELVSRFLSEFDRE
KSRYDRILQILEFGRKGVTMPTKNLKTSSKTLSKNSQSRKYVKNEKRAKIQSLKIGAVLN
VTINHRGRKEADAISYGFGKMIVIHPFFVGKQSINKNVRVIIYKKSKNNKILYTYPANPF
YLHLENLRKLNSLDVAKLFSHSNNQISSKFNKKPLKKSITRKSKRSISALILMNGAKMLP
KTNLERRKRVNKDSATNLTENKQIKKEFSPSVFVRIVAAKCLNSKSKQKLLNVSSFQSSL
KKFNPSFSKDPVAYREKYVYTKTSRALRLFGTRFYATILKNTRDVKNSSFIERKKFDSRI
IGKTPKVNLTKTTRKAEAKAPDFYSSIFKKSSDNQSQNFLNAELLKRESQNSSFSSTFNN
SNKVEFSNGASLPVTFKSGKVNNTLIPKLNSNKLADQSVNAQLMNVPALFKQYKNIFSKL
SSLKNQKNFKKPKYTNLRSKILTNFLFESKNLSLNLNHFHNFIFVVFSKFCKVLVSKTNL
NFKIDNILSISQNFKEFFKHRNFEAKSIDSKKFSMDILLFSLKLKHFTKLNNKNVGTEIG
KTREFELSSSILDKKIQNDISLLLLLDKMKTHLQTSLNFVNQFFSSEILMNGIRPYFSSI
FSFVLKSEELVKTILTNVINSIKTVLTCSKLTTLKISQQSFLGSAASEKLEKTESFFLEN
NLIEKLVNLNSNQSNTLYKKIINFAMVSIPKAVFLVDYSENQISLLNKYSLYSAKKQKIQ
MVKTLNFLKRYNLINYSNFENFKQMIQLNIKNHQTILVHFKDSILNKSKLNLRRNKLSPD
SELVLNLEQKLLINQTKCKISLLESLKSNSKLSLFSQFNQNCNQILCRTKIFLNILNKKN
SLFQKLNLLKDFHLIKGKDYESLKNLLNNQFQLLQKLKSLVSIVDFPQSVSQSSLLNRVL
NKNLEKITETFGSISTLWKVLILQKQNKLNVTESVKQNILQNISVAKAEFTQTVLITILN
KTDLSAFIKHYQSSKINNFTFISKLHKINLLDKLNKLNLLDDTVFVQMFSDIREKLAVKK
LKQTNVVLTKFLEFGTMSKLSQTELLSLDSFIQTAFLNSNSLTNNIQHLGHKERDNFVRS
SSFWKKFSFLLTPSLIKKGLENLKTLNSISSSQYSYLILKLQNLYDQNKTLNLGLNKQYV
HEKLRIIKTLLVLIAKNVNITTLTSLNLFKNTTWGSKTLLNLGLNRQKELKLMSGLLISQ
KIKYSSSFQKWEKDEFSFLMTNYIENQYNYLISKILMRGILTKEEAYFYSRENCQDYLTE
KLSFLHSETKQILTSIKLYLLRYQFMVQNNKNSSFSPWNLRFQEENFSLKSSISERTLNS
SRIFSLKKQKSVLLDKTMYQKLKNFLQKEILSKNMKSLLENKHFILAYLNKELKAKLFYK
VSRLQRKNTLLTETEIYEFSNTFKKLTASNLSVKLATNLSTLIAPTNLSTNFTKKIFKIY
KSTNIPVYYNLLSNDTTFLDNKIDNVYLSKVLNKLKLQLNNNKLDLLMVNYTNDKLLRLR
AKSSMYQTFAKLAFIENGMILNKHLFPVRSAKSVYLKQFNKNLKFVIYLYNLNMLRSRDI
LSVQQYEQFKDNYENIFKLIKRKTFVISILNKRKKWKFINYGTYQTLFKNISKNLTTKIL
SLFQQSTEKNFFQKEKVNKQVQYSLEVEALVQQTLEQLVNSSESPNHSLTSLIYRFIEKS
IVFTHPSSNSLKQLTIENKTVIKPVIKKALQRLISLQKKLKSLGPWSSKEKIMLQNKQKT
LILQSLVSYLQKQETPFNSNFKTVSNKFKQISMFQTLSFAQRKTVLNKLCLNTYFTPNLQ
TISNAKDLSNEKKSRALALSGQLTNLQFKLQKEYLTKLNVSLLKAEKNESDLISFNESIL
LMLQKTTGVDLTNYKQFSRIVTNNSNIDKGSLAKEIYTTKLLFEFIRQNLMDNYRKDKFN
FELKQVSKFQKSQFLTNILFKGGSFSSRLSLKKCQNNLIKLKISKLLNHIFSVMPFVNNQ
TTTTGFISYDHIDTLVSFGIISSTMANVLNKKINVQIQKQKLRKTLLSLQNLQKMTFTSS
NKFNRLIYSSILMDVFSRLYELKKAKTITERKYVLIKQKLKIFSLFSALNYKLLDLKEKG
SISSSKALELKNQIIQKISQKMKKVKTFAKFKQSLKALKGENFSSRLSYLPKDLDSQKTL
EKAKAFEFNPSLLKILKSRGRWARVTVKQLVKQKLLTTKQQEKLQTIVDKQNLMKMKKLR

RLVSVFVYCRQIVETQRNTVANNSNYEPLMQNVISSVLKSFNGPWKRVLLNLLYKQNFIS
ENLFLSYLTNNTQKTKSSKTKVISLNNNVDTTYTKTKLKRLLTMYYKQICKLRQFQTNRE
ILKGEFEQKLSAILSSVILVLEKGGLDAFKVIYNTKWVNELCQSNLKINNQKNKNSQISS
AIREFVSKYNFLKDQYLNTYKKLQLNDKMKLFKMYKTNLLQELVNLEKTIKSPNAQSFEK
SEILSISRLEQLKMQGLISTKICNKLTVMLNNSLQRLIKLDRLFALQNLYLVTSEAKAIS
ENASYMNTGAVLEANSSKVQIQTESNIIQNNEQYIKLKQKIFQSYFRYEYKHIEKSVIKQ
KLLLQRLDSYNLKQKTTKQYKTNLRRKKSKLLSSEQFNSFFQQLLNFLDSRYKSAGRNRR
NPRINSIIRRLNQKLSFDKTLTKKFGDHLQTFIDKRFGPALPIPPHLELKRWKIKTSKLQ
SKQKLNLKYFILPVGIVRDLAPRRSVGLPILERLIVEYYSRN
>Q42588
MATCIDTCRTGNTQDDDSRFCCIKNFFRPGFSVNRKIHHTQIEDDDDVWIKMLEEAKSDV
KQEPILSNYYYASITSHRSLESALAHILSVKLSNLNLPSNTLFELFISVLEESPEIIEST
KQDLIAVKERDPACISYVHCFLGFKGFLACQAHRIAHTLWKQNRKIVALLIQNRVSESFA
VDIHPGAKIGKGILLDHATGVVIGETAVVGDNVSILHGVTLGGTGKQSGDRHPKIGDGVL
IGAGSCILGNITIGEGAKIGSGSVVVKDVPARTTAVGNPARLIGGKENPRKHDKIPCLTM
DQTSYLTEWSDYVI
>P28645
MALNLNPVSTPFQCRRLPSFSPRQTPSRRSPKFFMASTLSSSSPKEAESLKKPFSPPREV
HVQVTHSMPQEKIEIFKSLEGWAEENLLVHLKPVEKCWQPQDYLPDPASEDFRDQVKEIQ
ERAKEIPDDLYVVLVGDMITEEALPTYQTMLNTLDGAKDETGASPTSWAVWTRAWTAEEN
RHGDLLNKYLYLSGRVDMRSIEKTIQYLIGSGMDPRTENNPYLGFVYTSFQERATFVSHG
NSARLAKEHGDLKMAQICGIIASDEKRHETAYTKIVEKLFEIDPDATVLAFADMMKKKIS
MPAHLMYDGRDDNLFDHFSAVAQRLGVYTAKDYADILEFLVGRWEVEKLTGLSSEGQKAQ
DYVCSLPPRIRRLEERARERAKQAPSMPFSWIFDRQVKL
>A1E9W1
MIQPQTLLNVADNSGARKLMCIRVIGAAGNQRYARIGDVIIAVIKDAVPKMPLERSEVIR
AVIVRTRKEFKGDDGIIIRYDDNAAVIIDQKGNPKGTRVFGAVAEELRELNFTKIVSLAP
EVL
>Q9FV52
MASSVFLSSFSSSSSLQLCSSFHGEYLAPSRCFLGAPVTSSSLSLSGKKNSYSPRQFHVS
AKKVSGLEEAIRIRKMRELETKSKVRRNPPLRRGRVSPRLLVPDHIPRPPYVESGVLPDI
SSEFQIPGPEGIAKMRAACELAARVLNYAGTLVKPSVTTNEIDKAVHDMIIEAGAYPSPL
GYGGFPKSVCTSVNECMCHGIPDSRQLQSGDIINIDVTVYLDGYHGDTSRTFFCGEVDEG
FKRLVKVTEECLERGIAVCKDGASFKKIGKRISEHAEKFGYNVVERFVGHGVGPVFHSEP
LIYHYRNDEPGLMVEGQTFTIEPILTIGTTECVTWPDNWTTLTADGGVAAQFEHTILITR
TGSEILTKC
>A4QJK7
MSPQTETKASVGFKAGVKEYKLTYYTPEYETKDTDILAAFRVTPQPGVPPEEAGAAVAAE
SSTGTWTTVWTDGLTSLDRYKGRCYHIEPVPGEESQFIAYVAYPLDLFEEGSVTNMFTSI
VGNVFGFKALAALRLEDLRIPPAYTKTFQGPPHGIQVERDKLNKYGRPLLGCTIKPKLGL
SAKNYGRAVYECLRGGLDFTKDDENVNSQPFMRWRDRFLFCAEAIYKSQAETGEIKGHYL
NATAGTCEEMIKRAVFARELGVPIVMHDYLTGGFTANTSLAHYCRDNGLLLHIHRAMHAV
IDRQKNHGMHFRVLAKALRLSGGDHIHAGTVVGKLEGDRESTLGFVDLLRDDYVEKDRSR
GIFFTQDWVSLPGVLPVASGGIHVWHMPALTEIFGDDSVLQFGGGTLGHPWGNAPGAVAN

RVALEACVQARNEGRDLAVEGNEIIREACKWSPELAAACEVWKEIRFNFPTIDTIDKLDP
SVDKVA
>P25306
MEFLCLAPTRSFSTNPKLTKSIPSDHTSTTSRIFTYQNMRGSTMRPLALPLKMSPIVSVP
DITAPVENVPAILPKVVPGELIVNKPTGGDSDELFQYLVDILASPVYDVAIESPLELAEK
LSDRLGVNFYIKREDKQRVFSFKLRGAYNMMSNLSREELDKGVITASAGNHAQGVALAGQ
RLNCVAKIVMPTTTPQIKIDAVRALGGDVVLYGKTFDEAQTHALELSEKDGLKYIPPFDD
PGVIKGQGTIGTEINRQLKDIHAVFIPVGGGGLIAGVATFFKQIAPNTKIIGVEPYGAAS
MTLSLHEGHRVKLSNVDTFADGVAVALVGEYTFAKCQELIDGMVLVANDGISAAIKDVYD
EGRNILETSGAVAIAGAAAYCEFYKIKNENIVAIASGANMDFSKLHKVTELAGLGSGKEA
LLATFMVEQQGSFKTFVGLVGSLNFTELTYRFTSERKNALILYRVNVDKESDLEKMIEDM
KSSNMTTLNLSHNELVVDHLKHLVGGSANISDEIFGEFIVPEKAETLKTFLDAFSPRWNI
TLCRYRNQGDINASLLMGFQVPQAEMDEFKNQADKLGYPYELDNYNEAFNLVVSE
>P52410
MQALQSSSLRASPPNPLRLPSNRQSHQLITNARPLRRQQRSFISASASTVSAPKRETDPK
KRVVITGMGLVSVFGNDVDAYYEKLLSGESGISLIDRFDASKFPTRFGGQIRGFSSEGYI
DGKNERRLDDCLKYCIVAGKKALESANLGGDKLNTIDKRKAGVLVGTGMGGLTVFSEGVQ
NLIEKGHRRISPFFIPYAITNMGSALLAIDLGLMGPNYSISTACATSNYCFYAAANHIRR
GEADMMIAGGTEAAIIPIGLGGFVACRALSQRNDDPQTASRPWDKARDGFVMGEGAGVLV
MESLEHAMKRGAPIVAEYLGGAVNCDAHHMTDPRADGLGVSSCIERCLEDAGVSPEEVNY
INAHATSTLAGDLAEINAIKKVFKSTSGIKINATKSMIGHCLGAAGGLEAIATVKAINTG
WLHPSINQFNPEQAVDFDTVPNEKKQHEVDVAISNSFGFGGHNSVVAFSAFKP
>P41347
MTSTVTTTVGCGGLPVRPLSTATRGRPRRCAVRAQAAGADASNDKSVEVMRKFSEQYARR
SNTFFCADKTVTAVVIKGLADHRDTLGAPLCPCRHYDDKAAEVAQGFWNCPCVPMRERKE
CHCMLFLTPDNDFAGKDQVISFEEIKEATSKF
>Q9T052
MAYHPVYNETMSMGGGSSNEFGQWLDKQLVPFDTSSGSLRVELLHGNLDIWVKEAKHLPN
MDGFHNTLVGGMFFGLGRRNHKVDGENSSKITSDPYVTVSISGAVIGRTFVISNSENPVW
MQHFDVPVAHSAAKVHFVVKDSDIIGSQIIGAVEIPTEQLCSGNRIEGLFPILNSRGKPC
KQGAVLSLSIQYIPMERMRLYQKGVGFGVECVGVPGTYFPLRKGGRVTLYQDAHVDDGTL
PSVHLDGGIQYRHGKCWEDMADAIRRARRLIYITGWSVFHPVRLVRRNNDPTQGTLGELL
KVKSQEGVRVLVLVWDDPTSRSLLGFSTKGLMNTSDEETRRFFKHSSVQVLLCPRYGGKG
HSFIKKSEVETIYTHHQKTMIVDAEAAQNRRKIVAFVGGLDLCNGRFDTPKHPLFRTLKT
IHKDDFHNPNFVTTADDGPREPWHDLHSKIDGPAAYDVLANFEERWMKASKPRGIGRLRT
SSDDSLLRLDRIPDIMGLSEASSANDNDPESWHVQVFRSIDSSSVKGFPKDPKEATGRNL
LCGKNILIDMSIHAAYVKAIRSAQHFIYIENQYFLGSSFNWDSNKNLGANNLIPMEIALK
IANKIRAREKFAAYIVIPMWPEGAPTSNPIQRILYWQHKTMQMMYQTIYKALVEVGLDGQ
LEPQDFLNFFCLGTREVGTREVPDGTVSVYNSPRKPPQLNAAQVQALKSRRFMIYVHSKG
MVVDDEFVLIGSANINQRSLEGTRDTEIAMGGYQPHHSWAKKGSRPRGQIFGYRMSLWAE
HLGFLEQEFEEPENMECVRRVRQLSELNWRQYAAEEVTEMPGHLLKYPVQVDRTGKVSSL
PGYETFPDLGGKIIGSFLVVEENLTI
>O20142
MTHRFRNTTFMRYLSNSRFKKKLILRKIKVSGPFVIQRTVHPQSAHNPNENCGNFDQRKV

LGYFFKMFFYHNYNFVFTKLKLICLFLIISISSKKNFKLKVLILFFSIQKFTKSLDRNMS
LAFDCTHKRVT
>O82782
MRTLSSQLYSNGGLTWFQKKNQSSLFIKHLRVSKPSRVQLISAVQFRPCIDIHKGKVKQI
VGSTLRDLKEDGSVLVTNFESDKSAEEYAKMYKEDGLTGGHVIMLGADPLSQAAAIGALH
AYPGGLQVGGGINSENCMSYIEEGASHVIVTSYVFNNGKIDLERLKDIVSIVGKQRLILD
LSCRKKDGRYAIVTDRWQKFSDVILDEKSLEFLGGFSDEFLVHGVDVEGKKLGIDEELVA
LLGNYSPIPVTYAGGVTVMDDVERIKDAGKGRVDVTVGSALDIFGGNLPYKDVVAWHHKQ
HSLH
>O20143
MIKKHFKKITEHFSLVKISAILVGVVCGLRVYCSLNNKGAANLNFSQDEFFFKPTVAQIA
HESRVSKPMSHFFDEANTGNSNEERKVMFLSPINPATSSASPKKFKWVNTISIGPSLSLN
QNSVSSSVNTSPAREKISEIREKVKEFGEELASNDLVPTQEDVRGWEDLGNRYLEYLELK
LKPQLEQHNLAGFQFVNYGNLAERAKDMLANNTLCGLKVVKTNGESKLISISQFNKIFTI
LGENQLELEHHTISTLQDVRLMAQGVQQKQAVAMRLEPTLQNLLTTLRNQISNFEKTENW
TIEPSFKNLMVYTAPSPFRLQGSMCCHLGEKSLSLGVGETTFTQFSESNPHFGLITGWNF
GPKFSFGQETGLVSFSGYGIHAFCGLGFRI
>P30924
MEINFKVLSKPIRGSFPSFSPKVSSGASRNKICFPSQHSTGLKFGSQERSWDISSTPKSR
VRKDERMKHSSAISAVLTDDNSTMAPLEEDVKTENIGLLNLDPTLEPYLDHFRHRMKRYV
DQKMLIEKYEGPLEEFAQGYLKFGFNREDGCIVYREWAPAAQEDEVIGDFNGWNGSNHMM
EKDQFGVWSIRIPDVDSKPVIPHNSRVKFRFKHGNGVWVDRIPAWIKYATADATKFAAPY
DGVYWDPPPSERYHFKYPRPPKPRAPRIYEAHVGMSSSEPRVNSYREFADDVLPRIKANN
YNTVQLMAIMEHSYYGSFGYHVTNFFAVSSRYGNPEDLKYLIDKAHSLGLQVLVDVVHSH
ASNNVTDGLNGFDIGQGSQESYFHAGERGYHKLWDSRLFNYANWEVLRFLLSNLRWWLEE
YNFDGFRFDGITSMLYVHHGINMGFTGNYNEYFSEATDVDAVVYLMLANNLIHKIFPDAT
VIAEDVSGMPGLGRPVSEGGIGFDYRLAMAIPDKWIDYLKNKNDEDWSMKEVTSSLTNRR
YTEKCIAYAESHDQSIVGDKTIAFLLMDKEMYSGMSCLTDASPVVDRGIALHKMIHFFTM
ALGGEGYLNFMGNEFGHPEWIDFPREGNNWSYDKCRRQWNLADSEHLRYKFMNAFDRAMN
SLDEKFSFLASGKQIVSSMDDDNKVVVFERGDLVFVFNFHPKNTYEGYKVGCDLPGKYRV
ALDSDAWEFGGHGRTGHDVDHFTSPEGIPGVPETNFNGRQIPSKCCLLREHVWLITELMN
ACQKLKITRQTFVVSYYQQPISRRVTRNLKIRYLQISVTLTNACQKLKFTRQTFLVSYYQ
QPILRRVTRKLKDSLSTNIST
>P0C586
MSSAVVASSTTFLVALASSASRGGPRRGRVVGVAAPPALLYDGRAGRLALRAPPPPRPRP
RRRDAGVVRRADDGENEAAVERAGEDDEEEEEFSSGAWQPPRSRRGGVGKVLKRRGTVPP
VGRYGSGGDAARVRGAAAPAPAPTQDAASSKNGALLSGRDDDTPASRNGSVVTGADKPAA
ATPPVTITKLPAPDSPVILPSVDKPQPEFVIPDATAPAPPPPGSNPRSSAPLPKPDNSEF
AEDKSAKVVESAPKPKATRSSPIPAVEEETWDFKKYFDLNEPDAAEDGDDDDDWADSDAS
DSEIDQDDDSGPLAGENVMNVIVVAAECSPWCKTGGLGDVAGALPKALARRGHRVMVVVP
RYGDYAEAQDVGIRKYYKAAGQDLEVKYFHAFIDGVDFVFIDAPLFRHRQDDIYGGNRQE
IMKRMILFCKAAVEVPWHVPCGGVPYGDGNLVFLANDWHTALLPVYLKAYYRDNGMMQYT
RSVLVIHNIAYQGRGPVDEFPYMELPEHYLDHFKLYDPVGGEHANIFGAGLKMADRVVTV
SPGYLWELKTTEGGWGLHDIIRENDWKMNGIVNGIDYREWNPEVDVHLQSDGYANYTVAS

LDSGKPRCKAALQRELGLEVRDDVPLIGFIGRLDGQKGVDIIGDAMPWIAGQDVQLVLLG
SGRRDLEVMLQRFEAQHNSKVRGWVGFSVKMAHRITAGADVLVMPSRFEPCGLNQLYAMA
YGTVPVVHAVGGLRDTVSAFDPFEDTGLGWTFDRAEPHKLIEALGHCLETYRKYKESWRG
LQVRGMSQDLSWDHAAELYEEVLVKAKYQW
>Q9MUL5
MSNTRLTLFFTNIFQLLDSKIQNYSINFPIKILLLLLGFFIATVLATVFGQTGDWDVLVA
GILVAMIEILGNKMYSKKYISKKQVFDISFLSLIGINYIKIGLIFGLFVDAFKLGS
>Q9MUL6
MYFHILILNHTLMKKKSYSTYNLTIWQHIWTIILLFISISCIKQYNFSFQSFNRGEYSNF
TTFDSFNKKQVCNLISFDNALLKILLKNKICINTIEKQPIYKQIQICPDFCRLIFTSHSY
NPIGIINNVSDLSIIGFVDLYGNKWEEGSAFIAHKYFLENQKLPNIIGINNMNSYKLAQM
CFVLKNNKSTHSFVIDLRKEKNIILINNLGKVNIGNDFASLIKKLIFLKSFTSICKEGDD
ILSKIDINNLSRPIVQYSSYL
>Q06801
MAIHTCFSLIPSSFSSPKLPYPKNTTFQSPIPKLSRPTFMFDRKGSFQNGTAAVPAVGED
FPIDYADWLPKRDPNDRRRAGILLHPTSFPGPYGIGDLGPQAFKFLDWLHLAGCSLWQVL
PLVPPGKRGNEDGSPYSGQDANCGNTLLISLEELVDDGLLKMEELPEPLPTDRVNYSTIS
EIKDPLITKAAKRLLSSEGELKDQLENFRRDPNISSWLEDAAYFAAIDNSVNTISWYDWP
EPLKNRHLAALEEVYQSEKDFIDIFIAQQFLFQRQWKKVRDYARSKGISIMGDMPIYVGY
HSADVWANKKQFLLNRKGFPLIVSGVPPDAFSETGQLWGSPLYDWKAMEKDGFSWWVRRI
QRATDLFDEFRIDHFRGFAGFWAVPSEEKIAILGRWKVGPGKPLFDAILQAVGKINIIAE
DLGVITEDVVQLRKSIEAPGMAVLQFAFGSDAENPHLPHNHEQNQVVYTGTHDNDTIRGW
WDTLPQEEKSNVLKYLSNIEEEEISRGLIEGAVSSVARIAIIPMQDVLGLGSDSRMNIPA
TQFGNWSWRIPSSTSFDNLDAEAKKLRDILATYGRL
>P52418
MAAASNLSTLSSSSLSKPSSSFPFRNTPTNNNASFLHNKSLPNQNSLSHKLSSPLPLACN
PKNQNTCVFFDDEDQKPREECGVVGIYGDPEASRLCSLALHALQHRGQEGAGIVAVHDNH
LQSVTGVGLVSDVFEQSKLSRLPGTSAIGHVRYSTAGQSMLKNVQPFLADYRFAAVAVAH
NGNFVNYRSLRARLEHNNGSIFNTTSDTEVVLHLIATSKHRPFLLRIVDACEHLQGAYSL
VFVTEDKLVAVRDPFGFRPLVMGRRTNGAVVLASETCALDLIEATYEREVYPGEVIVVDH
TGIQSLCLVSHPEPKQCIFEHIYFALPNSVVFGRSVYESRKKFGEILASESPVECDVVIA
VPDSGVVAALGYAAKAGVPFQQGLIRSHYVGRTFIEPSQKIRDFGVKLKLSPVHAVLEGK
RVVVVDDSIVRGTTSSKIVRLLKEAGAKEVHMRIACPPIVASCYYGVDTPSSEELISNRM
SVEEIRKFIGSDSLAFLPLDKLKTLLGDDALNYCYACFSGKYPVEPEELQMKRLGVAHFN
WDDDFNGNFESIDVGGWVTNQDGFKIGSV
>Q9MUL7
MRNKIPYDSKKILYDSELLLNSASNRYILTMKVANRANLRRYEEFETMNHSSIKPIARTI
IEMVDDKNFLVVKKK
>A2YMU2
MPPLHAVSPAAAAAPPRALSSAARVPQRPGCVPERPNILSSSTNFMSLRAGPMRFYSRPL
ILQNSDKRAVLRHATIEEIEAEKSVIEDQARERMEKAIETVQNNFNTVRTGRANPAMLDR
IEVEYYGTPVNLKSIAQINTPDATSLLIQPYDKSSLKLIEKTIVAANLGVTPSNDGEVIR
VTVPPLTSDRRKELAKTVAKLAEEGKVAIRNIRRDAIKAYDKLEKEKKLSEDNVKDLSAD
LQKVTDEYMKKIEAIQKQKEQELMKI

>Q20EV4
MAYSVSRLVCSDGSIFSSNTNFETPFQPRVFHPSTSSEINERPDLNLLEGTPRTIVVTSG
KGGVGKTTATANLGMSIARLGYRVVLVDADIGLRNLDLLLGLENRVLYTAMDILDGQCRL
DQALIRDKRWKNLSLLAISKNRQRYNVTRKRMNMLIESLQKQGYDYILIDCPAGIDVGFI
NAVSPAKEAIIVTTPEITSIRDADRVAGLLESNGIYNVKLLVNRVRSEMIQQNDMMSVRD
VQEMLGIPLLGAIPEDNHVIISTNRGEPLVLKKKLTLSGIAFENAARRLIGKQDYFIDLQ
TPYRNVFQRFQQFLGF
>Q42736
MMSSLFVEGMLLKSADESCLPAKGKQRRTGDLRRLNHHRQPAFVRWICRRKLSGVSRIEF
HSGGLTPPRAVLNPVSPPVTTTKKRVFTFGKGRSEGNKDMKSLLGGKGANLAEMASIGLS
VPPGLTISTEACEEYQQNGKKLPPGLWDEILEGLRYVQKEMSASLGDPSKPLLLSVRSGA
AISMPGMMDTVLNLGLNDEVVAGLAGKSGARFAYDSYRRFLDMFGNVVMGIPHSLFDEKL
EEMKAEKGVHLDTDLTAADLKDLVEQYKNVYVEAKGEKFPTDPKKQLELAVNAVFDSWDS
PRANKYRSINQITGLKGTAVNIQCMVFGNMGNTSGTGVLFTRNPSTGEKKLYGEFLVNAQ
GEDVVAGIRTPEDLATMETCMPEAYRELVENCKILERHYKDMMDIEFTVQENRLWMLQCR
TGKRTGKGAVRIAVDMVNEGLIDTRTAIKRVETQHLDQLLHPQFENPSAYKSHVVATGLP
ASPGAAVGQVVFSAEDAETWHAQGKSAILVRTETSPEDVGGMHAAAGILTARGGMTSHAA
VVARGWGKCCVSGCADIRVNDDMKVLTIGDRVIKEGDWLSLNGSTGEVILGKQLLAPPAM
SNDLETFMSWADQVRRLKVMANADTPNDALTARNNGAQGIGLCRTEHMFFASDERIKAVR
KMIMAVTPEQRKAALDLLLPYQRSDFEGIFRAMDGLPVTIRLLDPPLHEFLPEGDLEHIV
NELAVDTGMSEDEIYSKIEKLSEVNPMLGFRGCRLGISYPELTEMQVRAIFQAAVSMNNQ
GVTVIPEIMVPLVGTPQELRHQIGVIRGVAANVFAEMGLTMDYKVGTMIEIPRAALIAEE
IAKEAEFFSFGTNDLTQMTFGYSRDDVGKFLQIYLSQGILQHDPFEVLDQKGVGQLIKMA
TEKGRAANPNLKVGICGEHGGEPSSVAFFDGVGLDYVSCSPFRVPIARLAAAQVVV
>P35006
MGRGLSTKKFYFKIGVQLKSNWNPLIDKLISILKLNSFYSSFVLRLIKLCLTKYLLLYAQ
QH
>P52420
MSSLCASNCYPSSSSINLFSNNNNPTKPFLLSLRFASSNSLPFVAPLKFSTTNHVLSNSR
FSSNRIQRRLFLLRCVSEESQPSLSIGNGGSEERVNVLVIGGGGREHALCHALKRSPSCD
SVLCAPGNAGISSSGDATCVPDLDISDSLAVISFCQKWNVGLVVVGPEVPLVAGLANDLV
KAGILTFGPSSQAAALEGSKNFMKNLCHKYNIPTAKYKTFSDASAAKEYIQEQGAPIVIK
ADGLAAGKGVTVAMELEEAFEAVDSMLVKGVFGSAGCQVVVEEFLEGEEASFFALVDGEN
AIPLESAQDHKRVGDGDTGPNTGGMGAYSPAPVLTKELQDFVMESIIHPTVKGMAEEGCK
FVGVLFAGLMIEKKSGLPKLIEFNVRFGDPECQVLMMRLESDLAKVLLAACKGELSGVSL
DWSKDSAMVVVMASNGYPGSYEKGSIIKNLEEAERVAPGVKVFHAGTGLDSEGNVVATGG
RVLGVTAKGKDLEEARERAYSAVQQINWPGGFFRHDIGWRALRQKQVATKEE
>P52423
MEAQQIISRFCPKSSLAPSIPMVKQPFSLNFPPLHSLSSYPFLQSQNLGFPTGALHAISF
VHKEVCSSSWRIWCSKSSSSTAEPEEDHEVRAQVTVRRKKLAVFVSGGGSNFRSIHEASK
KGSLHGDVTVLVTNKSECGGAQYARNNGIPVILFPKAKDEPKGLSPCDLVDTLRKFEVDF
VLLAGYLKLIPVELIRAFERSIFNIHPSLLPAFGGKGYYGMKVHKAVIASGARFSGPTIH
FVDEHYDTGRILAQRVVPVLANDTAEELAARVLNEEHQLYVEVVEALCEERIVWRKDGVP
LIQSRENPNEFL

>O82796
MEALTTSRVVPVQVPCRKLSSLFANFSCLELRRYPCRGLVSIMNHPKLLRPVTASVQPHE
LSTLGHEGNIVPSKEILDLWRSVEAVCFDVDSTVCVDEGIDELAEFCGAGKAVAEWTARA
MGGSVPFEEALAARLSLFKPSLSKVEEYLDKRPPRLSPGIEELVKKLRANNIDVYLISGG
FRQMINPVASILGIPRENIFANNLLFGNSGEFLGFDENEPTSRSGGKAKAVQQIRKGRLY
KTMAMIGDGATDLEARKPGGADLFICYAGVQLREAVAANADWLIFKFESLINSLD
>Q9MTP2
MKFRVKGKSVDFKNREGSSPSIPKKRSFDSLPINPYFVLFVLLSVQNSLSFFYSFYSFPK
GCKRLFFSINPRLVLNMLHVQKNFVLARDQHLNDSQSIISILGLPLKKSFFFFFIFLSKK
ALGLGKTFHTFFVFLLIDIERSHLVKCQGSG
>P42804
MAVSSAFVGCPKLETLLNHHNLSPSSSSSSSVSQTPLGLNGVRVLPKNNRTRRGLIQKAR
CELSASSDSASNAASISALEQLKNSAADRYTKERSSIVVIGLSIHTAPVEMREKLAIPEA
EWPRAIAELCGLNHIEEAAVLSTCNRMEIYVLALSQHRGVKEVTEWMSKTSGIPVSEICQ
HRFLLYNKDATQHIFEVSAGLDSLVLGEGQILAQVKQVVKVGQGVNGFGRNISGLFKHAI
TVGKRVRTETNIASGAVSVSSAAVELALMKLPQSSNVSARMCVIGAGKMGKLVIKHLMAK
GCTKVVVVNRSEERVSAIREEMPGIEIIYRPLDEMLACASEADVVFTSTASETPLFLKEH
VENLPQASPEVGGLRHFVDISVPRNVGSCVGEVETARVYNVDDLKEVVAANKEDRMRKAM
EAQTIITEESTQFEAWRDSLETVPTIKKLRAYAERIRVAELEKCMSKMGDDINKKTTRAV
DDLSRGIVNRFLHGPMQHLRCDGSDSRTLSETLENMHALNRMYGLEKDILEEKLKAMAEQ
QQK
>Q9MUM6
MSLQVPVAKEDNRESNSHLATLSGNLYTRMNLPHEPRARAGIVFFMEKQSNLALHSEFLL
HVIIN
>P42732
MAQMVAMPVAHSLSLICNWAKSNPLSRNTLALPASNTPNKQSLSIRCARVGGVEIPANKR
IEYSLQYIHGIGRTRARQILVDLQMENKITKDMAEEELIIILRDEVSKYMIEGDLRRFNAL
AIKRLKEIQCYRGVRHIQGLPCRGQRTKNNCRTLKGKKIAIAGKKKVSK
>Q9MUM7
MILIKKFIVAIELFIFNRNNCRYILLCTDNKVKNIQTSLSICILCAISYALIKKIQQTFQ
LSSKFAASCS
>Q2WGC1
MGGVENPVSTDKAIRLPERKQYSSNAEPNPSKTEVKRRIERFPRVRIVAINSHRLPAGAA
RGSAGSVTGRRVRRKRMITTLRLNHPIPLFPGKQNGFVS
>O20159
MATQPSENTPKVITEKKIFLPTEKKINLFNIIDRFNNHLKKLKEKLQEAHFQKQANKLTS
KEQELKDNDDGKSKSKNENDSTSPEKKDVLTGTGVALPGLIQLAGGFTVSITGNAGTAQL
GGRSSRNGNQRQSISRRIIIRSENQRTQNLTESVESVHTSQKKNEVRQALMISAIKKATH
GTGVNQKKPEEAVRINAEASRKIKPEVNYNLKIFLAEKTLTSKEKKEAAKLIKKINHEIS
EWLTKFFTTRTGRQRELQKNLIWSLIKKFLPKVIGLERSGNHVHAYFMLSKLYGLLRMAC
LKVISQKKTILSRRNKMPKERIRVKDEIIRLEKEIKHLTNPAEIGLKEKQLLGQKEELDS
LNAEDVLFQGFEIKLTPTESQFWDEACLTEKQEISRQLHYDNWEDYKKNLTETKTQKRIY
HNILSKLITLGTKKENKNLLEEHFVFFVTKGNFYIRAFLEIQFAHLFGAARRGENEPEDS
SVQGTNSHLNLVYLETFSHSISCLYEMGGYIANVELTTGAILKLLSQSLDSLLLSSSNSS

VDERNSYVKTKASLQSLKQLLLEYCNFHNDPESFDVNTTQELKQRLKDLSSVSLSKKKEL
ELYKELLKAFKLCFQDIMNALSEVLKKLEKRIPEKTFTELKELFLLHQSHLIKLKQVCGA
LSSLFGFFLIELKSLWQLPLDEYIGVFIGSEPRKK

>P0C306

MVYGYGKSNMPHPNRKRKGTDTQYDYWEELLVMVSGLYVLFCVFLVLFIFFDSFKQESNK
LELSGKEEKKKLNGENRLSRDIQNLLYIK

>P0C308

MGAFPSPPPWGWSTGFITTPLTTGRLPSQHLDPALPKLFWFTPTLPTCPTVAKQFWDTKR
TSPDGNLKVADLPSFAISFATAPAALANCPPLPRVISMLCMAVPKGISVEVDSSFLSKNP
FPNCTSFFQSIRLSRCI

>Q9MTP8

MDLLKKERKANEIKIMNYKESSGSKMNCKESSGSNSIDNMDGPVYNDRPMDGFFKLQIWI
HPLDILKMGWLNVKIHSLKFNK

>Q9TL05

MSGEVAQDQPPIRSPRSTRRSQEIVNSAITVQSSAKLDTDKSGAQADKVSSKKSRTTSPE
LLEDQVVSNESKTSKKKETKASQLFSKRKEIFSTRDLRFLKLRIASPTRIRSWGTHKLPN
GKQVGRITKAETINYRTYKPEMDGLFCERAFGPVKDWECHCGRTKGQERNKDGIPIPRVC
THCGVELRDSKIRRHRMGYIELVYPVVHIWYLKSIPSYLGVLLDKPRRELEAITYCTNYA
SSQDSMAFSASLLFPTTGSFRQSKDSMKWEYLNWFHIETYLGLKEATNSALVHYGKRIQD
SPIEVGLPLSEDPRQFSIGAQAIACQLRALNLRSVSRLLSRDLYIIDARETRFGALEEEE
MKRRSKLIRRLQLIHYFMQTKAQPEWMVIKALPVLPPDLRPIVQLEGGRFATTDLNDLYR
RVLNRNNRFMKLHKMVAPETLIRSEKRLLQEAVDGLFDNGKRGKPVLNSSNRPLKSLADA
LKGKQGRFRQNLLGKRVDYSGRSVIVVGPKLRLHQCGLPKEMALELFQPLVIRLLLKRKV
APNIRYAKKLMHHAVARGPENPTIIDAVVWDTLAAVVEGYPILLNRAPTLHRLGIQAFEP
VLINGRAIQLHPLVCTGFNADFDGDQMGVHIPLSAEARAEAKLLMLASHNLLSPATGQPI
VVPSQDMVLGWYYLTTENPWIESTEGLYFSGLSDVEHAYHQGQIHLHSIIWVRWGGESEG
SLGDEFEDNPLEIRIDANGHSWHIYSQYQLRYDDEGDLISQFIRTTTGRVVFNQLVHRHI
EWSLHDQVMEEIETEFPESVLPPDVMRCLVRLYSAHAIGERPAMLGLASPGTPSPGRACA
YPPRDDYADPNQIKEYLNRIGHW

>P42738

MRSSCCSSAIGGFFDHRRELSTSTPISTLLPLPSTKSSFSVRCSLPQPSKPRSGTSSVHA
VMTLAGSLTGKKRVDESESLTLEGIRNSLIRQEDSIIFGLLERAKYCYNADTYDPTAFDM
DGFNGSLVEYMVKGTEKLHAKVGRFKSPDEHPFFPDDLPEPMLPPLQYPKVLHFAADSIN
INKKIWNMYFRDLVPRLVKKGDDGNYGSTAVCDAICLQCLSKRIHYGKFVAEAKFQASPE
AYESAIKAQDKDALMDMLTFPTVEDAIKKRVEMKTRTYGQEVKVGMEEKEEEEEEGNESH
VYKISPILVGDLYGDWIMPLTKEVQVEYLLRRLD

>Q94KU5

MATLFTVTTTSRPFFPANPSKTFSPSISLKPNALSFSLTHHRPPRPLRFSKIRSSLPSESD
SEPEGGYSITDEWGEQPAEPESPPDNAPSAVSDEWGEKSESVPEESVTRFAESDPPTNED
EWEEREADDGVDKTWELKRCLADTVYGTELGFRAGSEVRAEVLEIVNQLEALNPTQAPVE
NPELLDGNWVLLYTAFSELLPLLAAGSTPLLKVKSISQSIDTKSLSIDNSTTLSSPFADF
SFSATASFEVRTPSRIEVSFKEGTLKPPEIKSSVDLPESVGVFGQEINLSFLKQSLNPLQ
DVAANISRAISGQPPLKLPFPGNRGSSWLLTTYLDKDLRISRGDGGLFVLAREGSSLLEL

>Q9M385

MAIPMSMAMATPTDSVSRVWSMSSLKSALPSTASLRLPSSSSRRPVTLRLPISSPSLPSF
SGLSPVNPLLSIGLPDWQSFENGFKIVDGGGRIYAMRHGRRVPKLNRPPDQRKALLRGLT
TQLLKHGRIKTTRARASAMRKFVDKMITLAKDGSLHKRRQALGYIYEKQIVHALFAEVPD
RYGERNGGYTRIIRTLPRRGDNAPMAYIELV

>Q9SUI6

MASFATIAAVQPSAAVKGLGGSSLAGAKLFIKPSRQSFKTKSTRAGAVVAKYGDKSVYFD
LEDLGNTTGQWDVYGSDAPSPYNPLQSKFFETFAAPFTKRGLLLKFLILGGGSLLTYVSA
NSTGDVLPIKRGPQEPPKLGPRGKL

>P17597

MAAATTTTTTSSSISFSTKPSPSSSKSPLPISRFSLPFSLNPNKSSSSSRRRGIKSSSPS
SISAVLNTTTNVTTTPSPTKPTKPETFISRFAPDQPRKGADILVEALERQGVETVFAYPG
GASMEIHQALTRSSSIRNVLPRHEQGGVFAAEGYARSSGKPGICIATSGPGATNLVSGLA
DALLDSVPLVAITGQVPRRMIGTDAFQETPIVEVTRSITKHNYLVMDVEDIPRIIEEAFF
LATSGRPGPVLVDVPKDIQQQLAIPNWEQAMRLPGYMSRMPKPPEDSHLEQIVRLISESK
KPVLYVGGGCLNSSDELGRFVELTGIPVASTLMGLGSYPCDDELSLHMLGMHGTVYANYA
VEHSDLLLAFGVRFDDRVTGKLEAFASRAKIVHIDIDSAEIGKNKTPHVSVCGDVKLALQ
GMNKVLENRAEELKLDFGVWRNELNVQKQKFPLSFKTFGEAIPPQYAIKVLDELTDGKAI
ISTGVGQHQMWAAQFYNYKKPRQWLSSGGLGAMGFGLPAAIGASVANPDAIVVDIDGDGS
FIMNVQELATIRVENLPVKVLLLNNQHLGMVMQWEDRFYKANRAHTFLGDPAQEDEIFPN
MLLFAAACGIPAARVTKKADLREAIQTMLDTPGPYLLDVICPHQEHVLPMIPSGGTFNDV
ITEGDGRIKY

>P29463

MESRVLTGGATAIRGGLPLLRKPAAVMKFTTAAHAISRDFPAGAVTAKPVGPLIAGPNLI
WGRQLRPAILLETSPKRESIKPCSAAASSSAGSSDSSGDAKVGFFNKATLTTGFFFFMWY
FLNVIFNILNKKIYNYFPYPYFVSVIHLAVGVVYCLVSWGVGLPKRAPIDSTQLKLLTPV
AFCHALGHVTSNVSFAAVRVSFTHTVKALEPFFNAAASQFILGQQIPLALWLSLAPVVLG
VSMASLTELSFNWLGFTSAMISNISFTYRSIYSKKAMTDMDSTNVYAYISIIALIFCLPP
AIFIEGPQLLQHGFNDAIAKVGLTKFVTDLFWVGMFYHLYNQVATNTLERVAPLTHAVGN
VLKRVFVIGFSIVIFGNKISTQTGIGTCIAIAGVAIYSFIKAKMEEEKRQKKAA

>Q00865

MGFVDMDYDIISYSDQMPLAQHYSYFIIYSPSKSRFHVIPYKDDEYLNRFYWLQISLGFS
IDASNISSQYSANPTINLSLILD

>Q9ZST0

MAATAVTLPSSPAPFPVTTTASSSRNVRLLLRSPPPRRALRVAASAAADAPPKPAPPPTS
PSGIVLVDPTEAQKVHRLKAVYDQKVVPLITEEFGYTNVHQVPKVEKIVVNCGLGAEAGN
SKGLESAMKDLAMITGQWPVKTKAKKSVASFKIREGNTIGIAVTLRGRVMFNFLDRLINL
GLPRTMDFLGVNPNSFDGHGNFTIGLRDQGVFPEIPYEVGGKKNGMDVCIVTTAKTDNEA
LRLLTLLGMPFAEHIKSSVVIRKKRLKRHHFMSKGRGRR

>P27793

MASSLSTKPFLSGSRRRSTTDGSGWSYFQTSDLRQLSNQSVQISVRRQTAPLKLVVQASG
SSFGKVFQVTTYGESHGGGVGCVIDGCPPRFPISEADIQSDLDRRRPGQSRITTPRKETD
TCKIYSGVADGFTTGSPIHISVPNTDQRGNDYSEMAKAYRPSHADATYDFKYGVRSVQGG
GRSSARETIGRVAAGALAKKILKAYAGTEVLAYVSQAHKVVLPEGLVDHETLSLEQIESN

IVRCPDSEYAEKMIAAIDAVRVKGDSVGGVVTCIMRNVPRGLGSPVFDKLEAELAKACMS
LPATKGFEFGSGFSGTFLTGSEHNDEFYTDENGRIRTRTNRSGGIQGGISNGEIINMRIA
FKPTSTIGKKQNTVTREREEIELIARGRHDPCVVPRAVPMVEAMVALVLLDQLMLQHAQG
NLFSINPALQEPLSETVSSAAASLQGV
>Q9SR43
MALSMEFGFSIGSCFKAPNPPVLISASPNKINFTLRRRKKRFLLRVSAVSYKEFAESALE
ETRKRIVLEPSHLQEKYSSMTGLDGKTELQMLAFKSSKIRLLRSMAIENETMQVFDFAGF
MEPEYDTPIFCANFFTSTNVNIVVLDLNPLHQLTDQTDYQDKYYNKIMSIYHKYAETFPW
GGKLTGESIKFFSPLVMWTRFSSSKEKHKALFSAFLEYYQAWLEMTIQVREEMEPSHVRA
NCEAQHKYLTWRAQKDPGHGLLKRLVGEAKAKELLRDFLFNGVDELGTKTFIDYFPEYQT
EDGTVSDKRSIIGKSYETRPWDLTGQFIG
>P29102
MAAALQTNIRPVKFPATLRALTKQSSPAPFRVRCAAASPGKKRYNITLLPGDGIGPEVIS
IAKNVLQQAGSLEGLEFSFQEMPVGGAALDLVGVPLPEETVSAAKESDAVLLGAIGGYKW
DKNEKHLKPETGLLQLRAGLKVFANLRPATVLPQLVDASTLKREVAEGVDLMVVRELTGG
IYFGVPRGIKTNENGEEVGYNTEVYAAHEIDRIARVAFETARKRRGKLCSVDKANVLDAS
ILWRRRVTALAAEYPDVELSHMYVDNAAMQLVRDPKQFDTIVTNNIFGDILSDEASMITG
SIGMLPSASLSDSGPGLFEPIHGSAPDIAGQDKANPLATILSAAMLLKYGLGEEKAAKRI
EDAVLGALNKGFRTGDIYSAGTKLVGCKEMGEEVLKSVDSHVQASV
>O80333
MPKRSNGSLVVRCAVSVVRFSKENVSCDLASENFTFSRDSFPVVSTVLRVSEAVYRMAAV
ASLGSALSVSSAALSQNVSVSNNATKESAFLGLRMGEVAKFGGALLSVSTVAANLKSKPG
VLSVNAVTAPAETMNKPSSKKTATKSTCIITGASSGLGLATAKALADTGEWHVIMACRDF
LKAERAARSVGIPKDSYTVIHCDLASFDSVRAFVDNFRRTERQLDVLVCNAAVYFPTDKE
PKFSAEGFELSVGTNHMGHFLLARLLMEDLQKAKDSLKRMIIVGSITGNSNTVAGNVPPK
ANLGHLRGLAGGLNGVNSSSMIDGGEFDGAKAYKDSKVCNMFTMQEFHRRYHAETGITFS
SLYPGCIAETGLFRNHVTLFRTLFPPFQKYITKGYVSEEEAGKRMAQVVSDPKLSKSGVY
WSWNKDSGSFENELSEEASNPEKAKRLWELSERLSGLV
>O20162
MVTEFRDAWEARYGLVPGACCLKNSVTFSSLESGQGLERNPNQEALADRKNKKCQFKNEY
VACHATVFPHFVNPENPKAVEFLKNHPLNIPVNMDMTFGPLKDTPAFCYGGHKLGLCNSL
YDSIIYDDTYQGKGEHNLKKFFSEAIATCFLYQNAIKPINYKPKNMKHSVIGVSLYY
>P0C310
MTKDETLLVFTLVVSSVSVFLFGILLFMVLISATRDFRERTKSKLVKIMIWAGIVVITFA
IAVRIYPIFIFLLKERIKPLVEALYDKLPWIWEVSLSRYWDRLIDFLDRYLWACAQRIQT
GIRKQKGEFVVTFSCRVKKRLYARAIEVGIHLSLLSNLFWILKTTLAVGYRLLWVLYYII
SFEGFLGSFRLYLVYFGFYCLLFSGKWLRTSEDRGERQAQISGILLRGMLIECAFSVLCL
EEDSNLHAL
>Q9MUN5
MICKNSIVTSSSKLFYKMFFIAISIEILEYNYIYPLCIEIAQHSGNIKIALCLKEILQKK
KYNLILLKIASIVISNFLSTNTYSFIYTPYDVEILFSNLLLEKYEKDKEMSYMIDVFTKL
SHLKQNIIYPYLFNQLKHDCNKKHIFLLLKKIDYTDISTFTHSIFIK
>Q9TL10
MLVVGNRIVDVSRHTSIGTIVIATVDKLAPGLGIAFVSWTHGQGKGGLPSTKKHYGILPL

RSWRGRGPLDFATTHELTGENLILQHGDFVLVQIVQDGNHAKVHLVSGHIALTTSRLVVW
PGLSSKDWIFSHQIGQKINNYLHVRKLVSYMRRDQILCTGPQRWMKEQYALEHQWENFVL
EFIEQPTGISYLTSMTEKFVSPVCAEWFQHPLSVWIIGCNLQIRESMTKWMITHVPHKSR
HIEITTLDAWKNWYNLHRAAIVQPQIPLRSGGTMIIEFTEIGWSFDINSGIGLEIGSKTC
ANEEAIYAIAQQILLRSMHGFILIDFIGDIDLEKLRVNLIQFTSLLEQDSYHIRIISISA
DGLVCVIRHRRSKLI
>Q20EX1
MNIYLRQNSFLVPDFIAVQRNSFSQFLESGLIQEISKRNPITNSTKELELCFYPQYYKLS
PPELNAKQSILKNKTYSCRLYIPVKLTNKRTKTLKLQWVVLGNLPLMTKRGHFIINGSPR
VIINQMVRSPGVYFKEVSHADQKLTYYADLIAYRGAWLRIELDKKADIWARMKKTPKLPM
LILLQALGLNLPTILKSLNYPKFFENLGHDGNFMKQLKKGRVSSRSSSQELSGSFNLSED
VSLRLDDSSKNMGAENVQRAPLQKSTKKATLFGDFSRSSVYCKTQEEALWALYALTHPLK
NPDEVTANLGKSFLYRKFMNSRVYDLSPLGRLRLNQKLNLSVNENITTITAQDLLFITNT
LINLNYGVGKVDDIDNLKNRRIRTAGELVQNQFGIGLLRLEKIIREKLKQQQTKLSIGSL
INTKPINGALREFFGSSPLSQFMDQTNPLAEITHKRRLSSLGPGGVNRETAGMAIRGIHP
THYGRICPIETPEGQNAGLVNSLTIYSRVNPYGFIETPFYKVVKGQVQNSTRPIFFSAYQ
EEKKLLAPGDIYANKLKYLPKSELPIRKLAEFTRTTREKIDYISVSPLQMISIATSLIPF
LEHDDANRALMGSNMQRQAVPLMTAEAPVVGTGLECRVASDSGHVLQAKKSGFVTYSSAE
KITILSPVDSTPAVPKLSKGNNSPQHFHESVGGSCKESQSLQAAAEGYLSGVASSSSSKF
TGSSFSTISALNDGVLRRALTQKVLTTGTNCSSMLKNQRFFVEPKAQLYTGAQTPVGRVE
TILQPNHAEGYLSEVETIQQTQNKLPLQVRFGVEAPKSSFDSEATKISSTLNSIPQHPVL
GEDSPSKQVGWALKDPQIQTMLKPKWHHSAQTMSRENWYQNLKGQNVKTFTQVESGRLVT
KPTNTKMPMSSLAGTANSKKTGEALYKLASSESATKSMDFSTKTQLKPITYELQKLFRSN
QDTSIMHRPVVREGEWVNRGDLLADNSTTVGGELSLGKNLLLAYMPWEGYNFEDAILISE
RLVSDDAYTSLHIERYEVEIRETKFGLEQITSQIPDEGKLSHLDHFGIAKPGTWVEEGDI
LIGKVAPMPQKNLSRYEKLLYDVVGKKISTTRDTSLRVPRGVSGRVIHVEILNAENLPPE
FVFEGPSRVNLYIAEKRKIHVGDKMAGRHGNKGIISNILPRQDMPYLPDGTPLDMVLNPL
GVPSRMNVGQIYECLLGLAGRYLGQKFKVRPFDEIYGPQTSRSLVYSKLYEARLRTGQRW
LFNPASPGKTRLFDGRTGECFSQPVTVGQAYMLKLIHLVDEKIHARSIGPYSLVTQQPLR
GRSKHGGQRLGEMEVWALEGFGAAYTLQELLTVKSDDIKGREQVMESIQKNKTISLGTPE
SFKVLIRELQSLCLDVGVYAVDGSGKIKQVDTMKLP
>Q96291
MASVASSTTLISSPSSRVFPAKSSLSSPSVSFLRTLSSPSASASLRSGFARRSSLSSTSR
RSFAVKAQADDLPLVGNKAPDFEAEAVFDQEFIKVKLSDYIGKKYVILFFYPLDFTFVCP
TEITAFSDRHSEFEKLNTEVLGVSVDSVFSHLAWVQTDRKSGGLGDLNYPLISDVTKSIS
KSFGVLIHDQGIALRGLFIIDKEGVIQHSTINNLGIGRSVDETMRTLQALQYIQENPDEV
CPAGWKPGEKSMKPDPKLSKEYFSAI
>Q93ZN9
MSSTHQLVSSMISSSSSTFLAPSNFNLRTRNACLPMAKRVNTCKCVATPQEKIEYKTKVS
RNSNMSKLQAGYLFPEIARRRSAHLLKYPDAQVISLGIGDTTEPIPEVITSAMAKKAHEL
STIEGYSGYGAEQGAKPLRAAIAKTFYGGLGIGDDDVFVSDGAKCDISRLQVMFGSNVTI
AVQDPSYPAYVDSSVIMGQTGQFNTDVQKYGNIEYMRCTPENGFFPDLSTVGRTDIIFFC
SPNNPTGAAATREQLTQLVEFAKKNGSIIVYDSAYAMYMSDDNPRSIFEIPGAEEVAMET
ASFSKYAGFTGVRLGWTVIPKKLLYSDGFPVAKDFNRIICTCFNGASNISQAGALACLTP

EGLEAMHKVIGFYKENTNIIIDTFTSLGYDVYGGKNAPYVWVHFPNQSSWDVFAEILEKT
HVVTTPGSGFGPGGEGFVRVSAFGHRENILEACRRFKQLYK
>Q85WU2
MIRVLGLLWDPLSSWVEVSGPIILFGLYYGFIATLPFGPSKIYSMRSFFLGETLYGIIAI
SGSITGQLIVFLSMYYSPIYAALWKPHAITLLVIPYTFCRVFRSLEKPSSPESTHPMNSI
KNPKILSLFMGGLILQLLNPILLANPVLTRLVNLFLFRYSDNISFMISSFCGWLGGHILF
INLTKLVSLRLVSFRIERNSPIDHTSLRRYIHQTFSVLLISYFSFYLGRSPLIFHRKKKD
NKKKDRSAAMAKKDRSVAMAKKNCSVAMVKKDRSVAEDEDRSVAMAKKGRSVAEDEDRSE
PLAMVMVQEARSVSFIAKKARSVAEDKDPEDEHRSVAMAKKARSVAEDKDPEDEHRSVAM
AKKDRSVAEDEDRSVAMAKKDRSVAEDEDRSVAEDEDRSVAEDEDRSVAEDEDRSVAEDE
DRSVAEDEDRFVAEDEDRSVAEDEDRSVAEDEDRSVAEDEDPEDEDRSVPREQKKLKGLP
ISWFKQPCPIKFFDPDRIYQPIRYIGNSPFHCLKPVMRTEVSQYFFGAYSSDGKKRISFT
LLPSVLALGEKLGKYRDLLDTSCLSEDPYHRWNHTMKRRRDSLENEFSDRVKALSHGSPA
ENVIERRVKFSNSQGDSFTEMYDPLLNGALRGTIDQFESPKMLNDLIISIISNLSDFIEI
PIKDCKEGFPNDQFGYGYHISEHWQELEHKSFRLPWEPLPTDTFRSLVPSTKSSRRGKVE
PISKRLYPLAERIIPNKAKKIFEEYLSNIDSSFGKLIYPKDLLEMQIQEIYTKDDDSLIH
FLWLEIAFRVAYMDLAPEIASCNERIYTNSLVNIYNRIDGRNVAPTGTVKWELILSLFTT
KQIFLFESLAQHEWTILRNCRGNVSTDDSTQTKDFIDLYGKILLNEPLQFREIKKHLPRW
PSDLMRAERDGDGDDRGPIQISTSRIRTRKVKSKLTLDFGKERIVLKRYHAESDYRRSLI
RGSMRAKRRKIMIWKRVQPNVHSLFFLRRMEIPTYPKDYYDTFDSGRVNQEQIQKEVREK
IHRGKYLDPVLHNTTLAEQVCLAWPEVHYFRGLILVAQSNIRKNIILPSLIIAKNIGRIL
LFKAPEFAQDWEEMKEELHIKCGSDGTEFSKKGFPDKWYKYGMQIKLIFPFRLKPWHSQS
KKRLRLRWSYLTTLGFETDIPFGDPKPKLGIFSEFFQPIFKKVKKGLKKGLKKGLKKGLK
RELILFKKVKRRLMGSTGIRRVSRVRYIDKSELNDRIQNKLLSETETTPMGSANDSSEVN
DQFGYETQTINVKDPDDWTTTMKERIESIAIINSSPITGMSLMDSEIHTGSKGSFNILGS
TLKKRLVQIRRIPGRFRNKSVQLIRKRFYSMKLMKLFLKRMDRYLLLSVIHFIGSNIKFW
IRSAWIRSTGNIATIYGRIFIINNDISKINRGKITNYYSINEKRKDFEIRPDRNMLSMSQ
AYVFHKIWQIGAIDRSYSKYFLKYRAQTSYPLIKKKIKELLDIQRILDHEKPQDLKENDW
KQWLRCFDRYKLSPQIWSRINPQKWRNRVNKQCTCYEERFIPYEKKKDYIFATVIEPSLG
LLRKMNKRYRYDLLSYSYLNSTKELDILNLTKDSDILNLTKDSDILNLTKDSDILNLTKD
SDILNLTKDSDTLKIEKRRSGLDLKFWLFPELSGKENIYDNSKFIPGYSILSEAQERKKI
EEKEREERIKVIEERIGIIRSDVQNKKVEEKQTGTGEVEEKQTGKVKQKQFGLKVEDQKT
DGKKKTNQVLVQHKILKDIGEEDISDLAGMCRILIEIEDPTKFMQIYEENINLNLMFLII
YEDLKGYEKYINARDSNANDINANDINANDINAKDSNANDINAKDSNANDINANDSNAKD
SNANDINAKDINAKDSNANDINAKDSNADVPKKKEDEIPKTVVSSEPYRLSSIVNDNLLI
YKIVSMWLKSEKIKRAGLGDDKNILSSFNLEDILLPKRRREFRILNRFDLENDHVGFFNG
KSIQNDEELMGRDQHLSVDTTQRIKRFLWPSYRLEDLLCMNRYWFNTNDGSRSAMLRIRM
YPLNVN
>P08927
MASTFSATTSSCNLSSSAAISSFPLAAGKRNANKVVLPRKNRNVKVSAMAKELHFNKDGS
AIKKLQNGVNKLADLVGVTLGPKGRNVVLESKYGSPKIVNDGVTVAKEVELEDPVENIGA
KLVRQAAAKTNDLAGDGTTTSVVLAQGLIAEGVKVVAAGANPVLITRGIEKTSKALVAEL
KKMSKEVEDSELADVAAVSAGNNHEVGNMIAEALSKVGRKGVVTLEEGKSAENSLYVVEG
MQFDRGYISPYFVTDSEKMTVEFENCKLLLVDKKITNARDLINILEDAIRSGFPIVIIAE

DIEQEALATLVVNKLRGSLKIAALKAPGFGERKSQYLDDIAILTGGTVIREEVGLTLDKA
DKEVLGNAAKVVLTKDTTTIVGDGSTQEAVNKRVSQIKNQIEAAEQEYEKEKLSERIAKL
SGGVAVIQVGAQTETELKEKKLRVEDALNATKAAVEEGIVVGGGCTLLRLASKVDAIKDT
LANDEEKVGADIVKRALSYPLKLIAKNAGVNGSVVSEKVLSSDNPKYGYNAATGKYEDLM
AAGIIDPTKVVRCCLEHASSVAKTFLMSDCVVVEIKEPESAPVGNPMDNSGYGNI
>P38365
MTTGVAVMSSATAASTATATAAATARIPLFLSRNNSSATVCSTLRCRTITRTRTRARLAI
CCEVALKSDSSTGFDSSSSSPPEEDEELKKNLEKVGCKVKVKSPLKVYHVPKLPEVELTP
DMVGVIKQYVGFWKGKYISPNYPFKVEYRIDVPDRGSVKLVVHLKEEEFEIIAE
>Q20EX8
MRNTVLNTLNAFPTQLGSNKSTLSSKIFHSYLGVLRPPTPLNTSMEVLGEGAHQNREAKR
SSLRFEFCSALKECQKGKVPFGSSLRLEAAENLTFECETGNYHTFCPISCVRWLYQQIAD
SFFLVIGTKTCGYFLQNAMGVMIFAEPRYAMAELEEGDIAAQLNDYKELKRLCLQIKHDR
NPSVIVWIGTCTTEIIKMDLENLAKLIEAELKVPIVVARANGLDYAFTQGEDTVLASLVN
RCPSSHESSLDSMKLPSGGREKQINDVNTSKPEGYLSEVISLTSNGDDINKKSCTKPVPK
KSLVLFGSVPNSVQTQLTLELAKQGINVDGWLPSRYSELPVLNKDVYVCGINPFLSRTAT
SLMRRRKCHLISAPFPIGPDGTRRWIEKICTVLNTDKSSTSLEEVQKNLQQREEKVWKSL
QSYLDLVKKKSVFFMGDNLLEISLARFFIRCGMIVYEIGIPYMDRRYQAAELALLEQTCL
EMNVPLPRIVEKPDNYNQIQRIRELQPDIVVTGLAHSNPLEARGVTTKWSTEFTFAQIHG
FANSRDVLELITRPVRRNQNLDALGFTSLVKN
>Q20EX9
MKLAVYGKGGIGKSTVSCNLSIALAKRGKKVLQIGCDPKHDSTFTLTGFLIPTIIDTLQS
KDYHYEDIWPEDVIYAGYGGVDCVEAGGPPAGAGCGGYVVGETVKLLKELNAFYEYDVIL
FDVLGDVVCGGFAAPLNYADYCLIVTDNGFDALFAANRIVASVREKARTHPLRLAGLIGN
RTATRELIDKYVEVCRMPVLEVLPLIEEIRISRVKGKTIFELEELEPNLKSIGEYYLNIA
DQLLAQPEGIVPQELADRDLFTLLSSFYLSDQQTEKAYSSIQHVHGSVGEGCSAQNSEQN
QTANDYSFEFI
>Q40147
MAAVNGVGLSWPSKLTKNQTPKWGFSPSHRRCNPSSSSSATIRMTASVDEKKKTFTLEKS
EEAFSKAKELMPGGVNSPVRAFKSVGGQPIIIDSVKGSRMRDIDGNEYIDYVGSWGPAII
GHADDEVLAALAETMKKGTSFGAPCLLENTLAEMVISAVPSIEMVRFVNSGTEACMGVLR
LARAFTCRPKIIKFEGCYHGHADPFLVKAGSGVATLGLPDSPGVPKAATIDTLTAPYNDI
SAVESLFEEHKGEIAAVILEPVVGNAGFIPPKLEFLAAIRKITKENDALLIFDEVMTGFR
LAYGGAQEYFGITPDLTTLGKIIGGGLPVGAYGGRRDIMEMVAPAGPMYQAGTLSGNPLA
MTAGIHTLKRLQGQGTYEHLDKITAELTQGILDAGKKTGHAMCGGSIRGMFGFFFADGPI
YNFSDAKKSDTEKFGRFYRGMLEEGVYFAPSQFEAGFTSLAHTPEDIQRTVAAAEKVLKQ
I
>Q9FWS4
MAVSLPNSFLQISPCVPSLQLRKPVMAAVKGGKQSVRRSSNTVVQITCRKKELHPEFHED
AKVYCNGELVMTTGGTKKEYVVDVWSGNHPFYLGNRSALMVDADQVEKFRKRFAGLSEIM
EIPVLKGEIIMPTKKSKGPKGKKK
>A1E9Z1
MPNILSLTCICFNSVLYPTTSFFFAKLPEAYAIFNPIVDVMPVIPLFFFLLAFVWQAAVS
FR

>Q8HUH1
MNVYVTLKTKLVAELRQIKQTLQTERNIIRVLRRKLKQIAAQKRFIKFLPKLRYLPTPVT
KIEQTARFLVKKFVDPKMKYPMDSIYDKKLSRQSKKVAASRKKKWQRLEKYLGGISNMTK
IKEKQIANNVAIIIGQQEEMNAVRECQKLGIKMFHIVDTNCNPGLADHFIPANDDARNSI
KFILGKFLTRIRLAHKIKVKFKKTSLKK
>Q8S9K3
MNNSTRLISLFSPHPPPLFLLRGLYISRIANLRRFHRRAFPPSSVASTNLCSFRPLVSLP
PLIPTFPIGRFYNHQVRVSAADFVPSYHNQQLPEWTELLQSLSKAGYFSDSGSISGLESE
FFPGFPDELLRPALACLALARERPELLEMLSRRDVEVLVENGKPFLFKTGPDSLKRMSLY
LRSGLQGIGKLMDMEKASTVDLMRLILSYVVDVASSEESKQHNKEIMESSVRSLLSQIAK
MSLRPPESNVHDTMQNQYSDRDGQGVRSFQNNVEMKRGDWICSRCSGMNFARNVKCFQCD
EARPKRQLTGSEWECPQCDFYNYGRNVACLRCDCKRPRDSSLNSANSDYSSDPELERRLV
ENEKKAQRWLSKVAQGGSDANSVDTDEDFPEIMPLRKGVNRYVVSTRKPPLERRLANTEN
RVATDGNSKRSDDNALGSKTTRSLNEILGSSSSLTSRSDDKNVSSRRFESSQGINTDFVP
FVPLPSDMFAKKPKEETQIGLIDNIQVDGFSGGNQNVYQEDKSDANHSGKETDRLEKEDH
KSEEPARWFKRVTELHNVSDLESAIPQEISPEKMPMRKGENRFVVSRKKDRSLTSPAYKR
PEDSDFVPFVPFPPDYFAKEKQPKESIDTLPAPATENVSQVVQQEPREPSINKSDTVAVK
IRNGKSLEGSLVKESDLLDMSEEAKAERWFKRVAEIKNISELSEIPDEDFPSIMPMRKGV
NRFVVSKRKTPLERRLTSQRHQRNPHITNSDPTGKGDK
>O04130
MAFSSSCSSVKAVNSRWTSPSPSPSSRFAVLPAFLHRRYATSVKLTAISAALKTVEQTTL
TEDNRFSTVGSDSDEYNPTLPKPRILVTEKLGEAGVNLLREFGDVDCSYDLSPEDLKKKV
AESDALIVRSGTKVTREVFEAAKGRLKVVGRAGVGIDNVDLQAATEHGCLVVNAPTANTV
AAAEHGIALLASMARNVAQADASIKAGKWERSKYVGVSLVGKTLAVMGFGKVGTEVARRA
KGLGMTVISHDPYAPADRARALGVDLVSFDQAISTADFVSLHMPLTPATKKVFNDETFSK
MKKGVRLINVARGGVIDEDALVRALDAGIVAQAALDVFCEEPPSKDSRLIQHENVTVTPH
LGASTKEAQEGVAIEIAEAVAGALKGELSATAVNAPMVAPEVLSELTPYIVLAEKLGRLA
VQLASGGKGVQSIRVVYRSARDRDDLDTRLLRAMITKGIIEPISDSYVNLVNADFIAKQK
GLRISEERMVVDSSPEYPVDSIQVQILNVESNFAGAVSDAGDISIEGKVKYGVPHLTCVG
SFGVDVSLEGNLILCRQVDQPGMIGQVGNILGEQNVNVNFMSVGRTVLRKQAIMAIGVDE
EPDNKTLERIGGVSAIEEFVFLKL
>O20173
MNKKESILREFALLCYFFIVYLLLPKNAKHPLADKSFLDPHPVARVSPFGSRPPFLLMVP
STSVPNASLESGQSSTISPRRQKESSIPVLQASGSVSANKTFQSTESSALHQPKSSSSER
TGKIITRQRPHSGPTSPRVTPGELLPTLEALKLQEALDFAQLKLKTRQVRYTINLRSQKL
LDTICELNDEKRAEVEEQAAYIFTLQKELVETGYEKVSSYNFTYSRLKQKAEELKYASKK
EVAQLVNSL
>P48496
MAVVSTSLASQITNPNSAVSTQFSGLRRSFLKLENSVSTQSSFFQNVDSHLRLSSSSRRC
PRGVVAMAGSGKFFVGGNWKCNGTKESITKLVSDLNSATLEADVDVVVAPPFVYIDQVKS
SLTGRVEISAQNCWIGKGGAFTGEISVEQLKDLGCQWVILGHSERRHVIGEQNEFIGKKA
AYALNQGVGVIACIGELLEEREAGKTFDVCYQQLKAFADALPSWDNVVVAYEPVWAIGTG
KVASPDQAQEVHVAVRDWLKKNVSEEVASKTRIIYGGSVNGGNCAELAKQEDIDGFLVGG
ASLKGPEFATIVNSVTAKKVAA

>P34802

MASVTLGSWIVVHHHNHHHPSSILTKSRSRSCPITLTKPISFRSKRTVSSSSSIVSSSVV
TKEDNLRQSEPSSFDFMSYIITKAELVNKALDSAVPLREPLKIHEAMRYSLLAGGKRVRP
VLCIAACELVGGEESTAMPAACAVEMIHTMSLIHDDLPCMDNDDLRRGKPTNHKVFGEDV
AVLAGDALLSFAFEHLASATSSDVVSPVRVVRAVGELAKAIGTEGLVAGQVVDISSEGLD
LNDVGLEHLEFIHLHKTAALLEASAVLGAIVGGGSDDEIERLRKFARCIGLLFQVVDDIL
DVTKSSKELGKTAGKDLIADKLTYPKIMGLEKSREFAEKLNREARDQLLGFDSDKVAPLL
ALANYIAYRQN

>P30956

MATMVAGISLRGPVMSSHRTFSVTKRASLPQSKLSSELSFVTSQLSGLKISSTHFISSSA
PLSVPFKPSLQPVARRICPFTGKKSNRANKVSHSNHKTKKLQFVNLQYKRIWWEAGKRYV
KLRLSTKAIKTIEKNGLDAVAKKAGIDLSKK

>O49937

MATSTSSSLSLSFFSSSLFSSKSRNFSSKPILKLPSSSHSQTSLSLSIKSELIPLPILNF
SGEKVGETFLNLKTAPPEKARAVVHRGLITHLQNKRRGTASTLTRAEVRGGGRKPYPQKK
TGRARRGSQGSPLRPGGGVIFGPKPRDWTIKMNKKERRLALSTAIASAVGNSFVVEEFAE
NFEKPKTKDFIAAMQRWGLDPAEKSLFFLMDLVENVEKSGRNIRTLKLLTPRSLNLFDVL
NAEKLVFTEGTIQYLNQRYGVDTLEDEDEEEEEEEEGEEVDDGVEDGTPEPAE

>P42757

MAFPIPARQLFIDGEWREPLLKNRIPIINPSTEEIIGDIPAATAEDVEVAVVAARKAFKR
NKGRDWAALWSHRAKYLRAIAAKITEKKDHFVKLETLDSGKPRDEAVLDIDDVATCFEYF
EYFAGQAEALDAKQKAPVTLPMERFKSHVLRQPIGVVGLISPWNYPLLMDTWKIAPALAA
GCTTVLKPSELASVTCLEFGEVCNEVGLPPGVLNILTGLGPDAGAPIVSHPDIDKVAFTG
SSATGSKIMASAAQLVKPVTLELGGKSPVIMFEDIDIETAVEWTLFGVFWTNGQICSATS
RLLVHESIAAEFVDRMVKWTKNIKISDPFEEGCRLGPVISKGQYDKIMKFISTAKSEGAT
ILCGGSRPEHLKKGYYIEPTIITDITTSMQIWKEEVFGPVICVKTFKTEDEAIELANDTE
YGLAGAVFSKDLERCERVTKALEVGAVWVNCSQPCFVHAPWGGVKRSGFGRELGEWGIEN
YLNIKQVTSDISDEPWGWYKSP

>Q10712

MATLRVSSLFASSSSSLHSNPSVFTKYQSSPKWAFSFPVTPLCSKRSKRIVHCIAGDTLG
LTRPNESDAPKISIGAKDTAVVQWQGDLLAIGATENDMARDENSKFKNPLLQQLDSELNG
LLSAASSEEDFSGKSGQSVNLRFPGGRITLVGLGSSASSPTSYHSLGQAAAAAAKSSQAR
NIAVALASTDGLSAESKINSASAIATGVVLGSFEDNRFRSESKKSTLESLDILGLGTGPE
IERKIKYAEHVCAGVILGRELVNAPANIVTPAVLAEEAKKIASTYSDVISVNILDAEQCK
ELKMGAYLAVAAAATENPPYFIHLCFKTPTKERKTKLALVGKGLTFDSGGYNLKVGARSR
IELMKNDMGGAAAVLGAAKALGEIRPSRVEVHFIVAACENMISAEGMRPGDIVTASNGKT
IEVNNTDAEGRLTLADALIYACNQGVEKIIDLATLTGAIMVALGPSVAGAFTPNDDLARE
VVEAAEASGEKLWRMPMEESYWESMKSGVADMINTGPGNGGAITGALFLKQFVDEKVQWL
HLDVAGPVWSDEKKNATGYGVSTLVEWVLRN

>Q9TL28

MPYIKHIETKRISARTYYGRFCVLPLPAGQGITLGNALRRILLGDLVGFAATSANLAGAS
HEFDTLPGIRESVLEILLNIKQLVFKQISSRPKDTNRFAMRASLNLTGPATVTAKDLVLP
SWMKVVDPSQYIATLASGASLEFEIQLSQGSGYRLRRTSLVPPGFTLPPPRDPLEPENDS
KSETKSKSKGKSKNTSTSDVQLADTDVNAQIIDTDSNSTETEKEAPHIPSMRDDHMTLHI

DAVFFPVTRVNYRVEEEVINGRRREELVIDIWTNGSLSPRKALDQAAVILIRMLASLQAP
PPLLIEPEKKPTTKTIAKEIALTPIESLDLSVRSFNCLKRANITNVGKLIAYTRQELLQL
KNFGTKSASEVVDVLNSRFKLALKGEEVTDQEQVVNQPSSQIATKKGARKKVNRASRPID
SKETRRSRNPVKSTASEVPEKMLRKSSKTKVKAPKSETLPKPSKSANLQQAEESLQVPKL
RRKSELSSSQNPEET

>P30524

MSSMQFSSVLPLEGKACVSPVRREGSACERLKIGDSSSIRHERASRRMCNGGARGPAATG
AQCVLTSDASPADTLVLRTSFRRNYADPNEVAAVILGGGTGTQLFPLTSTRATPAVPIGG
CYRLIDIPMSNCFNSGINKIFVMTQFNSASLNRHIHRTYLGGGINFTDGSVEVLAATQMP
GEAAGWFRGTADAVRKFIWVLEDYYKHKSIEHILILSGDQLYRMDYMELVQKHVDDNADI
TLSCAPVGESRASEYGLVKFDSSGRVIQFSEKPKGDDLEAMKVDTSFLNFAIDDPAKYPY
IASMGVYVFKRDVLLNLLKSRYAELHDFGSEILPRALHDHNVQAYVFTDYWEDIGTIRSF
FDANMALCEQPPKFEFYDPKTPFFTSPRYLPPTKSDKCRIKEAIISHGCFLRECKIEHSI
IGVRSRLNSGSELKNAMMMGADSYETEDEISRLMSEGKVPIGVGENTKISNCIIDMNARI
GRDVVISNKEGVQEADRPEEGYYIRSGIVVIQKNATIKDGTVV

>Q06RB0

MEKSERPLIKKKRPFRKKKRSFRKRRSPIESGHQIDFTNTSLLNQFISQQGKILPRKVTR
LTLKQQRFMTSAIKQARILSLLPFVFDDKKVKKKQKEEFQKKQKEEFQKKQKEEFQKKQK
EEFQKKQKEEFQKKQKEEFQKKQFQKKEFQRTKSTARTTNEKQTNEKQTKSTARTTNEKQ
TKSTARTTNEKQTKSTARTTNEKQTKSTARTTNEKQTKSNDRTTDLRTRKK

>Q9S7W5

MNIVSWKDANDEVAGGATTRREREVKEDQEETEVRATSGKTVIKKQPTSISSSSSSWMKS
KDPRIVRVSRAFGGKDRHSKVCTLRGLRDRRVRLSVPTAIQLYDLQERLGVDQPSKAVDW
LLDAAKEEIDELPPLPISPENFSIFNHHQSFLNLGQRPGQDPTQLGFKINGCVQKSTTTS
REENDREKGENDVVYTNNHHVGSYGTYHNLEHHHHHHQHLSLQADYHSHQLHSLVPFPSQ
ILVCPMTTSPTTTTIQSLFPSSSSAGSGTMETLDPRQMVSHFQMPLMGNSSSSSSQNIST
LYSLLHGSSSNNGGRDIDNRMSSVQFNRTNSTTTANMSRHLGSERCTSRGSDHHM

>Q32063

MTCTRTLLVLNRMLALKANFVLHSVKSYLNVSGVNKTLPAKGSAGVKNGFILSHLGCQ

>Q85CT7

MNIINLPELNYEVFFVFIELGLIFGSLGVVFLTNIVYSAFLLGLVFVCISFLYLLLDADF
VATAQILIYVGAVNILIVFAVMLINKPQSLQFLPSWTVGDTITLILCTSLFFLLISMILS
ISWSNIFSIAQSNKIGEQVLKSSVQGIGSSLLIDFLLPFELLSIVLLVALIGAITIARRE
KKVKLQKNRTLQVTKDSFIL

>Q93WX6

MEGVAMKLPSFPNAISIGHRSFSRVRCSSSLSVCSAAAASSATISTDSESVSLGHRVRKD
FRILHQEVNGSKLVYLDSAATSQKPAAVLDALQNYYEFYNSNVHRGIHYLSAKATDEFEL
ARKKVARFINASDSREIVFTRNATEAINLVAYSWGLSNLKPGDEVILTVAEHHSCIVPWQ
IVSQKTGAVLKFVTLNEDEVPDINKLRELISPKTKLVAVHHVSNVLASSLPIEEIVVWAH
DVGAKVLVDACQSVPHMVVDVQKLNADFLVASSHKMCGPTGIGFLYGKSDLLHSMPPFLG
GGEMISDVFLDHSTYAEPPSRFEAGTPAIGEAIALGAAVDYLSGIGMPKIHEYEVEIGKY
LYEKLSSLPDVRIYGPRPSESVHRGALCSFNVEGLHPTDLATFLDQQHGVAIRSGHHCAQ
PLHRYLGVNASARASLYFYNTKDDVDAFIVALADTVSFFNSFK

>Q32065

MTFLNHYTYLFSIPEKQADKVSGILRLAQARPIETLQNERINKQLNAFLKTYKFEKLITN
YKKMQSFIPNNSLNGNKTNSSTNKLYATSLNVFPENPPLMVRKAVSDEADKFSKFTYSKV
QVVTNNLNNGMNSKEFIKANNLKPSLRAAESLVLNHLTYNKFKENLYFKTNNIQPTKSKS
TSLFFLNILSNSKPRTCSDFLSSPKIRKTWFRNTAWSLQTQQHRSSNGINLSLQLPYALG
PSVPAGASGQNMYELPVAQSSSRFGTYYFLQKLLSKYLDVWNASADNGSVLSNSENIKLN
FSMVSLLDSKMAIQTPNSLYFVFTQLNQKTFLSYWLLPVAGLALLTPTLLTLTGQSVSVQ
KFNSFINKKTDMMVLSNTEMPSKSFGTPTLFGTSVEIYLPNSYMPKGEGESGINRVNSSI
NAVKKNTVTANLVLDSESQEVATSFQNDLISIKYCFNNLYNYISNKTALSTKNLFLFSAI
KSNATKHKRTQSFFSVENTTTLGNNSNFVKGHFKSSINAFSSYLPSTNVHSMIPLTSLPY
LKAISPLYSKFMIDHSLKFITPKTTLKLLQHKLNKSPKQMYTKTQNFTGLRDLRALNSFS
FGQVNFRTNHFLHSNSRPLNHYNQALKLINGYEQYKNNLQINCNKTLDLNTKNKLVYQVH
KSHLFNQKCSQIVYKQSLYNRDLCTIRGTGTKVVDYFSHGDKLSNKNGIVLDYFVYSNLL
FDNKTNTIINKDGKQNITKLKLNLTKTTVPFKTLIKKYTSINSLVANEQTRNNLNLGLIH
FNGHLSVVSNANLLTGRPVKFIYYKFDKRLNSYLIYVNQNLKKFIQLNNNFLKPKPLSHQ
KNKPVEDFNQYATNNSSPPKTNVFEKSFVEDSSLRKPLTSLRGSKQFLNSLTILFKHQKM
FKKKTLKAHKWHSDTQGIFRKHTNSSFGSANFSNGPEESSLSTRLHIQKKRKAKKQRLET
RRQKKRTRFFPRPVWLRSRMFLNFLTERNKYYLNSTITKQGFSLPSKDVVTTKLDWLKED
MRPSSLGAYQYKSLLTQKAGNKFQRQSFTEVVSTMEYINGIHKALNNSIFNKIVRKSLLS
SSQNPLKLRLVANYSKMQFMHRVKLPFYRTLKHSEGTKNLANKKQNLRDIKIKANYNNFK
SQKANNQPQQNDKDKDKDTMFRDFWVWSYNNTQTNAFNQNLWWLLPNLTTKQSNLEFLTS
TYPTAKETQRAKEEIHGNSIPTASKNQIALIRLNWALNKTNINTFTDYSKRNNLWTTQKL
RNQSKNNKTKSLEKQFITNWEKFFLNKNLNIFSKKIISKVKQKKQKLNYMTSYLNVQSEH
NVKIFHNSWWTHLNIKNLVNNQDMVIPVREGYFSVGNFNSEFINSAIIKSINNKTLVENY
VYSPSSEKETMQLLLMSSSILLHLCAIISLVSISQVRCFVKFHLILLYKLSNVYNAILNQ
LSNKLQKNLPIYNNINKLNSRYFYMNHQKSQIKQRKKLLTYFSLTLLKKQFVTVKPLQIR
NFASIKNQSSNNSNLTYTDMLPLSLRANKFRGSKYDISIREEEGQSAHIKPSKSMYAKLN
ILSLKTIFLKQLLMNKKPSALPSNVGLKSNRETQKSQLIQRIKTKELQISLKKNIIGFSK
VTKNHILKILFNVIEVFQTAVRNISSFFEKPAEFTTTWIAYGFLVEWSSDFITIIPENVD
IYIWNVFSKIYRTIPLSFISTTLGPASTVFDPVTNSTIPIQMGNFNYQKMVAFPILLSLS
HLLHRRILYLFDTLFSTITQPDTDLIARQEKGTLFWDIWADFLVTAADYYNVNVAALSTI
KAEQNSLIENISNDFDNLTMSSKKPFFMPNKGVSNIKNIFWIKKLKEPQLPESIVQNREV
FVRERKRTLKGLFNIYAPQEETLWNNPTSPKNLSDEKISFKLFNQLNLQLFAEKNKIKPY
FEAYFSTTQQKTNIMQSAFPEANLNRWSVNQFITYQSWHSHNGSNNSNGDLFIDYHPPKT
FSHIPALKYNSILQQPIGSLVCQIYSGLFNKQISKNILLVNPKTTSNNLVDYNVLLIQAL
AGETEMKIITDNAQRYALVNRGFAIGIKLLREVFDAIALNTPCIFLLEDIHAIGERRPML
ISDFGGGMSDDNGSFKEDFFGSQRDEVHEKNQVVYQLTRHAITHYKKPFKGDYSLAIPTN
LYVTDLFLKLPTQSISNLTNVENHNLSIKNKIQHNGTQSLTETKRNLGGDINKNSYLQLT
QFTKTLAPPSTSPFSVLLLKEEKRLKPNKIVEELPWTSLPGEQLATKPRTSYSVRAKVAM
LAELSLSNLSAKLDMITDLLVIIDSVRSNKGFVVFATTDIPHVLDPALRRPGRLDETICL
PNIHTSNILNFTKNYEIFKSAKDTSNFGKKIILNEMQNLTTTSTQRDMYLSCLPTNNQTH
KTKREGVLTMNLKDYNILLNQVYFAEGTGGILNSQMHKDSLQKSLNFALISHSKKLKELN
VSKLIGSNGTVSQGNVDQLGVFAGQIVNKQKKSLQQHLPNSKKSFKKKYKDKAIIYYEVG
KFVLNYFLNNQLTQSSIIDKPVSVTNKQTNDITIFGNDFLNLKTINYLSLYNSKNKILLQ
LMLIFGGKISQLLSSKNLVKSLKQASINSYMVEEESGSISSAGMPLGQTHLLPKALSVLA

KPMIFSDGYNNQNLKTATTLLLSFIHKRYLYRKNLIVPKLLSFADGNILDEPPSPPFSSL
LIPAKRFENYKRFFRDTLTGDKMGQRKSQITLLEKLQYHMQLRSIKQLNATFSSQENLDF
QSNAALTSQKLDTLMSLSTNNLLQNPTNINWYYQNRILKRHGQYLTNQWWNGQLSEHNAE
TVFLSDIDWRSSFIKNKNINITKSKNLYRLTQQKNNTDGLDVLLDFPDTDQYYNPKRRRW
LLNNGSWNFWFNFDKLYSEEIVTTWILESLIQTYKYLHKNTELLDFVTNKFITLGYIAPE
NANLQNISGFPSQSELLSTKEIILTNSFKRF

>P06258
MFLLYEYDFFWAFLIISILVPILAFLISGVLAPISKGPEKLSTYESGIEPMGDAWLQFRI
RYYMFALVFVVFDVETVFLYPWAMSFDVLGVSVFIEAFIFVLILIIGLVYAWRKGALEWS

>P24613
MASATLAFSCSSLCATLKLPQNLNPLLLNVPPLSKPFSGVVSPPSLSRLSLLPVAAKRRR
FQEIPEELKAEFEEFQRPPNQKPQLSDVLPDDFQAPEPGTPEYNDIINQFLPKKGPPPPR
EEIFAVVVIGSRQYIVIPGRWIYTQRLKGATVNDKIVLNKVLLVGTKASTYIGTPIVTNA
AVHAVVEEQLLDDKVIVFKYKKKKNYRRNIGHRQPITRIKITGITGYEDYPASTLEAEVE
AKEEAEAEAEAEAVPV

>P56809
MLLLKHGRIEILDQNTMYGWYELPKQEFLNSEQPELLLTTSKKFPLIKDGNPLENQKYAC
RIKLLLLSVPITNQLNN

>P28759
MASLGGLQNVSGINFLIKEGPKVNAKFELKPPPYPLNGLEPVMSQQTLEFHWGKHHKTYV
ENLKKQVVGTELDGKSLEEIIVTSYNKGDILPAFNNAAQVWNHDFFWECMKPGGGGKPSG
ELLELIERDFGSFVKFLDEFKAAAATQFGSGWAWLAYRARKFDGENVANPPSPDEDNKLV
VLKSPNAVNPLVWGGYYPLLTIDVWEHAYYLDFQNRRPDYISVFMDKLVSWDAVSSRLEQ
AKALITSA

>Q06GS8
MVKLRLKRCGRKQRAVYRIVAIDVRSRREGRDLQKVGFYDPIKNQTYLNVPAVLYFLGKG
AQPTGTVHDISKKAEIFKELRVNQTIRVNQTKGGLHI

>Q43064
MTASSSLFSCSMHMEVLTPKISKWPKDFVSCHSKISYVETNYLKSTCYPISRFLCINNLS
KCDKMIKTRQRDGIHCFSEGQKFQLDDVIEAQQFDRDILNAIFEIARDMENIERNSPESQ
ILKGYLMATLFYEPSTRTRLSFESAMRRLGGEVLTTENAREFSSAAKGETLEDTIRTVEG
YSDLIVLRHFESGAARRAAAIAGIPIVNAGDGPGQHPSQALLDVYTIEREIGKLDGIKVG
LVGDLANGRTVRSLAYLLAKYKDVKIYFVSPEVVKMKDDIKDYLTSKGVDWEESSDLVEV
ASECDVVYQTRIQKERFGERLDLYEKARGKFIVNQNILNAMQRHAVIMHPLPRLDEITVD
VDADPRAAYFRQAKYGLYIRMALLKLLLVGW

>A4GYN8
MPTIKQLIRNTRQPIRNVTKSPALGGCPQRRGTCTRVYSGSKGDLSVNFSTITPKKPNSA
LRKVARVRLTSGFEITAYIPGIGHNSQEHSVVLVRGGRVKDLPGVRYHIVRGTLDAVGVK
DRQQGRSQYGVKKPK

>Q7YKY4
MNLISKKNELKQGFYPLFFLEEFYIRVLIHMNHNHILKIGNIKQSKLIHVNRICHYLLIK
RLIRQIRKQSHKYNGISEFPNYETEFYFQYKNRFYNLMIENVFLLILQMIWQHQKTRKND
PCILIHRSIQSTFPFLENKIIHCIWIIHGNIQLFHTIQQLNFLFLLLYERIRDKSFLHLL

KNIFNLKKELLIEAFYCDKFHLIELSMFFRNLYINEFDSFIVYHIVKTWKLAYLLNPSQA
IDDSSFIQKNHILLNIKRKQKSLPLVSWLANRSFYSLYGNIHYVRRDLSFLMAIQAGKHI
SRFWKYNSINFLQLKLGFPCSLDVLYLKSLFNQDFLFLGYRIVNKLWKKNFKIRAVSWYS
PILFFFKGRRISTKMPVLNLIHRLSVMHLCNLEGYPIHKAAWSVFNDKQIMNIFSNLLRN
IILYYSGCSNRSDLGKIQYILEFSCMKTLAFKHKSSIRSTWTQYKKHVSLLSLVKNRHKN
GKTSVDLYFLFQKTNKLWLLDLSKIQDSLACFIFID
>P34811
MAAESSLRVATPTLCNLNGSQRRPTTTTLSPLRFMGFRPRPSSHSLTSSSLSHFFGSTRI
HSNSSSSYSSISRQHAPRRNFSVFAMSADGDAKRSVPLKDYRNIGIMAHIDAGKTTTTER
ILYYTGRNYKIGEVHEGTATMDWMEQEQERGITITSAATTTFWNKHRINIIDTPGHVDFT
LEVERALRVLDGAICLFDSVAGVEPQSETVWRQADKYGVPRICFVNKMDRLGANFYRTRD
MIVTNLGAKPLVIQLPIGSEDNFKGVIDLVRNKAIVWSGEELGAKFDIVDIPEDLQEQAQ
DYRAQMIENIVEFDDQAMENYLEGIEPDEETIKKLIRKGTISASFVPVMCGSAFKNKGVQ
PLLDAVVDYLPSPLDLPAMKGSDPENPEATIERLASDDEPFAGLAFKIMSDPFVGSLTFV
RVYAGKLGAGSYVLNANKGKKERIGRLLEMHANSRDDVKVALAGDIIALAGLKDTITGET
LCDPDNPIVLERMDFPDPVIKVAIEPKTKADVDKMATGLIKLAQEDPSFHFSRDEEINQT
VIEGMGELHLEIIVDRLKREFKVEANVGAPQVNYRESISKISEVKYVHKKQSGGQGQFAD
ITVRFEPMDPGSGYEFKSEIKGGAVPREYIPGVMKGLEECMSNGVLAGFPVVDVRAVLTD
GSYHDVDSSVLAFQLAARGAFREGIRKAGPRMLEPIMKVEVVTPEEHLGDVIGDLNSRRG
QINSFGDKPGGLKVVDSLVPLAEMFQYVSTLRGMTKGRASYTMQLAMFDVVPQHIQNQLA
TKEQEVAA
>Q9LE95
MATAAPPSLSLCYASSSFQYQQDPSFQTHFKPLLLNSSLCRLTLNQRERSCLKWVDFTSQ
KQSPKSVSFRVLAAVAAEAEVADMEEEEGESGGVATLPSPTKPKKGKAALPLKSDRTRSK
RFLEIQKLREIKQEYDLKTALSLMKQMSSTKFVETAEAHFRLNIDPKYNDQQLRATVSLP
KGTGKTVKIAVLAQGDKIDEAKAAGADIVGGEELIEQIKGGFMDFDKLIATSDMMAKVAS
LGRILGPRGLMPTPKAGTVTPNVAQAVEEFKKGKVEFRVDKTGIVHIPFGKLNFEEEDLL
INLFATIKSVETNKPTGAKGVYWKSAHISSSMGPSIRLNIREMLDYKPPSNA
>Q9MUP3
MYFQNIDALIFSKLLDTLLNIFFLLFFLVLGAEFTLYIINVYFPNLLLYGNALGIKFVLS
ILGSLIAFPIGLVFQNNYKNFLNYINNFTVDIFFTSLLSIIGALFIANLYFSFLDISSIL
KEFLFIKYLLRLLIQTILCYSGFILVTFHSDYLLNILVPEMAEAIFSYNKIVRPSITRVL
HTSYIINHNIIAIFQSQFVEGLVLIPKSTIQELQILNNSKNFKDKYLGRLGFSRLNELKY
NYFDQIILESSLTNCHENVLFKYNKTYNNEQQNIVLMSSCSNNIKESNISQLLISNLFPL
IININKLIELLKLPYLSGESFYLQIVRKGKRLGQGIGYLEDGTMVIVNAGSKYIGQKIKV
IVKKVWQKSTGKILFTQPIKNS
>P25851
MAATAATTTSSHLLLSSSRHVASSSQPSILSPRSLFSNNGKRAPTGVRNHQYASGVRCMA
VAADAAETKTAARKKSGYELQTLTGWLLRQEMKGEIDAELTIVMSSISLACKQIASLVQR
AGISNLTGVQGAVNIQGEDQKKLDVISNEVFSNCLRSSGRTGIIASEEEDVPVAVEESYS
GNYVVVFDPLDGSSNIDAAVSTGSIFGIYSPNDECIVDDSDDISALGSEEQRCIVNVCQP
GNNLLAAGYCMYSSSVIFVLTLGKGVFSFTLDPMYGEFVLTQENIEIPKAGRIYSFNEGN
YQMWDDKLKKYIDDLKDPGPTGKPYSARYIGSLVGDFHRTLLYGGIYGYPRDAKSKNGKL
RLLYECAPMSFIVEQAGGKGSDGHSRVLDIQPTEIHQRVPLYIGSTEEVEKLEKYLA

>P27456

MNQAMATPLSLSCCSPTLTRSTLFFTKTFPFSRSFSTPLPLSTKTLISLSPPHRTFAVRA
ESQNGADPARQYDFDLFTIGAGSGGVRASRFASNFGASSAVCELPFSTISSDTTGGVGGT
CVIRGCVPKKLLVYASKFSHEFEESNGFGWRYDSEPKHDWSSLIANKNAELQRLTGIYKN
TLKNAGVKLIEGRGKIVDAHTVDVDGKLYSAKHILVSVGGRPFIPDIPGKEYAIDSDAAL
DLPSKPQKIAIVGGGYIALEFAGIFNGLKSEVHVFIRQKKVLRGFDEEIRDFVAENMALR
GIEFHTEESPVAITKAADGSLSLKTNKGTEEGFSHIMFATGRSPNTKDLGLESVGVKVAK
DGSIEVDEYSQTSVPSIWAIGDATNRVNLTPVALMEGVALAKTLFQNEPTKPDYRAIPSA
VFSQPPIGGVGLTEEQAAEQYGDIDVFTANFRPMKATLSGLPDRVFMKLIVSAETNVVLG
LHMCGEDAAEIAQGFAVGIKAGLTKADFDATVGIHPTAAEEFVTMRTPTRKVRKNQASQG
KSDSKAKAVAGS

>Q85FL6

MVLTSDHLDKKNIRKIEYKGEDSFSNSMSKLPLQGGMSDSIFMASISDFSNWSRLSSLWP
LLYGTSCCFIEFASLIGSRFDFDRYGLVPRSSPRQADLIVTAGTITMKMAPSLIRLYEQM
PEPKYVIAMGACTITGGMFSTDSYSTVRGVDKLIPVDIYLPGCPPKPEAIMDAVTKLRKK
IARNRFVNRASRPIRTKYFSISHQLNLVPGTCAGKYNWDRENCGTKLAPANFSENCQKFA
NEHDELKSAI

>Q93Y52

MALAIRSSLRAAAMGRKAFRQAVPVRVAPAQRVRSVTTASAEITAYSVEEKGPKDSLEYR
MFFKQGAKEVSCWHEIPLYAGDGHLHYICEIPKETSAKMEVATDEPRTPIKQDVKKGKLR
FYPYNINWNYGMLPQTWEDPGHTDATLGAAGDNDPVDVVEIGAAAAKRGGVYKVKPVGVL
AMIDDGELDWKVIAISADDPKAALCNDVEDVEKHFPGEIQKVLEWFRDYKIPDGKPANKF
GYDNKCMNKEFTLNVIKETHEAYVKLKSGARANSEELSLI

>Q9M4G5

MAMESALTSTRVSIPSLCSGISSSHHHHRSLSFLNFPKLSSFKYSFRTISPVPFVVSASS
VSPSSPSTSVAQSQDLKIKSVPTKPIEGQKTGTSGLRKKVKVFMQDNYLANWIQALFNSL
PLEDYKNGLLVLGGDGRYFNREAAQIIIKIAAGNGVGKILVGKDGILSTQAVSAVIRKRE
ANGGFIMSASHNPGGPEYDWGIKFNYSSGQPAPESITDKIYGNTLSISEIKIADIPDVDL
SQLGVTKYGNFSVEVVDPVADYLELMENVFDFSLIRSLVSRPDFRFVFDAMHAVTGAYAK
PIFVDKLGASLESIANGVPLEDFGHGHPDPNLTYAEDLVNILYGENGPDFGAASDGDGDR
NMILGRSFFVTPSDSVAIIAAQCQYAIHYFQSGPKGLARSMPTSGSLDRVAQKLNLPFFE
VPTGWKFFGNLMDAGKLSICGEESFGTGSDHIREKDGIWAVLAWLSILAYRNKDKKSGEK
LVSVADVVKDHWATYGRNFFSRYDYEECESEGANNMIEYLRDLISKSKAGDKYGSYSLDF
ADDFAYTDPVDGSVASKQGVRFVFSDGSRIIFRLSGTGSAGATVRIYIEQFEPDVSKHDM
DAQIALKPLIDLALSVSKLKDFTGREKPTVIT

>Q96529

MSLSSLTLDSNPRFAVGGPYHRRYPPLHHPRSFVSCSAKRPAVSASLSVAADSAATESLG
RIGSLSQVSGVLGCQWGDEGKGKLVDILAQHFDIVARCQGGANAGHTIYNSEGKKFALHL
VPSGILNEDTTCVIGNGVVVHLPGLFKEIDGLESNGVSCKGRILVSDRAHLLFDFHQEVD
GLRESELAKSFIGTTKRGIGPAYSSKVIRNGIRVGDLRHMDTLPQKLDLLLSDAAARFQG
FKYTPEMLREEVEAYKRYADRLEPYITDTVHFINDSISQKKKVLVEGGQATMLDIDFGTY
PFVTSSSPSAGGICTGLGIAPSVVGDLIGVVKAYTTRVGSGPFPTENLGTGGDLLRLAGQ
EFGTTTGRPRRCGWLDIVALKFSCQINGFASLNLTKLDVLSDLNEIQLGVAYKRSDGTPV
KSFPGDLRLLEELHVEYEVLPGWKSDISSVRNYSDLPKAAQQYVERIEELVGVPIHYIGI

GPGRDALIYK

>P42698

MLGGLYGDLPPPTDDEKPSGNSSSVWSSSTKMAPPTLRKPPAFAPPQTILRPLNKPKPIV

SAPYKPPPPSNSSQSVLIPANESAPSHQPALVGVTSSVIEEYDPARPNDYEEYKREKKRK

ATEAEMKREMDKRRQEDEERDKREREEREKERERDNSDPSRLNISGEEAWKRRAAMSGGG

SGGKGRSSSPPGNVDGFSIGKSETSGLGVGAGGQMTAAQRMMAKMGWKQGQGLGKSEQGI

TTPLMAKKTDRRAGVIVNASENKSSSAEKKVVKSVNINGEPTRVLLLRNMVGPGQVDDEL

EDEVGGECGKYGTVTRVLIFEITEPNFPVHEAVRIFVQFSRPEETTKALVDLDGRYFGGR

TVRATFYDEEKFSKNELAPVPGEIPGY

>Q9XIR4

MLLVSPACRGVYLQTIDPKPIDFSARASYALCFQIPTSIPKRECLMRLGTVFCFNQKHRE

QTSFKKRYVSTQNVDLPPILPKNKKKPYPIPFKQIQEEARKDKKLAQMGIEKQLDPPKNG

LLVPNLVPVADQVIDNWKLLIKGLAQLLHVVPVFACSECGAVHVANVGHNIRDCNGPTNS

QRRGSHSWVKGTINDVLIPVESYHMYDPFGRRIKHETRFEYERIPALVELCIQAGVEIPE

YPCRRRTQPIRMMGKRVIDRGGYHKEPEKPQTSSSLSSPLAELDTLGVFERYPPPTPEDI

PKIAQETMDAYEKVRLGVTKLMRKFTVKACGYCSEVHVGPWGHSVKLCGEFKHQWRDGKH

GWQDALVDEVFPPNYVWHVRDLKGNPLTGNLRRFYGKAPALVEICMHSGARVPQRYKAMM

RLDIIVPDSQEADMVA

>Q06FW2

MAKGKDNRGKVTLECTGCVQNRVNNKKKKKSAGIYRYLTQKNPKNTPSPLELRKFCPYCN

KHTIHGEIKE

>Q84Y95

MALRSVKTPTLITSVAVVSSSVTNKPHSIRFSLKPTSALVVHNHQLSFYGSNLKLKPTKF

RCSASALTPQLKDTLEKLVNSEKVVLFMKGTRDFPMCGFSNTVVQILKNLNVPFEDVNIL

ENEMLRQGLKEYSNWPTFPQLYIGGEFFGGCDITLEAFKTGELQEEVEKAMCS

>O24474

MALVSISPLASKSCLRKSLISSIHEHKPPYRTIPNLGMRRRGKSVTPSMSISLATAAPDD

GVQRRIGDYHSNIWDDDFIQSLSTPYGEPSYQERAERLIVEVKKIFNSMYLDDGRLMSSF

NDLMQRLWIVDSVERLGIARHFKNEITSALDYVFRYWEENGIGCGRDSIVTDLNSTALGF

RTLRLHGYTVSPEVLKAFQDQNGQFVCSPGQTEGEIRSVLNLYRASLIAFPGEKVMEEAE

IFSTRYLKEALQKIPVSALSQEIKFVMEYGWHTNLPRLEARNYIDTLEKDTSAWLNKNAG

KKLLELAKLEFNIFNSLQQKELQYLLRWWKESDLPKLTFARHRHVEFYTLASCIAIDPKH

SAFRLGFAKMCHLVTVLDDIYDTFGTIDELELFTSAIKRWNSSEIEHLPEYMKCVYMVVF

ETVNELTREAEKTQGRNTLNYVRKAWEAYFDSYMEEAKWISNGYLPMFEEYHENGKVSSA

YRVATLQPILTLNAWLPDYILKGIDFPSRFNDLASSFLRLRGDTRCYKADRDRGEEASCI

SCYMKDNPGSTEEDALNHINAMVNDIIKELNWELLRSNDNIPMLAKKHAFDITRALHHLY

IYRDGFSVANKETKKLVMETLLESMLF

>P05466

MAQVSRICNGVQNPSLISNLSKSSQRKSPLSVSLKTQQHPRAYPISSSWGLKKSGMTLIG

SELRPLKVMSSVSTAEKASEIVLQPIREISGLIKLPGSKSLSNRILLLAALSEGTTVVDN

LLNSDDINYMLDALKRLGLNVETDSENNRAVVEGCGGIFPASIDSKSDIELYLGNAGTAM

RPLTAAVTAAGGNASYVLDGVPRMRERPIGDLVVGLKQLGADVECTLGTNCPPVRVNANG

GLPGGKVKLSGSISSQYLTALLMSAPLALGDVEIEIVDKLISVPYVEMTLKLMERFGVSV

EHSDSWDRFFVKGGQKYKSPGNAYVEGDASSASYFLAGAAITGETVTVEGCGTTSLQGDV

KFAEVLEKMGCKVSWTENSVTVTGPPRDAFGMRHLRAIDVNMNKMPDVAMTLAVVALFAD
GPTTIRDVASWRVKETERMIAICTELRKLGATVEEGSDYCVITPPKKVKTAEIDTYDDHR
MAMAFSLAACADVPITINDPGCTRKTFPDYFQVLERITKH
>P03938
MAYSSCLNRSLKPNKLLLRRIDGAIQVRSHVDRTFYSLVGSGRSGGGPPGLLSSRESIHP
LSVYGELSLEHRLRFVLNGKMEHLTTHLHRPRTTRSPLSFWGDGGIVPFEPFFHAFPGGL
EKAVINRTSLILPS
>O80439
MASLILGAPPRVTVALPSSRLSSSHSETAGVSLSCFTHQFSLSTSSSSSIPLVYCGRGDR
KTAKGKRFNHSFGNARPRNKSKGRGPERVPVPPAPPRKDKFENDEKIKIDIDESLFSN
>Q9MUQ5
MNKFFSINIILWALIGLNFHILSYSLKISISNFPWQWPQEGLETYMIHTDYYLGSILFIS
FLGGRFASLISQLAYQIIQINIVQLIIFFSKNLHISFLLPNILNQLINLDTNRGTITEYT
CIQDHIINNGWLSYFVVNLISYDLFENTLNQYNTQAIDSFFNKSVIFLIRLYIMNILFSL
LTSNSLNEWIMYSIKDNFLLLPNQLIIICSVVLLVFLLQFCVY
>Q9ZS97
MASLLANGISSFSPQPTSDSSKSPKGFHPKPESLKFPSPKSLNPTRPIFKLRADVGIDSR
PIGASESSSSGTSTVSSTDKLQQYFQNLDYDKKYGFVEDIDSFTIPKGLSEETIRLISKL
KEEPDWMLEFRFKAYAKFLKLEEPKWSDNRYPSINFQDMCYYSAPKKKPTLNSLDEVDPQ
LLEYFDKLGVPLTEQKRLANVAVDAVIDSVSIATTHRKTLEKSGVIFCSISEAIREYPDL
IKKYLGRVVPSDDNYYAALNSAVFSDGSFCYIPKNTRCPMPISTYFRINAMETGQFERTL
IVAEEGSFVEYLEGCTAPSYDTNQLHAAVVELYCGKGAEIKYSTVQNWYAGDEQGKGGIY
NFVTKRGLCAGDRSKISWTQVETGSAITWKYPSVVLEGDDSVGEFYSVALTNNYQQADTG
TKMIHKGKNTKSRIISKGISAGHSRNCYRGLVQVQSKAEGAKNTSTCDSMLIGDKAAANT
YPYIQVKNPSAKVEHEASTSKIGEDQLFYFQQRGIDHERALAAMISGFCRDVFNKLPDEF
GAEVNQLMSIKLEGSVG
>P27608
MALSSSSTTNSLLPNKSQLVQNQSLLPSPLKNVSFTTNSTKPVRFVQPISAIHSSDSSKN
PIVSDKPSSKPSPPAATVTAAATTVTKTEWTVESWKSKKALQLPEYPNQEELQSVLKTIE
EFPPIVFAGEARSLEERLGEAAMGRAFLLQGGDCAESFKEFNANNIRDTFRILLQMGAVL
MFGGQMPVIKVGRMAGQFAKPRSDNFEEKNGVKLPSYRGDNVNGDAFDAKSRTPDPQRLI
RAYCQSAATLNLLRAFATGGYAAMQRINQWNLDFTEHSEQGDRYRELANRVDEALGFMAA
AGLTVDHPIMKTTEFWTSHECLLLPYEQSLTRLDSTSGLYYDCSAHFIWVGERTRQLDGA
HVEFLRGVANPLGIKVSDKMDPSALVKLIEILNPDNKAGRITIIITRMGAENMRVKLPHLI
RAVRRAGQIVTWVSDPMHGNTIKAPCGLKTRPFDSIRAEVRAFFDVHEQEGSHPGGVHLE
MTGQNVTECIGGSRTVTFDDLSSRYHTHCDPRLNASQSLELAFIIAERLRKRRLGSQNVL
GQ
>Q9MUQ8
MINIEFGPSTILGIAVVCGGILLYITRTIKPEISRDHDIFFSSVALLIGGILIFQGWRLD
PILLFGQMLSTGTALCFIIESLKLRVPKENNKSLFSTQNKNNSNNRIIKFQPPSKYDINL
NQRGKKEKKNNNFQLKENSKYTKDSLVPSNWNDINISSIDYEKPIDYQKK
>Q9LWB5
MATTAPNLHSLSSSFAFSNPSSNVSATSFTFQIPNKKAQISCISSKKLHTQKSFNFHDAV
TPMNKPSFGRDLMVAQATEAVAPTTEEAATSQPKTSKKAKKLKYPRRILDVYQILQSPII

TEAAIKNIADENSLLFTVDVRADKKMIREAISNFFGVKVRKVNTLIRPDGTKKAYIMLNK
EYNASELAKKIGIFPGGN
>P09753
MSRKKTIKDYENLAATRNHEVISVSNKETPSQGDITLLCKTCNKEFTTTTISYQNARKTG
CPHCKATSASLYWTGRARTKTPEQAKKNAEIKEHINKTRKEKGKAFANIKNKEDLKEKLT
NDLYLPNGEKNAYNDFILKRLNDPVTGKMMEKHHIIPLHAGGPDEKWNLISLTPEDHIEA
HNLRYLVYNETGDKNTIKFRNKTPNVTDQISKAKALGNETRRAQGTGIYEPGMSSKAGKI
GGSVKSVEKDLKQSTKMTSGVYDALYNGSRWKHTKTNTEIVIPPNTIVKMPQLVEKLIEA
LPPCEEKTRLAGAKLTTATSALARVIKGKNEGGRSSYFGWSICKE
>Q9M4W3
MAMATSFYCSTAIPSKKTNQNRENFLCSPVGGSKTTPSYIRLSTRQSRTLSLVVSAAASG
AAVEAEPKFAAVTPSKILSFRVGHGFDLHRLEPGYPLIIGGINIPHDRGCEAHSDGDVLL
HCVVDAILGALGLPDIGQIFPDTDPKWKGAPSSVFIKEAVRLMDEAGYELGNLDATLILQ
RPKVSPHKEAIRQNLCQLLGADPCVVNLKAKTHEKVDSLGENRSIAAHTVVLLMRK
>P09754
MDFYNICCKYLKEIELTREDYSADRNSPQYNLFCSHHINPKFNKGGDEPENLVLLHYFEH
AYVHLFRWLITDNPRDLGGFTSAMNAKERRELQIEKRRENPPKLTLPPLPPPAEERKKPA
KTAKAIRAGKAVGSKYQLQSLKRANPFTMFMANLVLKFINNNGIEVIHKPSDNIKDVSVN
SASAIGRALNNVYPTETLTNSPDGISKLLKGTNKIVQNWRIDSLFIDDFEYEITQEVLLK
YQDLFETLTTYFSENQELSILELTKSMADIRNSNPKEIALIQKMFTFIKKWSFLIKSLSE
GKADDEIDLPTD
>Q6JJ61
MAAYSAAALSWSSSSLLGNYKLSKNAPLGTAKLVERTSNFTIVCQKKTKKIRKIILKEDV
QELGKKGQLMNVRAGYYRNYLLPMGMAQIVTPQLLKEMKIEEERIEAEKKRVKEEAQQLA
LIFETVGGFKVKRKGGKGKQIFGSVTAQDLVDIIKAQLQREVDKRIVSLPEIRETGAYTA
ELKLHPEVTARVQVIVSAN
>P23326
MAMASATATLSFKTPSLSLSPPSTRCSAAQGISLTHFNKQLKSTLNLSSSSSISSSKVQP
IVLKNKRISTVDSSVSTSSPSFTVFAAKGYKMKTHKASAKRFRVTGKGKIVRRRAGKQHL
LAKKNTKRKNRLSKLIQVDRSDYDNVIGALPYLKVNRKV
>Q9TKU9
MAVPKKRKSKSRANSQNHVWKREIVKQARRAVSLAKALLGGNTNFLLVSPGPTTPIKPNP
KKQTGRRPRSQRRRT
>P26302
MAFCSPHTTTSLRSPCTTIPNSGFRQNQVIFFTTRSSRRSNTRHGARTFQVSCAVEQPIV
IGLAADSGCGKSTFMRRLTSVFGGAAEPPKGGNPDSNTLISDTTTVICLDDYHSLDRTGR
KEKGVTALDPKANDFDLMYEQVKAIKEGKAIEKPIYNHVTGLLDPAELIQPPKIFVIEGL
HPMYDERVRELLDFSIYLDISNEVKFAWKIQRDMAERGHSLESIKASIEARKPDFDAFID
PQKQYADAVIEVLPTQLIPDDNEGKVLRVKLIMKEGIKFFNPVYLFDEGSTINWIPCGRK
LTCSYPGIKFSYGPDTYFGQEVSVLEMDGQFDRLDELIYVESHLSNLSTKFYGEVTQQML
KHADFPGSNNGTGLFQTIVGLKIRDLYEQIIAERAGVPAEAAKV
>Q9XJ27
MASITNLASSLSSLSFSSQVSQRPNTISFPRANSVFALPAKSARRASLSITATVSAPPEE
EEIVELKKYVKSRLPGGFAAQKIIGTGRRKCAIARVVLQEGTGKVIINYRDAKEYLQGNP

LWLQYVKVPLVTLGYENSYDIFVKAHGGGLSGQAQAITLGVARALLKVSADHRSPLKKEG
LLTRDARVVERKKAGLKKARKAPQFSKR
>Q9LS01
MASSSAAMSLESISMTTLNNLSRNHQSHRSSLLGFSRSFQNLGISSNGPDFSSRSRSTTS
KNLNVTRAFFWNWGKKTENSRPSKIQELNVYELNEGDRNSPAVLKLGKKPTELCLGDLVP
FTNKLYTGDLKKRVGITAGLCVLIQHVPEKSGDRFEASYSFYFGDYGHLSVQGQYLTYED
TFLAVTGGSGIFEGAYGQVKLRQLVYPTKLFYTFYLKGLANDLPLELTGTAVTPSKDVKP
APEAKAMEPSGVISNFTN
>P13564
MQVRRDDDGAGGCAGDAVPGGGEGQDGVPARQPAGRVWGVSRAARATSGFKVLALGPETT
GVIQRMQQLLDMDTTPFTDKIIAEYIWVGGSGIDLRSKSRTISKPVEDPSELPKWNYDGS
STGQAPGEDSEVILYPQAIFKDPFRGGNNILVICDTYTPQGEPIPTNKRHMAAQIFSDPK
VTSQVPWFGIEQEYTLMQRDVNWPLGWPVGGYPGPQGPYYCAVGSDKSFGRDISDAHYKA
CLYAGIEISGTNGEVMPGQWEYQVGPSVGIDAGDHIWASRYILERITEQAGVVLTLDPKP
IQGDWNGAGCHTNYSTLSMREDGGFDVIKKAILNLSLRHDLHIAAYGEGNERRLTGLHET
ASISDFSWGVANRGCSIRVGRDTEAKGKGYLEDRRPASNMDPYTVTALLAETTILWEPTL
EAEALAAKKLALKV
>P46489
MAVVKLSPWANYSSSKSEIKSSSSSSSSKSSLSAYVINVSSSPRLSFYNPYPRRLHHQRL
SSPASIRCSVTSSDQIQAPLPAKQKPECFGVFCLTYDLKAEEETKSWKKIINVAVSGAAG
MISNHLLFKLASGEVFGPDQPISLKLLGSERSFAALEGVAMELEDSLYPLLRQVSIGIDP
YEIFQDAEWALLIGAKPRGPGMERADLLDINGQIFAEQGKALNAVASPNVKVMVVGNPCN
TNALICLKNAPNIPPKNFHALTRLDENRAKCQLALKAGVFYDKVSNVTIWGNHSTTQVPD
FLNAKIHGIPVTEVIRDRKWLEDEFTNMVQTRGGVLIKKWGRSSAASTAVSIVDAIRSLV
TPTPEGDWFSTGVYTNGNPYGIAEDIVFSMPCRSKGDGDYEFVKDVIFDDYLSKKIKKSE
DELLAEKKCVAHLTGEGIAVCDLPEDTMLPGEM
>Q9FYA6
MERSAVASGFHRNYILCASRAATSTTRLHSLSSLRNFPSSSLRIRHCPSPISSNFIVSEV
SRNRRCDAVSSSTTDVTELAEIDWDKIDFGLKPTDYMYAMKCSRDGEFSQGQLQPFGNID
INPAAGVLNYGQGLFEGLKAYRKQDGNILLFRPEENAIRMRNGAERMCMPSPTVEQFVEA
VKTTVLANKRWIPPPGKGSLYIRPLLMGTGAVLGLAPAPEYTFLIFVSPVGNYFKEGVAP
INLIVETEFHRATPGGTGGVKTIGNYAAVLKAQSIAKAKGYSDVLYLDCLHKRYLEEVSS
CNIFIVKDNVISTPEIKGTILPGITRKSIIEVARSQGFKVEERNVTVDELVEADEVFCTG
TAVVLSPVGSITYKSQRFSYGEDGFGTVSKQLYTSLTSLQMGLSEDNMNWTVQLS
>P08889
MPPCSSKSILSTKSSMFILVSFALLRFIFYFVEFYRLVIGMNNLEIKRMNQKIVRNSERW
EDPFDRIISFHYIDNFKKLFIL
>O22886
MSILQVSTSSLSSSTLLSISPRKSLSSTKSCRIVRCSVEGTTVTERKVSATSEPLLLRAV
KGEVVDRPPVWLMRQAGRYMKSYQTLCEKYPSFRDRSENADLVVEISLQPWKVFKPDGVI
LFSDILTPLSGMNIPFDIVKGKGPIIFNPPQSAADVAQVREFVPEESVPYVGEALRRLRN
EVNNEAAVLGFVGAPFTLSSYVIEGGSSKNFTQIKRLAFSQPKVLHALLQKFTTSMITYI
RYQADSGAQAVQIFDSWATELSPVDFEEFSLPYLKQIVEAVKQTHPNLPLILYASGSGGL
LERLARTGVDVVSLDWTVDMAEGRDRLGRDIAVQGNVDPGVLFGSKEFITSRIHDTVKKA

GRDKHILNLGHGIKVGTPEENVAHFFEVAQEIRY
>P24493
MASTFNIPCNAGTIKNFNNSQRNLGFSSNLGINFAKTRFSNCGDSGRIPSQLVVRASERR
DNLTQQKTGLSIEECEAAVVAGNAPSAPPVPPTPKAPSGTPSVSPLSLGRRPRRNRTSPV
FRAAFQETTLSPANVVYPLFIHEGEEDTPIGAMPGCYRLGWRHGLVEEVAKARDVVVNSI
VVFPKPDALKSPTGDEAYNENGLVPRTIRMLKDKFPDLIIYTDVALDPYYYDGHDGIVTQ
HGVIMNDETVHQLCKQAVAQARAGADVVSPSDMMDGRVGAIRAALDAEGYSNVSIMSYTA
KYASSFYGPFREALDSNPRFGDKKTYQMNPANYREALIETQEDESEGADILLVKPGLPYL
DIIRLLRDNSDLPIAAYQVSGEYSMIKAGGVLKMIDEEKVMLESLLCLRRAGADIILTYF
ALQAARCLCGEKR
>Q9MUR1
MAQLENNSTLLVRENELKGLVSNWLIEMKLMHRPLGFDYQGVETLEVKAQNLTSVAIALY
AYGFNYLRSQCAYDVSPGGDLASVYHLTKVDDNADQPQEVCIKVFVPRTKPIIPSVFWIW
KTADFQERESYDMFGIYYEGHPHLKRILMPEHWIGWPLRKDYITPDFYELQDAY
>Q9MUR2
MSPIIINSVRLISSLLTIVFIMLPKVDTFSKIMKNKKILLNWQENISTLSIIKWISIILF
LSSHCALFITL
>Q02073
MAATHLTSTSSLTINTLPSFEGLRSASGISKINVSVAYPSFTSRSFRGLVVRAASITTSK
YTSVKPLGDRVLIKTKIVEEKTTSGIFLPTAAQKKPQSGEVVAIGSGKKVGDKKLPVAVK
TGAEVVYSKYTGTEIEVDGSSHLIVKEDDIIGILETDDVKDLKPLNDRLLIKVAEVENKT
SGGLLLAESSKEKPSFGTVVATGPGVLDEEGNRIPLPVCSGNTVLYSKYAGNDFKGVDGS
DYMVLRVSDVMAVLS
>P36437
MKLAYWMYAGPAHIGVLRVSSSFKNVHAIMHAPLGDDYFNVMRSMLERERDFTPVTASIV
DRHVLARGSQEKVVENITRKNKEETPDLILLTPTCTSSILQEDLHNFVESALAKPVQIDE
HADHKVTQQSALSSVSPLLPLEENTLIVSELDKKLSPSSKLHINMPNICIPEGEGEGEQT
KNSIFVKSATLTNLSEEELLNQEHHTKTRNHSDVILADVNHYRVNELQAADRTLEQIVRY
YISQAQKQNCLNITKTAKPSVNIIGIFTLGFHNQHDCRELKRLFNDLGIQINEIIPEGGN
VHNLKKLPQAWFNFVPYREIGLMTAMYLKSEFNMPYVAITPMGLIDTAACIRSICKIITT
QLLNQTATVQEPSKFIYPKATSLEQTNILETSQKETILKDNPDSGNTLSTTVEEIETLFN
KYIDQQTRFVSQAAWFSRSIDCQNLTGKKAVVFGDATHSAAMTKLLAREMGIKVSCAGTY
CKHDADWFREQVSGFCDQVLITDDHTQVGDMIAQLEPAAIFGTQMERHVGKRLDIPCGVI
SAPVHIQNFPLGYRPFLGYEGTNQIADLVYNSFNLGMEDHLLQIFGGHDSENNSSIATHL
NTNNAINLAPGYLPEGEGSSRTSNVVSTISSEKKAIVWSPEGLAELNKVPGFVRGKVKRN
TEKYALQKNCSMITVEVMYAAKEALSA
>Q94FY8
MNLAVAAALPSVTPRTGVVLPRSSRRHCPRGVVPRAASSSVSSFTSPSAAAAPIYTPTPQ
DRSLRTPHSGYHFDGTARPFFEGWYFKVSIPECRQSFCFMYSVENPLFRDGMSDLDKLLY
RPRFTGVGAQILGADDKYICQFSEKSNNFWGSRHELMLGNTFISNKESTPPQGEVPPQDF
SRRVLEGLQVTPIWHQGFIRDDGRSNYVPNVQTARWEYSTRPVYGWGDVKSKQLSTAGWL
AAFPFFEPHWQICMARGLSTGWIEWDGERFEFENAPSYSEKNWGGGFPRKWYWIQCNVFP
GASGEVSLTAAGGLRKIGLGDTYESPSLIGIHYEGQFFEFVPWTGTVSWDIGLWGLWKMS
GENKTHLVEIEATTAESGTALRAPTIEAGLVPACKDTCYGDLRLQLWEKKYDGSKGEMIL

DATSNMAALEVGGGPWFNGWKGTTVVNEVVNNIVGTPVDVESLLPIPFLKPPGL

>Q9MV14

MAAMVDAKPAASVQGTPLLATATLPVFTRGIYSTKRITLETSSPSSPPPPKPLIIVTPAG
KGTFNVILFLHGTSLSNKSYSKIFDHIASHGFIVVAPQLYTSIPPPSATNELNSAAEVAE
WLPQGLQQNLPENTEANVSLVAVMGHSRGGQTAFALSLRYGFGAVIGLDPVAGTSKTTGL
DPSILSFDSFDFSIPVTVIGTGLGGVARCITACAPEGANHEEFFNRCKNSSRAHFVATDY
GHMDILDDNPSDVKSWALSKYFCKNGNESRDPMRRCVSGIVVAFLKDFFYGDAEDFRQIL
KDPSFAPIKLDSVEYIDASSMLTTTHVKV

>P25873

MATPLSISSNPLTSRHCYRLHLSSTSFKGNVSVLGANPSQILSLKLNQTLKTRNQQQFAR
PLVVVSQTAATSSAVVAPERFRLDNLGPQPGSRKKQKRKGRGISAGQGASCGFGMRGQKS
RSGPGIMRGFEGGQTALYRRLPKLRGIAGGMRSGLPKYLPVNIKDIETAGFQEGDEVSLE
TLKQKGLINPSGRERKLPLKILGTGELSMKLTFKARAFSTQAKEKLEASGCTLTVLPGRK
KWVKPSVAKNQARADEYFAKKRAAAAEAATSEPAASA

>P23408

MALSLAINVPPVRDNLLRPQPFQSQFRPNLLKFPIPSSARIRCSSSSSFNDISLKTVTPD
NKNSFVCRFNATQLEVQETNQPYAETYAVGRHIRMSADKARRVVDQIRGRPYEASLMVLE
LMPYRACEAIIKIVFSAGANASHNLGLSKSSLFISKAEVNEGKTLKRVRARAQGRANQIL
KRTCHITITVRGLPDESDKEENSS

>O48593

MAATFLPATSLRLTQNSTLRFLSFFTISNPSYSLFRPLRRRVLPPFDAFPANSRRRCFCT
AVSESLGSGDGNKVESYEKRFGSKVGEFRKKLRIAEVKGGADEGLSRVGQSLNIMGWVRT
LRSQSSVTFIEINDGSCLSNLQCVMTSDAEGYDQVESGSILTGASVSVQGTIVASQGTKQ
KVELKVEKIIVVGECDSSYPIQKKRVSREFLRTKAHLRPRTNTFGAVARVRNTLAYATHK
FFQESGFVWVASPIITASDCEGAGEQFCVTTLIPSSHENTDTSIDAIPKTKGGLIDWSQD
FFGKPAFLTVSGQLNGETYATALSDVYTFGPTFRAENSNTSRHLAEFWMIEPELAFADLD
DDMACATAYLQYVVKYVLDNCKEDMEFFDTWIEKGIIRRLSDVAEKEFLQLGYTDAIEIL
LKANKKFDFPVKWGLDLQSEHERYITEEAFGGRPVIIRDYPKEIKAFYMRENDDGKTVAA
MDMLVPRIGELIGGSQREERLEVLEARLDELKLNKESYWWYLDLRRYGSVPHAGFGLGFE
RLVQFVTGIDNIRDVIPFPRTPASAEF

>Q9FN52

MASLLLTSSSMITTSCRSMVLRSGLPIGSSFPSLRLTRPYDKATLFVSCCSAESKKVATS
ATDLKPIMERRPEYIPNKLPHKNYVRVLDTTLRDGEQSPGAALTPPQKLEIARQLAKLRV
DIMEVGFPVSSEEEFEAIKTIAKTVGNEVDEETGYVPVICGIARCKKRDIEATWEALKYA
KRPRVMLFTSTSEIHMKYKLKKTKEEVIEMAVNSVKYAKSLGFKDIQFGCEDGGRTEKDF
ICKILGESIKAGATTVGFADTVGINMPQEFGELVAYVIENTPGADDIVFAIHCHNDLGVA
TANTISGICAGARQVEVTINGIGERSGNAPLEEVVMALKCRGESLMDGVYTKIDSRQIMA
TSKMVQEHTGMYVQPHKPIVGDNCFVHESGIHQDGILKNRSTYEILSPEDVGIVKSENSG
IVLGKLSGRHAVKDRLKELGYEISDEKFNDIFSRYRELTKDKKRITDADLKALVVNGAEI
SSEKLNSKGINDLMSSPQISAVV

>P36443

MTLALMQSMGKLKFLHCLLPLPFRDVPFPLRASKLRDFNAINKFVPLRDVSGSCEVLILY
INIECLHTPKEDVSGSGTATAILILRRSSGGPTATKIYLPEDVLPTAEANEF

>Q9SE20

MATSSAYLSCPATSATGKKHVFPNGSPGFLVFGGTRLSNRLVTRKSVIRADLDSMVSDMS
TNAPKGLFPPEPEHYRGPKLKVAIIGAGLAGMSTAVELLDQGHEVDIYESRTFIGGKVGS
FVDRRGNHIEMGLHVFFGCYNNLFRLLKKVGAEKNLLVKEHTHTFVNKGGEIGELDFRFP
VGAPLHGINAFLSTNQLKIYDKARNAVALALSPVVRALVDPDGALQQIRDLDNVSFSEWF
LSKGGTRASIQRMWDPVAYALGFIDCDNMSARCMLTIFALFATKTEASLLRMLKGSPDVY
LSGPIKKYIMDKGGRFHLRWGCREVLYETSSDGSMYVSGLAMSKATQKKIVKADAYVAAC
DVPGIKRLVPQKWRELEFFDNIYKLVGVPVVTVQLRYNGWVTELQDLERSRQLKRAAGLD
NLLYTPDADFSCFADLALASPDDYYIEGQGSLLQCVLTPGDPYMPLSNDEIIKRVTKQVL
ALFPSSQGLEVTWSSVLKIGQSLYREGPGKDPFRPDQKTPVENFFLAGSYTKQDYIDSME
GATLSGRQASAYICNVGEQLMALRKKITAAELNDISKGVSLSDELSLV
>P12347
MCLPFFTSINSSGLFKAVSWRIIESFLLELFFIFYSSFSWSSSLSSSEGTGAGTGTGTGT
GTGTGTGTGTGTGTGTGTGTGTGTGTGTGTGTGTGTGTGTGTGTGTGTGTGTGTGTGTGT
GTGTGTGTGTGTGTGTGTIWEGELVFFYAECKGYPKIKGVFWVVSFFYDTAKNV
>P37825
MYLGISGRSPYGSSVAVACQLPNINISGQIYLLLPPSEYINIGFNTRQWQYHCHWRPKFT
CPKGKEAVAVPLPLKFICPKGTSCQLPIFIYSEVYLYASEY
>Q8WHX7
MKFANIFALEHLLTLGAYLFCIGFYGLITSQNMIKALMCLELVLNAVNINFVTFSNYFDT
QHIKGEIFVLFIMAIAAAEAAIGLAIVLTIYRDRKSIRIDQFNLLK
>Q9SMH4
MRTRAGAFFGKQRSTSPSGSSTSASRQWLRSSPGRTQRPAAHRVLAVCVRNPARPAADHP
LVSTTQAAAQRQARQWDSRSKPPQLQPQLRHTHVSRQAQRRQQQQVQQQAEAGALVVPTV
ITSFPEHLRPEVLANGVLLVDKPPHWEVPEVVAAVQRATGADKVASVAPLDARASGLMLL
CFGSATRLAPRVERAAKRYTGTLVLGGSSLSGDVRGGSFRAAQLPAEHLTDEDLREAAQG
LVTAAAGAPVHGAVATGHQGGGGLALRVLPRTWRLRQLPSSTEYYEERVEPSMRTLDMEL
LDFRVWRESALSHNDGAAGAEYDQHDQHKQELGRGLVWTRSPPHPRPVVLRFSALLVGRS
HVRSLIAMYGRRLRTAACLDDLRRTEIGSFNVEEAWPLEALVPVLQRHAH
>P80030
MAATAAASSLQMATTRPSISAASSKARTYVVGANPRNAYKIACTPHLSNLGCLRNDSALP
ASKKSFSFSTKAMSESSESKASSGLPIDLRGKRAFIAGIADDNGYGWAVAKSLAAAGAEI
LVGTWVPALNIFETSLRRGKFDQSRVLPDGSLMEIKKVYPLDAVFDNPEDVPEDVKANKR
YAGSSNWTVQEAAECVRQDFGSIDILVHSLANGPEVSKPLLETSRKGYLAAISASSYSFV
SLLSHFLPIMNPGGASISLTYIASERIIPGYGGGMSSAKAALESDTRVLAFEAGRKQNIR
VNTISAGPLGSRAAKAIGFIDTMIEYSYNNAPIQKTLTADEVGNAAAFLVSPLASAITGA
TIYVDNGLNSMGVALDSPVFKDLNK
>Q9SWI6
MATMSLAAASPLASIPRGIAAQAPCAAFLSIRLGGATATRFAGLAVASQPAERRAAAMVA
MAKREQELEEIRAMTTEQMEEEVVDLKGELFLLRLKRSARQEFKNSEFSRMRKRIARMLT
VKREREIEQGINKRLSRKLDRKWKQSIVVRPPPSLRGNKEE
>O82499
MLQLCSTFRPQLLLPCQFRFTNGVLIPQINYVASNSVVNIRPMIRCQRASGGRGGANRSK
PAKPQVKEGSNKTVIEGLVTESLPNGMFRVDLENGDNILGYICGKIRKNFIRILPGDKVK
VEMSVYDSTKGRIIFRMSSRD

>P53797

MVVAILRVVSAIEIPIRLGFSEANWRFSSPKYDNLGRKKSRLSVYSLYTTSKYACVGFEA
ENNGKFLIRSSLVANPAGEATISSEQKVYDVVLKQAALVKDQTKSSRKSTDVKPDIVLPG
TVYLLKDAYDRCGEVCAEYAKTFYLGTLLMTPERRRAIWAIYVWCRRTDELVDGHNASHI
TPSALDRWEARLEDLFAGRPYDMFDAALSDTVSRFPVDIQPFMDMVEGMRMDLKKSRYKN
FDELYLYCYYVAGTVGLMSVPVMGIAPESLAEAESVYNAALALGIANQLTNILRDVGEDA
RRGRIYLPQDELAEAGLSDEDVFTGKVTDKWRSFMKRQIKRARTFFEQAEKGVTELSQAS
RWPVWASLLLYRQILDEIEANDYNNFTKRAYVSKVKRLAALPLAYGKSLLIPLSLRPPSL
SKA

>Q43316

MDIASSSLSQAHKVVLTRQPSSRVNTCSLGSVSAIGFSLPQISSPALGKCRRKQSSSGFV
KACVAVEQKTRTAIIRIGTRGSPLALAQAYETREKLKKKHPELVEDGAIHIEIIKTTGDK
ILSQPLADIGGKGLFTKEIDEALINGHIDIAVHSMKDVPTYLPEKTILPCNLPREDVRDA
FICLTAATLAELPAGSVVGTASLRRKSQILHKYPALHVEENFRGNVQTRLSKLQGGKVQA
TLLALAGLKRLSMTENVASILSLDEMLPAVAQGAIGIACRTDDDKMATYLASLNHEETRL
AISCERAFLETLDGSCRTPIAGYASKDEEGNCIFRGLVASPDGTKVLETSRKGPYVYEDM
VKMGKDAGQELLSRAGPGFFGN

>Q1ACG0

MLSPRKTKFRKHHRGRMKGISTRGNSIVFGKFALQALEPSWITSRQIEAGRRSMTRYARR
GGKIWIRIFPDKPVTMRPAETRMGSGKGSPEYWVAVVKPGRILYEMDGVPENIARAAMKI
AAYKMPNKTQFLIRDNKDLIDSKI

>P12859

MATHAALASTRIPTNTRFPSKTSHSFPSQCASKRLEVGEFSGLKSTSCISYVHSARDSSF
YDVVAAQLTSKANGSTAVKGVTVAKLKVAINGFGRIGRNFLRCWHGRKDSPLEVIVVNDS
GGVKNASHLLKYDSMLGTFKAEVKILNNETITVDGKPIKVVSSRDPLKLPWAELGIDIVI
EGTGVFVDGPGAGKHIQAGAKKVIITAPAKGADIPTYVIGVNEQDYGHEVADIISNASCT
TNCLAPFAKVLDEEFGIVKGTMTTTHSYTGDQRLLDASHRDLRRARAAALNIVPTSTGAA
KAVSLVLPQLKGKLNGIALRVPTPNVSVVDLVVNVAKKGISAEDVNAAFRKAAEGPLKGI
LDVCDVPLVSVDFRCSDVSTTIDSSLTMVMGDDMVKVVAWYDNEWGYSQRVVDLAHLVAN
KWPGTPKVGSGDPLEDFCETNPADEECKVYE

>Q94IC4

MALSCSKVLSFYLSPVVGGGDVPKKLTFSSFLGLSKGVGGSRSSRVCAASNAPAPLAGVI
FEPFQELKKDYLAVPIAHNVXLARQNYADDSESAINEQINVEYNVSYVYHALFAYFDRDN
IALKGLAKFFKESSEEEREHAEQLIKYQNIRGGRVVLHPITSPPSEFEHSEKGDALYAME
LALSLEKLTNEKLLHVHSVAERNNDPQXADFIESEFLYEQVKSIKKIAEYVAQLRLVGKG
HGVWHFDQKLLHDEDHV

>P08474

MASSILSSAAVASVNSASPAQASMVAPFTGLKSSAGFPITRKNNVDITTLASNAGKVQCM
KVWPPLGLRKFETLSYLPDMSNEQLSKECDYLLRNGWVPCVEFDIGSGFVYRENHRSPGF
YDGRYWTMWKLPMFGCTDSSQVIQEIEEAKKEYPDAFIRVIGFDNVRQVQCISFIAYKPQ
VLLFLSSIC

>Q01292

MAATAATTFSLSSSSSTSAAASKALKQSPKPSALNLGFLGSSSTIKACRSLKAARVLPSG
ANGGGSALSAQMVSAPSINTPSATTFDFDSSVFKKEKVTLSGHDEYIVRGGRNLFPLLPD

AFKGIKQIGVIGWGSQAPAQAQNLKDSLTEAKSDVVVKIGLRKGSNSFAEARAAGFSEEN
GTLGDMWETISGSDLVLLLISDSAQADNYEKVFSHMKPNSILGLSHGFLLGHLQSLGQDF
PKNISVIAVCPKGMGPSVRRLYVQGKEVNGAGINSSFAVHQDVDGRATDVALGWSIALGS
PFTFATTLEQEYKSDIFGERGILLGAVHGIVECLFRRYTESGMSEDLAYKNTVECITGVI
SKTISTKGMLALYNSLSEEGKKDFQAAYSASYYPSMDILYECYEDVASGSEIRSVVLAGR
RFYEKEGLPAFPMGKIDQTRMWKVGEKVRSVRPAGDLGPLYPFTAGVYVALMMAQIEILR
KKGHSYSEIINESVIEAVDSLNPFMHARGVSFMVDNCSTTARLGSRKWAPRFDYILSQQA
LVAVDNGAPINQDLISNFLSDPVHEAIGVCAQLRPSVDISVTADADFVRPELRQA
>Q9MUT3
MSTIDLIKNVTKREERPVYPFTAIVGQEEMKLALILNVIDPDIGGVMIMGDRGTGKSTTI
RALVDLLPEIEVVTNDPFNSDPRDPDLMSDEVREKINNKQEVPTIKTKIKIVDLPLGATE
DRVCGTIDIERALNEGVKAFEPGLLAKANRGILYVDEVNLLDDHLVDILLDSAASGWNTV
EREGISVRHPAKFILVGSGNPEEGELRPQLLDRFGMHAEIRTVKDPDLRVKIVEERSSFD
ENPQVFRKAYEQSQEDVKSQIIQARKNLANVQMDRELRIKVSQICSELDVDGLRGDLVIN
RAAKALAAFEGRDKVLPKDILKIITLCLRHRLRKDPLESIDSGSKVESKFYEVFGLLEEN

>Q85FP0
MKVNYGSPAKSGPLHKNGEFYINKNCFLHPLLFLSEENFYLTDGKRRSHGADANLVFGAW
STVAVKRLIGSVRDPNLNFLKIYDSVSVRNLTDGLDVDLYLHPLLKMTYLILGIALFPKI
RAETSSKSKMLQSIHSMFLFLEDRFSKSNHILEADLPHNLHLETLIRLFRRQIKDVSFLH
LLRIVFRKRKIFCGKTFYSPGGGQDGSVDIPVRNFYIFEIDSLLLIPWKQVYKFRVNYLS
PIDSCNIIRKEIYASAYKFKWNKASIDYSFSRSLWIHYGRWRNKFLIASEGTHYFVKKML
YYLWILLKYHFHYRIKSNEPWIRKLLPTSCVSFLGYTLLAQLVSKNVRIETVTDLYISIL
GGKKFYPKIPNSIIITTLAKQRFCDFTGRPIGKSAWVTSTDDKIIDGYVQLWQVFSLYYG
ASMNQYRLRRLIFLLQMSCDSTLAGKHRSTIRLLRCKSNVEALNQILASRKFELSSSRRV
WRSSSIRSVLVQFTVLDIGL
>Q93YW0
MPSLSTPPSQNLAFSPAASATSSRLTPSSKRSFYPHRLPDPTALCRCSSSSGSNSSSSSS
SDDNPRWDSAIQDVLKSAIKRFDSVLSWYATLDNDDGEQGSENVEKIDDDWDWDRWKKHF
DQVDDQDRLLSVLKSQLNRAIKREDYEDAARLKVAIAATATNDAVGKVMSTFYRALLEER
YKDAVYLRDKAGAGLVGWWSGISEDVKDPFGLIVQITAEHGRYVARSYNPRQLSTSAAGA
PLFEIFLTLDGKGNYKKQAVYLKWKEIFPDVPTMPSRTLTPGRFLTSPGRKEDTGNLAVE
SSEDEESDNSDDDSDLLEESSGFQSFLRDMIPGVKVKVMKVTAPGRVDKDFISKVIEQIA
DEEDEENDLDIEDIDVEDDTKAEIDEKNADIELESVTDEIIDNNGGREIAVKFVIGDIVD
RLSGNQPLKESLRSPANLESVENSSFYLRLEKDLNVKESKGVEGTTLVDGKGSRQSRRRI
ENIMGDLAKSIEKEKKISVKMLKDVGELLSLTLSQAQNRQQLSGLTKFRRIDVTPSLDPL
DGLYIGAHGLYTSEVIHLKRKFGQWKGGKESKKPTDIEFYEYVEAVKLTGDPYVPAGKVA
FRAKIGRRYELPHKGLIPEEFGVIARYKGQGRLADPGFRNPRWVDGELVILDGKYVKGGP
VVGFVYWAPEYHFVMFFNRLRLQA
>P24226
MSFDLSRLSLTSSPRLSFLTRTATKKGFVRCSMKSYRLSELSFSQVENLKARPRIDFSSI
FTTVNPIIDAVRSKGDTAVKEYTERFDKVQLNKVVEDVSELDIPELDSAVKEAFDVAYDN
IYAFHFAQMSTEKSVENMKGVRCKRVSRSIGSVGLYVPGGTAVLPSTALMLAIPAQIAGC
KTVVLATPPTKEGSICKEVLYCAKRAGVTHILKAGGAQAIAAMAWGTDSCPKVEKIFGPG

NQYVTAAKMILQNSEAMVSIDMPAGPSEVLVIADEHASPVYIAADLLSQAEHGPDSQVVL
VVVGDGVNLKAIEEEIAKQCKSLPRGEFASKALSHSFTVFARDMIEAITFSNLYAPEHLI
INVKDAEKWEGLIENAGSVFIGPWTPESVGDYASGTNHVLPTYGYARMYSGVSLDSFLKF
MTVQSLTEEGLRNLGPYVATMAEIEGLDAHKRAVTLRLKDIEAKQTQTK
>Q00497
MEARVSQSLQLSSWINSDKVVRKPSGLLRFSEKWNEKPRHRVVVSCHLQPRKAAHSDRRV
QLKVSCSPQNVQASVLESGCFSASIDEIETLKNKAEEVEEYLDGRCVYLVGMMGCGKTTV
GRILAETLGYSFFDCDRLIEQAVGGITVAEIFELRGESFFRDNETEVLHKLSLMHRLVVS
TGGGAVVRPINWRHMHKGISVWLDVPLEALAKRITTEGTKSRPLLHEESGDVYDTTLKRL
TTLMETRGENYANASARVSLENIALKREKDVCHITPAEITLEVLIQIENFLKTQKSVVVL

>P09856
MALHLSLSHQSWTSPAHPITSSDPTRSSVPGTGLSRRVDFLGSCKINGVFVVKRKDRRRM
RGGEVRASMEQALGTQEMEAIVGKVTEVNKDTFWPIVKAAGDKPVVLDMFTQWCGPCKAM
APKYEKLAEEYLDVIFLKLDCNQENKTLAKELGIRVVPTFKILKENSVVGEVTGAKYDKL
LEAIQAARSS
>Q39513
MVATAASSAFFPLPSADTSSRPGKLGNKPSSLSPLKPKSTPNGGLQVKANASAPPKINGS
PVGLKSGGLKTQEDAHSAPPPRTFINQLPDWSMLLAAITTVFLAAEKQWMMLDWKPKRPD
MLVDPFGLGSIVQDGLVFRQNFSIRSYEIGADRTASIETVMNHLQETALNHVKIAGLSND
GFGRTPEMYKRDLIWVVAKMQVMVNRYPTWGDTVEVNTWVAKSGKNGMRRDWLISDCNTG
EILTRASSVWVMMNQKTRRLSKIPDEVRNEIEPHFVDSPPVIEDDDRKLPKLDEKTADSI
RKGLTPRWNDLDVNQHVNNVKYIGWILESTPPEVLETQELCSLTLEYRRECGRESVLESL
TAMDPSGGGYGSQFQHLLRLEDGGEIVKGRTEWRPKNGVINGVVPTGESSPGDYS
>Q8LPT9
MSNSIGRNVLHQSLLCSTVFEHQSNRHSSGIPANSLFQAVSINQPAGASAARKSPLSTKF
YGTSLNARPKMAMGRHRPVLITPRAVLAVDSASELAGKFNLEGNVELQITVGAPTPGSLT
QVNIEISYSSNSLLLHWGAIRDKKEKWVLPSRPPDGTKILKNRALRTPFVSSGSKSLVKL
EIDDPAIEAVEFLILDEAQNKWFKNNGANFHVKLPSERSLIQNVSVPEDLVQTQAYLRWE
RKGKQIYTPEQEKEEYEAARTELLEEIVRGTSVEDLRAKLTNKNDRQEIKESSSHGTKNA
IPDDLVQIQSYIRWERAGKPNYSADQQLREFEEARKELQSELEKGISLDEIWKKITKGEI
QTKVSDQLKTKKYFRTERIQRKQRDFMQILNKHVAEPTEKKNISVEPKALTPVELFVGAT
EEQEGDSILNKKIYKLAGKELLVLVHKPGGKTKIHLATDGKEPLILHWALSKKAGEWLAP
PPSVLPAGSVLLSGSVETTFTTSSLADLPYQVQSIEIEIEEEGYVGMPSVLQSGGNWIKN
KGSDFYVDFSYESKQVQQDFGDGKGTAKALLEKIAGLEIEAQKSFMHRFNIAADLIQEAK
EAGELGFAGILVWMRFMATRQLIWNKNYNVKPREISKAQDRLTDLLQNVYISNPEYREIV
RMILSTVGRGGEGDVGQRIRDEILVIQRNNNCKGGMMEEWHQKLHNNTSPDDVIICQALI
DYIKSDFDISAYWKTLNDNGITKERLLSYDRAIHSEPNFRRDQKDGLLRDLGNYMRTLKA
VHSGADLESAITNCLGYRSEGQGFMVGVQINPIPNLPSGFPELLQFVSEHVEDRNVEALL
EGLLEARQEIRPLLCKHNDRLKDLLFLDIALESSVRTAIEKGYEELNEAGPEKIMYFVSL
ILENLALSLDDNEDLIYCLKGWSNALSMSKSKSDNWALFAKSVLDRTRLALAGKADWYQK
VLQPSAEYLGTLLSVDKWAVDIFTEEMIRAGSAAALSLLLNRLDPVLRKTASLGSWQVIS
PVEVFGYVAVVDELLAVQDKSYDQPTILLARRVKGEEEIPHGTVAVLTADMPDVLSHVSV
RARNCKVCFATCFDPNILADLQSNEGKMLHLKPTSADIAYSVVEGSELQDSSSANLKEED

GPSSSVALVKKQFAGRYAITSDEFTGELVGAKSRNIAYLKGKVPSWIGIPTSVALPFGVF
EKVLSDDINQAVAEKLQILKQKLGEEDHSALREIRETVLQMKAPNQLVQELKTEMKSSGM
PWPGDEGEQRWEQAWMAIKKVWASKWNERAFFSTRRVKLDHEYLCMAVLVQEIINADYAF
VIHTTNPSSGDSSEIYAEVVKGLGETLVGAYPGRALSFVCKKNDLKSPRVLGYPSKPIGL
FIRRSIIFRSDSNGEDLEGYAGAGLYDSVPMDEAEKVVLDYSSDHLITDGHFQQSILSSI
ARAGCEIEELFGSAQDIEGVVRDGKIYVVQTRPQM

>Q05728

MEARILQSSSSCYSSLYAVNRSRFSSVSSPKPFSVSFAQTTRTRTRVLSMSKKDGRTDKD
DDTDSLNYKDSGVDIDAGAELVKRIAKMAPGIGGFGGLFPLGDSYLVAGTDGVGTKLKLA
FETGIHDTIGIDLVAMSVNDIITSGAKPLFFLDYFATSRLDVDLAEKVIKGIVEGCRQSE
CALLGGETAEMPDFYAEGEYDLSGFAVGIVKKTSVINGKNIVAGDVLIGLPSSGVHSNGF
SLVRRVLARSNLSLKDALPGGSSTLGDALMAPTVIYVKQVLDMIEKGGVKGLAHITGGGF
TDNIPRVFPDGLGAVIHTDAWELPPLFKWIQQTGRIEDSEMRRTFNLGIGMVMVVSPEAA
SRILEEVKNGDYVAYRVGEVVNGEGVSYQ

>P42379

MPIGVPRIIYCWGEELPAQWTDIYNFIFRRRMVFLMQYLDDELCNQICGLLINIHMEDRS
KELEKKEIERSGLFKGGPKTQKGGTGAGETGASSIQNKKSNSSSFEDLLAADEDLGIDEN
NTLEQYTLQKITMEWLNWNAQFFDYSDEPYLFYLAEMLSKDFNKGDARMLFSNNNKFSMP
FSQMLNTGSMSDPRRPQSTNGANWNSSEQNNSLDIYSPFRMLANFEAQDYDFKQINPSLA
SKEEVFKLFNNTILKNGGQRNNNMSKLLTELAQRNWENKTNSQENLYKSTEKALSQRNLR
KEYIKDRTLNNYSSDPFNTKGYVNAQGASTGPSPRTRGMHADGSLNYLDFYSYNDSYNDF
KTAPRGKQAERAFQEEESKKVFVIINSFGGSVGNGITVHDALQFIKAGSLTLALGVAASA
ASLALAGGTIGERYVTEGCHVMIHQPECLTSDHTVLTTRGWIPIADVTLDDKVAVLDNNT
GEMSYQNPQKVHKYDYEGPMYEVKTAGVDLFVTPNHRMYVNTTNNTTNQNYNLVEASSIF
GKKVRYKNDAIWNKTDYQFILPETATLTGHTNKISSTPAIQPEMNAWLTFFGLWIANGHT
TKIAEKTAENNQQKQRYKVILTQVKEDVCDIIEQTLNKLGFNFIRSGKDYTIENKQLWSY
LNPFDNGALNKYLPDWVWELSSQQCKILLNSLCLGNCLFTKNDDTLHYFSTSERFANDVS
RLALHAGTTSTIQLEAAPSNLYDTIIGLPVEVNTTLWRVIINQSSFYSYSTDKSSALNLS
NNVACYVNAQSALTLEQNSQKINKNTLVLTKNNVKSQTMHSQRAERVDTALLTQKELDNS
LNHEILINKNPGTSQLECVVNPEVNNTSTNDRFVYYKGPVYCLTGPNNVFYVQRNGKAVW
TGNSSIQGQASDIWIDSQEIMKIRLDVAEIYSLATYRPRHKILRDLDRDFYLTATETIHY
GLADEIASNEVMQEIIEMTSKVWDYHDTKQQRLLESRDSTTSGADTQSQN

>Q9SHI1

MPSLASLVSLGGACASVSGTSSSDASYALVKRVSLSRRSVKGTKKWLCRYSVSSSTTTTT
ADFIADQNNNSVSIDSNSFRGSKDGDDSEVVLKQTPKPVLKPPVARVERGLGVNTAPWSK
DLSNGGKFDGEEERNKVIESLGEVLDKAEKLEIPKPGNKEGGEAVKPSQPSANSSNSRNG
SYANASDGGTRKTKTMKSVWRKGDAVAAVQKVVKESPKIFNRGVQTEPRTREEGEVNAKA
GTPLAPPQPPFRPQPPVRPQPMLQGKPMVAPPVKKSPILKDLGMAAKPLVSEEVDSSVKS
KERKPILVDKFASKKKGVDPAASQAVLAPTKPGKGPPSNKFRVEHRNKKNASASPRRRIV
AEDDGDDDASISRSGRKGRKWSKASRKAVRLQAAKDAAPVKAEILEVEEEGMSIEDLAYN
LAIGEGDILGYLYSKGIRPDGVHTLDREMVKMICRDYDVEVLDADSVKVEEMAKKRQTFD
EEDLDKLEDRPPVITIMGHVDHGKTTLLDYIRKSKVAASEAGGITQGIGAYKVSVPVDGK
LQSCVFLDTPGHEAFGAMRARGARVTDIAIIVVAADDGIRPQTNEAIAHAKAAAVPIVIA
INKIDKEGASPDRVMQELSSIGLMPEDWGGDVPMVQISALKGENVDDLLETVMLVAELQE

LKANPHRNAKGIVIEAGLDKAKGPFATFIVQKGTLKRGDVVVCGEAFGKVRALFDHSGER
VDEAGPSIPVQVIGLNNVPIAGDEFEIVSSLDVAREMAEARAVSLRDERISAKAGDGKVT
LSSLASAVSAKKMSGLDLHQLNIILKVDVQGSIEAVRQALQVLPQENVTLKFLLQATGDV
SNSDVDLASASEAIVFGFNVKASGSVKKAAENKGVEIRLYRVIYELIDDVRNAMEGLLES
VEEQIPIGSAEVRATFSSGSGRVAGCMVNEGKFVKDCGIRVVRKGKTVHVGVLDSLKRVK
ENVKEVSAGLECGIGMDDYDDWIEGDIIEAFNAVQKRRTLEEASASMSAAIEEAGV
>Q09FY3
MAIHLYKTSTPSTRNGAVDSQVKSNPRNNLIYGQHRCGKGRNARGIITAGHRGGGHKRLY
RKIDFRRNKKNIEGRIVTIEYDPNRNAYICLIHYGDGEKRYILHPRGAIIGDSIVSGTEV
SIKMGNALRLGNALPLTDMPLGTAIHNIEITLGKGGQLARAAGAVAKLIAKEGKSATLKL
PSGEVRLISKNCSATFGQVGNVGVNQKSLGRAGSKCWLGKRPVVRGVVMNPVDHPHGGGE
GRAPIGRKNPTTPWGYPALGRRSRKRNKYSDSLILRRRTK
>P0C452
MAVPKKRTSMSKKRIRKNLWKKKTYFSIVQSYSLAKSRSFSGVSEHPKPKGFSRQQTNNR
VLG
>Q52TG1
MEEFKKYFERDSYWQQHFLYPLLFQEYIYALAHIHNHGLNGSIFYESGEVLGYDNKSSLI
LVKRLILRMYQQNFLIHSSNESHQNGFVGHKNPFFSQMISGGFAVIVEIPFSLPSLIEKK
KKEITKSQNLRSIHSIFPFLVDQFSHLNYVSDILIPYPIHLEILIQIIQSWIQDVPSLHL
LRFFLYEYHNWNSFITRKKSLSTFGKENPRFFWFLYNSYVSEYESVFCFLRKHSSYLLST
SYRNLIERTHFYGKIEHLPVVCSNDFHKNLQLFKDPCMHYVRYQGKAILASKGTSLLMKK
WKWYLVNFWECNFSFWFQPYRIHINQLSNSSFLFWGYFSIVLINPLPVRSQMLGYSCLID
NTLTKKLETLVPIIPLIGSLSKAKFCNVSGHPASKPIWTDLSDSDIIDRFVRICRTLSHY
HSGSSKKQSLYRMKYILRLSCARTLARKHKTTVRAFFQRLGSGFLEEFFTEEQKFLSLVL
PRTSLASGSDRVYKERIWYLDIIRINDLVNYSRLAMELCTGNITKRIEKRSKIAFLFSTD
MLKCSCSGS
>P18823
MINEDPSSLTDMDNNIDSWKNNSENSSYSHADSLADVSNIDNLLSDKIFSIRDSNSNIYD
IYYAYDTNDTNITKYKWTNNINRCIESYLRSQICEDIDFNSDICDKVQRTIIILIRSTND
TNDISDTNDISDTNDTNDTNAIYDPFDISDTNDTNEIYDPFFILDINDTNDTNDIYGIYD
PDDIYETNIKDICERYSEIYPRNREKSTFVPIDYSDPNCMEKLARLWVQCETCYGLNFKQ
FFRPKMNICEHCGEHLKMSSSDRIDLSIDRDTWNPMDEDMVSVDPIKFDSIKELGSEEES
SKDRLDEDMLSPDPIELDSEEESSKDRVDSEEEKDQSYIDRLDSYQEKTGLPETVQTGTD
QREEIHPLFEDIMNQLDLYLQTAKNRVDSEEEKDQSYIDRLDSYQEKTGLPEAVQTGTGQ
LNGIPLALAVMDSEFIAGSMGCVVGEKITRLIEYATNLLLPLIIVCASGGARMQEGSLSL
MQMAKISSALYNYQINQKLFYVAILTSPTTGGVTASFGMLGDIIIAEPNATIAFAGKRVI
EQLLNKEVPEGSQSADLLFDRGLLDAVVPRHLLKEFLTELFQFHGFVPLT
>P56350
MVELNFTQKQKQFLKDRKSLKSLVNQHLASDLGETSLSQYLDEFQILYNRFFCFYFFSYG
ENVFCSEEIFSSQLKNGFFPTKTKDEILSSPALNVIFSEKDFLLLKNIRIFLGMDSNNFF
LTEELFN
>Q9SZ30
MEATAAPFSSIVSSRQNFSSSSSIRASSPASLFLSQKSIGNVNRKFKSPRSLSVRASSTS
DSVVTLLDYGAGNVRSIRNALRHLGFSIKDVQTPGDILNADRLIFPGVGAFAPAMDVLNR

TGMAEALCKYIENDRPFLGICLGLQLLFDSSEENGPVKGLGVIPGIVGRFDASAGIRVPH
IGWNALQVGKDSEILDDVGNRHVYFVHSYRAIPSDENKDWISSTCNYGESFISSIRRGNV
HAVQFHPEKSGEVGLSVLRRFLHPKLPATQKPMEGKASKLAKRVIACLDVRTNDKGDLVV
TKGDQYDVREQSNENEVRNLGKPVDLAGQYYKDGADEISFLNITGFRDFPLGDLPMIQVL
RQTSKNVFVPLTVGGGIRDFTDASGRYYSSLEVAAEYFRSGADKISIGSDAVSAAEEFIK
SGVKTGKSSLEQISRVYGNQAVVVSIDPRRVYVNHPDDVPYKVIRVTNPGPNGEEYAWYQ
CTVSGGREGRPIGAFELAKAVEELGAGEILLNCIDCDGQGKGFDIDLVKLISDSVGIPVI
ASSGAGTPDHFSEVFEKTNASAALAAGIFHRKEVPIQSVKEHLQEERIEVRI
>P94026
MASVFSVHPLPSSSFLCPLKTTKSRTKHHQTFYTYQKTILINSLQLTELDPKIPQPVQTF
WQWLCKEGVVTTKTPVKPGIVPEGLGLVAKRDIAKGETVLQVPKRFWINPDAVAESEIGN
VCSGLKPWISVALFLLREKWRDDSKWKYYMDVLPKSTDSTIYWSEEELSEIQGTQLLSTT
MSVKDYVQNEFQKVEEEVILRNKQLFPFPITLDDFFWAFGILRSRAFSRLRNQNLILVPF
ADLTNHNARVTTEDHAHEVRGPAGLFSWDLLFSLRSPLKLKAGDQLFIQYDLNKSNADMA
LDYGFIEPSSARDAFTLTLEISESDEFYGDKLDIAETNGIGETAYFDIKIGQSLPPTMIP
YLRLVALGGTDAFLLESIFRNSVWGHLGLPVSRANEELICKVVRDACKSALSGYHTTIEE
DEKLMEEGNLSTRLQIAVGIRLGEKRVLKQIDDIFRERELELDELEYYGERRLKDLGLVG
EQGDIIFWEPK
>Q32S00
MKLHKKKLNITMTKQFIQKFPSNTVGSGGKTLLSKFQENSGLTETQIYILTQRVARLSSH
LKNHNKDYSSQRGLRKLLGKRKRLLAYLSNEDVERYENLLIQLGIRGLKKI
>Q42961
MASATASHTLCGIPATSSSTTNKAIAPSSARFLAKTPLRRLGFAGAAADSLFTNHVATKL
RSLKSSSKPIRGVASMAKKSVGDLTAAELKGKKVFVRADLNVPLDDNQNITDDTRIRAAV
PTIKHLMANGAKVILSSHLGRPKGVTPKYSLAPLVPRLSELLGIQVVKVEDCIGPEVEKL
VASLPEGGVLLLENVRFYKEEEKNEPEFAKKLASLADLYVNDAFGTAHRAHASTEGVTKF
LKPSVAGFLLQKELDYLVGAVSNPKRPFAAIVGGSKVSSKIGVIESLLEKCDILLLGGGM
IFTFYKAQGLSVGSSLVEEDKLELATSLLEKAKAKGVSLLLPSDVVIADKFAPDANSKIV
PASAIPDGWMGLDIGPDSVKTFNDALDTTKTVIWNGPMGVFEFDKFAVGTEAIAKKLADL
SGKGVTTIIGGGDSVAAVEKVGVASVMSHISTGGGASLELLEGKVLPGVIALDEADAPVA
V
>Q9MTI5
MKRSLKNNPFVANPLLRKMEKLNRREDKILIRTWSRASTIILTMIGHTIAIHNGKEHLPI
YITDYMVGHKLGEFAPTINFHEHAKNDNKSRRSKMRIDY
>P05720
MALSNILLFKIEFYIFSSLLMIKKKLVFSILKLLQLTEKDSKILVNESLTNKNFSKLIEF
LDNYKVEKAKSITLQQLQSVLQNIKLNNSQKSEIIENIYSKLDAANHLIF
>P05721
MFILGKSNYTNFIYGHLVIRHIKYKLFDDYKKLIKKLSSKLKTSNSTKANLGDNFSLTLP
TWPFFILSWFLHIRTLEFSSNGNKTANSISSVFFLFPKLLNVV
>Q32S08
MNNYPWLTIITLFPISAGLLIPLIPNRGNNLIRWYALGICLIDFLLMTYVFGSQFDFYGQ
GIQLKEDISWINVIDFHWRVGVDGLSIALVLLTGFITTLATLAAWPVTRNPRLFYFLMLA
MYSGQLGLFLAQDLLLFFFMWELELIPVYLLLSMWGGRRRLYAATKFILYTAGGSIFLLA

AILTISLWGPNGPILDMETLSKQSYPLGLEILVYLGFLIAYAVKLPVFPFHTWLPDTHGE
AHYSTCMLLAGILLKMGGYGFIRINIEMLSDAHRIFAPWLVALGAGQIVYAALVSIAQKN
LKRRIAYSSVSHMGFVLIGAGSFSDLGLSGAILQMISHGLIGAGLFFLAGTTYDRSRTLI
LDDMGGWGSPLPKTFSTFTACAMASLALPGMSGFVAELMIFLGIVASSVYSPLFKAIITC
VEGIGIILTPIYLLSMVRKMFYGYNDISLKTLKDSLILDASPREVFIILSLLVPMLGIGF
YPDLTLQLWNQKAQEIVSLPSGNEKSLTLISYSYPVEKNFSLHH
>P48269
MNFVNLEQIENSLRNATFCMLFLTTFLYWFYTAFYSTNPQQIINPLSLTNIKTNFTYPDG
FQANNINVSTTYLPINPVLNTEREEQPEANGTNGLLGVSSLVVNLKSIAIPRIMMGVSNL
LLVLLLLVRWEKSGHFPLSNLYESLMFLAWCCTFLYLLYCTSFTLLVEKMLGSLIAPCSL
LMNAFATFSLPKEMQQASPLVPALQSNWLMMHVTVMIISYATLIIGSLLSILFLILFKHK
KGTPKKYDNFINNLDALSYRIIGLGFPFLTIGILSGAVWANEAWGSYWSWDPKETWALLT
WLVFAIYLHTRLTKGWEGEKPAIIAAVGFLVVWFCYLGVNLIGEGLHSYGFFN
>P05722
MYWSLTACSAVLFCLRDSLIFLADDVNPERGNEGVAVGTGGIPGRGIPPLITANGIGLYT
FTSIGLLVPFTLGLKTQNQQIKIVVKSKISETNKLVIFCCLKHIISRKARSWLNDSR
>P46666
MLIVLFRSKDIRGGRFVRPILIFRTKRSWILFRIGPERRREAEMPTDLCLFSNSPDPIVP
VFGTSSAKVTEWVSHQSNPFDKSGVILDIIFYIYRNIIE
>P05723
MICFKQQKITSLFVSLILLLTTILICWFCVLSPKVNGTNSPIEVNVYNPIPLAVMRGGIP
LPGMPPVPTATPSLPRSGFTSSAKKIKESRKQKSTALQAVKDQYILRVARL
>P05724
MQGLEYTLEPEQAKKCKTHQLVDLAKIDLNSWSQENVRPKTLLGRTSKKRLTSQFWYEFT
KKQKLKNALYYFVIYDITDNSILPVETLNQKNPLSTSFPFPFGRPLRGINFSGSCLHCLL
RVLK
>P05725
MNTKYNKEFLLYLAGFVDGDGSIIAQIKPNQSYKFKHQLSLAFQVTQKTQRRWFLDKLVD
EIGVGYVRDRGSVSDYILSEIKPLHNFLTQLQPFLKLKQKQANLVLKIIWRLPSAKESPD
KFLEVCTWVDQIAALNDSKTRKTTSETVRAVLDSLSEKKKSSP
>Q9SCX9
MRFRSFFFSSSIFSLSHSRSPSLSSSRFSSLSAAMSPALEKSRQGNGGCNDDSKSKVTVV
GSGNWGSVAAKLIASNALKLPSFHDEVRMWVFEEVLPNGEKLNDVINKTNENVKYLPGIK
LGRNVVADPDLENAVKDANMLVFVTPHQFMDGICKKLDGKITGDVEAISLVKGMEVKKEG
PCMISSLISKQLGINCCVLMGANIANEIAVEKFSEATVGYRGSREIADTWVQLFSTPYFM
VTPVHDVEGVELCGTLKNVVAIAAGFVDGLEMGNNTKAAIMRIGLREMKALSKLLFPSVK
DSTFFESCGVADVITTCLGGRNRRVAEAFAKSRGKRSFDELEAEMLQGQKLQGVSTAREV
YEVLKHCGWLEMFPLFSTVHQICTGRLQPEAIVQYRENKL
>O47027
MLNKKPPYLILSLIMIKTQKSKQTAVRKLIPGNVISLKITAMGANNVGINEFTFGIPVLV
PNAKLGETVQAKVLKILASKKIAIAKLIKVVTKTNATETNNLAALTPGATLDVTITKLGP
NSTGIADLGNNYNNVNKLIVKKSAITIGEKVTVLVTRVKNGYAFGVATVSTQRLNQVSTS
LTQNSFKGTKFTVVLPKNAKRYLKHLVFKVTTATAQNLSVNGFEQTLFKKGAVGLDTRIN
NQNGSAGNLTTVPTNTILFVKPNLGAKLGDKVQIQIIKFASIENGTLTYNVAIAKIIKLN

PLSTPQKKAFVRSSLRQMLKSGMHYGEKAIKCNARMKNYVWTRKKGTDTKVEARPLIKKG
RNLINLLKTRRCLTKALAQLTKYAAKGKTFLFVGTKKAASGLVARAALFSKKAFFVNTRW
LGGMLTNWKTILKSISKIRPILKEKQMIIKDILEKRQTIKARLIQKALLLRKKSKLMLKK
GRLLIQMLKQNNSNSTSGKQAVRFLFTEKTNLLNTKRKEFVSKGILLLEKRQQLVVKRQE
LITQSQTLKSKAIQLTNTISQFIKQFNLFT
>P32761
MSNFILKPGEKLPQDKLEELKKINDAVKKTKNFSKYLIDLRKLFQIDEVQVTSESKLFLA
GFLEGEASLNISTKKLATSKFGLVVDPEFNVTQHVNGVKVLYLALEVFKTGRIRHKSGSN
ATLVLTIDNRQSLEEKVIPFYEQYVVAFSSPEKVKRVANFKALLELFNNDAHQDLEQLVN
KILPIWDQMRKQQGQSNEGFPNLEAAQDFARNYKKGIK
>Q8MA04
MERNKNYYINVFHKQAENDLNFNKFYFYFFQETFYCLAYKQVSYKFLQNNVFIKKKKKFS
FFNLKRTIRSMRNQNYKESFFFIEQKKLSKKLLFSKLFYELLQEILKCILEISFKIKKNP
KISSYSTMSSIHGPFISFEENLIYFPVAIQSYLPNRVHPELIVRILRSYNLDVNLFHFLR
NIVHNGLYILSPPFLLNFGFRSLSTIFFNIYAYEIDLGFLSFLKLQKDLPQKYQTELDIF
SSSRKITFYFKNYSDFNNLKKIDQIERKYNNIIDYGPIYYLRYSNILLISLNLIKKNANF
FQLIYIRFFHLRFHFLFAKNLVKKISFNQDQIFFLGFLVFFFYTKIQIRIKLKKCLVNFI
KLEKKICINVPIKLLIIFLSKNGFCDISGNSKSKLSWSVLQDIEIIEKFRRLWLTISGYY
SGSSNKYCLKIVLYILRYSCAKTLACKHKMSLKKIWKKYTLNLSVTLKFQTGKKKFLSFS
HFKDFHKDEKSWQLNLKETNSIVYTFWN
>Q42533
MASSSFSVTSPAAAASVYAVTQTSSHFPIQNRSRRVSFRLSAKPKLRFLSKPSRSSYPVV
KAQSNKVSTGASSNAAKVDGPSSAEGKEKNSLKESSASSPELATEESISEFLTQVTTLVK
LVDSRDIVELQLKQLDCELVIRKKEALPQPQAPASYVMMQQPNQPSYAQQMAPPAAPAAA
APAPSTPASLPPPSPPTPAKSSLPTVKSPMAGTFYRSPAPGEPPFIKVGDKVQKGQVLCI
VEAMKLMNEIESDHTGTVVDIVAEDGKPVSLDTPLFVVQP
>P30222
MACKTLTCSASPLVSNGVVSATSRTNNKKTTTAPFSVCFPYSKCSVRKPASRLVAQATGD
NKDTSVDVHVSNNNQGGNNQGSAVERRPRRMALDVSPFGLLDPMSPMRTMRQMMDTMDRL
FEDTMTFPGSRNRGTGEIRAPWDIKDDENEIKMRFDMPGLSKEEVKVSVEDDVLVIKGEH
KKEESGKDDSWGRNYSSYDTRLSLPDNVDKDKVKAELKNGVLLISIPKTKVEKKVTDVEI
K
>P46307
MGQKVHPSGFRVGITKKHQSQWFARFNKNKYSQTVLEDRMIRDTLNKLFPELLNPSTKGG
SAPKRHQNARIKKSRITQIKIERNIVPYQIGIQIHAENCKLLKSSIKNLQVKKDILVKLQ
KTRQYLTNLKIKLDKLTNAPAETQVKTNFEDSTKRSVGQLELSHEGLNTKLSKIKSLRKK
LAKLKTGTSTTRANTTGTRQKKLSSKTKRSISQLLKMKLLRAKLAKLVQPKLKRKALEAS
TIEKTTAKTLKTKKKVSLKRKNKFETKLTKRQLKRQRVFKKRLKLRKFIRLRDRLLISKG
LFLQKKGHKITKKIVLAPTPKFLKDFSLNPKVYLLPVKRIEKLLTPKKAAKGGQKSAAVK
TVRTSLQTNIYNSRIQKKFVTMYVEQMKKKFLPHLNELFLEYTSKGFDSNNKAVLSLGYM
KQWDFERRVDNLKVQPIEKLDRLVHNLRDKFVMKLNLLRKDFITFGSFSSNSADILGLFQ
LYNFLMKLKNLVNGLKLNLKHKIKTRLAISKMNGSLNTSRNEPELRSRETSQASDKLRIV
DSTNSSLPQKVFRKKLENISNEYRKMKFIEYLKDVVQKHRTDNIYLYLASISESRRKLKE
IKNEVKQHLDLYLPGANSIKELNQKSSEDSINLATNEVNNVLMKLAKKHPFDRTFLDCKF

EELERRKTMWVQNLQLVPKISIKFFSVNQKALNLKASIVSESVVDALEKRKAFRKVIKTT
KENLMRNSEIKGVKIQVAGRLNGAEIARTEWVRAGRVPLQTLRANLDYSYRTANTIYGII
GVKVWIFKGFTKLVTESTGA
>Q42463
MASICTSNFHFLCRKNNSSPISHHLLLSPSSLSFSRCGGLRLCRCAAVKTGSEGGGIRSD
NAELLRKPVISTELETTSESEELVKEESDDEVGKKSGDGEGWVDWEDQILEDTVPLVGFV
RMILHSGKYAIGDRLSPDHQRTILQRLLPYHPECDKKIGPGVDYITVGYHPDFENSRCLF
IVRKDGETVDFSYWKCIKGLIRKNYPLYADSFILRHFRKRRRND
>Q06R99
MAKGTRFINFRRYGRFGSRRYRRFGSHKSVRKIPRGVIHIQTSFHNTIVTVTDGRGQVVS
WSSAGTCRFKGPRKRTPFAAQIVAADAIGPVVDQGLQRAKIKLKGTGRGRDAALRTISKS
GIGFTSVQDVTPVPHNGCRPPNKKRQ
>Q3ZJ77
MIKIIIKETFSFKSVLMHFLKEKVSNNKISKKNKIMPSYTLLSCIDSRVENLTKFYGRFE
LGPFAPGQALTVANALRRSLLSQLPGTSITLVEVRGASNEYEIITGVRESILDILLNLKQ
IVLTSDFEIFSPQIGFLSVEGPGVIRANDLKLPSFIYAVDPNQYIATLSNSGRLNMKFLI
CCGKNYITYNPNDSQYFEWLSLLKKSKPLIKSNSPKLNKGNINSEIDSDISIDKKNDEVI
FFQNANTRVVDSQTALKKGLVLKNTLRINKSSFLINSNYQSFFPKTQQKAFSIFRKNSKT
FLSTMGFYKEWKKEREFLKKDLYKNQEDFNKSYDFQETKKKLQTKLIKNFETPKKKLFKV
QNSANPFNKAFIPLTEEKVDYDSDFNLDHKSTKIGYFPIDAIFMPINRVNYLIESTEDIK
LKIKDRVILEVWTNGSIHPRHAIHKAAKALIQLFLPLQQIRTNLFLISDNSHFEEGQDKI
KKRIDNFKKEALLKANPQIQANPQLKKRNFDHRLLELDIANLELTARPYSCLKLANINTI
EDLISYSQEDLLSIKNFGRRSLIEVQKALQLMKLTLK
>Q5YLB4
MALLKPFPLQHSLRCMASRFLLHSHYHTHTLSFSSISLSRPSIVLNPRVINKLRRLSDAI
LIRNMVSLRAFMSSSTTTEAFQENTKSKGYGSEQIQVLEGLDPVRKRPGMYIGSTGPRGL
HHLVYEILDNAVDEAQAGFATKIDVVLHADNSVSIADNGRGIPTELHPVTKKSSLETVLT
VLHAGGKFGGSSSGYNVSGGLHGVGLSVVNALSQALEVTIWRDGKEYQQKYSRGKPITTL
ICHDLPVEMRDRQGTAIRFWPDKEVFTTEMQFDYNTIAGRIRELAFLNPELTIALKKEDI
DPEKIQCNEYFYAGGLVEYVKWLNADKKPLHDVLGFRKEADGITIDMALQWCSDAYSDTM
LGYANSIRTIDGGTHIDGVKAALTRILNNLGKKSKTIKEKDISLSGEHVREGLTCVISVK
VPNPEFEGQTKTRLGNPEVRKVVDQSVQEYLTEYLELHPDVLDSILSKSLNALKAALAAK
RARELVRQKSVLKSSSLPGKLADCSATNPEEAEIFIVEGDSAGGSAKQGRDRRFQAILPL
RGKILNIERKDEAAMYKNEEIQNLILGLGLGVKGEDFKKEALRYHKIIILTDADVDGAHI
RTLLLTFFFRYQRALFEEGCIYVGVPPLYKVERGKQVYYCYDDAELKKVQRSFPSNASYN
IQRFKGLGEMMPAQLWETTMNPETRLLKQLVVEDAAEANVVFSSLMGSRVDIRKQLIQNS
ASMMNLEQLDI
>Q8SAY0
MLASPALAGARAFAATVSGSLGIPIPAISAPSPSQARRRASLVVVAKVKVSTPQADRIAR
HVRLRKKVSGTTERPRLSVFRSNKHLYAQVIDDTKSCTLVSASTMHKSLSKDLEYSAGPT
VEVAQKIGEVIAKSCLEKGITKVVFDRGGFLYHGRIKALADAARENGLDF
>Q9ST69
MATTATTTPSATSLTTLHRRIPLFPTTTTLLSLSSSSKPLFLSLSSTRSFPTHLYCIKKD
DIDITFFEQDNPDEEITFDPPEKPEGYIPPRAVDEPPFESEEEIALAYEELYGAAYSGES

LLGNDVYAMDSKIKKATGFGSKSKKEKIRDGFEENVVQVRRVTKVVKGGKHMRFRAIVVV
GDKKGQVGVGVGKAKEVVSAVQKAAVDARRNIITVPMTKYLTFPHRNEADYGAARVMLRP
AAPGTGVIAGGAVRTVLEMAGVENALGKQLGSNNALNNARATIVAVQTMRQFSDVARDRG
IPMEELWK

>O24163

MTTTPIANHPNIFTHQSSSSPLAFLNRTSFIPFSSISKRNSVNCNGWRTRCSVAKDYTVP
SSAVDGGPAAELDCVIVGAGISGLCIAQVMSANYPNLMVTEARDRAGGNITTVERDGYLW
EEGPNSFQPSDPMLTMAVDCGLKDDLVLGDPNAPRFVLWKGKLRPVPSKLTDLAFFDLMS
IPGKLRAGFGAIGLRPSPPGHEESVEQFVRRNLGGEVFERLIEPFCSGVYAGDPSKLSMK
AAFGKVWKLEETGGSIIGGTFKAIKERSSTPKAPRDPRLPKPKGQTVGSFRKGLRMLPDA
ISARLGSKLKLSWKLSSITKSEKGGYHLTYETPEGVVSLQSRSIVMTVPSYVASNILRPL
SVAAADALSNFYYPPVGAVTITYPQEAIRDERLVDGELKGFGQLHPRTQGVETLGTIYSS
SLFPNRAPKGRVLLLNYIGGAKNPEILSKTESQLVEVVDRDLRKMLIKPKAQDPLVVGVR
VWPQAIPQFLVGHLDTLSTAKAAMNDNGLEGLFLGGNYVSGVALGRCVEGAYEVASEVTG
FLSRYAYK

>P49079

MRSLTVASRHPGAAFSTRRRPLLHPAAAGRDSTFQRCWRWEKTQDSSFGSSLRTSRLPRT
VHGDILKNLLAPTAGAVSVEQAEAIADLPKGDMWSVHKFGGTCMGTSERIHNVADIVLRD
PSERKLVVVSAMSKVTDMMYNLVNKAQSRDDSYIAVLDEVFDKHMTTAKDLLAGEDLARF
LSQLHADISNLKAMLRAIYIAGHATESFSDFVVGHGELWSAQMLSYAIQKSGTPCSWMDT
REVLVVNPSGANQVDPDYLESEKRLEKWFSRCPAETIIATGFIASTPENIPTTLKRDGSD
FSAAIIGSLVKARQVTIWTDVDGVFSADPRKVSEAVILSTLSYQEAWEMSYFGANVLHPR
TIIPVMKYNIPIVIRNIFNTSAPGTMICQQPANENGDLEACVKAFATIDKLALVNVEGTG
MAGVPGTANAIFGAVKDVGANVIMISQASSEHSVCFAVPEKEVALVSAALHARFREALAA
GRLSKVEVIHNCSILATVGLRMASTPGVSATLFDALAKANINVRAIAQGCSEYNITIVLK
QEDCVRALRAAHSRFFLSKTTLAVGIIGPGLIGRTLLNQLKDQAAVLKENMNIDLRVMGI
AGSRTMLLSDIGVDLTQWKEKLQTEAEPANLDKFVHHLSENHFFPNRVLVDCTADTSVAS
HYYDWLKKGIHVITPNKKANSGPLDRYLKLRTLQRASYTHYFYEATVGAGLPIISTLRGL
LETGDKILRIEGIFSGTLSYIFNNFEGARTFSDVVAEAKKAGYTEPDPRDDLSGTDVARK
VIILARESGLGLELSDIPVRSLVPEALKSCTSADEYMQKLPSFDEDWARERKNAEAAGEV
LRYVGVVDVVSKKGQVELRAYKRDHPFAQLSGSDNIIAFTTSRYKDQPLIVRGPGAGAEV
TAGGVFCDILRLSSYLGAPS

>Q38802

MSLQYHVLNSIPSTTFLSSTKTTISSSFLTISGSPLNVARDKSRSGSIHCSKLRTQEYIN
SQEVQHDLPLIHEWQQLQGEDAPQISVGSNSNAFKEAVKSVKTILRNLTDGEITISAYDT
AWVALIDAGDKTPAFPSAVKWIAENQLSDGSWGDAYLFSYHDRLINTLACVVALRSWNLF
PHQCNKGITFFRENIGKLEDENDEHMPIGFEVAFPSLLEIARGINIDVPYDSPVLKDIYA
KKELKLTRIPKEIMHKIPTTLLHSLEGMRDLDWEKLLKLQSQDGSFLFSPSSTAFAFMQT
RDSNCLEYLRNAVKRFNGGVPNVFPVDLFEHIWIVDRLQRLGISRYFEEEIKECLDYVHR
YWTDNGICWARCSHVQDIDDTAMAFRLLRQHGYQVSADVFKNFEKEGEFFCFVGQSNQAV
TGMFNLYRASQLAFPREEILKNAKEFSYNYLLEKREREELIDKWIIMKDLPGEIGFALEI
PWYASLPRVETRFYIDQYGGENDVWIGKTLYRMPYVNNNGYLELAKQDYNNCQAQHQLEW
DIFQKWYEENRLSEWGVRRSELLECYYLAAATIFESERSHERMVWAKSSVLVKAISSSFG
ESSDSRRSFSDQFHEYIANARRSDHHFNDRNMRLDRPGSVQASRLAGVLIGTLNQMSFDL

FMSHGRDVNNLLYLSWGDWMEKWKLYGDEGEGELMVKMIILMKNNDLTNFFTHTHFVRLA
EIINRICLPRQYLKARRNDEKEKTIKSMEKEMGKMVELALSESDTFRDVSITFLDVAKAF
YYFALCGDHLQTHISKVLFQKV
>O48917
MAHLLSASCPSVISLSSSSSKNSVKPFVSGQTFFNAQLLSRSSLKGLLFQEKKPRKSCVF
RATAVPITQQAPPETSTNNSSSKPKRVMVIGGDGYCGWATALHLSKKNYEVCIVDNLVRR
LFDHQLGLESLTPIASIHDRISRWKALTGKSIELYVGDICDFEFLAESFKSFEPDSVVHF
GEQRSAPYSMIDRSRAVYTQHNNVIGTLNVLFAIKEFGEECHLVKLGTMGEYGTPNIDIE
EGYITITHNGRTDTLPYPKQASSFYHLSKVHDSHNIAFTCKAWGIRATDLNQGVVYGVKT
DETEMHEELRNRLDYDAVFGTALNRFCVQAAVGHPLTVYGKGGQTRGYLDIRDTVQCVEI
AIANPAKAGEFRVFNQFTEQFSVNELASLVTKAGSKLGLDVKKMTVPNPRVEAEEHYYNA
KHTKLMELGLEPHYLSDSLLDSLLNFAVQFKDRVDTKQIMPSVSWKKIGVKTKSMTT
>A2CI50
MAVRFLFEALQKAIDEEMEREKRVVLIGEDIGHYGGSYKVTQGLYGKYGKHRVIDTPIAE
YSFVGAAVGAAATGLIPVVEGMNMAFILLAYSQISNNMGMLCATSGGHFQVPMVLRGPGG
IGKQLGAEHSQRLESYFQSVPGLQIVTCSTPYNAKGLLKSAIRSKNPILFIEHVLLYNLK
GEVPDNDYLLPLEKAELVREGSDITVLTYSRQRYNVIQAVKVLVEEGYDPEVIDLISLKP
FDMETIGKSIQKTHKVLIVEECMMTGGISNVLQSLIIDNFFDALDAAPLILSSPNVPTPY
TGPLEEATVVQTIDIIESIEYGITGKPPKPRTAKK
>Q38732
MATINLSLLPKTLTPNSKTLAPLLSILSTSSLSFLPCTRPHPIKSRSAAYPTVRALTDGE
YSSRRNNNNNNSGEERETIMLPGCDYNHWLIVMEFPKDPAPTREQMIDTYLNTLATVLGS
MEEAKKNMYAFSTTTYTGFQCTVTEETSEKFKGLPGVLWVLPDSYIDVKNKDYGGDKYVN
GEIIPCQYPTYQPKQSRSSKYKSKAYVRQRDGPPAEQRRPKQEATPESST
>Q948J9
MELLNGVETLVSGIHHHHRTNAKRNRLVRSVKILNSGNHEIPRKCLCFDLYDKLVPYKKA
WSWQKSIVEEKKTLIDRNQDCADTVILLQHSPVYTMGTASTEDYLNFDIKDAPFNVYRTE
RGGEVTYHGPGQLVMYPIINLRNHEMDLHWYLRMLEEIVIRVLSSTFSIKASRLDGLTGV
WVGNQKVAAIGIRVSKWITYHGLALNVTTDLTPFNWIVPCGIRDRKVGNIKGLLEDGEHG
MVDDLRLIDIVHESLLKEFSEAFQLQIEKQTVSDPNIL
>Q42545
MAIIPLAQLNELTISSSSSSFLTKSISSHSLHSSCICASSRISQFRGGFSKRRSDSTRSK
SMRLRCSFSPMESARIKVIGVGGGGNNAVNRMISSGLQSVDFYAINTDSQALLQSSAENP
LQIGELLTRGLGTGGNPLLGEQAAEESKDAIANALKGSDLVFITAGMGGGTGSGAAPVVA
QISKDAGYLTVGVVTYPFSFEGRKRSLQALEAIEKLQKNVDTLIVIPNDRLLDIADEQTP
LQDAFLLADDVLRQGVQGISDIITIPGLVNVDFADVKAVMKDSGTAMLGVGVSSSKNRAE
EAAEQATLAPLIGSSIQSATGVVYNITGGKDITLQEVNRVSQVVTSLADPSANIIFGAVV
DDRYTGEIHVTIIATGFSQSFQKTLLTDPRAAKLLDKMGSSGQQENKGMSLPHQKQSPST
ISTKSSSPRRLFF
>Q06FN2
MTNNKNFADWDSLECKDLHNDLLYGRFALSPLTAKESRLLKKGLREALLTGILCLRFTHA
KIQNACKNLNLMNIVGIQESLDEILKNFGKIILTGKLEEFVGKGPFVAILDVRGPLNAMA
VDIELPPGIKVEIETQHIATITEPIPFVVELRIELVSSTSKGETGITDEEGFSIDPNPPI
QKVNSSIQGYEYGGQTFQTLFIEILSTSPTVPNKALLLVSMKIMNLFQIVLQAKYLDYKE

LEKGIHVGVFCVSALRAEQSKWIKTILEDALYMVGGRKHQGPLTDEEDDSIDSNFTPVQN
LDCRIESYEEEGQTFQRLFLEIWTKSPTEPQEALWEASAKILELFSLFLQTSKENEKDLK
QIIKDWTENKRKHQEVLRLLDSEESGSIGWITKMKLAYMHMTLLSMNAMYILLVHRLKPD
YDRYNSITDQIVQELRASLNKLREIQKGEYSEQRILVHSIAQEIEAALQKYETTYKLDDF
VIKAKKDMITMIWDKERADLESSLIRWESDEDYLNLKKMNPVEMDRSFAELQYQETLRKE
QDEQSSQQQKDQMEKRRWERQNRERERKRGNREF
>P53536
MASMTMRFHPNSTAVTESVPRRGSVYGFIGYRSSSLFVRTNVIKYRSVKRNLEFRRRSAF
SVKCGSGNEAKQKVKDQEVQQEAKTSPSSFAPDTTSIVSSIKYHAEFTPLFSPEKFELPQ
AFIATAQSVRDALIINWNATYDYYEKLNVKQAYYLSMEFLQGRALLNAIGNLELTGPYAE
ALSQLSYKLEDVAHQEPDAALGNGGLGRLASCFLDSLATLNYPAWGYGLRYKYGLFKQRI
TKDGQEEVAEDWLEMGNPWEIVRNDVSYPVRFYGKVVSGSDGKKHWVGGEDIKAVAHDVP
IPGYKTRSTINLRLWSTKAASEEFDLNAFNSGRHTEASEALANAEKICYILYPGDESIEG
KTLRLKQQYTLCSASLQDIIARFERRSGASVNWEDFPEKVAVQMNDTHPTLCIPELMRIL
IDIKGLSWKDAWNITQRTVAYTNHTVLPEALEKWSMDLMEKLLPRHVEIIEMIDEELIRT
IIAEYGTADSDLLDKKLKEMRILENVELPAEFADILVKTKEATDISSEEVQISKEGGEEE
ETSKEGGEEEEEKEVGGGREEGDDGKEDEVEKAIAEKDGTVKSSIGDKKKKLPEPVPVPP
KLVRMANLCVVGGHAVNGVAEIHSEIVKDDVFNAFYKLWPEKFQNKTNGVTPRRWIRFCN
PDLSKIITQWIGTEDWILNTEKLAELRKFADNEDLQTQWREAKRNNKVKVAAFLRERTGY
SVSPDSMFDIQVKRIHEYKRQLLNIFGIVYRYKKMKEMNAAERKENFVPRVCIFGGKAFA
TYVQAKRIVKFITDVGATVNHDPEIGDLLKVIFVPDYNVSVAEMLIPASELSQHISTAGM
EASGTSNMKFAMNGCLQIGTLDGANVEIREEVGADNFFLFGAKAREIVGLRKERARGKFV
PDPRFEEVKKFVRSGVFGSYNYDELIGSLEGNEGFGRADYFLVGQDFPSYLECQEEVDKA
YRDQKKWTRMSILNTAGSSKFSSDRTIHEYAREIWNIEPVKLE
>Q43349
MSASASSLSAFNPKSLPLCVSRPASVSVLPPSLSFKLHSDHLVSIFASSALKCSSPAEYP
SRFVRNVAVSSDFEVEEDDMFADGDDSAPVERNSFSPDLKLFVGNLSFNVDSAQLAQLFE
SAGNVEMVEVIYDKVTGRSRGFGFVTMSTAAEVEAAAQQFNGYEFEGRPLRVNAGPPPPK
REESFSRGPRSGGYGSERGGGYGSERGGGYGSERGGGYGSERGGGYGSQRSGGGYGGSQR
SSYGSGSGSGSGSGSGNRLYVGNLSWGVDDMALENLFNEQGKVVEARVIYDRDSGRSKGF
GFVTLSSSQEVQKAINSLNGADLDGRQIRVSEAEARPPRGQF
>Q9C5W3
MFLCFCPCHVPIMSRLSPATGISSRLRFSIGLSSDGRLIPFGFRFRRNDVPFKRRLRFVI
RAQLSEAFSPDLGLDSQAVKSRDTSNLPWIGPVPGDIAEVEAYCRIFRSAERLHGALMET
LCNPVTGECRVPYDFSPEEKPLLEDKIVSVLGCILSLLNKGRKEILSGRSSSMNSFNLDD
VGVAEESLPPLAVFRGEMKRCCESLHIALENYLTPDDERSGIVWRKLQKLKNVCYDAGFP
RSDNYPCQTLFANWDPIYSSNTKEDIDSYESEIAFWRGGQVTQEGLKWLIENGFKTIVDL
RAEIVKDTFYQTALDDAISLGKITVVQIPIDVRMAPKAEQVELFASIVSDSSKRPIYVHS
KEGVWRTSAMVSRWKQYMTRPITKEIPVSEESKRREVSETKLGSNAVVSGKGVPDEQTDK
VSEINEVDSRSASSQSKESGRFEGDTSASEFNMVSDPLKSQVPPGNIFSRKEMSKFLKSK
SIAPAGYLTNPSKILGTVPTPQFSYTGVTNGNQIVDKDSIRRLAETGNSNGTLLPTSSQS
LDFGNGKFSNGNVHASDNTNKSISDNRGNGFSAAPIAVPPSDNLSRAVGSHSVRESQTQR
NNSGSSSDSSDDEAGAIEGNMCASATGVVRVQSRKKAEMFLVRTDGVSCTREKVTESSLA
FTHPSTQQQMLLWKTTPKTVLLLKKLGQELMEEAKEAASFLYHQENMNVLVEPEVHDVFA

RIPGFGFVQTFYIQDTSDLHERVDFVACLGGDGVILHASNLFKGAVPPVVSFNLGSLGFL
TSHPFEDFRQDLKRVIHGNNTLDGVYITLRMRLRCEIYRKGKAMPGKVFDVLNEIVVDRG
SNPYLSKIECYEHDRLITKVQGDGVIVATPTGSTAYSTAAGGSMVHPNVPCMLFTPICPH
SLSFRPVILPDSAKLELKIPDDARSNAWVSFDGKRRQQLSRGDSVRIYMSQHPLPTVNKS
DQTGDWFRSLIRCLNWNERLDQKAL
>Q9FLN4
MAMATSMSLNLIGAFKGLSLSSTSSFLRGDLSFSPKTSFTVTLPLENLQAPIPLTIESAH
KKGAGSTKNGRDSPGQRLGVKIYGDQVAKPGAIIVRQRGTKFHAGKNVGIGKDHTIFSLI
DGLVKFEKFGPDRKKISVYPREIVPENPNSYRARKRENFRLQREKKKARRENYSYTLPTP
ELVLASASVDDAEANPEC
>P46248
MASLMLSLGSTSLLPREINKDKLKLGTSASNPFLKAKSFSRVTMTVAVKPSRFEGITMAP
PDPILGVSEAFKADTNGMKLNLGVGAYRTEELQPYVLNVVKKAENLMLERGDNKEYLPIE
GLAAFNKATAELLFGAGHPVIKEQRVATIQGLSGTGSLRLAAALIERYFPGAKVVISSPT
WGNHKNIFNDAKVPWSEYRYYDPKTIGLDFEGMIADIKEAPEGSFILLHGCAHNPTGIDP
TPEQWVKIADVIQEKNHIPFFDVAYQGFASGSLDEDAASVRLFAERGMEFFVAQSYSKNL
GLYAERIGAINVVCSSADAATRVKSQLKRIARPMYSNPPVHGARIVANVVGDVTMFSEWK
AEMEMMAGRIKTVRQELYDSLVSKDKSGKDWSFILKQIGMFSFTGLNKAQSDNMTDKWHV
YMTKDGRISLAGLSLAKCEYLADAIIDSYHNVS
>P82244
MATLSLLSTGVGAAITNRTPSASLTFITGSRTTNKRVSFNGGSARSGSLHCSFLAPSSSL
SSNFSGLSLGLDLTSNTGVSTDRCRRFVVRAGKAALCLTKRSRSRKSLARTHGFRLRMST
TSGRALLKRRRAKGRKILCTKTNPSSGKRASP
>A1E9S2
MAKKSLIQREKKRHKLEQKYHLIRRSSKKKIRSKVSPLSLSEKTKMQEKLQSLPRNSAPT
RLHRRCFLTGRPRANYRDFGLSGHILREMVYACLLPGATRSSW
>P02902
MAHCLAAVSSFSPSAVRRRLSSQVANVVSSRSSVSFHSRQMSFVSISSRPSSLRFKICCA
AMGEAQAKKETVDKVCMIVKKQLAVPDGTPVTAESKFSELGADSLDTVEIVMGLEEEFNI
TVDETSAQDIATVQDAANLIEKLVTEKTA
>A2Y3Q5
MWSSSQASTRGVIEVGRVEAGPSHFPKRPAPRNSSRVNLSRTYAIKSCSVSSRTGLCLGQ
CYHKKSSACKCKLGWSSQPLSSLRHHLRVHSSASEAVLTSQSDFTKLLVGNEKIGVLLLN
LGGPETLDDVQPFLFNLFADPDIIRLPRLFRFLQKPLAQFISVVRAPKSKEGYASIGGGS
PLRQITDAQAEALRKALCDKDIPAKVYVGMRYWHPFTEEAIEQIKRDGITKLVVLPLYPQ
FSISTSGSSLRLLEGIFREDEYLVNMQHTVIPSWYQREGYIKAMATLIEKELRTFSEPQK
VMIFFSAHGVPLAYVEEAGDPYKAEMEECVDLIMEELEKRGITNSCTLAYQSRVGPVEWL
RPYTDETIIELGQKGVKSLLAVPISFVSEHIETLEEIDVEYKELALESGIKHWGRVPALG
CEPTFITDLADAVIESLPYVGAMAVSNLEARQPLVPLGSVEELLAAYDSKRDELPPPVTV
WEWGWTKSAETWNGRAAMLAVLALLVLEVTTGEGFLHQWGILPLFH
>Q8VYN6
MDALSQAISSGISVPYKNNSSSLVPSHGLTSLILRKSRSPVNPSSRSRVSVRASEIQHSK
TSASSIDLSDPDWKLKYEKDFEQRFSIPHITDVLPDAEAIRSTFCLKMRSPTEDFVGGYP
SDEEWHGYINNNDRVLLKVISYSSPTSAGAECLDHDCSWVEQWIHRAGPREKIYFRPEEV

KAAIITCGGLCPGLNDVIRHIVITLEIYGVKNIVGIPFGYRGFSDKDLTEMPLSRKVVQN
IHLSGGSLLGVSRGGPSVSEIVDSMEERGINMLFVLGGNGTHAGANAIHNECRKRKIKVA
VVGVPKTIDNDILHMDKTFGFDTAVEEAQRAINSAYIEAHSAYHGIGVVKLMGRNSGFIA
MQASLASGQVDICLIPEVPFNLHGPNGVLKHLKYLIETKGSAVICVAEGAGQNFLEKTNA
KDASGNAVLGDFGVYIQQETKKYFKEISTPIDVKYIDPTYMIRAVRANASDGILCTVLGQ
NAVHGAFAGYSGITVGIINTHYAYLPITEVIAYPKSVDPNSRMWHRCLTSTGQPDFI
>Q3ZJ89
MTNKLDCLKLINFIEKSNLFTTNKIRDKKLLIAFSGGQDSSCLLTIFYILSKKWGFKLGA
VYCNHCWTDSPQSSLSAFDTLQMFDIPFYFVESPNSEPMKPEQKARDWRYSSFYTISKWE
DYDFVLTGHSLSDCVETVLFNLFRGSGLKGICSLKEDQKFSNLKTNDFFFQKTVCFLDPF
FYFSDKKGNPFFRKYRLFQSMSFLSIFPKFDFFAFGENCAIDKSNFPLKNKSKGKNLQFL
TSFDIKNWFGIKKICRFCFLDKIGQTGIKKKRGTNRVPILIFSRRVNKTTSPLALNFQTK
NKRVDRKSFLFRFSLAFALPLAFALRAKGEKVRKNALLNQIKPVSFFFKTKKDKTETNLF
HRYLQTIKKLQNRDKISSFITIFRPFILTLGNVKIKGKKIKNKNLPFQIKKLIRKKEKEK
GILWNSLSKLDNKDKLISNQIQKKGFKLIPKFILTKDKKSFSNFPPFIFDQLEKTFRKEK
VDTKPRNSSPQFEVLFNKLSQPSSDKKNVDFIVFRPLIKITRETLFLFSNNLKIPIYYDK
SNKDLNITRNYIRKVLVPLLKKINPRVEENIYKFSKIIEFYYQVFGDLKCPSDRFDIFNP

>P0C471
MKKILFSMFYSILVGEEPDSVFLKKEGKQNQVKMIWVAPSSCAKDLTISEGTGATFLFNF
HSRVSYLFPRPFLRPRNMKWTNSFS
>P94044
MSTATAPRLPAPRSGASYHYQTTAAPAANTLSFAGHARQAARASGPRLSSRFVASAAAVL
HKVKLVGPDGTEHEFEAPDDTYILEAAETAGVELPFSCRAGSCSTCAGRMSAGEVDQSEG
SFLDDGQMAEGYLLTCISYPKADCVIHTHKEEDLY
>Q84KJ5
MAASSLSLSSPLSNPLRRFTLHHLHLSKKPLSSSSLFLCSAAKMNDHIHRVPALTEEEID
SVAIKTFERYALPSSSSVKRKGKGVTILWFRNDLRVLDNDALYKAWSSSDTILPVYCLDP
RLFHTTHFFNFPKTGALRGGFLMECLVDLRKNLMKRGLNLLIRSGKPEEILPSLAKDFGA
RTVFAHKETCSEEVDVERLVNQGLKRVGNSTKLELIWGSTMYHKDDLPFDVFDLPDVYTQ
FRKSVEAKCSIRSSTRIPLSLGPTPSVDDWGDVPTLEKLGVEPQEVTRGMRFVGGESAGV
GRVFEYFWKKDLLKVYKETRNGMLGPDYSTKFSPWLAFGCISPRFIYEEVQRYEKERVAN
NSTYWVLFELIWRDYFRFLSIKCGNSLFHLGGPRNVQGKWSQDQKLFESWRDAKTGYPLI
DANMKELSTTGFMSNRGRQIVCSFLVRDMGLDWRMGAEWFETCLLDYDPCSNYGNWTYGA
GVGNDPREDRYFSIPKQAQNYDPEGEYVAFWLQQLRRLPKEKRHWPGRLMYMDTVVPLKH
GNGPMAGGSKSGGGFRGSHSGRRSRHNGP
>P31541
MMARALVQSTNILPSVAGERAGQFNGSRKDQRTVRMLCNVKCCSSRLNNFAGLRGCNALD
TLLVKSGETLHSKVAAATFVRRPRGCRFVPKAMFERFTEKAIKVIMLAQEEARRLGHNFV
GTEQILLGLIGEGTGIAAKVLKSMGINLKDARVEVEKIIGRGSGFIAVEIPFTPRAKRVL
ELSLEEARQLGHNYIGSEHLLLGLLREGEGVAARVLENLGADPTNIRTQVIRMVGESSEA
VGASVGGGTSGLKMPTLEEYGTNLTKLAEEGKLDPVVGRQAQIERVTQILGRRTKNNPCL
IGEPGVGKTAIAEGLAQRIANGDVPETIEGKKVITLDMGLLVAGTKYRGEFEERLKKLME
EIKQSDEIILFIDEVHTLIGAGAAEGAIDAANILKPALARGELQCIGATTLDEYRKHIEK

DPALERRFQPVKVPEPSVDETIQILKGLRERYEIHHKLHYTDEAIEAAAKLSHQYISDRF
LPDKAIDLIDEAGSRVRLRHAQLPEEARELEKELRQITKEKNEAVRGQDFEKAGELRDRE
MDLKAQISALIDKNKEKSKAESEAGDAAGPIVTEADIQHIVSSWTGIPVEKVSTDESDRL
LKMEETLHTRVIGQDEAVKAISRAIRRARVGLKNPNRPIASFIFSGPTGVGKSELAKSLA
TYYFGSEEAMIRLDMSEFMERHTVSKLIGSPPGYVGYTEGGQLTEAVRRRPYTVVLFDEI
EKAHPDVFNMMLQILEDGRLTDSKGRTVDFKNTLLIMTSNVGSSVIEKGGRRIGFDLDFD
EKDSSYNRIKSLVTEELKQYFRPEFLNRLSEMIVFRQLTKLEVKEIADIMLKEVFVRLKN
KEIELQVTERFRDRVVDEGYNPSYGARPLRRAIMRLLEDSMAEKMLAGEIKEGDSVIVDV
DSDGNVTVLNGTSGAPSDSAPEPILV
>O65837
MECVGVQNVGAMAVLTRPRLNRWSGGELCQEKSIFLAYEQYESKCNSSSGSDSCVVDKED
FADEEDYIKAGGSQLVFVQMQQKKDMDQQSKLSDELRQISAGQTVLDLVVIGCGPAGLAL
AAESAKLGLNVGLVGPDLPFTNNYGVWEDEFKDLGLQACIEHVWRDTIVYLDDDEPILIG
RAYGRVSRHFLHEELLKRCVEAGVLYLNSKVDRIVEATNGQSLVECEGDVVIPCRFVTVA
SGAASGKFLQYELGSPRVSVQTAYGVEVEVDNNPFDPSLMVFMDYRDYLRHDAQSLEAKY
PTFLYAMPMSPTRVFFEETCLASKDAMPFDLLKKKLMLRLNTLGVRIKEIYEEEWSYIPV
GGSLPNTEQKTLAFGAAASMVHPATGYSVVRSLSEAPKCASVLANILRQHYSKNMLTSSS
IPSISTQAWNTLWPQERKRQRSFFLFGLALILQLDIEGIRSFFRAFFRVPKWMWQGFLGS
SLSSADLMLFAFYMFIIAPNDMRKGLIRHLLSDPTGATLIRTYLTF
>P16096
MASASLLKTSPVLDNPEFLKGQTLRIPSVAGVRFTPSGSSSLTVRASSYADELVKTAKTV
ASPGRGILAMDESNATCGKRLASIGLENTEANRQAYRTLLISAPGLGQYVSGAILFEETL
YQSTTDGKKMVDVLIEQGIVPGIKVDKGWLPLPGSNDESWCQGLDGLACRSAAYYQQGAR
FAKWRTVVSIPNGPSALAVKEAAWGLARYAAITQDNGLDPILEPEIMLDGEHGIDRTFRV
AQQVWAEVFFNLAENNVLLEGSSLKPSMVGPGALSARKGPPEQVADYPLKLLHRRRGPVV
PGIMVLSGGQSEVEATLNLNAMNQSPNPWHVSFSYARALQNTCLKTWVEGQENVKAQDFA
CAKSNSLAQLGKYTGEGESEERKKDMFVKATLTY
>Q06SG0
MINDTISDMLTRIRNASIVRKKSVLIPYTKMNLKIAEILEKEGYILSFEINTNKAISITK
KTSETNLRMLKIYLKYYKKYKTNFYKLNVIKNKKALSDFKNLIKLLKGKQGNKKTPCITN
LKRISKPGLRIYVNHKELPRVLAGTGIYILSTSKGILTDREARFRGIGGEVLCSVW
>P59287
MALPSAAMQSNPEKLNLFHRLSSLPTTSLEYGNNRFPFFSSSAKSHFKKPTQACLSSTTH
QEVRPLAYFPPTVWGNRFASLTFNPSEFESYDERVIVLKKKVKDILISSTSDSVETVILI
DLLCRLGVSYHFENDIEELLSKIFNSQPDLVDEKECDLYTAAIVFRVFRQHGFKMSSDVF
SKFKDSDGKFKESLRGDAKGMLSLFEASHLSVHGEDILEEAFAFTKDYLQSSAVELFPNL
KRHITNALEQPFHSGVPRLEARKFIDLYEADIECRNETLLEFAKLDYNRVQLLHQQELCQ
FSKWWKDLNLASDIPYARDRMAEIFFWAVAMYFEPDYAHTRMIIAKVVLLISLIDDTIDA
YATMEETHILAEAVARWDMSCLEKLPDYMKVIYKLLLNTFSEFEKELTAEGKSYSVKYGR
EAFQELVRGYYLEAVWRDEGKIPSFDDYLYNGSMTTGLPLVSTASFMGVQEITGLNEFQW
LETNPKLSYASGAFIRLVNDLTSHVTEQQRGHVASCIDCYMNQHGVSKDEAVKILQKMAT
DCWKEINEECMRQSQVSVGHLMRIVNLARLTDVSYKYGDGYTDSQQLKQFVKGLFVDPIS
I
>Q05753

MQSLSTPHTISLLLPRTSPSRLSPSLHSLAFPTRLRSLSYSSQTSILPDAGDDFIVGDCL
VYEDGVFEDPYLDKEVTQVAKQERKKNRRGGAKRLDESEIEPENLVPEEWRDIQAEVNLT
KKDKRKIAQEMEFGVRVEKKRQGLIPLRKVDLNDFLTYKEAKLAQLRPVILDKPGNFSDD
SGASSDGETAVSSPSERVAPKNPRWAVYGKGFDHVAKFFNSDKYDPSDKKSDGPRKLLSK
EEKFMLNSRNPDLAVATSKKWLPLHTLAACGEFYLVDSLLKHNLDINATDVGGLTVLHRA
IIGKKQAITNYLLRESANPFVLDDEGATLMHYAVQTASAPTIKLLLLYNADINAQDRDGW
TPLHVAVQARRSDIVKLLLIKGADIEVKNKDGLTPLGLCLYLGREIRTYEVMKLLKEFPL
SRHKKRLVTTDEDIE
>P55217
MAVSSFQCPTIFSSSSISGFQCRSDPDLVGSPVGGSSRRRVHASAGISSSFTGDAGLSSR
ILRFPPNFVRQLSIKARRNCSNIGVAQIVAAKWSNNPSSALPSAAAAAATSSASAVSSAA
SAAAASSAAAAPVAAAPPVVLKSVDEEVVVAEEGIREKIGSVQLTDSKHSFLSSDGSLTV
HAGERLGRGIVTDAITTPVVNTSAYFFKKTAELIDFKEKRSVSFEYGRYGNPTTVVLEDK
ISALEGAESTLVMASGMCASTVMLLALVPAGGHIVTTTDCYRKTRIFMENFLPKLGITVT
VIDPADIAGLEAAVNEFKVSLFFTESPTNPFLRCVDIELVSKICHKRGTLVCIDGTFATP
LNQKALALGADLVVHSATKYIGGHNDVLAGCICGSLKLVSEIRNLHHVLGGTLNPNAAYL
IIRGMKTLHLRVQQQNSTAFRMAEILEAHPKVSHVYYPGLPSHPEHELAKRQMTGFGGVV
SFEIDGDIETTIKFVDSLKIPYIAPSFGGCESIVDQPAIMSYWDLPQEERLKYGIKDNLV
RFSFGVEDFEDVKADILQALEAI
>Q06SG5
MARKGAPKKRLLLPDPIYNKVSVHMLVNRILKNGKKSIAYRIVYSVFRKISETMNQNPVE
VWEKALNNVKPRVEVKPRRRAGSIQQVPRVMPSAERARAIAIRWIVSACQKKAGKDMISK
LFSEISEASNKNGAAFRKKEELHKMALSNLMNSRRPDKIVKAITEQSEINDDVY
>A2CI69
MNDLPFTLDQLLILKAIAAQGSFKKAADSLYISQPAVSMQVQNIEKQLNVQLLDRGGRRA
NLTDAGHLLLRYGDRILALCDETCRALEDLRNLQTGTLIIGASQTTGTYLMPQLISLFRK
KYPQIMVQLHVDSTRHICWNVANGQIDIAIIGGEVPLELREILQVTPYADDELALILPCS
HPFSQRQEIQKEDLYQLHFIALYASSTIRKVVDNILREHDIHSSRFFIEMELNSIEAIKS
AVQSELGAAFVSASAITKELQLGLLHWAKIQNVILNRNLSIVTNPNRYRSKAAEKFSCEI
LASFQPPTVHPCG
>Q3ZJ90
MFIKKKKKKVSSFEFSSLRSTFSFVHWAKLKHQTSSIIENHSDQISPIQIDFYNRPFEKG
RLKALVSWSISYFGEKKTVDLVENLKSIGYAYATKAGISLGIDDLKIPPSKKDYITKAEQ
ILEFTNQDVKKGYLTSIEYFSKVIETWNKTSESLKEEVISNFKKTDELNPVFIMAFSGAR
GNISQVRQLTSMRGLMSDPQGRIINFPIQSNFREGLTLTEYLISCYGARKGVVDTALRTA
TSGYLTRRLVDVAHHVIVRGFNCGTKKGLYLSDLKKGDKILLSLKNRIIGRRLAEDIYNS
KKQLIATKNQEISSNLSSLITKNKTSIFVRSPLTCQDKNYVCQLCYGWSLANHRLVPSGE
AIGIIAAQSIGEPGTQLTMRTFHTGGVFSGEVTDEIKAPFRGIVFFNTSIPGKLIRTTYG
QIAFLTKQESFLTLIPENPKQQWLDSSKKLGFSKQKEPDFQKDSLFLQKKVEFKIPAYTL
LFVKNNQLVEKNQVLGEASTFLTRQNQSIESYQTIYSEFSGEVKFQHSKGVQVLKKELDF
KIDDQELSSVLKNKLRKLKSLFNPSANSSTGEFWILSAQKQTISKPVNLLVKPGDFLHQK
ALLYLAKKASYSNYFETIALDPSHNLPTLPLPILFYDFGVNSLAKNLNKNAFFNLALLNE
QLYLRKGNLFLMQDRSTQKKANRSTIKILPSLQKQTLQNFKKVCSDLPFVSRSLFPISES
SRDKLNSIQNGVKLQSTIVGKHRFLSLNIIFTQIAKKKFFKMNLNSEVKIKSLKQFDLNH

ESNEMKQIGVFNKDKTPTGGFGIQEIFYTSFESKLKTSPLFLLAQFSSDKTLIDLTKQNV
QDFKFKNLNYQYFHPFLVLASDPPFCVSEKTLHFMQSCGQSLNEIDPNPKIETFFSKSAP
SFLDKNRRNKRFKSLKKTMEKNHLLSLYYFLTQPLSDDNDDTIMNSFNRKSKLNKFSTKR
LNKKQIVFGKLNHHFFADKGGDDFHFKNFIHGGLSFKEVCEVDLYLKERVEVFNWKIATL
GTPVSDIITKAEKFVLFHSPTNACGSFILEQPLKNPCFYAFGKIGFLRGKTDYGQPNKIF
SSNLFNLKKGWKTKTEGRFTNRPNKQKFKSFINSVEKSGPLNKKIFQNEGPFALNDFTKI
QPIVGNQLNENFFTNAAELLKKRPGAAKQEFQLTSFLEKFINRHNRLFWFPKDQNILNFA
DQQSKGPTNLLFQRKFLKDLTLFSDGDPSFSCRSFVFQTKFYLSKKLFENLPRHNFENYD
IISGPINFQEFEILQKIPFSFYPLSSRSTQKFGPFFILPREVTKKQSFQIFKYPLLFELK
TYSPTFFSTVFFFIRNFGPKFHKKRERKLIKAIFRPLPRLIGFSTLKSPLDSGQTTTEKA
GLQKPVYRFIKKSELFFSIFDYQILKNYRRLASNLILKQNGLNLLNYSPILNSLLKSALL
FNIASAKKNSVFEKLGLKISAAYSESKTFGIATEKNSLNSNSVVLFQKKQSGFLIRELNP
FHNTLGSPFFDRSKKVDNPQSSLTFDRPQSANERKQILKKARQKLRLFPLNLNEKKNRFS
SVTLDLLRDQTTLHKMQSCGEAESGNLKTKETLFKKVKRENKKITEIFTFCPFCPQLKSK
GKRKSKGDQLFQEPCNLNLGENFLSCLPFGFEYPVKARRRLVKEQRLPFSLSLILPDKLN
YYQSLTSFPFSKQMGNRLLKNLTQLNFLNVGCIRLSQLDSKILRSDSFNPQRKELKSKKF
NNFSINELSDSFMNLGWTGFSQKNLILKYLDTDQIFKKGGPLKSNLQDSQLVECFFKTKK
VHFLFISKLLKESSLNSFFYNFYYSGSSFNSFADIQKFENKASFFQTKKKLYNFEKKDQW
QKKMSFLFFIQNKKTHLFLPNSKGFFLKKKKDISCERFSTNVILSKSQSKNETKELKKAS
LANQNLKKHSTISTENALKNKMNQSFSFSHFNKEKKPLSDCRAQTKGAIQKFSTKLPPTQ
TGWIFAILNPKIYLNKHNCVEFAGNSNLSDISFDNYVTLTCFLTIRFVNTFFELHANVDF
WLKTLNTFHLGLLKQSSFDFKASKIKLQKTPFLFPSFPPNYIELVFVKKAILRRVAKKDS
TFCNLYSFENFLLDKRFQKFKTHFNLQSHLKLAPSFCVDLQGSKGLENFAWTGKLRSQSQ
ASWILETNLFSPCFADKQSKSPLLEFENKKCPAFAKGKLQKSKIEKFEFTYNDIKNKLVY
FNKSFFLTSDQRPVFQRSFPKKIHRKTLFITHPSIIWKSRFESSLKSLTFFKMKAKQNGT
GSLSFFEKKSLETSLNKTSVLFKKVLAIKTFLLFSFSSLNLVSGKLNFLSSVDQNKKSNL
KIQSIFENFAYQQSKGSNEINFSRSAALHSMQSFVFSEKLRKTLSLRISNFHKIQKYANQ
NLESGIGFFSFFQKSLSIVFFDSAYHFSPQNTSTKDSFEIKKENKTVINNYCRFSNFKLL
KLPVFKNRFLNKYSLFKSFLNYLSYSFDVKTSKQILVRLPLLKETCFHFNKNSRFKPKLL
ILNQANSQQLLATCFVQPYSEFSNSSFVFNSKSAHLHSQTAVKHRQNFEKKSKIIFDERK
TFSFISSSTQVLFVSKKATHYLNENFRSQNYKKKTYDFIDNANVLKNRFFERLSPVEFHR
KREGFLSKDQKQMTFKYQNMQGGLIPALDSTSTFAPFARSSKARGSAKAIFSQAQRLWGE
ESFINDKQKSIRNQIIFAKNSRFKNLLILNNKNENEKLFYLNLKKKVSEQSTMNFFVPAL
YKKLFYTKQSISKFLEVKIQPNLQIQWTFFNSNISKHEKQQKFLLPLFDETFNIQGSNLK
NGLNFGMLSLSYSTLDPFFECLKKRVNSSWFFNGKQTFKKKKKIAKEGAFFNHSFFLDAK
KSKQLNKKIQKKFTKRLQTLNFSEIKKGFFISEKFKTRLSCLIKKPFLISTFFLSYRLKK
PKLALNFNYQSLGNNSKKFSLIRLNSIDFNLSKSQRGWFHNQNVSKQFRFFKHNRSVNLF
QIHFDFENSCDPCFAMQKQTTSSKPVLFLYNLKPLKTDFFQKGFQTTSQLLFKHINHSIV
PLKDDANHLSSFLNQANFRGAFEPKAKTIADKLISQNVAITKPNLPKSNFSSLKGEVFFV
TNSRQFKLVDLSVFKKSSREIQLLTNLDLITFRIKNRNFPSKHIEEQKPNLIEKQLAQSK
NKLQIYIGQLLRYGKEISPGIGLNQSGQILILQSNKLVLRYAKPFLLASGGICDLVQGDF
VKNQSPLLNLKYKSLKTEDIVQGIPKIEQLFEARENFQDELGINNLLKNKFLVYKTLYHP
KEAVRKSFEFIQHYIIDGIQYVYQSQGVNISDKHIEIIVKQMTSKVRILEPRNSGLLRGD
VVDLDWIERINLDILTGKKAQYEPIVLGITKASLDRRGFISAASFQETIKVLTKATILQR

RDYLRGLKENVILGHLINSGTGSTLYSILKEKKSNFLNRFLQ
>Q8L770
MASCLQASMNSLLPRSSSFSPHPPLSSNSSGRRNLKTFRYAFRAKASAKIPMPPINPKDP
FLSTLASIAANSPEKLLNRPVNADVPPYLDIFDSPQLMSSPAQVERSVAYNEHRPRTPPP
DLPSMLLDGRIVYIGMPLVPAVTELVVAELMYLQWLDPKEPIYIYINSTGTTRDDGETVG
MESEGFAIYDSLMQLKNEVHTVCVGAAIGQACLLLSAGTKGKRFMMPHAKAMIQQPRVPS
SGLMPASDVLIRAKEVITNRDILVELLSKHTGNSVETVANVMRRPYYMDAPKAKEFGVID
RILWRGQEKIIADVVPSEEFDKNAGIKSVV
>P29344
MASLAQQLAGGLRCPPLSNSNLSKPFSPKHTLKPRFSPIVSAVAVSNAQTRERQKLKQLF
EDAYERCRNAPMEGVSFTIDDFHTALDKYDFNSEMGSRVKGTVFCTDANGALVDITAKSS
AYLPLAEACIYRIKNVEEAGIIPGVREEFVIIGENEADDSLILSLRQIQYELAWERCRQL
QAEDVVVKGKIVGANKGGVVALVEGLRGFVPFSQISSKSSAEELLEKEIPLKFVEVDEEQ
SRLVMSNRKAMADSQAQLGIGSVVTGTVQSLKPYGAFIDIGGINGLLHVSQISHDRVSDI
ATVLQPGDTLKVMILSHDRERGRVSLSTKKLEPTPGDMIRNPKLVFEKAEEMAQTFRQRI
AQAEAMARADMLRFQPESGLTLSSDGILGPLTSDLPAEGLDLSVVPPAVES
>P05314
MASLPVNKIIPSSTTLLSSSNNNRRRNNSSIRCQKAVSPAAETAAVSPSVDAARLEPRVE
ERDGFWVLKEEFRSGINPAEKVKIEKDPMKLFIEDGISDLATLSMEEVDKSKHNKDDIDV
RLKWLGLFHRRKHHYGRFMMRLKLPNGVTTSEQTRYLASVIKKYGKDGCADVTTRQNWQI
RGVVLPDVPEIIKGLESVGLTSLQSGMDNVRNPVGNPLAGIDPHEIVDTRPFTNLISQFV
TANSRGNLSITNLPRKWNPCVIGSHDLYEHPHINDLAYMPATKNGKFGFNLLVGGFFSIK
RCEEAIPLDAWVSAEDVVPVCKAMLEAFRDLGFRGNRQKCRMMWLIDELGMEAFRGEVEK
RMPEQVLERASSEELVQKDWERREYLGVHPQKQQGLSFVGLHIPVGRLQADEMEELARIA
DVYGSGELRLTVEQNIIIPNVENSKIDSLLNEPLLKERYSPEPPILMKGLVACTGSQFCG
QAIIETKARALKVTEEVQRLVSVTRPVRMHWTGCPNSCGQVQVADIGFMGCMTRDENGKP
CEGADVFVGGRIGSDSHLGDIYKKAVPCKDLVPVVAEILINQFGAVPREREEAE
>O80575
MKSLASPPCLRLIPTAHRQLNSRQSSSACYIHGGSSVNKSNNLSFSSSTSGFASPLAVEK
ELRSSFVQTAAVRHVTGSLIRGEGLRFAIVVARFNEVVTKLLLEGAIETFKKYSVREEDI
EVIWVPGSFEIGVVAQNLGKSGKFHAVLCIGAVIRGDTTHYDAVANSAASGVLSASINSG
VPCIFGVLTCEDMDQALNRSGGKAGNKGAETALTALEMASLFEHHLK
>P92959
MATMSALQSSFTSLSLSPSSSFLGQRLISPISLSVTSPVKPAENPCLVLAKLKRWERKEC
KPNSLPILHKMHVKFGDTVKVISGRDKGKIGEVTKIFTHNSTIVIKDVNLKTKHMKSREE
GEPGQIVKIEAPIHSSNVMLYSKEKDVVSRVGHKVLEDGQKVRYLIKTGELIDTIEKWKL
LKEAKDKETTQVAVTSAS
>Q9XFS9
MMTLNSLSPAESKAISFLDTSRFNPIPKLSGGFSLRRRNQGRGFGKGVKCSVKVQQQQQP
PPAWPGRAVPEAPRQSWDGPKPISIVGSTGSIGTQTLDIVAENPDKFRVVALAAGSNVTL
LADQVRRFKPALVAVRNESLINELKEALADLDYKLEIIPGEQGVIEVARHPEAVTVVTGI
VGCAGLKPTVAAIEAGKDIALANKETLIAGGPFVLPLANKHNVKILPADSEHSAIFQCIQ
GLPEGALRKIILTASGGAFRDWPVEKLKEVKVADALKHPNWNMGKKITVDSATLFNKGLE
VIEAHYLFGAEYDDIEIVIHPQSIIHSMIETQDSSVLAQLGWPDMRLPILYTMSWPDRVP

CSEVTWPRLDLCKLGSLTFKKPDNVKYPSMDLAYAAGRAGGTMTGVLSAANEKAVEMFID
EKISYLDIFKVVELTCDKHRNELVTSPSLEEIVHYDLWAREYAANVQLSSGARPVHA
>P49235
MAPLLAAAMNHAAAHPGLRSHLVGPNNESFSRHHLPSSSPQSSKRRCNLSFTTRSARVGS
QNGVQMLSPSEIPQRDWFPSDFTFGAATSAYQIEGAWNEDGKGESNWDHFCHNHPERILD
GSNSDIGANSYHMYKTDVRLLKEMGMDAYRFSISWPRILPKGTKEGGINPDGIKYYRNLI
NLLLENGIEPYVTIFHWDVPQALEEKYGGFLDKSHKSIVEDYTYFAKVCFDNFGDKVKNW
LTFNEPQTFTSFSYGTGVFAPGRCSPGLDCAYPTGNSLVEPYTAGHNILLAHAEAVDLYN
KHYKRDDTRIGLAFDVMGRVPYGTSFLDKQAEERSWDINLGWFLEPVVRGDYPFSMRSLA
RERLPFFKDEQKEKLAGSYNMLGLNYYTSRFSKNIDISPNYSPVLNTDDAYASQEVNGPD
GKPIGPPMGNPWIYMYPEGLKDLLMIMKNKYGNPPIYITENGIGDVDTKETPLPMEAALN
DYKRLDYIQRHIATLKESIDLGSNVQGYFAWSLLDNFEWFAGFTERYGIVYVDRNNNCTR
YMKESAKWLKEFNTAKKPSKKILTPA
>Q8HSW1
MAAMIRLFRSSSSSSSNSISLISRSLSTAAASETVKSQSYPHNPHSTSVDPKAKTVQWVF
LGCPGVGKGTYASRLSTLLGVPHIATGDLVRDELKSSGPLSKQLAEIVNQGKLVSDEIIL
NLLSKRLESGEAKGEAGFILDGFPRTVRQAEILTEVTDIDLVVNLKLPERVLVEKCLGRR
ICSECGKNFNVASIDVAGENGAPRISMARLNPPFTVCFKLITRADDTEAIVKERLSIYWD
KSQPVEDFYRSQGKLLEFDLPGGIPESWPKLLEVLNLDEQEYKLSPAA
>P19587
MNKKIQDFFLSCKECILENPRSFYGSFSLGPFKNSQSLTVANALRRTLLAELSNIAITHL
EIEGVTHEYSTLVGVRESVLDLLLNFKGIALKNTSPVTKPLFGYLQVRGPGVVRASDLKF
PPMIQCVDPDQYIATLNENGKLILKFRISDFKNSQENLNFEVSKTFLNIPNFGHFDTSSF
QNFENSNFFSSSFFASQKMIKSKRKTKFSQLKVDYLSSFGFSTQHKRSGLNQTQKFAMQH
QNPFRIEKNQQDFNKNKNGNFDSQKNKTNSLWVDPLFNPILKVNYIIETIEPMQKNIPNE
IVFIELWTNGSIHPRKAFYTALLYLKTMFDKLDCMRLLNYEFSNTMLESEKTSTKFFKTF
EYDFRFYNSREDKTMKSFTPEKFFLPEEIEDVEKDYLLNLKNQADNTWNDLPLTNLNLPY
RITKILAKNNFFVVGDLLKISPNELKKLSGIGNYCVFILQKRFEKLGLKLGSRNEKNL
>P93184
MLTATKPLVGGACAAPSSSARRRTFVVPEARRKPGNGRRTSVSKVGSTSTSTTTTTTTTL
SADSNGAAVGTVTRPDVHVQDRTHATEMKATVTVHMSKAAGVRDFLYDLILKTWLHVDLV
SSELDPQTGQEREPISGAVKHSGRVDDEWDMYEATFKVPASFGPIGAVQVTNYHHSEMLL
GDIEVFPTGQEESAVTFHCKSWIDPSHCTPDKRVFFPAHSYLPSQTPKGVEGLRKRELEI
LRGTGCGERKEHDRIYDYDVYNDLGNPDDDNNPTTRPVLGGKEHPYPRRCRTGRPRSKKD
PFSEERSHKEHIYVPRDEAFTERKMGAFDTKKFMSQLHALTTGLKTAKHKSQSFPSLSAI
DQLYDDNFRNQPVQPEGGKLRFVIDLLETELLHLFKLEGAAFLEGIRRVFKFETPEIHDR
DKFAWFRDEEFARQTIAGMNPMSIQLVTEFPIKSNLDEATYGPADSLITKEVVEEQIRRV
MTADEAVQNKKLFMLDYHDLLLPYVHKVRKLDGTTLYGSRALFFLTADGTLRPIAIELTR
PKSKKKPQWRQVFTPGCDGSVTGSWLWQLAKAHILAHDAGVHQLVSHWLRTHACTEPYII
AANRQLSQMHPVYRLLHPHFRFTMEINAQARAMLINAGGIIEGSFVPGEYSLELSSVAYD
QQWRFDMEALPEDLIRRGMAVRNPNGELELAIEDYPYANDGLLVWDAIKQWALTYVQHYY
PCAADIVDDEELQAWWTEVRTKGHADKQDEPWWPELDSHENLAQTLATIMWVTSGHHAAV
NFGQYPMAGYIPNRPTMARRNMPTEIGGDDMRDFVEAPEKVLLDTFPSQYQSAIVLAILD
LLSTHSSDEEYMGTHEEPAWTKDGVINQAFEEFKESTRKIVEQVDEWNNDPDRKNRHGAG

MVPYVLLRPSDGDPTDGDPTDEKMVMEMGIPNSISI

>O20118

MENATVPNDQNEDYIDLTKPMSKSYWKKMKVKGFYRWVLVRDFKKINDILNTNEIVPILV
SGAKTFTKSRFSAMRWSLNEFAFVDGFFFLLLFFFYKKKKRSKISNTSKRKKRTKKKRTR
NLGNLCFLRKNLASILFF

>Q5SD32

MTRVKRGSVARKRRKNILKLASGFQGAHSTIFRTGNQQIIKALASSYRDRGKRKRDFRRL
WITRINAAARNNGVSYTKFIRHLYKNQVSSNRKVLAQIAIFDTNSFSTILKKLSSGESIR
YRNFSPEFYSGEVRTP

>Q32001

MSLTQQQKDLIFGSLLGDGNLQTGSVGRTWRYRALHKSEHQTYLFHKYEILKPLCGENTL
PTESIVFDERTNKEVKRWFFNTLTNPSLKFFADMFYTYDQNTQKWVKDVPVKVQTFLTPQ
ALAYFYIDDGALKWLNKSNAMQICTESFSQGGTIRIQKALKTLYNIDTTLTKKTLQDGRI
GYRIAIPEASSGAFREVIKPFLVDCMRYKVSDGNKGHL

>Q9S7B5

MASSCLFNASVSSLNPKQDPIRRHRSTSLLRHRPVVISCTADGNNIKAPIETAVKPPHRT
EDNIRDEARRNRSNAVNPFSAKYVPFNAAPGSTESYSLDEIVYRSRSGGLLDVEHDMEAL
KRFDGAYWRDLFDSRVGKSTWPYGSGVWSKKEWVLPEIDDDDIVSAFEGNSNLFWAERFG
KQFLGMNDLWVKHCGISHTGSFKDLGMTVLVSQVNRLRKMKRPVVGVGCASTGDTSAALS
AYCASAGIPSIVFLPANKISMAQLVQPIANGAFVLSIDTDFDGCMKLIREITAELPIYLA
NSLNSLRLEGQKTAAIEILQQFDWQVPDWVIVPGGNLGNIYAFYKGFKMCQELGLVDRIP
RMVCAQAANANPLYLHYKSGWKDFKPMTASTTFASAIQIGDPVSIDRAVYALKKCNGIVE
EATEEELMDAMAQADSTGMFICPHTGVALTALFKLRNQGVIAPTDRTVVVSTAHGLKFTQ
SKIDYHSNAIPDMACRFSNPPVDVKADFGAVMDVLKSYLGSNTLTS

>P24846

MPYLQPPRPHPHPHPTSRLSRASPPSPFPFFPAGTSRSGRLQPVPVSGHSASRVSKGKFA
VAAVTLDDYLPMRSTEVKNRTSTDGIKSLRLITAVKTPYLPDGRFDLEAYDSLINTQING
GAEGVIVGGTTGEGHLMSWDEHIMLIGHTVNCFGANIKVIGNTGSNSTREAVHATEQGFA
VGMHAALHVNPYYGKTSTEGLISHFKEVLPMGPTIIYNVPSRTSQDIPPPVIEALSSYSN
MAGVKECVGHERVKCYTDKGISIWSGNDDECHDSRWKYGATGVISVASNLVPGLMHSLMF
EGENAALNEKLLPLMKWLFCEPNPIGLNTALAQLGVVRPVFRLPYTPLPLEKRVEFVRIV
EAIGRENFVGQKESRVLDDDDFVLISRY

>Q9FY99

MAALSSSVTTRSYHSGYLASFSPVNGDRHRSLSFLSASPQGLNPLDLCVRFQRKSGRASV
FMQDGAIVTNSNSSESKTSLKGLKDEVLSALSQEAAKVGVESDGQSQSTVSITVVGASGD
LAKKKIFPALFALYYEGCLPEHFTIFGYSRSKMTDVELRNMVSKTLTCRIDKRANCGEKM
EEFLKRCFYHSGQYDSQEHFTELDKKLKEHEAGRISNRLFYLSIPPNIFVDAVKCASTSA
SSVNGWTRVIVEKPFGRDSETSAALTKSLKQYLEEDQIFRIDHYLGKELVENLSVLRFSN
LIFEPLWSRQYIRNVQFIFSEDFGTEGRGGYFDNYGIIRDIMQNHLLQILALFAMETPVS
LDAEDIRNEKVKVLRSMRPIRVEDVVIGQYKSHTKGGVTYPAYTDDKTVPKGSLTPTFAA
AALFIDNARWDGVPFLMKAGKALHTRSAEIRVQFRHVPGNLYNRNTGSDLDQATNELVIR
VQPDEAIYLKINNKVPGLGMRLDRSNLNLLYSARYSKEIPDAYERLLLDAIEGERRLFIR
SDELDAAWSLFTPLLKEIEEKKRIPEYYPYGSRGPVGAHYLAAKHKVQWGDVSIDQ

>Q43295

MIAAGAKSLLGLSMASPKGIFDSNSMSNSRSVVVVRACVSMDGSQTLSHNKNGSIPEVKS
INGHTGQKQGPLSTVGNSTNIKWHECSVEKVDRQRLLDQKGCVIWVTGLSGSGKSTLACA
LNQMLYQKGKLCYILDGDNVRHGLNRDLSFKAEDRAENIRRVGEVAKLFADAGIICIASL
ISPYRTDRDACRSLLPEGDFVEVFMDVPLSVCEARDPKGLYKLARAGKIKGFTGIDDPYE
PPLNCEISLGREGGTSPIEMAEKVVGYLDNKGYLQA

(4) $\mathbb{S}_4$: 182 cytoplasm proteins

>Q9SLX0
MSLRPSERAEVRRSRYKVAVDADEGRRRREDNMVEIRKSRREESLLKKRRDGLPAAAAAA
AAASPLLAHSSALQQKLEGLPAMVQAVQSDDSAVQLEATTQFRKLLSIERSPPIEEVINT
GVVPRFIAFLQREDYPQLQFEAAWALTNIASGTSDNTKVVVESGAVPIFVKLLSSPSEDV
REQAVWALGNVAGDSPKCRDLVLASGGLYPLLQQLNEHAKLSMLRNATWTLSNFCRGKPQ
PNFEQVKPALSALQRLIHSQDEEVLTDACWALSYLSDGTNDKIQAVIESGVFPRLVELLM
HPSASVLIPALRTVGNIVTGDDMQTQCVIDHQALPCLLNLLTNNHKKSIKKEACWTISNI
TAGNREQIQAVINANIIAPLVHLLQTAEFDIKKEAAWAISNATSGGTHDQIKYLVAQGCI
KPLCDLLVCPDPRIVTVCLEGLENILKVGEAEKNLGAGDVNSYAQMIDDAEGLEKIENLQ
SHDNTEIYEKAVKMLESYWLEEEDDAMPSGDNAQNGFNFGNQQPNVPSGGFNFG
>P25141
MSSNTAGQVIRCKAAVAWEAGKPLVIEEVEVAPPQKMEVRLKILFTSLCHTDVYFWEAKG
QTPLFPRIFGHEAGGIVESVGEGVTDLKPGDHVLPVFTGECQQCRHCKSEESNMCDLLRI
NTDRGVMIHDGQTRFSKDGKPIYHFVGTSTFSEYTVCHSGCVTKIDPQAPLDKVCVLSCG
ISTGLGATLNVAKPTKGSTVAIFGLGAVGLAAAEGARIAGASRIIGVDLNPSRFNDAKKF
GVTEFVNPKDHGDKPVQQVIAEMTDGGVDRSVECTGNVNAMISAFECVHDGWGVAVLVGV
PNKDDAFKTHPMNLLNERTLKGTFFGNYKPKSDIPSVVDKYMKKELELEKFITHQVPFSE
INKAFDYMLKGESIRCMITMEH
>P26301
MAVTITWVKARQIFDSRGNPTVEVDVGLSDGSYARGAVPSGASTGIYEALELRDGGSDYL
GKGVLKAVSNVNNIIGPAIVGKDPTEQVEIDNFMVQQLDGTSNEWGWCKQKLGANAILAV
SLAVCKAGAMVKKIPLYQHIANLAGNKTLVLPVPAFNVINGGSHAGNKLAMQEFMILPTG
ASSFKEAMKMGVEVYHNLKSIIKKKYGQDATNVGDEGGFAPNIQENKEGLELLKAAIEKA
GYTGKVVIGMDVAASEFFGEKDKTYDLNFKEENNDGSNKISGDSLKDLYKSFVSEYPIES
IEDPFDQDDWSTYAKLTDEIGQKVQIVGDDLLVTNPTRVAKAINEKTCNALLLKVNQIGS
VTESIEAVRMSKRAGWGVMASHRSGETEDTFIADLSVGLSTGQIKTGAPCRSERLAKYNQ
LLRIEEELGDAAVYAGAKFRAPVEPY
>P17990
MKILIILTILAMATTFATSEMQVNPSVQVQPTQQQPYPESQQPFISQSQQQFPQPQQPFP
QQPQQPFPQSQQQCLQQPQHQFPQPTQQFPQRPLLPFTHPFLTFPDQLLPQPPHQSFPQP
PQSYPQPPLQPFPQPPQQKYPEQPQQPFPWQQPTIQLYLQQQLNPCKEFLLQQCRPVSLL
SYIWSKIVQQSSCRVMQQQCCLQLAQIPEQYKCTAIDSIVHAIFMQQGQRQGVQIVQQQP
QPQQVGQCVLVQGQGVVQPQQLAQMEAIRTLVLQSVPSMCNFNVPPNCSTIKAPFVGVVT
GVGGQ
>P19595
MATATTLSPADAEKLNNLKSAVAGLNQISENEKSGFINLVGRYLSGEAQHIDWSKIQTPT
DEVVVPYDKLAPLSEDPAETKKLLDKLVVLKLNGGLGTTMGCTGPKSVIEVRNGLTFLDL

IVKQIEALNAKFGCSVPLLLMNSFNTHDDTLKIVEKYANSNIDIHTFNQSQYPRLVTEDF
APLPCKGNSGKDGWYPPGHGDVFPSLMNSGKLDALLAKGKEYVFVANSDNLGAIVDLKIL
NHLILNKNEYCMEVTPKTLADVKGGTLISYEGKVQLLEIAQVPDEHVNEFKSIEKFKIFN
TNNLWVNLSAIKRLVEADALKMEIIPNPKEVDGVKVLQLETAAGAAIKFFDRAIGANVPR
SRFLPVKATSDLLLVQSDLYTLTDEGYVIRNPARSNPSNPSIELGPEFKKVANFLGRFKS
IPSIIDLDSLKVTGDVWFGSGVTLKGKVTVAAKSGVKLEIPDGAVIANKDINGPEDI
>A2ZDX9
MEHQGQHGHVTSRVDEYGNPVGTGAGHGQMGTAGMGTHGTTGGMGTHGTTGGMGTHGTTG
TGGGQFQPMREEHKTGGVLQRSGSSSSSSSEDDGMGGRRKKGIKEKIKEKLPGGNKGEQQ
HAMGGTGGAYGQQGHGTGMTTGTTGAHGTTTTDTGEKKGIMDKIKEKLPGQH
>P16181
MAEQTEKAFLKQPKVFLSSKKSGKGKRPGKGGNRFWKNIGLGFKTPREAIDGAYVDKKCP
FTGTVSIRGRILAGTCHSAKMQRTIIVRRDYLHFVKKYQRYEKRHSNIPAHVSPCFRVKE
GDHIIIGQCRPLSKTVRFNVLKVIPAGSSSSFGKKAFTGM
>Q41261
MAAKVSSDPLVIGRVIGDVVDHFTSTVKMSVIYNSNNSIKHVYNGHELFPSAVTSTPRVE
VHGGDMRSFFTLIMTDPDVPGPSDPYLREHLHWIVTDIPGTTDSSFGKEVVSYEMPRPNI
GIHRFVFLLFKQKKRGQAMLSPPVVCRDGFNTRKFTQENELGLPVAAVFFNCQRETAARR
R
>O04016
MAAAAFVPIPTDTYKLGFIGAGKMAESIARGVVKSGVLPASRIRTAHLGSARREAFESFG
VKVLDRNDQVVEDSDVIIFSVKPQIVKEVVLQLRPLLSEKQLLVSIVAGVKLKELEDWAG
HSRFIRVMPNTPAAVGEAASVMSLGATATGEDGELITKLFGAIGKIWKADEKLFDAVTGL
SGSGPAYIFLAIEALADGGVAAGLPRELALGLASQTVLGAASMVARGGKHPGQLKDDVAS
AGGTTIAGIHELEKGGFRGTLMNAVVSATKRSQEIFKR
>P26307
MEASSGSSPPHSQENPPEHGGDMGGAPAEEIGGEAADDFMFAEDTFPSLPDFPCLSSPSS
STFSSNSSSNSSSAYTNTAGRAGGEPSEPASAGEGFDALDDIDQLLDFASLSMPWDSEPF
PGVSMMLENAMSAPPQPVGDGMSEEKAVPEGTTGGEEACMDASEGEELPRFFMEWLTSNR
ENISAEDLRGIRLRRSTIEAAAARLGGGRQGTMQLLKLILTWVQNHHLQRKRPRDVMEEE
AGLHVQLPSPVANPPGYEFPAGGQDMAAGGGTSWMPHQQAFTPPAAYGGDAVYPSAAGQQ
YSFHQGPSTSSVVVNSQPFSPPPVGDMHGANMAWPQQYVPFPPPGASTGSYPMPQPFSPG
FGGQYAGAGAGHLSVAPQRMAGVEASATKEARKKRMARQRRLSCLQQQRSQQLSLGQIQT
SVHLQEPSPRSTHSGPVTPSAGGWGFWSPSSQQQVQNPLSKSNSSRAPPSSLEAAAAAPQ
TKPAPAGARQDDIHHRLAAASDKRQGAKADKNLRFLLQKVLKQSDVGSLGRIVLPKKEAE
VHLPELKTRDGISIPMEDIGTSRVWNMRYRFWPNNKSRMYLLENTGEFVRSNELQEGDFI
VIYSDVKSGKYLIRGVKVRPPPAQEQGSGSSGGGKHRPLCPAGPERAAAAGAPEDAVVDG
VSGACKGRSPEGVRRVRQQGAGAMSQMAVSI
>P49608
MAAENPFKENLTSLPKPGGGEFGKYYSLPSLNDPRIDRLPYSIRILLESAIRNCDNFQVK
KEDVEKIIDWENSSPKQVEIPFKPARVLLQDFTGVPAVVDLACMRDAMNKLGSDSNKINP
LVPVDLVIDHSVQVDVARSENAVQANMELEFQRNKERFAFLKWGSNAFQNMLVVPPGSGI
VHQVNLEYLGRVVFNTSGLLYPDSVVGTDSHTTMIDGLGVAGWGVGGIEAEAAMLGQPMS
MVLPGVVGFKLSGKLRNGVTATDLVLTVTQMLRKHGVVGKFVEFYGDGMEELSLADRATI

ANMSPEYGATMGFFPVDHVTLQYLKLTGRSDETVSMIEAYLRANKMFVDYKEPQQEKVYS
SYLQLDLTDVEPCISGPKRPHDRVPLKEMKSDWHACLDNKVGFKGFAIPKEAQENVAKFS
FHGQPAELKHGSVVIAAITSCTNTSNPSVMLGAALVAKKACELGLQVKPWVKTSLAPGSG
VVTKYLLKSGLQPYLNQQGFHIVGYGCTTCIGNSGDLDESVSAAISDNDIVAAAVLSGNR
NFEGRVHPLTRANYLASPPLVVAYALAGTVDIDFEKEPIGKGKDGKDVYFRDIWPSTEEI
AEVVQSSVLPDMFKSTYESITKGNPMWNQLSVPSGTLYSWDPNSTYIHEPPYFKNMTMDP
PGAHGVKDAYCLLNFGDSITTDHISPAGSIHKDSPAAKYLLERGVDRKDFNSYGSRRGND
EVMARGTFANIRLVNKLLDGEVGPKTVHVPTGEKLSVFEAAEKYKSAGQDTIVLAGAEYG
SGSSRDWAAKGPMLLGVKAVIAKSFERIHRSNLVGMGIIPLCFKSGEDADSLGLTGHERY
TIDLPDDISKIRPGQDVTVTTDSGKSFTCTVRFDTEVELAYFNNGGILPYVIRNLIKQ
>O23826
MSNKEKGVNVQVLLRCRPFSNDELRNNAPQVVTCNDYQREVAVSQNIAGKHIDRIFTFDK
VFGPSAQQRDLYDQAIVPIVNEVLEGFNCTIFAYGQTGTGKTYTMEGECKRSKSGPNGEL
PQEAGVIPRAVKQVFDTLESQNAEYSVKVTFLELYNEEITDLLAPEDLKVALEDRQKKQL
PLMEDGKGGVLVRGLEEEIVTSANEIFTLLERGSAKRRTAETLLNKQSSRSHSLFSITIH
IKEATPEGEELIKCGKLNLVDLAGSENISRSGAREGRAREAGEINKSLLTLGRVINALVE
HLGHIPYRDSKLTRLLRDSLGGRTKTCIIATVSPAVHCLEETLSTLDYAHRAKNIKNKPE
VNQKMMKSTLIKDLYGEIERLKAEVYAAREKNGVYIPKERYYQEENERKAMADQIEQMGV
SIENHQKQFEELQSRHDSQVQQCSDLTCKLDVTQKQLNQTSKLLAYTEEQLRQSQYTLKE
RDFIISEQKKAENALAHQACVLRADLEKSIQENASLFQKIAREDKLSTDNRSLVNNFQAE
LAKQLGSLSSTLATSVCRQTEHLQCVEKFCHNFLDSHDKAVLDLKRKINSSMALYISHFE
AMQNVVRLHKATSNATLEEVSTLASSNSISTKEFLDAEAVEANSMFDELQSTLSTHQGEM
AHFARELRQRFNDSTEHLTNISAIIQRFFDKLLDESKRLEKHATTVDEIQTNSIAEFEKA
YEEQSKSDAEKLIADVTSLVSNHMRRQKELVGARLVDLRETVSGNRTFLDGHVSSMEGIT
TDAKRKWQDFYMQAEGETKENADFSAAKHCRMESLMQKCVSTAETALKRWQSTHELVNDM
GNQHVLTMHSVVRNICDNNEQHVTDFDSTRESAEEDVKRNSEDIIKSIDSLSGEERGSIS
GVLDTTSAHSETLDVLKKDHCMQSTSIEQIALETFQQKYMDYEPTGATPIRSEPDVPSKV
TIESLRAMPMEVLLEEFRENNSFESFQVKEVKPSLIPRSPFSQINN
>Q9SZF7
MDTLFRLVSLQQQQQSDSIITNQSSLSRTSTTTTGSPQTAYHYNFPQNDVVEECFNFFMD
EEDLSSSSSHHNHHNHNNPNTYYSPFTTPTQYHPATSSTPSSTAAAAALASPYSSSGHHN
DPSAFSIPQTPPSFDFSANAKWADSVLLEAARAFSDKDTARAQQILWTLNELSSPYGDTE
QKLASYFLQALFNRMTGSGERCYRTMVTAAATEKTCSFESTRKTVLKFQEVSPWATFGHV
AANGAILEAVDGEAKIHIVDISSTFCTQWPTLLEALATRSDDTPHLRLTTVVVANKFVND
QTASHRMMKEIGNRMEKFARLMGVPFKFNIIHHVGDLSEFDLNELDVKPDEVLAINCVGA
MHGIASRGSPRDAVISSFRRLRPRIVTVVEEEADLVGEEEGGFDDEFLRGFGECLRWFRV
CFESWEESFPRTSNERLMLERAAGRAIVDLVACEPSDSTERRETARKWSRRMRNSGFGAV
GYSDEVADDVRALLRRYKEGVWSMVQCPDAAGIFLCWRDQPVVWASAWRPT
>Q9SE83
MEAIDELSQLSDSMKQAASLLADEDPDETSSSKRPATFLNVVALGNVGAGKSAVLNSLIG
HPVLPTGENGATRAPIIIELSRESSLSSKAIILQIDNKSQQVSASALRHSLQDRLSKGAS
GKNRDEINLKLRTSTAPPLKLVDLPGLDQRIVDESMIAEYAQHNDAILLVIVPASQASEI
SSSRALKIAKEYDPESTRTIGIIGKIDQAAENSKALAAVQALLSNQGPPKTTDIPWVAVI
GQSVSIASAQSGSGENSLETAWRAESESLKSILTGAPQSKLGRIALVDTLASQIRSRMKL

RLPSVLSGLQGKSQIVQDELARLGEQLVNSAEGTRAIALELCREFEDKFLLHLAGGEGSG
WKVVASFEGNFPNRIKQLPLDRHFDLNNVKRVVLEADGYQPYLISPEKGLRSLIKIVLEL
AKDPARLCVDEVHRVLVDIVSASANATPGLGRYPPFKREVVAIASAALDGFKNEAKKMVV
ALVDMERAFVPPQHFIRLVQRRMERQRREEELKGRSSKKGQDAEQSLLSRATSPQPDGPT
AGGSLKSMKDKPSPQDKETPEVSGLKTAGPEGEITAGYLMKKSAKTNGWSRRWFVLNEKT
GKLGYTKKQEERNFRGTITLEECTIEEIPEDEVEKSKSSKDKKANGPDSKGPGLVFKITC
KVPYKTVLKAHNALVLKAESVVDKNEWINKLQKVIQARGGQVGSVSMRQSLSEGSLDKMV
RKPIDPEEELRWMSQEVRGYVEAVLNSLAANVPKAVVLCQVEKAKEDMLNQLYSSISAIG
NERIESLIQEDQNVKRRRERYQKQSSLLSKLTRQLSIHDNRAAAASSYSDNSGTESSPRA
SGGSSGDDWMNAFNSAANGPSDSLSKYGSGGHSRRYSDPAQNGDAASPGSGSNRRTTPNR
LPPAPPPTGSAYRY

>P24632

MDAVMFGLETPLMAALQHLLDVPDGDAGAGGDNKTGSGGSATRTYVRDARAMAATPADVK
ELPGAYAFVVDMPGLGTGDIRVQVEDERVLVVSGERRREEREDDAKYLRMERRMGKFMRK
FVLPDNADVDKVAAVCRDGVLTVTVEKLPPPEPKKPKTIEVKVA

>Q8GWZ6

METTVKNSSSDGEWKVVLPSKGRQGRRRKPKPKGQAEEEEQPWKSDDLEIDPQRQARLKQ
KMEISLKKIESSSFYTAFLEQLKSPEVSNQIRLVLGSETQLQMVMYGIGSIESYESPRFQ
LSIAILMKREFDWVGDNIEVFDPVLSATESSYLESLGCSVLSVNEQARREALKPTLFFMP
HCEANLYSNLLQANWRMDRLSKIALFGNSFQMYEEQVSFDAEVICATKRIIAAQRVTSEF
AIETESDDYFAAFHDSSWHFFSSGIDSELPLFVSD

>P28996

MVKFTIDQIRGLMEYQNNIRNMSVIAHVDHGKSTLTDSLVAAAGIIAFEQAGDQRLTDTR
ADEQERGITIKSTGISLYYQMTDEQLKGFTGERQGNDFLINLIDSPGHVDFSSEVTAALR
ITDGALVVVDCIEGVCVQTETVLRQALGERIRPVLTINKIDRCFLELMLDPEEAYLAYRR
VIENANVIMATYADEHLGDTQTHPEAGTVSFSAGLHGWAFTLTVFANMYAAKFGTDTKRM
MEKLWGDNFFDATTRKWTKKHTGADTCKRGFCQFIYEPIKTVIEAAMNDNKDKLFDLLKK
LNVYSKLKPEDRELMGKPLMKRVMQTWLPAHEALLEMMIWHLPSPAKAQKYRVDVLYEGP
LDDTYATAVRNCDADGPLMMYVSKMIPAADKGRFYAFGRVFSGRIATGRKVRIMGPNYVP
GQKKDLYVKTVQRTVLCMGRRQEAVEDVPCGNTVALVGLDQFITKNATLTDEKCEDAHTI
KAMKFSVSPVVRVAVEPKVASDLPKLVEGLKRLAKSDPMVQCTIEETGEHIIAGAGELHL
EICLKDLQDDFMGGAEIRVSEPVVSFRETVIGTSDHVVMSKSPNKHNRLYMQARPMEDGL
AEAIDEGKIGPRDDPKVRSKILSEEFGWDKELAKKILAFGPDTTGPNMVTDITKGVQYLN
EIKDSVVAAFQWASKEGVLAEENMRGIVFEVCDVVLHADAIHRGGGQIIPTARRSMYAAQ
LTAQPRLLEPVYLVEIQCPEQAMGGVYSVLNQKRGMVFEELQRPGTPIFNLKAYLPVIES
FGFTSTLRAATAGQAFPQCVFDHWEAMGSDPTQVGSQANTLVMDIRKRKGLKPEPAALSE
YEDKL

>Q94JU3

MDIEQKQAEIIDQLVKRASTCKSEALGPLIIEATSHPSLFAFSEILALPNVAQLEGTTDS
VYLDLLRLFAHGTWGDYKCNATRLPHLSPDQILKLKQLTVLTLAESNKVLPYDTLMVELD
VSNVRELEDFLINECMYAGIVRGKLDQLKRCFEVPFAAGRDLRPGQLGNMLHTLSNWLNT
SENLLISIQDKIKWADNMSEMDKKHRKEAEEGVEEVKKSLSMKGDVDIRGNKEMFGEPSG
VMDYEEDGIRPKRRRHPVTR

>P30986

MENKTPIFFSLSIFLSLLNCALGGNDLLSCLTFNGVRNHTVFSADSDSDFNRFLHLSIQN
PLFQNSLISKPSAIILPGSKEELSNTIRCIRKGSWTIRLRSGGHSYEGLSYTSDTPFILI
DLMNLNRVSIDLESETAWVESGSTLGELYYAITESSSKLGFTAGWCPTVGTGGHISGGGF
GMMSRKYGLAADNVVDAILIDANGAILDRQAMGEDVFWAIRGGGGGVWGAIYAWKIKLLP
VPEKVTVFRVTKNVAIDEATSLLHKWQFVAEELEEDFTLSVLGGADEKQVWLTMLGFHFG
LKTVAKSTFDLLFPELGLVEEDYLEMSWGESFAYLAGLETVSQLNNRFLKFDERAFKTKV
DLTKEPLPSKAFYGLLERLSKEPNGFIALNGFGGQMSKISSDFTPFPHRSGTRLMVEYIV
AWNQSEQKKKTEFLDWLEKVYEFMKPFVSKNPRLGYVNHIDLDLGGIDWGNKTVVNNAIE
ISRSWGESYFLSNYERLIRAKTLIDPNNVFNHPQSIPPMANFDYLEKTLGSDGGEVVI
>O23979
MSQEDSTSAAAAQQPTSRPAPKLNERILSSLSRRGGGAHPWHDLEIGPGAPAVFNVVVEI
TKGSKVKYELDKKTGLIKVDRVLYSSVVYPHNYGFIPRTLCEDNDPMDVLVLMQEPVIPG
SFLRARAIGLMPMIDQGEKDDKIIAVCADDPEYRHYSTSVSLLPRLQEIKRLEDYKKNEN
KEVAVDAFLPATTAREAIQYSMDLYAQYILQSLRQ
>P49972
MVLAELGGSISRALQQMSNATIIDEKVLNECLNEITRALLQADVQFKLVRDMTTNIKKIV
NLDDLAAGHNKRRIIQQAVFNELCKILDPGKPSFTPKKGKPSIVMFVGLQGSGKTTTCTK
YAYYHQKKGWKPALVCADTFRAGAFDQLKQNATKAKIPFYGSYTESDPVKIAVDGVETFK
KENCDLIIVDTSGRHKQEAALFEEMRQVAEATKPDLVIFVMDSSIGQAAFDQAQAFKQSV
AVGAVIVTKMDGHAKGGGALSAVAATKSPVIFIGTGEHMDEFEVFDVKPFVSRLLGMGDW
SGFMDKIHEVVPMDQQPELLQKLSEGHFTLRIMYEQFQNILKMGPIGQVFSMLPGFSAEL
MPKGRENESQAKIKRYMTMMDSMTNEELDSSNPKLMTDSRIMRIARGSGRQVHEVMDMME
EYKRLAKIWSKMKGLKIPKKGEMSALSRNMNAQHMSKVLPPQMLKQIGGMGGLQNLMKQM
GSAKDMMGGMGGMFGGGDK
>Q9M571
MAASAMGVLQEREVFKKYWIEHSVDLTVEAMMLDSQASDLDKVERPEVLSMLPPYEGKSV
LELGAGIGRFTGELAEKASQVIALDFIESVIKKNESINGHYKNVKFMCADVTSPSLNISP
NSVDIIFSNWLLMYLSDEEVERLVERMLKWLKPGGYIFFRESCFHQSGDHKRKSNPTHYR
EPRFYTKIFKECHMQDDSGNSYELSLIGCKCIGAYVKSKKNQNQISWLWQKVDSEDDKGF
QRFLDSSQYKFNSILRYERVFGPGYVSTGGLETTKEFVSKLDLKPGQKVLDVGCGIGGGD
FYMAENYDVEVVGIDLSINMISFALERSIGLKCAVEFEVADCTKKDYPENSFDVIYSRDT
ILHIQDKPALFRSFHKWLKPGGKVLISDYCKSAGTPSAEFAAYIRQRGYDLHDVKAYGKM
LKDAGFVEVIAENRTDQFIQVLQKELDALEQEKDDFIDDFSEEDYNDIVDGWKAKLVRTT
EGEQQWGLFIAKKM
>Q00834
MVEEKAFIAKDVTELIGKTPLVYLNTVADGCVARVAAKLEGMEPCSSVKDRIGFSMITDA
EKSGLITPGESVLIEPTSGNTGIGLAFIAAAKGYKLIITMPASMSLERRTILRAFGAELI
LTDPAKGMKGAVQKAEEIRDKTPNSYILQQFENPANPKVHYETTGPEIWKGTGGKIDIFV
SGIGTGGTITGAGKYLKEQNPDVKLIGLEPVESAVLSGGKPGPHKIQGLGAGFIPGVLDV
NIIDEVVQISSEESIEMAKLLALKEGLLVGISSGAAAAAAIKVAKRPENAGKLIVAVFPS
FGERYLSSVLFDSVRKEAESMVIES
>Q9T041
MSTPTTSGSAAKPTRPARSSSLATRSCSNSGSLTSFQAMVELKQKILTSISKLADRDTYQ
IAVEDLEKTIQSLTPETLPMFLNCLYDSCSDPKPAVKKECLHLLSYVCSLHCDSTAAHLT

KIIAQIVKRLKDSDSGVRDACRDTIGALSGIYLKGKEEGTNTGSASLAVGLFVKPLFEAM
GEQNKVVQSGASMCMARMVESAASPPVTSFQKLCPRICKLLSNSSFLAKASLLPVVSSLS
QVGAIAPQSLESLLESIHDCLGSTDWVTRKAAAETLTALASHSSGLIKEKTDSTITVLET
CRFDKIKPVRESVTEALQLWKKISGKYVDGASDDSKLSASEQLGSEKNGEKRSNLADLMK
KEASDGSTLSPDSASKGKGCFPEKAVGLLKKKAPVLSDKDFNPEFFQRLERRQSVEVVVP
RRCKNNDEEESGLDDLNAMGSSNRLKNTQADDKQVKGRFDGNGSQARTSGDDKAGVVNGK
ETPGHHAPVSNTDNQSEGSFTSNRGNWSAIQRQLLQLERQQTNLMNMLQEFIGGSHDSMV
TLEGRVRGLERIVEDMARDLSISSGRRANLTAGFGKYNSFANYPTGKYNGRAPGERGSQT
DGAMRGRMWNSDMADDWFIPPHAASRNGQAGPRRSPRSEQYENEHMGNGRRGWDNKASGT
IRFGEGPSARSVWQASKDEATLEAIRVAGEDGAVPRPTRVAVAPEAEAMGDDDNEGQERD
PIWVSWSNAMHSLRVGDIDAAYAEVLCAGDQHLVIKLMDKTGPSLDQMSNEIANEALNFI
SQFLLDHSLYDICLSWSQQLLELVLQDGADTFGVPMELKTEILYNLQDACSTMDPPEDWE
GPAPEQLVVQLASVWEIDLQQFDK
>Q9FV49
MAIGNPEVATMGKENTEAESSNGNESQLSSDLTKSLDLAEVKEDEKDNNQEEEDGLKAEA
STKKKKKKSKSKKKKSSLQQTDPPSIPVLELFPSGDFPQGEIQQYNDDNLWRTTSEEKRE
MERLQKPIYNSLRQAAEVHRQVRKYMRSILKPGMLMIDLCETLENTVRKLISENGLQAGI
AFPTGCSLNNVAAHWTPNSGDKTVLQYDDVMKLDFGTHIDGHIVDSAFTVAFNPMFDPLL
AASRDATYTGIKEAGVDVRLCDVGAAVQEVMESYEVEINGKVYQVKSIRNLNGHSIGRYQ
IHAEKSVPNVRGGEQTKMEEGELYAIETFGSTGKGYVREDLECSHYMKNYDVGHVPLRLP
RAKQLLATINKNFSTLAFCRRYLDRLGETKYLMALKNLCDSGIIEPCPPVCDVKGSYISQ
FEHTILLRPTCKEIISKGDDY
>Q944C1
MEGKKEMGSYKFCLIFTRKFRMTESGPVEDVRDLFEKYTEGDAHMSPEQLQKLMTEEGGE
GETSLEEAERIVDEVLRRKHHIAKFTRRNLTLDDFNYYLFSTDLNPPIADQVHQNMDAPL
SHYFIFTGHNSYLTGNQLSSNCSELPIADALRRGVRVVELDLWPRGTDDVCVKHGRTLTK
EVKLGKCLESIKANAFAISKYPVIITLEDHLTPKLQFKVAKMITQTFGDMLYYHDSQGCQ
EFPSPEELKEKILISTKPPKEYLEANDTKEKDNGEKGKDSDEDVWGKEPEDLISTQSDLD
KVTSSVNDLNQDDEERGSCESDTSCQLQAPEYKRLIAIHAGKPKGGLRMALKVDPNKIRR
LSLSEQLLEKAVASYGADVIRFTQKNFLRIYPKGTRFNSSNYKPQIGWMSGAQMIAFNMQ
GYGRALWLMEGMFRANGGCGYVKKPDFLMDASPNGQDFYPKDNSSPKKTLKVKVCMGDGW
LLDFKKTHFDSYSPPDFFVRVGIAGAPVDEVMEKTKIEYDTWTPIWNKEFTFPLAVPELA
LLRVEVHEHDVNEKDDFGGQTCLPVSEIRQGIRAVPLFNRKGVKYSSTRLLMRFEFV
>O24496
MKIFHVPCLQDNYSYLIIDESTGDAAVVDPVDPEKVIASAEKHQAKIKFVLTTHHHWDHA
GGNEKIKQLVPDIKVYGGSLDKVKGCTDAVDNGDKLTLGQDINILALHTPCHTKGHISYY
VNGKEGENPAVFTGDTLFVAGCGKFFEGTAEQMYQSLCVTLAALPKPTQVYCGHEYTVKN
LEFALTVEPNNGKIQQKLAWARQQRQADLPTIPSTLEEELETNPFMRVDKPEIQEKLGCK
SPIDTMREVRNKKDQWRG
>Q9ZSY2
MSAKKLEGSSAPANRRDPYEVLCVSKDANDQEIKSAYRKLALKYHPDKNANNPDASELFK
EVAFSYSILSDPEKRRHYDNAGFEALDADGMDMEIDLSNLGTVNTMFAALFSKLGVPIKT
TVSANVLEEAMNGTVTVRPLPIGTSVSGKVEKQCAHFFGVTISEQQAESGVVVRVTSTAQ
SKFKLLYFEQDSSGGYGLALQEEREKTGKVTSAGMYFLHFQVYRMDTTVNALAAAKDPES

AFFKRLEGLQPCEVSELKAGTHIFAVYGDNFFKTASYTIEALCAKTYEDTTEKLKEIEAQ
ILRKRNELRQFETEYRKALARFQEVTNRYTQEKQTVDELLKQRDTIHSTFSVVKTPSGNN
LSNGSSSKAQGDESKGDGDSAGEEGGTENRDKSKRKWFNLNLKGSDKKLG
>Q9LK23
MGSGQWHMEKRSTLKNDSFVKEYNPVTETGSLSIIVLGASGDLAKKKTFPALFNLFHQGF
LNPDEVHIFGYARSKITDEELRDKIRGYLVDEKNASKKTEALSKFLKLIKYVSGPYDSEE
GFKRLDKAILEHEISKKTAEGSSRRLFYLALPPSVYPPVSKMIKAWCTNKSDLGGWTRIV
VEKPFGKDLESAEQLSSQIGALFEEPQIYRIDHYLGKELVQNMLVLRFANRLFLPLWNRD
NIANVQIVFREDFGTEGRGGYFDEYGIIRDIIQNHLLQVLCLVAMEKPISLKPEHIRDEK
VKVLQSVIPIKDEEVVLGQYEGYRDDPTVPNDSNTPTFATTILRINNERWEGVPFILKAG
KAMSSKKADIRIQFKDVPGDIFKCQNQGRNEFVIRLQPSEAMYMKLTVKQPGLEMQTVQS
ELDLSYKQRYQDVSIPEAYERLILDTIRGDQQHFVRRDELKAAWEIFTPLLHRIDKGEVK
SVPYKQGSRGPAEADQLLKKAGYMQTHGYIWIPPTL
>P20075
MASRKDERAAKEERAQAAAELAAKELRDVNQDRERGIKVVEHKEEVSGGPGVIGSILKSV
QGTLGQAKEVVVGKAHDTAEVSRENTDYAYDKGREGGDVAAQKAEEAKEKAKMAKDTTMG
KAGEYKDYTAQKAEEAKEKAAQKAEETKEKAGEYKNYTAQKAGEAKDTTLGKAGEYKDYA
AQKAAEAKDTTAQKAAEAKEKTGEYKDYAAQKAAEAKVLAAQKAAEAKDTTGKDGEYKDY
AAQKAAEAKDATMQKTGEYKDYAAQKTAETKDATMEKAKEYKEYAAQKAAEAKDATMQKT
GEYKDYSAQKAAETKDATMEKTKEYKDYTAQKAAETKDATMEKAKEAKDTTVQKTGEYKD
YAAEKAKEGKDVTVEKAKEGKDTTVGKMTELKDSAADAARKAMDMFLGKKEEVKGKAGET
AEAAKEKYEDTEFAARKKMEELKLQEEGVKDEAKQRAEADRETAGDRGSAAKGTIFGAMG
SVKDAIVGKLTMPSDVVKDKQQQEAVIKVDETRPGAVAEALKAADQMHGQAFNDVGKMGD
EEVIVERKETRQGKM
>Q8LDP4
MAKASFILLGTLFLFGAIASIQAKEDLKEVTHKVYFDVEIDGKSAGRVVIGLFGKAVPKT
AENFRALCTGEKGVGKSGKPLHYKGSKFHRIIPSFMIQGGDFTHGNGMGGESIYGQKFAD
ENFKLKHTGPGVLSMANSGEDTNGSQFFITTVTTSWLDGRHVVFGKVVQGMDVVYKIEAE
GKQSGTPKSKVVIADSGELPL
>Q2QYF3
MGSSSLLLFPSSSSSATHSSYSPSSSSHAITSLLPPLPSDHHLLLYLDHQEQHHLAAAMV
RKRPASDMDLPPPRRHVTGDLSDVTAAAAGAPTLSASAQLPALPTQLPAFHHTDMDLAAP
APPAPQQVAAGEGGPPSTAWVDGIIRDIIASSGAAVSVAQLIHNVREIIRPCNPDLASIL
ELRLRSLLNSDPAPPPPPPPSHPALLPPDATAPPPPPTSVAALPPPPPAQPDKRRREPQCQ
EQEPNQPQSPKPPTAEETAAAAAAAAAAAAAAAKERKEEQRRKQRDEEGLHLLTLLLQCA
ESVNADNLDEAHRALLEIAELATPFGTSTQRVAAYFAEAMSARLVSSCLGLYAPLPSPSP
AGARVHGRVAAAFQVFNGISPFVKFSHFTANQAIQEAFEREERVHIIDLDIMQGLQWPGL
FHILASRPGGPPRVRLTGLGASMEALEATGKRLSDFADTLGLPFEFCPVADKAGNLDPEK
LGVTRREAVAVHWLRHSLYDVTGSDSNTLWLIQRLAPKVVTMVEQDLSHSGSFLARFVEA
IHYYSALFDSLDASYSEDSPERHVVEQQLLSREIRNVLAVGGPARTGDVKFGSWREKLAQ
SGFRVSSLAGSAAAQAALLLGMFPSDGYTLIEENGALKLGWKDLCLLTASAWRPIQASGR

>Q9LEK8
MSSEKVRASHILIKHQGSRRKSSWKDPDGSLISATTRDDAVSQLQSLRQELLSDPASFSD

LASRHSHCSSAKRGGDLGPFGRGQMQKPFEEATFALKVGEISDIVDTDSGVHIIKRTG
>Q8W5S1
MADFQTSTQRAKWIFTPQKLAERYKAANQRAVQMLEKCGTTQVEVDASGSLTYPKDKVGS
GDQADKKLKPLSADEERFMRAFYEAKVQEVCSAFAFPHKIQATALQYFKRFYLQWSVMQH
HPKEIMLTCVYAACKIEENHVSAEEIGKGINQDHRIILKYEMAVLQSLEFDLIVYAPYRA
IEGFVNNMEEFLQARDDEIQKLESLLKGATAEADKVMLTDAPLLFPPGQLALASLRIANG
VLGVIDFDRYLENIVSQPNSEHTTSELTKLLDNIEYLVKNYKCPSEKDMKHINRKLKSCL
GHSSSHDESKKREKRSKHKSHRSSNDTPNGAPPPIG
>P42791
MGIDLIAGGKSKKTKRTAPKSDDVYLKLTVKLYRFLVRRTNSKFNGVILKRLFMSKVNKA
PLSLSRLVEFMTGKEDKIAVLVGTITDDLRVHEIPAMKVTALRFTERARARIEKAGGECL
TFDQLALRAPLGQNTVLLRGPKNSREAVKHFGPAPGVPHSHSKPYVRAKGRKFEKARGKR
KSRGFKV
>Q39214
MASATVDFGIGRILSVLENETLLLSGVHGEIDKMKKELLIMKSFLEDTHKHGGNGSTTTT
TQLFQTFVANTRDLAYQIEDILDEFGYHIHGYRSCAKIWRAFHFPRYMWARHSIAQKLGM
VNVMIQSISDSMKRYYHSENYQAALLPPIDDGDAKWVNNISESSLFFSENSLVGIDAPKG
KLIGRLLSPEPQRIVVAVVGMGGSGKTTLSANIFKSQSVRRHFESYAWVTISKSYVIEDV
FRTMIKEFYKEADTQIPAELYSLGYRELVEKLVEYLQSKRYIVVLDDVWTTGLWREISIA
LPDGIYGSRVMMTTRDMNVASFPYGIGSTKHEIELLKEDEAWVLFSNKAFPASLEQCRTQ
NLEPIARKLVERCQGLPLAIASLGSMMSTKKFESEWKKVYSTLNWELNNNHELKIVRSIM
FLSFNDLPYPLKRCFLYCSLFPVNYRMKRKRLIRMWMAQRFVEPIRGVKAEEVADSYLNE
LVYRNMLQVILWNPFGRPKAFKMHDVIWEIALSVSKLERFCDVYNDDSDGDDAAETMENY
GSRHLCIQKEMTPDSIRATNLHSLLVCSSAKHKMELLPSLNLLRALDLEDSSISKLPDCL
VTMFNLKYLNLSKTQVKELPKNFHKLVNLETLNTKHSKIEELPLGMWKLKKLRYLITFRR
NEGHDSNWNYVLGTRVVPKIWQLKDLQVMDCFNAEDELIKNLGCMTQLTRISLVMVRREH
GRDLCDSLNKIKRIRFLSLTSIDEEEPLEIDDLIATASIEKLFLAGKLERVPSWFNTLQN
LTYLGLRGSQLQENAILSIQTLPRLVWLSFYNAYMGPRLRFAQGFQNLKILEIVQMKHLT
EVVIEDGAMFELQKLYVRACRGLEYVPRGIENLINLQELHLIHVSNQLVERIRGEGSVDR
SRVKHIPAIKHYFRTDNGSFYVSLSS
>P41127
MKHNNVIPNGHFKKHWQNYVKTWFNQPARKTRRRIARQKKAVKIFPRPTSGPLRPVVHGQ
TLKYNMKVRTGKGFTLEELKAAGIPKKLAPTIGIAVDHRRKNRSLEGLQTNVQRLKTYKT
KLVIFPRRARKVKAGDSTPEELANATQVQGDYLPIVREKPTMELVKLTSEMKSFKAFDKI
RLERTNKRHAGARAKRAAEAEKEEKK
>Q9XG54
MENKVVEEKQVDKIPLMSPCKMGKFELCHRVVLAPLTRQRSYGYIPQPHAILHYSQRSTN
GGLLIGEATVISETGIGYKDVPGIWTKEQVEAWKPIVDAVHAKGGIFFCQIWHVGRVSNK
DFQPNGEDPISCTDRGLTPQIRSNGIDIAHFTRPRRLTTDEIPQIVNEFRVAARNAIEAG
FDGVEIHGAHGYLIDQFMKDQVNDRSDKYGGSLENRCRFALEIVEAVANEIGSDRVGIRI
SPFAHYNEAGDTNPTALGLYMVESLNKYDLAYCHVVEPRMKTAWEKIECTESLVPMRKAY
KGTFIVAGGYDREDGNRALIEDRADLVAYGRLFISNPDLPKRFELNAPLNKYNRDTFYTS
DPIVGYTDYPFLETMT
>P37392

MREILHIQGGQCGNQIGAKFWEVVCAEHGIDSTGRYGGDNELQLERVNVYYNEASCGRFV
PRAVLMDLEPGTMDSVRSGPYGQIFRPDNFVFGQSGAGNNWAKGHYTEGAELIDSVLDVV
RKEAENCDCLQGFQVCHSLGGGTGSGMGTLLISKIREEYPDRMMLTFSVFPSPKVSDTVV
EPYNATLSVHQLVENADECMVLDNEALYDICFRTLKLTTPSFGDLNHLISATMSGVTCCL
RFPGQLNSDLRKLAVNLIPFPRLHFFMVGFAPLTSRGSQQYRALTVPELTQQMWDAKNMM
CAADPRHGRYLTASAMFRGKMSTKEVDEQMINVQNKNSSYFVEWIPNNVKSTVCDIPPTG
LKMASTFIGNSTSIQEMFRRVSEQFTAMFRRKAFLHWYTGEGMDEMEFTEAESNMNDLVS
EYQQYQDATADEDGYEYEDEEEIGEEA
>Q8GYN5
MARSNVPKFGNWEAEENVPYTAYFDKARKTRAPGSKIMNPNDPEYNSDSQSQAPPHPPSS
RTKPEQVDTVRRSREHMRSREESELKQFGDAGGSSNEAANKRQGRASQNNSYDNKSPLHK
NSYDGTGKSRPKPTNLRADESPEKVTVVPKFGDWDENNPSSADGYTHIFNKVREERSSGA
NVSGSSRTPTHQSSRNPNNTSSCCCFGFGGK
>P19825
MAHQHTISGLVGKLITESEVNCNADKYYQIFKHHEDLPSAIPHIYTSVKAVEGHGTTSGC
VKEWCYILEGKPLTVKEKTTYNDETRTINHNGIEGGMMNDYKKFVATLVVKPKANGQGSI
VTWIVDYEKINEDSPVPFDYLAFFQQNIEDLNSHLCASD
>P08688
MTKTGYINAAFRSSQNNEAYLFINDKYVLLDYAPGTSNDKVLYGPTPVRDGFKSLNQTVF
GSYGVDCSFDTDNDEAFIFYEKFCALIDYAPHSNKDKIILGPKKIADMFPFFEGTVFENG
IDAAYRSTRGKEVYLFKGDQYARIDYETNSMVNKEIKSIRNGFPCFRNTIFESGTDAAFA
SHKTNEVYFFKGDYYARVTVTPGATDDQIMDGVRKTLDYWPSLRGIIPLEN
>Q9SWI1
MVTLTPSSASTPKTSFDFMKNNNSHSSLYVSSSSYLSSKEDALVTTKKLMEPSKTLNMSI
NPKQEEFGDEKKMVKKAPEDPEIGVFGAEKYFNGDMDSDQGSSVLSLTNPEVERTVVDSK
QSAKKSTGTPSVRSESSWNSQSVLLQNKLVNSCNSSFKEKKNSNGQIQKVTNNKKSFLAN
LGCKCACSDGDSVDVEEKTSVKRSADPNISVITMRSSADMNTELIKIQKQEELSQRKSLE
VFGSPVAIEKKSSVVQKKLPLPPWKSRTEEDDTKSEGSDSSSDLFEIEGLTGNPKPFLTR
QGSDPASPTCYAPSEVSVEWSIVTASAADFSVMSECATSPVRRNRPTQIPRIPITAKSAP
QRRKSSSSSGGNGFLMSCKSHKSVMVSGDLDRRSSMNKTQPSYVPRFPMETTKPKSFETR
RRISNSSISHTQSSLLYSQ
>Q84M92
MYGGDEVSAIVVDLGSHTCKAGYAGEDAPKAVFPSVIGAVDGVEAMDVDVDSTKTNSNSE
DSKTESEKEKSKRKLYVGSQAMSYRRDHMEVLSPIKDGIVSDWDLVDNIWEHAFKSCLMI
DPTEHPMLLAEPPLNTQQQREKAAELMFEKYKVPALFMAKNPVLTSFATGRATSLVVDCG
GGSTTISPVHDGYVLQKAVVSSPLGGEFLTDCLLKSLESKGIKIRPRYSFKRKEVRAGEF
QVEDVDIPDTTESYKLFCQRMIVGDIKDSICRVPDTPYDDKSYSNIPTTSYELPDGQTLE
IGADRFKVPDVMFNPSIVQTIPGMEKYAEMIPSVRGLPHMVMESINKCDVDIRRELYSSI
LLAGGTSSMQQLKERLEKDLIEESPHSARVKVLASGNTTERRFSVWIGGSILASLGSFQQ
MWFSKSEYEEHGASYIQRKCP
>Q56WK6
MAQEEVQKSADVAAAPVVKEKPITDKEVTIPTPVAEKEEVAAPVSDEKAVPEKEVTPEKE
APAAEAEKSVSVKEEETVVVAEKVVVLTAEEVQKKALEEFKELVREALNKREFTAPVTPV
KEEKTEEKKTEEETKEEEKTEEKKEETTTEVKVEEEKPAVPAAEEEKSSEAAPVETKSEE

KPEEKAEVTTEKASSAEEDGTKTVEAIEESIVSVSPPESAVAPVVVETVAVAEAEPVEPE
EVSIWGVPLLQDERSDVILTKFLRARDFKVKEALTMLKNTVQWRKENKIDELVESGEEVS
EFEKMVFAHGVDKEGHVVIYSSYGEFQNKELFSDKEKLNKFLSWRIQLQEKCVRAIDFSN
PEAKSSFVFVSDFRNAPGLGKRALWQFIRRAVKQFEDNYPEFAAKELFINVPWWYIPYYK
TFGSIITSPRTRSKMVLAGPSKSADTIFKYIAPEQVPVKYGGLSKDTPLTEETITEAIVK
PAANYTIELPASEACTLSWELRVLGADVSYGAQFEPTTEGSYAVIVSKTRKIGSTDEPVI
TDSFKVGEPGKIVITIDNQTSKKKKVLYRFKTQ

>P42798

MVRISVLNDALKSMYNAEKRGKRQVMIRPSSKVIIKFLIVMQKHGYIGEFEYVDDHRSGK
IVVELNGRLNKCGVISPRFDVGVKEIEGWTARLLPSRQFGYIVLTTSAGIMDHEEARRKN
VGGKVLGFFY

>Q40987

MAFYRTNLPTRELFSLVSVVIVLLATNINSVQALSFNFTKLTTANSGVTFQGDAQILPSG
LIALTKSSPFPPGQYFTTVGRALSSNLVPLWDSATGKAASFVTSFSFVIDTTEGPITDGL
IFFIAPPGTVIPQNSTTPFLGVVDSETSINRFVGLEFDLYRNSWDPEGRHIGIDINSIIS
TKTVTYNLVSGSLTKVIIIYDSPSSTLSAAIIYENGKISTISQVIDLKTVLPNTVQIGLS
AATLTGESYSIHSWSFVSDLETTASYVSNI

>P48534

MGKSYPTVSPDYQKAIEKAKRKLRGFIAEKKCAPLILRLAWHSAGTFDSKTKTGGPFGTI
KHQAELAHGANNGLDIAVRLLEPIKEQFPIVSYADFYQLAGVVAVEITGGPEVPFHPGRE
DKPEPPPEGRLPDATKGSDHLRDVFGKAMGLSDQDIVALSGGHTIGAAHKERSGFEGPWT
SNPLIFDNSYFTELLTGEKDGLLQLPSDKALLTDSVFRPLVEKYAADEDVFFADYAEAHL
KLSELGFAEA

>O23627

MRIFSTFVFHRRQQIFNLRQFQTTTILRNPISIAPIQIPMDATEQSLRQSLSEKSSSVEA
QGNAVRALKASRAAKPEIDAAIEQLNKLKLEKSTVEKELQSIISSSGNGSLNREAFRKAV
VNTLERRLFYIPSFKIYSGVAGLFDYGPPGCAIKSNVLSFWRQHFILEENMLEVDCPCVT
PEVVLKASGHVDKFTDLMVKDEKTGTCYRADHLLKDYCTEKLEKDLTISAEKAAELKDVL
AVMEDFSPEQLGAKIREYGITAPDTKNPLSDPYPFNLMFQTSIGPSGLIPGYMRPETAQG
IFVNFKDLYYYNGKKLPFAAAQIGQAFRNEISPRQGLLRVREFTLAEIEHFVDPENKSHP
KFSDVAKLEFLMFPREEQMSGQSAKKLCLGEAVAKGTVNNETLGYFIGRVYLFLTRLGID
KERLRFRQHLANEMAHYAADCWDAEIESSYGWIECVGIADRSAYDLRAHSDKSGTPLVAE
EKFAEPKEVEKLVITPVKKELGLAFKGNQKNVVESLEAMNEEEAMEMKATLESKGEVEFY
VCTLKKSVNIKKNMVSISKEKKKEHQRVFTPSVIEPSFGIGRIIYCLYEHCFSTRPSKAG
DEQLNLFRFPPLVAPIKCTVFPLVQNQQFEEVAKVISKELASVGISHKIDITGTSIGKRY
ARTDELGVPFAITVDSDTSVTIRERDSKDQVRVTLKEAASVVSSVSEGKMTWQDVWATFP
HHSSAAADE

>Q9T052

MAYHPVYNETMSMGGGSSNEFGQWLDKQLVPFDTSSGSLRVELLHGNLDIWVKEAKHLPN
MDGFHNTLVGGMFFGLGRRNHKVDGENSSKITSDPYVTVSISGAVIGRTFVISNSENPVW
MQHFDVPVAHSAAKVHFVVKDSDIIGSQIIGAVEIPTEQLCSGNRIEGLFPILNSRGKPC
KQGAVLSLSIQYIPMERMRLYQKGVGFGVECVGVPGTYFPLRKGGRVTLYQDAHVDDGTL
PSVHLDGGIQYRHGKCWEDMADAIRRARRLIYITGWSVFHPVRLVRRNNDPTQGTLGELL
KVKSQEGVRVLVLVWDDPTSRSLLGFSTKGLMNTSDEETRRFFKHSSVQVLLCPRYGGKG

HSFIKKSEVETIYTHHQKTMIVDAEAAQNRRKIVAFVGGLDLCNGRFDTPKHPLFRTLKT
IHKDDFHNPNFVTTADDGPREPWHDLHSKIDGPAAYDVLANFEERWMKASKPRGIGRLRT
SSDDSLLRLDRIPDIMGLSEASSANDNDPESWHVQVFRSIDSSSVKGFPKDPKEATGRNL
LCGKNILIDMSIHAAYVKAIRSAQHFIYIENQYFLGSSFNWDSNKNLGANNLIPMEIALK
IANKIRAREKFAAYIVIPMWPEGAPTSNPIQRILYWQHKTMQMMYQTIYKALVEVGLDGQ
LEPQDFLNFFCLGTREVGTREVPDGTVSVYNSPRKPPQLNAAQVQALKSRRFMIYVHSKG
MVVDDEFVLIGSANINQRSLEGTRDTEIAMGGYQPHHSWAKKGSRPRGQIFGYRMSLWAE
HLGFLEQEFEEPENMECVRRVRQLSELNWRQYAAEEVTEMPGHLLKYPVQVDRTGKVSSL
PGYETFPDLGGKIIGSFLVVEENLTI
>Q38707
MAKSSEIEHPVKAFGWAARDTTGLLSPFKFSRRATGEKDVRLKVLFCGVCHSDHHMIHNN
WGFTTYPIVPGHEIVGVVTEVGSKVEKVKVGDNVGIGCLVGSCRSCESCCDNRESHCENT
IDTYGSIYFDGTMTHGGYSDTMVADEHFILRWPKNLPLDSGAPLLCAGITTYSPLKYYGL
DKPGTKIGVVGLGGLGHVAVKMAKAFGAQVTVIDISESKRKEALEKLGADSFLLNSDQEQ
MKGARSSLDGIIDTVPVNHPLAPLFDLLKPNGKLVMVGAPEKPFELPVFSLLKGRKLLGG
TINGGIKETQEMLDFAAKHNITADVEVIPMDYVNTAMERLVKSDVRYRFVIDIANTMRTE
ESLGA
>Q8VXU6
MDSNWINCPSVFSSSSSSSRRCQSRSDLYLGGGYEDLEGEDDLKAEFICPFCAEDFDIVG
LCCHIDEEHPVEAKNGVCPVCTKRVGLDIVGHITTQHANFFKVQRRRRLRRGGYSSTYLA
LKKELREANLQSLLGGSSSFTSSTNIDSDPLLSSFMFNSPSVNQSANKSATPVTVGNAAT
KVSIKESLKRDIQEAPLSGEDQEKAKKSEFVRGLLLSTMLEDDF
>P12783
MATKRSVGTLGEADLKGKKVFVRADLNVPLDDAQKITDDTRIRASIPTIKYLLEKGAKVI
LASHLGRPKGVTPKFSLKPLVARLSELLGLEVVMAPDCIGEEVEKLAAALPDGGVLLLEN
VRFYKEEEKNDPEFAKKLASVADLYVNDAFGTAHRAHASTEGVTKFLRPSVAGFLMQKEL
DYLVGAVANPKKPFAAIVGGSKVSSKIGVIESLLAKVDILILGGGMIFTFYKAQGLAVGK
SLVEEDKLELATSLIETAKSKGVKLLLPTDVVVADKFAADAESKIVPATAIPDGWMGLDV
GPDSIKTFAEALDTTKTVIWNGPMGVFEFEKFAAGTDAIAKQLAELTGKGVTTIIGGGDS
VAAVEKAGLADKMSHISTGGGASLELLEGKPLPGVLALDEA
>Q9FVL0
MGTLDTKGFTEEQEALVVKSWNAMKKNSAELGLKLFLKIFEIAPSAQKLFSFLKDSKVPL
EQNTKLKPHAMSVFLMTCESAVQLRKSGKVTVRESSLKKLGANHFKYGVVDEHFEVTKFA
LLETIKEAVPEMWSPAMKNAWGEAYDQLVNAIKSEMKPSS
>P27058
MGTPSYDIKNKGDDMQEEPKVKLHHEKGGDEKEKIIEKETPSQDINNKDTISSYVLRDDT
QEIPKMEHEEGGYVKEKIVEKETISQYIIKIEGDDDAQEKLKVEYEEEEYEKEKIVEKET
PSQDINNKGDDAQEKPKVEHEEGDDKETPSQDIIKMEGEGALEITKVVCEKIIVREDLAV
QSKPPSKRDPPKMQTDNNKL
>O48963
MEPTEKPSTKPSSRTLPRDTRGSLEVFNPSTQLTRPDNPVFRPEPPAWQNLSDPRGTSPQ
PRPQQEPAPSNPVRSDQEIAVTTSWMALKDPSPETISKKTITAEKPQKSAVAAEQRAAEW
GLVLKTDTKTGKPQGVGVRNSGGTENDPNGKKTTSQRNSQNSCRSSGEMSDGDVPGGRSG
IPRVSEDLKDALSTFQQTFVVSDATKPDYPIMYASAGFFNMTGYTSKEVVGRNCRFLQGS

GTDADELAKIRETLAAGNNYCGRILNYKKDGTSFWNLLTIAPIKDESGKVLKFIGMQVEV
SKHTEGAKEKALRPNGLPESLIRYDARQKDMATNSVTELVEAVKRPRALSESTNLHPFMT
KSESDELPKKPARRMSENVVPSGRRNSGGGRRNSMQRINEIPEKKSRKSSLSFMGIKKKS
ESLDESIDDGFIEYGEEDDEISDRDERPESVDDKVRQKEMRKGIDLATTLERIEKNFVIT
DPRLPDNPIIFASDSFLELTEYSREEILGRNCRFLQGPETDLTTVKKIRNAIDNQTEVTV
QLINYTKSGKKFWNIFHLQPMRDQKGEVQYFIGVQLDGSKHVEPVRNVIEETAVKEGEDL
VKKTAVNIDEAVRELPDANMTPEDLWANHSKVVHCKPHRKDSPPWIAIQKVLESGEPIGL
KHFKPVKPLGSGDTGSVHLVELVGTDQLFAMKAMDKAVMLNRNKVHRARAEREILDLLDH
PFLPALYASFQTKTHICLITDYYPGGELFMLLDRQPRKVLKEDAVRFYAAQVVVALEYLH
CQGIIYRDLKPENVLIQGNGDISLSDFDLSCLTSCKPQLLIPSIDEKKKKKQQKSQQTPI
FMAEPMRASNSFVGTEEYIAPEIISGAGHTSAVDWWALGILMYEMLYGYTPFRGKTRQKT
FTNVLQKDLKFPASIPASLQVKQLIFRLLQRDPKKRLGCFEGANEVKQHSFFKGINWALI
RCTNPPELETPIFSGEAENGEKVVDPELEDLQTNVF
>O50016
MIRFILLQNRQGKTRLAKYYVPLEDSEKHKVEYEVHRLVVNRDPKFTNFVEFRTHKVIYR
RYAGLFFSICVDITDNELAYLECIHLFVEILDHFFSNVCELDLVFNFHKVYRYLILDEFI
LAGELQETSKRQ
>O49044
MGVIKAVAVINGNNNVKGSLQFIQDSTGVTHVKGRITGLTPGLHGFHIHALGDTTNGCNS
TGPHFNPLKKDHGAPFDTERHAGDLGNVVAGADGVAEVSVRDTQIPLSGQHSILGRAVVV
HADQDDLGRTGHELSKTTGNAGARVRCGIIGLQASV
>Q42806
MANIDIEGILKQQQPYDGRVPKTKIVCTLGPASRSVEMTEKLLRAGMNVARFNFSHGTHD
YHQETLNNLKTAMHNTGILCAVMLDTKGPEIRTGFLKDGKPIQLKEGQEVTITTDYDIKG
DPEMISMSYKKLPVHLKPGNTILCSDGTITLTVLSCDPDAGTVRCRCENTATLGERKNVN
LPGVVVDLPTLTEKDKEDILGWGVPNKIDMIALSFVRKGSDLVNVRKVLGPHAKNIQLMS
KVENQEGVLNFDEILRETDAFMVARGDLGMEIPVEKIFLAQKMMIYKCNLVGKPVVTATQ
MLESMIKSPRPTRAEATDVANAVLDGTDCVMLSGESAAGAYPELAVKIMARICIEAESSL
DYGAIFKEMIRSTPLPMSPLESLASSAVRTANKAKAKLIVVLTRGGSTAKLVAKYRPAVP
ILSVVVPVLSTDSFDWTCSDETPARHSLIYRGLIPILGEGSAKATDAESTEVILEAALKS
ATERALCKPGDAVVALHRIGAASVIKICIVK
>Q8VYK7
MAKSSFKQEHDLEKRRAEAARIREKYPDRIPVIVEKAEKSDIPTIDKKKYLVPADLTVGQ
FVYVIRKRIKLSAEKAIFIFVDNVLPPAGALMSSVYEEKKDDDGFLYVTYSGENTFGFGS
P
>P34923
MVGSAKIKVGINGFGRIGRLVARVALERDDIELVAINDPFITPEYMTYMFKYDSTHGQWK
KTEVTLHSEGHLTFGGNPVAVYACRDPSEIPWGKHGADFVVESTGVFTDKDKAAAHLKGG
AKKVVISAPSKDAPMFVMGVNENKYSDEDIVSNASCTTNCLAPLAKVINDKFGILEGLMT
TVHATTATQKTVDGPSSKDWRGGRSAATNIIPSATGAAKAVGKVLPELNGKLTGMAFRVP
TTDVSVVDLTVRLEKPASYDAIKTAIKEASEGQMKGILGYTEDDVVSTDFITDSRSSIFD
AKAGIALSDTFVKLVAWYDNEWGYSNRVVDLIVHMAKQGTSQ
>P38414
MASLLFGRGQKLKGTVILMQKNVLDINALTAAQSPSGIIGGAFGVVGSIAGSIIDTATAF

LGRSVRLRLISATVADASGKGKVSKEAFLEGLLTSIPTLGDKQSAFSVHFEWDSNMGTPG
AFYIENFMQGGEFFLVSLTLDDVPNVGSIKFACNSWIYNDKKYQSDRIFFANKTYLPSAT
PAPLVSYRQEELKTLRGDGTGERQEWDRIYDYDVYNDLGAPDQKATLGRPVLGGSSTLPY
PRRGRTGRKKTVKEPQSESRSDTVYLPRDEAFGHVKSSDFLVYILKSASQNIVPQLRSVV
TLQLNNPEFNTFEDVRSLYDGGIKLPTDVLSKISPIPLFSELFRSDGEAALKFPPPKVIQ
VDHSAWMTDEEFAREMIAGVNPHIIKEVLSFPIKSKLDSQLYGDNTSKITKEHLEPNLGG
VTVEGAIQTNRLFTPDHHDALFPYLRKINATATKAYATRTVLFLQDNGTLKPLAIELSTP
HPDGDSFGPVSKVYLPASEGVEASIWLLAKAFVVVNDSCYHQLVSHWLNTHAVVEPFIIA
TNRHLSVVHPIHKLLLPHYRDTMNINALARNVLVNAEGIIESTFLWGNYAMEMSAVVYKD
WVFPDQGLPNDLIKRGVAVKDPSSPHGVRLLIEDYPYASDGLEIWAAIKSWVEEYVNFYY
KSDAAIAQDAELQAFWKELVEVGHGDLKSATWWFKMQNRKELIEACSILIWIASALHAAV
NFGQYPYGGYILNRPTKSRKFMPEKGTPEYDDLAKNYEKAYLRTITPKNDTLTDLTIIEV
LSRHASDEQYLGERIEGDDWTTDSVPKEAFKRFGKKLAEIEEKLTQRNNDESLRNRYGPV
KMPYTLLYPSSEEGLTCRGIPNSISI

>Q9ZNR6

MADQAMTDQDQGAVTLYSGTAITDAKKNHPFSVKVGLAQVLRGGAIVEVSSVNQAKLAES
AGACSVIVSDPVRSRGGVRRMPDPVLIKEVKRAVSVPVMARARVGHFVEAQILESLAVDY
IDESEIISVADDDHFINKHNFRSPFICGCRDTGEALRRIREGAAMIRIQGDLTATGNIAE
TVKNVRSLMGEVRVLNNMDDDEVFTFAKKISAPYDLVAQTKQMGRVPVVQFASGGITTPA
DAALMMQLGCDGVFVGSEVFDGPDPFKKLRSIVQAVQHYNDPHVLAEMSSGLENAMESLN
VRGDRIQDFGQGSV

>Q84KG5

MGEVAKEEVEERRSIVAVNPQPSKGLVSSAVDLIEKAVVYLFHDKSKPCHYLSGNFAPVV
DETPPCPDLPVRGHLPECLNGEFVRVGPNPKFMPVAGYHWFDGDGMIHGMRIKDGKATYA
SRYVKTSRLKQEEYFEGPKFMKIGDLKGFFGLFMVQMQLLRAKLKVIDVSYGVGTGNTAL
IYHHGKLLALSEADKPYVVKVLEDGDLQTLGLLDYDKRLSHSFTAHPKVDPFTDEMFTFG
YAHTPPYVTYRVISKDGVMRDPVPITIPASVMMHDFAITENYSIFMDLPLYFQPKEMVKG
GKLIFSFDATKKARFGVLPRYAKDDSLIRWFELPNCFIFHNANAWEEGDEVVLITCRLEN
PDLDMVNGAVKEKLENFKNELYEMRFNMKTGAASQKQLSVSAVDFPRINESYTTRKQRYV
YGTILDNITKVKGIIKFDLHAEPEAGKKKLEVGGNVQGIFDLGPGRYGSEAVFVPRERGI
KSEEDDGYLIFFVHDENTGKSEVNVIDAKTMSAEPVAVVELPNRVPYGFHAFFVNEEQLQ
WQQTDV

>A2YDY2

MASEKKQSNPMREIKVQKLVLNISVGESGDRLTRASKVLEQLSGQSPVFSKARYTVRSFG
IRRNEKIACYVTVRGEKAMQLLESGLKVKEYELLRRNFSETGCFGFGIQEHIDLGIKYDP
STGIYGMDFYVVLERAGYRVARRRRCKSRVGIQHRVTKEDAMKWFQVKYEGVILNKAQAN
TS

>P25892

MAETNPELSDLMAQTNKKIVPKFTEIFPVEDVNYPYSAFIASVRKDVIKHCTDHKGIFQP
VLPPEKKVPELWFYTELKTRTSSITLAIRMDNLYLVGFRTPGGVWWEFGKAGDTHLLGDN
PRWLGFGGRYQDLIGNKGLETVTMGRAEMTRAVNDLAKKKKMATLEEEEVQMQMQMPEAA
ELAAAAAAADPQADTKSKLVKLVVMVCEGLRFNTVSRTVDAGFNSQHGVTLTVTQGKQVQ
KWDRISKAAFEWADHPTAVIPDMQKLGIKDKNEAARIVALVKNQTTAAAAAATAASADND
DDEA

>O65735
MSNFKSKYHDELIANAAYIGTPGKGILAADESTGTIGKRLASINVENVETNRRALRELLF
TAPNVLQYLSGVILFEETLYQSTAAGKPFVDVLNEAGVLPGIKVDKGTVELAGTDGETTT
QGLDGLGARCAKYYEAGARFAKWRAVLKIGPNEPSLSILSIENAYGLARYAVICQENGLV
PIVELEILVDGSHDIHKCAAITERVLAATYKALSDHHVLLEGTLLKPNMVTPGSDSPKVA
PEVVAEHTVRALQRTVPAAVPAVVFLSGGQSEEEATVNLNAINQVKGKKPWTLSFSFGRA
LQQSTLKAWSGKEENVKNAQDALLTRAKANSEATLGTYKGNSQLGEGASESLHVKDYKY
>Q2QSB9
MPNVVDPPQISFAAKDMDLTEWEGDILAVLVTETDVSKATSSSSRFTNAAAALAKLDGEL
GGLLSEASAEEEFAGRAGQSVALRLPTAPGLHGFKRVCLVGVGNNMPSSAAACRSTGETI
AAVAKSAQARSAAVALASPPPGWVQGEDLRLNAAAAVASGVVLGLHEDRRYKSDSKKVHL
KQVDLIGFGSGQEMGRKLQYANHVSSAVIFAKELVNSPANVLTPAVLAEEASNIASSYSD
VLTATILDEEKCRELKMGSYLAVAAASANPPHFIHLCYKPPGGNVKRKLAIVGKGLTFDR
FYLSLDNLLIVTKFVCIGGYNIKIGAVTTIELMKKDMGGSAALFGAAKALGQIKPPGVEV
HFISAACENMISGTGMRPGDIVTASNGKTIEVDNTDAEGRLTLADALVYACKLGVDKIID
LATLTGYCRIALGPSIAGILTPSDELDKEVAAAYEASGEKFWRLPLEESYWEQMKSSVAD
MLNTGSPLGGAITAGLFLKQFVDEKVKWMHVDMAGPVWNYKKQEATGFGVSTLVEWVLIN
SS
>A3AZ88
MSCCGGNCGCGSGCQCGGGCGGCKMFPDVEATATTKTFVLAAPSNKASSGGMEMAVESGE
NGGCGCNTCKCGTSCSGCSCCSCN
>Q9SBK6
MEVMRILHMNKGNGETSYAKNSIVQSNIISLGRRVMDEALKKLMIRNSEILSFGIADLGC
SSGPNSLLSISNIVETIQNLCHDLDRPVPELSLSLNDLPSNDFNYIFASLPEFYDRVKKR
DNNYESLGFEHGSGGPCFVSAVPGSFYGRLFPRRSLHFVHSSSSLHWLSQVPCGEVNKKD
GVVITADLDNRGKIYLSKTSPKSAHKVYALQFQTDFSVFLRSRSEELVPGGRMVLSFLGR
SSPDPTTEESCYQWELLAQALMSLAKEGIIEEENIDAFNAPYYAASPEELKMAIEKEGSF
SIDRLEISPVDWEGGSISDDSYDIVRFKPEALASGRRVAKTIRAVVEPMLEPTFGQKVMD
ELFERYAKLVGEYVYVSSPRYTIVIVSLLRMG
>P42801
MFIESFKVESPNVKYTENEIHSVYDYETTEVVHEKTVNGTYQWIVKPKTVKYDFKTDIRV
PKLGVMLVGLGGNNGSTLTAGVIANKEGISWATKDKVQQANYFGSLTQASSIRVGSFNGE
EIYAPFKSLLPMVNPDDVVFGGWDISDMNLADAMARARVLDIDLQKQLRPYMENIVPLPG
IFDPDFIAANQGSRANHVIKGTKKEQVDHIIKDMREFKEKNKVDKVVVLWTANTERYSNV
VVGMNDTMENLMESVDRDEAEISPSTLYAIACVLEGIPFINGSPQNTFVPGLIDMAIRNN
VLIGGDDFKSGQTKMKSVLVDFLVGAGIKPTSIVSYNHLGNNDGMNLSAPQTFRSKEISK
SNVVDDMVASNGILFEPGEHPDHVVVIKYVPYVADSKRAMDEYTSEIFMGGKNTIVMHNT
CEDSLLAAPIILDLVLLAELSTRIQFKSEGEGKFHSFHPVATILSYLTKAPLVPPGTPVI
NALSKQRAMLENIMRACVGLAPENNMIMEFK
>P30792
MGSSGFSWTLPDHPKLPKGKSVAVVVLDGWGEANPDQYNCIHVAQTPVMDSLKNGAPEKW
RLVKAHGTAVGLPSDDDMGNSEVGHNALGAGRIFAQGAKLVDQALASGKIYDGDGFNYIK
ESFESGTLHLIGLLSDGGVHSRLDQLQLLLKGVSERGAKKIRVHILTDGRDVLDGSSIGF
VETLENDLLELRAKGVDAQIASGGGRMYVTMDRYENDWDVVKRGWDAQVLGEAPYKFKSA

LEAVKTLRAQPKANDQYLPPFVIVDDSGNAVGPVLDGDAVVTINFRADRMVMLAKALEYA
DFDNFDRVRVPKIRYAGMLQYDGELKLPSRYLVSPPEIDRTSGEYLVKNGIRTFACSETV
KFGHVTFFWNGNRSGYFDATKEEYVEVPSDSGITFNVAPNMKALEIAEKARDALLSGKFD
QVRVNLPNGDMVGHTGDIEATVVACKAADEAVKIILDAVEQVGGIYLVTADHGNAEDMVK
RNKSGKPLLDKNDRIQILTSHTLQPVPVAIGGPGLHPGVKFRNDIQTPGLANVAATVMNL
HGFEAPADYEQTLIEVADN

>Q8LAD0

MTVGVLALQGSFNEHIAALRRLGVQGVEIRKADQLLTVSSLIIPGGESTTMAKLAEYHNL
FPALREFVKMGKPVWGTCAGLIFLADRAVGQKEGGQELVGGLDCTVHRNFFGSQIQSFEA
DILVPQLTSQEGGPETYRGVFIRAPAVLDVGPDVEVLADYPVPSNKVLYSSSTVQIQEED
ALPETKVIVAVKQGNLLATAFHPELTADTRWHSYFIKMTKEIEQGASSSSSKTIVSVGET
SAGPEPAKPDLPIFQ

>Q500Y9

MAIRKLLLLLKPIDPYPFLQTEGASLIKNPQVLQYLESRCKVHKNAIKFCQEILSKKPVE
WKPISRNDLSHPIRDVDMVITVGGDGTLLHASHFIDDSVPVLGVNSDPTQAHEVEELSDQ
FDASRSTGHLCAATVENFEQVLDDILFGRVVPAKVSRISLKLNSETLLSHALNDILIAQP
CPAAVSRFSFKIKNKDGASSPKTVNCRSSGLRICTAAGSTAAMQSAGGFVMPMLSRDLQF
MVREPISPGSTASLMHSTFKPDQFMDVNWYSDHGTIYIDGCQVQHSVQLGDTIEISSDAP
VLNVFLSHGISQIRSRY

>Q9XFA2

MASPNGGVTTYDYHDSDSAAPVNAQTIEELHSLQRKAATTTKDGASPLQSISASLASLAR
EYGPNLVKGDPEATKGAPPVPIKHQQPSAAAATIAASDSSLKFTHVLYNLSPAELYEQAF
GQKKSSFITSTGALATLSGAKTGRSPRDKRVVKDETTSQELWWGKGSPNIEMDERQFVIN
RERALDYLNSLDKVYVNDQFLNWDSENRIKVRIITSRAYHALFMHNMCIRPTEEELESFG
TPDFTIYNAGEFPANRYANYMTSSTSINISLARREMVILGTQYAGEMKKGLFGVMHYLMP
KRGILSLHSGCNMGKEGDVALFFGLSGTGKTTLSTDHNRLLIGDDEHCWSDNGVSNIEGG
CYAKCIDLSQEKEPDIWNAIKFGTVLENVVFNERTREVDYADKSITENTRAAYPIEFIPN
AKIPCVGPHPKNVILLACDAYGVLPPVSKLNLAQTMYHFISGYTAIVAGTEDGVKEPTAT
FSACFGAAFIMYHPTKYAAMLAEKMQKYGATGWLVNTGWSGGRYGVGNRIKLPYTRKIID
AIHSGELLNASYKKTEVFGLEIPTAINGVPSEILGPVNTWTDKAAYKETLLKLAGLFKNN
FEVFASYKIGNNNSLTEQILAAAPNF

>Q9SWR3

MAAVTGLYGSIDEFLNHCSQSGDSAYSALRSLLERLEKPDTRTEARIFLAHLQKKLDNDG
ASQRCLETYHFQIQDIYLDRNEGTGYQNRKKFTMMVIPSIFMPEDWSFTFYEGINRHPDS
IFKDKTVAELGCGNGWISIAIAEKWLPLKVYGLDINPRAVKISWINLYLNAFDEDGQPVY
DSESKTLLDRVEFYESDLLSYCRDNHIELERIVGCIPQILNPNPDAMSKLVTENASEEFL
HSLSNYCALQGFVEDQFGLGLIARAVEEGIDVIKPMGIMIFNMGGRPGQGVCKRLFERRG
LSVNKLWQTKILQASDTDISALVEIEKNNPHRFEFFMGLVGDRPICARTAWAFGKACGRI
SHALSVYSCQLRHPNEVKKIFEFLKNGFHDISNSLDLSFEDDSVADEKIPFLAYLAGVLK
DGSRFPYEPPTGNKRFRDLIASFMKTYHHVPLSTDNVAIFPSRATAIENSLRLFTPRLAI
VEEHLTCNLPRQWLTSLEIEQTRDSKTPIDGITVIEAPRQSDLMIELIKKLKPQVVVTGI
AQFEAVTSSAFEHLLRVTREIGSRLFIDISDQFELSSLPSSIGVLKYLARTPLPSHAAII
CGLLRNRVYTDLEVAFVISEEQTIFDALTRTVELLQGNTALISQYYYGCLFHELLSFQIP
DRRQTAEREAENVEASDIDMIGFSSSAISVLSQSELSVRVTEKSSLLHMDVDQIFLPTPT

PVKAAIFESFARQNVTETECDVTPILRQFILNTWNFSVEHSAEFIYADFPLALFNKLVLC
CIEEGGSLCMPAGSNGNYAAAAKFLNANIMSIPTEAVGFKLTAKQLSSVLETVHKPWVY
ISGPTINPTGLLYSNEEMKSLLTVCARYGARTIIDTSFSGIKFNSQDWDGWNLDASLAGL
TGNPSFSVCLLGGLFFKIPTGGLSYGFLVLKSGFLADSFRSSFSGLNKPHNTVRYTAKKL
LELGEQKGNLTGAAQGQEKLLATRLKRLKETLENCGWEVIEARGGVSVIAKPSAYLGKNI
KLEKDGSTWVTKLDGTNIREAMLRATGLCINGPSWTGIPDYCRFTFALEDGDFDRALDCI
VKFNQLVK
>Q5XPJ9
MPLTRYQSRNEYGLADPDLYQAADKDDPEALLEGVAMAGLVGILRQLGDLAEFAAEMFHD
LHEEVMATASRSHGLMARVQQLEAEFPSIEKALLCQTDHSPFFSNKGVEWHPNLQLEQSV
VTSGDLPRCVMDSYEECRGPPRLFLLDKFDISGAGACLKRYTDPSFVRLETSSYEESWDD
IQREKKSQKAKRRASQWRNGGTPENALSSHAKLHELFLEEHLEAHHSDPARVVKLKTRKL
DGCSLISKSGESYMEKFVQTRVDSKISYEIITQNPGLLTWNMDSARDVVTDIPEISMVGA
MDKSHGGSRAEVSFPSEQENVANVNMNGGFIEKDIETVPESTYNEVRGTTITQDSQTVLN
GKPGFFQQRSYSEDLTSEADNYVDAPATMESETETDDECRPKSRSDTLKDGNHHIYSDAV
EERMEDPPQFSFSHSNGNTPVSENGRSSFGKKSTSYSYSDTASISIDDQSDGEKLSGCLP
STSSFKSELVDSMSHVTPEANKVSHDLNVQESVSSSNVDGQTSLSSNGTCSSPRPVSQND
QSCSLTVQSLASEVVETSPELVRLDLMKGGNDGRKVDPFDSSKSCASFDAKNSDLPSETS
SISSTSEGSRCDSTIEKNCMVASNLVNSGTSPQAFVDSQTGKQLPIADTDFETNSIVACS
EVLANSGSDPEERDGRCLTGKLVPCSAGVGMEVSPDTPSKVCGPSSADGIHLKDTLDDET
DCVSVTNVVVDVDSKNSVADVGSQSSVADIDSQSSVAEISDEHSCAFGNTADVSVSESHE
DTLENGMSVPSDFNSGVEKLAGDASPTCSKCDDHISHEGFHDLSGLDNATTDIVPNVELD
VSDNDNDTSSGGVNHAVSLSSTRGKGSLPWISTNTYQSSSDAGEIFHDTVVESDGTLLED
NNPESEIKMHKSPLEVSSEGLSTEPDNKDVESIESTSPKPSLDQRNRDTETKSPGESILD
DNCIDSTQVYNLNLLESEAIDQAVREQTSYASHEVADEELLQSNVFRGLEFEPQSAGLEF
APQSAGIELNRPKQELNLDPTFPSFGFIPETIPPNPEDMPPLPPMQWLIGKVPHSFPTFM
GESVETSSSALSAAPPIGSSLNVQIGSPPSELSVSLGSDESERLPGGFVHNASEKPLQSS
IQFPTMSTDLNSQYDSSELPTIPYQECIEDFGSEENNLLADHAAQNHELVYSQASSLQLP
QVKHEDFKDDADVHESQSSSDDHHCPETKSLTPTQSTKVEDKGHSVPDASNAETAESSNT
SVQKINPVSVGDAMWPVSCFSVAPTLDTYKTEVVPTVRLPRPRSPLVDAVAAHDRRKMKK
VSEMVHPPIKSKQDDKDSLLAQIRNKSVNLKPAVTTRPSIQTGPRTDLRVAAILEKANTI
RMAMAGSDEDEDSDSWSDS
>P49202
MGSLVQGDDFQHILRLLNTNVDGKNKIMYAMTAIRGIGRRFSNLVCKKAEVDLRKAAGEC
SADELERMMGIVANPRAYKIPDWFLNRQKDHKTGRFSQLTSSQLDTVMRDDLERLKKIRG
HRGLRHYWGLRVRGQHTKTTGRAGKTVGVAKKK
>Q9FK12
MDEYIDFRPLKYTEHKTSMTKYTKKSSEKLSGGKSLKKVSICYTDPDATDSSSDEDEEDF
LFPRRRVKRFVNEITVEPSCNNVVTGVSMKDRKRLSSSSDETQSPASSRQRPNNKVSVSG
QIKKFRGVRQRPWGKWAAEIRDPEQRRRIWLGTFETAEEAAVVYDNAAIRLRGPDALTNF
SIPPQEEEEEEEPEPVIEEKPVIMTTPTPTTSSSESTEEDLQHLSSPTSVLNHRSEEIQQ
VQQPFKSAKPEPGVSNAPWWHTGFNTGLGESDDSFPLDTPFLDNYFNESPPEMSIFDQPM
DQIFCENDDIFNDMLFLGGETMNIEDELTSSSIKDMGSTFSDFDDSLISDLLVA
>P80196

MMKSQKKLLIKIIVVQCLLVLCVTSQDFDFFYFVQQWPASYCDTRRSCCYPTTGKPDEDF
SIHGLWPNYKDGKWPQNCDRESSLDESEFSDLISTMEKNWPSLACPSSDGLKFWSHEWLK
HGTCSALNQHAYFQTALDFKTKSNLLQNLNNAGIKPRNGDYYGVESIKKAIEKGVGHTPF
IECNVDSQGNHQLYQVYLCVDSSASKFIDCPIFPHGGKCGSKIEFPSFSTNDDHDEF
>Q38869
MEKPNPRRPSNSVLPYETPRLRDHYLLGKKLGQGQFGTTYLCTEKSSSANYACKSIPKRK
LVCREDYEDVWREIQIMHHLSEHPNVVRIKGTYEDSVFVHIVMEVCEGGELFDRIVSKGC
FSEREAAKLIKTILGVVEACHSLGVMHRDLKPENFLFDSPSDDAKLKATDFGLSVFYKPG
QYLYDVVGSPYYVAPEVLKKCYGPEIDVWSAGVILYILLSGVPPFWAETESGIFRQILQG
KIDFKSDPWPTISEGAKDLIYKMLDRSPKKRISAHEALCHPWIVDEHAAPDKPLDPAVLS
RLKQFSQMNKIKKMALRVIAERLSEEEIGGLKELFKMIDTDNSGTITFEELKAGLKRVGS
ELMESEIKSLMDAADIDNSGTIDYGEFLAATLHINKMEREENLVVAFSYFDKDGSGYITI
DELQQACTEFGLCDTPLDDMIKEIDLDNDGKIDFSEFTAMMKKGDGVGRSRTMRNNLNFN
IAEAFGVEDTSSTAKSDDSPK
>P54609
MSTPAESSDSKSKKDFSTAILERKKSPNRLVVDEAINDDNSVVSLHPATMEKLQLFRGDT
ILIKGKKRKDTVCIALADETCEEPKIRMNKVVRSNLRVRLGDVISVHQCPDVKYGKRVHI
LPVDDTVEGVTGNLFDAYLKPYFLEAYRPVRKGDLFLVRGGMRSVEFKVIETDPAEYCVV
APDTEIFCEGEPVKREDEERLDDVGYDDVGGVRKQMAQIRELVELPLRHPQLFKSIGVKP
PKGILLYGPPGSGKTLIARAVANETGAFFFCINGPEIMSKLAGESESNLRKAFEEAEKNA
PSIIFIDEIDSIAPKREKTNGEVERRIVSQLLTLMDGLKSRAHVIVMGATNRPNSIDPAL
RRFGRFDREIDIGVPDEIGRLEVLRIHTKNMKLAEDVDLERISKDTHGYVGADLAALCTE
AALQCIREKMDVIDLEDDSIDAEILNSMAVTNEHFHTALGNSNPSALRETVVEVPNVSWN
DIGGLENVKRELQETVQYPVEHPEKFEKFGMSPSKGVLFYGPPGCGKTLLAKAIANECQA
NFISVKGPELLTMWFGESEANVREIFDKARQSAPCVLFFDELDSIATQRGGGSGGDGGGA
ADRVLNQLLTEMDGMNAKKTVFIIGATNRPDIIDSALLRPGRLDQLIYIPLPDEDSRLNI
FKAALRKSPIAKDVDIGALAKYTQGFSGADITEICQRACKYAIRENIEKDIEKEKRRSEN
PEAMEEDGVDEVSEIKAAHFEESMKYARRSVSDADIRKYQAFAQTLQQSRGFGSEFRFEN
SAGSGATTGVADPFATSAAAAGDDDDLYN
>Q9SYT0
MATLKVSDSVPAPSDDAEQLRTAFEGWGTNEDLIISILAHRSAEQRKVIRQAYHETYGED
LLKTLDKELSNDFERAILLWTLEPGERDALLANEATKRWTSSNQVLMEVACTRTSTQLLH
ARQAYHARYKKSLEEDVAHHTTGDFRKLLVSLVTSYRYEGDEVNMTLAKQEAKLVHEKIK
DKHYNDEDVIRILSTRSKAQINATFNRYQDDHGEEILKSLEEGDDDDKFLALLRSTIQCL
TRPELYFVDVLRSAINKTGTDEGALTRIVTTRAEIDLKVIGEEYQRRNSIPLEKAITKDT
RGDYEKMLVALLGEDDA
>P34937
MGRKFFVGGNWKCNGTVEQVEAIVQTLNAGQIVSPDVVEVVVSPPYVFLPIVKAKLRPEI
QVAAQNCWVKKGGAFTGEVSAEMLANLGVPWVILGHSERRSLLGESSEFVGEKVAYALAQ
GLKVIACVGETLEQREAGSTMEVVAEQTKAIAGKIKDWSNGVVAYEPVWAIGTGKVATPA
QAQEVHANLRDWLKTNVSPEVAESTRIIYGGSVTGASCKELAAQADVDGFLVGGASLKPE
FIDIINAAAVKSA
>P12365
MDPYKHRPSSAFNAPYWTTNSGAPVWNNDSSLTVGARGPILLEDYHCEKLANFDRERIPE

RVVHARGASAKGFFEVTHDITHLTCADFLRAPGVQTPVIVRFSTVIHERGSPETLRDPRG
FAVKFYTREGNWDLVGNNFPVFFIRDGIKFPDMVHALKPNPRTHIQDNWRILDFFSHHPE
SLHMFSFLFDDVGIPADYRHMDGSGVHTYTLVSRAGTVTYVKFHWRPTCGVRSLMDDEAV
RCGANHSHATKDLTDAIAAGNFPEWTLYIQTMDPEMEDRLDDLDPLDVTKTWPEDTFPLQ
PVGRLVLNRNIDNFFAENEQLAFCPGLIVPGIYYSDDKLLQTRIFSYSDTQRHRLGPNYL
LLPANAPKCAHHNNHYDGSMNFMHRHEEVDYFPSRYDAVRNAPRYPIPTAHIAGRREKTV
ISKENNFKQPGERYRAMDPARQERFITRWVDALSDPRLTHEIRTIWLSNWSQADRSLGQK
LASRLSAKPSM
>P48407
MSVGMGIDLEAFRKSQRADGFASILAIGTANPPNVVDQSTYPDYYFRVTNNEDNTDLKDK
FKRICERSAIKKRHMYLTEEILKKNPELCAFLEVPSLDTRQAMLAAEVPRLGKEAAEKAI
EEWGQPKSRITHLIFCTTTTPDLPGADFEVAKLLGLHPSVKRVGVFQHGCFAGGTVLRLA
KDLAENNRGARVLVVCSENTAVTFRGPSETHLDGLVGLALFGDGASALIVGADPIPQVEK
PCFEIVWTAQTVVPNSDGAISGKLREVGLTFQLKGAVPDLISTNIEKCLVEAFSQFNISD
WNQLFWIAHPGGHAILDQVEASLNLDPTKLRATRHVMSEYGNMSSACVHFILDETRKASR
QNGCSTSGGGFQMGVLFGFGPGLTVETVVLKSIPFP
>P31166
MQTIIISPLVSHRLCLARAVPCNRLLNNHHRAPPSIRLSNHRSTTSLRLFSSAAASRDSE
MATEDVQDPRIAKIASSIRVIPDFPKPGIMFQDITTLLLDTEAFKDTIALFVDRYKDKGI
SVVAGVEARGFIFGPPIALAIGAKFVPMRKPKKLPGKVISEEYSLEYGTDTIEMHVGAVE
PGERAIIIDDLIATGGTLAAAIRLLERVGVKIVECACVIELPELKGKEKLGETSLFVLVK
SAA
>Q8LBI1
MVFVKSTKSNAYFKRYQVKFRRRRDGKTDYRARIRLINQDKNKYNTPKYRFVVRFTNKDI
VAQIVSASIAGDIVKASAYAHELPQYGLTVGLTNYAAAYCTGLLLARRVLKMLEMDDEYE
GNVEATGEDFSVEPTDSRRPFRALLDVGLIRTTTGNRVFGALKGALDGGLDIPHSDKRFA
GFHKENKQLDAEIHRNYIYGGHVSNYMKLLGEDEPEKLQTHFSAYIKKGVEAESIEELYK
KVHAAIRADPNPKKTVKPAPKQHKRYNLKKLTYEERKNKLIERVKALNGAGGDDDDEDDE
E
>Q9SE42
MAAAAAAKIAPSMLSSDFANLAAEADRMVRLGADWLHMDIMDGHFVPNLTIGAPVIQSLR
KHTKAYLDCHLMVTNPSDYVEPLAKAGASGFTFHIEVSRDNWQELIQSIKAKGMRPGVSL
RPGTPVEEVFPLVEAENPVELVLVMTVEPGFGGQKFMPEMMEKVRALRKKYPSLDIEVDG
GLGPSTIDVAASAGANCIVAGSSIFGAAEPGEVISALRKSVEGSQNKS
>O65743
MVLKTELCRFSGAKIYPGRGIRFIRGDSQVFLFVNSKCKRYFHNRLKPSKLTWTAMFRKQ
HKKDAAQEAVKKRRRATKKPYSRSIVGATLEVIQKKRTEKPEVRDAAREAALREIKERIK
KTKDEKKAKKAEVASKAQKSQGKGNVQKGALPKGPKMGGGGGKA
>P28011
MASQNITPSPTASSDSVFAHLVRAPEDPILGVTVAYNKDPSPIKLNLGVGAYRTEEGKPL
VLDVVRRVERQLLNDMSRNKEYIPIVGLADFNKLSAKLIFGADSPAIQENRVTTVQGLSG
TGSLRVGGEFLAKHYHQRIIYLPTPTWGNHTKVFNLAGLTVKTYRYYAPATRGLDFQGLL
EDLGSAPSGSVVLLHACAHNPTGVDPTLEQWEQIRQLIRSKSLLPFFDSAYQGFASGSLD
ADAQPVRLFVADGGELLVAQSYAKNMGLYGERVGALSIVSKSADVSSRVESQLKLVIRPM

YSSPPIHGASIVAAILKDRDLYNDWTIELKAMADRIINMRQQLFDALRARGTPGDWSHII
KQIGMFTFTGLNPEQVSILTKEYHIYLTSDGRISMAGLSSKTVPHLAHAIHAVVTRVA
>Q8W575
MIGAVNSVEAVITSIQGLSGSPEDLSALHDLLRGAQDSLRAEPGVNFSTLDQLDASKHSL
GYLYFLEVLTCGPVSKEKAAYEIPIIARFINSCDAGQIRLASYKFVSLCKILKDHVIALG
DPLRGVGPLLNAVQKLQVSSKRLTALHPDVLQLCLQAKSYKSGFSILSDDIVEIDQPRDF
FLYSYYGGMICIGLKRFQKALELLYNVVTAPMHQVNAIALEAYKKYILVSLIHNGQFTNT
LPKCASTAAQRSFKNYTGPYIELGNCYNDGKIGELEALVVARNAEFEEDKNLGLVKQAVS
SLYKRNILRLTQKYLTLSLQDIANMVQLGNAKEAEMHVLQMIQDGQIHALINQKDGMVRF
LEDPEQYKSSEMIEIMDSVIQRTIGLSKNLLAMDESLSCDPLYLGKVGRERQRYDFGDDF
DTVPQKFSM
>Q8LPU4
MALKQKDTDAAATATGTKKRRRVFFSDTDAGVEANECMKVFLVWNPGEVSSVDCTAIQPF
DLNHFFGEDGKIYGYKNLKINVWISAKSFHGYADVSFDETSDGGKGITDLKPVLQNIFGE
NLVEKEEFLHTFSKECEYIRTAVTNGSAIKHDGSYESDPAVEIVRVELQGAAAFLYSRLV
PLVLLLVEGSTPIDIGEHGWEMLLVVKKATQEAGSKFELLGFAAVHNFYHYPESIRLRIS
QILVLPPYQGEGHGLGLLEAINYIAQSENIYDVTIESPSDYLQYVRSSIDCLRLLMFDPI
KPALGAIVLSLKETNLSKRAQSLRMVPPADLMETVRQKLKINKKQFLRCWEILVFLSLDS
QDHKSMDNFRACIYDRMKGEILGSASGTNRKRLLQMPTSFNKEASFAVYWTQEIEDEDEQ
TVEQQPEDLKTQEQQLNELVDIQIEEIAGVAKNVTSRCKDKMTELVVQ
>P46011
MSMQQETSHMTAAPQTNGHQIFPEIDMSAGDSSSIVRATVVQASTVFYDTPATLDKAERL
LSEAAENGSQLVVFPEAFIGGYPRGSTFELAIGSRTAKGRDDFRKYHASAIDVPGPEVER
LALMAKKYKVYLVMGVIEREGYTLYCTVLFFDSQGLFLGKHRKLMPTALERCIWGFGDGS
TIPVFDTPIGKIGAAICWENRMPSLRTAMYAKGIEIYCAPTADSRETWLASMTHIALEGG
CFVLSANQFCRRKDYPSPPEYMFSGSEESLTPDSVVCAGGSSIISPLGIVLAGPNYRGEA
LITADLDLGDIARAKFDFDVVGHYSRPEVFSLNIREHPRKAVSFKTSKVMEDESV
>O04847
MPKSWPIVISSHSFCFLPNSEQERKMKDLNFHAATLSEEESLRELKAFDETKAGVKGIVD
TGITKIPRIFIDQPKNLDRISVCRGKSDIKIPVINLNGLSSNSEIRREIVEKIGEASEKY
GFFQIVNHGIPQDVMDKMVDGVRKFHEQDDQIKRQYYSRDRFNKNFLYSSNYVLIPGIAC
NWRDTMECIMNSNQPDPQEFPDVCRDILMKYSNYVRNLGLILFELLSEALGLKPNHLEEM
DCAEGLILLGHYYPACPQPELTFGTSKHSDSGFLTILMQDQIGGLQILLENQWIDVPFIP
GALVINIADLLQLITNDKFKSVEHRVLANKVGPRISVAVAFGIKTQTQEGVSPRLYGPIK
ELISEENPPIYKEVTVKDFITIRFAKRFDDSSSLSPFRLNN
>Q39097
MGDKNDDDKNIEIWKIKKLIKSLEAARGNGTSMISLIMPPRDQVSRVTKMLGDEYGTASN
IKSRVNRQSVLGAITSAQQRLKLYNRVPPNGLVLYTGTIVNEDGKEKKVTIDFEPFRPIN
ASLYLCDNKFHTEALNELLESDDKFGFIVMDGNGTLFGTLSGNTREVLHKFSVDLPKKHG
RGGQSALRFARLRMEKRHNYVRKTAELATQYYINPATSQPNVSGLILAGSADFKTELSQS
DMFDPRLAAKILNVVDVSYGGENGFNQAIELSAEILANVKFIQEKRLIGKYFEEISQDTG
KYVFGVEDTLNALESGAIETLIVWENLDINRYVMKNSATGETVIKHLNKEQEANTENFKV
ADSDLALDVEEKLSLLEWLANEYRRFGCALEFVTNKSQEGSQFCRGFGGIGGILRYQLDM
TAFDSEDGEALDDDSE

>Q9FVU9
MEGSSSTIARKTWELENSILTVDSPDSTSDNIFYYDDTSQTRFQQEKPWENDPHYFKRVK
ISALALLKMVVHARSGGTIEIMGLMQGKTDGDTIIVMDAFALPVEGTETRVNAQDDAYEY
MVEYSQTNKLAGRLENVVGWYHSHPGYGCWLSGIDVSTQRLNQQHQEPFLAVVIDPTRTV
SAGKVEIGAFRTYSKGYKPPDEPVSEYQTIPLNKIEDFGVHCKQYYSLDVTYFKSSLDSH
LLDLLWNKYWVNTLSSSPLLGNGDYVAGQISDLAEKLEQAESHLVQSRFGGVVPSSLHKK
KEDESQLTKITRDSAKITVEQVHGLMSQVIKDELFNSMRQSNNKSPTDSSDPDPMITY
>P43254
MEEISTDPVVPAVKPDPRTSSVGEGANRHENDDGGSGGSEIGAPDLDKDLLCPICMQIIK
DAFLTACGHSFCYMCIITHLRNKSDCPCCSQHLTNNQLYPNFLLDKLLKKTSARHVSKTA
SPLDQFREALQRGCDVSIKEVDNLLTLLAERKRKMEQEEAERNMQILLDFLHCLRKQKVD
ELNEVQTDLQYIKEDINAVERHRIDLYRARDRYSVKLRMLGDDPSTRNAWPHEKNQIGFN
SNSLSIRGGNFVGNYQNKKVEGKAQGSSHGLPKKDALSGSDSQSLNQSTVSMARKKRIHA
QFNDLQECYLQKRRQLADQPNSKQENDKSVVRREGYSNGLADFQSVLTTFTRYSRLRVIA
EIRHGDIFHSANIVSSIEFDRDDELFATAGVSRCIKVFDFSSVVNEPADMQCPIVEMSTR
SKLSCLSWNKHEKNHIASSDYEGIVTVWDVTTRQSLMEYEEHEKRAWSVDFSRTEPSMLV
SGSDDCKVKVWCTRQEASVINIDMKANICCVKYNPGSSNYIAVGSADHHIHYYDLRNISQ
PLHVFSGHKKAVSYVKFLSNNELASASTDSTLRLWDVKDNLPVRTFRGHTNEKNFVGLTV
NSEYLACGSETNEVYVYHKEITRPVTSHRFGSPDMDDAEEEAGSYFISAVCWKSDSPTML
TANSQGTIKVLVLAA
>P93804
MGLFTVTKKATTPFDGQKPGTSGLRKKVTVFQQPHYLQNFVQSTFNALPVDQVRGATIVV
SGDGRYFSKDAVQIITKMAAANGVRRVWVGQNSLMSTPAVSAVIRERVGADGSKATGAFI
LTASHNPGGPKEDFGIKYNMGNGGPAPESVTDKIFSNTTTISEYLISEDLPDVDISVVGV
TSFSGPEGPFDVDVFDSSVDYIKLMKTIFDFEAIKKLLTSPKFTFCYDALHGVAGAYAKH
IFVEELGADESSLLNCVPKEDFGGGHPDPNLTYAKELVERMGLGKSSSNVEPPEFGAAAD
GDADRNMILGKRFFVTPSDSVAIIAANAVQSIPYFASGLKGVARSMPTSAALDVVAKNLN
LKFFEVPTGWKFFGNLMDAGMCSICGEESFGTGSDHIREKDGIWAVLAWLSIIAFKNKDN
LGGDKLVTVEDIVRQHWATYGRHYYTRYDYENVDAGAAKELMANLVSMQSSLSDVNKLVK
EIRSDVSEVVAADEFEYKDPVDGSVSKHQGIRYLFGDGSRLVFRLSGTGSVGATIRVYIE
QYERDSSKTGRDSQDALAPLVDVALKLSKMQEYTGRSAPTVIT
>P46302
MDTQVKLAVVVKVMGRTGSRGQVTQVRVKFLDDQNRLIMRNVKGPVCEGDILTLLESERE
ARRLR
>Q9XGN1
MDNSAPDSLSRSETAVTYDSPYPLYAMAFSSLRSSSGHRIAVGSFLEDYNNRIDILSFDS
DSMTVKPLPNLSFEHPYPPTKLMFSPPSLRRPSSGDLLASSGDFLRLWEINEDSSTVEPI
SVLNNSKTSEFCAPLTSFDWNDVEPKRLGTCSIDTTCTIWDIEKSVVETQLIAHDKEVHD
IAWGEARVFASVSADGSVRIFDLRDKEHSTIIYESPQPDTPLLRLAWNKQDLRYMATILM
DSNKVVILDIRSPTMPVAELERHQASVNAIAWAPQSCKHICSGGDDTQALIWELPTVAGP
NGIDPMSVYSAGSEINQLQWSSSQPDWIGIAFANKMQLLRV
>Q09152
MSVSCCCRNLGKTIKKAIPSHHLHLRSLGGSLYRRRIQSSSMETDLKSTFLNVYSVLKSD
LLHDPSFEFTNESRLWVDRMLDYNVRGGKLNRGLSVVDSFKLLKQGNDLTEQEVFLSCAL

GWCIEWLQAYFLVLDDIMDNSVTRRGQPCWFRVPQVGMVAINDGILLRNHIHRILKKHFR
DKPYYVDLVDLFNEVELQTACGQMIDLITTFEGEKDLAKYSLSIHRRIVQYKTAYYSFYL
PVACALLMAGENLENHIDVKNVLVDMGIYFQVQDDYLDCFADPETLGKIGTDIEDFKCSW
LVVKALERCSEEQTKILYENYGKPDPSNVAKVKDLYKELDLEGVFMEYESKSYEKLTGAI
EGHQSKAIQAVLKSFLAKIYKRQK
>P15494
MGVFNYETETTSVIPAARLFKAFILDGDNLFPKVAPQAISSVENIEGNGGPGTIKKISFP
EGFPFKYVKDRVDEVDHTNFKYNYSVIEGGPIGDTLEKISNEIKIVATPDGGSILKISNK
YHTKGDHEVKAEQVKASKEMGETLLRAVESYLLAHSDAYN
>Q9SYM4
MPGNKYNCSSSHIPLSRTERLLRDRELREKRKSNRARNPNDVAGSSENSENDLRLEGDSS
RQYVEQYLEGAAAAMAHDDACERQEVRPYNRQRLLVVANRLPVSAVRRGEDSWSLEISAG
GLVSALLGVKEFEARWIGWAGVNVPDEVGQKALSKALAEKRCIPVFLDEEIVHQYYNGYC
NNILWPLFHYLGLPQEDRLATTRSFQSQFAAYKKANQMFADVVNEHYEEGDVVWCHDYHL
MFLPKCLKEYNSKMKVGWFLHTPFPSSEIHRTLPSRSELLRSVLAADLVGFHTYDYARHF
VSACTRILGLEGTPEGVEDQGRLTRVAAFPIGIDSDRFIRALEVPEVIQHMKELKERFAG
RKVMLGVDRLDMIKGIPQKILAFEKFLEENANWRDKVVLLQIAVPTRTDVPEYQKLTSQV
HEIVGRINGRFGTLTAVPIHHLDRSLDFHALCALYAVTDVALVTSLRDGMNLVSYEFVAC
QEAKKGVLILSEFAGAAQSLGAGAILVNPWNITEVAASIGQALNMTAEEREKRHRHNFHH
VKTHTAQEWAETFVSELNDTVIEAQLRISKVPPELPQHDAIQRYSKSNNRLLILGFNATL
TEPVDNQGRRGDQIKEMDLNLHPELKGPLKALCSDPSTTIVVLSGSSRSVLDKNFGEYDM
WLAAENGMFLRLTNGEWMTTMPEHLNMEWVDSVKHVFKYFTERTPRSHFETRDTSLIWNY
KYADIEFGRLQARDLLQHLWTGPISNASVDVVQGSRSVEVRAVGVTKGAAIDRILGEIVH
SKSMTTPIDYVLCIGHFLGKDEDVYTFFEPELPSDMPAIARSRPSSDSGAKSSSGDRRPP
SKSTHNNNKSGSKSSSSSNSNNNNKSSQRSLQSERKSGSNHSLGNSRRPSPEKISWNVLD
LKGENYFSCAVGRTRTNARYLLGSPDDVVCFLEKLADTTSSP
>P45432
MERDEEASGPMMEMCTNGGEETSNRRPIISGEPLDIEAYAALYKGRTKIMRLLFIANHCG
GNHALQFDALRMAYDEIKKGENTQLFREVVNKIGNRLGEKYGMDLAWCEAVDRRAEQKKV
KLENELSSYRTNLIKESIRMGYNDFGDFYYACGMLGDAFKNYIRTRDYCTTTKHIIHMCM
NAILVSIEMGQFTHVTSYVNKAEQNPETLEPMVNAKLRCASGLAHLELKKYKLAARKFLD
VNPELGNSYNEVIAPQDIATYGGLCALASFDRSELKQKVIDNINFRNFLELVPDVRELIN
DFYSSRYASCLEYLASLKSNLLLDIHLHDHVDTLYDQIRKKALIQYTLPFVSVDLSRMAD
AFKTSVSGLEKELEALITDNQIQARIDSHNKILYARHADQRNATFQKVLQMGNEFDRDVR
AMLLRANLLKHEYHARSARKL
>Q43621
MARKMLNDGEPDLKKGEEQGKVYDFDLFIIGAGSGGVRAARFSSNFGAKVGICELPFHPI
SSETIGGVGGTCVIRGCVPKKILVYGASYGGELQDARNFGWELNENVDFNWKKLLQKKTD
EINRLNGIYKRLLSNAGVKLFEGEGKIASPNEVEVTQLDGTKLSYSAKHILIATGSRAQR
PNIPGQELGITSDEALSLEEFPKRAVILGGGYIAVEFASIWRGMGSSVNLVFRKELPLRG
FDDEMRAVVARNLEGRGINLHPRTNLAQLIKTEDGIKVITDHGEELIADVVLFATGRSPN
SKRLNLEKVGVEFDKAGAIVVDEYSRTNIPSIWAVGDVTNRLNLTPVALMEASLFAKTVF
GGQASKPDYNDIPYAVFCIPPLSVVGLSEEQAVEQTKGDVLIFTSTFNPMKNTISGRQEK
TVMKLVVDAQTDKVLGASMCGPDAPEIVQGIAIAIKCGATKAQFDSTVGIHPSSAEEFVT

MRSETRRVTGGVKPKTNL
>Q40577
MASAAVANYEEEIVRPVADFSPSLWGDQFLSFSIKNQVAEKYAKEIEALKEQTRNMLLAT
GMKLADTLNLIDTIERLGISYHFEKEIDDILDQIYNQNSNCNDLCTSALQFRLLRQHGFN
ISPEIFSKFQDENGKFKESLASDVLGLLNLYEASHVRTHADDILEDALAFSTIHLESAAP
HLKSPLREQVTHALEQCLHKGVPRVETRFFISSIYDKEQSKNNVLLRFAKLDFNLLQMLH
KQELAQVSRWWKDLDFVTTLPYARDRVVECYFWALGVYFEPQYSQARVMLVKTISMISIV
DDTFDAYGTVKELEAYTDAIQRWDINEIDRLPDYMKISYKAILDLYKDYEKELSSAGRSH
IVCHAIERMKEVVRNYNVESTWFIEGYTPPVSEYLSNALATTTYYYLATTSYLGMKSATE
QDFEWLSKNPKILEASVIICRVIDDTATYEVEKSRGQIATGIECCMRDYGISTKEAMAKF
QNMAETAWKDINEGLLRPTPVSTEFLTPILNLARIVEVTYIHNLDGYTHPEKVLKPHIIN
LLVDSIKI
>Q42539
MKQFWSPSSINKNKAMVENLQNHGIVTSDEVAKAMEAVDRGVFVTDRSSAYVDSPMSIGY
NVTISAPHMHAMCLQLLEKHLKPGMRVLDVGSGTGYLTACFAVMVGTEGRAIGVEHIPEL
VASSVKNIEASAASPFLKEGSLAVHVGDGRQGWAEFAPYDAIHVGAAAPEIPEALIDQLK
PGGRLVIPVGNIFQDLQVVDKNSDGSVSIKDETSVRYVPLTSREAQLRGD
>P52580
MASEKSKILVVGGTGYLGRHVVAASARLGHPTSALVRDTAPSDPAKAALLKSFQDAGVTL
LKGDLYDQASLVSAVKGADVVISVLGSMQIADQSRLVDAIKEAGNVKRFFPSEFGLDVDR
TGIVEPAKSILGAKVGIRRATEAAGIPYTYAVAGFFAGFGLPKVGQVLAPGPPADKAVVL
GDGDTKAVFVEEGDIATYTVLAADDPRAENKVLYIKPPANTLSHNELLSLWEKKTGKTFR
REYVPEEAVLKQIQESPIPLNIILAIGHAAFVRGEQTGFEIDPAKGVDASELYPDVKYTT
VDEYLNRFL
>Q7G188
MSGYVGVVVSDPWLQSQFTQVELRTLNSKYVSVKNQNGKVTIEDLPPLFAKLKALSATFK
EDEIKGMLGELGSDTSTDVSFEEFLKIYLNLLSKAAEKSGGHHKNSSSFLKACTTTLLHT
IYQSEKGPFVQHINRYLGDDPFLKQFLPLDPHSNQLYELVKDGVLLCKLINVAVPGTIDE
RAINTKRVLNPWERNENHTLCLNSAKAVGCSVVNIGTQDLAEGRPHLVLGLISQLIKIQV
LADLNLKKTPQLVELLEDSDDVEELLRLPPEKVLLKWMNFHLKKGGYKKTVSNFSADLKD
AQAYAFLLNVLAPEHCDPATLDAKDPLERAELVLSHAERMNCKRYLTAEEIVEGSSTLNL
AFVAQIFHERNGLNKDGKYAFAEMMTEDVETCRDERCYRLWINSLGIDSYVNNVFEDVRN
GWILLEVLDKVSPSSVNWKHASKPPIKMPFRKVENCNQVIKIGKQLKFSLVNVAGNDIVQ
GNKKLILGLLWQLMRFHMLQLLKSLRSRTLGKEMTDADILSWANRKVRTMGRKLQIESFK
DKSLSSGLFFLNLLWAVEPRVVNWNLVTKGETDDEKRLNATYIVSVARKLGCSVFLLPED
IVEVNQKMILILTASIMYWSLQRHSRESSDSSSTQSTTTTCTSTASSPAPSVTEEEEVSS
LSGEVTSLAVGDAVSEITTVSEEASIE
>O04630
MLLRLTARSIRRFTTSSSSLPLLSSSSFCTVPTMAANHPKDEAYLSAVIPKRIKLFEQIQ
ANQLENLKSLPHDPIKVTLPDGNVKEGKKWETTPMDIAAQISKGLANSALISAVDDVLWD
MNRPLEGDCKLELFKFDSDKGRDTLWHSSAHILGQALEQEYGCQLCIGPCTTRGEGFYYD
GFYGELGLSDNHFPSIEAGAAKAAKEAQPFERIEVTKDQALEMFSENNFKVELINGLPAD
MTITVYRCGPLVDLCRGPHIPNTSFVKAFKCLRASSAYWKGDKDRESLQRVYGISYPDQK
QLKKYLQFLEEAKKYDHRLLGQKQELFFSHQLSPGSYFFLPLGTRVYNRLMDFIKNQYWH

RGYTEVITPNMYNMELWQTSGHADNYKDNMFTFNIEKQEFGLKPMNCPGHCLIFQHRVRS
YRELPMRLADFGVLHRNEASGALSGLTRVRRFQQDDAHIFCTTEQVKGEVQGVLEFIDYV
YKVFGFTYELKLSTRPEKYLGDLETWDKAEADLKEAIEAFGKPLVLNEGDGAFYGPKIDI
TVSDAMNRKFQCATLQLDFQLPIRFNLEYAAEDEAKKSRPVMIHRAVLGSVERMFAILLE
HYKGKWPFWISPRQAIVCPISEKSQQYAEKVQKQIKDAGFYVDADLTDRKIDKKVREAQL
AQYNYILVVGETEAATGQVSVRVRDNAAHSVKSIEDLLEEFKAKTAEFV
>O81644
MSTKVLDPAFQGAGQKPGTEIWRIENFEAVPVPKSEHGKFYMGDTYIVLQTTQNKGGAYL
FDIHFWIGKDTSQDEAGTAAVKTVELDAVLGGRAVQHREIQGHESDKFLSYFKPCIIPLE
GGVASGFKTVEEEVFETRLYTCKGKRAIRLKQVPFARSSLNHDDVFILDTEEKIYQFNGA
NSNIQERAKALEVVQYLKDKYHEGTCDVAIVDDGKLDTESDSGAFWVLFGGFAPIGRKVA
NDDDIVPESTPPKLYCITDGKMEPIDGDLSKSMLENTKCYLLDCGAEIYIWVGRVTQVDE
RKAASQSAEEFLASENRPKATHVTRVIQGYESHSFKSNFDSWPSGSATPGNEEGRGKVAA
LLKQQGVGLKGIAKSAPVNEDIPPLLESGGKLEVWYVNGKVKTPLPKEDIGKLYSGDCYL
VLYTYHSGERKDEYFLSCWFGKKSIPEDQDTAIRLANTMSNSLKGRPVQGRIYEGKEPPQ
FVALFQPMVVLKGGLSSGYKSSMGESESTDETYTPESIALVQVSGTGVHNNKAVQVETVA
TSLNSYECFLLQSGTSMFLWHGNQSTHEQLELATKVAEFLKPGITLKHAKEGTESSTFWF
ALGGKQNFTSKKASSETIRDPHLFSFAFNRGKFQVEEIYNFAQDDLLTEDIYFLDTHAEV
FVWVGQCVEPKEKQTVFEIGQKYIDLAGSLEGLHPKVPIYKINEGNEPCFFTTYFSWDAT
KAIVQGNSFQKKASLLFGTHHVVEDKSNGGNQGLRQRAEALAALNSAFNSSSNRPAYSSQ
DRLNESHDGPRQRAEALAALSSAFNSSSSSTKSPPPPRPVGTSQASQRAAAVAALSQVLV
AENKKSPDTSPTRRSTSSNPADDIPLTEAKDEEEASEVAGLEAKEEEEVSPAADETEAKQ
ETEEQGDSEIQPSGATFTYEQLRAKSENPVTGIDFKRREAYLSEEEFQSVFGIEKEAFNN
LPRWKQDLLKKKFDLF
>O82803
MAEEVEEERLKYLDFVRAAGVYAVDSFSTLYLYAKDISGPLKPGVDTIENVVKTVVTPVY
YIPLEAVKFVDKTVDVSVTSLDGVVPPVIKQVSAQTYSVAQDAPRIVLDVASSVFNTGVQ
EGAKALYANLEPKAEQYAVITWRALNKLPLVPQVANVVVPTAVYFSEKYNDVVRGTTEQG
YRVSSYLPLLPTEKITKVFGDEAS
>P83326
MAFSYCSSSLFVSLLLVILFISPLSQRPSVKAENHLISEICPKTRNPSLCLQALESDPRS
ASKDLKGLGQFSIDIAQASAKQTSKIIASLTNQATDPKLKGRYETCSENYADAIDSLGQA
KQFLTSGDYNSLNIYASAAFDGAGTCEDSFEGPPNIPTQLHQADLKLEDLCDIVLVISNL
LPGSK
>P04711
MASTKAPGPGEKHHSIDAQLRQLVPGKVSEDDKLIEYDALLVDRFLNILQDLHGPSLREF
VQECYEVSADYEGKGDTTKLGELGAKLTGLAPADAILVASSILHMLNLANLAEEVQIAHR
RRNSKLKKGGFADEGSATTESDIEETLKRLVSEVGKSPEEVFEALKNQTVDLVFTAHPTQ
SARRSLLQKNARIRNCLTQLNAKDITDDDKQELDEALQREIQAAFRTDEIRRAQPTPQAE
MRYGMSYIHETVWKGVPKFLRRVDTALKNIGINERLPYNVSLIRFSSWMGGDRDGNPRVT
PEVTRDVCLLARMMAANLYIDQIEELMFELSMWRCNDELRVRAEELHSSSGSKVTKYYIE
FWKQIPPNEPYRVILGHVRDKLYNTRERARHLLASGVSEISAESSFTSIEEFLEPLELCY
KSLCDCGDKAIADGSLLDLLRQVFTFGLSLVKLDIRQESERHTDVIDAITTHLGIGSYRE
WPEDKRQEWLLSELRGKRPLLPPDLPQTDEIADVIGAFHVLAELPPDSFGPYIISMATAP

SDVLAVELLQRECGVRQPLPVVPLFERLADLQSAPASVERLFSVDWYMDRIKGKQQVMVG
YSDSGKDAGRLSAAWQLYRAQEEMAQVAKRYGVKLTLFHGRGGTVGRGGGPTHLAILSQP
PDTINGSIRVTVQGEVIEFCFGEEHLCFQTLQRFTAATLEHGMHPPVSPKPEWRKLMDEM
AVVATEEYRSVVVKEARFVEYFRSATPETEYGRMNIGSRPAKRRPGGGITTLRAIPWIFS
WTQTRFHLPVWLGVGAAFKFAIDKDVRNFQVLKEMYNEWPFFRVTLDLLEMVFAKGDPGI
AGLYDELLVAEELKPFGKQLRDKYVETQQLLLQIAGHKDILEGDPFLKQGLVLRNPYITT
LNVFQAYTLKRIRDPNFKVTPQPPLSKEFADENKPAGLVKLNPASEYPPGLEDTLILTMK
GIAAGMQNTG

>Q94JY4

MAKAGGITNAVNVGIAVQADWENREFISHISLNVRRLFEFLVQFESTTKSKLASLNEKLD
LLERRLEMLEVQVSTATANPSLFAT

>Q39242

MCWISMSQSRFIIKSLFSTAGGFLLGSALSNPPSLATAFSSSSSSSSAAAAVDMETHKTK
VCIVGSGPAAHTAAIYASRAELKPLLFEGWMANDIAPGGQLTTTTDVENFPGFPEGILGI
DIVEKFRKQSERFGTTIFTETVNKVDFSSKPFKLFTDSRTVLADSVIISTGAVAKRLSFT
GSGEGNGGFWNRGISACAVCDGAAPIFRNKPLVVIGGGDSAMEEANFLTKYGSKVYIIHR
RDTFRASKIMQQRALSNPKIEVIWNSAVVEAYGDENGRVLGGLKVKNVVTGDVSDLKVSG
LFFAIGHEPATKFLDGQLELDEDGYVVTKPGTTKTSVVGVFAAGDVQDKKYRQAITAAGT
GCMAALDAEHYLQEIGSQEGKSD

>O48626

MPEIDALFESINVRDLLAGHDLNDPTTPLSAPDLRLLINRLESHSLRIKSKVQSYLVAHH
SDFSELFSLCQDTVSRTRLISDDVSDVLQLVSDRPIDVEIRSVVDEITEKTKEVKLKRES
LDLVNAIVGICEALQETKEALKNGRFRFAAERIRELKVVLRIGEEEDGEPVAYALLRKEW
SNCFDEIQEVLAKFMENAVRFELDSSRIRIKYQLSVGETAGIALSTVLEAMEVIGILDYG
LAKAADSIFKHVITPAVTHASTFAAVEDLCKSAGEVTEATLRLEQSSDHKFEDVDGDAMY
SGILKVVKFICSSLCFGNVTWIHSFGRLTWPRISELIISKFLSKVVPEDASKLADFQKII
ERTSQFEAALKELNFVSSSDAESRLSKYAEDVEVHFASRKKIEILAKARNLLLQCNFTIP
QDIAMRNAKHIVCLLFSSERCVVSEAASQLMNLVHKTLEDVCVSSARVASEFYNAARDSI
LLYEAVVPVKLEKQLDGLNEAAVLLHNDCLYLFEEILGLAFEYRASFPSSIKEYAVFADI
APRFKLMAEEVLQKQVHLVISSLREAIDSADGFQNTHQIKQFKSAEFSIDQVVFSLKNVH
MIWEPVLRPKTYKQSMCAVLESVFRRIARDILLLDDMAADETFELQKLIYLMLKNLSSVL
DSVRSADETSRPLDDIIPSLRKTRKLAELLDMPLMSITSAWESGELFRCNFTRTEVQDFI
KAIFTDSPLRKECLWRIDEVNQ

>Q9C9C9

MESETLTAKATITTTTLPSHDETKTESTEFEKNQKRYQDLISTFPHEKGWRPKEPLIEYG
GYWWLPSLLEGCIHAQEFFQARPSDFLVCSYPKTGTTWLKALTFAIANRSRFDDSSNPLL
KRNPHEFVPYIEIDFPFFPEVDVLKDKGNTLFSTHIPYELLPDSVVKSGCKMVYIWREPK
DTFISMWTFLHKERTELGPVSNLEESFDMFCRGLSGYGPYLNHILAYWKAYQENPDRILF
LKYETMRADPLPYVKSLAEFMGHGFTAEEEEKGVVEKVVNLCSFETLKNLEANKGEKDRE
DRPGVYANSAYFRKGKVGDWSNYLTPEMAARIDGLMEEKFKGTGLLEHGK

>Q42688

MAAGSVGVFATDEKIGSLLDQSITRHFLSTVTDQQGKICAEYVWIGGSMHDVRSKSRTLS
TIPTKPEDLPHWNYDGSSTGQAPGHDSEVYLIPRSIFKDPFRGGDNILVMCDCYEPPKVN
PDGTLAAPKPIPTNTRFACAEVMEKAKKEEPWFGIEQEYTLLNAITKWPLGWPKGGYPAP

QGPYYCSAGAGVAIGRDVAEVHYRLCLAAGVNISGVNAEVLPSQWEYQVGPCEGITMGDH
MWMSRYIMYRVCEMFNVEVSFDPKPIPGDWNGSGGHTNYSTKATRTAPDGWKVIQEHCAK
LEARHAVHIAAYGEGNERRLTGKHETSSMSDFSWGVANRGCSIRVGRMVPVEKSGYYEDR
RPASNLDAYVVTRLIVETTILL
>P14624
MQIFVKTLTGKTITLEVESSDTIENVKAKIQDKEGIPPDQQRLIFAGKQLEDGRTLADYN
IQKESTLHLVLRLRGG
>Q43776
MDSSVSTEPLSKNALKREKKAKEKEQLEQEKKAAAVAKRQMEQHNLPENDDLDPTQYLAN
RLRNIESLRESGINPYPHKFFITMSIPEFISRYAHLNTGEFPEDIDMSLAGRVISKRASS
SKLYFYELLGGGARVQVLASARDSDVDAVQFSNYQSGVKRGDIIGVRGYPGKSKRGELSI
FAKPFIVLAPCLHMLPRRLTSSIVDETRTQNFQGITAYDTWTPGDLRNPESYVLRDQETR
YRQRYLDLMMNPEVRALFRTRARIISYIRSFLDNLEFLEVETPSMNLTAGGASARPFITH
HNELDTELLIRVSPELYLKKLVVGGFDRVYELGKHFRNEGMDLTHSPEFTMCELYMAYAD
YNDLMDLTEQLLSGMVKDLTGSYKIRYHANGLDNEPIEIDFTPPFRKIDMLSELEKVANI
SIPRDLSSESANKHLVDVCEKFDVKCPPPHTTTRLLDKLVGHFIEVNCINPTFIINHPEI
MSPLAKSRRSEPGLTERFNLFVNRRELCDAYTELNDPTAQRERFAEQLKDRQLGDDEAMD
LDESFITALEYGLPPTGGLGMGIDRLTMLLTDSQNVKEVILFPAMRLQ
>Q9SMZ4
MNSNGHEEEKKLGNGVVGILAETVNKWERRTPLTPSHCARLLHGGKDRTGISRIVVQPSA
KRIHHDALYEDVGCEISDDLSDCGLILGIKQPELEMILPERAYAFFSHTHKAQKENMPLL
DKILSERVTLCDYELIVGDHGKRLLAFGKYAGRAGLVDFLHGLGQRKLILGYSTPFLSLG
ASYMYSSLAAAKAAVISVGEEIASQGLPLGICPLVFVFTGTGNVSLGAQEIFKLLPHTFV
EPSKLPELFVKDKGISQNGISTKRVYQVYGCIITSQDMVEHKDPSKSFDKADYYAHPEHY
NPVFHEKISPYTSVLVNCMYWEKRFPCLLSTKQLQDLTKKGLPLVGICDITCDIGGSIEF
VNRATLIDSPFFRFNPSNNSYYDDMDGDGVLCMAVDILPTEFAKEASQHFGDILSGFVGS
LASMTEISDLPAHLKRACISYRGELTSLYEYIPRMRKSNPEEAQDNIIANGVSSQRTFNI
LVSLSGHLFDKFLINEALDMIEAAGGSFHLAKCELGQSADAESYSELEVGADDKRVLDQI
IDSLTRLANPNEDYISPHREANKISLKIGKVQQENEIKEKPEMTKKSGVLILGAGRVCRP
AADFLASVRTISSQQWYKTYFGADSEEKTDVHVIVASLYLKDAKETVEGISDVEAVRLDV
SDSESLLKYVSQVDVVLSLLPASCHAVVAKTCIELKKHLVTASYVDDETSMLHEKAKSAG
ITILGEMGLDPGIDHMMAMKMINDAHIKKGKVKSFTSYCGGLPSPAAANNPLAYKFSWNP
AGAIRAGQNPAKYKSNGDIIHVDGKNLYDSAARFRVPNLPAFALECFPNRDSLVYGEHYG
IESEATTIFRGTLRYEGFSMIMATLSKLGFFDSEANQVLSTGKRITFGALLSNILNKDAD
NESEPLAGEEEISKRIIKLGHSKETAAKAAKTIVFLGFNEEREVPSLCKSVFDATCYLME
EKLAYSGNEQDMVLLHHEVEVEFLESKRIEKHTATLLEFGDIKNGQTTTAMAKTVGIPAA
IGALLLIEDKIKTRGVLRPLEAEVYLPALDILQAYGIKLMEKAE
>Q9LG26
MRRHKRWPLRSLVCSFSSSAAETVTTSTAASATAAFPLKHVTRSNFETTLNDLRSLVKAA
DFVAIDLEMTGVTSAPWRDSLEFDRYDVRYLKVKDSAEKFAVVQFGVCPFRWDSRTQSFV
SYPHNFFVFPRQELTFDPPAHEFLCQTTSMDFLAKYQFDFNTCIHEGISYLSRREEEEAS
KRLKMLHGEDGIDSSGETEELKLVRLADVLFAARMEKLLNEWRSGLLHGGNASSEFPRIS
NGSNQSMETVFHHMRPALSLKGFTSHQLRVLNSVLRKHFGDLVYIHSNDKSSSSRDIVVY
TDSDSDKENLMKEAKDERKRLAERKIQSAIGFRQVIDLLASEKKLIVGHNCFLDIAHVYS

KFVGPLPSTAEKFVASINSHFPYIVDTKILLNVNPMLHQRMKKSSTSLSSAFSSLCPQIE
FSSRSSDSFLQQRVNIDVEIDNVRCSNWNAGGKHEAGYDAFMTGCIFAQACNHLGFDFKQ
HSQLDDFAQNEKLEKYINRLYLSWTRGDIIDLRTGHSNADNWRVSKFKYENIVLIWNFPR
KLKARGIKECICKAFGSASVTSVYHVDDSAVFVLFKNSELVWDFLALKRQLESSDGPVSV
LHPLSKILEGGNTGAADYEAYKEICSSHVSEVMFSDQAETVGVKSRTRPNAQCETETREE
NTVTVTHKASDLIDAFLANRVEVETATSN
>Q93ZG7
MADTVEKVPTVVESSSSSTVEASNSAEKTEPTTEKKKWGDVEDDDDEEEAVSELNSLSIK
EEEKPDSILEEPEDSNIKAVTSGDTPYTSASRFEDLNLSPELMKGLYVEMKFEKPSKIQA
ISLPMIMTPPHKHLIAQAHNGSGKTTCFVLGMLSRVDPTLREPQALCICPTRELANQNME
VLQKMGKFTGITAELAVPDSTRGAPAATRGAPVSAHVVIGTPGTLKKWMAFKRLGLNHLK
ILVFDEADHMLATDGFRDDSLKIMKDIGRVNPNFQVLLFSATFNETVKDFVARTVKDPNQ
LFVKREDLALDSVKQYKVVCPKEQNKIEVIKDQIMELGDIGQTIIFVKTKASAQKVHKAL
AEMGYDVTSVHGNLTESDRDKIVKEFKECLTQVLIATDVIARGFDQQRVNLVVNYNLPTK
YETGEPDYEVYLHRVGRAGRFGRKGAVFNLLLDDGWDKEVMEKIEKYFEANVKEIKSWNS
EEEYKSALKEAGLLDE
>Q9SLN5
MASESDASSIATLSCARCEKPAHLQCPKCIDLKLPREQASFCTQECFKAAWSSHKSVHVK
AQLSSIGDQNSDLISQGWLYCVKKGQARTPKLPHFDWTGPLKQYPISTKRVVPAEIEKPD
WAIDGTPKVEPNSDLQHVVEIKTPEQIQRMRETCKIAREVLDAAARVIHPGVTTDEIDRV
VHEATIAAGGYPSPLNYYFFPKSCCTSVNEVICHGIPDARKLEDGDIVNVDVTVCYKGCH
GDLNETYFVGNVDEASRQLVKCTYECLEKAIAIVKPGVRFREIGEIVNRHATMSGLSVVR
SYCGHGIGDLFHCAPNIPHYARNKAVGVMKAGQTFTIEPMINAGGWRDRTWPDGWTAVTA
DGKRSAQFEHTLLVTETGVEVLTARLPSSPDVYPWLTK
>Q9SSX0
MSSAAAATRFIKCVTVGDGAVGKTCMLICYTCNKFPTDYIPTVFDNFSANVSVDGSVVNL
GLWDTAGQEDYSRLRPLSYRGADVFILSFSLISRASYENVQKKWMPELRRFAPGVPVVLV
GTKLDLREDRAYLADHPASSIITTEQGEELRKLIGAVAYIECSSKTQRNIKAVFDTAIKV
VLQPPRHKDVTRKKLQSSSNRPVRRYFCGSACFA
>Q43272
MALAGTGVFAEILDGEVYRYYADGEWRTSASGKSVAIVNPTTRKTQYRVQACTQEEVNKA
MDAAKVAQKAWARTPLWKRADVLHKAAAILKEHKAPIAECLVKEIAKPAKDAVSEVVRSG
DLVSYTAEEGVRILGEGKLVVSDSFPGNERNKYCLSSKIPLGVVLAIPPFNYPANLAGSK
IGPALIAGNALVLKPPTQGAVAALHMVHCFHLAGFPKGLISCVTGKGSEIGDFLTMHPGV
NCISFTGGDTGIAISKKAGMVPLQMELGGKDACIVLEDADLDLVSANIVKGGFSYSGQRC
TAVKVVLIMESIADAVVQKVNAKLAKLKVGPPEDDSDITPVVTESSANFIEGLVMDAKEK
GATFCQEYRREGNLIWPLLLDHVRPDMRIAWEEPFGPVLPVIRINSVEEGIHHCNASNFG
LQGCIFTRDINKAILISDAMETGTVQINSAPARGPDHFSFQGLKDSGIGSQGITNSINMM
TKVKSTVINLPSPSYTMG
>P29333
MATPALISETEAWKDLKAHLEGIKRTHLRELMGDTERCQSMMVEFDNIFLDYSRQQASPD
TINKLYKLAEAAHLKQKIDRMYNGDHINSTENRSVLHVALRAPRNSAICSDGKNVVPDVW
NVLDKIKDFSERVRNGSWVGATGKELKDVIAVGIGGSFLGPLFVHTALQTDPEASKNARG
RELRFLANVDPIDAARNISGLNPETTLVVVVSKTFTTAETMLNARTLREWISSALGVAAV

AKHMVAVSTNLPLVEKFGIDPNNAFAFWDWVGGRYSVCSAVGVLPLSLQYGFAVVEKFLQ
GAHNIDQHFSSAPFEKNIPVLLGLLSVWNVSFLGYPARAILPYSQALEKLAPHIQQVSME
SNGKGVSIDGLPLPFESGEIDFGEPGTNGQHSFYQLIHQGRVIPCDFIGVVKSQQPVYLK
GEVVNNHDELMSNFFAQPDALAYGKTPEELKKENVSEHLIPHKTFTGNRPCLSILLPTLD
AYRIGQLLAIYEHRVAVQGFVWGINSFDQWGVELGKSLATQVRKQLHASRVKGEPVEEGF
NFSTKTLLTRYLQATTDVPADPSTLLPNI
>O22424
MARGLKKHLKRLNAPKHWMLDKLGGAFAPKPSSGPHKSRECLPLILIIRNRLKYALTYRE
VISILMQRHVLVDGKVRTDKTYPAGFMDVISIPKTNENYRLLYDTKGRFRLHPIRDEDAK
FKLCKVRSVQFGQKGIPYLNTYDGRTIRYPDPLIKANDTIKIDLETNKIVDFIKFDVGNV
VMVTGGRNTGRVGVIKNREKHKGSFETIHVEDSLGHQFATRMGNVFTIGKGNKPWVSLPK
GKGIKLSIIEEQRKRDAAAQAAANA
>P53537
MGFKVETNGGDGSLVSAKVPPLANPLAEKPDEIASNISYHAQYTPHFSPFKFQLQQAYYA
TAESVRDRLIQQWNETYLHFHKVDPKQTYYLSMEFLQGRALTNAIGNLNIQDAYADALRK
FGLELEEITEQEKDAALGNGGLGRLASCFLDSMATLNLPAWGYGLRYRYGLFKQIITKEG
QEEVAEDWLEKFSPWEIVRHDVLYPIRFFGQVEVNPDGSRQWIGGEVIQALAYDVPIPGY
QTKNTISLRLWEAKACADDFDLFLFNDGQLESASVLHSRAQQICSVLYPGDATEGGKLLR
LKQQYFLCSASLQDIISRFKERRQGPWNWSEFPTKVAVQLNDTHPTLSIPELMRLLMDDE
GLGWDEAWAVTSKTVAYTNHTVLPEALEKWSQPVMWKLLPRHMEIIEEIDRRFVALISKT
RLDLEDEVSNMRILDNNLQKPVVRMANLCVVSSHTVNGVAQLHSDILKSELFASYVSIWP
TKFQNKTNGITPRRWINFCSPELSRIITKWLKTDKWVTNLDLLTGLREFADNEDLQAEWL
SAKRANKQRLAQYVLQVTGENIDPDSLFDIQVKRIHEYKRQLLNILGVIYRYKKLKEMSP
EERKSTTARTVMIGGKAFATYTNAKRIVKLVDDVGSVVNSDPEVNSYLKVVFVPNYNVSV
AEVLIPGSELSQHISTAGMEASGTSNMKFALNRVLIIGTLDGANVEIREEIGEENFFLFG
ATADEVPRLRKERENGLFKPDPRFEEAKKFIRSGVFGSYDYNPLLDSLEGNSGYGRGDYF
LVGYDFPSYMDAQEKVDEAYRDKKRWLKMSILSTAGSGKFSSDRTIAQYAKEIWNIEECR
VP
>Q8S3N1
METDSIDSVIDDDEIHQKHQFSSTKSQGGATVVISPATSVYELLECPVCTNSMYPPIHQC
HNGHTLCSTCKSRVHNRCPTCRQELGDIRCLALEKVAESLELPCKYYNLGCLGIFPYYSK
LKHESQCNFRPYSCPYAGSECAAVGDITFLVAHLRDDHKVDMHTGCTFNHRYVKSNPREV
ENATWMLTVFQCFGQYFCLHFEAFQLGMAPVYMAFLRFMGDEDDARNYTYSLEVGGSGRK
QTWEGTPRSVRDSHRKVRDSHDGLIIQRNMALFFSGGDKKELKLRVTGRIWKEQQNPDSG
VCITSMCSS
>P93026
MKLGLFTLSFLLILNLAMGRFVVEKNNLKVTSPDSIKGIYECAIGNFGVPQYGGTLVGTV
VYPKSNQKACKSYSDFDISFKSKPGRLPTFVLIDRGDCYFTLKAWIAQQAGAAAILVADS
KAEPLITMDTPEEDKSDADYLQNITIPSALITKTLGDSIKSALSGGDMVNMKLDWTESVP
HPDERVEYELWTNSNDECGKKCDTQIEFLKNFKGAAQILEKGGHTQFTPHYITWYCPEAF
TLSKQCKSQCINHGRYCAPDPEQDFTKGYDGKDVVVQNLRQACVYRVMNDTGKPWVWWDY
VTDFAIRCPMKEKKYTKECADGIIKSLGIDLKKVDKCIGDPEADVENPVLKAEQESQIGK
GSRGDVTILPTLVVNNRQYRGKLEKGAVLKAMCSGFQESTEPAICLTEDLETNECLENNG
GCWQDKAANITACRDTFRGRLCECPTVQGVKFVGDGYTHCKASGALHCGINNGGCWRESR

GGFTYSACVDDHSKDCKCPLGFKGDGVKNCEDVDECKEKTVCQCPECKCKNTWGSYECSC
SNGLLYMREHDTCIGSGKVGTTKLSWSFLWILIIGVGVAGLSGYAVYKYRIRSYMDAEIR
GIMAQYMPLESQPPNTSGHHMDI
>P42036
MSKRKTKEPKVENVTLGPAVREGEQVFGVVHVFASFNDTFIHVTDLSGRETLVRITGGMK
VKADRDESSPYAAMLAAQDVAQRCKELGITAIHVKLRATGGNKTKTPGPGAQSALRALAR
SGMKIGRIEDVTPIPTDSTRRKGGRRGRRL
>Q9LPM9
MAPSSSPLRTTSETDEKYANVKWEELGFALTPIDYMYVAKCRQGESFTQGKIVPYGDISI
SPCSPILNYGQGLFEGLKAYRTEDDRIRIFRPDQNALRMQTGAERLCMTPPTLEQFVEAV
KQTVLANKKWVPPPGKGTLYIRPLLLGSGATLGVAPAPEYTFLIYASPVGDYHKVSSGLN
LKVDHKYHRAHSGGTGGVKSCTNYSPVVKSLLEAKSAGFSDVLFLDAATGRNIEELTACN
IFIVKGNIVSTPPTSGTILPGVTRKSISELAHDIGYQVEERDVSVDELLEAEEVFCTGTA
VVVKAVETVTFHDKKVKYRTGEAALSTKLHSMLTNIQMGVVEDKKGWMVDIDPCQG
>P12670
MGYLTSSFVLFFLLCVTYTYAATIEVRNNCPYTVWAASTPIGGGRRLNRGQTWVINAPRG
TKMARIWGRTGCNFNAAGRGTCQTGDCGGVLQCTGWGKPPNTLAEYALDQFSNLDFWDIS
LVDGFNIPMTFAPTKPSGGKCHAIHCTANINGECPRALKVPGGCNNPCTTFGGQQYCCTQ
GPCGPTELSKFFKKRCPDAYSYPQDDPTSTFTCPGGSTNYRVVFCPNGVADPNFPLEMPA
STDEVAK
>Q39255
MSAKKIVLKSSDGESFEVEEAVALESQTIAHMVEDDCVDNGVPLPNVTSKILAKVIEYCK
RHVEAAASKAEAVEGAATSDDDLKAWDADFMKIDQATLFELILAANYLNIKNLLDLTCQT
VADMIKGKTPEEIRTTFNIKNDFTPEEEEEVRRENQWAFE
>P15290
MGYSKTLVAGLFAMLLLAPAVLATDPDPLQDFCVADLDGKAVSVNGHTCKPMSEAGDDFL
FSSKLAKAGNTSTPNGSAVTELDVAEWPGTNTLGVSMNRVDFAPGGTNPPHIHPRATEIG
IVMKGELLVGILGSLDSGNKLYSRVVRAGETFLIPRGLMHFQFNVGKTEASMVVSFNSQN
PGIVFVPLTLFGSNPPIPTPVLTKALRVEARVVELLKSKFAAGF
>Q9ZT50
MGLQGQLSDVSSDSIPLMLLSLLAVFINHLRSFLLRLTSKSNPNLPVDDVSIASGLANII
VLADQLSLNRLFSYRCGDGGGGGSDCVVCLSKLKEGEEVRKLECRHVFHKKCLEGWLHQF
NFTCPLCRSALVSDDCVSKTQRSVGRDLISCFSLH
>P25998
MGRLIRAQRKGAGSVFKSHTHHRKGPARFRSLDFGERNGYLKGVITEVIHDPGRGAPLAR
VTFRYPFRYNHQKELFVAAEGMYTGQFVYCGKKATLMVGNVLPLRSIPEGAVVCNVEHKV
GDRGVFARCSGAYAIVISHNPDNGTTRIKLPSGSKKIVPSGCRRMIGQVAGGGRTEKPML
KAGNAYHKYRVKRNCWPKVRGVAMNPVEHPHGGGNHQHIGHASTVARDAPPGQKVGLIAG
RRTGRLRGQAAATAAKADKA
>A2WWV5
MAGSGVLEVHLVDAKGLTGNDFLGEIGKIDPYVVVQYRSQERKSSVARDQGKNPSWNEVF
KFQINSTAATGQHKLFLRLMDHDTFSRDDFLGEATINVTDLISLGMEHGTWEMSESKHRV
VLADKTYHGEIRVSLTFTASAKAQDHAEQVGGWAHSFRQ
>Q96301

MVGLEDDTERERSPVVENGFSNGSRSSSSSAGVLSPSRKVTQGNDTLSYANILRARNKFA
DALALYEAMLEKDSKNVEAHIGKGICLQTQNKGNLAFDCFSEAIRLDPHNACALTHCGIL
HKEEGRLVEAAESYQKALMADASYKPAAECLAIVLTDLGTSLKLAGNTQEGIQKYYEALK
IDPHYAPAYYNLGVVYSEMMQYDNALSCYEKAALERPMYAEAYCNMGVIYKNRGDLEMAI
TCYERCLAVSPNFEIAKNNMAIALTDLGTKVKLEGDVTQGVAYYKKALYYNWHYADAMYN
LGVAYGEMLKFDMAIVFYELAFHFNPHCAEACNNLGVLYKDRDNLDKAVECYQMALSIKP
NFAQSLNNLGVVYTVQGKMDAAASMIEKAILANPTYAEAFNNLGVLYRDAGNITMAIDAY
EECLKIDPDSRNAGQNRLLAMNYINEGLDDKLFEAHRDWGWRFTRLHPQYTSWDNLKDPE
RPITIGYISPDFFTHSVSYFIEAPLTHHDYTKYKVVVYSAVVKADAKTYRFRDKVLKKGG
VWKDIYGIDEKKIASMVREDKIDILVELTGHTANNKLGTMACRPAPVQVTWIGYPNTTGL
PTVDYRITDSLADPPDTKQKQVEELVRLPDCFLCYTPSPEAGPVCPTPALSNGFVTFGSF
NNLAKITPKVLQVWARILCAVPNSRLVVKCKPFCCDSIRQRFLTTLEQLGLESKRVDLLP
LILFNHDHMQAYSLMDISLDTFPYAGTTTTCESLYMGVPCVTMAGSVHAHNVGVSLLTKV
GLGHLVAKNEDEYVQLSVDLASDVTALSKLRMSLRDLMAGSPVCNGPSFAVGLESAYRNM
WKKYCKGEVPSLRRMEMLQKEVHDDPLISKDLGPSRVSVTGEATPSLKANGSAPVPSSLP
TQSPQLSKRMDSTS

>Q43139

MDHAADAHRTDLMTITRHVLNEQSRNPESRGDFTILLSHIVLGCKFVASAVNKAGLAQLI
GLAGETNVQGEEQKKLDVLSNEVFVKALVSSGRTCVLVSEEDEETTFVDPKLRGKYCVCF
DPLDGSSNIDCGVSIGTIFGIYMIKDKDNVTLSDVLQPGKDMLAAGYCMYGSSCTLVLST
GTGVNGFTLDPSLGEFILTHPDIKIPKKGKIYSVNEGNAKNWDVPVAKFVEKCKYPKDGS
PPKSLRYIGSMVADVHRTLLYGGVFLYPADQKSPNGKLRVLYEVFPMSFLMEQAGGQSFT
GKERALDLVPTKIHERSPIFLGSYDDVEEIKALYAEQAKSSSA

>Q06197

MAAFQKIKVANPIVEMDGDEMTRVIWKSIKDKLILPFLELDIKYYDLGLPYRDETDDKVT
IESAEATLKYNVAIKCATITPDEARVKEFGLKSMWKSPNGTIRNILNGTVFREPILCKNI
PRLVPGWTKAICIGRHAFGDQYRATDTVIKGAGKLKLVFVPEGQGEETEFEVFNFTGEGG
VSLAMYNTDESIRSFAEASMATALEKKWPLYLSTKNTILKKYDGRFKDIFQEVYEASWKS
KFEAAGIWYEHRLIDDMVAYALKSEGGYVWACKNYDGDVQSDFLAQGFGSLGLMTSVLVC
PDGKTIEAEAAHGTVTRHFRVHQKGGETSTNSIASIFAWTRGLAHRAKLDDNAKLLDFTE
KLEAACIGVVEAGKMTKDLALILHGSKLSREHYLNTEEFIDAVAAELSARLSA

>P48578

MGANSLPTDATLDLDEQISQLMQCKPLSEQQVRALCEKAKEILMDESNVQPVKSPVTICG
DIHGQFHDLAELFRIGGKCPDTNYLFMGDYVDRGYYSVETVTLLVGLKVRYPQRITILRG
NHESRQITQVYGFYDECLRKYGNANVWKIFTDLFDYFPLTALVESEIFCLHGGLSPSIET
LDNIRNFDRVQEVPHEGPMCDLLWSDPDDRCGWGISPRGAGYTFGQDISEQFNHTNNLKL
IARAHQLVMDGFNWAHEQKVVTIFSAPNYCYRCGNMASILEVDDCRNHTFIQFEPAPRRG
EPDVTRRTPDYFL

>O04147

MEEVKKDVYSVWALPDEESEPRFKKLMEALRSEFTGPRFVPHVTVAVSAYLTADEAKKMF
ESACDGLKAYTATVDRVSTGTFFFQCVFLLLQTTPEVMEAGEHCKNHFNCSTTTPYMPHL
SLLYAELTEEEKKNAQEKAYTLDSSLDGLSFRLNRLALCKTDTEDKTLETWETVAVCNLN
P

>Q84JU6

MDTREINGFASAARSISLPTQPNYSSKPVQEALKHLASINLRELCNEAKVERCRATRDLA
SCGRFVNYVLNPCGHASLCTECCQRCDVCPICRSTLPKFGDRLRLRLYYECVEAGLISRT
HEEASQDSDEDEHQLAADVHRLYSLFDVAMNNNLISVVCHYITNVCMDETAVSSDPVIAF
LLDEVVVKDWVKRTFRSTLAELQEIYNLETKEMQAWLDKLLRCSKQVAGICSVLEVMESA
FKGSVSPQLQDVQTLRENIGKTKQHLDIMVWCIRHGFLDDVRSRYSNFTSWNALVGERKS
NAVKRAWPDAVDQSSDCSVQSASLFIEDALENLEREPEYSQEIGADLEVGRLQKDKRSFL
RSKIEGTSGSYPFENLRTAADMLFLHGGSDLVVAKQAIFLYYLFDRHWTTPEKYWKHTID
DFAATFGITRHSLLESFVFYLLDDHSEEALQEACRILPEICGPETYPKVAQVLLERDNPE
TALMVLRWSGRDGVSELVSIGEAVTALRVRVECGLLSEAFTYQRTLCLKVKENNLKNGAV
KHASDDLDIWSWTEWMEILVNEFCCLSIRRNLVDRIIELPWNPDEEKYLHRCLLDSATDD
PSSAVGSLLVVFYLQRYRYIQAYQVDLRLQKIEEAFVSDNQIGEEVMFRMRSQSHWRKEL
VDRAIDILPVIQQQQVRSGQFSEMEDASEGAKKSDLPDAPDMITSSVPFATTNSVFLQSA
NNARAREPVANNGSPFQPGHMIGNASHDLSHGRLFTNANRGQKSEVRSVTKNLKFGEMST
PFKDLNRARGNSQLQGKRTEESSPEVNVDRYIENNMSSPYLRRITANNPVTVKSSSNHLN
GSSQKPESTFFGTRMQPDKDNFVDLDDPMDMSSSLKDNNNNVLATESRNNSGGLRWRSDE
TSDDEDELTSFGSMPVKGRRRRRFAAR
>Q8W2B8
MACINGENRDFSSSSSLSSLPMIVSRNFSARDDGETGDEFPFERIFPVYARGTLNPVADP
VLLDFTNSSYDPIWDSIREEAKLEAEEEPVLSSFLYASILSHDCLEQALSFVLANRLQNP
TLLATQLMDIFCNVMVHDRGIQSSIRLDVQAFKDRDPACLSYSSAILHLKGYLALQAYRV
AHKLWKQGRKLLALALQSRVSEVFGIDIHPAARIGKGILLDHGTGVVIGETAVIGDRVSI
LHGVTLGGTGKETGDRHPNIGDGALLGACVTILGNIKIGAGAMVAAGSLVLKDVPSHSMV
AGNPAKLIGFVDEQDPSMTMEHDATREFFQNVAVAYRETIPNGSSVSGSCRERRH
>O04433
MVVLQPDPFLSELTSMYERSTEKGSVWVTMKRSSMKCQARLKKMAAKGEAVEYRCLVRAT
DGKKNICTALSAKEYLKFQASYATVLKAHMHALKKRERKDKKKAAEVEKIPEKAPKKQKK
APSSKKSAGSKS
>Q9ZWR8
MEQIQMVKVLEKCQVTPPSDTTDVELSLPVTFFDIPWLHLNKMQSLLFYDFPYPRTHFLD
TVIPNLKASLSLTLKHYVPLSGNLLMPIKSGEMPKFQYSRDEGDSITLIVAESDQDFDYL
KGHQLVDSNDLHGLFYVMPRVIRTMQDYKVIPLVAVQVTVFPNRGIAVALTAHHSIADAK
SFVMFINAWAYINKFGKDADLLSANLLPSFDRSIIKDLYGLEETFWNEMQDVLEMFSRFG
SKPPRFNKVRATYVLSLAEIQKLKNKVLNLRGSEPTIRVTTFTMTCGYVWTCMVKSKDDV
VSEESSNDENELEYFSFTADCRGLLTPPCPPNYFGNCLASCVAKATHKELVGDKGLLVAV
AAIGEAIEKRLHNEKGVLADAKTWLSESNGIPSKRFLGITGSPKFDSYGVDFGWGKPAKF
DITSVDYAELIYVIQSRDFEKGVEIGVSLPKIHMDAFAKIFEEGFCSLS
>Q9FML6
MAINEPEFATAVAVATKKRTFKKFSFRGFNVDALLKMSNVDLAKLFNARVRRRFYRGLKK
QPLILIKKLRRAKKEASDENKMKPEVVKTHLRNMIIVPEMIGSVVGVHNGKKFNEIVIKP
EMIGHYLAEFSMTCKKVNHHRPRICGCCCFRRSTRFIPLR
>Q42484
MDFISSLIVGCAQVLCESMNMAERRGHKTDLRQAITDLETAIGDLKAIRDDLTLRIQQDG
LEGRSCSNRAREWLSAVQVTETKTALLLVRFRRREQRTRMRRRYLSCFGCADYKLCKKVS
AILKSIGELRERSEAIKTDGGSIQVTCREIPIKSVVGNTTMMEQVLEFLSEEEERGIIGV

YGPGGVGKTTLMQSINNELITKGHQYDVLIWVQMSREFGECTIQQAVGARLGLSWDEKET
GENRALKIYRALRQKRFLLLLDDVWEEIDLEKTGVPRPDRENKCKVMFTTRSIALCNNMG
AEYKLRVEFLEKKHAWELFCSKVWRKDLLESSSIRRLAEIIVSKCGGLPLALITLGGAMA
HRETEEEWIHASEVLTRFPAEMKGMNYVFALLKFSYDNLESDLLRSCFLYCALFPEEHSI
EIEQLVEYWVGEGFLTSSHGVNTIYKGYFLIGDLKAACLLETGDEKTQVKMHNVVRSFAL
WMASEQGTYKELILVEPSMGHTEAPKAENWRQALVISLLDNRIQTLPEKLICPKLTTLML
QQNSSLKKIPTGFFMHMPVLRVLDLSFTSITEIPLSIKYLVELYHLSMSGTKISVLPQEL
GNLRKLKHLDLQRTQFLQTIPRDAICWLSKLEVLNLYYSYAGWELQSFGEDEAEELGFAD
LEYLENLTTLGITVLSLETLKTLFEFGALHKHIQHLHVEECNELLYFNLPSLTNHGRNLR
RLSIKSCHDLEYLVTPADFENDWLPSLEVLTLHSLHNLTRVWGNSVSQDCLRNIRCINIS
HCNKLKNVSWVQKLPKLEVIELFDCREIEELISEHESPSVEDPTLFPSLKTLRTRDLPEL
NSILPSRFSFQKVETLVITNCPRVKKLPFQERRTQMNLPTVYCEEKWWKALEKDQPNEEL
CYLPRFVPN
>Q9C5X3
MSYSDSDSSSHGGEYKNFRQITRERLLYEMLRSAKTGSSKSTWKVLIMDKLTVKIMSYAC
KMADITQEGVSLVEDIFRRRQPLPSMDAIYFIQPTKENVIMFLSDMSGKSPLYKKAFVFF
SSPVSKELVGHIKKDSSVLPRIGALREMNLEFFAIDSQGFITDHERALEDLFGDEETSRK
GDACLNVMASRIATVFASLREFPAVRYRAAKSLDASTMTTLRDLIPTKLAAGIWNCLAKH
KQSIENFPQTETCELLILDRSIDQIAPVIHEWTYDAMCHDLLNMEGNKYVHVIPSKSGGQ
PEKKDVLLEEHDPIWLELRHAHIADASERLHDKMTNFLSKNKAAQLQGKRDGAELSTRDL
QKMVQALPQYSEQIDKLSLHVEIARKLNDLIREQGLRELGQLEQDLVFGDAGMKDVIKYL
STQEEASREGKLRLLMILATIYPEKFEGEKGQNLMKLAKLSSDDMTAVNNMSLLGSAVDA
KKNTPGGFTLKFDLHKKKRAVRKERQEEAAWQLSRFYPMIEELIEKLSKGELPKEDFPCM
NDPSPSFHGSTSLSSAASSSQGQAAQSMRSRRTPTWAKPRGSDDGYSSDSVLRHASSDFR
KMGQRIFVFIVGGATRSELKVCHKLSTKLKREVILGSTSLDDPPQFITKLKLLTANDDLS
LDDLQI
>Q9C5X4
MACFSNETQIEIDVHDLVEAPIRYDSIESIYSIPSSALCCVNAVGSHSLMSKKVKAQKLP
MIEQFEIEGSGVSASDDCCRSDDYKLRIQRPEIVRVYYRRRKRPLRECLLDQAVAVKTES
VELDEIDCFEEKKRRKIGNCELVKSGMESIGLRRCKENNAFSGNKQNGSSRRKGSSSKNQ
DKATLASRSAKKWVRLSYDGVDPTSFIGLQCKVFWPLDALWYEGSIVGYSAERKRYTVKY
RDGCDEDIVFDREMIKFLVSREEMELLHLKFCTSNVTVDGRDYDEMVVLAATLDECQDFE
PGDIVWAKLAGHAMWPAVIVDESIIGERKGLNNKVSGGGSLLVQFFGTHDFARIKVKQAI
SFIKGLLSPSHLKCKQPRFEEGMQEAKMYLKAHRLPERMSQLQKGADSVDSDMANSTEEG
NSGGDLLNDGEVWLRPTEHVDFRHIIGDLLIINLGKVVTDSQFFKDENHIWPEGYTAMRK
FTSLTDHSASALYKMEVLRDAETKTHPLFIVTADSGEQFKGPTPSACWNKIYNRIKKVQN
SDSPNILGEELNGSGTDMFGLSNPEVIKLVQDLSKSRPSSHVSMCKNSLGRHQNQPTGYR
PVRVDWKDLDKCNVCHMDEEYENNLFLQCDKCRMMVHAKCYGELEPCDGALWLCNLCRPG
APDMPPRCCLCPVVGGAMKPTTDGRWAHLACAIWIPETCLSDVKKMEPIDGVNKVSKDRW
KLMCTICGVSYGACIQCSNNSCRVAYHPLCARAAGLCVELENDMSVEGEEADQCIRMLSF
CKRHRQTSTACLGSEDRIKSATHKTSEYLPPPNPSGCARTEPYNCFGRRGRKEPEALAAA
SSKRLFVENQPYVIGGYSRLEFSTYKSIHGSKVSQMNTPSNILSMAEKYRYMRETYRKRL
AFGKSGIHGFGIFAKLPHRAGDMMIEYTGELVRPSIADKREQLIYNSMVGAGTYMFRIDD
ERVIDATRTGSIAHLINHSCVPNCYSRVITVNGDEHIIIFAKRHIPKWEELTYDYRFFSI

GERLSCSCGFPGCRGVVNDTEAEEQHAKICVPRCDLIDWTAE
>O64394
MAASAATATATAAAVGAGEVISVHSLEQWTMQIEEANAAKKLVVIDFTASWCGPCRIMAP
IFADLAKKFPAAVFLKVDVDELKPIAEQFSVEAMPTFLFMKEGDVKDRVVGAIKEELTTK
VGLHAAQ
>Q943Z6
MDSGTINIKKWVVIYPVYINSKKTVAEGRRISVSKSCENPNCIEISDCCKHLKLPSAVEI
DKAYPRDFMQVGRVRVQLKREDGTLLNPAITSRKHLMQKIAELVPRHPERVKKQEAQKAK
KQEPQATTSTSGTSSKSGKGGKKKR
>Q9LV58
MPSRYPGAVTQDWEPVVLHKSKQKSQDLRDPKAVNAALRNGVAVQTVKKFDAGSNKKGKS
TAVPVINTKKLEEETEPAAMDRVKAEVRLMIQKARLEKKMSQADLAKQINERTQVVQEYE
NGKAVPNQAVLAKMEKVLGVKLRGKIGK
>Q04522
MTIKVHGNPRSTATQRVLVALYEKHLEFEFVPIDMGAGGHKQPSYLALNPFGQVPALEDG
EIKLFESRAITKYLAYTHDHQNEGTSLIHKEKHEMAAQLVWEEVEAHQFDPVASKLAWEL
VFKGIFGMQTDTTVVEENEAKLAKVLDVYEARLTESEYLGANDSFTLVDLHHLPLLGYLM
GTQVKKLFEERAHVSAWCKKILARPSWEKTLALQKQA
>Q8RY95
MDEVGAQVAAPMFIHQSLGRKRDLYYPMSNRLVQSQPQRRDEWNSKMWDWDSRRFEAKPV
DVEVQEFDLTLRNRSGEERGLDLNLGSGLTAVEETTTTTQNVRPNKKVRSGSPGGNYPMC
QVDNCTEDLSHAKDYHRRHKVCEVHSKATKALVGKQMQRFCQQCSRFHLLSEFDEGKRSC
RRRLAGHNRRRRKTTQPEEVASGVVVPGNHDTTNNTANANMDLMALLTALACAQGKNAVK
PPVGSPAVPDREQLLQILNKINALPLPMDLVSKLNNIGSLARKNMDHPTVNPQNDMNGAS
PSTMDLLAVLSTTLGSSSPDALAILSQGGFGNKDSEKTKLSSYENGVTTNLEKRTFGFSS
VGGERSSSSNQSPSQDSDSRGQDTRSSLSLQLFTSSPEDESRPTVASSRKYYSSASSNPV
EDRSPSSSPVMQELFPLQASPETMRSKNHKNSSPRTGCLPLELFGASNRGAADPNFKGFG
QQSGYASSGSDYSPPSLNSDAQDRTGKIVFKLLDKDPSQLPGTLRSEIYNWLSNIPSEME
SYIRPGCVVLSVYVAMSPAAWEQLEQKLLQRLGVLLQNSPSDFWRNARFIVNTGRQLASH
KNGKVRCSKSWRTWNSPELISVSPVAVVAGEETSLVVRGRSLTNDGISIRCTHMGSYMAM
EVTRAVCRQTIFDELNVNSFKVQNVHPGFLGRCFIEVENGFRGDSFPLIIANASICKELN
RLGEEFHPKSQDMTEEQAQSSNRGPTSREEVLCFLNELGWLFQKNQTSELREQSDFSLAR
FKFLLVCSVERDYCALIRTLLDMLVERNLVNDELNREALDMLAEIQLLNRAVKRKSTKMV
ELLIHYLVNPLTLSSSRKFVFLPNITGPGGITPLHLAACTSGSDDMIDLLTNDPQEIGLS
SWNTLRDATGQTPYSYAAIRNNHNYNSLVARKLADKRNKQVSLNIEHEVVDQTGLSKRLS
LEMNKSSSSCASCATVALKYQRRVSGSQRLFPTPIIHSMLAVATVCVCVCVFMHAFPIVR
QGSHFSWGGLDYGSI
>Q94AH6
MERKTIDLEQGWDYMQTGITKLKRILEGLNEPAFDSEQYMMLYTTIYNMCTQKPPHDYSQ
QLYDKYREAFEEYINSTVLPALREKHDEFMLRELFKRWSNHKVMVRWLSRFFYYLDRYFI
ARRSLPPLNEVGLTCFRDLVYNELHSKVKQAVIALVDKEREGEQIDRALLKNVLDIYVEI
GMGQMERYEEDFESFMLQDTSSYYSRKASSWIQEDSCPDYMLKSEECLKKERERVAHYLH
SSSEPKLVEKVQHELLVVFASQLLEKEHSGCRALLRDDKVDDLSRMYRLYHKILRGLEPV
ANIFKQHVTAEGNALVQQAEDTATNQVANTASVQEQVLIRKVIELHDKYMVYVTECFQNH

TLFHKALKEAFEIFCNKTVAGSSSAELLATFCDNILKKGGSEKLSDEAIEDTLEKVVKLL
AYISDKDLFAEFYRKKLARRLLFDRSANDDHERSILTKLKQQCGGQFTSKMEGMVTDLTL
ARENQNSFEDYLGSNPAANPGIDLTVTVLTTGFWPSYKSFDINLPSEMIKCVEVFKGFYE
TKTKHRKLTWIYSLGTCHINGKFDQKAIELIVSTYQAAVLLLFNTTDKLSYTEILAQLNL
SHEDLVRLLHSLSCAKYKILLKEPNTKTVSQNDAFEFNSKFTDRMRRIKIPLPPVDERKK
VVEDVDKDRRYAIDAAIVRIMKSRKVLGHQQLVSECVEQLSRMFKPDIKAIKKRMEDLIT
RDYLERDKENPNMFRYLA

>Q9LN63

MTSDGATSTSAAAAAAAMATRRKPSWRERENNRRRERRRRAVAAKIYTGLRAQGNYNLPK
HCDNNEVLKALCSEAGWVVEEDGTTYRKGHKPLPGDMAGSSSRATPYSSHNQSPLSSTFD
SPILSYQVSPSSSSFPSPSRVGDPHNISTIFPFLRNGGIPSSLPPLRISNSAPVTPPVSS
PTSRNPKPLPTWESFTKQSMSMAAKQSMTSLNYPFYAVSAPASPTHHRQFHAPATIPECD
ESDSSTVDSGHWISFQKFAQQQPFSASMVPTSPTFNLVKPAPQQLSPNTAAIQEIGQSSE
FKFENSQVKPWEGERIHDVAMEDLELTLGNGKAHS

>Q6EUP4

MSQPAELSREENVYMAKLAEQAERYEEMVEFMEKVAKTVDSEELTVEERNLLSVAYKNVI
GARRASWRIISSIEQKEESRGNEDRCTLIKEYRGKIETELSKICDGILKLLDSHLVPSST
APESKVFYLKMKGDYYRYLAEFKTGAERKDAAENTMVAYKAAQDIALAELPPTHPIRLGL
ALNFSVFYYEILNSPDRACNLAKQAFDEAISELDTLSEESYKDSTLIMQLLRDNLTLWTS
DISEDAAEEIKEAPKGESGDGQ

>A2ZB00

MAAALTRPPPGTVQCFGRKKTAVAVSYCKPGRGLIKVNGVPIELIRPEMLRLKAFEPILL
AGRSRFKDIDMRIRVRGGGKTSQIYAIRQAIAKALVAYYQKYVDEASKKEVKDIFARYDR
TLLVADPRRCEPKKFGGRGARARFQKSYR

>O04005

MPGITLGDTVPNLEVETTHDKFKLHDYFANSWTVLFSHPGDFTPVCTTELGAMAKYAHEF
DKRGVKLLGLSCDDVQSHKDWIKDIEAFNHGSKVNYPIIADPNKEIIPQLNMIDPIENGP
SRALHIVGPDSKIKLSFLYPSTTGRNMDEVLRALDSLLMASKHNNKIATPVNWKPDQPVV
ISPAVSDEEAKKMFPQGFKTADLPSKKGYLRHTEVS

>P14713

MVSGVGGSGGGRGGGRGGEEEPSSSHTPNNRRGGEQAQSSGTKSLRPRSNTESMSKAIQQ
YTVDARLHAVFEQSGESGKSFDYSQSLKTTTYGSSVPEQQITAYLSRIQRGGYIQPFGCM
IAVDESSFRIIGYSENAREMLGIMPQSVPTLEKPEILAMGTDVRSLFTSSSSILLERAFV
AREITLLNPVWIHSKNTGKPFYAILHRIDVGVVIDLEPARTEDPALSIAGAVQSQKLAVR
AISQLQALPGGDIKLLCDTVVESVRDLTGYDRVMVYKFHEDEHGEVVAESKRDDLEPYIG
LHYPATDIPQASRFLFKQNRVRMIVDCNATPVLVVQDDRLTQSMCLVGSTLRAPHGCHSQ
YMANMGSIASLAMAVIINGNEDDGSNVASGRSSMRLWGLVVCHHTSSRCIPFPLRYACEF
LMQAFGLQLNMELQLALQMSEKRVLRTQTLLCDMLLRDSPAGIVTQSPSIMDLVKCDGAA
FLYHGKYYPLGVAPSEVQIKDVVEWLLANHADSTGLSTDSLGDAGYPGAAALGDAVCGMA
VAYITKRDFLFWFRSHTAKEIKWGGAKHHPEDKDDGQRMHPRSSFQAFLEVVKSRSQPWE
TAEMDAIHSLQLILRDSFKESEAAMNSKVVDGVVQPCRDMAGEQGIDELGAVAREMVRLI
ETATVPIFAVDAGGCINGWNAKIAELTGLSVEEAMGKSLVSDLIYKENEATVNKLLSRAL
RGDEEKNVEVKLKTFSPELQGKAVFVVVNACSSKDYLNNIVGVCFVGQDVTSQKIVMDKF
INIQGDYKAIVHSPNPLIPPIFAADENTCCLEWNMAMEKLTGWSRSEVIGKMIVGEVFGS

CCMLKGPDALTKFMIVLHNAIGGQDTDKFPFPFFDRNGKFVQALLTANKRVSLEGKVIGA
FCFLQIPSPELQQALAVQRRQDTECFTKAKELAYICQVIKNPLSGMRFANSLLEATDLNE
DQKQLLETSVSCEKQISRIVGDMDLESIEDGSFVLKREEFFLGSVINAIVSQAMFLLRDR
GLQLIRDIPEEIKSIEVFGDQIRIQQLLAEFLLSIIRYAPSQEWVEIHLSQLSKQMADGF
AAIRTEFRMACPGEGLPPELVRDMFHSSRWTSPEGLGLSVCRKILKLMNGEVQYIRESER
SYFLIILELPVPRKRPLSTASGSGDMMLMMPY
>Q9FVX0
MSSSSRNLSQENPIPRPNLAKTRTSLRDVGNRRAPLGDITNQKNGSRNPSPSSTLVNCSN
KIGQSKKAPKPALSRNWNLGILDSGLPPKPNAKSNIIVPYEDTELLQSDDSLLCSSPALS
LDASPTQSDPSISTHDSLTNHVVDYMVESTTDDGNDDDDDEIVNIDSDLMDPQLCASFAC
DIYEHLRVSEVNKRPALDYMERTQSSINASMRSILIDWLVEVAEEYRLSPETLYLAVNYV
DRYLTGNAINKQNLQLLGVTCMMIAAKYEEVCVPQVEDFCYITDNTYLRNELLEMESSVL
NYLKFELTTPTAKCFLRRFLRAAQGRKEVPSLLSECLACYLTELSLLDYAMLRYAPSLVA
ASAVFLAQYTLHPSRKPWNATLEHYTSYRAKHMEACVKNLLQLCNEKLSSDVVAIRKKYS
QHKYKFAAKKLCPTSLPQELFL
>Q8LGE3
MSNIVVLDNGGGLIKAGQGGERDPTTVIPNCLYKPLSSKKFIHPSPLTTLSDEIDLTSAA
VRRPIDRGYLINSDLQREIWSHLFTSLLHIAPSSSSLLLTEAPLSIPSVQRTTDELVFED
FGFSSLYIAHPQSLVHLYEASRQPDSILSKTQCSLVVDCGFSFTHAVPVLHNFTLNHAIK
RIDLGGKAFTNYLKELVSYRSINVMDETFLVDDAKEKLCFVSLDLLRDLRLARNGNTLIK
STYVLPDGVTHTKGYVKDPQAAKRFLSLSEKESVVVMDKVGERKKADMNKNEIDLTNERF
LVPETLFQPADLGMNQAGLAECIVRAINSCHSYLQPVLYQSIILTGGSTLFPQLKERLEG
ELRPLVPDHFDVKITTQEDPILGVWRGGSLLASSPDFESMCVTKAEYEELGSARCRRRFF
H
>P33444
MAVGKNKRISKGKKGGKKKAADPFAKKDWYDIKAPSVFSVRNVGKTLVTRTQGTKIASEG
LEHRVFEISLADLQGDEDHSFRKIRLRAEDIQGKNVLTNFWGMDFTTDKLRSLVRKWQSL
IEAHVDVKTTDSYTLRMFCIGFTKKRANQQKRTCYAQSSQIRQIRRKMREIMVNQAQSCD
LKDLVQKFIPESIGREIEKATSSIYPLQNVFIRKVKILKAPKFDLGKLMEVHGDYNEDIG
TKLDRPAEEAVAEPTEVIGA
>Q8GZA8
MANNEGEMQCGSMLFKQEELQEMSGVNVGGDYVEVMCGCTSHRYGDAVARLRVFPTGDLE
ITCECTPGCDEDKLTPAAFEKHSGRETARKWKNNVWVIIGGEKVPLSKTVLLKYYNESSK
KCSRSNRSQGAKVCHRDEFVGCNDCGKERRFRLRSRDECRLHHNAMGDPNWKCSDFPYDK
ITCEEEEERGSRKVYRGCTRSPSCKGCTSCVCFGCELCRFSECTCQTCVDFTSNVKA
>P28769
MSISAQNPDISGDRQSGQDVRTQNVMACQAVSNIVKTSLGPVGLDKMLVDDIGDVTITND
GATILRMLEVEHPAAKVLVELAELQDREVGDGTTSVVIVAAELLKRANDLVRNKIHPTSI
ISGYRLAMRESCKYIEEKLVTKVEKLGKVPLINCAKTSMSSKLISGDSDFFANLVVEAVL
SVKMTNQRGEIKYPIKGINILKAHGQSARDSYLLNGYALNTGRAAQGMPLRVSPAKIACL
DFNLQKTKMQLGVQVVVNDPRELEKIRQREADMTKERIEKLLKAGANVILTTKGIDDMAL
KYFVEAGAIAVRRVRKEDMRHVAKATGATLVTTFADMEGEETFDPAHLGSADEVVEERIA
DDDVILIKGTKTSSAVSLILRGANDYMLDEMERALHDALCIVKRTLESNTVVAGGGAVES
ALSVYLEHLATTLGSREQLAIAEFADALLIIPKVLAVNAAKDATELVAKLRAYHHTAQTK

ADKKHYSSMGLDLVNGTIRNNLEAGVIEPAMSKVKIIQFATEAAITILRIDDMIKLVKDE
SQGEE
>P22738
MSHRKFEHPRHGSLGFLPRKRASRHRGKVKAFPKDDPTKPCRLTSFLGYKAGMTHIVRDV
EKPGSKLHKKETCEAVTIIETPPMVVVGVVGYVKTPRGLRSLCTVWAQHLSEELRRRFYK
NWAKSKKKAFTRYSKKHETEEGKKDIQSQLEKMKKYCSVIRVLAHTQIRKMKGLKQKKAH
LNEIQINGGDIAKKVDYACSLFEKQVPVDAIFQKDEMIDIIGVTKGKGYEGVVTRWGVTR
LPRKTHRGLRKVACIGAWHPARVSYTVARAGQNGYHHRTEMNKKVYRVGKVGQETHSAMT
EYDRTEKDITPMGGFPHYGIVKEDYLMIKGCCVGPKKRVVTLRQTLLKQTSRLAMEEIKL
KFIDAASNGGHGRFQTSQEKAKFYGRTIKA
>P37223
MGGSNALNEMTNGSDGITGGVADVYGEEFATQDQLVTPWSFSVACGHSLLRDPQHNKGLA
FTEKERDAHFLRGLLPPVVLSQELQEKKFLTTLRQYQVPLQKYMAMMDLQERNEKLFYKL
LVDHVEELLPLVYTPTVGEGCQKYGSIFRRPQGLFISLKDKGRILELLRNWPEKKIQVIV
VTDGERILGLGDLGCQGMGIPVGKLSLYSALGGVCPSACLPITLDVGTNNQKLLDDEFYI
GLKQKRATGEEYAEFVQEFMSAVKQNYGEKILVQFEDFANHNAFELLEKYRTTHLVFNDD
IQGTASVVLAGLIASLKLLGGTLADHKFLFLGAGEAGTGIAELIALEMSKKTKAPVEQMR
KKIWLVDSKGLVVSSRKETLQQFKLPWAHEHEPITTLIDAVQAIKPTVLIGTSGKGKQFT
KEVVEAMANINAKPLILALSNPTSQSECTAEEAYTWSQGHAIFASGSPFDPVEYEGRTFV
PGQANNAYIFPGFGLGLIMCGAIRVHDDMLLAASEALASQVTGEHFIKGLIYPPFKDIRK
ISAHIAAGVAAKAYELGLASRLPQPADLVKFAESCMYNPTYRSFR
>Q94AA4
MSTVESSKPKIINGSCGYVLEDVPHLSDYLPGLPTYPNPLQDNPAYSVVKQYFVDADDSV
PQKIVVHKDGPRGIHFRRAGPRQKVYFESDEVHACIVTCGGLCPGLNTVIREIVSSLSYM
YGVKRILGIDGGYRGFYAKNTVSLDSKVVNDIHKRGGTILGTSRGGHDTTKIVDSIQDRG
INQVYIIGGDGTQRGASVIFEEIRRRGLKVAVIGIPKTIDNDIPVIDKSFGFDTAVEEAQ
RAINAAHVEAESIENGIGVVKLMGRYSGFIAMYATLASRDVDCCLIPESPFYLEGEGGLF
EYIEKRLKESGHMVLVIAEGAGQDLMSKSMESMTLKDASGNKLLKDVGLWLSQSIKDHFN
QKKMVMNLKYIDPTYMIRAVPSNASDNVYCTLLAQSAVHGAMAGYTGYISGLVNGRQTYI
PFYRITEKQNHVVITDRMWARLLSSTNQPSFLGPKDVFDNKEKPMSALLDDGNCNGVVDV
PPVTKEITK
>P93836
MGHQNAAVSENQNHDDGAASSPGFKLVGFSKFVRKNPKSDKFKVKRFHHIEFWCGDATNV
ARRFSWGLGMRFSAKSDLSTGNMVHASYLLTSGDLRFLFTAPYSPSLSAGEIKPTTTASI
PSFDHGSCRSFFSSHGLGVRAVAIEVEDAESAFSISVANGAIPSSPPIVLNEAVTIAEVK
LYGDVVLRYVSYKAEDTEKSEFLPGFERVEDASSFPLDYGIRRLDHAVGNVPELGPALTY
VAGFTGFHQFAEFTADDVGTAESGLNSAVLASNDEMVLLPINEPVHGTKRKSQIQTYLEH
NEGAGLQHLALMSEDIFRTLREMRKRSSIGGFDFMPSPPPTYYQNLKKRVGDVLSDDQIK
ECEELGILVDRDDQGTLLQIFTKPLGDRPTIFIEIIQRVGCMMKDEEGKAYQSGGCGGFG
KGNFSELFKSIEEYEKTLEAKQLVG
>Q9LZ98
MAVTEEEKKRNSTVEISGLRFTYPGIDGHPPPGSKPLIEDFSITLNSSDRCLLVGSNGAG
KTTILKILGGKHMVEPHMVRVLGRSAFHDTGLTSSGDLCYLGGEWRRDVAFAGFEVPIQM
DISAEKMIFGVAGIDPQRRDELIKVLDIDISWRLHKVSDGQRRRVQICMGLLKPFKVLLL

DEITVDLDVLARADLLKFLRKECEERGATIIYATHIFDGLEDWPTHIVYVANGKLQLALP
MEKVKETSKKSLMRTVESWLRKERDEERKRRKERKANGLPEFETRTEESRVTGDPARMLN
NGWAAGRLHSTVAGGEDNFVLSSNRVLR
>P36428
MRLVKAASLLISSTKPPSRVFYSSHLRRPFFSHFRFSSSSSTSSSVAVMPGSEPSETQWP
AKRVRDTYVDFFRGKGHKFWPSSPVVPHNDPTLLFANAGMNQYKPIFLGTADPNTELSKL
SRACNTQKCIRAGGKHNDLDDVGKDTYHHTFFEMLGNWSFGDYFKKEAIEWAWELLTKVY
GLPTDRIYATYFGGDEKAGLQPDNEARDIWLKVLPSGRVLPFGCKDNFWEMGDTGPCGPC
TEIHYDRIGNRDAASLVNNDDPTCLEIWNLVFIQFNRESDGSLKPLPAKHVDTGMGFERL
TSVLQNKMSNYDTDVFMPIFDDIQKATGARPYSGKVGPEDVDRVDMAYRVVADHIRTLSF
AIADGSRPGNEGREYVLRRILRRAVRYGKEILKAEEGFFNGLVSSVIRVMGDVFTELKEH
EKKITDIIKEEEASFCKTLAKGIEKFRKAGQAVQGNTLSGDDAFILWDTYGFPLDLTQLM
AEERGLLVDVDGFNKAMEEARERSRSAQNKQAGGAIVMDADATSTLHKAGVSATDDSFKY
IWFQDHESELKAIYTGSTFLESSAASDNVGLVLGSTSFYAEQGGQIFDTGLIEGSFGTFN
VCNVQIFGGFVLHIGYLSKETGEVSVGDKVICKVDYERRKLIAPNHTCTHMLNYALKEVL
GDHIDQKGSIVLPEKLRFDFSHGKPVDPEDLRRIESIVNKQIKDELDVFSKEAVLSEAKR
IKGLRAVFGEVYPDPVRVVSIGRKVEDLLADPENNEWSLLSSEFCGGTHITNTREAKAFA
LLSEEGIAKGIRRVTAVTTECAFDALNAASLLEREVEDASRAEGSALEKKVSALKSRVDA
AIIPAAKKADIRTKIASLQNEVRKAQKKIAEQNLKKSVKLATEAAESAASDGKTFCIIQL
DVGLDAAAVREAVSKVMEKKGMSIMVFSTDESTNKAVVCAGVPEKSDQFKPLDVTEWLTT
ALGPLKGRCGKGKGGLASGQGTDASQVQAALDMASSFASMKLN
>Q9LTR9
MADNNSPPGSVEQKADQIVEANPLVKDDTSLETIVRRFQDSMSEAKTHKFWETQPVGQFK
DIGDTSLPEGPIEPATPLSEVKQEPYNLPSVYEWTTCDMNSDDMCSEVYNLLKNNYVEDD
ENMFRFNYSKEFLRWALRPPGYYQSWHIGVRAKTSKKLVAFISGVPARIRVRDEVVKMAE
INFLCVHKKLRSKRLAPVMIKEVTRRVHLENIWQAAYTAGVILPTPITTCQYWHRSLNPK
KLIDVGFSRLGARMTMSRTIKLYKLPDAPITPGFRKMEPRDVPAVTRLLRNYLSQFGVAT
DFDENDVEHWLLPREDVVDSYLVESPETHDVTDFCSFYTLPSTILGNPNYTTLKAAYSYY
NVATQTSFLQLMNDALIVSKQKGFDVFNALDVMHNESFLKELKFGPGDGQLHYYLYNYRL
KSALKPAELGLVLL
>Q96247
MSEGVEAIVANDNGTDQVNGNRTGKDNEEHDGSTGSNLSNFLWHGGSVWDAWFSCASNQV
AQVLLTLPYSFSQLGMLSGIVLQIFYGLLGSWTAYLISVLYVEYRARKEKEGKSFKNHVI
QWFEVLDGLLGSYWKALGLAFNCTFLLFGSVIQLIACASNIYYINDHLDKRTWTYIFGAC
CATTVFIPSFHNYRIWSFLGLGMTTYTAWYLAIASIIHGQAEGVKHSGPTKLVLYFTGAT
NILYTFGGHAVTVEIMHAMWKPQKFKYIYLMATLYVFTLTIPSAAAVYWAFGDALLDHSN
AFSLMPKNAWRDAAVILMLIHQFITFGFACTPLYFVWEKVIGMHDTKSICLRALARLPVV
IPIWFLAIIFPFFGPINSAVGALLVSFTVYIIPSLAHMLTYRSASARQNAAEKPPFFMPS
WTAMYVLNAFVVVWVLIVGFGFGGWASVTNFVRQVDTFGLFAKCYQCKPAAAAAHAPVSA
LHHRL
>Q9C826
MSTNTESSSYSSLPSQRLLGKVALITGGATGIGESIVRLFHKHGAKVCIVDLQDDLGGEV
CKSLLRGESKETAFFIHGDVRVEDDISNAVDFAVKNFGTLDILINNAGLCGAPCPDIRNY
SLSEFEMTFDVNVKGAFLSMKHAARVMIPEKKGSIVSLCSVGGVVGGVGPHSYVGSKHAV

LGLTRSVAAELGQHGIRVNCVSPYAVATKLALAHLPEEERTEDAFVGFRNFAAANANLKG
VELTVDDVANAVLFLASDDSRYISGDNLMIDGGFTCTNHSFKVFR
>Q41764
MANARSGVAVNDECMLKFGELQSKRLHRFITFKMDDKFKEIVVDQVGDRATSYDDFTNSL
PENDCRYAIYDFDFVTAEDVQKSRIFYILWSPSSAKVKSKMLYASSNQKFKSGLNGIQVE
LQATDASEISLDEIKDRAR
>O24047
MAVEPLRVLVTGAAGQIGYALVPMIARGIMLGANQPVILHMLDIPPAAEALNGVKMELVD
AAFPLLKGVVATTDAAEACKGVNVAVMVGGFPRKEGMERKDVMSKNVSIYKAQASALEQH
AAPNCKVLVVANPANTNALILKEFAPSIPEKNISCLTRLDHNRALGQISERLNVQVSDVK
NVIIWGNHSSTQYPDVNHATVKTQGVDKPVRELVADDAWLNGEFITTVQQRGAAIIKARK
LSSALSAASSACDHIHDWVLGTPEGTWVSMGVYSDGSYNVPAGIIYSFPVTCKNGEWTIV
QGLPIDDDSRKKMDATAAELVEEKTLAYSCLT
>O22518
MATATNAAAAPPRQLSQKEADIQMMLAADVHLGTKNCDFQMERYIFKRRNDGIYIINLGK
TWEKLQLAARVIVAIENPQDIIVQSARPYGQRAVLKFAQYTGAHAIAGRHTPGTFTNQLQ
TSFSEPRLLILTDPRTDHQPIKEAALGNIPTIAFCDTDSPMRYVDIGIPANNKGKHSIGC
LFWLLARMVLQMRGTIRPGLKWDVMVDLFFYREPEEAKQQEEEEAPAVDYAITDFNAGAI
AADGQWPGTIDQSWSDAVPQPIPAVPGVNWGAPAEAPAAAGGDWGEAVPPPQQIPVPPSG
IDTVQPSGWD

(5) $\mathbb{S}_5$: 42 endoplasmic reticulum proteins

>Q9SLG2
MEAQNIFLYLLIVFLSLHFVFTTLKGRLSPANTRRLIRLLHIPIKSPVAAAIFARKDTRE
FLDSSIKLVNEEDDFGFSFDFKPYMISKAETINRALDEAIPLIEPLNIHKAMRYAILAGG
KRVRPILCLAACELVGGEERLAIQAACAVEMIHTMSLIKDDLPCMDNDDLRRGKPTTHKV
FGESVAILSGGALLALAFEHLTEADVSSKKMVRAVKELAKSIGTKGLVAGQAKDLSSEGL
EQNDVGLEDLEYIHVHKTGSLLEASAVIGAVIGGGTEKEIEKVRNFARCIGLLFQVVDDI
LDETKSSEELGKTAGKDKVAGKLTYPKVIGVEKSKEFVEKLKRDAREHLQGFDSDKVKPL
IALTNFIANRNH
>Q9SE50
MVRFEKVHLVLGLALVLTLVGAPTKAQGPVCGAGLPDKFSRLNFPEGFIWGTATAAFQVE
GAVNEGCRGPSMWDTFTKKFPHRCENHNADVAVDFYHRYKEDIQLMKDLNTDAFRLSIAW
PRIFPHGRMSKGISKVGVQFYHDLIDELLKNNIIPLVTVFHWDTPQDLEDEYGGFLSGRI
VQDFTEYANFTFHEYGHKVKHWITFNEPWVFSRAGYDNGKKAPGRCSPYIPGYGQHCQDG
RSGYEAYQVSHNLLLSHAYAVDAFRNCKQCAGGKIGIAHSPAWFEPQDLEHVGGSIERVL
DFILGWHLAPTTYGDYPQSMKDRVGHRLPKFTEAEKKLLKGSTDYVGMNYYTSVFAKEIS
PDPKSPSWTTDSLVDWDSKSVDGYKIGSKPFNGKLDVYSKGLRYLLKYIKDNYGDPEVII
AENGYGEDLGEKHNDVNFGTQDHNRKYYIQRHLLSMHDAICKDKVNVTGYFVWSLMDNFE
WQDGYKARFGLYYIDFQNNLTRHQKVSGKWYSEFLKPQFPTSKLREEL
>Q949U1
MMSFTTSLPYPFHILLVFILSMASITLLGRILSRPTKTKDRSCQLPPGPPGWPILGNLPE
LFMTRPRSKYFRLAMKELKTDIACFNFAGIRAITINSDEIAREAFRERDADLADRPQLFI
METIGDNYKSMGISPYGEQFMKMKRVITTEIMSVKTLKMLEAARTIEADNLIAYVHSMYQ

RSETVDVRELSRVYGYAVTMRMLFGRRHVTKENVFSDDGRLGNAEKHHLEVIFNTLNCLP
SFSPADYVERWLRGWNVDGQEKRVTENCNIVRSYNNPIIDERVQLWREEGGKAAVEDWLD
TFITLKDQNGKYLVTPDEIKAQCVEFCIAAIDNPANNMEWTLGEMLKNPEILRKALKELD
EVVGRDRLVQESDIPNLNYLKACCRETFRIHPSAHYVPSHLARQDTTLGGYFIPKGSHIH
VCRPGLGRNPKIWKDPLVYKPERHLQGDGITKEVTLVETEMRFVSFSTGRRGCIGVKVGT
IMMVMLLARFLQGFNWKLHQDFGPLSLEEDDASLLMAKPLHLSVEPRLAPNLYPKFRP
>P49333
MEVCNCIEPQWPADELLMKYQYISDFFIAIAYFSIPLELIYFVKKSAVFPYRWVLVQFGA
FIVLCGATHLINLWTFTTHSRTVALVMTTAKVLTAVVSCATALMLVHIIPDLLSVKTREL
FLKNKAAELDREMGLIRTQEETGRHVRMLTHEIRSTLDRHTILKTTLVELGRTLALEECA
LWMPTRTGLELQLSYTLRHQHPVEYTVPIQLPVINQVFGTSRAVKISPNSPVARLRPVSG
KYMLGEVVAVRVPLLHLSNFQINDWPELSTKRYALMVLMLPSDSARQWHVHELELVEVVA
DQVAVALSHAAILEESMRARDLLMEQNVALDLARREAETAIRARNDFLAVMNHEMRTPMH
AIIALSSLLQETELTPEQRLMVETILKSSNLLATLMNDVLDLSRLEDGSLQLELGTFNLH
TLFREVLNLIKPIAVVKKLPITLNLAPDLPEFVVGDEKRLMQIILNIVGNAVKFSKQGSI
SVTALVTKSDTRAADFFVVPTGSHFYLRVKVKDSGAGINPQDIPKIFTKFAQTQSLATRS
SGGSGLGLAISKRFVNLMEGNIWIESDGLGKGCTAIFDVKLGISERSNESKQSGIPKVPA
IPRHSNFTGLKVLVMDENGVSRMVTKGLLVHLGCEVTTVSSNEECLRVVSHEHKVVFMDV
CMPGVENYQIALRIHEKFTKQRHQRPLLVALSGNTDKSTKEKCMSFGLDGVLLKPVSLDN
IRDVLSDLLEPRVLYEGM
>P35016
MRKWTVPSVLFLLCPSLSSSCQGRKIHANAEADSDAPVDPPKVEDKIGAVPNGLSTDSDV
AKREAESMSMRNLRSDAEKFEFQAEVSRLMDIIINSLYSNKDIFLRELISNASDALDKIR
FLALTDKEILGEGDTAKLEIQIKLDKEKKILSIRDRGIGMTKEDLIKNLGTIAKSGTSAF
VEKMQTSGDLNLIGQFGVGFYSVYLVPDYVEVISKHNDDKQYIWESKADGAFAISEDVWN
EPLGRGTEIRLHLRDEAQEYLDEFKLKELVKRYSEFINFPIYLWASKEVEVEVPAEEDDS
SDDEDNKSESSSSEEGEEEETEKEEDEKKPKTKKVKETTYEWELLNDMKAIWLRNPKDVT
DDEYTKFYHSLAKDFSEEKPLAWSHFTAEGDVEFKAFTLLPPKAPQDLYESYYNSNKSNL
KLYVRRVFISDEFDELLPKYLNFLKGLVDSDTLPLNVSREMLQQHSSLKTIKKKLIRKAL
DMIRKIADEDPDEANDKDKKEVEESTDNDEKKGQYAKFWNEFGKSIKLGIIEDAANRNRL
AKLLRFESTKSEGKLTSLDQYISRMKSGQKDIFYITGTSKEQLEKSPFLERLTKKNYEVI
LFTDPVDEYLMQYLMDYEDKKFQNVSKEGLKIGKDSKDKELKESFKELTKWWKGALASEN
VDDVKISNRLANTPCVVVTSKYGWSSNMERIMQSQTLSDASKQAYMRGKRVLEINPRHPI
IKELRERVVKDAEDESVKQTARLMYQTALMESGFMLNDPKEFASSIYDSVKSSLKISPDA
TVEEEDDTEEAEAESGTTESSAAEDAGAETLDLKDEL
>P48630
MGLAKETTMGGRGRVAKVEVQGKKPLSRVPNTKPPFTVGQLKKAIPPHCFQRSLLTSFSY
VVYDLSFAFIFYIATTYFHLLPQPFSLIAWPIYWVLQGCLLTGVWVIAHECGHHAFSKYQ
WVDDVVGLTLHSTLLVPYFSWKISHRRHHSNTGSLDRDEVFVPKPKSKVAWFSKYLNNPL
GRAVSLLVTLTIGWPMYLAFNVSGRPYDSFASHYHPYAPIYSNRERLLIYVSDVALFSVT
YSLYRVATLKGLVWLLCVYGVPLLIVNGFLVTITYLQHTHFALPHYDSSEWDWLKGALAT
MDRDYGILNKVFHHITDTHVAHHLFSTMPHYHAMEATNAIKPILGEYYQFDDTPFYKALW
REARECLYVEPDEGTSEKGVYWYRNKY
>A2XMB2

MAAYTSKIFALFALIALSASATTAITTMQYFPPTLAMGTMDPCRQYMMQTLGMGSSTAMF
MSQPMALLQQQCCMQLQGMMPQCHCGTSCQMMQSMQQVICAGLGQQQMMKMAMQMPYMCN
MAPVNFQLSSCGCC
>Q8LG50
MVIAAAVIVPLGLLFFISGLAVNLFQAVCYVLIRPLSKNTYRKINRVVAETLWLELVWIV
DWWAGVKIQVFADNETFNRMGKEHALVVCNHRSDIDWLVGWILAQRSGCLGSALAVMKKS
SKFLPVIGWSMWFSEYLFLERNWAKDESTLKSGLQRLSDFPRPFWLALFVEGTRFTEAKL
KAAQEYAASSELPIPRNVLIPRTKSFVSAVSNMRSFVPAIYDMTVTIPKTSPPPTMLRLF
KGQPSVVHVHIKCHSMKDLPESDDAIAQWCRDQFVAKDALLDKHIAADTFPGQQEQNIGR
PIKSLAVVLSWACVLTLGAIKFLHWAQLFSSWKGITISALGLGIITLCMQILIRSSQSER
STPAKVVPAKPKDNHHPESSSQTETEKEK
>P93508
MANPKSLSLFLLSLLAIASAEVFFEERFEDGWENRWVKSDWKKDENTAGEWNYTSGKWNG
DPNDKGIQTSEDYRFYAISAEFPEFSNKDKTLVFQFSVKHEQKLDCGGGYMKLLSSSTDQ
KKFGGDTPYSIMFGPDICGYSTKKVHAILNYNDTNHLIKKEVPCETDQLTHVYTLVIRPD
ATYSILIDNVEKQTGSLYTDWDLLPPKKIKDPEAKKPEDWDEKEYIPDPEDKKPEGYDDI
PKEIPDPDAKKPEDWDDEEDGEWTAPTIANPEYKGPWKPKKIKNPNYKGKWKAPMIDNPD
FKDDPEIYVYPNLKYVGIELWQVKSGTLFDNVLICNDPEYAKQLAEETWGKNKDAEKAAF
EEAEKKKEEEESKDDPADSDADEDDDDADDTEGEDDGESKSDAAEDSAEDVHDEL
>P38389
MVGSGAPQRGSAAATASMRRRKPTSGAGGGGASGGAAGSMLQFYTDDAPGLKISPNVVLI
MSIGFIAFVAVLHVMGKLYFVK
>P17048
MKIIFVFALLAIVACNASARFDPLSQSYRQYQLQSHLLLQQQVLSPCSEFVRQQYSIVAT
PFWQPATFQLINNQVMQQQCCQQLRLVAQQSHYQAISIVQAIVQQLQLQQFSGVYFDQTQ
AQAQTLLTFNLPSICGIYPNYYSAPRSIATVGGVWY
>P35402
MNIFRFAGDMSHLISVLILLLKIYATKSCAGISLKTQELYALVFLTRYLDLFTDYVSLYN
SIMKIVFIASSLAIVWCMRRHPLVRRSYDKDLDTFRHQYVVLACFVLGLILNEKFTVQEV
FWAFSIYLEAVAILPQLVLLQRSGNVDNLTGQYVVFLGAYRGLYIINWIYRYFTEDHFTR
WIACVSGLVQTALYADFFYYYYISWKTNTKLKLPA
>P93339
MSVTELKERHMAATQTVNDLREKLKQKRLQLLDTDVSGYARSQGKTPVTFGPTDLVCCRI
LQGHTGKVYSLDWTPEKNRIVSASQDGRLIVWNALTSQKTHAIKLPCAWVMTCAFSPSGH
SVACGGLDSVCSIFNLNSPIDKDGNHPVSRMLSGHKGYVSSCQYVPDEDTHLITSSGDQT
CVLWDITTGLRTSVFGGEFQFGHTADVQSVSISSSNPRLFVSGSCDTTARLWDTRVASRA
QRTFYCHEGDVNTVKFFPDGNRFGTGSEDGTCRLFDIRTGHQLQVYYQPHGDGDIPHVTS
MAFSISGRLLFVRYSNGDCYVWDTLLAKVVLNLGAVQNSHEGXISCLGLSADGXXLCTGS
WDTNLKIWAFGGHRSVI
>Q8GYE0
MEIEEASRESGHVVCGSWIRRPKKVNWVLIAKASKRRGSSVSSPALLNIFSFDPITASLS
SSPLATHTLKDSDGDPVAVSVHPGGDYFVCSTSKGGCKLFELVGGATGITILAKELLPLQ
NAGLQKCMAFSFDGSKLAVGGVDGCLRIMEWPNLSVILDEPKAHKSIRDMDFSLDSEFLA
TTSTDGSARIWKAEDGFPLSTLERSGDENIELCRFSKDGTKPFLFCAAQRGDTPMVNVYD

ISTWKKLGFKKLSRKTASTMAVSLDGKYIALGGKDGDVSVAEVKTMEIYHYSKRLHLGQS
IASLEFCPSERVMLTTSSEWGEMVTKLTVPKEWKEWQIYALLFCLFMASVIAAYVFFENS
DSFWKLPMGKDQKRPKISLFGGSSSTPSEDHSRWNLDL

>Q8VXU6

MDSNWINCPSVFSSSSSSSSRRCQSRSDLYLGGGYEDLEGEDDLKAEFICPFCAEDFDIVG
LCCHIDEEHPVEAKNGVCPVCTKRVGLDIVGHITTQHANFFKVQRRRRLRRGGYSSTYLA
LKKELREANLQSLLGGSSSFTSSTNIDSDPLLSSFMFNSPSVNQSANKSATPVTVGNAAT
KVSIKESLKRDIQEAPLSGEDQEKAKKSEFVRGLLLSTMLEDDF

>Q9LDU6

MAETVHSPIVTYASMLSLLAFCPPFVILLWYTMVHQDGSVTQTFGFFWENGVQGLINIWP
RPTLIAWKIIFCYGAFEAILQLLLPGKRVEGPISPAGNRPVYKANGLAAYFVTLATYLGL
WWFGIFNPAIVYDHLGEIFSALIFGSFIFCVLLYIKGHVAPSSSDSGSCGNLIIDFYWGM
ELYPRIGKSFDIKVFTNCRFGMMSWAVLAVTYCIKQYEINGKVSDSMLVNTILMLVYVTK
FFWWEAGYWNTMDIAHDRAGFYICWGCLVWVPSVYTSPGMYLVNHPVELGTQLAIYILVA
GILCIYINYDCDRQRQEFRRTNGKCLVWGRAPSKIVASYTTTSGETKTSLLLTSGWWGLA
RHFHYVPEILSAFFWTVPALFDNFLAYFYVIFLTLLLFDRAKRDDDRCRSKYGKYWKLYC
EKVKYRIIPGIY

>Q84K11

MPGMEAENSNASRAEELKQLANEAFKGHKYSQAIDLYTQAIELNGENAVYYANRAFAHTK
LEEYGSAIQDGTRAIEIDPRYSKGYYRRGAAYLAMGKFKDALKDFQQVKKLCPNDPDATK
KLKECEKAVMKLKFEEAISVPESQRRSVADSIDYRSVGSGPGSSYVPTKTTAVSAAAALM
GVLVVYMGTKAATMVAAAASAALLVVLITFLWGRCSDGFFTKSRTLELEVEPQYAGARIE
GDVVTLDFVKKMLDDFKNQKNLHKRYAYQIVLQTREMLRALPSLVDIVVPEGKHFTVCGD
VHGQFYDLLNIFELNGLPSEDNPYLFNGDFVDRGSFSLEVILTLFAFKCMCPSAIHLARG
NHESKSMNKIYGFEGEVRSKLSEIFVELFAEVFCCLPLAHVINEKVFVVHGGLFSVDGVK
LSDIRAIDRFCEPPEEGLMCELLWSDPQPQPGRGPSKRGVGLSFGGDVTKRFLQENNLDL
VVRSHEVKDEGYEIEHDGKLITVFSAPNYCDQMGNKGAFIRFEAPDMKPNIVTFSAVPHP
DVKPMAYANNFLRMFS

>Q9M8W5

MMGVLKSAIGDMLMTFSWVVLSATFGIQTAAIISAGDFQAITWAPLVILTSLIFVYVSIF
TVIFGSASFNPTGSAAFYVAGVPGDTLFSLAIRLPAQAIGAAGGALAIMEFIPEKYKHMI
GGPSLQVDVHTGAIAETILSFGITFAVLLIIILRGPRRLLAKTFLLALATISFVVAGSKYT
GPAMNPAIAFGWAYMYSSHNTWDHIYVYWISSFVGALSAALLFRSIFPPPRPQKKKQKKA

>Q4G2J3

MAQAVEEWYRQMPIITRSYLTAAVVTTVGCTLEIISPYHLYLNPKLVVQHYEIWRLVTNF
LYFRKMDLDFLFHMFFLARYCKLLEENSFRGRTADFFYMLLFGATVLTGIVLIGGMIPYI
SETFARILFLSNSLTFMMVYVWSKHNPFIHMSFLGLFTFTAAYLPWVLLGFSILVGSSTW
VDLLGMIAGHVYYFLEDVYPRMTGRRPLKTPSFIKALFADDNVVVAQPPNAGIGAGARFG
AIGVDPQAQ

>Q9FZ33

MAIITEEEEDPKTLNPPKNKPKDSDFTKSESTMKNPKPQSQNPFPFWFYFTVVVSLATII
FISLSLFSSQNDPRSWFLSLPPALRQHYSNGRTIKVQVNSNESPIEVFVAESGSIHTETV
VIVHGLGLSSFAFKEMIQSLGSKGIHSVAIDLPGNGFSDKSMVVIGGDREIGFVARVKEV

YGLIQEKGVFWAFDQMIETGDLPYEEIIKLQNSKRRSFKAIELGSEETARVLGQVIDTLG
LAPVHLVLHDSALGLASNWVSENWQSVRSVTLIDSSISPALPLWVLNVPGIREILLAFSF
GFEKLVSFRCSKEMTLSDIDAHRILLKGRNGREAVVASLNKLNHSFDIAQWGNSDGINGI
PMQVIWSSEASKEWSDEGQRVAKALPKAKFVTHSGSRWPQESKSGELADYISEFVSLLPK
SIRRVAEEPIPEEVQKVLEEAKAGDDHDHHHGHGHAHAGYSDAYGLGEEWTTT
>O64967
MEASRRSAVKPVIVLKPSKRFPLVEDTPGKASDALPLPLYLTNAVFFTLFFTVVYFLLSR
WREKIRASIPLHAVTFPEIVAIFAFVASLIYLLGFFGIDFVQSLIIRPSGDVWSGEDYEE
ENEVLLHEEDARTVPCGQALDCSVPSLPHMARNVTAQRLFDEKPVRVATEEDARKVSCGQ
AVDCSLHSLPPRPPIVTSQKLFHEKTVIVTTEEDEEIIKSVVAGTLPSYSLESKLGDCKR
AAAIRREALQRLTGRSLSGLPLDGFDYESILGQCCEMPVGYVQIPVGIAGPLLLNGREYS
VPMATTEGCLVASTNRGCKAIHLSGGATSILLKDGMTRAPVVRFSTAKRAAELKFYLEDP
ENFDTLAVVFNRSSRFGRLQSIKCAIAGKNLYLRFTCSTGDAMGMNMVSKGVQNVLDFLQ
TDFPDMDVIGISGNFCSDKKPAAVNWIEGRGKSVVCEAIIEGDVVRKVLKTSVESLVELN
MLKNLTGSAMAGALGGFNAHASNIVTAIYIATGQDPAQNVESSHCITMMEAVNDGKDLHI
SVTMPSIEVGTVGGGTQLASQSACLNLLGVKGASKDVAGANSRMLATIVTGAVLAGELSL
MSALAAGQLVKSHMKYNRSSKDMSNLSS
>Q8W037
MDDISVSKSNHGNVVVLNIKASSLADTSLPSNKHESSSPPLLSVHFLQKLLAELVGTYYL
IFAGCAAIAVNAQHNHVVTLVGIAVVWGIVIMVLVYCLGHLSAHFNPAVTLALASSQRFP
LNQVPAYITVQVIGSTLASATLRLLFDLNNDVCSKKHDVFLGSSPSGSDLQAFVMEFIIT
GFLMLVVCAVTTTKRTTEELEGLIIGATVTLNVIFAGEVSGASMNPARSIGPALVWGCYK
GIWIYLLAPTLGAVSGALIHKMLPSIQNAEPEFSKTGSSHKRVTDLPL
>Q03685
MAGAWKRRASLIVFAIVLFGSLFAFSIAKEEATKLGTVIGIDLGTTYSCVGVYKNGHVEI
IANDQGNRITPSWVAFTDGERLIGEAAKNQAAVNPERTIFDVKRLIGRKFDDKEVQRDKK
LVPYEIVNKDGKPYIQVKIKDGETKVFSPEEISAMILTKMKETAEAYLGKKIKDAVVTVP
AYFNDAQRQATKDAGVIAGLNVARIINEPTAAAIAYGLDKKGGEKNILVFDLGGGTFDVS
ILTIDNGVFEVLATNGDTHLGGEDFDQRIMEYFIKLIKKKHGKDISKDNRALGKLRREAE
RAKRALSSQHQVRVEIESLFDGVDFSEPLTRARFEELNNDLFRKTMGPVKKAMEDAGLEK
NQIDEIVLVGGSTRIPKVQQLLKDYFDGKEPNKGVNPDEAVAYGAAVQGGILSGEGGDET
KDILLLDVAPLTLGIETVGGVMTKLIPRNTVIPTKKSQVFTTYQDQQTTVTISVFEGERS
LTKDCRLLGKFDLTGIAPAPRGTPQIEVTFEVDANGILNVKAEDKASGKSEKITITNDKG
RLSQEEIERMVKEAEEFAEEDKKVKERIDARNSLETYVYNMRNQINDKDKLADKLESDEK
EKIETATKEALEWLDDNQSAEKEDYDEKLKEVEAVCNPIITAVYQRSGGAPGGASEESNE
DDDSHDEL
>Q05001
MDSSSEKLSPFELMSAILKGAKLDGSNSSDSGVAVSPAVMAMLLENKELVMILTTSVAVL
IGCVVVLIWRRSSGSGKKVVEPPKLIVPKSVVEPEEIDEGKKKFTIFFGTQTGTAEGFAK
ALAEEAKARYEKAVIKVIDIDDYAADDEEYEEKFRKETLAFFILATYGDGEPTDNAARFY
KWFVEGNDRGDWLKNLQYGVFGLGNRQYEHFNKIAKVVDEKVAEQGGKRIVPLVLGDDDQ
CIEDDFAAWRENVWPELDNLLRDEDDTTVSTTYTAAIPEYRVVFPDKSDSLISEANGHAN
GYANGNTVYDAQHPCRSNVAVRKELHTPASDRSCTHLDFDIAGTGLSYGTGDHVGVYCDN
LSETVEEAERLLNLPPETYFSLHADKEDGTPLAGSSLPPPFPPCTLRTALTRYADLLNTP

KKSALLALAAYASDPNEADRLKYLASPAGKDEYAQSLVANQRSLLEVMAEFPSAKPPLGV
FFAAIAPRLQPRFYSISSSPRMAPSRIHVTCALVYEKTPGGRIHKGVCSTWMKNAIPLEE
SRDCSWAPIFVRQSNFKLPADPKVPVIMIGPGTGLAPFRGFLQERLALKEEGAELGTAVF
FFGCRNRKMDYIYEDELNHFLEIGALSELLVAFSREGPTKQYVQHKMAEKASDIWRMISD
GAYVYVCGDAKGMARDVHRTLHTIAQEQGSMDSTQAEGFVKNLQMTGRYLRDVW
>Q9LTS3
MASYNLRSQVRLIAITIVIIITLSTPITTNTSPQPWNILSHNEFAGKLTSSSSSVESAAT
DFGHVTKIFPSAVLIPSSVEDITDLIKLSFDSQLSFPLAARGHGHSHRGQASAKDGVVVN
MRSMVNRDRGIKVSRTCLYVDVDAAWLWIEVLNKTLELGLTPVSWTDYLYLTVGGTLSNG
GISGQTFRYGPQITNVLEMDVITGKGEIATCSKDMNSDLFFAVLGGLGQFGIITRARIKL
EVAPKRAKWLRFLYIDFSEFTRDQERVISKTDGVDFLEGSIMVDHGPPDNWRSTYYPPSD
HLRIASMVKRHRVIYCLEVVKYYDETSQYTVNEEMEELSDSLNHVRGFMYEKDVTYMDFL
NRVRTGELNLKSKGQWDVPHPWLNLFVPKTQISKFDDGVFKGIILRNNITSGPVLVYPMN
RNKWNDRMSAAIPEEDVFYAVGFLRSAGFDNWEAFDQENMEILKFCEDANMGVIQYLPYH
SSQEGWVRHFGPRWNIFVERKYKYDPKMILSPGQNIFQKINSS
>O23051
MAETTSWIPVWFPLMVLGCFGLNWLVRKVNVWLYESSLGENRHYLPPGDLGWPFIGNMLS
FLRAFKTSDPDSFTRTLIKRYGPKGIYKAHMFGNPSIIVTTSDTCRRVLTDDDAFKPGWP
TSTMELIGRKSFVGISFEEHKRLRRLTAAPVNGHEALSTYIPYIEENVITVLDKWTKMGE
FEFLTHLRKLTFRIIMYIFLSSESENVMDALEREYTALNYGVRAMAVNIPGFAYHRALKA
RKTLVAAFQSIVTERRNQRKQNILSNKKDMLDNLLNVKDEDGKTLDDEEIIDVLLMYLNA
GHESSGHTIMWATVFLQEHPEVLQRAKAEQEMILKSRPEGQKGLSLKETRKMEFLSQVVD
ETLRVITFSLTAFREAKTDVEMNGYLIPKGWKVLTWFRDVHIDPEVFPDPRKFDPARWDN
GFVPKAGAFLPFGAGSHLCPGNDLAKLEISIFLHHFLLKYQVKRSNPECPVMYLPHTRPT
DNCLARISYQ
>P11090
MARLSSLLSFSLALLTFLHGSTAQQFPNECQLDQLNALEPSHVLKAEAGRIEVWDHHAPQ
LRCSGVSFVRYIIESKGLYLPSFFSTARLSFVAKGEGLMGRVVLCAETFQDSSVFQPSGG
SPFGEGQGQGQQGQGQGHQGQGQGQQGQQGQQGQQSQGQGFRDMHQKVEHIRTGDTIATH
PGVAQWFYNDGNQPLVIVSVLDLASHQNQLDRNPRPFYLAGNNPQGQVWIEGREQQPQKN
ILNGFTPEVLAKAFKIDVRTAQQLQNQQDNRGNIIRVQGPFSVIRPPLRSQRPQEEVNGL
EETICSARCTDNLDDPSNADVYKPQLGYISTLNSYDLPILRFLRLSALRGSIRQNAMVLP
QWNANANAVLYVTDGEAHVQVVNDNGDRVFDGQVSQGQLLSIPQGFSVVKRATSEQFRWI
EFKTNANAQINTLAGRTSVLRGLPLEVISNGYQISLEEARRVKFNTIETTLTHSSGPASY
GGPRKADA
>A8MQY1
MMKGLIGYRFSPTGEEVINHYLKNKLLGKYWLVDEAISEINILSHKPSKDLPKLARIQSE
DLEWYFFSPIEYTNPNKMKMKRTTGSGFWKPTGVDREIRDKRGNGVVIGIKKTLVYHEGK
SPHGVRTPWVMHEYHITCLPHHKRKYVVCQVKYKGEAAEISYEPSPSLVSDSHTVIAITG
EPEPELQVEQPGKENLLGMSVDDLIEPMNQQEEPQGPHLAPNDDEFIRGLRHVDRGTVEY
LFANEENMDGLSMNDLRIPMIVQQEDLSEWEGFNADTFFSDNNNNYNLNVHHQLTPYGDG
YLNAFSGYNEGNPPDHELVMQENRNDHMPRKPVTGTIDYSSDSGSDAGSISTTSYQGTSS
PNISVGSSSRHLSSCSSTDSCKDLQTCTDPSIISREIRELTQEVKQEIPRAVDAPMNNES
SLVKTEKKGLFIVEDAMERNRKKPRFIYLMKMIIGNIISVLLPVKRLIPVKKL

>Q9ZSY2
MSAKKLEGSSAPANRRDPYEVLCVSKDANDQEIKSAYRKLALKYHPDKNANNPDASELFK
EVAFSYSILSDPEKRRHYDNAGFEALDADGMDMEIDLSNLGTVNTMFAALFSKLGVPIKT
TVSANVLEEAMNGTVTVRPLPIGTSVSGKVEKQCAHFFGVTISEQQAESGVVVRVTSTAQ
SKFKLLYFEQDSSGGYGLALQEEREKTGKVTSAGMYFLHFQVYRMDTTVNALAAAKDPES
AFFKRLEGLQPCEVSELKAGTHIFAVYGDNFFKTASYTIEALCAKTYEDTTEKLKEIEAQ
ILRKRNELRQFETEYRKALARFQEVTNRYTQEKQTVDELLKQRDTIHSTFSVVKTPSGNN
LSNGSSSKAQGDESKGDGDSAGEEGGTENRDKSKRKWFNLNLKGSDKKLG
>Q7X9I4
MAETDVGSVKGKEKGSGKRWILLIGAIAAVLLAVVVAVFLNTQNSSISEFTGKICNCRQA
EQQKYIGIVEDCCCDYETVNRLNTEVLNPLLQDLVKTPFYRYFKVKLWCDCPFWPDDGMC
RLRDCSVCECPESEFPEVFKKPLSQYNPVCQEGKPQATVDRTLDTRAFRGWTVTDNPWTS
DDETDNDEMTYVNLRLNPERYTGYIGPSARRIWEAIYSENCPKHTSEGSCQEEKILYKLV
SGLHSSISVHIASDYLLDEATNLWGQNLTLLYDRVLRYPDRVQNLYFTFLFVLRAVTKAE
DYLGEAEYETGNVIEDLKTKSLVKQVVSDPKTKAACPVPFDEAKLWKGQRGPELKQQLEK
QFRNISAIMDCVGCEKCRLWGKLQILGLGTALKILFTVNGEDNLRHNLELQRNEVIALMN
LLHRLSESVKYVHDMSPAAERIAGGHASSGNSFWQRIVTSIAQSKAVSGKRS
>Q8VY97
MAPIFRSTSLIAFSLFFFFFASTLPISSGSEDSYTITGRVRVPASTVIGHAAKFSNIKVI
LNGGQHVTFLRPDGYFTFHKVPAGTHLIEVYALGYFFSPVRVDVSARHRGKVQATLTETR
RSLTELVLEPLRAEQYYEMREPFSVMSIVKSPMGLMVGFMVVVVFLMPKLMENIDPEEMK
SAQEQMRSQGVPSLTSLLPASR
>P12412
MAMKKLLWVVLSLSLVLGVANSFDFHEKDLESEESLWDLYERWRSHHTVSRSLGEKHKRF
NVFKANVMHVHNTNKMDKPYKLKLNKFADMTNHEFRSTYAGSKVNHHKMFRGSQHGSGTF
MYEKVGSVPASVDWRKKGAVTDVKDQGQCGSCWAFSTIVAVEGINQIKTNKLVSLSEQEL
VDCDKEENQGCNGGLMESAFEFIKQKGGITTESNYPYTAQEGTCDESKVNDLAVSIDGHE
NVPVNDENALLKAVANQPVSVAIDAGGSDFQFYSEGVFTGDCNTDLNHGVAIVGYGTTVD
GTNYWIVRNSWGPEWGEQGYIRMQRNISKKEGLCGIAMMASYPIKNSSDNPTGSLSSPKD
EL
>P46422
MAGIKVFGHPASIATRRVLIALHEKNLDFELVHVELKDGEHKKEPFLSRNPFGQVPAFED
GDLKLFESRAITQYIAHRYENQGTNLLQTDSKNISQYAIMAIGMQVEDHQFDPVASKLAF
EQIFKSIYGLTTDEAVVAEEEAKLAKVLDVYEARLKEFKYLAGETFTLTDLHHIPAIQYL
LGTPTKKLFTERPRVNEWVAEITKRPASEKVQ
>P45434
MMNLRVLFLALLLLASPLLQVARCQSDAEDHSSLVDDVVGENTDDAVEEDDHDLDMNLSS
FPGVETVCVFPKNSAKLVPAGEETELLVGLKNEGKTRVGVMGIRASVHLPYDHKLLVQNL
TMLRLNNASIPTSLQATFPYIFAVSQYLQPGAFDLVGYIIYDVEGKPYQSVFYNGTIEVV
ESGGLLSGESVFLLTLGIGLLLLLGLWAYSQVQRLTKKTKKVSKVEVGTRSTEASLDEWL
EGTTLAKTSSGKTKNKKN
>Q8GSP8
MLRSAGRVAAVALLALFALGCVSAAAKPSTDRLVAAIKSKNLSAVVDALSVKGLDVNTPD
STRRLPLVEAARSRDARLVGALLDQGALARVSDGTTTPLHLSMQGGSAAIVKLLLAHGAD

PNAKDKTGASARSTAAAVKELADLLKQWDARGAMAFEDEPGAWIREERDGQSYYWKPANG
ESRWAVPPSCAWQRVTVQGHPIKYINSLTGQETTRVPPALAWARVTAADGSALWLNWASR
VASAAATAPAELPAELAAELAMHPNRRWYNTATREYVYTDPAYATPWRELVDEASGAPFF
FNVETGDTTWELPAALAWTEVIESSSGADGESGSGSEAGPRYFHNTVSGEVAWSAPEGSR
HVFVEASAADL

>P33488

MVRRRPATGAAPRPHLAAVGRGLLLASVLAAAASSLPVAESSCPRDNSLVRDISRMQQRN
YGREGFSHITVTGALAHGTKEVEVWLQTFGPGQRTPIHRHSCEEVFIVLKGKGTLLLGSS
SLKYPGQPQEVPVFQNTTFSIPVNDPHQVWNSNEHEDLQVLVIISRPPVKIFIYDDWSMP
HTAAKLKFPYFWDEDCLPAPKDEL

>Q5NE24

MDLMDMDAINDLHFSGHSSLTNTPTSDEDYGCTWNHWSPIVNWDTFTGAPDDFHHLMDTI
IEDRTTVLEQLSPSITTTTTTTTTTDEEEEEMETTTTTTTTAIKTHEVGDDSKGLKLVHL
LMAGAEALTGSTKNRDLARVILIRLKELVSQHANGSNMERLAAHFTEALHGLLEGAGGAH
NNHHHHNNNKHYLTTNGPHDNQNDTLAAFQLLQDMSPYVKFGHFTANQAIIEAVAHERRV
HVIDYDIMEGVQWASLIQSLASNNNGPHLRITALSRTGTGRRSIATVQETGRRLTSFAAS
LGQPFSFHHCRLDSDETFRPSALKLVRGEALVFNCMLNLPHLSYRAPESVASFLNGAKTL
NPKLVTLVEEEVGSVIGGFVERFMDSLHHYSAVFDSLEAGFPMQNRARTLVERVFFGPRI
AGSLGRIYRTGGEEERRSWGEWLGEVGFRGVPVSFANHCQAKLLLGLFNDGYRVEEVGVG
SNKLVLDWKSRRLLSASLWTCSSSDSDL

>Q8LDP4

MAKASFILLGTLFLFGAIASIQAKEDLKEVTHKVYFDVEIDGKSAGRVVIGLFGKAVPKT
AENFRALCTGEKGVGKSGKPLHYKGSKFHRIIPSFMIQGGDFTHGNGMGGESIYGQKFAD
ENFKLKHTGPGVLSMANSGEDTNGSQFFITTVTTSWLDGRHVVFGKVVQGMDVVYKIEAE
GKQSGTPKSKVVIADSGELPL

>O80458

MLSLLSSDSSLLSLLFLFLIPCLFITSYIGFPVFLLKLIGLIKIKAARDNEKRDEGTYVV
REDGLQRELMPRHVAFILDGNRRWAKRAGLTTSQGHEAGAKRLIDIAELCFELGVHTVSA
FAFSTENWGRDKIEIDNLMSLIQHYRNKSNIKFFHRSEVRVSVIGNKTKIPESLLKEIHE
IEEATKGYKNKHLIMAVDYSGKFDIMHACKSLVKKSEKGLIREEDVDEALIERELLTNCS
DFPSPDLMIRTSGEQRISNFFLWQLAYSELFFSPVFWPDFDKDKLLEALASYQRRERRFG
CRV

>P92939

MGKGSEDLVKKESLNSTPVNSDTFPAWAKDVAECEEHFVVSREKGLSSDEVLKRHQIYGL
NELEKPEGTSIFKLILEQFNDTLVRILLAAAVISFVLAFFDGDEGGEMGITAFVEPLVIF
LILIVNAIVGIWQETNAEKALEALKEIQSQQATVMRDGTKVSSLPAKELVPGDIVELRVG
DKVPADMRVVALISSTLRVEQGSLTGESEAVSKTTKHVDENADIQGKKCMVFAGTTVVNG
NCICLVTDTGMNTEIGRVHSQIQEAAQHEEDTPLKKKLNEFGEVLTMIIGLICALVWLIN
VKYFLSWEYVDGWPRNFKFSFEKCTYYFEIAVALAVAAIPEGLPAVITTCLALGTRKMAQ
KNALVRKLPSVETLGCTTVICSDKTGTLTTNQMAVSKLVAMGSRIGTLRSFNVEGTSFDP
RDGKIEDWPMGRMDANLQMIAKIAAICNDANVEQSDQQFVSRGMPTEAALKVLVEKMGFP
EGLNEASSDGDVLRCCRLWSELEQRIATLEFDRDRKSMGVMVDSSSGNKLLLVKGAVENV
LERSTHIQLLDGSKRELDQYSRDLILQSLRDMSLSALRCLGFAYSDVPSDFATYDGSEDH
PAHQQLLNPSNYSSIESNLIFVGFVGLRDPPRKEVRQAIADCRTAGIRVMVITGDNKSTA

EAICREIGVFEADEDISSRSLTGIEFMDVQDQKNHLRQTGGLLFSRAEPKHKQEIVRLLK
EDGEVVAMTGDGVNDAPALKLADIGVAMGISGTEVAKEASDMVLADDNFSTIVAAVGEGR
SIYNNMKAFIRYMISSNIGEVASIFLTAALGIPEGMIPVQLLWVNLVTDGPPATALGFNP
PDKDIMKKPPRRSDDSLITAWILFRYMVIGLYVGVATVGVFIIWYTHSSFMGIDLSQDGH
SLVSYSQLAHWGQCSSWEGFKVSPFTAGSQTFSFDSNPCDYFQQGKIKASTLSLSVLVAI
EMFNSLNALSEDGSLVTMPPWVNPWLLLAMAVSFGLHFVILYVPFLAQVFGIVPLSLNEW
LLVLAVSLPVILIDEVLKFVGRCTSGYRYSPRTLSTKQKEE

>Q94CC0

MSRYFSFFFLALFLHYRIIVASPFEGFDAEEDDVTDDSSHLLHHSLPPPLLTQSHSSLSD
PDPEPEPSSAECKSDLITESDLEHQSDSKTPSSTPFEYWDEDEFEGLPVEIETLESPLIT
ENGTHADPKTPDLKTSSEAQGDTNDQTKKKKSYAVEIACVCFLIALAINYFVGKRENESL
ALAWAAKFASKDTIFQKNFSMLGVSELEDSPLLLKEALNVFKFYASGRRYCHGLLATMEL
KSRHDLISRVFNLVVPCKDEITFEVYMNEETMDHVVFAMTKKKAAKTMQKEMRDLQRFAG
IVSPPAGRKWVSEEFALISESKEVAADLITDTVLDQVFGDKAVDKYGKNFMSMHISDQHP
GKHKKMMLFKFSLPDAKHMDDIVRLVALIPYYIDLVGRYRLSSQARNKTESGRQKAAEEA
YKELHNARQEALQKKKAEKKKMMEEAEAKMSAEVIRKKEAKERARQVKKAVPKMKMSRSH

>Q8L586

MAAPRNLTGDGGARQLVKDEESPAASSAAKGLLNDDSPTGKRTKSERFPLSRWEFAVFFT
VFLVFTTGLFCIYLTMPAAEYGKLKVPRTISDLRLLKENLGSYASEYQARFILGYCSTYI
FMQTFMIPGTIFMSLLAGALFGVVRGFVLVVLNATAGACSCFFLSKLVGRPLVNWLWPEK
LRFFQAEIAKRRDRLLNYMLFLRITPTLPNLFINLSSPIVDIPFHVFFLATLVGLMPASY
ITVRAGLALGDLRSVKDLYDFKTLSVLFLIGSISIFPALLKRKRVYE

(6) $\mathbb{S}_6$: 22 extracell proteins

>Q40966

MGITGMTYMFTMVLSLIVLIFSASTVGFDYFQFTQQYQPAVCNSNPTPCNDPTDKLFTVH
GLWPSNRNGPDPEKCKTTTMNSQKIGNMTAQLEIIWPNVLNRSDHVGFWEREWLKHGTCG
YPTIKDDMHYLKTVIKMYITQKQNVSAILSKATIQPNGNNRSLVDIENAIRSGNNNTKPK
FKCQKNTRTTTELVEVTLCSNRDLTKFINCPHGPPKGSRYFCPANVKY

>P00693

MGKNGSLCCFSLLLLLLLAGLASGHQVLFQGFNWESWKQSGGWYNMMMGKVDDIAAAGVT
HVWLPPPSHSVSNEGYMPGRLYDIDASKYGNAAELKSLIGALHGKGVQAIADIVINHRCA
DYKDSRGIYCIFEGGTSDGRLDWGPHMICRDDTKYSDGTANLDTGADFAAAPDIDHLNDR
VQRELKEWLLWLKSDLGFDAWRLDFARGYSPEMAKVYIDGTSPSLAVAEVWDNMATGGDG
KPNYDQDAHRQNLVNWVDKVGGAASAGMVFDFTTKGILNAAVEGELWRLIDPQGKAPGVM
GWWPAKAATFVDNHDTGSTQAMWPFPSDKVMQGYAYILTHPGIPCIFYDHFFNWGFKDQI
AALVAIRKRNGITATSALKILMHEGDAYVAEIDGKVVVKIGSRYDVGAVIPAGFVTSAHG
NDYAVWEKNGAAATLQRS

>Q9T0N8

MAVVYYLLLAGLIACSHALAAGTPALGDDRGRPWPASLAALALDGKLRTDSNATAAASTD
FGNITSALPAAVLYPSSTGDLVALLSAANSTPGWPYTIAFRGRGHSLMGQAFAPGGVVVN
MASLGDAAAPPRINVSADGRYVDAGGEQVWIDVLRASLARGVAPRSWNDYLYLTVGGTLS
NAGISGQAFRHGPQISNVLEMDVITGHGEMVTCSKQLNADLFDAVLGGLGQFGVITRARI

AVEPAPARARWVRFVYTDFAAFSADQERLTAPRPGGGGASFGPMSYVEGSVFVNQSLATD
LANTGFFTDADVARIVALAGERNATTVYSIEATLNYDNATAAAAAVDQELASVLGTLSYV
EGFAFQRDVAYAAFLDRVHGEEVALNKLGLWRVPHPWLNMFVPRSRIADFDRGVFKGILQ
GTDIVGPLIVYPLNKSMWDDGMSAATPSEDVFYAVSLLFSSVAPNDLARLQEQNRRILRF
CDLAGIQYKTYLARHTDRSDWVRHFGAAKWNRFVEMKNKYDPKRLLSPGQDIFN
>Q40161
MHTKIHLPPCILLLLLFSLPSFNVVVGGDGESGNPFTPKGYLIRYWKKQISNDLPKPWFL
LNKASPLNAAQYATYTKLVADQNALTTQLHTFCSSANLMCAPDLSPSLEKHSGDIHFATY
SDKNFTNYGTNEPGIGVNTFKNYSEGENIPVNSFRRYGRGSPRDNKFDNYASDGNVIDQS
FNSYSTSTAGGSGKFTNYAANANDPNLHFTSYSDQGTGGVQKFTIYSQEANAGDQYFKSY
GKNGNGANGEFVSYGNDTNVIGSTFTNYGQTANGGDQKFTSYGFNGNVPENHFTNYGAGG
NGPSETFNSYRDQSNVGDDTFTTYVKDANGGEANFTNYGQSFNEGTDVFTTYGKGGNDPH
INFKTYGVNNTFKDYVKDTATFSNYHNKTSQVLASLMEVNGGKKVNNRWVEPGKFFREKM
LKSGTIMPMPDIKDKMPKRSFLPRVIASKLPFSTSKIAELKKIFHAGDESQVEKMIGDAL
SECERAPSAGETKRCVNSAEDMIDFATSVLGRNVVVRTTEDTKGSNGNIMIGSVKGINGG
KVTKSVSCHQTLYPYLLYYCHSVPKVRVYEADILDPNSKVKINHGVAICHVDTSSWGPSH
GAFVALGSGPGKIEVCHWIFENDMTWAIAD
>P28493
MANISSIHILFLVFITSGIAVMATDFTLRNNCPTTVWAGTLAGQGPKLGDGGFELTPGAS
RQLTAPAGWSGRFWARTGCNFDASGNGRCVTGDCGGLRCNGGGVPPVTLAEFTLVGDGGK
DFYDVSLVDGYNVKLGIRPSGGSGDCKYAGCVSDLNAACPDMLKVMDQNNVVACKSACER
FNTDQYCCRGANDKPETCPPTDYSRIFKNACPDAYSYAYDDETSTFTCTGANYEITFCP
>P33154
MNFTGYSRFLIVFVALVGALVLPSKAQDSPQDYLRVHNQARGAVGVGPMQWDERVAAYAR
SYAEQLRGNCRLIHSGGPYGENLAWGSGDLSGVSAVNMWVSEKANYNYAANTCNGVCGHY
TQVVWRKSVRLGCAKVRCNNGGTIISCNYDPRGNYVNEKPY
>P93164
MPNDSVLSLFFFVTLFTCLLSATSHDDHIFLPSQLHDDDSVSCTATDPSLNYKPVIGILT
HPGDGASGRLSNATGVSYIAASYVKFVESGGARVIPLIYNESPENLNKKLDLVNGVLFTG
GWAVSGPYLDTLGNIFKKALERNDAGDHFPVIAFNLGGNLVIRIVSEQTDILEPFTASSL
PSSLVLWNEANAKGSLFQRFPSDLLTQLKTDCLVLHNHRYAISPRKLQYNTKLSDFFEIL
ATSGDRDGKTFVSTARGRKYPVTVNLWQPEKNAFEWATSLKAPHTEDAIRVTQSTANFFI
SEARKSTNTPDAQKVRDSLIYNYKPTFGGTAGKGYDQVYLFE
>P80691
VAPKPIVDIDGKPVLYGVDYFVVSAIWGAGGGGLTVYGPGNKKKCPLSVVQDPFDNGEPI
IFSAIKNVKDNIVFESVDLNVKFNITINCNETTAWKVDRFPGVIGWTVTLGGEKGYHGFE
STHSMFKIKKAGLPFSYKFHFCPSYPRTRLIPCNNVDIFFDKYRIRRLILTNDAKEFVFI
KTNR
>Q05091
MELKFSTFLSLTLLFSSVLNPALSDLCNPDDKKVLLQIKKAFGDPYVLASWKSDTDCCDW
YCVTCDSTTNRINSLTIFAGQVSGQIPALVGDLPYLETLEFHKQPNLTGPIQPAIAKLKG
LKSLRLSWTNLSGSVPDFLSQLKNLTFLDLSFNNLTGAIPSSLSELPNLGALRLDRNKLT
GHIPISFGQFIGNVPDLYLSHNQLSGNIPTSFAQMDFTSIDLSRNKLEGDASVIFGLNKT
TQIVDLSRNLLEFNLSKVEFPTSLTSLDINHNKIYGSIPVEFTQLNFQFLNVSYNRLCGQ

IPVGGKLQSFDEYSYFHNRCLCGAPLPSCK
>P33432
MKALFLIGLLALVASTAFAQYSEVVGSYDVAGGGGAQQCPVETKLNSCRNYLLDRCSTMK
DFPVTWRWWKWWKGGCQELLGECCSRLGQMPPQCRCNIIQGSIQGDLGGIFGFQRDRASK
VIQEAKNLPPRCNQGPPCNIPGTIGYYW
>Q9S7Y7
MASSSSSLAFSLSLLLALILCFSPTQSYKTIGKGYRLVSIEESPDGGFIGYLQVKQKNKI
YGSDITTLRLFVKHETDSRLRVHITDAKQQRWEVPYNLLPREQPPQVGKVIGKSRKSPIT
VQEISGSELIFSYTTDPFTFAVKRRSNHETLFNTTSSLVFKDQYLEISTSLPKEASLYGL
GENSQANGIKLVPNEPYTLYTEDVSAINLNTDLYGSHPMYMDLRNVGGKAYAHAVLLLNS
NGMDVFYRGDSLTYKVIGGVFDFYFIAGPSPLNVVDQYTQLIGRPAPMPYWSLGFHQCRW
GYHNLSVVEDVVDNYKKAKIPLDVIWNDDDHMDGHKDFTLNPVAYPRAKLLAFLDKIHKI
GMKYIVINDPGIGVNASYGTFQRAMAADVFIKYEGKPFLAQVWPGPVYFPDFLNPKTVSW
WGDEIKRFHDLVPIDGLWIDMNEVSNFCSGLCTIPEGKQCPSGEGPGWVCCLDCKNITKT
RWDDPPYKINATGVVAPVGFKTIATSATHYNGVREYDAHSIYGFSETIATHKGLLNVQGK
RPFILSRSTFVGSGQYAAHWTGDNQGTWQSLQVSISTMLNFGIFGVPMVGSDICGFYPQP
TEELCNRWIEVGAFYPFSRDHANYYSPRQELYQWDTVADSARNALGMRYKILPFLYTLNY
EAHMTGAPIARPLFFSFPEYTECYGNSRQFLLGSSFMISPVLEQGKTEVEALFPPGSWYH
MFDMTQAVVSKNGKRVTLPAPLNFVNVHLYQNTILPTQQGGLISKDARTTPFSLVIAFPA
GASEGYATGKLYLDEDELPEMKLGNGQSTYVDFYASVGNGTMKMWSQVKEGKFALSKGWV
IEKVSVLGLRGAGQVSEIQINGSPMTKKIEVSSKEHTYVIGLEDEEENKSVMVEVRGLEM
LVGKDFNMSWKMGIN
>Q8GW72
MNSQITLFFFFFSILSLSQISNSSSLLKPHPCPILPLPSSQQLQWQLGSMAMFLHFGPNT
FTDSEWGTGKANPSIFNPTHLNASQWVQIAKDSGFSRVILTAKHHDGFCLWPSEYTDYSV
KSSQWRNGAGDVVAGLASAAKEAGIGLGLYLSPWDRHEQCYGKTLEYNEFYLSQMTELLT
KYGEIKEVWLDGAKGDGEKDMEYFFDTWFSLIHQLQPKAVIFSDAGPDVRWIGDEAGLAG
STCWSLFNRTNAKIGDTEPSYSQEGDGYGQDWVPAECDVSIRPGWFWHASESPKPAVQLL
DIYYNSVGRNCLFLLNVPPNSSGLISEQDIKVLEEFSEMKNSIFSNNLARKAFVNSSSIR
GDQSSQFGPKNVLEEGLDKYWAPEENQNEWVLYLEFKDLVSFNVLEIREPIHMGQRIASF
HLETRKTGSGEWERVVSGTTVGNKRLLRFLNVVESRSLKLVVDKARTDPLISYLGLYMDK
FSGSSRNTTKITITRTLKEEQQLHDL
>Q38716
MATVQKSQHSHFFLLVGCIVHLSNFCSTTTAQFDYFKLVLQWPNSYCSLKTTHCPRTRLP
SQFTIHGLWPDNKSWPLSNCRDTSADVLKITDKGLIQDLAVHWPDLTRRQRKVPGQKFWV
TQWKKHGACALPMYSFNDYFVKALELKKRNNVLDMLSRKSLTPGDQRVDVSDVNGAITKV
TGGIAILKCPEGYLTEVIICFDPSGFPVIDCPGPFPCKDDPLEFQVLSRRKFQDL
>P93349
MGVKGLLFSIVLINLSLLGLCGYPRKPVDVPFWKNYEPSWASHHIKYLSGGSTVDLVLDR
SSGAGFQSKKSYLFGHFSMKLKLVGGDSAGVVTAFYLSSNNAEHDEIDFEFLGNRTGQPY
ILQTNVFTGGKGDREQRIYLWFDPTKGYHSYSVLWNTFQIVIFVDDVPIRAFKNSKDLGV
KFPFNQPMKIYSSLWDADDWATRGGLEKTDWSNAPFTASYTSFHVDGCEAATPQEVQVCN
TKGMRWWDQKAFQDLDALQYRRLRWVRQKYTIYNYCTDRKRYPTLPPECTKDRDI
>Q94EG3

MAAFGINSKIFQSMEMAILFLLAISIDRYCFAADEDMLQDVCVADLHSKVKVNGFPCKTN
FTAADFSSLAISKPGATNNKFGSVVTTANVEQVPGLNTLGVSLARIDYAPGGINPPHTHP
RASEMVFVMEGELDVGFITTANVLVSKKIIKGEVFVFPRGLVHFQKNNGEVPAAVISAFN
SQLPGTQSIPITLFGASPPVPDDVLAQTFQINTEDVQQIKSKFAPVKKF
>Q9FXE5
MNPILSSLFALSLLSSLSPSTHAHQCHFPAIFNFGDSNSDTGGLSAAFGQAGPPHGSSFF
GSPAGRYCDGRLVIDFIAESLGLPYLSAFLDSVGSNFSHGANFATAGSPIRALNSTLRQS
GFSPFSLDVQFVQFYNFHNRSQTVRSRGGVYKTMLPESDSFSKALYTFDIGQNDLTAGYF
ANKTVEQVETEVPEIISQFMNAIKNIYGQGGRYFWIHNTGPIGCLAYVIERFPNKASDFD
SHGCVSPLNHLAQQFNHALKQAVIELRSSLSEAAITYVDVYSLKHELFVHAQGHGFKGSL
VSCCGHGGKYNYNKGIGCGMKKIVKGKEVYIGKPCDEPDKAVVWDGVHFTQAANKFIFDK
IAPGLSKACKRQ
>P81131
MMRKALLALCVATAFAVAQAQNVAYPNFPYCQCIKSPSPYSLEPVVKSNRTGQYCFTLRV
TKPSPSATGYCATKADIKKIEINVNQVCDVFGNVVNATLNGVPTKVGPAFDTPPDGPNTS
RILRFTQLNLGLDSDGAMLCITLGQNDKGKGCTTLEDLCAPPAGAPKGTCSLALFDSKPD
CCPISRVSPPAPPPPPPPPPPPEPVAVPITPPCKTCVYATITALPPLLFPFQLTPSICQSV
ADKIAGDLEMIVTSYSIGYSGATITCSGNVIKVCASFSLPPGAPYTGLQADISNALTFWL
SLLAPSTGCPAYFANHQVTVTVGGDGDPNSVTCLEGTATTTCKPGNPDFPKCECETKPAA
TRFAALPTLTQEPGRPSNRTNSTLYCFTLQVVAPLNPNGLCGNTTTLLKAELWGNDIPTQ
RRKILALAFKAAGASSPLRYLSPSWGSAGEQTLKVSGLNWDASQADGAKICMELSNDTNL
KTFCNTGQDTCWINLFSPDKQCCPLFAASLTP
>P42820
MTLLLKNTLYLALIISVISSFPTSLFAQNCGCAPNLCCSNFGFCGTGTPYCGVGNCQSGP
CEGGTPTTPTTPTTPTTPGTGGGGSSVSDIVSQAFFDGIIGQAAASCPGKNFYTRAAFLS
AVDPKFGNEGSSDDNKREIAAFFAHISHETTNLCHIEERDGDVGDAYCDQDKAAQYPCAA
GKKYYGRGPLQLSWNYNYALAGQAIGFDGLGNPEKVATDVNTSFKAAMWFWMTNVHSVMN
QGFGATTKAINGALECNGQNQDQANDRIQFYKKYCADFGVAPGDNLTC
>P19172
MTNMTLRKHVIYFLFFISCSLSKPSDASRGGIAIYWGQNGNEGNLSATCATGRYAYVNVA
FLVKFGNGQTPELNLAGHCNPAANTCTHFGSQVKDCQSRGIKVMLSLGGGIGNYSIGSRE
DAKVIADYLWNNFLGGKSSSRPLGDAVLDGIDFNIELGSPQHWDDLARTLSKFSHRGRKI
YLTGAPQCPFPDRLMGSALNTKRFDYVWIQFYNNPPCSYSSGNTQNLFDSWNKWTTSIAA
QKFFLGLPAAPEAAGSGYIPPDVLTSQILPTLKKSRKYGGVMLWSKFWDDKNGYSSSILA
SV
>P05117
MVIQRNSILLLIIIFASSISTCRSNVIDDNLFKQVYDNILEQEFAHDFQAYLSYLSKNIE
SNNNIDKVDKNGIKVINVLSFGAKGDGKTYDNIAFEQAWNEACSSRTPVQFVVPKNKNYL
LKQITFSGPCRSSISVKIFGSLEASSKISDYKDRRLWIAFDSVQNLVVGGGGTINGNGQV
WWPSSCKINKSLPCRDAPTALTFWNCKNLKVNNLKSKNAQQIHIKFESCTNVVASNLMIN
ASAKSPNTDGVHVSNTQYIQISDTIIGTGDDCISIVSGSQNVQATNITCGPGHGISIGSL
GSGNSEAYVSNVTVNEAKIIGAENGVRIKTWQGGSGQASNIKFLNVEMQDVKYPIIIDQN
YCDRVEPCIQQFSAVQVKNVVYENIKGTSATKVAIKFDCSTNFPCEGIIMENINLVGESG
KPSEATCKNVHFNNAEHVTPHCTSLEISEDEALLYNY

>Q9M099
MARTHFIFLLLVALLSTTFPSSSSSREQEKDRIKALPGQPKVAFSQYSGYVNVNQSHGRA
LFYWLTESSSPSPHTKPLLLWLNGGPGCSSIAYGASEEIGPFRINKTGSNLYLNKFAWNK
DANLLFLESPAGVGYSYTNTSSDLKDSGDERTAQDNLIFLIKWLSRFPQYKYRDFYIAGE
SYAGHYVPQLAKKINDYNKAFSKPIINLKGFLVGNAVTDNQYDSIGTVTYWWTHAIISDK
SYKSILKYCNFTVERVSDDCDNAVNYAMNHEFGDIDQYSIYTPTCVAAQQKKNTTGFFVR
MKNTLLRRRLVSGYDPCTESYAEKYFNRPDVQRAMHANVTGIRYKWTACSDVLIKTWKDS
DKTMLPIYKELAASGLRIWIFSGDTDSVVPVTATRFSLSHLNLPVKTRWYPWYTDNQVGG
WTEVYKGLTFATVRGAGHEVPLFEPKRALILFRSFLAGKELPRSY
>P52399
MALCIKNGFLAAALVLVGLLMCSIQMIGAQSIGVCYGKIANNLPSEQDVINLYKANGIRK
MRIYYPDKNIFKALKGSNIEIILDVPNQDLEALANSSIANGWVQDNIRSHFPYVKFKYIS
IGNEVSPINNGQYSQFLLHAMENVYNALAASGLQDKIKVTTATYSGLLANTYPPKASIFR
GEFNSFINPIIQFLAQNNLPLLANVYPYFVHISNTADVPLSYALFTQRGKNSAGYQNLFD
AILDSMYFAVEKAGGPNVEIIVSESGWPSEGNSAATIENAQTYYRNLIDHVKRGAGTPKK
PGKSIETYLFAMFDENVKKGEITEKHFGLFSPDQRAKYQLNFNSLMPIYIDISRVI

(7) $\mathbb{S}_7$: 21 Golgi apparatus proteins

>Q9M2Y6
MARRQVGSTRRVGDGGSFPFAGALHSKSRSSPLLSICLVLVGACLLIGYAYSGPGIFKSI
KEVSKVTGDYSCTAEVQRAIPVLKKAYGDGMRKVLHVGPDTCSVVSSLLKEEETEAWGVE
PYDIEDADSHCKSFVSKGLVRVADIKFPLPYRAKSFSLVIVSDALDYLSPKYLNKTVPEL
ARVASDGVVLFAGLPGQQRAKVAELSKFGRPAKMRSASWWNRFFVQTNLEENDAPSKKFE
QAVSKGLYKPACQVFHLKPLH
>Q9SA77
MFSFGRARSQGRQNRSMSLGGLDYADPKKKNNYLGKILLTASLTALCIFMLKQSPTFNTP
SVFSRHEPGVTHVLVTGGAGYIGSHAALRLLKESYRVTIVDNLSRGNLAAVRILQELFPE
PGRLQFIYADLGDAKAVNKIFTENAFDAVMHFAAVAYVGESTQFPLKYYHNITSNTLVVL
ETMAAHGVKTLIYSSTCATYGEPDIMPITEETPQVPINPYGKAKKMAEDIILDFSKNSDM
AVMILRYFNVIGSDPEGRLGEAPRPELREHGRISGACFDAARGIMPGLQIKGTDYKTADG
TCVRDYIDVTDLVDAHVKALQKAKPRKVGIYNVGTGKGSSVKEFVEACKKATGVEIKIDY
LPRRAGDYAEVYSDPSKIRKELNWTAKHTNLKESLETAWRWQKLHRNGYGLTTSSVSVY
>Q9SWH4
MATRNRTTVYRKHRDACKSARAPLSLSASDSFGGPVIEMVSGSFSRSNHSSYAPLNSYDP
GPSSSDAFTIGMPPAWVDDSEEITFNIQKVRDKMNELAKAHSKALMPTFGDNKGIHREVE
MLTHEITDLLRKSEKRLQMLSTRGPSEESNLRKNVQRSLATDLQNLSMELRRKQSTYLKR
LQQQKEGQDEVDLEFNVNGKMSRLDEEDELGGMGFDEHQTIKLKEGQHVSAERERERIQQV
LGSVNDLAQIMKDLSALVIDQGTIVDRIDYNVQNVSTSVEEGYKQLQKAERTQREGAMVK
CATILLVLCLIMIVLLILKNILF
>Q8W486
MRRLGHHRLHGKTGGVGTKGMVAKLSIGVIVLLICTLSLLFSANIGSNREPTRPSKINVE
ELWESAKSGGWRPSSAPRSDWPPPTKETNGYLRVRCNGGLNQQRSAICNAVLAARIMNAT
LVLPELDANSFWHDDSGFQGIYDVEHFIETLKYDVKIVGKIPDVHKNGKTKKIKAFQIRP
PRDAPIEWYLTTALKAMREHSAIYLTPFSHRLAEEIDNPEYQRLRCRVNYHALRFKPHIM

KLSESIVDKLRSQGHFMSIHLRFEMDMLAFAGCFDIFNPEEQKILRKYRKENFADKRLIY
NERRAIGKCPLTPEEVGLILRAMRFDNSTRIYLAAGELFGGEQFMKPFRTLFPRLDNHSS
VDPSEELSATSQGLIGSAVDYMVCLLSDIFMPTYDGPSNFANNLLGHRLYYGFRTTIRPD
RKALAPIFIAREKGKRAGFEEAVRRVMLKTNFGGPHKRVSPESFYTNSWPECFCQMNPKK
SSDKCPPNNVIEILDSRLESIRDPDSTSQTNSTVTGLER
>Q941R4
MKLYEHDGVDLEDGKTVKSGGDKPIPRKIHNRALLSGLAYCISSCSMILVNKFVLSSYNF
NAGIFLMLYQNFVSVIIVVGLSLMGLITTEPLTLRLMKVWFPVNVIFVGMLITSMFSLKY
INVAMVTVLKNVTNVITAVGEMYLFNKQHDNRVWAALFLMIISAVSGGITDLSFNAVGYA
WQIANCFLTASYSLTLRKTMDTAKQVTQSGNLNEFSMVLLNNTLSLPLGLLLSYFFNEMD
YLYQTPLLRLPSFWMVMTLSGLLGLAISFTSMWFLHQTGATTYSLVGSLNKIPLSIAGIV
LFNVPTSLQNSASILFGLVAGVVFARAKMREKS
>Q946Y7
MSSAQDPFYIVKEEIQDSIDKLQSTFHKWERISPDMGDQAHVAKELVATCGSIEWQVDEL
EKAITVAAKDPSWYGIDEAELEKRRRWTSNARTQVRNVKSGVLAGKVSSGAGHASEVRRE
LMRMPNSGEASRYDQYGGRDDDGFVQSESDRQMLLIKQQDEELDELSKSVQRIGGVGLTI
HDELVAQERIIDELDTEMDSTKNRLEFVQKKVGMVMKKAGAKGQMMMICFLLVLFIILFV
LVFLT
>Q8RXL8
MSEGQKFQLGTIGALSLSVVSSVSIVICNKALISTLGFTFATTLTSWHLLVTFCSLHVAL
WMKMFEHKPFDPRAVMGFGILNGISIGLLNLSLGFNSVGFYQMTKLAIIPCTVLLETLFF
RKKFSRKIQFSLTILLLGVGIATVTDLQLNMLGSVLSLLAVVTTCVAQIMTNTIQKKFKV
SSTQLLYQSCPYQAITLFVTGPFLDGLLTNQNVFAFKYTSQVVFFIVLSCLISVSVNFST
FLVIGKTSPVTYQVLGHLKTCLVLAFGYVLLRDPFDWRNILGILVAVIGMVVYSYYCSIE
TQQKASETSTQLPQMKESEKDPLIAAENGSGVLSDGGGGVQQKTVAPVWNSNKDFQA
>O04300
MASLPKPTPLLKDELDIVIPTIRNLDFLEMWRPFFEQYHLIIVQDGDPSKVIKVPEGFDY
ELYNRNDINRILGPKASCISFKDSACRCFGYMVSKKKYIYTIDDDCFVAKDPTGHEINAL
EQHIKNLLSPSTPFFFNTLYDPYREGTDFVRGYPFSLREGVPTAVSHGLWLNIPDYDAPT
QLVKPHERNTRFVDAVLTIPKGSLFPMCGMNLAFNRELIGPAMYFGLMGDGQPIGRYDDM
WAGWCIKVICDHLGYGVKTGLPYIWHSKASNPFVNLKKEYKGIFWQEEIIPFFQAATLSK
DCTSVQKCYIELSKQVKEKLGTIDPYFIKLADAMVTWVEAWDEINNNKSEETTSTKASEV
AATK
>Q9SE83
MEAIDELSQLSDSMKQAASLLADEDPDETSSSKRPATFLNVVALGNVGAGKSAVLNSLIG
HPVLPTGENGATRAPIIIELSRESSLSSKAIILQIDNKSQQVSASALRHSLQDRLSKGAS
GKNRDEINLKLRTSTAPPLKLVDLPGLDQRIVDESMIAEYAQHNDAILLVIVPASQASEI
SSSRALKIAKEYDPESTRTIGIIGKIDQAAENSKALAAVQALLSNQGPPKTTDIPWVAVI
GQSVSIASAQSGSGENSLETAWRAESESLKSILTGAPQSKLGRIALVDTLASQIRSRMKL
RLPSVLSGLQGKSQIVQDELARLGEQLVNSAEGTRAIALELCREFEDKFLLHLAGGEGSG
WKVVASFEGNFPNRIKQLPLDRHFDLNNVKRVVLEADGYQPYLISPEKGLRSLIKIVLEL
AKDPARLCVDEVHRVLVDIVSASANATPGLGRYPPFKREVVAIASAALDGFKNEAKKMVV
ALVDMERAFVPPQHFIRLVQRRMERQRREEELKGRSSKKGQDAEQSLLSRATSPQPDGPT
AGGSLKSMKDKPSPQDKETPEVSGLKTAGPEGEITAGYLMKKSAKTNGWSRRWFVLNEKT

GKLGYTKKQEERNFRGTITLEECTIEEIPEDEVEKSKSSKDKKANGPDSKGPGLVFKITC
KVPYKTVLKAHNALVLKAESVVDKNEWINKLQKVIQARGGQVGSVSMRQSLSEGSLDKMV
RKPIDPEEELRWMSQEVRGYVEAVLNSLAANVPKAVVLCQVEKAKEDMLNQLYSSISAIG
NERIESLIQEDQNVKRRRERYQKQSSLLSKLTRQLSIHDNRAAAASSYSDNSGTESSPRA
SGGSSGDDWMNAFNSAANGPSDSLSKYGSGGHSRRYSDPAQNGDAASPGSGSNRRTTPNR
LPPAPPPTGSAYRY

>Q7XJ98

MFPRVSMRRRSAEVSPTEPMEKGNGKNQTNRICLLVALSLFFWALLLYFHFVVLGTSNID
KQLQLQPSYAQSQPSSVSLRVDKFPIEPHAAPSKPPPKEPLVTIDKPILPPAPVANSSST
FKPPRIVESGKKQEFSFIRALKTVDNKSDPCGGKYIYVHNLPSKFNEDMLRDCKKLSLWT
NMCKFTTNAGLGPPLENVEGVFSDEGWYATNQFAVDVIFSNRMKQYKCLTNDSSLAAAIF
VPFYAGFDIARYLWGYNISRRDAASLELVDWLMKRPEWDIMRGKDHFLVAGRITWDFRRL
SEEETDWGNKLLFLPAAKNMSMLVVESSPWNANDFGIPYPTYFHPAKDSEVFEWQDRMRN
LERKWLFSFAGAPRPDNPKSIRGQIIDQCRNSNVGKLLECDFGESKCHAPSSIMQMFQSS
LFCLQPQGDSYTRRSAFDSMLAGCIPVFFHPGSAYTQYTWHLPKNYTTYSVFIPEDDVRK
RNISIEERLLQIPAKQVKIMRENVINLIPRLIYADPRSELETQKDAFDVSVQAVIDKVTR
LRKNMIEGRTEYDYFVEENSWKYALLEEGQREAGGHVWDPFFSKPKPGEDGSSDGNGGTT
ISADAAKNSWKSEQRDKTQ

>Q9LS42

MEVSQDGSERDKTPPPSSSSSSSSPIPVVTNFWKEFDLEKEKSLLDEQGLRIAENQENSQ
KNRRKLAESTRDFKKASPENKLSMFNSLLKGYQEEVDNITKRAKFGENAFLNIYQKLYEA
PDPFPALASIAEQDRKLSEVESENRKMKVELEEFRTEATHLKNQQATIRRLEERNRQLEQ
QMEEKIKEVVEIKQRNLAEENQKTMELLKDREQALQDQLRQAKDSVSTMQKLHELAQNQL
FELRAQSDEETAGKQSEVSLLMDEVERAQTRLLTLEREKGHLRSQLQTANEDTDNKKSDN
IDSNSMLENSLTAKEKIISELNMEIHNVETALANERESHVAEIKKLNSLLNKKDTIIEEM
KKELQERPSAKLVDDLRKKVKILQAVGYNSIEAEDWDAATTGEEMSKMESLLLDKNRKME
HEVTQLKVQLSEKASLLEKAEAKGEELTAKVNEQQRLIQKLEDDILKGYGSKERKGALFD
EWEFSEAGVAEQSEPMDQKHVPSEQDQSSMLKVICSQRDRFRARLRETEEEIRRLKEKIG
FLTDELEKTKADNVKLYGKIRYVQDYNHDKVVSRGSKKYVEDLESGFSSDVESKYKKIYE
DDINPFAAFSKKEREQRIKDLGIRDRITLSSGRFLLGNKYARTFAFFYTIGLHVLVFTCL
YRMSAYSYLSHGAEETLMTEATTNLPHGL

>Q56ZN6

MMMDEDVEQASLMSFNDRPRAFPNMRSKTYSPLIFRIIRKLNVRVLSIILLFCFGAIFYM
GASTSPIIVFVFTVCIISFLLSIYLTKWVLAKDEGPPEMVEISDAIRDGAEGFFRTQYST
ISKMAILLAFVILCIYLFRSLTPQQEAAGLGRAMSAYITVAAFLLGALCSGIAGYVGMWV
SVRANVRVSSAARRSAREALQIAVRAGGFSALVVVGMAVIGIAILYSTFYVWLGVGSPGS
MNVTDLPLLLVGYGFGASFVALFAQLGGGIYTKGADVGADLVGKVEQGIPEDDPRNPAVI
ADLVGDNVGDCAARGADLFESIAAEIISAMILGGTMAKKCKIEDPSGFILFPLVVHSFDL
IISSIGILSIKGTRDASVKSPVEDPMAVLQKGYSLTIILAVITFGASTRWLLYTEQAPSA
WFNFALCGLVGIITAYIFVWISKYYTDYKHEPVRTLALASSTGHGTNIIAGVSLGLESTA
LPVLTISVAIISAYWLGNTSGLVDENGIPTGGLFGTAVATMGMLSTAAYVLTMDMFGPIA
DNAGGIVEMSQQPESVREITDLLDAVGNTTKATTKGFAIGSAALASFLLFSAYMDEVSAF
ANVSFKEVDIAIPEVFVGGLLGAMLIFLFSAWACAAVGRTAQEVVNEVRRQFIERPGIME
YKEKPDYSRCVAIVASAALREMIKPGALAIASPIVVGLVFRILGYYTGQPLLGAKVVASM

LMFATVCGILMALFLNTAGGAWDNAKKYIETGALGGKGSEAHKAAVTGDTVGDPFKDTAG
PSIHVLIKMLATITLVMAPVFL
>Q9SA23
MASSSDSWMRAYNEALKLSEEINGMISERSSSAVTGPDAQRRASAIRRKITIFGNKLDSL
QSLLAEIHGKPISEKEMNRRKDMVGNLRSKANQMANALNMSNFANRDSLLGPDIKPDDSM
SRVTGMDNQGIVGYQRQVMREQDEGLEQLEGTVMSTKHIALAVSEELDLQTRLIDDLDYH
VDVTDSRLRRVQKSLAVMNKNMRSGCSCMSMLLSVLGIVGLAVVIWMLVKYM
>Q9ZSY2
MSAKKLEGSSAPANRRDPYEVLCVSKDANDQEIKSAYRKLALKYHPDKNANNPDASELFK
EVAFSYSILSDPEKRRHYDNAGFEALDADGMDMEIDLSNLGTVNTMFAALFSKLGVPIKT
TVSANVLEEAMNGTVTVRPLPIGTSVSGKVEKQCAHFFGVTISEQQAESGVVVRVTSTAQ
SKFKLLYFEQDSSGGYGLALQEEREKTGKVTSAGMYFLHFQVYRMDTTVNALAAAKDPES
AFFKRLEGLQPCEVSELKAGTHIFAVYGDNFFKTASYTIEALCAKTYEDTTEKLKEIEAQ
ILRKRNELRQFETEYRKALARFQEVTNRYTQEKQTVDELLKQRDTIHSTFSVVKTPSGNN
LSNGSSSKAQGDESKGDGDSAGEEGGTENRDKSKRKWFNLNLKGSDKKLG
>Q9LK31
MANKPDHHHHHHQSSRRLMLVLYFTSVLGIGFIAAFLCLSSSIPSVSAVFSIWVPVNRPE
IQIPIIDSKIVQKRSKQSNDTKDHVRFLSAIFADIPAPELKWEEMESAPVPRLDGYSVQI
NNLLYVFSGYGSLDYVHSHVDVFNFTDNKWCDRFHTPKEMANSHLGIVTDGRYVYVVSGQ
LGPQCRGPTSRSFVLDSFTKTWLEFPSLPAPRYAPATQIWRGRLHVMGGSKENRNAVAFD
HWSIAVKDGKALDEWREEVPIPRGGPHRACVVANDKLLVIGGQEGDFMAKPNSPIFKCSR
RREIFNGEVYMMDEEMKWKMLPPMPKNNSHIESAWIIVNNSIVIVGGTTDWHPVTKRLVL
VGEIFRFQLDTLTWSVIGRLPYRVKTAMAGFWNGYLYFTSGQRDRGPDNPQPGKVIGEMW
RTKLKF
>Q9SEL5
MSDVFEGYERQYCELSTNLSRKCHSASVLSNGEEKKGKIAEIKSGIDEADVLIRKMDLEA
RSLQPSAKAVCLSKLREYKSDLNQLKKEFKRVSSADAKPSSREELMESGMADLHAVSADQ
RGRLAMSVERLDQSSDRIRESRRLMLETEEVGISIVQDLSQQRQTLLHAHNKLHGVDDAI
DKSKKVLTAMSRRMTRNKWIITSVIVALVLAIILIISYKLSH
>Q5W7F2
MHFTKLDDSPMFRKQLQSMEESAEILRERSLKFYKGCRKYTEGLGEAYDGDIAFASALET
FGGGHNDPISVAFGGPVMTKFTIALREIGTYKEVLRSQVEHILNDRLLQFANMDLHEVKE
ARKRFDKASLTYDQAREKFLSLRKGTKSDVAAALEQELHTSRSMFEQARFNLVTALSNVE
AKKRFEFLEAVSGTMDAHLRYFKQGYELLHQMEPYINQVLTYAQQSRERSNYEQAALNEK
MQEYKRQVDRESRWGSNGSNGSPNGDGIQAIGRSSHKMIDAVMQSAARGKVQTIRQGYLS
KRSSNLRGDWKRRFFVLDSRGMLYYYRKQCSKPSGSGSQLSGQRNSSELGSGLLSRWLSS
NNHGHGGVHDEKSVARHTVNLLTSTIKVDADQSDLRFCFRIISPTKNYTLQAESALDQMD
WIEKITGVIASLLSSQVPEQRLPGSPMGSGHHRSASESSSYESSEYDHPTTEEFVCERSF
LGYNERPSRSFQPQRSIRKGEKPIDALRKVCGNDKCADCGAPEPDWASLNLGVLVCIECS
GVHRNLGVHISKVRSLTLDVKVWEPSVISLFQALGNTFANTVWEELLHSRSAIHFDPGLT
VSDKSRVMVTGKPSYADMISIKEKYIQAKYAEKLFVRRSRDSDFPQSAAQQMWDAVSGND
KKAVYRLIVNGDADVNYVYDQTSSSSLTLSRVILVPERPKREDVLLRLRNELLDRTGSSS
NISPEGSGGSSLLHCACEKADLGMVELLLQYGANVNASDSSGQTPLHCCLLRGKVTIARL
LLTRGADPEAMNREGKTALDIAAESNFTDPEVLALLSDTNGYNHRQC

>Q6UDF0
MRNLIFEEPEGIPGNSSSSLRYAWQSIRAPVIIPLLKLAVIVCSVMSIMLFVERVAMAAV
ILIVKVLRKKRYTKYNLEAMKQKLERSKKYPMVLIQIPMYNEKEVYKLSIGAVCGLSWPA
DRFIVQVLDDSTNPVLRELVEMECQKWIQKGVNVKYENRRNRNGYKAGALKEGLEKQYVE
DCEFVAIFDADFQPDADFLWNTIPYLLENPKLGLVQARWKFVNSEECMMTRLQEMSLDYH
FSVEQEVGSSTYSFFGFNGTAGVWRIQAIKDAGGWKDRTTVEDMDLAVRASLHGWEFVFV
GDVKVKNELPSTFKAYRFQQHRWSCGPANLFKKMTKEIICCKRVPLLKRLHLIYAFFFVR
KIVAHWVTFFFYCIVIPACVIVPEVNLKKQIAIYIPATITILNAVSTPRSMHLLVLWILF
ENVMSLHRTKAAIIGLLEANRVNEWVVTEKLGNAMKQRNNARPSRASRFRIIERIHPLEI
IVGMYMLHCATYDLLFGHDHFFVYLLLQAGAFFTMGFGLVGTIVPT
>O49048
MVLVTSVRDYINRMLQDISGMKVLILDSETVSNVSIVYSQSELLQKEVFLVEMIDSISVS
KESMSHLKAVYFIRPTSDNIQKLRYQLANPRFGEYHLFFSNLLKDTQIHILADSDEQEVV
QQVQEYYADFVSGDPYHFTLNMASNHLYMIPAVVDPSGLQRFSDRVVDGIAAVFLALKRR
PVIRYQRTSDTAKRIAHETAKLMYQHESALFDFRRTESSPLLLVIDRRDDPVTPLLNQWT
YQAMVHELIGLQDNKVDLKSIGSLPKDQQVEVVLSSEQDAFFKSNMYENFGDIGMNIKRM
VDDFQQVAKSNQNIQTVEDMARFVDNYPEYKKMQGNVSKHVTLVTEMSKLVEARKLMTVS
QTEQDLACNGGQGAAYEAVTDLLNNESVSDIDRLRLVMLYALRYEKENPVQLMQLFNKLA
SRSPKYKPGLVQFLLKQAGVEKRTGDLFGNRDLLNIARNMARGLKGVENVYTQHQPLLFQ
TMESITRGRLRDVDYPFVGDHFQQGRPQEVVIFMVGGTTYEESRSVALQNATNSGVRFIL
GGTAVLNSKRFLKDLEEAQRISRSGSHMV
>P93026
MKLGLFTLSFLLILNLAMGRFVVEKNNLKVTSPDSIKGIYECAIGNFGVPQYGGTLVGTV
VYPKSNQKACKSYSDFDISFKSKPGRLPTFVLIDRGDCYFTLKAWIAQQAGAAAILVADS
KAEPLITMDTPEEDKSDADYLQNITIPSALITKTLGDSIKSALSGGDMVNMKLDWTESVP
HPDERVEYELWTNSNDECGKKCDTQIEFLKNFKGAAQILEKGGHTQFTPHYITWYCPEAF
TLSKQCKSQCINHGRYCAPDPEQDFTKGYDGKDVVVQNLRQACVYRVMNDTGKPWVWWDY
VTDFAIRCPMKEKKYTKECADGIIKSLGIDLKKVDKCIGDPEADVENPVLKAEQESQIGK
GSRGDVTILPTLVVNNRQYRGKLEKGAVLKAMCSGFQESTEPAICLTEDLETNECLENNG
GCWQDKAANITACRDTFRGRLCECPTVQGVKFVGDGYTHCKASGALHCGINNGGCWRESR
GGFTYSACVDDHSKDCKCPLGFKGDGVKNCEDVDECKEKTVCQCPECKCKNTWGSYECSC
SNGLLYMREHDTCIGSGKVGTTKLSWSFLWILIIGVGVAGLSGYAVYKYRIRSYMDAEIR
GIMAQYMPLESQPPNTSGHHMDI
>Q8H166
MSAKTILSSVVLVVLVAASAAANIGFDESNPIRMVSDGLREVEESVSQILGQSRHVLSFA
RFTHRYGKKYQNVEEMKLRFSIFKENLDLIRSTNKKGLSYKLGVNQFADLTWQEFQRTKL
GAAQNCSATLKGSHKVTEAALPETKDWREDGIVSPVKDQGGCGSCWTFSTTGALEAAYHQ
AFGKGISLSEQQLVDCAGAFNNYGCNGGLPSQAFEYIKSNGGLDTEKAYPYTGKDETCKF
SAENVGVQVLNSVNITLGAEDELKHAVGLVRPVSIAFEVIHSFRLYKSGVYTDSHCGSTP
MDVNHAVLAVGYGVEDGVPYWLIKNSWGADWGDKGYFKMEMGKNMCGIATCASYPVVA

(8) $\mathbb{S}_2$: 150 mitochondrion proteins

>P46269
MGKQPVKLKAVVYAISPFQQKIMPGLWKDLPGKIHHKVSENWISATLLLGPLVGTYSYVQ

HFLEKEKLEHRY
>Q8L6J3
MSSTKTPISLTIKLNQFTDKPTGLDINRYHNSPIMWRNIIKQLSSRTPQKLLFSSKNRTY
SFLGFGQDSVFKDNTKFRSLIPISCSNIVMGFQNLGEYLPGDEFLSRPLLKNQVNSNDFC
CRKSYASVAEAVAVSSTDAEEDVSVVDEVQELLTELKKEEKKQFAFRRRKQRMLTSGMGH
RKYQTLKRRQVKVETEAWEQAAKEYKELLFDMCEQKLAPNLPYVKSLFLGWFEPLRDKIA
EEQELCSQGKSKAAYAKYLYQLPADMMAVITMHKLMGLLMTGGDHGTARVVQAALVIGDA
IEQEVRIHNFLEKTKKQKAEKDKQKEDGEHVTQEQEKLRKKVTNLMKKQKLRAVGQIVRR
QDDSKPWGQDAKAKVGSRLIELLLQTAYIQPPANQLAVDPPDIRPAFLHSVRTVAKETKS
ASRRYGIIQCDELVFKGLERTARHMVIPYMPMLVPPVKWTGYDKGGHLYLPSYVMRTHGA
RQQREAVKRASRNQLQPVFEALDTLGSTKWRINKRVLSVIDRIWAGGGRLADLVDRDDAP
LPEEPDTEDEALRTKWRWKVKSVKKENRERHSQRCDIELKLAVARKMKDEEGFFYPHNVD
FRGRAYPMHPHLNHLGSDICRGVLVFAEGRPLGESGLRWLKIHLANLFAGGVEKLSLEGR
IAFTENHMDDIFDSADKPLEGRRWWLNAEDPFQCLAVCINLSEAVRSSSPETSISHIPVH
QDGSCNGLQHYAALGRDELGAAAVNLVAGEKPADVYSGIAARVLDIMKRDAQRDPAEFPD
AVRARALVNQVDRKLVKQTVMTSVYGVTYIGARDQIKRRLKERGAIADDSELFGAACYAA
KVTLTALGEMFEAARSIMTWLGECAKIIASENEPVRWTTPLGLPVVQPYRKIGRHLIKTS
LQILTLQQETEKVMVKRQRTAFPPNFIHSLDGSHMMMTAVACRRAGLNFAGVHDSYWTHA
CDVDKLNRILREKFVELYETPILEKLLESFQTSYPTLLFPPLPERGDFDLRDVLESPYFF
N
>Q8L785
MAILHFSLPLIVSFLRPHASPRFFLLPRSLSQSPFLSRRRFHRTSAVSSAAVHHQSYRNP
DDDVTRAVSVPTFQQAIQRLQEYWASVGCAVMQPSNTEVGAGTMNPCTFLRVLGPEPWNV
AYVEPSIRPDDSRYGENPNRLQRHTQFQVILKPDPGNSQQLFINSLSALGIDVTAHDIRF
VEDNWESPVLGAWGLGWEIWMDGMEITQFTYFQQAGSLPLSPVSVEITYGLERIIMLLQE
VDHFKKILYADGITYGELFLENEKEMSSYYLEHASVDRLQKHFDYFDEEARSLLALGLPI
PAYDQLLKTSHAFNILDARGFIGVTERARYFGRMRSLARQCAQLWLATRESLGHPLGVAS
EPVPPVCHRAALEKVAEKVSEDPRSFIIEIGTEEMPPQDVINASEQLRVLVLELLENQRL
RHGAVKAFGTPRRLVVLVDAMSSKQLEEEVEVRGPPASKAFDDEGNPTKAAEGFSRRYGV
PLEKLYRKVSGKTEYVHARVTEPARLALEVLSEDLPGILAKISFPKSMRWNSSVMFSRPI
RWVMALHGDLVVPFSFAGISSGNVSCGLRNTASASLLVQNAESYEDTMRNSGINIEIEER
KKIILEKSNALAKSVSGRLVVPQNLLNEVANLVEAPVPLIGKFKESFLELPEELLTIVMQ
KHQKYFSIIDESGQLLPYFIAVANGAINEDVVKKGNEAVLRARYEDAKFFYEVDTRKRFS
EFRDQLQGILFHEKLGTMLDKMNRLKKMVSKLCLALKIDEDLLPVVEDAASLAMSDLATA
VVTEFTALSGIMARHYALRDGYSEQIAEALLEITLPRFSGDVIPKTDAGMVLAIGDRLDS
LVGLFAAGCQPSSTNDPFGLRRISYGLVQILVEKDKNVNFKRVLELAASVQPTKVEANTV
EDVYQFVTRRLEQLLVDNGVSPEVVRSVLAERGNNPCLAARTAYKTEKLSKGEMFPKIVE
AYSRPTRIVRGKDVGVGVEVDENAFETPQERTLWSTYTSIKDRIHTGIEIEDFTEISMQL
VEPLEDFFNNVFVMVEEERVRKNRLALLNNIANLPKGVIDLSFLPGF
>P42055
MGKGPGLYTEIGKKARDLLYKDYQSDHKFSITTYSPTGVVITSSGSKKGDLFLADVNTQL
KNKNVTTDIKVDTNSNLFTTITVDEAAPGLKTILSFRVPDQRSGKLEVQYLHDYAGICTS
VGLTANPIVNFSGVVGTNIIALGTDVSFDTKTGDFTKCNAGLSFTNADLVASLNLNNKGD
NLTASYYHTVSPLTSTAVGAEVNHSFSTNENIITVGTQHRLDPLTSVKARINNFGKASAL

LQHEWRPKSLFTVSGEVDTKSVDKGAKFGLALALKP
>Q9SAK4
MVIGAAARVAIGGCRKLISSHTSLLLVSSQCRQMSMDAQSVSEKLRSSGLLRTQGLIGGK
WLDSYDNKTIKVNNPATGEIIADVACMGTKETNDAIASSYEAFTSWSRLTAGERSKVLRR
WYDLLIAHKEELGQLITLEQGKPLKEAIGEVAYGASFIEYYAEEAKRVYGDIIPPNLSDR
RLLVLKQPVGVVGAITPWNFPLAMITRKVGPALASGCTVVVKPSELTPLTALAAAELALQ
AGVPPGALNVVMGNAPEIGDALLTSPQVRKITFTGSTAVGKKLMAAAAPTVKKVSLELGG
NAPSIVFDDADLDVAVKGTLAAKFRNSGQTCVCANRVLVQDGIYDKFAEAFSEAVQKLEV
GDGFRDGTTQGPLINDAAVQKVETFVQDAVSKGAKIIIGGKRHSLGMTFYEPTVIRDVSD
NMIMSKEEIFGPVAPLIRFKTEEDAIRIANDTIAGLAAYIFTNSVQRSWRVFEALEYGLV
GVNEGLISTEVAPFGGVKQSGLGREGSKYGMDEYLEIKYVCLGDMNRH
>Q9FN42
MMRGLVSGAKMLSSTPSSMATSIATGRRSYSLIPMVIEHSSRGERAYDIFSRLLKERIIC
INGPINDDTSHVVVAQLLYLESENPSKPIHMYLNSPGGHVTAGLAIYDTMQYIRSPISTI
CLGQAASMASLLLAAGAKGQRRSLPNATVMIHQPSGGYSGQAKDITIHTKQIVRVWDALN
ELYVKHTGQPLDVVANNMDRDHFMTPEEAKAFGIIDEVIDERPLELVKDAVGNESKDKSS
S
>P25076
MSLGKKIRIGFDGFGRINRFITRGAAQRNDSKLPSRNDALKHGLDGLGSAGSKSFRALAA
IGAGVSGLLSFATIAYSDEAEHGLECPNYPWPHEGILSSYDHASIRRGHQVYQQVCASCH
SMSLISYRDLVGVAYTEEETKAMAAEIEVVDGPNDEGEMFTRPGKLSDRFPQPYANEAAA
RFANGGAYPPDLSLITKARHNGQNYVFALLTAYRDPPAGVSIREGLHYNPYFPGGAIAMP
KMLNDGAVEYEDGIPATEAQMGKDVVSFLSWAAEPEMEERKLMGFKWIFVLSLALLQAAY
YRRLRWSVLKSRKLVLDVVN
>Q9SJ12
MAYASRFLSRSKQLQGGLVILQQQHAIPVRAFAKEAARPTFKGDEMLKGVFFDIKNKFQA
AVDILRKEKITLDPEDPAAVKQYANVMKTIRQKADMFSESQRIKHDIDTETQDIPDARAY
LLKLQEIRTRRGLTDELGAEAMMFEALEKVEKDIKKPLLRSDKKGMDLLVAEFEKGNKKL
GIRKEDLPKYEENLELSMAKAQLDELKSDAVEAMESQKKKEEFQDEEMPDVKSLDIRNFI

>Q949J1
MSFYRGTASHPWHDLHPGNDAPNFVSCVIEIPRGSKVKYELDKDTGLCFVDRILYSSVVY
PHNYGFVPKTLCEDGDPLDVLVLMQEPVVPMCFLRAKPIGVMQMLDQGERDDKLIAVHAD
DPEYKGFTDISQLPPHRLAEIKRFFEDYKKNEHKEVVVDDFLGAEEAKKVVKDSLNMYQE
HYVPRKLRNVYE
>Q9FNC9
MAAKRIGAGKSGGGDPNILARISNSEIVSQGRRAAGDAVEVSKKLLRSTGKAAWIAGTTF
LILVVPLIIEMDREAQINEIELQQASLLGAPPSPMQRGL
>Q9SMN1
MATSLARISKRSITSAVSSNLIRRYFAAEAVAVATTETPKPKSQVTPSPDRVKWDYRGQR
QIIPLGQWLPKVAVDAYVAPNVVLAGQVTVWDGSSVWNGAVLRGDLNKITVGFCSNVQER
CVVHAAWSSPTGLPAQTLIDRYVTVGAYSLLRSCTIEPECIIGQHSILMEGSLVETRSIL
EAGSVLPPGRRIPSGELWGGNPARFIRTLTNEETLEIPKLAVAINHLSGDYFSEFLPYST
IYLEVEKFKKSLGIAI

>O99869
MSEFAPICIYLVISPLVSLIPLGLPFLFSSNSSTYPEKSSAYECGLDPSGDARSRVDIRF
YLVSILFIIPDPEVTFSFPWAVPPNKIDPFGSWSMMAFLLILTIGSLYEWKRGASDRE
>Q8LG77
MTMTAFLARRLIGNGSSQILGTSSSSSGPFISVSRAFFSSSTPIKATLFPGDGIGPEIAE
SVKQVFTAADVVIDWDEQFVGTEVDPRTNSFLTWDNLQSVLKNKVGLKGPMATPIGKGHR
SLNLTLRKELNLYANVRPCYSLPGYKTRYDDVDLITIRENTEGEYSGLEHQVVKGVVESL
KIITRKASMRVAEYAFLYAKTHGRKKVSAIHKANIMQKTDGLFLQCCDEVAAKYPEIYYE
KVVIDNCCMMLVKNPALFDVLVMPNLYGDIISDLCAGLVGGLGLTPSMNIGEDGIALAEA
VHGSAPDIAGMNLANPTALLLSGVMMLRHLKLNKQAEQIHSAIINTIAEGKYRTADLGGS
STTTDFTKAICDHL
>Q9ASY8
MESTISLKVNKGKGKGSKGASSSDDKSKFDVVKEWTNWSLKKAKVVTHYGFIPLVIFVGM
NSDPKPHLFQLLSPV
>Q96252
MFKQASRLLSRSVAAASSKSVTTRAFSTELPSTLDSTFVEAWKKVAPNMDPPQTPSAFMK
PRPSTPSSIPTKLTVNFVLPYTSELTGKEVDMVIIPASTGQMGVLPGHVPTIAELKPGIM
SVHEGTDVKKYFLSSGFAFLHANSVADIIAVEAVPLDHIDPSQVQKGLAEFQQKLASATT
DLEKAEAQIGVEVHSAINAALSG
>Q02500
MRLLAPAFKFHFKGGRRTMILSVLSSPALVSGLMVVRAKNPVHSVLFPILVFCDTSGLLI
LLGLDFSAMISPVVHIGAIAVSFLFVVMMFNIQIAEIHEEVLRYLPVSGIIGLIFWWEMF
FILDNETIPLLPTHRNTTSLRYTVYAGKVRSWTNLETLGNLLYTYYSVWFLVSSLILLVA
MIGAIVLTMHRTTKVKRQDVFRRNALDSRSHIMNRTISPFGHSHRRSFSSGAGGPPDNYK
ETFKMWI
>P08740
MIELDLCFGLLLLILFGLLSLRNGHVSLAHIRFICQCWLVMITPLEVQDFALCILTVVLL
QSFHSFEALLFLLLAYIGQLYMMHSCNLVSFYVCLEAQTLCVVVLCGLLARGASTSFSVE
AALKFLLLSAMVSGMALFWFSAMYQRTGSLDMVGQETFWILLVMLFKLGVAPMHMWSVDL
YGSIPKSLLLYLSTAPKLSLFTFWASSWHHDFSVGVFILFSMFIGSIGAYGQPALRSLFA
YSTINEIGLLLLAVETAGFHTLYQHLGIYIITQLLLWNLTDKRLFALCAVSLAGLPPFAG
FFGKAWIFWHAMSVQAFSLLAAALFCTLLSLVYYLRVIRLFWTAPVHTAASFTGAPNQTT
LTSACAVALAFAPVMLVKPFVI
>P52902
MALSRLSSSSSSSNGSNLFNPFSAAFTLNRPISSDTTATLTIETSLPFTAHNCDPPSRSV
TTSPSELLSFFRTMALMRRMEIAADSLYKANLIRGFCHLYDGQEAVAVGMEAGTTKKDCI
ITAYRDHCTFLGRGGTLLRVYAELMGRRDGCSKGKGGSMHFYKKDSGFYGGHGIVGAQVP
LGCGLAFGQKYLKDESVTFALYGDGAANQGQLFEALNISALWDLPAILVCENNHYGMGTA
TWRSAKSPAYFKRGDYVPGLKVDGMDALAVKQACKFAKEHALKNGPIILEMDTYRYHGHS
MSDPGSTYRTRDEISGVRQERDPIERVRKLLLSHDIATEKELKDTEKEVRKEVDEAIAKA
KDSPMPDPSDLFSNVYVKGYGVEAFGVDRKEVRVTLP
>Q42577
MAMITRNTATRLPLLLQSQRAVAAASVSHLHTSLPALSPSTSPTSYTRPGPPSTSPPPPG
LSKAAEFVISKVDDLMNWARTGSIWPMTFGLACCAVEMMHTGAARYDLDRFGIIFRPSPR

QSDCMIVAGTLTNKMAPALRKVYDQMPEPRWVISMGSCANGGGYYHYSYSVVRGCDRIVP
VDIYVPGCPPTAEALLYGLLQLQKKINRRKDFLHWWNK
>Q9CAF6
MTPVLCHSTASIPNPNSLMSLSSTLRLSSSLLRRSFFRFPLTDPLCRLRRTEPSATRFFS
SRTPRSGKFVVGAGKRGDEQVKEESGANNGGLVVSGDESRIVPFELHKEATESYMSYALS
VLLGRALPDVRDGLKPVHRRILFAMHELGMSSKKPYKKCARVVGEVLGKFHPHGDTAVYD
SLVRMAQSFSLRCPLIQGHGNFGSIDADPPAAMRYTECRLDPLAEAVLLSDLDQDTVDFV
ANFDNSQKEPAVLPARLPALLLNGASGIAVGMATNIPPHNLGELVDVLCALIHNPEATLQ
ELLEYMPAPDFPTGGIIMGNLGVLDAYRTGRGRVVVRGKAEVELLDPKTKRNAVIITEIP
YQTNKATLVQKIAELVENKTLEGISDIRDESDRNGMRVVIELKRGGDPALVLNNLYRHTA
LQSSFSCNMVGICDGEPKLMGLKELLQAFIDFRCSVVERRARFKLSHAQQRKHIIEGIVV
GLDNVDEVIELITKASSHSSATAALQSEYGLSEKQAEAILEITLRRLTALERKKFTDESS
SLTEQITKLEQLLSTRTNILKLIEQEAIELKDRFSSPRRSMLEDSDSGDLEDIDVIPNEE
MLMAVSEKGYVKRMKADTFNLQHRGTIGKSVGKLRVDDAMSDFLVCHAHDHVLFFSDRGI
VYSTRAYKIPECSRNAAGTPLVQILSMSEGERVTSIVPVSEFAEDRYLLMLTVNGCIKKV
SLKLFSGIRSTGIIAIQLNSGDELKWVRCCSSDDLVAMASQNGMVALSTCDGVRTLSRNT
KGVTAMRLKNEDKIASMDIIPASLRKDMEEKSEDASLVKQSTGPWLLFVCENGYGKRVPL
SSFRRSRLNRVGLSGYKFAEDDRLAAVFVVGYSLAEDGESDEQVVLVSQSGTVNRIKVRD
ISIQSRRARGVILMRLDHAGKIQSASLISAADEEETEGTLSNEAVEAVSL
>Q04613
MRLSITNMDGRKMLFAAILSICALSSKKILIYNEEMIVALCFIGFIIFSRKSLGTTFKVT
LDGSLQAIQEELQQFPNPNEVVLLESNEQQRLLRISLRICGTVVESLPMARCAPKCEKTV
QALLCRNLNVKLATLTNAISSRRIRFQDDLVTKFYTLVGKQFAYSCISKAERVEFIRESL
VVLRMVRGGVFS
>P83291
MATSFFRRLARSAPITFPVAFGSQSKSGSGAFRFSTGAIAALSGGFSYYYLTSGNNLVYL
DQAKEETGPKTALNPDKWLEFKLQDTARVSHNTQLFRFSFDPSAELGLHVASCLLTRAPL
GYNAEGKTKYVIRPYTPISDPEAKGYFDLLIKVYPDGKMSQHFASLKPGDVLEVKGPVEK
FKYSPNMKKHIGMIAGGSGITPMLQVIDAIVKNPEDNTQISLLYANVSPDDILLKQKLDV
LQANHPNLKIFYTVDNPTKNWKGGVGYISKDMALKGLPLPTDDTLILVCGPPGMMEHISG
GKAPDWSQGEVKGILKELGYTEEMVFKF
>P07513
MPFLVGLSPPFLYFELIGHFQVEPSPTPTIKGRKWWRLSLFLIFWGERRVKKETKANDC
>O48593
MAATFLPATSLRLTQNSTLRFLSFFTISNPSYSLFRPLRRRVLPPFDAFPANSRRRCFCT
AVSESLGSGDGNKVESYEKRFGSKVGEFRKKLRIAEVKGGADEGLSRVGQSLNIMGWVRT
LRSQSSVTFIEINDGSCLSNLQCVMTSDAEGYDQVESGSILTGASVSVQGTIVASQGTKQ
KVELKVEKIIVVGECDSSYPIQKKRVSREFLRTKAHLRPRTNTFGAVARVRNTLAYATHK
FFQESGFVWVASPIITASDCEGAGEQFCVTTLIPSSHENTDTSIDAIPKTKGGLIDWSQD
FFGKPAFLTVSGQLNGETYATALSDVYTFGPTFRAENSNTSRHLAEFWMIEPELAFADLD
DDMACATAYLQYVVKYVLDNCKEDMEFFDTWIEKGIIRRLSDVAEKEFLQLGYTDAIEIL
LKANKKFDFPVKWGLDLQSEHERYITEEAFGGRPVIIRDYPKEIKAFYMRENDDGKTVAA
MDMLVPRIGELIGGSQREERLEVLEARLDELKLNKESYWWYLDLRRYGSVPHAGFGLGFE
RLVQFVTGIDNIRDVIPFPRTPASAEF

>P26969
MERARRLANRATLKRLLSEAKQNRKTESTSTTTTTPLPFSLSGSSSRYVSSVSNSILRGR
GSKPDNNVSRRVGGFLGVGYPSQSRSISVEALKPSDTFPRRHNSATPDEQTKMAESVGFD
TLDSLVDATVPKSIRLKEMKFNKFDGGLTEGQMIEHMKDLASKNKVFKSFIGMGYYNTHV
PPVILRNIMENPAWYTQYTPYQAEISQGRLESLLNFQTMITDLTGLPMSNASLLDEGTAA
AEAMSMCNNIQKGKKKTFIIASNCHPQTIDICQTRADGFELKVVVKDLKDIDYKSGDVCG
VLVQYPGTEGEVLDYGEFIKKAHANEVKVVMASDLLALTVLKPPGEFGADIVVGSAQRFG
VPMGYGGPHAAFLATSQEYKRMMPGRIIGVSVDSSGKQALRMAMQTREQHIRRDKATSNI
CTAQALLANMAAMYAVYHGPEGLKAIAQRVHGLAGVFALGLKKLGLEVQDLGFFDTVKVK
TSNAKAIADAAIKSEINLRVVDGNTITAAFDETTTLEDVDKLFKVFAGGKPVSFTAASLA
PEFQNAIPSGLVRESPYLTHPIFNTYQTEHELLRYIHRLQSKDLSLCHSMIPLGSCTMKL
NATTEMMPVTWPSFTDLHPFAPTEQAQGYQEMFNNLGDLLCTITGFDSFSLQPNAGAAGE
YAGLMVIRAYHLSRGDHHRNVCIIPASAHGTNPASAAMVGMKIVTIGTDAKGNINIEELK
KAAEKHKDNLSAFMVTYPSTHGVYEEGIDDICKIIHDNGGQVYMDGANMNAQVGLTSPGW
IGADVCHLNLHKTFCIPHGGGGPGMGPIGVKKHLAPFLPSHPVVPTGGIPAPENPQPLGS
ISAAPWGSALILPISYTYIAMMGSQGLTDASKIAILNANYMAKRLESYYPVLFRGVNGTV
AHEFIIDLRGFKNTAGIEPEDVAKRLMDYGFHGPTMSWPVAGTLMIEPTESESKAELDRF
CDALISIRKEIAEVEKGNADVHNNVLKGAPHPPSLLMADAWTKPYSREYAAFPAAWLRGA
KFWPTTGRVDNVYGDRNLVCTLLPASQAVEEQAAATA
>Q8LB02
MAFGLIGRVVGTKSSRLSTAARLIPARWTSTGSEAQSKASTGGGGASLKTFQIYRWNPDN
PGKPELQDYKIDLKDCGPMVLDALIKIKNEMDPSLTFRRSCREGICGSCAMNIDGCNGLA
CLTKIESGSKETTITPLPHMFVIKDLVVDMTNFYNQYKSIEPWLKRKNPASVPGKEILQS
KKDRAKLDGMYECILCACCSTSCPSYWWNPESYLGPAALLHANRWISDSRDEYTKERLEA
IDDEFKLYRCHTILNCARACPKGLNPGKQITHIKQLQKSG
>O24495
MPVISKASSTTTNSSIPSCSRIGGQLCVWPGLRQLCLRKSLLYGVMWLLSMPLKTLRGAR
KTLKITHFCSISNMPSSLKIELVPCSKDNYAYLLHDEDTGTVGVVDPSEAAPVIEALSRK
NWNLTYILNTHHHDDHIGGNAELKERYGAKVIGSAVDKDRIPGIDILLKDSDKWMFAGHE
VRILDTPGHTQGHISFYFPGSATIFTGDLIYSLSCGTLSEGTPEQMLSSLQKIVSLPDDT
NIYCGRENTAGNLKFALSVEPKNETLQSYATRVAHLRSQGLPSIPTTVKVEKACNPFLRI
SSKDIRKSLSIPDSATEAEALRRIQRARDRF
>Q05492
MFPLNFHYEDVLRQDLLLKLNLANVMEVPGLCEIRIVPKTSSTYDFIIKNGKLAMEILRG
QKFIQTERGSTGKSFRSNPFLESNKDKGYVSDLARQSILRGHGMSNFLVRILTVMSLLDS
RVEIRENSIQFSMETEFCEFSPELEDHFEIFEHIRRFNVTIVTSANTQDETLLLWSGFLQ
KDEGESFKWKTP
>Q42290
MAMKNLLSLARRSQRRLFLTQATRSSSSFSAIDSVPASASPTALSPPPPHLMPYDHAAEI
IKNKIKKLENPDKRFLKYASPHPILASHNHILSAPETRVTTLPNGLRVATESNLSAKTAT
VGVWIDAGSRFESDETNGTAHFLEHMIFKGTDRRTVRALEEEIEDIGGHLNAYTSREQTT
YYAKVLDSNVNQALDVLADILQNSKFEEQRINRERDVILREMQEVEGQTDEVVLDHLHAT
AFQYTPLGRTILGPAQNVKSITREDLQNYIKTHYTASRMVIAAAGAVKHEEVVEQVKKLF
TKLSSDPTTTSQLVANEPASFTGSEVRMIDDDLPLAQFAVAFEGASWTDPDSVALMVMQT

MLGSWNKNVGGGKHVGSDLTQRVAINEIAESIMAFNTNYKDTGLFGVYAVAKADCLDDLS
YAIMYEVTKLAYRVSDADVTRARNQLKSSLLLHMDGTSPIAEDIGRQLLTYGRRIPTAEL
FARIDAVDASTVKRVANKYIYDKDIAISAIGPIQDLPDYNKFRRRTYWNRY
>Q0WQF7
MVLPLFRRAAIARTSSLLRARLFAPASEFHSRFSNGLYHLDDKISSSNGVRSASIDLITR
MDDSSPKPILRFGVQNFSSTGPISQTVLAMPALSPTMSHGNVVKWMKKEGDKVEVGDVLC
EIETDKATVEFESQEEGFLAKILVTEGSKDIPVNEPIAIMVEEEDDIKNVPATIEGGRDG
KEETSAHQVMKPDESTQQKSSIQPDASDLPPHVVLEMPALSPTMNQGNIAKWWKKEGDKI
EVGDVIGEIETDKATLEFESLEEGYLAKILIPEGSKDVAVGKPIALIVEDAESIEAIKSS
SAGSSEVDTVKEVPDSVVDKPTERKAGFTKISPAAKLLILEHGLEASSIEASGPYGTLLK
SDVVAAIASGKASKSSASTKKKQPSKETPSKSSSTSKPSVTQSDNNYEDFPNSQIRKIIA
KRLLESKQKIPHLYLQSDVVLDPLLAFRKELQENHGVKVSVNDIVIKAVAVALRNVRQAN
AFWDAEKGDIVMCDSVDISIAVATEKGLMTPIIKNADQKSISAISLEVKELAQKARSGKL
APHEFQGGTFSISNLGMYPVDNFCAIINPPQAGILAVGRGNKVVEPVIGLDGIEKPSVVT
KMNVTLSADHRIFDGQVGASFMSELRSNFEDVRRLLL
>P19173
MAGGHVAHLVYKGPSVVKELVIGFSLGLVAGGFWKMHHWNSQRRTKEFYDMLEKGQISVV
ADEE
>Q9LKA5
MATHTISRSILCRPAKSLSFLFTRSFASSAPLAKSPASSLLSRSRPLVAAFSSVFRGGLV
SVKGLSTQATSSSLNDPNPNWSNRPPKETILLDGCDFEHWLVVVEPPQGEPTRDEIIDSY
IKTLAQIVGSEDEARMKIYSVSTRCYYAFGALVSEDLSHKLKELSNVRWVLPDSYLDVRN
KDYGGEPFIDGKAVPYDPKYHEEWIRNNARANERNRRNDRPRNNDRSRNFERRRENMAGG
PPPQRPPMGGPPPPPHIGGSAPPPPHMGGSAPPPPHMGQNYGPPPPNNMGGPRHPPPYGA
PPQNNMGGPRPPQNYGGTPPPNYGGAPPANNMGGAPPPNYGGGPPPQYGAVPPPQYGGAP
PQNNNYQQQGSGMQQPQYQNNYPPNRDGSGNPYQG
>P92983
MATRLLRTNFIRRSYRLPAFSPVGPPTVTASTAVVPEILSFGQQAPEPPLHHPKPTEQSH
DGLDLSDQARLFSSIPTSDLLRSTAVLHAAAIGPMVDLGTWVMSSKLMDASVTRGMVLGL
VKSTFYDHFCAGEDADAAAERVRSVYEATGLKGMLVYGVEHADDAVSCDDNMQQFIRTIE
AAKSLPTSHFSSVVVKITAICPISLLKRVSDLLRWEYKSPNFKLSWKLKSFPVFSESSPL
YHTNSEPEPLTAEEERELEAAHGRIQEICRKCQESNVPLLIDAEDTILQPAIDYMAYSSA
IMFNADKDRPIVYNTIQAYLRDAGERLHLAVQNAEKENVPMGFKLVRGAYMSSEASLADS
LGCKSPVHDTIQDTHSCYNDCMTFLMEKASNGSGFGVVLATHNADSGRLASRKASDLGID
KQNGKIEFAQLYGMSDALSFGLKRAGFNVSKYMPFGPVATAIPYLLRRAYENRGMMATGA
HDRQLMRMELKRRLIAGIA
>P15688
MLSLTIKGKARRRKERAFGDRDFLTFSSKTKKTENVNLSFEKGTRFFDRGGMIFGPSPRS
ARWPIGIAAFGLCLLFLIKNSGSARESAGNNRKEGVHVAAASAPFLVNRAAGSATTTKER
IHFKITNASAMAACGMAGSDLFGYIIQVESGVTGTAGLMENNFHGSVQRALFSLRILRSL
RVNSLARIQNFWGPSIPSSSPAKTPLPFGLNIFFDSYMWAPDIYEGSPTPVTAFFSIAPE
RSISANILRVFIYGSYGATLQQIFFFCSIALRLRSTGAMANEGKASSSIGQLDYGGLYFV
LVLMWNREGIQSLLIGLFIYASMDDRCFAIVSALRQTRVKYIADLGALAKTNPISAITFS
ITMFSYAGIPPLAGFCSKFYLFFAALGCGAYFLAPVGVVTSVIGCWAAGRLPRVSQFGDR

RQFSVHRTRSLPNQLRHGWECMLRKIGSSLIHQPSVYSISLYESTITTRDEPWFGEFELA
LGVIGLPVTAHDRILRCSPPVVGTTRAGPGLNSER
>Q9LIG6
MKGIARLVTSLSRIGGRKVVSGTSTVTSSSSSSLLLSRRSLFISATNLLNSRTKDSALPS
LNSSLLAQKWNFLGGQRRTMFIQTQSTPNPSSLMFYPGKPVMEVGSADFPNVRSALGSPL
AKSIYSIDGVVRVFFGSDFVTVTKSDDVSWDILKPEIFAAVMDFYSSGQPLFLDSQAAAA
KDTAISEDDSETVAMIKELLETRIRPAVQDDGGDIEYCGFDPESGIVKLRMQGACSGCPS
SSVTLKSGIENMLMHYVSEVKGVEQEFDGEDEEGTLSGEMRVE
>Q9LSW8
MKILVAVKRVVDYAVKIRVKPDKTGVETQNVKMSMNPFCEIALEEALRIKEAGFAKEVIA
VSIGPSQCVDTLRTGLAMGADRGIHVETNSIFLPLTIAKILKSLADVENPGLIFLGKQAI
DDDCNQTGQMVAALLGWPQATFASKVVLDKDKNVATVDREVDGGLETLNVDLPAVITTDL
RLNQPRYASLPNIMKAKSKPIKKMTVQDLKVDIKSDIEILEVTEPPKRKSGVMVSSVDEL
IDKLKNEAHVV
>Q9FV50
MAGVKSLQPRLISSFLGNNSIRSTQPLIHLFRFDLGRRHVSMQLSRTFSGLTDLLFNRRN
EDEVIDGKRKRLRPGNVSPRRPVPGHITKPPYVDSLQAPGISSGLEVHDKKGIECMRASG
ILAARVRDYAGTLVKPGVTTDEIDEAVHNMIIENGAYPSPLGYGGFPKSVCTSVNECICH
GIPDSRPLEDGDIINIDVTVYLNGYHGDTSATFFCGNVDEKAKKLVEVTKESLDKAISIC
GPGVEYKKIGKVIHDLADKHKYGVVRQFVGHGVGSVFHADPVVLHFRNNEAGRMVLNQTF
TIEPMLTIGSRNPIMWDDNWTVVTEDASLSAQFEHTILITKDGAEILTKC
>Q39218
MLRSSSPHINHHSFLLPSFVSSKFKHHTLSPPPSPPPPPPMAACIDTCRTGKPQISPRDS
SKHHDDESGFRYMNYFRYPDRSSFNGTQTKTLHTRPLLEDLDRDAEVDDVWAKIREEAKS
DIAKEPIVSAYYHASIVSQRSLEAALANTLSVKLSNLNLPSNTLFDLFSGVLQGNPDIVE
SVKLDLLAVKERDPACISYVHCFLHFKGFLACQAHRIAHELWTQDRKILALLIQNRVSEA
FAVDFHPGAKIGTGILLDHATAIVIGETAVVGNNVSILHNVTLGGTGKQCGDRHPKIGDG
VLIGAGTCILGNITIGEGAKIGAGSVVLKDVPPRTTAVGNPARLLGGKDNPKTHDKIPGL
TMDQTSHISEWSDYVI
>P46643
MALAMMIRNAASKRGMTPISGHFGGLRSMSSWWKSVEPAPKDPILGVTEAFLADPSPEKV
NVGVGAYRDDNGKPVVLECVREAEKRLAGSTFMEYLPMGGSAKMVDLTLKLAYGDNSEFI
KDKRIAAVQTLSGTGACRLFADFQKRFSPGSQIYIPVPTWSNHHNIWKDAQVPQKTYHYY
HPETKGLDFSALMDDVKNAPEGSFFLLHACAHNPTGVDPTEEQWREISQLFKAKKHFAFF
DMAYQGFASGDPARDAKSIRIFLEDGHHIGISQSYAKNMGLYGQRVGCLSVLCEDPKQAV
AVKSQLQQLARPMYSNPPLHGAQLVSTILEDPELKSLWLKEVKVMADRIIGMRTTLRESL
EKLGSPLSWEHVTKQIGMFCYSGLTPEQVDRLTSEYHIYMTRNGRISMAGVTTGNVGYLA
NAIHEVTKSS
>Q9C6I6
MTRTVLLRALTKNKFVASNAPRSISISITSLSRCISTLILAEHESGTIKPQTVSTVVAAN
SLGESSSISLLLAGSGSSLQEAASQAASCHPSVSEVLVADSDKFEYSLAEPWAKLVDFVR
QQGDYSHILASSSSFGKNILPRVAALLDVSPITDVVKILGSDQFIRPIYAGNALCTVRYT
GAGPCMLTIRSTSFPVTPITANSESKKATVSQIDLSNFEDDSVSKSRYVGRSTQDTERPD
LGSARVVITGGRALKSVENFKMIEKLAEKLGGAVGATRAAVDAGYVPNDLQVGQTGKIVA

PELYMAFGVSGAIQHLAGIKDSKVIVAVNKDADAPIFQVADYGLVGDLFEVIPELLEKLP
EKK
>Q37617
MNRNEISFIYYVKIKLYLYNSFFIMSYIEMVLAIPLLGAIALLFVPSWKTQTIRNIALNS
SLLTFLISLLLWIEFDSSSALFQFTDGVCSPNVYSDVTLAKAASSSSFSALNFALGVDGI
SLFFIILTTLLVPICILVSWNNIEVYVKEYCIAFLVLETLMLTVFSVLDLLLFYIFFESV
LIPMFIIIGVWGSRERKIRAAYQFFLYTLFGSVLMLLAILLIYFQTGTLDIEMLYLSDFS
ETRQCILWLAFFASFAVKVPMVPVHIWLPEAHVEAPTAGSVILAGILLKLGTYGFLRFSI
PLFPYACIYFTPLIYTMSVIAIVYTSCTTIRQIDLKKIIAYSSVAHMNFVTIGLFSQNTQ
GIEGSILLMISHGLVSPALFLCVGVLYDRHKTRLLRYYSGCGQTMPIFALLFVFFTMANI
SLPGTSSFPGEFLVFIGSYQNNSFVAFCAATGMVLGAAYALWLCNRLIYGVSKPDFINTW
SDVNRREFFMFAPLIAGILWIGVYPEPFLDAMHCSCIYLLYAQ
>O49543
MASKVISATIRRTLTKPHGTFSRCRYLSTAAAATEVNYEDESIMMKGVRISGRPLYLDMQ
ATTPIDPRVFDAMNASQIHEYGNPHSRTHLYGWEAENAVENARNQVAKLIEASPKEIVFV
SGATEANNMAVKGVMHFYKDTKKHVITTQTEHKCVLDSCRHLQQEGFEVTYLPVKTDGLV
DLEMLREAIRPDTGLVSIMAVNNEIGVVQPMEEIGMICKEHNVPFHTDAAQAIGKIPVDV
KKWNVALMSMSAHKIYGPKGVGALYVRRRPRIRLEPLMNGGGQERGLRSGTGATQQIVGF
GAACELAMKEMEYDEKWIKGLQERLLNGVREKLDGVVVNGSMDSRYVGNLNLSFAYVEGE
SLLMGLKEVAVSSGSACTSASLEPSYVLRALGVDEDMAHTSIRFGIGRFTTKEEIDKAVE
LTVKQVEKLREMSPLYEMVKEGIDIKNIQWSQH
>P68209
MSRQVARLIGSLSSKARRCSTGGSEVFPSCQSLTSLTQSRSFASDPHPPAAVFVDKNTRV
LCQGITGKNGTFHTEQAIEYGTKMVAGVTPKKGGTEHLGLPVFNSVAEAKADTKANASVI
YVPAPFAAAAIMEGIEAELDLIVCITEGIPQHDMVRVKHALNSQSKTRLIGPNCPGIIKP
GECKIGIMPGYIHKPGKIGIVSRSGTLTYEAVFQTTAVGLGQSTCVGIGGDPFNGTNFVD
CLEKFFVDPQTEGIVLIGEIGGTAEEDAAALIKASGTEKPVVAFIAGLTAPPGRRMGHAG
AIVSGGKGTAQDKIKSLNDAGVKVVESPAKIGSAMYELFQERGLLKQ
>O23627
MRIFSTFVFHRRQQIFNLRQFQTTTILRNPISIAPIQIPMDATEQSLRQSLSEKSSSVEA
QGNAVRALKASRAAKPEIDAAIEQLNKLKLEKSTVEKELQSIISSSGNGSLNREAFRKAV
VNTLERRLFYIPSFKIYSGVAGLFDYGPPGCAIKSNVLSFWRQHFILEENMLEVDCPCVT
PEVVLKASGHVDKFTDLMVKDEKTGTCYRADHLLKDYCTEKLEKDLTISAEKAAELKDVL
AVMEDFSPEQLGAKIREYGITAPDTKNPLSDPYPFNLMFQTSIGPSGLIPGYMRPETAQG
IFVNFKDLYYYNGKKLPFAAAQIGQAFRNEISPRQGLLRVREFTLAEIEHFVDPENKSHP
KFSDVAKLEFLMFPREEQMSGQSAKKLCLGEAVAKGTVNNETLGYFIGRVYLFLTRLGID
KERLRFRQHLANEMAHYAADCWDAEIESSYGWIECVGIADRSAYDLRAHSDKSGTPLVAE
EKFAEPKEVEKLVITPVKKELGLAFKGNQKNVVESLEAMNEEEAMEMKATLESKGEVEFY
VCTLKKSVNIKKNMVSISKEKKKEHQRVFTPSVIEPSFGIGRIIYCLYEHCFSTRPSKAG
DEQLNLFRFPPLVAPIKCTVFPLVQNQQFEEVAKVISKELASVGISHKIDITGTSIGKRY
ARTDELGVPFAITVDSDTSVTIRERDSKDQVRVTLKEAASVVSSVSEGKMTWQDVWATFP
HHSSAAADE
>P38482
MLSSVRLAALRAGKTNSVFQAVRAFAAEPAAAATTDAGFVSQVIGPVVDVRFDGELPSIL

SALEVQGHNVRLVLEVAQHMGDNTVRCVAMDSTDGLVRGQKVVNTGSPIKVPVGRGTLGR
IMNVIGEPVDEQGPIECSEVWSIHREAPEFTEQSTEQEILVTGIKVVDLLAPYQRGGKIG
LFGGAGVGKTVLIMELINNVAKAHGGFSVFAGVGERTREGNDLYREMIESGVIKLGDKRG
ESKCTLVYGQMNEPPGARARVALTGLTVAEYFRDVEGQDVLLFVDNIFRFTQANSEVSAL
LGRIPSAVGYQPTLATDLGGLQERITTTTKGSITSVQAVYVPADDLTDPAPATTFAHLDA
TTVLSRSIAELGIYPAVDPLDSTSRMLNPNIIGAEHYNIARGVQKVLQDYKNLQDIIAIL
GMDELSEEDKLTVARARKIQRFLSQPFQVAEVFTGTPGKYVDLKDTISAFTGILQGKYDD
LPEMAFYMVGGIHEVVEKADKLAKDVAARKDESKKAKSSEALKDVPSLEKMAGEIKDEVI
DADDSLEEDFKAEAISSENMVLNEKGEKVPLPKK

>Q37619

MALEKIITAPKYKNFTINFGPQHPAAHGVLRLVLEMNGEVVQRSDPHIGLLHRGTEKLIE
YKNYLQALPYFDRLDYVSMMCQEHAYSLAVEKLLNISKDIPLRAQYIRVLFSEITRILNH
LLAVTCHAMDVGALTPFLWGFEEREKLMEFYERVSGARMHAAYIRPGGVALDLPLGLCED
IYKFSKQFASRIDEIEEMLTSNRIWKQRLVDVGVVSAEQALDWSFSGVLLRGSGIAWDLR
KTQPYEVYDRMKFNIPVGTRGDCYDRYLIRVQEMRESLRIVMQTINEMSKGIIRLDDRKI
TPPTRDQMKQSMESLIHHFKFYTGGFVVPAGETYTAVEAPKGEFGVYLVSNGTSKPYRCK
IRAPGFAHLQGLDFMARNHMLADVVTIIGTQDIVFGEVDR

>Q8W487

MALAWCVVRRSASKFASVYGGRVRSISAVANRASLARNPSSIRPFVSRALNYSTAIDRIS
SEQTLIRVIDSEINSALQSDNIDSDEEMTPGSFPFRIEDKPGNQNVTLTRDYNGEHIKVV
VSMPSLVSDENDDDDDDDEGPSNESSIPLVVTVTKKSGLTLEFSCMAFPDEIAIDALSVK
HPGDSLEDQLANEGPDFEDLDENLKKTFYKFLEIRGVKASTTNFLHEYMTRKVNREYFLW
LKNVKEFMEQ

>Q9SX77

MGTLGRAIHTVGNRIRGTAQAQARVGSLLQGSHHIEKHLSRHRTLITVAPNASVIGDVQI
NKGSFISYASVSRDLQYPRAMGMGQVRRFSEDVSHMPEMEDSDVLNAFKDLMAADWAELP
SAVVKDAKTAISKNTDDKAGQEALKNVFRAAEAVEEFGGILTSIKMEIDDSIGMSGEGVK
PLPNDITDALRTAYQRYAEYLDSFEPEEVYLKKKVEMELGTKMIHLKMRCSGLGSEWGKV
TVLGTSGLSGSYVEQRA

>P32746

MAGRAATSSAKWAREFLFRRVSSNPLGATRNCSSVPGASSAPKVPHFSKRGRILTGATIG
LAIAGGAYVSTADEATFCGWLFNATKVVNPFFALLDAEFAHKLAVSAAARGWVPREKRPD
PAILGLEVWGRKFSNPIGLAAGFDKNAEATEGLLGMGFGFVEVGSVTPVPQEGNPKPRIF
RLSQEGAIINRCGFNSEGIVVVAKRLGAQHGKRMLAETSATSSSPSDDVKPGGKSGPGIL
GVNLGKNKTSEDAAADYVQGVHNLSQYADYLVINVSSPNTAGLRMLQGRKQLKDLVKKVQ
AARDEMQWGDEGPPPLLVKIAPDLSRGELEDIAAVALALHLDGLIISNTTVSRPDAVSNN
PVATETGGLSGKPLFALSTNMLRDMYTLTRGKIPLIGCGGVSSGEDAYKKIRAGATLVQL
YTGFAYGGPALIPQIKEELVKCLERDGFKSIHEAIGADHR

>P05499

MFRRIFLFDEDSLNSSVTSYTNASQSTTTIMDYSLKSSDTQGSSSGIFTDHPGLNPCSER
IVELQYDIRLKLGALMPKESAQKVLEASEALHGESNNIAFLEYLLEDLQQNGVGGEAYKD
AVDLSKDLVSSPLEQFEIISLIPMKIGNLYFSFTNPSLFMLLTLSLVLLLVYFVTKKGGG
NSVPNAWQSLVELIYDFVLNPVNEQIGGLSGNVKQKFSPRISVTFTFSLFCNPQGMIPYS
FTVTSHFLITLGLSFSIFIGITIVGFQKNGLHFLSFLLPAGVPLPLAPFLVLLELIPYCF

RALSSGIRLFANMMAGHSSVKILSGFAWTMLCMNDLLYFIGDLGPLFIVLALTGLELGVA
ISQAHVSTILICIYLNDAINLHQSASFFIIEQKRV
>O82067
MLKPKGKNTKKAAAADEDDGAVAVVGKFVKEWGTWTAKKAKVITHYGFIPLVIIIGMNSE
PKPSLSQLLSPV
>Q9CA19
MLGLRRSATTLFDISQSLLRNVTFHGLRVQGIRVGNAEVPNNKPLKTGLQEVYGIGRRKS
HQVLCHLGITNKLARDLTGKELIDLREEVGQHQHGDELRRRVGSEIQRLVEVDCYRGSRH
RHGLPCRGQRTSTNARTKKGKAVAIAGKKKAPRK
>Q9XI00
MTSDALTIPSELESALRLRTVQYFITKRPWLDLYGVHVRPVPPFGSTSRKPHFDPALIHR
CLPDELLFEVFARMMPYDLGRASCVCRKWRYTVRNPMFWRNACLKAWQTAGVIENYKILQ
SKYDGSWRKMWLLRSRVRTDGLYVSRNTYIRAGIAEWKITNPVHIVCYYRYIRFYPSGRF
LYKNSSQKLKDVAKYMNFKASKSENLYKGTYTLSMSDDKIEAAVLYPGTRPTVLRIRLRL
RGTAIGANNRMDLLSLVTSGVNDEEISSTEEDILGLVEGWEDDETHNPDIPAVSHKRGMT
AFVFVPFEEVDESVLNLPPEKMDYYVTG
>Q9S7E4
MAMRQAAKATIRACSSSSSSGYFARRQFNASSGDSKKIVGVFYKANEYATKNPNFLGCVE
NALGIRDWLESQGHQYIVTDDKEGPDCELEKHIPDLHVLISTPFHPAYVTAERIKKAKNL
KLLLTAGIGSDHIDLQAAAAAGLTVAEVTGSNVVSVAEDELMRILILMRNFVPGYNQVVK
GEWNVAGIAYRAYDLEGKTIGTVGAGRIGKLLLQRLKPFGCNLLYHDRLQMAPELEKETG
AKFVEDLNEMLPKCDVIVINMPLTEKTRGMFNKELIGKLKKGVLIVNNARGAIMERQAVV
DAVESGHIGGYSGDVWDPQPAPKDHPWRYMPNQAMTPHTSGTTIDAQLRYAAGTKDMLER
YFKGEDFPTENYIVKDGELAPQYR
>P27927
MEKHLVMYLTRKSIMLLRKYLLVTEFQVSKCGSHIVKIRRDVLYPKRTKYSKYSKCRCSR
GREPDGTQLGFGRYGTKSSRAGRLSYRAIEAARRATIGQFRRAMSGQFRRNCKIWVRVLA
DLPITGKPAEVRMGRGKGNPTGWIARVSTGQIPFEMDGVSLSNARQAARLAAHKPCSSTK
FVQWS
>P05716
MSEKRNIRDHKRRLLAAKYELRRKLYKAFCKDSDLPSDMRDKLRYKLSKLPRNSSFARVR
NRCISTGRPRSVYELFRISRIVFRSLASRGPLMGIKKSSW
>Q8GXR9
MAVLSSVSSLIPFSYGATRLTSKASLASRTSGFNLSSRWNSTRNSPMLYLSRAVTNNSGT
TEISDNETAPRTYSWPDNKRPRVCILGGGFGGLYTALRLESLVWPEDKKPQVVLVDQSER
FVFKPMLYELLSGEVDVWEIAPRFSDLLTNTGIQFLRDRVKTLLPCDHLGVNGSEISVTG
GTVLLESGFKIEYDWLVLALGAESKLDVVPGAMELAFPFYTLEDAIRVNEKLSKLERKNF
KDGSAIKVAVVGCGYAGVELAATISERLQDRGIVQSINVSKNILTSAPDGNREAAMKVLT
SRKVQLLLGYLVQSIKRASNLEEDEGYFLELQPAERGLESQIIEADIVLWTVGAKPLLTK
LEPSGPNVLPLNARGQAETDETLRVKGHPRIFALGDSSSLRDSNGKILPTTAQVAFQEAD
FTGWNIWAAINNRPLLPFRFQNLGEMMTLGRYDAAISPSFIEGLTLEGPIGHAARKLAYL
IRLPTDEHRFKVGISWFAKSAVDSIALLQSNLTKVLSGS
>Q06450
MASNAAVPFWRAAGMTYITYSNLCANMVRNCLKEPYRAEALSREKVHFSFSKWVDGKPQK

PAIRSDTGEE
>P52424
MSLSACAELSRCFAAAASAKPNSGKSNSTAATSLVISSPIGHDGAVSSVSRSRKTSRIVA
EASQGLTYRDAGVDIDAGAELVRRIAKMAPGIGGFGGLYPLGDSYLVAGTDGVGTKLMLA
FETGIHDTIGIDLVAMSVNDIVTSGAKPLFFLDYFATGRLDVDVAEKVVKGIVDGCKQSD
CVLLGGETAEMPGLYKEGEYDLSGCAVGIVKKDSVINGKNIVAGDVIIGLPSSGVHSNGF
SLVRRVLAQSGLSLKDQLPGSNITLAEALMAPTVIYVKQVLDLISKGGVKGIAHITGGGF
TDNIPRVFPEGLGALIYDGSWEVPAVFRWLQEAGKIEDSEMRRTFNMGIGMILVVSPEAA
NRILENKGQADKFYRIGEIISGNGVTFS
>Q9GCB9
MTIHAAVIQKLLNTGAHLGRRAAEHHFKQYAYGTRNGMTIIDSDKTLICLRSAAHFVANL
AHMRGNIFFVNTNPLFDEIIELTSRRIQGDSYNHNRAMNLWKMGGFLTNSYSPKKFRSRH
KKLCFGPTTMPDCVVVFDSERKSSVILEASKLQIPVVAIVDPNVPLEFFEKITYPVPARD
SVKFVYLFCNVITKCFVAEQMKLGIKEGSNEDLMKDLAA
>O22043
MRPRYSLILSAMRLIRPSNRRLSSIASSDSEFISYMKNKAKSINKALDNSIPLCNNFVPL
WEPVLEVHKAMRYTLLPGGKRVRPMLCLVACELVGGQESTAMPAACAVEMIHAASLILDD
LPCMDDDSLRRGKPTNHKVFGEKTSILASNALRSLAVKQTLASTSLGVTSERVLRAVQEM
ARAVGTEGLVAGQAADLAGERMSFKNEDDELRYLELMHVHKTAVLVEAAAVVGAIMGGGS
DEEIERLKSYARCVGLMFQVMDDVLDETKSSEELGKTAGKDLITGKLTYPKVMGVDNARE
YAKRLNREAQEHLQGFDSDKVVPLLSLADYIVKRQN
>Q42523
MSMMTVWALRRNVRRKNHSMLVRYISGSASMKPKEQCIEKILVANRGEIACRIMRTAKRL
GIQTVAVYSDADRDSLHVKSADEAVRIGPPSARLSYLSGVTIMEAAARTGAQAIHPGYGF
LSESSDFAQLCEDSGLTFIGPPASAIRDMGDKSASKRIMGAAGVPLVPGYHGHEQDIDHM
KSEAEKIGYPIIIKPTHGGGGKGMRIVQSGKDFADSFLGAQREAAASFGVNTILLEKYIT
RPRHIEVQIFGDKHGNVLHLYERDCSVQRRHQKIIEEAPAPNISEKFRANLGQAAVSAAR
AVGYYNAGTVEFIVDTESDQFYFMEMNTRLQVEHPVTEMIVGQDLVEWQIRVANGEPLPL
SQSEVPMSGHAFEARIYAENVPKGFLPATGVLNHYRPVAVSPSVRVETGVEQGDTVSMHY
DPMIAKLVVWGGNRGEALVKLKDCLSNFQVAGVPTNINFLQKLASHKEFAVGNVETHFIE
HHKSDLFADESNPAATEVAYKAVKHSAALVAACISTIEHSTWNESNHGKVPSIWYSNPPF
RVHHEAKQTIELEWNNECEGTGSNLISLGVRYQPDGSYLIEEGNDSPSLELRVTRAGKCD
FRVEAAGLSMNVSLAAYLKDGYKHIHIWHGSEHHQFKQKVGIEFSEDEEGVQHRTSSETS
SHPPGTIVAPMAGLVVKVLVENEAKVDQGQPILVLEAMKMEHVVKAPSSGSIQDLKVKAG
QQVSDGSALFRIKG
>P82873
MEFSTADFERLIMFEHARKNSEAQYKNDPLDSENLLKWGGALLELSQFQPIPEAKLMLND
AISKLEEALTINPGKHQALWCIANAYTAHAFYVHDPEEAKEHFDKATEYFQRAENEDPGN
DTYRKSLDSSLKAPELHMQFMNQGMGQQILGGGGGGGGGMASSNVSQSSKKKKRNTEFT
YDVCGWIILACGIVAWVGMAKSLGPPPPAR
>Q8LGI2
MNPTQKPEPVYDMVILGASGFTGKYVVREALKFLQTPSSSPLKSLALAGRNPTRLTQSLE
WAARPNPPPSSVAILTADTSDPDSLRRLCTQTKLILNCVGPFRIHGDPVVSACADSGCDY
LDISGEPEFMERMEANYHDRAEETGSLIVSACGFDSIPAELGLLFNAKQWVSPSVPNQIE

AYLSLESDKKIAGNFGTYESAVLGVANAEKLKELRRSRPRRPRPTICGPPAKGPTLENQK
TIGLWALKLPSADAVVVRRTLTTLTEKPHGLPGINESPEQIQKREAFWSSIKPAHFGVKI
TSKSLFGIFRYVTLGVSLGLLSKFSFGRWLLLKFPSVFSLGWFQKKGPSEEEVESATFKM
WFIGRGYSEESLASQGETKPDLEIITRISGPEIGYITTPITLVQCGLIVLGQRESLVKGG
VYTPGIVFGSTDIQQRLEDNGISFELISKIKTQG
>P93655
MLKLFTSSASRVHHLTPVSRVVGSSPVESPLFKALSQITGWNRRSTSLGHRAFFCSEPTN
GEAAAEAETKAVESDSEVSDSKSSSAIVPTNPRPEDCLTVLALPVPHRPLFPGFYMPIYV
KDPKVLAALQESRRRQAPYAGAFLLKDDPSADSSSSTDAEKNINELKGKELLNRLHEVGT
LAQISSIQGDQVILVGHRRLRIKEMVSEEPLTVKVDHLKDNPFDMDDDVVKATSFEVIST
LRDVLKTSSLWRDHVQTYTQHIGDFTYPRLADFGAAICGANRHQAQEVLEELDVHKRLRL
TLELMKKEMEISKIQETIAKAIEEKISGEQRRYLLNEQLKAIKKELGVETDDKSALSAKF
KERIEPNKEKIPAHVLQVIEEELTKLQLLEASSSEFNVTRNYLDWLTILPWGNYSNENFD
VARAQTILDEDHYGLSDVKERILEFIAVGRLRGTSQGKIICLSGPPGVGKTSIGRSIARA
LNRKFFRFSVGGLADVAEIKGHRRTYVGAMPGKMVQCLKSVGTANPLVLIDEIDKLGRGH
AGDPASALLELLDPEQNANFLDHYLDVTIDLSKVLFVCTANVIDMIPNPLLDRMEVISIA
GYITDEKVHIARDYLEKTARGDCGVKPEQVEVSDAALLSLIENYCREAGVRNLQKQIEKI
YRKIALKLVREGAVPEEPAVASDPEEAEIVADVGESIENHTVEENTVSSAEEPKEEAQTE
KIAIETVMIDESNLADYVGKPVFHAEKLYEQTPVGVVMGLAWTSMGGSTLYIETTVVEEG
EGKGGLNITGQLGDVMKESAQIAHTVARKIMLEKEPENQFFANSKLHLHVPAGATPKDGP
SAGCTMITSLLSLATKKPVRKDLAMTGEVTLTGRILPIGGVKEKTIAARRSQIKTIIFPE
ANRRDFDELAENVKEGLNVHFVDDYGKIFELAFGYDKQED
>P10329
MFFENSAILLCALLSIAVGYTKSPFMSLMYSVMLFINSSFVLMMLGFEFLALVNLLVYVG
ALAVLFLFVIMLLEIPATELRAYSRGWSTLGIFVFIINGVFQITPSMGPRGIITGLPGAE
SITNLGHALYLYFADLLILNSLVLTVALFGRFAIAPVRTTGR
>P22778
MAMTGRARSMGFSILQKALSSAQRSNAHRSILCPTLSNSELLRNYATASASKEQKIKVPL
TMYGVSGNYASALYLAAVKSNTLEKVESELYDLVEASKKSPTFSQFMRDPSVPVDTRVNA
IKEICAQAKFGDTTQNFLLILAENGRLKHIDRIVKRFKELTMAHRGEVKATVTTVIPLPA
DEEKELKATLQEMVGQGKSVQIEQKIDPTILGGLVVEFGQKVFDMSIRTRARQMERFLRE
PLNF
>Q08276
MATAALLRSLRRREFATSSISAYRTLASNTKPSWCPSLVGAKWAGLARPFSSKPAGNEII
GIDLGTTNSCVAVMEGKNPKVIENSEGARTTPSVVAFNQKGELLVGTPAKRQAVTNPTNT
LSGTKRLIGRRFDDPQTQKEMKMVPYKIVRGSNGDAWVEANGQQYSPTQIGAFILTKMKE
TAEAYLGKSINKAVITVPAYFNDAQRQAIKDAGAIAGLDVQRIINEPTAAALSYGMNSKE
GLVAVFDLGGGTFDVSILEISNGVFEVKATNGDTFLGGEDFDNALLEFLVSEFKRTEGID
LSKDKLALQRLREAAEKAKIELSSTSQTDINLPFITADASGAKHLNITLTRSKFETLVNH
LIERTRNPCKNCLKDAGVSLKDVDEVLLVGGMTRVPKVQEIVSEIFGKSPSKGVNPDEAV
AMGAALQGGILRGDVKELLLLDVTPLARGIETLGGIFTRLINRNTTIPTKKSQVFSTAAD
NQTQVGIKVLQGEREMASDNKLLGEFDLVGIPPAPKGYCPQIEVIFDIDANGMVTVSAKD
KATSKEQQITIRSSGGLSEDEIDKMVREAEMHAQRIKNARHLLISGIVQSTTIYSIEKSL
SEYKEKVPKEVVTEIETAISDLRAAMGTENIDDIKAKLDAANKAVSKIGEHMAGGSSGGA

SGGGGAQGGDQPPEAEYEEVKK
>O23138
MASFDEAPPGNAKAGEKIFRTKCAQCHTVEAGAGHKQGPNLNGLFGRQSGTTAGYSYSAA
NKNKAVEWEEKALYDYLLNPKKYIPGTKMVFPGLKKPQDRADLIAYLKESTAPK
>P80269
MAAILARKSLSALRSRQLVLAGHTIEGTNGYNRTLLGTRSFATKHSFSTDKDDEEREQLA
KELSKDWNSVFERSINTLFLTEMVRGLMLTLKYFFEKKVTINYPFEKGPLSPRFRGEHAL
RRYATGEERCIACKLCEAICPAQAITIEAEEREDGSRRTTRYDIDMTKCIYCGFCQEACP
VDAIVEGPNFEFATETHEELLYDKEKLLENGDRWETEIAENLRSESLYR
>Q9FT52
MSGAGKKIADVAFKASRTIDWDGMAKVLVTDEARREFSNLRRAFDEVNTQLQTKFSQEPE
PIDWDYYRKGIGAGIVDKYKEAYDSIEIPKYVDKVTPEYKPKFDALLVELKEAEQKSLKE
SERLEKEIADVQEISKKLSTMTADEYFEKHPELKKKFDDEIRNDNWGY
>P05502
MTNLVRWLFSTNHKDIGTLYFIFGAIAGVMGTCFSVLIRMELARPGDQILGGNHQLYNVL
ITAHAFLMIFFMVMPAMIGGFGNWFVPILIGAPDMAFPRLNNISFWLLPPSLLLLLSSAL
VEVGSGTGWTVYPPLSGITSHSGGAVDLAIFSLHLSGVSSILGSINFITTIFNMRGPGMT
MHRLPLFVWSVLVTAFLLLLSLPVLAGAITMLLTDRNFNTTFFDPAGGGDPILYQHLFWF
FGHPEVYILILPGFGIISHIVSTFSRKPVFGYLGMVYAMISIGVLGFLVWAHHMFTVGLD
VDTRAYFTAATMIIAVPTGIKIFSWIATMWGGSIQYKTPMLFAVGFIFLFTIGGLTGIVL
ANSGLDIALHDTYYVVAHFHYVLSMGAVFALFAGFYYWVGKIFGRTYPETLGQIHFWITF
FGVNLTFFPMHFLGLSGMPRRIPDYPDAYAGWNALSSFGSYISVVGIRRFFVVVAITSSS
GKNKRCAESPWAVEQNPTTLEWLVQSPPAFHTFGELPTIKETQGELQTRK
>Q38799
MLGILRQRAIDGASTLRRTRFALVSARSYAAGAKEMTVRDALNSAIDEEMSADPKVFVMG
EEVGQYQGAYKITKGLLEKYGPERVYDTPITEAGFTGIGVGAAYAGLKPVVEFMTFNFSM
QAIDHIINSAAKSNYMSAGQINVPIVFRGPNGAAAGVGAQHSQCYAAWYASVPGLKVLAP
YSAEDARGLLKAAIRDPDPVVFLENELLYGESFPISEEALDSSFCLPIGKAKIEREGKDV
TIVTFSKMVGFALKAAEKLAEEGISAEVINLRSIRPLDRATINASVRKTSRLVTVEEGFP
QHGVCAEICASVVEESFSYLDAPVERIAGADVPMPYAANLERLALPQIEDIVRASKRACY
RSK
>P53665
MALRNAILRHLRVPVQTLGLNQSKIGFLGTIRSFSSHDDHLSREAVVDRVLDVVKSFPKV
DPSKVTPEVHFQNDLGLDSLDTVEIVMAIEEEFKLEIPDKEADKIDSCSLAIEYVYNHPM
SS
>Q8LCU7
MAALMESVVGRALKFSSTANFRSIRRGETPTLCIKSFSTIMSPPSKAIVYEEHGSPDSVT
RLVNLPPVEVKENDVCVKMIAAPINPSDINRIEGVYPVRPPVPAVGGYEGVGEVYAVGSN
VNGFSPGDWVIPSPPSSGTWQTYVVKEESVWHKIDKECPMEYAATITVNPLTALRMLEDF
VNLNSGDSVVQNGATSIVGQCVIQLARLRGISTINLIRDRAGSDEAREQLKALGADEVFS
ESQLNVKNVKSLLGNLPEPALGFNCVGGNAASLVLKYLREGGTMVTYGGMSKKPITVSTT
SFIFKDLALRGFWLQSWLSMGKVKECREMIDYLLGLARDGKLKYETELVPFEEFPVALDK
ALGKLGRQPKQVITF
>Q9XH48

MDSYSSPPMGGSGSSVSPEVMMESVKTQLAQAYAEELIETLRTKCFDKCVTKPGSSLGGS
ESSCISRCVERYMEATAIISRSLFTQR
>P04709
MADQANQPTVLHKLGGQFHLRSIISEGVRARNICPSVSSYERRFATRNYMTQSLWGPSMS
VSGGINVPVMQTPLCANAPAEKGGKNFMIDFMMGGVSAAVSKTAAAPIERVKLLIQNQDE
MIKSGRLSEPYKGIVDCFKRTIKDEGFSSLWRGNTANVIRYFPTQALNFAFKDYFKRLFN
FKKDRDGYWKWFAGNLASGGAAGASSLFFVYSLDYARTRLANDAKAAKGGGERQFNGLVD
VYRKTLKSDGIAGLYRGFNISCVGIIVYRGLYFGLYDSIKPVVLTGNLQDNFFASFALGW
LITNGAGLASYPIDTVRRRMMMTSGEAVKYKSSLDAFQQILKKEGPKSLFKGAGANILRA
IAGAGVLSGYDQLQILFFGKKYGSGGA
>Q8VYE2
MASIVLRSRLLPRLAKLRSRNLRCFSAEASSTNSTSRYSGVTSTQSMFSDFPPPNQPPPP
PPPQVEAAAAAATGKERKGLKYLGYALLWALTGATAATGYASFAYTIDEVNEKTKAFRES
ATKTPVIKSSGIDVIDKYQTKLYSAAMTGSARAIDKYLELREIVEEQVKGFTEPLSEKLL
PDLHPAEQHVFTLVLDLNETLLYTDWKRERGWRTFKRPGVDAFLEHLGKFYEIVVYSDQM
EMYVLPVCEKLDPNGYIRYKLARGATKYENGKHYRDLSKLNRDPKKILFVSANAFESTLQ
PENSVPIKPYKLEADDTALVDLIPFLEYVARNSPADIRPVLASFERKDIAKEFIDRSIEY
QKRKQGQLGQGRFWRR
>Q04716
MARKGNPISVRLDLNRSSDSSWFSYYYGKSVYQDVNLRSYFGSIRPPTRLTFGFRLGRCI
ILHFPKRTFIHFFLPRRPRRLKRREKSRPVKEKGRWGAFGKVGPIGCLHSSDGTEEERNE
VRGRGAGKRVESIRLDDREKQNEIRIWPKKKQGYGYHDRSPSIKKNLSKSLRVSGAFKHP
KYAGIENDIAFLIENDDSFRKTNLFKFFFPKKSRSDRPTSHLLKRTLPAVRPSLNYSVMQ
YLLNTKNKIHFDPVVVLNHFVAPGVAEPSTMGGANAQGRSLDKRIASCIAFFVENSTSEK
KCLAEAKKRVTHFIRQANDLRFAGTTKTTISLFPFFGATFFFPRDGVGVYNNLFFEDARE
QLLGQLRRKCWNLMGKDKVMELIEKFIDLNRIGELIRGIEMMIEIILRNRRIPYGYNYYL
NEVKKMRSLLYNRTNTNTLIESVKIKSVYQSASPIAQDISFQPRNKTRSFRSIFSKIVKD
IPLVMKKGVEGIRICCSGRLEGAEIARTECGKYGKTSRNVFNQKIDYAPAEVSTRYGISG
VKVWISYSKKKKGRAISETYEI
>P68514
MITFFEKLSTFCHNLTPTECKVSVISFFLLAFLLMAHIWLSWFSNNQHCLRTMRHLEKLK
IPYEFQYGWLGVKITIKSNVPNDEVTKKVSPIIKGEIEGKEEKKEGKGEIEGKEEKKEGK
GEIEGKEEKKEVENGPRK
>Q09152
MSVSCCCRNLGKTIKKAIPSHHLHLRSLGGSLYRRRIQSSSMETDLKSTFLNVYSVLKSD
LLHDPSFEFTNESRLWVDRMLDYNVRGGKLNRGLSVVDSFKLLKQGNDLTEQEVFLSCAL
GWCIEWLQAYFLVLDDIMDNSVTRRGQPCWFRVPQVGMVAINDGILLRNHIHRILKKHFR
DKPYYVDLVDLFNEVELQTACGQMIDLITTFEGEKDLAKYSLSIHRRIVQYKTAYYSFYL
PVACALLMAGENLENHIDVKNVLVDMGIYFQVQDDYLDCFADPETLGKIGTDIEDFKCSW
LVVKALERCSEEQTKILYENYGKPDPSNVAKVKDLYKELDLEGVFMEYESKSYEKLTGAI
EGHQSKAIQAVLKSFLAKIYKRQK
>O49203
MSSQICRSASKAAKSLLSSAKNARFFSEGRAIGAAAAVSASGKIPLYASNFARSSGSGVA
SKSWITGLLALPAAAYMIQDQEVLAAEMERTFIAIKPDGVQRGLISEIISRFERKGFKLV

GIKVIVPSKDFAQKHYHDLKERPFFNGLCDFLSSGPVIAMVWEGDGVIRYGRKLIGATDP
QKSEPGTIRGDLAVTVGRNIIHGSDGPETAKDEISLWFKPQELVSYTSNSEKWLYGDN
>Q39818
MASSLIAKRFLSSSLLSRSLLRPAASASHRSFDTNAMRQYDNRADDHSTDIDRHSERSFP
STARRDDIFLRCVGSIFSDSEFEPGSEHDGPGHGQSVPLRVARDRSWRWSGRGWDARETE
DALHLRVDMPGLAKEDVKISVEQNTLIIKGEGAKEGDEEESARRYTSRIDLPDKLYKIDQ
IRAEMKNGVLKVVVPKMKEEERKDVISVKVE
>Q9LHE5
MADLLPPLTAAQVDAKTKVDEKVDYSNLPSPVPYEELHREALMSLKSDNFEGLRFDFTRA
LNQKFSLSHSVMMGPTEVPAQSPETTIKIPTAHYEFGANYYDPKLLLIGRVMTDGRLNAR
LKADLTDKLVVKANALITNEEHMSQAMFNFDYMGSDYRAQLQLGQSALIGATYIQSVTNH
LSLGGEIFWAGVPRKSGIGYAARYETDKMVASGQVASTGAVVMNYVQKISDKVSLATDFM
YNYFSRDVTASVGYDYMLRQARVRGKIDSNGVASALLEERLSMGLNFLLSAELDHKKKDY
KFGFGLTVG
>Q38946
MNALAATNRNFRHASRILGLDSKIERSLMIPFREIKVECTIPKDDGTLVSYIGFRVQHDN
ARGPMKGGIRYHPEVDPDEVNALAQLMTWKTAVADIPYGGAKGGIGCSPRDLSLSELERL
TRVFTQKIHDLIGIHTDVPAPDMGTNAQTMAWILDEYSKFHGHSPAVVTGKPIDLGGSLG
REAATGRGVVFATEALLAEYGKSIQGLTFVIQGFGNVGTWAAKLIHEKGGKVVAVSDITG
AIRNPEGIDINALIKHKDATGSLNDFNGGDAMNSDELLIHECDVLIPCALGGVLNKENAG
DVKAKFIVEAANHPTDPDADEILSKKGVIILPDIYANAGGVTVSYFEWVQNIQGFMWEEE
KVNLELQKYMTRAFHNIKTMCHTHSCNLRMGAFTLGVNRVARATQLRGWEA
>O04630
MLLRLTARSIRRFTTSSSSLPLLSSSSFCTVPTMAANHPKDEAYLSAVIPKRIKLFEQIQ
ANQLENLKSLPHDPIKVTLPDGNVKEGKKWETTPMDIAAQISKGLANSALISAVDDVLWD
MNRPLEGDCKLELFKFDSDKGRDTLWHSSAHILGQALEQEYGCQLCIGPCTTRGEGFYYD
GFYGELGLSDNHFPSIEAGAAKAAKEAQPFERIEVTKDQALEMFSENNFKVELINGLPAD
MTITVYRCGPLVDLCRGPHIPNTSFVKAFKCLRASSAYWKGDKDRESLQRVYGISYPDQK
QLKKYLQFLEEAKKYDHRLLGQKQELFFSHQLSPGSYFFLPLGTRVYNRLMDFIKNQYWH
RGYTEVITPNMYNMELWQTSGHADNYKDNMFTFNIEKQEFGLKPMNCPGHCLIFQHRVRS
YRELPMRLADFGVLHRNEASGALSGLTRVRRFQQDDAHIFCTTEQVKGEVQGVLEFIDYV
YKVFGFTYELKLSTRPEKYLGDLETWDKAEADLKEAIEAFGKPLVLNEGDGAFYGPKIDI
TVSDAMNRKFQCATLQLDFQLPIRFNLEYAAEDEAKKSRPVMIHRAVLGSVERMFAILLE
HYKGKWPFWISPRQAIVCPISEKSQQYAEKVQKQIKDAGFYVDADLTDRKIDKKVREAQL
AQYNYILVVGETEAATGQVSVRVRDNAAHSVKSIEDLLEEFKAKTAEFV
>Q8S944
MTIEEVSGETPPSTPPSSSTPSPSSSTTNAAPLGSSVIPIVNKLQDIFAQLGSQSTIALP
QVVVVGSQSSGKSSVLEALVGRDFLPRGNDICTRRPLVLQLLQTKSRANGGSDDEWGEFR
HLPETRFYDFSEIRREIEAETNRLVGENKGVADTQIRLKISSPNVLNITLVDLPGITKVP
VGDQPSDIEARIRTMILSYIKQDTCLILAVTPANTDLANSDALQIASIVDPDGHRTIGVI
TKLDIMDKGTDARKLLLGNVVPLRLGYVGVVNRCQEDILLNRTVKEALLAEEKFFRSHPV
YHGLADRLGVPQLAKKLNQILVQHIKVLLPDLKSRISNALVATAKEHQSYGELTESRAGQ
GALLLNFLSKYCEAYSSLLEGKSEEMSTSELSGGARIHYIFQSIFVKSLEEVDPCEDLTD
DDIRTAIQNATGPRSALFVPDVPFEVLVRRQISRLLDPSLQCARFIFEELIKISHRCMMN

ELQRFPVLRKRMDEVIGDFLREGLEPSEAMIGDIIDMEMDYINTSHPNFIGGTKAVEAAM
HQVKSSRIPHPVARPKDTVEPDRTSSSTSQVKSRSFLGRQANGIVTDQGVVSADAEKAQP
AANASDTRWGIPSIFRGGDTRAVTKDSLLNKPFSEAVEDMSHNLSMIYLKEPPAVLRPTE
THSEQEAVEIQITKLLLRSYYDIVRKNIEDSVPKAIMHFLVNHTKRELHNVFIKKLYREN
LFEEMLQEPDEIAVKRKRTQETLHVLQQAYRTLDELPLEADSVSAGMSKHQELLTSSKYS
TSSSYSASPSTTRRSRRAGDQHQNGYGF

>Q9LQI7

MSRFRSLLQASVNATKKALTWNVEEWVPPAEKHIFKFHSKEDLKKWHLYSDSEYGGVFSG
NLSVDLSEGSKWNISRSGFCGMRSKKFDGFIDLDGYDAIALRIRGDGRCYISTIYTENWV
NSPGQSEDNSWQAFVFAPKDSWYTAKIPLARYLPTWRGNVIDVEMEMNPGRVLGMSLSVN
AEGGAVGAKSGAGDFRVEIDWIKALRLP

>Q9SHJ5

MVLPELLVILAEWVLYRLLAKSCYRAARKLRGYGFQLKNLLSLSKTQSLHNNSQHHLHNH
HQQNHPNQTLQDSLDPLFPSLTKYQELLLDKNRACSVSSDHYRDTFFCDIDGVLLRQHSS
KHFHTFFPYFMLVAFEGGSIIRAILLLLSCSFLWTLQQETKLRVLSFITFSGLRVKDMDN
VSRSVLPKFFLENLNIQVYDIWARTEYSKVVFTSLPQVLVERFLREHLNADDVIGTKLQE
IKVMGRKFYTGLASGSGFVLKHKSAEDYFFDSKKKPALGIGSSSSPQDHIFISICKEAYF
WNEEESMSKNNALPRERYPKPLIFHDGRLAFLPTPLATLAMFIWLPIGFLLAVFRISVGV
FLPYHVANFLASMSGVRITFKTHNLNNGRPEKGNSGVLYVCNHRTLLDPVFLTTSLGKPL
TAVTYSLSKFSEFIAPLKTVSLKRDRKKDGEAMQRLLSKGDLVVCPEGTTCREPYLLRFS
PLFAELTEDIVPVAVDARVSMFYGTTASGLKCLDPIFFLMNPRPVYCLEILKKLPKEMTC
AGGKSSFEVANFIQGELARVLGFECTNLTRRDKYLVLAGNEGIVR

>Q93Y32

MALRRCLPQYSTTSSYLSKIWGFRMHGTKAAASVVEEHVSGAEREDEEYADVDWDNLGFS
LVRTDFMFATKSCRDGNFEQGYLSRYGNIELNPAAGILNYGQGLIEGMKAYRGEDGRVLL
FRPELNAMRMKIGAERMCMHSPSVHQFIEGVKQTVLANRRWVPPPGKGSLYLRPLLFGSG
ASLGVAAASEYTFLVFGSPVQNYFKEGTAALNLYVEEVIPRAYLGGTGGVKAISNYGPVL
EVMRRAKSRGFSDVLYLDADTGKNIEEVSAANIFLVKGNTIVTPATSGTILGGITRKSII
EIALDLGYKVEERSVPVEELKEAEEVFCTGTAAGVASVGSITFKNTRTEYKVGDGIVTQQ
LRSILVGIQTGSIQDTKDWVLQIA

>P26851

MDLVKYLTFSMILFLLGIWGIFLNRKNILIMLMSIELMLLAVNLNFLVFSVYLDDMMGQL
FALFVLTVAAAESAIGLAILVITFRIRGTIAVEFINCMKG

>P93303

MPQLDKFTYFSQFFWLCLFFFTFYIFICNDGDGVLGISRILKLRNQLLSHRGKTIRSKDP
NSLEDLLRKGFSTGVSYMYASLFEVSQWCKAVDLLGKRRKITLISCFGEISGSRGMERNI
LYNISKSSPSNTGRWITCRNCRNDIMLIHVVHGQGSIK

>P34944

MDNQLFFKSLIATLPKWIHKCQTSKHENILYTNPNSLFQLLYFLKYHTNTRFKVLIDICG
VDYPSRKRRFEVVYNLLSIDYNTRIRILTSVDEITPICSVVSIFPSAGWWERETWDMFGV
YFSNHPDLRRILTDYGFEGHPLRKDFPLSGYVEVRYDDSEKRVVSEPIEMTQEFRYFDFA
SPWEQMSRSDESNQK

>O24164

MAPSAGEDKHSSAKRVAVIGAGVSGLAAAYKLKIHGLNVTVFEAEGKAGGKLRSVSQDGL

IWDEGANTMTESEGDVTFLIDSLGLREKQQFPLSQNKRYIARNGTPVLLPSNPIDLIKSN
FLSTGSKLQMLLEPILWKNKKLSQVSDSHESVSGFFQRHFGKEVVDYLIDPFVAGTCGGD
PDSLSMHHSFPELWNLEKRFGSVILGAIRSKLSPKNEKKQGPPKTSANKKRQRGSFSFLG
GMQTLTDAICKDLREDELRLNSRVLELSCSCTEDSAIDSWSIISASPHKRQSEEESFDAV
IMTAPLCDVKSMKIAKRGNPFLLNFIPEVDYVPLSVVITTFKRENVKYPLEGFGVLVPSK
EQQHGLKTLGTLFSSMMFPDRAPNNVYLYTTFVGGSRNRELAKASRTELKEIVTSDLKQL
LGAEGEPTYVNHLYWSKAFPLYGHNYDSVLDAIDKMEKNLPGLFYAGNHRGGLSVGKALS
SGCNAADLVISYLESVSTDSKRHC
>P26854
MNKLAGAELSTLLEQRITNYYTKLQVDEIGRVVSVGDGIARVYGLNKIQAGEMVEFASGV
KGMALNLENENVGIVIFGSDTAIKEGDIVKRTGSIVDVPVGKGMLGRVVDALGVPIDGKG
ALSAVERRRVEVKAPGIIARKSVHEPMQTGLKAVDSLVPIGRGQRELIIGDRQTGKTAIA
IDTILNQKQINAQGTSDSEKLYCVYVAIGQKRSTVAQLVKILSEAGALEYSIIVAATASD
PAPLQFLAPYSGCAMGEYFRDNGMHALIIYDDLSKQSVAYRQMSLLLRRPPGREAFPGDV
FYLHSRLLERAAKMSDQTGAGSLTALPVIETQAGDVSAYIPTNVISITDGQIFLETELFY
RGSRPAINVGLSVSRVGSAAQLKAMKQVCGSLKLELAQYREVAAFAQFGSDLDAATQYLL
NRGARLTEILKQAQYSPIPIEKQIVVIYAAVKGYLDQIPVALITHYEQELLKSIDPGLLS
AIVQQKNITEQISSQLATFCQKFTQSFLATHQS
>Q9XIA7
MFPGMFMRKPDKAEALKQLRTHVALFGSWVVIIRAAPYVLSYFSDSKDELKIDF
>O82662
MRGLVNKLVSRSLSISGKWQNQQLRRLNIHEYQGAELMGKYGVNVPKGVAASSLEEVKKA
IQDVFPNESELVVKSQILAGGRGLGTFKSGLKGGVHIVKRDEAEEIAGKMLGQVLVTKQT
GPQGKVVSKVYLCEKLSLVNEMYFSIILDRKSAGPLIIACKKGGTSIEDLAEKFPDMIIK
VPIDVFAGITDEDAAKVVDGLAPKAADRKDSIEQVKKLYELFRKTDCTMLEINPLAETST
NQLVAADAKLNFDDNAAFRQKEVFAMRDPTQEDPREVAAAKVDLNYIGLDGEIGCMVNGA
GLAMATMDIIKLHGGTPANFLDVGGNASEHQVVEAFKILTSDDKVKAILVNIFGGIMKCD
VIASGIVNAAKEVALKVPVVVRLEGTNVEQGKRILKESGMKLITADDLDDAAEKAVKALA
H
>Q42686
MSLQSSIRADSNCTLPNNPVCVLLPVDFIVAAMASSTSSAMAKWAAQAARGFAAAAPSSG
KGRKVAVLGAAGGIGQPLSMLMKMNSQVSSLSLYDIAGTPGVAADVSHINTKAQVKGFDK
DGLAEALRGCDLVIIPAGVPRKPGMTRDDLFKINAGIVRDLVTAVGQHCPGAVLNIISNP
VNSTVPIAAEQLKKMGVYDKRKVMGVTTLDVVRAKTFYAEKNGLDVASVDVPVVGGHAGV
TILPLFSQATPKATMSAEVLDALTKRTQDGGTEVVQAKAGKGSATLSMAYAAALFADSCL
RGLNGAPVVECTYVESTVTDAPYFASKVKLSTEGVDKIHDLGPLSDYEKAGLKAMMPELL
ASIEKGVQFVKGA
>P46741
MNSTKIKEIEFQNVIVDPKINNLHLKTNTDISSNYYKAGTHIGHKSIHLSETHSWHPSMA
QYHLGIRDNITICNVQQTQKCLSRAFYVLTQILDNGGNVLIVNTNPEFYKLSINSMKFIE
KNLPFQTFSAISYCFYKWIGGTLTNYKQISKSIYCYVKFTQRCGKFCEKNNIDFTRYQKI
KKCFQGYGSVSGDSIVMSLQKKPDVIFIFNPGDNKNLILEANRLQIPVIGCTDMSSNTNG
ISYPIPCNNTSVEFTLYLYKKLYKVLHYISTKKK
>O82663

MWRCVSRGFRAPASKTSSLFDGVSGSRFSRFFSTGSTDTRSSYTIVDHTYDAVVVGAGGA
GLRAAIGLSEHGFNTACITKLFPTRSHTVAAQGGINAALGNMSEDDWRWHMYDTVKGSDW
LGDQDAIQYMCREAPKAVIELENYGLPFSRTEEGKIYQRAFGGQSLDFGKGGQAYRCACA
ADRTGHALLHTLYGQAMKHNTQFFVEYFALDLLMASDGSCQGVIALNMEDGTLHRFRSSQ
TILATGGYGRAYFSATSAHTCTGDGNAMVARAGLPLQDLEFVQFHPTGIYGAGCLITEGS
RGEGGILRNSEGERFMERYAPTAKDLASRDVVSRSMTMEIREGRGVGPHKDHIYLHLNHL
PPEVLKERLPGISETAAIFAGVDVTKEPIPVLPTVHYNMGGIPTNYHGEVVTIKGDDPDA
VIPGLMAAGEAACASVHGANRLGANSLLDIVVFGRACANRVAEISKPGEKQKPLEKDAGE
KTIAWLDRLRNSNGSLPTSTIRLNMQRIMQNNAAVFRTQETLEEGCQLIDKAWESFGDVQ
VKDRSMIWNSDLIETLELENLLINASITMHSAEARKESRGAHAREDFTKREDGEWMKHTL
GYWEDEKVRLDYRPVHMDTLDDEIDTFPPKARVY
>P46743
MKISLKCLNTHTAEIYRKCLLLSDSTVNVSSVDFTNQSKLNLLKQEKKSIESFNKNSLLN
ISNFRDLERQEKDIQKKTSKKVYKKSYSRSTKSIYSRTKIINERNKLHTLNQQIIFSTLA
RIHLKKKINLLFPVQEFLFELSKHRNKSAGSKSREYLEILLCVKKLKMFYGFIPLKQLHK
ILVQAKAMPGYFSKNFFSLIEKRLDVVLYRSGFTKTIVAARQACRHSQIYVNSKVCRIPS
TILESGDIISYKNQLDSLKTAEIKNNLFSNINTNVSGNEVSNNVSNKVSNKISSTLALNK
QSLLLLLFCAKSIYNESFFVSNKRKFKSNEVTFDNKKEITSAIDYTKYLQIKNSRKKYKL
ITLNNNLDSLSFNIFDKEILNSLSSKQNLPVNKQNIVEIENKKTNKFNTFSLLFQNLTSF
SKSIESYSSVLALKLQDHLLNIKGKRNEVSKNINKIERSQKDNVFINILQKINRPTHLEI
SSITNSIIFLYSPQRIYLPFYVDIDILRKSL
>Q9SD80
MVNNVVSIEKMKALWHSEVHDEQKWAVNMKLLRALGMFAGGVVLMRSYGDLMGV
>P46746
MLVKKSQISRKFIEKKSGIVHIQSTTNNTLITLTDLEGNTQFFVSAGTLGFKNSRKSTVY
ASGAAAEALASKAFNEGYRTIIVKIKGLGYGKKSAIRGLQKSNLVIKQIQEVTPIAHNGC
RPPKKRRV
>P49364
MRGGLWQLGQSITRRLANGGDKKAVARRCFATESELKKTVLYDFHVAHGGKMVPFAGWSM
PIQYKDSIMDSTLNCRQNGSLFDVSHMCGLSLKGKDVVSFLEKLVIADVAALAHGTGTLT
VFTNEKGGAIDDSVITKVTDDHLYLVVNAGCRDKDLAHIEEHMKAFKAKGGDVSWHIHDE
RSLLALQGPLAAPVLQHLTKEDLSKLYFGEFRVLDINGSQCFLTRTGYTGEDGFEISVPS
EHGVELAKALLEKSEGKIRLTGLGARDSLRLEAGLCLYGNDLEQHITPIEAGLTWAIGKR
RRAEGGFLGADVILKQLADGPSIRRVGFISSGPPPRSHSEIQDEGGNNIGEVTSGGFSPC
LKKNIAIGYVKSGLHKAGTKVKIIIRGKQNEGVVTKMPFVPTKYYKPS
>P46748
MKTLSILDTIIKIPEKIEIHPTTTEYIYTITGPLGSSSINLKKLDKNGIACINFDIQNKQ
VLIRSLYPKYNGLYKKLIENKFLGVSRGFCVYLEIVGVGYRAALLSSSLQNSTKDTNDTI
VLKLGHSHDIHYKVPNGVRVFLQSPSEICIFGVDLNQVTQVAHSIRNTRPPSVYKGKGIR
YTNEKIVTKTGKRK
>P46749
MAIYKTKNTLKKMNLDYSFRTNSTLVSNSEMFSLLYADTTLQKYYKNIVCQDLCLKQDYK
NIMECVSLNKIVCNTSSKHYAGEKESILPAFTALEMITGQKPKYTCAKKSISTFKLRQNQ
ILGCKNSLRGNTMYRFLEKYISIVSTRIKDWSENYSSSNKSNSLTTAYNKDYVVLNSNLF

PELQQHDELFQNVTGIEISFSTLSNNVKTSTKTSRYKKDGILLYSAFQMPK
>Q43127
MAQILAASPTCQMRVPKHSSVIASSSKLWSSVVLKQKKQSNNKVRGFRVLALQSDNSTVN
RVETLLNLDTKPYSDRIIAEYIWIGGSGIDLRSKSRTIEKPVEDPSELPKWNYDGSSTGQ
APGEDSEVILYPQAIFRDPFRGGNNILVICDTWTPAGEPIPTNKRAKAAEIFSNKKVSGE
VPWFGIEQEYTLLQQNVKWPLGWPVGAFPGPQGPYYCGVGADKIWGRDISDAHYKACLYA
GINISGTNGEVMPGQWEFQVGPSVGIDAGDHVWCARYLLERITEQAGVVLTLDPKPIEGD
WNGAGCHTNYSTKSMREEGGFEVIKKAILNLSLRHKEHISAYGEGNERRLTGKHETASID
QFSWGVANRGCSIRVGRDTEAKGKGYLEDRRPASNMDPYIVTSLLAETTLLWEPTLEAEA
LAAQKLSLNV
>Q9LEV3
MQGVIRSFVSGGNVVKGSVLQHLRVINPAIQPSVFCSRSESTQPARMEESGFESTTISDV
MKSKGKSADGSWLWCTTDDTVYDAVKSMTQHNVGALVVVKPGEQQALAGIITERDYLRKI
IVQGRSSKSTKVGDIMTEENKLITVTPETKVLRAMQLMTDNRIRHIPVIKDKGMIGMVSI
GDVVRAVVHEHREELQRLNAYIQGGY
>P51428
MTTKIRIVIRSFDHPFLENHFGGLPPYTWKIGLPESRVLYTVLRSPHIDKKSREQFEMEI
KKKYLVIKTEKHELRKKFFWLKRQRLFGAQYEILFFCKTRSDKGKLQRLL
>Q94BZ7
MALLQRASYLRLYYLRLMGSRPRLFSSSLSPALHRHSSTLSSPPFSSPSPSFRLKFQLTS
VLSQRLIQRNAISSRFLSTEASQETTTSKGYSSEQIQVLEGLDPVRKRPGMYIGSTGSRG
LHHLVYEILDNAIDEAQAGYASKVDVVLHADGSVSVVDNGRGIPTDLHPATKKSSLETVL
TVLHAGGKFGGTSSGYSVSGGLHGVGLSVVNALSEALEVSVWRDGMEHKQNYSRGKPITT
LTCRVLPLESKGTKGTSIRFWPDKEVFTTAIEFDHNTIAGRIRELAFLNPKVTISLKKED
DDPEKTQYSEYSFAGGLTEYVSWLNTDKNPIHDVLGFRREINGATVDVALQWCSDAYSDT
MLGYANSIRTIDGGTHIEGVKASLTRTLNTLAKKSKTVKEKDISLSGEHVREGLTCIVSV
KVPNPEFEGQTKTRLGNPEVRKIVDQSVQEYLTEFLELHPDILESIISKSLNAYKAALAA
KRARELVRSKSVLKSSSLPGKLADCSSTDPEVSEIFIVEGDSAGGSAKQGRDRRFQAILP
LRGKILNIERKDEAAMYKNEEIQNLILGLGLGVKGEDFKKENLRYHKIIILTDADVDGAH
IRTLLLTFFFRYQRALFDAGCIYVGVPPLFKVERGKNAQYCYDDADLKKITSNFPANASY
NIQRFKGLGEMMPEQLWETTMNPETRILKQLVVDDIAEANMTFSSLMGARVDVRKELIKN
AATRINLQRLDI
>P49727
MLRVAGRRLSSSLSWRPAAAVARGPLAGAGVPDRDDDSARGRSQPRFSIDSPFFVASRGF
SSTETVVPRNQDAGLADLPATVAAVKNPNPKVVYDEYNHERYPPGDPSKRAFAYFVLSGG
RFIYASLLRLLVLKFVLSMSASKDVLALASLEVDLSSIEPGTTVTVKWRGKPVFIRRRTE
DDIKLANSVDVASLRHPEQDAERVKNPEWLVVIGVCTHLGCIPLPNAGDFGGWFCPCHGS
HYDISGRIRKGPAPFNLEVPTYSFLEENKLLVG
>Q93XM7
MADAWKDLASGTVGGAAQLVVGHPFDTIKVKLQSQPTPAPGQLPRYTGAIDAVKQTVASE
GTKGLYKGMGAPLATVAAFNAVLFTVRGQMEGLLRSEAGVPLTISQQFVAGAGAGFAVSF
LACPTELIKCRLQAQGALAGASTTSSVVAAVKYGGPMDVARHVLRSEGGARGLFKGLFPT
FAREVPGNATMFAAYEAFKRFLAGGSDTSSLGQGSLIMAGGVAGASFWGIVYPTDVVKSV
LQVDDYKNPRYTGSMDAFRKILKSEGVKGLYKGFGPAMARSVPANAACFLAYEMTRSSLG

>Q8L3X9

MATSNLRRHLSASRLRLNRFISTSSSYHSHRRVVVTGLGMVTPLGRGVETTWRRLIDGEC
GIRGLTLDDLKMKSFDEETKLYTFDQLSSKVAAFVPYGSNPGEFDEALWLNSKAVANFIG
YAVCAADEALRDAEWLPTEEEEKERTGVSIGGGIGSICDIVEAAQLICEKRLRRLSPFFI
PKILVNMASGHVSMKYGFQGPNHAAVTACATGAHSIGDATRMIQFGDADVMVAGGTESSI
DALSVAGFSRSRALSTKFNSSPQEASRPFDCDRDGFVIGEGSGVIVLEEYEHAKRRGAKI
YAELCGYGMSGDAHHITQPPEDGKGAVLAMTRALRQSGLCPNQIDYVNAHATSTPIGDAV
EARAIKTVFSEHATSGTLAFSSTKGATGHLLGAAGAVEAIFSILAIHHGVAPMTLNVKNP
DPIFDKRFMPLTTSKKMLVRTAMSNSFGFGGTNASLLFASI

>Q01607

MSGGRWISNLRQVFGRSRASISSSLLPFPPTSISSGFHVPRLLDAWKNPVAAAAAFRLPQ
SSGFATMNQLIRHGREEKHRSDRKRALGKCPQKQGVCLRVSTRTPKKPNSALGKIAKVRL
SNRNDVFAYIPGEGHNLQEHSMVLVRGGRVKDLPGVKFHCIRGVKDLLGIPDRRRGRSKY
GAEKPKST

>Q9M7T0

MAMSILKLRNLSALRSAANSARIGVSSRGFSKLAEGTDITSAAPGVSLQKARSWDEGVSS
KFSTTPLSDIFKGKKVVIFGLPGAYTGVCSQQHVPSYKSHIDKFKAKGIDSVICVSVNDP
FAINGWAEKLGAKDAIEFYGDFDGKFHKSLGLDKDLSAALLGPRSERWSAYVEDGKVKAV
NVEEAPSDFKVTGAEVILGQI

>Q8VY06

MLRSLTCSSTITSTSLFFRSFRQLPRSYLSPSSSTTVVGASGRNIRRLSTLEAAGRRLFL
RRGLKLLSAASRGLNGQFSRLSIRAVATQSAPSSYPGQDEAEKLGFEKVSEEFISECKSK
AVLFKHKKTGCEVMSVSNDDENKVFGIVFRTPPKDSTGIPHILEHSVLCGSRKYPMKEPF
VELLKGSLHTFLNAFTYPDRTCYPVASTNKKDFYNLVDVYLDAVFFPKCVDDVHTFQQEG
WHYELNDPSEDISYKGVVFNEMKGVYSQPDNILGRVTQQALCPENTYGVDSGGDPKDIPK
LTFEKFKEFHRQYYHPSNARIWFYGDDDPVHRLRVLSEYLDMFDASPARDSSKVEPQKLF
SRPRRIVEKYPAGEDGDLKKKHMVCLNWLLSDKPLDLQTQLALGFLDHLMLGTPASPLRK
ILLESGLGEALVNSGMEDELLQPQFSIGLKGVSDDNVQKVEELVMNTLRKLADEGFDTDA
VEASMNTIEFSLRENNTGSSPRGLSLMLQSIAKWIYDMDPFEPLKYEEPLKSLKARIAEK
GSKSVFSPLIEEYILNNPHCVTIEMQPDPEKASLEEAEEKSILEKVKASMTEEDLTELAR
ATEELRLKQETPDPPDALKCVPSLNLSDIPKEPIYVPTEVGDINGVKVLRNDLFTNNILY
TEVVFDMGSVKHELLQLIPLFCQSLLEMGTQDLTFVQLNQLIGRKTGGISVYPLTSSVYG
RDDPCSKIIVRGKSMVGRAEDLFNLMNCVLQEVRFTDQQRFKQFVSQSRARMENRLRGSG
QGIAAARMDAMLNVAGWMSEQMGGLSYLEFLHTLEQKVDQDWEGISSSLEEIRRSFLSRN
GCIVNMTADGKSLTNTEKYVGKFLDLLPENPSGELVTWDARLPLRNEAIVIPTQVNYVGK
AGNIYSSGYKLDGSSYVISKHISNTWLWDRVRVSGGAYGGSCDFDSHSGVFSFLSYRDPN
LLKTLDIYDGTGDFLRGLDVDEDTLTKAIIGTIGDVDSYQLPDAKGYTSLLRHLLNVTDE
ERQIRREEILSTSLKDFKEFAEAIDSVSDKGVAVAVASQEDIDAANRERSNFFEVKKAAL

>P26863

MSFSQLFPKYNSSFNPLRGSAIQCSVIQLQQNKVLVDTGLKTPIICFQHELKRVPITKQA
RFHFGIEDVEVFGEPKMLLPKPLEIKCKRKLVWIELTKIWRSDQNLVKGFILNSVKGGYA
VAIAGYIAFLPKSLLRSRKVFYSQWRIFSILNMKPKISNIVVKEIGDGKIDYFSPTKSHQ

KQTKYLGAKLKHWRNMKKNTNVKKKYIFSEKVPTTKKTKQGFKHLGPKPLAYTEKKRETT
KQSTKNNVFQLKDQGQGKSLVFVDVLTQSS
>Q84KJ5
MAASSLSLSSPLSNPLRRFTLHHLHLSKKPLSSSSLFLCSAAKMNDHIHRVPALTEEEID
SVAIKTFERYALPSSSSVKRKGKGVTILWFRNDLRVLDNDALYKAWSSSDTILPVYCLDP
RLFHTTHFFNFPKTGALRGGFLMECLVDLRKNLMKRGLNLLIRSGKPEEILPSLAKDFGA
RTVFAHKETCSEEVDVERLVNQGLKRVGNSTKLELIWGSTMYHKDDLPFDVFDLPDVYTQ
FRKSVEAKCSIRSSTRIPLSLGPTPSVDDWGDVPTLEKLGVEPQEVTRGMRFVGGESAGV
GRVFEYFWKKDLLKVYKETRNGMLGPDYSTKFSPWLAFGCISPRFIYEEVQRYEKERVAN
NSTYWVLFELIWRDYFRFLSIKCGNSLFHLGGPRNVQGKWSQDQKLFESWRDAKTGYPLI
DANMKELSTTGFMSNRGRQIVCSFLVRDMGLDWRMGAEWFETCLLDYDPCSNYGNWTYGA
GVGNDPREDRYFSIPKQAQNYDPEGEYVAFWLQQLRRLPKEKRHWPGRLMYMDTVVPLKH
GNGPMAGGSKSGGGFRGSHSGRRSRHNGP
>P26867
MNLFGKSNFNCVFSSSLDWFHSSRLSEKVGTKKNRICRETESFYALCLSHRRYLCYALEG
LLPSRPRGRRASTYNCSDNLGYIRGLNGKQKQLIKKLVHICMIDGKKTRSRAIVYKTFHR
LAPHGDVIKLLVNAIENVKPICEVKKVRISGTTRLVPSIIATNRQETLAIRWMLESAAKR
RMGKKSISLDQCLYAEILEASQKMGIARKKRDDLHKLAEANRSFSHYRWW
>P26868
MHTLSNLLSSIKNAQKAQKRVLYFSSFKKISKRKKRFVCSACKMMPRVFVSRLCWDFCRI
LYNEGYIHGFSQEADGSLRIVLKYHSSGIGVIKKMKTISKPGFRIYSSKNRLSKKREGLG
ITILSTSKGNLICDREAQKTNFGGGEILCQVF
>P18123
MTMDPTKFRPSSSHDTTVTTTNAGAPVWNDNEALTVGPRGPILLEDYHLIEKVAHFARER
IPERVVHARGASAKGFFECTHDVTSLTCADFLRAPGVRTPVIVRFSTVIHERGSPETIRD
PRGFAVKFYTREGNWDLLGNNFPVFFIRDGIKFPDVIHAFKPNPRSHVQEYWRVFDFLSH
LPESLHTFFFLFDDVGVPSDYRHMEGFGVNTYTFVSAAGKAQYVKFHWKPTCGVRCILTD
EEAALVGGRNHSHATQDLYDSIAAGSFPEWTLYVQVMDPDTEEQYDFDPLDDTKTWPEDL
LPLRPVGRLVLDRNVDNFFNENEQLAFGPGLVVPGIYYSDDKMLQCRVFAYADTQRYRLG
PNYLMLPVNAPRCAHHNNHYDGAMNFMHRDEEVDYYPSRHAPLRQAAPPTPLPPRPVAGR
REKATIRKPNDFKQPGERYRSWDADRQDRFVRRFADSLGHPKVSQELRSIWIDLLAKCDA
SLGMKIATRLNMKANM
>Q38813
MRRFNWVLRHVQARRTFDSAIGLRQGSQKPLFERYIHATGINNSSARNYYDVLGVSPKAT
REEIKKSFHELAKKFHPDTNRNNPSAKRKFQEIREAYETLGNSERREEYDKLQYRNSDYV
NNDGGDSERFRRAYQSNFSDTFHKIFSEIFENNQIKPDIRVELSLSLSEAAEGCTKRLSF
DAYVFCDSCDGLGHPSDAAMSICPTCRGVGRVTIPPFTASCQTCKGTGHIIKEYCMSCRG
SGIVEGTKTAELVIPGGVESEATITIVGAGNVSSRTSQPGNLYIKLKVANDSTFTRDGSD
IYVDANISFTQAILGGKVVVPTLSGKIQLDIPKGTQPDQLLVLRGKGLPKQGFFVDHGDQ
YVRFRVNFPTEVNERQRAILEEFAKEEINNELSDSAEGSWLYQKLSTG
>P09233
MALRTLASKKVLSFPFGGAGRPLAAAASARGVTTVTLPDLSYDFGALEPAISGEIMRLHH
QKHHATYVANYNKALEQLETAVSKGDASAVVQLQAAIKFNGGGHVNHSIFWKNLKPISEG
GGEPPHGKLGWAIDEDFGSFEALVKKMNAEGAALQGSGWVWLALDKEAKKVSVETTANQD

PLVTKGASLVPLLGIDVWEHAYYLQYKNVRPDYLNNIWKVMNWKYAGEVYENVLA
>P20115
MVFFRSVSAFTRLRSRVQGQQSSLSNSVRWIQMQSSTDLDLKSQLQELIPEQQDRLKKLK
SEHGKVQLGNITVDMVIGGMRGMTGLLWETSLLDPEEGIRFRGLSIPECQKVLPTAQSGA
EPLPEGLLWLLLTGKVPSKEQVEALSKDLANRAAVPDYVYNAIDALPSTAHPMTQFASGV
MALQVQSEFQKAYENGIHKSKFWEPTYEDCLNLIARVPVVAAYVYRRMYKNGDSIPSDKS
LDYGANFSHMLGFDDEKVKELMRLYITIHSDHEGGNVSAHTGHLVGSALSDPYLSFAAAL
NGLAGPLHGLANQEVLLWIKSVVEECGEDISKEQLKEYVWKTLNSGKVIPGYGHGVLRNT
DPRYVCQREFALKHLPDDPLFQLVSKLYEVVPPVLTELGKVKNPWPNVDAHSGVLLNHYG
LTEARYYTVLFGVSRSLGICSQLIWDRALGLALERPKSVTMDWLEAHCKKASSA
>O64967
MEASRRSAVKPVIVLKPSKRFPLVEDTPGKASDALPLPLYLTNAVFFTLFFTVVYFLLSR
WREKIRASIPLHAVTFPEIVAIFAFVASLIYLLGFFGIDFVQSLIIRPSGDVWSGEDYEE
ENEVLLHEEDARTVPCGQALDCSVPSLPHMARNVTAQRLFDEKPVRVATEEDARKVSCGQ
AVDCSLHSLPPRPPIVTSQKLFHEKTVIVTTEEDEEIIKSVVAGTLPSYSLESKLGDCKR
AAAIRREALQRLTGRSLSGLPLDGFDYESILGQCCEMPVGYVQIPVGIAGPLLLNGREYS
VPMATTEGCLVASTNRGCKAIHLSGGATSILLKDGMTRAPVVRFSTAKRAAELKFYLEDP
ENFDTLAVVFNRSSRFGRLQSIKCAIAGKNLYLRFTCSTGDAMGMNMVSKGVQNVLDFLQ
TDFPDMDVIGISGNFCSDKKPAAVNWIEGRGKSVVCEAIIEGDVVRKVLKTSVESLVELN
MLKNLTGSAMAGALGGFNAHASNIVTAIYIATGQDPAQNVESSHCITMMEAVNDGKDLHI
SVTMPSIEVGTVGGGTQLASQSACLNLLGVKGASKDVAGANSRMLATIVTGAVLAGELSL
MSALAAGQLVKSHMKYNRSSKDMSNLSS
>P26360
MAMAALRREGRRLAAAPFTSPTPLNALRSSLVSPSEEIGLSGVRSISTQVVRNRMKSVKN
IQKITKAMKMVAASKLRAIQTRAENSRGLWQPFTALLGDTPSVDVKKNVIITISSDKGLC
GGINSTSVKTSRNIHKLNSGPEKENKYVILGEKAKAQLVRDSKKDIELIITELQKNPLNY
TQVSVVADDILKNVEFDALRIVFNKFQSVVSFVPTMSTVLSPEVVERESESGGKLGDLDS
YEIEGAESKSEVLQNLTEFQFSSVLFNAVLENACSEQGARMSAMDSSSRNAGEMLDRLTL
TYNRTRQASITTELIEIISGASALEG
>Q42191
MAFRQTLSIRSRLFARRNQPVYHIIPRESDHERDSFCQETSQRSYHSFLHQRSVNNSDFS
KVSGGSLHLPLAPTSGFAFYRYMSSAPGVGSEKIGVMSDIAEVITDSTLQDVPAQAAAAV
SEVTLAAADSFFPIAALQQCIDMVHTFTGFEWWASIVVATILIRSSTVPLLIKQMKDTTK
LALMRPRLESIREEMQNKGMDSVTMAEGQKKMKNLFKEYGVTPFTPMKGMFIQGPLFICF
FLAIRNMAEKVPSFQTGGALWFTDLTTPDSLYILPVITGLTFLITVECNAQEGMEGNPMA
GTVKTVCRVFALLTVPMTMSFPQAIFCYWITSNLFSLMYGLVIKRPQVKKMLRIPDLPPP
PPGQQPSFDLFSALKKMKAMTQDHTQNQIEPPSPVNPRLSSTSLSPVSKRLKALESQVKG
RKKNSSKKK
>Q9FE64
MAMARRSASRLLSSFRPFSLLLQPLDDAPSLSAAAAAASARRGMSSASALRARDEKEVAR
WRESMDRMRNIGISAHIDSGKTTLTERVLYYTGRIHEIHEVRGRDGVGAKMDSMDLEREK
GITIQSAATYCTWNGYQVNIIDTPGHVDFTIEVERALRVLDGAILVLCSVGGVQSQSITV
DRQMRRYEIPRVAFINKLDRMGADPWKVLNQARSKLRHHNAAVQVPIGLEEEFEGLVDLV
ELKAYKFEGGSGQNVVASDVPSNMQDLVMEKRRELIEVVSEVDDQLAEAFLNDEPIQANQ

LKAAIRRATVARKFIPVYMGSAFKNKGVQPLLDGVLDYLPCPMEVESYALDQNKSEEKVL
LAGTPAEPLVALAFKLEEGRFGQLTYLRIYDGVIRKGDFIYNVNTGKKIKVPRLVRMHSN
EMEDIQEAHAGQIVAVFGVDCASGDTFTDGSVKYTMTSMNVPEPVMSLAVSPISKDSGGQ
FSKALNRFQKEDPTFRVGLDPESGETIISGMGELHLDIYVERIRREYKVDAKVGKPRVNF
RETITQRAEFDYLHKKQSGGQGQYGRVCGYIEPLPSESDGKFEFDNMIIGQAIPSNFIPA
IEKGFKEACNSGSLIGHPVENIRIVLTDGASHAVDSSELAFKLASIYAFRQCYAAARPVI
LEPVMKVELKVPTEFQGTVTGDMNKRKGIIVGNDQEGDDTVVVCHVPLNNMFGYSTALRS
MTQGKGEFSMEYLEHNTVSQDVQMQLVNTYKASRGTE
>P27168
MSFTGIFHFFTNSPCDAAEPWQLGSQDAATPMMQGIIDLHHDIFFFLILILVFVSRILVR
ALWHFHSKKNPIPQRIVHGTTIEILRTIFPSIIPMFIAIPSFALLYSMDEVVVDPAMTIK
AIGHQWYRTYEYSDYNSSDEQSLTFDSYTIPEDDPELGQSRLLEVDNRVVVPAKTHLRII
VTSADVPHSWAVPSSGVKCDAVPGRLNQISISVQREGVYYGQCSEICGTNHAFTPIVVEA
VSRKDYGSRVSNQLIPQTGEA
>Q9ZUX4
MATRNALRIVSRRFSSGKVLSEEERAAENVFIKKMEQEKLQKLARQGPGEQAAGSASEAK
VAGATASASAESGPKVSEDKNRNYAVVAGVVAIVGSIGWYLKAGGKKQPEVQE
>P93033
MSIYVASRRLSGGTTVTALRYATSLRSYSTSFREERDTFGPIQVPSDKLWGAQTQRSLQN
FEIGGERERMPEPIVRAFGVLKKCAAKVNMEYGLDPTIGKAIMQAAQEVAEGKLNDHFPL
VVWQTGSGTQSNMNANEVIANRAAEILGRKRGEKCVHPNDHVNRSQSSNDTFPTVMHIAA
ATEINSRLIPSLKTLHSTLESKSFEFKDIVKIGRTHTQDATPLTLGQEFGGYATQVKYGL
NRVTCTLPRLYQLAQGGTAVGTGLNTKKGFDVKIAAAVAEETNLPFVTAENKFEALAAHD
ACVETSGSLNTIATSLMKIANDIRFLGSGPRCGLGELVLPENEPGSSIMPGKVNPTQCEA
LTMVCAQVMGNHVAVTVGGSNGHFELNVFKPVIASALLHSVRLIADASASFEKNCVRGIE
ANRERISKLLHESLMLVTSLNPKIGYDNAAAVAKKAHKEGCTLKEAALNLGVLTAEEFDT
LVVPEKMIGPSD
>P27456
MNQAMATPLSLSCCSPTLTRSTLFFTKTFPFSRSFSTPLPLSTKTLISLSPPHRTFAVRA
ESQNGADPARQYDFDLFTIGAGSGGVRASRFASNFGASSAVCELPFSTISSDTTGGVGGT
CVIRGCVPKKLLVYASKFSHEFEESNGFGWRYDSEPKHDWSSLIANKNAELQRLTGIYKN
TLKNAGVKLIEGRGKIVDAHTVDVDGKLYSAKHILVSVGGRPFIPDIPGKEYAIDSDAAL
DLPSKPQKIAIVGGGYIALEFAGIFNGLKSEVHVFIRQKKVLRGFDEEIRDFVAENMALR
GIEFHTEESPVAITKAADGSLSLKTNKGTEEGFSHIMFATGRSPNTKDLGLESVGVKVAK
DGSIEVDEYSQTSVPSIWAIGDATNRVNLTPVALMEGVALAKTLFQNEPTKPDYRAIPSA
VFSQPPIGGVGLTEEQAAEQYGDIDVFTANFRPMKATLSGLPDRVFMKLIVSAETNVVLG
LHMCGEDAAEIAQGFAVGIKAGLTKADFDATVGIHPTAAEEFVTMRTPTRKVRKNQASQG
KSDSKAKAVAGS
>P48502
MASSFSRWLVDPKKNPLAAIHMKTLSSRLRNYGLRHDDLYDPMYDLDVKEALNRLPREIV
DARNQRLLRAMDLSMKHQYLPEDLQAMQTPFRNYLQEMLALVKRESAEREALGALPLYQR
TLP
>P48504
MSDEEVVDPKATLEVSCKPKCVRQLKEYQACTKRVEGDESGHKHCTGQYFDYWHCIDKCV

AAKLFDHLK
>P42043
MQATALSSGFNPLTKRKDHRFPRSCSQRNSLSLIQCDIKERSFGESMTITNRGLSFKTNV
FEQARSVTGDCSYDETSAKARSHVVAEDKIGVLLLNLGGPETLNDVQPFLYNLFADPDII
RLPRPFQFLQGTIAKFISVVRAPKSKEGYAAIGGGSPLRKITDEQADAIKMSLQAKNIAA
NVYVGMRYWYPFTEEAVQQIKKDKITRLVVLPLYPQYSISTTGSSIRVLQDLFRKDPYLA
GVPVAIIKSWYQRRGYVNSMADLIEKELQTFSDPKEVMIFFSAHGVPVSYVENAGDPYQK
QMEECIDLIMEELKARGVLNDHKLAYQSRVGPVQWLKPYTDEVLVDLGKSGVKSLLAVPV
SFVSEHIETLEEIDMEYRELALESGVENWGRVPALGLTPSFITDLADAVIESLPSAEAMS
NPNAVVDSEDSESSDAFSYIVKMFFGSILAFVLLLSPKMFHAFRNL
>P48505
MTSPAAAGNGLFKFLRPKLRPQSTDIQAAAGWGVAAVTGALWVIQPWDFLRKTFIEKQEE
EK
>Q8RY99
MGRLSWASPIQRFRFFSYLSQLNGRRSVLACSGYENRYLSSLVEASDCELDEVPDDRKVA
EKDTALHLALSQLSGDFDKDSKLSLQRFYRKRRVSVISTGSLNLDLALGVGGLPKGRMVE
VYGKEASGKTTLALHIIKEAQKLGGYCAYLDAENAMDPSLAESIGVNTEELLISRPSSAE
KMLNIVDVLTKSGSVDVIVVDSVAALAPQCELDAPVGERYRDTQSRIMTQALRKIHYSVG
YSQTLIVFLNQVRSHVKSNMHFPHAEEVTCGGNALPFHAAIRLKMIRTGLIKTANKISGL
NVCVQVVKNKLAPGKKKSELGIHFGHGFYVEREVLELACEHGVILREGTSYFIEGEVIEG
KDAAEKYLVENKEALDTVVAILRNQLFKM
>P92812
MRQSIKGRALRHFTLSTGKSAGRNSSGRITVFHRGGGSKRLQRKIDLKRSTSSIGIVERI
EYDPNRSSRIALVRWIEGVLPGRQRKFKTIEEFALPRKILESTTATIFCLFSFSSLSSPL
AQGETASLSFGSSLGFPRIAVAGAKPAFFAERMREKKIGKKTFSLCEIRKWRTHCVLWAH
RIKRKAALSWQSLRQQKTLELVGAAEHNESKLKADQGSLLPRQVLAYALCSGRPSYLHAS
RSFYKALLPVEASRFGSLPAKPPIGEGPKDGAYKVDRAPVTYILASHQLEAGNMVINCDC
SKPSKSGFLRPAQNAHTYLRFQELGRTVNKGRVEGGSQLAASWPRPPAYRHEILDLNSKV
GNSIPLADIRMGTWVHDIECHPGQGAKLARAAGTYAKIIKEPASQCLVRLPSGVEKLIDS
RCRATIGIVSNPNHGARKLRKAGQSRWSGRRPIVRGVAMNPVDHPHGGGEGRTKGGRPSV
SPWGKPTKAGFRAGVGVGKRRI
>Q9LDD8
MLRILGRRVVSASKELTSIQQWRIRPGTDSRPDPFRTFRGLQKGFCVGILPDGVDRNSEA
FSSNSIAMEGILSELRSHIKKVLAGGGEEAVKRNRSRNKLLPRERIDRLLDPGSSFLELS
QLAGHELYEEPLPSGGIITGIGPIHGRICMFMANDPTVKGGTYYPITIKKHLRAQEIAAR
CRLPCIYLVDSGGAYLPKQAEVFPDKENFGRVFYNESVMSSDGIPQIAIVLGSCTAGGAY
IPAMADESVMVKGNGTIFLAGPPLVKAATGEEVSAEDLGGATVHCTVSGVSDYFAQDELH
GLAIGRNIVKNLHMAAKQGMEGTFGSKNLVYKEPLYDINELRSIAPVDHKQQFDVRSIIA
RIVDGSEFDEFKKQYGTTLVTGFARIYGQTVGIIGNNGILFNESALKGAHFIELCSQRKI
PLVFLQNITGFMVGSRAEANGIAKAGAKMVMAVSCAKVPKITIITGASFGAGNYAMCGRA
YSPDFMFIWPNARIGIMGGAQAAGVLTQIERATKKRQGIKWTEEEEEAFKKKTVDAYERE
ANPYYSTARLWDDGVIDPCDTRKVLGLCLSAALNRPLEDTRFGVFRM
>P34899
MAMAMALRKLSSSVNKSSRPLFSASSLYYKSSLPDEAVYDKENPRVTWPKQLNSPLEVID

PEIADIIELEKARQWKGLELIPSENFTSLSVMQAVGSVMTNKYSEGYPGARYYGGNEYID
MAETLCQKRALEAFRLDPAKWGVNVQPLSGSPSNFQVYTALLKPHDRIMALDLPHGGHLS
HGYQTDTKKISAVSIFFETMPYRLDESTGYIDYDQLEKSATLFRPKLIVAGASAYARLYD
YARIRKVCDKQKAVLLADMAHISGLVAAGVIPSPFDYADVVTTTTHKSLRGPRGAMIFFR
KGLKEVNKQGKEVFYDYEDKINQAVFPGLQGGPHNHTITGLAVALKQATTPEYRAYQEQV
LSNSSKFAKALSEKGYDLVSGGTENHLVLVNLKNKGIDGSRVEKVLELVHIAANKNTVPG
DVSAMVPGGIRMGTPALTSRGFVEEDFVKVAEYFDAAVSLALKVKAESKGTKLKDFVEAL
QTSSYVQSEISKLKHDVEEFAKQFPTIGFEKATMKYNK
>P37221
MAIFSNQMRLSSTLLKRLHQRVAAAVNSSSSRNFTTTEGHRPTIVHKRSLDILHDPWFNK
GTAFSFTERDRLHIRGLLPPNVMSFEQQIARFMADLKRLEVQARDGPSDPYVLAKWRILN
RLHDRNETLYYKVLMENIEEYAPIVYTPTVGLVCQKYSGLFRRPRGMYFSAEDRGEMMSM
VYNWPADQVDMIVVTDGSRILGLGDLGIQGIGIAIGKLDLYVAAAGINPQRVLPVMIDVG
TDNENLLKDPLYLGLQDHRLDGEEYIEVIDEFMEAVFTRWPHVIVQFEDFQSKWAFKLLQ
RYRNNYRMFNDDIQGTAGVAIAGLLGAVRAQGRPMIDFPKMKIVVAGAGSAGIGVLNAAR
KTMARMLGNTEIAFESARSQFWVVDAKGLITEARENVDPDARPFARKIKEIERQGLSEGA
TLAEVVREVKPDVLLGLSACGGLFSKEVLEALKHSTSTRPAIFPMSNPTRNAECTPEEAF
SILGENIIFASGSPFKDVDLGNGHVGHCNQANNMFLFPGIGLGTLLSGSRIVSDGMLQAA
AECLAAYITEEEVLKGIIYPSISRIRDITKEVAAAVVKEAIEEDLAEGYREMDSRELRKL
DEAQISEFVENNMWSPDYPTLVYKKD
>P93834
MGKVAVATTVVCSVAVCAAAALIVRRRMKSAGKWARVIEILKAFEEDCATPIAKLRQVAD
AMTVEMHAGLASEGGSKLKMLISYVDNLPSGDETGFFYALDLGGTNFRVMRVLLGGKHDR
VVKREFKEESIPPHLMTGKSHELFDFIVDVLAKFVATEGEDFHLPPGRQRELGFTFSFPV
KQLSLSSGTLINWTKGFSIDDTVDKDVVGELVKAMERVGLDMLVAALVNDTIGTLAGGRY
TNPDVVVAVILGTGTNAAYVERAHAIPKWHGLLPKSGEMVINMEWGNFRSSHLPLTEYDH
SLDVDSLNPGEQILEKIISGMYLGEILRRVLLKMAEEAAFFGDIVPPKLKIPFIIRTPNM
SAMHSDTSPDLKVVGSKLKDILEVQTSSLKMRKVVISLCNIIASRGARLSAAGIYGILKK
IGRDATKDGEAQKSVIAMDGGLFEHYTQFSESMKSSLKELLGDEVSESVEVILSNDGSGV
GAALLAASHSQYLELEDDSETS
>P35480
MYRLISSIASKARVARNCTSQIGSRLSSTRNYAAKDIRFGVEGRALMLRGVEELADAVKV
TIPPKGRNVIIEQSWGAPKVTKDGVTVAKSIEFKDRVKNVGASLVKQVANRPTQLNRCLG
DGTTCATVLTRAIFTEGCKSVAAGMNAMDLRRGIKLAVDTVVTKLKSRARMISTSEEIAQ
VGTISANGDRELVTDCKAMESVGKEGVITIQDGKTLFNELEVVEGMKIDRGYISPYFITN
QKNQKCELEDPLILIHEKKISNLNSMVKVLELALKSQRSLLIVAADLESDALAVLILNKL
RAGIKVCAVKAPGFGENRKANMHDLATLTGAQVITEELGMNLEKIDLSMLGNCKKITVSK
DDTVFLGWGAGDKKAIGERCEQIRSMVEASESDYDKEKLQERLAKLSGGVAVLKIGGASE
SEVGEKKDRVTDALNATKAAVEEGIVPGGGVALLYASKELDKLSTANFDHKIGVQIIQNA
LKTPVYTIASNAGVEGAVIVGKLLESDNPDLGYDAAKGEYVDMVKSGIIDPVKVIRTALV
DAASVSSLLTTTEAVVTEIPTKEDASPAMGGGGGGMGGMGGMGGMGF
>P36428
MRLVKAASLLISSTKPPSRVFYSSHLRRPFFSHFRFSSSSSTSSSVAVMPGSEPSETQWP
AKRVRDTYVDFFRGKGHKFWPSSPVVPHNDPTLLFANAGMNQYKPIFLGTADPNTELSKL

SRACNTQKCIRAGGKHNDLDDVGKDTYHHTFFEMLGNWSFGDYFKKEAIEWAWELLTKVY
GLPTDRIYATYFGGDEKAGLQPDNEARDIWLKVLPSGRVLPFGCKDNFWEMGDTGPCGPC
TEIHYDRIGNRDAASLVNNDDPTCLEIWNLVFIQFNRESDGSLKPLPAKHVDTGMGFERL
TSVLQNKMSNYDTDVFMPIFDDIQKATGARPYSGKVGPEDVDRVDMAYRVVADHIRTLSF
AIADGSRPGNEGREYVLRRILRRAVRYGKEILKAEEGFFNGLVSSVIRVMGDVFTELKEH
EKKITDIIKEEEASFCKTLAKGIEKFRKAGQAVQGNTLSGDDAFILWDTYGFPLDLTQLM
AEERGLLVDVDGFNKAMEEARERSRSAQNKQAGGAIVMDADATSTLHKAGVSATDDSFKY
IWFQDHESELKAIYTGSTFLESSAASDNVGLVLGSTSFYAEQGGQIFDTGLIEGSFGTFN
VCNVQIFGGFVLHIGYLSKETGEVSVGDKVICKVDYERRKLIAPNHTCTHMLNYALKEVL
GDHIDQKGSIVLPEKLRFDFSHGKPVDPEDLRRIESIVNKQIKDELDVFSKEAVLSEAKR
IKGLRAVFGEVYPDPVRVVSIGRKVEDLLADPENNEWSLLSSEFCGGTHITNTREAKAFA
LLSEEGIAKGIRRVTAVTTECAFDALNAASLLEREVEDASRAEGSALEKKVSALKSRVDA
AIIPAAKKADIRTKIASLQNEVRKAQKKIAEQNLKKSVKLATEAAESAASDGKTFCIIQL
DVGLDAAAVREAVSKVMEKKGMSIMVFSTDESTNKAVVCAGVPEKSDQFKPLDVTEWLTT
ALGPLKGRCGKGKGGLASGQGTDASQVQAALDMASSFASMKLN

>P39697

MAFCTKLGGHWKQGVNVPVSSMLGSLRYMSTKLYIGGLSPGTDEHSLKDAFSSFNGVTEA
RVMTNKVTGRSRGYGFVNFISEDSANSAISAMNGQELNGFNISVNVAKDWPSLPLSLDES
IEEAEKKENKMMSRSVWKDPFVDAFLMKKKNAALNRKIWSRRSTILPEYVDSAVRIYNGK
THVRCKITEGKVGHKFGEFAFTRKVKKHAKAK

>Q940M2

MALQRQLLKRATSDIYHRRAISLLRTDFSTSPSIADAPPHIPPFVHQPRPYKGPSADEVL
QKRKKFLGPSLFHYYQKPLNIVEGKMQYLYDESGRRYLDAFAGIVTVSCGHCHPDILNAI
TEQSKLLQHATTIYLHHAIGDFAEALAAKMPGNLKVVYFVNSGSEANELAMMMARLYTGS
LEMISLRNAYHGGSSNTIGLTALNTWKYPLPQGEIHHVVNPDPYRGVFGSDGSLYAKDVH
DHIEYGTSGKVAGFIAETIQGVGGAVELAPGYLKSVYEIVRNAGGVCIADEVQTGFGRTG
SHYWGFQTQDVVPDIVTMAKGIGNGLPLGAVVTTPEIASVLASKILFNTFGGNPVCSAGG
LAVLNVIDKEKRQEHCAEVGSHLIQRLKDVQKRHDIIGDVRGRGLMVGIELVSDRKDKTP
AKAETSVLFEQLRELGILVGKGGLHGNVFRIKPPMCFTKDDADFLVDALDYSISRL

>Q9LQL0

MALRMWASSTANALKLSSSASKSHLLPAFSISRCFSSVLEGLKYANSHEWVKHEGSVATI
GITDHAQDHLGEVVFVELPEANSSVSKEKSFGAVESVKATSEILSPISGEVIEVNTKLTE
SPGLINSSPYEDGWMIKVKPSSPAELEALMGPKEYTKFCEEEDAAH

>Q94A28

MYRRATSGVRSASARLSSSLSRIASSETASVSAPSASSLRNQTNRSKSFSSALRSFRVCS
ASTRWSHGGSWGSPASLRAQARNSTPVMEKFERKYATMASEHSYKDILTSLPKPGGGEYG
KYYSLPALNDPRIDKLPFSVRILLESAIRNCDNYQVTKDDVEKILDWENTSTKQVEIAFK
PARVILQDFTGVPVLVDLASMRDAVKNLGSDPSKINPLVPVDLVVDHSIQVDFARSEDAA
QKNLELEFKRNKERFTFLKWGSTAFQNMLVVPPGSGIVHQVNLEYLGRVVFNSKGFLYPD
SVVGTDSHTTMIDGLGVAGWGVGGIEAEAAMLGQPMSMVLPGVVGFKLDGKLKEGVTATD
LVLTVTQILRKHGVVGKFVEFYGEGMSELSLADRATIANMSPEYGATMGFFPVDHVTLEY
LKLTGRSDETVSMIESYLRANNMFVDYNEPQQERAYTSYLQLELGHVEPCISGPKRPHDR
VPLKDMKADWHACLDNPVGFKGFAVPKEKQEEVVKFSYNGQPAEIKHGSVVIAAITSCTN
TSNPSVMIGAALVAKKASDLGLKVKPWVKTSLAPGSRVVEKYLDRSGLRESLTKQGFEIV

GYGCTTCIGNSGNLDPEVASAIEGTDIIPAAVLSGNRNFEGRVHPQTRANYLASPPLVVA
YALAGTVDIDFEKEPIGTRSDGKSVYLRDVWPSNEEVAQVVQYSVLPSMFKSSYETITEG
NPLWNELSAPSSTLYSWDPNSTYIHEPPYFKNMTANPPGPREVKDAYCLLNFGDSVTTDH
ISPAGNIQKTSPAAKFLMDRGVISEDFNSYGSRRGNDEVMARGTFANIRIVNKLLKGEVG
PNTVHIPTGEKLSVFDAASKYKTAEQDTIILAGAEYGSGSSRDWAAKGPLLLGVKAVIAK
SFERIHRSNLAGMGIIPLCFKAGEDAETLGLTGHERYTVHLPTKVSDIRPGQDVTVTTDS
GKSFVCTLRFDTEVELAYYDHGGILPYVIRSLSAK
>Q43725
MVAMIMASRFNREAKLASQILSTLLGNRSCYTSMAATSSSALLLNPLTSSSSSSTLRRFR
CSPEISSLSFSSASDFSLAMKRQSRSFADGSERDPSVVCEAVKRETGPDGLNIADNVSQL
IGKTPMVYLNSIAKGCVANIAAKLEIMEPCCSVKDRIGYSMVTDAEQKGFISPGKSVLVE
PTSGNTGIGLAFIAASRGYRLILTMPASMSMERRVLLKAFGAELVLTDPAKGMTGAVQKA
EEILKNTPDAYMLQQFDNPANPKIHYETTGPEIWDDTKGKVDIFVAGIGTGGTITGVGRF
IKEKNPKTQVIGVEPTESDILSGGKPGPHKIQGIGAGFIPKNLDQKIMDEVIAISSEEAI
ETAKQLALKEGLMVGISSGAAAAAAIKVAKRPENAGKLIAVVFPSFGERYLSTPLFQSIR
EEVEKMQPEV
>Q9SWG0
MQRFFSARSILGYAVKTRRRSFSSRSSSLLFDDTQLQFKESVSKFAQDNIAPHAERIDKT
NSFPKDVNLWKLMGEFNLHGITAPEEYGGLGLGYLYHCIAMEEISRASGSVALSYGAHSN
LCINQLVRNGTAAQKEKYLPKLISGEHVGALAMSEPNAGSDVVGMKCKAEKVDGGYILNG
NKMWCTNGPSAETLVVYAKTDTKAGSKGITAFIIEKGMTGFSTAQKLDKLGMRGSDTCEL
VFENCFVPEENILDKEGKGVYVLMSGLDLERLVLAAGPLGIMQACLDNVLPYIRQREQFG
RPVGEFQFIQGKVADMYTALQSSRSYVYSVARDCDNGKVDPKDCAGTILCAAERATQVAL
QAIQCLGGNGYINEYATGRLLRDAKLYEIGAGTSEIRRIVIGRELFKEE

(9) $\mathbb{S}_9$: 152 nucleus proteins

>P93045
MKGLVSTGWKGPVKFRMPTAENLVPIRLDIQFEGQRYKDAFTWNPSDPDNEVVIFAKRTV
KDLKLPYAFVTQIAQSIQSQLSDFRAYEGQDMYTGEKIIPIKLDLRVNHTLIKDQFLWDL
NNFESDPEEFARTLCKDLGVEDPEVGPAVAFAIREQLYEIAIQSVASARESRLSKKGRRG
SDHGSASKASGLSMDLMKLFSFKSSVVRKRKDLDVYEPVVDLLTSEEVDALEAREERHAR

>O22446
MDTGGNSLASGPDGVKRKVCYFYDPEVGNYYYGQGHPMKPHRIRMTHALLAHYGLLQHMQ
VLKPFPARDRDLCRFHADDYVSFLRSITPETQQDQIRQLKRFNVGEDCPVFDGLYSFCQT
YAGGSVGGSVKLNHGLCDIAINWAGGLHHAKKCEASGFCYVNDIVLAILELLKQHERVLY
VDIDIHHGDGVEEAFYATDRVMTVSFHKFGDYFPGTGHIQDIGYGSGKYYSLNVPLDDGI
DDESYHLLFKPIMGKVMEIFRPGAVVLQCGADSLSGDRLGCFNLSIKGHAECVKFMRSFN
VPLLLLGGGGYTIRNVARCWCYETGVALGVEVEDKMPEHEYYEYFGPDYTLHVAPSNMEN
KNSRQMLEEIRNDLLHNLSKLQHAPSVPFQERPPDTETPEVDEDQEDGDKRWDPDSDMDV
DDDRKPIPSRVKREAVEPDTKDKDGLKGIMERGKGCEVEVDESGSTKVTGVNPVGVEEAS
VKMEEEGTNKGGAEQAFPPKT
>P48513
MSDAFCSDCKRQTEVVFDHSAGDTVCSECGLVLESHSIDETSEWRTFANESGDNDPNRVG

GPSNPLLTDGGLSTVIAKPNGGGGGEFLSSSLGRWQNRGSNPDRALIQAFKTIATMSDRL
GLVATIKDRANEIYKRVEDQKSSRGRNQDALLAACLYIACRQEDKPRTVKEICSVANGAT
KKEIGRAKEYIVKQLGLENGNAVEMGTIHAGDFMRRFCSNLCMNNQAVKAAQEAVQKSEE
FDIRRSPISIAAAVIYIITQLSDDKKPLKDISLATGVAEGTIRNSYKDLYPHVSKIIPNW
YAKEEDLKNLCSP
>Q8VWG3
MESPPLYEISSSSSSEKPRHHFQSLDLFPNLNQNSCINNTLIEPLPLIDRINLNSNLDLN
PNPLYAEEGEQEEEEEEEEDREVDVDLHIGLPGFGKPSNDAKQLKKRNGKEIATYDAGKG
IENELSGKAYWIPAPEQILIGFTHFSCHVCFKTFNRYNNLQMHMWGHGSQYRKGPESLKG
TQPRAMLGIPCYCCVEGCRNHIDHPRSKPLKDFRTLQTHYKRKHGHKPFSCRLCGKLLAV
KGDWRTHEKNCGKRWVCVCGSDFKHKRSLKDHVKAFGSGHGPYPTGLFEEQASNSSVSET
LFF
>P48732
MFTSGNVTARVFERQIRTPPPGASVNRARHFYENLVPSYTLYDVESPDHCFRKFTEDGLF
LISFSRNHQELIVYRPSWLTYSTTDDSTTTLPPLPRRASKFDSFFTQLYSVNLASSNELI
CKDFFLYHQTRRFGLFATSTAQIHDSSSPSNDAVPGVPSIDKITFVLLRLDDGVVLDERV
FLHDFVNLAHNMGVFLYDDLLAILSLRYQRIHLLQIRDSGHLVDARAIGYFCREDDELFL
NSSSQAMMSQDKSKQQSLSGSKEDDTGENGLRHSLSQPSGSNSFLSGVKQRLLSFIFREI
WNEESDNRVQSLKKKFYFHFQDYVDLIIWKVQFLDRQHLLIKFGSVDGGVTRSADHHPAF
FAVYNMETTDIVAFYQNSAEDLYQLFEQFSDHFTVSSSTPFMNFVTSHSNNVYALEQLKY
TKNKSNSFSQFVKKMLLSLPFSCQSQSPSPYFDQSLFRFDEKLISAADRHRQSSDNPIKF
ISRRQPQTLKFKIKPGPECGTADGRSKKICSFLFHPHLPLAISIQQTLFMPPSVVNIHFR
R
>Q93WK5
MNANEEGEGSRYPITDRKTGETKFDRVESRTEKHSEEEKTNGITMDVRNGSSGGLQIPLS
QQTAATVCWERFLHVRTIRVLLVENDDCTRYIVTALLRNCSYEVVEASNGIQAWKVLEDL
NNHIDIVLTEVIMPYLSGIGLLCKILNHKSRRNIPVIMMSSHDSMGLVFKCLSKGAVDFL
VKPIRKNELKILWQHVWRRCQSSSGSGSESGTHQTQKSVKSKSIKKSDQDSGSSDENENG
SIGLNASDGSSDGSGAQSSWTKKAVDVDDSPRAVSLWDRVDSTCAQVVHSNPEFPSNQLV
APPAEKETQEHDDKFEDVTMGRDLEISIRRNCDLALEPKDEPLSKTTGIMRQDNSFEKSS
SKWKMKVGKGPLDLSSESPSSKQMHEDGGSSFKAMSSHLQDNREPEAPNTHLKTLDTNEA
SVKISEELMHVEHSSKRHRGTKDDGTLVRDDRNVLRRSEGSAFSRYNPASNANKISGGNL
GSTSLQDNNSQDLIKKTEAAYDCHSNMNESLPHNHRSHVGSNNFDMSSTTENNAFTKPGA
PKVSSAGSSSVKHSSFQPLPCDHHNNHASYNLVHVAERKKLPPQCGSSNVYNETIEGNNN
TVNYSVNGSVSGSGHGSNGPYGSSNGMNAGGMNMGSDNGAGKNGNGDGSGSGSGSGSGNL
ADENKISQREAALTKFRQKRKERCFRKKVRYQSRKKLAEQRPRVRGQFVRKTAAATDDND
IKNIEDS
>P26307
MEASSGSSPPHSQENPPEHGGDMGGAPAEEIGGEAADDFMFAEDTFPSLPDFPCLSSPSS
STFSSNSSSNSSSAYTNTAGRAGGEPSEPASAGEGFDALDDIDQLLDFASLSMPWDSEPF
PGVSMMLENAMSAPPQPVGDGMSEEKAVPEGTTGGEEACMDASEGEELPRFFMEWLTSNR
ENISAEDLRGIRLRRSTIEAAAARLGGGRQGTMQLLKLILTWVQNHHLQRKRPRDVMEEE
AGLHVQLPSPVANPPGYEFPAGGQDMAAGGGTSWMPHQQAFTPPAAYGGDAVYPSAAGQQ
YSFHQGPSTSSVVVNSQPFSPPPVGDMHGANMAWPQQYVPFPPPGASTGSYPMPQPFSPG

FGGQYAGAGAGHLSVAPQRMAGVEASATKEARKKRMARQRRLSCLQQQRSQQLSLGQIQT
SVHLQEPSPRSTHSGPVTPSAGGWGFWSPSSQQQVQNPLSKSNSSRAPPSSLEAAAAAPQ
TKPAPAGARQDDIHHRLAAASDKRQGAKADKNLRFLLQKVLKQSDVGSLGRIVLPKKEAE
VHLPELKTRDGISIPMEDIGTSRVWNMRYRFWPNNKSRMYLLENTGEFVRSNELQEGDFI
VIYSDVKSGKYLIRGVKVRPPPAQEQGSGSSGGGKHRPLCPAGPERAAAAGAPEDAVVDG
VSGACKGRSPEGVRRVRQQGAGAMSQMAVSI
>Q4QSC8
MTSTAAEGAGSGSLNPPHSNPSGDGGPRIRSPPGKGNKPVALADITNTGKPNAARSITVP
DLVKENTKLLTLLNEKTKIIDLSRVEIYKLRLALQASKQQNLHLTQTNSQMLAEINTGKD
RIKMLQHELSCTTALLKVKDSELDRKKNAGNVQQKGVKSQVLKTKASTVAVEAHHVGDSV
TSGVEHHVVESQSAVSSNTVCQEPPQDGKQKRMPQRRRSSRLNQGSCEIRGVSQNTLHEN
PVVPVAPSTLSLEKQYGQTTGKHMKSLQNECSATVHEVIMASEFEKTEINELPQKTDLKE
IPEACSSETEVQSHKIGDKAFNSKQNHLTGSQSSLSFNTVDTPEPPEDNTVKRCSKKRSS
IEDVNAKLDTITSEPLRHEKKRKSRRKISARLNSVSSEHTDIVVETEHKDVIVSLAGSTS
NVSMEQRTNQEQDGDCFSRKSNENQILGRRSLRRAAEKVVSYKEMPLNVKMRRP
>Q8GWZ6
METTVKNSSSDGEWKVVLPSKGRQGRRRKPKPKGQAEEEEQPWKSDDLEIDPQRQARLKQ
KMEISLKKIESSSFYTAFLEQLKSPEVSNQIRLVLGSETQLQMVMYGIGSIESYESPRFQ
LSIAILMKREFDWVGDNIEVFDPVLSATESSYLESLGCSVLSVNEQARREALKPTLFFMP
HCEANLYSNLLQANWRMDRLSKIALFGNSFQMYEEQVSFDAEVICATKRIIAAQRVTSEF
AIETESDDYFAAFHDSSWHFFSSGIDSELPLFVSD
>O22161
MSRRVRRKLEEEKGKDKVVVLPSYPETSISNEEDLVAPELLHGFVDWISLPYDTVLQLFT
CLNYRDRASLASTCKTWRCLGASSCLWTSLDLRPHKFDASMAASLASRCVNLHYLRFRGV
ESADSLIHLKARNLIEVSGDYCKKITDATLSMIVARHEALESLQLGPDFCERITSDAIKA
VAFCCPKLKKLRLSGIRDVTSEAIEALAKHCPQLNDLGFLDCLNIDEEALGKVVSVRYLS
VAGTSNIKWSIASNNWDKLPKLTGLDVSRTDIGPTAVSRFLTSSQSLKVLCALNCHVLEE
DESLISYNRFKGKVLLALFTNVFDGLASIFADNTKKPKDIFAYWRELMKTTKDKTINDFI
HWIEWIISHTLLRTAECNPEGLDDFWLNEGAALLLNLMQSSQEDVQERSATGLATFVVVD
DENASIDCGRAEAVMKDGGIRLLLELAKSWREGLQSEAAKAIANLSVNANIAKSVAEEGG
IKILAGLAKSMNRLVAEEAAGGLWNLSVGEEHKNAIAQAGGVKALVDLIFRWPNGCDGVL
ERAAGALANLAADDKCSMEVAKAGGVHALVMLARNCKYEGVQEQAARALANLAAHGDSNN
NNAAVGQEAGALEALVQLTKSPHEGVRQEAAGALWNLSFDDKNRESISVAGGVEALVALA
QSCSNASTGLQERAAGALWGLSVSEANSVAIGREGGVPPLIALARSEAEDVHETAAGALW
NLAFNPGNALRIVEEGGVPALVHLCSSSVSKMARFMAALALAYMFDGRMDEYALMIGTSS
SESTSKNISLDGARNMALKHIEAFVLSFIDPHIFESPVVSSTPTMLAQVTERARIQEAGH
LRCSGAEIGRFVTMLRNPDSTLKACAAFALLQFTIPGGRHAMHHVSLMQNGGESRFLRSA
AASAKTPREAKIFTKILLRNLEHHQAESSI
>Q9LRY0
MGKYIKKSKVAGAVSVKDKSHPPALGFRTRAAAAKNLALHRLRSHSDEADSFNYLQLRSR
RLVKLPLLTNTRKQQKQQLIPSVNQCQTKNPRASSGPAKKLEPDTTTEEACGDNERISRS
DCNFGDKGFDLESENRSMISDSKSIQSEIEDFFASAEQQQQRFFIQKYNFDIVSDNPLPG
RYEWVKVMP
>Q8LJT8

MLKQFTHYCEMQAELIPEGPNGEGRLSNQNSNPNLLSSASISITQFPAKKPTRQWAAWTH
QEEESFFTALRQVGKNFEKITSRVQSKNKDQVRHYYYRLVRRMNKLLGPDLSLDAKNPKD
TNAAMLRWWSLLEKYSCKASKLHLKPRRFKLFIEALEHQLLKDRRKSIRKRTCQGENLSS
ASLGNISSHSRERGLDNRPFKLILSDGQNVKKLGPGRASTKHGESLSVNLGDEKEDTAFG
RGGRQRRKQGYRKWEKAAIDGVSLVADAAEHLERTSIDKDMDDQTDLGPTRYLTGKSPLS
LCSAGDVPLSDANMQFSAKLKLQLFPIDECTRRSLEMDKHNPHLELTLSNRKKISSVLEH
LNRKWGSSSCATGELLLFPYNARKETVTCHQRWTHDSFLSAAEVHSMVGSPSVFRLRYGW
FVHDASGSIISQVPTSDPCPSLEDDMNVDRLNEVNMLLTESGPLSVHSTAEQTTSVEPSQ
GLVCASGVHDRPARSRDDYEPASTSITPLEHLSGGNAQSPGEWADSLTNISIGDLLSEVP
DDIDSDGVDPPATEGSHYLLRDVPFTSDSFDAAIAAHILRHQNKPSAQLPLTSGSSSLWD
DEETRDAFSFQKNRFANSTELASVASPKGVGRVNGEPSQLVEASSGDEGSYNPHDDGDPM
EEGPADPHTMDSPGKTPCGLADVYWPDSLGPLDLDIRSSKYTDDLILSESLGGLSRLIAT
SLDAFQNCSLFGFDNKKDKSNMV
>Q94JU3
MDIEQKQAEIIDQLVKRASTCKSEALGPLIIEATSHPSLFAFSEILALPNVAQLEGTTDS
VYLDLLRLFAHGTWGDYKCNATRLPHLSPDQILKLKQLTVLTLAESNKVLPYDTLMVELD
VSNVRELEDFLINECMYAGIVRGKLDQLKRCFEVPFAAGRDLRPGQLGNMLHTLSNWLNT
SENLLISIQDKIKWADNMSEMDKKHRKEAEEGVEEVKKSLSMKGDVDIRGNKEMFGEPSG
VMDYEEDGIRPKRRRHPVTR
>P43299
MKDHDFDGDKGLAKGFLENFADANGRSKYMEILQEVSNRKIRAIQVDLDDLFNYKDESEE
FLGRLTENTRRYVSIFSAAVDELLPEPTEAFPDDDHDILMTQRADDGTDNPDVSDPHQQI
PSEIKRYYEVYFKAPSKGRPSTIREVKASHIGQLVRISGIVTRCSDVKPLMAVAVYTCED
CGHEIYQEVTSRVFMPLFKCPSSRCRLNSKAGNPILQLRASKFLKFQEAKMQELAEHVPK
GHIPRSMTVHLRGELTRKVSPGDVVEFSGIFLPIPYTGFKALRAGLVADTYLEATSVTHF
KKKYEEYEFQKDEEEQIARLAEDGDIYNKLSRSLAPEIYGHEDIKKALLLLLVGAPHRQL
KDGMKIRGDVHICLMGDPGVAKSQLLKHIINVAPRGVYTTGKGSSGVGLTAAVMRDQVTN
EMVLEGGALVLADMGICAIDEFDKMDESDRTAIHEVMEQQTVSIAKAGITTSLNARTAVL
AAANPAWGRYDLRRTPAENINLPPALLSRFDLLWLILDRADMDSDLELAKHVLHVHQTEE
SPALGFEPLEPNILRAYISAARRLSPYVPAELEEYIATAYSSIRQEEAKSNTPHSYTTVR
TLLSILRISAALARLRFSESVAQSDVDEALRLMQMSKISLYADDRQKAGLDAISDTYSII
RDEAARSKKTHVSYANALNWISRKGYSEAQLKECLEEYAALNVWQIDPHTFDIRFI
>A1YKT1
MNNNIFSTTTTINDDYMLFPYNDHYSSQPLLPFSPSSSINDILIHSTSNTSNNHLDHHHQ
FQQPSPFSHFEFAPDCALLTSFHPENNGHDDNQTIPNDNHHPSLHFPLNNTIVEQPTEPS
ETINLIEDSQRISTSQDPKMKKAKKPSRTDRHSKIKTAKGTRDRRMRLSLDVAKELFGLQ
DMLGFDKASKTVEWLLTQAKPEIIKIATTLSHHGCFSSGDESHIRPVLGSMDTSSDLCEL
ASMWTVDDRGSNTNTTETRGNKVDGRSMRGKRKRPEPRTPILKKLSKEERAKARERAKGR
TMEKMMMKMKGRSQLVKVVEEDAHDHGEIIKNNNRSQVNRSSFEMTHCEDKIEELCKNDR
FAVCNEFIMNKKDHISNESYDLVNYKPNSSFPVINHHRSQGAANSIEQHQFTDLHYSFGA
KPRDLMHNYQNMY
>Q6L545
MAGSDEVNRNECKTVVPLHTWVLISNFKLSYNILRRADGTFERDLGEYLDRRVPANARPL
EGVSSFDHIIDQSVGLEVRIYRAAAEGDAEEGAAAVTRPILEFLTDAPAAEPFPVIIFFH

GGSFVHSSASSTIYDSLCRRFVKLSKGVVVSVNYRRAPEHRYPCAYDDGWTALKWVMSQP
FMRSGGDAQARVFLSGDSSGGNIAHHVAVRAADEGVKVCGNILLNAMFGGTERTESERRL
DGKYFVTLQDRDWYWKAYLPEDADRDHPACNPFGPNGRRLGGLPFAKSLIIVSGLDLTCD
RQLAYADALREDGHHVKVVQCENATVGFYLLPNTVHYHEVMEEISDFLNANLYY
>Q06364
MTQDVEMKEVPAPAPSNSVTAATPSTLQHLKEIASLIESGAYAREVRRILRAVRLTIALR
KKLNASVVNAFLNFSLVPGSEVHARLASYLPKEDEHDMEVDTAMSATTTLAKHSLPELEI
YCYLLVLIFLIDQKKYSEAKACSSASIARVKNLNRRTVEVLASRLYFYYSLSYELTGDLA
EIRGNLLALHRIATLRHDELGQETLLNLLLRNYLHYNLYDQAEKLRSKAPRFEAHSNQQF
CRYLFYLGKIRTIQLEYTDAKESXLQAARKAPVAALGFRVQCNKWAVIVRLLLGEIPERT
VFMQKGMEKALRPYFXLTNAVRIGDLELFRXVADKFASTFTADRTHNLIVRLRHNVIRTG
LRNISISYSRISLVDVARKLRLDSPNPVADAESIVSKAIRDGAIDATIDHANGWMVSKET
GDIYSTNEPQAAFNSRIAFCLNMHNEAVRALRFPANSHKDKESAEKRRERQQQEQELAKH
IAEEDDDEF
>Q9LX99
MADQRSKTNRWNWEVSGFEPRKSSSNASFAESTGHRTTGPLLRRNSISTPSLPPKQAIAS
KVNGLKEKVKLAKEDYLELRQEATDLQEYSNAKLDRVTRYLGVLAEKSRKLDQFVLETEA
RISPLINEKKRLFNDLLTAKGNIKVFCRARPLFEDEGPSVIEFPGDCTICVNTSDDTLSN
PKKDFEFDRVYGPHVGQAALFSDVQPFVQSALDGSNVSILSYGQTNAGKTYTMEGSNHDR
GLYARCFEELFDLANSDSTSTSRFSFSLSVFEIYNEQIRDLLSETQSNLPNINMDLHESV
IELGQEKVDNPLEFLGVLKSAFLNRGNYKSKFNVTHLIVSIHIYYSNTITGENIYSKLSL
VDLAGSEGLIMENDSGDHVTDLLHVMNSISALGDVLSSLTSGKDSIPYDNSILTRVLADS
LGGSSKTLMIVNICPSVQTLSETISCLNYAARARNTVPSLGNRDTIKKWRDVASDARKEL
LEKERENQNLKQEVVGLKKALKDANDQCVLLYSEVQRAWKVSFTLQSDLKSENIMLVDKH
RLEKEQNSQLRNQIAQFLQLDQEQKLQMQQQDSAIQNLQAKITDLESQVSEAVRSDTTRT
GDALQSQDIFSPIPKAVEGTTDSSSVTKKLEEELKKRDALIERLHEENEKLFDRLTERSM
AVSTQVLSPSLRASPNIQPANVNRGEGYSAEAVALPSTPNKNNGAITLVKSGTDLVKTTP
AGEYLTAALNDFDPEEYEGLAAIADGANKLLMLVLAAVIKAGASREHEILAEIRDSVFSF
IRKMEPRRVMDTMLVSRVRILYIRSLLARSPELQTIRVSPVECFLEKPNTGRSKSTSRGS
SPGRSPVRYLDTQIHGFKVNIKAERRNKLASVVSRMRGLEQDAGRQQVTGVKLREMQDEA
KSFAIGNKALAALFVHTPAGELQRQIRLWLAENFEFLSVTSDDVSGGNGGQLELLSTAIM
DGWMAGLGAAVPPHTDALGQLLSEYAKRVYTSQMQHMKDIAGTLAAEEAEDAGQVSKLRS
ALESVDHKRRKILQQMKSDAALLNLEEGSSPIPNPSTAAEDSRLASLISLDGILKQVKEI
TRQASVHVLSKSKKKALLESLDELTERMPSLLDIDHPCAQREIATAHQLVETIPEQEDTN
ILEQSHDRRPSLESISSGETDVSQWNVLQFNTGSSAPFIIKCGGNNNSELVIKADARVQE
PKGGEIVRVVPRPSVLVNMSLEEMKQMFVQLPEALSLLALARTADGTRARYSRLYKTLAM
KVPSLKDLVSELE
>Q38847
MGRGKIEIKRIENANSRQVTFSKRRSGLLKKARELSVLCDAEVAVIVFSKSGKLFEYSST
GMKQTLSRYGNHQSSSASKAEEDCAEVDILKDQLSKLQEKHLQLQGKGLNPLTFKELQSL
EQQLYHALITVRERKERLLTNQLEESRLKEQRAELENETLRRQVQELRSFLPSFTHYVPS
YIKCFAIDPKNALINHDSKCSLQNTDSDTTLQLGLPGEAHDRRTNEGERESPSSDSVTTN
TSSETAERGDQSSLANSPPEAKRQRFSV
>Q40478

MASPQENCTTLDLIRQHLLDDNVFMEHYCPQPILYSQSSSSSESLNSIASELNNETFSFE
PTLKYADTAQSSNLDISSFFNNSKTEFDSFEFETKPNVSAARISSNSPKQTSFKERKPSL
NIAIPMKQQEVVQKVEVVPTEKKHYRGVRQRPWGKFAAEIRDPNRKGTRVWLGTFDTAIE
AAKAYDRAAFKLRGSKAIVNFPLEVANFKQQDNEILQPANSGRKRMRETENEEIVIKKEV
KREERVPAAAAPLTPSSWSAIWEGEDGKGIFEVPPLSPLSPHMAYSQLVMI
>Q00265
MLELRLVQGGLLKKVLESIKDLVNDANFDCSASGFSLQAMDSSHVALVAVLLRSEGFEHY
RCDRNISMGMNLGNMAKMLRCAGNDDIVTMKADDDGDVITFMFESPTQDKISDFEMKLMD
IDSEHLGIPESEYEAIVRMPSAEFARICKDLSTIGDTVVISVTKEGVKFSTRGDIGTANI
VCRQNTSVDKPEDATIIEMQETVSLTFALRYMNSFTKATPLANQVTISLSSELPVVVEYK
IAEMGYIRYYLAPKIEEEDEAANYAQPAQNSAAAATSNNGTKKNEGNNKVDSKKRAIKSE
FVDDSEAATDAQPQAKAKTKTEAGEDDDVEVMDTKPKNEPDDGDEVMETKPKTESNGEVE
VMDIE
>Q4VYC8
MTMEPNPTSDHILDWLEGSVSFFPSFLDDPYNNGYIHEYEIWNQNQDISNQYQIDANTNS
SNATNSTTNIVAASTTTSTTSLEPNSFNNIPFSDLPKKRNAEDELSLKKQPQNQKNKRLK
SRPMNESDNGDAALEGTVVRKSGGNKKGAAKANGSNSNNGNNKDGRWAEQLLNPCAVAIT
GGNLNRVQHLLYVLHELASTTGDANHRLAAHGLRALTHHLSSSSSSTPSGTITFASTEPR
FFQKSLLKFYEFSPWFSFPNNIANASILQVLAEEPNNLRTLHILDIGVSHGVQWPTFLEA
LSRRPGGPPPLVRLTVVNASSSTENDQNMETPFSIGPCGDTFSSGLLGYAQSLNVNLQIK
KLDNHPLQTLNAKSVDTSSDETLIVCAQFRLHHLNHNNPDERSEFLKVLRGMEPKGVILS
ENNMECCCSSCGDFATGFSRRVEYLWRFLDSTSSAFKNRDSDERKMMEGEAAKALTNQRE
MNERREKWCERMKEAGFAGEVFGEDAIDGGRALLRKYDNNWEMKVEENSTSVELWWKSQP
VSFCSLWKLDKQPE
>Q94CL9
MSETKPKRDSEYEGSNIKRMRLDDDDDVLRSPTRTLSSSSSSSLAYSVSDSGGFCSVALS
EEEDDHLSSSISSGCSSSETNEIATRLPFSDLEAHEISETEISTLLTNNFRKQGISSSEN
LGETAEMDSATTEMRDQRKTEKKKKMEKSPTQAELDDFFSAAERYEQKRFTEKYNYDIVN
DTPLEGRYQWVSLKP
>Q2QYF3
MGSSSLLLFPSSSSSATHSSYSPSSSSHAITSLLPPLPSDHHLLLYLDHQEQHHLAAAMV
RKRPASDMDLPPPRRHVTGDLSDVTAAAAGAPTLSASAQLPALPTQLPAFHHTDMDLAAP
APPAPQQVAAGEGGPPSTAWVDGIIRDIIASSGAAVSVAQLIHNVREIIRPCNPDLASIL
ELRLRSLLNSDPAPPPPPPPSHPALLPPDATAPPPPPTSVAALPPPPPAQPDKRRREPQCQ
EQEPNQPQSPKPPTAEETAAAAAAAAAAAAAAAKERKEEQRRKQRDEEGLHLLTLLLQCA
ESVNADNLDEAHRALLEIAELATPFGTSTQRVAAYFAEAMSARLVSSCLGLYAPLPSPSP
AGARVHGRVAAAFQVFNGISPFVKFSHFTANQAIQEAFEREERVHIIDLDIMQGLQWPGL
FHILASRPGGPPRVRLTGLGASMEALEATGKRLSDFADTLGLPFEFCPVADKAGNLDPEK
LGVTRREAVAVHWLRHSLYDVTGSDSNTLWLIQRLAPKVVTMVEQDLSHSGSFLARFVEA
IHYYSALFDSLDASYSEDSPERHVVEQQLLSREIRNVLAVGGPARTGDVKFGSWREKLAQ
SGFRVSSLAGSAAAQAALLLGMFPSDGYTLIEENGALKLGWKDLCLLTASAWRPIQASGR

>Q9LEK8
MSSEKVRASHILIKHQGSRRKSSWKDPDGSLISATTRDDAVSQLQSLRQELLSDPASFSD

LASRHSHCSSAKRGGDLGPFGRGQMQKPFEEATFALKVGEISDIVDTDSGVHIIKRTG
>Q9LJD7
MMTPGGSGRLRPLPTAMYAGYSGTASSWVAKTSVSASGKRIQREMAELNIDPPPDCSAGP
KGDNLYHWIATIIGPSGTPYEGGIFFLDIIFPSDYPFKPPKLVFKTRIYHCNVDTAGDLS
VNILRDSWSPALTITKVLQAIRSIFLKPEPYSPALPVIARLYLTDREKHDEVAKEWTLRF
AK
>Q39211
MDGATYQRFPKIKIRELKDDYAKFELRETDVSMANALRRVMISEVPTVAIDLVEIEVNSS
VLNDEFIAHRLGLIPLTSERAMSMRFSRDCDACDGDGQCEFCSVEFRLSSKCVTDQTLDV
TSRDLYSADPTVTPVDFTIDSSVSDSSEHKGIIIVKLRRGQELKLRAIARKGIGKDHAKW
SPAATVTFMYEPDIIINEDMMDTLSDEEKIDLIESSPTKVFGMDPVTRQVVVVDPEAYTY
DEEVIKKAEAMGKPGLIEISPKDDSFIFTVESTGAVKASQLVLNAIDLLKQKLDAVRLSD
DTVEADDQFGELGAHMRGG
>Q8W5S1
MADFQTSTQRAKWIFTPQKLAERYKAANQRAVQMLEKCGTTQVEVDASGSLTYPKDKVGS
GDQADKKLKPLSADEERFMRAFYEAKVQEVCSAFAFPHKIQATALQYFKRFYLQWSVMQH
HPKEIMLTCVYAACKIEENHVSAEEIGKGINQDHRIILKYEMAVLQSLEFDLIVYAPYRA
IEGFVNNMEEFLQARDDEIQKLESLLKGATAEADKVMLTDAPLLFPPGQLALASLRIANG
VLGVIDFDRYLENIVSQPNSEHTTSELTKLLDNIEYLVKNYKCPSEKDMKHINRKLKSCL
GHSSSHDESKKREKRSKHKSHRSSNDTPNGAPPPIG
>P52914
MELLIKLITFLLFSMPAITSSQYLGNNLLTSRKIFLKQEEISSYAVVFDAGSTGSRIHVY
HFNQNLDLLHIGKGVEYYNKITPGLSSYANNPEQAAKSLIPLLEQAEDVVPDDLQPKTPV
RLGATAGLRLLNGDASEKILQSVRDMLSNRSTFNVQPDAVSIIDGTQEGSYLWVTVNYAL
GNLGKKYTKTVGVIDLGGGSVQMAYAVSKKTAKNAPKVADGDDPYIKKVVLKGIPYDLYV
HSYLHFGREASRAEILKLTPRSPNPCLLAGFNGIYTYSGEEFKATAYTSGANFNKCKNTI
RKALKLNYPCPYQNCTFGGIWNGGGGNGQKNLFASSSFFYLPEDTGMVDASTPNFILRPV
DIETKAKEACALNFEDAKSTYPFLDKKNVASYVCMDLIYQYVLLVDGFGLDPLQKITSGK
EIEYQDAIVEAAWPLGNAVEAISALPKFERLMYFV
>P93203
MATSCFPPFSASSSSLCSSQFTPLLSCPRNTQICRKKRPVMASMHSENQKESNVCNRRSI
LFVGFSVLPLLNLRARALEGLSTDSQAQPQKEETEQTIQGSAGNPFVSLLNGLGVVGSGV
LGSLYALARNEKAVSDATIESMKNKLKDKEDAFVSMKKQFESELLSEREDRNKLIRREGE
ERQALVNQLKSAKTTVISLGQELQNEKKLAEDLKFEIKGLQNDLMNTKEDKKKLQEELKE
KLDLIQVLEEKITLLTTEIKDKEVSLRSNTSKLAEKESEVNSLSDMYQQSQDQLMNLTSE
IKELKDEIQKRERELELKCVSEDNLNVQLNSLLLERDESKKELHAIQKEYSEFKSNSDEK
VASDATLGEQEKRLHQLEEQLGTALSEASKNEVLIADLTREKENLRRMVDAELDNVNKLK
QEIEVTQESLENSRSEVSDITVQLEQLRDLSSKLEREVSKLQMELEETRASLQRNIDETK
HSSELLAAELTTTKELLKKTNEEMHTMSDELVAVSENRDSLQTELVNVYKKREHTRNELK
QEKTIVRTLEEELKFLESQITREKELRKSLEDELEKATESLDEINRNVLALAEELELATS
RNSSLEDEREVHRQSVSEQKQISQEAQENLEDAHSLVMKLGKERESLEKRAKKLEDEMAA
AKGEILRLRSQINSVKAPVEDEEKVVAGEKEKVNVQQ
>O22607
MESDEAAAVSPQATTPSGGTGASGPKKRGRKPKTKEDSQTPSSQQQSDVKMKESGKKTQQ

SPSVDEKYSQWKGLVPILYDWLANHNLVWPSLSCRWGPQLEQATYKNRQRLYLSEQTDGS
VPNTLVIANCEVVKPRVAAAEHISQFNEEARSPFVKKYKTIIHPGEVNRIRELPQNSKIV
ATHTDSPDVLIWDVETQPNRHAVLGAANSRPDLILTGHQDNAEFALAMCPTEPFVLSGGK
DKSVVLWSIQDHITTIGTDSKSSGSIIKQTGEGTDKNESPTVGPRGVYHGHEDTVEDVAF
SPTSAQEFCSVGDDSCLILWDARTGTNPVTKVEKAHDADLHCVDWNPHDDNLILTGSADN
TVRLFDRRKLTANGVGSPIYKFEGHKAAVLCVQWSPDKSSVFGSSAEDGLLNIWDYDRVS
KKSDRAAKSPAGLFFQHAGHRDKVVDFHWNASDPWTIVSVSDDCETTGGGGTLQIWRMSD
LIYRPEEEVVAELEKFKSHVMTCASKP
>O82491
MADSRNGNARAPPSGVPPKAGNTYSIDVKNFISRARALYEHWKKHSADLWGSADALAIAT
PPASDDLRYLKSSALNIWLLGYEFPDTIMVFTKKQIHFLCSRNKASLLEVVKKPAHDELK
LDVIMHVKPKGDDGTGLMDAIFRAIRDLSRGDGNDSQVVGHIAREAPEGKLLETWTERLK
NANFQFVDITGGLSDLFAVKDDTEVMSVKKAAYLAYSVMKNVVVPNLESAIDEEKDVTHS
ALMDLTEKAILEPTKASVKLKPENVDICYPPIFQSGGKFDLKPSAASNDELLTYDPASII
ICAVGARYNSYCSNVARTYLIDATSLQSKAYEVLLKAHEAAIDALRSGRKINTVYQAALS
VVEKNAPEFVDKLTKSAGTGIGLEFRESGLNINAKNDKVLRPKMAFNVSLGFQNLECESE
SRSKNKKFSLLLADTVLVTDQKPELLTKCSKSVKDVAYSFKEDEEEEKPRKKARTSGSEN
YITKTALRSDDHVVSKEELRKQHQAELARQKNEETARRLAGDSSGAGDSRSTAKTSADVV
AYKNVNDMPHKELMIQVDTRNEAVLLPIYGSLVPFHVATIRTVSGNQDTNRNCYIRIIFN
VPGTPFNPHDSNSLKNQGAIYLKEVSFRTKDSRHSSEVTQQIKTLRRQVMARESERAERA
TLVTQEKLQLAGNKFKPLRLSELWIRPPFSGRKKIPGTLEAHANGFRYSTTRPDERVDVL
FANIKHAFFQPAEKEMITLLHFHLHNHIMVGTKKTKDVQFYVEVMDVVQSLGGGRRSAYD
PDEIDEEQRERDRKNKINMDFNHFANRVNDMWQLPQFASLDLEFDQPLRELGFHGVPHKT
SAFIIPTSSCLVELIEYPFLVVSLSEIEIVNLERVGFGQKNFDMAIIFKDFKKDVLRVDS
VPTSSLEGIKEWLDTTDIKYYESKLNLNWRQILKTITDDPQSFIDDGGWEFLNLDGSDSE
SGGSEESDKGYEPSDVEVESESEDEASESESLVESDDDEEEDSEQESEEEKGKTWDELER
EATNADREHGVESDSEEERKRRKMKAFGKSRPGTSGGGGSSSMKNMPPSKRKHR
>Q84M92
MYGGDEVSAIVVDLGSHTCKAGYAGEDAPKAVFPSVIGAVDGVEAMDVDVDSTKTNSNSE
DSKTESEKEKSKRKLYVGSQAMSYRRDHMEVLSPIKDGIVSDWDLVDNIWEHAFKSCLMI
DPTEHPMLLAEPPLNTQQQREKAAELMFEKYKVPALFMAKNPVLTSFATGRATSLVVDCG
GGSTTISPVHDGYVLQKAVVSSPLGGEFLTDCLLKSLESKGIKIRPRYSFKRKEVRAGEF
QVEDVDIPDTTESYKLFCQRMIVGDIKDSICRVPDTPYDDKSYSNIPTTSYELPDGQTLE
IGADRFKVPDVMFNPSIVQTIPGMEKYAEMIPSVRGLPHMVMESINKCDVDIRRELYSSI
LLAGGTSSMQQLKERLEKDLIEESPHSARVKVLASGNTTERRFSVWIGGSILASLGSFQQ
MWFSKSEYEEHGASYIQRKCP
>Q9XG57
MARMKHTARMSTGGKAPRKQLASKALRKAPPPPTKGVKQPTTTTSGKWRFARFHRKLPFQ
GLVRKIWQDLKTHLRFKNHSVPPLEEVTEVYPCQTIGGCY
>Q7G7J6
MKREYQEAGGSSGGGSSADMGSCKDKVMAGAAGEEEDVDELLAALGYKVRSSDMADVAQK
LEQLEMAMGMGGVSAPGAADDGFVSHLATDTVHYNPSDLSSWVESMLSELNAPLPPIPPA
PPAARHASTSSTVTGGGGSGFFELPAAADSSSSTYALRPISLPVVATADPSAADSARDTK
RMRTGGGSTSSSSSSSSSLGGGASRGSVVEAAPPATQGAAAANAPAVPVVVVDTQEAGIR

LVHALLACAEAVQQENFAAAEALVKQIPTLAASQGGAMRKVAAYFGEALARRVYRFRPAD
STLLDAAFADLLHAHFYESCPYLKFAHFTANQAILEAFAGCHRVHVVDFGIKQGMQWPAL
LQALALRPGGPPSFRLTGVGPPQPDETDALQQVGWKLAQFAHTIRVDFQYRGLVAATLAD
LEPFMLQPEGEADANEEPEVIAVNSVFELHRLLAQPGALEKVLGTVHAVRPRIVTVVEQE
ANHNSGSFLDRFTESLHYYSTMFDSLEGGSSGQAELSPPAAGGGGGTDQVMSEVYLGRQI
CNVVACEGAERTERHETLGQWRNRLGRAGFEPVHLGSNAYKQASTLLALFAGGDGYRVEE
KEGCLTLGWHTRPLIATSAWRVAAA
>Q9FVL0
MGTLDTKGFTEEQEALVVKSWNAMKKNSAELGLKLFLKIFEIAPSAQKLFSFLKDSKVPL
EQNTKLKPHAMSVFLMTCESAVQLRKSGKVTVRESSLKKLGANHFKYGVVDEHFEVTKFA
LLETIKEAVPEMWSPAMKNAWGEAYDQLVNAIKSEMKPSS
>Q9M0V3
MSSWNYVVTAHKPTSVTHSCVGNFTSPQELNLIVAKCTRIEIHLLTPQGLQPMLDVPIYG
RIATLELFRPHGEAQDFLFIATERYKFCVLQWDPESSELITRAMGDVSDRIGRPTDNGQI
GIIDPDCRLIGLHLYDGLFKVIPFDNKGQLKEAFNIRLEELQVLDIKFLFGCAKPTIAVL
YQDNKDARHVKTYEVSLKDKDFVEGPWSQNSLDNGADLLIPVPPPLCGVLIIGEETIVYC
SASAFKAIPIRPSITKAYGRVDVDGSRYLLGDHAGMIHLLVITHEKEKVTGLKIELLGET
SIASTISYLDNAVVFVGSSYGDSQLVKLNLHPDAKGSYVEVLERYINLGPIVDFCVVDLE
RQGQGQVVTCSGAFKDGSLRVVRNGIGINEQASVELQGIKGMWSLKSSIDEAFDTFLVVS
FISETRILAMNLEDELEETEIEGFLSQVQTLFCHDAVYNQLVQVTSNSVRLVSSTTRELR
DEWHAPAGFTVNVATANASQVLLATGGGHLVYLEIGDGKLTEVQHALLEYEVSCLDINPI
GDNPNYSQLAAVGMWTDISVRIFSLPELTLITKEQLGGEIIPRSVLLCAFEGISYLLCAL
GDGHLLNFQMDTTTGQLKDRKKVSLGTQPITLRTFSSKSATHVFAASDRPTVIYSSNKKL
LYSNVNLKEVSHMCPFNSAAFPDSLAIAREGELTIGTIDDIQKLHIRTIPLGEHARRICH
QEQTRTFGICSLGNQSNSEESEMHFVRLLDDQTFEFMSTYPLDSFEYGCSILSCSFTEDK
NVYYCVGTAYVLPEENEPTKGRILVFIVEDGRLQLIAEKETKGAVYSLNAFNGKLLAAIN
QKIQLYKWMLRDDGTRELQSECGHHGHILALYVQTRGDFIVVGDLMKSISLLLYKHEEGA
IEERARDYNANWMSAVEILDDDIYLGAENNFNLLTVKKNSEGATDEERGRLEVVGEYHLG
EFVNRFRHGSLVMRLPDSEIGQIPTVIFGTVNGVIGVIASLPQEQYTFLEKLQSSLRKVI
KGVGGLSHEQWRSFNNEKRTAEARNFLDGDLIESFLDLSRNKMEDISKSMNVQVEELCKR
VEELTRLH
>Q7XAK4
MKFRSDSSGGDEPRAPAAGDGGGGGDEPAKRQRTDPSSSSSQGEASSSSQPPPQQQQEEQ
PPEDAGEGEQPRVPDLGEDLVFEVLRRAEARTLAAAACVSRGWRQLAEDERLWEAACVRE
WANLGFSERQLRAVVLSLGGFRRLHAVYIRPLQWRGAGVPRQQGRRQPPVRLGRDQVQLS
LSLFSIGFFQNMPCPKKDKGNDSDKNGGGQCG
>A2XLV9
MAGADVDVGTELRLGLPGGGGGAAEAAAKAAKRGFEETIDLKLKLPTAGMEEAAAGKAEA
PAAEKAKRPAEAAAADAEKPPAPKAQAVGWPPVRSFRRNIMTVQSVKSKKEEEADKQQQQ
PAANASGSNSSAFVKVSMDGAPYLRKVDLKMYNSYKDLSLALQKMFGTFTATGNNMNEVN
GSDAVTTYEDKDGDWMLVGDVPWQMFVESCKRLRIMKGSEAIGLAPRAKDKYKNKS
>Q9FN69
MATGQNRTTVPENLKKHLAVSVRNIQWSYGIFWSVSASQSGVLEWGDGYYNGDIKTRKTI
QASEIKADQLGLRRSEQLSELYESLSVAESSSSGVAAGSQVTRRASAAALSPEDLADTEW

YYLVCMSFVFNIGEGMPGRTFANGEPIWLCNAHTADSKVFSRSLLAKSAAVKTVVCFPFL
GGVVEIGTTEHITEDMNVIQCVKTSFLEAPDPYATILPARSDYHIDNVLDPQQILGDEIY
APMFSTEPFPTASPSRTTNGFDQEHEQVADDHDSFMTERITGGASQVQSWQLMDDELSNC
VHQSLNSSDCVSQTFVEGAAGRVAYGARKSRVQRLGQIQEQQRNVKTLSFDPRNDDVHYQ
SVISTIFKTNHQLILGPQFRNCDKQSSFTRWKKSSSSSSGTATVTAPSQGMLKKIIFDVP
RVHQKEKLMLDSPEARDETGNHAVLEKKRREKLNERFMTLRKIIPSINKIDKVSILDDTI
EYLQELERRVQELESCRESTDTETRGTMTMKRKKPCDAGERTSANCANNETGNGKKVSVN
NVGEAEPADTGFTGLTDNLRIGSFGNEVVIELRCAWREGVLLEIMDVISDLHLDSHSVQS
STGDGLLCLTVNCKHKGSKIATPGMIKEALQRVAWIC
>Q38859
MNAPERYERFVVPEGTKKVSYDRDTKIINAASFTVEREDHTIGNIVRMQLHRDENVLFAG
YQLPHPLKYKIIVRIHTTSQSSPMQAYNQAINDLDKELDYLKNQFEAEVAKFSNQF
>Q8LK56
MNSRADPGDRYFRVPLENQTQQEFMGSWIPFTPKKPRSSLMVDERVINQDLNGFPGGEFV
DRGFCNTGVDHNGVFDHGAHQGVTNLSMMINSLAGSHAQAWSNSERDLLGRSEVTSPLAP
VIRNTTGNVEPVNGNFTSDVGMVNGPFTQSGTSQAGYNEFELDDLLNPDQMPFSFTSLLS
GGDSLFKVRQYGPPACNKPLYNLNSPIRREAVGSVCESSFQYVPSTPSLFRTGEKTGFLE
QIVTTTGHEIPEPKSDKSMQSIMDSSAVNATEATEQNDGSRQDVLEFDLNKTPQQKPSKR
KRKFMPKVVVEGKPKRKPRKPAELPKVVVEGKPKRKPRKAATQEKVKSKETGSAKKKNLK
ESATKKPANVGDMSNKSPEVTLKSCRKALNFDLENPGDARQGDSESEIVQNSSGANSFSE
IRDAIGGTNGSFLDSVSQIDKTNGLGAMNQPLEVSMGNQPDKLSTGAKLARDQQPDLLTR
NQQCQFPVATQNTQFPMENQQAWLQMKNQLIGFPFGNQQPRMTIRNQQPCLAMGNQQPMY
LIGTPRPALVSGNQQLGGPQGNKRPIFLNHQTCLPAGNQLYGSPTDMHQLVMSTGGQQHG
LLIKNQQPGSLIRGQQPCVPLIDQQPATPKGFTHLNQMVATSMSSPGLRPHSQSQVPTTY
LHVESVSRILNGTTGTCQRSRAPAYDSLQQDIHQGNKYILSHEISNGNGCKKALPQNSSL
PTPIMAKLEEARGSKRQYHRAMGQTEKHDLNLAQQIAQSQDVERHNSSTCVEYLDAAKKT
KIQKVVQENLHGMPPEVIEIEDDPTDGARKGKNTASISKGASKGNSSPVKKTAEKEKCIV
PKTPAKKGRAGRKKSVPPPAHASEIQLWQPTPPKTPLSRSKPKGKGRKSIQDSGKARGPS
GELLCQDSIAEIIYRMQNLYLGDKEREQEQNAMVLYKGDGALVPYESKKRKPRPKVDIDD
ETTRIWNLLMGKGDEKEGDEEKDKKKEKWWEEERRVFRGRADSFIARMHLVQGDRRFSPW
KGSVVDSVIGVFLTQNVSDHLSSSAFMSLAARFPPKLSSSREDERNVRSVVVEDPEGCIL
NLNEIPSWQEKVQHPSDMEVSGVDSGSKEQLRDCSNSGIERFNFLEKSIQNLEEEVLSSQ
DSFDPAIFQSCGRVGSCSCSKSDAEFPTTRCETKTVSGTSQSVQTGSPNLSDEICLQGNE
RPHLYEGSGDVQKQETTNVAQKKPDLEKTMNWKDSVCFGQPRNDTNWQTTPSSSYEQCAT
RQPHVLDIEDFGMQGEGLGYSWMSISPRVDRVKNKNVPRRFFRQGGSVPREFTGQIIPST
PHELPGMGLSGSSSSAVQEHQDDTQHNQQDEMNKASHLQKTFLDLLNSSEECLTRQSSTKQ
NITDGCLPRDRTAEDVVDPLSNNSSLQNILVESNSSNKEQTAVEYKETNATILREMKGTL
ADGKKPTSQWDSLRKDVEGNEGRQERNKNNMDSIDYEAIRRASISEISEAIKERGMNNML
AVRIKDFLERIVKDHGGIDLEWLRESPPDKAKDYLLSIRGLGLKSVECVRLLTLHNLAFP
VDTNVGRIAVRMGWVPLQPLPESLQLHLLELYPVLESIQKFLWPRLCKLDQRTLYELHYQ
LITFGKVFCTKSRPNCNACPMRGECRHFASAYASARLALPAPEERSLTSATIPVPPESYP
PVAIPMIELPLPLEKSLASGAPSNRENCEPIIEEPASPGQECTEITESDIEDAYYNEDPD
EIPTIKLNIEQFGMTLREHMERNMELQEGDMSKALVALHPTTTSIPTPKLKNISRLRTEH
QVYELPDSHRLLDGMDKREPDDPSPYLLAIWTPGETANSAQPPEQKCGGKASGKMCFDET

CSECNSLREANSQTVRGTLLIPCRTAMRGSFPLNGTYFQVNELFADHESSLKPIDVPRDW
IWDLPRRTVYFGTSVTSIFRGLSTEQIQFCFWKGFVCVRGFEQKTRAPRPLMARLHFPAS
KLKNNKT
>Q94KE2
MDRNREARRVPMAAAGNGLSRRRHRAGSFRDSPEEEGPVELPEAARLRDRGGSNKKDRDR
ERDRDRERERERDRERDRLNSRSKRRRGERLMMVHGNLDDGGDDSSEESVNDDEEYDDGG
VGPPSSLKMLPPTSNNISAASFSSSLSNHHNGGSGNLHHHHHSHNNNHQRKNNFPPTKVF
RSSPSPAPVSPLVSTWKAADEMIGVSVPRKARSACTKRPHESWASSTTGGGVFASGEQIH
RQISSTSPANRVSPASILASPSPPAPTSPSSSSISVRKKLPSGTKQKPLPPKSSSSKLSS
PVAVQDEIEIEIAEVLYGMMRMPSTSKQEAAGNDLTEAAKSTVEVKSRVSSPISNPQTLP
QSSITLAANSSSSNVSAIAPKRKKPRHVKYEDDNSSRVTTIKSEAEAPSKSQVPFSNQLK
SSGSGEGNSSVLDSIIPLTRESNASLDSEKKENNLSKDETILPKVESSSGFRSDGEGAKS
SSPEKEKFEIDLMAPPPVRSSSERGGEMMECVAAEAKPKVTEVETEAKPLLKEDRSDPAI
HDSQEKKRPRMVAEAEHHKFERNCELKLDLDKSDHVGLVNKHHVQKPPPQQQLSVPDKTA
QASHLPLHMSMPGWPGGLPTMGYMAPTQGVVPTDTSSLSAAAMQPPPHLLFNQPRPKRCA
THCYIARNIQSHQQFTKMNPFWPAAAGSAPMYGTKACNLSLMPPTELQGSVLGRSSNPVQ
DKNSQSTSKSSETAQRNQLMLQQALPPGAANSILHGPTFIFPLGQQPHAAATIAAASVRP
PNSGITSSGPTATSTSMNGSASATPAGAPTMSFSYPAMPGNETQYLAILQNNGYPFPVPA
HVGAQPAYRGAPGQPMPFFNGSFYSSQMIQPPHHQPQKQHQQQLTGQMLQSHAPNNQNGS
ASTGSSAAQKHLQNQQLRPPINHGNSQGFPTHKVQSQPLNFQQRQQPRENATQHSETVGE
DSPSTADSRGSRSNVAYGQNYGMQMQPTNLGLMSSPAPGGGVVGSSSSHGEKKSQQQVSK
AGVESFQSPGYAMTFATFNGANTAPTLNMSSIAQNHAMFHSMPEAARQGYQMMAAQAAQQ
KMNYGASLEDGKSGSIGGAATANNTPEEQRKSGGGAIGKTSGGNGGQSIAFSNKQDLADA
SVSAVTSGSIVDSSSRLLNLGSALPQSSGSLPTSHHQQLLQQQQQQHMQRSQSQQPYTTM
YLQKQQRYATSVAASAARTKGPVVSNGSGFPDHNMTTSPAGTTKFANANSGFPQNLVQSS
SNQVQSQQWKNNSPRTTNTTQAQSPSMLSPSTSVAAASSLRNIPHKQQSRPQQSQISFAA
NSKPMTSGSPMQQVQGGTNHQAPSPPMLVGSPSTSSVSKNASGSPRTTASASSAANKGGQ
ASTTTHSASQPSKNLQPASAASSAGGRNNGPSVLGNPTTSSGSKSQQQQQLPKHGLQPQA
QLFFSNPYMQAQHQHQQQQITISPSGGYYIQRHQQQSGSAPAVPVTGAVTATSDPAKAIA
AASAANNMKGGGGMGKTQQHQLGPPGFTNVHAVSSAVQVKPVDQKQQAGE
>Q9ZSZ8
MEFSSQDDDFGGDDSAANATRASGNRRSFGDLEDDEDDIFGSTTVAPGVRTGMILSLRGS
LKNCKDDLASCQNELESAKTEIQKWKSAFQNESFVPAGKSPEPRFLIDYIQNLKSSEKSL
KEQLEIAKRKEASCIVQYAKREQEMAELKSAVRDLKSQLKPASMQARRLLLDPAIHEEFS
RLKNLVEEKDKKIKELQDNIAAVTFTPQSKNGKMLMAKCRTLQEENEEIGHQAAEGKIHE
LAIKLAMQKSQNAELRSQFEGLYKHMEELTNDVERSNETVIILQEKLEEKEKEIERVKKG
LEIVSELVGDKKDEVDEIDEDAKEEIAGGE
>Q9S7T7
MLRLESLIVTVWGPATLLARKAPLGQIWMAATLHAKINRKKLDKLDIIQICEEILNPSVP
MALRLSGILMGGVVIVYERKVKLLFDDVNRFLVEINGAWRTKSVPDPTLLPKGKTHARKE
AVTLPENEEADFGDFEQTRNVPKFGNYMDFQQTFISMRLDESHVNNNPEPEDLGQQFHQA
DAENITLFEYHGSFQTNNETYDRFERFDIEGDDETQMNSNPREGAEIPTTLIPSPPRHHD
IPEGVNPTSPQRQEQQENRRDGFAEQMEEQNIPDKEEHDRPQPAKKRARKTATSAMDYEQ
TIIAGHVYQSWLQDTSDILCRGEKRKVRGTIRPDMESFKRANMPPTQLFEKDSSYPPQLY

QLWSKNTQVLQTSSSESRHPDLRAEQSPGFVQERMHNHHQTDHHERSDTSSQNLDSPAEI
LRTVRTGKGASVESMMAGSRASPETINRQAADINVTPFYSGDDVRSMPSTPSARGAASIN
NIEISSKSRMPNRKRPNSSPRRGLEPVAEERPWEHREYEFEFSMLPEKRFTADKEILFET
ASTQTQKPVCNQSDEMITDSIKSHLKTHFETPGAPQVESLNKLAVGMDRNAAAKLFFQSC
VLATRGVIKVNQAEPYGDILIARGPNM
>Q9FIE3
MQAASLSKIWRFDGNVGPENMDSSSFEDNECIETCKPNVLNVSERRELIHALSNQPEEAS
ELLNSWSRNEIMKIICAEMGKERKYTGLNKPKLIENLLNLVSRPLGETSCSDRRNSRKKE
KKMIGYIICCENLACRAALGCDDTFCRRCSCCICQKFDDNKDPSLWLTCDACGSSCHLEC
GLKQDRYGIGSDDLDGRFYCAYCGKDNDLLGCWRKQVKVAKETRRVDVLCYRLSLGQKLL
RGTTKYRNLLELMDEAVKKLEGDVGPLSGWAMKMARGIVNRLSSGVHVQKLCSQAMEALD
KVVSPSESVSGQGDKMTVRVEEIQARSVTVRVDSEEPSSSTQNKITGFRLFCRKSKDEEC
SSQGNCVVYLPETTSAIQGLEPDTEFCLRVVSFNEEGDLDESELRFTTLKDDGDEAGDQQ
SPLTNSSSGLCSNPSLPEDESNNVNKSCSKGNGDKDNTEHCSAGEVESELEEERLVKRKA
NKIDGRDLLVTPCKRDIYKGKQGGNKRFKSRTVSLNEKPEINNAANGVGDKDLGHIVKTI
RCLEEEGHIDKSFRERFLTWYSLRATHREVRVVKIFVETFMEDLSSLGQQLVDTFSESIL
SKRSSTNGVVPAGICLKLWH
>O04479
MASSSTNSPCAACKFLRRKCQPECVFAPYFPPDQPQKFANVHKVFGASNVTKLLNELHPS
QREDAVNSLAYEADMRLRDPVYGCVGVISLLQHQLRQLQIDLSCAKSELSKYQSLGILAA
THQSLGINLLAGAADGTATAVRDHYHHHQFFPREQMFGGLDVPAGNNYDGGILAIGQITQ
FQQPRAAAGDDGRRTVDPS
>Q9ATB4
MGRSRGNFQNFEDPTQRTRKKKNAANVENFESTSLVPGAEGGGKYNCDYCQKDITGKIRI
KCAVCPDFDLCIECMSVGAEITPHKCDHPYRVMGNLTFPLICPDWSADDEMLLLEGLEIY
GLGNWAEVAEHVGTKSKEQCLEHYRNIYLNSPFFPLPDMSHVAGKNRKELQAMAKGRIDD
KKAEQNMKEEYPFSPPKVKVEDTQKESFVDRSFGGKKPVSTSVNNSLVELSNYNQKREEF
DPEYDNDAEQLLAEMEFKENDTPEEHELKLRVLRIYSKRLDERKRRKEFIIERNLLYPNP
FEKDLSQEEKVQCRRLDVFMRFHSKEEHDELLRNVVSEYRMVKRLKDLKEAQVAGCRSTA
EAERYLGRKRKRENEEGMNRGKESGQFGQIAGEMGSRPPVQASSSYVNDLDLIGFTESQL
LSESEKRLCSEVKLVPPVYLQMQQVMSHEIFKGNVTKKSDAYSLFKIDPTKVDRVYDMLV
KKGIAQL
>Q0J4I1
MAALHHQAAAAPVTTTTDGGELRAMDLYEKLEKVGEGTYGKVYKAREKATGRIVALKKTR
LPEDDEGVPPTALREVSLLRMLSQDSHVVRLLDLKQGQNKEGQTILYLVFEYMDTDLKKF
IRAHRQNLQKIPVPTVKILMYQLCKGVAFCHGRGVLHRDLKPHNLLMDRKTMALKIADLG
LSRSFTVPLKKYTHEILTLWYRAPEVLLGAAHYSTPVDIWSVGCIFAELATNQPLFAGDS
EVQQLLHIFKLLGTPNEQVWPGVSKLPNWHEYPQWNPSKVSDLVHGLDADALDLLEKMLQ
YEPSKRISAKKAMEHPYFNDVNKELY
>Q0JBF0
MMSSAPETFSLDHLSQHQQQQPPPLAEQEQLCYVHCNFCDTILAVGVPCSSLFKTVTVRC
GHCANLLSVNLRGLLLPAAASTANQLPFGQALLSPTSPHGLLDEVPSFQAPASLMTEQAS
PNVSSITSSNSSCANNAPATSMASAANKATQREPQQPKNAPSANRTSEKRQRVPSAYNRF
IKDEIQRIKASNPDITHREAFSAAAKNWAHFPHIHFGLMPDQGLKKTGIQSQDGAGECML

FKDGLYAAAAAAAAATAASSMGVTPF
>Q9SBK6
MEVMRILHMNKGNGETSYAKNSIVQSNIISLGRRVMDEALKKLMIRNSEILSFGIADLGC
SSGPNSLLSISNIVETIQNLCHDLDRPVPELSLSLNDLPSNDFNYIFASLPEFYDRVKKR
DNNYESLGFEHGSGGPCFVSAVPGSFYGRLFPRRSLHFVHSSSSLHWLSQVPCGEVNKKD
GVVITADLDNRGKIYLSKTSPKSAHKVYALQFQTDFSVFLRSRSEELVPGGRMVLSFLGR
SSPDPTTEESCYQWELLAQALMSLAKEGIIEEENIDAFNAPYYAASPEELKMAIEKEGSF
SIDRLEISPVDWEGGSISDDSYDIVRFKPEALASGRRVAKTIRAVVEPMLEPTFGQKVMD
ELFERYAKLVGEYVYVSSPRYTIVIVSLLRMG
>Q9XGM2
MSREDFSDTLRVLVATDCHLGYMEKDEIRRHDSFKAFEEICSIAEEKQVDFLLLGGDLFH
ENKPSRTTLVKAIEILRRHCLNDKPVQFQVVSDQTVNFQNAFGQVNYEDPHFNVGLPVFS
IHGNHDDPAGVDNLSAIDILSACNLVNYFGKMVLGGSGVGQITLYPILMKKGSTTVALYG
LGNIRDERLNRMFQTPHAVQWMRPEVQEGCDVSDWFNILVLHQNRVKSNPKNAISEHFLP
RFLDFIVWGHEHECLIDPQEVSGMGFHITQPGSSVATSLIDGESKPKHVLLLEIKGNQYR
PTKIPLTSVRPFEYTEIVLKDESDIDPNDQNSILEHLDKVVRNLIEKASKKAVNRSEIKL
PLVRIKVDYSGFMTINPQRFGQKYVGKVANPQDILIFSKASKKGRSEANIDDSERLRPEE
LNQQNIEALVAESNLKMEILPVNDLDVALHNFVNKDDKLAFYSCVQYNLQETRGKLAKDS
DAKKFEEDDLILKVGECLEERLKDRSTRPTGSSQFLSTGLTSENLTKGSSGIANASFSDD
EDTTQMSGLAPPTRGRRGSSTANTTRGRAKAPTRGRGRGKASSAMKQTTLDSSLGFRQSQ
RSASAAASAAFKSASTIGEDDVDSPSSEEVEPEDFNKPDSSSEDDESTKGKGRKRPATTK
RGRGRGSGTSKRGRKNESSSSLNRLLSSKDDDEDEDDEDREKKLNKSQPRVTRNYGALRR

>Q8W420
MQNQMEWDSDSDLSGGDEVAEDGWFGGDNGAIPFPVGSLPGTAPCGFVVSDALEPDNPII
YVNTVFEIVTGYRAEEVIGRNCRFLQCRGPFTKRRHPMVDSTIVAKMRQCLENGIEFQGE
LLNFRKDGSPLMNKLRLVPIREEDEITHFIGVLLFTDAKIDLGPSPDLSAKEIPRISRSF
TSALPIGERNVSRGLCGIFELSDEVIAIKILSQLTPGDIASVGCVCRRLNELTKNDDVWR
MVCQNTWGTEATRVLESVPGAKRIGWVRLAREFTTHEATAWRKFSVGGTVEPSRCNFSAC
AVGNRIVIFGGEGVNMQPMNDTFVLDLGSSSPEWKSVLVSSPPPGRWGHTLSCVNGSRLV
VFGGYGSHGLLNDVFLLDLDADPPSWREVSGLAPPIPRSWHSSCTLDGTKLIVSGGCADS
GALLSDTFLLDLSMDIPAWREIPVPWTPPSRLGHTLTVYGDRKILMFGGLAKNGTLRFRS
NDVYTMDLSEDEPSWRPVIGYGSSLPGGMAAPPPRLDHVAISLPGGRILIFGGSVAGLDS
ASQLYLLDPNEEKPAWRILNVQGGPPRFAWGHTTCVVGGTRLVVLGGQTGEEWMLNEAHE
LLLATSTTAST
>Q9LRE6
MSSGGRGGKRRGAPPPGPSGAAAKRAHPGGTPQPPPPAATAAAPVAEEEDMMDEDVFLDE
TILAEDEEALLLLDRDEALASRLSRWRRPALPADLASGCSRNVAFQQLEIDYVIGESHKV
LLPNSSGPAAILRIFGVTREGHSVCCQVHGFEPYFYISCPMGMGPDDISRFHQTLEGRMK
DSNRNSNVPRFVKRIELVQKQTIMHYQPQQSQPFLKIVVALPTMVASCRGILERGITIEG
LGSKSFLTYESNILFALRFMIDCNIVGGNWIEVPAGKYMKAARIMSYCQLELDCLYSDLV
SHAAEGEHSKMAPFRILSFDIECAGRKGHFPEPTHDPVIQIANLVTLQGEGQPFVRNVMT
LKSCSPIVGVDVMSFDTERDVLLAWRDFIREVDPDIIIGYNICKFDLPYLIERAEVLKIV
EFPILGRIRNSRVRVRDTTFSSRQYGMRESKDVAVEGRVQFDLLQAMQRDYKLSSYSLNS

VSAHFLGEQKEDVHHSIISDLQNGNSETRRRLAVYCLKDAYLPQRLLDKLMYIYNYVEMA
RVTGVPISFLLSRGQSIKVLSQLLRKAKQKNLVIPNIKGQASGQDTFEGATVLEARAGFY
EKPIATLDFASLYPSIMMAYNLCYCTLVPPEDARKLNLPPESVNKTPSGETFVKPDVQKG
ILPEILEELLAARKRAKADLKEAKDPFERAVLDGRQLALKISANSVYGFTGATVGQLPCL
EISSSVTSYGRQMIEHTKKLVEDKFTTLGGYEHNAEVIYGDTDSVMVQFGVSTVEDAMKL
GREAADYISGTFIKPIKLEFEKIYFPYLLISKKRYAGLYWTNPEKFDKMDTKGIETVRRD
NCLLVKNLVTECLHKILVDRDVPGAVQYVKNTISDLLMNRVDLSLLVITKGLTKTGEDYA
VKAAHVELAERMRKRDAATAPTVGDRVPYVIIKAAKGAKAYERSEDPIYVLDNNIPIDPQ
YYLENQISKPLLRIFEPILKNASRELLHGSHTRAVSISTPSNSGIMKFAKKQLTCLGCKA
VISGSNQTLCFHCKGREAELYCKTVGNVSELEMLFGRLWTQCQECQGSLHQDVLCTSRDC
PIFYRRRKAQKDMAEARVQLQRWDF
>Q11207
MANKLKVDELRLKLAERGLSTTGVKAVLVERLEEAIAEDTKKEESKSKRKRNSSNDTYES
NKLIAIGEFRGMIVKELREEAIKRGLDTTGTKKDLLERLCNDANNVSNAPVKSSNGTDEA
EDDNNGFEEEKKEEKIVTATKKGAAVLDQWIPDEIKSQYHVLQRGDDVYDAILNQTNVRD
NNNKFFVLQVLESDSKKTYMVYTRWGRVGVKGQSKLDGPYDSWDRAIEIFTNKFNDKTKN
YWSDRKEFIPHPKSYTWLEMDYGKEENDSPVNNDIPSSSSEVKPEQSKLDTRVAKFISLI
CNVSMMAQHMMEIGYNANKLPLGKISKSTISKGYEVLKRISEVIDRYDRTRLEELSGEFY
TVIPHDFGFKKMSQFVIDTPQKLKQKIEMVEALGEIELATKLLSVDPGLQDDPLYYHYQQ
LNCGLTPVGNDSEEFSMVANYMENTHAKTHSGYTVEIAQLFRASRAVEADRFQQFSSSKN
RMLLWHGSRLTNWAGILSQGLRIAPPEAPVTGYMFGKGVYFADMFSKSANYCYANTGAND
GVLLLCEVALGDMNELLYSDYNADNLPPGKLSTKGVGKTAPNPSEAQTLEDGVVVPLGKP
VERSCSKGMLLYNEYIVYNVEQIKMRYVIQVKFNYKH
>Q9LHS9
MMDRGECLMSMKLRPMVTRPSSDGTLFWPFREERAFASAEEYGGGGGCMWPPRSYSCSFC
GREFKSAQALGGHMNVHRRDRARLKQQSLSPSSTDQATPPECDRQQQVLDVGSKVLVQEE
TRKPNGTKREISDVCNNNVLESSMKRYEHDNGEVKTDLSVGLLSTEFDPRKKQLINGSSS
SWKRAKTDVSRFPMMLGLVIGISEINGHHEELDLELRLGADPPKVN
>Q9FF80
MERNGGHYTDKTRVLDIKPLRTLRPVFPSGNQAPPFVCAPPFGPFPPGFSSFYPFSSSQA
NQHTPDLNQAQYPPQHQQPQNPPPVYQQQPPQHASEPSLVTPLRSFRSPDVSNGNAELEG
STVKRRIPKKRPISRPENMNFESGINVADRENGNRELVLSVLMRFDALRRRFAQLEDAKE
AVSGIIKRPDLKSGSTCMGRGVRTNTKKRPGIVPGVEIGDVFFFRFEMCLVGLHSPSMAG
IDYLVVKGETEEEPIATSIVSSGGYYDNDEGNPDVLIYTGQGGNADKDKQSSDQKLERGNL
ALEKSLRRDSAVRVIRGLKEASHNAKIYIYDGLYEIKESWVEKGKSGHNTFKYKLVRAPG
QPPAFASWTAIQKWKTGVPSRQGLILPDMTSGVESIPVSLVNEVDTDNGPAYFTYSTTVK
YSESFKLMQPSFGCDCANLCKPGNLDCHCIRKNGGDFPYTGNGILVSRKPMIYECSPSCP
CSTCKNKVTQMGVKVRLEVFKTANRGWGLRSWDAIRAGSFICIYVGEAKDKSKVQQTMAN
DDYTFDTTNVYNPFKWNYEPGLADEDACEEMSEESEIPLPLIISAKNVGNVARFMNHSCS
PNVFWQPVSYENNSQLFVHVAFFAISHIPPMTELTYDYGVSRPSGTQNGNPLYGKRKCFC
GSAYCRGSFG
>P52572
MPGLTIGDTVPNLELDSTHGKIRIHDYVGNGYVILFSHPGDFTPVCTTELAAMANYAKEF
EKRGVKLLGISCDDVQSHKEWTKDIEAYKPGSKVTYPIMADPDRSAIKQLNMVDPDEKDA

QGQLPSRTLHIVGPDKVVKLSFLYPSCTGRNMDEVVRAVDSLLTAAKHKVATPANWKPGE
CVVIAPGVSDEEAKKMFPQGFETADLPSKKGYLRFTKV
>Q38796
MDAFKEEIEIGSSVESLMELLDSQKVLFHSQIDQLQDVVVAQCKLTGVNPLAQEMAAGAL
SIKIGKRPRDLLNPKAVKYLQAVFAIKDAISKRESREISALFGITVAQVREFFVTQKTRV
RKQVRLSREKVVMSNTHALQDDGVPENNNATNHVEPVPLNSIHPEACSISWGEGETVALI
PPEDIPPDISDSDKYFVENIFSLLRKEETFSGQVKLMEWIMQIQDASVLIWFLSKGGVLI
LTTWLSQAASEEQTSVLLLILKVLCHLPLHKASPENMSAILQSVNGLRFYRISDISNRAK
GLLSRWTKLFAKIQAMKKQNRNSSQIDSQSQLLLKQSIAEIMGDSSNPEDILSLSNGKSE
NVRRIESSQGPKLLLTSADDSTKKHMLGSNPSYNKERRKVQMVEQPGQKAAGKSPQTVRI
GTSGRSRPMSADDIQKAKMRALYMQSKNSKKDPLPSAIGDSKIVAPEKPLALHSAKDSPP
IQNNEAKTEDTPVLSTVQPVNGFSTIQPVNGPSAVQPVNGPLAVQPVNGPSALQPVNGPS
AVIVPVQADEIKKPSTPPKSISSKVGVMMKMSSQTILKNCKRKQIDWHVPPGMELDELWR
VAAGGNSKEADVQRNRNRRERETTYQSLQTIPLNPKEPWDREMDYDDSLTPEIPSQQPPE
ESLTEPQDSLDERRIAAGAATTSSSLSSPEPDLELLAALLKNPDLVYALTSGKPSNLAGQ
DMVKLLDVIKTGAPNSSSSSNKQVEERVEVSLPSPTPSTNPGMSGWGQEGIRNPFSRQNQ
VGTAVARSGTQLRVGSMQWHQTNEQSIPRHAPSAYSNSITLAHTEREQQQYMQPKLHHNL
HFQQQQQQPISTTSYAVREPVGQMGTGTSSSWRSQQSQNSYYSHQENEIASASQVTSYQG
NSQYMSSNPGYESWSPDNSPSRNQLNMRGQQQQASRKHDSSTHPYWNQNKRWR
>P54609
MSTPAESSDSKSKKDFSTAILERKKSPNRLVVDEAINDDNSVVSLHPATMEKLQLFRGDT
ILIKGKKRKDTVCIALADETCEEPKIRMNKVVRSNLRVRLGDVISVHQCPDVKYGKRVHI
LPVDDTVEGVTGNLFDAYLKPYFLEAYRPVRKGDLFLVRGGMRSVEFKVIETDPAEYCVV
APDTEIFCEGEPVKREDEERLDDVGYDDVGGVRKQMAQIRELVELPLRHPQLFKSIGVKP
PKGILLYGPPGSGKTLIARAVANETGAFFFCINGPEIMSKLAGESESNLRKAFEEAEKNA
PSIIFIDEIDSIAPKREKTNGEVERRIVSQLLTLMDGLKSRAHVIVMGATNRPNSIDPAL
RRFGRFDREIDIGVPDEIGRLEVLRIHTKNMKLAEDVDLERISKDTHGYVGADLAALCTE
AALQCIREKMDVIDLEDDSIDAEILNSMAVTNEHFHTALGNSNPSALRETVVEVPNVSWN
DIGGLENVKRELQETVQYPVEHPEKFEKFGMSPSKGVLFYGPPGCGKTLLAKAIANECQA
NFISVKGPELLTMWFGESEANVREIFDKARQSAPCVLFFDELDSIATQRGGGSGGDGGGA
ADRVLNQLLTEMDGMNAKKTVFIIGATNRPDIIDSALLRPGRLDQLIYIPLPDEDSRLNI
FKAALRKSPIAKDVDIGALAKYTQGFSGADITEICQRACKYAIRENIEKDIEKEKRRSEN
PEAMEEDGVDEVSEIKAAHFEESMKYARRSVSDADIRKYQAFAQTLQQSRGFGSEFRFEN
SAGSGATTGVADPFATSAAAAGDDDDLYN
>Q9FT54
MADSVPGHVAGGGLQGFSVDAECIKQRVDEVLQWVDSLEHKLKEVEEFYSSIGVSNSGSI
GKDTEKGRHVVGIRKIQQEAARREAVAAKRMQDLMRQFGTIFRQITQHKCAWPFMHPVNV
EGLGLHDYFEVIDKPMDFSTIKNQMEAKDGTGYKHVMQIYADMRLVFENAMNYNEETSDV
YSMAKKLLEKFEEKWAHFLPKVQEEEKIREEEEKQAAKEALLAKEASHIKTTRELGNEIC
HANDELEKLMRKVVERCRKITIEEKRNIGLALLKLSPDDLQKVLGIVAQANPSFQPRAEE
VSIEMDILDEPTLWRLKFFVKDALDNAMKKKKEEETKTRELSGAQKKEVSKKRNATTKLA
ERKTKRSRI
>P16867
MAPKKEEKPASQAAEAPEVKAEAKPKAVKAPKKKEKKAPAKKVAKEPSAGGEDGDKKSKK

KAKVAKSETYKLYIYKVLKQVHPDTGISSKAMSIMNSFINDIFEKVATEASKLSRYNKKP
TVTSREIQTAVRLVLPGELAKHAVSEGTKAVTKFTSA
>Q9S775
MSSLVERLRIRSDRKPVYNLDDSDDDDFVPKKDRTFEQVEAIVRTDAKENACQACGESTN
LVSCNTCTYAFHAKCLVPPLKDASVENWRCPECVSPLNEIDKILDCEMRPTKSSEQGSSD
AEPKPIFVKQYLVKWKGLSYLHCSWVPEKEFQKAYKSNHRLKTRVNNFHRQMESFNNSED
DFVAIRPEWTTVDRILACREEDGELEYLVKYKELSYDECYWESESDISTFQNEIQRFKDV
NSRTRRSKDVDHKRNPRDFQQFDHTPEFLKGLLHPYQLEGLNFLRFSWSKQTHVILADEM
GLGKTIQSIALLASLFEENLIPHLVIAPLSTLRNWEREFATWAPQMNVVMYFGTAQARAV
IREHEFYLSKDQKKIKKKKSGQISSESKQKRIKFDVLLTSYEMINLDSAVLKPIKWECMI
VDEGHRLKNKDSKLFSSLTQYSSNHRILLTGTPLQNNLDELFMLMHFLDAGKFGSLEEFQ
EEFKDINQEEQISRLHKMLAPHLLRRVKKDVMKDMPPKKELILRVDLSSLQKEYYKAIFT
RNYQVLTKKGGAQISLNNIMMELRKVCCHPYMLEGVEPVIHDANEAFKQLLESCGKLQLL
DKMMVKLKEQGHRVLIYTQFQHMLDLLEDYCTHKKWQYERIDGKVGGAERQIRIDRFNAK
NSNKFCFLLSTRAGGLGINLATADTVIIYDSDWNPHADLQAMARAHRLGQTNKVMIYRLI
NRGTIEERMMQLTKKKMVLEHLVVGKLKTQNINQEELDDIIRYGSKELFASEDDEAGKSG
KIHYDDAAIDKLLDRDLVEAEEVSVDDEEENGFLKAFKVANFEYIDENEAAALEAQRVAA
ESKSSAGNSDRASYWEELLKDKFELHQAEELNALGKRKRSRKQLVSIEEDDLAGLEDVSS
DGDESYEAESTDGEAAGQGVQTGRRPYRRKGRDNLEPTPLMEGEGRSFRVLGFNQSQRAI
FVQTLMRYGAGNFDWKEFVPRLKQKTFEEINEYGILFLKHIAEEIDENSPTFSDGVPKEG
LRIEDVLVRIALLILVQEKVKFVEDHPGKPVFPSRILERFPGLRSGKIWKEEHDKIMIRA
VLKHGYGRWQAIVDDKELGIQELICKELNFPHISLSAAEQAGLQGQNGSGGSNPGAQTNQ
NPGSVITGNNNASADGAQVNSMFYYRDMQRRLVEFVKKRVLLLEKAMNYEYAEEYYGLGG
SSSIPTEEPEAEPKIADTVGVSFIEVDDEMLDGLPKTDPITSEEIMGAAVDNNQARVEIA
QHYNQMCKLLDENARESVQAYVNNQPPSTKVNESFRALKSINGNINTILSITSDQSKSHE
DDTKPDLNNVEMKDTAEETKPLRGGVVDLNVVEGEENIAEASGSVDVKMEEAKEEEKPKN
MVVD
>Q39234
MEMMSSSSSTTQVVSFRDMGMYEPFQQLSGWESPFKSDINNITSNQNNNQSSSTTLEVDA
RPEADDNNRVNYTSVYNNSLEAEPSSNNDQDEDRINDKMKRRLAQNREAARKSRLRKKAH
VQQLEESRLKLSQLEQELVRARQQGLCVRNSSDTSYLGPAGNMNSGIAAFEMEYTHWLEE
QNRRVSEIRTALQAHIGDIELKMLVDSCLNHYANLFRMKADAAKADVFFLMSGMWRTSTE
RFFQWIGGFRPSELLNVVMPYVEPLTDQQLLEVRNLQQSSQQAEEALSQGLDKLQQGLVE
SIAIQIKVVESVNHGAPMASAMENLQALESFVNQADHLRQQTLQQMSKILTTRQAARGLL
ALGEYFHRLRALSSLWAARPREHT
>Q8LBI1
MVFVKSTKSNAYFKRYQVKFRRRRDGKTDYRARIRLINQDKNKYNTPKYRFVVRFTNKDI
VAQIVSASIAGDIVKASAYAHELPQYGLTVGLTNYAAAYCTGLLLARRVLKMLEMDDEYE
GNVEATGEDFSVEPTDSRRPFRALLDVGLIRTTTGNRVFGALKGALDGGLDIPHSDKRFA
GFHKENKQLDAEIHRNYIYGGHVSNYMKLLGEDEPEKLQTHFSAYIKKGVEAESIEELYK
KVHAAIRADPNPKKTVKPAPKQHKRYNLKKLTYEERKNKLIERVKALNGAGGDDDDEDDE
E
>Q05153
MADGHSFNNISLSGRGGKNPGLLKINSGGIQWKKQGGGKAVEVDRSDIVSVSWTKVTKSN

QLGVKTKDGLYYKFVGFRDQDVPSLSSFFQSSYGKTPDEKQLSVSGRNWGEVDLHGNTLT
FLVGSKQAFEVSLADVSQTQLQGKNDVTLEFHVDDTAGANEKDSLMEISFHIPNSNTQFV
GDENRPPSQVFNDTIVAMADVSPGVEDAVVTFESIAILTPRGRYNVELHLSFLRLQGQAN
DFKIQYSSVVRLFLLPKSNQPHTFVVISLDPPIRKGQTMYPHIVMQFETDTVVESELSIS
DELMNTKFKDKLERSYKGLIHEVFTTVLRWLSGAKITKPGKFRSSQDGFAVKSSLKAEDG
VLYPLEKGFFFLPKPPTLILHDEIDYVEFERHAAGGANMHYFDLLIRLKTDHEHLFRNIQ
RNEYHNLYTFISSKGLKIMNLGGAGTADGVAAVLGDNDDDDAVDPHLTRIRNQAADESDE
EDEDFVMGEDDDGGSPTDDSGGDDSDASEGGVGEIKEKSIKKEPKKEASSSKGLPPKRKT
VAADEGSSKRKKPKKKKDPNAPKRAMSGFMFFSQMERDNIKKEHPGIAFGEVGKVLGDKW
RQMSADDKEPYEAKAQVDKQRYKDEISDYKNPQPMNVDSGNDSDSN
>Q9SE43
MEMAVANHRERSSDSMNRHLDSSGKYVRYTAEQVEALERVYAECPKPSSLRRQQLIRECS
ILANIEPKQIKVWFQNRRCRDKQRKEASRLQSVNRKLSAMNKLLMEENDRLQKQVSQLVC
ENGYMKQQLTTVVNDPSCESVVTTPQHSLRDANSPAGLLSIAEETLAEFLSKATGTAVDW
VQMPGMKPGPDSVGIFAISQRCNGVAARACGLVSLEPMKIAEILKDRPSWFRDCRSLEVF
TMFPAGNGGTIELVYMQTYAPTTLAPARDFWTLRYTTSLDNGSFVVCERSLSGSGAGPNA
ASASQFVRAEMLSSGYLIRPCDGGGSIIHIVDHLNLEAWSVPDVLRPLYESSKVVAQKMT
ISALRYIRQLAQESNGEVVYGLGRQPAVLRTFSQRLSRGFNDAVNGFGDDGWSTMHCDGA
EDIIVAINSTKHLNNISNSLSFLGGVLCAKASMLLQNVPPAVLIRFLREHRSEWADFNVD
AYSAATLKAGSFAYPGMRPTRFTGSQIIMPLGHTIEHEEMLEVVRLEGHSLAQEDAFMSR
DVHLLQICTGIDENAVGACSELIFAPINEMFPDDAPLVPSGFRVIPVDAKTGDVQDLLTA
NHRTLDLTSSLEVGPSPENASGNSFSSSSSRCILTIAFQFPFENNLQENVAGMACQYVRS
VISSVQRVAMAISPSGISPSLGSKLSPGSPEAVTLAQWISQSYSHHLGSELLTIDSLGSD
DSVLKLLWDHQDAILCCSLKPQPVFMFANQAGLDMLETTLVALQDITLEKIFDESGRKAI
CSDFAKLMQQGFACLPSGICVSTMGRHVSYEQAVAWKVFAASEENNNNLHCLAFSFVNWS
FV
>Q39238
MSDDMVLHFSSNSSNQSDHSLPDKIAKLEARLTGKTPSSAKPPQQQQQQQQQVSLWSSAS
AAVKVVTSTPPGLSETSISDSDDENTGDFLIRANTKKRQKVQESNNFSVVDHVEPQEAAY
DGRKNDAESKTGLDVSKKKQGRGRASSTGRGRGSKTNNDVTKSQFVVAPVSAASQLDASD
QKDFRPDGQLRNGECSLQDEDLKSLRAKIAMLEEELRKSRQDSSEYHHLVRNLENEVKDL
KDQEQQGKQKTTKVISDLLISVSKTERQEARTKVRNESLRLGSVGVLRTGTIIAETWEDG
QMLKDLNAQLRQLLETKEAIERQRKLLKKRQNGDKNDGTDTESGAQEEDIIPDEVYKSRL
TSIKREEEAVLRERERYTLEKGLLMREMKRIRDEDGSRFNHFPVLNSRYALLNLLGKGGF
SEVYKAYDLVDHRYVACKLHGLNAQWSEEKKQSYIRHANRECEIHKSLVHHHIVRLWDKF
HIDMHTFCTVLEYCSGKDLDAVLKATSNLPEKEARIIIVQIVQGLVYLNKKSQKIIHYDL
KPGNVLFDEFGVAKVTDFGLSKIVEDNVGSQGMELTSQGAGTYWYLPPECFELNKTPMIS
SKVDVWSVGVLFYQMLFGKRPFGHDQSQERILREDTIIKAKKVEFPVTRPAISNEAKDLI
RRCLTYNQEDRPDVLTMAQDPYLAYSKK
>Q8W575
MIGAVNSVEAVITSIQGLSGSPEDLSALHDLLRGAQDSLRAEPGVNFSTLDQLDASKHSL
GYLYFLEVLTCGPVSKEKAAYEIPIIARFINSCDAGQIRLASYKFVSLCKILKDHVIALG
DPLRGVGPLLNAVQKLQVSSKRLTALHPDVLQLCLQAKSYKSGFSILSDDIVEIDQPRDF
FLYSYYGGMICIGLKRFQKALELLYNVVTAPMHQVNAIALEAYKKYILVSLIHNGQFTNT

LPKCASTAAQRSFKNYTGPYIELGNCYNDGKIGELEALVVARNAEFEEDKNLGLVKQAVS
SLYKRNILRLTQKYLTLSLQDIANMVQLGNAKEAEMHVLQMIQDGQIHALINQKDGMVRF
LEDPEQYKSSEMIEIMDSVIQRTIGLSKNLLAMDESLSCDPLYLGKVGRERQRYDFGDDF
DTVPQKFSM
>Q8LPU4
MALKQKDTDAAATATGTKKRRRVFFSDTDAGVEANECMKVFLVWNPGEVSSVDCTAIQPF
DLNHFFGEDGKIYGYKNLKINVWISAKSFHGYADVSFDETSDGGKGITDLKPVLQNIFGE
NLVEKEEFLHTFSKECEYIRTAVTNGSAIKHDGSYESDPAVEIVRVELQGAAAFLYSRLV
PLVLLLVEGSTPIDIGEHGWEMLLVVKKATQEAGSKFELLGFAAVHNFYHYPESIRLRIS
QILVLPPYQGEGHGLGLLEAINYIAQSENIYDVTIESPSDYLQYVRSSIDCLRLLMFDPI
KPALGAIVLSLKETNLSKRAQSLRMVPPADLMETVRQKLKINKKQFLRCWEILVFLSLDS
QDHKSMDNFRACIYDRMKGEILGSASGTNRKRLLQMPTSFNKEASFAVYWTQEIEDEDEQ
TVEQQPEDLKTQEQQLNELVDIQIEEIAGVAKNVTSRCKDKMTELVVQ
>Q5YGP7
MNSNNWLAFPLSPTHSSLPPHIHSSQNSHFNLGLVNDNIDNPFQNQGWNMINPHGGGGEG
GEVPKVADFLGVSKSGDHHTDHNLVPYNDIHQTNASDYYFQTNSLLPTVVTCASNAPNNY
ELQESAHNLQSLTLSMGSTGAAAAEVATVKASPAETSADNSSSTTNTSGGAIVEATPRRT
LETFGQRTSIYRGVTRHRWTGRYEAHLWDNSCRREGQSRKGRQVYLGGYDKEEKAARAYD
LAALKYWGPSTTTNFPITNYEKEVEEMKNMTRQEFVASIRRKSSGFSRGASMYRGVTRHH
QHGRWQARIGRVAGNKDLYLGTFSTEEEAAEAYDIAAIKFRGLNAVTNFEINRYDVKAIL
ESNTLPIGGGAAKRLKEAQALESSRKREEMIALGSNFHQYGAASGSSSVASSSRLQLQPY
PLSIQQPFEHLHHHQPLLTLQNNNDISQYHDSFSYIQTQLHLHQQQTNNYLQSSSHTSQL
YNAYLQSNPGLLHGFVSDNNNTSGFLGNNGIGIGSSSTVGSSAEEEFPAVKVDYDMPPSG
GATGYGGWNSGESAQGSNPGGVFTMWNE
>Q9FVU9
MEGSSSTIARKTWELENSILTVDSPDSTSDNIFYYDDTSQTRFQQEKPWENDPHYFKRVK
ISALALLKMVVHARSGGTIEIMGLMQGKTDGDTIIVMDAFALPVEGTETRVNAQDDAYEY
MVEYSQTNKLAGRLENVVGWYHSHPGYGCWLSGIDVSTQRLNQQHQEPFLAVVIDPTRTV
SAGKVEIGAFRTYSKGYKPPDEPVSEYQTIPLNKIEDFGVHCKQYYSLDVTYFKSSLDSH
LLDLLWNKYWVNTLSSSPLLGNGDYVAGQISDLAEKLEQAESHLVQSRFGGVVPSSLHKK
KEDESQLTKITRDSAKITVEQVHGLMSQVIKDELFNSMRQSNNKSPTDSSDPDPMITY
>P43254
MEEISTDPVVPAVKPDPRTSSVGEGANRHENDDGGSGGSEIGAPDLDKDLLCPICMQIIK
DAFLTACGHSFCYMCIITHLRNKSDCPCCSQHLTNNQLYPNFLLDKLLKKTSARHVSKTA
SPLDQFREALQRGCDVSIKEVDNLLTLLAERKRKMEQEEAERNMQILLDFLHCLRKQKVD
ELNEVQTDLQYIKEDINAVERHRIDLYRARDRYSVKLRMLGDDPSTRNAWPHEKNQIGFN
SNSLSIRGGNFVGNYQNKKVEGKAQGSSHGLPKKDALSGSDSQSLNQSTVSMARKKRIHA
QFNDLQECYLQKRRQLADQPNSKQENDKSVVRREGYSNGLADFQSVLTTFTRYSRLRVIA
EIRHGDIFHSANIVSSIEFDRDDELFATAGVSRCIKVFDFSSVVNEPADMQCPIVEMSTR
SKLSCLSWNKHEKNHIASSDYEGIVTVWDVTTRQSLMEYEEHEKRAWSVDFSRTEPSMLV
SGSDDCKVKVWCTRQEASVINIDMKANICCVKYNPGSSNYIAVGSADHHIHYYDLRNISQ
PLHVFSGHKKAVSYVKFLSNNELASASTDSTLRLWDVKDNLPVRTFRGHTNEKNFVGLTV
NSEYLACGSETNEVYVYHKEITRPVTSHRFGSPDMDDAEEEAGSYFISAVCWKSDSPTML
TANSQGTIKVLVLAA

>O24591
MEFWGLEVKPGSTVKCEPGYGFVLHLSQAALGESKKSDNALMYVKIDDQKLAIGTLSVDK
NPHIQFDLIFDKEFELSHTSKTTSVFFTGYKVEQPFEEDEMDLDSEDEDEELNVPVVKEN
GKADEKKQKSQEKAVAAPSKSSPDSKKSKDDDDSDEDETDDSDEDETDDSDEGLSSEEGD
DDSSDEDDTSDDEEEDTPTPKKPEVGKKRPAESSVLKTPLSDKKAKVATPSSQKTGGKKG
AAVHVATPHPAKGKTIVNNDKSVKSPKSAPKSGGSVPCKPCSKSFISETALQAHSRAKMG
ASESQVQ
>Q84Q77
MSLIRRSNVFDPFSLDLWDPFDGFPFGSGGSSSGSIFPSFPRGASSETAAFAGARIDWKE
TPEAHVFKADVPGLKKEEVKVEVDDGNILQISGERNKEQEEKTDQWHRVERSSGKFLRRF
RLPDNAKPEQIKASMENGVLTVTVPKEEAKKPDVKSIQISG
>Q32SG5
MTEDRAHKVADEPAASGRQSPERKKRKWDQPAEDLVSAAVTAAAVSGMPVMNFGALPGVV
LPGVTAYGAATLPSVVPVPYSLPPHIAPSVLQNAAAAAQKLSQAKIPDEVIAREIVINDA
DPSVRYKLTKRQTQEEIQKCTNTVIITRGKYHPPNLLPDGEKPLYLHISAGSQLKDTAER
IKAVDRAASMIEEILKQGTTSESISVPFSSSTGQAVRPFSASVFLGFDADPSLNITARIR
GPNDQYINHIMKETGVTVVLRGKDSENLGSCHSEASQQPLHLYLTSMHLKNLEAAKVLAE
NLLDTVAAEFGASRISSSKVYGAVPPPQQLLAGVDTSGTKSDVHYIVGPNVLSGATHSFA
STGVIAPVVAPAVTVQSGAPTYSGVPLPSNMAYPIPPANGGAFYSGYGDIYPQATPLQQL
AFTLKHASSSATQAVPVTSTPTSMATKGNSILDAEMDKRSRRKFQELPVSKGPATESQNS
QQGSKFVKTGLDSSGNIGSSSIAPPKKVHPGSNGMLPQEEADMPSHLSISTKMLPPPLKS
MLPLPPRSMPPPPPKSMPPPPPKFPSDEFLSRNENKFFPLKEPTAPPRSFDAISVLPSER
RPREPKEEKNKRHTCV
>Q9ZVD3
MAFAQSVYNQSSVLKINVMVVDDNRVFLDIWSRMLEKSKYREITVIAVDYPKKALSTLKN
QRDNIDLIITDYYMPGMNGLQLKKQITQEFGNLSVLVMSSDPNKEEESLSCGAMGFIPKP
IAPTDLPKIYQFALTYKRNGKSTLSTEQNQKDANVSVPQQIMLVPEQAYVLKTKKKNCSS
KSDTRTVNSTNVSHVSTNGSRKNRKRKPKGGPSDDGESLSQPPKKKKIWWTNPLQDLFLQ
AIQHIGYDKVVPKKILAIMNVPYLTRENVASHLQKYRLFVKRVVHQGRFSMLSDRGKDSM
FRQTHIKEPYVNYYTPSTSWYETSLNNRSFYSESVHGHSRLLSEAREPVRYNQMSYNYMN
RNISFENQPSQNEETRTVFEPPVMANKISQTSQVLGFGQLGPSAISGHNFNTNMMSSYGS
LTPNQPGTSHFSYGMQSVLNNENATYNPQPPANATTQPNLDELPQLENLNLYNDLGNTSE
LPYNISNFQSDDNKKQGEEDGDWTFVNINQDQSNGESSNTIATPETNTPNFNINPNQNQG
QAVPEFTDWSFLDQQELVDDDFMNSLFNNDMN
>Q32SG6
MILEKPSWIRHEGLQIFSIDIQTGGLRFATGGGDQKVRIWSMESVHKDNTNNDSKQRLLA
TLRDHFGSVNCVRWAKHGRYLASGSDDQVILIHERKAGSGTSEFGSGEPPDAENWKVIMT
WRGHTADVVDLSWSPDDSTLASGSLDNTIHIWNMNNGICTAVLRGHTSLVKGVTWDPIGS
FIASQSDDKTVMIWRTSDWSLAHKTEGHWTKSLGSTFFRRLAWSPCCHFITTTHGFQKPR
HSAPVLERGEWAATFDFLGHNAPIVVVKFNNSTFRKNFSSDQDPKAAPVGWANGASKTPT
KEQQPYNVIAIGSQDRTITVWTTASARPLFVARHFFSQSVVDLSWSPDGYSLFACSLDGS
AANFHFEVKELGHRLSDSEMDEWKRNRYGDVGGRQSNLAESPAQLLLEQASAKQSAGEKV
TSIVEQGKAPPKVSAGVPNPGLVVLSLEVPEVSHEDSKKTAGPTADDVKKGNQLSSPVKQ
REYRRPDGRKRIIPEAVGFASNQDNIPNHSQNHPVNFSSLDQRMNGTKPSYGSNSNSNNC

GVKDRTSVTARANITESLVIQKASAGAGNDGRLSIEHTRSMAPSSLTPCSALSIHVINKN
GNEDALPVCLEARPVERGAGDMIGVGALSTKETEIKCIKGTKTLWSDRISGKVTVLAGNA
NFWAVGCEDGFLQVYTRCGVRAMPAMMMGSAAVFIDCDDSWKLLLVTGRGLMYIWNLYDR
ACILHDSLASLVASPDESSAKDAGTVKVISATFSRCGSPLVALASRHAFLYDMSLKCWLR
IADDCFPASNFASSFSFPQGGELGKLQIDIGKFMARKPIWSRVTDDGLQTRAHLENQLAS
SLALKSAQEYRQCLLSYVRFLAREADESRLREVCESFLGPPMGKVGSASPTDPKNLAWDP
DVLGMKKHKLLKEDILPSMASNRKVQRLLNEFMDLLLEYETDVTLIPQPGTEGNGNGNDK
VMTS

>P45432

MERDEEASGPMMEMCTNGGEETSNRRPIISGEPLDIEAYAALYKGRTKIMRLLFIANHCG
GNHALQFDALRMAYDEIKKGENTQLFREVVNKIGNRLGEKYGMDLAWCEAVDRRAEQKKV
KLENELSSYRTNLIKESIRMGYNDFGDFYYACGMLGDAFKNYIRTRDYCTTTKHIIHMCM
NAILVSIEMGQFTHVTSYVNKAEQNPETLEPMVNAKLRCASGLAHLELKKYKLAARKFLD
VNPELGNSYNEVIAPQDIATYGGLCALASFDRSELKQKVIDNINFRNFLELVPDVRELIN
DFYSSRYASCLEYLASLKSNLLLDIHLHDHVDTLYDQIRKKALIQYTLPFVSVDLSRMAD
AFKTSVSGLEKELEALITDNQIQARIDSHNKILYARHADQRNATFQKVLQMGNEFDRDVR
AMLLRANLLKHEYHARSARKL

>P42744

MQAVKRSRRHVEEEPTMVEPKTKYDRQLRIWGEVGQAALEEASICLLNCGPTGSEALKNL
VLGGVGSITVVDGSKVQFGDLGNNFMVDAKSVGQSKAKSVCAFLQELNDSVNAKFIEENP
DTLITTNPSFFSQFTLVIATQLVEDSMLKLDRICRDANVKLVLVRSYGLAGFVRISVKEH
PIIDSKPDHFLDDLRLNNPWPELKSFVETIDLNVSEPAAAHKHIPYVVILVKMAEEWAQS
HSGNLPSTREEKKEFKDLVKSKMVSTDEDNYKEAIEAAFKVFAPRGISSEVQKLINDSCA
EVNSNSSAFWVMVAALKEFVLNEGGGEAPLEGSIPDMTSSTEHYINLQKIYLAKAEADFL
VIEERVKNILKKIGRDPSSIPKPTIKSFCKNARKLKLCRYRMVEDEFRNPSVTEIQKYLA
DEDYSGAMGFYILLRAADRFAANYNKFPGQFDGGMDEDISRLKTTALSLLTDLGCNGSVL
PDDLIHEMCRFGASEIHVVSAFVGGIASQEVIKLVTKQFVPMLGTYIFNGIDHKSQLLKL

>Q9LR78

MAPDQSYQYPSPSYESIQTFYDTDEDWPGPRCGHTLTAVFVNNSHQLILFGGSTTAVANH
NSSLPEISLDGVTNSVHSFDVLTRKWTRLNPIGDVPSPRACHAAALYGTLILIQGGIGPS
GPSDGDVYMLDMTNNKWIKFLVGGETPSPRYGHVMDIAAQRWLVIFSGNNGNEILDDTWA
LDTRGPFSWDRLNPSGNQPSGRMYASGSSREDGIFLLCGGIDHSGVTLGDTYGLKMDSDN
VWTPVPAVAPSPRYQHTAVFGGSKLHVIGGILNRARLIDGEAVVAVLDTETGEWVDTNQP
ETSASGANRQNQYQLMRRCHHAAASFGSHLYVHGGIREDVLLDDLLVAETSQSSSPEPEE
DNPDNYMLLDDYLMDEPKPLSSEPEASSFIMRSTSEIAMDRLAEAHNLPTIENAFYDSAI
EGYVPLQHGAETVGNRGGLVRTASLDQSTQDLHKKVISTLLRPKTWTPPANRDFFLSYLE
VKHLCDEVEKIFMNEPTLLQLKVPIKVFGDIHGQYGDLMRLFHEYGHPSVEGDITHIDYL
FLGDYVDRGQHSLEIIMLLFALKIEYPKNIHLIRGNHESLAMNRIYGFLTECEERMGESY
GFEAWLKINQVFDYLPLAALLEKKVLCVHGGIGRAVTIEEIENIERPAFPDTGSMVLKDI
LWSDPTMNDTVLGIVDNARGEGVVSFGPDIVKAFLERNGLEMILRAHECVIDGFERFADG
RLITVFSATNYCGTAQNAGAILVIGRDMVIYPKLIHPHPPPISSSEEDYTDKAWMQELNI
EMPPTPARGESSE

>O82804

MKRGKDEEKILEPMFPRLHVNDADKGGPRAPPRNKMALYEQLSIPSQRFGDHGTMNSRSN
NTSTLVHPGPSSQPCGVERNLSVQHLDSSAANQATEKFVSQMSFMENVRSSAQHDQRKMV
REEEDFAVPVYINSRRSQSHGRTKSGIEKEKHTPMVAPSSHHSIRFQEVNQTGSKQNVCL
ATCSKPEVRDQVKANARSGGFVISLDVSVTEEIDLEKSASSHDRVNDYNASLRQESRNRL
YRDGGKTRLKDTDNGAESHLATENHSQEGHGSPEDIDNDREYSKSRACASLQQINEEASD
DVSDDSMVDSISSIDVSPDDVVGILGQKRFWRARKAIANQQRVFAVQLFELHRLIKVQKL
IAASPDLLLDEISFLGKVSAKSYPVKKLLPSEFLVKPPLPHVVVKQRGDSEKTDQHKMES
SAENVVGRLSNQGHHQQSNYMPFANNPPASPAPNGYCFPPQPPPSGNHQQWLIPVMSPSE
GLIYKPHPGMAHTGHYGGYYGHYMPTPMVMPQYHPGMGFPPPGNGYFPPYGMMPTIMNPY
CSSQQQQQQQPNEQMNQFGHPGNLQNTQQQQQRSDNEPAPQQQQQPTKSYPRARKSRQGS
TGSSPSGPQGISGSKSFRPFAAVDEDSNINNAPEQTMTTTTTTTRTTVTQTTRDGGGVTR
VIKVVPHNAKLASENAARIFQSIQEERKRYDSSKP
>P41153
MEPNSSGSGKAAVGDGGGGGAPMLQPAPAPAPMPSANAPPPFLVKTYDMVDDPSTDKIVS
WSPTNNSFVVWDPPEFAKDLLPKYFKHNNFSSFVRQLNTYGFRKVDPDRWEFANEGFLRG
QKHLLKSISRRKPAHGHAQQQQQPHGHAQQQMQPPGHSASVGACVEVGKFGLEEEVERLK
RDKNVLMQELVRLRQQQQSTDNQLQGMVQRLQGMELRQQQMMSFLAKAVNSPGFLAQFVQ
QQNESNKRIAEGSKKRRIKQDIESQDPSVTPADGQIVKYQPGINEAAKAMLRELSKLDSS
PRLENFSNSPESFLIGDGSPQSNASSGRVSGVTLQEVPPTSGKPLLNTASAIAGQSLLPA
TSEMQSSHLGTCSEIINNQLSNIIPLVGGEDLHPGSLSASDMIMPELSQLQGILPENNTD
VIGCDSFMDTIAVEGKMGLDIGSLSPGADIDWQSGLLDEIQEFPSVGDPFWEKFLQSPSS
PDAAMDDDISNTSETKPQINGWDKTQNMEHLTEQMGATNIKQQKHMI
>Q9ZNT9
MTLKAEVVENFSCPFCLIPCGGHEGLQLHLKSSHDAFKFEFYRAEKDHGPEVDVSVKSDT
IKFGVLKDDVGNPQLSPLTFCSKNRNQRRQRDDSNNVKKLNVLLMELDLDDLPRGTENDS
THVNDDNVSSPPRAHSSEKISDILTTTQLAIAESSEPKVPHVNDGNVSSPPRAHSSAEKN
ESTHVNDDDDVSSPPRAHSLEKNESTHVNEDNISSPPKAHSSKKNESTHMNDEDVSFPPR
TRSSKETSDILTTTQPAIVEPSEPKVRRVSRRKQLYAKRYKARETQPAIAESSEPKVLHV
NDENVSSPPEAHSLEKASDILTTTQPAIAESSEPKVPHVNDENVSSTPRAHSSKKNKSTR
KNVDNVPSPPKTRSSKKTSDILTTTQPTIAESSEPKVRHVNDDNVSSTPRAHSSKKNKST
RKNDDNIPSPPKTRSSKKTSNILATTQPAKAEPSEPKVTRVSRRKELHAERCEAKRLERL
KGRQFYHSQTMQPITFEQVMSNEDSENETDDYALDISERLRLERLVGVSKEEKRYMYLWN
IFVRKQRVIADGHVPWACEEFAKLHKEEMKNSSSFDWWWRMFRIKLWNNGLICAKTFHKC
TTILLSNSDEAGQFTSGSAANANNQQSMEVDE
>Q8LEA8
MAESVFSCIPEDVVFNIFFKLQDDPRNWARLACVCTKFSSIVRNVCCKTQCYSAIPTVIS
DLLPLPPSAAASASSSTAADSSLTPPGGWASLYKLAVCCPGLFHAGILLENSDFGLEREL
GPDQNLDPKPTTTDLALNDEEVSKPVGSGLETTSFWSLYDDLYTDTIPAPPPEDSIDDQE
EEIETSEIRPGRDLPVRKRRKICRSLGSHLASGGWNLSREQGNKLLASRFRGDCLYICNW
PGCIHVEEKRNYMLFRGVFKDFKRSRVWRTINDGNRSKTSGLKCAFCLCDETWDLHSSFC
LRRVFGFHDDGEPVVRAYVCENGHVSGAWTALPLYT
>P40280
MDSTGTGAGGKGKKGAAGRKVGGPRKKSVSRSVKAGLQFPVGRIGRYLKKGRYAQXVGTG
APVYLAAVLEYLAAEVLELAGNAARDNKKTRIIPRHVLLAIRNDEELGKLLGGVTIAHGG

VLPNINPVLLPKKTAEKASSGGSKEAKSPKKAAKSPKKA
>Q8W1K8
MARGPGDTDMDEASADAAIPSSTPNPTVAFRCTHALSGHTKAVAAVKFSPDGSLLASGSA
DRTVALWDAATGARVNTLAGHSCGVSDVAWNPNGRYLATAADDHSLKLWDAETGACLRTL
TGHTNYVFCCNFDGAAGHLLASGSFDETLRLWDVRSGRCLREVPAHSDPVTSAAFSYDGS
MVVTSSLDGLIRLWDTQTGHCLKTLFDRDSPPVSFAAFTPNAKYVLCNTLDGRAKLWDYA
AGRTRRTYAGGHVNTQFCISSGFLGGSSSASFDLGCSMVVTGSEDGSLAAYDISTGHVVG
RGAAAAAAAEGGGDEGSAAAAAAGGVAGGHTAAVLSVNVHPSAPLVATGGHHPDNSVRVW
AASRTEPAAA
>O24454
MGAAGSKLEKALGDQFPEGERYFGFENFGNTCYCNSVLQALYFCVPFREQLLEYYTSNKS
VADAEENLMTCLADLFSQISSQKKKTGVIAPKRFVQRLKKQNELFRSYMHQDAHEFLNYL
LNEVVDILEKEAKATKTEHETSSSSSPEKIANGLKVPQANGVVHKEPIVTWVHNIFQGIL
TNETRCLRCETVTARDETFLDLSLDIEQNSSITSCLKNFSSTETLHAEDKFFCDKCCSLQ
EAQKRMKIKKPPHILVIHLKRFKYIEQLGRYKKLSYRVVFPLELKLSNTVEPYADVEYSL
FAVVVHVGSGPNHGHYVSLVKSHNHWLFFDDENVEMIEESAVQTFFGSSQEYSSNTDHGY
ILFYESLGPTK
>P14624
MQIFVKTLTGKTITLEVESSDTIENVKAKIQDKEGIPPDQQRLIFAGKQLEDGRTLADYN
IQKESTLHLVLRLRGG
>Q9FUY2
MSQTNWEADKMLDVYIHDYLVKRDLKATAQAFQAEGKVSSDPVAIDAPGGFLFEWWSVFW
DIFIARTNEKHSEVAASYIETQMIKAREQQLQQSQHPQVSQQQQQQQQQQIQMQQLLLQR
AQQQQQQQQQHHHHQQQQQQQQQQQQQQQQQQQQQHQNQPPSQQQQQQSTPQHQQQPTPQ
QQPQRRDGSHLANGSANGLVGNNSEPVMRQNPGSGSSLASKAYEERVKMPTQRESLDEAA
MKRFGDNVGQLLDPSHASILKSAAASGQPAGQVLHSTSGGMSPQVQTRNQQLPGSAVDIK
SEINPVLTPRTAVPEGSLIGIPGSNQGSNNLTLKGWPLTGFDQLRSGLLQQQKPFMQSQS
FHQLNMLTPQHQQQLMLAQQNLNSQSVSEENRRLKMLLNNRSMTLGKDGLGSSVGDVLPN
VGSSLQPGGSLLPRGDTDMLLKLKMALLQQQQQNQQQGGGNPPQPQPQPQPLNQLALTNP
QPQSSNHSIHQQEKLGGGGSITMDGSISNSFRGNEQVLKNQSGRKRKQPVSSSGPANSSG
TANTAGPSPSSAPSTPSTHTPGDVISMPNLPHSGGSSKSMMMFGTEGTGTLTSPSNQLAD
MDRFVEDGSLDDNVESFLSQEDGDQRDAVTRCMDVSKGFTFTEVNSVRASTTKVTCCHFS
SDGKMLASAGHDKKAVLWYTDTMKPKTTLEEHTAMITDIRFSPSQLRLATSSFDKTVRVW
DADNKGYSLRTFMGHSSMVTSLDFHPIKDDLICSCDNDNEIRYWSINNGSCTRVYKGGST
QIRFQPRVGKYLAASSANLVNVLDVETQAIRHSLQGHANPINSVCWDPSGDFLASVSEDM
VKVWTLGTGSEGECVHELSCNGNKFQSCVFHPAYPSLLVIGCYQSLELWNMSENKTMTLP
AHEGLITSLAVSTATGLVASASHDKLVKLWK
>P62787
MSGRGKGGKGLGKGGAKRHRKVLRDNIQGITKPAIRRLARRGGVKRISGLIYEETRGVLK
IFLENVIRDAVTYTEHARRKTVTAMDVVYALKRQGRTLYGFGG
>Q67Y93
MVRKYRKAKGIVEAGVSSTYMQLRSRRIVYVRSEKSSSVSVVGDNGVSSSCSGSNEYKKK
ELIHLEEEDKDGDTETSTYRRGTKRKLFENLREEEKEELSKSMENYSSEFESAVKESLDC
CCSGRKTMEETVTAEEEEKAKLMTEMPTESEIEDFFVEAEKQLKEKFKKKYNFDFEKEKP

LEGRYEWVKLE
>Q67EU8
MSSAAQQQQKAAAAEQEEVEHGPFPIEQLQASGIAALDVKKLKDSGLHTVEAVAYTPRKD
LLQIKGISEAKADKIIEAASKIVPLGFTSASQLHAQRLEIIQVTTGSRELDKILEGGIET
GSITEIYGEFRSGKTQLCHTPCVTCQLPLDQGGGEGKALYIDAEGTFRPQRLLQIADRFG
LNGADVLENVAYARAYNTDHQSRLLLEAASMMIETRFALMVVDSATALYRTDFSGRGELS
ARQMHMAKFLRSLQKLADEFGVAVVITNQVVAQVDGSAMFAGPQFKPIGGNIMAHASTTR
LALRKGRGEERICKVISSPCLAEAEARFQLASEGIADVKD
>P84634
MRDEVDLSLTIPSKLLGKRDREQKNCEEEKNKNKKAKKQQKDPILLHTSAATHKFLPPPL
TMPYSEIGDDLRSLDFDHADVSSDLHLTSSSSVSSFSSSSSSLFSAAGTDDPSPKMEKDP
RKIARRYQVELCKKATEENVIVYLGTGCGKTHIAVMLIYELGHLVLSPKKSVCIFLAPTV
ALVEQQAKVIADSVNFKVAIHCGGKRIVKSHSEWEREIAANEVLVMTPQILLHNLQHCFI
KMECISLLIFDECHHAQQQSNHPYAEIMKVFYKSESLQRPRIFGMTASPVVGKDGPPLDS
FTYFSGSFQSENLSKSINSLENLLNAKVYSVESNVQLDGFVSSPLVKVYYYRSALSDASQ
STIRYENMLEDIKQRRANVYCLRFLEAAKIQLNSDHNVQDEPVGKNPKSKICDTYLSMAA
EALSSGVAKDENASDLLSLAALKEPLFSRKLVQLIKILSVFRLEPHMKCIIFVNRIVTAR
TLSCILNNLELLRSWKSDFLVGLSSGLKSMSRRSMETILKRFQSKELNLLVATKVGEEGL
DIQTCCLVIRYDLPETVTSFIQSRGRARMPQSEYAFLVDSGNEKEMDLIENFKVNEDRMN
LEITYRSSEETCPRLDEELYKVHETGACISGGSSISLLYKYCSRLPHDEFFQPKPEFQFK
PVDEFGGTICRITLPANAPISEIESSLLPSTEAAKKDACLKAVHELHNLGVLNDFLLPDS
KDEIEDELSDDEFDFDNIKGEGCSRGDLYEMRVPVLFKQKWDPSTSCVNLHSYYIMFVPH
PADRIYKKFGFFMKSPLPVEAETMDIDLHLAHQRSVSVKIFPSGVTEFDNDEIRLAELFQ
EIALKVLFERGELIPDFVPLELQDSSRTSKSTFYLLLPLCLHDGESVISVDWVTIRNCLS
SPIFKTPSVLVEDIFPPSGSHLKLANGCWNIDDVKNSLVFTTYSKQFYFVADICHGRNGF
SPVKESSTKSHVESIYKLTEPQELDEYFIEIPPELSHLKIKGLSKDIGSSLSLLPSIMHR
MENLLVAIELKHVLSASIPEIAEVSGHRVLEALTTEKCHERLSLERLEVLGDAFLKFAVS
RHLFLHHDSLDEGELTRRRSNVYIRDQALDPTQFFAFGHPCRVTCDEVASKEVHSLNRDL
GILESNTGEIRCSKGHHWLYKKTIADVVEALVGAFLVDSGFKGAVKFLKWIGVNVDFESL
QVQDACIASRRYLPLTTRNNLETLENQLDYKFLHKGLLVQAFIHPSYNRHGGGCYQRLEF
LGDAVLDYLMTSYFFTVFPKLKPGQLTDLRSLSVNNEALANVAVSFSLKRFLFCESIYLH
EVIEDYTNFLASSPLASGQSEGPRCPKISPIKELIELCQSYKWDREISATKKDGAFTVEL
KVTKNGCCLTVSATGRNKREGTKKAAQLMITNLKAHENITTSHPLEDVLKNGIRNEAKLI
GYNEDPIDVVDLVGLDVENLNILETFGGNSERSSSYVIRRGLPQAPSKTEDRLPQKAIIK
AGGPSSKTAKSLLHETCVANCWKPPHFECCEEEGPGHLKSFVYKVILEVEDAPNMTLECY
GEARATKKGAAEHAAQAAIWCLKHSGFLC
>Q9LG26
MRRHKRWPLRSLVCSFSSSAAETVTTSTAASATAAFPLKHVTRSNFETTLNDLRSLVKAA
DFVAIDLEMTGVTSAPWRDSLEFDRYDVRYLKVKDSAEKFAVVQFGVCPFRWDSRTQSFV
SYPHNFFVFPRQELTFDPPAHEFLCQTTSMDFLAKYQFDFNTCIHEGISYLSRREEEEAS
KRLKMLHGEDGIDSSGETEELKLVRLADVLFAARMEKLLNEWRSGLLHGGNASSEFPRIS
NGSNQSMETVFHHMRPALSLKGFTSHQLRVLNSVLRKHFGDLVYIHSNDKSSSSRDIVVY
TDSDSDKENLMKEAKDERKRLAERKIQSAIGFRQVIDLLASEKKLIVGHNCFLDIAHVYS
KFVGPLPSTAEKFVASINSHFPYIVDTKILLNVNPMLHQRMKKSSTSLSSAFSSLCPQIE

FSSRSSDSFLQQRVNIDVEIDNVRCSNWNAGGKHEAGYDAFMTGCIFAQACNHLGFDFKQ
HSQLDDFAQNEKLEKYINRLYLSWTRGDIIDLRTGHSNADNWRVSKFKYENIVLIWNFPR
KLKARGIKECICKAFGSASVTSVYHVDDSAVFVLFKNSELVWDFLALKRQLESSDGPVSV
LHPLSKILEGGNTGAADYEAYKEICSSHVSEVMFSDQAETVGVKSRTRPNAQCETETREE
NTVTVTHKASDLIDAFLANRVEVETATSN
>Q9SCJ9
MSRPNTRNKNKRQRPDAVDSSSQILRKIHEANDVTDDDINQLFMIWKPVCQGCRVNTRDN
PNCFCGLVPPLNGSRKSGLWQKTSEIIQSLGPDPTLDRRDSESTPAGLTNLGATCYANSI
LQCLYMNTAFREGVFSVEVHVLKQNPVLDQIARLFAQLHASQKSFVDSDAFVKTLELDNG
VQQDTHEFLTLLLSLLERCLLHSGVKAKTIVQDLFSGSVSHVTTCSKCGRDSEASSKMED
FYALELNVKGLKSLDASLNDYLSLEQLNGDNQYFCGSCNARVDATRCIKLRTLPPVITFQ
LKRCIFLPKTTAKKKITSSFSFPQVLDMGSRLAESSQNKLTYDLSAVLIHKGSAVNSGHY
VAHIKDEKTGLWWEFDDEHVSELGKRPCNEASSSTPQSESNGTASSGNITDGIQSGSSDC
RSAIKSEVFSSSDAYMLMYSLRCDKQENQEGQKENPIDITKGEVKQLKGGYLPKHLSEWI
NNMNAVFLESCKQYNLRKEKELNALTERRQEVRTILSEAAVQSLEEQYFWISTDWLRLWA
DTTLPPALDNTPLLCSHGKVHASKVNCMKRISELAWIKLESKFNGGPKLGKGDYCRDCLM
DGARMVVSSDSYRDRRTFMKSIANDVLSGKCEDGMYYISRAWLQQWIKRKNLDAPTEADA
GPTNAITCNHGELMPEQAPGAKRVVVPENFWSFLFEDALKVMSEDTLDCTCFPVDSSQCC
HCTEVLSEVACFEDSLRTLKVKQRQNHEKLATGKGIPLTPQSRYFLLPSPWLVQWRIYIN
MTGKNSSSAPEPERLDGVINTLKCKKHTRLLERLPELVCRRGSYFQKNPSTDKLTIIPEL
DWKYFCDEWGGLMENGISAFIEVGNTDQSSSPDVIDLEKDSSPDDNMDVDAQQLILRASP
EICEECIGERESCELMQKLSYSEGDVFVCFVRGKGAPKAMLEASDSSFEVDRRTSKRSRR
TNYGNLTSLKVSATTTVYQLKMMIWELLEVMKENQELHKGSKVIDQESATLADMNIFPGD
RLWVRDTEMHEHRDIADELCEKKPGAQDIEEGFRGTLLTGNISSEAC
>Q9C801
MKLSFSLPSKSKPKVTATTADGNNAVDDGTSKEFVTEFDPSKTLANSIPKYVIPPIENTW
RPHKKMKNLDLPLQSGNAGSGLEFEPEVPLPGTEKPDNISYGLNLRQKVKDDSIGGDAVE
ERKVSMGEQLMLQSLRRDLMSLADDPTLEDFESVPVDGFGAALMAGYGWKPGKGIGKNAK
EDVEIKEYKKWTAKEGLGFDPDRSKVVDVKAKVKESVKLDKKGVGINGGDVFFVGKEVRI
IAGRDVGLKGKIVEKPGSDFFVIKISGSEEEVKVGVNEVADLGSKEEEKCLKKLKDLQLN
DREKDKKTSGRGRGAERGSRSEVRASEKQDRGQTRERKVKPSWLRSHIKVRIVSKDWKGG
RLYLKKGKVVDVVGPTTCDITMDETQELVQGVDQELLETALPRRGGPVLVLSGKHKGVYG
NLVEKDLDKETGVVRDLDNHKMLDVRLDQVAEYMGDMDDIEY
>Q9C519
MDRGWSGLTLDSSSLDLLNPNRISHKNHRRFSNPLAMSRIDEEDDQKTRISTNGSEFRFP
VSLSGIRDREDEDFSSGVAGDNDREVPGEVDFFSDKKSRVCREDDEGFRVKKEEQDDRTD
VNTGLNLRTTGNTKSDESMIDDGESSEMEDKRAKNELVKLQDELKKMTMDNQKLRELLTQ
VSNSYTSLQMHLVSLMQQQQQQNNKVIEAAEKPEETIVPRQFIDLGPTRAVGEAEDVSNS
SSEDRTRSGGSSAAERRSNGKRLGREESPETESNKIQKVNSTTPTTFDQTAEATMRKARV
SVRARSEAPMISDGCQWRKYGQKMAKGNPCPRAYYRCTMATGCPVRKQVQRCAEDRSILI
TTYEGNHNHPLPPAAVAMASTTTAAANMLLSGSMSSHDGMMNPTNLLARAVLPCSTSMAT
ISASAPFPTVTLDLTHSPPPPNGSNPSSSAATNNNHNSLMQRPQQQQQQMTNLPPGMLPH
VIGQALYNQSKFSGLQFSGGSPSTAAFSQSHAVADTITALTADPNFTAALAAVISSMING
TNHHDGEGNNKNQ

>P12959
MEHVISMEEILGPFWELLPPPAPEPEREQPPVTGIVVGSVIDVAAAGHGDGDMMDQQHAT
EWTFERLLEEEALTTSTPPPVVVVPNSCCSGALNADRPPVMEEAVTMAPAAVSSAVVGDP
MEYNAILRRKLEEDLEAFKMWRADSSVVTSDQRSQGSNNHTGGSSIRNNPVQNKLMNGED
PINNNHAQTAGLGVRLATSSSSRDPSPSDEDMDGEVEILGFKMPTEERVRKKESNRESAR
RSRYRKAAHLKELEDQVAQLKAENSCLLRRIAALNQKYNDANVDNRVLRADMETLRAKVK
MGEDSLKRVIEMSSSVPSSMPISAPTPSSDAPVPPPPIRDSIVGYFSATAADDDASVGNG
FLRLQAHQEPASMVVGGTLSATEMNRVAAATHCAGAMELIQTAMGSMPPTSASGSTPPPQ
IMSCWVQMGPYTWTCIRHCGFRDRWEHFICRRR
>P26356
MAAAAVDPMVLGLGTSGGASGSGVVGGGVGRAGGGGAVMEGAQPVDLARHPSGIVPVLQN
IVSTVNLDCRLDLKQIALQARNAEYNPKRFAAVIMRIRDPKTTALIFASGKMVCTGAKSE
EHSKLAARKYARIVQKLGFPATFKDFKIQNIVASCDVKFPIRLEGLAYSHGAFSSYEPEL
FPGLIYRMKQPKIVLLVFVSGKIVLTGAKVRDEIYAAFENIYPVLTEYRKSQQ
>Q6Q4D0
MGRLDVAAAKRAYRKAEEVGDRREQARWANNVGDILKNHGEYVDALKWFRIDYDISVKYL
PGKDLLPTCQSLGEIYLRLENFEEALIYQKKHLQLAEEANDTVEKQRACTQLGRTYHEMF
LKSEDDCEAIQSAKKYFKKAMELAQILKEKPPPGESSGFLEEYINAHNNIGMLDLDLDNP
EAARTILKKGLQICDEEEVREYDAARSRLHHNLGNVFMALRSWDEAKKHIEMDINICHKI
NHVQGEAKGYINLAELHNKTQKYIDALLCYGKASSLAKSMQDESALVEQIEHNTKIVKKS
MKVMEELREEELMLKKLSAEMTDAKGTSEERKSMLQVNACLGSLIDKSSMVFAWLKHLQY
SKRKKKISDELCDKEKLSDAFMIVGESYQNLRNFRKSLKWFIRSYEGHEAIGNLEGQALA
KINIGNGLDCIGEWTGALQAYEEGYRIALKANLPSIQLSALEDIHYIHMMRFGNAQKASE
LKETIQNLKESEHAEKAECSTQDECSETDSEGHANVSNDRPNACSSPQTPNSLRSERLAD
LDEANDDVPLISFLQPGKRLFKRKQVSGKQDADTDQTKKDFSVVADSQQTVAGRKRIRVI
LSDDESETEYELGCPKDSSHKVLRQNEEVSEESMYFDGAINYTDNRAIQDNVEEGSCSYT
PLHPIKVAPNVSNCRSLSNNIAVETTGRRKKGSQCDVGDSNGTSCKTGAALVNFHAYSKT
EDRKIKIEIENEHIALDSCSHDDESVKVELTCLYYLQLPDDEKSKGLLPIIHHLEYGGRV
LKPLELYAILRDSSENVVIEASVDGWVHKRLMKLYMDCCQSLSEKPSMKLLKKLYISEVE
DDINVSECELQDISAAPLLCALHVHNIAMLDLSHNMLGNGTMEKLKQLFASSSQMYGALT
LDLHCNRFGPTALFQICECPVLFTRLEVLNVSRNRLTDACGSYLSTIVKNCRALYSLNVE
HCSLTSRTIQKVANALDSKSGLSQLCIGYNNPVSGSSIQNLLAKLATLSSFAELSMNGIK
LSSQVVDSLYALVKTPSLSKLLVGSSGIGTDGAIKVTESLCYQKEETVKLDLSCCGLASS
FFIKLNQDVTLTSSILEFNVGGNPITEEGISALGELLRNPCSNIKVLILSKCHLKLAGLL
CIIQALSDNKNLEELNLSDNAKIEDETVFGQPVKERSVMVEQEHGTCKSVTSMDKEQELC
ETNMECDDLEVADSEDEQIEEGTATSSSLSLPRKNHIVKELSTALSMANQLKILDLSNNG
FSVEALETLYMSWSSSSSRTGIAQRHVKEETVHFYVEGKMCCGVKSCCRKD
>P93022
MKAPSSNGVSPNPVEGERRNINSELWHACAGPLISLPPAGSLVVYFPQGHSEQVAASMQK
QTDFIPSYPNLPSKLICMLHNVTLNADPETDEVYAQMTLQPVNKYDRDALLASDMGLKLN
RQPNEFFCKTLTASDTSTHGGFSVPRRAAEKIFPALDFSMQPPCQELVAKDIHDNTWTFR
HIYRGQPKRHLLTTGWSVFVSTKRLFAGDSVLFIRDGKAQLLLGIRRANRQQPALSSSVI
SSDSMHIGVLAAAAHANANNSPFTIFYNPRAAPAEFVVPLAKYTKAMYAQVSLGMRFRMI
FETEECGVRRYMGTVTGISDLDPVRWKNSQWRNLQIGWDESAAGDRPSRVSVWDIEPVLT

PFYICPPPFFRPRFSGQPGMPDDETDMESALKRAMPWLDNSLEMKDPSSTIFPGLSLVQW
MNMQQQNGQLPSAAAQPGFFPSMLSPTAALHNNLGGTDDPSKLLSFQTPHGGISSSNLQF
NKQNQQAPMSQLPQPPTTLSQQQQLQQLLHSSLNHQQQQSQSQQQQQQQQLLQQQQQLQS
QQHSNNNQSQSQQQQQLLQQQQQQQLQQQHQQPLQQQTQQQQLRTQPLQSHSHPQPQQLQ
QHKLQQLQVPQNQLYNGQQAAQQHQSQQASTHHLQPQLVSGSMASSVITPPSSSLNQSFQ
QQQQQSKQLQQAHHHLGASTSQSSVIETSKSSSNLMSAPPQETQFSRQVEQQQPPGLNGQ
NQQTLLQQKAHQAQAQQIFQQSLLEQPHIQFQLLQRLQQQQQQQFLSPQSQLPHHQLQSQ
QLQQLPTLSQGHQFPSSCTNNGLSTLQPPQMLVSRPQEKQNPPVGGGVKAYSGITDGGDA
PSSSTSPSTNNCQISSSGFLNRSQSGPAILIPDAAIDMSGNLVQDLYSKSDMRLKQELVG
QQKSKASLTDHQLEASASGTSYGLDGGENNRQQNFLAPTFGLDGDSRNSLLGGANVDNGF
VPDTLLSRGYDSQKDLQNMLSNYGGVTNDIGTEMSTSAVRTQSFGVPNVPAISNDLAVND
AGVLGGGLWPAQTQRMRTYTKVQKRGSVGRSIDVNRYRGYDELRHDLARMFGIEGQLEDP
QTSDWKLVYVDHENDILLVGDDPWEEFVNCVQSIKILSSAEVQQMSLDGNFAGVPVTNQA
CSGGDSGNAWRGHYDDNSATSFNR
>Q8GYJ3
MGKYIRKSKIDGAGAGAGGGGGGGGGGGESSIALMDVVSPSSSSSLGVLTRAKSLALQQQQ
QRCLLQKPSSPSSLPPTSASPNPPSKQKMKKKQQQMNDCGSYLQLRSRRLQKKPPIVVIR
STKRRKQQRRNETCGRNPNPRSNLDSIRGDGSRSDSVSESVVFGKDKDLISEINKDPTFG
QNFFDLEEEHTQSFNRTTRESTPCSLIRRPEIMTTPGSSTKLNICVSESNQREDSLSRSH
RRRPTTPEMDEFFSGAEEEQQKQFIEKYNFDPVNEQPLPGRFEWTKVDD
>Q8S3N1
METDSIDSVIDDDEIHQKHQFSSTKSQGGATVVISPATSVYELLECPVCTNSMYPPIHQC
HNGHTLCSTCKSRVHNRCPTCRQELGDIRCLALEKVAESLELPCKYYNLGCLGIFPYYSK
LKHESQCNFRPYSCPYAGSECAAVGDITFLVAHLRDDHKVDMHTGCTFNHRYVKSNPREV
ENATWMLTVFQCFGQYFCLHFEAFQLGMAPVYMAFLRFMGDEDDARNYTYSLEVGGSGRK
QTWEGTPRSVRDSHRKVRDSHDGLIIQRNMALFFSGGDKKELKLRVTGRIWKEQQNPDSG
VCITSMCSS
>Q08655
MEEEKHHHHHLFHHKDKAEEGPVDYEKEIKHHKHLEQIGKLGTVAAGAYALHEKHEAKKD
PEHAHKHKIEEEIAAAAAVGAGGFAFHEHHEKKDAKKEEKKKLRGDTTISSKLLF
>Q680Q4
MDLEANCKEKLSYFRIKELKDVLTQLGLSKQGKKQELVDRILTLLSDEQAARLLSKKNTV
AKEAVAKLVDDTYRKMQVSGASDLASKGQVSSDTSNLKVKGEPEDPFQPEIKVRCVCGNS
LETDSMIQCEDPRCHVWQHVGCVILPDKPMDGNPPLPESFYCEICRLTRADPFWVTVAHP
LSPVRLTATTIPNDGASTMQSVERTFQITRADKDLLAKPEYDVQAWCMLLNDKVLFRMQW
PQYADLQVNGVPVRAINRPGGQLLGVNGRDDGPIITSCIRDGVNRISLSGGDVRIFCFGV
RLVKRRTLQQVLNLIPEEGKGETFEDALARVRRCIGGGGGDDNADSDSDIEVVADFFGVN
LRCPMSGSRIKVAGRFLPCVHMGCFDLDVFVELNQRSRKWQCPICLKNYSVEHVIVDPYF
NRITSKMKHCDEEVTEIEVKPDGSWRVKFKRESERRELGELSQWHAPDGSLCPSAVDIKR
KMEMLPVKQEGYSDGPAPLKLGIRKNRNGIWEVSKPNTNGLSSSNRQEKVGYQEKNIIPM
SSSATGSGRDGDDASVNQDAIGTFDFVANGMELDSISMNVDSGYNFPDRNQSGEGGNNEV
IVLSDSDDENDLVITPGPAYSGCQTDGGLTFPLNPPGIINSYNEDPHSIAGGSSGLGLFN
DDDEFDTPLWSFPSETPEAPGFQLFRSDADVSGGLVGLHHHSPLNCSPEINGGYTMAPET
SMASVPVVPGSTGRSEANDGLVDNPLAFGRDDPSLQIFLPTKPDASAQSGFKNQADMSNG

LRSEDWISLRLGDSASGNHGDPATTNGINSSHQMSTREGSMDTTTETASLLLGMNDSRQD
KAKKQRSDNPFSFPRQKRSNNEQDHQTRHRSLNKICIILCAGKN
>Q9SF32
MVKKAKWLKNVKKAFSPDSKKLKHESVECQDSVISYPVLIATSRSSSPQFEVRVDEVNYE
QKKNLYPPSSDSVTATVAHVLVDSPPSSPESVHQAIVVNRFAGKSKEEAAAILIQSTFRG
HLARRESQVMRGQERLKLLMEGSVVQRQAAITLKCMQTLSRVQSQIRSRRIRMSEENQAR
HKQLLQKHAKELGGLKNGGNWNYSNQSKEQVEAGMLHKYEATMRRERALAYAFTHQQNLK
SFSKTANPMFMDPSNPTWGWSWLERWMAGRPWESSEKEQNTTNNDNSSVKNSTNRNSQGG
ETAKSSNRNKLNSSTKPNTPSASSTATRNPRKKRPIPSSIKSKSSDDEAKSSERNRRPSI
ARPSVSDDETLSSSTARRSSNLIPTTKSARGKPKSQTSSRVAVTTSTTEESSILPEKAPA
KKRLSTSASPAPKPRRSSAPPKVEKGVLKAERTP
>Q8S307
MTSDGATSTSAAAAAAAAAAARRKPSWRERENNRRRERRRAVAAKIYTGLRAQGDYNLP
KHCDNNEVLKALCVEAGWVVEEDGTTYRKGCKPLPGEIAGTSSRVTPYSSQNQSPLSSAF
QSPIPSYQVSPSSSSFPSPSRGEPNNNMSSTFFPFLRNGGIPSSLPSLRISNSCPVTPPV
SSPTSKNPKPLPNWESIAKQSMAIAKQSMASFNYPFYAVSAPASPTHRHQFHTPATIPEC
DESDSSTVDSGHWISFQKFAQQQPFSASMVPTSPTFNLVKPAPQQMSPNTAAFQEIGQSS
EFKFENSQVKPWEGERIHDVGMEDLELTLGNGKARG
>O24606
MMFNEMGMCGNMDFFSSGSLGEVDFCPVPQAEPDSIVEDDYTDDEIDVDELERRMWRDKM
RLKRLKEQDKGKEGVDAAKQRQSQEQARRKKMSRAQDGILKYMLKMMEVCKAQGFVYGII
PENGKPVTGASDNLREWWKDKVRFDRNGPAAITKYQAENNIPGIHEGNNPIGPTPHTLQE
LQDTTLGSLLSALMQHCDPPQRRFPLEKGVPPPWWPNGKEDWWPQLGLPKDQGPAPYKKP
HDLKKAWKVGVLTAVIKHMFPDIAKIRKLVRQSKCLQDKMTAKESATWLAIINQEESLAR
ELYPESCPPLSLSGGSCSLLMNDCSQYDVEGFEKESHYEVEELKPEKVMNSSNFGMVAKM
HDFPVKEEVPAGNSEFMRKRKPNRDLNTIMDRTVFTCENLGCAHSEISRGFLDRNSRDNH
QLACPHRDSRLPYGAAPSRFHVNEVKPVVGFPQPRPVNSVAQPIDLTGIVPEDGQKMISE
LMSMYDRNVQSNQTSMVMENQSVSLLQPTVHNHQEHLQFPGNMVEGSFFEDLNIPNRANN
NNSSNNQTFFQGNNNNNNVFKFDTADHNNFEAAHNNNNNSSGNRFQLVFDSTPFDMASFD
YRDDMSMPGVVGTMDGMQQKQQDVSIWF
>Q39255
MSAKKIVLKSSDGESFEVEEAVALESQTIAHMVEDDCVDNGVPLPNVTSKILAKVIEYCK
RHVEAAASKAEAVEGAATSDDDLKAWDADFMKIDQATLFELILAANYLNIKNLLDLTCQT
VADMIKGKTPEEIRTTFNIKNDFTPEEEEEVRRENQWAFE
>P45951
MNNVLQFGLQSSAIYVAKFLVVPLRSLRVGSSFVGVGVGTRSFNKRLMSNATAFSINNSK
RKELKIPGAAIDQNCHQMGSDTDRDEMGTLQDDRKEIEAMTVQELRSTLRKLGVPVKGRK
QELISTLRLHMDSNLPDQKETSSSTRSDSVTIKRKISNREEPTEDECTNSEAYDIEHGEK
RVKQSTEKNLKAKVSAKAIAKEQKSLMRTGKQQIQSKEETSSTISSELLKTEEIISSPSQ
SEPWTVLAHKKPQKDWKAYNPKTMRPPPLPEGTKCVKVMTWNVNGLRGLLKFESFSALQL
AQRENFDILCLQETKLQVKDVEEIKKTLIDGYDHSFWSCSVSKLGYSGTAIISRIKPLSV
RYGTGLSGHDTEGRIVTAEFDSFYLINTYVPNSGDGLKRLSYRIEEWDRTLSNHIKELEK
SKPVVLTGDLNCAHEEIDIFNPAGNKRSAGFTIEERQSFGANLLDKGFVDTFRKQHPGVV
GYTYWGYRHGGRKTNKGWRLDYFLVSQSIAANVHDSYILPDINGSDHCPIGLILKL

>O24463

MDMISGSTAATSTPHNNQQAVMLSSPIIKEEARDPKQTRAMPQIGGSGERKPRPQLPEAL
KCPRCDSNNTKFCYYNNYSMSQPRYFCKACRRYWTHGGTLRNVPIGGGCRKNKHASRFVL
GSHTSSSSSATYAPLSPSTNASSSNMSINKHMMMVPNMTMPTPTTMGLFPNVLPTLMPTG
GGGGFDFTMDNQHRSLSFTPMSLPSQGPVPMLAAGGSEATPSFLEMLRGGIFHGSSSYNT
SLTMSGGNNGMDKPFSLPSYGAMCTNGLSGSTTNDARQLVGPQQDNKAIMKSSNNNNGVS
LLNLYWNKHNNNNNNNNNNNNNNNNNKGQ

>Q64MA3

MGVGGSFWDLLKPYARHEGAGYLRGRRVAVDLSFWVVSHSAAIRARSPHARLPHLRTLFF
RTLSLFSKMGAFPVFVVDGQPSPLKSQVRAARFFRGSGMDLAALPSTEAEASADALVQPR
NAKFTRYVEDCVELLEYLGMPVLRAKGEGEALCAQLNNQGHVDACITSDSDAFLFGAKTV
IKVLRSNCKEPFECYNMADIESGLGLKRKQMVAMALLVGSDHDLHGVPGFGPETALRFVQ
LFDEDNVLAKLYEIGKGVYPFIGVSAPNIDDLPSPSTKSLPRARSPHCSHCGHPGNKKNH
IKDGCNFCLVDSLENCVEKPAGFICECPSCDKARDLKVQRRNENWQIKVCKRIAAETNFP
NEEIINLYLNDDNLDNENGVPLLTWNKPDMEILVDFLSFKQNWEPAYIRQRMLPMLSTIY
LREMASSQSKSFLLYDQYKFHSIQRIKIRYGHPYYLVKWKRVTRSMISNDPPSKQTELEG
KNDKVEVLDGDDEVVDEEEEEPTMISETTELLDEPDVPQVLDDDKDCFLLTDEDIELVNA
AFPDEAQRFQEEQRLKEAKSIARKSKLNVAGFETPKGPRPSGVQLSIKEFYRSKKGLSGD
SGKDGSRKSSDVDLSKNLPKSVRRRLLFD

>Q8LGD5

MDPSEYFAGGNPSDQQNQKRQLQICGPRPSPLSVHKDSHKIKKPPKHPAPPPNRDQPPPY
IPREPVVIYAVSPKVVHATASEFMNVVQRLTGISSGVFLESGGGGDVSPAARLASTENAS
PRGGKEPAARDETVEINTAMEEAAEFGGYAPGILSPSPALLPTASTGIFSPMYHQGGMFS
PAIPLGLFSPAGFMSPFRSPGFTSLVASPTFADFFSHIWDQD

>Q9ZT50

MGLQGQLSDVSSDSIPLMLLSLLAVFINHLRSFLLRLTSKSNPNLPVDDVSIASGLANII
VLADQLSLNRLFSYRCGDGGGGGSDCVVCLSKLKEGEEVRKLECRHVFHKKCLEGWLHQF
NFTCPLCRSALVSDDCVSKTQRSVGRDLISCFSLH

>Q96301

MVGLEDDTERERSPVVENGFSNGSRSSSSSAGVLSPSRKVTQGNDTLSYANILRARNKFA
DALALYEAMLEKDSKNVEAHIGKGICLQTQNKGNLAFDCFSEAIRLDPHNACALTHCGIL
HKEEGRLVEAAESYQKALMADASYKPAAECLAIVLTDLGTSLKLAGNTQEGIQKYYEALK
IDPHYAPAYYNLGVVYSEMMQYDNALSCYEKAALERPMYAEAYCNMGVIYKNRGDLEMAI
TCYERCLAVSPNFEIAKNNMAIALTDLGTKVKLEGDVTQGVAYYKKALYYNWHYADAMYN
LGVAYGEMLKFDMAIVFYELAFHFNPHCAEACNNLGVLYKDRDNLDKAVECYQMALSIKP
NFAQSLNNLGVVYTVQGKMDAAASMIEKAILANPTYAEAFNNLGVLYRDAGNITMAIDAY
EECLKIDPDSRNAGQNRLLAMNYINEGLDDKLFEAHRDWGWRFTRLHPQYTSWDNLKDPE
RPITIGYISPDFFTHSVSYFIEAPLTHHDYTKYKVVVYSAVVKADAKTYRFRDKVLKKGG
VWKDIYGIDEKKIASMVREDKIDILVELTGHTANNKLGTMACRPAPVQVTWIGYPNTTGL
PTVDYRITDSLADPPDTKQKQVEELVRLPDCFLCYTPSPEAGPVCPTPALSNGFVTFGSF
NNLAKITPKVLQVWARILCAVPNSRLVVKCKPFCCDSIRQRFLTTLEQLGLESKRVDLLP
LILFNHDHMQAYSLMDISLDTFPYAGTTTTCESLYMGVPCVTMAGSVHAHNVGVSLLTKV
GLGHLVAKNEDEYVQLSVDLASDVTALSKLRMSLRDLMAGSPVCNGPSFAVGLESAYRNM
WKKYCKGEVPSLRRMEMLQKEVHDDPLISKDLGPSRVSVTGEATPSLKANGSAPVPSSLP

TQSPQLSKRMDSTS
>Q9FGN8
MFVKRNPIRETTAGKISSPSSPTLNVAVAHIRAGSYYEIDASILPQRSPENLKSIRVVMV
SKITASDVSLRYPSMFSLRSHFDYSRMNRNKPMKKRSGGGLLPVFDESHVMASELAGDLL
YRRIAPHELSMNRNSWGFWVSSSSRRNKFPRREVVSQPAYNTRLCRAASPEGKCSSELKS
GGMIKWGRRLRVQYQSRHIDTRKNKEGEESSRVKDEVYKEEEMEKEEDDDDGNEIGGTKQ
EAKEITNGNRKRKLIESSTERLAQKAKVYDQKKETQIVVYKRKSERKFIDRWSVERYKLA
ERNMLKVMKEKNAVFGNSILRPELRSEARKLIGDTGLLDHLLKHMAGKVAPGGQDRFMRK
HNADGAMEYWLESSDLIHIRKEAGVKDPYWTPPPGWKLGDNPSQDPVCAGEIRDIREELA
SLKRELKKLASKKEEEELVIMTTPNSCVTSQNDNLMTPAKEIYADLLKKKYKIEDQLVII
GETLRKMEEDMGWLKKTVDENYPKKPDSTETPLLLEDSPPIQTLEGEVKVVNKGNQITES
PQNREKGRKHDQQERSPLSLISNTGFRICRPVGMFAWPQLPALAAATDTNASSPSHRQAY
PSPFPVKPLAAKRPLGLTFPFTIIPEEAPKNLFNV
>Q38819
MGKENAVSRPFTRSLASALRASEVTSTTQNQQRVNTKRPALEDTRATGPNKRKKRAVLGE
ITNVNSNTAILEAKNSKQIKKGRGHGLASTSQLATSVTSEVTDLQSRTDAKVEVASNTAG
NLSVSKGTDNTADNCIEIWNSRLPPRPLGRSASTAEKSAVIGSSTVPDIPKFVDIDSDDK
DPLLCCLYAPEIHYNLRVSELKRRPLPDFMERIQKDVTQSMRGILVDWLVEVSEEYTLAS
DTLYLTVYLIDWFLHGNYVQRQQLQLLGITCMLIASKYEEISAPRIEEFCFITDNTYTRD
QVLEMENQVLKHFSFQIYTPTPKTFLRRFLRAAQASRLSPSLEVEFLASYLTELTLIDYH
FLKFLPSVVAASAVFLAKWTMDQSNHPWNPTLEHYTTYKASDLKASVHALQDLQLNTKGC
PLSAIRMKYRQEKYKSVAVLTSPKLLDTLF
>A8MQY1
MMKGLIGYRFSPTGEEVINHYLKNKLLGKYWLVDEAISEINILSHKPSKDLPKLARIQSE
DLEWYFFSPIEYTNPNKMKMKRTTGSGFWKPTGVDREIRDKRGNGVVIGIKKTLVYHEGK
SPHGVRTPWVMHEYHITCLPHHKRKYVVCQVKYKGEAAEISYEPSPSLVSDSHTVIAITG
EPEPELQVEQPGKENLLGMSVDDLIEPMNQQEEPQGPHLAPNDDEFIRGLRHVDRGTVEY
LFANEENMDGLSMNDLRIPMIVQQEDLSEWEGFNADTFFSDNNNNYNLNVHHQLTPYGDG
YLNAFSGYNEGNPPDHELVMQENRNDHMPRKPVTGTIDYSSDSGSDAGSISTTSYQGTSS
PNISVGSSSRHLSSCSSTDSCKDLQTCTDPSIISREIRELTQEVKQEIPRAVDAPMNNES
SLVKTEKKGLFIVEDAMERNRKKPRFIYLMKMIIGNIISVLLPVKRLIPVKKL
>P37218
MATEEPVIVNEVVEEQAAPETVKDEANPPAKSGKAKKETKAKKPAAPRKRSATPTHPPYF
EMIKDAIVTLKERTGSSQHAITKFIEEKQKSLPSNFKKLLLTQLKKFVASEKLVKVKNSY
KLPSGSKPAAAAVPAKKKPAAAKSKPAAKPKAAVKPKAKPAAKAKPAAKAKPAAKAKPAA
KAKPAAKAKPAAKAKPVAKAKPKAAAAAKPKAAVKPKAAPAKTKAAVKPNLKAKTTTAKV
AKTATRTTPSRKAAPKATPAKKEPVKKAPAKNVKSPAKKATPKRGRK
>Q84J70
MDSDSWSDRLASATRRYQLAFPSRSDTFLGFEEIDGEEEFREEFACPFCSDYFDIVSLCC
HIDEDHPMEAKNGVCPVCAVRVGVDMVAHITLQHANIFKMHRKRKPRRGGSYSTLSILRR
EFPDGNFQSLFGGSSCIVSSSSSSNVAADPLLSSFISPIADGFFTTESCISAETGPVKKT
TIQCLPEQNAKKTSLSAEDHKQKLKRSEFVRELLSSTILDDSL
>Q84JU6
MDTREINGFASAARSISLPTQPNYSSKPVQEALKHLASINLRELCNEAKVERCRATRDLA

SCGRFVNYVLNPCGHASLCTECCQRCDVCPICRSTLPKFGDRLRLRLYYECVEAGLISRT
HEEASQDSDEDEHQLAADVHRLYSLFDVAMNNNLISVVCHYITNVCMDETAVSSDPVIAF
LLDEVVVKDWVKRTFRSTLAELQEIYNLETKEMQAWLDKLLRCSKQVAGICSVLEVMESA
FKGSVSPQLQDVQTLRENIGKTKQHLDIMVWCIRHGFLDDVRSRYSNFTSWNALVGERKS
NAVKRAWPDAVDQSSDCSVQSASLFIEDALENLEREPEYSQEIGADLEVGRLQKDKRSFL
RSKIEGTSGSYPFENLRTAADMLFLHGGSDLVVAKQAIFLYYLFDRHWTTPEKYWKHTID
DFAATFGITRHSLLESFVFYLLDDHSEEALQEACRILPEICGPETYPKVAQVLLERDNPE
TALMVLRWSGRDGVSELVSIGEAVTALRVRVECGLLSEAFTYQRTLCLKVKENNLKNGAV
KHASDDLDIWSWTEWMEILVNEFCCLSIRRNLVDRIIELPWNPDEEKYLHRCLLDSATDD
PSSAVGSLLVVFYLQRYRYIQAYQVDLRLQKIEEAFVSDNQIGEEVMFRMRSQSHWRKEL
VDRAIDILPVIQQQQVRSGQFSEMEDASEGAKKSDLPDAPDMITSSVPFATTNSVFLQSA
NNARAREPVANNGSPFQPGHMIGNASHDLSHGRLFTNANRGQKSEVRSVTKNLKFGEMST
PFKDLNRARGNSQLQGKRTEESSPEVNVDRYIENNMSSPYLRRITANNPVTVKSSSNHLN
GSSQKPESTFFGTRMQPDKDNFVDLDDPMDMSSSLKDNNNNVLATESRNNSGGLRWRSDE
TSDDEDELTSFGSMPVKGRRRRRFAAR
>Q570C0
MQKRIALSFPEEVLEHVFSFIQLDKDRNSVSLVCKSWYEIERWCRRKVFIGNCYAVSPAT
VIRRFPKVRSVELKGKPHFADFNLVPDGWGGYVYPWIEAMSSSYTWLEEIRLKRMVVTDD
CLELIAKSFKNFKVLVLSSCEGFSTDGLAAIAATCRNLKELDLRESDVDDVSGHWLSHFP
DTYTSLVSLNISCLASEVSFSALERLVTRCPNLKSLKLNRAVPLEKLATLLQRAPQLEEL
GTGGYTAEVRPDVYSGLSVALSGCKELRCLSGFWDAVPAYLPAVYSVCSRLTTLNLSYAT
VQSYDLVKLLCQCPKLQRLWVLDYIEDAGLEVLASTCKDLRELRVFPSEPFVMEPNVALT
EQGLVSVSMGCPKLESVLYFCRQMTNAALITIARNRPNMTRFRLCIIEPKAPDYLTLEPL
DIGFGAIVEHCKDLRRLSLSGLLTDKVFEYIGTYAKKMEMLSVAFAGDSDLGMHHVLSGC
DSLRKLEIRDCPFGDKALLANASKLETMRSLWMSSCSVSFGACKLLGQKMPKLNVEVIDE
RGAPDSRPESCPVERVFIYRTVAGPRFDMPGFVWNMDQDSTMRFSRQIITTNGL
>Q6NPP4
MADRGSFGFAPRLDIKQLLSEAQHRWLRPAEICEILRNHQKFHIASEPPNRPPSGSLFLF
DRKVLRYFRKDGHNWRKKKDGKTVKEAHEKLKVGSIDVLHCYYAHGEDNENFQRRCYWML
EQDLMHIVFVHYLEVKGNRMSTSGTKENHSNSLSGTGSVNVDSTATRSSILSPLCEDADS
GDSRQASSSLQQNPEPQTVVPQIMHHQNASTINSYNTTSVLGNRDGWTSAHGNRVKGSNS
QRSGDVPAWDASFENSLARYQNLPYNAPLTQTQPSTFGLIPMEGKTEKGSLLTSEHLRNP
LQSQVNWQTPVQESVPLQKWPMDSHSGMTDATDLALFGQGAHENFGTFSSLLGSQDQQSS
SFQAPFTNNEAAYIPKLGPEDLIYEASANQTLPLRKALLKKEDSLKKVDSFSRWVSKELG
EMEDLQMQSSSGGIAWTSVECENAAAGSSLSPSLSEDQRFTMIDFWPKWTQTDSEVEVMV
IGTFLLSPQEVTSYSWSCMFGEVEVPADILVDGVLCCHAPPHEVGRVPFYITCSDRFSCS
EVREFDFLPGSTRKLNATDIYGANTIETSLHLRFENLLALRCSVQEHHIFENVGEKRRKI
SKIMLLKDEKEPPLPGTIEKDLTELEAKERLIREEFEDKLYLWLIHKVTEEGKGPNILDE
DGQGVLHLAAALGYDWAIKPILAAGVSINFRDANGWSALHWAAFSGREDTVAVLVSLGAD
AGALADPSPEHPLGKTAADLAYGNGHRGISGFLAESSLTSYLEKLTVDAKENSSADSSGA
KAVLTVAERTATPMSYGDVPETLSMKDSLTAVLNATQAADRLHQVFRMQSFQRKQLSELG
GDNKFDISDELAVSFAAAKTKKSGHSSGAVHAAAVQIQKKYRGWKKRKEFLLIRQRIVKI
QAHVRGHQVRKQYRAIIWSVGLLEKIILRWRRKGSGLRGFKRDTISKPTEPVCPAPQEDD
YDFLKEGRKQTEERLQKALTRVKSMAQYPEARAQYRRLLTVVEGFRENEASSSSALKNNT

EEAANYNEEDDLIDIDSLLDDDTFMSLAFE
>Q9C5X4
MACFSNETQIEIDVHDLVEAPIRYDSIESIYSIPSSALCCVNAVGSHSLMSKKVKAQKLP
MIEQFEIEGSGVSASDDCCRSDDYKLRIQRPEIVRVYYRRRKRPLRECLLDQAVAVKTES
VELDEIDCFEEKKRRKIGNCELVKSGMESIGLRRCKENNAFSGNKQNGSSRRKGSSSKNQ
DKATLASRSAKKWVRLSYDGVDPTSFIGLQCKVFWPLDALWYEGSIVGYSAERKRYTVKY
RDGCDEDIVFDREMIKFLVSREEMELLHLKFCTSNVTVDGRDYDEMVVLAATLDECQDFE
PGDIVWAKLAGHAMWPAVIVDESIIGERKGLNNKVSGGGSLLVQFFGTHDFARIKVKQAI
SFIKGLLSPSHLKCKQPRFEEGMQEAKMYLKAHRLPERMSQLQKGADSVDSDMANSTEEG
NSGGDLLNDGEVWLRPTEHVDFRHIIGDLLIINLGKVVTDSQFFKDENHIWPEGYTAMRK
FTSLTDHSASALYKMEVLRDAETKTHPLFIVTADSGEQFKGPTPSACWNKIYNRIKKVQN
SDSPNILGEELNGSGTDMFGLSNPEVIKLVQDLSKSRPSSHVSMCKNSLGRHQNQPTGYR
PVRVDWKDLDKCNVCHMDEEYENNLFLQCDKCRMMVHAKCYGELEPCDGALWLCNLCRPG
APDMPPRCCLCPVVGGAMKPTTDGRWAHLACAIWIPETCLSDVKKMEPIDGVNKVSKDRW
KLMCTICGVSYGACIQCSNNSCRVAYHPLCARAAGLCVELENDMSVEGEEADQCIRMLSF
CKRHRQTSTACLGSEDRIKSATHKTSEYLPPPNPSGCARTEPYNCFGRRGRKEPEALAAA
SSKRLFVENQPYVIGGYSRLEFSTYKSIHGSKVSQMNTPSNILSMAEKYRYMRETYRKRL
AFGKSGIHGFGIFAKLPHRAGDMMIEYTGELVRPSIADKREQLIYNSMVGAGTYMFRIDD
ERVIDATRTGSIAHLINHSCVPNCYSRVITVNGDEHIIIFAKRHIPKWEELTYDYRFFSI
GERLSCSCGFPGCRGVVNDTEAEEQHAKICVPRCDLIDWTAE
>Q84Y18
MPATAGRVRMPANNRVHSSAALQTHGIWQSAIGYDPYAPTSKEEPKTTQQKTEDPENSYA
SFQGLLALARITGSNNDEARGSCKKCGRVGHLTFQCRNFLSTKEDKEKDPGAIEAAVLSG
LEKIRRGVGKGEVEEVSSEEEEESESSDSDVDSEMERIIAERFGKKKGGSSVKKTSSVRK
KKKRVSDESDSDSDSGDRKRRRRSMKKRSSHKRRSLSESEDEEEGRSKRRKERRGRKRDE
DDSDESEDEDDRRVKRKSRKEKRRRRSRRNHSDDSDSESSEDDRRQKRRNKVAASSDSEA
NVSGDDVSRVGRGSSKRSEKKSRKRHHRKERE
>Q84VX3
MVKFAIINTLTVNETWAKLKSFGVMESSIEGSSESTTVTTSPSRRVRELLALCFSSVEEA
GGFQDFESFVTELVSCLDSLYENVALDANNELENDVIEEVLDEILKVLSSPQMDQDVIDA
LSFHLPKVTSKFADISSRCLQLVEEIVDRFVEACNPRDMLSILCEALDAARCYHSASTCS
TPLLHGLSKVFILIQRRHYEQLKVAVPIVLNVLKDISLETDVQVEDLFDKALGIASSIRD
VSSKLNNEEEAKVRCLLCLYVIQITAIISVSIRDKAASCIPLVIQLEPFLTSCGLTHLGL
ITGNDTEKLMSTVAGDDDEFITSFPDISLGASLLFICAKISHEVAEAANAVLGSVVDELQ
NNPVKRWQAYGMLKYILSSGDLLWEFKRHAIEFLLDITKGVTSSQCNDEQIDCSDYTPGI
YATLQAVTLLIMYAPDADLRKKTFEALKRVLSDIPAPHRFDVLRALVTNSRSPSMTAILL
GLVKDSMSKSSLQDTDCAAVDTHVIELVELVLRPPQGGPPLLPDQSDAILAALNLYRFAL
LFESRECEAGKERSKVGSDILSKKNLEKAYKEWLLPLRTLVSCSIAENLKEDHGQESSLD
DVGLLNPIELVLYRCIELVEEKLKSH
>Q38897
MARDQFYGHNNHHHQEQQHQMINQIQGFDETNQNPTDHHHYNHQIFGSNSNMGMMIDFSK
QQQIRMTSGSDHHHHHHQTSGGTDQNQLLEDSSSAMRLCNVNNDFPSEVNDERPPQRPSQ
GLSLSLSSSNPTSISLQSFELRPQQQQQQGYSGNKSTQHQNLQHTQMMMMMMNSHHQNNN
NNNHQHHNHHQFQIGSSKYLSPAQELLSEFCSLGVKESDEEVMMMKHKKKQKGKQQEEWD

TSHHSNNDQHDQSATTSSKKHVPPLHSLEFMELQKRKAKLLSMLEELKRRYGHYREQMRV
AAAAFEAAVGLGGAEIYTALASRAMSRHFRCLKDGLVGQIQATSQALGEREEDNRAVSIA
ARGETPRLRLLDQALRQQKSYRQMTLVDAHPWRPQRGLPERAVTTLRAWLFEHFLHPYPS
DVDKHILARQTGLSRSQVSNWFINARVRLWKPMIEEMYCEETRSEQMEITNPMMIDTKPD
PDQLIRVEPESLSSIVTNPTSKSGHNSTHGTMSLGSTFDFSLYGNQAVTYAGEGGPRGDV
SLTLGLQRNDGNGGVSLALSPVTAQGGQLFYGRDHIEEGPVQYSASMLDDDQVQNLPYRN
LMGAQLLHDIV

>Q9C9E3

MSTGQAPEKSNFSQRCSLLSRYLKEKGSFGNINMGLARKSDLELAGKFDLKGQQNVIKKV
ETSETRPFKLIQKFSIGEASTSTEDKAIYIDLSEPAKVAPESGNSQLTIFFGGKVMVFNE
FPEDKAKEIMEVAKEANHVAVDSKNSQSHMNLDKSNVVIPDLNEPTSSGNNEDQETGQQH
QVVERIARRASLHRFFAKRKDRAVARAPYQVNQHGSHLPPKPEMVAPSIKSGQSSQHIAT
PPKPKAHNHMPMEVDKKEGQSSKNLELKL

>Q9LV58

MPSRYPGAVTQDWEPVVLHKSKQKSQDLRDPKAVNAALRNGVAVQTVKKFDAGSNKKGKS
TAVPVINTKKLEEETEPAAMDRVKAEVRLMIQKARLEKKMSQADLAKQINERTQVVQEYE
NGKAVPNQAVLAKMEKVLGVKLRGKIGK

>Q9SKK0

MSQIFSFAGENDFYRRGAIYPNPKDASLLLSLGSFADVYFPPSKRSRVVAPTIFSAFEKK
PVSIDVLPDECLFEIFRRLSGPQERSACAFVSKQWLTLVSSIRQKEIDVPSKITEDGDDC
EGCLSRSLDGKKATDVRLAAIAVGTAGRGGLGKLSIRGSNSAKVSDLGLRSIGRSCPSLG
SLSLWNVSTITDNGLLEIAEGCAQLEKLELNRCSTITDKGLVAIAKSCPNLTELTLEACS
RIGDEGLLAIARSCSKLKSVSIKNCPLVRDQGIASLLSNTTCSLAKLKLQMLNVTDVSLA
VVGHYGLSITDLVLAGLSHVSEKGFWVMGNGVGLQKLNSLTITACQGVTDMGLESVGKGC
PNMKKAIISKSPLLSDNGLVSFAKASLSLESLQLEECHRVTQFGFFGSLLNCGEKLKAFS
LVNCLSIRDLTTGLPASSHCSALRSLSIRNCPGFGDANLAAIGKLCPQLEDIDLCGLKGI
TESGFLHLIQSSLVKINFSGCSNLTDRVISAITARNGWTLEVLNIDGCSNITDASLVSIA
ANCQILSDLDISKCAISDSGIQALASSDKLKLQILSVAGCSMVTDKSLPAIVGLGSTLLG
LNLQQCRSISNSTVDFLVERLYKCDILS

>Q38899

MEDIENIEEDEYGFSRNYFLAKELGGASKRSAHKLSDIHIVDEQELRETASTIEMKHSKE
ISELMSDYKTMYSKWVFELRCGFGLLMYGFGSKKALVEDFASASLTDYSVVVINGYLPSV
NLKQVLLALAELLSELLKCKRKSSGSLSKGQETFPSRSMDDILSFLHGPQSGDKDCFICV
VVHNIDGPALRDPESQQTLARLSSCSHIRLVASIDHVNAPLLWDKKMVHKQFNWLWHHVP
TFAPYNVEGVFFPLVLAQGSTAQTAKTAAIVLQSLTPNGQNVFKILAEYQLSHPDEDGMP
TDDLYSASRERFFVSSQVTLNSHLTEFKDHELVKTKRNSDGQECLNIPLTSDAIRQLLLD
LNQ

>Q8RY95

MDEVGAQVAAPMFIHQSLGRKRDLYYPMSNRLVQSQPQRRDEWNSKMWDWDSRRFEAKPV
DVEVQEFDLTLRNRSGEERGLDLNLGSGLTAVEETTTTTQNVRPNKKVRSGSPGGNYPMC
QVDNCTEDLSHAKDYHRRHKVCEVHSKATKALVGKQMQRFCQQCSRFHLLSEFDEGKRSC
RRRLAGHNRRRRKTTQPEEVASGVVVPGNHDTTNNTANANMDLMALLTALACAQGKNAVK
PPVGSPAVPDREQLLQILNKINALPLPMDLVSKLNNIGSLARKNMDHPTVNPQNDMNGAS
PSTMDLLAVLSTTLGSSSPDALAILSQGGFGNKDSEKTKLSSYENGVTTNLEKRTFGFSS

VGGERSSSSNQSPSQDSDSRGQDTRSSLSLQLFTSSPEDESRPTVASSRKYYSSASSNPV
EDRSPSSSPVMQELFPLQASPETMRSKNHKNSSPRTGCLPLELFGASNRGAADPNFKGFG
QQSGYASSGSDYSPPSLNSDAQDRTGKIVFKLLDKDPSQLPGTLRSEIYNWLSNIPSEME
SYIRPGCVVLSVYVAMSPAAWEQLEQKLLQRLGVLLQNSPSDFWRNARFIVNTGRQLASH
KNGKVRCSKSWRTWNSPELISVSPVAVVAGEETSLVVRGRSLTNDGISIRCTHMGSYMAM
EVTRAVCRQTIFDELNVNSFKVQNVHPGFLGRCFIEVENGFRGDSFPLIIANASICKELN
RLGEEFHPKSQDMTEEQAQSSNRGPTSREEVLCFLNELGWLFQKNQTSELREQSDFSLAR
FKFLLVCSVERDYCALIRTLLDMLVERNLVNDELNREALDMLAEIQLLNRAVKRKSTKMV
ELLIHYLVNPLTLSSSRKFVFLPNITGPGGITPLHLAACTSGSDDMIDLLTNDPQEIGLS
SWNTLRDATGQTPYSYAAIRNNHNYNSLVARKLADKRNKQVSLNIEHEVVDQTGLSKRLS
LEMNKSSSSCASCATVALKYQRRVSGSQRLFPTPIIHSMLAVATVCVCVCVFMHAFPIVR
QGSHFSWGGLDYGSI
>P25209
MAEAPASPGGGGGSHESGSPRGGGGGGGSVREQDRFLPIANISRIMKKAIPANGKIAKDAK
ETVQECVSEFISFITSEASDKCQREKRKTINGDDLLWAMATLGFEDYIEPLKVYLQKYRE
MEGDSKLTAKSSDGSIKKDALGHVGASSSAAEGMGQQGAYNQGMGYMQPQYHNGDISNV
>Q94AH6
MERKTIDLEQGWDYMQTGITKLKRILEGLNEPAFDSEQYMMLYTTIYNMCTQKPPHDYSQ
QLYDKYREAFEEYINSTVLPALREKHDEFMLRELFKRWSNHKVMVRWLSRFFYYLDRYFI
ARRSLPPLNEVGLTCFRDLVYNELHSKVKQAVIALVDKEREGEQIDRALLKNVLDIYVEI
GMGQMERYEEDFESFMLQDTSSYYSRKASSWIQEDSCPDYMLKSEECLKKERERVAHYLH
SSSEPKLVEKVQHELLVVFASQLLEKEHSGCRALLRDDKVDDLSRMYRLYHKILRGLEPV
ANIFKQHVTAEGNALVQQAEDTATNQVANTASVQEQVLIRKVIELHDKYMVYVTECFQNH
TLFHKALKEAFEIFCNKTVAGSSSAELLATFCDNILKKGGSEKLSDEAIEDTLEKVVKLL
AYISDKDLFAEFYRKKLARRLLFDRSANDDHERSILTKLKQQCGGQFTSKMEGMVTDLTL
ARENQNSFEDYLGSNPAANPGIDLTVTVLTTGFWPSYKSFDINLPSEMIKCVEVFKGFYE
TKTKHRKLTWIYSLGTCHINGKFDQKAIELIVSTYQAAVLLLFNTTDKLSYTEILAQLNL
SHEDLVRLLHSLSCAKYKILLKEPNTKTVSQNDAFEFNSKFTDRMRRIKIPLPPVDERKK
VVEDVDKDRRYAIDAAIVRIMKSRKVLGHQQLVSECVEQLSRMFKPDIKAIKKRMEDLIT
RDYLERDKENPNMFRYLA
>Q6EUP4
MSQPAELSREENVYMAKLAEQAERYEEMVEFMEKVAKTVDSEELTVEERNLLSVAYKNVI
GARRASWRIISSIEQKEESRGNEDRCTLIKEYRGKIETELSKICDGILKLLDSHLVPSST
APESKVFYLKMKGDYYRYLAEFKTGAERKDAAENTMVAYKAAQDIALAELPPTHPIRLGL
ALNFSVFYYEILNSPDRACNLAKQAFDEAISELDTLSEESYKDSTLIMQLLRDNLTLWTS
DISEDAAEEIKEAPKGESGDGQ
>P14713
MVSGVGGSGGGRGGGRGGEEEPSSSHTPNNRRGGEQAQSSGTKSLRPRSNTESMSKAIQQ
YTVDARLHAVFEQSGESGKSFDYSQSLKTTTYGSSVPEQQITAYLSRIQRGGYIQPFGCM
IAVDESSFRIIGYSENAREMLGIMPQSVPTLEKPEILAMGTDVRSLFTSSSSILLERAFV
AREITLLNPVWIHSKNTGKPFYAILHRIDVGVVIDLEPARTEDPALSIAGAVQSQKLAVR
AISQLQALPGGDIKLLCDTVVESVRDLTGYDRVMVYKFHEDEHGEVVAESKRDDLEPYIG
LHYPATDIPQASRFLFKQNRVRMIVDCNATPVLVVQDDRLTQSMCLVGSTLRAPHGCHSQ
YMANMGSIASLAMAVIINGNEDDGSNVASGRSSMRLWGLVVCHHTSSRCIPFPLRYACEF

LMQAFGLQLNMELQLALQMSEKRVLRTQTLLCDMLLRDSPAGIVTQSPSIMDLVKCDGAA
FLYHGKYYPLGVAPSEVQIKDVVEWLLANHADSTGLSTDSLGDAGYPGAAALGDAVCGMA
VAYITKRDFLFWFRSHTAKEIKWGGAKHHPEDKDDGQRMHPRSSFQAFLEVVKSRSQPWE
TAEMDAIHSLQLILRDSFKESEAAMNSKVVDGVVQPCRDMAGEQGIDELGAVAREMVRLI
ETATVPIFAVDAGGCINGWNAKIAELTGLSVEEAMGKSLVSDLIYKENEATVNKLLSRAL
RGDEEKNVEVKLKTFSPELQGKAVFVVVNACSSKDYLNNIVGVCFVGQDVTSQKIVMDKF
INIQGDYKAIVHSPNPLIPPIFAADENTCCLEWNMAMEKLTGWSRSEVIGKMIVGEVFGS
CCMLKGPDALTKFMIVLHNAIGGQDTDKFPFPFFDRNGKFVQALLTANKRVSLEGKVIGA
FCFLQIPSPELQQALAVQRRQDTECFTKAKELAYICQVIKNPLSGMRFANSLLEATDLNE
DQKQLLETSVSCEKQISRIVGDMDLESIEDGSFVLKREEFFLGSVINAIVSQAMFLLRDR
GLQLIRDIPEEIKSIEVFGDQIRIQQLLAEFLLSIIRYAPSQEWVEIHLSQLSKQMADGF
AAIRTEFRMACPGEGLPPELVRDMFHSSRWTSPEGLGLSVCRKILKLMNGEVQYIRESER
SYFLIILELPVPRKRPLSTASGSGDMMLMMPY
>Q9C895
MENQESDEPMQKKPHLLDSVSPNSMARNSSPSHPIAKSVSFFDCDFSLLCLRLVDYEIDV
DATVLQLQNQKLVQQLDLQKKQLYDVESKIQELQLNQTSYDDELISVNQLWNQLVDDLIL
LGVRAGANQEALNYLDIVDKKRVPPCAADETFLCRLLQVDSLDTSKSDEVVRKVEEALAL
RHSSTMELMGLFENTIDTQKTKAESISQSLHAVKSTEDATIQLSSINDLMKEESKNLREM
IDALHVRHKEHSEQIQAYISSHSTDQSELKHLKGQLEEIKAELEENRRKLITLKMQKDAA
CEGHVTSPAIANGSLSPEKPVDKTKLRELKDSIDEIKIMAEGRLSELQASQEYNLSLSRQ
CQDIENELKDDQYIYSSRLYSLINDRIHHWNAELDRYKILTEAIQAERSFVMRRDKELNL
RAESLEAANHKTTTVGSRIEVLEKKLQSCIIEKNGLELETEEAIQDSERQDIKSEFIAMA
STLSKEMEMMEAQLKRWKDTAQDALYLREQAQSLRVSLSNKADEQKGLEDKCAKQMAEIK
SLKALIEKLLKEKLQLQNLASICTRECNDDRGLAEIKDSQRKAQAQAEELKNVLDEHFLE
LRVKAAHETESACQERLATAKAEIAELRTQLDLSEREVLELKEGIKVKEQEAEASIAEME
TIGQAYEDMQTQNQHLLQQVAERDDYNIKLVSESVKTKHAYNTHLSEKQVMEKQLHQVNA
SVENFKARIAHNEEQMKGCFSEAYKLIQEDRHLVISLETTKWEVADADKEFRWLKSAVSS
SEKEYEQISRRTDDIKLELDDEREKKKLEEELMELNKELEELGSESVEAAIVRLQEEVKN
CKNILKCGVCFDRPKEVVIVKCYHLFCQQCIQRSLEIRHRKCPGCGTAFGQNDVRLVKM
>Q9SXZ2
MSINIRDPLIVSRVVGDVLDPFNRSITLKVTYGQREVTNGLDLRPSQVQNKPRVEIGGED
LRNFYTLVMVDPDVPSPSNPHLREYLHWLVTDIPATTGTTFGNEIVCYENPSPTAGIHRV
VFILFRQLGRQTVYAPGWRQNFNTREFAEIYNLGLPVAAVFYNCQRESGCGGRRL
>Q946J8
MKGASGAVKKKPQVLNEAGEAETAVETVGESRKISGDGGFGSDDGGGGGGGGSGESILRE
IGDDRPTEDGDEEEEEDEDEDDGGDEEDEEGEGEGGQEERPKLDEGFYEIEAIRRKRVRK
GKVQYLIKWRGWPETANTWEPLENLQSIADVIDAFEGSLKPGKPGRKRKRKYAGPHSQMK
KKQRLTSTSHDATEKSDSSTSLNNSSLPDIPDPLDLSGSSLLNRDVEAKNAYVSNQVEAN
SGSVGMARQVRLIDNEKEYDPTLNELRGPVNNSNGAGCSQGGGIGSEGDNVRPNGLLKVY
PKELDKNSRFIGAKRRKSGSVKRFKQDGSTSNNHTAPTDQNLTPDLTTLDSFGRIARMGN
EYPGVMENCNLSQKTKIEELDITKILKPMSFTASVSDNVQEVLVTFLALRSDGKEALVDN
RFLKAHNPHLLIEFYEQHLKYNRTP
>Q8LGE3
MSNIVVLDNGGGLIKAGQGGERDPTTVIPNCLYKPLSSKKFIHPSPLTTLSDEIDLTSAA

VRRPIDRGYLINSDLQREIWSHLFTSLLHIAPSSSSLLLTEAPLSIPSVQRTTDELVFED
FGFSSLYIAHPQSLVHLYEASRQPDSILSKTQCSLVVDCGFSFTHAVPVLHNFTLNHAIK
RIDLGGKAFTNYLKELVSYRSINVMDETFLVDDAKEKLCFVSLDLLRDLRLARNGNTLIK
STYVLPDGVTHTKGYVKDPQAAKRFLSLSEKESVVVMDKVGERKKADMNKNEIDLTNERF
LVPETLFQPADLGMNQAGLAECIVRAINSCHSYLQPVLYQSIILTGGSTLFPQLKERLEG
ELRPLVPDHFDVKITTQEDPILGVWRGGSLLASSPDFESMCVTKAEYEELGSARCRRRFF
H
>Q9FQ20
MFYSHCLVSRKGPLGAIWVAAYFFKKLKKSQVKATHIPSSVDQILQKELDALTYRVLAYL
LLGVVRIYSKKVDFLFDDCNKALIGVKEFVAKERNREKTGVSLPASIECFSIALPERFEL
DAFDLGVLEDFHGGNVKPHEDITLKDGSQETERMDMYSMERFDMEEDLLFTFHETFSTNH
NENKHESFAHDMELDAENVRDTTEEASVRVVEAEPLDSNEPSRDHQNASRHREDPESDDI
LLEPQMSEDIRIAQEEDTVRETICTIVQRLVDSHESSGDNLHRDGHTENLESEKTSKKTS
CEEMQHDRSLPSECGIPEAIHGIEDQPSGATRINGEKEIPEMSTLEKPEPVSVTGSRDLQ
EGVEKCRDHNEAEMADFELFHGSHKEQSETSEVNLHGSEKGFLSDMTVSKDPSSEFNATD
TPVTVTPKTPSRLKISEGGTSPQFSIIPTPAAKESSRVSRKRKCLIDDEVIIPNKVMKEM
IEDSSKLLAKRRNVPHTDCPERRTKRFANPFRSFLEPLIQYGSSDLQSLFCQPIKLKNWA
TTGTPKDTKIARHKEKSSLDTVRSPGVILSSDQTENTQEIMETPQAAALAGLKVTAGNSN
VVSVEMGASSTTSGTAHQTENAAETPVKPSVIAPETPVRTSEQTVIAPETPVVSEQVEIA
PETPVRESMSKRFFKDPGTCYKKSRPASPFTSFEEHPSVYYVENRDLDTILMNDEQVNAD
ERQDLQQETWSSRTRNVAKFLEKTFLEQREREEEEKVSLLQLCRGRTQKESARLFYETLV
LKTKGYVEVKQNHPYSDVFLMRVSRPQKAC
>Q8GZA8
MANNEGEMQCGSMLFKQEELQEMSGVNVGGDYVEVMCGCTSHRYGDAVARLRVFPTGDLE
ITCECTPGCDEDKLTPAAFEKHSGRETARKWKNNVWVIIGGEKVPLSKTVLLKYYNESSK
KCSRSNRSQGAKVCHRDEFVGCNDCGKERRFRLRSRDECRLHHNAMGDPNWKCSDFPYDK
ITCEEEEERGSRKVYRGCTRSPSCKGCTSCVCFGCELCRFSECTCQTCVDFTSNVKA
>Q8RVQ9
MARTKHRVTRSQPRNQTDAAGASSSQAAGPTTTPTRRGGEGGDNTQQTNPTTSPATGTRR
GAKRSRQAMPRGSQKKSYRYRPGTVALKEIRHFQKQTNLLIPAASFIREVRSITHMLAPP
QINRWTAEALVALQEAAEDYLVGLFSDSMLCAIHARRVTLMRKDFELARRLGGKGRPW
>Q9SQI2
MASSSSSERWIDGLQFSSLLWPPPRDPQQHKDQVVAYVEYFGQFTSEQFPDDIAELVRHQ
YPSTEKRLLDDVLAMFVLHHPEHGHAVILPIISCLIDGSLVYSKEAHPFASFISLVCPSS
ENDYSEQWALACGEILRILTHYNRPIYKTEQQNGDTERNCLSKATTSGSPTSEPKAGSPT
QHERKPLRPLSPWISDILLAAPLGIRSDYFRWCSGVMGKYAAGELKPPTIASRGSGKHPQ
LMPSTPRWAVANGAGVILSVCDDEVARYETATLTAVAVPALLLPPPTTSLDEHLVAGLPA
LEPYARLFHRYYAIATPSATQRLLLGLLEAPPSWAPDALDAAVQLVELLRAAEDYASGVR
LPRNWMHLHFLRAIGIAMSMRAGVAADAAAALLFRILSQPALLFPPLSQVEGVEIQHAPI
GGYSSNYRKQIEVPAAEATIEATAQGIASMLCAHGPEVEWRICTIWEAAYGLIPLNSSAV
DLPEIIVATPLQPPILSWNLYIPLLKVLEYLPRGSPSEACLMKIFVATVETILSRTFPPE
SSRELTRKARSSFTTRSATKNLAMSELRAMVHALFLESCAGVELASRLLFVVLTVCVSHE
AQSSGSKRPRSEYASTTENIEANQPVSNNQTANRKSRNVKGQGPVAAFDSYVLAAVCALA
CEVQLYPMISGGGNFSNSAVAGTITKPVKINGSSKEYGAGIDSAISHTRRILAILEALFS

LKPSSVGTPWSYSSSEIVAAAMVAAHISELFRRSKALTHALSGLMRCKWDKEIHKRASSL
YNLIDVHSKVVASIVDKAEPLEAYLKNTPVQKDSVTCLNWKQENTCASTTCFDTAVTSAS
RTEMNPRGNHKYARHSDEGSGRPSEKGIKDFLLDASDLANFLTADRLAGFYCGTQKLLRS
VLAEKPELSFSVVSLLWHKLIAAPEIQPTAESTSAQQGWRQVVDALCNVVSATPAKAAAA
VVLQAERELQPWIAKDDEEGQKMWKINQRIVKVLVELMRNHDRPESLVILASASDLLLRA
TDGMLVDGEACTLPQLELLEATARAIQPVLAWGPSGLAVVDGLSNLLKCRLPATIRCLSH
PSAHVRALSTSVLRDIMNQSSIPIKVTPKLPTTEKNGMNSPSYRFFNAASIDWKADIQNC
LNWEAHSLLSTTMPTQFLDTAARELGCTISLSQ

>Q75RY2

MKLKMNKACDIASISVLPPRRTGGSSGASASGSVAVAVASQPRSQPLSQSQQSFSQGASA
SLLHSQSQFSQVSLDDNLLTLLPSPTRDQRFGLHDDSSKRMSSLPASSASCAREESQLQL
AKLPSNPVHRWNPSIADTRSGQVTNEDVERKFQHLASSVHKMGMVVDSVQSDVMQLNRAM
KEASLDSGSIRQKIAVLESSLQQILKGQDDLKALFGSSTKHNPDQTSVLNSLGSKLNEIS
STLATLQTQMQARQLQGDQTTVLNSNASKSNEISSTLATLQTQMQADIRQLRCDVFRVFT
KEMEGVVRAIRSVNSRPAAMQMMADQSYQVPVSNGWTQINQTPVAAGRSPMNRAPVAAGR
SRMNQLPETKVLSAHLVYPAKVTDLKPKVEQGKVKAAPQKPFASSYYRVAPKQEEVAIRK
VNIQVPAKKAPVSIIIESDDDSEGRASCVILKTETGSKEWKVTKQGTEEGLEILRRARKR
RRREMQSIVLAS

>Q8L4H0

MFEKNGRTLLAKRKTQGTIKTRASKKIRKMEGTLERHSLLQFGQLSKISFENRPSSNVAS
SAFQGLLDSDSSELRNQLGSADSDANCGEKDFILSQDFFCTPDYITPDNQNLMSGLDISK
DHSPCPRSPVKLNTVKSKRCRQESFTGNHSNSTWSSKHRVDEQENDDIDTDEVMGDKLQA
NQTERTGYVSQAAVALRCRAMPPPCLKNPYVLNQSETATDPFGHQRSKCASFLPVSTSGD
GLSRYLTDFHEIRQIGAGHFSRVFKVLKRMDGCLYAVKHSTRKLYLDSERRKAMMEVQAL
AALGFHENIVGYYSSWFENEQLYIQLELCDHSLSALPKKSSLKVSEREILVIMHQIAKAL
HFVHEKGIAHLDVKPDNIYIKNGVCKLGDFGCATRLDKSLPVEEGDARYMPQEILNEDYE
HLDKVDIFSLGVTVYELIKGSPLTESRNQSLNIKEGKLPLLPGHSLQLQQLLKTMMDRDP
KRRPSARELLDHPMFDRIRG

>Q9S7B2

MKERQRWRPEEDAVLRAYVRQYGPREWHLVSQRMNVALDRDAKSCLERWKNYLRPGIKKG
SLTEEEQRLVIRLQAKHGNKWKKIAAEVPGRTAKRLGKWWEVFKEKQQRELRDSRRPPPE
PSPDERGRYEWLLENFAEKLVGERPQQAAAAPSPLLMAAPVLPPWLSSNAGPAAAAAAAV
AHPPPRPPSPSVTLSLASAAVAPGPPAPAPWMPDRAAADAAPYGFPSPSQHGGAAPPGMA
VVDGQALAELAECCRELEEGRRAWAAHRREAAWRLKRVEQQLEMEREMRRREVWEEFEAK
MRTMRLEQAAAAERVERDHREKVAELRRDAQVKEEKMAEQWAAKHARVAKFVEQMGGCSR
SWSSATDMNC

>Q9FEF8

MRPPVTGGRGGGGFRGGRDGGGRGFGGGRSFGGGRSGDRGRSGPRGRGRGAPRGRGGPPR
GGMKGGSKVIVEPHRHAGVFIAKGKEDALVTKNLVPGEAVYNEKRISVQNEDGTKVEYRV
WNPFRSKLAAAILGGVDNIWIKPGAKVLYLGAASGTTVSHVSDLVGPEGCVYAVEFSHRS
GRDLVNMAKKRTNVIPIIEDARHPAKYRMLVGMVDVIFSDVAQPDQARILALNASFFLKT
GGHFVISIKANCIDSTVAAEAVFQSEVKKLQQEQFKPAEQVTLEPFERDHACVVGGYRMP
KKQKTPAS

>P42774

MGTSEDKMPFKTTKPTSSAQEVPPTPYPDWQNSMQAYYGGGGTPNPFFPSPVGSPSPHPY
MWGAQHHMMPPYGTPVPYPAMYPPGAVYAHPSMPMPPNSGPTNKEPAKDQASGKKSKGNS
KKKAEGGDKALSGSGNDGASHSDESVTAGSSDENDENANQQEQGSIRKPSFGQMLADASS
QSTTGEIQGSVPMKPVAPGTNLNIGMDLWSSQAGVPVKDERELKRQKRKQSNRESARRSR
LRKQAECEQLQQRVESLSNENQSLRDELQRLSSECDKLKSENNSIQDELQRVLGAEAVAN
LEQNAAGSKDGEGTN
>P49311
MASPDVEYRCFVGGLAWATDERSLETAFSQFGELVDSKIINDRETGRSRGFGFVTFKDEK
SMKDAIEGMNGQDLDGRSITVNEAQSRGSGAGGGGRGGGGGYRGGGGYGGGGGGYGGGRR
EGGGYSGGGGGYSSRGGGGGGYGGGGRRDGGGYGGGEGGGYGGGGGGGW
>Q9XF62
MSAQGQALQNHNNELVKCIEDLREKREEIIKQLREDDAEKAKITQELQILTKRLAQVNES
IARKTETKNEYDKVISETEAAYLKILESSQTLLTVLKREAVNIAKKKQASS

(10) $\mathbb{S}_{10}$: 21 peroxisome proteins

>Q43743
MEFRGDAYKRIAMISAHLQPSFTPQMEAKNSVMGLESCRAKGGNPGFKVAILGAAGGIGQ
SLSLLMKMNPLVSLLHLYDVVNAPGVTADVSHMDTGAVVRGFLGAKQLEDALTGMDLVII
PAGVPRKPGMTRDDLFKINAGIVRTLCEGVAKCCPNAIVNLISNPVNSTVAIAAEVFKKA
GTYDPKKLLGVTTLDVARANTFVAEVLGLDPREVDVPVVGGHAGVTILPLLSQVKPPSSF
TPSEIEYLTNRIQNGGTEVVEAKAGAGSATLSMAYAAAKFADACLRGLRGDANVIECSFV
ASQVTELAFFATKVRLGRTGAEEVFQLGPLNEYERVGLEKAKEELAGSIQKGVDFIRK
>P05414
MEITNVNEYEAIAKQKLPKMVYDYYASGAEDQWTLAENRNAFSRILFRPRILIDVTNIDM
TTTILGFKISMPIMIAPTAMQKMAHPEGEYATARAASAAGTIMTLSSWATSSVEEVASTG
PGIRFFQLYVYKDRNVVAQLVRRAERAGFKAIALTVDTPRLGRREADIKNRFVLPPFLTL
KNFEGIDLGKMDKANDSGLSSYVAGQIDRSLSWKDVAWLQTITSLPILVKGVITAEDARL
AVQHGAAGIIVSNHGARQLDYVPATIMALEEVVKAAQGRIPVFLDGGVRRGTDVFKALAL
GAAGVFIGRPVVFSLAAEGEAGVKKVLQMMRDEFELTMALSGCRSLKEISRSHIAADWDG
PSSRAVARL
>Q84P23
MLTKTNDSRLIDRSSGFDQRTGIYHSLRPSLSLPPIDQPLSAAEFALSLLLKSSPPATAG
KNIEALTYLVNSSSGDNLTYGELLRRVRSLAVSLRERFPSLASRNVAFILSPSSLDIPVL
YLALMSIGVVVSPANPIGSESEVSHQVEVSEPVIAFATSQTVKKLQSSSLPLGTVLMDST
EFLSWLNRSDSSSVNPFQVQVNQSDPAAILFSSGTTGRVKGVLLTHRNLIASTAVSHQRT
LQDPVNYDRVGLFSLPLFHVFGFMMMIRAISLGETLVLLGRFELEAMFKAVEKYKVTGMP
VSPPLIVALVKSELTKKYDLRSLRSLGCGGAPLGKDIAERFKQKFPDVDIVQGYGLTESS
GPAASTFGPEEMVKYGSVGRISENMEAKIVDPSTGESLPPGKTGELWLRGPVIMKGYVGN
EKASAETVDKEGWLKTGDLCYFDSEDFLYIVDRLKELIKYKAYQVPPVELEQILHSNPDV
IDAAVVPFPDEDAGEIPMAFIVRKPGSNLNEAQIIDFVAKQVTPYKKVRRVAFINAIPKN
PAGKILRRELTKIAVDGNASKL
>O80845
MGTTLDVSRAELALVVMYLNKAEARDKLCRAIQYGSKFLSGGQPGTAQNVDKSTSLARKV
FRLFKFVNDLHGLISPVPKGTPLPLVLLGKSKNALLSTFLFLDQIVWLGRSGIYKNKERA

ELLGRISLFCWMGSSVCTTLVEVGEMGRLSSSMKKIEKGLKNGNKYQDEDYRAKLKKSNE
RSLALIKSAMDIVVAAGLLQLAPTKITPRVTGAFGFITSIISCYQLLPTRPKIKTP
>P13443
MAKPVQIEVWNPNGKYRVVSTKPMPGTRWINLLIEQDCRVEICTEKKTILSVEDILALIG
DKCDGVIGQLTEDWGEVLFSALSRAGGKAFSNMAVGYNNVDVNAANKYGVAVGNTPGVLT
ETTAELAASLSLAAARRIVEADEFMRAGRYDGWLPNLFVGNLLKGQTVGVIGAGRIGSAY
ARMMVEGFKMNLIYFDLYQSTRLEKFVTAYGEFLKANGEAPVTWRRASSMDEVLREADVI
SLHPVLDKTTFHLVNKESLKAMKKDAILINCSRGPVIDEAALVDHLRDNPMFRVGLDVFE
DEPYMKPGLADMKNAIIVPHIASASKWTREGMATLAALNVLGKIKGYPVWSDPNRVEPFL
DENVSPPAASPSIVNAKALGNA
>P25890
MDPYKHRPSSAFNSPFWTTNSGAPVWNNNSSLTVGSRGPILLEDYHLVEKLAQFDRERIP
ERVVHARGASAKGFFEVTHDISHLTCADFLRAPGVQTPVIVRFSTVIHERGSPETLRDPR
GFAVKFYTREGNYDLVGNNFPVFFVHDGMNFPDMVHALKPNPQTHIQENWRILDFFYNFP
ESLHMFSFLFDDVGVPQDYRHMDGFGVNTYTLINKAGKSVYVKFHWKPTCGVKCLLEEEA
IQVGGSNHSHATKDLYDSIAAGNYPEWKLYIQTIDPAHEDRFEFDPLDVTKTWPEDIIPL
QPVGRMVLNKNIDNFFAENEQLAFCPAIMLPGIYYSDDKMLQTRVFSYADSQRHRLGPNY
LQLPVNAPKWSHHNNHHEGFMNAIHRDEEVNYFPSRHDTVRHAERVPIPTTHLSARREKC
NIPKQNHFKQAGERYRTWAPDRQERFLRRWVEALSDTDPRITHEIRSIWVSYWSQADRSL
GQKLASHLNMRPSI
>O24174
MAAPSAIPRRGLFIGGGWREPSLGRRLPVVNPATEATIGDIPAATAEDVELAVSAARDAF
GRDGGRHWSRAPGAVRAKYLKAIAAKIKDKKSYLALLETLDSGKPLDEAAGDMEDVAACF
EYYADLAEALDGKQRAPISLPMENFESYVLKEPIGVVGLITPWNYPLLMATWKVAPALAA
GCTAVLKPSELASLTCLELGGICAEIGLPPGVLNIITGLGTEAGAPLASHPHVDKIAFTG
STETGKRIMITASQMVKPVSLELGGKSPLIVFDDVDIDKAVEWAMFGCFANAGQVCSATS
RLLLHEKIAKRFLDRLVAWAKSIKISDPLEEGCRLGSVVSEGQYQKIMKFISTARCEGAT
ILYGGARPQHLKRGFFIEPTIITNVSTSMQIWREEVFGPVICVKEFRTEREAVELANDTH
YGLAGAVISNDLERCERISKAIQSGIVWINCSQPCFVQAPWGGNKRSGFGRELGQWGLDN
YLSVKQVTKYCSDEPYGWYRPPSKL
>P08216
MGSLGMYSESGLTKKGSSRGYDVPEGVDIRGRYDEEFAKILNKEALLFIADLQRTFRNHI
KYSMECRREAKRRYNEGGLPGFDPATKYIRDSEWTCAPVPPAVADRRVEITGPVERKMII
NALNSGAKVFMADFEDALSPNWENLMRGQINLKDAVDGTISFHDRVRNRVYKLNDRTAKL
FVRPRGWHLPEAHIFIDGEPATGCLVDFGLYFFHNHANFRRSQGQGYGPFFYLPKMEHSR
EAKIWNSVFERAEKMAGIERGSIRATVLIETLPAVFQMNEILYELRDHSVGLNCGRWDYI
FSYVKTFQAHPDRLLPDRVLVGMTQHFMRSYSDLLIRTCHRRGVHAMGGMAAQIPIRDDP
KANEVALELVRKDKLREVKAGHDGTWAAHPGLIPACMEVFTNNMGNAPNQIRSMRRDDAA
NLTEEDLLQQPRGVRTMEGLRLNTRVGIQYLAAWLTGAGSVPLYNLAEDAATAEISRVQN
WQWLKYGVELDGDGLGVRVNKELFGRVVEEEMERIEREVGKERFKKGMYKEACKMFTRQC
TAPNLDDFLTLDAYNYIVIHHPRELSKL
>Q9ZS51
MGSSPPKKTTLQRYLSQLQQHPLRTKAITAGVLSGVSDVVSQKLSGIQKIQLRRVLLKVI
FAGGFLGPAGHFFHTYLDKFFKGKKDTQTVAKKVILEQLTLSPLNHLLFMIYYGVVIERT

PWTLVRERIKKTYPTVQLTAWTFFPVVGWINYKYVPLHFRVILHSLVAFFWGIFLTLRAR
SMTLALAKAK

>O04104

MAQQEVVEGFKFEQRHGKERVRVARVWKTRQGQHFVVEWRVGITLFSDCVNSYLRDDNSD
IVATDTMKNTVYAKAKECSDILSAEDFAILLAKHFVSFYKKVTGAIVNIVEKPWERVIVD
GQPHEHGFKLGSEKHTTEAIVQKSGSLQLTSGIEGLSVLKTTQSGFVNFIRDKYTALPDT
RERILATEVTALWRYSYESQYSLPQKPLYFTEKYQEVKKVLADTFFGPPNGGVYSPSVQN
TLYLMAKATLNRFPDIAYVSLKMPNLHFLPVNISNKDGPIVKFEDDVYLPTDEPHGSIQA
SLSRLWSKL

>O64894

MASPGEPNRTAEDESQAAARRIERLSLHLTPIPLDDSQGVEMETCAAGKAKAKIEVDMGS
LSLYMRGKHREIQERVFEYFNSRPELQTPVGISMADHRELCMKQLVGLVREAGIRPFRFV
NEDPAKYFAIMEAVGSVDVSLAIKMGVQFSLWGGSVINLGTKKHRDRFFDGIDNVDYPGC
FAMTELHHGSNVQGLQTTATFDPITDEFIINTPNDGAIKWWIGNAAVHGKFATVFAKLVL
PTHDSRKTADMGVHAFIVPIRDLKSHKTLPGIEIHDCGHKVGLNGVDNGALRFRSVRIPR
DNLLNRFGEVSRDGKYKSSLPSINKRFAATLGELVGGRVGLAYSSASVLKIASTIAIRYS
LLRQQFGPPKQPEVSILDYQSQQHKLMPMLASTYAFHFSTMQLVEKYAQMKKTHDEELVG
DVHALSAGLKAYVTSYTAKSLSTCREACGGHGYAVVNRFGTLRNDHDIFQTFEGDNTVLL
QQVAAYLLKQYQEKFQGGTLAVTWNYLRESMNTYLSQPNPVTARWESADHLRDPKFQLDA
FQYRTSRLLQSVAVRLRKHTKNLGSFGAWNRCLNHLLTLAESHIESVILAQFIESVQRCP
NANTQATLKLVCDLYALDRIWNDIGTYRNVDYVAPNKAKAIHKLTEYLCFQVRNIAQELV
DAFDLPDHVTRAPIAMKSNAYSQYTQYIGF

>Q9FZF1

MATKAPEKITKPKDRDFLNHLETYLSKRDGVDKLLKISRYATKIILASSLIPETRSIIPR
LKSFESSVGVSRKAFRLGKFVQDINALRSSRWDSNHELVLLIIAYGGEGLYYFVEQFIWL
TKSGLIDAKHSKWLQKISAWAELVGYVGSVSIKIRDLRKLNDEESCVASTIEISVSRGLA
CDGEDEKMKMIKEKKTLKVLSILQDLADGLMTIADIRDGKGVLSAPNVISSAGLFSAIVS
THKNWISC

>P49299

MPTDMELSPSNVARHRLAVLAAHLSAASLEPPVMASSLEAHCVSAQTMVAPPELVKGTLT
IVDERTGKRYQVQVSEEGTIKATDLKKITTGPNDKGLKLYDPGYLNTAPVRSSISYIDGD
LGILRYRGYPIEELAESSTYVEVAYLLMYGNLPSQSQLADWEFAISQHSAVPQGLVDIIQ
AMPHDAHPMGVLVSAMSALSVFHPDANPALRGQDLYKSKQVRDKQIARIIGKAPTIAAAA
YLRLAGRPPVLPSSNLSYSENFLYMLDSLGNRSYKPNPRLARVLDILFILHAEHEMNCST
SAARHLASSGVDVFTALSGAVGALYGPLHGGANEAVLKMLSEIGTVNNIPEFIEGVKNRK
RKMSGFGHRVYKNYDPRAKVIRKLAEEVFSIVGRDPLIEVAVALEKAALSDEYFVKRKLY
PNVDFYSGLIYRAMGFPPEFFTVLFAIPRMAGYLAHWRESLDDPDTKIIRPQQVYTGEWL
RHYIPPNERLVPAKADRLGQVSVSNASKRRLSGSGI

>Q56YA5

MDYMYGPGRHHLFVPGPVNIPEPVIRAMNRNNEDYRSPAIPALTKTLLEDVKKIFKTTSG
TPFLFPTTGTGAWESALTNTLSPGDRIVSFLIGQFSLLWIDQQKRLNFNVDVVESDWGQG
ANLQVLASKLSQDENHTIKAICIVHNETATGVTNDISAVRTLLDHYKHPALLLVDGVSSI
CALDFRMDEWGVDVALTGSQKALSLPTGLGIVCASPKALEATKTSKSLKVFFDWNDYLKF
YKLGTYWPYTPSIQLLYGLRAALDLIFEEGLENIIARHARLGKATRLAVEAWGLKNCTQK

EEWISNTVTAVMVPPHIDGSEIVRRAWQRYNLSLGLGLNKVAGKVFRIGHLGNVNELQLL
GCLAGVEMILKDVGYPVVMGSGVAAASTYLQHHIPLIPSRI
>Q43097
MAIYSAQAPNSILEEEARFEAEVSETQAWWNSTDLFRLTRRPYTARDVVRLRGSMRQSYA
SNEMAKKLWRTLKTHQANKTASRTFGALDPVQVSMMAKYLDSIYVSGWQCSSTHTTTNEP
GPDLADYPYDTVPNKVEHLFFAQQFHDRKQKEARMSMTREERSKTPYIDYLKPIIADGDT
GFGGATATVKLCKLFVERGAAGVHIEDQASVTKKCGHMAGKVLVSVGEHVNRMVAARLQF
DIMGVETLLVARTDAVAATLIQTNVDARDHQFILGATNPNLKGKPLADVLARAMASGKSG
ADLQAVEDEWMAMADLKLFSDCVVDGIKALNVSEQEKGRRLGEWMQQTGGNTGNVLSYYQ
AKELAEKLGISNLFWDWDLPRTREGFYRFQGSVKAAIVRGWAFGPHADIIWMETSSPDMV
ECRDFALGVKSKHPEIMLAYNLSPSFNWDASRMTDEQMKNFIPEIARLGYCWQFITLAGF
HADALVIDTFAKDFAQRGMLAYVEKIQRQEMMNGVDTLAHQKWSGANYYDQLLKTVQGGI
SATAAMAKGVTEDQFEETQSSTLALESNIGAGTVLAKSRM
>Q39659
MGSNAKGRTVMEVGTDGVAIITIINPPVNSLSFDVLFSLRDSYEQALRRDDVKAIVVTGA
KGKFSGGFDITAFGVLQGGKGEQPNVRNISIEMITDIFEAARKPAVAAIDGLALGGGLEV
AMACHARISTPTAQLGLPELQLGIIPGFGGTQRLPRLVGLSKALEMMLTSKPIKGQEAHS
LGLVDAIVPPEELINTARRWALEILERRRPWVHSLHRTDKLESLAEARKIFNLARAQAKK
QYPNLKHTIACIDAVETGVVSGPRAGLWKEAEEFQGLLHSDTCKSLIHIFFAQRSTTKVP
GVTDLGLVPRQIKKVAIVGGGLMGSGIATALILSNYHVVLKEVNDKFLQAGIDRVRANLQ
SRVKKGNMTNEKFEKSISLLKGVLNYESFKDVDMVIEAVIENVSLKQQIFSDLEKYCPPH
CMLATNTSTIDLELIGERIKSRDRIIGAHFFSPAHIMPLLEIVRTKHTAAQVIVDLLDVG
KNIKKTPVVVGNCTGFAVNRMFFPYSQAAILLAEHGVDPYQIDRAISKFGMPMGPFRLCD
LVGFGVAAATASQFVQAFPERTYKSMLIPLMQEDKNAGESTRKGFYVYDKNRKAGPNPEL
KKYIEKARNSSGVSVDPKLTKLPEKDIVEMIFFPVVNEACRVLAEGIAVKAADLDIAGVM
GMGFPSYRGGLMFWADSLGSNYIYSRLEEWSKQYGGFFKPCGYLAERAVQGATLSAPGGH
AKPRM
>Q56WD9
MEKAIERQRVLLEHLRPSSSSSHNYEASLSASACLAGDSAAYQRTSLYGDDVVIVAAHRT
PLCKSKRGNFKDTYPDDLLAPVLRALIEKTNLNPSEVGDIVVGTVLAPGSQRASECRMAA
FYAGFPETVAVRTVNRQCSSGLQAVADVAAAIKAGFYDIGIGAGLESMTTNPMAWEGSVN
PAVKKFAQAQNCLLPMGVTSENVAQRFGVSRQEQDQAAVDSHRKAAAATAAGKFKDEIIP
VKTKLVDPKTGDEKPITVSVDDGIRPTTTLASLGKLKPVFKKDGTTTAGNSSQVSDGAGA
VLLMKRSVAMQKGLPVLGVFRTFAAVGVDPAIMGIGPAVAIPAAVKAAGLELDDIDLFEI
NEAFASQFVYCRNKLGLDPEKINVNGGAMAIGHPLGATGARCVATLLHEMKRRGKDCRFG
VVSMCIGTGMGAAAVFERGDGVDELRNARKVEAQGLLSKDAR
>Q96329
MAVLSSADRASNEKKVKSSYFDLPPMEMSVAFPQATPASTFPPCTSDYYHFNDLLTPEEQ
AIRKKVRECMEKEVAPIMTEYWEKAEFPFHITPKLGAMGVAGGSIKGYGCPGLSITANAI
ATAEIARVDASCSTFILVHSSLGMLTIALCGSEAQKEKYLPSLAQLNTVACWALTEPDNG
SDASGLGTTATKVEGGWKINGQKRWIGNSTFADLLIIFARNTTTNQINGFIVKKDAPGLK
ATKIPNKIGLRMVQNGDILLQNVFVPDEDRLPGVNSFQDTSKVLAVSRVMVAWQPIGISM
GIYDMCHRYLKERKQFGAPLAAFQLNQQKLVQMLGNVQAMFLMGWRLCKLYETGQMTPGQ
ASLGKAWISSKARETASLGRELLGGNGILADFLVAKAFCDLEPIYTYEGTYDINTLVTGR

EVTGIASFKPATRSRL
>Q42564
MAAPIVDAEYLKEITKARRELRSLIANKNCAPIMLRLAWHDAGTYDAQSKTGGPNGSIRN
EEEHTHGANSGLKIALDLCEGVKAKHPKITYADLYQLAGVVAVEVTGGPDIVFVPGRKDS
NVCPKEGRLPDAKQGFQHLRDVFYRMGLSDKDIVALSGGHTLGRAHPERSGFDGPWTQEP
LKFDNSYFVELLKGESEGLLKLPTDKTLLEDPEFRRLVELYAKDEDAFFRDYAESHKKLS
ELGFNPNSSAGKAVADSTILAQSAFGVAVAAAVVAFGYFYEIRKRMK
>Q9S850
MPGIRGPSEYSQEPPRHPSLKVNAKEPFNAEPPRSALVSSYVTPVDLFYKRNHGPIPIVD
HLQSYSVTLTGLIQNPRKLFIKDIRSLPKYNVTATLQCAGNRRTAMSKVRNVRGVGWDVS
AIGNAVWGGAKLADVLELVGIPKLTASTNLGARHVEFVSVDRCKEENGGPYKASITLSQA
TNPEADVLLAYEMNGETLNRDHGFPLRVVVPGVIGARSVKWLDSINVIAEESQGFFMQKD
YKMFPPSVNWDNINWSSRRPQMDFPVQSAICSVEDVQMVKPGKVSIKGYAVSGGGRGIER
VDISLDGGKNWVEASRTQEPGKQYISEHSSSDKWAWVLFEATIDVSQTTEVIAKAVDSAA
NVQPENVESVWNLRGVLNTSWHRVLLRLGHSNL
>Q9FEW9
MASSAQDGNNPLFSPYKMGKFNLSHRVVLAPMTRCRALNNIPQAALGEYYEQRATAGGFL
ITEGTMISPTSAGFPHVPGIFTKEQVREWKKIVDVVHAKGAVIFCQLWHVGRASHEVYQP
AGAAPISSTEKPISNRWRILMPDGTHGIYPKPRAIGTYEISQVVEDYRRSALNAIEAGFD
GIEIHGAHGYLIDQFLKDGINDRTDEYGGSLANRCKFITQVVQAVVSAIGADRVGVRVSP
AIDHLDAMDSNPLSLGLAVVERLNKIQLHSGSKLAYLHVTQPRYVAYGQTEAGRLGSEEE
EARLMRTLRNAYQGTFICSGGYTRELGIEAVAQGDADLVSYGRLFISNPDLVMRIKLNAP
LNKYNRKTFYTQDPVVGYTDYPFLQGNGSNGPLSRL

(11) $\mathbb{S}_{11}$: 39 plastid proteins

>P28643
MATATAAGCSGAVALKSLGGRRLCIPQQLSPVLAGFGSHAAKSFPILSTRSIATSGIRAQ
VATAEKVSAGAGQSVESPVVIVTGASRGIGKAIALSLGKAGCKVLVNYARSSKEAEEVSK
EIEAFGGQALTFGGDVSKEEDVEAMIKTAVDAWGTVDILVNNAGITRDGLLMRMKKSQWQ
EVIDLNLTGVFLCTQAAAKIMMKKKKGRIINIASVVGLVGNAGQANYSAAKAGVIGFTKT
VAREYASRNINVNAVAPGFISSDMTSKLGDDINKKILETIPLGRYGQPEEVAGLVEFLAI
NPASSYVTGQVFTIDGGMTM
>P28645
MALNLNPVSTPFQCRRLPSFSPRQTPSRRSPKFFMASTLSSSSPKEAESLKKPFSPPREV
HVQVTHSMPQEKIEIFKSLEGWAEENLLVHLKPVEKCWQPQDYLPDPASEDFRDQVKEIQ
ERAKEIPDDLYVVLVGDMITEEALPTYQTMLNTLDGAKDETGASPTSWAVWTRAWTAEEN
RHGDLLNKYLYLSGRVDMRSIEKTIQYLIGSGMDPRTENNPYLGFVYTSFQERATFVSHG
NSARLAKEHGDLKMAQICGIIASDEKRHETAYTKIVEKLFEIDPDATVLAFADMMKKKIS
MPAHLMYDGRDDNLFDHFSAVAQRLGVYTAKDYADILEFLVGRWEVEKLTGLSSEGQKAQ
DYVCSLPPRIRRLEERARERAKQAPSMPFSWIFDRQVKL
>Q85UK6
MLKNKTKKTEVYALCRHISLSADKARRVIDQIRGRSYEETLMILELMPYRACYPILKLVY
SAASNAAYSMDSAEVNLVISKAEVNEGTITKKFKPRARGRSYVIKRTTCHITIVVKDISL
DKYEEIYSFKNPIWKNTIDVYSNGVVWHKK

>P31562
MERREGGRDNSSCSNVNQLFGVKDSESLLYDDVFIVRDRNGDSYFAYWDIEKNTFLSEPF
YSYRSRNSSYLPKIKAFMSEDRSQIHEVKNGFRSEDHSKINKINGVENLFHNYNMNVLTD
DYNFKMGMNGFHRPQSKIHINRFIDSYLQSQICIATTPGSGSDNDSYIHGSRVYGESESY
TRSEGRSSSIRTRTKGVELTLRERPGILDRTKKYMYLWLQCDNCYGLNYKKVLKSKMTIC
EQCGYHLQMSSSDRIELLIDPGTWDPMDEDMVSRDPIKFDSGGGEAYKDRLYFYQRKTGL
TEAVQTGIGQLNGIPVAIGVMDFKFMGGSMGSVVGEKITRLIEHATNKFLPLIIVSASGG
ARMQEGSLSLMQMAKISSALYDYQSNKRLVYVSILTSPTAGGVTASFGMLGDIIIVEPRA
YVAFAGKRVIEQTLNQTIPNDSQEAEFLFHKGLFDLIIPRHLLKSVISELFTLHDLFPLN
QNSNQYSQYRALLNPIF
>Q2EEW0
MDFISKTIGLRLGYNKTHKLIWSLSKNNPIFIQTNHFLEYIIKNDPFFCGLILIDFQLMQ
YIVVHKNQLGNFSPLKNNLPNSINLFLKLKIATEIQSNFNSVFILEERLHFLFFKYLNKM
KNLKMLNNLKIKVKIKFLNNPILEPQWIFYKLHQDLYKGINIRRALAKISSNVIKKGAEG
VKIQIKGRINGVDKATVIVEEKGKMPLQNLSSEINYYSRALKTVYGLLGVKIWVFKGFLL
K
>Q2EEW1
MIYIPKKIRYRKHHRGRLKGVASKGNNLFFASCGLQALESSWITSQQMEAARRACTRKVK
RHGKLFMRFFPDKPVTYRSAETRMGSGKGTPRDWVAVIKPGKILYELKGIPEPLARKALK
LASAKLPLKTKFIFKKE
>Q2EEW2
MIQAQTILQVADNTGAKEIMCIRVLRGSSKKGARVGDIIVAVVKKTKKQTSVKKSEIVRA
VIVRTRTTIHRANGMHLKFNENAAVLVTKELHPKGTRLFGPIPYECSEAGLNSIISLAPY
II
>Q2EEW3
MKNDFFNYYKTKIIPQYLKDYNKKKALEVPVIKKIVISRGIGKANTLEINNYIEESIKEF
LIMTGQKPKLNRAKKHIAAFKIKKKMILGLSVTLRGAKMYAFLYKFINIILPNIRQFQGI
ENKQFDSLGNITFPIYSQSNIPELPYNPRVEKRGFNVTINIKAKNVSEAKALCAYLGFPI
I
>Q2EEW4
MPRDLFSDALTRLRNALRVNKQTVIIPFSNLNYSFSNILLEEHLLEKKTILSKKYIKLEG
LKFYNKKSDHTLFNFFKNCQRLSKPSNRLYIKAGDIPLIDNGLGFVILSTSYGLMTGKKA
RALNIGGELLCQFNFQ
>Q2EEW6
MPFLLNNFKFSRFRSFATKLEKTKGFFNYQPLTKFKQKYSLDVAYVYIISSYNNTLISLT
DFLGNVLKNESCGSCNYKGRFKRKFIASKQAAENIVSFCKFQRIKRLVIILHGYGKGSEM
VIRTIREKELKILDIKIKDKKPYNGCRQKKKRRI
>Q2EEW7
MLIKQLSSSYNEYNNNYAASFLIGPLPEGIAQSLGFNIRSVLLKEFPYFAVKNIRFNPEN
NNYKTHIGTKESIETIINTILGIDFNYSFDFYKAMGLYKSKPKALFQENLSKAKFLCSAK
QVGEGILYAKDLMLPVGIKCTTPYKIIAHVTKQNFLGFTFELGFLFGLNNNIKFENKLYS
IKTYPIQNINIEIINSKKSFNLNSESIILSFTTKSSLQPIDVIKILSYLLLNKHLILIKA
FQQFLD
>P30060

MPTIKQLIRKKRQPNLNVTKSPALRGCPQRRGTCTRVYTITPKKPNSALRKVARVRLTSG
IEITAYIPGIGHNLQEHSSVLVRGGRVKDLPGVRYHIIRGTLDAVGVKDRQQGRSKYGVK
IKNK
>Q2EEW8
MTFNKKINEIITLLSDLNILELNSLINEIRNQFQIPDIVSTIQTGNKTKPEANIIEQKES
AEIKKSNFELILQTVPTEKRVAVLKAIRNTTSLDLKGAKDSIADLPKKLLDNLSLEEAEK
AKTLLEEAGATVILA
>Q9TJQ6
MMLNLLQYPVIKTPKATRLLENNQYSFDIDKKLTKPQIKKLVEEYFNIKVLAINTHRPPR
KIKRSGSAPRSKRVIITVSANINLLG
>P30061
MYKFKRSFRRRLSPIGSGNLIYYRNMSLISRFISEQGKILSRRVNRLTLKQQRLITIAIK
QARILSLLPFINNEKQFERIESITRVKGFIKK
>Q2EEW9
MKYFIIRSLINKSKNFYNTIIKYNKTKLFALELSIISPQEIQNSAEYKREDGSIVGKVED
STFYDLEKVNNKNLFSQQIFGPLIDFTCACGKKLNRKNKEINVCTKCGIEFLPSSIRSKR
KGYIKLNYAMLHPFYIDYCEKLLMKSKKSLHLLLNMDSFFYFPSYKNWVNFLSEKNHIYL
FLLKKILIYNRYSYIYKYSTFFKNRSKFPNNKKMNKLFFYNALGFYGLNSRKTWTVLDFI
KGYNFFLGNFSKYYKKLSWYAYQQKISLNKYKSNNISINNFIDNTFNFYKNEVNSLCFKV
GGDGIEMFFLQDMAVYSIINKFLKLRVLKLNNLINVNNKYYGKINTIIQNSKNYQYYFKH
FYLKKIAPVWMTLRRIPVLPPNLRPILDLSGKNYQKTASSGTLLFMRDIMHEGNLFISDI
NTFYREIIIHNKKAFNFFSSLPTLYYLNELNIEYVNSKLWLLKYYLMPIQKSITSLFDKN
PETINNFSNKKFTDLILDNVKPTSILDSLKGKYGKIRFNLLGKRVDYSGRSVIISAPHLK
IYECGIPYEMALTLYYPFLSEYFYKKNNNILKKNYNKSELVLYSKSLIFTKSLQSILLSH
PIIINRAPTLHRLGIQSFLPKLTHSKAIELHPLVCPAFNADFDGDQMAIHVPITEIAKLE
AIQLMASSLFVYAPASGLPLLIPTQDIILGFNFYTNDLLLKTSREDKSIKQAMNQGFINE
CHIPYWIKINTKDIFFKFLAYPLRNYIIPIELQLNIKGFSKIIRLNTFKVTNLVFDSLDL
LIKKNYISNWLILNNTKFKYLLISFLQNKNLLTNKQVYLRTTLGNIYFNKYLNL
>P30401
MSPQTETKASVGFKAGVKDYKLTYYTPDYETKDTDILAAFRVTPQPGVPPEEAGAAVAAE
SSTGTWTTVWTDGLTSLDRYKGRCYRIERVIGEKDQYIAYVAYPLDLFEEGSVTNLFTSI
VGNVFGFKALRALRLEDLRIPPAYTKTFQGPPHGIQVERDKLNKYGRPLLGCTIKPKLGL
SAKNYGRAVYECLRGGLDFTKDDENVNSQPFMRWRDRFLFCAEAIYKSQAETGEIKGHYL
NATAGTCEEMLKRALFARELGVPIIMHDYLTGGFTANTSLAHYCRENGLLLHIHRAMHAV
IDRQKNHGIHFRVLAKALRLSGGDHIHSGTVVGKLEGEREITLGFVDLLRDDFVEQDRSR
GIYFPQDWVSLPGVMPVASGGIHVWHMPALTEIFGDDSVLQFGGGTLGHPWGNAPGAVAN
RVALEACVQARNEGRDLAQEGNDILRQAGKWSPELAAACEVWKEIRFDFKPVDTLDPNDK
KQRDNEDTLADKLFGDKG
>Q9TJQ7
MTNFKDIIKKIEYKFSKHQLPDIKVGDLIRLGISIQESGKQRVQPFEGTVIALHKAGLNT
TITVRKILQGIGVERVFPIHASCLTSIQVLRRSQVSRAKLYYLRNRTGKATRLKEKFEKL
PPIWVNKLP
>Q9TIR8
MEQIRRYLQLERFQQHDFLYPXFFQEYIYVFAHGRGFSRSIWSENENTGYDNKSSLRVMK

RLITRMYQQNNFIILPNDFNKKNPFLAKKKFFYSQIIAEGFAFIVEIPFSLRFISYLEGK
KKIVKSQNLRSIHSIFPFLEDNFSHLNFVLDILILHPVHVEILVQILRYWVKDASSLHLL
RFFLNKYWNSLITPNKASSSLSKKNKRLFVFLYNSHVGGYESSFVFIRNQSSHLGSTPFG
VLLERIYFYGKIERLVNVFVKVKDFRTNLWFVKEPYIHYIRYQRKWILASKGTFLFVKKW
KCYLIIFWQWHFSLWFYPRRIYINQLSNHYFEFLGYLSNLRMTPSVVRSQSLENTFIINN
AIKKIDNFVPIISMIASLAKAKFCNVFGHPISKPVRADLSDSNIIDQFGCICRKFFHYYS
GSSKKKSLYRIKYILRLACARTLARKHKSTVRTFLKRLGSELLEEFLLSEEDVLFLTFPK
ASSSLQGVYRSRIWYLDIIYINDLVDHKYKL
>Q9TJQ9
MSRRRTAKKRIVMPDPVYKNSLLELIVRQVMRNGKKLLAYRIMYNSMIKIAEMTQKDPLD
VLEKAIRNVTPLIEVKARRVGGSTYQVPLEVLPERGTTLAIRWILAACRKNRGKPMYIKL
TNELIDASNKSGSAIKKKDEIHRMAEANKAFAKQRF
>P82412
MLSMAVQPNINAIAKPSIYQSPKLSLKPFKTPAFANPKPFFSSPSFSQLKKKNNWSLFVA
PETISDVAIMGNEVDIDDDLLVNKEKLKVLVKPMDKPRLVLKFIWMEKNIGLALDQTIPG
HGTVPLSPYYFWPRKDAWEELKVLLENKPWISQKQMIILLNQATDIINLWQQSGGNLAS
>P30067
MTRIKRGYIARRRIKKFRLFASSFLNAHSRLTRTITQQKIRALVSSDRDRNNKKRKFRSL
WITRINAVIREEGVSYSYKNFIYAQYKIQLLINRKILAQIAILNRNFFYMIFNEIRKEAD
LKEYIRIN
>P30068
MAKSKDARVAVILECTSCIRNSVNKVLTGISRYITQKNRRNTPNRLELRKFCPYCYKHMI
HGEIKK
>A8W3F3
MVREKITVSTRTLQWKCVESRTDSKRLYYGRFILSPLRKGQADTIGIAMRRALLAEIEGT
RITRVKFAKASHEYSTIAGIQESVHEIFMNLKEIVLRSNLYGTCDASISIKGPGHVTAKD
IILPPHVEIVDSTQHIAWLTEPIDLCIGLKIERNRGYFIKTHPNFEDGSYPIDAVFMPVR
NANHSIHSYGNEKQEILFLEIWTNGSLTPKEALHEASRNLIDLFIPFLHMEEENLHLEDA
DHTISLSPFTVYDKVAKLRKNKKKRSLESIFIDQLEFPPKIYNCLKKSNIFTLLDLLNNS
QEDLIKIEHFRLEDVKQILGILGKHFALDLPKKKF
>P25699
MALAPSKVSPFSGFSLSDGVGAVRNPTCSVSLSFLNKKVGSRNLGVSASTVPLTGVIFEP
FEEVKKEELAVPTAGQVSLARQYYADECESAINEQINVEYNASYVYHSLFAYFDRDNVAL
KGFARFFKESSEEEREHAEKLMKYQNTRGGRVVLHPIKNVPSEFEHVEKGDALYAMELAL
SLEKLVNEKLRSVHSVADRNKDPQLADFIESEFLSEQVEAIKKISEYVAQLRMVGKGHGV
WHFDQSLLHDGHAA
>A7M988
MPIGVPRVRFQYRRDRSRVWIDIYNRLYRERCLFLAHVVESQIANQLVGLFIYLGVQDET
KDIFLFINSPGGGIISGFAIYDTMQFVRPDIQTICVGLAASMGSFLLVGGAITKRLAFPH
ARVMIHQPMSAFFETQTVEAILEAEELLKLRESLAKVYVQRTGKPDWVIAEDMERDVFLS
ATEAQSYGIVDVVGVALASKG
>O47032
MSRYRGPRLPIIRRLGELPGFSKKIDRNHTPPGQHGWKKKASDQKKSKESQYGIRLKEKQ
KLRYNYGINERQLINYVREARRRKGSTGEVLLQLLEMRLDNIIYRLGFAPTIPAARQLIN

HGHINVNNKNINIPSYICKINDIISVLKNSQQLIKNYLQNGGISELSTCLNLNKEKLEAS
INNIIPRDLVKLEINELLVIEYYSRKL
>Q2EEX0
MIKTSQIAKYIFLLKKAEFIKSEKKHVILNKTTLKKMVFILQNKYDEQTVFFILEKLKEI
GFHLATQSGLSLGIDDLKSFPDKKKLITETRTDLNNNEEHFLLQNISPLEKSKYLITKWN
DVNNFIIQRIKKVFLKKEPLNPLALMAFSGARGNISQVSQLIGLRGLMTDPLGKLVEFPI
QGNFREGMTLTEYFISCYGARKGLVDTALRTATAGYLTRRLVYVVQHIFISIEDCNVTNG
ISVEINEKSSNSLIGLTLLKNFYNKNIFIAKNTIISPTLVKLLLNSYNYLSHKKSIVCRS
ILTCQLQHTLCQFCYGWDNARAKRVAIGEAIGIIAAQSIGEPGTQLTLRTFHTGGVGGFS
GANWISYNSPFDGFIKFKKPVIGNILRSWIVMPHYTSLNLVYKLTPSFNELNLTLFIFDK
LKNELYNKTLKNEGLLFVREGDFIKKGQLLFSEININFKNSNLLKTYIPYYSLETGLIIS
PKLALNKYLYPLWIFLFNIIKLNLDSLNSKKFTVYKNYSILEKNDLIDLKTILFEIHFIQ
KSKKFNLKKQEYLLYQNLKMSLCKSHYDYSTYKKLNVAFQQNNQKLNFFYNKKLKSSLVS
NNINLLWTSSSYSINILTKSIFNIIVFEYLYLSLLIKNKYILWILSKNLKKKDKFHFFKY
TSTSSSKIEIFRLNSKKNSLNIRKNKLFFYSQNYKKNKVSNILFGNSIFLKFPLKKINTF
FSQIKQKTINLKEKKTSNVEGEFIGFQLKNNIKFWYSISPNSLFSFKKDNKKNRYNFNFI
RKNSNISGFLVSNTIKTFTLQKGIPLIFPSDTIYHKMEKDFIFKNELLCSFKSLKPETQD
IIQGIPRVEKLLEGRPLPFYKPKVELYKIWQFFLKNLGFLKIKNSLFNLKKVESKVYLNN
FKPNLNIGDICYLAYFHSKSNFGLDIASIISNVYLQEGVNISFKHIELIVRELTSLVEII
NPGDSGFIVKEKIHWLLIYNYNKRLKSLNLKEISYMPIFLGMTEITKKKNSFGVAGSFQN
LREIIIDHTFTRKTDYLLGLHENVLFNKIIPAGTGFFF
>Q2EEX2
MLLNDQKFLPINIHYLNQGRFDLNKLKNFNLLINQRENWRFFLINGLKLAFKEFEHFKIK
NNNFIIDIVFQEKFLFFKQIRQAYLKKVETFGYNIFIPIIITQKILNKKNNLNLTFFKWL
DLGILPSLSPKTSFYIDGISRAVSTQKKKSPGLMFDFDLTKKSSLEKREGEAFLKIIAPR
GSWFKISYSEDHIIFIEIRNIAYKIPIFTFLCALGFSLESIFQFFNYNINNFKLIPNKYD
NTNALIKARNDIFILYSDLLPLLNKTKKNFEIKYIINHYFFSYIWNSNTRYCGEATRLHL
YNKVGSPLSLNSLFLEEIDFIYITKIFIYRLINNNFKDENNDNLKNMRFRNGGDYIYLQT
RQGLFDFESFLNINLHNIENNSFFCNITTNKKKEDIYSIKKKKNIKNFNLSKISFLKHIL
LKSWKSFFLSGTLSQFAENLNPLSYITHARRFTSLGPGSLKKAEASIEVRSINPSYNGRV
CPIETPEGFNAGLVHSFSLYSFKGVNGELLLPFYFIYKNIKQSYYLPPIFFNIEEESNQS
IITGDLIHEKWNDFLNKKLSLRNNFHLDKNDISKISFQSLSPYHMISLATSLIPFLEHND
ANRALMGSNMQRQTVPVLGAHNFIVRTGLDTKFFSDISSIPISQINGFNFESNNNYCYFY
KIDNLITLNNISYNYIKKTNHNSLYINESIKKKNPWHQQGDFLINNSVIENGKLALGPNL
LLAYLPWHGYNYEDAVIINERCVSQKILTSLHIITLTNSLELAYREINKKKEFFYEVPVK
LEILFSFFAPFYYNLKYYKNQEFSQFEKKNPSYYTNNINIGTFLKKGDCIFSKCRIRFSF
SRELRLQYKELFILLREAFPTFEDFKNFANIFAYDKYKQKLDRKKQKKNTRLKTDNFIFN
KNSTLFKKHKLKALINYYNIEDSTIYAQKNQEGLIISKHLKKVPMTYTSITPFDLEEFLS
TKESKKKEYYNLFFTKSINIEILQLRDIQLGDKCAGRHGNKGIISKICPINEMPLLPDGT
PIDIILNPLGIPSRMNVGQIFESLLGLAGFYLNETYTINIFDEQFGLEASRSFVLSKLYK
ASILSNKPWLFLKKNAGKTNIIDGLTGKYFQQPITIGYSSILKLIHMVTDKLHGRSIGGY
STLTLQPTKGKSLLGGQRVGEMELWALQGYGASWALQELYTLKSDLVEARQHFQNYINSF
NIESNIYDRNDISLTELESLIISDTFKPYTLECFLEDLKALCIYIDY
>Q2EEV7

MAREKFERIKPHINIGTIGHVDHGKTTLTAAITMALASIGNTKGKNYADIDSAPEEKARG
ITINTTHVEYETAKRHYAHVDCPGHADYIKNMITGAAQMDGAILVVSGADGPMPQTREHI
VLAKQVGVPSMVVFINKEDQVDDPEILELVELEVRDLLTSYKFEGEEVPVITGSALLALE
AFIKNPKILKGENPWVDKIYNLMDSVDSYIPTPVREIDKPFLMAIEDVFSISGRGTVATG
RIERGKIKMGDSIEIIGGSLRKTTTVTGIEMFQKTLTDGVAGDNVGILMRGIQKKEIDRG
MVLTKPKSIDPLTSFEAQVYLLTKEEGGRSKGFTIGYRPQFYVRTTDVTGAILNMLSDDN
TPLKIASPGDRITMSVKLIQPIALEKNMRFAIREGGKTVGAGVVSKLIN
>Q2EEX3
MAKKSLIVREYKKNILVNKYYKKRLLLKSQLNNTINSTDCILKILNKLQKLPKKSSPTKL
RNRCTLTGRARGVYSEYKISRFVFRNLALNGLLPGVFKASW
>Q2EEV8
MTLKKLNPITPGSRHRVIIDYSKQSKLLKPSTKNAPLKKNTLKIMTTEGRNFHGVITTRH
RGGGHKRLYRIIEFNRNKLGVSGKVNNIEYDPNRTANIARIHYQDGSKKYIIHPEGLEKG
ANIIASAIAPLSIGNSLPLKSIPIGTEIHNIEFHPGKGGQLARSAGTGALVSYKTNSFVT
LKLPSGRSRYFDNKCWATIGKVSKAEHKFINLGKAGRSRWLGIRPTVRGSAQNAVDHPHG
GGEGKAPIGRIPSTPWGKPALGIKTRHRHKWSERFFKGLKKK
>P27068
MTRRYWNIDLEEMIGAGVSFGHGTRKWNPKMAPYISVKHKGIHFTNLTKTARFLSEACDL
VFYAASRGKQFLIVDTKNKAADSAAWAAIKARCHCVNKKWPGGMLTNWSTTETRLHKLRD
LIMEQKAGRLNRLKKKDEAAVKRQLARLQTYLGGIKYMTRLPDIVIIVDQHEEYKALHEC
ITIGIPTIGLIDTNCDPDLADISIPANDDAISSIRLILNKLVFAICEGRSGYIIKNI
>Q2EEV9
MGINNINKIPVRNNKISFVAVHLIKALTSKKQEKLVKLQRKKKIISVIKTWSRSSTIVPL
MIGEIIAVHNGREHIPVYITEEMVGFKLGEFATTRIWRGHLKKTTHKKNNKTYGLYF
>P30070
MKEQKWIHEGLITESLTNGMFWVRLDNKDLIIGYVSGNIRHSFIRILPGDKVKIEVSRYN
STRGRIIYRLRNKYYKD
>Q2EEX9
MLKNIIYSYITQLKNLLSKKLNVLCTFSAGQDSFFLLYCLIHIISNKNNKIKLQHNHHFL
QSSNILSFWQCIKVASIFKIPLVINLLEINLANKDFMTENEARKWRYDSFLRNSLFQNEK
PSIFIGHTGSDLLETFFWHFLRNSIIDYQLIKKKLLWNIPYYISNFSSVGYKNSSKVKIK
LTNKKKKINCLINHNLMLNKAKKSFLKKDLTQLVLITRPLVNLHRQDIFLFRKNLKLPVI
TDKSNDNNYYYRNRIRNILFPIMRILFNKKTDKNLIKLFL
>P32034
MYGWYELPKQEFLNSKQPVHIFTTKKYWILFRIGPERRRKAGMPKASIISFTRPNPVWER
PVPKSLTGLFNVLYLLG
>P32035
MEYMKRVEYLYRSVMSYRTQLDIQLLRFELSGECLIDISKRWTIKPLSRFTQRGE
>A7M934
MGQKINPLGLRLGTNQDHYSIWFSQPKAYSKSLQEDQKIRSFIRNYIKNMKISPSGVEGL
ARISIYKRIELIEVRIFLGFPKLLLENRPEGLEELQITLQKELNCGNRRLNILITKVEKP
YSNPNILAEFIAGQLKNRVSVRQAMKKAIELAEEADTKGIQVQVAGRLNGQDIARVQWIR
EGRVPRQTIRATFDYCSYPVRTIYGILGIKIWIFVGEDK

(12) $\mathbb{S}_{12}$: 52 vacuole proteins

>P08031
MKVLIVALALLALAASAASSTSGGCGCQTPPFHLPPPFYMPPPFYLPPQQQPQPWQYPTQ
PPQLSPCQQFGSCGVGSVGSPFLGQCVEFLRHQCSPAATPYGSPQCQALQQQCCHQIRQV
EPLHRYQATYGVVLQSFLQQQPQGELAALMAAQVAQQLTAMCGLQLQQPGPCPCNAAAGG
VYY
>Q9LDC0
MDESLEHQTQTHDQESEIVTEGSAVVHSEPSQEGNVPPKVDSEAEVLDEKVSKQIIKEGH
GSKPSKYSTCFLHYRAWTKNSQHKFEDTWHEQQPIELVLGKEKKELAGLAIGVASMKSGE
RALVHVGWELAYGKEGNFSFPNVPPMADLLYEVEVIGFDETKEGKARSDMTVEERIGAAD
RRKMDGNSLFKEEKLEEAMQQYEMAIAYMGDDFMFQLYGKYQDMALAVKNPCHLNIAACL
IKLKRYDEAIGHCNIVLTEEEKNPKALFRRGKAKAELGQMDSARDDFRKAQKYAPDDKAI
RRELRALAEQEKALYQKQKEMYKGIFKGKDEGGAKSKSLFWLIVLWQWFVSLFSRIFRRH
RVKAD
>P15797
MSTSSHKHNTPQMAAITLLGLLLVASSIDIAGAQSIGVCYGMLGNNLPNHWEVIQLYKSR
NIGRLRLYDPNHGALQALKGSNIEVMLGLPNSDVKHIASGMEHARWWVQKNVKDFWPDVK
IKYIAVGNEISPVTGTSYLTSFLTPAMVNIYKAIGEAGLGNNIKVSTSVDMTLIGNSYPP
SQGSFRNDARWFTDPIVGFLRDTRAPLLVNIYPYFSYSGNPGQISLPYSLFTAPNVVVQD
GSRQYRNLFDAMLDSVYAALERSGGASVGIVVSESGWPSAGAFGATYDNAATYLRNLIQH
AKEGSPRKPGPIETYIFAMFDENNKNPELEKHFGLFSPNKQPKYNINFGVSGGVWDSSVE
TNATASLVSEM
>P07052
MNFLKSFPFYAFLCFGQYFVAVTHAATFDIVNQCTYTVWAAASPGGGRQLNSGQSWSINV
NPGTVQARIWGRTNCNFDGSGRGNCETGDCNGMLECQGYGKPPNTLAEFALNQPNQDFVD
ISLVDGFNIPMEFSPTNGGCRNLRCTAPINEQCPAQLKTQGGCNNPCTVIKTNEFCCTNG
PGSCGPTDLSRFFKARCPDAYSYPQDDPPSLFTCPPGTNYRVVFCP
>P04347
MGKPFFTLSLSSLCLLLLSSACFAITSSKFNECQLNNLNALEPDHRVESEGGLIETWNSQ
HPELQCAGVTVSKRTLNRNGSHLPSYLPYPQMIIVVQGKGAIGFAFPGCPETFEKPQQQS
SRRGSRSQQQLQDSHQKIRHFNEGDVLVIPLGVPYWTYNTGDEPVVAISPLDTSNFNNQL
DQNPRVFYLAGNPDIEHPETMQQQQQQKSHGGRKQGQHRQQEEEGGSVLSGFSKHFLAQS
FNTNEDTAEKLRSPDDERKQIVTVEGGLSVISPKWQEQEDEDEDEDEEYGRTPSYPPRRP
SHGKHEDDEDEDEEEDQPRPDHPPQRPSRPEQQEPRGRGCQTRNGVEENICTMKLHENIA
RPSRADFYNPKAGRISTLNSLTLPALRQFGLSAQYVVLYRNGIYSPDWNLNANSVTMTRG
KGRVRVVNCQGNAVFDGELRRGQLLVVPQNPAVAEQGGEQGLEYVVFKTHHNAVSSYIKD
VFRVIPSEVLSNSYNLGQSQVRQLKYQGNSGPLVNP
>P07053
MGFFLFSQMPSFFLVSTLLLFLIISHSSHAQNSQQDYLDAHNTARADVGVEPLTWDNGVA
AYAQNYVSQLAADCNLVHSHGQYGENLAQGSGDFMTAAKAVEMWVDEKQYYDHDSNTCAQ
GQVCGHYTQVVWRNSVRVGCARVKCNNGGYVVSCNYDPPGNVIGQSPY
>P19825
MAHQHTISGLVGKLITESEVNCNADKYYQIFKHHEDLPSAIPHIYTSVKAVEGHGTTSGC
VKEWCYILEGKPLTVKEKTTYNDETRTINHNGIEGGMMNDYKKFVATLVVKPKANGQGSI
VTWIVDYEKINEDSPVPFDYLAFFQQNIEDLNSHLCASD

>P08688

MTKTGYINAAFRSSQNNEAYLFINDKYVLLDYAPGTSNDKVLYGPTPVRDGFKSLNQTVF
GSYGVDCSFDTDNDEAFIFYEKFCALIDYAPHSNKDKIILGPKKIADMFPFFEGTVFENG
IDAAYRSTRGKEVYLFKGDQYARIDYETNSMVNKEIKSIRNGFPCFRNTIFESGTDAAFA
SHKTNEVYFFKGDYYARVTVTPGATDDQIMDGVRKTLDYWPSLRGIIPLEN

>P04706

MRVLLVALALLALAASATSTHTSGGCGCQPPPPVHLPPPVHLPPPVHLPPPVHLPPPVHL
PPPVHLPPPVHVPPPVHLPPPPCHYPTQPPRPQPHPQPHPCPCQQPHPSPCQLQGTCGVG
STPILGQCVEFLRHQCSPTATPYCSPQCQSLRQQCCQQLRQVEPQHRYQAIFGLVLQSIL
QQQPQSGQVAGLLAAQIAQQLTAMCGLQQPTPCPYAAAGGVPH

>Q2V4F9

MVAFGKYLQRKQIEEWSGYYINYKLMKKKVKQYAEQIQGGSQHPRHVLKDFSRMLDTQIE
TTVLFMLEQQGLLSGRLAKLRESHDAILEQPDISRIFELREAYRDVGRDLLQLLKFVELN
AIGLRKILKKFDKRFGYRFADYYVKTRANHPYSQLQQVFKHVGVGAVVGAISRNLHELQE
NEGSFYSIYDQPVLPAQDPVVEAINNAVDKLTFSTNFLNFLAQHALIMQDDLVTPSEDTI
DERSYHFNSLLLNLGNTFLYMVNTYIIVPTADDYSMSLGAAATVCGVVIGSMAVAQVFSS
VYFSAWSNKSYFKPLVFSSIALFIGNLMYALAYDANSIALLLLGRVCCGLGSARAVNRRY
ISDCVPLRIRMQASAGFVSASALGMACGPALAGLLQIKFKFYKFTFNQSTLPGWVMAVAW
LFYLVWLCISFREPLRDTEDGEKNNRNETTSDRVESSRVEEGLRLPLLITSGIKPEDEEE
CDESEESPEDSHKPANSFIEAYRLLTPSVKVQLLIYFMLKYSMEILLSESSVITSYYFSW
TTSSVAIFLACLGLTVLPINILVGSYISNMFEDRQILLTSEIIVFLGILFSFNLFVPYTV
PQYVISGLIMFVAAEVLEGVNLSLLSRVMSSRLSKGTYNGGLLSTEAGTLARVVADATIT
LGGYLGRGHLLNATLLPSLVICIGSIVATCCTYNSLY

>Q9M8U1

MSCDGGKPAPAKLGDEQLAELREIFRSFDQNKDGSLTELELGSLLRSLGLKPSQDQLDTL
IQKADRNNNGLVEFSEFVALVEPDLVKCPYTDDQLKAIFRMFDRDGNGYITAAELAHSMA
KLGHALTAEELTGMIKEADRDGDGCIDFQEFVQAITSAAFDNAWG

>P17990

MKILIILTILAMATTFATSEMQVNPSVQVQPTQQQPYPESQQPFISQSQQQFPQPQQPFP
QQPQQPFPQSQQQCLQQPQHQFPQPTQQFPQRPLLPFTHPFLTFPDQLLPQPPHQSFPQP
PQSYPQPPLQPFPQPPQQKYPEQPQQPFPWQQPTIQLYLQQQLNPCKEFLLQQCRPVSLL
SYIWSKIVQQSSCRVMQQQCCLQLAQIPEQYKCTAIDSIVHAIFMQQGQRQGVQIVQQQP
QPQQVGQCVLVQGQGVVQPQQLAQMEAIRTLVLQSVPSMCNFNVPPNCSTIKAPFVGVVT
GVGGQ

>P49175

MIPAVADPTTLDGGGARRPLLPETDPRGRAAAGAEQKRPPATPTVLTAVVSAVLLLVLVA
VTVLASQHVDGQAGGVPAGEDAVVVEVAASRGVAEGVSEKSTAPLLGSGALQDFSWTNAM
LAWQRTAFHFQPPKNWMNDPNGPLYHKGWYHLFYQWNPDSAVWGNITWGHAVSRDLLHWL
HLPLAMVPDHPYDANGVWSGSATRLPDGRIVMLYTGSTAESSAQVQNLAEPADASDPLLR
EWVKSDANPVLVPPPGIGPTDFRDPTTACRTPAGNDTAWRVAIGSKDRDHAGLALVYRTE
DFVRYDPAPALMHAVPGTGMWECVDFYPVAAGSGAAAGSGDGLETSAAPGPGVKHVLKAS
LDDDKHDYYAIGTYDPATDTWTPDSAEDDVGIGLRYDYGKYYASKTFYDPVLRRRVLWGW
VGETDSERADILKGWASVQSIPRTVLLDTKTGSNLLQWPVVEVENLRMSGKSFDGVALDR
GSVVPLDVGKATQLDIEAVFEVDASDAAGVTEADVTFNCSTSAGAAGRGLLGPFGLLVLA

DDDLSEQTAVYFYLLKGTDGSLQTFFCQDELRASKANDLVKRVYGSLVPVLDGENLSVRI
LVDHSIVESFAQGGRTCITSRVYPTRAIYDSARVFLFNNATHAHVKAKSVKIWQLNSAYI
RPYPATTTSL
>Q01594
MVESYKKIGSCNKMPCLVILTCIIMSNSLVNNNNMVQAKMTWTMKAAEEAEAVANINCSE
HGRAFLDGIISEGSPKCECNTCYTGPDCSEKIQGCSADVASGDGLFLEEYWKQHKEASAV
LVSPWHRMSYFFNPVSNFISFELEKTIKELHEVVGNAAAKDRYIVFGVGVTQLIHGLVIS
LSPNMTATPDAPESKVVAHAPFYPVFREQTKYFNKKGYVWAGNAANYVNVSNPEQYIEMV
TSPNNPEGLLRHAVIKGCKSIYDMVYYWPHYTPIKYKADEDILLFTMSKFTGHSGSRFGW
ALIKDESVYNNLLNYMTKNTEGTPRETQLRSLKVLKEVVAMVKTQKGTMRDLNTFGFKKL
RERWVNITALLDQSDRFSYQELPQSEYCNYFRRMRPPSPSYAWVKCEWEEDKDCYQTFQN
GRINTQNGVGFEASSRYVRLSLIKTQDDFDQLMYYLKDMVKAKRKTPLIKQLFIDQTETA
SRRPFI
>P59229
MASSGFSGDETAPFFGFLGAAAALVFSCMGAAYGTAKSGVGVASMGVMRPELVMKSIVPV
VMAGVLGIYGLIIAVIISTGINPKAKSYYLFDGYAHLSSGLACGLAGLSAGMAIGIVGDA
GVRANAQQPKLFVGMILILIFAEALALYGLIVGIILSSRAGQSRAE
>Q9C8G9
MGFEPLDWYCKPVPNGVWTKTVDYAFGAYTPCAIDSFVLGISHLVLLILCLYRLWLITKD
HKVDKFCLRSKWFSYFLALLAAYATAEPLFRLVMRISVLDLDGAGFPPYEAFMLVLEAFA
WGSALVMTVVETKTYIHELRWYVRFAVIYALVGDMVLLNLVLSVKEYYGSFKLYLYISEV
AVQVAFGTLLFVYFPNLDPYPGYTPVGTENSEDYEYEELPGGENICPERHANLFDSIFFS
WLNPLMTLGSKRPLTEKDVWHLDTWDKTETLMRSFQKSWDKELEKPKPWLLRALNNSLGG
RFWWGGFWKIGNDCSQFVGPLLLNELLKSMQLNEPAWIGYIYAISIFVGVVLGVLCEAQY
FQNVMRVGYRLRSALIAAVFRKSLRLTNEGRKKFQTGKITNLMTTDAESLQQICQSLHTM
WSAPFRIIVALVLLYQQLGVASIIGALFLVLMFPIQTVIISKTQKLTKEGLQRTDKRIGL
MNEVLAAMDTVKCYAWENSFQSKVQTVRDDELSWFRKAQLLSAFNMFILNSIPVLVTVVS
FGVFSLLGGDLTPARAFTSLSLFSVLRFPLFMLPNIITQMVNANVSLNRLEEVLSTEERV
LLPNPPIEPGQPAISIRNGYFSWDSKADRPTLSNINLDIPLGSLVAVVGSTGEGKTSLIS
AMLGELPARSDATVTLRGSVAYVPQVSWIFNATVRDNILFGAPFDQEKYERVIDVTALQH
DLELLPGGDLTEIGERGVNISGGQKQRVSMARAVYSNSDVCILDDPLSALDAHVGQQVFE
KCIKRELGQTTRVLVTNQLHFLSQVDKILLVHEGTVKEEGTYEELCHSGPLFQRLMENAG
KVEDYSEENGEAEVDQTSVKPVENGNANNLQKDGIETKNSKEGNSVLVKREERETGVVSW
KVLERYQNALGGAWVVMMLVICYVLTQVFRVSSSTWLSEWTDSGTPKTHGPLFYNIVYAL
LSFGQVSVTLINSYWLIMSSLYAAKKMHDAMLGSILRAPMVFFQTNPLGRIINRFAKDMG
DIDRTVAVFVNMFMGSIAQLLSTVILIGIVSTLSLWAIMPLLVVFYGAYLYYQNTSREIK
RMDSTTRSPVYAQFGEALNGLSSIRAYKAYDRMAEINGRSMDNNIRFTLVNMAANRWLGI
RLEVLGGLMVWLTASLAVMQNGKAANQQAYASTMGLLLSYALSITSSLTAVLRLASLAEN
SLNSVERVGNYIEIPSEAPLVIENNRPPPGWPSSGSIKFEDVVLRYRPELPPVLHGVSFL
ISPMDKVGIVGRTGAGKSSLLNALFRIVELEKGRILIDECDIGRFGLMDLRKVLGIIPQA
PVLFSGTVRFNLDPFSEHNDADLWESLERAHLKDTIRRNPLGLDAEVTEAGENFSVGQRQ
LLSLARALLRRSKILVLDEATAAVDVRTDVLIQKTIREEFKSCTMLIIAHRLNTIIDCDK
VLVLDSGKVQEFSSPENLLSNGESSFSKMVQSTGTANAEYLRSITLENKRTREANGDDSQ
PLEGQRKWQASSRWAAAAQFALAVSLTSSHNDLQSLEIEDDNSILKKTKDAVVTLRSVLE

GKHDKEIEDSLNQSDISRERWWPSLYKMVEGLAVMSRLARNRMQHPDYNLEGKSFDWDNV
EM
>P23472
MAKRTQAILLLLLAISLIMSSSHVDGGGIAIYWGQNGNEGTLTQTCSTRKYSYVNIAFLN
KFGNGQTPQINLAGHCNPAAGGCTIVSNGIRSCQIQGIKVMLSLGGGIGSYTLASQADAK
NVADYLWNNFLGGKSSSRPLGDAVLDGIDFDIEHGSTLYWDDLARYLSAYSKQGKKVYLT
AAPQCPFPDRYLGTALNTGLFDYVWVQFYNNPPCQYSSGNINNIINSWNRWTTSINAGKI
FLGLPAAPEAAGSGYVPPDVLISRILPEIKKSPKYGGVMLWSKFYDDKNGYSSSILDSVL
FLHSEECMTVL
>P30941
MKCLFLLCLCLVPIVVFSSTFTSKNPINLPSDATPVLDVAGKELDSRLSYRIISTFWGAL
GGDVYLGKSPNSDAPCANGIFRYNSDVGPSGTPVRFIGSSSHFGQGIFENELLNIQFAIS
TSKLCVSYTIWKVGDYDASLGTMLLETGGTIGQADSSWFKIVKSSQFGYNLLYCPVTSTM
SCPFSSDDQFCLKVGVVHQNGKRRLALVKDNPLDVSFKQVQ
>P46032
MGSIEEEARPLIEEGLILQEVKLYAEDGSVDFNGNPPLKEKTGNWKACPFILGNECCERL
AYYGIAGNLITYLTTKLHQGNVSAATNVTTWQGTCYLTPLIGAVLADAYWGRYWTIACFS
GIYFIGMSALTLSASVPALKPAECIGDFCPSATPAQYAMFFGGLYLIALGTGGIKPCVSS
FGADQFDDTDSRERVRKASFFNWFYFSINIGALVSSSLLVWIQENRGWGLGFGIPTVFMG
LAIASFFFGTPLYRFQKPGGSPITRISQVVVASFRKSSVKVPEDATLLYETQDKNSAIAG
SRKIEHTDDCQYLDKAAVISEEESKSGDYSNSWRLCTVTQVEELKILIRMFPIWASGIIF
SAVYAQMSTMFVQQGRAMNCKIGSFQLPPAALGTFDTASVIIWVPLYDRFIVPLARKFTG
VDKGFTEIQRMGIGLFVSVLCMAAAAIVEIIRLHMANDLGLVESGAPVPISVLWQIPQYF
ILGAAEVFYFIGQLEFFYDQSPDAMRSLCSALALLTNALGNYLSSLILTLVTYFTTRNGQ
EGWISDNLNSGHLDYFFWLLAGLSLVNMAVYFFSAARYKQKKASS
>Q9SZW5
MAEGEESKKMNLQTSYFDVVGICCSSEVSIVGNVLRQVDGVKEFSVIVPSRTVIVVHDTF
LISPLQIVKALNQARLEASVRPYGETSLKSQWPSPFAIVSGVLLVLSFFKYFYSPLEWLA
IVAVVAGVFPILAKAVASVTRFRLDINALTLIAVIATLCMQDFTEAATIVFLFSVADWLE
SSAAHKASIVMSSLMSLAPRKAVIADTGLEVDVDEVGINTVVSVKAGESIPIDGVVVDGS
CDVDEKTLTGESFPVSKQRESTVMAATINLNGYIKVKTTALARDCVVAKMTKLVEEAQKS
QTKTQRFIDKCSRYYTPAVVVSAACFAVIPVLLKVQDLSHWFHLALVVLVSGCPCGLILS
TPVATFCALTKAATSGFLIKTGDCLETLAKIKIVAFDKTGTITKAEFMVSDFRSLSPSIN
LHKLLYWVSSIECKSSHPMAAALIDYARSVSVEPKPDIVENFQNFPGEGVYGRIDGQDIY
IGNKRIAQRAGCLTDNVPDIEATMKRGKTIGYIYMGAKLTGSFNLLDGCRYGVAQALKEL
KSLGIQTAMLTGDNQDAAMSTQEQLENALDIVHSELLPQDKARIIDDFKIQGPTMMVGDG
LNDAPALAKADIGISMGISGSALATETGDIILMSNDIRKIPKGMRLAKRSHKKVIENVVL
SVSIKGAIMVLGFVGYPLVWAAVLADAGTCLLVILNSMMLLRDEREAVSTCYRASTSSPV
KLEEDEVEDLEVGLLQKSEETSKKSCCSGCCSGPKDNQQK
>Q56XP4
MTMFASLTSKMLSVSTSDHASVVSLNLFVALLCACIVIGHLLEENRWMNESITALLIGLG
TGVVILLISRGKNSHLLVFSEDLFFIYLLPPIIFNAGFQVKKKQFFRNFVTIMAFGAIGT
VVSCTIISLGAIQFFKKLDIGTFDLGDFLAIGAIFAATDSVCTLQVLNQDETPLLYSLVF
GEGVVNDATSVVLFNAIQSFDLTHLNHEAAFQFLGNFFYLFLLSTGLGVATGLISAYVIK

KLYFGRHSTDREVALMMLMAYLSYMLAELFALSGILTVFFCGIVMSHYTWHNVTESSRIT
TKHAFATLSFLAETFIFLYVGMDALDIEKWRFVSDSPGTSVAVSSILMGLVMLGRAAFVF
PLSFLSNLAKKHQSEKISIKQQVVIWWAGLMRGAVSMALAYNKFTRSGHTELRGNAIMIT
STITVCLFSTMVFGMLTKPLIRYLMPHQKATTSTTSMLSDDSTPKSIHIPLLDGEQLDSF
ELPGSHQDVPRPNSLRGFLMRPTRTVHYYWRQFDDAFMRPVFGGRGFVPFVPGSPTERSS
HDLSKP
>O22213
MGLTSSLRFHRQNNKTFLGIFMILVLSCIPGRTNLCSNHSVSTPKELPSSNPSDIRSSLV
SLDLEGYISFDDVHNVAKDFGNRYQLPPLAILHPRSVFDISSMMKHIVHLGSTSNLTVAA
RGHGHSLQGQALAHQGVVIKMESLRSPDIRIYKGKQPYVDVSGGEIWINILRETLKYGLS
PKSWTDYLHLTVGGTLSNAGISGQAFKHGPQINNVYQLEIVTGKGEVVTCSEKRNSELFF
SVLGGLGQFGIITRARISLEPAPHMVKWIRVLYSDFSAFSRDQEYLISKEKTFDYVEGFV
IINRTDLLNNWRSSFSPNDSTQASRFKSDGKTLYCLEVVKYFNPEEASSMDQETGKLLSE
LNYIPSTLFSSEVPYIEFLDRVHIAERKLRAKGLWEVPHPWLNLLIPKSSIYQFATEVFN
NILTSNNNGPILIYPVNQSKWKKHTSLITPNEDIFYLVAFLPSAVPNSSGKNDLEYLLKQ
NQRVMNFCAAANLNVKQYLPHYETQKEWKSHFGKRWETFAQRKQAYDPLAILAPGQRIFQ
KTTGKLSPIQLAKSKATGSPQRYHYASILPKPRTV
>Q00326
MKLLHGLALVFLLAAASCKADEEITCEENNPFTCSNTDILSSKNFGKDFIFGVASSAYQI
EGGRGRGVNVWDGFSHRYPEKAGSDLKNGDTTCESYTRWQKDVDVMGELNATGYRFSFAW
SRIIPKGKVSRGVNQGGLDYYHKLIDALLEKNITPFVTLFHWDLPQTLQDEYEGFLDRQI
IQDFKDYADLCFKEFGGKVKHWITINQLYTVPTRGYAIGTDAPGRCSPMVDTKHRCYGGN
SSTEPYIVAHNQLLAHATVVDLYRTKYKFQKGKIGPVMITRWFLPFDESDPASIEAAERM
NQFFHGWYMEPLTKGRYPDIMRQIVGSRLPNFTEEEAELVAGSYDFLGLNYYVTQYAQPK
PNPYPSETHTAMMDAGVKLTYDNSRGEFLGPLFVEDKVNGNSYYYPKGIYYVMDYFKTKY
GDPLIYVTENGFSTPSSENREQAIADYKRIDYLCSHLCFLRKVIKEKGVNVRGYFAWALG
DNYEFCKGFTVRFGLSYVNWEDLDDRNLKESGKWYQRFINGTVKNAVKQDFLRSSLSSQS
QKKRFADA
>P18417
MANFSESKSMMAVFFMFFLLLLSSSSSSSSSPILKKIFIESPSYAPNAFTFDSTDKGFY
TSVQDGRVIKYEGPNSGFTDFAYASPFWNKAFCENSTDPEKRPLCGRTYDISYDYKNSQM
YIVDGHYHLCVVGKEGGYATQLATSVQGVPFKWLYAVTVDQRTGIVYFTDVSSIHDDSPE
GVEEIMNTSDRTGRLMKYDPSTKETTLLLKELHVPGGAEISADGSFVVVAEFLSNRIVKY
WLEGPKKGSAEFLVTIPNPGNIKRNSDGHFWVSSSEELDGGQHGRVVSRGIKFDGFGNIL
QVIPLPPPYEGEHFEQIQEHDGLLYIGSLFHSSVGILVYDDHDNKGNSYVSS
>P06673
MKMVIVLVVWLALSAASASAMQMPCPCAGLQGLYGAGAGLTTMMGAGGLYPYAEYLRQPQ
CSPLAAAPYYAGCGQTSAMYQPLRQQCCQQQMRMMDVQSVAQQLQMMMQLERAATASSSL
YEPALMQQQQQLLAAQGLNPMAMMMAQNMPAMGGLYQYQYQLPSYRTNPCGVSAAIPPYY

>P15478
MATTNSFTILIFMILATTSSTFATLGEMVTVLSIDGGGIKGIIPATILEFLEGQLQEVDN
NTDARLADYFDVIGGTSTGGLLTAMITTPNETNRPFAAAKDIVPFYFEHGPKIFQSSGSI
FGPKYDGKYLMQVLQEKLGETRVHQALTEVAISSFDIKTNKPVIFTKSNLAKSPELDAKM

YDICYSTAAAPTFFPPHYFATNTSNGDKYEFNLVDGAVATVDDPALLSISVATKLAQVDP
KFASIKSLNYKQMLLLSLGTGTTSEFDKTYTAEETAKWGTARWMLVIQKMTSAASSYMTD
YYLSTAFQALDSQNNYLRVQENALTGTTTELDDASEANMQLLVQVGEDLLKKSVSKDNPE
TYEEALKRFAKLLSDRKKLRANKASY
>Q8RUW5
MSLKIKFLLLLVLYHHVDSASIVKFLPGFEGPLPFELETGYIGIGEDENVQFFYYFIKSE
NNPKEDPLLIWLNGGPGCSCLGGIIFENGPVGLKFEVFNGSAPSLFSTTYSWTKMANIIF
LDQPVGSGFSYSKTPIDKTGDISEVKRTHEFLQKWLSRHPQYFSNPLYVVGDSYSGMIVP
ALVQEISQGNYICCEPPINLQGYMLGNPVTYMDFEQNFRIPYAYGMGLISDEIYEPMKRI
CNGNYYNVDPSNTQCLKLTEEYHKCTAKINIHHILTPDCDVTNVTSPDCYYYPYHLIECW
ANDESVREALHIEKGSKGKWARCNRTIPYNHDIVSSIPYHMNNSISGYRSLIYSGDHDIA
VPFLATQAWIRSLNYSPIHNWRPWMINNQIAGYTRAYSNKMTFATIKGGGHTAEYRPNET
FIMFQRWISGQPL
>O04057
MASYHSKAAFLCLFLLVSFNIVSSASNDGLLRVGLKKIKLDPENRLAARVESKDAEILKA
AFRKYNPKGNLGESSDTDIVALKNYLDAQYYGEIAIGTPPQKFTVIFDTGSSNLWVLCEC
LFSVACHFHARYKSSRSSSYKKNGTSASIRYGTGAVSGFFSYDNVKVGDLVVKEQVFIEA
TREPSLTFLVAKFDGLLGLGFQEIAVGNAVPVWYNMVEQGLVKEPVFSFWLNRNVEEEEG
GEIVFGGVDPKHYRGKHTYVPVTQKGYWQFDMGDVLIDGEPTGFCDGGCSAIADSGTSLL
AGPTPVITMINHAIGAKGVVSQQCKAVVAQYGQTIMDLLLSEADPKKICSQINLCTFDGT
RGVSMGIESVVDENAGKSSDSLHDGMCSVCEMTVVWMQNQLRQNQTKERIINYINELCDR
MPSPMGQSAVDCGQLSSMPTVSFTIGGKIFDLAPEEYILKVGEGPVAQCISGFTAFDIPP
PRGPLWILGDVFMGRYHTVFDFGKLRVGSAEAA
>Q9SYM4
MPGNKYNCSSSHIPLSRTERLLRDRELREKRKSNRARNPNDVAGSSENSENDLRLEGDSS
RQYVEQYLEGAAAAMAHDDACERQEVRPYNRQRLLVVANRLPVSAVRRGEDSWSLEISAG
GLVSALLGVKEFEARWIGWAGVNVPDEVGQKALSKALAEKRCIPVFLDEEIVHQYYNGYC
NNILWPLFHYLGLPQEDRLATTRSFQSQFAAYKKANQMFADVVNEHYEEGDVVWCHDYHL
MFLPKCLKEYNSKMKVGWFLHTPFPSSEIHRTLPSRSELLRSVLAADLVGFHTYDYARHF
VSACTRILGLEGTPEGVEDQGRLTRVAAFPIGIDSDRFIRALEVPEVIQHMKELKERFAG
RKVMLGVDRLDMIKGIPQKILAFEKFLEENANWRDKVVLLQIAVPTRTDVPEYQKLTSQV
HEIVGRINGRFGTLTAVPIHHLDRSLDFHALCALYAVTDVALVTSLRDGMNLVSYEFVAC
QEAKKGVLILSEFAGAAQSLGAGAILVNPWNITEVAASIGQALNMTAEEREKRHRHNFHH
VKTHTAQEWAETFVSELNDTVIEAQLRISKVPPELPQHDAIQRYSKSNNRLLILGFNATL
TEPVDNQGRRGDQIKEMDLNLHPELKGPLKALCSDPSTTIVVLSGSSRSVLDKNFGEYDM
WLAAENGMFLRLTNGEWMTTMPEHLNMEWVDSVKHVFKYFTERTPRSHFETRDTSLIWNY
KYADIEFGRLQARDLLQHLWTGPISNASVDVVQGSRSVEVRAVGVTKGAAIDRILGEIVH
SKSMTTPIDYVLCIGHFLGKDEDVYTFFEPELPSDMPAIARSRPSSDSGAKSSSGDRRPP
SKSTHNNNKSGSKSSSSSNSNNNNKSSQRSLQSERKSGSNHSLGNSRRPSPEKISWNVLD
LKGENYFSCAVGRTRTNARYLLGSPDDVVCFLEKLADTTSSP
>O22218
MSNLLRDFEVEAKNPSLEARQRWRSSVSIVKNRTRRFRNIRDLDKLADYENKKHQIQEKI
RVAFFVQKAALHFIDAAARPEYKLTDEVKKAGFSIEADELASMVRKNDTKSLAQKGGVEE
LAKKVSVSLSEGIRSSEVPIREKIFGENRYTEKPARSFLMFVWEALHDITLIILMVCAVV

SIGVGVATEGFPRGMYDGTGILLSILLVVMVTAISDYKQSLQFRDLDREKKKIIVQVTRD
GSRQEISIHDLVVGDVVHLSIGDQVPADGIFISGYNLEIDESSLSGESEPSHVNKEKPFL
LSGTKVQNGSAKMLVTTVGMRTEWGKLMETLVDGGEDETPLQVKLNGVATIIGKIGLSFA
VLTFVVLCIRFVLDKATSGSFTNWSSEDALTLLDYFAISVTIIVVAVPEGLPLAVTLSLA
FAMKKLMSDRALVRHLAACETMGSSTCICTDKTGTLTTNHMVVNKVWICDKVQERQEGSK
ESFELELSEEVQSTLLQGIFQNTGSEVVKDKDGNTQILGSPTERAILEFGLLLGGDFNTQ
RKEHKILKIEPFNSDKKKMSVLIALPGGGARAFCKGASEIVLKMCENVVDSNGESVPLTE
ERITSISDIIEGFASEALRTLCLVYKDLDEAPSGELPDGGYTMVAVVGIKDPVRPGVREA
VQTCQAAGITVRMVTGDNISTAKAIAKECGIYTEGGLAIEGSEFRDLSPHEMRAIIPKIQ
VMARSLPLDKHTLVSNLRKIGEVVAVTGDGTNDAPALHEADIGLAMGIAGTEVAKENADV
IIMDDNFKTIVNVARWGRAVYINIQKFVQFQLTVNVVALIINFVSACITGSAPLTAVQLL
WVNMIMDTLGALALATEPPNEGLMKRAPIARTASFITKTMWRNIAGQSVYQLIVLGILNF
AGKSLLKLDGPDSTAVLNTVIFNSFVFCQVFNEINSREIEKINVFKGMFNSWVFTWVMTV
TVVFQVIIVEFLGAFASTVPLSWQHWLLSILIGSLNMIVAVILKCVPVESRHHHDGYDLL
PSGPSSSNSA
>P52706
MEKSTMSAILLVLHLFVLLLQYSEVHSLATTSNHDFSYLRFAYDATDLELEGSYDYVIVG
GGTSGCPLAATLSEKYKVLVLERGSLPTAYPNVLTADGFVYNLQQEDDGKTPVERFVSED
GIDNVRGRVLGGTSMINAGVYARANTSIYSASGVDWDMDLVNKTYEWVEDTIVFKPNYQP
WQSVTGTAFLEAGVDPNHGFSLDHEAGTRITGSTFDNKGTRHAADELLNKGNSNNLRVGV
HASVEKIIFSNAPGLTATGVIYRDSNGTPHRAFVRSKGEVIVSAGTIGTPQLLLLSGVGP
ESYLSSLNIPVVLSHPYVGQFLHDNPRNFINILPPNPIEPTIVTVLGISNDFYQCSFSSL
PFTTPPFSFFPSTSYPLPNSTFAHFASKVAGPLSYGSLTLKSSSNVRVSPNVKFNYYSNP
TDLSHCVSGMKKIGELLSTDALKPYKVEDLPGIEGFNILGIPLPKDQTDDAAFETFCRES
VASYWHYHGGCLVGKVLDGDFRVTGIDALRVVDGSTFPYTPASHPQGFYLMLGRYVGIKI
LQERSASDLKILDSLKSAASLVL
>Q9M3D6
MNLSLSGRKIAMTRVSAETQYITPIGSPTLDELLKDCDSFRKGDSGDGVKSDDPAHHIID
VEALYVKPVPYVLNFNNLQYDVTLRRRFGFSRQNGVKTLLDDVSGEASDGDILAVLGASG
AGKSTLIDALAGRVAEGSLRGSVTLNGEKVLQSRLLKVISAYVMQDDLLFPMLTVKETLM
FASEFRLPRSLSKSKKMERVEALIDQLGLRNAANTVIGDEGHRGVSGGERRRVSIGIDII
HDPIVLFLDEPTSGLDSTNAFMVVQVLKRIAQSGSIVIMSIHQPSARIVELLDRLIILSR
GKSVFNGSPASLPGFFSDFGRPIPEKENISEFALDLVRELEGSNEGTKALVDFNEKWQQN
KISLIQSAPQTNKLDQDRSLSLKEAINASVSRGKLVSGSSRSNPTSMETVSSYANPSLFE
TFILAKRYMKNWIRMPELVGTRIATVMVTGCLLATVYWKLDHTPRGAQERLTLFAFVVPT
MFYCCLDNVPVFIQERYIFLRETTHNAYRTSSYVISHSLVSLPQLLAPSLVFSAITFWTV
GLSGGLEGFVFYCLLIYASFWSGSSVVTFISGVVPNIMLCYMVSITYLAYCLLLSGFYVN
RDRIPFYWTWFHYISILKYPYEAVLINEFDDPSRCFVRGVQVFDSTLLGGVSDSGKVKLL
ETLSKSLRTKITESTCLRTGSDLLAQQGITQLSKWDCLWITFASGLFFRILFYFALLFGS
RNKRT
>Q8VYK7
MAKSSFKQEHDLEKRRAEAARIREKYPDRIPVIVEKAEKSDIPTIDKKKYLVPADLTVGQ
FVYVIRKRIKLSAEKAIFIFVDNVLPPAGALMSSVYEEKKDDDGFLYVTYSGENTFGFGS
P

>P22895
MGFLVLLLFSLLGLSSSSSISTHRSILDLDLTKFTTQKQVSSLFQLWKSEHGRVYHNHEE
EAKRLEIFKNNSNYIRDMNANRKSPHSHRLGLNKFADITPQEFSKKYLQAPKDVSQQIKM
ANKKMKKEQYSCDHPPASWDWRKKGVITQVKYQGGCGRGWAFSATGAIEAAHAIATGDLV
SLSEQELVDCVEESEGSYNGWQYQSFEWVLEHGGIATDDDYPYRAKEGRCKANKIQDKVT
IDGYETLIMSDESTESETEQAFLSAILEQPISVSIDAKDFHLYTGGIYDGENCTSPYGIN
HFVLLVGYGSADGVDYWIAKNSWGFDWGEDGYIWIQRNTGNLLGVCGMNYFASYPTKEES
ETLVSARVKGHRRVDHSPL
>Q39962
MKMKRSPYCFCCSFALLLLVSFLKDRHFCSADPTDGFTEVPLTEDNFVIQKPYDKPLNDR
YSYKNGIRRLWVYENDKPFKVGSPTRPRTEIRIKGHDYSSGVWQFEGQVHVPEGTSGVTV
MQVFGAVNKATALQLRVYNGDLKSYKSNSVATDIYNKWLRVNVIHKVGKGEITVFINGQQ
KLVVNDDGPAEHYFKCGVYAAPDGSSNYMESRWKNIKLYKSDNKLEGCNNNHGTWLVQ
>Q8H1Z0
MVAFLSAWPWENFGNLKYLLYAPLAAQVVYSWVYEEDISKVLWCIHILIICGLKALVHEL
WSVFNNMLFVTRTLRINPKGIDFKQIDHEWHWDNYIILQAIIVSLICYMSPPLMMMINSL
PLWNTKGLIALIVLHVTFSEPLYYFLHRSFHRNNYFFTHYHSFHHSSPVPHPMTAGNATL
LENIILCVVAGVPLIGCCLFGVGSLSAIYGYAVMFDFMRCLGHCNVEIFSHKLFEILPVL
RYLIYTPTYHSLHHQEMGTNFCLFMPLFDVLGDTQNPNSWELQKKIRLSAGERKRVPEFV
FLAHGVDVMSAMHAPFVFRSFASMPYTTRIFLLPMWPFTFCVMLGMWAWSKTFLFSFYTL
RNNLCQTWGVPRFGFQYFLPFATKGINDQIEAAILRADKIGVKVISLAALNKNEALNGGG
TLFVNKHPDLRVRVVHGNTLTAAVILYEIPKDVNEVFLTGATSKLGRAIALYLCRRGVRV
LMLTLSMERFQKIQKEAPVEFQNNLVQVTKYNAAQHCKTWIVGKWLTPREQSWAPAGTHF
HQFVVPPILKFRRNCTYGDLAAMKLPKDVEGLGTCEYTMERGVVHACHAGGVVHMLEGWK
HHEVGAIDVDRIDLVWEAAMKYGLSAVSSLTN
>Q39119
MATTMTRVSVGVVLFVLLVSLVAVSAARSGPDDVIKLPSQASRFFRPAENDDDSNSGTRW
AVLVAGSSGYWNYRHQADICHAYQLLRKGGLKEENIVVFMYDDIANNYENPRPGTIINSP
HGKDVYQGVPKDYTGDDVNVDNLFAVILGDKTAVKGGSGKVVDSGPNDHIFIFYSDHGGP
GVLGMPTSPYLYANDLNDVLKKKHALGTYKSLVFYLEACESGSIFEGLLPEGLNIYATTA
SNAEESSWGTYCPGEEPSPPPEYETCLGDLYSVAWMEDSGMHNLQTETLHQQYELVKRRT
APVGYSYGSHVMQYGDVGISKDNLDLYMGTNPANDNFTFADANSLKPPSRVTNQRDADLV
HFWEKYRKAPEGSARKTEAQKQVLEAMSHRLHIDNSVILVGKILFGISRGPEVLNKVRSA
GQPLVDDWNCLKNQVRAFERHCGSLSQYGIKHMRSFANICNAGIQMEQMEEAASQACTTL
PTGPWSSLNRGFSA
>Q9SCB9
MATRYWIAALPVADDNVAAGKTALWARLQEAISRHSFDTPLYRFTVPDLRPGTLDSLLAL
SDDLVKSNIFIEGVSHKIRRQIEDLERAGGVEPGTLTVDGVPVDSYLTRFVWDEGKYPVN
APLKETVASIQSQVAKIEDDMKVRVAEYGNVKSQLGAINRKQTGSLAVRDLSNLIKPEDM
VTSEHLVTLLSIVPKYSQKDWLASYESLDTFVVPRSSKKLYEDNEYALYTVTLFAKVVDN
FKVHAREKGFQIRDFEYSPEAQESRKQELEKLLQDQEVMRTSPIAMGAMLATVRVFSSWD
AFSSAVRVFVESILRYGSACTVPVCCPSTIYKEREKSKEHLGRAMRQYQQLLEI
>P26587
MATSARRAYGFGRADEATHPDSIRATLAEFLSTFVFVFAAEGSILSLDKLYWEHAAHAGT

NTPGGLILVALAHAFALFAAVSAAINVSGGHVNPAVTFGALVGGRVTAIRAIYYWIAQLL
GAILACLLLRLTTNGMRPVGFRLASGVGAVNGLVLEIILTFGLVYVVYSTLIDPKRGSLG
IIAPLAIGLIVGANILVGGPFSGASMNPARAFGPALVGWRWHDHWIYWVGPFIGSALAAL
IYEYMVIPTEPPTHHAHGVHQPLAPEDY
>P11827
MMRARFPLLLLGVVFLASVSVSFGIAYWEKQNPSHNKCLRSCNSEKDSYRNQACHARCNL
LKVEEEEECEEGQIPRPRPQHPERERQQHGEKEEDEGEQPRPFPFPRPRQPHQEEEHEQK
EEHEWHRKEEKHGGKGSEEEQDEREHPRPHQPHQKEEEKHEWQHKQEKHQGKESEEEEED
QDEDEEQDKESQESEGSESQREPRRHKNKNPFHFNSKRFQTLFKNQYGHVRVLQRFNKRS
QQLQNLRDYRILEFNSKPNTLLLPHHADADYLIVILNGTAILTLVNNDDRDSYNLQSGDA
LRVPAGTTFYVVNPDNDENLRMIAGTTFYVVNPDNDENLRMITLAIPVNKPGRFESFFLS
STQAQQSYLQGFSKNILEASYDTKFEEINKVLFGREEGQQQGEERLQESVIVEISKKQIR
ELSKHAKSSSRKTISSEDKPFNLGSRDPIYSNKLGKLFEITQRNPQLRDLDVFLSVVDMN
EGALFLPHFNSKAIVVLVINEGEANIELVGIKEQQQRQQQEEQPLEVRKYRAELSEQDIF
VIPAGYPVMVNATSDLNFFAFGINAENNQRNFLAGSKDNVISQIPSQVQELAFPRSAKDI
ENLIKSQSESYFVDAQPQQKEEGNKGRKGPLSSILRAFY
>Q9FRK5
MADSQNGKSAFFFFFVSLILLFLSPSYSDVTASESDPIPYENSDASPGVVTSSESDRQGV
SLHRLEELVRNLTELVARLDAKLSETPFKVKKEITRDEIEEKAKAFSVTKYSPFWSERFE
FTSAVKLDSEATCINVLPFRDHEGLSKYFAVGDSSGRVFVFLRNGDVLVEFFTTCDSPIT
AMVSYMSVYKNESFVVTGHQSGVILLHRLREGSIGEDLNSAVMENVGKFDGTEDGLQVTL
LEVHHVGRVRYILATDLSGKLTVFTENRTVYGSVSPTSRPLVFLKQRLLFLTETGAGSLD
LRSMKIRESECEGLNHSLARSYVFDASERTKAYGFTSEGEIIHVLLLGDIMNFKCRVRSK
KKVQMEEPVALQAIKGYLLIVNQEKVFVYNVSTQHYVRTTGPRLLFPAALEDIRSTFLSH
RESTKTTDHQKLEKVTPLIASDREKLLVMGLGDGYVATYKSKLPISKAEFNTMLWSSPVF
FFILFLFGAWHFFSKKKESLTAWGPDDPFSSTTMSSSSTTTAQNSSAFSESTRRNDDHMD
LRRRYVSPSRYPPGAATGAYRSVGSNDPSSRAPVETTNYRTTAQEMKYRGGSGLDSGGFG
KRRESLFGNNKALDDES
>Q8LBL1
MSSDAARTPLLPTEKIDTMAQDFNLNSRTSSSRKRRLRRSRSAPRGDCMYNDDVKIDEPP
PHPSKIPMFSDLNPNLRRVIMFLALYLTIGTLCFYLVRDQISGHKTSGVVDALYFCIVTM
TTVGYGDLVPNSSASRLLACAFVFSGMVLVGHLLSRAADYLVEKQEALLVRAFHLRQSFG
PTDILKELHTNKLRYKCYATCLVLVVLFIVGTIFLVMVEKMPVISAFYCVCSTVTTLGYG
DKSFNSEAGRLFAVFWILTSSICLAQFFLYVAELNTENKQRALVKWVLTRRITNNDLEAA
DLDEDGVVGAAEFIVYKLKEMGKIDEKDISGIMDEFEQLDYDESGTLTTSDIVLAQTTSQ
IQR
>Q8GTM0
MARSLCFMAFAILAMMLFVAYEVQARECKTESNTFPGICITKPPCRKACISEKFTDGHCS
KILRRCLCTKPCVFDEKMTKTGAEILAEEAKTLAAALLEEEIMDN
>P93654
MSFQDLESGRGRSTRKFNGGRQDSTQAVASGIFQINTGVSTFQRLVNTLGTPKDTPELRE
KLHKTRLHIGQLVKDTSAKLKEASETDHQSGVNPSKKIADAKLARDFQAVLKEFQKAQQT
AAERETTYTPFVPQSALPSSYTAGEVDKVPEQRAQLQESKRQELVLLDNEIAFNEAVIEE
REQGIQEIHQQIGEVNEIFKDLAVLVNDQGVMIDDIGTHIDNSRAATSQGKSQLVQAAKT

QKSNSSLTCLLLVIFGIVLLIVIIVLAA
>Q9ZT63
MESSSPHHSHIVEVNVGKSDEERIIVASKVCGEAPCGFSDSKNASGDAHERSASMRKLCI
AVVLCLVFMSVEVVGGIKANSLAILTDAAHLLSDVAAFAISLFSLWAAGWEATPRQTYGF
FRIEILGALVSIQLIWLLTGILVYEAIIRIVTETSEVNGFLMFLVAAFGLVVNIIMAVLL
GHDHGHSHGHGHGHGHDHHNHSHGVTVTTHHHHHDHEHGHSHGHGEDKHHAHGDVTEQLL
DKSKTQVAAKEKRKRNINLQGAYLHVLGDSIQSVGVMIGGAIIWYNPEWKIVDLICTLAF
SVIVLGTTINMIRNILEVLMESTPREIDATKLEKGLLEMEEVVAVHELHIWAITVGKVLL
ACHVNIRPEADADMVLNKVIDYIRREYNISHVTIQIER
>P19171
MPPQKENHRTLNKMKTNLFLFLIFSLLLSLSSAEQCGRQAGGALCPNGLCCSEFGWCGNT
EPYCKQPGCQSQCTPGGTPPGPTGDLSGIISSSQFDDMLKHRNDAACPARGFYTYNAFIT
AAKSFPGFGTTGDTATRKKEVAAFFGQTSHETTGGWATAPDGPYSWGYCFKQEQNPASDY
CEPSATWPCASGKRYYGRGPMQLSWNYNYGLCGRAIGVDLLNNPDLVANDAVIAFKAAIW
FWMTAQPPKPSCHAVIAGQWQPSDADRAAGRLPGYGVITNIINGGLECGRGQDGRVADRI
GFYQRYCNIFGVNPGGNLDCYNQRSFVNGLLEAAI
>P58908
ADTIVAVELDSYPNTDIGDPNYPHIGIDIKSIRSKSTARWNMQTGKVGTVHISYNSVAKR
LSAVVSYTGSSSTTVSYDVDLNNVLPEWVRVGLSATTGLYKETNTILSWSFTSKLKTNSI
ADANSLHFTFNQFSQNPKDLILQGDATTDSDGNLELTKVSSSGDPQGNSVGRALFYAPVH
IWEKSAVVASFDATFTFLIKSPDRDPADGITFFIANPDTSIPSGSGGRLLGLFPDAN
>Q39649
MARLTSIIALFAVALLVADAYAYRTTITTVEVEENRQGREERCRQMSAREELRSCEQYLR
QQSRDVLQMRGIENPWRREGGSFDECCRELKNVDEECRCDMLEEIAREEQRQARGQEGRQ
MLQKARNLPSMCGIRPQRCDF
>Q8LG88
MNGGDVTVAGSDDLKSPLLPVVHNDEPFERQTVGQQLRTIFTPKNCYIALGPLLCAVVCL
CVDLGGDETTTARNMLGVLVWMFAWWLTEAVPMPITSMTPLFLFPLFGISAADDVANSYM
DDVISLVLGSFILALAVEHYNIHRRLALNITLVFCVEPLNAPLLLLGICATTAFVSMWMH
NVAAAVMMMPVATGILQRLPSSSSTTEVVHPAVGKFSRAVVLGVIYSAAVGGMSTLTGTG
VNLILVGMWKSYFPEADPISFSQWFFFGFPLALCIFVVLWCVLCVMYCPKGAGQALSPYL
HKSHLRRELDLLGPMNFAEKMVLAVFGGLVVLWMTRNITDDIPGWGRIFAGRAGDGTVSV
MMATLLFIIPSNIKKGEKLMDWNKCKKLPWNIVLLLGAGFAIADGVRTSGLAEVLSKGLV
FLETAPYWAIAPTVCLIAATITEFTSNNATTTLLVPLLIEIAKNMGIHPLLLMVPGAIGA
QFAFLLPTGTPSNVVGFTTGHIEIKDMIKTGLPLKIAGTIFLSILMPTLGAYVFASMGGV

>Q67VS5
MKSPSPVDPESPSSPDCKGGSSSKRRRLPWRMTMSLAYQSLGVVYGDLSTSPLYVYKAAF
AEDIQHSETNEEILGVLSFVFWTLTLVPLLKYVCVVLRADDNGEGGTFALYSLLCRHARA
ALLPPGGGGGGGEPGDEDQFLDAGADKKAAANGNALALSGRGGGGGAAAGVRRLLERHKV
LQRVLLVLALVGTCMVIGDGVLTPAISVFSAVSGLELSMEKHQHKYVEVPIACFVLVCLF
CLQHYGTHRVGFLFAPIVITWLLCISMIGVYNIVHWEPNVYRALSPYYMYKFLKKTQRGG
WMSLGGILLCITGSEAMFADLGHFNQLSIQIAFTCMVYPSLILAYMGQAAYLCKHHIIES
DYRIGFYVSVPEKIRWPVLAIAILAAVVGSQAVITGTFSMIKQCTALGCFPRVKIVHTSD

KVHGQIYIPEINWILMILCLAITIGFRDTKHLGNASGLAVITVMLVTTCLMSLVIVLCWH
KSIFLAFGFIIFFGTIEALYFSASLIKFREGAWVPIVLAFIFMAIMCIWHYGTIKKYEFD
LQNKVSINWLLGLSPNLGIVRVRGIGLIHTELDSGIPAIFSHFVTNLPAFHQVLIFLCIK
NVPIPHVSPEERFLVGRIGPKEYRIYRCIVRYGYHDVHKDDQEFEKELVCSVAEFIRSGA
AAAADAAASSKPKNVCGGGAEESEKEEEERMSVIPSGSIRMMEEDGGAGAPSSEDTVGGS
GSGSGRGSSRGGGGAREIMSPSPSPPPVVVAPRKRVRFVLPAASPRPDAGVREELQELMD
AREAGMAFILGHSYVKAKSGSSFFRRLVINFCYDFLRRNSRGPNYAVTIPHASTLEVGMI
YYV

>Q38882

MAQHLLHGTLHATIYEVDALHGGGVRQGFLGKILANVEETIGVGKGETQLYATIDLQKAR
VGRTRKIKNEPKNPKWYESFHIYCAHLASDIIFTVKDDNPIGATLIGRAYIPVDQVINGE
EVDQWVEILDNDRNPIQGGSKIHVKLQYFHVEEDRNWNMGIKSAKFPGVPYTFFSQRQGC
KVSLYQDAHIPDNFVPRIPLAGGKNYEPQRCWEDIFDAISNAKHLIYITGWSVYAEIALV
RDSRRPKPGGDVTIGELLKKKASEGVRVLLLVWDDRTSVDVLKKDGLMATHDEETENFFR
GSDVHCILCPRNPDDGGSIVQSLQISTMFTHHQKIVVVDSEMPSRGGSEMRRIVSFVGGI
DLCDGRYDTPFHSLFRTLDTVHHDDFHQPNFTGAAITKGGPREPWHDIHSRLEGPIAWDV
MYNFEQRWSKQGGKDILVKLRDLSDIIITPSPVMFQEDHDVWNVQLFRSIDGGAAAGFPE
SPEAAAEAGLVSGKDNIIDRSIQDAYIHAIRRAKDFIYVENQYFLGSSFAWAADGITPED
INALHLIPKELSLKIVSKIEKGEKFRVYVVVPMWPEGLPESGSVQAILDWQRRTMEMMYK
DVIQALRAQGLEEDPRNYLTFFCLGNREVKKDGEYEPAEKPDPDTDYMRAQEARRFMIYV
HTKMMIVDDEYIIIGSANINQRSMDGARDSEIAMGGYQPHHLSHRQPARGQIHGFRMSLW
YEHLGMLDETFLDPSSLECIEKVNRISDKYWDFYSSESLEHDLPGHLLRYPIGVASEGDI
TELPGFEFFPDTKARILGTKSDYLPPILTT

>P31414

MVAPALLPELWTEILVPICAVIGIAFSLFQWYVVSRVKLTSDLGASSSGGANNGKNGYGD
YLIEEEEGVNDQSVVAKCAEIQTAISEGATSFLFTEYKYVGVFMIFFAAVIFVFLGSVEG
FSTDNKPCTYDTTRTCKPALATAAFSTIAFVLGAVTSVLSGFLGMKIATYANARTTLEAR
KGVGKAFIVAFRSGAVMGFLLAASGLLVLYITINVFKIYYGDDWEGLFEAITGYGLGGSS
MALFGRVGGGIYTKAADVGADLVGKIERNIPEDDPRNPAVIADNVGDNVGDIAGMGSDLF
GSYAEASCAALVVASISSFGINHDFTAMCYPLLISSMGILVCLITTLFATDFFEIKLVKE
IEPALKNQLIISTVIMTVGIAIVSWVGLPTSFTIFNFGTQKVVKNWQLFLCVCVGLWAGL
IIGFVTEYYTSNAYSPVQDVADSCRTGAATNVIFGLALGYKSVIIPIFAIAISIFVSFSF
AAMYGVAVAALGMLSTIATGLAIDAYGPISDNAGGIAEMAGMSHRIRERTDALDAAGNTT
AAIGKGFAIGSAALVSLALFGAFVSRAGIHTVDVLTPKVIIGLLVGAMLPYWFSAMTMKS
VGSAALKMVEEVRRQFNTIPGLMEGTAKPDYATCVKISTDASIKEMIPPGCLVMLTPLIV
GFFFGVETLSGVLAGSLVSGVQIAISASNTGGAWDNAKKYIEAGVSEHAKSLGPKGSEPH
KAAVIGDTIGDPLKDTSGPSLNILIKLMAVESLVFAPFFATHGGILFKYF
