# Supplementary material for: Plant-mPLoc: A Top-Down Strategy to Augment the Power for Predicting Plant Protein Subcellular Localization
Source: PLoS One. 2010 Jun 28;5(6):e11335. doi: 10.1371/journal.pone.0011335 (PMC2893129; doi:10.1371/journal.pone.0011335)
Supplement: Table S2 — The degenerate testing dataset used for comparing the performance between TargetP (Emanuelsson, et al. J. of Mol. Biol. 2000, 300: 1005–1016) and Plant-mPLoc of this paper. The dataset contains 1,775 plant proteins classified into 2 subcellular locations: (1) chloroplast, and (2) mitochondrion. To avoid bias, none of the proteins included here occurs in the training dataset of TargetP, nor in the training dataset of Plant-mPLoc. See the text of the paper for further explanation. (0.91 MB PDF) [file pone.0011335.s002.pdf]

Table S2. The degenerate testing dataset used for comparing the performance between TargetP (Emanuelsson, et al. J. of Mol. Biol. 2000, 300: 1005-1016) and Plant-mPLoc of this paper. The dataset contains 1,775 plant proteins classified into 2 subcellular locations: (1) chloroplast, and (2) mitochondrion. To avoid bias, none of the proteins included here occurs in the training dataset of TargetP, nor in the training dataset of Plant-mPLoc. See the text of the paper for further explanation.

---

(1) 1500 chloroplast proteins

```
>A0A325 | RPOC2_COFAR
MEVLMAERANLVFHNKSIDGTAMKRLISRLIDHFGMAYTSHILDQVKTLGFKQATATSIS
LGIDDLLTIPSKGWLVQDAEQQSLILEKHHHYGNVHAVEKLRQSIEIWYATSEYLRQEMN
PNFRMTDPFNPVHIMSFSGARGNVSQVHQLVGMRGLMSDPQGQMIDLPIQSNLREGLSLT
EYIISCYGARKGVVDTAVRTSDAGYLTRRLVEVVQHIVVRRTDCGTVRGISVSPPNGMMP
EKFFIQTLVGRVLADDIYMGPRCIATRNQDIGIELVNRFITFRAQPISIRTPFTCRSTSW
ICRLCYGRSPTHGDLVELGEAVGIIAGQSIGEPGTQLTLRTFHTGGVFAGGIAEHVRAPS
NGKIKFNEDLVHPTRTRHGHPAFLCFIDLYVTIKSENILHNVNIPPKSFLLVQNDQYVEA
EQVIAEIRAGTSTLSFKEKVRKHIYSDSDGEIHWSTDVYHAPQFTYGNVHLLPKTSHLWI
LLGGPCRSSLVSLSLHKDQDQMNTHSLSVKRRYTYKLSVTNDRVRYKFFSSDFYGKKKNR
IPDYLDLNRIICTGYCNRIYPAILHENSDLLSKRRRNRFIMPLQSIQERENESPGISIEI
PINGIFRRNSILSYFDDPRYRRKSSGITKYGTIEMHSLVKKEDLIEYRGVKEFRPKYQMK
VDRFFFIPEEVHILPGSSSIMVQNNSIIGIDTQITLNIRSRVGGLVRVERKKKGIELKIF
SGDIHFPGETDKISRHSGVLIPLPPGTGKINSKESKKLKNWIYVQRITPSKKGYFALVRP
VITYEKRDGLNLATLFPPDLLQERDNVRLQVVNYILYGNGKPIRGISDTSIQLVRTCLVL
NWDQDKKSSSSEEAPASFVEIRTNGLIRHFLRIDLVKSPISYIVKRNDPSGSGLLSDNGS
DCTNINPFSSIYSYSKARIQQFLNPNQGTIHTLLNKNREFQSLIILLSSNCSRMGPFAGV
KYHNVIKESIKNKKDPRISIRNSLGPLGSAPPIANFFSFSHLLTHNQILVTNYLQLDNIK
ETFQVIKYYSMDENGKIYNPDPCNNIILNPLNLNWYFRHHNYCEETSTIISLGQFICENV
CIAKNRPHLKPGQVIFVQVDSVVIRSAKPYLATPGATVHGHYGETLYEGDILVTFIYEKS
RSGDITQGLPKVEQILEVRSIDSISMNLQKRIEGWNKCITRILGTPWGFLISAELTIVQS
QISLVNKIQKVYRSQGVQIHNKHIEIIVRQITSKVLISEDGMSNVFSPGELIGLLRAERM
GRALEEAICYRAVLLGITRASLNTQSFISEASFQETARVLAKAALRGRIDWLKGLKENVV
LGGMIPVGTGFKGLVKPSKQHNKALLETKKKNLFEGEMRDILFHHRNFFDSFLLKKFHDP
SEQSFIGFNDS
>A0A327 | RPOB_COFAR
MLGDGNEGMATIPGFNQIQFEGFCRFIDQGLAEELYKFPKIEDTDQEIEFQLFVETYQLV
EPLIKERDAVYELLTYSSELYVSAGLIWKTGRDMQEQTIFIGNIPLMNSLGTFIVNGVYR
ILINQILQSPGIYYRSELDHNGISVYTGTIISDWGGRSELEIDRKARIWARVSRKQKISI
LVLSSAMGSNLREILENVCYPEIFLSFLNDKERKKIGSKENAILEFYQQFACVGGDPVFS
ESLCKELQKKFFQQRCELGRIGRRNMNRRLNLDIPQNNPFLLPRDILAAADHLIGLKFGM
GTLDDMNHLKNKRIRSVADLLQDQFGLALVRLENAVRGTICGAIRHKLIPTPQNLVTSTP
LTTTYESFFGLHPLSQVLDGTNPLTQIVHGRKLSYLGPGGLTGRTANFRIRDIHPSHYGR
ICPIDTSEGINVGLIGSLSIHARIGHWGFLESPFYEISERRVLFLLPGRDEYYKVAAGNS
LALNQDIQEKQVVPARYRQEFLTIAWEQVHLRSIFPFQYFSIGASLIPFIEHNDANRALM
```

SSNMQRQAVPLSRSEKCIVGTGLEQQAALDSGALSIAEREGKVLYTDTDKILLSGNGGTL
SIPLVMYQRSNKNTCMHQKPRVQRGKCIKKGQILADAAATVGGELSLGKNVLVAYMPWEG
YNFEDAVLISERLVYEDIYTSFHIRKYEIQTHVTSQGPERVTNEIPHLEAHLLRNLDKSG
IVMLGSWVETGDILVGKLTPQMVKESSYAPEDRLLRAILGIQVSTSKETCLKLPIGGRGR
VIDVRWIQKKGGSSYNPEMIRVYISQKREIKVGDKVAGRHGNKGIISKILTRQDMPYLQD
GRPVDMVFNPLGVPSRMNVGQIFECSLGLAGSLLDRHYRIAPFDERYEQEASRKLVFSEL
YQASKQTANPWVFEAEYPGKSRIFDGRTGNPFEQPVLIGKPYILKLIHQVDDKIHGRSSG
HYALVTQQPLRGRAKQGGQRVGEMEVWALEGFGVAHILQEMLTYKSDHIRARQEVLGTTI
VGGTIPNPEDAPESFRLLVRELRSLALELKHFLISEKNFRIHRKEA
>A0A333 | RR14_COFAR
MARKGLIQREKKRQKLEQKYHLIRRSSKKEISKVQSLNDKWEIYRKLQSPPRNSAPTRLH
RRCFATGRPRANYRDFGLSGHILREMVHACLLPGARRSSW
>A0A337 | RR4_COFAR
MSRYRGPRFKKIRRLGALPGLTNKKPRAGNDLRNQLRSGKKSQYRIRLEEKQKLRFHYGL
TERQLLKYVRIAGKAKGSTGQVLLQLLEMRLDNILFRLGMASTIPAARQLVNHRHILVNG
RIVDIPSYRCKPRDIITGKDEQKSRALIQKSLDSSPQEELPSHLTLHPFQYKGLVNQIID
SKWVGLKINELLVVEYYSRQT
>A0A356 | RK33_COFAR
MAKGKDVRVTVILECTDCVRNSVNKVSTGISRYITQKNRHNTPNRLELKKFCPYCYKHTV
HGEIKK
>A0A357 | RR18_COFAR
MDKSKRSFLKSKRSVRRRLPPIQSGDLIDYRNMSLISRFISEQGKILSRRVNRLTLKQQR
LITVAIKQARILSLLPFLNNEKQFERTESTARTTGLRTRNK
>A0A371 | RR8_COFAR
MGRDTVAEIITSIRNADMDKKRVVRITSTNITENIVKILLREGFIENARKHRENNKYFLV
LTLRHRRNRKGPHRNIFNLKRISRPGLRIYANSQRIPRILGGMGIVIFSTSRGIMTDREA
RLEGIGGEILCYIW
>A0A374 | RR3_COFAR
MGQKINPLGFRLGTTQGHHSLWFTQAKNYSEGLQEDQQIRNCIKNYVQKNTKISSGVEGI
ARIEIQKRIDLIQVIIFMGFPKPLLENQPRGIEELQTNLQKKCNSVNRKFNIAITKIAKP
YGNPNILAEYIAGQLKNRVSFRKAMKKAIELTEQANTKGIQVQIAGRIDGKEIARVEWIR
RGRVPLQTIRAKIDYCSYTVRTIYGVLGIKIWIFIDEE
>A0A375 | RK22_COFAR
MLKKKKTEVYALGQHISMSADKARRVIDQIRGRSYEETLIILELMPYRACYPIFKLVYSA
AANASYNMSSNDANLLISKAEVNEGTTIKKFKPRARGRSYPIKRPTCHITIVMKDISLDD
EYVKINSLKKPRWKNKHTAMVYHDMYSSGGVWDKK
>A0A376 | RR19_COFAR
MTRSLKKNPFVANHLLKKIDKLNTKAEKEIIVTWSRASTIIPTMIGHTIGIHNGKEHLPI
YITDRMVGHKLGEFAPTLNFRGHAKSDNRSRR
>A0A381 | RR7_COFAR
MSRRGAAEEKTAKSDPIYRNRLVNMLVNRILKHGKKSLAYQIIYRAVKKIQQKTETNPLS
VLRQAIRGVTPDITVKARRVGGSTHQVPIEIGSTQGKALAIRWLLAASRKRPGRNMAFKL
SSELVDAAKGSGDAIRKKEETHRMAEANRAFAHFR
>A0A385 | NU4C_COFAR

MNYFPWLTIIVILPIFAGSLIFFLPHRGNRVIRWYTIFICIFELLLTAYTFCYHFQTDDP
LIQLMEDYKWIQLFDFHWRLGIDGLSIGPILLTGFITTLATLAAWPVTRDSRLFNFLMLA
MYSAQIGLFSSRDLLLFFIMWELELIPVYLLLSMWGGKKRLYSATKFILYTAGGSIFLLM
GVLGIGLYGSNEPTLNFETSANQSYPLPLEIIFYIGFFIAFAVKLPIIPLHTWLPDTHGE
AHYSTCMLLAGILLKMGAYGLVRINMELLSHAHSIFSPWLLIVGTIQIIYAASTSLGQRN
LKKRIAYSSVSHMGFIIIGIGSITDTGLNGALLQIISHGFIGAALFFLAGTTYDRIRLVY
LDEMGGIAILMPKIFTIFSTFSMASLALPGMSGFVAELIVFFGIITSQKYLLMPKILITF
VMAIGMILTPIYSLSMSRQMFYGYKFFNIPNSYFFDSGPRELFLSISLFLPVLGIGMYPD
FIFSLSVDKVEVILSNSFSK
>A0A392 | RR15_COFAR
MVKNTLISVISEEEKRGSVEFQVFRFTNKIRRLTSHLELHKKDYLSQRGLRKILGKRQRL
LAYLAKKNRVRYKELIGLLSIRETKTR
>A0ZZ25 | RPOC2_GOSBA
MAERANLVFHNKVIDGTAIKRLISRLIDHFGMAYTSHILDQVKALGFQQATATSISLGID
DLLTIPSKGWLVQDAEQQSLILEKHHHFGNVHAVEKLRQSIEIWYATSEYLRQEMNPNFR
MTDPFNPVHIMSFSGARGNASQVHQLVGMRGLMSDPQGQMIDLPIQSNLREGLSLTEYII
SCYGARKGVVDTAVLTSDAGYLTRRLVEVVQHIVVRRTDCGTTRGISVSPQKRTLPERIF
IQTLIGRVLADDIYMGPRCIAIRNQDIGLGLVDRFRAFRTQPISIRTPFTCRSTSWICRL
CYGRSPTHGDLVELGEAVGIIAGQSIGEPGTQLTLRTFHTGGVFTGGTAEHVRAPFNGKI
KFNEDLVHPTRTRHGHPAFLCYRDLYVIIESEDIIHKVTIPPKSFLLVQNDQYVESEQVI
AEIRAGTYTLNLKERVRKHIYSDSEGEMHWSTDVYHSPEYTYSNVHLLPKTSHLWILSGG
SYKFSVVPFSLHKDQDQINIHYLSAERRYISRFSVNNDQVRHNLFSSDFSDEKEERIYDY
SELNRIIGTGHCDFIYSAILHENADLLAKRRRNRFIIPFQLIQDQEKELMLHSHSGISME
IPINGIFRRKGILAFFDDPRYRRKSSGITKYGTLGAHSIVKREDVIEYRGVKKVKPKYQM
KVDRFFFIPEEVHILSESSSIMVRNNSIIGVDTPITLNTRSQVGGLVRVERKKKRIELKI
FSGNIYFPGERDKISRHSGILIPPGTGKTNSKESKKLKNWIYVQRITPTKKKYFVLVRPV
TPYEIPDGLNLATLFPQDPFQEKDNMQLRAVNYILYGNGKPTRRISDTSIQLVRTCLVLS
WDQDNKSSFAEEVCASFVEVRTNGLIRDFLRIDLVKSHIFYIRKRNDPSGSELISDNRSD
RTNKNPFYSIYSNARIQQSFSQNHGTIHTLLNRNKESQSLIILSASNCFRMGPFNDVKYH
NVIKQSIKKDPLIPIKNLLGPLGTAPKIANFYSSFYPLITHNQTSVAKYFELDNLKQAFQ
VLNYYLIAENGRIYNFDPCRNIFLNAVNLNWYFPHHHYNYCEETSTIISLGQFICENVCI
AKSGPRLKSGQVFIVQADSIVIRSAKPYLATPGATVHGHYGETLYEGDTLVTFIYEKSRS
GDITQGLPKVEQVLEVRSIDSISMNLEKRIEGWNECITRILGIPWGFVIGAELTIVQSRL
SLVNKIQKVYRSQGVQIHNRHIEIIVRQITSKVLVSEDGMSNVFLPGELIGLLRAERTGR
ALEEAICYRAVLLGITRASLNTQSFISEASFQETARVLAKAALRGRIDWLKGLKENVVLG
GMIPAGTGFKGLVHRSRQHNNILLETKKKNFFGGEMRDIFFHHRELFDSCFSNNLHDTSG
RSFIGIEFNDS
>A0ZZ26 | RPOC1_GOSBA
MIDRYKHQQLRIGSVSPQQISAWAKKILPNGETVGEVTKPYTFHYKTNKPEKDGLFCERI
FGPIKSGICACGNYRVIGNQKEGPKFCEQCGVEFVDSRIRRYQMGYIRLACPVTHVWYLK
RLPSYIANLLDKPLKELEGLVYCDFSFARPIAKKPTFLRLRGSFEYEIQSWKYSIPLFFT
TQGFDTFRSREISTGAGAIREQLADLDLRILIDYSVVEWKELGEEGLTGNEWEDRKIGRR
KDFLVRRMELAKHFIRTNIEPEWMVLCLLPVLPPELRPIIQIDGGKLMSSDINELYRRVI
YRNNTLTDLLTTSRSTPGELVMCQEKLVQEAVDTLLDNGIRGQPMRDGHNKVYKSFSDVI

EGKEGRFRETLLGRRVDYSGRSVIVVGPSLSLHRCGLPREIAIELFQTFVIRGLIRQHLA
PNIGVAESKIREKGPIVWEILQEVMRGHPVLLNRAPTLHRLGIQAFQPILVEGRAICLHP
LVCKGFNADFDGDQMAVPFSLEMQAEARLLMFSHMNLFSPAIGDPISILTQDMLIGLYVL
TSGNRRGICANRYNPWNLKSYQNQRFDNNNYKSTREPFFFFLIHVMRLELMGRKESIQIV
LCDSGGNQSNALLLQEKLPSKFTMNLRVPIMRFMDIIQQQEVQKKKFFVYTFEPLLVIFL
FTEKSKKLYKGFFGPTHMIPNHMVSKLKNSMTPI
>A0ZZ27 | RPOB_GOSBA
MLGDENGEMSTIPGLNQIQFEGFCGFMDRGLTEELYKFPKIEDTEQEIEFQLFVETYQLV
EPLIKERDAVYESLTYSSELYVSAGLIWKTSKDMQEQTIFIGNIPLMNSLGTSIVNGIYR
IVINQILQSPGIYYRSELDHNGISVYTGTIISDWGGRLELEIDRKARIWARVSRKQKISI
LVLSSAMGSNLREILENVCYPEIFLSFLTDKEKKKIGSKENAILEFYQQFSCVGGDPVFS
ESLCKELQKKFFQQRCELGRIGRRNMNQRLNLNIPQNNTFLLPRDILAAADRLIGMKFGM
GPLDDMNHLKNKRIRSVADLLQDQFGLALVRLENVVRGTICGAIRHKLIPTPQNLVTSTP
LTTTYESFFGLHPLSQVLDRTNPLTQIVHGRKLSYLGPGGLTGRTANFRIRDIHPSHYGR
ICPIDTSEGINVGLIGSLAIHARIGHWGSLESPFYKIFERSKKAQMLYLSPSRDEYYMVA
AGNSLALNQGIQEEQVVPARYRQEFLTIAWEQVHLRSIFPFQYFSIGASLIPFIEHNDAN
RALMSSNMQRQAVPLSRSEKCIVGTGLERQVALDSGVPAIADHEGKIISTDTDKIILSGN
GDALGIPLVMYQRSNKNTCMHQTARVRRGKCIKKGQILADGAATVGGELALGKNVLVAYM
PWEGYNFEDAVLISERLVYEDIYTSFHIRKYEIQTHVTSQGPERITNEIPHLEAHLLRNL
DKNGIVMLGSWVETGDILVGKLTPQVAKESSYAPEDRLLRAILGIQVSTSKETCLKLPIG
GRGRVIDVRWVQKKGGSSYNPETIRVYISQKREIKVGDKVAGRHGNKGIISKILPRQDMP
YLQDGRPVDMVFNPLGVPSRMNVGQLFECSLGLAGSLLDRHHRIAPFDERYEQEASRKLV
FSELYQASKQTANPWVFEPEYPGKSRIFDGRTGGPFEQPVIIGKPYILKLIHQVDDKIHG
RSSGYYALVTQQPLRGRSKQGGQRVGEMEVWALEGFGVAHILQEMLTYKSDHIRARQEVL
GTTIIGGTIPKPEDAPESFRLLVRELRSLALELNHFLVSEKNFQINRKEA
>A0ZZ33 | RR14_GOSBA
MAKKSLIHREKKRQKLEQKYHLIRRSSKKEISKVPSLSEKWKIHGKLQSSPRNSAPTRLH
RRCFSTGRPRANYRDFGLSGHILREMVHACLLPGATRSSW
>A0ZZ43 | RBL_GOSBA
MSPQTETKASVGFKAGVKEYKLTYYTPEYEVKDTDILAAFRVTPQPGVPPEEAGAAVAAE
SSTGTWTTVWTDGLTSLDRYKGRCYDIEPVPGEEDQYICYVAYPLDLFEEGSVTNMFTSI
VGNVFGFKALRALRLEDLRVPTAYIKTFQGPPHGIQVERDKLNKYGRPLLGCTIKPKLGL
SAKNYGRAVYECLRGGLDFTKDDENVNSQPFMRWRDRFLFCAEAIFKSQAETGEIKGHYL
NATAGTCEEMIKRAMCARELGVPIVMHDYLTGGFTANTSLAHYCRDNGLLLHIHRAMHAV
IDRQKNHGMHFRVLAKALRMSGGDHIHAGTVVGKLEGERDITLGFVDLLRDDVIEKDRSR
GIYFTQDWVSMPGVLPVASGGIHVWHMPALTEIFGDDSVLQFGGGTLGHPWGNAPGAVAN
RVALEACVQARNEGRDLAREGNEIIREASKWSPELAAACEVWKAIKFEFDAVDKLDKPAS

>A0ZZ58 | RK20_GOSBA
MTRIKRGYIARRRRKKISLFASSFRGAHSRLTRTITQQRIRALVSAHRDRDRKKRDFRRL
WITRINAVIRGVGVSYSYSRLIHNLYKKQLLLNRKILAQIAISNRNCLYMISNEIRKEGD
RKESNEML
>A0ZZ70 | RR8_GOSBA
MGKDTIADIITSIRNADMNRKGTIQIGSTNITENIVQILLREGFIDNVRKHRERNKYFLV

LTLRHRRNRKGPHRTILNLRRISRPGLRIYSNYQQIPRILGGMGIVILSTSRGIMTDREA
RLEGIGGEILCYIW
>A0ZZ74 | RK22_GOSBA
MIKIKKHRRNPYTTSDEVYALGQHICMSAHKARRIIDQIRGRSYEETLMILELMPYRACY
PILKLVYSAAANARHNRGFNEASLIISQVAVNEGTTLKRLNPRARGRSYLIKRPTCHITI
ALKDLEFEPLDRYMLRPKPKNTGWLGWLKKG
>A0ZZ75 | RR19_GOSBA
MARSLKKNPFVANHLLKKIERLNTKAEKEIIITWSRASTIIPTMIGHTIAIHNGKEHLPI
YITDRMVGHKLGEFAPTINFRGHAKNDNKSRR
>A0ZZ77 | RK23_GOSBA
MDGIKYVVVTDKSIRLLVKNQYTSNVESGSTRTEIKHWVELFFGVKVIAMNSHRLPGKGR
RMGPIMGHTMHYRRMIITLQPGYSIPPLRTKRT
>A0ZZ83 | RK32_GOSBA
MAVPKKRTSTSKKRIRKNVWKKKGYWAALKAFSLAKSLSTGNSKSFFVRQINLE
>A0ZZ85 | NU4C_GOSBA
MNYFPWLTIIVFLPISAGSLLFFLPHKGNKLIKWYTICICILELLLTTYAFCYHFRLDDP
LIQLAEDYKWINFFDFYWRLGIDGLSIGPILLTGFITTLATLAAWPVTRDSRLFHFLMLA
MYSGQIGSFSSRDLLLFFIMWEFELIPVYLLLSMWGGKKRLYSATKFILYTAGGSVFLLI
GVLGLGLYGSNEPTLNFETLANQSYPVALEIIFYIGFLIAFAVKSPIIPLHTWLPDTHGE
AHYSTCMLLAGILLKMGAYGLVRINMELLPHAHSIFSPWLIIVGTMQIIYAASTSLGQRN
LKKRIAYSSVSHMGFIIIGIGSITDTGLNGAILQIISHGFIGAALFFLAGTSYDRMRLVY
LDEMGGMAVSIPKIFTMFSILSMASLALPGMSGFVAELIVFFGIITSQKYFLMPKILITF
VMAIGMILTPIYSLSMSRQMFYGYKLFNAPSSYFFDSGPRELFVSISIFLPVIGIGIYPD
FVLSLSGEKVETILYNYFYR
>A0ZZ92 | RR15_GOSBA
MVKNSFISVIFQEKKEENRGSAEFQIVSFTNKIRRLTSHLELHKKDYLSQRGLRKILGKR
QRLLSYLSKTNKIRYKELIGELDIRESKNR
>A1E9M3 | RR11_HORVU
MAKAIPKIGSRKKVRIGLRRNARFSLRKSARRITKGVIHVQASFNNTIITVTDPQGRVVF
WSSAGTCGFKSSRKASPYAGQRTAVDAIRTVGLQRAEVMVKGAGSGRDAALRAIAKSGVR
LSCIRDVTPMPHNGCRPPKKRRL
>A1E9N8 | RK32_HORVU
MAVPKKRTSMSKKRIRKNIWKKKTYFSIVQSYSLVKSRSFSSGNEHPKPKGFSGQQTNK
>A1E9Q2 | RR12_SORBI
MPTVKQLIRNARQPIRNARKSAALKGCPQRRGTCARVYTINPKKPNSALRKVARVRLTSG
FEITAYIPGIGHNLQEHSVVLVRGGRVKDLPGVRYRIIRGTLDAVAVKNRQQGRSKYGAK
KPKK
>A1E9R5 | RPOC1_SORBI
MIDQYKHKQLQIGLVSPQQIKAWAKKILPNGEVVGEVTRPSTFHYKTDKPEKDGLFCERI
FGPIKSGICACGNSRASVAENEDERFCQKCGVEFVDSRIRRYQMGYIKLACPVTHVWYLK
GLPSYIANLLDKPLKKLEGLVYGDFSFARPSAKKPTFLRLRGLFEDEISSCNHSISPFFS
TPGFATFRNREIATGAGAIREQLADLDLRIIIENSLVEWKELEDEGYSGDEWEDRKRRIR
KVFLIRRMQLAKHFIQTNVEPEWMVLCLLPVLPPELRPIVYRSGDKVVTSDINELYKRVI
RRNNNLAYLLKRSELAPADLVMCQEKLVQEAVDTLLDSGSRGQPMRDGHNKVYKSLSDVI

EGKEGRFRETLLGKRVDYSGRSVIVVGPSLSLHQCGLPLEIAIKLFQLFVIRDLITKRAT
SNVRIAKRKIWEKEPIVWEILQEVMRGHPVLLNRAPTLHRLGIQAFQPTLVEGRTICLHP
LVCKGFNADFDGDQMAVHLPLSLEAQAEARLLMFSHMNLLSPAIGDPICVPTQDMLIGLY
VLTIGNRRGICANRYNSCGNSPNKKINYNNNNYYKYTKDKEPHFSSSYDALGAYRQKRIG
LNSPLWLRWKLDQRIVGSREVPIEVQYESFGTYHEIYAHYLVVGNRKKEIRSIYIRTTLG
HISFYREIEEAIQGFSRAYSYTI
>A1E9S6 | RR4_SORBI
MSRYRGPRLKKIRRLGALPGLTRKTPKSGSNQKKKFHSGKKEQYRIRLQEKQKLRFHYGL
TERQLLRYVHIAGKAKRSTGQVLLQLLEMRLDNILFRLGMASTIPGARQLVNHRHILVNG
RIVDIPSFRCKPRDIITTKDNQRSKRLVQNYIASSDPGKLPKHLTVDTLQYKGLVKKILD
RKWVGLKINELLVVEYYSRQT
>A1E9T2 | RBL_SORBI
MSPQTETKASVGFKAGVKDYKLTYYTPEYETKDTDILAAFRVTPQLGVPPEEAGAAVAAE
SSTGTWTTVWTDGLTSLDRYKGRCYHIEPVPGDPDQYICYVAYPLDLFEEGSVTNMFTSI
VGNVFGFKALRALRLEDLRIPPAYVKTFQGPPHGIQVERDKLNKYGRPLLGCTIKPKLGL
SAKNYGRACYECLRGGLDFTKDDENVNSQPFMRWRDRFVFCAEAIYKAQAETGEIKGHYL
NATAGTCEEMIKRAVFAKELGVPIVMHDYLTGGFTANTTLSHYCRDNGLLLHIHRAMHAV
IDRQKNHGMHFRVLAKALRMSGGDHIHSGTVVGKLEGEREITLGFVDLLRDDFIEKDRSR
GIFFTQDWVSMPGVIPVASGGIHVWHMPALTEIFGDDSVLQFGGGTLGHPWGNAPGAAAN
RVALEACVQARNEGRDLAREGNEIIKAACKWSAELAAACEIWKEIKFDTFKAMDTL
>A1E9T3 | RK23A_SORBI
MDNGIKYAVFTEKSLRLLGKNQYTFNVESGFTKTEIKHWVELFFGVKVVAVNSHRLPGKG
RRMGPILGHTMHYRRMIITLQLGILFHFYPLNSRVF
>A1E9U5 | RK33_SORBI
MAKGKDVRIRVILECISCVRKGTNKESTGISRYSTQKNRHNTPGQLELRKFCRYCRKHTT
HNEIKK
>A1E9U6 | RR18_SORBI
MYISKQPFRKSKQPFRKSKQTFHKSKQPFRKFKQPFRKSKQPFRRRSRIGPGDRIDYRNM
SLINRFISEQGKILSRRINRLTLKQQRLITVAIKQARILSFLPFRNYENEKQFQAQAISI
ITGPRHRKNRHIPQLTQKFNSNRNLRNSNQNLRNNNRNLSSDC
>A1E9U7 | RK20_SORBI
MTRVPRGYIARRRRTKMRSFASNFRGAHLRLNRMITQQVRRAFVSSHRDRGRQKRDFRRL
WITRINAATRVYNVFNSYSKLIHNLSKKELILNRKMLAQVAVSNPNNLYTISNKIRTIN
>A1E9W0 | RR8_SORBI
MGKDTIADLLTSIRNADMNKKGTVRVVSTNITENIVKILLREGFIESVRKHQESNRYFLV
STLRHQRRKTRKGIYRTRTFLKRISRPGLRIYANYQGIPKVLGGMGIAILSTSRGIMTDR
EARLNRIGGEVLCYIW
>A1E9W2 | RK16_SORBI
MLSPKRTRFRKQHRGRMKGKSCRGNHICFGRYALQVLEPAWITARQIEAGRRAMTRYARR
GGKIWVRIFPDKPVTIRPTETRMGSGKGSPEYWVAVVKPGRILYEMSGVSETVARAAISI
AASKMPIRSQFLRLEI
>A1E9W3 | RR3_SORBI
MGQKINPLGFRLGTTQNHHSFWFAQPKNYSEGLQEDKKIRNCIKNYIQKNRKKGSNRKME
SDSSSEVITHIEIQKEIDTIHVIIHIGFPNLLKKKGAIEELEKDLQKEVNSVNQRLNIAI

EKVKEPYRQPNILAEYIAFQLKNRVSFRKAMKKAIELTKKADIKGIKIQIAGRLAGKEIA
RAECIKKGRLPLQTIRAKIDYCCYPIRTIYGVLGVKIWIFVEEE
>A1E9W6 | RK2_SORBI
MAKHLYKTPIPSTRKGTVDRQVKSNPRNKLIHGRHRCGKGRNARGIITARHRGGGHKRLY
RKIDFRRNQKDISGRIVTIEYDPNRNAYICLIHYGDGEKRYILHPRGAIIGDTIVSGTKV
PISMGNALTDMPLGTAIHNIEITRGRGGQLARAAGAVAKLIAKEGKLATLRLPSGEVRLV
SQNCLATVGQVGNVGVNQKSLGRAGSKCWLGKRPVVRGVVMNPVDHPHGGGEGKAPIGRK
KPTTPWGYPALGRRTRKRKKYSDSFILRRRK
>A1E9X2 | RK32_SORBI
MAVPKKRTSMSKKRIRKNLWKKKTYFSIVQSYSLAKSRSFSSGNEHPKPKGFSGQQANK
>A1E9X4 | NU4C_SORBI
MSYFPWLTILVVLPIFAGSLIFFLPHKGNKIVRWYTIAICLLEFLIMTYAFCYHFQLEDP
LIQLKEDSKWIDVFDFHWRLGIDGLSLGSILLTGFITTLATLAAWPVTRNSQLFYFLMLA
MYSGQIGLFSSRDLLLFFIMWELELIPVYLLLSMWGGKRRLYSATKFILYTAGGSIFFLI
GVLGMGLYGSNEPGLDLERLINQSYPTTLEILLYFGFLIAYAVKLPIIPLHTWLPDTHGE
AHYSTCMLLAGILLKMGAYGLIRVNMELLPHAHYLFSPWLVIIGAVQIIYAASTSLGQRN
FKKRIAYSSVSHMGFIIIGIGSITNIGLNGAILQILSHGFIGATLFFLAGTACDRMRLVY
LEELGGISIPMPKIFTMFSSFSMASLALPGMSGFVAELVVFFGLITSPKFMLMPKMLITF
VMAIGMILTPIYLLSMLRQMFYGYKLFHVPNKNFVDSGPRELFLLICIFLPVIGIGIYPD
FVLSLSVDRVEALLSNYYTK
>A1E9Y7 | RR19_AGRST
MTRKKTNPFVAHHLLAKIEKVNRKEEKETIVTWSRASSILPTMVGHTIAIHNGKEHIPIY
ITNPMVGRKLGEFVPTRHFTSYENARKDTKSRR
>A1E9Z0 | RR16_AGRST
MVKLRLKRCGRKQQAVYRIVAIDVRSRREGRDLRKVGFYDPIKNQTCLNVPAILYFLEKG
AQPTRTVYDILRKAELFKEKERILS
>A1EA17 | RK23A_AGRST
MDNGIKYAVFTEKSLRLLGKNQYTFNVESGFTKTEIKHWVELFFGVKVVAVNSHRLPGKG
RRIGPILGHTMHYRRMIIILQPGYSIPLLDREKN
>A1EA30 | RR18_AGRST
MYTSKQPFLKSKQPFRKSKQTFNKSKQTFRKSKQTFRKFKQPFRKSKQPFRRRPRIGPGD
RIDYRNMSLINRFISEQGKILSRRINRLTLKQQRLITLAIKQARILSFLPFRNYENEKQF
QAQSISIITGSRPRKNRHIPQLTEKYNSNRNLRNYNQNLRNINRNLSSD
>A1EA31 | RK20_AGRST
MTRVPRGYIARRRRTKMRSFASNFRGAHLRLNRMITQQVKRAFVSSHRDRGRQKRDFRRL
WITRINAATRIYKVFDSYSKLIHNLYKKKLILNRKMLAQVAVSNPNNLYTISNKIKIIN
>A1EA41 | RR11_AGRST
MAKAIPKIGSRKKVRIGLRRNARFSLRKSARRITKGVIHVQASFNNTIITVTDPQGRVVF
WSSAGTCGFKSSRKASPYAGQRTAVDAIRTVGLQRAEVMVKGAGSGRDAALRAIAKSGVR
LSCIRDVTPMPHNGCRPPKKRRL
>A1EA43 | IF1C_AGRST
MTEKKNRREKKNPREAKVTFEGLVTEALPNGMFRVRLENDTIILGYISGKIRSSSIRILM
GDRVKIEVSRYDSSKGRIIYRLPHKDSKRMEDSKDIEDLKDSEDSKD
>A1EA45 | RK14_AGRST

MIQPQTLLNVADNSGARKLMCIRVIGAAGNQRYARIGDVIVAVIKDALPQMPLERSEVIR
AVIVRTCKEFKCEDGIIIRYDDNAAVIIDQKGNPKGTRVFGAIAEELRELNFTKIVSLAP
EVL
>A1EA50 | RK2_AGRST
MAKHLYKTPIPSTRKGTVDRQVKSNPRNNLIHGRHRCGKGRNSRGIITARHRGGGHKRLY
RKIDFRRNQKDISGRIVTIEYDPNRNAYICLIHYGDGEKRYILHPRGAIIGDTIVSGTKV
PISMGNALPLTDMPLGTAMHNIEITRGRGGQLARAAGAVAKLIAKEGKSATLRLPSGEVR
LVSQNCLATVGQVGNVGVNQKSLGRAGSKCWLGKRPVVRGVVMNPVDHPHGGGEGKAPIG
RKKPTTPWGYPALGRRTRKRKKYSDSFILRRRK
>A1XFT4 | RR12_NUPAD
MPTIKQLIRNTRQPMRNVTKSPALRGCPQRRGTCTRVYTITPKKPNSALRKVARVRLTSG
FEITAYIPGIGHNLQEHSVVLVRGGRVKDLPGVRYHIVRGTLDAVGVKDRQQGRSKYGVK
KPK
>A1XFZ7 | RK2_NUPAD
MAIHLYKTSTPSTRKGAVDSQVKSNPRTNLIYGQHRCGKGRNARGIITARHRGGGHKRLY
RKIDFRRNEKDISGRIVTIEYDPNRNAYICLIHYGDGEKRYILHPRGAIIGDTIVSGTEV
PISMGNALPLTDMPLGTAIHNIEITLGKGGQLARAAGAVAKLIAKEGKSATLRLPSGEVR
LISKNCSATVGQVGNVGVNQKSLGRAGSKCWLGKRPVVRGVVMNPVDHPHGGGEGRAPIG
RKKPTTPWGYPALGRRSRKRNKYSDIFILRRRK
>A2CI70 | RK32_CHLAT
MAVPKKRVSKSKRDMRKTTWKNKASKEAKKALSLAKSVSTGKSKSKGFQIKSSN
>A2T365 | RPOA_ANGEV
MIQDEVPVSAQTIQWRCIESKIESKRLHYGRFAISPFRKGQANTVGIAIRRSLLGEIEGT
AITYAKSKNVIHEYSTIIGIEESINDILINFKEIVLRSDSYETQKAYISITGPKDITAED
ILLPPSVQAIDDSQHIATITKDITLDIEIEIQKDRGYRIQDSKESQAGEFFIDAVFMPIR
KANYSVHSFGNNKKFQEILFIEIWTDGSLTPKEALYEASRNLIDLFLPFLHTEEEEIISD
RDEKSESNGNILLSNSISTDIDRMAKEVAFKHIFIDQLELPARAYNCLKKVDVHTISDLL
KYSQDDLRKIKNFGKKSVDQVLEALQERFAINLPRNKFSID
>A2T381 | RK32_ANGEV
MAVPKKRTSKSKKRIRKSVWREKTKKIASKAFSLAQSILTNRSKSFYYTTNEKISESTE
>A2XDA1 | CRTI_ORYSI
MDTGCLSSMNITGTSQARSFAGQLPTHRCFASSSIQALKSSQHVSFGVKSLVLRNKGKRF
RRRLGALQVVCQDFPRPPLENTINFLEAGQLSSFFRNSEQPTKPLQVVIAGAGLAGLSTA
KYLADAGHKPILLEARDVLGGKIAAWKDEDGDWYETGLHIFFGAYPNIQNLFGELGINDR
LQWKEHSMIFAMPNKPGEFSRFDFPETLPAPLNGIWAILRNNEMLTWPEKVKFALGLLPA
MVGGQAYVEAQDGFTVSEWMKKQGVPDRVNDEVFIAMSKALNFINPDELSMQCILIALNR
FLQEKHGSKMAFLDGNPPERLCMPIVDHVRSLGGEVRLNSRIQKIELNPDGTVKHFALTD
GTQITGDAYVFATPVDILKLLVPQEWKEISYFKKLEKLVGVPVINVHIWFDRKLKNTYDH
LLFSRSSLLSVYADMSVTCKEYYDPNRSMLELVFAPAEEWVGRSDTEIIEATMQELAKLF
PDEIAADQSKAKILKYHVVKTPRSVYKTIPDCEPCRPLQRSPIEGFYLAGDYTKQKYLAS
MEGAVLSGKLCAQSVVEDYKMLSRRSLKSLQSEVPVAS
>A2Y205 | RBS1_ORYSI
MAPSVMASSATTVAPFQGLKSTAGMPVARRSGNSSFGNVSNGGRIRCMQVWPIEGIKKFE
TLSYLPPLTVEDLLKQIEYLLRSKWVPCLEFSKVGFVYRENHRSPGYYDGRYWTMWKLPM

FGCTDATQVLKELEEAKKAYPDAFVRIIGFDNVRQVQLISFIAYKPPGCEESGGN
>A2YQD9 | FER1_ORYSI
MAATALSSQVRLPMSLRVATAPAPARVSVLPASNKLGDRLRMQATYNVKLITPDGEVELQ
VPDDVYILDQAEEEGIDLPYSCRAGSCSSCAGKVVSGEIDQSDQSFLDDDQVAAGWVLTC
HAYPKSDVVIETHKEDDLI
>A4GG84 | RR3_PHAVU
MGQKINPLGFRLGTTQSHDSIWFAQPTKYSENIQEDKKIRDWIKNYIQKNIRISSGVEGI
GEIKIQKRIDLIQVIIYMGFPKLLIEGKPHKIEEFQTNMHKKLNCVNKKLNIAIVKITNA
YKHPNILAEFIAGQLKNRVSFRKAMKKAIELTEQAGTKGVQVQIAGRIDGKEIARVEWIR
EGRVPLQTIRAKIEYCCYTVRTIYGILGIKVWIFSK
>A4GG95 | RR4_PHAVU
MSRYRGPCFKKIRRLGYLPGLTSKKPTVKNELRNQLRFSKKSQYRIRLEEKQKLRFHYGL
TERQLLKYVRISGKAKGSTGQVLLQLLEMRLDNILFRLGMAATIPQARQFINHRHVLVNG
RIVDIPSYRCKPQDIITAKDEQKSKTLIQNYLDSAPRDKLPNHLTVHPFQYKGLINQIID
NKWVGLKINELLVVEYYSRQT
>A4GGD7 | RPOA_PHAVU
MVQEKLRVSTRTLQWKCVESRIDSKRLYYGRFILSPLMKGQADTIGIAIRRILLGEIEGT
CITCVKSEKIPHEYSTIIGIEESVHEIFMNLKEIVLKSNMYGIQDASISFKGPGYITAQD
IILPPSVEIVDNRQHIANVTEPVHLCIELKIERNRGYRIKTLKNFQDGSYDIDARFMPVR
NVNYSIHSYVNGNEKQEILFLEIWTNGSLTPKEALYEASQNLIELFIPFLHAEEDNFHLE
KNQHKVTLPLFTFHDILVKDKLRKNQKEIALKSIFIDQLELPPRIYNCLKRSNIHTLLEL
LSNSQEDLLKFEHFHVEDGKSILDILKIQKYFA
>A4GGE4 | RK32_PHAVU
MAVPKKRTSISKKIIRNTLWKKKGYFTALKAFSLAQSLFTGNSKSFFCNKYKR
>A4GYQ0 | RPOB_POPTR
MLGDGNGGMSTIPGFNQIQFEGFCRFIDQGLAEELYKFPKIEDRDQEIEFQLFVETYQLV
EPSIKERDAVYESLTYSSELYVSGGLIWKNSRDMQEQTIFIGNIPLMNSLGTSIVNGIYR
IVINQILQSPGIYYRSELNHNGISVYTGTIISDWGGRVELEIDKKARIWARVSRKQKISI
LVLSSAMGLNLREILENVCYPEIFLSFLSDKEKKKIGSRENAILEFYQQFTCVGGGPVFS
ESLCKELQKKFFQQRCELGRIGRLNMNQRLNLDIPHNNTFLLPRDILAAADHLIGMKFGM
GTLDDMNHLKNKRIRSVADLLQDQFGLALIRLENVVRGTICGAIRHKLIPTPQNLVTSTP
LTTTYESFFGLHPLSQVLDRTNPLTQIVHGRKSSYLGPGGLTGRTASFRIRDIHPSHYGR
ICPIDTSEGINVGLIGSLTIHAKIGHLGSLESPFYEISARSKKVRMLYLSPNRDEYYMIA
AGNCLALNRGAREEQVVPARYRQEFLTIAWEQVRLRSFFPFQYFSIGASLIPFIEHNDAN
RALMSSNMQRQAVPLARSEKCIVGTGLERQVALDSGVPAIAEHEGKIIYTDIDKIILSGN
GYTVSIPLVMYQRSNKNTCMHQKTQVQRGKCIKRGQVLADGAATVGGELALGKNILVAYM
PWEGYNFEDAVLISERLVYEDVYTSFHIRKYEIQTHVTSQGPERITNEIPHLEAHLLRNL
DKNGIVMLGSWVETGDILIGKLTPQLAKESSYAPEDRLLRAILGIQVSTSKETCLKLPTG
GRGRVIDVRWIQKKGGSSYNPETIRVYILQKREIKVGDKVAGRHGNKGIISKILPRQDMP
YLQDGAPVDMVFNPLGVPSRMNVGQIFECSLGLAGSLLDRHYRVAPFDERYEQEASRKLV
FSELYEAGKQTGNPWVFEPECPGKSRIFDGRTGDPFEQPVIIGKPYILKLIHQVADKIHG
RSSGHYALVTQQPLRGRAKQGGQRVGEMEVWALEGFGVSHILQEMLTYKSDHIRARQEVL
GTTISGRTIPKPEDAPESFRLLVRELRSLALELKHFLISEKNFQINRKEV
>A4GYR2 | RR4_POPTR

MSRYRGPRFKKIRRLGALPGLTSKRPRAGSDLRNQSRAGKKSQYRIRLEEKQKLRFHYGL
TERQLLKYVRIAAKAKGSTGQVLLQLLEMRLDNILFRLGMASTIPRARQLVNHRHILVNG
RIVDIPSYRCKPRDIITAKDEQKSRVMIQNSLDSFPQEELPKHLTLHPFQYKGLVNHIID
SKWIGLKINELLVVEYYSRQT
>A4GYR8 | RBL_POPTR
MSPQTETKAGVGFKAGVKDYKLTYYTPDYETKDTDILAAFRVTPQPGVPPEEAGAAVAAE
SSTGTWTTVWTDGLTSLDRYKGRCYDIEPVAGEENQFIAYVAYPLDLFEEGSVTNMFTSI
VGNVFGFKALRALRLEDLRIPPAYVKTFQGPPHGIQVERDKLNKYGRPLLGCTIKPKLGL
SAKNYGRAVYECLRGGLDFTKDDENVNSQPFMRWRDRFLFCAEALYKAQAETGEIKGHYL
NATAGTCEEMIKRAVFARELGVPIVMHDYLTGGFTANTSLAHYCRDNGLLLHIHRAMHAV
IDRQKNHGIHFRVLAKALRMSGGDHIHSGTVVGKLEGERDITLGFVDLLRDDFVEKDRSR
GIYFTQDWVSLPGVLPVASGGIHVWHMPALTEIFGDDSVLQFGGGTLGHPWGNAPGAVAN
RVALEACVQARNEGRDLAREGNEIIREASKWSPELAAACEVWKEIKFEFQAMDTL
>A4GYT5 | RR12A_POPTR
MPTIKQLIRNTRQPIRNVTKSPALGGCPQRRGTCTRVYTITPKKPNSALRKVARVRLTSG
FEITAYIPGIGHNSQEHSVVLVRGGRVKDLPGVRYHIVRGTLDAVGVKDRQQGRSQYGVK
KPK
>A4GYT6 | CLPP_POPTR
MPIGVPKVPFRNPGEDSSNWIDVYNRLYRERLLFLGQDIDSEISNQLIGLMVYLSTESEI
KDLYLFINSPGGWVIPGIAIYDTMQFVRPDVQTVCMGLAASMGSFILVGGKITKRLAFPH
ARVMIHQPFAAFYEAQIGEFVLEAEELLKLREILTRVYAQRTGKPLWVVSEDMERDVFMS
AAEAQVHGIVDLVAVA
>A4GYU3 | RPOA_POPTR
MVREKVRISTRTLQWKCVESRADSKRLYYGRFILSPLMKGQADTIGIAMRRALLGEIEGT
CITRAKSKKIPHEFSTITGIQESIHEILMNLKEIVLRSNLYGTHDASICVKGPGCVTAQD
IILPPSVEIIDNTQHIASLREPIDLHIGLEIERNRGYCMKPPKNFQDGSYSIDAVFMPVR
NANHSVHSYGNGNEKQEILFLEIWTNGSLTPKEALHEASRNLIDLFIPFLHAEEENFHLE
KNQHKITLPLFAFHDRLAKLRKNQKEIALKSIFIDQLELAPKIYNCLKRSNIHTLWDLLK
NSQEDLMKIEHFRIEDVKHIFGILKIEKHFTINLPKNKF
>A4GYU6 | RR8_POPTR
MGRDTLADIITSIRNADMDRKGTVRIPSTNITENIIKILLREGFIENVRKHQEGNFFFFA
LTLRHRRNRKGPCRTSLNLKRISRPGLRIYSNYQQIPRILGGMGIVILSTSRGIMTDREA
RLERIGGEILCYIW
>A4GYU9 | RR3_POPTR
MGQKINPLGFRLGTTQDHYSLWFAQPKNFFEGLQEDQKIRNCIKNYVQKNMKISSGVEGI
GHIEIQKRIDVIQVIIYLGFPKFLTEGKPKRIKELQINVQKELNCMNRKLNISITRIENP
YMHPNVLAEFIAGQLKNRVSFRKAMKKAIELTEQSNTKGIQVQIAGRLDGKEIARAEWVR
EGRVPLQTLRAKINYCSYTVRTIYGVLGIKIWIFVDEE
>A4GYV2 | RK2_POPTR
MAIHLYKTSTPSTRNGAVDSQVKSNTRNNLIYGQHRCGKGRNSRGIITARHRGGGHKRLY
RKIDFRRNEKYIYGRIVTIEYDPNRNAYICLIHYGDGEKRYILHPRGAIIGDTIISGTEV
PIKMGNALPLTDMPLGTAIHNIEITLGRGGQLARAAGAVAKLIAKEGKSATLKLPSGEVR
LISKNCSATVGQVGNAGVNQKSLGRAGSKCWLGKRPVVRGVVMNPVDHPHGGGEGRAPIG
RKKPATPWGYPALGRRSRKRNKYSDNLILRRRSK

>A4QJA7 | RPOB_AETCO
MLGDGKEGTSTIPGFNQIQFEGFYRFIDQGLIEEVSKFPKIEDIDQEIEFQLFVETYQLV
EPLIKERDAVYESLTYSSELYVSAGLIWKTSRNMQEQRIFIGNIPLMNSLGTSIVNGIYR
IVINQILQSPGIYYQSELDHNGISVYTGTIISDWGGRLELEIDKKARIWARVSRKQKISI
LVLSSAMGLNLREILENVCYPEIFLSFLTDKEKKQIGSKENAILEFYQQFSCVGGDPIFS
ESLCKELQKKFFHQRCELGRIGRRNINWRLNLTIPQNNIFLLPRDILAAADHLIGMKFGM
GTLDDMNHLQNKRIRSVADLLQDQFGLALARLENVVKGTICGAIKHKLIPTPQNLVTATP
LTTTYESFFGLHPLSQVLDRTNPLTQIVHGRKLSYLGPGGLTGRTANFRIRDIHPSHYGR
ICPIDTSEGINVGLIGSLAIHARIGDWGSLESPFYELFEKSKKARIRMLFLSPSQDEYYM
IAAGNSLALNRGIQEEQAVPARYRQEFLTIAWEEVHLRSIFPFQYFSIGASLIPFIEHND
ANRALMSSNMQRQAVPLSRSEKCIVGTGLERQVALDSGVPAIAEQEGKILYTDTKKIILS
GYGDNTLGIPLIRYQRSNKNTCMHQKPQVRRGKCIKKGQILADGAATVGGELALGKNILV
GYMPWEGYNFEDAVLISECLVYGNIYTSFHIRKYEIQTHVTTQGPERITKEIPHLEGRLL
RNLDKNGIVMLGSWVETGDILVGKLTPQVAKESSYAPEDRLLRAILGIQVSTSKETCLKL
PIGGRGRVIDVRWVQKKGGSSYNPEKIRVYISQKREIKVGDKVAGRHGNKGIISKILPRQ
DMPYLQDGRPVDMVFNPLGVPSRMNVGQIFECSLGLAGSLLDRHYRIAPFDERYEQEASR
KLVFSELYEASKQTANPWVFEPEYPGKSRIFDGRTGDPFEQPVIIGKPYILKLIHQVDDK
IHGRSSGHYALVTQQPLRGRSKQGGQRVGEMEVWALEGFGVAHILQEMLTYKSDHIRARQ
EVLGTTIIGGTIPKPEDAPESFRLLVRELRSLALDLNHFLVSEKNFQINRKEV
>A4QJC3 | RBL_AETCO
MSPQTETKASVGFKAGVKEYKLTYYTPEYETKDTDILAAFRVTPQPGVPPEEAGAAVAAE
SSTGTWTTVWTDGLTSLDRYKGRCYHIEPVPGEESQFIAYVAYPLDLFEEGSVTNMFTSI
VGNVFGFKALAALRLEDLRIPPAYTKTFQGPPHGIQVERDKLNKYGRPLLGCTIKPKLGL
SAKNYGRAVYECLRGGLDFTKDDENVNSQPFMRWRDRFLFCAEAIYKSQAETGEIKGHYL
NATAGTCEEMIKRAVFARELGVPIVMHDYLTGGFTANTSLAHYCRDNGLLLHIHRAMHAV
IDRQKNHGMHFRVLAKALRLSGGDHIHAGTVVGKLEGDRESTLGFVDLLRDDYVEKDRSR
GIFFTQDWVSLPGVLPVASGGIHVWHMPALTEIFGDDSVLQFGGGTLGHPWGNAPGAVAN
RVALEACVQARNEGRDLAVEGNEIIREACKWSPELAAACEVWKEIRFNFPTIDKLDPSAE
KVA
>A4QJE0 | CLPP_AETCO
MPIGVPKVPFRSPGEGDTSWVDIYNRLYRERLFFLGQEVDTDISNQLISLMIYLSIEKDT
KDLYLFINSPGGWVISGMAIYDTMQFVRPDVQTICMGLAASIASFILVGGAITKRIAFPH
ARVMIHQPASSFYEAQTGEFILEAEELLKLRETITRVYVQRTGKPIWVVSEDMERDVFMS
ATEAQAHGIVDLVAVQ
>A4QJE7 | RPOA_AETCO
MVREKVKVSTRTLQWKCVESRRDSKRLYYGRFILSPLMKGQADTIGIAMRRALLGEMEGT
CITRAKSENIPHDYSNIVGIQESVHEILMNLQEIVLRSNLYGTCNALICVQGPGSITARD
IILPPSVEIIDNTQHIATLTEPIDLCIGLQIERNRGYSLKMPNHFEDGSYPIDALFMPVQ
NANHSIHSYGTGNEKQEILFLEIWTNGNLTPKEALHEASRNLINLFIPFLHVEEETFYLE
NNQHKVTLPLFPFHNRLVKLRKKKKELVFQYIFIDQLELPPRIYNCLKKSNIHTLLDLLN
NSQEDLIKIEHFHIEDVKKILEILEKNRKFISN
>A4QJF0 | RR8_AETCO
MGKDTIADIITSIRNADMNRKGTVRIGSTNITESIVKILLREGFIENVRKHRENNQYFLI
LTLRHRRNKKESYKTILNLKRISRPGLRIYSNSQRIPRILGGIGIVILSTSRGIMTDREA

RLKRVGGEILCYIW
>A4QJF3 | RR3_AETCO
MGQKINPLGFRLGTTQSHHSLWFAQPKKYCEGLEEDKKIRDCIKNYVQKNIRISSGMEGI
ARIEIQKRIDLIQVIIYMGFPKLLIEDKPRRVEELQMNVQKELNCVNRKLNIAITRISTP
YGHPNILAEFIAGQLKNRVSFRKAMKKAIELTEQANTKGIQVQIAGRIDGKEIARVEWIR
EGRVPLQTIEAKIDYCSYTVRTIYGVLGIKIWIFVDEE
>A4QJF6 | RK2_AETCO
MAIHLYKTSTPSTRNGAVDSQVKSNPRNNLIYGQHRCGKGRNARGIITARHRGGGHKRLY
RKIDFRRNAKDIYGRIVTIEYDPNRNAYICLIHYGDGEKRYILHPRGAIIGDTIVSGTEV
PIKMGNALPLTDMPLGTAIHNIEITLGKGGQLARAAGAVAKLIAKEGKSATIKLPSGEVR
LISKNCSATVGQVGNVGVNQKSLGRAGSKCWLGKRPVVRGVVMNPVDHPHGGGEGRAPIG
RKKPATPWGYPALGRRTRKRKKYSETLILRRRSK
>A4QJJ1 | RPOB_AETGR
MLGDGKEGTSTIPGFNQIQFEGFYRFIDQGLIEEVSKFPKIEDIDQEIEFQLFVETYQLV
EPLRKERDAVYESLTYSSELYVSAGLIWKTSRKMQEQRIFIGNIPLMNSLGTSIVNGIYR
IVINQILQSPGIYYQSELDHNGISVYTGTIISDWGGRLELEIDKKARIWARVSRKQKISI
LVLSSAMGSNLREILENVCYPEIFLSFLTDKEKKKIGSKENAILEFYQQFSCVGGDPIFS
ESLCKELQKKFFHQRCELGRIGRRNINWRLNLTIPQNNIFLLPRDILAAADHLIGMKFGM
GTLDDMNHLKNKRIRSVADLLQDQFGLALARLENVVKGTICGAIKHKLIPTPQNLVTATP
LTTTYESFFGLHPLSQVLDRTNPLTQIVHGRKLSYLGPGGLTGRTANFRIRDIHPSHYGR
ICPIDTSEGINVGLIGSLAIHARIGDWGSLESPFYELFEKSKKARIRMLFLSPSQDEYYM
IAAGNSLALNRGIQEEQAVPARYRQEFLTIAWEEVHLRSIFPFQYFSIGASLIPFIEHND
ANRALMSSNMQRQAVPLSRSEKCIVGTGLERQVALDSGVPAIAEQEGKILYTDTKKIILS
GNGDNTLGIPLIMYQRSNKNTCMHQKSQVRRGKCIKKGQILADGAATVGGELALGKNILV
AYMPWEGYNFEDAVLISECLVYGDIYTSFHIRKYEIQTHVTTQGPERITKEIPHLEGHLL
RNLDKNGIVMLGSWVETGDILVGKLTPQVAKESSYAPEDRLLRAILGIQVSTSKETCLKL
PIGGRGRVIDVGWVQKKGGSSYNPEKIRVYISQKREIKVGDKVAGRHGNKGIISKILPRQ
DMPYLQDGRPVDMVFNPLGVPSRMNVGQIFECSLGLAGSLLDRHYRIAPFDERYEQEASR
KLVFSELYEASKQTANPWVFEPEYPGKSRIFDGRTGDPFEQPVIIGKPYILKLIHQVDDK
IHGRSSGHYALVTQQPLRGRSKQGGQRVGEMEVWALEGFGVAHILQEMLTYKSDHIRARQ
EVLGTTIVGGTIPKPEDAPESFRLLVRELRSLALDLNHFLVSEKNFQINRKEV
>A4QJK1 | RR4_AETGR
MSRYRGPRFKKIRRLGALPGLTSKRPRAGSDLRNQSRSGKKSQYRIRLEEKQKLRFHYGL
TERQLLKYVRIAGKAKGSTGQVLLQLLEMRLDNTLFRLGMALTIPQARQLVNHGHILVNG
RIVDIPSYRCKPRDIITVKDEQNSRTLVQNLLDSSAPEELPKHLTLHTFQYEGLVNQIID
RKCVGLKINELLVVEYYSRQT
>A4QJM3 | RR12_AETGR
MPTIKQLIRNTRQPIRNVTKSPALRGCPQRRGTCTRVYTITPKKPNSALRKVARVRLTSG
FEITAYIPGIGHNLQEHSVVLVRGGRVKDLPGVRYHIVRGTLDAVGVKDRQQGRSKYGVK
KPK
>A4QJM4 | CLPP_AETGR
MPIGVPKVPFRSPGEGDTSWVDIYNRLYRERLFFLGQEVDTDISNQLISLMIYLSIEKDT
KDLYLFINSPGGWVISGMAIYDTMQFVRPDVQTICMGLAASIASFILVGGAITKRIAFPH
ARVMIHQPASSFYEAQTGEFILEAEELLKLRETITRVYVQRTGKPIWVVSEDMERDVFMS

ATEAQAHGIVDLVAVQ
>A4QJN7 | RR3_AETGR
MGQKINPLGFRLGTTQSHHSLWFAQPKKYCEGLEEDKKIRDCIKNYVQKNIRISSGMEGI
ARIEIQKRIDLIQVIIYMGFPKLLIEDKPRRVEELQMNVQKELNCVNRKLNIAITRISTP
YGHPNILAEFIAGQLKNRVSFRKAMKKAIELTEQANTKGIQVQIAGRIDGKEIARVEWIR
EGRVPLQTIEAKIDYCSYTVRTIYGVLGIKIWIFVDEE
>A4QJV5 | RR12A_OLIPU
MPTIKQLIRNTRQPIRNVTKSPALRGCPQRRGTCTRVYTITPKKPNSALRKVARVRLTSG
FEITAYIPGIGHNLQEHSVVLVRGGRVKDLPGVRYHIVRGTLDAVGVKDRQQGRSKYGVK
KPK
>A4QJV6 | CLPP_OLIPU
MPIGVPKVPFRSPGEGDTSWVDIYNRLYRERLFFLGQEVDTEISNQLISLMIYLSIEKDT
KDLYLFINSPGGWVISGMAIYDTMQFVRPDVQTICMGLAASIASFILVGGAITKRIAFPH
ARVMIHQPASSFYEAQTGEFILEAEELLKLRETITRVYVQRTGKPIWVVSEDMERDVFMS
ATEAQAHGIVDLVAVQ
>A4QJW6 | RR8_OLIPU
MGKDTIADIITSIRNADMNRKGTVRIGSTNITESIVKILLREGFIENVRKHRESNQYFLI
LTLRHRRNKKESYKTILNLKRISRPGLRIYSNSQRIPRILGGIGIVILSTSQGIMTDREA
RLKRIGGEILCYIW
>A4QJY0 | RK32_OLIPU
MAVPKKRTSISKKRIRKKIWKRKGYWTSLKAFSLGKSLSTGNSKSFFVQQNK
>A4QK51 | RR11_ARAHI
MAKPILRIGSRKNTRSGSRKNVRRIPKGIIHVQASFNNTIVTVTDVRGRVISWSSAGTCG
FRGTRRGTPFAAQTAAGNAIRAVVDQGMQRAEVRIKGPGLGRDAALRAIRRSGILLSFVR
DVTPMPHNGCRPPKKRRV
>A4QK53 | RR8_ARAHI
MGKDTIADIITSIRNADMNRKGTVRIESTNITESIVKILLREGFIENVRKHRENNQYFLI
LTLRHRRNKKESYKTILNLKRISRPGLRIYSNSQRIPRILGGIGIVILSTSQGIMTDREA
RLKRIGGEILCYIW
>A4QK68 | RK32_ARAHI
MAVPKKRTSISKKRIRKKNWKRKGYWTSLKAFSLGKSLSTGNSKSFFVQQNK
>A4QKD0 | CLPP_BARVE
MPIGVPKVPFRSPGEGDTSWVDIYNRLYRERLFFLGQEVDTEISNQLISLMIYLSIEKDT
KDLYLFINSPGGWVISGMAIYDTMQFVRPDVQTICMGLAASIASFILVGGAITKRIAFPH
ARVMIHQPASSFYEAQTGEFILEAEELLKLRETITRVYVQRTGKPIWVVSEDMERDVFMS
ATEAQAHGIVDLVAVQ
>A4QKD7 | RPOA_BARVE
MVREKVKVSTRTLQWKCVESRRDSKRLYYGRFILSPLMKGQADTIGIAMRRALLGEIEGT
CITRAKSENIPHDYSNIVGIQESVHEILMNLNEIVLRSNLYGTRNALICVQGPGYITARD
IILPPSVEIIDNTQHIATLTEPIDLCIGLKIERNRGYSLKMSNNFEDRSYPIDAVFMPVQ
NANHSIHSYGNGNEKQEILFLEIWTNGSLTPKEALHEASRNLINLFIPFLHVEEETFYLE
NNQHQVTLPLFPFHNRLVNLRKKKKEQGFQYIFIDQLELPPRIYNCLKKSNIHTLLDLLN
NSQEDLIKIEHFHIEDVKKILDILEKK
>A4QKD8 | RR11_BARVE

MAKPILRIGSRKNTRSGSRKNVRRIPKGVIHVQASFNNTIVTVTDVRGRVISWSSAGTCG
FKGTRRGTPFAAQTAAGNAIRAVVDQGMQRAEVRIKGPGLGRDAALRAIRRSGILLSFVR
DVTPMPHNGCRPPKKRRV
>A4QKF5 | RK32_BARVE
MAVPKKRTSISKKRIRKKIWKRKGYWTSLKAFSLGKSLSTGNSKSFFVQQNK
>A4QKJ4 | RR4_CAPBU
MSRYRGPRFKKIRRLGALPGLTSKRPKAGSDLRNQSRSVKKSQYRIRLEEKQKLRFHYGL
TERQLLKYVRIAGKAKGSTGQVLLQLLEMRLDNILFRLGMALTIPQARQLVNHGHILVNG
RIVDIPSYRCKPRDIITVKDEQNSRTLVQNLLDSSAPEELPNHLTLHTFQYEGLVNQIID
RKCVGLKINELLVVEYYSRQT
>A4QKK0 | RBL_CAPBU
MSPQTETKASVGFKAGVKEYKLTYYTPEYETKDTDILAAFRVTPQPGVPPEEAGAAVAAE
SSTGTWTTVWTDGLTSLDRYKGRCYHIEPVPGEETQFIAYVAYPLDLFEEGSVTNMFTSI
VGNVFGFKALAALRLEDLRIPPAYTKTFQGPPHGIQVERDKLNKYGRPLLGCTIKPKLGL
SAKNYGRAVYECLRGGLDFTKDDENVNSQPFMRWRDRFLFCAEAIYKSQAETGEIKGHYL
NATAGTCEEMIKRAVFARELGVPIVMHDYLTGGFTANTSLSHYCRDNGLLLHIHRAMHAV
IDRQKNHGMHFRVLAKALRLSGGDHIHAGTVVGKLEGDRESTLGFVDLLRDDYVEKDRSR
GIFFTQDWVSLPGVLPVASGGIHVWHMPALTEIFGDDSVLQFGGGTLGHPWGNAPGAVAN
RVALEACVQARNEGRDLAVEGNEIIREACKWSPELAAACEVWKEIRFNFPTIDKLDGQE
>A4QKL7 | CLPP_CAPBU
MPIGVPKVPFRSPGEGDTSWVDIYNRLYRERLFFLGQEVDTEISNQLISLMIYLSIEKDT
KDLYLFINSPGGWVISGMAIYDTMQFVRPDVQTICMGLAASIASFILVGGEITKRIAFPH
ARVMIHQPASSFYEAQTGEFILEAEELLKLRETITRVYVQRTGKPIWVVSEDMERDVFMS
ATEAQAHGIVDLVAVQ
>A4QKM5 | RR11_CAPBU
MAKPILRIGSRKNTRSGSRKNVRRIPKGVIHVQASFNNTIVTVTDVRGRVISWSSAGTCG
FRGTRRGTPFAAQTAAGNAIRAVVDQGMQRAEVRIKGPGLGRDAALRAIRRSGILLSFVR
DVTPMPHNGCRPPKKRRV
>A4QKN0 | RR3_CAPBU
MGQKINPLGFRLGTTQSHHSLWFAQPKKYSEGLEEDKKIRDCIKNYVQKNIRISSGMEGI
ARIEIQKRIDLIQIIIYMGFPKLLIEDKPRRVEELQMNVQKELNCVNRKLNIAITRISNP
YGDPNILAEFIAGQLKNRVSFRKAMKKAIELTEQANTKGIQVQIAGRIDGKEIARVEWIR
EGRVPLQTIEAKIDYCSYTVRTIYGVLGIKIWIFVDEE
>A4QKN3 | RK2_CAPBU
MAIYLYKTSTPSTRNGAVDSQVKSNPRNNLIYGQHHCGKGRNARGIITARHRGGGHKRLY
RKIDFRRNANDIYGRIVTIEYDPNRNAYICLIHYGDGEKRYILHPRGAIIGDTIVSGTEV
PIKMGNALPLTDMPLGTAIHNIEITLGKGGQLARAAGAVAKLIAKEGKSATLKLPSGEVR
LISKNCSATVGQVGNVGVNQKSLGRAGSKRWLGKRPVVRGVVMNPVDHPHGGGEGRAPIG
RKKPVTPWGYPALGRRTRKRKKYSETLILRRRSK
>A4QKP3 | RK32_CAPBU
MAVPKKRTSISKKRIRKNIWKRKGYWTSLKAFSLGKSLSTGNSKSFFVQQNK
>A4QKT3 | RR4_CRUWA
MSRYRGPRFKKIRRLGALPGLTSKRPKAGSDLRNQSRSVKKSQYRIRLEEKQKLRFHYGL
TERQLLKYVRIAGKAKGSTGQVLLQLLEMRLDNILFRLGMALTIPQARQLVNHGHILVNG

RIVDIPSYRCKPRDIITVKDEQNSRTLVQNLIDSSAPEELPNHLTLHTFQYEGLVNQIID
RKCVGLKINELLVVEYYSRQT
>A4QKT9 | RBL_CRUWA
MSPQTETKASVGFKAGVKEYKLTYYTPEYETKDTDILAAFRVTPQPGVPPEEAGAAVAAE
SSTGTWTTVWTDGLTSLDRYKGRCYHIEPVPGEETQFIAYVAYPLDLFEEGSVTNMFTSI
VGNVFGFKALAALRLEDLRIPPAYTKTFQGPPHGIQVERDKLNKYGRPLLGCTIKPKLGL
SAKNYGRAVYECLRGGLDFTKDDENVNSQPFMRWRDRFLFCAEAIYKSQAETGEIKGHYL
NATAGTCEEMIKRAVFARELGVPIVMHDYLTGGFTANTSLAHYCRDNGLLLHIHRAMHAV
IDRQKNHGMHFRVLAKALRLSGGDHIHAGTVVGKLEGDRESTLGFVDLLRDDYVEKDRSR
GIFFTQDWVSLPGVLPVASGGIHVWHMPALTEIFGDDSVLQFGGGTLGHPWGNAPGAVAN
RVALEACVQARNEGRDLAVEGNEIIREACKWSPELAAACEVWKEIRFNFPTIDKLDGQE
>A4QKV5 | RR12_CRUWA
MPTIKQLIRNTRQPIRNVTKSPALRGCPQRRGTCTRVYTITPKKPNSALRKVARVRLTSG
FEITAYIPGIGHNLQEHSVVLVRGGRVKDLPGVRYHIVRGTLDAVGVKDRQQGRSKYGVK
KPK
>A4QKW3 | RPOA_CRUWA
MVREKVKVSTRTLQWKCVESRRDSKRLYYGRFILSPLMKGQADTIGIAMRRALLGEMEGT
CITRVKSENIPHDYSNIVGIQESVHEILMNLNEIVLRSNLYGTRNALICVQGPGYITARD
IILPPSVEIVDNTQHIATLTEPIDLCIGLKIERNRGYSLKISNNLEDRSYPIDAVFMPVQ
NANHSIHSYGNGNEKQEILFLEIWTNGSLTPKEALHEASRNLINLFIPFLHVEEETFYLE
NNQHQVTLPFFPFHNRFFNLRKKKKELAFQYIFIDQLELPPRIYNCLKKSNIHTLLDLLN
NSQEDLIKIEHFHVEDVKKILDILEKK
>A4QL21 | RR4_DRANE
MSRYRGPRFKKIRRLGALPGLTSKRPRAGSDPRNQSRSGKKSQYRIRLEEKQKLRFHYGL
TERQLLKYVRIAGKAKGSTGQVLLQLLEMRLDNTLFRLGMALTIPQARQLVNHGHILVNG
RIVDIPSYRCKPRDIITVKDEQNSRTLVQNLLDSSTPEELPNHLTLHTFQYEGLVNQIID
RKCVGLKINELLVVEYYSRQT
>A4QL44 | CLPP_DRANE
MPIGVPKVPFRSPGEGDTSWVDIYNRLYRERLFFLGQEVDTEISNQLISLMIYLSIEKDT
KDLYLFINSPGGWVISGMAIYDTMQFVRPDVQTICMGLAASIASFILVGGAITKRIAFPH
ARVMIHQPASSFYEAQTGEFILEAEELIKIRETITRVYVQRTGKPIWVVSEDMKRDVFMS
ATEAQAHGNVDLVTVQ
>A4QL57 | RR3_DRANE
MGQKINPLGFRLGTTQSHHSLWFAQPKKYSEGLEEDKKIRDCIKNYVQNNIRISSGMEGI
ARIEIQKRIDLIQIIIYMGFPKLLIEDKPRRIEELQINVQKELNCVNRKLNIAITRISNP
YGHPNILAEFIAGQLKNRVSFRKAMKKAIELTEQANTKGIQVQIAGRIDGKEIARVEWIR
EGRVPLQTIDAKIDYCSYTVRTIYGVLGIKIWIFVDEE
>A4QLB4 | RBL_LEPVR
MSPQTETKASVGFKAGVKEYKLTYYTPEYETKDTDILAAFRVTPQPGVPPEEAGAAVAAE
SSTGTWTTVWTDGLTSLDRYKGRCYHIEPVPGEETQFIAYVAYPLDLFEEGSVTNMFTSI
VGNVFGFKALAALRLEDLRIPPAYTKTFQGPPHGIQVERDKLNKYGRPLLGCTIKPKLGL
SAKNYGRAVYECLRGGLDFTKDDENVNSQPFMRWRDRFLFCAEAIYKSQAETGEIKGHYL
NATAGTCEEMIKRAVFARELGVPIVMHDYLTGGFTANTSLAHYCRDNGLLLHIHRAMHAV
IDRQKNHGMHFRVLAKALRLSGGDHIHAGTVVGKLEGDRESTLGFVDLLRDDYVEKDRSR

GIFFTQDWVSLPGVLPVASGGIHVWHMPALTEIFGDDSVLHFGGGTLGHPWGNAPGAVAN
RVALEACVQARNEGRDLAVEGNEIVREACKWSPELAAACEVWKEIRFNFPTIDKLDGQ
>A4QLD0 | RR12_LEPVR
MPTIKQLIRNTRQPIRNVTKSPALRGCPQRRGTCTRVYTITPKKPNSALRKVARVRLTSG
FEITAYIPGIGHNLQEHSVVLVRGGRVKDLPGVRYHIVRGTLDAVGVKDRQQGRSKYGVK
KPK
>A4QLD1 | CLPP_LEPVR
MPIGVPKVPFRSPGEGDTSWVDIYNRLYRERLFFLGQDVDTEISNQLISLMIYLSIEKDT
KDLYLFINSPGGWVISGMAIYDTMQFVRPDVQTICMGLAASIASFILVGGAITKRIAFPH
ARVMIHQPASSFYEAQTGEFILEAEELLKLRETITRVYVQRTGKPIWVVSEDMERDVFMS
ATEAQAHGIVDLVAVQ
>A4QLD9 | RR11_LEPVR
MAKPILRIGSRKNTRSGSRKNVRRIPKGVIHVQASFNNTIVTVTDVRGRVISWSSAGTCG
FRGTRRGTPFAAQTAAGNAIRAVVDQGMQRAEVRIKGPGLGRDAALRAIRRSGILLSFVR
DVTPMPHNGCRPPKKRRV
>A4QLE1 | RR8_LEPVR
MGKDTIADIITSIRNADMNRKGTVRIRSTNITESIVKILLREGFIENVRKHRENNQYFLI
LTLRHKRNKKESYKTILNLKRISRPGLRIYSNSQRIPRILGGIGIVILSTSQGIMTDREA
RRKSIGGEILCYIW
>A4QLE4 | RR3_LEPVR
MGQKINPLGFRLGTTQSHHSLWFAQPKKYSEGLEEDKKIRDCIKKYVQKNIRISSGMEGI
ARIEIQKRIDLIQIIIYMGFPKLLIEDKPRRVEELQMNVQKELNCVNRKLNIAITRISNP
YGDPNILAEFIAGQLKNRVSFRKAMKKAIELTEQANTKGIQVQIAGRIDGKEIARVEWIR
EGRVPLQTIEAKIDYCSYTVRTIYGVLGIKIWIFVDEE
>A4QLF7 | RK32_LEPVR
MAVPKKRTSISKKRIRKKIWKRKGYWTSLKAFSLGKSLSTGNSKSFFVQQNK
>A4QLI6 | RPOB_LOBMA
MLGDGKEGTSTIPGFNQIQFEGFYRFIDQGLIEELSKFPKIEDIDHEIEFQLFVETYQLV
EPLIKERDAVYESLTYSSELYVSAGLIWKTSRNMQEQRIFIGNIPLMNSLGTSIVNGIYR
IVINQILQSPGIYYQSELDHNGISVYTGTIISDWGGRLELEIDKKARIWARVSRKQKISI
LVLSSAMGSNLREILENVCYPEIFLSFLTDKEKKKIGSKENAILEFYKQFSCVGGDPIFS
ESLCKELQKKFFHQRCELGRIGRRNINWRLNLNIPQNNIFLLPRDILAAADHLIGMKFGM
GTLDDMNHLKNKRIRSVADLLQDQLGLALARLENVVKGTIGGAIRHKLIPTPQNLVTSTP
LTTTYESFFGLHPLSQVLDRTNPLTQIVHGRKLSYLGPGGLTGRTANFRIRDIHPSHYGR
ICPIDTSEGINVGLIGSLSIHARIGDWGSLESPFYELFEKSKKARIRMLFLSPSQDEYYM
IAAGNSLALNRGIQEEQAVPARYRQEFLTIAWEEVHLRSILPFQYFSIGASLIPFIEHND
ANRALMSSNMQRQAVPLSRSEKCIVGTGLERQVALDSGVPAIAEHEGKILYTDTEKIILS
GNGDTFSIPLIMYQRSNKNTCMHQKPQVRRGKYIKKGQILADGAATVGGELSLGKNILVA
YMPWEGYNFEDAVLISESLVYDDIYTSFHIRKYEIQTHVTTQGPERITKEIPHLEGRLLR
NLDKNGIVMLGSWVETGDILVGKLTPQVAKESSYAPEDRLLRAILGIQVSTSKETCLKLP
IGGRGRVIDVRWVQKKGGSSYNPEKIRVYISQKREIKVGDKVAGRHGNKGIISKILPRQD
MPYLQDGRPVDMVFNPLGVPSRMNVGQIFECSLGLAGSLLDRHYRIAPFDERYEQEASRK
LVFSELYQASKQTANPWVFEPEYPGKSRIFDGRTGDPFEQPVIIGKPYILKLIHQVDDKI
HGRSSGHYALVTQQPLRGRSKQGGQRVGEMEVWALEGFGVAHILQEMLTYKSDHIRARQE

VLATTIIGGTIPKPEDAPESFRLLVRELRSLALELNHFLVSEKNFQINRKAV
>A4QLJ6 | RR4_LOBMA
MSRYRGPRFKKIRRLGALPGLTSKRPRAGSDLRNQSRSGKKSQYRIRLEEKQKLRFHYGL
TERQLLKYVRIAGKAKGSTGQVLLQLLEMRLDNILFRLGMALTIPQARQLVNHGHILVNG
RIVDIPSYRCKPRDIITVKDEQNSRTLVQNLLDSSAPEELPNHLTLHTFQYEGLVNQIID
RKCVGLKINELLVVEYYSRQT
>A4QLK2 | RBL_LOBMA
MSPQTETKASVGFKAGVKEYKLTYYTPEYETKDTDILAAFRVTPQPGVPPEEAGAAVAAE
SSTGTWTTVWTDGLTSLDRYKGRCYHIEPVPGEETQFIAYVAYPLDLFEEGSVTNMFTSI
VGNVFGFKALAALRLEDLRIPPAYTKTFQGPPHGIQVERDKLNKYGRPLLGCTIKPKLGL
SAKNYGRAVYECLRGGLDFTKDDENVNSQPFMRWRDRFLFCAEAIYKSQAETGEIKGHYL
NATAGTCEEMIKRAVFARELGVPIVMHDYLTGGFTANTSLAHYCRDNGLLLHIHRAMHAV
IDRQKNHGMHFRVLAKALRLSGGDHIHAGTVVGKLEGDRESTLGFVDLLRDDYIEKDRSR
GIFFTQDWVSLPGVLPVASGGIHVWHMPALTEIFGDDSVLQFGGGTLGHPWGNAPGAVAN
RVALEACVQARNEGRDLAVEGNEIVREACKWSPELAAACEVWKEIRFNFPTIDKLDGQE
>A4QLL8 | RR12_LOBMA
MPTIKQLIRNTRQPIRNVTKSPALRGCPQRRGTCTRVYTITPKKPNSALRKVARVRLTSG
FEITAYIPGIGHNLQEHSVVLVRGGRVKDLPGVRYHIVRGTLDAVGVKDRQQGRSKYGVK
KPK
>A4QLM6 | RPOA_LOBMA
MVREKVKVSTRTLQWKCVESRKDSKRLYYGRFILSPLMKGQADTIGIAMRRALLGEIEGT
CITRAKSENIPHDYSNIVGIQESVHEILMNLNEIVLRSNLYGTRNALICVQGPGYITAQD
IILPPSVEIIDNTQHIATLTEPIDLCIELKIERNRGYSLKMSNNFEDRSYPIDAVFMPVQ
NANHSIHSYGNGNEKQEILFLEIWTNGSLTPKEALHEASRNLINLFIPFLHVEEETFYLE
NNQHQVTLPLFPFHNRLVNLRKKKKELAFQYIFIDQLELPPRIYNCLKKSNIHTLLDLLN
NSQEDLIKIEHFHMEDVKKILDILEKK
>A4QLM9 | RR8_LOBMA
MGKDTIADIITSIRNADMNRKGTVRIGSTNITESIVKILLREGFIENVRKHRENNQYFLI
LTLRHRRNKKESHKTILNLKRISRPGLRIYSNSQRIPRILGGIGIVILSTSQGIMTDREA
RLKRIGGEILCYIW
>A4QLN2 | RR3_LOBMA
MGQKINPLGFRLGTTQSHRSLWFAQPKKYSEGLEEDKKIRDCIKNYVQKNIRISSGMEGI
ARIEIQKRIDLIQIIIYMGFPKLLIEDKPRRVEELQMTVQKELNCVNRKLNIAITRISNP
YGHPNILAEFIAGQLKNRVSFRKAMKKAIELTEQANTKGIQVQIAGRIDGKEIARVEWIR
EGRVPLQTIEAKIDYCSYTVRTIYGVLGIKIWIFVDEE
>A4QLP5 | RK32_LOBMA
MAVPKKRTSISKKRIRKKIWKRKGYWTSLKAFSLGKSLSTGNSKSFFVQQKN
>A4QLS5 | RPOB_NASOF
MLGDGKEGTSTIPGFNQIQFEGFYRFIDQGLIEELSKFPKIEDIDHEIEFQLFMETYQLV
EPLIKERDAVYESLTYSSELYVSAGLIWKTSRNMQEQRIFIGNIPLMNSLGTSIVNGIYR
IVINQILQSPGIYYQSELDHNGISVYTGTIISDWGGRLELEIDKKARIWARVSRKQKISI
LVLSSAMGSNLREILENVCYPEIFLSFLTDKEKKKIGSKENAILEFYQQFSCVGGDPIFS
ESLCKELQKKFFHQRCELGRIGRRNINWRLNLNIPQNNIFLLPRDILAAADHLIGMKFGM
GTLDDMNHLKNKRIRSVADLLQDQLGLALSRLENVVKGTISGAIRHKLIPTPQNLVTSTP

LTTTYESFFGLHPLSQVLDRTNPLTQIVHGRKLSYLGPGGLTGRTANFRIRDIHPSHYGR
ICPIDTSEGINVGLIGSLSIHARIGDWGSLESPFYELFEKSKKARIRMLFLSPSQDEYFM
IAAGNSLALNRGIQEEQAVPARYRQEFLTIAWEEVHLRSIFPFQYFSIGASLIPFIEHND
ANRALMSSNMQRQAVPLSRSEKCIVGTGLERQVALDSGVPAIAEHEGKILYTDTEKIVFS
GNGDTLSIPLIMYERSNKNTCMHQKPQVRRGKCIKKGQILADGAATVGGELALGKNILVA
YMPWEGYNFEDAVLISECLVYGDIYTSFHIRKYEIQTHVTTQGPERITKEIPHLEGRLLR
HLDKNGIVMLGSWVETGDILVGKLTPQVAKESSYAPEDRLLRAILGIQVSTSKETCLKLP
IGGRGRVIDVRWVQKKGGSSYNPEIIRVYISQKREIKVGDKVAGRHGNKGIISKILPRQD
MPYLQDGRPVDMVFNPLGVPSRMNVGQIFECSLGLAGSLLDRHYRIAPFDERYEQEASRK
LVFSELYKASKQTANPWVFEPEYPGKSRIFDGRTGDPFEQPVIIGKPYILKLIHQVDDKI
HGRSSGHYALVTQQPLRGRSKQGGQRVGEMEVWALEGFGVAHILQEMLTYKSDHIRARQE
VLGTTIIGGTIPKPEDAPESFRLLVRELRSLALELNHFLVSEKNFQINRKEV
>A4QLU1 | RBL_NASOF
MSPQTETKANVGFKAGVKEYKLTYYTPEYETKDTDILAAFRVTPQPGVPPEEAGAAVAAE
SSTGTWTTVWTDGLTSLDRYKGRCYHIEPVPGEETQFIAYVAYPLDLFEEGSVTNMFTSI
VGNVFGFKALAALRLEDLRIPPAYTKTFQGPPHGIQVERDKLNKYGRPLLGCTIKPKLGL
SAKNYGRAVYECLRGGLDFTKDDENVNSQPFMRWRDRFLFCAEAIYKSQAETGEIKGHYL
NATAGTCEEMIKRAVFARELGVPIVMHDYLTGGFTANTSLAHYCRDNGLLLHIHRAMHAV
IDRQKNHGMHFRVLAKALRLSGGDHVHAGTVVGKLEGDRESTLGFVDLLRDDYVEKDRSR
GIFFTQDWVSLPGVLPVASGGIHVWHMPALTEIFGDDSVLQFGGGTLGHPWGNAPGAVAN
RVALEACVQARNEGRDLAIEGNTIIREACKWSPELAAACEVWKEIRFNFPTVDTLDTQE
>A4QLV7 | RR12_NASOF
MPTIKQLIRNTRQPIRNVTKSPALRGCPQRRGTCTRVYTITPKKPNSALRKVARVRLTSG
FEITAYIPGIGHNLQEHSVVLVRGGRVKDLPGVRYHIVRGTLDAVGVKDRQQGRSKYGVK
KPK
>A4QLV8 | CLPP_NASOF
MPIGVPKVPFRSPGEGDTSWVDIYNRLYRERLFFLGQEVDTEISNQLISLMIYLSIEKDT
KDLYLFINSPGGWVISGMAVYDTMQFVRPDVQTICMGLAASIASFILVGGAITKRIAFPH
ARVMIHQPASSFYEAQTGEFILEAEELLKVRETITRVYVQRTGKPIWVVSEDMERDVFMS
ATEAQAHGIVDLVAVQ
>A4QLW6 | RR11_NASOF
MAKPILRIGSRKNTRSSSRKNVRRIPKGVIHVQASFNNTIVTVTDVRGRVISWSSAGTCG
FRGTRRGTPFAAQTAAGNAIRAVVDQGMQRAEVRIKGPGLGRDAALRAIRRSGILLSFVR
DVTPMPHNGCRPPKKRRV
>A6BM50 | RPOB_GNEPA
MQFDEKKEMFTIPEFGQIQLEGFCRFIEYDLLDKFVKFPKIANTQKEVEFLFDKNYKIIE
PSIKEKDAVYQRLTFSSKLFVPAFFIYWNKIKKKKIIYLGEIPLMNNNGTFLINGNYRVV
VNQLIRSPGIYYSLERKRTGNIYTSTLISDCGGRLKFEIDIKQNIWIRISRKKNVSILVF
FFAMGLDIEEILKNTYYIKGFEGWGLICNEKLKHKLSRKKGAIFTFYEELGSRGDNNDFV
FSESLSESLYKKFTNFLSKRCKLGRIGRRNLNKKLNLEIPDNEIFLLPQDVLAIIDYLIK
VSYGVGTVDNIDHLQNRRFFSVADLLKKEVGLALNRVKVLIQKTMQTIEMLRKKQNKRIM
LPEIPLVSTQITKTLKHFFGLHPLSQFLEQTNSLAEILHARKVSFLGPGGLTERTANFRA
RDIHPSYYGRFCPINTPEGQNAGLIASLAISARINFGFLESPFYNVAKKYQKAKKIVYLS
PSEDVYYRIALGNCLSVDQKIPEKKNTPTQYHQEFLSIAWEQIHFRCFLPLQYFSIGVSL

IPFLEHNDATRALMGSNMQRQAVPSVQPEKCIVGTGLEGQVALDSGALAISTQEGRIQYS
DAATIVSVLKGNTTQTELQIYQRSNSNTLMHQKTHASQAKYVRKGQILADGAAMLGGEIC
LGKNILVAYMPWQGYNFEDAILISECLIYKDIFTSFHITRYETTICTAECEKMTREIPRL
ATYSLRHLDKNGLVRVGSWVQPGDVLVGKLRPRSSEDFSRFPELRLLQDLFCTSPIKETC
LRANGKGRVIDVNWSKLQAFCKDDLEKGHQDDDTEDIYDEAEDALEKVYQLNNYNLSSDS
EKVHVYLLEKRKIQVGDKVAGRHGNKGIVSIVLSRQDMPFLQSGISLDMVLNPLSVPSRM
NVGQIFECLLGLAGTMNKHYRIPPFDERYEQEASRKLVFYELYKASEQTANPWIFELEHL
GKTQIFDGRTGEIFEQPVTTGNAYILKLIHQVNDKMHARSTGNYARITQQPVQGKSKGGG
QRLGEMEVWALESFGVAYVLREMLTVKADHIRARKKILRSILDGHSVPKADSATESFRVL
SKELNSLALELNHTIISGKYFNLDRIEV
>A6H5H5 | RR4_CYCTA
MSRYRGPRLKIIRRLRTLPGLTNKRPKSRNDPTNQSSSRKISQYRIRPEEKQKLRFHYGL
TERQLLKYVRIAGRAKGSTGQILLQLLEMRLDNIIFRLGMALTIPGARQLVNHRHILVND
RVVDIPSYRCKPRDVITIIDQRRSRAMIGKNLDLSQKDQMPNHLTLHSSQYKGLVNQIID
SKWISLKINELLVVEYYSRQA
>A6H5K6 | CLPP_CYCTA
MPIGVPKVPFRIPGEEDNRLYRERLLFPGQQVDDEIANQLIGIMMYLNGEDENKDIYPYI
NSPGGAVIPGISIYDAMQFVVPDVHTICMGLAASMGSSVLTGGEITKRIALPHARVMIHQ
PASSYYDGQAGECIMEAEEVLKLRDSITNVYVQRTGKPLWVISGDMERDVFMSATEAQAY
GIVDLVAVENTEDLL
>A6H5L3 | RPOA_CYCTA
MIRDEISVSTQTPRWRCIGSGADSKRLHYGRFALSPLRKGQASTIGIAMRRALLGEIEGT
CITHAKLEKVTHEYSAIIGIEESVHDILINLKEIVLRSDPYGTREASICIVGPRNVTAQD
IILPPSVRIIDATQHIASLTKSITFDIRLWIEKDRGYRIQSPKNYQDGIFPIDAVFMPVR
NANYSIHSYGNGNDIQEILFLEIWTNGSLAPREALYRASRNLIDLFIPFLRAEEQNIDGM
DNQNGSNMPSFSFSNISADMERMEEEVAFKHIFIDQSELPPRVYNCLGRVNIHTLSDLLN
YSQEDLMRIGHFGKKSVEQVSEVLQKHFAVDLPKNKFQIH
>A6H5L7 | RR8_CYCTA
MGNDTIANIITSIRNADIVKKKTVRIIATNTTKNVVRILLQEGFIEDAREHREGQKSFSV
LTLRYRGRKEKTYITTSKRTSKPGLRIYSNSQKVPKVLGGMGVVILSTSQGIMTDREARR
RRIGGEILCYVR
>A6H5M3 | RK2_CYCTA
MAIRSYRTYTPSTRNRPISSYDGRVRSNPQKKLTSGQHRCGKGRNSRGIITARHRGGGHK
RLYRQIDFQRNEKYIFGEIVTIEYDPNRSAYICLVHYGDGEKKYILHPRGVIIGDTITSG
PRAPISIGNALPLTDVPLGTAIHSIEITLGKGGQLARAAGAVAELIAKEGRSTTLRLPSG
EIRLISENCSATIGQVGNVNANNGTLGKAGSKRWLGKRPRVRGVVMNPVDHPHGGGEGRT
PIGRKKPVTPWGYAALGRKSRKNNKYSDASILRRRK
>A6H5Q1 | CHLN_CYCTA
MSAKISETLTFECETGNYHTFCPISCVSWLYQKIEDSFFLMVGTKTCGYFLQNTLGVMIF
AEPRYAMAELEEGDISAQLNDYEELKRLCIRIKKDRDPNVIIWIGTCTTEIIKMDLEGMA
PKLESEIGIPIIVARANGLDHAFTQGEDTVLAAMAHRCLEQRLFVRERNGTIQKFPPPLE
KEGEFIEYGDHPSLALFGSLPSNVASQLSPELRRQSVKVSGWLPAQRYTHLPSLGNGVYV
CGINPFLSRTAATLVRRERCRLIGAPFPIGPDGTRAWIEKICPVFDIETQGLEEREKQIW
ESLKDYISLVHGKSVFFMGDNLLEISLARFLIRCGMIVYEIGIPYMDKRYQAAELALLRD

TCIKMCVPIPRIVEKPDNYNQLRRIRELQPDLAITGMAHADPLEARGMNTKWSVEFTFAQ
IHGFANARNVLELVTRPLRCNDNLEDLGRTTLVK
>A6MM38 | RR4_BUXMI
MSRYRGPRFKKIRRLGALPGLTSKRPRAGSDLRNQSRSGKRSQYRIRLEEKQKLRFHYGL
TERQLLKYVRIAGKAKGSTGQVLLQLLEMRLDNILFRLGMASTIPGARQLVNHRHILVNG
RIVDIPSYRCKPRDIITAKDEQKSRALIQNYLDSSPHEELPKHLTLHSFQYKGLVNQIID
SKWVGLKINELLVVEYYSRQT
>A6MM69 | RR11_BUXMI
MAKPIPRIGSRKNGRIGSRKSGRRIPKGVIHVQASFNNTIVTVTDVRGRVVSWSSAGTCG
FRGTRRGTPFAAQTAAGNAIRTVMDQGMQRAEVMIKGPGLGRDAALRAIRRSGLLLSFVR
DVTPMPHNGCRPPKKRRV
>A6MM72 | RR8_BUXMI
MGRDTIANIITSIRNADMDKKGTVRIASTNITENIVKILLREGFIENVRKHRESNKYFLV
STLRHRRSRKGPYRTILKRISRPGLRIYSNYQRIPRILGGMGIVILSTSRGIMTDREARL
EGIGGEILCYIW
>A6MMF4 | RPOA_CHLSC
MVREEIAVSTRTLQWKCVESRADSKRLYYGRFMLSPLMKGQADTIGIAMRRALLGEIEGT
CITRAKLDKVPHEYSTIVGIEESIHEIFMNLKEIVLRSNLYGTRNASICVRGPRYVTAQD
IISPPSVEIVDTTQHIASLTEPIDLCIELQIQRDRGYRMKTPNKYQNGSYPIDAVSMPVR
NANHSIHSYGNGNEKQEILFLEIWTNGSLTPKEALHEASRNLIDLFIPFLHAEEEGIRLE
DNPNRFNIPFFTFHDGLANTNIRKKKKGIALKCIFIDQSELPPRTYNYLKRSNINTLLDL
LSNSQEDFLKIEHFRIEDVKQILDILQKHFTIDC
>A6MMF8 | RR8_CHLSC
MGKDTIADIITSIRNADMEKKGTVRIASTNINISENIVQILLREGFIENVRKHRENNKYF
LVSTLRHRRNRKGAYRNILKRISRPGLRIYSNYQRIPRILGGMGIVILSTSRGIMTDREA
RLEGIGGEILCYIW
>A6MMG1 | RR3_CHLSC
MGQKINPLGFRLGATQGHHSLWFAQPKSYSRGLQEDEKIKDCIKNYVQKNMRISSDYEGI
AHIEIKKRIDLIQVIIYMGSKNSLMLIEGPTRGIEELQINVQKEFHSANQRLNIDITEVA
NPYGQPNLLAEYIARQLKKRVSFRKAMKKTIEFAEQADTKGIQVQISGRIDGKEIARVEW
VREGRVPLQTIRAKIDYCSYTVRTIHGVLGIKIWVFVDDK
>A6MMI6 | RK2B_CHLSC
MAIHLYKTSTPSTRNGAVDSQVKSNPRNNLIYGQHHCGKGRNARGIITAGHRGGGHKRLY
RKIDFRRNEKDISGRIVTIEYDPNRNAYICLIHYGDGEKRYILHPRGAIIGDTIVSGTEV
PISMGNALPLTDMPLGTAIHNIEITLGKGGQLARAAGAVAKLIAKEGKSATLRLPSGEVR
LISKNCSATVGQVGNVGVNQKSLGRAGSKCWLGKRPVVRGVVMNPVDHPHGGGEGRSPIG
RKKPTTPWGYPALGRRSRKRNKYSDSLILRRRK
>A6MMJ9 | RPOB_DIOEL
MIRDGNEGMYTIPGFNQIQFEGFCRFINQGLMEELHKFPKIEDTDQEIEFQLFVETYQLV
EPLIKERDAVYESLTYSSELYVPAGLIWKTSRAMQEQTIFIGNIPLMNSLGTSIINGIYR
IVINQILQNPGVYYRSELDHNGISVYTSTIISDWGGRLELEIDRKARIWARVSRKQKISI
LVLLSAMGSNLREILDNVCYPEIFLSFPNDKEKKKIGSKENAILEFYQQFACVGGDPVFS
ESLCKELQKKFFQQRCELGRIGRRNTNQRLNLDIPQKNTFLLPRDVLAAADHLIRMKFGM
GTLDDMNHLKNKRIRSVADLLQDQFGLALVRLENGVRGTICGAIRHKLIPNPKNLVTSTS

FTTTYESFFGLHPLSQVLDRTNPLTQIAHGRRLSYLGPGGLTGRTASFRIRDIHPSHYGR
ICPIDTSEGINVGLIGSLAIHVRIGHWGSIESPFYEISERSKKAQMIYLSPSRDEYYMVA
AGNSLALNRGIQEEQVVPARYRQEFLTIAWEQIHLRSIFPFQYFSIGASLIPFIEHNDAN
RALMSSNMQRQAVPLSRSEKCIVGTGLERQTALDSGVSAIAEREGKIIYTDTHKIIFSSN
GDTMSIPLVMYQRSNKNTCMHQKPQVPRGKCIKKGQILADGATTVGGELALGKNVLVAHM
PWEGYNSEDAVLISERLVYEDIYTSFYIRKYEIQTHVTSQGPERITKEIPHLEDHLLRNL
DRNGIVMLGSWIETGDILVGKLTPQTATESSYAPEDRLLRAILGIQVSTAKETSLKLSIG
GRGRVIDVRWIQKRGGSIYNPEMIRVYISQKREIKVGDKVAGRHGNKGIISKILPRQDMP
YLQDGTPVDMVFNPLGVPSRMNVGQIFECSLGLAGDLLKRHYRIAPFDERYEQEASRKLV
FSELYSASKQTKNPWVFEPEYPGKSRIFDGRTGDPFEQPVLIGKPYILKLIHQVDDKIHG
RSSGHYALVTQQPLRGRAKQGGQRVGEMEVWALEGFGVAHILQEMLTYKSDHIRARQEVL
GATIVGGTVPNPEDAPESFRLLVRELRSLALELNHFLVSEKNFQINRKEA
>A6MMK9 | RR4_DIOEL
MSRYRGPRFKKIRRLGALPGLTSKRPRSGSDFKNQSRFGKKSQYRIRLEEKQKLRFHYGL
TEQQLLRYVHIAGKAKGSTDQVLLQLLEMRLDNILFRLGMASTIPAARQLVNHRHILVNG
RIVDIPSYRCKPRDIITSKDKQRSKVLIQNSIESSPHEELPKHLTIDSFQYKGLVNQIID
SKLIGLKINELLVVEYYSRQT
>A6MMN2 | CLPP_DIOEL
MPIGVPKVPFRSPGEEDAVWVDVNRLHRERLLFLGQEVDNEVSNQLVGLMVYLSIEDATK
DLYLFINSPGGWVIPGMAIYDTMQFVSPDVHTICMGLAASMGSLILVGGEITKRLAFPHA
RVMIHQPASSFYEAQAGEFILEAEELLKLRETLTKVYVQRTGNPLWAVSEDMERDAFMSA
TEAQAHGIVDLVAVENENTNDFV
>A6MMN9 | RPOA_DIOEL
MVREEIAESTRTLQWKCVESRVDSKRLYYGRFILSPLMKGQADTIGISMRRALLGEIEGT
CITRAKSEKVPHEYSTVVGIEESVHEILFNLKEIVLRSNLYEIRDASICVRGPRYVTAQD
IISPPYVEIVDTTQYIANLTEPIDLCIELEIKRDRGYRMKPITNSQDGSYPIDAVFMPVR
NANHSIHSYGNGNEKQEILFLEIWTNGSLTPKEALYEASRNLIDLFVPFLHAEEEDINFE
ENKNKFTLPPLTFQDRLSNLKKNKKGIPLKYIFIDQLELPSRTYNCLKRSNIHTLLDLLS
KGQENLIKMEYFHIEDVKQILDTLQKHFAVDLPKVLI
>A6MMP0 | RR11_DIOEL
MTKPIPRIGSRRNGRIGSRKNARRIPKGVIHVQASFNNTIVTVSDVRGQVISWASAGTSG
FKGTRRGTPYAAQAAAVNAIRTIIDQDMQRAEVMIKGAGFGRDAALRAIRRSGILLSFVR
DVTPMPHNGCRPPQKRRV
>A6MMS3 | RR12_ILLOL
MPTIKQLIRNTRQPIKNVTKSPALRGCPQRRGTCTRVTITPKKPNSALRKVARVRLTSGF
EITAYIPGIGHNSQEHSVVLVRGGRVKDLPGVRYHIVRGTLDAVGVKDRQQGRSKYGVKK
PK
>A6MMT6 | RPOB_ILLOL
MLRDGNEGMSTIPGSSQIQFEGFCKFIDQGLAEELHKFPKMEDTDQEMEFQLFVETYQLV
EPLIKERDAVYESLTYSSELYVSAGLIWRAGRDMRKQTVFIGNIPLMNSLGTSLVNGIYR
IVVNQILQSPGIYYRSELDHNGISVYTGTIISDWGGRSELEIDRKARIWARVSRKQKISI
LVPSSAMGSNLRDILDNVCYPEIFLSFPNDKEKKKIGSKENAVLEFYQQFACVGGDPVFS
ESLCKELQKKFFQQRCELGRIGRRNMNQRLNLDIPQNSTFLLPRDVLAAADHLIGMKFGM
GTLDDLNHLKNKRIRSVADLLQDQFGLALVRLENVVRGTICGAIRHKLIPTPHNLVTSTP

LTTTYESFFGLHPLSQVLDRTNPLTQIVHGRKWSYLGPGGLTGRTASFRIRDIHPSHYGR
ICPIDTSEGINVGLIGSLAIHARIGDWGSIESPFYEISERLKEEQMVYLSPRRDEYYMVA
AGNSLALNRGIQEEQVVPARYRQEFLTIAWEQIHLRSIYPFQYFSIGASLIPFIEHNDAN
RALMSSNMQRQAVPLSHSEKCIVGTGLERQAALDSGGSAIAEHEGKITYTDTEKIVLSGN
GDTISIPLVMYQRSNKNTCMHQKPQVHRGKCVKKGQILADGAAIIGGELALGKNILVAYM
PWEGYNSEDAVLISERLVYGDIYTSFHIRKYEIQTHVTSQGPERITNEIPHLEAHLLRNL
DKNGIVMLGSWVETGDILVGKLTPQTAKESSYAPEDRLLRAILGIQVSTAKETCLKLPIG
GRGRVIDVRWIQKKRGSSSNPETIRVYISQKREIKVGDKVAGRHGNKGIISKILPRQDMP
YLQDGTPVDMVFNPLGIPSRMNVGQMFECSIGLAGNLLNRHYRIIPFDERYEQEASRKLV
FPELYKASKQTANPWVFEPEYPGKSRIFDGRTGDPFEQPVIIGKSYMMKLIHQVDDKIHG
RSSGHYALVTQQPLRGRAKHGGQRVGEMEVWALEGFGVAHISQEMLTYKSDHIRARQEVL
GTTIIGGTIPNPDGAPESFRLLVRELRSLALELNHFLVSEKSFQINRKEA
>A6MMY0 | RR8_ILLOL
MGRDTIADIITSIRNADMGTKGMVRIASTNITENIVKILLREGFIENVRKHRESNKYFLV
STLRHRRNRKGTYRNILKRISRPGLRIYSNYQGIPRILGGMGVVILSTSRGIMTDREARL
EGIGGEILCYIW
>A6MMY3 | RR3_ILLOL
MGQKINPLGFRLGTTQSHHCLWFAQPKNYSGGLQEDEKIRDCIKNYVQKHMRVSSGFEGI
ARIGIQKKIDLIQVIIHIGFPHFLIEGRARGIEELQINVQKKLNSVNRRLNIAITRVAKP
YGQPTILAEYIALQLKNRVSFRKAMKKAIELTEQADTKGIQVQISGRINGKEIARVEWIR
EGRVPLQTIRAKIDHCSYAVRTIYGVLGIKIWIFLDEE
>A6YGA4 | CHLL_LEPTE
MKLAVYGKGGIGKSTTSCNISIALARRGKKVLQIGCDPKHDSTFTLTGFLIPTIIDTLQS
KDYHYEDVWPEDVIYQGYGGVDCVEAGGPPAGAGCGGYVVGETVKLLKELNAFYEYDVIL
FDVLGDVVCGGFAAPLNYADYCIIVTDNGFDALFAANRIVASVREKARTHPLRVAGLVGN
RTDARDLIDKYVEVCPMPVLEVLPLIEDIRISRVKGQTLFEIAETQTAVSYVCDYFLNIA
DQLLSQPEGVVPNELGDRELFSLLSDFYLNPTSNSEKNANISGLEPDSLDFLIV
>A6YGC4 | RK2_LEPTE
MGIRFYKPYTPGTRNRSMAEFDQITETKPEKHLTSWIPRSKGRNNRGIITSRHRGGGHKR
LYRNIDFKRNKLGILGQVATIEYDPNRNARIAKVHYQDGEKRYILYPRGLQLGQTILSNF
NAPITIGNSLPLHQIPLGTEIHNIELKPGSGGQLARAAGSVAQLVAKEGNFVTLRLPSGE
IRLVSKSCWATIGQVGNVEQMNLTVGKAGKSRWLGRRPKVRGVVMNPVDHPHGGGEGRAP
IGRSRPVTPWGRPALGQRTRNATKYSRNLIIRRRK
>A6YGD0 | RR8_LEPTE
MVNDTISDMLTRIRNANMAKNDLVLIPFTHINLEICQILLKEGFIQSFKTLTLDVSKYSK
LNAQKNLNIVVKLKYLGRQKNSCITNLLRISKPGNRIYAKHKIPDMLGGLGIIIVSTSAG
IMTDREAREQKIGGELLCSVW
>A6YGD3 | RR11_LEPTE
MAKLTRKIPKKTKRKFSRGLVHIQVSFNNTIVTITNLKGDVLAWSSAGACGFKGARKGTP
FAAQIVAETAARKSFDRGLKQAQVLVKGPGPGRDKALVGLFKAGIQISLIRDITAIPHNG
CRPPKKRRL
>A6YGD9 | RPOB1_LEPTE
MEFSVKNKNLLIQNLVEIQQQSFNELLCVGLKRQLLQLNFIENKTRNIKLIFHAKNYKFI
LPEITPKEAVLKSKTFASALFIPVEYRAKNSIGLYWVLLGHLPFMTKRGHFIINGVPRVV

LNQMVRSPGLYYKTTANPQNVSNQVSINDSSHTSPVFYSDIIPNRGTWLRLEIDRKKNIW
IRMKRVDKIPMMLFLQALGYDLHCIEKLLYSRKFLNCDYLLNTDHPADSNSALKKIKVEI
EQQKVLKLAALEALDDDDPDIAKQLDYIEKTTTPEMGKDFLFQSFFNPASYDLSPIGRFQ
LNKKLNLSIPSDYTVLTEFDIYFATKELIKRATTMSNSDDIDHLQTRRIRSCGELLEIQL
GEGIERLQKTIIDKINNTNLKKFAKHSSTYKRGTSSSIYRQDRQVINTDKNSKFLKNNLP
TKFNRITLEPNLLPNGTSFIHKNNKLNQVSSNKLKLLSKKLIYLNLLINQKFIIAKLKSA
NLLANKSSFDKFLIHLVDESPRQKFVKNKTGLYHKFIPTPEIYIVEPNLNLVDMKSQQKF
NLLKMDLYKVGLAESFLKSNLNNLNIYLQMCLRKPNTYFRDISKICSAQITQQLKQKSFL
KLLLINPRLISQNNLLVLNRAVNFLKKIQFNLKDSPNLNDYQNFEINKLKAHIFKCQIKT
QNNLTCCKYIYIKTKFNYINLPIKQFFFTKRFLITEQKLLLGGKFINSNLALTSLLKLHK
FSEVVSNSFLIYNANNPNPNPTSIMSMIRPVNKSEIYRQLNRRFVRSRLQLNVLKSELTN
SHSQLIKANNDPIDRELSIQILKQDVQKSNPFNKKGIYNQRFINKKFLDTSQFIDQELLH
FLKTHKIFLDRQLTSLKTKQQNEKLKFIKWKKLRNFKNALKNPIQNLITTNAINGALQEL
FGLNPLSQFMDQINPLSEITHKRRISSMGPGGVNRDNASMDVRSIHPTHYGRICPIETPE
GHNAGLVNSPTIYARINNYGFLETPFFKVNSGQIQAKAFYLNAQKEQKFKVSPPDLSCSE
LQFLPLGPTKIESEVPMRKDFKFQRMAATQIEFIGISPLQMISVATSLIPFLEHDDANRA
LMGSNMQRQAVPLLIAERPVVGTGLEGRVIADSSYIMQTKQSGVISYVSNEQVIVQTFHL
NN
>A7Y3B9 | RPOB_IPOPU
MEGGGMTTIPGFNQIQFEGFCRFIDQGLTEELSKFPKIEDIDQEIEFQLFVETYQLVEPL
IKERDAVYDSLTYSSELYVSARLIWKTSRDMQEQTIFIGSIPLMNSQGTSIVNGIYRIVI
NQILQSPGIYYRSELDHNGISVYTGTIISDWGGRSELEIDRKARIWARVSRKQKISILVL
SSAMGSNLREILENVCYPEIFLSFLSDKEKKKIGSKENAILEFYQQFACVDGDPIFSESL
WKELQKKFFQQRCELGRIGRRNMNRRLNLDIPQNNTFLLPRDLLAAADHLIGLKFGMGTL
DDMNHLQNKRIRSVADLLQDQFGLALVRLENAVRGTICGAIRHKLIPTPQNLVTSTPLTT
TYESFFGLHPLSQVLDRTNPLTQIVHGRKFSSLGPGGLTGRTASFRIRDIHPSHYGRICP
IDTSEGINVGLIGSLAIHARIGHWGSLESPFYEISERSTGVRMLYLSPGRDEYYMVAAGN
SLALNQDIQEDQVVPARYRQEFLTIAWEQVHLRSIFPFQYFSIGASLIPFIEHNDANRAL
MSSNMQRQAVPLSRSEKCIVGTGVERQAALDSGALVIAEREGRVVYTDTDKILFSGDGET
LSIPLVMYKRSNKNTCMHQKPQVQRGKCIKKGQILADGAATVEGELALGKNVLVAYMPWE
GYNSEDAVLISERLVYEDIYTSFHIKKYEIQTHVTSQGPEKVTNEIPHLEAHFIRNLDKN
GIVKQGSWVETGDVLVGKLTPQVVKESSYAPEDRLLRAILGIQVSTSKETCLKVPIGGRG
RVIDVRWIQKKGGSSYNPEMIRVYILQKREIKVGDKVAGRHGNKGIISKILPRQDMPYLQ
DGRSVDLVFNPLGVPSRMNLGQIFECSLGLAGSLLDRHYRIAPFDERYEQEASRKVVFSE
LYEASKQTANPWAFEPEYPGKSRIFDGRTGNPFEQPVLIGKPYILKLIHQVDDKIHGRSS
GHYALVTQQPLRGRAKQGGQRVGEMEVWALEGFGVAHILQEMLTYKSDHIRARQEVLGTT
IVGGTIPNPKDAPESFRLLVRELRSLALELNHFLVSEKNFQIHRKEA
>A7Y3I9 | RR3_IPOPU
MGQKINPLGFRLGTTQGHHSLWFSQPKNYSESLQEDQKIRNFIKNYVQKNMITSSGVTGI
ARIDIQKGIDLIKVIIFMGFPKLLIENRPRATEELQMTLQKELNCVNRKLNIAITRIAKP
YGNPKILAEFIAGQLKNRVSFRKAMKKAIELAEKADTKGIQVQIAGRIDGKEIARVEWIR
EGRVPRQTIRAKIDYCSYPVRTIYGVLGIKIWIFLDEA
>A8SEA5 | RR4_CERDE
MSRYRGPRFKKIRRLGALPGLTSKRPSPGSDLRNQSRSGKRSQYRIRLEEKQKLRFHYGL

TERQLLRYVRIAGKAKGSTGQVLLQLLEMRLDNILFRLGMASTIPGARQLVNHRHILVNG
RIVDIPSYRCKPQDIITTRDEQRSRALIQKSIDSSPHEEVPKHLTLYPFQYKALVNKIID
SQWIGLKINELLVVEYYSRQT
>A8SED5 | RPOA_CERDE
MVREEVTISTRTLQWKCVESRVDSKRLYYGRFILSPLMKGQADTIGISMRRALLGEIEGT
CITRAKFDKVTHEYSTIVGIEESVHEILMNLKEIVLRSNLYGTLDASICVRGPRSVTAQD
IISPPSVEIVDTTQHIANLTEPIDLCIGLQIERDRGYRLKTPNNYQDGSYPIDTVFMPVR
NANHSIHSYGNGTEKQEILFLEIWTNGSLTPKEALYEASRNLINLFIPFLHAEEQEILLE
DSENISTVPLFTFHNELANLKKNKKGIALKCIFIDQLELPSRTYNCLKRSNIHTLFDLLS
NSKEDLMRIEHFRVEDVKQILDILQKNFAMNLPKDFF
>A8SED6 | RR11_CERDE
MTKAIQKIGSRRNGRIASRKNGRRIPKGVIHVQASFNNTIVTVTDVRGQVVSWSSAGTCG
FRGTRRGTPFAAQTAAANAIRTVVDQGMQRAEVMIKGPGLGRDAALRAIRRSGILLSFVR
DVTPMPHNGCRPPKKRRV
>A8SEE2 | RR3_CERDE
MGQKINPLGFRLGATQSHRSFWFAQPKNYSDGLQEDEKIRDCIKNYVQKEMRISSGFEGI
ARIGIQKRIDLIQVIIYIGFPNLLIEGRTRGIEELQSNVQKEFNSGNRRLNVVITRVAKP
YGQPNILAEYIAGQLKKRVSFRKAMKKAIELTEQADTKGIQVQIAGRIDGKEIARVEWIR
EGRVPLQTIRAKIDHCSYMVRTIYGVLGIKIWIFVDED
>A8Y9B8 | RPOA_LOLPR
MVREEVAGSTQTLQWKCVESRVDSKRLYYGRFILSPLRKGQADTVGIALRRALLGEIEGT
CIARAKFGSVPHEYSTIAGIEESVQEILLNLKEIVLRSNLYGVRDASICVKGPRYITAQD
IILPPSVEIVDTSQPIANLTEPIDFCIELQIKRDRGYQTELRKNYQDGSYPIDAVSMPVR
NVNYSIFSCGNGNEKHEILFLEIWTNGSLTPKEALYEASRNLIDLFLPFLHAEEEGTSFE
ENKNRFTPPLFTFQKRLTNLNLKKNKKGIPLNCIFIDQLELPSRTYNCLKRANIHTLLDL
LSKTEEDLMRIDSFHMEDGKQIWDTLEKYLPIDLLKNKLSF
>A8Y9B9 | RR11_LOLPR
MAKAIPKIGSRKKVRIGLRRNARFSLRKSARRITKGVIHVQASFNNTIITVTDPQGRVVF
WSSAGTCGFKSSRKASPYAGQRTAVDAIRTVGLQRAEVMVKGAGSGRDAALRAIAKSGVR
LSCIRDVTPMPHNGCRPPKKRRL
>A8Y9C2 | RR8_LOLPR
MGKDTIADLLTSIRNADMNKKGTVRVISTNITENIVKILLREGFIESVRKHQERNRYFLV
STLRHQKRKTRKGIYRTRTFLKRISRPGLRIYTNYQGIPKVLGGMGIAILSTSRGIMTDR
EARLNRIGGEVLCYIW
>A8Y9C5 | RR3_LOLPR
MGQKINPLGFRLGTTQKHHSFWFAQPKNYSEGLQEDKKIRDCIKNYIQKNRKKSSNRKLE
SDSSSEVITHNRKNDSGSSSEVITRIEIQKEIDTIHVIIHIGFPNLLKKKGAIEELEKDL
QKEIHSVNQRLNISIEKVKEPYREPNILAEYIAFQLKNRVSFRKAMKKAMELTKKADIKG
VKVKIAGRLGGKEIARAECIKKGRLPLQTIRAKIDYCCYPIRTIYGVLGVKIWIFVDEE
>A8Y9G0 | RPOB_LOLPR
MLRNGNEGMSTIPGFSQIQFEGFCRFINQALAEELNKFPAIKDPDHEIAFQLFAKGYQLL
EPSIKERDAVYESLTYSSELYVSARLIFGFDVQKQTISIGNIPIMNSLGTFIINGIYRIV
INQILLSPGIYYRSELDHKGISIYTGTIISDWGGRSELAIDKKERIWARVSRKQKISILV
LSSAMGSNLREILDNVSYPEIFLSFPNAKEKKRIESKEKAILEFYQQFACVGGDLVFSES

LCEELQKKFFQQKCELGRIGRRNMNRRLNLDIPQNNTFLLPRDVLAATDHLIGMKFGTGI
LDDDDMNHLKNKRIRSVADLLQDQFGLALGRLQHAVQKTIRRVFIRQSKPTPQTLVTPTS
TSILLITTYETFFGTYPLSQVFDQTNPLTQTVHGRKVSCLGPGGLTGRTSSFRSRDIHPS
HYGRICPIDTSEGINVGLTGSLAIHARINHLWGSIESPFYEISAEKAKKKKSRQVVYLSP
NRDEYYMIAAGNSLSLNQGIQEEQVVPARYRQEFLTIAWEQIHVRSIFPFQYFSIGGSLI
PFIEHNDANRALMSSNMQRQAVPLSRSEKCIVGTGLERQTALDSGVSVIAEREGKILSTD
SHKILLSSSGKTLSIPLVNHRRSNKNNCMHQKSRVPRGKSIKKGQILAEGAATVGGELAL
GKNVLVAYMPWEGYNFEDAVLISERLVYEDIYTSFHIRKYEIQTDTTSQGSAEKITKEIP
HLEAHLLRNLDRNGVVRLGSWVETSDILVGKLTPQIASESSYIAEAGLLRAIFGLEVSTS
KETSLKLPIGGRGRVIDVKWIQRDPLDIMVRVYILQKREIKVGDKVAGRHGNKGIISKIL
PRQDMPYLQDGTPVDMVFNPLGVPSRMNVGQIFESSLGLAGDLLKKHYRIAPFDERYEQE
ASRKLVFSELYEASKETKNPWVFEPEYPGKSRIFDGRTGDPFEQPVLIGKSYILKLIHQV
DEKIHGRSTGPYSLVTQQPVRGRAKQGGQRVGEMEVWALEGFGVAHILQEILTYKSDHLI
ARQEILNATIWGKRIPNHEDPPESFRVLVRELRSLALELNHFLVSEKNFQVTREEV
>A9LYH7 | RPOB_ACOAM
MGFLFSINRKLKMPRDGNEGMFTIPGFSQIQFEGFCRFIDQGLMEEFHKFPKIEDTDQEI
EFQLFVERYQLVEPLIKERDAVYESLTYSSELYVPAGLIWKTGRDMQEQTIFIGNIPLMN
SLGTFIVNGIYRIVINQILQSPGIYYRSELDHNGISVYTSTIISDWGGRSELEIDRKSRI
WARVSRKQKISILVLSSAMGSNLREILDNVCYPEIFLSFLNDREKKKIGSKENAILEFYQ
QFACVGGDPVFSESLCKELQKKFFQQRCELGRIGRRNMNRRLNLDIPQSNTFLLPRDVLA
AADHLIGMKFGMGTLDDMNHLKNKRIRSVADLLQDQFGLALVRLENAVRGTICGAIRHKL
ILTPQNLVSSTSLTTTYESFFGLHPLSQVLDRTNPLTQIVHGRKLSYLGPGGLTGRTASF
RIRDIHPSHYGRICPIDTSEGINVGLIGSLAIHARIGHWGSIESPFYEVYQRSKETKMVF
LSPSRDEYYTVATGNSLALNRGGIQEEQIVPARYRQEFLTIAWEQIHLRSIFPFQYFSIG
ASLIPFIEHNDANRALMSSNMQRQAVPLSRSEKCIVGTGLERQAALDSGVSAIAECEGKI
IHTDTHKIVLSGHGDTISIPLVMYQRSNKNTCMHQNPQVRRGKCIKKGQILADGAATVGG
ELALGKNVLVAYMPWEGYNFEDAVLISERLVYEDIYTSFHIRKYEIQTHVTSQGPERITH
EIPHLEAHLLRNLDRNGIVALGSWVETGDILVGKLTPQTANESSYAPEDRLLRAILGIQV
STAKETCLKLPIGGRGRVIDVRWIQKKGGSSYNPETIRVYISQKREIKVGDKVAGRHGNK
GIISKILSRQDMPYLQDGTPVDMVFNPLGVPSRMNVGQIFECSLGLAGDLLDRHYRIAPF
DERYEQEASRKLVFSELYEASKQTANPWVFEPEYPGKSRIFDGRTGDPFEQPVLIGKSYI
LKLIHQVDDKIHGRSSGHYALVTQQPLRGRAKQGGQRVGEMEVWALEGFGVAHILQEMLT
YKSDHIRARQEVLGTTIIGGTIPTPEDAPESFRLLVRELRSLALELNHFLVSEKNFQINR
KEA
>O03060 | NU4C_HORVU
MSYFPWLTILVVLPIFAGSLIFFLPHKGNKIVRWYTISICLLEFLLMTYAFCYHFQLEDP
LIQLKEDYKWIDVFDFHWRLGIDGLSLGSILLTGFITTLATLAAWPITRNSRLFYFLMLA
MYSGQIGLFSSRDLLLFFIMWELELIPVYLLLSMWGGKRRLYSATKFILYTAGGSIFFLI
GVLGMGLYGSNEPGLDLERLINQSYPATLEILLYFGFLIAYAVKLPIIPLHTWLPDTHGE
AHYSTCMLLAGILLKMGAYGLIRINMELLPHAHYLFSPWLVIIGAIQIIYAALTSLGQRN
FKKRIAYSSVSHMGFIIIGIGSITNIGLNGAILQILSHGFIGATLFFLAGTASDRMRLVY
LEELGGISIPMPKIFTMFSSFSMASLALPGMSGFVAELVVFFGLITSPKFLLMPKALITF
VMAIGMILTPIYLLSMLRQMFYGYKLFNVPNANFVDSGPRELFILICIFLPVIGIGIYPD
FVLSLSVDRVEALLSNYYPK

>O04683 | FER1_MESCR
MAATTAALSGATMSTAFAPKTPPMTAALPTNVGRALFGLKSSASRGRVTAMAAYKVTLVT
PEGKQELECPDDVYILDAAEEAGIDLPYSCRAGSCSSCAGKVTSGSVNQDDGSFLDDDQI
KEGWVLTCVAYPTGDVTIETHKEEELTA
>O04997 | SODCP_SOLCS
MAAHCILFSSPAATTSLIFPISNPNTAVSLPSSSFHGVSLKSTINRQSLTLSAAASAAPK
PLTVFAATKKAVAVLKGTSSVEGVVTLTQEEDGPTTVNVKITGLTPGPHGFHLHEFGDTT
NGCISTGPHFNPNGNTHGAPEDENRHAGDLGNIIANADGVAEATIVDNQIPLTGPNAVVG
RAFVVHELADDLGKGGHELSLSTGNAGGRLACGVVGLTPV
>O19816 | MATK_ALLCA
MEAIQRYLQFDRSQQLSFLYPLIFQEYIYAFARDHSLNRAILLENPGYDNKSSFLIVKRL
ITRMYQQNHFIISANDSSQNPFFGRNKNLYSKMISEGFSFIIEIPLSTRLISSEELKGIL
KSPNLRSIHSIFPFLEDNFSHFHFVLDILIPHPVHLEILFQTLRYWLKDAPSLHLLRXXX
XXXXXXXXXXXXXXXXXXXXXXXXXXXXXXXXXXXXXXXXXXXXXXXXXXXXXXXXXXXX
XXXXXXXXXXXXXXXXXXXXXXXXXXXXXXXXXXXXXXXXXXXXXXXXXXXXXXXXXXXX
XXXXXXXXXXLWFHSERVYIKQLSNHSLDFMGYFSIVRLNPSMVRSQMLENXFLINNAIK
KFDTLVPMIPLIGSLSKAKFCNLFGHPISKSARTDLSDSDIMDRFGRICRNLSHYHSGSS
KKKSLYRIKYILRLSCAKTLARKHKSTVRAFLKRLGSEFFEEFLMSEEETLCLTFPRVSS
PFWGVYGSRIWYLDITCINDLANQQ
>O20163 | TILS_CHLVU
MKKDSTIISTCNEFSLSSVKTKRVYKNNHLLCSVSGGQDSIVSFFFLVNFLYLSKNFKLI
KSKLSSFSVASKSAEKKRCFDCFFSLKKNKKINALINKEKKLSRLDLVFCQHFWQTQNFF
CVEFLFQLTFFFDIPYTFVLSKNILVSENRARGWRKKTFSRLLKIQKLSTIVTGQTKTDT
AEKSITNLLRGTSPKSFSLITKANSKTTSSFFCFRLFRPKTFYLTLTGNKRERKVNRRIS
FSFYRTQKKDSKKLRETRAPFFIEMASFSAFFGNTFQKKKSSNSFLLYKTRLSFSFCFSG
ETREHSINSLKPLQNRNRYHISKLVKFYKFPLTIDATNFSLNFSRNKIRHLFIPFIRFLF
KLKFETLLLNFLYLVNLEHEQIENENLELKRVLNFLRINYLKKNFFSSNRRSFFVIQTGH
KNNNVFPTKKKEKRWFYCNEIGISKIRKQCKIHEDPILETSLVIYFTSVLTTSRKYSLLQ
NLLYNYREIEINFRQISKLENSFW
>O22077 | RBS_FAGCR
MASSMISSATVATVSRATPAQATMVAPFTGLKSTAAFPATRKSNNDITSLASNGGRVQCM
KVWPPLGLQKFETLSYLPPLSIESLAKQIEYLILKGWIPCLEFELEHPFVYRENNRSPGY
YDGRYWVMWKLPMFGCTDATQVLAELQEASKTYPTSHIRIIGFDNKRQVQCISFIAYKPP
AK
>O22340 | TPSDA_ABIGR
MALLSIVSLQVPKSCGLKSLISSSNVQKALCISTAVPTLRMRRRQKALVINMKLTTVSHR
DDNGGGVLQRRIADHHPNLWEDDFIQSLSSPYGGSSYSERAETVVEEVKEMFNSIPNNRE
LFGSQNDLLTRLWMVDSIERLGIDRHFQNEIRVALDYVYSYWKEKEGIGCGRDSTFPDLN
STALALRTLRLHGYNVSSDVLEYFKDEKGHFACPAILTEGQITRSVLNLYRASLVAFPGE
KVMEEAEIFSASYLKKVLQKIPVSNLSGEIEYVLEYGWHTNLPRLEARNYIEVYEQSGYE
SLNEMPYMNMKKLLQLAKLEFNIFHSLQLRELQSISRWWKESGSSQLTFTRHRHVEYYTM
ASCISMLPKHSAFRMEFVKVCHLVTVLDDIYDTFGTMNELQLFTDAIKRWDLSTTRWLPE
YMKGVYMDLYQCINEMVEEAEKTQGRDMLNYIQNAWEALFDTFMQEAKWISSSYLPTFEE
YLKNAKVSSGSRIATLQPILTLDVPLPDYILQEIDYPSRFNELASSILRLRGDTRCYKAD

RARGEEASAISCYMKDHPGSIEEDALNHINAMISDAIRELNWELLRPDSKSPISSKKHAF
DITRAFHHVYKYRDGYTVSNNETKNLVMKTVLEPLAL
>O22573 | RBS3_FRIAG
MASSATMLSSVATAARAAPAQASMVAPFVGLKSASAFPVTQKPATGLSTLPSNGGRVQCM
KVWPIVGLKKFETLSYLPTLSVESLLKQIEYLIRNGWVPCLEFSLEGFVSRDNNKSPGYY
DGRYWTMWKLPMFGCTDAAQVVKEAAECKKEYPAAFIRVIGFDNVRQVQCVSFIVEKPE
>O24310 | EFTU_PEA
MALSSTAATTSSKLKLSNPPSLSHTFTASASASVSNSTSFRPKLTLTRLSSSFLNPSTIL
HLTPSQRTNRPSSSPFTVRAARGKFERKKPHLNIGTIGHVDHGKTTLTAALTMALACLGN
SAPKKYDEIDAAPEERARGITINTATVEYETETRHYAHVDCPGHADYVKNMITGAAQMDG
AILVVSGADGPMPQTKEHILLAKQVGVPSVVVFLNKQDQVDDEELLELVELEVRELLSSY
EFPGDDIPIVSGSALLALEALMANPTLKRGNNQWVDKIYQLMDEVDKYIPIPQRQTELPF
LLAIEDVFSITXRGTVATGRIERGLVKVGDVVDLVGLRETRNTTVTGVEMFQKILDDAMA
GDNVGLLLRGIQKIDIQRGMVLAKPGTITPHSKFSAIVYVLKKEEGGRHSPFFAGYRPQF
YMRTTDVTGKVTSIMNDKDEESKMVMPGDRVKIVVELIVPVAIEQGMRFAIREGGKTVGA
GVIGAIIE
>O24600 | RPOT3_ARATH
MASAAAASPSLSLNPTSHFQHQTSLVTWLKPPPSSALFRRKTLPFFERHSLPISASSSSS
SSSSTSLSVHEKPISNSVHFHGNLIESFENQDSSYAGTIKGASLIEELENPVERNGLSGR
RRLFMQDPPWISALFLKGLSKMVDQTLKIERKDIDKRKFDSLRRRQVKEETEAWERMVDE
YRDLEKEMCEKNLAPNLPYVKHMFLGWFQPLKDVIEREQKLQKNKSKKVRAAYAPHIELL
PADKMAVIVMHKMMGLVMSGHEDGCIQVVQAAVSIGIAIEQEVRIHNFLKRTRKNNAGDS
QEELKEKQLLRKRVNSLIRRKRIIDALKVVKSEGTKPWGRATQAKLGSRLLELLIEAAYV
QPPLTQSGDSIPEFRPAFRHRFKTVTKYPGSKLVRRYGVIECDSLLLAGLDKSAKHMLIP
YVPMLVPPKRWKGYDKGGYLFLPSYIMRTHGSKKQQDALKDISHKTAHRVFEALDTLGNT
KWRVNRNILDVVERLWADGGNIAGLVNREDVPIPEKPSSEDPEELQSWKWSARKANKINR
ERHSLRCDVELKLSVARKMKDEEGFYYPHNLDFRGRAYPMHPHLNHLSSDLCRGTLEFAE
GRPLGKSGLHWLKIHLANLYAGGVEKLSHDARLAFVENHLDDIMDSAENPIHGKRWWLKA
EDPFQCLAACVILTQALKSPSPYSVISHLPIHQDGSCNGLQHYAALGRDSFEAAAVNLVA
GEKPADVYSEISRRVHEIMKKDSSKDPESNPTAALAKILITQVDRKLVKQTVMTSVYGVT
YVGAREQIKRRLEEKGVITDERMLFAAACYSAKVTLAALGEIFEAARAIMSWLGDCAKII
ASDNHPVRWITPLGLPVVQPYCRSERHLIRTSLQVLALQREGNTVDVRKQRTAFPPNFVH
SLDGTHMMMTAVACREAGLNFAGVHDSYWTHACDVDTMNRILREKFVELYNTPILEDLLQ
SFQESYPNLVFPPVPKRGDFDLKEVLKSQYFFN
>O24634 | RBS1_FRIAG
MAASSTMLSSVATAACAAPAQASMVAPFVGLKSTSAFPVTQKPATGLSTLPSNGGRVQCM
KVWPIVGLKKFETLSYLPTLSVESLLKQIEYLIRNGWVPCLEFSLEGFVSRDNNKSPGYY
DGRYWTMWKLPMFGCTDAAQVVKEAAECKKEYPAAFIRVIGFDNVRQVQCVSFIVERPE
>O47039 | ACCD_PICAB
MSIREWFEDRRKITGLLKNSVERDSKDVNEMERNKNLSIDYVKINRLWVQCDNCESLLYI
RFLRENKSVCEECGYYLQMNSSDRIELLIDRGTRHPMDEDMYTLDVLQFHSENEPAHSDP
LHSEDESYKDHITFCQIETGLTDAIQTGIGQLNGLPIALGVMDFKFMGGSMGSVVGEKIT
RLIERATEESLPVIMVCASGGARMQEGSFSSMQMAKIASALYIPQKDNRLLYVSILTSPT
TGGVTASFGMLGDIIIAEPKAYIAFAGKIVIDQTLGQKVIEDFQVTEHLFGHGLFDLIVP

RNLLKCVLSELFYVLQSSS
>O47041 | CHLL_PICAB
MKIAVYGKGGIGKSTTSCNISVALARRGQKVLQIGCDPKHDSTFTLTGFLIPTIIDTLQS
KDYHYEDIWPEDVIHKGYGGVDCVEAGGPPAGAGCGGYVVGETVKLLKELNAFYEYDIIL
FDVLGDVVCGGFAAPLNYADYCVIITDNGFDALFAANRITASIREKARTHPLRLAGLVGN
RTSRRDLINKYVEACPMPVIEVLPIIEDIRVSRVKGKTLFEMVGFEPSLNYVCNYYLGIA
DQILSQPEGIVPKEIPDRELFSLLSDLYLNPIGGGGQKKKNQENLLGFTRI
>O47125 | MATK_LYOLU
MEEFKRYLELDRSQQHDFIYPLIFQEYIYALARGRVLNGSIFFENAGYDNKSSLLIVKRL
ITHLITQMYQQNHFLFYTNDFNPNKFLGCNTNLYYQMIFEGFAVVVEIPFYLRLVSFLEG
KERVKSHNLRSLHSIFPFLEDKFSHLNSLLDILIPHPVHLEILVQTLRYWVKDPSSLHLL
RFFLHEYPNWNSLMTPKKFSFYFSKRNQRFFFFLYNFHVCEYESIFVFLRNQSSHLCSIS
SEIFLERISFYKKIELEEVFTKNFKAFLWAFKDPFLHYVRYRGKSFLASKDXXLLMNKWK
YYLVNFWECYFSIWSQPRRIHINQLSNNCVDFLGYLLSVRLKPSMVRSQMIENSFLIENA
SKQFDTLVPITLLIRYLSKAKFCNVLGHPMSKPVWAALSDSDIIERFGRIYRNLSHYHSG
SLKKISLYRIKYILRLSCARTLARKHKSTVRSFLKRLGMGLLEEFFTEEEQVFYLTFPKA
SSTSGKLYQGRIWYLDIFCINDPANHE
>O47129 | MATK_LYOFE
MEEFKRYLELHRFQQHDFIYPLIFQEYIYALAHGRGLNGSIFFENAGYDNKSSLLIVKRL
ITHLITQMYQQNHFLFYTNDFNPNKFLGCNTNLYSQMIFEGFAVVVEIPFYLRLLSFLEG
KERVKSHNLRSLHSIFPFLEDKFSHLNSLLDILIPHPVHLEILVQTLRYWVKDPSSLHLL
RFFLHEYPNWNSLMTPKKSSFSFSKRNQRFFFFLYNFYVCEYESIFVFLRNQSSHLCSTS
SETLSERICFYKKIELEEVFTKDFKAILWVFKDPFLHYVRYRGKSILASKDSSLLMNKWK
YYLVNIWECYFYIWSQPRRIHINQLSNNSLDFLGYLLSVRLKPSMVRSQMIENSFLIENA
IKQFDIIVPITPLVTSLSKAKFCNVLGHPYSKPSWAESSDSDIIERFGRIYRNLSHYHSG
SLKKISLYRIKYILRLSCARTLARKHKSTVRSFLKRLGVGLLEEFFTEEEQVFYLTFPRA
SSTSGKLYQRRIWYLDIFCINDPANHE
>O47170 | MATK_RHOHI
MEEFKRNLELDRSQQHDFIYPLIFQEYIYALAHDRGLNRSIFLENTGYENKSSLLIVKRL
ITHLITQMYQQNHFLFSGNDSNQNKFLGYNTNLYSQMIFGGFAVVVEIPFYLRLLSFLEA
KERVKSHNLRSIHSIFPFLEDKFSHLVYVLDILISHPIHLEIVIQTLRYWVKDASSLHLL
RFFLHEYPIWNSLITPKKSSFSFSIRNQRFFLFLYNFHVCEYESIFVFLRNQSSHLRSIS
SETFLERISFYRKIELEVFTKDFKAIIWVFKEPFLHYVRYRGKAILASKGTSLLMNKWKY
YLVNFWQCYFYMWSQPRRIHINQLSNHSLDFLGYLSTVRLKPLMVRSQMIENSFIIGNAS
KKFDTLMPITPMIGSLSKAKFCNVLGHPMSKPVWAALSDSDIIERFGRIYRNLSHYYSGS
LKKMSLYRIKYILRLSCARTLARKHKSTVRAFLKRLGVGLLEEFFTEEEQVFYLTFAKAS
SNSGELYRRRVWYLDIICINDLANYE
>O50039 | OTC_ARATH
MAAAMASHVSTARSPALSFSSSSSSFFPGTTLRRFSAVSLPSPALPRLRVSCQASSVTSP
SSPSDVKGKSDLKDFLAIDDFDTATIKTILDKASEVKALLKSGERNYLPFKGKSMSMIFA
KPSMRTRVSFETGFFLLGGHALYLGPNDIQMGKREETRDVARVLSRYNDIIMARVFAHQD
ILDLANYSSVPVVNGLTDHNHPCQIMADALTMIEHIGQVEGTKVVYVGDGNNMVHSWLEL
ASVIPFHFVCACPKGYEPDKERVSKAKQAGLSKIEITNDPKEAVIGADVVYSDVWASMGQ
KDEAEARRKAFQGFQVDEALMKLAGQKAYFMHCLPAERGVEVTNGVVEAPYSIVFPQAEN

RMHAQNAIMLHLLGF
>O62951 | RR3_PICAB
MAQKINPLGFRLGVTQNDRSHWFAQQRNYSKDLREDQKIRTCIENYVRTHIKSSSNYGGI
ARVEISRKIDLIQVKIYIGFPNLLLIEGRGFQGIEKLKNDVLNMLDSVDRKLHIAIEKVA
KPYRKPNILAEYIALQLEKRVPFRKTMKKAIELAEREEVEGIQIQIAGRLDGKEIARVEW
DRGGRVPLQTIRARIDYCYYPVQTIYGVLGIKIWILEE
>O62953 | RR19_PICAB
MARSLKKNPFVANHSLRKIQNLNIKEEKKIIVTWSRASVIVPAMIGHTIAVHNGREHLPI
YVTDRMVDHKLGEFAPTLLFQGHARNDKKSRR
>O62961 | RK23_PICAB
MDEVKYPVLTEKSIRLLERNQYTFNVDSQLNKTKMKIWIEHFFDVKVIAMNSYRLPEKGG
KGGSMIGHPIRCKRMIITLKPGDSIPLFSEQ
>O62964 | RBL_VIGUN
MSPQTETKASVGFKAGVKDYKLNYYTPEYETKDTDILAAFRVTPQPGVPPEEAGASVAAE
SSTGTWTTVWTDGLTSLDRYKGRCYHIEPVAGEENQYIAYVAYPLDLFEEGSVTNMFTSI
VGNVFGFKALRALRLEDLRIPNAYIKTFQGPPHGIQVERDKLNKYGRPLLGCTIKPKLGL
SAKNYGRAVYECLRGGLDFTKDDENVNSQPFMRWRDRFLFCAEAIFKSQAETGEIKGHYL
NATAGTCEEMMKRAVFARELGVPIVMHDYLTGGFTANTSLAHYCRDNGLLLHIHRAMHAV
IDRQKNHGMHFRVLAKALRLSGGDHVHSGTVVGKLEGERDITLGFVDLLRDDFVEKDRSR
GIYFTQDWVSLPGVLPVASGGIHVWHMPALTEIFGDDSVLQFGGGTLGHPWGNAPGAVAN
RVALEACVKARNEGRDLAREGNEIIREASKWSPELAAACEVWKEIKFEFPAMD
>O62992 | MATK_LEDPA
MEEFKRNLELDRSQQHDFIYPLIFQEYIYALAHDRGLNRSIFLENTGYDNKSSLLIVKRL
ITHLITQMYQQNRFLFSGNDSNQKKFFGDNTNLYSQMIFEGFAVVVEIPFYLRLLSFLEG
KERVKSHNLRSIHSIFPFLEDKFSHLVYVLDILISHPIHLEIVVQTLRYWVKDASSLHLL
RFFLHEYPIWNSLITPKKSSFSFSIRNQRFFLFLYNFHVCEYESIFVFLRNQSSHLRSIS
SETFLERISFYRKIELEVFAKDFKAILWVFKEPFLHYVRYRGKAILASKGTSLLMNKWKY
YLVNFWQCYFYMWSQPRRIHINQLSNHSLDFLGYLSTVRLKPLMVRSQMIENSFLIENAS
KKFDTLMPITPMIGSLSKAKFCNVLGHPMSKPVWAALSDSDIIERFGRIYRNLSHYYSGS
LKKMSLYRIKYILRLSCARTLARKHKSTVRAFLKRLGVGLLEEFFTEEEQVFYLTFAKAS
SNSGELYRRRVWYLDIICINDLANYE
>O63070 | MATK_PICGL
MDEFHRYGKEDSSWQQCFLYPLFFQEDLYAISHDHYLDGSSSSEPMEHLSSNDQFSFLTV
KRLIGQIRQQNHSIVLFVNCDPNPLVDRKKSSYSESVLEGLTLVLEVPFSIRSKYSVEGM
NEWKSFRSIHSIFPFLEDKFPHSNYVSDTRIPYSIHPEILVRTFRRWIGDAPSLHPLRSI
LYEYRNSSESLQRSIIVVPKVNTRFFLFLWNNYVYECESILVSLLKRSSHSRSLSHGSFP
QRTHFHRKIKNIFLFSRRNSFQSIWSLKDPNIHYVRYGERSIIAIKGTNLLVKKYRYYLP
IFRQCYFHLWNEPYRVCSHQLSKNCSSSLGYFLRFRMKPLLVKTKMLDELFIADLITDEF
DPIVPIVPIIGLLSREKFCDISGRPISKLSWTSLTDDDILDRFDRIWRNLFHYYSGSFGR
DGLYRIKYILSLSCAKTLACKHKSTIRVVRKELGPELFKKSFSKERELDSPPFSSKAAAR
SQRERIWHSDIPQINPLAHSWQKIQDLKIENLFDQ
>O64416 | RBS_MARPA
MASVVASAAVVTPFAASAASTTKSSQIVSVQAGLKAGVFGGKSEWQTKTQTNGSRVSCMQ
VWEPYNNLKFETLSYLPPLSQDALAKQIDYVIKSGWAPCIEFDVQGTVTREGSTMPGYYD

GRYWTMWKLPMFGCTDSASVLREIEECKKLYGKKCYIRCLGFDNTRQVQCASFIVHQPTL

>O65037 | RK27_ORYSJ
MASMAFTLVGAFKGMSLSSPCHSSSSASFLRADRVSLSVGGGVGMGVPMTMPVRRLTIQM
AHKKGAGSTKNGRDSPGQRLGVKIYGDQVAKPGAIIIRQRGTRVYPGNNVGMGKDHTLFS
LIDGLVKFEKYGPDKKKVSVYPYEKQPENPNSYRARKREYFRMQRERKKARAEGIVEVQL
VLAAADESPEVNADC
>O65349 | RBS_CAPAN
MASSVMSTATVATGANAAQASMIASFNGLKSAASFPVTRKQDLDITSIASNGGRVECMLV
WPPINKKKYETLSYLPDLSDEQLLKEIEYLLQKGWVPCLEFETEHGFVYREHHRSPGYYD
GRYWTMWKLPMFGCTDATQVLNEVQEAKKAYPQAWIRIIGFDNVRQVQCISFIAYKPEAT
KFSMFNV
>O78248 | MATK_ROSFO
MEEFQGYLELDRSQQHDFLYPLIFREYIYALAHDRGLNRSVLLDNVSYDKKSSLLIIKRL
ISRMYQQNHFIISVNDSNQNKFFGYNKNLYSQMISEGFAVIVEIPFSLRLVSSLEETETV
KSYNLRSIHSIFPFFEDKFPHLNYASDVLIPYPIHLEILVQTLRYCVKDPSSLHLLRLFL
HEYYNWNTLITPKKSIFAKSNQRLFLLLYNSYVCEYESILLFLRNQSNHLRLTSSGILFE
RIRFYEKIKYPVEEVFANDFPATLWFFKDPFIQYVRYQGKSILASKDTPLLMNKWKYYLV
HFWQCHFYVWSQPGRIHINQLSKHSFDFLGYLSSIRPNISVVRSQLLENSFLMDNAMKKL
DTLFPIIPMIGSLAKVKFCNTSGHPISKSSWADSSDSDIIDRFVRIGENLSHYYSGSSKK
KSLYRIKYILRLSCVKTLARKHKSTVRTFLKRLGPKLLDEFFTEEEQIFSLLFPRTSSTL
KRFYRGRIWYLDILCINDLVNHE
>O78253 | MATK_ROSRU
MEEFQGYLELYRSQQHDFLYPLIFREYIYALAHDRGLNRSVLLDNVGYDKKSSLLIIKRL
ISRMYQQNHFLISVNDSNQNKFFGYNKNLYSQIISEGFAVIVEIPFSLRLVSSLKETETV
KSYNLRSIHSIFPFFEDKFPHLNYASDVLIPYPIHLEILVQTLRYCVKDPSSLHLLRLFL
HEYYNWNTLITPKKSIFAKSNQRLFLLLYNSYVCEYESILLFLRNQSNHLRLTSFGILFE
RIRFYEKIKYPVEEVFANDFPATLWFFKDPFIQYVRYQGKSILASKDTPLLMNKWKYYLV
HFWQCHFYVWFQPGRIHINQLSKHSFDFLGYLSSIRPNISVVRSQLLENSFLMDNAMKKL
DTLFPIIPMIGSLAKVKFCNTSGHPISKSSWADSSDSDIIDRFVRIGGNLSHYYSGSSKK
KSLYRIKYILRLSCVKTLARKHKSTVRTFLKRLGPKLLDEFFTEEEQIFSLLFPRTSSTL
KRFYRGRIWYLDILCINDLVNHE
>O78310 | SODCP_ARATH
MAATNTILAFSSPSRLLIPPSSNPSTLRSSFRGVSLNNNNLHRLQSVSFAVKAPSKALTV
VSAAKKAVAVLKGTSDVEGVVTLTQDDSGPTTVNVRITGLTPGPHGFHLHEFGDTTNGCI
STGPHFNPNNMTHGAPEDECRHAGDLGNINANADGVAETTIVDNQIPLTGPNSVVGRAFV
VHELKDDLGKGGHELSLTTGNAGGRLACGVIGLTPL
>O80363 | RK17_TOBAC
MASASTTWSMSCLKSALPSIQPISSSSLRFSCGPSPSRLRICKPKSSSRLLHSFVGLAPL
HPLLSLSSQDSTSFEHSFTVIDNGGRVFAMRHGRKVPKLNRPPDQRRALLRGLTTQLLKH
GRIKTTKARARAVRKYVDKMITMAKDGSLHKRRQALGFIYEKQIVHALFAEVPDRYGERN
GGYTRIIRTLPRRGDNAPMAYIELV
>O81192 | BPPS_SALOF
MSIISMNVSILSKPLNCLHNLERRPSKALLVPCTAPTARLRASCSSKLQEAHQIRRSGNY

QPALWDSNYIQSLNTPYTEERHLDRKAELIVQVRILLKEKMEPVQQLELIHDLKYLGLSD
FFQDEIKEILGVIYNEHKCFHNNEVEKMDLYFTALGFRLLRQHGFNISQDVFNCFKNEKG
IDFKASLAQDTKGMLQLYEASFLLRKGEDTLELAREFATKCLQKKLDEGGNEIDENLLLW
IRHSLDLPLHWRIQSVEARWFIDAYARRPDMNPLIFELAKLNFNIIQATHQQELKDLSRW
WSRLCFPEKLPFVRDRLVESFFWAVGMFEPHQHGYQRKMAATIIVLATVIDDIYDVYGTL
DELELFTDTFKRWDTESITRLPYYMQLCYWGVHNYISDAAYDILKEHGFFCLQYLRKSVV
DLVEAYFHEAKWYHSGYTPSLDEYLNIAKISVASPAIISPTYFTFANASHDTAVIDSLYQ
YHDILCLAGIILRLPDDLGTSYFELARGDVPKTIQCYMKETNASEEEAVEHVKFLIREAW
KDMNTAIAAGYPFPDGMVAGAANIGRVAQFIYLHGDGFGVQHSKTYEHIAGLLFEPYA
>O81193 | SSS_SALOF
MSSISINIAMPLNSLHNFERKPSKAWSTSCTAPAARLRASSSLQQEKPHQIRRSGDYQPS
LWDFNYIQSLNTPYKEQRHFNRQAELIMQVRMLLKVKMEAIQQLELIDDLQYLGLSYFFQ
DEIKQILSSIHNEPRYFHNNDLYFTALGFRILRQHGFNVSEDVFDCFKIEKCSDFNANLA
QDTKGMLQLYEASFLLREGEDTLELARRFSTRSLREKFDEGGDEIDEDLSSWIRHSLDLP
LHWRVQGLEARWFLDAYARRPDMNPLIFKLAKLNFNIVQATYQEELKDISRWWNSSCLAE
KLPFVRDRIVECFFWAIAAFEPHQYSYQRKMAAVIITFITIIDDVYDVYGTIEELELLTD
MIRRWDNKSISQLPYYMQVCYLALYNFVSERAYDILKDQHFNSIPYLQRSWVSLVEGYLK
EAYWYYNGYKPSLEEYLNNAKISISAPTIISQLYFTLANSIDETAIESLYQYHNILYLSG
TILRLADDLGTSQHELERGDVPKAIQCYMNDTNASEREAVEHVKFLIREAWKEMNTVTTA
SDCPFTDDLVAAAANLARAAQFIYLDGDGHGVQHSEIHQQMGGLLFQPYV
>O81220 | DCUP_MAIZE
MATACPPLSLPSTSLFRGRSARAGPNAGSSRPSAAAPSERRSWRRPRPDGGRAAAGERNQ
REEVERPPVWLMRQAGRYMKSYQLLCERYPSFRERSENVDLVVEISLQPWKVFKPDGVIL
FSDILTPLPGMNIPFDIVKGKGPVIYDPLRTAAAVNEVREFVPEEWVPYVGQALNILRQE
VKNEAAVLGFVGAPFTLASYCVEGGSSKNFTLIKKMAFSEPAILHNLLQKFTTSMANYIK
YQADNGAQAVQIFDSWATELSPADFEEFSLPYLKQIVDSVRETHPDLPLILYASGSGGLL
ERLPLTGVDVVSLDWTVDMAEGRKRLGSNTAVQGNVDPGVLFGSKEFITRRIYDTVQKAG
NVGHVLNLGHGIKVGTPEENVAHFFEVAKGIRY
>O98451 | RK23_SPIMX
MDKIKYRPIGMKAIRLLERRQYTFDVDVKATKTEVKRWIEGFFSVKVVGMNSHRPPKKKK
RMGSVIGYPVRYKRMIVTLKVGDSIPLS
>O98455 | RR3_SPIMX
MGQKIHPLGFRLGVTQEHLSNWFARPSRYSDLLEEDEKIRNCIKEYVRTHIRNSSNYGGI
SRVKIQRKTDLVQVDIHTGFPALLIEGRGKGLLVLKQSVLNSISTERKLKITLSEIDKYY
AEANILAEYIALQLESRVAFRRTMKKAIQLAMEQGKVKGIKIQIAGRLNGAEIARIEWAR
EGRVPLQTLRACIDYCHYPAQTTYGVLGIKVWIFKGEE
>O98456 | RK16_SPIMX
MLSPRRTKFRKQHRGRMKGVATRGNQIAFGRFALQAMEASWITSRQIEAGRRAMTRYARR
GGKLWIKIFPDKPITMRPAETRMGSGKGAPEYWVAVVKPGRILYEMSGISETIARAAMRI
AAYKMPIKTRFIVANLSKNPTND
>O98458 | RR8_SPIMX
MSNDIISNMITSIRNAILSKTKTVDILATTTTRSIAKILLQEGFIDGLRERQENGRRLLL
LTLHYERKNKSKITTSLRRISKPGLRVYSNHQEIPKVLGGIGIVILSTSKGIMVDREARH
KKIGGEIICYVS

>O98461 | RR11_SPIMX
MAKTANKINIRKVKRKTPKAIIHVQASFNNTIVTVTDVQGQVISSCSAGACGFKGAKKNT
PFAAQTAAENAIRLLIDQGLKQAEVMISGPGRGRDTALRAIRNSGITLSLVRDVTPLPHN
GCRRPKTRRV
>O98634 | MATK_BRASC
MDKLQYELQGYLQIDRYRKQRFLYPLLFREYIYALAHDHGLNSSIFYEPTENLGYDNKFS
SLIVKRLITRLHQQNHLITSVNYSRFVGPNRSFYSQTIPEGFAGIMEISFSMRLVSSLER
IAKYQNLRSIHSIFPFLEDKLSHLSYVSDILIPYPIHLEILLQTLRTRIRDAPSLHLLRC
FLHEHHNCNSPITPKKCISIENQRLFLFLYNSHVYECESILVFLRKQSSHLRSISFLAFL
ERTHFYGKIKHLVVAPRNDSQRTLPLWFFKEPLMHYVRYQGKSIMASRCTNLLMXKWKYY
LVNFWQCHFHLWSQPSRIHINELSNHSFYFLGYLSGVRLTPWVIRSQMVENSFMIDTAIK
RFDTIVPIFPLIGSLVKAKFCNVSGHPTSKSVWADLSDSDIIARFGWICRNLSHYHSGSS
KKHSLCRIKYILRLSCARTLARKHKSTVRAICKRLGSKLLEEFLTEEQEIVSFIFRRTRL
HSERIWYLDIIRINGLVPNS
>O98636 | MATK_BARLO
MEKLQYELQGYLEIERYRKQRFLYPLLFREYIYALAHDHGLNSSIFYEPTENLGYDNDNK
SSSLIVKRLITRLHQQNHLTISVNDSRFVGPNRSFYSQTIPEGFAGIMEIPFSMRLVSSL
ERLTKYQNFRSIHSIFSFLEDKLSHLYYVSDILIPHPIHLEILLQTLRTRIRDAPSLHLL
RCFLHEHNNWNSLITLNKSISIFSKENQRLFLFLYNSHVYECESVLVFLRKQSSHLRSIS
SLAFLERTHFYGKIKHLVVTLRNDSQRTLPLWFFKEPLMHYVRYQGKSIMASRCTNLLMK
KWKYYLVNFWQCHFHLWSQPGGIHINELSNHSFHFLGYLSGVRLTPWVIRSQMLEDSFMI
DTAIKRFDTIVPIFPLIGSLVKAKFCNVSGYPISKSVWADSSDSDIIARFGWICTNLSHY
HSGSSKKHSLCRIKNILRLSCARTLARKHKSTVRAICKRLGXKLLEEFFTEEHEIVSFIF
RRTRLRSERIWYLDIIRINGLVPHS
>O98691 | NDHH_HORVU
MSLPLTKKDLMIVNMGPQHPSMHGVLRLIVTLDGEDVIDCEPILGYLHRGMEKIAENRTI
IQYLPYVTRWDYLATMFTEAITVNAPEFLENIQIPQRASYIRVIMLELSRIASHLLWLGP
FMADLGAQTPFFYIFREReliyDLFEAATGMRMMHNYFRIGGVAADLPYGWIDKCLDFCD
YFLRGVVEYQQLITQNPIFLERVEGVGFISGEEAVNWGLSGPMLRASGIQWDLRKVDPYE
SYNQFDWKVQWQKEGDSLARYLVRVGEMSESIKIIQQAIEKIPGGPYENLEVRRFKKEKN
SEWNDFEYKFLGKKPSPNFELSRQELYVRVEAPKGELGIYLVGDDSLFPWRWKIRPPGFI
NLQILPQLVKKMKLADIMTILGSIDIIMGEVDR
>P00225 | FER_LEUGL
AFKVKLLTPDGPKEFECPDDVYILDQAEELGIELPYSCRAGSCSSCAGKLVEGDLDQSDQ
SFLDDEQIEEGWVLTCAAYPRSDVVIETHKEEELTG
>P00230 | FER1_PHYES
ATYKVTLVTPSGTQTIDCPDDTYVLDAAEEAGLDLPYSCRAGSCSSCAGKVTAGTVDQED
QSFLDDDQIEAGFVLTCVAYPKGDVTIETHKEEDIA
>P00231 | FER2_PHYAM
AASYKVTFVTPSGTNTITCPADTYVLDAAEESGLDLPYSCRAGACSSCAGKVTAGAVNQE
DGSFLEEEQMEAGWVLTCVAYPTSDVTIETHKEEDLTA
>P00232 | FER2_PHYES
AASYKVTFVTPSGTKTITCPADTYVLDAAEDTGLDLPYSCRAGACSSCAGKVTAGSVNQE
DGSFLDEEQMEAGWVLTCVAYPTSDVTIETHKEEDLSA

>P00233 | FER_GLEJA
AIFKVKFLTPDGERTIEVPDDKFILDAGEEAGLDLPYSCRAGACSSCTGKLLDGRVDQSE
QSFLDDDQMAEGFVLTCVAYPAGDITIETHAEEKL
>P00234 | FER1_EQUTE
AYKTVLKTPSGEFTLDVPEGTTILDAAEEAGYDLPFSCRAGACSSCLGKVVSGSVDQSEG
SFLDDGQMEEGFVLTCIAIPESDLVIETHKEEELF
>P00235 | FER1_EQUAR
AYKTVLKTPSGEFTLDVPEGTTILDAAEEAGYDLPFSCRAGACSSCLGKVVSGSVDESEG
SFLDDGQMEEGFVLTCIAIPESDLVIETHKEEELF
>P00238 | FER_SCEQU
ATYKVTLKTPSGDQTIECPDDTYILDAAEEAGLDLPYSCRAGACSSCAGKVEAGTVDQSD
QSFLDDSQMDGGFVLTCVAYPTSDCTIATHKEEDLF
>P00240 | FER2_DUNSA
AYKVTLKTPSGDQTIEVSPDAYILDAAEEAGLDLPYSCRAGACSSCAGKVEAGTIDQSDQ
SFLDDDQQGRGFVLTCVAYATSDCTISTHQEESLY
>P00870 | RBS1_SPIOL
MQVWPPLGLKKFETLSYLPPLTTEQLLAEVNYLLVKGWIPPLEFEVKDGFVYREHDKSPG
YYDGRYWTMWKLPMFGGTDPAQVVNEVEEVKKAPPDAFVRFIGFNDKREVQCISFIAYKP
AGY
>P00873 | RBS1_CHLRE
MAAVIAKSSVSAAVARPARSSVRPMAALKPAVKAAPVAAPAQANQMMVWTPVNNKMFETF
SYLPPLTDEQIAAQVDYIVANGWIPCLEFAEADKAYVSNESAIRFGSVSCLYYDNRYWTM
WKLPMFGCRDPMQVLREIVACTKAFPDAYVRLVAFDNQKQVQIMGFLVQRPKTARDFQPA
NKRSV
>P04045 | PHSL1_SOLTU
MATANGAHLFNHYSSNSRFIHFTSRNTSSKLFLTKTSHFRRPKRCFHVNNTLSEKIHHPI
TEQGGESDLSSFAPDAASITSSIKYHAEFTPVFSPERFELPKAFFATAQSVRDSLLINWN
ATYDIYEKLNMKQAYYLSMEFLQGRALLNAIGNLELTGAFAEALKNLGHNLENVASQEPD
AALGNGGLGRLASCFLDSLATLNYPAWGYGLRYKYGLFKQRITKDGQEEVAEDWLEIGSP
WEVVRNDVSYPIKFYGKVSTGSDGKRYWIGGEDIKAVAYDVPIPGYKTRTTISLRLWSTQ
VPSADFDLSAFNAGEHTKACEAQANAEKICYILYPGDESEEGKILRLKQQYTLCSASLQD
IISRFERRSGDRIKWEEFPEKVAVQMNDTHPTLCIPELMRILIDLKGLNWNEAWNITQRT
VAYTNHTVLPEALEKWSYELMQKLLPRHVEIIEAIDEELVHEIVLKYGSMDLNKLEEKLT
TMRILENFDLPSSVAELFIKPEISVDDDTETVEVHDKVEASDKVVTNDEDDTGKKTSVKI
EAAAEKDIDKKTPVSPEPAVIPPKKVRMANLCVVGGHAVNGVAEIHSEIVKEEVFNDFYE
LWPEKFQNKTNGVTPRRWIRFCNPPLSAIITKWTGTEDWVLKTEKLAELQKFADNEDLQN
EWREAKRSNKIKVVSFLKEKTGYSVVPDAMFDIQVKRIHEYKRQLLNIFGIVYRYKKMKE
MTAAERKTNFVPRVCIFGGKAFATYVQAKRIVKFITDVGATINHDPEIGDLLKVVFVPDY
NVSVAELLIPASDLSEHISTAGMEASGTSNMKFAMNGCIQIGTLDGANVEIREEVGEENF
FLFGAQAHEIAGLRKERADGKFVPDERFEEVKEFVRSGAFGSYNYDDLIGSLEGNEGFGR
ADYFLVGKDFPSYIECQEKVDEAYRDQKRWTTMSILNTAGSYKFSSDRTIHEYAKDIWNI
EAVEIA
>P04713 | SSG1_MAIZE
MAALATSQLVATRAGLGVPDASTFRRGAAQGLRGARASAAADTLSMRTSARAAPRHQQQA

RRGGRFPSLVVCASAGMNVVFVGAEMAPWSKTGGLGDVLGGLPPAMAANGHRVMVVSPRY
DQYKDAWDTSVVSEIKMGDGYETVRFFHCYKRGVDRVFVDHPLFLERVWGKTEEKIYGPV
AGTDYRDNQLRFSLLCQAALEAPRILSLNNNPYFSGPYGEDVVFVCNDWHTGPLSCYLKS
NYQSHGIYRDAKTAFCIHNISYQGRFAFSDYPELNLPERFKSSFDFIDGYEKPVEGRKIN
WMKAGILEADRVLTVSPYYAEELISGIARGCELDNIMRLTGITGIVNGMDVSEWDPSRDK
YIAVKYDVSTAVEAKALNKEALQAEVGLPVDRNIPLVAFIGRLEEQKGPDVMAAAIPQLM
EMVEDVQIVLLGTGKKKFERMLMSAEEKFPGKVRAVVKFNAALAHHIMAGADVLAVTSRF
EPCGLIQLQGMRYGTPCACASTGGLVDTIIEGKTGFHMGRLSVDCNVVEPADVKKVATTL
QRAIKVVGTPAYEEMVRNCMIQDLSWKGPAKNWENVLLSLGVAGGEPGVEGEEIAPLAKE
NVAAP
>P05346 | RBS1_BRANA
MASSMLSSAAVVTSPAQATMVAPFTGLKSSSAFPVTRKANNDITSIVSNGGRVSCMKVWP
PVGKKKFETLSYLPDLTEVELGKEVDYLLRNKWIPCVEFELEHGFVYREHGSTPGYYDGR
YWTMWKLPLFGCTDSAQVLKEVQECKTEYPNAFIRIIGFDNNRQVQCISFIAYKPPSFTG
A
>P05698 | RBL_HORVU
MSPQTETKAGVGFQAGVKDYKLTYYTPEYETKDTDILAAFRVSPQPGVPPEEAGAAVAAE
SSTGTWTTVWTDGLTSLDRYKGRCYHIEPVAGEDSQWICYVAYPLDLFEEGSVTNMFTSI
VGNVFGFKALRALRLEDLRIPPTYSKTFQGPPHGIQVERDKLNKYGRPLLGCTIKPKLGL
SAKNYGRACYECLRGGLDFTKDDENVNSQPFMRWRDRFVFCAEAIYKSQAETGEIKGHYL
NATAGTCEEMIKRAVFARELGVPIVMHDYLTGGFTANTTLAHYCRDNGLLLHIHRAMHAV
IDRQKNHGMHFRVLAKALRMSGGDHIHSGTVVGKLEGEREMTLGFVDLLRDDFIEKDRAR
GIFFTQDWVSMPGVIPVASGGIHVWHMPALTEIFGDDSVLQFGGGTLGHPWGNAPGAAAN
RVALEACVQARNEGRDLAREGNEIIRAACKWSPELAAACEVWKAIKFEFEPVDTIDKKV
>P05727 | RK16_CHLSP
MLSPKRTKFRKPHRGHLRGKATRGNTIVFGDFALQAQEPCWITSRQIEAGRRVLTRYVRR
GGKLWIRIFPDKAVTMRPAGTRMGSGKGAPDYWVAVVHPGKILYEMQGVSETIARQAMRI
AAYKMPVKTKFLTKQA
>P06260 | NU4LC_MARPO
MLEHILTLSAFLFCIGVFGLITSRNMVRALMCLELIFNAVNINLVAFSNFLDSSQIKGEI
FSIFIIAIAAAEATIGLAIVLAIYRNRKSTRIDQFNLLKW
>P06263 | NU4C_MARPO
MNHFPWLTIIVLFPISAGLVIPFLPSTGNKIIRWYTLGVCLLEFLLITYIFCYHYQFNDH
LIQLKEDYNWISFINFHWRLGIDGFSIGLILLTGFITTLATLAAWPVTRNPRLFYFLMLA
MYSGQIGLFASQDILLFFFMWELELLPVYLLLAMWGGKRRLYAATKFILYTAAGSLFILI
GGLIMAFYNSNEFTFDFQFLINKKYPLELEIIIYLSFLIAYAVKLPIIPFHTWLPDTHGE
AHYSTCMLLAGILLKMGAYGLIRINMELLPHAHSFFAPWLVIVGAIQIVYAALTSLSQRN
LKRRIAYSSVSHMGFVLIGIGSITNLGLNGAILQMISHGLIGASLFFLAGISYDRTRTLV
LDQMGGIGNSMPKIFTLFTSCSMASLALPGMSGFIAELMIFLGVIDNPNYSSLFKIIIII
IQGIGIILTPIYLLSMLRQMFYGYKFSNTLEPYFMDAGPREIFILICLFFPIISIGIYPN
FVLSIWNSKVNFLLSNNFF
>P06269 | RPOA_TOBAC
MVREKVTVSTRTLQWKCVESRTDSKRLYYGRFILSPLMKGQADTIGIAMRRALLGEIEGT
CITRVKSEKVPHEYSTITGIQESVHEILMNLKEIILRSNLYGTSDASICVKGPGSVTAQD

IILPPYVEIVDNTQHIASLTEPIDFCIGLQIERNRGYLIKTPHNFQDGSYPIDAVFMPVR
NANHSIHSYGNGNEKQEILFLEIWTNGSLTPKEALHEASRNLIDLFIPFLHMEEDNLYLQ
DNQHTVPLSPFTFHDKLAKLIKNKKKIALKSIFIDQSELPSRIYNCLKMSNIYTLLDLLN
NSQEDLMKIEHFRSEDVKRILGILEKYFVIDLAKNKF
>P06271 | RPOB_TOBAC
MLGDGNEGISTIPGFNQIQFEGFCRFIDQGLTEELYKFPKIEDTDQEIEFQLFVETYQLV
EPLIKERDAVYESLTYSSELYVSAGLIWKNSRDMQEQTIFIGNIPLMNSLGTSIVNGIYR
IVINQILQSPGIYYRSELDHNGISVYTGTIISDWGGRSELEIDRKARIWARVSRKQKISI
LVLSSAMGLNLREILENVCYPEIFLSFLSDKERKKIGSKENAILEFYQQFACVGGDPVFS
ESLCKELQKKFFQQRCELGRIGRRNMNRRLNLDIPQNNTFLLPRDILAAADHLIGLKFGM
GALDDMNHLKNKRIRSVADLLQDQFGLALVRLENVVRGTICGAIRHKLIPTPQNLVTSTP
LTTTYESFFGLHPLSQVLDRTNPLTQIVHGRKLSYLGPGGLTGRTASFRIRDIHPSHYGR
ICPIDTSEGINVGLIGSLAIHARIGHWGSLESPFYEISERSTGVRMLYLSPGRDEYYMVA
AGNSLALNQDIQEEQVVPARYRQEFLTIAWEQVHLRSIFPFQYFSIGASLIPFIEHNDAN
RALMSSNMQRQAVPLSRSEKCIVGTGLERQAALDSGALAIAEREGRVVYTNTDKILLAGN
GDILSIPLVIYQRSNKNTCMHQKLQVPRGKCIKKGQILADGAATVGGELALGKNVLVAYM
PWEGYNSEDAVLISERLVYEDIYTSFHIRKYEIQTHVTSQGPEKVTNEIPHLEAHLLRNL
DKNGIVMLGSWVETGDILVGKLTPQVVKESSYAPEDRLLRAILGIQVSTSKETCLKLPIG
GRGRVIDVRWIQKRGGSSYNPETIRVYILQKREIKVGDKVAGRHGNKGIISKILPRQDMP
YLQDGRSVDMVFNPLGVPSRMNVGQIFECSLGLAGSLLDRHYRIAPFDERYEQEASRKLV
FSELYEASKQTANPWVFEPEYPGKSRIFDGRTGNPFEQPVIIGKPYILKLIHQVDDKIHG
RSSGHYALVTQQPLRGRAKQGGQRVGEMEVWALEGFGVAHILQEMLTYKSDHIRARQEVL
GTTIIGGTIPNPEDAPESFRLLVRELRSLALELNHFLVSEKNFQINRKEA
>P06272 | RPOB_MARPO
MEIFILPEFGKIQFEGFNRFINQGLSEELSNFPIIEDIDQEFEFQIFGEQYKLAEPLLKE
RDAVYQSITYSSDVYVPAQLTQKKKGKIQKQIVFLGSIPLMNSQGTFVVNGVARVIINQI
LRSPGIYYNSELDHNGIPIYTGTLISNWGGRLKLEIDGKTRIWARISKKRKVSILVLLLA
MGLNLQNILDSVCYPKIFLEFIKKNTKKEYPNSTEDAIVELYKHLYCIGGDLFFSESIRK
ELQKKFFQQRCELGKIGRLNLNKKLNLNVPENEIFVLPQDILAAVDYLIKLKFGIGTIDD
IDHLKNRRVCSVADLLQDQLKLALNRLENSVLFFFRGATKRKRLPTPKSLVTSTPLIMTF
KEFFGSHPLSQFLDQTNPLTEIVHKRRLSSLGPGGLTRRTASFQVRDIHASHYGRICPIE
TSEGMNAGLIASLAIHAKISILGCLESPFYKISKLSNLEEIINLSAAEDEYYRIATGNCL
ALDQNSQEEQITPARYRQDFVAIAWEQVHLRSIFPLQYFSVGASLIPFLEHNDANRALMG
SNMQRQAVPLLKPEKCIVGTGIESQTALDSGSVTVSSHGGKIEYLDGNQIILSLKKKKID
KNLIIYQRSNNSTCMHQKPKVEKQKYIKKGQILADGAATANGELALGKNILVAYMPWEGY
NFEDAILINERLIYEDIYTSIHIERYEIEARVTSQGPEKFTNEIPHLDDYLLRHLDQNGI
VLTGSWVETGDVLVGKLTPQETEENLRAPEGKLLQAIFGIQVATSKETCLKVPPGGRGRV
IDIRLISQEDNSANTAQIIHIYILQKRKIQIGDKVAGRHGNKGIISKILPRQDMPFLQDG
TPIDMILSPLGVPSRMNVGQIFECLLGLAGSFLHKNYRIIPFDERYEREASRKLVFSELY
KASKKTTNPWLFEPDNPGKNRLIDGRTGEIFEQPITIGKAYMLKLIHQVDDKIHARSSGP
YALVTQQPLRGRSRRGGQRVGEMEVWALEGFGVAYILQEMLTIKSDHIRARYEVLGAIVT
GEPIPKPNTAPESFKLLVRELRSLALEINHVIICEKNLKLKLKEI
>P06273 | RPOC1_MARPO
MTYQKKHQHLRIELASPEQIRNWAERVLPNGEIVGQVTKPYTLHYKTHKPEKDGLFCEKI

FGPIKSGICACGKYQGIEKKKENIKFCEQCGVEFIESRIRRYRMGYIKLACSVTHVWYLK
RLPSYIANLLAKPLKELESLVYCDLFLARPITKKPTLLKLQGLFKYEDQSWKDIFPRFFS
PRGFEVFQNREIATGGDAIQKQLTNLNLQNVINLAHLEWKEFAEQKSTGNEWEDRKIQRR
KDLLVRRIKLAKHFIQTNIKPEWMVLSLLPVLPPELRPMIELGEGELITSDLNELYRRVI
YRNNTLLDFLARSGSTPGGLVVCQKRLVQEAVDALIDNGIRGQPMKDSHNRPYKSFSDLI
EGKEGRFRENLLGKRVDYSGRSVIVVGPFLPLHQCGLPREMAIELFQAFVIRGLIGRNFA
PNLRAAKTMIQNKEPIIWKVLQEVMQGHPILLNRAPTLHRLGIQAFQPILVNGRAIHLHP
LVCGGFNADFDGDQMAVHIPLSLEAQAEARLLMLSHKNLLSPATGEPISVPSQDMLLGLY
ILTIENNQGIYGNKYNPSKKYDSKKKFSQIPYFSSYDNVFRALQQKQIYLHSSLWLRWQI
NLRIITLLNQEGPIEIQYKSFGNSFQIYEHYQLRKNKNQEIISTYICTTAGRILFNQQIE
EAIQGTYKASLKQKTFVQKIEKNG
>P06356 | RR3_MARPO
MGQKINPLGFRLGITQNHRSYWFANKKYSKVFEEDKKIRDCIELYVQKHIKNSSNYGGIA
RVEIKRKTDLIQVEIYTGFPALLVESRGQGIEQLKLNVQNILSSEDRRLRMTLIEIAKPY
GEPKILAKKIALKLESRVAFRRTMKKAIELAKKGNIKGIKIQIAGRLNGAEIARVEWARE
GRVPLQTIRARINYCYYAAQTIYGVLGIKVWIFQDEE
>P06357 | RR3_TOBAC
MGQKINPLGFRLGTTQGHHSLWFSQPKNYSEGLQEDQKIRDCIKNYVQKNMRTSSGVEGI
ARIEIQKRIDLIQVIIFMGFPKLLIESRPRGIEELQTTLQKEFHCVNRKLNIAVTRIAKP
YGNPNILAEFIAGQLKNRVSFRKAMKKAIELTEQADTKGIQIQIAGRIDGKEIARVEWIR
EGRVPLQTIRAKIDYCSYTVRTIYGVLGIKIWIFLDEE
>P06359 | RR4_TOBAC
MSRYRGPRFKKIRRLGALPGLTNKKPRNGSDLRNQSRSGKKSQYRIRLEEKQKLRFHYGL
TERQLLKYVRIARKAKGSTGQVLLQLLEMRLDNILFRLGMASTIPAARQLVNHRHILVNG
RIVDIPSYRCKPRDIITAKDEQKSRALIQISLDSSPHEELPNHLTLHPFQYKGLVNQIID
SKWVGLKINELLVVEYYSRQT
>P06360 | RR7_MARPO
MSRKSIAEKQVAKPDPIYRNRLVNMLVNRILKNGKKSLAYRILYKAMKNIKQKTKKNPLF
VLRQAVRKVTPNVTVKARRIDGSTYQVPLEIKSTQGKALAIRWLLGASRKRSGQNMAFKL
SYELIDAARDNGIAIRKKEETHKMAEANRAFAHFR
>P06362 | RR8_MARPO
MGNDTIANMITSIRNANLGKIKTVQVPATNITRNIAKILFQEGFIDNFIDNKQNTKDILI
LNLKYQGKKKKSYITTLRRISKPGLRIYSNHKEIPKVLGGMGIVILSTSRGIMTDREARQ
KKIGGELLCYVW
>P06373 | RR15_TOBAC
MVKNSVISVISQEEKRGSVEFQVFNFTNKIRRLTSHLELHKKDYLSQRGLKKILGKRQRL
LAYLSKKNRVRYKELINQLDIRETKTR
>P06378 | RK2_MARPO
MAIRLYRAYTPGTRNRSVPKFDEIVKCQPQKKLTYNKHIKKGRNNRGIITSQHRGGGHKR
LYRKIDFQRNKKYITGKIKTIEYDPNRNTYICLINYEDGEKRYILYPRGIKLDDTIISSE
EAPILIGNTLPLTNMPLGTAIHNIEITPGKGGQLVRAAGTVAKIIAKEGQLVTLRLPSGE
IRLISQKCLATIGQIGNVDVNNLRIGKAGSKRWLGKRPKVRGVVMNPIDHPHGGGEGRAP
IGRKKPLTPWGHPALGKRSRKNNKYSDTLILRRRKNS
>P06382 | RK14_TOBAC

MIQPQTHLNVADNSGARELMCIRIIGASNRRYAHIGDVIVAVIKEAVPNMPLERSEVVRA
VIVRTCKELKRDNGMIIRYDDNAAVVIDQEGNPKGTRIFGAIARELRELNFTKIVSLAPE
VL
>P06384 | RK16_TOBAC
MLSPKRTRFRKQHRGRMKGISHRGNHISFGKYALQALEPAWITSRQIEAGRRAMTRNARR
GGKIWVRIFPDKPVTLRPAETRMGSGKGSPEYWVAVVKPGRILYEMGGVTENIARRAISL
AASKMPIRTQFIIS
>P06386 | RK20_TOBAC
MTRIKRGYIARRRRTKIRLFASSFRGAHSRLTRTITQQKIRALVSAHRDRDRKKRDFRRL
WITRINAVIRERGVSYSYSRLIHDLYKRQLLLNRKILAQIAISNRNCLYMISNEIIKEVD
WKESTRII
>P06388 | RK22_MARPO
MQTNTSNKKIRAVAKHIHMSPHKVRRVVSQIRGRSYEQALMILEFMPYRACNPILQLLSS
AAANANHNFGLSKTNLFISEIQVNKGTFFKRFQPRAQGRGYPIHKPTCHITIVLNILPK
>P06506 | RR11_SPIOL
MAKPIPKIGSRRNGRISSRKSARKIPKGVIHVQASFNNTIVTVTDVRGRVVSWASAGTCG
FRGTKRGTPFAAQTAAGNAIRTVVEQGMQRAEVMIKGPGLGRDAALRAIRRSGILLSFVR
DVTPMPHNGCRPPKKRRV
>P06586 | RR3_MAIZE
MGQKINPLGFRLGTTQNHHSFWFAQPKNYSEGLQEDKKIRNCIKNYIQKNRKKGSNRKME
SDSSSEVITHIEIQKEIDTIHVIIHIGFPNLLKKKGAIEELEKDLQKEVNSVNQRLNIAI
EKVKEPYRQPNILAEYIAFQLKNRVSFRKAMKKAIELTKKADIKGIKIQIAGRLAGKEIA
RAECIKKGRLPLQTIRAKIDYCCYPIRTIYGVLGVKIWIFVEEE
>P07088 | ACP_BRACM
MSTTFCSSVSMQATSLAATTRISFQKPALVSTTNLSFNLRRSIPTRFSISCAAKPETVEK
VSKIVKKQLSLKDDQKVVAETKFADLGADSLDTVEIVMGLEEEFDIEMAEEKAQKIATVE
EAAELIEELVQLKK
>P07089 | RBS_FLATR
MASIPATVAAVPQTNMVAPFTGLKANAAFPVTKKVNGFSTLPSNGGRVQCMKVWPPVGKK
KYETLSYLPELTEAQLAKEVDYLLRNKWVPCLEFELEHGFVYRENASSPGYYDGRYWTMW
KLPMFGCTDSAQVMKELQECKKEYPQAWIRIIGFDNVRQVQCVSFIASKPTGF
>P07689 | RBS3_PEA
MASMISSSAVTTVSRASTVQSAAVAPFGGLKSMTGFPVKKVNTDITSITSNGGRVKCMQV
WPPIGKKKFETLSYLPPLTRDQLLKEVEYLLRKGWVPCLEFELEKGFVYREHNKSPGYYD
GRYWTMWKLPMFGTTDASQVLKELDEVVAAYPQAFVRIIGFDNVRQVQCISFIAHTPESY

>P07838 | FER_BRYMA
ASYKVTLKLDDGSEAVIDCDEDSFILDVAEEEGIDIPFSCRSGSCSTCAGKIEGGTVDQS
EQTFLDDDQMEEGYVLTCVAYPTSDCTILTHQEEEMIG
>P07839 | FER_CHLRE
MAMAMRSTFAARVGAKPAVRGARPASRMSCMAYKVTLKTPSGDKTIECPADTYILDAAEE
AGLDLPYSCRAGACSSCAGKVAAGTVDQSDQSFLDDAQMGNGFVLTCVAYPTSDCTIQTH
QEEALY
>P08211 | RBL_CHLMO

MVPQTETKAGAGFKAGVKDYRLTYYTPDYVVKDTDILAAFRMTPQPGVPAEECGAAVAAE
SSTGTWTTVWTDGLTSLDRYKGRCYDIEPVPGEDNQYIAYVAYPIDLFEEGSVTNLFTSI
VGNVFGFKALRALRLEDLRIPPAYVKTFSGPPHGIQVERDKINKYGRGLLGCTIKPKLGL
SAKNYGRAVYECLRGGLDFTKDDENVNSQPFMRWRDRFLFCAEAIYKAQAETGEVKGHYL
NATAGTSEEMIKRAVCAKEFGVPIIMHDYLTGGFTANTSLSNYCRDHGLLLHIHRAMHAV
IDRQRNHGIHFRVLAKALRMSGGDHLHSGTVVGKLEGEREVTLGFVDLMRDNYIEKDRSR
GIYFTQDWCSMAGVMPVASGGIHVWHMPALVEIFGDDACLQFGGGTLGHPWGNAPGAVAN
RVALEACTQARNEGRDLAREGGDVIRSACKWSPELAAACEVWKEIKVEFDTIDKL
>P08241 | RR2_PEA
MTKRYWNITFEEMMEAGVHFGHDTRKWNPRMAPFISAKRKGIHITNLTKTARFLSEACDL
AFDAASKGKQFLIVGTKKKAADSVTRAAIRARCHYVNKKWLRGMLTNWYTTETRLGKFRD
LRTEQKTGKLNSLPKRDAAMLKRQLSHFETYLGGIKYMTGLPDIVIIVDQQKEYTALQEC
ITLGIPTICLIDTNCDPDLADMSIPANDDAIASIRLILNKLVFAICEGRSSSIRNY
>P08242 | RR2_SPIOL
MTRRYWNINLEEMMEAGVHFGHGTRKWNPRMSPYISAKCKGIHIINLTRTARFLSEACDL
VFDASSRGKQFLIVGTKNKAADSVARAAIRARCHYVNKKWLGGMLTNWSTTETRLHKFRD
LRMEQTAGRLARLPKRDAAVVKRQLSHLQTYLGGIKYMTGLPDIVIIVDQQEEYTALREC
ITLGIPTICLIDTNCNPDLADISIPANDDAIASIRLILTKLVFAICEGRSSYIRNP
>P08527 | RR14_MAIZE
MAKKSLIQREKKRQKLEQKYHLIRRSSKKKIRSKVSPLSLSEKTKMQEKLQSLPRNSAPT
RLHRRCFLTGRPRANYRDFGLSGHILREMVYACLLPGATRSSW
>P08528 | RK16_MAIZE
MLSPKRTRFRKQHRGRMKGKSCRGNHICFGRYALQVLEPAWITARQIEAGRRAMTRYARR
GGKIWVRIFPDKPVTIRPTETRMGSGKGSPEYWVAVVKPGRILYEMSGVSETVARAAISI
AASKMPIRSQFLRLEI
>P08529 | RK14_MAIZE
MIQPQTLLNVADNSGARKLMCIRVIGAAGNQRYARIGDVIIAVIKDAVPQMPLERSEVIR
AVIVRTRKEFKGDDGIIIRYDDNAAVIIDQKGNPKGTRVFGAVAEELRELNLTKIVSLAP
EVL
>P08530 | RR8_MAIZE
MGKDTIADLLTSIRNADMNKKGTVRVVSTNITENIVKILLREGFIESVRKHQESNRYFLV
STLRHQRRKTRKGIYRTRTFLKRISRPGLRIYANYQGIPKVLGGMGIAILSTSRGIMTDR
EARLNRIGGEVLCYIW
>P08698 | IF1C_SPIOL
MKEQKWIHEGLITESLPNGMFWVRLDNEDPILGYVSGRIRRSSIRILPGDRVKIEVSRYD
STRGRIIYRLRNKDSND
>P08705 | RBS_HELAN
MASISSSVATVSRTAPAQANMVAPFTGLKSNAAFPTTKKANDFSTLPSNGGRVQCMKVWP
PLGLKKYETLSYLPPLTETQLAKEVDYLLRKKWVPCLEFELEHGFVYRENARSPGYYDGR
YWTMWKLPMFGCTDSAQVMKELAECKKEYPQAWIRIIGFDNVRQVQCIMFIASRPDGY
>P08706 | RBS1_SOLLC
MASSIVSSAAAATRSNVAQASMVAPFTGLKSAASFPVTKKNNNVDITSLASNGGRVRCMQ
VWPPINMKKYETLSYLPDLSDEQLLSEIEYLLKNGWVPCLEFETERGFVYRENNSSPGYY
DGRYWTMWKLPMFGCTDATQVLAEVQEAKKAYPQAWVRIIGFDNVRQVQCISFIAYKPEG

F
>P08817 | ACP2_HORVU
MASAAASAVSFARPVKAICVNSVSFSALRKDNVSFRLQPVPQRFSVCCAAKKETVEKVCD
IVKSQLALSDDTEVSGSSTFADLGADSLDTVEIVMGLEEAFGISVEESSAQTIATVEDAA
NLIDSLVGK
>P08974 | YCF15_SPIOL
MLLLKHGRIEILDQNTMYGWYELPKQEFLNSEQPEPITHSIKKFPLMKDVNPLENQKYAC
LMK
>P09364 | MATK_SINAL
MEKFQGYLEFDGARQQSFLYPLFFREYIYVLSYDHGLNRLNRNRSIFLENADYDKKYSSL
IVKRLILRMYEQNRLIIPTKDLNTNLGHTNLFYYQMISVLFAVIVEIPFSLRLGSSFEGK
NLKKSYNLQSIHSIFPFLEDKFSHFNYVLDVLIPYPIHLEILVQTLRYRVKDASSLHFFR
FCLYEYCNWKNFDSKKKSILNPRFFLFLYNSHVCEYESIFFFLRKQSSHLRSTSYEVFFE
RILFYGKIQHFLKVFVNNFPAILGLLKDPFLHYVRYHGKYILATKDTPLMMNKWKYYFVN
LWQCYFSVWFQSQKININQLSKDNFEFLGYFSSLRLNSLVVRSQMLENSFLIDNVRRKLD
SNIQISSIIGSLAKDKFCNVLGHPISKATWMDSSDSDILNRFVRICRNISHYYSGSAKKK
NLYRIKYILRLCCVKTLARKHKSTVRAFLKRLGSGLLEEFLTGEDQVLSLIFPRSDYASK
RLYRVRVWYLDILYLNDLVNHE
>P09594 | RK22_SPIOL
MGFFKKKEKKAEFDVFRLYGLHHPEPGKCDEITTRGYSISMSVDKARRVIDQIRGRSYAE
TLMILELMPYRACYPIFKLIYSAAANASHNKQFNKANLIISKAEVNKGITLKKVKPRARG
RSYMIKRPTCHITIVLRDITHFDSYDKFLESLTPKKLIALLGLMSTGRRRELLCGRFREN
HKIKSFLYKIALFKRYEVM
>P09597 | RR8_SPIOL
MGKDTIADIITCIRNADMNRKGTVRIVSTNITENIVKILLREGFIENARKHQERNKYFLV
LTLRHRRNKKGPYLNTFHLKRVSRPGLRIYSNYQRIPRILGGMGIAILSTSRGIMTDREA
RLEGIGGEILCYIW
>P09735 | FER_MARPO
TFKVTLNTPTGQSVIDVEDDEYILDAAEEAGLSLPYSCRAGACSSCAGKVTAGEVDQSDE
SFLDDDQMDEGYVLTCIAYPTSDLTIDTHQEEALI
>P09842 | SSG1_HORVU
MAALATSQLATSGTVLGVTDRFRRPGFQGLRPRNPADAALGMRTIGASAAPKQSRKAHRG
SRRCLSVVVSATGSGMNLVFVGAEMAPWSKTGGLGDVLGGLPPAMAANGHRVMVVSPRYD
QYKDAWDTSVISEIKVADEYERVRFFHCYKRGVDRVFIDHPWFLEKVRGKTKEKIYGPDA
GTDYEDNQQRFSLLCQAALEAPRILNLNNNPYFSGPYGEDVVFVCNDWHTGLLACYLKSN
YQSNGIYRTAKVAFCIHNISYQGRFSFDDFAQLNLPDRFKSSFDFIDGYDKPVEGRKINW
MKAGILQADKVLTVSPYYAEELISGEARGCELDNIMRLTGITGIVNGMDVSEWDPTKDKF
LAVNYDITTALEAKALNKEALQAEVGLPVDRKVPLVAFIGRLEEQKGPDVMIAAIPEILK
EEDVQIILLGTGKKKFEKLLKSMEEKFPGKVRAVVRFNAPLAHQMMAGADLLAVTSRFEP
CGLIQLQGMRYGTPCVCASTGGLVDTIVEGKTGFHMGRLSVDCNVVEPADVKKVATTLKR
AVKVVGTPAYQEMVKNCMIQDLSWKGPAKNWEDVLLELGVEGSEPGIVGEEIAPLAMENV
AAP
>P0C2Y3 | ACCD_ORYSI
MALQSLRGSMRSVVGKRICPLIEYAIFPPLPRIIVYASRRARMQRGNYSLIKKPKKVSTL

RQYQSTKSPMYQSLQRICGVREWLNKYCMWKEVDEKDFGFEIGAFD
>P0C305 | YCF68_ORYSI
MAYSSCLNRSLKPNKLLLRRIDGAIQVRSHVDLTFYSLVGSGRSGGGTTAPLFSRIHTSL
ISVWRAISRAQVEVRPQWENGAPNNASSQTKNYEITLSFWGDGGIVPFEPFFHAFPGGLE
KAAINRTSLILPS
>P0C312 | CLPP_ORYSA
MPIGVPKVPYRIPGDEEATWVDLYNVMYRERTLFLGQEIRCEVTNHITGLMVYLSIEDGI
SDIFLFINSPGGWLISGMAIFDTMQTVTPDIYTICLGIAASMASFILLGGEPTKRIAFPH
ARIMLHQPASAYYRARTPEFLLEVEELHKVREMITRVYALRTGKPFWVVSEDMERDVFMS
ADEAKAYGLVDIVGDEMLDEHCDTDPVWFPEMFKDW
>P0C313 | CLPP_ORYSI
MPIGVPKVPYRIPGDEEATWVDLYNVMYRERTLFLGQEIRCEVTNHITGLMVYLSIEDGI
SDIFLFINSPGGWLISGMAIFDTMQTVTPDIYTICLGIAASMASFILLGGEPTKRIAFPH
ARIMLHQPASAYYRARTPEFLLEVEELHKVREMITRVYALRTGKPFWVVSEDMERDVFMS
ADEAKAYGLVDIVGDEMLDEHCDTDPVWFPEMFKDW
>P0C321 | NU3C_ORYSI
MFLLHEYDIFWAFLIIASLIPILAFWISALLAPVREGPEKLSSYESGIEPMGGAWLQFRI
RYYMFALVFVVFDVETVFLYPWAMSFDVLGISVFIEAFIFVLILVVGLVYAWRKGALEWS

>P0C322 | NU3C_ORYSJ
MFLLHEYDIFWAFLIIASLIPILAFWISALLAPVREGPEKLSSYESGIEPMGGAWLQFRI
RYYMFALVFVVFDVETVFLYPWAMSFDVLGISVFIEAFIFVLILVVGLVYAWRKGALEWS

>P0C324 | NU4C_ORYSI
MSSFPWLTILVVLPIFAGSLIFFLPHRGNKIVRWYTMSICLLEFLLMTYAFCYHFQLEDP
LIQLKEDSKWIDVFNFHWRLGIDGLSLGSILLTGFMTTLATLAAWPVTRNSRLFYFLMLA
MYSGQIGLFSSRDLLLFFIMWELELIPVYLLLSMWGGKRRLYSATKFILYTAGGSIFFLI
GVLGMGLYGSNEPRLDLERLINQSYPATLEILFYFGFLIAYAVKLPIIPLHTWLPDTHGE
AHYSTCMLLAGILLKMGAYGLIRINMELLPHAHYLFSPWLVIIGAMQIIYAASTSLGQRN
FKKRIAYSSVSHMGFIIIGIGSITNIGLNGAILQILSHGFIGATLFFLAGTACDRMRLVY
LEELGGVSIPMPKIFTMFSSFSMASLALPGMSGFVAELVVFFGLITSPKFLLMPKMLITF
VMAIGMILTPIYLLSMLRQMFYGYKLFHVPNENFEDSGPRELFLLICIFLPVIGIGIYPD
FVLSLSVDRVEALLSNYYPK
>P0C325 | NU4C_ORYSJ
MSSFPWLTILVVLPIFAGSLIFFLPHRGNKIVRWYTMSICLLEFLLMTYAFCYHFQLEDP
LIQLKEDSKWIDVFNFHWRLGIDGLSLGSILLTGFMTTLATLAAWPVTRNSRLFYFLMLA
MYSGQIGLFSSRDLLLFFIMWELELIPVYLLLSMWGGKRRLYSATKFILYTAGGSIFFLI
GVLGMGLYGSNEPRLDLERLINQSYPATLEILFYFGFLIAYAVKLPIIPLHTWLPDTHGE
AHYSTCMLLAGILLKMGAYGLIRINMELLPHAHYLFSPWLVIIGAMQIIYAASTSLGQRN
FKKRIAYSSVSHMGFIIIGIGSITNIGLNGAILQILSHGFIGATLFFLAGTACDRMRLVY
LEELGGVSIPMPKIFTMFSSFSMASLALPGMSGFVAELVVFFGLITSPKFLLMPKMLITF
VMAIGMILTPIYLLSMLRQMFYGYKLFHVPNENFEDSGPRELFLLICIFLPVIGIGIYPD
FVLSLSVDRVEALLSNYYPK
>P0C329 | NU6C_ORYSA

MDLPGPIHEILVLFGGFVLLLGGLGVVLLTNPTFSAFSLGLVLVCISLFYILLNSYFVAV
AQLLIYVGAINVLIIFAVMFVNGSEWSKDKNFWTIGDGFTSLVCITIPFSLMTTIPDTSW
YGILWTTRSNQIVEQGLINNVQQIGIHLATDFYLPFELISIILLVSLIGAITMARQ
>P0C331 | NU6C_ORYSJ
MDLPGPIHEILVLFGGFVLLLGGLGVVLLTNPTFSAFSLGLVLVCISLFYILLNSYFVAV
AQLLIYVGAINVLIIFAVMFVNGSEWSKDKNFWTIGDGFTSLVCITIPFSLMTTIPDTSW
YGILWTTRSNQIVEQGLINNVQQIGIHLATDFYLPFELISIILLVSLIGAITMARQ
>P0C333 | NU4LC_ORYSI
MMFEHVLFLSVYLFSIGIYGLITSRNMVRALICLELILNSINLNLVTFSDLFDSRQLKGD
IFAIFVIALAAAEAAIGLSILSSIHRNRKSTRINQSNFLNN
>P0C334 | NU4LC_ORYSJ
MMFEHVLFLSVYLFSIGIYGLITSRNMVRALICLELILNSINLNLVTFSDLFDSRQLKGD
IFAIFVIALAAAEAAIGLSILSSIHRNRKSTRINQSNFLNN
>P0C335 | NDHH_ORYSA
MSLPLTRKDLMIVNMGPQHPSMHGVLRLIVTLDGEDVIDCEPILGYLHRGMEKIAENRTI
IQYLPYVTRWDYLATMFTEAITVNAPEFLENIQIPQRASYIRVIMLELSRIASHLLWLGP
FMADLGAQTPFFYIFRERELIYDLFEAATGMRMMHNYFRIGGVAADLPYGWIDKCLDFCD
YFLRGVIEYQQLITQNPIFLERVEGVGFISGEEAVNWGLSGPMLRASGIQWDLRKVDLYE
SYNQFDWKVQWQKEGDSLARYLVRIGEMRESIKIIQQAVEKIPGGPYENLEVRRFKKAKN
SEWNDFEYRFLGKKPSPNFELSKQELYARVEAPKGELGIYLVGDDSLFPWRWKIRPPGFI
NLQILPQLVKKMKLADIMTILGSIDIIMGEVDR
>P0C336 | NDHH_ORYSI
MSLPLTRKDLMIVNMGPQHPSMHGVLRLIVTLDGEDVIDCEPILGYLHRGMEKIAENRTI
IQYLPYVTRWDYLATMFTEAITVNAPEFLENIQIPQRASYIRVIMLELSRIASHLLWLGP
FMADLGAQTPFFYIFRERELIYDLFEAATGMRMMHNYFRIGGVAADLPYGWIDKCLDFCD
YFLRGVIEYQQLITQNPIFLERVEGVGFISGEEAVNWGLSGPMLRASGIQWDLRKVDLYE
SYNQFDWKVQWQKEGDSLARYLVRIGEMRESIKIIQQAVEKIPGGPYENLEVRRFKKAKN
SEWNDFEYRFLGKKPSPNFELSKQELYARVEAPKGELGIYLVGDDSLFPWRWKIRPPGFI
NLQILPQLVKKMKLADIMTILGSIDIIMGEVDR
>P0C338 | NDHJ_ORYSA
MQQGWLSNWLVKHEVVHRSLGFDHRGIETLQIKAEDWDSIAVILYVYGYNYLRSQCAYDV
APGGSLASVYHLTRIQYGIDNPEEVCIKVFAQKDNPRIPSVFWIWRSSDFQERESFDMVG
ISYDNHPRLKRILMPESWIGWPLRKDYITPNFYEIQDAH
>P0C341 | NDHK_ORYSA
MVLTEYSDKKKKEGKDSIKTVMSLIEFPLLDQTSSNSVISTTLKDLSNWSRLSSLWPLLY
GTSCCFIEFASLIGSRFDFDRYGLVPRSSPRQADLILTAGTVTMKMAPSLVRLYEQMPEP
KYVIAMGACTITGGMFSTDSYSTVRGVDKLIPVDVYLPGCPPKPEAVIDALTKLRKKISR
EIVEDRTLSQKKNRCFTTSHKLYVRRSTNTGTYEQELLYQSPSTLDISSETFFKSKSPVS
SYKLVN
>P0C375 | CCSA_ORYSA
MLFATLEHILTHISFSTISIVITIHLITLLVRELGGLRDSSEKGMIATFFCITGFLVSRW
ASSGHFPLSNLYESLIFLSWALYILHMIPKIQNSKNDLSTITTPSTILTQGFATSGLLTE
MHQSTILVPALQSQWLMMHVSMMLLSYATLLCGSLLSAALLMIRFRKNLDFFSKKKKNVL
SKTFFFNEIEYFYAKRSALKSTFFPLFPNYYKYQLIERLDSWSYRVISLGFTLLTIGILC

GAVWANEAWGSYWNWDPKETWAFITWTIFAIYLHSRTNPNWKGTKSAFVASIGFLIIWIC
YFGINLLGIGLHSYGSFTLPI
>P0C379 | IF1C_ORYSI
MTEKKNRREKKNPREAKITFEGLVMEALPNGMFRVRLENDTIILGYISGKIRSSSIRILM
GDRVKIEVSRYDSSKGRIIYRLPHKDSKRTEDSKDTEDLKDTKDSKG
>P0C381 | MATK_ORYSA
MEKFEGYSEKLKFPRQYFVYPLLFQEYIYVFAHDYGLNGSELVEIIGSNNKKFSSLLVKR
LMIRMYQQNFWINLVNHPNQDRLLDYNNFFYSEFYSQILSEGFAIVVEIPFSLREQSCPE
EKEIPKFQNLRSIHSIFPFLEDKFLHLHYLAHIEIPYPIHLDILLQLLQYRIQDVPSLHL
LRFFLNYYSNWNSFITSMKSIFILKKENKRLFRFLYNSYVSEYEFFLLFLRKQSSCLRLT
SSGTFLERIIFSRKMEHFGLMYPAFFRKTIWFVMDPLMHYVRYQGKAILASKGTLLLKKK
WKCYLVRLWQYSFSFWTQPQRIHLNQLENSCFDFLGYFSSVPINSLLVRNQMLENSFLID
TQMKKFDTKVPVTPLIGSLAKAQFCTGSGHPISKPIWTDLSDWDILDRFGRICRNLFHYH
SGSSKKKTLYRLKYILRLSCARTLARKHKSTVRAFMQWLGSVFLEEFFTEEEQVFSLMFA
KTTYFSFRGSHSERIWYLDILRINDLVNPLN
>P0C408 | PSBK_ORYSA
MPNILSLTCICFNSVIYPTSFFFAKLPEAYAIFNPIVDFMPVIPVLFFLLAFVWQAAVSF
R
>P0C439 | RK14_ORYSI
MIQPQTLLNVADNSGARKLMCIRVIGAASNQRYARIGDVIVAVIKDAVPQMPLERSEVIR
AVIVRTCKEFKCEDGIIIRYDDNAAVIIDQKGNPKGTRVFGAIAEELRELNFTKIVSLAP
EVL
>P0C440 | RK14_ORYSJ
MIQPQTLLNVADNSGARKLMCIRVIGAASNQRYARIGDVIVAVIKDAVPQMPLERSEVIR
AVIVRTCKEFKCEDGIIIRYDDNAAVIIDQKGNPKGTRVFGAIAEELRELNFTKIVSLAP
EVL
>P0C449 | RK23_ORYSA
MDGIKYAVFTEKSLRLLGKNQYTFNVESGFTKTEIKHWVELFFGVKVVAVNSHRLPGKGR
RMGPILGHTMHYRRMIITLQPGYSIPLLDREKN
>P0C453 | RK32_ORYSI
MAVPKKRTSMSKKRIRKNLWKKKTYFSIVQSYSLAKSRSFSGVSEHPKPKGFSRQQTNK
>P0C456 | RK33_ORYSI
MAKGKDVRIRVILQCVSCVRKGANEESAGISRYSTQKNRHNTPGQLELRKFCRYCRKHTI
HAEIKK
>P0C457 | RK33_ORYSJ
MAKGKDVRIRVILQCVSCVRKGANEESAGISRYSTQKNRHNTPGQLELRKFCRYCRKHTI
HAEIKK
>P0C460 | RR12_ORYSA
MPTVKQLIRNARQPIRNARKSAALKGCPQRRGTCARVYTINPKKPNSALRKVARVRLTSG
FEITAYIPGIGHNLQEHSVVLVRGGRVKDLPGVRYRIIRGALDAVAVKNRQQGRSKYGVK
KPKK
>P0C463 | RR11_ORYSI
MTKAIPKIGSRRKVRIGLRRNARFSLRKSARRITKGVIHVQASFNNTIITVTDPQGRVVF
WSSAGTCGFKSSRKASPYAGQRTAVDAIRTVGLQRAEVMVKGAGSGRDAALRAIAKSGVR

LSCIRDVTPMPHNGCRPPKKRRL
>P0C473 | RR16_ORYSA
MLKLRLKRCGRKQRAVYRIVAIDVRSRREGRDLRKVGFYDPIKNQTCLNVPAILYFLEKG
AQPTRTVSDILRKAEFFKEKERTLS
>P0C474 | RR16_ORYSI
MLKLRLKRCGRKQRAVYRIVAIDVRSRREGRDLRKVGFYDPIKNQTCLNVPAILYFLEKG
AQPTRTVSDILRKAEFFKEKERTLS
>P0C475 | RR18_ORYSA
MYTSKQPFHKSKQTFHKSKQTFRKSKQTFRKFKQPFRKPKQPFRRRPRIGPGDRIDYRNM
SLINRFISEQGKILSRRINRLTLKQQRLITLAIKQARILSFLPFRNYENEKQFQAQSISI
ITGPRPRKNRHIPPLTQKFNSNRNLRNSNQTLRNNNRNLSSDC
>P0C477 | RR18_ORYSJ
MYTSKQPFHKSKQTFHKSKQTFRKSKQTFRKFKQPFRKPKQPFRRRPRIGPGDRIDYRNM
SLINRFISEQGKILSRRINRLTLKQQRLITLAIKQARILSFLPFRNYENEKQFQAQSISI
ITGPRPRKNRHIPPLTQKFNSNRNLRNSNQTLRNNNRNLSSDC
>P0C478 | RR19_ORYSA
MTRKKTNPFVAHHLLAKIEKVNMKEEKETIVTWSRASSILPAMVGHTIAIHNGKEHIPIY
ITNPMVGRKLGEFVPTRHFTSYESARKDTKSRR
>P0C484 | RR3_ORYSI
MGQKINPLGFRLGTTQNHHSFWFAQPKNYSEGLQEDKKIRNCIKNYIQKNRKKGSNRKIE
ADSSFEVITHNKKMDSGSSSEVITHIEIQKEIDTIHVIIHIGFPNLLKKKGAIEELEKDL
QKEVNSVNQRLNIGIEKVKEPYRQPNILAEYIAFQLKNRVSFRKAMKKAIELTKKTDIKG
VKVKIAGRLAGKEIARAECIKKGRLPLQTIRAKIDYCCYPIRTIYGVLGVKIWIFVDEE
>P0C485 | RR3_ORYSJ
MGQKINPLGFRLGTTQNHHSFWFAQPKNYSEGLQEDKKIRNCIKNYIQKNRKKGSNRKIE
ADSSFEVITHNKKMDSGSSSEVITHIEIQKEIDTIHVIIHIGFPNLLKKKGAIEELEKDL
QKEVNSVNQRLNIGIEKVKEPYRQPNILAEYIAFQLKNRVSFRKAMKKAIELTKKTDIKG
VKVKIAGRLAGKEIARAECIKKGRLPLQTIRAKIDYCCYPIRTIYGVLGVKIWIFVDEE
>P0C487 | RR4_ORYSI
MSRYRGPRFKKIRRLGALPGLTRKTPKSGSNLKKKFHSGKKEQYRIRLQEKQKLRFHYGL
TERQLLRYVHIAGKAKSSTGQVLLQLLEMRLDNILFRLGMASTIPEARQLVNHRHILVNG
RIVDIPSFRCKPRDIITTKDNQRSKRLVQNSIASSDPGKLPKHLTIDTLQYKGLVKKILD
RKWVGLKINELLVVEYYSRQT
>P0C488 | RR4_ORYSJ
MSRYRGPRFKKIRRLGALPGLTRKTPKSGSNLKKKFHSGKKEQYRIRLQEKQKLRFHYGL
TERQLLRYVHIAGKAKSSTGQVLLQLLEMRLDNILFRLGMASTIPEARQLVNHRHILVNG
RIVDIPSFRCKPRDIITTKDNQRSKRLVQNSIASSDPGKLPKHLTIDTLQYKGLVKKILD
RKWVGLKINELLVVEYYSRQT
>P0C491 | RR7_ORYSJ
MSRRGTAEKRTAKSDPIFRNRLVNMVVNRIMKDGKKSLAYQILYRAVKKIQQKTETNPLL
VLRQAIRRVTPNIGVKTRRNKKGSTRKVPIEIGSKQGRALAIRWLLEASQKRPGRNMAFK
LSSELVDAAKGGGGAIRKKEATHRMAEANRALAHFR
>P0C492 | RR8_ORYSA
MGKDTIADLLTSIRNADMNKKGTVRVVSTNITENIVKILLREGFIESVRKHQESNRYFLV

STLRHQKRKTRKGIYRTRTFLKRISRPGLRIYANYQGIPKVLGGMGIAILSTSRGIMTDR
EARLNRIGGEVLCYIW
>P0C498 | RPOA_ORYSA
MVREEVAGSTQTLQWKCVESRVDSKRLYYGRFILSPLRKGQADTVGIALRRALLGETEGT
CITHAKFGSVPHEYSTIAGIEESVQEILLNLKEIVLRSNLYGVRTASICVKGPRYITAQD
IILPPSVEIVDTAQPIANLTEPTDFRIELRIKRDRGYHTEVRKNTQDGSYPIDAVSMPVR
NVNYSIFACGNGNAKYEILFLEIWTNGSLTPKEALYEASRNLIDLFLPFLHTEEEGTRFQ
ENKNRFTSPLLSFQKRLTNLKKNKKRIPLNCIFIDQLELPSRTYNCLKRANIHTLLDLLS
KTEEDLMRIDSFRMQDGKQIWDTLEKHLPMDLPKNKF
>P0C500 | RPOA_ORYSJ
MVREEVAGSTQTLQWKCVESRVDSKRLYYGRFILSPLRKGQADTVGIALRRALLGETEGT
CITHAKFGSVPHEYSTIAGIEESVQEILLNLKEIVLRSNLYGVRTASICVKGPRYITAQD
IILPPSVEIVDTAQPIANLTEPTDFRIELRIKRDRGYHTEVRKNTQDGSYPIDAVSMPVR
NVNYSIFACGNGNAKYEILFLEIWTNGSLTPKEALYEASRNLIDLFLPFLHTEEEGTRFQ
ENKNRFTSPLLSFQKRLTNLKKNKKRIPLNCIFIDQLELPSRTYNCLKRANIHTLLDLLS
KTEEDLMRIDSFRMQDGKQIWDTLEKHLPMDLPKNKF
>P0C501 | RPOB_ORYSA
MLRNGNEGMSTIPGFSQIQFEGFCRFINQGLAEELEKFPTIKDPDHEISFQLFAKGYQLL
EPSIKERDAVYESLTYSSELYVSARLIFGFDVQKQTISIGNIPIMNSLGTFIINGIYRIV
INQILLSPGIYYRSELDHKGISIYTGTIISDWGGRSELAIDKKERIWARVSRKQKISILV
LSSAMGSNLKEILDNVSYPEIFLSFPNAKEKKRIESKEKAILEFYQQFACVGGDLVFSES
LCEELQKKFFQQKCELGRIGRRNMNRRLNLDIPQNSTFLLPRDVLAATDHLIGMKFETGI
LDDDDMNHLKNKRIRSVADLLQDQFGLALGRLQHAVQKTIRRVFIRQSKPTPQTLVTPTS
TSILLITTYETFFGTYPLSQVFDQTNPLTQTVHGRKVSCLGPGGLTGRTASFRSRDIHPS
HYGRICPIDTSEGINVGLTGSLAIHARIDHWWGSVESPFYEISEKAKKKKERQVVYLSPN
RDEYYMIAAGNSLSLNRGIQEEQVVPARYRQEFLTIAWEQIHVRSIFPFQYFSIGGSLIP
FIEHNDANRALMSSNMQRQAVPLSRSEKCIVGTGLERQTALDSRVSVIAEREGKIISTNS
HKILLSSSGKTISIPLVTHRRSNKNTCMHQKPRVPRGKSIKKGQILAEGAATVGGELALG
KNVLVAYMPWEGYNFEDAVLISERLVYEDIYTSFHIRKYEIQTDTTSQGSAEKITKEIPH
LEEHLLRNLDRNGVVKLGSWVETGDILVGKLTPQIASESSYIAEAGLLRAIFGLEVSTSK
ETSLKLPIGGRGRVIDVKWIQRDPLDIMVRVYILQKREIKVGDKVAGRHGNKGIISKILP
RQDMPYLQDGTPVDMVFNPLGVPSRMNVGQIFESSLGLAGDLLKKHYRIAPFDERYEQEA
SRKLVFSELYEASKQTKNPWVFEPEYPGKSRIFDGRTGDPFEQPVLIGKSYILKLIHQVD
EKIHGRSTGPYSLVTQQPVRGRAKQGGQRIGEMEVWALEGFGVAHILQEILTYKSDHLIA
RQEILNATIWGKRVPNHEDPPESFRVLVRELRSLALELNHFLVSQKNFQVNREEV
>P0C502 | RPOB_ORYSI
MLRNGNEGMSTIPGFSQIQFEGFCRFINQGLAEELEKFPTIKDPDHEISFQLFAKGYQLL
EPSIKERDAVYESLTYSSELYVSARLIFGFDVQKQTISIGNIPIMNSLGTFIINGIYRIV
INQILLSPGIYYRSELDHKGISIYTGTIISDWGGRSELAIDKKERIWARVSRKQKISILV
LSSAMGSNLKEILDNVSYPEIFLSFPNAKEKKRIESKEKAILEFYQQFACVGGDLVFSES
LCEELQKKFFQQKCELGRIGRRNMNRRLNLDIPQNSTFLLPRDVLAATDHLIGMKFETGI
LDDDDMNHLKNKRIRSVADLLQDQFGLALGRLQHAVQKTIRRVFIRQSKPTPQTLVTPTS
TSILLITTYETFFGTYPLSQVFDQTNPLTQTVHGRKVSCLGPGGLTGRTASFRSRDIHPS
HYGRICPIDTSEGINVGLTGSLAIHARIDHWWGSVESPFYEISEKAKKKKERQVVYLSPN

RDEYYMIAAGNSLSLNRGIQEEQVVPARYRQEFLTIAWEQIHVRSIFPFQYFSIGGSLIP
FIEHNDANRALMSSNMQRQAVPLSRSEKCIVGTGLERQTALDSRVSVIAEREGKIISTNS
HKILLSSSGKTISIPLVTHRRSNKNTCMHQKPRVPRGKSIKKGQILAEGAATVGGELALG
KNVLVAYMPWEGYNFEDAVLISERLVYEDIYTSFHIRKYEIQTDTTSQGSAEKITKEIPH
LEEHLLRNLDRNGVVKLGSWVETGDILVGKLTPQIASESSYIAEAGLLRAIFGLEVSTSK
ETSLKLPIGGRGRVIDVKWIQRDPLDIMVRVYILQKREIKVGDKVAGRHGNKGIISKILP
RQDMPYLQDGTPVDMVFNPLGVPSRMNVGQIFESSLGLAGDLLKKHYRIAPFDERYEQEA
SRKLVFSELYEASKQTKNPWVFEPEYPGKSRIFDGRTGDPFEQPVLIGKSYILKLIHQVD
EKIHGRSTGPYSLVTQQPVRGRAKQGGQRIGEMEVWALEGFGVAHILQEILTYKSDHLIA
RQEILNATIWGKRVPNHEDPPESFRVLVRELRSLALELNHFLVSQKNFQVNREEV
>P0C508 | RPOC2_ORYSI
MAERANLVFQNKEIDGTAMKRLISRLIDHFGMGYTSHILDQIKTLGFHQATTTSISLGIE
DLLTIPSKGWLVQDAEQQSFLLEKHYYYGAVHAVEKLRQSVEIWYATSEYLKHEMNSNFR
ITDPSNPVYLMSFSGARGNASQVHQLVGMRGLMADPQGQMIDLPIQSNLREGLSLTEYII
SCYGARKGVVDTAVRTADAGYLTRRLVEVVQHIIVRRRDCGTIQAISVSPQNGMTEKLFV
QTLIGRVLANDIYIGSRCIATRNQDIGIGLVNRFITTFRAQPFRAQPIYIRTPFTCRSTS
WICQLCYGRSSTHGDLVELGEAVGVIAGQSIGEPGTQLTLRTFHTGGVFTGGTADLVRSP
SNGKIQFNGDLVHPTRTRHGQPAFLCYIDLHITIQSQDILHSVTIPSKSLILVQNDQYVE
SEQVIAEIRAGTSALHFKEKVQKHIYSESDGEMHWSTDVYHAPEYQYGNLRRLPKTSHLW
ILSVSMCRSSIASFSLHKDQDQMNTYSFSVDGRYIFGLSMADDEVRHRLLDTFGKKDREI
LDYSTPDRIMSNGHWNFVYPSILQNNFDLLAKKRRNRFAIPLQYHQEQEKEPISCFGISI
EIPFMGVLRRNTIVAYFDDPRYKKDKKGSGIVKFRYRTLEDEYRTREKDSENEYGSPENE
YRTREEECKTLEDEYRTREEEYETLEDEYGIPENEYETLEDEYGILEDEYRTREEESEDE
YGSPENKYRPREDKYGTLEEDSEDEHGTLEEDSEEDSEDEYGNPEEDSVLKKGVLIEHRG
TKEFSLKYQKEVDRFFFILQELHILPRSSSLKVLDNSIIGVDTQLTKNTRSRLGGLVRVK
RKKSHTELKIFSGDIHFPEEADKILGGSLIPLEREKKDSKESKKRENWVYVQWKKILKSK
EKYFVLVRPAVAYEMNEGRNLATLFPQDLLQEEGNLQLRLVNFISHENSKLTQRIYHTNS
QFVRTCLVLNWEQEEKEEARASLVEIRANGLIRDFLRIGLIKSTISYTRKRYDSRSAGLI
LHNRLDRTNTNSFYSKAKIQSLSQHQEAIGTLLNRNKEYQSLMVLSASNCSRIGFFKNSK
NPNGVKESNPRIPIPKFLGLFRNFSGLLGTIAPSISNFSSSYYLLTYNQILLKKHLLLDN
LKQNFKVLQGLKHSLINENQRTSNFDSNIMLDPFQLNWHFLPHDSWEETSAKIHLGQFIC
ENVCLFKSHIKKSGQIFIVNIDSFVIRAAKPYLATTGATVHGHYGEILYKGDRLVTFIYE
KARSSDITQGLPKVEQIFEARSIDSLSPNLERRIEDWNERIPRILGGPWGFLIGAELTIA
QSRISLVNKIQKVYRSQGVQIHNRHIEIIIRQVTSKVRVSEDGMSNVFSPGELIGLLRAE
RAGRALDESIYYRAILLGITRVSLNTQSFISEASFQETARVLAKAALRGRIDWLKGLKEN
VVLGGIIPVGTGFQKFVHRYPQDKNLYFEIQKKKLFASEMRDILFLHTELVSSDSDVTNN
FYETSESPFTPFI
>P10797 | RBS2B_ARATH
MASSMFSSTAVVTSPAQATMVAPFTGLKSSASFPVTRKANNDITSITSNGGRVSCMKVWP
PIGKKKFETLSYLPDLSDVELAKEVDYLLRNKWIPCVEFELEHGFVYREHGNTPGYYDGR
YWTMWKLPLFGCTDSAQVLKEVEECKKEYPGAFIRIIGFDNTRQVQCISFIAYKPPSFTE
A
>P10798 | RBS3B_ARATH
MASSMLSSAAVVTSPAQATMVAPFTGLKSSAAFPVTRKTNKDITSIASNGGRVSCMKVWP

PIGKKKFETLSYLPDLSDVELAKEVDYLLRNKWIPCVEFELEHGFVYREHGNTPGYYDGR
YWTMWKLPLFGCTDSAQVLKEVEECKKEYPGAFIRIIGFDNTRQVQCISFIAYKPPSFTE
A
>P11422 | RBL_NICOT
MSPQTETKASVGFKAGVKEYKLTYYTPEYQTKDTDILAAFRVTPQPGVPPEEAGAAVAAE
SSTGTWTTVWTDGLTSLDRYKGRCYRIERVVGEKDQYIAYVAYPLDLFEEGSVTNMFTSI
VGNVFGFKALRALRLEDLRIPPAYVKTFQGPPHGIQVERDKLNKYGRPLLGCTIKPKLGL
SAKNYGRAVYECLRGGLDFTKDDENVNSQPFMRWRDRFLFCAEALYKAQTETGEIKGHYL
NATAGTCEEMIKRAVFARELGVPIVMHDYLTGGFTANTSLAHYCRDNGLLLHTHRAMHAV
IDRQKNHGIHFRVLAKALRMSGGDHIHSGTVVGKLEGERDITLGFVDLLRDDFVEQDRSR
GIYFTQDWVSLPGVLPVASGGIHVWHMPALTEIFGDDSVLQFGGGTLGHPWGNAPGAVAN
RLALEACVQARNEGRDLAQEGNEIIREACKWSPELAAACQVWKEIVFNFAAVDVLDK
>P11646 | NU4LC_MAIZE
MMFERVLFLSVYLFSIGIYGLITSRNMVRALICLELILNSINLNLVTFSDLFDSRQLKGD
IFAIFVIALAAAEAAIGLSILSSIHRNRKSTRINQSNFLNN
>P11705 | RPOC1_SPIOL
MIDQYKHQQLRIGSVSPQQISAWATKILPNGEIVGEVTKPYTFHYKTNKPEKDGLFCERI
FGPIKSGICACGNYRVIGDEKEDPKFCEQCGVEFVDSRIRRYQMGYIKLACPVTHVWYLK
RLPSYIANFLDKPLKELEGLVYCDFSFARPIAKKPTFLRLRGLFEYEIQSWKYSIPLFFT
TQGFDTFRNREISTGAGAIREQLADLDLRTIIDYSFAEWKELGEEGSTGNEWEDRKVGRR
KDFLVRRMELVKHFIRTNIEPEWMVLCLLPVLPPELRPIIQIDGGKLMSSDINELYRRVI
YRNNTLTDLLSTSRSTPGELVMCQEKLVQEAVDTLLDNGIRGQPMRDGHNKVYKSFSDVI
EGKEGRFRETLLGKRVDYSGRSVIVVGPSLSLHRCGLPREIAIELFQTFVIRGLIRQHLA
SNIGVAKRKIREKEPIVWKILQEVMQGHPVLLNRAPTLHRLGIQAFQPILVEGRAICLHP
LVCKGFNADFDGDQMAVHVPLSLEAQAEARLLMFSHMNLLSPAIGDPISVPTQDMLIGLY
ILTSGNRRGICANRYNPWNHKTYQNERIDDTNYKSMKEPFFCNFYDAIGAYRQKRIHLDS
PLWLRWQLDQRIIASKEAPIEVHYESLGTYHEIYAHYLIIRSVKKEIIDIYIRTTVGHIS
LYREIEEAIQGFYQACS
>P11893 | RK24_PEA
MVAMAMASLQSSMSSLSLSSNSFLGQPLSPITLSPFLQGKPTEKKCLIVMKLKRWERKEC
KPNSLPVLHKLHVKVGDTVKVISGHEKGQIGEITKIFKHNSSVIVKDINLKTKHVKSNQE
GEPGQINKVEAPIHSSNVMLYSKEKDVTSRVGHKVLENGKRVRYLIKTGEIIDSEENWKK
LKEANKKTAEVAAT
>P11964 | SODCP_PEA
MASQTLVSPSPLSSHSLLRTSFSGVSVKLAPQFSTLATSNFKPLTVVAAAKKAVSVLKGT
SAVEGVVTLTQDDEGPTTVNVRITGLTPGLHGFHLHEYGDTTNGCISTGPHFNPNKLTHG
APEDEIRHAGDLGNIVANAEGVAEATIVDNQIPLTGPNSVVGRALVVHELQDDLGKGGHE
LSLSTGNAGGRLACGVVGLTPV
>P12116 | RPOC1_TOBAC
MIDRYKHQQLRIGSVSPQQISAWATKILPNGEIVGEVTKPYTFHYKTNKPEKMDYFVKNF
GPIKSGICACGNYRVIGDEKEDPKFCEQCGVEFVDSRIRRYQMGYIKLACPVTHVWYLKR
LPSYIANLLDKPLKELEGLVYCDFSFARPITKKPTFLRLRGLFEYEIQSWKYSIPLFFTT
QGFDTFRNREISTGAGAIREQLADLDLRIIIENSLVEWEELGEEGHTGNEWEDRKVGRRK
DFLVRRVELAKHFIRTNIEPEWMVLCLLPVLPPELRPIIQIDGGKLMSSDINELYRRVIY

RNNTLTDLLTTSRSTPGELVMCQEKLVQEAVDTLLDNGIRGQPMRDGHNKVYKSFSDVIE
GKEGRFRETLLGKRVDYSGRSVIVVGPSLSLHRCGLPREIAIELFQTFVIRGLIRQHLAS
NIGVAKSKIREKEPIVWEILQEVMQGHPVLLNRAPTLHRLGIQAFQPVLVEGRAICLHPL
VCKGFNADFDGDQMAVHVPLSLEAQVEARLLMFSHMNLLSPAIGDPISVPTQDMLIGLYV
LTSGNHRGICVNRYNPCNRRNYQNQKRSDNSHYKYTKEPFFSNSYDAIGAYRQKRINLDS
PLWLRWRLDQRVIASRETPIEVHYESLGTFYEIYGHYLIVRSLKKQILFIYIRTTVGHIA
LYREIEEAIQGFSRAYSSGT
>P12136 | IF1C_TOBAC
LLNRLPNGLFRVCLDLIINYVSGKIRHSFIRILPGDRVKIEVSPYDSTKGRIIYRLHNKD
LKDSFFNFTIPFVGIQFEMKNFKKLIFFQEIDSELR
>P12139 | RK20_ORYSJ
MTRVPRGYIARRRRAKMRSFASNFRGAHLRLNRMITQQVRRAFVSSHRDRVRQKRDFRRL
WISRINAATRIHKVFDNYSKLIHNLYKKELILNRKILAQVAVLNSNNLYTISNKIKIIN
>P12149 | RR12_ORYSJ
MPTVKQLIRNARQPIRNARKSAALKGCPQRRGTCARVYTINPKKPNSALRKVARVRLTSG
FEITAYIPGIGHNLQEHSVVLVRGGRVKDLPGVRYRIIRGALDAVAVKNRQQGRSKYGVK
KPKK
>P12163 | PSBK_SPIOL
MLNIFSLICLNSALYSSSFFFGKLPEAYAFLSPIVDFMPVIPLFFFLLAFVWQAAVSFR
>P12174 | MATK_MARPO
MEHRIYNSNYFLDITIPYFFHPEILIRIFRRHIQDIPFLHFLRTLLYKNKCLNILNIENF
FYLKKNQFFCFLWNFYIYEFEYLLNDIWEKFYKFESVFFWNFIDKTNSIKKIKHILKKSK
KPIEKKIVKKISSIHYIRYKNNLIITLNDRNILILENWKDFFLIFWQKYFNVWFKSSRIL
IQNFYKNSFSFLGYMFRIESQIILIQIQIINLLRNVNLIKKEFCSIIPVIPLIRLLAKEK
FCDVLGRPLCKLSWTTLSDNEIFERFDQIIKHIFSYYSGCINKKGLYQLQYIFRFSCAKT
LACKHKSTIRTVWKKYGSNLLTSSIFFNKTKLISLNFSNKNPYKKNFWYLNIIQVNYLAH
SLQKSKLLKE
>P12196 | RK32_MARPO
MAVPKKRTSKSKTRIRKAIWKNKANKSALRAFSLAKSILTNRSKSFYYTINDKLLNSSKS
ISTSKLDES
>P12199 | NDHJ_MARPO
MLNILKNNNNKIQGRLSIWLIKHNLKHRPLGFDYQGIETLQIRSEDWPSLAVALYVYGFN
YLRSQCAYDVEPGGLLASVYHFTKITDNADQPEEICIKIFILRKNPKIPSIFWVWKSADF
QERESYDMFGIFYENHPCLKRILMPDSWLGWPLRKDYIVPNFYELQDAY
>P12210 | CLPP_TOBAC
MPIGVPKVPFRSPGEEDASWVDVYNRLYRERLLFLGQEVDSEISNQLIGLMVYLSIEDET
KDLYLFINSPGGWVIPGVAIYDTMQFVRPDVHTICMGLAASMGSFILVGGEITKRLAFPH
ARVMIHQPASSFYEAQTGEFVLEAEELLKLRETLTRVYVQRTGKPLWVVSEDMERDVFMS
ATEAQAYGIVDLVAVE
>P12216 | CCSA_TOBAC
MIFSTLEHILTHISFSIVSIVITIHLITFLVDEIVKLYDSSEKGIIVTFFCITGLLVTRW
ISSGHFPLSDLYESLIFLSWSFSLIHIIPYFKKNVLILSKITGPSAIFTQGFATSGILTE
IHQSVILVPALQSEWLIMHVSMMILGYAALLCGSLLSVALLVITFRKNRQLFYKSNGFLN
ESFFLGENVLQNTSFFSAKNYYRSQLIQQLDYWSYRVISLGFTFLTIGILSGAVWANEAW

GSYWNWDPKETWAFITWIVFAIYLHTRTNRNLRGANSAIVASIGFLIIWICYFGVNLLGI
GLHSYGSFPSTFN
>P12217 | ACCD_MARPO
MSLMNWFEDKRRFGGLIGAFIEKATKGYIFSEREKDRYIKIDTTKGLWTRCDNCENMLYV
RFLRQNKRICEECGYHLQMSSTERIELLIDRGTWYPMDEDMTARDVLKFSDEDSYKNRIA
FYQKRTGLTDAIQTGIGQLNGIPIALGVMDFQFMGGSMGSVVGEKITRLIEYATRASMPL
IIVCSSGGARMQEGTLSLMQMAKISSVLQIHQAQKRLLYIAILTYPTTGGVTASFGMLGD
IIIAEPKAYIAFAGKRVIEQTLRQKIPDGFQVAESLFDHGLLDLIVPRNLLKGVLSEIFE
LYNAAPCKKFQNSFFK
>P12299 | GLGL2_WHEAT
MSSMQFSSVLPLEGKACISPVRREGSASERLKVGDSSSIRHERASRRMCNGGRGPAATGA
QCVLTSDASPADTLVLRTSFRRNYADPNEVAAVILGGGTGTQLFPLTSTRATPAVPIGGC
YRLIDIPMSNCFNSGINKIFVMTQFNSASLNRHIHRTYLGGGINFTDGSVEVLAATQMPG
EAAGWFRGTADAVRKFIWVLEDYYKNKSIEHILILSGDQLYRMDYMELVQKHVDDNADIT
LSCAPVGESRASEYGLVKFDSSGRVVQFSEKPKGDDLEAMKVDTSFLNFAIDDPAKYPYI
ASMGVYVFKRDVLLNLLKSRYAELHDFGSEILPRALHDHNVQAYVFTDYWEDIGTIRSFF
DANMALCEQPPKFEFYDPKTPFFTSPRYLPPTKSDKCRIKEAIISHGCFLRECKIEHSII
GVRSRLNSGSELKNAMMMGADSYETEDEISRLMSEGKVPIGVGENTKISNCIIDMNARIG
RDVVISNKEGVQEADRPEEGYYIRSGIVVIQKNATIKDGTVV
>P12468 | RBS4_SOYBN
MASSMISSPAVTTVNRAGAGMVAPFTGLKSMAGLPTRKTNNDITSIASNGGRVQCMQVWP
PVGKKKFETLSYLPDLDDAQLAKEVEYLLRKGWIPCLEFELEHGFVYREHNRSLGYYDGR
YWTMWKLPMFGCTDASQVLKELQEAKTAYPNGFIRIIGFDNVRQVQCISFIAYKPPSF
>P13788 | RR4_SPIOL
MSRYRGPRFKKIRRLGALPGLTNKRPRAGSDLRNQSRSGKRSQYRIRLEEKQKLRFHYGI
TERQLLKYVRIARKAKGSTGQVLLQLLEMRLDNILFRLGMAPTIPGARQLVNHRHILVNG
RIVDIPSYRCKPQDTIMARDEQKSIALIQNSLDLSPREELPKHLTLNPFPYKGLVNQIID
SKWVGLKINELLVVEYYSRQT
>P14149 | RR12_CHLRE
MPTIQQLIRSARKKITKKTKSPALKSCPQRRGICLRVYTVTPKKPNSALRKVARVRLTTG
FEVTAYIPGVGHNLQEHAVVLVRGGRVKDLPGVRYHIVRGSLDTAGVKNRVQSRSKYGVK
MGSKTAAKTAGKK
>P15102 | GLNA4_PHAVU
MAQILAPSTQWQMRFTKSSRHASPITSNTWSSLLMKQNKKTSSAKFRVLAVKSDGSTINR
LEGLLNLDITPFTDKIIAEYIWIGGTGIDVRSKSRTISKPVEHPSELPKWNYDGSSTGQA
PGEDSEVILYPQAIFKDPFRGGNNILVICDAYTPAGEPIPTNKRHRAAEVFSNPRVIAEV
PWFGIEQEYTLLQTNVNWPLGWPVGGYPGPQGPYYCSAGADKSFGRDISDAHYKACLFAG
INISGTNGEVMPGQWEYQVGPSVGIEAGDHIWASRYILERITEQAGVVLSLDPKPIEGDW
NGAGCHTNYSTKSMREDGGFEVIKKAILNLSLRHKEHISAYGEGNERRLTGKHETASINT
FSWGVANRGCSIRVGRDTEKNGKGYLEDRRPASNMDPYVVTSLLAESTLLWEPTLEAEAL
AAQKLALKV
>P16025 | RPOC2_MAIZE
MAERANLVFHNKEIDGTAMKRLISRLIDHFGMGYTSHILDQIKTLGFHQATTTSISLGIE
DLLTIPSKGWLVQDAEQQSFLLEKHYYYGAIHAVEKLRQSVEIWYATSEYLKQEMNSNFR

ITDPSNPVYLMSFSGARGNASQVHQLVGMRGLMADPQGQMIHLPIQSNLREGLSLTEYII
SCYGARKGVVDTAVRTADAGYLTRRLVEVVQHIIVRRRDCGTIQGISVSPQNGMTEKLFV
QTLIGRVLADDIYIGSRCIASRNQDIGIGLVNRFITAFRAQPFRAQPIYIRTPFTCRSTS
WICQLCYGRSPTHGDLVELGEAVGIIAGQSIGEPGTQLTLRTFHTGGVFTGGTADLIRSP
SNGKIQFNEDLVHPTRTRHGQPAFLCYIDLHVTIQSQDILHSVNIPLKSLILVQNDQYVE
SEQVIAEIRAGTSTLHFKEKVQKHIYSESDGEMHWSTDVYHAPEYQYGNLRRLPKTSHLW
ILSVSMCRSSIASFSLHKDQDQMNTYSFSVDGRYIFDFSMANDQVSHRLLDTFGKKDREI
LDYLTPDRIVFNGHWNCFYPSILQDNSDLLAKKRRNRLVVPLQYHQEQEKERISCLGISM
EIPFMGVLRRNTIFAYFDDPRYRKDKRGSGIVKFRYRTLEEEYRTQEEEYRTREEEYRTR
EEDSEDEYESPENKYRTREGEGEYKILEDEYRTLEDEYETLEDEYGILEDEYRTLEKDSE
EEYGSLENKYRTREGEGEYEILEEESEEEYGSSEDGSEKEYGTLEEDSEEDSEEDSEDEY
GSPEEDSILKKEGFIEHRGTKEFSLKYQKEVDRFFFILQELHILPRSSSLKVLDNSIIGV
DTQLTKNTRSRLGGLVRVKRKKSHTELKIFSGDIHFPEEADKILGGSLIPPEREKKDSKE
SKKRKNWVYVQRKKFLKSKEKYFVSVRPAVAYEMDEGINLATLFPQDLLQEEDNLQLRLV
NFISHENSKLTQRIYHTNSQFVRTCLVVNWEQEEKEGARASLVEVKTNDLIRDFLRIELV
KSTILYTRRRYDRTSVGLIPNNRLDRNNTNSFYSKAKIQSLSQHQEVIGTLLNRNKEYPS
LMILLASNCSRIGLFKNSKYPNAVKESNPRIPIRDIFGLLGVIVPSISNFSSSYYLLTHN
QILLKKYLFLDNLKQTLQVLQGLKYSLIDENKRISNFDSNIMLEPFHLNWHFLHHDSWEE
TLAIIHLGQFICENLCLFKLHIKKSGQIFIVNMDSFVLRAAKPYLATIGATVHGHYGKIL
YKGDRLVTFIYEKSRSSDITQGLPKVEQIFEARSIDSLSPNLERRIEDWNERIPRILGVP
WGFLIGAELTIAQSRISLVNKIQKVYRSQGVQIHNRHIEIIIRQVTSKVRVSEDGMSNVF
LPGELIGLLRAERAGRALDESIYYRAILLGITRASLNTQSFISEASFQETARVLAKAALR
GRIDWLKGLKENVVLGGIIPVGTGFQKFVHRSPQDKNLYLEIQKKNLFASEMRDILFLHT
ELVSSDSDVTNNFYETSETPFTPIYTI
>P16032 | RBS1_MESCR
MASSMVSSAAVATVNRTPAQASMVAPFNGLKSVAAFPVTKKNNDITSVATNGGRVQCMQV
WPPLGKKKFETLSYLPPLSEESLMKEVQYLLNNGWVPCLEFEPTHGFVYREHGNTPGYYD
GRYWTMWKLPMFGCTDPSQVVAELEEAKKAYPEAFIRIIGFDNVRQVQCISFIAYKPASY
DA
>P16037 | RR2_MAIZE
MTRRYWNINLKEMIEAGVHFGHGIKKWNPKMAPYISAKRKGTHITNLARTARFLSEACDL
VFDAASQGKSFLIVGTKKRAADLVASAAIRSRCHYVNKKWFSGMLTNWSITKTRLSQFRD
LRAEEKMGKFHHLPKRDAAILKRKLSTLQRYLGGIKYMTRLPDIVIVLDQQKEYIALQEC
AILGIPTISLVDTNCDPDLANISIPANDDTMTSIRLILNKLVFAISEGRSLYIRNR
>P16131 | RBS3_ACECL
MATTMLNRSVIVNKEVAKTPNFPRATKNNKGFASNAAVQKCRDMMVWQPFNNKMFETFSY
LPPLTDEQISKQVDYILANSWTPCLEFAASDQAYAGNENCIRMGPVSSTYQDNRYWTMWK
LPMFGCTDGSQVLSEIQACTKAFPDAYIRLVCFDANRQVQISGFLVHRPPSATDYRLPAD
RQV
>P16132 | RBS4_ACECL
MAATMMNKTVVLSKGCTKPSAVPKVSINRKGFLNTAMNKKREMMVWQPFNNKMFETFSYL
PPLTDEQISKQVDYILANSWTPCLEFAASDQAYAGNENCIRMGPVASTYQDNRYWTMWKL
PMFGCTDGSQVLSEIQACTKAFPDAYIRLVCFDANRQVQISGFLVHRPPSATDYRLPADR
QV

>P16134 | RBS1_ACEAT
MASIMMNKSVVLSKECAKPLATPKVTLNKRGFATTIATKNREMMVWQPFNNKMFETFSFL
PPLTDEQISKQVDYILTNSWTPCLEFAASDQAYAGNENCIRMGPVASTYQDNRYWTMWKL
PMFGCTDGSQVLSEIQACTKAFPDAYIRLVCFDANRQVQISGFLVHRPPSATDYRLPADR
QV
>P16306 | RBL_AMAHP
MSPQTETKASVGFKAGVKDYRLTYYTPEYETQDTDILAAFRVSPQPGVPPEEAGAAVAAE
SSTGTWTSVWTDGLTNLDRYKGRCYNIEPVAGEENQYICYVAYPLDLFEEGSVTNMFTSI
VGNVFGFKALRALRLEDLRIPVAYVKTFQGPPHGIQVERDKLNKYGRPLLGCTIKPKLGL
SAKNYGRACYECLRGGLDFTKDDENVNSQPFMRWRDRFLFCAEAIYKSQAETGEIKGHYL
NATAGTCEEMIKRAVFARELGVPIVMHDYLTGGFTANTSLSQYCRDNGLLLHIHVAMHAV
IDRQKNHGMHFRVLAKALRLSGGDHIHSGTVVGKLEGERDITLGFVDLLRDDYTEKDRCR
GIFFTQSWVSTPGVLPVASGGIHVWHMPALTEIFGDDSVLQFGGGTLGHPWGNAPGAVAN
RVALEACVQARNEGRDLAREGNTIIREAAKWSPELAAACEVWKEIKFEFPAMDTI
>P17353 | RK16_SPIOL
MLSPKRTRFRKQHRGRMKGISYRGNRICFGRYALQALEPAWITSRQIEAGRRAMTRNARR
GGKIWVRIFPDKPVTVRPAETRMGSGKGSPEYWVAVVKPGRILYEISGVAENIARRAVAI
AASKMPIRTQFIISG
>P17537 | RBS_CHLMO
MASIAAKSVSLRAATRRAAPVAAPADARFKVWQPVNNKQYETFSYLPPLTNQKIGRQVDY
IINNGWTPCLEFADPSTSFVSNANAVRLQGVSAGYYDNRYWTMWKLPMFGCTDPSQVLRE
VSACQVAFPNVYIRLVAFDNVKQVQCMGFLVQRPRNAAEYCPLEKRSV
>P17652 | CHLB_CHLMO
MKLAYWMYAGPAHIGTLRVASSFKNVHAIMHAPLGDDYFNVMRSMLERERDFTPVTTSIV
DRHVLARGSQEKVVENITRKDNEESPDLIIILTPTCTSSILQEDLQNFVNRASMSENSTSD
VLLADVNHYRVNELQAADRTLEQIVRFYLEKEKSTLFRQGVSSVDDNNKTLTNNFLSIKT
EKPSANIIGIFTLGFHNQHDCRELKRLLNNLGIEINEVIPEGGSVKNLKNLPKAWFNIIP
YREVGLMSAIYLEKEFNMPYVAVSPIGIIDTAVCIREIERILNKIYFESLEGNVNTQSVL
TTNPYFNSHINSTNSETRDHNVKPFDFEFYIENQTRFISQAAWFSRSIDCQNLTGKKAVV
FGDATHAAGITKILAREMGIKVVCSGTYCKHDADWFREQVFGFCDQILITDDHTQVGDMI
AKLEPSAIFGTQMERHIGKRLDIPCGVISAPVHIQNFPLSYRPFLGYEGTNQIADLVYNS
FSLGMEDHLLEIFSGHDTKEPITKSLSTENELNWDAEALKELSNVPGFVRGKVKRNTEKY
ARQNAIPSITLDVLFAAKEALSA
>P17673 | RBS_TRIRP
MALISSAAVTTINRAPVQANLATPFTGLKSSAGFPVTKKNNDITSITSNGSRVNCMQVWP
PVGKKKFETLSYLPPLTDEQLLKEVEYLLRKGWVPCVEFELEKGFVHRQYNSSPGYYDGR
YWTMWRLPLFGTTDAAQVLKEVAECKAEYPEAFIRIIGFDNVRQVQCISFIASTPKVY
>P17688 | AROA_BRANA
MAQSSRICHGVQNPCVIISNLSKSNQNKSPFSVSLKTHQPRASSWGLKKSGTMLNGSVIR
PVKVTASVSTSEKASEIVLQPIREISGLIKLPGSKSLSNRILLLAALSEGTTVVDNLLNS
DDINYMLDALKKLGLNVERDSVNNRAVVEGCGGIFPASLDSKSDIELYLGNAGTAMRPLT
AAVTAAGGNASYVLDGVPRMRERPIGDLVVGLKQLGADVECTLGTNCPPVRVNANGGLPG
GKVKLSGSISSQYLTALLMAAPLALGDVEIEIIDKLISVPYVEMTLKLMERFGVSAEHSD
SWDRFFVKGGQKYKSPGNAYVEGDASSASYFLAGAAITGETVTVEGCGTTSLQGDVKFAE

VLEKMGCKVSWTENSVTVTGPSRDAFGMRHLRAVDVNMNKMPDVAMTLAVVALFADGPTT
IRDVASWRVKETERMIAICTELRKLGATVEEGSDYCVITPPAKVKPAEIDTYDDHRMAMA
FSLAACADVPVTIKDPGCTRKTFPDYFQVLESITKH
>P17703 | RR15_MAIZE
MKKKGGRKIFGFMVKEEKEENRGSVEFQVFSFTNKIRRLASHLELHKKDFSSERGLRRLL
GKRQRLLAYLAKKNRVRYKKLISQLDIREK
>P17933 | RR2_WHEAT
MTRRYWNINLKEMIEAGVHFGHGIKKWNPKMAPYISAKRKGTHIINLARTARFLSEACDL
VFDAASQGKSFLIVGTKKRATDLVASAAIRARCHYVNKKWFSGMLTNWSITKTRLSQFRD
LRAEEKMGKFHHLPKRDVAILKRKLSTLQRYLGGIKYMTRLPDIVIVLDQQKEYIALREC
AILGIPTISLVDTNCDPDLANISIPANDDTMTSIRLILNKLVFSICEGRSLYIRNR
>P18566 | RBS2_ORYSJ
MAPTVMASSATSVAPFQGLKSTAGLPVSRRSTNSGFGNVSNGGRIKCMQVWPIEGIKKFE
TLSYLPPLTVEDLLKQIEYLLRSKWVPCLEFSKVGFVYRENHRSPGYYDGRYWTMWKLPM
FGCTDATQVLKELEEAKKAYPDAFVRIIGFDNVRQVQLISFIAYKPPGCEESGGN
>P18905 | EFTU_COLOB
MERLLHMTFRNKKIHLNVGTIGHFSHGKTTLTAAITAVLAGIGYTQPKQNDAIDSTSEEK
ARNMSIYVHHVEYETAARHYSHLDCPGHVNYINNMITGVSQMDGAILVVSAVDGPMAQTK
EHILLAKLLGISSILVFINKEDELDDQEVLPMLIQNMRQILIYYGFPGHTSPILCGSALL
ALEAMNENPNFNRGKNKWVDKISSLIDHLDLYLPTPRRKLNKPFLMPIERVILIPSFGLV
GTGTIEKGHINIGESVEIVGFKDTQHSKVISLKMFNKTLEQAIAGDDIGIFLEGTNKNNF
QKGMVIAKPNTIQSWNHFEAQIYILRREEGGRRSPFFQGYCPQFYFRTIQITGRMESFEY
EIGGKTWMVMPGEKIKAIIQLIFPIALKKKMRFVIREGGFTIGVGIILELIKIKN
>P19044 | NU3C_MAIZE
MFLLHEYDIFWTFLIIASLIPILVFWISGLLAPVSEGPEKLSSYESGIEPMGGAWLQFRI
RYYMFALVFVVFDVETVFLYPWAMSFDVLGVSVFIEAFIFVLILVVGLVYAWRKGALEWS

>P19683 | ROC4_NICSY
MSCATKPIIKPSSMATNSCLISLPPLFATTTKSKSFAYPYLSNTLKPIKLLHLSCTYSPC
ILSPKKKTSVSALQEEENTLILDGQGQESGDLFNFEPSGEETEEEGFVEAVGDAGESDEV
EADEEEEEFQEPPEDAKLFVGNLPYDVDSEGLARLFEQAGVVEIAEVIYNRDTDQSRGFG
FVTMSTVEEAEKAVEMYNRYDVNGRLLTVNKAARRGERPERPPRTFEQSYRIYVGNIPWG
IDDARLEQLFSEHGKVVSARVVYDRETGRSRGFGFVTMASEAEMSDAIANLDGQSLDGRT
IRVNVAEDRSRRNTF
>P19824 | KPPR_CHLRE
MAFTMRAPAPRATAQSRVTANRARRSLVVRADKDKTVVIGLAADSGCGKSTFMRRMTSIF
GGVPKPPAGGNPDSNTLISDMTTVICLDDYHCLDRNGRKVKGVTALAPEAQNFDLMYNQV
KALKEGKSVDKPIYNHVSGLIDAPEKIESPPILVIEGLHPFYDKRVAELLDFKIYLDISD
DIKFAWKIQRDMAERGHSLESIKSSIAARKPDFDAYIDPQKKDADMIIQVLPTQLVPDDK
GQYLRVRLIMKEGSKMFDPVYLFDEGSTISWIPCGRKLTCSFPGIKMFYGPDTWYGQEVS
VLEMDGQFDKLEELIYVESHLSNTSAKFYGEITQQMLKNSGFPGSNNGTGLFQTIVGLKV
REVYERIVKKDVVPV
>P21357 | AROF_SOLTU
MALSSTSTTNSLLPNRSLVQNQPLLPSPLKNAFFSNNSTKTVRFVQPISAVHSSDSNKIP

IVSDKPSKSSPPAATATTAPAPAVTKTEWAVDSWKSKKALQLPEYPNQEELRSVLKTIDE
FPPIVFAGEARSLEERLGEAAMGRAFLLQGGDCAESFKEFNANNIRDTFRILLQMGAVLM
FGGQMPVIKVGRMAGQFAKPRSDSFEEKDGVKLPSYRGDNVNGDAFDVKSRTPDPQRLIR
AYCQSAATLNLLRAFATGGYAAMQRINQWNLDFTEHSEQGDRYRELASRVDEALGFMTAA
GLTMDHPIMKTTEFWTSHECLLLPYEQSLTRRDSTSGLYYDCSAHFLWVGERTRQLDGAH
VEFLRGIANPLGIKVSDKMDPSALVKLIEILNPQNKAGRITIITRMGAENMRVKLPHLIR
AVRRAGQIVTWVSDPMHGNTIKAPCGLKTRPFDSIRAEVRAFFDVHDQEGSHPGGVHLEM
TGQNVTECIGGSRTVTFDDLSSRYHTHCDPRLNASQSLELSFIIAERLRKRRLGSQSV
>P22243 | STAD_CARTI
MALRITPVTLQSERYRSFSFPKKANLRSPKFAMASTLGSSTPKVDNAKKPFQPPREVHVQ
VTHSMPPQKIEIFKSIEGWAEQNILVHLKPVEKCWQAQDFLPDPASEGFDEQVKELRARA
KEIPDDYFVVLVGDMITEEALPTYQTMLNTLDGVRDETGASLTPWAVWTRAWTAEENRHG
DLLHTYLYLSGRVDMRQIQKTIQYLIGSGMDPRTENSPYLGFIYTSFQERATFVSHGNTA
RHAKDHGDVKLAQICGTIASDEKRHETAYTKIVEKLFEIDPDGTVLAFADMMRKKISMPA
HLMYDGRDDNLFEHFSAVAQRLGVYTAKDYADILEFLVGRWKVADLTGLSGEGRKAQDYV
CGLPPRIRRLEERAQGRAKEGPVVPFSWIFDRQVKL
>P22337 | STAD_RICCO
MALKLNPFLSQTQKLPSFALPPMASTRSPKFYMASTLKSGSKEVENLKKPFMPPREVHVQ
VTHSMPPQKIEIFKSLDNWAEENILVHLKPVEKCWQPQDFLPDPASDGFDEQVRELRERA
KEIPDDYFVVLVGDMITEEALPTYQTMLNTLDGVRDETGASPTSWAIWTRAWTAEENRHG
DLLNKYLYLSGRVDMRQIEKTIQYLIGSGMDPRTENSPYLGFIYTSFQERATFISHGNTA
RQAKEHGDIKLAQICGTIAADEKRHETAYTKIVEKLFEIDPDGTVLAFADMMRKKISMPA
HLMYDGRDDNLFDHFSAVAQRLGVYTAKDYADILEFLVGRWKVDKLTGLSAEGQKAQDYV
CRLPPRIRRLEERAQGRAKEAPTMPFSWIFDRQVKL
>P22433 | RBS2_NICSY
MAFLIMSSAAAVATGTNAAQASMIAPFTGLKSATSFPVSRKQNLDITSIASNGGRVQCMQ
VWPPINKKKYETLSYLPDLSEEQLLREVEYLLKNGWVPCLEFETEHGFVYRENNKSPGYY
DGRYWTMWKLPMFGCTDATQVLAEVEEAKKAYPQAWIRIIGFDNVRQVQCISFIAYKPEG
Y
>P23981 | AROA1_TOBAC
MAQISSMGQGIRTPNLNSYLPKTQKVPLFSHSIFIGSKKITQNSAKSLWVSKEDSVLRVA
KSPFRISASVVTAQKPNEIVLQPIKDISGTVKLPGSKSLSNRILLLAALSKGRTVVDNLL
SSDDIHYMLGALKTLGLHVEDDNENQRAIVEGCGGQFPVGKKSEEEIQLFLGNAGTAMRP
LTAAVTVAGGHSRYVLDGVPRMRERPIGDLVDGLKQLGAEVDCFLGTNCPPVRIVSKGGL
PGGKVKLSGSISSQYLTALLMAAPLALGDVEIEIIDKLISVPYVEMTLKLMERFGVSVEH
TSSWDKFLVRGGQKYKSPGKAYVEGDASSASYFLAGAAVTGGTVTVEGCGTSSLQGDVKF
AEVLEKMGAEVTWTENSVTVKGPPRNSSGMKHLRAVDVNMNKMPDVAMTLAVVALFADGP
TAIRDVASWRVKETERMIAICTELRKLGATVVEGSDYCIITPPEKLNVTEIDTYDDHRMA
MAFSLAACADVPVTIKDPGCTRKTFPNYFDVLQQYSKH
>P24064 | CLPP_WHEAT
MPIGVPKVPYRIPGDEEATWVDLYNVMYRERTLFLGQEIRCEITNHITGLMVYLSIEDGI
SDIFLFINSPGGWLISGMAIFDTMQTVTPDIYTICLGIAASMASFILLGGEPTKRIAFPH
ARIMLHQPASAYYRARTPEFLLEVEELHKVREMITRVYAVRTGKPFWVVSEDMERDVFMS
ADEAKAYGLVDIVGDEMIDKHCDTDPVWFPEMFKDW

>P24674 | RBL_PICPU
MSPKTETKASVGFKAGVKDYRLTYYTPEYQTKDTDILAAFRVTPQPGVPPEEAGAAVAAE
SSTGTWTTVWTDGLTSLDRYKGRCYDIEPVAGEETQFIAFVAYPLDLFEEGSVTNLFTSI
VGNVFGFKALRALRLEDLRIPPAYSKTFQGPPHGIQVERDKLNKYGRPLLGCTIKPKLGL
SAKNYGRAVYECLRGGLDFTKDDENVNSQPFMRWRDRFVFCAEAIYKAQAETGEIKGHYL
NATAGTCEEMMKRAVFARELGVPIVMHDYLTGGFTANTSLAHYCRDNGLLLHIHRAMHAV
IDRQKNHGMHFRVLAKALRMSGGDHIHGGTVVGKLEGERDITLGFVDLLRDDFIEKDRSR
GIYFTQDWVSMPGVLPVASGGIHVWHMPALTEIFGDDSVLQFGGGTLGHPWGNAPGAVAN
RVALEACVQARNEGRDLAREGNEVIREASKWSPELAAACEVWKEIKFEFEAVDTI
>P24676 | RBL_PINWA
MSPKTETKASVGFKAGVKDYRLTYYTPEYQTKDTDILAAFRVTPQPGVPAEEAGAAVAAE
SSTGTWTTVWTDGLTSLDRYKGRCYDIEPVPGEENQFIAYVAYPLDLFEEGSVTNLFTSI
VGNVFGFKALRALRLEDLRIPPAYSKTFQGPPHGIQVERDKLNKYGRPLLGCTIKPKLGL
SAKNYGRAVYECLRGGLDFTKDDENVNSQPFMRWRDRFVFCAEAINKAQAETGEIKGHYL
NATAGTCEEMMKRAVFARELGVPIVMHDYLTGGFTANTSLAHYCRDNGLLLHIHRAMHAV
IDRQRNHGMHFRVLAKALRMSGGDHVHAGTVVGKLEGERDVTLGFVDLLRDDFIEKDRSR
GVYFTQDWVSMPGVLPVASGGIHVWHMPALTEIFGDDSVLQFGGGTLGHPWGNAPGAAAN
RVALEACVQARNEGRDLAREGNEVIREACKWSPELAAACEIWKEIKFEFDVIDRL
>P24677 | RBL_PINLO
MSPKTETKASVGFKAGVKDYRLTYYTPEYQTKDTDILAAFRVTPQPGVPAEEAGAAVAAE
SSTGTWTTVWTDGLTSLDRYKGRCYDIEPVPGEETQFIAYVAYPLDLFEEGSVTNLFTSI
VGNVFGFKALRALRLEDLRIPPAYSKTFQGPPHGIQVERDKLNKYGRPLLGCTIKPKLGL
SAKNYGRAVYECLRGGLDFTKDDENVNSQPFMRWRDRFVFCAEAIYKAQAETGEIKGHYL
NATAGTCEEMMKRAVFARELGVPIVMHDYLTGGFTANTSLAHYCRDNGLLLHIHRAMHAV
IDRQRNHGMHFRVLAKALRMSGGDHIHAGTVVGKLEGERDVTLGFVDLLRDDFIEKDRSR
GIYFTQDWVSMPGVLPVASGGIHVWHMPALTEIFGDDSVLQFGGGTLGHPWGNAPGAVAN
RVALEACVQARNEGRDLAREGNEVIREACKWSPELAAACEIWKEIKFEFDVIDRL
>P24678 | RBL_PINPI
MSPKTETKASVGFKAGVKDYRLTYYTPEYQTKDTDILAAFRVTPQPGVPPEEAGAAVAAE
SSTGTWTTVWTDGLTSLDRYKGRCYDIEPVPGEETQFIAYVAYPLDLFEEGSVTNLFTSI
VGNVFGFKALRALRLEDLRIPPSYSKTFQGPPHGIQVERDKLNKYGRPLLGCTIKPKLGL
SAKNYGRAVYECLRGGLDFTKDDENVNSQPFMRWRDRFVFCAEAINKAQAETGEIKGHYL
NATAGTCEEMIKRAVFARELGVPIVMHDYLTGGFTANTSLAHYCRDNGLLLHIHRAMHAV
IDRQRNHGMHFRVLAKALRMSGGDHIHAGTVVGKLEGERDVTLGFVDLLRDDFIEKDRSR
GIYFTQDWVSMPGVMPVASGGIHVWHMPALTEIFGDDSVLQFGGGTLGHPWGNAPGAVAN
RVALEACVQARNEGRDLAREGNEVIREACKWSPELAAACEIWKEIKFEFDVIDRL
>P24679 | RBL_PINRA
MSPKTETKASVGFKAGVKDYRLTYYTPEYQTKDTDILAAFRVTPQPGVPPEEAGAAVAAE
SSTGTWTTVWTDGLTSLDRYKGRCYDIEPVAGEETQFIAYVAYPLDLFEEGSVTNLFTSI
VGNVFGFKALRALRLEDLRIPPAYSKTFQGPPHGIQVERDKLNKYGRPLLGCTIKPKLGL
SAKNYGRAVYECLRGGLDFTKDDENVNSQPFMRWRDRFVFCAEAIYKAQAETGEIKGHYL
NATAGTCEEMMKRAIFARELGVPIVMHDYLTGGFTANTSLAHYCRDNGLLLHIHRAMHAV
IDRQRIHGMHFRVLAKALRMSGGDHIHAGTVVGKLEGERDVTLGFVDLLRDDFIEKDRSR
GIYFTQDWVSMPGVLPVASGGIHVWHMPALTEIFGDDSVLQFGGGTLGHPWGNAPGAVAN

RVALEACVQARNEGRDLAREGNEVIREACKWSPELAAACEIWKEIKFEFDVIDRL
>P24929 | RK12_TOBAC
MASTLSTITLRSPSPSTASSTHASIPFPKKALEFPIRTPKLHHRRATFLRPLAAVEAPEK
VVQLGDEISNLTLADAQKLVEYLQDKLGVTAASFAPAAVAAAPGAAAEAPAVVEEKTEFD
VVIDEVPSNARIATIKAVRALTSLALKEAKELIEGLPKKFKEGVSKDEAEDAKKQLEEAG
AKVSIA
>P25079 | RBL_SOLTU
MSPQTETKASVGFKAGVKEYKLTYYTPEYQTKDTDILAAFRVTPQPGVPPEEAGAAVAAE
SSTGTWTTVWTDGLTSLDRYKGRCYRIERVVGEKDQYIAYVAYPLDLFEEGSVTNMLTSI
VGNVFGFKALRALRLEDLRIPVAYVKTFQGPPHGIQVERDKLNKYGRPLLGCTIKPKLGL
SAKNYGRAVYECLRGGLDFTKDDENVNSQPFMRWRDRFLFCAEALFKAQAETGEIKGHYL
NATAGTCEEMMKRAVFARELGTPIVMHDYLTGGFTANTTLAHYCRDNGLLLHIHRAMHAV
IDRQKNHGMHFRVLAKALRMSGGDHIHSGTVVGKLEGERDITLGFVDLLRDDFIEQDRSR
GIYFTQDWVSLPGVLPVASGGIHVWHMPALTEIFGDDSVLQFGGGTLGHPWGNAPGAVAN
RVALEACVKARNEGRDLAREGNEIIREAAKWSPELAAACEVWKEIVFNFAAMDVLDK
>P25459 | RR18_MAIZE
MYISKQPFRKSKQPFRKSKQTFHKSKQPFRKFKQPFRKSKQPFRKSKQPFRRRSRIGPGD
RIDYRNMSLINRFISEQGKILSRRINRLTLKQQRLITLAIKQARILSFLPFRNYENEKQF
QAQAISIITGPRHRKNRHIPQLTQKFNSNRNLRNSNQNLRNNNRNLSSDC
>P25827 | RBL_AREDR
MSPQTETKASVGFKAGVKDYKLTYYTPDYETLDTDILAAFRVTPQPGVPAEEAGAAVAAE
SSTGTWTTVWTDGLTSLDRYKGRCYHIEPVAGDENQYICYVAYPLDLFEEGSVTNMFTSI
VGNVFGFKALRALRLEDLRIPVAYIKTFLGPPHGIQVERDKLNKYGRPLLGCTIKPKLGL
SAKNYGRAVYECLRGGLDFTKDDENVNSQPFMRWRDRFLFCAEAINKAQAETGEIKGHYL
NATAGTCEEMIKRAVFARELGVPIVMHDYITGGFTANTSLAHYCRDNGLLLHIHRAMHAV
IDRQKNHGMHFRVLAKALRLSGGDHIHSGTVVGKLEGERDITLGFVDLLRDDFTEKDRSR
GIYFTQSWVSTPGVLPVASGGIHVWHMPALTEIFGDDSVLQFGGGTLGHPWGNAPGAVAN
RVALEACVQARNEGRDLAREGNTIIREACKWSPELAAACEVWKEIKFEFQAMDTID
>P25828 | RBL_BASAL
MSPQTETKAFVGFKAGVKDYKLNYYTPQYQPLDTDILAAFRVTPQPGVPSEEAGAAVAAE
SSTGTWTTVWTDGLTSLDRYKGRCYHIDPVPGEDNQYICYVAYPLDLFEEGSVTNMFTSI
VGNVFGFKALRALRLEDLRIPVAYIKTFQGPPHGIQVERDKLNKYGRAILGCTIKPKLGL
SAKNYGRAVYECLRGGLDFTKDDENVNSQPFMRWRDRFLFCAEALYKAQAETGEIKGHYL
NATAGTCEEMIKRAVFARELGAPIVMHDYLTGGFTANTSLAHYCRDNGLLLHIHRAMHAV
IDRQKNHGMHFRVLAKALRLSGGDHIHAGTVVGKLEGERDITLGFVDLLRDDYIEIDDDR
GIYFTQPWVSTPGVLPVASGGIHVWHMPALTEIFGDDSVLQFGGGTLGHPWGNAPGAVAN
RVALEACVQARNEGRDLAREGATIIREAAKWSPELAAACEVWKEIKFEFPAVDTLDKKKG

>P25839 | RBL_TRIPO
MSPQTETKASVGFKAGVKDYKLTYYTPEYKPLDTDILAAFRVTPQPGVPSEEAGAAVAAE
SSTGTWTTVWTDGLTSLDRYKGRCYHIDPVPGEDNQYICYVAYPLHLFEEGSVTNIFTSI
VGNVFGFKALRALRLEDLRIPIAYIKTFQGPPHGIQVERDKLNKYGRKILGCTIKPKLGL
SAKNYGRAVYECLRGGLDFTKDDENVNSQPFMRWRDRFCFSAEAIYKAQAETGEIKGHYL
NATAGTCEEMIKRAVFARELGVPIVMHDYLTGGFTANTSLSHYCRDNGLLLHIHRAMHAV

IDRQKNHGMHFRVLAKALRLSGGDHIHAGTVVGKLEGERDITLGFVDLLRDDYTEIDDQR
GIYFTQSWASTPGVLPVASGGIHVWHMPALTEIFGDDSVLQFGGGTLGHPWGNAPGAVAN
RVALEACVQARNEGRDLAKEGNTIIRDAAKWSPELAAACEVWKEIKFEFPAVDVLDKKKK

>P25875 | RR16_HORVU
MVKLRLKRCGRKQQAVYRIVAIDVRSRREGRDLRKVGFYDPIKNQTCLNVPAILYFLEKG
AQPTRTVYDILRKAEFFKEKERTLS
>P26180 | CHLN_PINCO
MSTKIVETITLECETGNYHSFCPISCVSWLYQKIEDSFFLVVGTKTCGYFLQNALGVMIF
AEPRYAMAELEEGDISAHLNDYEELKTLCIRIRKDRDPSVIIWIGTCTTEIIKMDLEGMA
PKLEYEIGVPILVARANGLDYAFTQGEDTVLAVMAHRCPDQEFPIGESKETKKKLFPFPL
LKENNLVEYANHPPLVIFGSLPSNLVSQLDTELRRQFIKVSGWLPAQRYADLPSLGDGVY
VCGVNPFLSRTATTLIRRKKCELIVAPFPIGPDGTRAWIERICPVFGIEAQSLEEIEERI
WESLKDYLDLVRGKSVFFMGDNLIEISIARFLIRCGMIVYEIGIPYLDKRYQAAELALLK
KTCIRMCMPIPRIVEKPDNSNQIRRMRELKPDLAITGMAHANPLGARGIGTKWSVEFTFA
QIHGFANARDVLELVTRPLRRNENLDNLDRTTLVRKNNK
>P26289 | NU4LC_ARATH
MILEHVLVLSAYLFLIGLYGLITSRNMVRALMCLELILNAVNMNFVTFSDFFDNSQLKGE
IFCIFVIAIAAAEAAIGLAIVSSIYRNRKSIRINQSTLLNK
>P26573 | RBS8_NICPL
MASSVLSSAAVATRSNVAQANMVAPFTGLKSAASFPVSRKQNLDITSIASNGGRVQCMQV
WPPINKKKYETLSYLPDLSQEQLLSEIEYLLKSGWVPCLEFETERGFVYREHHHSPGYYD
GRYWTMWKLPMFGCTDATQVLAEVEEAKKAYPQAWVRIIGFDNVRQVQCISFIAYKPEGY

>P26574 | RBS1_SOLTU
MASSVISSAAVATRTNVTQAGSMIAPFTGLKSAATFPVSRKQNLDITSIASNGGRVRCMQ
VWPPINMKKYETLSYLPDLTDEQLLKEVEYLLKNGWVPCLEFETEHGFVYRENHKSPGYY
DGRYWTMWKLPMFGCTDATQVLAEVQECKKSYPQAWIRIIGFDNVRQVQCISFIAYKPEG
Y
>P26577 | RBSC_SOLTU
MASSVMSSAAVATRGNGAQASMVAPFTGLKSTASFPVSRKQNLDITSIASNGGRVRCMQV
WPPINMKKYETLSYLPDLSDEQLLKEVEYLLKNGWVPCLEFETEHGFVYRENNKSPGYYD
GRYWTMWKLPMFGCTDATQVLAEVEEAKKAYPQAWIRIIGFDNVRQVQCISFIAYKPEGY

>P26667 | RBS2_WHEAT
MAPAVMASSATTVAPFQGLKSTAGLPISCRSGSTGLSSVSNGGRIRCMQVWPIEGIKKFE
TLSYLPPLSTEALLKQVDYLIRSKWVPCLEFSKVGFVFREHNSSPGYYDGRYWTMWKLPM
FGCTDATQVLNEVEEVKKEYPDAYVRVIGFDNMRQVQCVSFIAFRPPGCEESGKA
>P26959 | RBL_CEDDE
MSPKTETKASVGFKAGVKDYRLTYYTPEYQTKDTDILAAFRVTPQPGVPPEEAGAAVAAE
SSTGTWTTVWTDGLTSLDRYKGRCYDIEPVPGEESQFIAYVAYPLDLFEEGSVTNLFTSI
VGNVFGFKALRALRLEDLRIPPAYSKTFQGPPHGIQVERDKLNKYGRPLLGCTIKPKLGL
SAKNYGRAVYECLRGGLDFTKDDENVNSQPFMRWRDRFVFCAEAIYKAQAETGEIKGHYL
NATAGTCEEMMKRAVFARELGVPIVMHDYLTGGFTANTSLAHYCRDNGLLLHIHRAMHAV

IDRQRIHGMHFRVLAKALRMSGGDHIHAGTVVGKLEGERDVTLGFVDLLRDDFIEKDRSR
GIYFTQDWVSMPGVLPVASGGIHVWHMPALTEIFGDDSVLQFGGGTLGHPWGNAPGAVAN
RVALEACVQARNEGRDLAREGNEVIREASKWSPELAAACEVWKEIKFEFEAIDVL
>P26960 | RBL_KETDA
MSPKTETKASVGFKAGVKDYRLTYYTPEYQTKDTDILAAFRVTPQPGVPPEEAGAAVAAE
SSTGTWTTVWTDGLTSLDRYKGRCYDIEPVPGEESQFIAYVAYPLDLFEEGSVTNLFTSI
VGNVFGFKALRALRLEDLRIPPAYSKTFQGPPHGTQVERDKLNKYGRPLLGCTIKPKLGL
SAKNYGRAVYECLRGGLDFTKDDENVNSQPFMRWRHRFVFCAEALNKAQAETGEIKGHYL
NATAGTCEEMMKRAIFARELGVPIVMHDYLTGGFTANTSLAHYCRDNGLLLHIHRAMHAV
IDRQRNHGMHFRVLAKALRMSGGDHIHAGTVVGKLEGERDVTLGFVDLLRDDFIEKDRSR
GIYFTQDWVSMPGVLPVASGGIHVWHMPALTEIFGDDSVLQFGGGTLGHPWGNAPGAVAN
RVAVEACVQARNEGRDLAREGNEVIREAAKWSPELAAACEVWKEIKFEFEAIDYL
>P26962 | RBL_PINBA
MSPKTETKASVGFKAGVKDYRLTYYTPEYQTKDTDILAAFRVTPQPGVPAEEAGAAVAAE
SSTGTWTTVWTDGLTSLDRYKGRCYDIEPVPGEENQFIAYVAYPLDLFEEGSVTNLFTSI
VGNVFGFKALRALRLEDLRIPPAYSKTFQGPPHGIQVERDKLNKYGRPLLGCTIKPKLGL
SAKNYGRAVYECLRGGLDFTKDDENVNSQPFMRWRDRFVFCAEAINKAQAETGEIKGHYL
NATAGTCEEMMKRAVFARELGVPIVMHDYLTGGFTANTSLAHYCRDNGLLLHIHRAMHAV
IDRQRNHGMHFRVLAKALRMSGGDHIHAGTVVGKLEGERDVTLGFVDLLRDDFIEKDRSR
GIYFTQDWVSMPGVLPVASGGIHVWHMPALTEIFGDDSVLQFGGGTLGHPWGNAPGAVAN
RVALEACVQARNEGRDLAREGNEVIREACKWSPELAAACEIWKEIKFEFDVIDRL
>P26964 | RBL_TSUHE
MSPKTETKASVGFKAGVKDYRLTYYTPEYQTKDTDILAAFRVTPQPGVPPEEAGAAVAAE
SSTGTWTTVWTDGLTSLDRYKGRCYDIEPVPGEESQFIAYVAYPLDLFEEGSVTNLFTSI
VGNVFGFKALRALRLEDLRIPPAYSKTFQGPPHGIQVERDKLNKYGRPLLGCTIKPKLGL
SAKNYGRAVYECLRGGLDFTKDDENVNSQPFMRWRDRFVFCAEALYKAQAETGEIKGHYL
NATAGTCEEMMKRAVFARELGVPIVMHDYLTGGFTANTTLAHYCRDNGLLLHIHRAMHAV
IDRQRNHGMHFRVLAKALRMSGGDHIHAGTVVGKLEGEREVTLGFVDLLRDNYIEKDRSR
GIYFTQDWVSMPGVLPVASGGIHVWHMPALTEIFGDDSVLQFGGGTLGHPWGNAPGAVAN
RVALEACVQARNEGRDLAREGNEVVREACKWSPELAAACEVWKEIKFEFDTIDYL
>P26985 | RBS_BATOE
MSFATTNKTIVPCATTKQIVRPRFLSNGTISKSRAMMVWEPFNNKFFETFSYLPPLTDDQ
ITKQVDYILRNNWTPCLEFAGSDQAYVTHDNTVRMGDCASTYQDNRYWTMWKLPMFGCID
GSQVLTEISACTKAFPDAYIRLVCFDANRQVQISGFLVHRPESATDYRLPADRQV
>P27064 | RBL_CUCSA
MSPQTETKASVGFKAGVKDYKLTYYTPEYETKDTDILAAFRVTPQPGVPPEEAGAAVAAE
SSTGTWTTVWTDGLTSLDRYKGRCYGIEPVAGEENQYIAYVAYPLDLFEEGSVTNMFTSI
VGNVFGFKALRALRLEDLRIPTAYIKTFQGPPHGIQVERDKLNKYGRPLLGCTIKPKLGL
SAKNYGRAVYECLRGGLDFTKDDENVNSQPFMRWRDRFLFCAEAIFKSQAETGEIKGHYL
NATAGTCEEMMKRAIFARELGAPIVMHDYLTGGFTANTSLAHYCRDNGLLLHIHRAMHAV
IDRQKNHGMHFRVLAKALRMSGGDHIHAGTVVGKLEGEREITLGFVDLLRDDFVEKDRSR
GIYFTQDWVSLPGVLPVASGGIHVWHMPALTEIFGDDSVLQFGGGTLGHPWGNAPGAVAN
RVALEACVQARNEGRDLAREGNEIIREASKWSPELAAACEVWKEIKFEFEAMDTL
>P27065 | RBL_SOLLC

MSPQTETKASVGFKAGVKEYKLTYYTPEYQTKDTDILAAFRVTPQPGVPPEEAGAAVAAE
SSTGTWTTVWTDGLTSLDRYKGRCYRIERVVGEKDQYIAYVAYPLDLFEEGSVTNMFTSI
VGNVFGFKALRALRLEDLRIPPAYVKTFQGPPHGIQVERDKLNKYGRPLLGCTIKPKLGL
SAKNYGRAVYECLRGGLDFTKDDENVNSQPFMRWRDRFLFCAEALFKAQTETGEIKGHYL
NATAGTCEEMIKRAVFARELGVPIVMHDYLTGGFTANTTLAHYCRDNGLLLHIHRAMHAV
IDRQKNHGIHFRVLAKALRMSGGDHIHSGTVVGKLEGERDITLGFVDLLRDDFVEQDRSR
GIYFTQDWVSLPGVLPVASGGIHVWHMPALTEIFGDDSVLQFGGGTLGHPWGNAPGAVAN
RVALEACVKARNEGRDLAREGNEIIREACKWSPELAAACEVWKEIVFNFAAVDVLDK
>P27107 | RK2_SINAL
MAIHLYKTSTPSTRNGAVDSQVKSNPRNNLIYGQHHCGKGRNARGIITVRHRGGGHKRLY
RKIDFRRNTKDIYGRIVTIEYDPNRNAYICLIHYGDGEKRYILHPRGAIIGDTIVSGTEV
PIKMGNALPLTDMPLGTAIHNIEITLGKGGQLARAAGAVAKLIAKEGKSATLKLPSGEVR
LISKNCSATVGQVGNVGVNQKSLGRAGSKCWLGKRPVVRGVVMNPVDHPHGGGEGRAPIG
RKKPVTPWGYPALGRRTRKRKKYSETLILRRRSK
>P27723 | RR16_MAIZE
MVKLRLKRCGRKQQAIYRIVAIDVRSRREGRDLRKVGFYDPIKNQTCLNVPAILYFLEKG
AQPTRTVYDILRKAEFFKDKERTLS
>P27789 | FER5_MAIZE
MATVLSSPRAPAFSFSLRAAPATTVAMTRGASSRLRAQATYNVKLITPEGEVELQVPDDV
YILDYAEEEGIDLPYSCRAGSCSSCAGKVVSGSLDQSDQSFLDDSQVADGWVLTCVAYPT
SDVVIETHKEDDLIS
>P27818 | ILVB1_BRANA
MAAATSSSPISLTAKPSSKSPLPISRFSLPFSLTPQKDSSRLHRPLAISAVLNSPVNVAP
PSPEKTDKNKTFVSRYAPDEPRKGADILVEALERQGVETVFAYPGGASMEIHQALTRSST
IRNVLPRHEQGGVFAAEGYARSSGKPGICIATSGPGATNLVSGLADAMLDSVPLVAITGQ
VPRRMIGTDAFQETPIVEVTRSITKHNYLVMDVDDIPRIVQEAFFLATSGRPGPVLVDVP
KDIQQQLAIPNWDQPMRLPGYMSRLPQPPEVSQLGQIVRLISESKRPVLYVGGGSLNSSE
ELGRFVELTGIPVASTLMGLGSYPCNDELSLQMLGMHGTVYANYAVEHSDLLLAFGVRFD
DRVTGKLEAFASRAKIVHIDIDSAEIGKNKTPHVSVCGDVKLALQGMNKVLENRAEELKL
DFGVWRSELSEQKQKFPLSFKTFGEAIPPQYAIQILDELTEGKAIISTGVGQHQMWAAQF
YKYRKPRQWLSSSGLGAMGFGLPAAIGASVANPDAIVVDIDGDGSFIMNVQELATIRVEN
LPVKILLLNNQHLGMVMQWEDRFYKANRAHTYLGDPARENEIFPNMLQFAGACGIPAARV
TKKEELREAIQTMLDTPGPYLLDVICPHQEHVLPMIPSGGTFKDVITEGDGRTKY
>P28252 | ACCD_ANGLY
MSLINWFEEKRKFGGLIGAFIEKATKGYVLSEREKDRYINVDTNKGLWTRCDNCENMLYI
KFLKQNKGVCEECGYHLQINSTERIELLIDRDTWIPMDEDMVAQDVLKFSDEDSYRNRIS
LSQKRTGLTDAVQTGIGNLNGTPVALGVMDFQSMGGSMGSVVGEKITRLIEYATQESLPL
IIVCASGGARMQEGTLSSMQMAKISSVSQIHQVQKKLLYIAVLTYPTTGGVTASFGMLGD
IIIAEPKAYIAFAGKRVIEQTLRQKIPDGFQVAESLFDHGLLDSIVPRNLLKGVLSEIFE
LYDLAPWKEKNNQV
>P28258 | RBL_ANGLY
MSPQTETKTGFGFKAGVKDYRLNYYTPEYETKDTDILAAFRMTPQPGVPPEEAGAAVAAE
SSTGTWTTVWTDGLTSLDRYKGRCYDIEPVAGEENQYIAYVAYPLDLFEEGSVTNMFTSI
VGNVFGFKALRALRLEDLRVPPAYSKTFQGPPHGIQAERDKLNKYGRPLLGCTIKPKLGL

SAKNYGRAVYECLRGGLDFTKDDENVNSQPFMRWRDRFLFVAEALFKSQAETGEVKGHYL
NATAGTCEEMMKRAIFARELGAPIVMHDYLTGGFTANTSLAHYCRDNGLLLHIHRAMHAV
IDRQRNHGMHFRVLAKALRMSGGDHVHAGTVVGKLEGEREVTLGFVDSLRDDYIEKDRSR
GIYFTQDWVSMPGVFPVASGGIHVWHMPALTEIFGDDSVLQFGGGTLGHPWGNAPGAVAN
RVASEACVQARNEGRDLAREGNEIIREASKWSPELAAACEVWKEIKFEFETIDTL
>P28399 | RBL_DIGPU
MSPQTETKASVGFKAGVKEYKLTYYTPEYETKDTDILAAFRVTPQPGVPPEEAGAAVAAE
SSTGTWTTVWTDGLTSLDRYKGRCYHIEPVPGEADQYICYVAYPLDLFEEGSVTNMFTSI
VGNVFGFKALRALRLEDLRIPVAYVKTFQGPPHGIQVERDKLNKYGRPLLGCTIKPKLGL
SAKNYGRACYECLRGGLDFTKDDENVNSQPFMRWRDRFLFCAEAIYKSQAETGEIKGHYL
NATAGTCEEMMKRAVFARELGVPIIMHDYLTGGFTANTSLAHYCRDNGLLLHIHRAMHAV
IDRQKNHGIHFRVLAKALRMSGGDHIHSGTVVGKLEGEREITLGFVDLLRDDFIEKDRSR
GIYFTQDWVSLPGVIPVASGGIHVWHMPALTEIFGDDSVLQFGGGTLGHPWGNAPGAVAN
RVALEACVKARNEGRDLAQEGNEIIREACKWSPELAAACEVWKEIVFNFAAVDVLDK
>P28441 | RBL_PLAOC
MSPQTETKASVGFKAGVKDYKLTYYTPDYETKDTDILAAFRVTPQPGVPPEEAGAAVAAE
SSTGTWTTVWTDGLTSLDRYKGRCYHIEPVAGEENQYIAYVAYPLDLFEEGSVTNMFTSI
VGNVFGFKALRALRLEDLRIPPAYSKTFQGPPHGIQVERDKLNKYGRPLLGCTIKPKLGL
SAKNYGRAVYECLRGGLDFTKDDENVNSQPFMRWRDRFLFCAEAIYKAQAETGEIKGHYL
NATAGTCEEMIKRAVFARELGVPIVMHDYLTGGFTANTSLAHYCRDNGLLLHIHRAMHAV
IDRQKNHGIHFRVLAKALRMSGGDHIHAGTVVGKLEGEREITLGFVDLLRDDFIEKDRSR
GIYFTQDWVSLPGVLPVASGGIHVWHMPALTEIFGDDSVLQFGGGTLGHPWGNAPGAVAN
RVALEACVQARNEGRDLAREGNEIIREACKWSPELAAACEVWKEIKFEFEAMDTL
>P28459 | RBL_VIBAC
MSPQTETKASAGFKAGVKDYKLTYYTPEYETKDTDILAAFRVTPQPGVPPEEAGAAVAAE
SSTGTWTTVWTDGLTSLDRYKGRCYHIEPVAGEESQFIAYVAYPLDLFEEGSVTNMFTSI
VGNVFGFKALXALRLEDLRIPVAYVKTFQGPPHGIQVERDKLNKYGRPLLGCTIKPKLGL
SAKNYGRAVYECLRGGLDFTKDDENVNSQPFMRWRDRFLFCAEAIYKAQAETGEIKGHYL
NATAGTCEDMMKRAVFARELGVPIVMHDYLTGGFTANTTLAHYCRDNGLLLHIHRAMHAV
IDRQKNHGMHFRVLAKALRMSGGDHIHAGTVVGKLEGEREITLGFVDLLRDDFIEKDRSR
GIYFTQDWVSLPGVLPVRSGGIHVWHMPALTEIFGDDSVLQFGGGTLGHPWGNAPGAVAN
RVALEACVQARNEGRDLAREGNEVIRXASKWSPELAAACEVWKEIKFEFEAMDTL
>P28553 | CRTI_SOYBN
MAACGYISAANFNYLVGARNISKFASSDATISFSFGGSDSMGLTLRPAPIRAPKRNHFSP
LRVVCVDYPRPELENTVNFVEAAYLSSTFRASPRPLKPLNIVIAGAGLAGLSTAKYLADA
GHKPILLEARDVLGGKVAAWKDKDGDWYETGLHIFFGAYPYVQNLFGELGINDRLQWKEH
SMIFAMPNKPGEFSRFDFPEVLPSPLNGIWAILRNNEMLTWPEKVKFAIGLLPAMLGGQP
YVEAQDGLSVQEWMKKQGVPERVADEVFIAMSKALNFINPDELSMQCILIALNRFLQEKH
GSKMAFLDGNPPERLCMPIVDYIQSLGGEVHLNSRIQKIELNDDGTVKSFLLNNGKVMEG
DAYVFATPVDILKLLLPDNWKGIPYFQRLDKLVGVPVINVHIWFDRKLKNTYDHLLFSRS
PLLSVYADMSVTCKEYYSPNQSMLELVFAPAEEWISRSDDDIIQATMTELAKLFPDEISA
DQSKAKILKYHVVKTPRSVYKTVPNCEPCRPIQRSPIEGFYLAGDYTKQKYLASMEGAVL
SGKLCAQAIVQDSELLATRGQKRMAKASVV
>P28644 | ROC1_SPIOL

CVAQTSEWEQEGSTNAVLEGESDPEGAVSWGSETQVSDEGGVEGGQGFSEPPEEAKLFVG
NLPYDVDSEKLAGIFDAAGVVEIAEVIYNRETDRSRGFGFVTMSTVEEAEKAVELLNGYD
MDGRQLTVNKAAPRGSPERAPRGDFEPSCRVYVGNLPWDVDTSRLEQLFSEHGKVVSARV
VSDRETGRSRGFGFVTMSSESEVNDAIAALDGQTLDGRAVRVNVAEERPRRAF
>P28805 | RK33_SPIOL
MAKGKDVRVKVILECTGCVRKSVNKGSRGVSRYITQKNRHNTPSRLELRKFCPYCYKHTI
HGEIKK
>P29036 | FRI1_MAIZE
MMLRVSPSPAAAVPTQLSGAPATPAPVVRVAAPRGVASPSAGAACRAAGKGKEVLSGVVF
QPFEEIKGELALVPQSPDKSLARHKFVDDCEAALNEQINVEYNASYAYHSLFAYFDRDNV
ALKGFAKFFKESSDEEREHAEKLMEYQNKRGGRVRLQSIVTPLTEFDHPEKGDALYAMEL
ALALEKLVNEKLHNLHGVATRCNDPQLTDFIESEFLEEQGEAINKISKYVAQLRRVGKGH
GVWHFDQMLLEEEA
>P29108 | STAD_BRANA
MALKLNPLASQPYNFPSSARPPISTFRSPKFLCLASSSPALSSKEVESLKKPFTPPKEVH
VQVLHSMPPQKIEIFKSMEDWAEQNLLTQLKDVEKSWQPQDFLPDPASDGFEDQVRELRE
RARELPDDYFVVLVGDMITEEALPTYQTMLNTLDGVRDETGASPTSWAIWTRAWTAEENR
HGDLLNKYLYLSGRVDMRQIEKTIQYLIGSGMDPRTENNPYLGFIYTSFQERATFISHGN
TARQAKEHGDLKLAQICGTIAADEKRHETAYTKIVEKLFEIDPDGTVMAFADMMRKKISM
PAHLMYDGRDESLFDNFSSVAQRLGVYTAKDYADILEFLVGRWKIESLTGLSGEGNKAQE
YLCGLTPRIRRLDERAQARAKKGPKVPFSWIHDREVQL
>P29684 | RBS_HEVBR
MASSMLSTAAVACINRASPAQASMVAPFTGLKSTSAFPTTRKTTTDITSIASNGGRVQCM
QVWPPRGKKFYETLSYLPPLTREQLAKEVEYLLRKGWVPCLEFELEHGTVYREYHRSPGY
YDGRYWTMWKLPMFGCTDAVQVLQELDEMIKAYPDCYGRIIGFDNVRQVQCISFLAYKPK
GAE
>P30523 | GLGS_WHEAT
MDVPLASKTFPSPSPSKREQCNIDGHKSSSKHADLNPHVDDSVLGIILGGGAGTRLYPLT
KKRAKPAVPLGANYRLIDIPVSNCLNSNISKIYVRTQFNSASLNRHLSRAYGSNIGGYKN
EGFVEVLAAQQSPDNPDWFQGTADAVRQYLWLFEEHNVMEYLILAGDHLYRMDYEKFIQA
HRETDADITVAALPMDEERATAFGLMKIDEEGRIIEFAEKPKGEQLKAMMVDTTILGLDD
ARAKEMPYIASMGIYVISKHVMLQLLREQFPGANDFGSEVIPGATSTGMRVQAYLYDGYW
EDIGTIEAFYNANLGITKKPIPDFSFYDRSAPIYTQPRHLPPSKVLDADVTDSVIGEGCV
IKNCKIHHSVVGLRSCISEGAIIEDTLLMGADYYETEADKKLLAEKGGIPIGIGKNSHIK
RAIIDKNARIGDNVMIINVDNVQEAARETDGYFIKSGIVTVIKDALLPSGTVI
>P30829 | RBL_MAGMA
MSPKTETKASVGFKAGVKEYKLTYYTPEYETKDTDILAAFRVTPQPGVPPEEAGAAVAAE
SSTGTWTTVWTDGLTSLDRYKGRCYHIEPVPGEESQFIAYVAYPLDLFEEGSVTNMFTSI
VGNVFGFKALRALRLEDLRIPTAYVKTFQGPPHGIQVERDKLNKYGRPLLGCTIKPKLGL
SAKNYGRAVYECLRGGLDFTKDDENVNSQPFMRWRDRFLFCAEALYKAQAETGEIKGHYL
NATAGTCEEMMKRAIFARELGVPIVMHDYLTGGFTANTSLAHYCRDNGLLLHIHRAMHAV
IDRQKNHGIHFRVLAKALRMSGGDHIHSGTVVGKLEGERDITLGFVDLLRDDFIEKDRSR
GIYFTQDWVSLPGVLPVASGGIHVWHMPALTEIFGDDSVLQFGGGTLGHPWGNAPGAVAN
RVALEACVQARNEGRDLASQGNEIIREASKWSPELAAACEVWKEIKFEFKAVDTL

>P30830 | RBL_PERAE
MSPKTETKASVGFKAGVKDYKLTYYTPDYETKSTDILAAFRVTPQPGVPPEEAGAAVAAE
SSTGTWTTVWTDGLTSLDRYKGRCYHIEPVAGEESQFIAYVAYPLDLFEEGSVTNMFTSI
VGNVFGFKALRALRLEDLRIPPAYSKTFQGPPHGIQVERDKLNKYGRPLLGCTIKPKLGL
SAKNYGRAVYECLRGGLDFTKDDENVNSQPFMRWRDRFLFCAEAIYKSQAETGEIKGHYL
NATAGTCEEMIKRAVFARELGVPIVMHDYLTGGFTANTTLAHYCRDNGLLLHIHRAMHAV
IDRQKNHGMHFRVLAKALRMSGGDHIHAGTVVGKLEGERDITLGFVDLLRDDFIEKDRSR
GIYFTQDWVSMPGVLPVASGGIHVWHMPALTEIFGDDSVLQFGGGTLGQPWGNAPRAVAN
RVALEACVQARNEGRDLAREGNEIIREASKWSPELAAACEVWKEIKFEFAEWIPCNQ
>P31162 | RR19_PEA
MTRSRKKNPFVANHLLKKIKKLNTKGEKAIIKTWSRKSTIIPIMIGHTIAIHNGKEHLPV
YITDRMVGHKLGEFSPTLNFGGFAKNDNKSRR
>P31163 | RK2_PEA
MAIHLSKTSSPSTRNGAVNSQVKSNSRNRLISGQHHCGKGRNPRGIITAGHRGGGHKRLY
RKIDFRRNEKDIYGRIITIEYDPNRNAHICLIHYGDGEKRYILHPKAIIGDTIVYGTEVP
IKMGNALPLTDMPLGTAIHNIEITLGKGGQLARAAGAVAKLIAKEGKSATLKLPSGEVRL
ISKNCSATVGQVGNVGVNQKSLGRVGAKRWRGKRPVVRGVVMNPVDHPHGGGEGRAPIGR
KKPSTPWGYPALGRRTRKSNKYSDNLILRRRSK
>P31593 | GSA_TOBAC
MAAVNGVGISWPSKLTQGQRPKLVFSPSPRRCTPSSSTIKMTASVDEKKKTFTLQKSEEA
FSKAKELMPGGVNSPVRAFKSVGGQPIIIDSVKGSRMRDIDGNEYIDYVGSWGPAIIGHA
DDEVLAALAETMKKGTSFGAPCLLENTLAEMVISAVPSIEMVRFVNSGTEACMGVLRLAR
AFTGRPKIIKFEGCYHGHADPFLVKAGSGVATLGLPDSPGGPKAATSDTLTAPYNDISAV
ESLFEEHKGEVAAIILEPVVGNAGFIQPNLDFLAAIRKITKENDALLIFDEVMTGFRLAY
GGAQEYFGITPDLTTLGKIIGGGLPVGAYGGRRDIMEMVAPAGPMYQAGTLSGNPLAMTA
GIHTLKRLQGPGTYEYLDKITGELTQGILDAGKKTGHAMCGGYIRGMFGFLFAEGPVNNF
SDAKKSDTEKFGRFYRGMLEEGVYFAPSQFEAGFTSLAHTSEDIQKTVAAAEKVLKQI
>P32087 | RR16_SOLTU
MVKLRLKRCGRKQRAVYRIVAIDVRSRREGKDLQKVGFYDPIKNQTYLNVPAILYFLEKG
AQPTETVQDILKKAEVFKELRLNQPKFN
>P32088 | MATK_SOLTU
MEEIHRYLQPDSSQQHNFLYPLIFQEYIYALAQDHGLNRNRSILLENSGYNNKLSFLIVK
RLITRMYQQNHFIISTNDSNKNPFLGCNKSLYSQMISEGFACIVEIPFSIRLISSLSSFE
GKKIFKSHNLRSIHSTFPFLEDNFAHLNYVLDILIPYPVHLEILVQTLRYWVKDASSLHL
LRFFLHEYCNLNSLITSKKPGYSFSKKNQRFFFFLYNSYVYECESTFVFLRNQSSHLRST
SFGALLERIYFYGKIERLVEVFAKDFQVTLWLFKDPFMHYVRYEGKSILASKGTFPLMNK
WKFYLVNFWQCHFSMYFHTGRIHINQLSNHSRDFMGYLSSVRLNHSMVRSQMLENSFLIN
NPIKKFETLVPIIPLIGSLAKAHFCTVLGHPISKPVWSDLSDSDIIDRFGRICRNLFHYY
SGSSKKKTLYRIKYILRLSCARTLARKHKSTVRTFLKRSGSELLEEFLTSEEQVLSLTFP
RASSSLWGVYRSRIWYLDIFCINDLANYQ
>P32976 | RR14_CHLVU
MAKKSMIERDRKRARLITKYAAKRKNLLVEIKTATSLEDKFNLHRKLQQLPRNSAPVRSH
NRCTITGRPRGYFRDFGLSRHVLREYALQGFLPGVVKASW
>P34915 | RBL_PHYPA

MSPRPEIKAGVGFKAGVKDYRLTYYTPDYQTKDTDILAAFRMTPQPGVPAEECGAAVAAE
SSTGTWTTVWTDGLTSLDRYKGRCYAIEPVAGEENQYIAYVAYPLDLFEEGSVTNLFTSI
VGNVFGFKALRALRLEDLRIPPAYSKTFQGPPHGIQVERDKLNKYGRPLLGCTIKPKLGL
SAKNYGRAVYECLRGGLDFTKDDENVNSQPFMRWRDRFLFCAEAIYKSQGETGEIKGHYL
NATAGTCEEMIKRAQFARELGMPIVMHDYLTGGFTANTSLAHYCRDNGLLLHIHRAMHAV
LDRQKNHGMHFRVLAKALRLSGGDHIHSGTVVGKLEGERQVTLGFVDLLRDDYIEKDRSR
GIYFTQDWVSLPGVLPVASGGIHVWHMPALTEIFGDDSVLQFGGGTLGHPWGNAPGAVAN
RVALEACVQARNEGRDLAREGNEIIREAAKWSPELAAACEVWKEIKFEFDTVDTL
>P36476 | RBL_ADICA
MSPQTETKAGVGFKAGVKDYRLTYYTPEYKTKDTDILAAFRMTPQPGVPAEEAGAAVAAE
SSTGTWTTVWTDGLTSLDRYKGRCYDIEPVAGEENQYIAYVAYPLDLFEEGSVTNMLTSI
VGNVFGFKALRALRLEDLRIPPAYSKTFLGPPHGIQVERDKLNKYGRPLLGCTIKPKLGL
SAKNYGRAVYECLRGGLDFTKDDENVNSQPFMRWRDRFLFVAEALFKSQAETGEIKGHYL
NATAGTCEEMMKRAVFARELGAPIVMHDYLTGGFTANTSLAFYCRDNGLLLHIHRAMHAV
IDRQKNHGIHFRVLAKALRMSGGDHIHAGTVVGKLEGEREVTLGFVDLLRDDYIEKDRSR
GIYFTQDWVSMPGVFPVASGGIHVWHMPALTEIFGDDSVLQFGGGTLGHPWGNAPGAVAN
RVALEACVQARNEGRDLAREGNEIIREASKWSPELAAACEIWKAIKFEFETIDTL
>P36493 | RK32_SOLLC
MAVPKKRTSTSKKRIRKNIWKRKGYWVALKAFSLAKSLSTGNSKSFFVRQTKINK
>P37215 | AROF_SOLLC
MALSNTLSLSSSKSLVQSHLLHNPTPQPRFSLFPTTQHGRRHPISAVHAAEPSKTAVKQG
KWSLDSWKTKKALQLPEYPDEKELESVLKTLEMNPPLVFAGEARSLEEKLGEAALGKAFL
LQGGDCAESFKEFNANNIRDTFRILLQMSVVLMFGGQVPVIKVGRMAGQFAKPRSDPFEE
INGVKLPSYKGDNINGDTFDEKSRIPDPHRLIRAYMQSAATLNLLRAFATGGYAAMQRVT
EWNLDFVENSEQGDRYQELAHRVDEALGFMAAAGLTVDHPIMSTTDFWTSHECLLLPYEQ
ALTREDSTSGLFYDCSAHMVWVGERTRQLDGAHVEFLRGVANPLGIKVSQKMDPKELIKL
IDILNPANKPGRITVIVRMGAENMRVKLSHLVRAVRGAGQIVTWVCDPMHGNTIKAPCGL
KTRAFDSIQAEVRAFFDVHEQEGSHPWCIHLEMTGQNVTECIGGSRTVTYDDLGSRYHTH
CDPRLNASQSLELSFIVAERLRRRRMSSQRL
>P37216 | AROG_SOLLC
MALSTNTTTNSLLSNKSLLQNQPLLSSPSKNAFFSNKSTKTVRFVQPIAAVHSSDSNKNP
IVSDKPTKSSPPAATATTAPAPAVTKTEWAVDSWKSKKALQLPEYPDQEELRSVLKTIDE
FPPIVFAGEARSLEERLGEAAMGRAFLLQGGDCAESFKEFNANNIRDTFRILLQMGAVLM
FGGQMPVIKVGRMAGQFAKPRSDSFEEKDGVKLPSYRGDNVNGDAFDVKSRTPDPQRLIR
AYCQSAATLNLLRAFATGGYAAMQRINQWNLDFTEHSEQGDRYRELASRVDEALGFMTAA
GLTMDHPIMKTTEFWTSHECLLLPYEQSLTRRDSTSGLHYDCSAHFLWVGERTRQLDGAH
VEFLRGIANPLGIKVSDKMDPSALVKLIEILNPQNKAGRITIITRMGAENMRVKLPHLIR
AVRRAGQIVTWVSDPMHGNTIKAPCGLKTRPFDSIRAEVRAFFDVHDQEGSHPGGVHLEM
TGQNVTECIGGSRTVTFDDLSSRYHTHCDPRLNASQSLELSFIIAERLRKRRLGSQSTLG
Q
>P38550 | RPOC2_TOBAC
MAERANLVFHNKAINGTAMKRLISRLIDHFGMAYTSHILDQVKTLGFQQATATSISLGID
DLLTIPSKGWLVQDAEQQSLILEKHHHYGNVHAVEKLRQSIEIWYATSEYLRQEMNPNFR
MTDPFNPVHIMSFSGARGNASQVHQLVGMRGLMSDPQGQMIDLPIQSNLREGLSLTEYII

SCYGARKGVVDTAVRTSDAGYLTRRLVEVVQHIVVRRTDCGTARGISVSPRNGMMPERIF
IQTLIGRVLADDIYMGPRCIATRNQDIGIGLVNRFITFRAQPISIRTPFTCRSTSWICRL
CYGRSPTHGDLVELGEAVGIIAGQSIGEPGTQLTLRTFHTGGVFTGGTAEHVRAPSNGKI
KFNEDLVHPTRTRHGHPAFLCSIDLYVTIESEDILHNVNIPPKSLLLVQNDQYVESEQVI
AEIRAGISTLNFKEKVRKHIYSDSDGEMHWSTDVYHAPEFTYGNVHLLPKTSHLWILLGR
PCRSSLVYLSIHKDQDQMNAHFLSGKRRYTSNLSVTNDQARQKLFSSDFSGKKEDRIPDY
SDLNRIICAGQYNLVYSPILHENSDLLSKRRRNKFIIPLHSIQELENELMPCSGISIEIP
VNGIFRRNSILAYFDDPRYRRKSSGIIKYGTVETHSVIKKEDLLEYRGVKEFRPKYQMKV
DRFFFIPEEVHILPGSSSIMVRNNSIVGVDTQITLNLRSRVGGLVRVERKKKRIELKIFS
GDIHFPGETDKISRHTGVLIPPGTGKRNSKESKKVKNWIYVQRITPSKKKFFVLVRPVVT
YEITDGINLATLFPPDPLQERDNVQLRIVNYILYGNGKPIRGISDTSIQLVRTCLVLNWN
QDKKSSSCEEARASFVEIRTNGLIRHFLRINLVKSPISYIGKRNDPSGSGLLSDNGSDCT
NINPFSSIYSYSKAKIQQSINQPQGTIHTLLNRNKECQSLIILSAANCSRMGPFKDVKYH
SVIKKSIKKDPLIPIRNSLGPLGTSLPIENFYSSYHLITHNQILVTNYLQLDNLKQTFQV
IKFKYYLMDENGKIFNPDPCRNIILNPFNLNWYFLHHNYCEETSKIISLGQFICENVCIA
KNGPPLKSGQVILVQVDSIVIRSAKPYLATPGATVHGHYGETLYEGDTLVTFIYEKSRSG
DITQGLPKVEQVLEVRSVDSISMNLEKRIEGWNKCITRILGIPWGFLIGAELTIAQSRIS
LVNKIQQVYRSQGVQIHNRHLEIIVRQITSKVLVSEDGMSNVFSPGELIGLLRAERMGRA
LEEAICYRVVLLGITRASLNTQSFISEASFQETARVLAKAALRGRIDWLKGLKENVVLGG
VIPVGTGFKGLVHPSKQHNNIPLETKKKNLFEGEMRDILFHHKKLFDSCLSKNFHDIPEQ
SFIGFNDS
>P41096 | RK2_HORVU
MAKHLYKTPIPSTRKGTVDRQVKSNPRNNLIHGRHRCGKGRNSRGIITARHRGGGHKRLY
RKIDFRRNQKDISGRIVTIEYDPNRNAYICLIHYGDGEKRYILHPRGAIIGDTIVSGTKV
PISMGNALPLTDMPLGTAMHNIEITRGRGGQLARAAGAVAKLIAKEGKSATLRLPSGEVR
LVSQNCLATVGQVGNVGVNQKSLGRAGSKCWLGKRPVVRGVVMNPVDHPHGGGEGKAPIG
RKKPTTPWGYPALGRRTRKRKKYSDSFILRRRK
>P41606 | RPOC2_PINTH
MKIWRFFLMKERTRLPFDNLPFYNKVMDKTAIKKLISRLIDHFGMTYTSHILDQLKTSGF
QQATDTAISLGIDDLLTAPSKGWLVQDAEQQGSVSEKQNHYGNVHAVEKLRQSIEIWYAT
SEYLRKEMNPNFSMTDPLNPVHVMSFSGARGSTSQVHQLVGMRGLMSDPQGQIIDLPIRR
NLREGLSLTEYIISCYGARKGVVDTAVRTADAGYLTRRLVEVVQHIVVRRTDCGTIQGIF
VSPIRGRERDRNEIVVRTQILIGRVLADDVYINRRCIATRNQDIGVGLANQLINLRTQPI
YIRTPFTCKSISRICQLCYGRSTTHSHLIELGEAVGIIAGQSIGEPGTQLTLRTFHTGGV
FTGDIAEHIRAPFNGKIEFNENLVYPTRTRNGHPAYLCHNNLSITIDGQDQVQNLTIPPQ
SLLLVQNDQYVESEQLIAEVRARTSSFKEKVRKNIYSDLEGEMHWSTNVCHAPEYVQGNV
HPILRTGYLWILSGGIYGSRVVPFPFHKYQDQVYVQPFVAKHQSLSDSYVDQVEHRSGDS
NCYEKEEQIFSYSETETDRTISNKHRDSIYVFYPNNYNIKGKKQMNRFIVSLQCDKEWEK
KIIPCPDAILRIPKSGILQRNSIFGYSNVEHGIPDGSNMTTPFSLDLSREGDNLQIQISY
SISYEDGERIQVMSDISIPLVRTCIGFDWEQIDSIESEAYVSLISVRTNKIVNNMVQISL
MKYPPFFMGRRDNKTSPNLMFHNNLDHTNLLSSNGASQLISKHQGIICSLSNGEEDSGSF
MVLSPSDYFRIVLFNDSKCYDTGNQSNRKDPMRKIIEFSGLLGNLHSITNRFPSSHFLTY
KKVLSKKHSIFHNSFNTFQVPKYYFMDENMIIYHFDPCRNIISNLLGPNWCSSSSESEFC
EKTFPVVSLGQLIPESVWISEDEPLPESGQIIAVDEESLVIRSAKPYLATRKATVHSHYG

KILDKGDTLITLIYERFKSSDIIQGLPKVEQLSEARLNNSISMNLKESFENWTGDMTRFL
GSLWGLFISARITMEQSQIHLVNQIQKVYRSQGVRIGDKHIEVIVRQMTSKVLISEDGTA
NVFSPGELIVLSRAQRMDRALEEAIYYQTMLLGITRASLNTQSFISEASFQETARVLAKA
ALQGRIDWLKGLKENVILGGMIPAGTGQHIHRSGKRNGIDPRIGNRNLFSNKVKDILFHH
DKVDFFSFQDNSYKYHNILKQQLK
>P41609 | CLPP_PINTH
MPVGVPKVPFRAPGDEDATWVDLYNRLYRERLLFLAQDINHEIANQLMGLMVYLSAEDSN
KDIFSFINCPGGSVIPGVGLFDMMQAIVPDVHTICMGVAASMGSFILIGGEMPKRIALPH
ARIMIHQPASSYYDGSAADFHNESKHVTMLRDYITRCYIERTDQPGEVIQRDLNRDVFMS
ATEAQAYGIVDVVAEG
>P41621 | RBL_PINTH
MSPKTETKASVGFKAGVKDYRLTYYTPEYQTKDTDILAAFRVTPQPGVPPEEAGAAVAAE
SSTGTWTTVWTDGLTSLDRYKGRCYDIEPVPGEETQFIAYVAYPLDLFEEGSVTNLFTSI
VGNVFGFKALRALRLEDLRIPPSYSKTFQGPPHGIQVERDKLNKYGRPLLGCTIKPKLGL
SAKNYGRAVYECLRGGLDFTKDDENVNSQPFMRWRDRFVFCAEALNKAQAETGEIKGHYL
NATAGTCEEMMKRAIFARELGVPIVMHDYLTGGFTANTSLAHYCRDNGLLLHIHRAMHAV
IDRQRNHGMHFRVLAKALRMSGGDHIHAGTVVGKLEGERDVTLGFVDLLRDDFIEKDRSR
GIYFTQDWVSMPGVLPVASGGIHVWHMPALTEIFGDDSVLQFGGGTLGHPWGNAPGAVAN
RVALEACVQARNEGRDLAREGNEVIREACKWSPELAAACEIWKEIKFEFDVIDRL
>P41630 | RR11_PINTH
MSKTIKRIGSRRNEHRVLKGVIYVQASFNNTIVTATDVRGQVLSWSSAGACGFKGTRRGT
PFAAQTAAENVIRALMDRGMERVEVMISGPGRGRDTALRTIRRSGILLSFVRDVTPMPHN
GCRPPKKRRV
>P41641 | RR14_PINTH
MARKSLIQREKKRQALERKYHLIRQSLEEKSKVSSLDDKWEIHRKLQSSPRNSAPTRLHR
RCSSTGRPRANYRDFGLSGHILREMAHACLLPGIKKSSW
>P41645 | CHLL_PINTH
MKIAVYGKGGIGKSTTSCNISVALARRGQKVLQIGCDPKHDSTFTLTGFLIPTIIDTLQS
KDYHYEDIWPEDVIHKGYGGVDCVEAGGPPAGAGCGGYVVGETVKLLKELNAFYEYDIIL
FDVLGDVVCGGFAAPLNYADYCVIITDNGFDALFAANRITASIREKARTHPLRLAGLVGN
RTSRRDLINKYVEACPMPVIEVLPIIEDIRVSRVKGKTLFEMVGFEPSLNYVCNYYLGIA
DQILSQPEGIVPKEIPDRELFSLLSDLYLNPIGGGGQKKNNKENLLGFTRI
>P41646 | CHLN_PINTH
MSTKIVETITLECETGNYHSFCPISCVSWLYQKIEDSFFLVVGTKTCGYFLQNALGVMIF
AEPRYAMAELEEGDISAHLNDYEELKTLCIRIRKDRDPSVIIWIGTCTTEIIKMDLEGMA
PKLEYEIGVPILVARANGLDYAFTQGEDTVLAVMAHRCPDQELPIGESKETKTKLFPFPL
LKEKNLVEYANHPPLVIFGSLPSNLVSQLDTELRRQFIKVSGWLPAQRYADLPSLGDGVY
VCGVNPFLSRTATTLIRRKKCELIVAPFPIGPDGTRAWIERICPVFGIEAQSLEEREERI
WESLKDYLDLVRGKSVFFMGDNLIEISIARFLIRCGMIVYEIGIPYMDKRYQAAELALLQ
NTCIRMCMPIPRIVEKPDNSNQIRRMRELQPDLAITGMAHANPLGARGIGTKWSVEFTFA
QIHGFANARDVLELVTRPLRRNENLDNLDRTTLVRKNNELYTSTPVK
>P41648 | RR15_PINTH
MINNLSISSSLIPDKQRGSVESQVFYLTNRVLRLTQHLQLHGRDYSSQRGLWKILSKRKQ
LLVYLSKRDKLRYDDLIGQLGIRGLKTR

>P41651 | RK32_PINTH
MAVPKKRTSRSKKKIRKNVRKGKAYRAAIKAFSLAKSISTGHSKSFYCIVNDDSSGSSES
KLTAIDLDDP
>P41652 | RR7_PINTH
MSRRSTAEKKTAKSDPIYHNRLVNMVVNRILKNGKKSLAYRILYRAIKKIQQKTDKNPLS
VLRQAIRRVTPNVTVKARRVGGSTYRVPTEIRSTQGKVLAIRWLLGASRKRPGRNMNFKL
SHELMDAARGNGNAIRKKEETHRMAEANRAFAHFR
>P41758 | PGKH_CHLRE
MALSMKMRANARVSGRRVAAVAPRVVPFSSASSSVLRSGFALRCLWTSAAWAALASVVEA
VKKSVGDLHKADLEGKRVFVRADLNVPLDKATLAITDDTRIRAAVPTLKYLLDNGAKVLL
TSHLGRPKGGPEDKYRLTPVVARLSELLGKPVTKVDDCIGPEVEKAVGAMKNGELLLLEN
CRFYKEEEKNEPEFAKKLAANADLYVNDAFGTAHRAHASTEGVTKFLKPSVAGFLLQKEL
DYLDGAVSNPKRPFVAIVGGSKVSSKITVIEALMEKCDKIIIGGGMIFTFYKARALKVGS
SLVEDDKIELAKKLEEMAKAKGVQLLLPTDVVVADKFDANANTQTVPITAIPDGWMGLDI
GPDSVKTFNDALADAKTVVWNGPMGVFEFPQVRQRTVSIANTLAGLTPKGCITIIGGGDS
VAAVEQAGVAEKMSHISTGGGASLELLEGKVLPGVAALDEK
>P42044 | HEMH_CUCSA
MDAASSSLALSNIKLHGSTNTLNSDQRISSLCSLPKSRVTFSCKTSGNLQVRDRSTGLVV
SCSSSNGDRDVIQGLHLSGPIEKKSRLGQACCSVGTFTVGEFALESQSQAVDDKVGVLLL
NLGGPETLDDVQPFLYNLFADPDIIRLPRLFRFLQEPLAKLISTYRAPKSKEGYASIGGG
SPLRKITDEQAQALKMALAEKNMSTNVYVGMRYWYPFTEEAIQQIKRDGITRLVVLPLYP
QYSISTTGSSIRVLQKMFREDAYLSSLPVSIIKSWYQREGYIKSMADLMQAELKNFANPQ
EVMIFFSAHGVPVSYVENAGDPYKDQMEECICLIMQELKARGIGNEHTLAYQSRVGPVQW
LKPYTDEVLVELGQKGIKSLLAVPVSFVSEHIETLEEIDMEYKHLALESGIQNWGRVPAL
NCNSSFISDLADAVIEALPSATALAPHTSSTDADDHDPFLYAIKLLFGSVLAFILLLSPK
AFMVFRNNFLLNYTRIYGYRGERSEFFWVRLIFT
>P42045 | HEMH_HORVU
MECVRSGALDLGRSGNFLGKSGSTTSCGKVRCSTNLAGSTKCEQNLHGKAKPLLLSASGK
ARGTSGLVHRSPVLKHQHHLSVRSTSTDVCTTFDEDVKGVSSHAVEEKVGVLLLNLGGPE
TLNDVQPFLFNLFADPDIIRLPRLFRFLQRPLAKLISTFRAPKSNEGYASIGGGSPLRKI
TDEQANALKVALKSKNLEADIYVGMRYWYPFTEEAIDQIKKDKITKLVVLPLYPQYSIST
SGSSIRVLQNIVKEDPYFAGLPISIIESWYQREGYVKSMADLIEKELSVFSNPEEVMIFF
SAHGVPLTYVKDAGDPYRDQMEDCIALIMEELKSRGTLNDHTLAYQSRVGPVQWLKPYTD
EVLVELGQKGVKSLLAVPVSFVSEHIETLEEIDMEYRELALESGIENWGRVPALGCTSSF
ISDLADAVVEALPSASAMATRKVKDTDSDMDMMHYLTKMFLGSVLAFFLLLSPRLVSAFR
NTLQ
>P48267 | RR7_CHLRE
MPRRPINKKRTLLPDPVYNSVSVHMLVNRVLKSGKKSVAYRIVYNALKEIGDVTQKNPVE
VFEKALDNVTPRVEVKPRRRAGAIQMVPRVLRLGDRARANSLRWIMEACDKRSGQPMVTK
LKSEILDAYKKTGFAIRKKEELHKIAIANAMYAKKPQVIINAINLLVD
>P48686 | RBL_BRAOL
MSPQTETKASVGFKAGVKEYKLNYYTPEYETKDTDILAAFRVTPQPGVPPEEAGAAVAAE
SSTGTWTTVWTDGLTSLDRYKGRCYHIEPVPGEETQFIAYVAYPLDLFEEGSVTNMFTSI
VGNVFGFKALAALRLEDLRIPPAYTKTFQGPPHGIQVERDKLNKYGRPLLGCTIKPKLGL

SAKNYGRAVYECLRGGLDFTKDDENVNSQPFMRWRDRFLFCAEAIYKSQAETGEIKGHYL
NATAGTCEEMMKRAIFARELGVPIVMHDYLTGGFTANTSLAHYCRDNGLLLHIHRAMHAV
IDRQKNHGMHFRVLAKALRLSGGDHVHAGTVVGKLEGDRESTLGFVDLLRDDYVEKDRSR
GIFFTQDWVSLPGVLPVASGGIHVWHMPALTEIFGDDSVLQFGGGTLGHPWGNAPGAVAN
RVALEACVQARNEGRDLAVEGNEIIREACKWSPELAAACEVWKEITFNFPTIDKLDGQD
>P48690 | RBL_CASSA
MSPQTETKASVGFKAGVKDYKLTYYTPDYQTKDTDILAAFRVTPQPGVPPEEAGAAVAAE
SSTGTWRTVWTDGLTSLDRYKGRCYHIEPVAGEENQFIAYVAYPLDLFEEGSVTNMFTSI
VGNVFGFKALRALRLEDLRIPTSYSKTFQGPPHGIQVERDKLNKYGRPLLGCTIKPKLGL
SAKNYGRAVYECLRGGLDFTKDDENVNSQPFMRWRDRFLFCAEAIYKAQAETGEIKGHYL
NATAGTCEEMIKRAVFARELGVPIVMHDYLTGGFTANTSLAHYCRDNGLLLHIHRAMHAV
IDRQKNHGIHFRVLAKALRMSGGDHIHAGTVVGKLEGEREITLGFVDLLRDDYIEKDRSR
GIYFTQDWVSLPGVLPVASGGIHVWHMPALTEIFGDDSVLQFGGGTLGHPWGNAPGAVAN
RVALEACVQARNEGRDLAREGNEIIREAAKWSPELAAACEVWKEIKFEFPAMDTL
>P48702 | RBL_EQUAR
MSPQTETKAGVGFKAGVKDYRLTYFTPDYETKDTDILAAFRMTPQPGVPPEEAGAAVAAE
SSTGTWTTVWTDGLTSLDRYKGRCYNIEPVAGEDNQFIAYVAYPLDLFEEGSVTNLFTSI
VGNVFGFKALRALRLEDLRIPPAYSKTFIGPPHGIQVERDKLNKYGRPLLGCTIKPKLGL
SAKNYGRAVYECLRGGLDFTKDDENVNSQPFMRWRDRFLFVAEALFKSQAETGEIKGHYL
NATAGTCEEMLKRAVFARELGAPIVMHDYLTGGFTANTSLAFYCRDNGLLLHIHRAMHAV
IDRQKNHGIHFRVLAKALRMSGGDHIHTGTVVGKLEGERDLTLGFVDLLRDDFIEKDRSR
GIYFTQDWVSMPGVLPVASGGIHVWHMPALTEIFGDDSVLQFGGGTLGHPWGNAPGAVAN
RVAVEACVQARNEGRDLATEGNDIIREAAKWSPELAAACEVWKEIKFEYEAMDTL
>P48713 | RBL_POPTM
MSPQTETKTGVGFKAGVKEYKLNYYTPEYETKDTDILAAFRVTPQPGVPPEEAGAAVAAE
SSTGTWTTVWTDGLTSLDRYKGRCYDIEPVAGEENQFIAYVAYPLDLFEEGSVTNMFTSI
VGNVFGFKALRALRLEDLRIPPAYTKTFQGPPHGIQVERDKLNKYGRPLLGCTIKPKLGL
SAKNYGRAVYECLRGGLDFTKDDENVNSQPFMRWRDRFLFCAEALYKAQTETGEIKGHYL
NATAGTCEEMMKRAIFARELGVPIVMHDYLTGGFTANTSLAHYCRDNGLLLHIHRAMHAV
IDRQKNHGIHFRVLAKALRMSGGDHIHSGTVVGKLEGERDITLGFVDLLRDDFVEKDRSR
GIYFTQDWVSLPGVLPVASGGIHVWHMPALTEIFGDDSVLQFGGGTLGHPWGNAPGAVAN
RVALEACVQARNEGRDLAREGNEIIREASKWSPELAAACEVWKEIKFEFEAMDTL
>P48714 | RBL_PSINU
MSPQTETKASSGFKAGVKDYRLTYYTPDYKVSDTDILAAFRMTPQPGVPPEEAGAAVAAE
SSTGTWTTVWTDGLTSLDRYKGRCYGIEPVAGEENQYIAYVAYPLDLFEEGSVTNMFTSI
VGNVFGFKALRALRLEDLRIPPAYSKTFMGPPHGIQVERDKLNKYGRPLLGCTIKPKLGL
SAKNYGRAVYECLRGGLDFTKDDENVNSQPFMRWRDRFLFVAEALFKSQAETGEIKGHYL
NATAGTCEEMMKRAVFARELGAPIVMHDYLTGGFTANTSLAFYCRDNGLLLHIHRAMHAV
IDRQRNHGMHFRVLAKALRMSGGDHVHAGTVVGKLEGERDVTLGFVDLLRDDYIEKDRSR
GVYFTQDWVSMPGVLPVASGGIHVWHMPALTEIFGDDSVLQFGGGTLGHPWGNAPGAVAN
RVALEACVQARNEGRDLAREGNDIIREASKWSPELAAACEVWKEIKFVFDTIDVL
>P48719 | RBL_WELMI
MSPKTETKASVGFQAGVKDYRLTYYTPEYQTKDTDILAAFRVTPQPGVPPEEAGAAVAAE
SSTGTWTTVWTDGLTSLDRYKGRCYDLEPVPGEDNQYIAYVAYPLDLFEEGSVTNMFTSI

VGNVFGFKALRALRLEDLRIPTSYIKTFQGPPHGIQVERDKLNKYGRPLLGCTIKPKLGL
SAKNYGRAVYECLRGGLDFTKDDENVNSQPFMRWRDRFVFCAEAIYKAQAETGEIKGHYL
NATAGTCEEMIKRAVFARELGVPIVMHDYLTGGFTANTTLAHYCRDNGLLLHIHRAMHAV
IDRQKNHGMHFRVLAKALRMSGGDHIHAGTVVGKLEGEREITLGFVDLLRDDFIEKDRSR
GIYFTQDWVSMPGVMPVASGGIHVWHMPALTDIFGDDAVLQFGGGTLGHPWGNAPGAVAN
RVALEACVQARNEGRDLAREGNEVIREAAKWSPELAAACEVWKEIKFEFESVDTL
>P49086 | CRTI_MAIZE
MDTGCLSSMNITGASQTRSFAGQLPPQRCFASSHYTSFAVKKLVSRNKGRRSHRRHPALQ
VVCKDFPRPPLESTINYLEAGQLSSFFRNSERPSKPLQVVVAGAGLAGLSTAKYLADAGH
KPILLEARDVLGGKVAAWKDEDGDWYETGLHIFFGAYPNIQNLFGELRIEDRLQWKEHSM
IFAMPNKPGEFSRFDFPETLPAPINGIWAILRNNEMLTWPEKVKFAIGLLPAMVGGQPYV
EAQDGLTVSEWMKKQGVPDRVNDEVFIAMSKALNFINPDELSMQCILIALNRFLQEKHGS
KMAFLDGNPPERLCMPIVDHIRSRGGEVRLNSRIKKIELNPDGTVKHFALSDGTQITGDA
YVCATPVDIFKLLVPQEWSEITYFKKLEKLVGVPVINVHIWFDRKLNNTYDHLLFSRSSL
LSVYADMSVTCKEYYDPNRSMLELVFAPADEWIGRSDTEIIDATMEELAKLFPDEIAADQ
SKAKILKYHIVKTPRSVYKTVPNCEPCRPLQRSPIEGFYLAGDYTKQKYLASMEGAVLSG
KLCAQSIVQDYSRLALRSQKSLQSGEVPVPS
>P49131 | TPT_FLAPR
MESRVLSSGATTISGIPRLTRPAGRTTTTTVVAVASPAKLNTNGGNLVWGRQLRPSLLNL
DHSSPVSLVTKPVKRDVLKPCTATASDSAGDAAPVGFFAKYPFLVTGFFFFMWYFLNVIF
NILNKKIYNYFPYPYFVSAIHLAVGVVYCLGGWAVGLPKRAPMDSNLLKLLIPVAFCHAL
GHVTSNVSFAAVAVSFTHTIKSLEPFFNAAASQFILGQSIPITLWLSLAPVVIGVSMASL
TELSFNWLGFISAMISNISFTYRSIYSKKAMTDMDSTNLYAYISIISLLFCIPPAIILEG
PQLLKHGFSDAIAKVGMTKFISDLFWVGMFYHLYNQLAINTLERVAPLTHAVGNVLKRVF
VIGFSIIVFGNKISTQTAIGTSIAIAGVAVYSLIKAKIEEEKRGLKSA
>P49132 | TPT_FLATR
MESRVLSSGATAISGVPRLTKPAGRITTTTVAVAFPARLNATGGNVVWGRQLRPSLLNLD
HSSPVSLVTKPVKRDVLKPCSATASDSAGDAAPVGFLAKYPFLVTGFFFFMWYFLNVIFN
ILNKKIYNYFPYPYFVSVIHLAVGVVYCLGSWTVGLPKRAPVDSNILKLLIPVGFCHALG
HVTSNVSFAAVAVSFTHTIKALEPFFNAAASQFVLGQSIPISLWLSLAPVVIGVSMASLT
ELSFNWLGFISAMISNISFTYRSIYSKKAMTDMDSTNLYAYISIIALLFCIPPAVLFEGP
QLLKHGFNDAIAKVGMIKFISDLFWVGMFYHLYNQIATNTLERVAPLTHAVGNVLKRVFV
IGFSIIVFGNKISTQTAIGTSIAIAGVAIYSLIKARIEEEKRRMKSA
>P49158 | ACCD_SOYBN
MEKWWFNSMLFNRKLEYRCELSKSMDSLGPIENTSLREDPKILTDIEKKIHRDLDYLEME
GFFSSDLNTVSKNDDDHYMYETQFSFNNNITSFIDSCIESFNLGDIDKYNDIYFYSYIFL
KGRNCSESDNSSTSIITSTNDTNDSDSTIGESSNNLDESQKYKHLWLECENCYGLNYKKF
FKSKMNICEYCGYHLKMGSSDRIELSIDSGTWNPMDEDMVSLDPIEFHSEEEPYKDRIDS
YQRKTGLTEAVQTGTGQLNGIPVAIGIMDFQFMGGSMGSAVGEKITRLVEYATNQLLPLI
LVCASGGARMQEGSLSLIQMAKISSALYDYQKNKKLFYVSILTSPTTGGVTASFGMLGDI
IIAEPNAYIAFAGKRVIEQTLNKAVPEGSQAAEYLFHKGLFDSIVPRNLLKGVLSELFQF
HNFFSLTKNDKA
>P49169 | RR18_PEA
MDKSKRLFVKSKQSIRRSSPLIQSGDRIDYKNLSLLFKFISRQGKILSRRVNKLTLKQQR

LITIAIKQARILSLLPFVNSSSFVNNAKKKYEKRKSITRTRTPVLKKKKK
>P49170 | RR18_SECCE
MYTSKQPFLKSKQPFSKSEQPFSKSEQPFRKSKQTFRKFKQPFRKSKQPFRRRPRIGPGD
RIDYRNMSLINRFISEQGKILSRRINRLTLKQQRLITLAIKQARILSFLPFRNYENEKQF
QAQSISIITGSRPRKNRHIPQLTQKYNSNRNLRNNNQNLRNNNRNLSSDC
>P49244 | FABH1_CUPWR
MANASGFLGSSVPALRRATQPQHSISSSRGSSSDFVFKRVFCCSAVQGSDRQSLGDSRSP
RLVSRGCKLIGSGSAIPSLQISNDDLAKIVDTNDEWISVRTGIRNRRVLTGKDSLTNLAS
EAARKALEMAQIDADDVDMVLMCTSTPEDLFGSAPQISKALGCKKNPLSYDITAACSGFV
LGLVSAACHIRGGGFNNVLVIGADSLSRYVDWTDRGTCILFGDAAGAVVVQSCDAEEDGL
FAFDLHSDGDGQRHLKAAIKEDEVDKALGSNGSIRDFPPRRSSYSCIQMNGKEVFRFACR
CVPQSIESALGKAGLNGSNIDWLLLHQANQRIIDAVATRLEVPQERIISNLANYGNTSAA
SIPLALDEAVRSGNVKPGHVIATAGFGAGLTWGSAIIRWG
>P49245 | FABH2_CUPWR
MANAYGFVGHSVPTMKRAAQFQQMGSGFCSADSISKRVFCCSVVQGADKPASGDSRTEYR
TPRLVSRGCKLVGSGSAMPALQVSNDDLSKIVDTNDEWISVRTGIRNRRVLTGKESLTNL
ATVAARKALEMAQVDANDVDMVLMCTSTPEDLFGSAPQIQKALGCKKNPLAYDITAACSG
FVLGLVSAACHIRGGGFNNILVIGADSLSRYVDWTDRGTCILFGDAAGAVLVQSCDAEED
GLFAFDLHSDGDGQRHLKAAITENGIDHAVGSNGSVSDFPPRSSSYSCIQMNGKEVFRFA
CRCVPQSIESALGKAGLNGSNIDWLLLHQANQRIIDAVATRLEVPQERVISNLANYGNTS
AASIPLALDEAVRGGKVKAGHLIATAGFGAGLTWGSAIVRWG
>P50362 | G3PA_CHLRE
MAAMMQKSAFTGSAVSSKSGVRAKAARAVVDVRAEKKIRVAINGFGRIGRNFLRCWHGRQ
NTLLDVVAINDSGGVKQASHLLKYDSTLGTFAADVKIVDDSHISVDGKQIKIVSSRDPLQ
LPWKEMNIDLVIEGTGVFIDKVGAGKHIQAGASKVLITAPAKDKDIPTFVVGVNEGDYKH
EYPIISNASCTTNCLAPFVKVLEQKFGIVKGTMTTTHSYTGDQRLLDASHRDLRRARAAA
LNIVPTTTGAAKAVSLVLPSLKGKLNGIALRVPTPTVSVVDLVVQVEKKTFAEEVNAAFR
EAANGPMKGVLHVEDAPLVSIDFKCTDQSTSIDASLTMVMGDDMVKVVAWYDNEWGYSQR
VVDLAEVTAKKWVA
>P50371 | EFTU_CHACO
MAQEVFQRTKPHVNIGTIGHVDHGKTTLTAAITMTLAVNSTCTPKKYDEIDAAPEERARG
ITINTAHVEYETALRHYAHVDCPGHADYIKNMITGAAQMDGAILVVSAADGPMPQTKEHI
LLAKQVGVPSIVVFLNKEDQVDDEEILQLVDLEVRESLINYEFPGDKVPVVSGSALMALQ
ALTEKPNTSRGENKWVDKIYELMDAVDSYIPTPKRDIEKPFLMPIEDVFSIQGRGTVATG
RIERGILKLGDIVELIGLNEKIRSTVVTGLEMFRRLLEQGFAGENIGVLLRGIEKKDIER
GMVIAQPGTIEPHTRFEAQVYILRKEEGGRHSPFFAGYRPQFFVRTADVTGVIEAFEYDN
GDKTRMVMPGDRVKMIVNLICPIAIEKKMRFAIREGGRTIGAGVVYKY
>P52422 | PUR3_ARATH
MESRVLFSSQFNFPVNSPFKTRETSIAPLTPSRNVLSFSFRSPAERCAMRIVPLVKAASS
TPQIVAEVDGSSHEPRRKKLAVFVSGGGSNFRKIHEGCSDGSVNGDVVLLVTNKKDCGGA
EYARSNGIPVLVFPKAKREPSDGLSPSELVDVLRKYGVDFVLLAGYLKLIPVELVQAFPK
RILNIHPALLPAFGGKGLYGIKVHKAVLESGARYSGPTIHFVNEEYDTGRILAQSAVRVI
ANDTPEELAKRVLHEEHKLYVEVVGAICEERIKWREDGVPLIQNKQNPDEYY
>P52733 | RPOC1_PINTH

MIDQNKHQQLRIGLASPEQICAWSEKILPNGEIVGQVTKPHTLHYETNKPERDGSFCERI
FGPIKSGVCSCGNSPGIGNEKIDAKFCTQCGVEFVDSRIRRYQMGYIKLACPVVHVWYLK
RLPSYIANLLAKTRKELEGPVYCDLFLARPIANKPTLLRSRGTFDYEIQSWREIIPHYLS
ARPYYLFPRGSGTFKEREIATGGDAIGKQLMGLDLQMIIDRSHMEWKNLVELKWNRLEEN
QESTVDRWEDEKIRRRKDFLVGRMKLAKHFLRTNIEPKWMVLCLLPVLPPEPRPIVQLGE
GGLITSSDLNELYRRVINRNNTLTNLLARSGSESFVIYQTKLIQEAVDALLDNGICRQPM
RDSHNRPYKSFSDVIEGKEGRFRENLLGKRVDYSGRSVIVVGPFLSLYQCGLPSEIAIEL
FQAFLIRSLIGRHIAPNLRTAKSMIRDKGPIVWEVLQEVMQGHPILLNRAPTLHKLGIQA
FQPILVEGRAIRLHPSVCGGFNADFDGDQMAVHVPLSLEARAEARLLMFSETNLLSPAIG
DPISIPTQDMLLGLYISTVQNSQGIYGNRYHPYHSENKSFSCKKPSFYSYDDVLRAYRQK
RIDLYSPLWLRWGEVDLRIITSVNQEAPIEVQYESLGTFHEIHEHYRIRKGRMGEILNIY
IRTTVGRTRFNREMEEAIQGFACSEHPNKSLPALRI
>P52762 | RR12_PINTH
MPTIQQLIRNARQPIENRKKSPALRGCPQRRGVCARVYTITPKKPNSALRKVARVRLTSG
FEITAYIPGIDHNLQEHSVVLVRGGRVKDLPGVRYHIVRGTLDAAEVKDRQQGRSKYGVK
KQK
>P52769 | ACCD_PINTH
MSIKEWFEDKRKITALLKNSVERDSKDANETEKNKNKSIDYAKIKKLWAQCDNCENLLYL
RFLRENQSVCKECGYYLQMNSSDRIELPIDRDTWRPMDEDMYTLDVLQFYSENEPSHSDN
LNSEDESYKDHITFYQIETGLTDAIQTGIGQLNGLTIALGVMDFQFMGGSMGSVVGEKIT
RLIERATAESLPLIMVCASGGARMQEGSFSLMQMAKIASALYIHQKEKKLLYISILTSPT
TGGVTASFGMLGDIIIAEPKAYIAFAGKRVIEQTLGQKVIEDFQVTEHLFGHGLFDLIVP
RNLLKGVLSELFWFYVLRSSL
>P52771 | RK22_PINTH
MESMGNNSPGPEIRALARNIRMSAHKARRVINQIRGRSYGQALMILELMPYGACYPISQL
IHSAAANANHNMGLNKANLLVGRVEVNEGAVFKRIQPRAQGRGYPIQKPTCHITIVSEEI
SRSNDPIMSIESRKKGYVWRRK
>P52772 | RK23_PINTH
MDEVKYPVLTEKSIRLLERNQYTFNVDSQSNKTKIKNWIENFFDVKVIAMNSYRLPEKGG
KRVSMIGHPIRCKRVIITLRTGDSIPLFSEQ
>P55229 | GLGL1_ARATH
MVVSADCRISLSAPSCIRSSSTGLTRHIKLGSFCNGELMGKKLNLSQLPNIRLRSSTNFS
QKRILMSLNSVAGESKVQELETEKRDPRTVASIILGGGAGTRLFPLTKRRAKPAVPIGGA
YRLIDVPMSNCINSGINKVYILTQYNSASLNRHLARAYNSNGLGFGDGYVEVLAATQTPG
ESGKRWFQGTADAVRQFHWLFEDARSKDIEDVLILSGDHLYRMDYMDFIQDHRQSGADIS
ISCIPIDDRRASDFGLMKIDDKGRVISFSEKPKGDDLKAMAVDTTILGLSKEEAEKKPYI
ASMGVYVFKKEILLNLLRWRFPTANDFGSEIIPFSAKEFYVNAYLFNDYWEDIGTIRSFF
EANLALTEHPGAFSFYDAAKPIYTSRRNLPPSKIDNSKLIDSIISHGSFLTNCLIEHSIV
GIRSRVGSNVQLKDTVMLGADYYETEAEVAALLAEGNVPIGIGENTKIQECIIDKNARVG
KNVIIANSEGIQEADRSSDGFYIRSGITVILKNSVIKDGVVI
>P55231 | GLGL3_ARATH
MDSCCNFSLGTKTVLAKDSFKNVENKFLGEKIKGSVLKPFSSDLSSKKFRNRKLRPGVAY
AIATSKNAKEALKNQPSMFERRRADPKNVAAIILGGGDGAKLFPLTKRAATPAVPVGGCY
RMIDIPMSNCINSCINKIFVLTQFNSASLNRHLARTYFGNGINFGDGFVEVLAATQTPGE

AGKKWFQGTADAVRKFLWVFEDAKNRNIENIIILSGDHLYRMNYMDFVQHHVDSKADITL
SCAPVDESRASEYGLVNIDRSGRVVHFSEKPTGIDLKSMQTDTTMHGLSHQEAAKSPYIA
SMGVYCFKTEALLKLLTWRYPSSNDFGSEIIPAAIKDHNVQGYIYRDYWEDIGTIKSFYE
ANIALVEEHPKFEFYDQNTPFYTSPRFLPPTKTEKCRIVNSVISHGCFLGECSIQRSIIG
ERSRLDYGVELQDTLMLGADSYQTESEIASLLAEGNVPIGIGRDTKIRKCIIDKNAKIGK
NVVIMNKDDVKEADRPEEGFYIRSGITVVVEKATIKDGTVI
>P55238 | GLGS_HORVU
MAMAAAASPSKILIPPHRASAVTAAASTSCDSLRLLCAPRGRPGPRGLVARPVPRRPFFF
SPRAVSDSKSSQTCLDPDASTSVLGIILGGGAGTRLYPLTKKRAKPAVPLGANYRLIDIP
VSNCLNSNISKIYVLTQFNSASLNRHLSRAYGSNIGGYKNEGFVEVLAAQQSPDNPDWFQ
GTADAVRQYLWLFEEHNVMEYLILAGDHLYRMDYEKFIQAHRETDADITVAALPMDEERA
TAFGLMKIDEEGRIIEFAEKPKGEQLKAMMVDTTILGLEDARAKEMPYIASMGIYVISKH
VMLQLLREQFPGANDFGSEVIPGATSTGMRVQAYLYDGYWEDIGTIEAFYNANLGITKKP
IPDFSFYDRSAPIYTQPRHLPPSKVLDADVTDSVIGEGCVIKNCKIHHSVVGLRSCISEG
AIIEDTLLMGADYYETEADKKLLAEKGGIPIGIGKNSHIKRAIIDKNARIGDNVMIINVD
NVQEAARETDGYFIKSGIVTVIKDALLPSGTVI
>P55243 | GLGL3_SOLTU
MGKKLKYTKFQLRSNVVKPNICMSLTTDIAGEAKLKDLERQKKGDARTVVAIILGGGAGT
RLFPLTKRRAKPAVPMGGAYRLIDVPMSNCINSGINKVYILTQFNSASLNRHIARAYNFG
NGVTFESGYVEVLAATQTPGELGKRWFQGTAHAVRQFHWLFEDARSKDIEDVLILSGDHL
YRMDYLHFVQSHRQSGADITISSLPIDDSRASDFGLMKIDDTGRVMSFSEKPKGDDLKAM
AVDTTVLGLSPEEAKEKPYIASIGKVYVFKKDILLNLLRWRFPTANDFGSEIIPASTKEF
CVKAYLFNDYWEDIGTIRSFFRANLALTEHPPRFSFYDATKPIYTSRRNLPPSAIDNSKI
VDSIVSHGIFLTNCFVEHSVVGIRSRIGTNVHLKDTVMLGADYYETDAEIRSQLAEGKVP
LGIGENTRIKDCIIDKNARIGKNVVIANSEGVQEADRSSEGFYMASGITVISKNSTIPDG
TVI
>P56293 | ACCD_CHLVU
MSILSWIENQRKLKLLNAPKYNHPESDVSQGLWTRCDHCGVILYIKHLKENQRVCFGCGY
HLQMSSTERIESLVDANTWRPFDEMVSPCDPLEFRDQKAYTERLKDAQERTGLQDAVQTG
TGLLDGIPIALGVMDFHFMGGSMGSVVGEKITRLIEYATQEGLPVILVCASGGARMQEGI
LSLMQMAKISAALHIHQNCAKLLYISVLTSPTTGGVTASFAMLGDLLFAEPKALIGFAGR
RVIEQTLQEQLPDDFQTAEYLLHHGLLDLIVPRSFLKQALSETLTLYKEAPLKEQGRIPY
GERGPLTKTREEQLRRFLKSSKTPEYLHIVNDLKELLGFLGQTQTTLYPEKLEFLNNLKT
QEQFLQKNDNFFEELLTSTTVKKALNLACGTQTRLNWLNYKLTEFRIRPKF
>P56303 | CHLN_CHLVU
MTNSKLTETLTFECETGNYHTFCPISCVAWLYQKIEDSFFLVIGTKTCGYFLQNALGVMI
FAEPRYAMAELEEADISAQLNDYKELKRLCLQIKQDRNPSVIVWIGTCTTEIIKMDLEGM
APRLEAEIQTPIVVARANGLDYAFTQGEDTVLAAMVQRCPSNAPEQNQIEKKSLVLFGSL
PTNVATQLNLELERCGIQVAGWLPSQRYADLPVLNQNVYVCGINPFLSRTATTLMRRRKC
KLISAPFPIGPDGTRAWLEKICSVFNVAPINLIERERLIWDSLEDYITLLRGKSVFFMGD
NLLEISLARFLVRCGMIVYEIGIPYLDKRFQSAELQLLEKTCSEMNVAMPRIVEKPDNYN
QIQRIRELQPDLAITGMAHANPLEARGINTKWSVEFTFAQIHGFTNARDILELVTRPLRR
NKALENLGWNQLVKM
>P56315 | CCSA_CHLVU

MMVNIPVIENFLSNSCFLILFLTTVYYWLKIGFGGKSSFSFTGLTGYGSALCCLTLQLIL
RWVDSGHFPLSNLYESLIFLAWCLLLLYIYIEVSTKTLFLGVLTSPAILCLVAFTDFSLP
TELQQSTPLVPALQSNWLVMHVTVMIASYAALLLGCFIAIAYLVLSKFFIKTTFVTAPLS
SQNTVLRQESFLEEKQKTDIVFEKNDKNLKFLFFLDNLSYRTIGIGFCFLTLGILSGAIW
ANETWGNYWSWDPKETWAFITWLTFACYLHSRLVGGWTGSKPALVASFGFLVVWVCYLGV
NLLGQGLHSYGFLNV
>P56317 | CLPP_CHLVU
MPIGVPKVPFRLPGEPSAQWVDLYNRLYRERVLFLCQELDDELANQLIGIMLYLNAEEQN
KGLYIYINSPGGSVTCGIAVYDAMNYIKSEVTTICVGTAASMASFILAGGDRGKRIALPH
SRIMVHQPEGGSQGQASEVLSESQEVMRIRRQVGRIYSERTGQTLSRVSRDMDRDQFLSA
REAKEYGLVDQVAVDTKWSTN
>P56351 | RR2_CHLVU
MLQKVKLEDMIQSGMHFGHSTREWNPRMAPYIYGERNGRHILDLVQTYYLLNKVLAFLEE
QAAQGKTFLFVGTKQQAAPLIAKTALACESFYVNQRWLGGMLTNWRTIQKSLRKLQEYRR
AEERGDWNLLKKQEVARKRREKDRLEKYLSGVENMSRLPGVVILIGQTEEIHAVKECRQL
GIPTVTLLDSNCDPNLADWFLPANDDSVSALRLILAWFQQAIQTGQLRSLEREAAKKQKI
KKTGVKISGNRRTSSITKKRNPASSKI
>P56353 | RR18_CHLVU
MAGIQQKRKTIKSFKTRRKVVPVLIPKKGQAVISNTGEPASRYIIDYKNTQLLVKFISPQ
GKILSRRATGLTAKQQRIMANAIKRARMGGLVPFVNYELGSKK
>P56355 | RR7_CHLVU
MSRRRTQKKRIVMPDPVYDSRLVELLVRQLMREGKKSLAYRICYESMNRVADATQQDPLV
IVEQAIRNATPLVEVKARRIGGSTYQVPLEVASERGTALAIRWILSVCRKKTGRPMAAKL
TAELLDAAKNSGLAVRKRDEIHKMADANKAFAKYRF
>P56357 | RK32_CHLVU
MAVPKKRKSKMKTRLRKAQWKSEASREAAKALSKAKTVIKSLLAANSANLESNSENSN
>P56358 | RR9_CHLVU
MIKITKTISQSTQSGGRKTAKARVQLLPGTGTKVMVNGKQASDYFQNNAVYLQNMFTPRD
LVKTETKYDVHILVEGGGLSAQAQAVKLALSKAFVDFFPEYRKVFKKPGLLTRDARIKER
RKYGLKKARKAPQFSKR
>P56359 | RR11_CHLVU
MAKKIEKSAKKKFREKVLLGVAHIQSTFNNTIVTITTNKGNVLAWSSAGACGFKGARKKT
PLAAKQAAENAAQTCVSQGMREIRVNVKGAGAGREAALRGLRDAGLNITIIRDITPIPHN
GCRPPKKRRI
>P56361 | RR8_CHLVU
MVNDTISDMLTRIRNANLAKKTSVSLPKTKVHEKMCQILEQEGFIKTFSFSETNTNELIV
DLKYQDFASFGNYGVGKPCITNLKRISKPGLRIYTNSREIPKVLGGMGILILSTSKGLMT
DRQARKLCLGGEILCSVW
>P56363 | RK14_CHLVU
MIQPQTYLRVADNTGARELMCIRVLGGGKQRAATVGDIIIAVVKDATPNMPVKRSDIVRA
VIVRTRKNVRRVNGTSIRFDENAAVIINKENNPRGTRVFGPVARELRDGNFTKIISLAPE
VL
>P56367 | RK2_CHLVU
MAIRFFKAATPGTRHGSVLDFSEITHKKPEKALTSWWSRSKGRNNRGIITSRHRGGGHKR

LYREIDFARAKVNVPAKVAYIEYDPNRNARIALVNYQDGEKKYILHPVGLQVGQTIIASP
EASIAIGNCLPLVKIPLGTEVHNIELQPGSGGQLVRAAGTVAQIVAKEGTWASLRLPSGE
VRLVSQNCWATIGRVGNIDAFNLTLGKAGRSRWLGRRPHVRGSAMNPVDHPHGGGEGRAP
IGRARPVSPWGRPALGAKTRKRKKFSAALILQRRK
>P56751 | NU3C_ARATH
MFLLYEYDIFWAFLLISSAIPVLAFLISGVLSPIRKGPEKLSSYESGIEPIGDAWLQFRI
RYYMFALVFVVFDVETVFLYPWAMSFDVLGVSAFIEAFIFVLILILGLVYAWRKGALEWS

>P56753 | NDHH_ARATH
MKRPVTGKDLMIVNMGPHHPSMHGVLRLIVTLDGEDVVDCEPILGYLHRGMEKIAENRAI
IQYLPYVTRWDYLATMFTEAITVNGPEQLGNIQVPKRASYIRVIMLELSRIASHLLWLGP
FMADIGAQTPFFYIFREREFVYDLFEAATGMRMMHNFFRIGGIAADLPYGWIDKCLDFCD
YFLTEVVEYQKLITRNPIFLERVEGVGIIGGEEAINWGLSGPMLRASGIPWDLRKIDRYE
SYDEFEWEIQWQKQGDSLARYLVRLSEMTESIKIIQQALEGLPGGPYENLESRGFDRKRN
PEWNDFEYRFISKKPSPTFELSKQELYVRVEAPKGELGIFLIGDQSGFPWRWKIRPPGFI
NLQILPELVKRMKLADIMTILGSIDIIMGEVDR
>P56782 | PSBK_ARATH
MLNIFNLICIFFNSTLFSSTFLVAKLPEAYAFLNPIVDVMPVIPLFFLLLAFVWQAAVSF
R
>P56784 | MATK_ARATH
MDKFQGYLEFDGARQQSFLYPLFFREYIYVLAYDHGLNRLNRNRYIFLENADYDKKYSSL
ITKRLILRMYEQNRLIIPTKDVNQNSFLGHTSLFYYQMISVLFAVIVEIPFSLRLGSSFQ
GKQLKKSYNLQSIHSIFPFLEDKLGHFNYVLDVLIPYPIHLEILVQTLRYRVKDASSLHF
FRFCLYEYCNWKNFYIKKKSILNPRFFLFLYNSHVCEYESIFFFLRKRSSHLRSTSYEVL
FERIVFYGKIHHFFKVFVNNFPAILGLLKDPFIHYVRYHGRCILATKDTPLLMNKWKYYF
VNLWQCYFSVWFQSQKVNINQLSKDNLEFLGYLSSLRLNPLVVRSQMLENSFLIDNVRIK
LDSKIPISSIIGSLAKDKFCNVLGHPISKATWTDSSDSDILNRFVRICRNISHYYSGSSK
KKNLYRIKYILRLCCVKTLARKHKSTVRTFLKRLGSGLLEEFLTGEDQVLSLIFPRSYYA
SKRLYRVRIWYLDILYLNDLVNHE
>P56791 | RK2_ARATH
MAIHLYKTSTPSTRNGAVDSQVKSNPRNNLICGQHHCGKGRNARGIITARHRGGGHKRLY
RKIDFRRNAKDIYGRIVTIEYDPNRNAYICLIHYGDGEKRYILHPRGAIIGDTIVSGTEV
PIKMGNALPLTDMPLGTAIHNIEITLGRGGQLARAAGAVAKLIAKEGKSATLKLPSGEVR
LISKNCSATVGQVGNVGVNQKSLGRAGSKCWLGKRPVVRGVVMNPVDHPHGGGEGRAPIG
RKKPVTPWGYPALGRRTRKRKKYSETLILRRRSK
>P56792 | RK14_ARATH
MIQPQTYLNVADNSGARELMCIRIIGASNRRYAHIGDVIVAVIKEAIPNTPLERSEVIRA
VIVRTCKELKRNNGTIIRYDDNAAVVIDQEGNPKGTRVFGAIPRELRQLNFTKIVSLAPE
VL
>P56794 | RK20_ARATH
MTRIKRGYIARRRRTKLRLFASSFRGAHSRLTRTMTQQRIRALVSAHRDRGKRKRDFRRL
WITRINAVIHEMGVFYSYNEFIHNLYKKQLLLNRKILAQIALLNRSCLYTISNDIKK
>P56797 | RR2_ARATH
MTKRYWNIDLEEMMRAGVHFGHGTRKWNPRMAPYISAKRKGIHIINLTRTARFLSEACDL

VFDAASRGKQFLIVGTKNKAADLVSRAAIRARCHYVNKKWLGGMLTNWSTTEKRLHKFRD
LRTEQKTEGFNRLPKRDAAVLKRQLSRLETYLGGIKYMTGLPDIVIILDQQEEYTALREC
ITLGIPTISLIDTNCNPDLADISIPANDDAIASIRFILNKLVFAICEGRSSYIQNS
>P56799 | RR4_ARATH
MSRYRGPRFKKIRRLGALPGLTSKRPKAGSDLRNQSRSVKKSQYRIRLEEKQKLRFHYGL
TEHQLLKYVRIAGKAKGSTGQVLLQLLEMRLDNILFRLGMALTIPQARQLVNHGHILVNG
RIVDIPSYRCKPRDIITVKDEQNSRTLVQNLLDSSAPEELPNHLTLHTFQYEGLVNQIID
RKCVGLKINELLVVEYYSRQT
>P56801 | RR8_ARATH
MGKDTIADIITSIRNADMNRKGTVRIGSTNITESIVKILLREGFIENVRKHRENNQYFLI
LTLRHRRNKKESYKTILNLKRISRPGLRIYSNSQRIPRILGGIGIVILSTSQGIMTDREA
RLKRIGGEILCYIW
>P58271 | MATK_WHEAT
MEKFEGYSEKHKSRQQYFVYPLLFQEYIYAFAPDYGLNGSEPVEIVGCNNKKFSSLLVKR
LIIRMYQQNFLDNSVNHPNQDRLLDYKNYFYSEFYSQILSEGFAIVVEIPFSLRELFCPK
EKEIPKFQNLRSIHSIFPFFEDKFLHLDYLSHIEIPYPIHLEILVQLLQYRIQDVPSLHL
LRFLLNYYSNWNSFITSMKSIFIFKKENKRLFRFLYNSYVSEYEFFLLFLRKQSSCLPLA
SSGTFLERIHFSRKMEHFGIMYPGFSRKTLWFFMDPLMHYVRYQGKAILASKGTFLLKKK
WKCYLINLWQYYFCFWTQPRRIHINQLANSCFDFMGYLSSVPKSSLLVRNQMLENSFLID
TRMKKFDTIVHATLLIGYLSKAQFCTGSGHPISKPIWTDLSDWDILDRFGRICRNLFHYH
SGSSKKKTLYRLKYILRLSCARTLGPKHKSTVRAFMQWLGSVFLEEFFREEEQVFSLMFA
KTTYFSFRGSHSERIWYLDILRINDLVNPLN
>P58419 | NU4C_OENHO
MNSFPWLTIIVVFPILTGSLIFLLPHRGNKVMKWYTLCICILELLLTTYTFCYHFQLDDP
LTQLTENYKWIHFFDFYWRLGIDGLSIGPILLTGFITTLATLAAWPVTRDAQLFHFLMLA
MYSGQIGSFSSRDLLLFFLMWEFELIPVYLLLSMWGGKKRLYSATKFILYTAGGSIFLLI
GVLGIGLYGSNEPTLNFETLANQSYPVALEVIFYVGFLIAFAVKLPIIPFHTWLPDTHGE
AHYSTCMLLAGILLKMGAYGLVRINMELLPHAHCLFSPGLIIVGAIQIIYAASTSPGQLN
LKKRIAYSSISHMGFIIIGIGSLSDTGLNGAILQIISHGFIGAALFFLAGTSYDRIRLLY
LDEMGGMAIPLPKLFTMLSILSMASLALPGLSGFVAELLVFFGIITSQKYLLMPKILIAF
LMAIGMILTPIYSLSMLRQMFYGYKLFNVPNYYFFDSGPRELFVSISLLLPIIGIGIYPD
FVLSLSVEKVEAIISHFFFSIVFKKKE
>P58420 | NU4C_WHEAT
MSYFPWLTILVVLPIFAGSLIFFLPHKGNKVVRWYTISICLLEFLLMTYAFCYHFQLEDP
LIQLKEDYKWIDVFDFHWRLGIDGLSLGSILLTGFITTLATLAAWPITRNSRLFYFLMLA
MYSGQIGLFSSRDLLLFFIMWELELIPVYLLLSMWGGKRRLYSATKFILYTAGGSIFFLI
GVLGMGLYGSNEPGLDLERLINQSYPATLEILLYFGFLIAYAVKLPIIPLHTWLPDTHGE
AHYSTCMLLAGILLKMGAYGLIRINMELLPHAHYLFSPWLVIIGAIQIIYAASTSLGQRN
FKKRIAYSSVSHMGFIIIGIGSITNIGLNGAILQILSHGFIGATLFFLAGTASDRMRLVY
LEELGGISIPMPKIFTMFSSFSMASLALPGMSGFVAELVVFFGLITSPKFLLMPKTLITF
VMAIGMILTPIYLLSMLRQMFYGYKLFNVPNANFVDSGPRELFILICIFLPVIGIGIYPD
FVLSLSVDRVEALLSNYYPK
>P59032 | RR8_CHAGL
MTSDVIANMITSIRNANLRKIKVIELPATKITKSIGKILLQEGFIDNLREREENNKNFLI

LTLRSQRRKHKASITTIRRISKPGLRIYSNHQEIPKVLGGIGIVILSTSQGIMSDRQARQ
NRIGGEILCYIW
>P59136 | RR4_CANTA
MSRYRGPRVKIIRRLGALPGLTNKTPQLKSSPINQSTSNKKISQYRIRLEEKQKLRFHYG
ITERQLLNYVRIARKAKGSTGEILLQLLEMRLDNVIFRLGMSPTIPGARQLVNHRHILVN
GHIVDIPSYRCKPQDFITIKNQRKSQAIINKNMNFYRKYKIPNHLIYNSLDKKGLVNQIL
DRESIGLKINELLVVEYYSRQA
>P59142 | RR4_HYPCP
MSRYRGPRVRIIRRLGTLPGLTNKTPQLKSSSINQSISNKKISQYRIRLEEKQKLRFHYG
ITERQLLNYVRIARKAKGSTGEVLLQLLEMRLDNVTFRLGMAPTIPGARQLVNHRHILVN
DCIVDIPSYRCKPQDFITIKNQRKSETIISKNIEFYQKSKIPNHLTYSSLEKKGLVNQIL
DRESIGLKINELLVVEYYSRQA
>P59145 | RR4_HYPLA
MSRYRGPRVRIIRRLGALPGLTNKTPQLKSSSINQSTSNKKISQYRIRLEEKQKLRFHYG
ITERQLLNYVRIARKAKGSTGEILLQLLEMRLDNVIFRLGMTPTIPGARQLVNHRHILVN
GYIVDIPSYRCKPQDFITIKNQRKSEAIISKNIEFYQKYKIPNHLTYSSLEKKGLVNQIL
NRKSIGLKINELLVVEYYSRQA
>P59146 | RR4_LEUSC
MSRYRGPRVRIIRRLGTLPGLTNKIPQLKSSSINQSTSNKKISQYRIRLEEKQKLRFHYG
ITERQLLNYVRIARKAKGSTGEVLLQLLEMRLDNIIFRLGMAPTIPGARQLVNHRHILVN
DCIVNIPSYRCKPQDFITIKNQRKSEAIISKNIEFYQNSKIPNHLTYSSLEKKGLVNQIL
DRESIGLKINELLVVEYYSRQA
>P59152 | RR4_WOOUN
MGRGKTPNLGEFRVATDQSASRKISQFCVRLEAKQRLRFNYGLTERQLLKYVRIARKTRG
STGQVPPQLLEMRLDNVIFRLGMASTIPAARQLVNHRHILVNNRIVDVPSYRCKPKDIIT
VRNRPTSCNALKGESPGGGETPDHLTASLSEGSRPTGLVNRIANRESVNLNINELLVVEY
YSRKA
>P60282 | RPOB_AMBTC
MLRDGNEGMSTIPGFSQIQFEGFCRFVDQGLAEELHKFPKIEDTDQEIEFQLLVETYQLA
EPLIKERDAVYESLTHSSELYVPAGLIWKVGRDMQEQTVFIGNIPLMNSLGTFIVNGIYR
IVINQILQSPGIYYSSELDHNGISVYTGTIISDRGGRSELEIDRKARIWARVSRKQKISI
LVLPSAMGSNLREILDNVCYPEILLYFPNEKEKKKIGSKENAILEFYQQFSCVGGDPVFS
ESLCKELQKRFFQQRCELGRIGRQNMNQRLNIDIPQNNTFLLPRDVLAATDHLIGMKFGM
GTLDDMNHLKNKRIRSVADLLQDQFGLALVRLENVVRGTICGAIRHKFIPTPQNLVTSTP
LTTTYESFFGLHPLSQVLDRTNPLTQIVHGRKSSYLGPGGLTGRTASFRIRDIHPSHYGR
ICPIDTSEGINVGLIGSLAIHARIGDWGSIRSPFYEISERSKEEQMVYLSPRRDEYYMVM
VAAGNSLALNQDIQDEQVVPARYRQEFVTIAWEHIDLRSIYPLQYFSIGASLIPFIEHND
ANRALMSSNMQRQAVPLSRSEKCIVGTGLERQAALDSGGSAIAQHEGKVIYTDTEKILLS
GNGDTISIPLLMYQRSNKNTCMHQKPQVHRDKYVKKGQVLADGAATVGGELALGKNVLVA
HMPWEGYNFEDAVLISERLVYGDIYTSFHIRKYEIQTHVTSHGPEKITNEIPHLEAHLLR
NLDRNGIVMLGSWVETGDVLVGKLTPQTAKESSYAPEDRLLRVILGIQVSTAKETCLKLP
IGGRGRVIDVRWIQKKGASSYNPEKIRVYISQKREIKVGDKVAGRHGNKGIISKILPRQD
MPYLQDGTPVDMVFNPLGVPSRMNVGQIFECSLGLAGDLLDRHYRITPFDERYEQEASRK
LVFPELYEASKRTANPWVFEPEYPGKSRIFDGRTGDPFEQPVIIGKSYMLKLIHQVDDKI

HGRSSGHYALVTQQPLRGRAKQGGQRVGEMEVWALEGFGVAHILQEMLTYKSDHIRARQE
LLGTTIVGGTIPKPEGAPESFRLLVRELRSLALELKHFLVSEKNFQINRKEA
>P60290 | RPOC2_PHYPA
MLFYNKVMDRTAIKQLISRLITHFGITYTTYILDQLKTVGFKQATQAAISLGIDDLLTAP
SKSWLIQDAEQQGYISEKHYRYGNVHAVEKLRQLIETWYATSEYLKQEMNPNFRMTDPLN
PVHMMSFSGARGSTSQVHQLVGMRGLMSDPQGQIIDLPIQSNFREGLSLTEYIISCYGAR
KGVVDTAVRTSDAGYLTRRLVEVVQHIVVRKVDCGTSENIFVTPLQNNYKKNNKLIGRIL
ADNIYINGRCIAIRNQDITTNLVISLINFQRKGIFIRSPLICKSMLWICQLCYGWSLTHG
NLIELGEAVGIIAGQSIGEPGTQLTLRTFHTGGVFTGDIAEHIRTPFNGIIQFDTNSVYP
TRTRHGHPAWICNNNLSVVIKSKKKLHNLVIPTQSLLLVQSNQYVESKQVIAEVRAKTSP
FKEKVQKYIYSNLSGEMHWSSKVQHSSEYIHSNVHLLRKTGHIWILAGNFDKDNKFSFIF
YQNQDKLDNKLPIAKQTLNYFQLKEHFLNNFWNSIYSSIILYNYRFLEKKNNKYEKKLLF
QFMLKLPKNGILKQNDIFAIFNDPKYRIKNSGIIKYGNIKVDLINKKNDIFEDQKTKTVR
PRYKILKEGNFFLLPEEVYILDQSSFSSILVKNNSFIKAGTKITFNISSKITGFVKIKKK
FNNFKIKILPGSIYYPKEKQKNFKQNGILIPPGEKIFEQFRAKNWIYLEWIVLSKDNSFF
LIRPAIEYKIIFNDNPLTLPIPFYLDLLKEQKKIKIQTVKYILYEDSEEVEINPDTDIQL
IQTCLILNWETKVFIKEAHISFIKIRINKIIKNFFQINLIENINLMNKKKNNNIILNYLF
KKKRYIINQKDCEKILLLSKTWGIIRTPSNKNQEKSFFLILSPFNLFQTILFDKTKQNLK
IENNVEKLFTYEPKKIIKTFNIEKRKNFVEFLGLLGYLQNITKSFQLFSCKKFSDKSIPI
NFSIIDNLKKKIKISKWFFLNENKKVQKFFLTQNTILSLLNWSFPIFDLAKKKTQLFNLG
HFFCDGLSIAEYPTFSESGQIIAIYDDLSLVIRLAKPYLATGGAIIHNNYGEIVKEGDIL
ITLIYERLKSGDIIQGLPKVEQLLEARLTNPVSINLEKGFGEWNKDMTNFFGSLWGYFLS
AQISMEQSQVNLVNQIQKVYRSQGVNISDKHIEIIVRQMTSKVFTLEDGMTNGFLPGELI
EFARAKRMNRALEEVIPYKPVLLGITKASLNTQSFISEASFQETTRVLAKAALRGRIDWL
KGLKENVILGGIIPTGTGCEEVLWQITLEKQKNILLKKNKSKLFHNKVKDIFLYKKLSIS
FTSEKIHKNY
>P60577 | RR19_WHEAT
MTRKKTNPFVAHHLLAKIEKVNMKEEKETIVTWSRASSILPTMVGHTIAIHNGKEHIPIY
ITNPMVGRKLGEFVPTRHFTSYENARKDTKSRR
>P61841 | RR7_ARATH
MSRRGTAEEKTAKSDPIYRNRLVNMLVNRILKHGKKSLAYQIIYRALKKIQQKTETNPLS
VLRQAIRGVTPDIAVKARRVGGSTHQVPIEIGSTQGKALAIRWLLGASRKRPGRNMAFKL
SSELVDAAKGSGDAIRKKEETHRMAEANRAFAHFR
>P61845 | RK23_ARATH
MDGIKYAVFTDKSIRLLGKNQYTFNVESGSTRTEIKHWVELFFGVKVIAMNSHRLPGKVK
RMGPILGHTMHYRRMIITLQPGYSIPPLRKKRT
>P62127 | RR12_NICPL
MPTIKQLIRNTRQPIRNVTKSPALRGCPQRRGTCTRVYTITPKKPNSALRKVARVRLTSG
FEITAYIPGIGHNLQEHSVVLVRGGRVKDLPGVRYHIVRGTLDAVGVKDRQQGRSKYGVK
KPK
>P62128 | RR12_SPIOL
MPTIKQLIRNTRQPIRNVTKSPALRGCPQRRGTCTRVYTITPKKPNSALRKVARVRLTSG
FEITAYIPGIGHNLQEHSVVLVRGGRVKDLPGVRYHIVRGTLDAVGVKDRQQGRSKYGVK
KPK

>P62729 | RR7_ATRBE
MSRRGTAEKKTAKSDPIYRNRLVNMLVNRILKHGKKSLAYQIIYRAVKKIQQKTETNPLS
VLRQAIRGVTPDITVKARRVGGSTHQVPIEIGSTQGKALAIRWLLAASRKRPGRNMAFKL
SSELVDAAKGSGDAIRKKEETHRMAEANRAFAHFR
>P62731 | RR7_SOLNI
MSRRGTAEKKTAKSDPIYRNRLVNMLVNRILKHGKKSLAYQIIYRAVKKIQQKTETNPLS
VLRQAIRGVTPDITVKARRVGGSTHQVPIEIGSTQGKALAIRWLLAASRKRPGRNMAFKL
SSELVDAAKGSGDAIRKKEETHRMAEANRAFAHFR
>P68158 | EFTU_TOBAC
MASISAATATSSTKLVSSNSTNPLLPSSTKPSKLILSSSFTPNCSTLFLHSPATPSSTAT
HRHRRFTVRAARGKFERKKPHVNIGTIGHVDHGKTTLTAALTMALASMGNSAPKKYDEID
AAPEERARGITINTATVEYETENRHYAHVDCPGHADYVKNMITGAAQMDGAILVCSGADG
PMPQTKEHILLAKQVGVPNMVVFLNKQDQVDDEELLQLVELEVRELLSSYEFPGDDIPII
SGSALLALEALMANPSIKRGENQWVDKIYELMDAVDSYIPIPVRQTELPFLMAIEDVFSI
TGRGTVATGRVERGTVRIGDTVDIVGLKDTRSTTVTGVEMFQKILDEAMAGDNVGLLLRG
IQKIDIQRGMVLAKPGTITPHTKFEAIVYVLKKEEGGRHSPFFSGYRPQFYMRTTDVTGK
VTSITTDKGEESKMVMPGDRVNLVVELIMPVACEQGMRFAIREGGKTVGAGVIQKIIE
>P68751 | MATK_LILMI
MEEFQGYLKKDRSLQQHFLYPLLLQEYIYTLAHDDSLNGSIFYEPIEFIGYDNKFSLVLV
KRLITRMYQQNFLIYLVNDSNQNRFGGHTNYFYSHFFYSKMVSKGFSVIVEIPFSLRLVS
SSEEKEIPKSQNLGSIHSIFPFLEDKLSHLNNVSDILIPHPIHFEILVQILQCWIQDVPS
LHLLRFFLHKYQNLNKTIQSNKTIYVFSKENKRLFWFLYNSYVSECEFLLVFFHKQSCYL
RSTSSGAFLERSHFYGKMEHIIIVCCNNFQKTLWPVKDPLIHYVRYQGKAILASRGTHLL
MKKWRYYFVNFWQYYFHFWSQPYRMHINSLLNYSFYFMGYLLRVLINPYAVKNQMLENSF
LIDTVIKKFDTIIPIIPLIGSLSKAKFCTFSGHPISKPIWADFSDFDIIDRFGRICRNLS
HYHNGSSKKQSLYRIKYILRLSCARTLACKHKSTARALLQRLGSGLLEEFFTEEEQVLSF
IFPKTTLFTLHGSHRERIWSLDIIRINDLVNN
>P68752 | MATK_LILSU
MEEFQGYLKKDRSLQQHFLYPLLLQEYIYTLAHDDSLNGSIFYEPIEFIGYDNKFSLVLV
KRLITRMYQQNFLIYLVNDSNQNRFGGHTNYFYSHFFYSKMVSKGFSVIVEIPFSLRLVS
SSEEKEIPKSQNLGSIHSIFPFLEDKLSHLNNVSDILIPHPIHFEILVQILQCWIQDVPS
LHLLRFFLHKYQNLNKTIQSNKTIYVFSKENKRLFWFLYNSYVSECEFLLVFFHKQSCYL
RSTSSGAFLERSHFYGKMEHIIIVCCNNFQKTLWPVKDPLIHYVRYQGKAILASRGTHLL
MKKWRYYFVNFWQYYFHFWSQPYRMHINSLLNYSFYFMGYLLRVLINPYAVKNQMLENSF
LIDTVIKKFDTIIPIIPLIGSLSKAKFCTFSGHPISKPIWADFSDFDIIDRFGRICRNLS
HYHNGSSKKQSLYRIKYILRLSCARTLACKHKSTARALLQRLGSGLLEEFFTEEEQVLSF
IFPKTTLFTLHGSHRERIWSLDIIRINDLVNN
>P69041 | IF1C_ANTMA
MKEQKWIHEGLITESLPNGMFRVRLDNEDLILGYVSGKIRRSFIRILPGDKVKIEVSRYD
STRGRIIYRLRNKDSKD
>P69250 | RBS1_NICSY
MASSVLSSAAVATRSNVAQANMVAPFTGLKSAASFPVSRKQNLDITSIASNGGRVQCMQV
WPPINKKKYETLSYLPDLSQEQLLSEVEYLLKNGWVPCLEFETEHGFVYRENNKSPGYYD
GRYWTMWKLPMFGCTDATQVLAEVEEAKKAYPQAWIRIIGFDNVRQVQCISFIAYKPEGY

>P69378 | NU4LC_HORVU
MMFEHVLFLSVYLFSIGIYGLITSRNMVRALICLELILNSINLNLVTFSDLFDSRQLKGD
IFAIFVIALAAAEAAIGLSILSSIHRNRKSTRINQSNLLNN
>P69379 | NU4LC_WHEAT
MMFEHVLFLSVYLFSIGIYGLITSRNMVRALICLELILNSINLNLVTFSDLFDSRQLKGD
IFAIFVIALAAAEAAIGLSILSSIHRNRKSTRINQSNLLNN
>P69570 | RBL_LAROX
MSPKTETKASVGFKAGVKDYRLTYYTPEYQTKDTDILAAFRVTPQPGVPPEEAGAAVAAE
SSTGTWTTVWTDGLTSLDRYKGRCYDIEAVPGEESQFIAYVAYPLDLFEEGSVTNLFTSI
VGNVFGFKALRALRLEDLRIPPAYSKTFQGPPHGIQVERDKLNKYGRPLLGCTIKPKLGL
SAKNYGRAVYECLRGGLDFTKDDENVNSQPFMRWRDRFVFCAEALYKAQAETGEIKGHYL
NATAGTCEEMMKRAVFARELGVPIVMHDYLTGGFTANTSLAHYCRDNGLLLHIHRAMHAV
IDRQRNHGMHFRVLAKALRMSGGDHIHAGTVVGKLEGERDVTLGFVDLLRDDFIEKDRSR
GIYFTQDWVSMPGVLPVASGGIHVWHMPALTEIFGDDSVLQFGGGTLGHPWGNAPGAVAN
RVALEACVQARNEGRDLAREGNEVIREATKWSPELAAACEVWKEIKFEFDTIDYL
>P69663 | RR7_CERJA
MSRRGTAEEKTAKSDPIYRNRLVNMLVNRILKHGKKSLAYQIIYRAVKKIQQKTETNPLS
VLRQAIRGVTPDIAVKARRVGGSTHQVPIEIGSTQGKALAIRWLLGASRKRPGRNMAFKL
SSELVDAAKGSGDAIRKKEETHRMAEANRAFAHFR
>P69664 | RR7_DRIWI
MSRRGTAEEKTAKSDPIYRNRLVNMLVNRILKHGKKSLAYQIIYRAVKKIQQKTETNPLS
VLRQAIRGVTPDIAVKARRVGGSTHQVPIEIGSTQGKALAIRWLLGASRKRPGRNMAFKL
SSELVDAAKGSGDAIRKKEETHRMAEANRAFAHFR
>P69667 | RK23_WHEAT
MDGIKYAVFTEKSLRLLGKNQYTFNVESGFTKTEIKHWVELFFGVKVVAVNSHRLPGKGR
RMGPILGHTMHYRRMIITLQPGYSIPLLDREKN
>P80042 | GGPPS_CAPAN
MRSMNLVDLWAQQACLVFNQTLSYKSFNGFMKIPLKNSKINPKLNKKRPFSPLTVSAIAT
TKEDERIEAAQTEEPFNFKIYVTEKAISVNKALDEAIIVKEPHVIHEAMRYSLLAGGKRV
RPMLCLAACELVGGNQENAMAAACAVEMIHTMSLIHDDLPCMDNDDLRRGKPTNHKIYGE
DVAVLAGDSLLAFAFEHIVNSTAGVTPSRIVGAVAELAKSIGTEGLVAGQVADIKCTGNA
SVSLETLEFIHVHKTAALLESSVVLGAILGGGTNVEVEKLRRFARCIGLLFQVVDDILDV
TKSSEELGKTAGKDLVVDKTTYPKLLGLEKAKEFAAELNREAKQQLEGFDSRKAAPLIAL
ADYIAYRDN
>P80093 | CRTI_CAPAN
MPQIGLVSAVNLRVQGNSAYLWSSRSSLGTDSQDGCSQRNSLCFGGSDSMSHRLKIRNPH
SITRRLAKDFRPLKVVCIDYPRPELDNTVNYLEAAFLSSSFRSSPRPTKPLEIVIAGAGL
GGLSTAKYLADAGHKPILLEARDVLGGKVAAWKDDDGDWYETGLHIFFGAYPNMQNLFGE
LGINDRLQWKEHSMIFAMPNKPGEFSRFDFPEALPAPLNGILAILKNNEMLTWPEKVKFA
IGLLPAMLGGQSYVEAQDGISVKDWMRKQGVPDRVTDEVFIAMSKALNFINPDELSMQCI
LIALNRFLQEKHGSKMAFLDGNPPERLCMPIVEHIESKGGQVRLNSRIKKIELNEDGSVK
CFILNDGSTIEGDAFVFATPVDIFKLLLPEDWKEIPYFQKLEKLVGVPVINVHIWFDRKL
KNTSDNLLFSRSPLLSVYADMSVTCKEYYDPNKSMLELVFAPAEEWVSRSDSEIIDATMK

ELAKLFPDEISADQSKAKILKYHVVKTPRSVYKTVPGCEPCRLLQRSPVEGFYLAGDYTK
QKYLASMEGAVLSGKLCAQAIVQDYELLVGRSQRKLAETSVV
>P81372 | FERA_ALOMA
ATYKVKLVTPQGQQEFDCPDDVYILDQAEEEGIDLPYSCRAGSCSSCAGKVKQGEVDQSD
GSFLDDEQMEQGWVLTCVAFPTSDVVIETHKEEELTA
>P82192 | RK5_SPIOL
AAGTAVFVDKAEAETINRLKTNYIEKMVPLLKEEFSYSNILEVPKVVKIVVNCGIGDASQ
NAKGLDAAINELALITGQRPVKTKAKTSIAGFKVREGMTLGIAVTLRGNLMYSFLDRLIN
LALPRTRDFQGVNPNSFDGHGNYSVGFREQSVFPEIKPEIVGKARGMDVCITTTAKTDKE
AYKLLSLMGMPFREGSGPSTLVRKKKLKSHHFDAKKGRRY
>P82277 | RRP2_SPIOL
MATISSILPCHGLLQHCSSSSSSSKPKFSSQNLVQLQSFSNGFGLKLKTRVSTNPPLLKV
RAVVTEETSSSSTASSSSDGEGARRLYVGNIPRNLNNDELRTIVEEHGAIEIAEVMYDKY
SGRSRRFGFVTMKTVEDANAVIEKLNDTEIGGRKIKVNITEKPLEGMDIATTQAEDSQFV
ESPYKVYIGNLAKTVTNELLKDFFSEKGKVLGAKVQRTPGTSKSNGFGFVSFSSEEEVEA
AIQALNNSVLEGQKIRVNKA
>P83524 | FER_PHYAF
ATYKVKLITPDGPVVFDCPDNEYILDAAEEQGHDLPYSCRAGSCSSCAGKVTAGTVDQSD
GNFLDDDQVADGFVLTCVAYPQSDVTIETHKEEELTA
>P83584 | FER_SOLLS
ASYKVKLITPDGPIEFNCPDDVYILDRAEEEGHDLPYSCRAGACSSCAGKIVDGSVDQSD
NSFLDDDQIGGGFVLTCVAYPKSNVTIETHKEEALVG
>P83585 | FER_SOLAB
ATYKVKLVTPDGPVEFECPDDEYILDRAEEEGHDLPYSCRAGSCSSCAGKIAAGSVDQSD
GNFLDDDQIADGFVLTCVAYPQSDVTIETHKEEELTA
>P92209 | RPOA_AGRCR
MVREEVAGSTQTLQWKCVESRVDSKRLYYGRFILSPLRKGQADTVGIALRRALLGEIEGT
CITRAKFGSVPHEYSTIAGIEESVQEILLNLKEIVLRSNLYGVRDASICVKGPRYITAQD
IILPPSVEIVDRAQPIANLTEPIDFCIDLQIKRDRGYQTELRKNYQDGSYPIDAVSMPVR
NVNYSIFSCGNGNEKHEILFLEIWTNGSLTPKEALYEASRNLIDLFLPFLHAEEEGTSFE
ENKNRFTPPLFTFQKRLTNLKKNKKGIPLNCIFIDQLELTSRTYNCLKRANIHTLLDLLS
KTEEDLLRIDSFRMEDRKHIWDTLEKHLPIDLLKNKLSF
>P92225 | RPOA_CRIDE
MVREEVAGSTQTLQWKCVESRVDSKRLYYGRFILSPLRKGQADTVGIALRRALLGEIEGT
CITRAKFGSVPHEYSTIAGIEESVQEILLNLKEIVLRSNLYGVRDASICVKGPRYITAQD
IILPPSVEIVDTAQPIANLTEPIDFCIDLQIKRDRGYQTELRKNYQDGSYPIDAVSMPVR
NVNYSIFSCGNGNEKHEILFLEIWTNGSLTPKEALYEASRNLIDLFLPFLHAEEEGASFE
ENKNRFTPPLFTFQKRLTNLKKNKKGIPLNCIFIDQLELTSRTYNCXKRXXXXTLLDLLS
KTEEDLLRIDSFRMEDRKHIWDTLEKHLPIDLLKNKLSF
>P92309 | NDHJ_LUPLU
MQGRLSAWLVKHGLIHRSLGFDYQGIETLQIKPEAWHSIAVILYVYGYNYLRSQCAYDVA
PGGLLASVYHLTRIECGIDQPEEVCIKVFVQGKILGIPSIFWVWKSADFQERESYDMLGI
SYYNHPRLKRILMPESWIGWPLRKDYIAPNFYEIQDAH
>P92429 | RPOA_AEGTA

MVREEVAGSTQTLQWKCVESRVDSKRLYYGRFILSPLRKGQADTVGIALRRALLGEIEGT
CITRAKFGSVPHEYSTIAGIEESVQEILLNLKEIVLRSNLYGVRDASICVKGPRYITAQD
IILPPSVEIVDTAQPIANLTEPIDFCIDLQIKRDRGYQTELRKNYQDGSYPIDAVSMPVR
NVNYSIFSCGNGNEKHEILFLEIWTNGSLTPKEALYEASRNLIDLFLPFLHAEEEGTSFE
ENKNRFTPPLFTFQKRLTNLKKNKKGIPLNSIFIDQLELTSRTYNCLKRANIHTLLDLLS
KTEEDLLRIDSFRMEDRKHIWDTLEKHLPIDLLKNKLSF
>P92892 | RPOA_AEGSP
MVREEVAGSTQTLQWKCVESRVDSKRLYYGRFILSPLRKGQADTVGIALRRALLGEIEGT
CITRAKFGSVPHEYSTIAGIEESVQEILLNLKEIVLRSNLYGVRDASICVKGPRYITAQD
IILPPSVEIVDTAQPIANLTEPIDFCIDLQIKRDRGYQTELRKNYQDGSYPIDAVSMPVR
NVNYSIFSCGNGNEKHEILFLEIWTNGSLTPKEALYEASRNLIDLFLPFLHAEEEGASFE
ENKNRFTPPLFTFQKRLTNLKKNKKGIPLNCIFIDQLELTSRTYNCLKRANIHTLLDLLS
KTEEDLLRIDSFRMEDRKHIWDTLEKHLPIDLLKNKLSF
>P93960 | RPOA_PSARU
MVREEVAGSTQTLQWKCVESRVDSKRLYYGRFILSPLRKGQADTVGIALRRALLGEIEGT
CITRAKFGXVPHEYSTIAGIEESVQEILLNLKEIVLRSNLYGVRDASICVKGPRYITAQD
IILPPSVEIVDTXQPIANLTEPIDFCIDLQIKRDRGYQTELRKNYQDGSYPIDAVSMPVR
NVNYSIFSCGNGNEKHEILFLEIWTNGSLTPKEALYEASRNLIDLFLPFLHAEEEGTSFE
ENKNRFTPPLFTFQKRLTNLKKNKKGIPLNYIFIDQLELTSRTYNCLKRANIHTLLDLLS
KTEEDLMRIDSFRMEDRKHIWDTLEKHLPIDLLKNKLSF
>P93964 | RPOA_SECST
MVREEVAGSTQTLQWKCVESRVDSKRLYYGRFILSPLRKGQADTVGIALRRALLGEIEGT
CITRAKFGSVPHEYSTIAGIEESVQEILLNLKEIVLRSNLYGVRDASICVKGPRYITAQD
IILPPSVEIVDTAQPIANLXEPIDFCIDLQIKRDRGYQTELRKNYQDGSYPIDAVSMPVR
NVNYSIFSCGNGNEKHEILFLEIWTNGSLTPKEALYEASRNLIDLFLPFLHAEEEGASFE
ENKNRFTPPLFTFQKRLTNLKKNKKGIPLNCIFIDQLELTSRTYNCLKRANIHTLLDLLS
KTEEDLLRIDXFRMEDRKHIWDTLEKHLPIDLLKNKLSF
>P93974 | RPOA_EREDI
MVREEVAGSTQTLQWKCVESRVDSKRLYYGRFILSPLRKGQADTVGIALRRALLGEIEGT
CITRAKFGSVPHEYSTIAGIEESVQEILLNLKEIVLRSNLYGVRDASICVKGPRYITAQD
IILPPSVEIVDTAQPIANLTEPIDFCIDLQIKRDRGYQTELRKNYQDGSYPIDAVSMPVR
NVNYSIFSCGNGNEKHEILFLEIWTNGSLTPKEALYEASRNLIDLFLPFLHAEEEGTSFE
ENKNRFTPPLFTFKKRLTNLKKNKKGIPLNCIFIDQLELTSRTYNCLKRANIHTLLDLLS
KTEEDLLRIDSFRMEDRKHIWDTLEKHLPIDLLKNKLSF
>Q00218 | AROG_ARATH
MVTLNASSPLTTKSFLPYRHAPRRPISFSPVFAVHSTDPKKSTQSASASVKWSLESWKSK
KALQLPDYPDQKDVDSVLQTLSSFPPIVFAGEARKLEDKLGQAAMGQAFMLQGGDCAESF
KEFNANNIRDTFRVLLQMGVVLMFGGQLPVIKVGRMAGQFAKPRSDPFEEKDGVKLPSYR
GDNINGDAFDEKSRIPDPHRMVRAYTQSVATLNLLRAFATGGYAAMQRVSQWNLDFTQHS
EQGDRYRELANRVDEALGFMGAAGLTSAHPIMTTTEFWTSHECLLLPYEQALTREDSTSG
LYYDCSAHMLWVGERTRQLDGAHVEFLRGIANPLGIKVSDKMVPSELVKLIEILNPQNKP
GRITVIVRMGAENMRVKLPNLIRAVRGAGQIVTWVSDPMHGNTIMAPGGLKTRSFDAIRA
ELRAFFDVHDQEGSFPGGVHLEMTGQNVTECVGGSRTITYNDLSSRYHTHCDPRLNASQS
LELAFIIAERLRKRRLGSGNLPSSIGV

>Q00775 | SSG1_SOLTU
MASITASHHFVSRSQTSLDTKSTLSQIGLRNHTLTHNGLRAVNKLDGLQSRTNTKVTPKM
ASRTETKRPGCSATIVCGKGMNLIFVGTEVGPWSKTGGLGDVLGGLPPALAARGHRVMTI
SPRYDQYKDAWDTSVAVEVKVGDSIEIVRFFHCYKRGVDRVFVDHPMFLEKVWGKTGSKI
YGPKAGLDYLDNELRFSLLCQAALEAPKVLNLNSSNYFSGPYGEDVLFIANDWHTALIPC
YLKSMYQSRGIYLNAKVAFCIHNIAYQGRFSFSDFPLLNLPDEFRGSFDFIDGYEKPVKG
RKINWMKAGILESHRVVTVSPYYAQELVSAVDKGVELDSVLRKTCITGIVNGMDTQEWNP
ATDKYTDVKYDITTVMDAKPLLKEALQAAVGLPVDKKIPLIGFIGRLEEQKGSDILVAAI
HKFIGLDVQIVVLGTGKKEFEQEIEQLEVLYPNKAKGVAKFNVPLAHMITAGADFMLVPS
RFEPCGLIQLHAMRYGTVPICASTGGLVDTVKEGYTGFHMGAFNVECDVVDPADVLKIVT
TVARALAVYGTLAFAEMIKNCMSEELSWKEPAKKWETLLLGLGASGSEPGVEGEEIAPLA
KENVATP
>Q00864 | CHLB_PINTH
MKLAHWMYAGPAHIGTLRVASSFKNVHAIMHAPLGDDYFNVMRSMLERERNFTPATASIV
DRHVLARGSRKRVVDHIIRKDKEEGPDLIILTPTCTSSILQEDLKNFVDRASIISDCNVI
FADVDHYQVNEIQAADRTLEQVVRYYLEKSHTLDQFVTDAPSVNIIGILTLGFHNRHDCR
ELRRLLKDLDIRINQIIPEGGSVEDPKNLPKARFNLIPYREVGLMTAMYLNKEFGMPYVS
TTPMGAVDMAECIRQIKKSLETLAAPILSSKRVDYESYIDGQTRFVSQAAWFSRSIDCQN
FTGKETVVFGDATHAASITKILAREMGIRVSCTGTYCKHDAEWFKEQIKDFCDEIIITDD
HAEVGDIISRVEPSAIFGTQMERHIGKRLEIPCGVISAPAHIQNFSLGYRPFLGYEGTNQ
IADLVYNSFALGMEDHLLDIFCGHDTKEIMTKSLSTDISPIWDPESRQELGKIPRFVRDE
VKRNTEKFARRKGILNVTVEVMHAAKEALS
>Q00866 | MATK_PINTH
MDEFHRCGKEDSFWQQCFLYPLFFQEDLYAISHDHYLDVSSSSRPMEHLSSNDQLSFLTV
KRLIGQIRQQNHSIVLFVNCDPNPLADRKKSFYSESVLEALTLVLEVPFSIWSKSSVEGM
NECKSFRSIHSIFPFLEDKFPHSNSILDARIPYSIHPEILVRTFRRWIRDAPSLHPLRSV
LYDYRNSPENLQRSIIVVPRVNTRFFLFLLNYYVCECESILFSRLKRSSHSRSLSHGSFP
QRTHFHRKIKHIIIFSRRNSLKSIWSLKDPKIHYVRYGERPIIAIKGADLLVKKCRYYLL
IFRQFYFHLWSEPYRVCSHQLSKNCSSSPGYFLRVRMNPLLVRTKTLDELFIPVLITNEM
DPIVPIVPIIGLLATEKFCDISGRPISKLSWTSLTDDDILDRFDQIWRNLFHYYSGSFDR
DGLYRIKYILLLSCAKTLACKHKSTIRVVRKELGPELFKKSFSKEREFDSLPFSSKAAAR
SQRERIWHSDIPQINPLANSWQKIQDLKIENLFDQ
>Q01923 | RPOC2_SORBI
MAERANLVFHNKEIDGTAMKRLISRLIDHFGMGYTSHILDQIKTLGFHQATTTSISLGIE
DLLTIPSKGWLVQDAEQQSFLLEKHYYYGAVHAVEKLRQSVEIWYATSEYLKQEMNSNFR
ITDPSNPVYLMSFSGARGNASQVHQLVGMRGLMADPQGQMIDLPIQSNLREGLSLTEYII
SCYGARKGVVDTAVRTADAGYLTRRLVEVVQHIIVRRRDCGTIQGISVSPQNGMTEKLFV
QTLIGRVLADDIYIGSRCIASRNQDIGIGLVNRFITAFRAQPFRAQPIYIRTPFTCRSTS
WICQLCYGRSPTHGDLVELGEAVGIIAGQSIGEPGTQLTLRTFHTGGVFTGGTADLIRSP
SNGKIQFNEDLVHPTRTRHGQPAFLCYIDLHVTIQSQDILHSVNIPLKSLILVQNDQYVE
SEQVIAEIRAGTSTLHFKEKVQKHIYSESDGEMHWSTDVYHAPEYQYGNLRRLPKTSHLW
ILSVSMCRSSIASFSLHKDQDQMNTYSFSVDGRYIFDFSMANDQVSHRLLDTFGKKDREI
LDYLTPDRIVSNGHWNCFYPSILQDNSDLLAKKRRNRFAVPLQYHQEQEKERISCLGISM
EIPFMGVLRRNTIFAYFDDPRYRKDKRGSGIVKFRYRTLEEEYRTREEEYRTREEDSEDE

YESPENKYRTREGEGEYEILEDEYRTLEDEYETLEDEYGILEDEYRTLEKDSEEEYGSLE
NKYRTREGEGEYEILEEDSEEEYGSSEDGSEKEYGTLEEDSEEDSEEDSEDEYGSPEEDS
ILKKEGFIEHRGTKEFSLKYQKEVDRFFFILQELHILPRSSSLKVLDNSIIGVDTQLTKN
TRSRLGGLVRVKRKKSHTELKIFSGDIHFPEEADKILGGSLIPPEREKKDSKESKKRKNW
VYVQRKKILKSKEKYFVSVRPAVSYEMDEGRNLATLFPQDLLQEEDNLQLRLVNFISHEN
SKLTQRIYHTNSQFVRTCLVVNWEQEVKEGARASLVEVRTNDLIRDFLRIELVKSTISYT
RRRYDRTSVGLIPNNRLDRNNTNSFYSKAKIQSLSQHQEVIGTLLNRNKEYPSLMILLAS
NCSRIGLFKNSKYPNAVKESNPRIPIRDVFGLLGVIVPSISNFSSSYYLLTHNQILLKKY
LFLDNLKQTFQVLQGLKYSLIDENQRISNFGSNIMLEPFHLNWHFLHHDSWEETLAIIHL
GQFICENLCLFKSHIKKSGQIFIVNMDSFVLRAAKPYLATIGATVHGHYGKILYKGDRLV
TFIYEKSRSSDITQGLPKVEQIFEARSIDSLSPNLERRIEDWNERIPRILGVPWGFLIGA
ELTIAQSRISLVNKIQKVYRSQGVQIHNRHIEIIIRQVTSKVRVSEDGMSNVFLPGELIG
LLRAERAGRALDESIYYRAILLGITRASLNTQSFISEASFQETARVLAKAALRGRIDWLK
GLKENVVLGGIIPVGTGFQKFVHRSPQDKNLYFEIKKKNLFASEMRDILFLHTELVSSDS
DVTNNFYETSETPFTPIYTI
>Q04450 | RBS2_MESCR
MASMMSNAAVVGRTTPAQASMVAPFTGLKSVSAFPVTKKSNDITSIASNGGRVQCMQVWP
PLGKKKFETLSYLPPLSEESLMKEVQYLLNNGWVPCLEFEPTHGFVYREHGNTPGYYDGR
YWTMWKLPMFGCTDPSQVVAELEEAKKAYPEAFTRIIGFDNVRQVQCISFIAYKPASYDA

>Q06021 | RBL_ALNIN
MSPQTETKASVGFKAGVKDYKLTYHTPDYETKDTDILAAFRVTPQPGVPPEEAGAAVAAE
SSTGTWTTVWTDGLTSLDRYKGRCYHIEPVAGEESQFIAYVAYPLDLFEEGSVTNMFTSI
VGNVFGFKALRALRLEDLRIPPAYSKTFQGPPHGIQVERDKLNKYGRPLLGCTIKPKLGL
SAKNYGRAVYECLRGGLDFTKDDENVNSQPFMRWRDRFLFCAEAIYKAQAETGEIKGHYL
NATAGTCEEMMKRAVFARELGVPIVMHDYLTGGFTANTSLAHYCRDNGLLLHIHRAMHAV
IDRQKNHGIHFRVLAKALRMSGGDHIHAGTVVGKLEGEREITLGFVDLLRDDYIEKDRSR
GIYFTQDWVSLPGVLPVASGGIHVWHMPALTEIFGDDSVLQFGGGTLGHPWGNAPGAVAN
RVALEACVQARNEGRDLAREGNEIIREAAKWSPELAAACEVWKEIKFEFPAMDTL
>Q06022 | RBL_BETPA
MSPQTETKASVGFKAGVKDYKLTYHTPDYETKDTDILAAFRVTPQPGVPPEEAGAAVAAE
SSTGTWTTVWTDGLTSLDRYKGRCYHIEPVAGEESQFIAYVAYPLDLFEEGSVTNMFTSI
VGNVFGFKALRALRLEDLRIPPAYSKTFQGPPHGIQVERDKLNKYGRPLLGCTIKPKLGL
SAKNYGRAVYECLRGGLDFTKDDENVNSQPFMRWRDRFLFCAEAIYKAQAETGEIKGHYL
NATAGTCEEMMKRAIFARELGVPIVMHDYLTGGFTANTSLAHYCRDNGLLLHIHRAMHAV
IDRQKNHGIHFRVLAKALRMSGGDHIHAGTVVGKLEGEREITLGFVDLLRDDYIEKDRSR
GIYFTQDWVSLPGVLPVASGGIHVWHMPALTEIFGDDSVLQFGGGTLGHPWGNAPGAVAN
RVALEACVQARNEGRDLAREGNEIIREAAKWSPELAAACEVWKEIKFEFPAMDTL
>Q06030 | RK121_SECCE
MASTTFSSAFSILSLPSSSPSPPPSPPRTLPVANRRRRAAAVASTATESPKVLELGDAIA
GLTLEEARNLVDHLQERLCVSAASFPPAAAGLRAAAVEEAPVEQTEFDVVIEEVPSSARI
ATIKIVRALTNLALKEAKDLIEGLPKKLKEAVSKDEAEEAKKQLEGVGAKVSIA
>Q06FM0 | RR7_PELHO
MSRRGPAEKKTVKRDPFYRNRVVNMLINRILKHGKKSLAYKILYRAMKKIQQKKKRNPLS

ILRQAIRRVTPKIAVKRRRVSGSAYQVPIEIKAPQGKVLAIRWLLEAARKRPGRNMAFKL
SSELMDATKGKGNAIRKKEETYRMAEANRTFANFR
>Q06FM3 | RK2_PELHO
MAIHLYKTSTPSTRKRAVDSQGKSNPRNHLIYGQHRCGKGRNARGIITAGHRGGGHKRLY
RQIDFRRNENNIYGRIVTIEYDPNRNAYICLIHYGDGEKRYILHPRGARIGDTIVSGTEV
PIKMGNALPLTNIPLGTAIHNIEITLGKGGQLARAAGAVAKLISKEGKSAAVKLPSGEVR
LISQNCSATVGQVGNVGVNRKSLGRAGSKRWLGKRPVVRGVAMNPVDHPHGGGEGKAPIG
RKKPATPWGRPALGIRTRKRKKYNDNLILRRRSK
>Q06FM9 | RR8_PELHO
MGKDTLSEIVTSIRNADMAKKETVRITFTNIAQNIVTILLREGFIKNAREHRESKKSFLV
LTLRHRRNRKGPSRTLFNLKRVSRPGLRIYSNYQKIPRILGGMGVAILSTSKGIMTDREA
RLKRIGGEILLYIW
>Q06FN4 | RPAL2_PELHO
MSNPNNGAEWQQVEADQLDSGLYYGRFALSPLTAKQASLLKKGLPEALLTEILCLRFTHA
KIQNECVNLMNIVGIQESLDEILKNFGKIILTGKLEEFVGKGPFVAILDVRGPLNAMAVD
IELPPGIKVEIETQHIATITEPIPFVVELKIELVSSTSKGETGITDEEGFSIDPNPPIQK
VDTSIQCYDYQGEPFQTLFLDIWTDRTIHPHEALAQASRKIFGLLSLVFQAEYFQYNELE
NGLRYGKFCLYPMTKEQFQWIQTALNEALALDMSGESKHEGPVTDEEGDSIDPTFTPVQK
WDITMNSYQYSGETFQGLLSRF
>Q06FP4 | RK20_PELHO
MTRIKRGSIARRRRAKTRLFASGARGAHSRLTRLIAQQTIRALASAHRDRDKKKRDFRRL
WITRINGVIRQNVLSYSYSQLITNLKKEQLLLNRKMLAQIAISNRNCLSIIYSAIIILET
KEEGQK
>Q06FP5 | RK32_PELHO
MTVPKKRLSSSKKRIRKNIWKGKGHWAALKALSLGKSLSTGNSKSFFRFFSMPTRFFSMP
TDNKKTKKS
>Q06FX0 | RPOC1_PELHO
MIDKYQEPKHHQLRIGLVSPQQIMAWANKTLPTGEIVGEVKNYRTFSYDRDSETYKPERD
GLFCERIFGPIKSGVCACGESRKIGDEKEKRTFCEKCGVEFVDSRIRRYQMGYIKLASPM
AHVWYLKRIPSYIANLLDEKIKDLENLVYRDFIFAGPLTKKPTLLRLRGSFPESKTKFSG
SRDILSQFLKTKSFQKLRDREFSAGAGAIRKRLANLDLEVIIYSSLAEWKKRKEQKAEIE
IKPTRTERTEEEDRKIEKIKRRMRVLVRRMELAKHFLETNVKPEWMILRLLPVLPPELRP
IFKISEVQFISSDLNTLYKEVISNNKILSDSLLGCKFVPEQLLTGQEKIVQVAVDQLLDN
RISGQPRRDRNKQLYKSFSDILAGKEGRFRQTLLGRRVDYSGRSVIVVGPSLALHQCGLP
REIALELFQTFIIRSLIRQRLASSVKTAKKQIREQERIVWDILEEVVQEHPVLLNRAPTL
HRLGIQAFQPILTKERAIYLHPLVCKGFNADFDGDQMAVHLPLSLEAQAEAYLLMFSTQN
LLSPGNGDPICVPTQDMLTGLYTLTLGNRRGICATRYNLWNRRNSQNERIDDKHYGSTKG
KELLFCNPDDAIGAYRQKRINLDSPFWLKVTWGQHRIVSKELPVEVHYESLGTHHEIYGH
CKIVRSVKTERHFIYIRTTVGHLFLDREIEDALKDFRARVV
>Q06FX1 | RPOC2_PELHO
MKEWPPNLAFHNKVIDGAAIKQLISRLIQRFGMLYTSHVLDQVKLLGFKQATASSISLGI
DDLLTIPSKRWLVQDAQQQSFLLEKYYQYGNVHAVEKLRQSIEIWDAISAYLRQEMTPNF
GMTDPLNPVHIMSFSGARGNASQVNQLIGMRGLMSDPLGQMIDLPIQSNLREGLSLTEYI
ISSYGARKGVVDTAIRTADAGYLTRRLVDVVQHIIVRGTDCGTTRGLSLSPQNGGVPEST

QTLIGRVLAKDIYIGPRCIAVRNQDLGGGLVNRLITFKKKPIYVRTPFTCRSTSWICRFC
YGRSPTSACGDLVELGEAVGIIAGQSIGEPGTQLTLRTFHTGGVFTGGTAEQVRAPFNGK
IKFNEDLVHPTRTRHGQPAFLCYIDLYLTIESEDIIHSVPIPPKSFLLVQNDQYVESEQV
IAEISAGTSTFAFKDRVRKHIYSDSSGEMHSNTGMYHARKFPFSNVHILPKTSHLWILLG
DLCGSGPVLFSMYKDQDQMSIHSLSVQGRNTPSLSVNNDQLKHRRLSLYDKNGTGGGSIP
ILNYSEMTRSLSTARCNLLYPAILHENFSLLAKKRRTRFLIPFQSIQEHRRKNERILCSD
ISIKIPRNGLFRGNSILAYFDDPRYKIGVSGITKYGTIEVDSIVTKESFINYGGVQPKDE
GKVDPLFFIPEEVHIFPETSSIMVRNNSIIGINTKITLNKRSRVGGLVRVKKIHRGIKLQ
IFSGDISFPRKRDKRFIPQNKDILIPPQIRKRNFNKSKKAKNWIYVQKMRPTNKNSFFLL
QPVVTYEIADGINLARLFPPDLLQEIDNIQLRVGNYIRYGDGELIPGISGKNPRIQLVRT
FLVLNWDQDKKDSSIEEAHASFVEVSTKGMVRDFLRIHLRKSQIAYAYMRKRNDPSGSGF
PSDNELDHSNRNPFSSSYPKARVRQSLNGTIRTLLNRNKECQSLLILSSSNCFRLGPFNN
VKYHNPKKESIKRDTDPLIPIRNFSGPLGTVPQIANFDVFYHLITKNRISVFHYLPPEKG
KLQVIQSFLMDENGRIYKSDPYSNIFLNPFNFNWYFLQHNYHKNYPNYCEETSTIINLGQ
FLFETVCIAKNGPRLKSGQVFILQVGYVIIRSGKPYLAIPGATVHGHSGQIVYGGDTLVT
FIYEKARSADITQGLPKVDAMFEVRSKNSISMRLKARDEYWNEYITRNLGIYWGLLIGAK
LTIAQSSLSLVNEIQKVYRSQGVQIDNRHIELIVRQITSKVLISEDGMSNLFLPGELIGL
LQAERTGRALEKGICYRAILVGITKASLNTQSFISEASFQQTARVLAKAALRGRIDWLKG
LKENVVLGGMSPAGTGFRGLVHPSSKKYKTLSLETQRKILFDGKVRDLLFHHKRIV

>Q06GJ6 | RK23_PIPCE

MDGIKYIVFTEKSIRLLGNNQYTSNVESGSTRTEIKHWVELFFGVKVIAMNSHRLQGKGR
RMGPIIGHTMHYRRMIITLQPGYSIPPLIEKRT

>Q06GL9 | RR19_PIPCE

MARSLKKSPFVANHLLKKIEKLNMKGGKEIIVTWSRASTIIPTMIGHTIAIHNGKEHLPI
YITDRMVGHKLGEFAPTLTFRGHARNDNRSRR

>Q06GM1 | RR3_PIPCE

MGKKINPLGFRLGANQSHRSIWFAQSKSYSRGLQEDEKIRDCIQHYVEKNTRISSGFEGK
GISHIEIQKKIDLIQVIIYMGSPNSLMEGRTRGIDELQTNVQKEFLSVNRRLNIAIIRVA
KPYAQPTILAEHIALQLKNRVSFRKAMKSAIELTEQADTKGIQIQIAGRIDGKEIARVEW
IREGRVPLQTIRAKIDYCSYTVRTIYGILGIKIWIFLDEK

>Q06GM5 | IF1C_PIPCE

MKEQKLIHEGLITESLPNGMFRVRLDNEDLILGYVSGRIRRSFIRILPGDRVKIEVSRYD
STRGRIIYRLRNKDSND

>Q06GM8 | RPOA_PIPCE

MVREEIAVATGAPRWKCVESRIDSKRLYYSRFILSPLMKGQADMIGIAMRRALLGEIEGT
CITRVKSEKVPHEFSTIVGIEESVHEIVMNLKEIVLRSNLYGTLDASICIRGPRYVTAQD
IILPPSVEIVDATQHIANLTERVDLCIKLQIERDRGYSMKTPHNDEDGSYPIDAIFMPVR
NANHSVHSYGNGNEKQEILFIEIWTNGSLTPKEALYEASRNLIDLFIPFLHAEEREIHLE
DNANGVPLALFTFHDEFAITNIRKNKKKMALKSIFIDQSELPSKTYNCLKKSNIHTLLDL
LNNSQEDLLKIEHFCMEDVKRILDILQKHFGFDLPKNGK

>Q06GN5 | CLPP_PIPCE

MPIGVPKVPFRSPGEEDAAWVDVYNRLHRERLLFLGQEVDSEISNQIVGLMVYLSIEDGT
RDLYLFINSPGGWVIPGIAIYDTMQFVSPDVHTICMGLAASMGSFILVGGEITKRLAFPH
AWVMIHQPASSFYEAPTGEFILEAEELLKLRETLTRVYVQRTGNPLWVVSEDLERDVFMS

ATEAQAHGIVDLVAVENSGNFT
>Q06GN7 | RK20_PIPCE
MTRVRRGYIARRRRTKIRLFASTFRGAHSRLTRTITQQKMRALVSAHRDRGRKKRDFRRL
WITRINAVIRGSGVSYSYSRLIHDLYKRQLLLNRKILAQIAISNRNCFYMISNQIIKSGE
CEEFNGII
>Q06GN8 | RR18_PIPCE
MDKTKRPLRKSKRSFRRRLPPPIGSGDRIDYRNMSLISRFISEQGKILSRRVNRLTLKQQ
RLITIAIKQARILSSLPFLNNEKQFERPESIPKTAGPSIRNK
>Q06GR2 | RR14_PIPCE
MARKSLIQRERKRQKLEQKFHSIRRSSKKEISKVSSLSGKWEIHGKLQSPPRNSAPIRLH
RRCFLTGRPRGNYRDFGLSGHILRERFHACLLPGATRSSW
>Q06GR8 | RPOB_PIPCE
MLRNRNEGMSTIPEFSQIQFEGFCRFIDWGLAEELHKFPKIEDTDQEIEFQLFVETYQLA
EPLIKERDAVYESLTYSSELYVPAGLIWKPGKDMQEQTVFIGNIPLMSSLGIFIVNGIYR
IVINQILQSPGIYYRSELDHNGIFIYTGTIISDWGGRSELEIDRKARIWARVSRKQKISI
LVSSSAMGSNLREILENVRYPEIFLSFLNERDKKKIGSRENAILEFYQQFACVGGDPVFS
ESLCRELQKKFFQQRCELGRIGRRNMNRRLNLDIPQNNTFLLPRDVLAAADHLIGMKFGM
GTLDDMNHLKNKRIRSVADLLQDQFGLALVRLENVVRGTICGAIRHKLIPTPQNLVTSTP
LTTTYESFFGLHPLSQVLDRTNPLTQIVHGRKSSYLGPGGLTGRTASFRIRDIHTSHYGR
ICPIDTSEGINVGLIGSLAIHARVGPWGSIKTPFYEISERSKRLQLVYLSPSVDEYHMVA
AGNSLALNRGIQEEQAVPARYRQEFLTIAWEQIHLRSIFPFQYFSMGASLIPFIEHNDAN
RALMSSNMQRQAVPLSRSEKCIVGTGLERQAALDSGVSAISEREGKVIYTNTDKIILSGN
GDTITIPLVMYQRSNKNTCMHQKTQVSRGKCIKKGQILANGAATVGGELALGKNVLVAYM
PWEGYNFEDAVLISERLVYEDIYTSFHIRKYEIQTHVTSQGPERITKEIPHLETHLLRNL
DRNGIVMLGSWVETGDILVGKLTPQTAKESSYAPEDRLLRAILGIQVSTSKETCLKLPIG
GRGRVIDVRWIQKKGGSSYNPETIRVYISQKREMKVGDKVAGRHGNKGIISKILPRQDMP
YLQDGTPVDMVFNPLGVPSRMNVGQIFECSLGLAGDFLGRHYRVAPFDERYEQEASRKLV
FSELYEASKQTANPWVFEPEYPGKSRIFDGRTGDPFDQPVLIGKSYILKLIHQVDDKIHG
RSSGHYALVTQQPLRGRAKQGGQRVGEMEVWALEGFGVAHILQEMLTYKSDHIRSRQEVL
GTTIVGGTIANPGDAPESFRLLVRELRSLALELNHFLLSEKDFQIHRKEA
>Q06GR9 | RPOC1_PIPCE
MIDRYKHQQLRIGPVSPQQISAWANKILPNGEIVGEVTKPYTFHYKTNKPEKDGLFCERI
SGPIKSGICACGNYRVIGDEKEDPNFCEQCGVEFADSRARRYQMGYIKLTCPVTHVWYLK
RLPSYIANLLDKPLKELEGLVYCDFSFARPVAKKPTFLRLRGLFEYEIQSRKYSIPLFFT
TQGFDTFRNREISTGASAIREQLADLDLRIIIDRSLVEWKELGEEGSTGNEWEDRKIGRR
KDFLVRRIELAKHFIRTNVEPERMVLSLLPVLPPELRPIIQIDGGKPMSSDINELYRRVI
YRNNTLTDLLTTSRSTPGELVMCQEKLVQEAVDTLLDNGIRGQPMRDGYNKVYKSFSDVI
EGKEGRFRETLLGKRVDYSGRSVIVVGPSLSLHRCGLPREIAIELFQTFLIRGLIRQRVA
SNIGIAKSKIREKEPIVWEILQEIMRGHPVLLNRAPTLHRLGIQAFQPILVEGRAICLHP
LVCKGFNADFDGDQMAVHVPLSLEAQAEARLLMFSHMNLLSPAIGSPISVPTQDMLIGLY
VLTIGNRQGVCANRYNPWNRRNYQNETVDHTKYDRTNYRYTKEKEPYFCSSYDALGAYRQ
KRIHLDTPLWLRWRLDQRIISLREVPIEVQYESLGTYHEIYRPYLIVKSVKKEILCIYIR
TTVGHISFYREIEEAIQGFCRAYSYDGT
>Q06GS0 | RPOC2_PIPCE

MEVLMAEQRADLVFHNKAIDGTAMKRLISRLIDHFGMAYTSHILDQVKTLGFQQATATSI
SLGIDDLLTIPSKGWLVRDAEQQGLILEKHHHYGNVHAVEKLRQSIEIWYATSEYLRQEM
HTNFRMTDPSNPVHIMSFSGARGNASQVHQLVGMRGLMSDPQGQMIDLPIQSNLREGLSL
TEYIISCYGARKGVVDTAVRTSDAGYLTRRLVEVVQHIVVRRTDCGTIRGISASPRNSME
EKIWIQRLIGHVLAYDIYIGPRCIAARNQDIGIGLVNRFITFQAKSIYIRTPFICRSTSW
ICRLCYGRSPTHGDLVELGEAVGVIAGQSIGEPGTQLTLRTFHTGGVFTGGTAEYVRAPS
NGKIKFHEDLVHPTRTRHGHPAFLCYIDLYVTIESRDILHKVNIPPKSFILVQNNQYVES
EQVIAEIRAGTSALSFKEKVRKHIYSDSEGEMHWSTNVYHTPQYTYGNVHLLPKTSHLWI
LSGNPCKSNGALFSLHKDQDQMNLYSLSLSLEQRSISDFSVTNDRVRRKSFSLDPSREGR
GGRFFDYSVPDKSIPNDCWNFIYSAILHENSYFLAKRRRNRFIIPFQYSQEGEKEPMPCS
GISIEIPKTGILRRNTILAYFDDPRYRRSGSGITKYGTVEVDSLVKKEDLVEYRGEKELR
TKYQTKVDRFFFIPEEVHILPGSSSIMVRNNSIIGVDTEIALNTRSRVGGLVRVERKKKR
TELKIFSGNIHFPGETDKISRHSGILLPPGMEKKNSKESKKWKNWIYVQRVTPIKKKYFV
SVRPVVTYEIADGINLATLFPQDSLQERDNVQLRVVNYILYGNGKPIRGISHTSIQLVRT
CLVLNWDQDNKKTSSIEEVFASFVEVRANDLIRDFIRVDLVKPRVLNTGKRNDTAGYGLN
PDNGSDCPNRNPFYSKIKIQSFTQDNGTIHTSLNINKQLPPFLILSSSNCFQAGPLKVNG
SKSDNGKKKSIKEDPAILIRNFFGPLGTIAKITNLDSRNYYLTDNQILLKKYLILDNFKN
LFQDVLYYLIDEKGRILNPDPCSNIILNAFDLNWCFLHHNSCEKTFSIISLGQLFYENGS
IYKYRSHIKSGQIVTVHGDSLVIRSAKLHLATPGATVHGHYGEIIYEGDTLVTFIYEKSR
SGDITQGLPKVEQVLEVRSIDSISMNLEKRVEGWNENITKILGIPWGFLIGAELTIVQSR
ISLVNKIQKVYRSQGVQIHNRHIEIIVRQITSKVLVSEDGMSNAFSPGELIGLLRAERTG
RALEETICYRAILLGLTRASLNTQSFISEASFQETARVLAKAALRGRIDWLKGLKENVVL
GGMIPVGTGFKGFVRRSRQHNNISLEKKRNNLVKGEIGNILFHHRELFSSCISKNKKKFS

>Q06GT3 | RK23_DRIGR
MDGIKYAVFTEKSIRLLGNNQYTSNVESGSTRTEIKHWVELFFGVKVIAMNSHRLPGKGR
RMGPIMGHTMHYRRMIITLQPGYSIPPLIEKRT
>Q06GT7 | RR7_DRIGR
MSRRGTAEEKTAKSDPIYRNRLVNMLVNRILKHGKKSLAYQIIYRAVKKIQQKTETNPLS
VLRQAIRGVTPDIAVKARRVGGSTHQVPIEIGSTQGKALAIRWLLGASRKRPGRNMAFKL
SSELVDAAKGSGDAIRKKEETHRMAEANRAFAHFR
>Q06GT9 | RR15_DRIGR
MVKNLFISVIPQEEKEKNKGSVEFQVFNFTNKIRRLTAHLEFHRKDYLSQRGLRKILGKR
QRLLAYLSKKNRVRYKELIGQLDIREPKTR
>Q06GU8 | RK32_DRIGR
MTVPKKRTSMSKKRIRKNIWKRKGYWAALKALSLAKSISTGHSKSFFVQQTSNKAAE
>Q06GV6 | RR19_DRIGR
MTRSLKKNPFVDNHLLGKIEKLNMREEKEIIVTWSRASTIIPTMIGHTIAIHNGKAHLPI
YIIDRMVGHKLGEFAPTLTFRGHARNNNVSRR
>Q06GV8 | RR3_DRIGR
MGKKINPLGFRLGANQSHRSLWFAQPKSYSRGLQEDEKIRDCIKNYVQKNMRSGSEGISH
IEIKKKIDLIQVIIYIGFPNLLIEGRTRGIEELRINVQKGFRSVNRRLNIAITRVAKPYG
QPNILAEYIALQLKNRVSFRKAMKKAIELTEQADTKGIQVQIAGRIDGKEIARVEWIREG
RVPLQTIRAKIDHCSYTVRTIYGVLGIKIWIFVDEQ

>Q06GW2 | IF1C_DRIGR
MKEQKLIHEGLITESLPNGMFRVRLDNEDLILGYVSGRIRRSFIRILPGDRVKIEVSSYD
STKGRIIYRLRNKDSND
>Q06GX3 | RR12_DRIGR
MPTIKQLIRNTRQPIKNVTKSPALRGCPQRRGTCTRVYTITPKKPNSALRKVARVRLTSG
FEITAYIPGIGHNLQEHSVVLVRGGRVKDLPGVRYHIVRGTLDAVGVKDRQQGRSKYGVK
KPK
>Q06GX4 | RK20_DRIGR
MTRVRRGYIARRRRTKIRLFTSTFRGAHSRLTRTTTQQKMRALVSSHRDRCRQKRDFRRL
WITRINAVTRENRVSYSYSRLMHDLYKRQLLLNRKILAQIAISNRNCIYMISNEIIK
>Q06GX5 | RR18_DRIGR
MDKSKRPFRKSKRSFRRRLPPIGSGDRIDYRNMSLISRFISEQGKILSRRVNRLTLKQQR
LITIAIKQARILSSLPFLNNEKQFERTESTPRTAGPKTRNK
>Q06GZ5 | RR4_DRIGR
MSRYRGPRFKKIRRLGALPGLTRKRPRSGSDLRNQSRSGKRSQYRIRLEEKQKLRFHYGL
TERQLLRYVRIAGKAKGSTGQVLLQLLEMRLDNILFRLGMASTIPGARQLVNHRHILVNG
RIVDIPSYRCKPQDIITTRDEQRSRALIQNYMDSSPHEELAKHLSLYSSQYKGLVNQIID
IKWIGLKINELLVVEYYSRQT
>Q06GZ9 | RR14_DRIGR
MARKSLIQRERKRQKLEQKYHLIRRSSKKEISKVSSLSDKWEIHGKLQSPPRNSAPIRLH
RRCFSTGRSRGNYRDFGLSGHILRERFHACLLPGATRSSW
>Q06H06 | RPOC1_DRIGR
MIDRYKHQQLRIGSVSPQQISAWANKILPNGEMVGEVTKPYTFHYKTNKPEKDGLFCERI
SGPIKSGICACGNYRVIGDEKEDPKFCEQCGVEFVDSRIRRYQMGYIKLACPVTHVWYFK
RLPSYIANLLDKPLKELESLVYCDFSFARPIVKKPTFLRLRGSFEYEIQSRKYSIPLFFT
TQGFDTFRNREISTGASAIREQLADPDLRIITDHSLVEWKELGEEGSAGNEWEDRKIGRR
KDFLVRRMELAKHFIRTNVEPERMVLCLLPVLPPELRPIIQIDGGKPMSSDINELYRRVI
YRNNTLTDPLTTSRSTPGESVMCQEKLVQEAVDTLLDNGIRGQPMRNGHNKVYKSFSDVI
EGKEGRFRETLLGKRVDYSGRSVIVVGPSLSLHRCGLPREIAIELFQTFVIRGLIRQHIA
SNIGIAKNKIREKEPIVWEILQEVMQGHPILLNRAPTLHRLGIQAFQPILAEGRAICLHP
LVRKGFNADFDGDQMAVHVPLSLEAQAEARLLMFSHMNLLSPAIGDPISIPTQDMLIGLY
VLTIGNRRGICANRYNPWNRRNYQNKTVYEYNNNYRYTKEKEPYFCSSYDALGAYRQKRI
NLDSPLWLRWRLDQRVIAPREVPIEVQYESLGTYHEIYGHYLLVRSVKKESLCIYIRTTV
GHISFYREIEEAIQGFCRAYS
>Q06H07 | RPOC2_DRIGR
MEVLMAERADLVFHNKVIDGTAMKRLISRLIDHFGMAYTSHILDQVKTMGFQQATATSIS
LGIDDLLTIPSKGWLVQDAEQQSLILEKHHHYGNVHAVEKLRQSIEIWYATSEYLRQEMH
PNFRMTDPSNPVHIMSFSGARGNASQVHQLVGMRGLMSDPQGQMIDLPIQSNLREGLSLT
EYLISCYGARKGVVDTAVRTSDAGYLTRRLVEVVQHIVVRRTDCGTIRGISVSPRNGIGM
TEKMLIQTLIGRVLADDIYMGLRCIAARNQDIGVGLVNRFIAFRAQSIYIRTPFICRSTS
WICRLCYGRSPTHGDLVELGEAVGIIAGQSIGEPGTQLTLRTFHTGGVFTGGTAEHVRAP
FNGKIKFNEDLVHPTRTRHGHPAFLCYIDLYVTIESQDILHNVNIPPKSFLLVQNDQYVE
SEQVIAEIRARTSTFNFKERVRKHIYSDSEGEMHWSTDVYHAPEYTYGNVHLLPKTSHLW
ILSGGPRRSSLVPFSLHKDQDQMNIHSLSVEQRESSDLSVTNDRARHKLFSSDPSGKKEG

KILDYSGPARIISNGHWNFIYPAILHENSYLLAKRRRNRFIIPFQYDQEREKELMPRSGI
SIEIPINGILRRNSILAYFDDPRYRRSSSGITKYGTVEVDSIVKKEDLIEYRGAKEFSPK
YQMKVDRFFFIPEEVHILPGSSSIMVRNNSIIGVDTRITLNTRSRIGGLVRVERKKKRIE
LKIFSGDIHFPGEADKISRHSGILIPPGTGKKNSKESKKLQNWIYVQRITPTKKKYFVSV
RPVVTYEIADGINLATLFPQDLLQERDNVKFRVVNSILYRNGKPIRGIYYTSIQLVRTCL
VLNWDQDRNGSIEKVKASIVEVRANDLIRDFIRIDLVKSPISYTGKRNDMAGSGLIPDNG
SDHTNINPFYSKVRRIQSLTQHQGTIRTLLNRNKECQSFLILSSSNCSRIGPFNGSKSHN
VTKESIQIKEDPMIPIRNSLGPLGTVPKIANFDSPYYLITHNQILLNKYLLLDNLKQTFQ
VLKYYLMDENGRIYNPYPCRNIIFHPFDLTWCFLHHDYCEKTSTIIVLGQFICENENVCI
SKYGPQIKSGQVLIVHVDSLVIRSAKPHLATPGATVHGHYGEILYEGDTLVTFIYEKSRS
GDITQGLPKVEQVLEVRSVDSISMNLEKRVEGWNEHIKRILGIPWGFLIGAELTIAQSRI
SLVNKIQKVYRSQGVQIHNRHIEIIVRQITSKVLVSEDGMSNVFSPGELIGLLRAERTGR
SLEEAICYRAILWGITRASLNTQSFISEASFQETARVLAKAALRGRIDWLKGLKENVVLG
GMIPVGTGFKGLVHRSRQDNNIPLEIKKKNLFEGEIRDILFHHRELFGSCIPNNFHNTPE
Q
>Q06H14 | PSBK_DRIGR
MLNIFSLICICLNSALHSSSFFFAKLPEAYAFFNPIVDFMPVIPVLFFLLALVWQAAVSF
R
>Q06R66 | RK2_JASNU
MAIHLYKTSTPSTRNGTVDSQVKSNPRNNLIYGQHHCGKGRNARGVITTRHRGGGHKRLY
RQIDFRRNKKDIYGRIVTIEYDPNRNAYICLIHYGDGEKRYILHPRGALIGDTIVSGTKV
PIKMGNALPLTHMPLGTAIHNIEITLGKGGQLVRAAGAVAKLIAKEGKSATLKLPSGEVR
LISQNCSATVGQVGNVGAKHKSLGKAGSKRWLGKRPVVRGVVMNPVDHPHGGGEGRAPIG
RKKPTTPWGYPALGSRSRKRNKYSENLILRRRSK
>Q06R80 | NU4C_JASNU
MNHFPWLTIIVVLPIFAGSLIFFLPHRGNRVIFWYTICISILELLLTTYAFCYHFQSDDP
LIQLVEDYKWIDFFDFHWRLGIDGLSIGPILLTGFITTLATLAARPVTRDARLFHFLMLV
MYSGQIGLFSCRDLLLFFLMWEFELIPVYLLLSMWGGKKRLYSATKFILYTAGGSVFLLM
GALGIGLYGSNEPTFNLEILANQSYPVALERIFYIGFFIAFAVKLPIIPLHTWLPDTHGE
AHYSTCMLLAGILLKMGAYGLVRINMELLSHAHSLFSPWLMIVGAMQIIYAASTSLGQRN
LKKRIAYSSVSHMGFLIIGIGSITDIGLDGALLQIISHGFIGAALFFLAGTTYDRIRLVY
LDEMGGIAIPMPKIFTMFSIFSMASLALPGMSGFVTEFIVFFGLITSPKYLLMAKILIPF
VMAIGIILTPIYSLSMSRQMFYGYKIFNAPNSYFLDSGPRELFLSISIFLPILGIGLYPD
FVLSLSVDKVEVILSNSFDR
>Q06R91 | RR19_JASNU
MARSLKKNPFVVHHLVRKIDKLNKKKKGIIKTWSRASTIIPTMIGHTIAVYKGKEHLPI
YITDYMTGHKLGEFAPTLYFLRGHPTERPTKNDNRSQR
>Q06RA9 | RK20_JASNU
MTRIKRGSIARRRRTKAHSFASKFREAHARLTRAITQQQIRALFSADRDRDKQKIDFRRL
WITRINALIREKGVFHNYSKFINDLYKSQLLLNRKILAQIAISNRNCLYMIANEIIKKVG
FESAVII
>Q06RB1 | RK33_JASNU
MAKGKDAPRVRVILECTGCVRNAVSRGVSRYITQKNRHNTSKQLELRKFCPYCHKHMIHG
EIKINK

>Q06RC1 | RBL_JASNU
MSPQTETKASVGFKAGVKEYKLTYYTPEYETKDTDILAAFRVTPQPGVPPEEAGAAVAAE
SSTGTWTTVWTDGLTSLDRYKGRCYHIEPVPGETDQYICYVAYPLDLFEEGSVTNMFTSI
VGNVFGFKALRALRLEDLRIPPAYIKTFQGPPHGIQVERDKLNKYGRPLLGCTIKPKLGL
SAKNYGRAVYECLRGGLDFTKDDENVNSQPFMRWRDRFLFCAEAIYKSQAETGEIKGHYL
NATAGTSEEMIKRAVFARELGVPIVMHDYLTGGFTANTSLAHYCRDNGLLLHIHRAMHAV
IDRQKNHGIHFRVLAKALRMSGGDHIHSGTVVGKLEGERDITLGFVDLLRDDFIEKDRSR
GIYFTQDWVSLPGVLPVASGGIHVWHMPALTEIFGDDSVLQFGGGTLGHPWGNAPGAVAN
RVALEACVKARNEGRDLASEGNVIIREAAKWSPELSAACEVWKEIRFDFKAVDTLDPEK
>Q06RD3 | RR14_JASNU
MARKSLIQREKKRQQLEQKYHLIRRSSKKEISKIPSLSYKSEFYRKLQSPPRNSAPTRLH
RRCFSTGRARAHYRDFGLSGYILREMFHACLLPGATRSSW
>Q06RD9 | RPOB_JASNU
MLGDGNEGMSTIPGLNQIQFEGFCRFIDQGLTEELSKFPKIEDTDQEIEFQLFMERYQLV
EPLRKERDAVYESLTYSSEFYVSAGLIWKTSRDMQEQTILIGNIPLMNSLGTSIVKGIYR
IVINQILQSPGIYYRSELDHNEISVYTGTIISDWGGRSELEIDRKARIWARVSRKQKISI
LVLSSAMGSNLKEILDNVFYPEIFLSFLKDKERKKIGSKENAILEFYQQFACVGGDPVFS
ESLCKELQTKFFQQRCELGRIGRRNMNRRLNLDIPQNNTFLLPRDILAAADHLIEQKFGM
GTLDDMNHLKNKRIRSVADLLQDQLGLALVRLEDEVRETICGPIRHKWRTTPHPQNLVTS
TPLTTTYESFFGSHPLSQVLDQTNPLTQIVHGRKWSYLDPGGLTGRTASFRIRDIHPSHY
GRICPIDTSEGINVGLIGSLAIHAKIGHWGSLESPFYEISERSTGVQMLYLSPGRDEYYT
VAAGNSLALNQDIQEEEVVPARYRQEFLTIAWEQVHFRSILPFQYFSIGASLIPFIEHND
ANRALMSSNMQRQAVPLSRPEKCIVGTGLERQVALDSGALAIAEREGKIVSTDTEKILFS
GNGDTLSIPLVMYQSSNKNTCMHQKPQVQRGKCIKKGQILADGAATVGGELALGKNVLVA
YMPWEGYNSEDAVLISERLVYEDIYTSFHIRKYEIQTAGTERITNEIPHLEAHLLRNLDK
NGIVMLGSWVETGAILVGKLTPEVVKESPANRLVYDIVGIQVSTSKDTCLKLPIGGRGRV
IDVRWIQKRGDNDNPETIRIYISQKREIKVGDKVAGRHGNKGIISKILPRQDMPYLQDGR
PVDMVFNPLGVPSRMNVGQIFECSLGLAGGLLDRHYRIVPFDERYEQEASRKLVFSELYE
ASKQTTNPWVFEPEYPGKSRIFDGRSGNPFEQPILIGKPYILKLIHQVDDKIHGRSVGNY
THITQQPLRGRAKGGGQRVGEMEVWALEGFGVAHILQEMLTYKSDHIRARQKIPGTTMIG
GTIPNPEDAPESFRVLVRELRSLALELNHFLVSEKNFQINRKEA
>Q06RE8 | PSBK_JASNU
MLNIFSLISICLNSALYSSSFFFGKLPEAYAFLNPIVDVMPVIPVFFFLLAFVWQAAVSF
R
>Q06SD8 | RR14_STIHE
MAKKNMIQRELKRQKCVLKYETRRQLLKKQITLASTLREKLMLHRQLQKLPRNSSAVRLR
NRCMITGRSRGYHRDFGLSRNVLREMAHQGLLPGVVKSSW
>Q06SE3 | CHLN_STIHE
MSQLITSPKINEKETLKFECETGNYHTFCPISCVSWLYQKIEDSFFLVIGTKTCGYFLQN
ALGVMIFAEPRYAMAELEEADISAQLNDYKELKRLCLQIKRDRNPSVIVWIGTCTTEIIK
MDLEGMAPRLETEIGIPIVVARANGLDYAFTQGEDTVLAAMACRCPDKISTTSDSKNLET
PMMIDNTQKNGQNSKALAHPPLVLFGSLPSAVVNVLTLELNKQGITVDGWLPSSRYTDLP
ALGEDVYVCGVNPFLSRTAMTLMRRKKCKLINAPFPIGPDGTRAWIEKICNVLGVIPTGL
EEREKKIWQSLENYLPLVRGKSVFFMGDNLLEISLARFLTRCGMIVYECGVPYLDKRYQA

SELLLLEQTCLEKNVPMPRIVEKPDNYYQIQRIRELQPDLVITGMALSNPLEARGINTKW
SVEFTFAQIHGFANSKDVLELVTRPLRRNMMQQVSNKTLVKPAK
>Q06SF4 | RPOB2_STIHE
MSTSQSLNIIPIEYSLQTYQRSNQDTSMLHRPVVKEGEWVQAGDLLSDCASSIGGEFSIG
QNILIAYLPWEGYNYEDAILISERLVYDDLYTSIHIERYDISTEKNPYGIEKITKDIVLL
KDTTELNHLDKNGIAQLGAWLKEGDILVGKITPTESKKEVARYVQLYNDILGKKINYAIR
DSSLRVPRGLEAKVIRVKTFPERKNETKNWENKIGLKSKALAFSVQHFLSKNQSNKKRTL
SLKSKWEKETSLSSFSSLAWQKKSPLFFVFKNGIFEKLKNFPKKKKKNERVFALKKKSSK
KFRFFELQKKFRFFFRNFFFFKAPKKKSPLFFFFKSETFDKERNFQKMSPNQSKQNWVKI
SIKKEWQKVGSFGKKKASKGKISFFPSFPQASLVGKASFFSPSRFEKKRDASNFLKSSCI
SSVHIYLAEKRKVQVGDKMAGRHGNKGIISQILPRQDMPYLPDGTPIDMALNPLGVPSRM
NVGQIYECLLGLAGKHLGEQYRIQPFDEAFGPEASRSFVFSKLYSAKTKTGQSWLFQPTN
PGKLKLFDGRTGNCFDQAITTGYSYMIKLVHLVDEKIHCLTVDHEVLTTKGWIPLNKVKT
SHFVATLKKNGQLVYQNPTNIYHYPEFKGELYHIKNVNLDLLVTLNHRMYVKNGIIEATS
SVDYQLIPAKDIVGQHKKYCKTAFWDKENYQFILPSVISNSIVIPEKTMNMEAWLQFFGI
WIAEGWALTNTISNNNVTNFNQSSSSPYVVQISIKKKKVLEILNNVIPILGYSFNYYDNN
ITICDKQLWAYLRPLSLGNPYRKLPIWVWDLSQDQARVLLLAMITVFKNGTNSKWEKAAS
LSSRLSLASVFPEFLKKRIDKGLSYYTSSVELADDISRLALHAGWSGNNYLLKKKGSISS
FDGKQIICQFDIWRISIIQSKNQPAVNHGYHSKGKEEVLPYQGAVYCLSVPNEIFYVRRN
GLSVWTGNSRSSGPYSLITQQPLKGRSKHGGQRLGEMEVWAIEAYGAAFTLLELLTIKSD
DVTGRLTIWDYVLYKKPLYIGTPASFKVLICELQALCLDIGIYKADKSNILKQINVSSMG

>Q06SF9 | RR18_STIHE
MGTSNTQKPQKQVPKRKKYKNLFSLKTKKKRGKKFSKRKKGGTLTLPVIPPKSIFILLKS
RNKKIYDRKIIDYKNRSLLQEYIYFTGKIIPKRKTGITTKQQRYLTKAIKTARILGLLPF
VKKEKGFFR
>Q06SG4 | RR12_STIHE
MPTIQQLVKSARQKLVNKTKAPALKSCPQRRGICLRVYTVTPKKPNSALRKVARVRLSSG
FEVTAYIPGIGHNLQEHAVVLVRGGRVKDLPGVRYHIVRGTLDTAGVKGRVQGRSKYGVK
KVSAKSAGKSK
>Q06SG6 | RK5_STIHE
MTQRLSKLYFEQISPKLIEKFSYKNMHQVPRVEKIVINRGVGEAAQSNKVLESSLKELNL
ISGQKGIITRSKKAIAGFKIREKVPVGVFVTLRGDRMYSFLDRLINLALPRIRDFQGISA
QSFDKYGNYSLGLEEQLMFPEIEYDKIDQLRGMDISIVTNARNSEEGLALLKEFGLPFKS

>Q06SG9 | RK16_STIHE
MLSPKRTKYRKYHRGRMKGKALRGSKIVYGGFALQATEPCWITSRQIEAGRRVLTRFVKR
GGKLWIRIFPDKPVTMRAAGSRMGSGKGAPAYWVAVVKPGRIIYEMKGVSDKIATRALKI
AGYKMPVKTKVLKPL
>Q06SI6 | RK20_STIHE
MTRVKRGIVARKRRKKILNYTKGFRGAASKLFRTANQRYMKALKLSFVNRRKKKRDFRSL
WISRLNAAVRKNGLNYNEFIFALKICNINLNRKTLSQISICDPQTFNLLYDTLKPLLIKH
LNTK
>Q08183 | RBS3_MESCR

MASSLMSNAATTMAAATTTAQANMVAPFNGLKSVSAFPVTRKNNDITSVASNGGRVQCMQ
VWPPLGKKKFETLSYLPPLSEESLMKEVQYLLNNGWVPCLEFEPTHGFVYREHGNTPGYY
DGRYWTMWKLPMFGCTDPSQVVAELEEAKKAYPEAFIRIIGFDNVRQVQCISFIAYKPAS
YDA
>Q08935 | ROC1_NICSY
MASSASSLHFLSLTPQTLPLPKPTSQTTSLSFFSLPPSSLNLSLSSSSSCFSSRFVRKVT
LSDFDQIEDVEDGDDGVEEERNFSPDLKIFVGNLPFSADSAALAELFERAGNVEMVEVIY
DKLTGRSRGFGFVTMSSKEEVEAACQQFNGYELDGRALRVNSGPPPEKRENSSFRGGSRG
GGSFDSSNRVYVGNLAWGVDQDALETLFSEQGKVVDAKVVYDRDSGRSRGFGFVTYSSAE
EVNNAIESLDGVDLNGRAIRVSPAEARPPRRQF
>Q09FP6 | RK2_NANDO
MAIHLYKTSTPSTRNGTVGSQVKSNPRNNLIYGQRRCGKGRNARGIITARHRGGGHKRLY
RKIDFRRNEKDISGRIVTIEYDPNRNAYICLIHYGDGEKRYILHPRGAIIGDTIVSGTEV
PISMGNALPLTDMPLGTAIHNIEITLGKGGQLARAAGAVAKLIAKEGKSATLKLPSGEVR
LISKNCSATVGQVGNVGVNQKSLGRAGSKRWLGKRPVVRGVVMNPVDHPHGGGEGRAPIG
RKKPTTPWGYPALGRRSRKRNKYSDSLILRRRSK
>Q09FR2 | RK32_NANDO
MAVPKKRTSISKKRIRKNIWKLKGHWAAKKAISLAKSISTGNSKSFFVQQTSNKVLE
>Q09FS5 | RR8_NANDO
MGRDTIADIITSIRNADMDKKGTVRIASTNITETIVKILLREGFIENVRKHRENDQFFLV
STLRHRRNRKGPYRTILKRISRPGLRIYSNYQRIPRILGGMGIVILSTSRGIMTDREARL
EGIGGEILCYIW
>Q09FS9 | RPOA_NANDO
MLREEVAVSTRTLQWRCVESRTDSKRLYYGRFVLSPLMKGQADTIGIAMRRALLGEIEGT
CITHAKSEKIPHEYSTIVGIEESVHEILMNLKEIVLRSNLYGTCDASICVKGPINITAKD
IISPPSVEIVDTTQHIASLTEPFDLCIGLQIERNRGYRMKTPNKAQNGSYPIDAVFMPVR
NANHSIHSYGNGNEKQEILFLEIWTNGSLTPKEALHEASRNLIDLFIPFLHAEEQDIILE
DNQNRVTLPFFTFHDGLAKLKKKNEIALKFIFIDQSELPSRTYNCLKKSNINTLLDLLNK
SQEDLLKIEHFRIEDVKRVLGILQKRFVIDLPKNKFSN
>Q09G10 | RR8_PLAOC
MGRDTIADIITSIRNADMDKKGRVRIGYTNIAENIIKILLREGFIENVRKHRESNKYFLV
STLRHRRNRKRPYKTILKRISRPGLRIYSNYQRIPRILGGIGIVILSTSRGIMTDREARL
EGIGGEILCYIW
>Q09G22 | RR12_PLAOC
MPTIKQLIRNTRQPVRNVTKSPALRGCPQRRGTCTRVYTITPKKPNSALRKVARVRLTSG
FEITAYIPGIGHNLQEHSVVLVRGGRVKDLPGVRYHIVRGTLDAVGVKDRQQGRSKYGVK
KPK
>Q09G54 | RPOB_PLAOC
MRRDGNEGMSTIPGFNQIQFEGFCRFIDQGLTEELYKFPKIEDTDQEIEFQLFVETYQLV
EPLIKERDAVYESLTYSSEVYVPAGLIWKPGRDMQEQTIFIGNIPLMNSLGTSIVNGIYR
IVINQILQSPGIYYRSESDHNGISVYTGTIISDWGGRSELEIDRKARIWARVSRKQKISI
LVPSSAMGSNLREILDNVCYPEIFLSFLNDKEKKKIGSKENAILEFYQQFACVGGDPVFS
ESLCKELQKKFFQQRCELGRIGRRNMNRRLNLDIPQNNTFLLPRDVLAAADHLIGMKFGM
GTLDDMNHLKNKRIRSVADLLQDQFGLALIRLENVVRGTICGAIKHKLIPTPQNLVTSTP

LTTTYESFFGLHPLSQVLDRTNPLTQMVHGRKLSYLGPGGLTGRTASFRIRDIHSSHYGR
ICPIDTSEGINVGLIGSLAIYARIGHWGSLESPFYEISDRSKEVQMLYLSPSRDEYYMVA
AGNSLALNQSIPEEQVVPARYRQEFLTIAWEQVHFRSIFPFQYFSIGASLIPFIEHNDAN
RALMSSNMQRQAVPLSQSEKCIVGTGLERQAALDSGVSTIAEHEGKIIYTDTDKIILLGN
GDTLSIPLVMYQRSNKNTCMYQKPQVRRGKFIKKGQIVADGAATVGGELALGKNVLVAYM
PWEGYNSEDAVLISERLVYGDIYTSFHIRKYEIQTHVTSQGPERITNEIPHLEAHLLRNL
DRNGIVMLGSWVETGDILVGKLTPQMAKESSYAPEDRLLRAILGIQVSTSKETCLKLPIG
GRGRVIDVRWIQKKGGSSYNPETICVYISQKREIKVGDKVAGRHGNKGIISKILPRQDMP
YLQDGTPIDMVFNPLGVPSRMNVGQIFECSLGLAGDLLDRHYRIAPFDERYEQEASRKLV
FSELYKASKQTANPWVFEPEYPGKSRIFDGRTGDPFEQPVLIGKSYILKLIHQVDDKIHG
RSSGHYALVTQQPLRGRAKQGGQRVGEMEVWALEGFGVAHILQEMLTYKSDHIRARQEVL
GTTIVGGTIPSPEDAPESFRLLVRELRSLALELNHFLVSEKNFQINRKEA
>Q09MB1 | RR19_CITSI
MTRSLKKNPFVANHLLRKIDKLNTKAEKEIIITWSRASTIIPTMVGHTIAIHNGREHLPI
YITDRMVGHKLGEFAPTINFRGHAKNDNRSRR
>Q09MB2 | RK2_CITSI
MAIHLYKTSTPSTRNGAVDSQVKSNPRNNLIYGQHRCGKGRNARGIITAGHRGGGHKRLY
RKIDFRRNEKDIYGRIVTIEYDPNRNAYICLIHYGDGEKRYILHPRGAIIGDTIVSGTEV
PIKMGNALPLTDMPLGTAIHNIEITLGKGGQLARAAGAVAKLIAKEGKSATLKLPSGEVR
LISKNCSATVGQVGNVGANQKSLGRAGSKCWLGKRPVVRGVVMNPVDHPHGGGEGRAPIG
RKRPATPWGYPALGRRSRKRNKYSDNLILRRRTK
>Q09MB9 | RR15_CITSI
MVKNAFISVRVQEKKEKNSGSVEFQVFRFTNTIRRLTSHLELHRKDYSSQRGLRKILEKR
QRLLAYLSKKNRVRYKELISKLNIRESKNR
>Q09MC6 | NU4C_CITSI
MNYFPWLTLIVVFPIAAGSLIFFLPHRGNKVTRGYTIYICVLELLLTTYAFCYNFQLDDP
LIQLAEDYKWITFFEFDWRLGIDGLSIGPILLTGFITTLATLAARPVTLNSRLFHFLMLA
MYSGQIGSFSSRDLLLFFIMWELELIPVYLLLSVWGGKKRLYSATKFILYTAGGSVFLLI
GVLGIGLYGSNEPTLNLETLVNRSYPVALEIIFYIGFFIAFAVKLPIIPFHTWLPDTHGE
AHYSTCMLLAGILLKMGAYGLIRINMELLPHAHSIFSPWFMIVGTVQIIYAASTSPGQRN
LKKRIAYSSVSHMGFIILGIGSITDTGLNGAVLQIISHGLISAALFFLAGTSYDRIRFAY
LDEMGGLAIPIPKIFTLFSILSMASLALPGMSGFVAELIVFFGIITSQKFFLMPKIVITF
VMAIGMILTPIYSLSMLRQMFYGYKLFNAPNSYFFDSGPRELFVLISILLPVIGIGIYPD
FVLSLSADKAEAIISNFFL
>Q09ME0 | RK16_CITSI
MLSNPKRTRFRKQHRGRMKGISYRGNHICFGRYALQALEPAWITSRQIEAGRRAMTRNVR
RGGKIWVRIFPDKPVTLRPTETRMGSGKGSPEYWVAVVKPGRILYEMSGVAENIARKAIS
IAASKMPIRTQFIISG
>Q09ME2 | RR8_CITSI
MGKDTIADIITSIRNADMNRKGTVRIASTNITENVVKILLREGFIENARKLVENKNKKEF
LVLTLRHRRNRKGPYRPILNLKRISRPGLRIYFNYRRIPRILDGMGIVILSTSRGIMTDR
AARLERIGGEILCYIW
>Q09ME5 | RPOA_CITSI
MVREKVKVSTRTLQWKCVESRADSKRLYYGRFILSPLMKGQADTIGIAMRRVLLGEIEGT

CITRAKSEKIPHEYSTIVGIQESVHEILMNLKDIVLRSNLYGTCDALICVKGPGYVTAQD
ILLPPSVEIVDNTQHIASLTEPIDLSIGLQIERSRGYNIKTPNTFQDGNCYPIDAVFMPV
RNANHSIQSYGNGNEKQEILFLEIWTNGSLTPKEALHEASRSLIDLFIPFLQAEDENLPL
ENNQYKVTLPFFTFHDRLAKLTKKKKEIALKSIFIDQSEMSPRIYNCLKKSNIHTLFDLL
NTRQEDLMKIEHFRIEDVKQIMSILEKK
>Q09MF3 | RR12_CITSI
MPTIKQLIRNPRQPIRNVTKSPALRGCPQRRGTCTRVYTITPKKPNSALRKVARVRLTSG
FEITAYIPGIGHNSQEHSVVLVRGGRVKDLPGVRYHIVRGTLDAVGVKDRQQGRSKYGVK
KPK
>Q09MF6 | RR18_CITSI
MDKTKRLFLKSKRSFRRRLPPIQSGDRIDYRNMTLISRFLSEQGKILSRRVNRLTLKEQR
LITIAIKQARILSSLPFLNNEKQFERSGLTARPPGLRTRKK
>Q09MF7 | RK33_CITSI
MAKGKEVRVRVILECTSCVRNGVNKESRGISRYITQKNRHNTPSRLELRKFCPYCYKHTL
HGEIKK
>Q09MI7 | RPOC1_CITSI
MIDRYKHQQLRIGSVSPQQIRAWANKILPNGEIIGEVTKPYTFHYKTNKPEKDGLFCERI
FGPIKSGICACGNYRIIGDEKEDPQFCEQCGVEFVDSRIRRYQMGYIKLGCPVTHVWYLK
RLPSYIANLLDKPLKELEGLVYCDFSFARPIAKKPTFLRLRGSFEYEIQSWKYSIPLFFT
TQGFDKFRNREISTGAVAIREQLADLDLRIILDNSLLEWKELGEEGPAGNDWEDRKIGRR
RDFLVRRMELAKHFLRTNIEPEWMVLCLLPVLPPELRPIIQIDGGKLMSSDINELYRRVI
YRNNTLIDLLTTSRSTPGELVMCQEKLVQEAVDTLLDNGIRGQPMRDGHNKIYKSFSDVI
EGKEGRFRETLLGKRVDYSGRSVIVVGPSLSLHQCGLPREIAIELFQTFVICGLIRQHLA
SNIGVAKSKIREKGPIIWEILQEVMQGHPVLLNRAPTLHRLGVQAFQPILVEGRAICLHP
LVCKGFNADFDGDQMAVHVPLSLEAQAEARLLMFSHMNLLSPTIGDPISIPTQDMLIGLY
VLTSGNRRGICANRYNTWNRRNYPDERIDDNSYTYTKEPLFCNSYDAIGAYRQKRINLDS
PLWLRWRLDQRLIASREAPIEVHYESLGTSHEIYGHYLIVRSVKKEILSIYIRTTVGHIS
LYREIEEAIQGFCRACSYGT
>Q09MI8 | RPOC2_CITSI
MAERVSLVFHNKMIDGTAIKRLISRLIDHFGMAYTSHILDQVKTLGFQQATATSISLGID
DLLTIPSKRWLVQDAEQQSFILEKHYHYGNVHAIEKLRQSIEIWYATSEYLRQEMNPNFR
MTEPFNPVHIMSFSGARGNASQVHQLVGMRGLMSDPQGQMIDLPIQSNLREGLSLTEYII
SCYGARKGVVDTAVRTSDAGYLTRRLVEVVQHIIVRRTDCGTVRGISVSPQNQNRMMSER
VFSQTLIGRVLADDIYMGQRSIAIRNQDIGIGLVNRLITLRTQAISIRTPFTCRSTSWIC
RLCYGRSPTHGDLVELGEAVGIIAGQSIGEPGTQLTLRTFHTGGVFTGGTAEHVRAPSNG
KIKFNEDLVHPTRTRHGHPAFLCSIDLDVIIEGEDIMHNVTIPPKSFLLVQNDQYVESEQ
VIAEIRAGAYTLNFKERVRKHIYSDSEGEMHWSTDVYHAPEFTYSNVHLLPKTSHLWILS
GGSRRSSVVSFSLHKDQDQMNIHSLSVERREISSLSLSVNNDQTGHKFFSSDFSAKKKGR
ILEMSDYSGFNRIISTGHCNLIHPAILHANSDLLAKRQRNGFLIPFHSIQEQEKELMPHS
GISIEIPVRGIFRRNSILAYFDDPRYRRKSSGITKWGTLGAHSIVKKEDLIEYRGLKKIK
PKYQMKIDRLFFIPEEVHIFPESSYLMVRNNSIIGVDTQITLNIRSRVGGLVRMERKRGG
VELKIFSGDIHFPGEIDKISRHSGILIPPESEKTKLKEATKESKKLKKWIYVQRITPTKK
KSFVLVRPVVTYEIANGINLATLFPQDPLREKDNMQFRVVNYVLYGKGKAAGGISDTSLQ
LVRTCLVLNWDQDNKSSSVEEVCASFVEVRTNGLIRNFLRINLVKSNISYLRKRDHPSGS

GLISDNGSARTNSNPFYSVFGKAGVEQSLRQNQGTIRTLLNKNKECQVLIIILSSSNYFRM
GPLNDVKYHNVIKQSIPIQKDSLTPIKTSLGPLGTFLQIANFYSFYYLITHNHISVTKYL
KLDNLKQPFQVLKYYLMDETGEIYNPDTDSKILLNPFNLNWYFLQPNYCEEMSPIISLGQ
FLCENVRITKRGPYLKSGQVLIVQVNSVVIRSAKPYLATPGATVHGHYGAILYEGDTLVT
FIYEKSRSGDITQGLPKVEQVLEVRSLDSISMNLEKRVEGWNASITRILGIPWGFLIGAE
LTIVQSRISLVNKIQKVYRSQGVQIHNRHIEIIVRQITSKVLVSEDGMSNIFLPGELIGL
LRAERMGRALEEVICYRAILLGITRASLNTQSFISEASFQETTRVLAKAALRGRIDWLKG
LKENVVLGGIIPVGTGFKGLVHCSRQHNNILLERQKGNLFGGEMRDIFLHHRELFDSCIS
TTFHDTSEHNCLEGLMSPRSEFF
>Q09MJ5 | PSBK_CITSI
MLNIFSLMYICLNSALYSSSFLFAKLPEAYAFLNPIVDVMPVIPVLFFLLAFVWQAAVSF
R
>Q09WV5 | RK2_MORIN
MAIHLYKTSTPSTRKGAVDRQVKSNPRNNLIYGQHRCGKGRNARGIITAGHRGGGHKRLY
RKIDFRRNEKDIYGKIVTIEYDPNRNAYICLIHYGGGEKRYILHPRGALIGDTIVSGTEV
PIKMGNALPLTDMPLGTAIHNIEITLGKGGQLARAAGAVAKLIAKEGKSATLKLPSGEVR
LIPKNCSATVGQVGNVGVNQKSLGRAGSKCWLGKRPVVRGVVMNPVDHPHGGGEGRAPIG
RKKPATPWGYPALGRRSRKRNKYSDNLILRRRSK
>Q09WV9 | RR7_MORIN
MSRRGTAEEKTAKSDPIYRNRLVNMLVNRILKHGKKSLAYQIIYRAMKKIQQKTETNPLS
VLRQAIRGVTPDIAVKARRVGGSTHQVPIEIGSTQGKALAIRWLLGASRKRPGRNMVFKL
SSELVDAAKGSGDAIRKKEETHRMAEANRAFAHFR
>Q09WW1 | RR15_MORIN
MIKKSFISVISHEEKEKNRGSVTFQILSLTNRIRKLSSHLELHRKDYLSQRGLRKILGKR
QRMLSYLSKNNRIRYKELINQLDIRESKTR
>Q09WX0 | RK32_MORIN
MAVPKKRTSISKKRIRKNIWKKKGYWAALKAFSLGKSLSTRNSKSFFYPTNK
>Q09WX8 | RK22_MORIN
MIKKRRTNRYTEVYAIAQYISMSAHKARRVIDQIRGRSYAETLIIELMPYRACYPILKL
VYSAAANANHNMRFNEANLIISKAEVNEGNTVKKLKPRARGRGYPIKRPTCHITIVLKDL
DVEEEYLDSFAFEDKYIHKK
>Q09WY0 | RK16_MORIN
MLSPKRTRFRKHHRGRMKGISYRGNHICFGRYALQALEPAWITSRQIEAGRRAMTRNARR
GGKIWVRIFPDKPITVRPTETRMGSGKGSPEYWVAVVKPGRILYEMSGVPENIARKAIAI
AASKMPIRTQFIISG
>Q09WY1 | RK14_MORIN
MIQPQTHLNVADNSGARKLMCIRIIGASNRRYAKIGDVVVAVIKEAIPNTPLERSEVIRA
VIVRTCKELKRDNGMIIRYDDNAAVVIDQERNPKGTRIFGAIARELRQLNFTKIVSLAPE
VL
>Q09WY5 | RPOA_MORIN
MGQEKVTVSTRPLQWKCVESRADSKRLYYGRFLLAPLMRGQADTIGIAMRRALLGEIEGT
CITRAKSEKIPHEYSTIVGIQESVHEILMNLKEIVLRSNLYGTRDASICVKGPGYVTAQD
IILPPSVEIVDNTQHIANLTEPITLCIELQIERNRGYRIKTPNNFQNGSYPIDAVFMPVR
NANHSIHSYVNGNENQEILFLEIWTNGSLTPKEALHEASRNLIDLFIPFFHAEEHLENNQ

HKGTLPLFNFHGRLAKPRKTKKEIALKYIYIDQSELPPRVYNCLKRSNINTFLELLNNSQ
EELMKIQDFRIEDVKHILDVLEI
>Q09WZ3 | RR12_MORIN
MPTIKQLIRNTRQPIKNVTKSPALRGCPQRRGTCTRVYTITPKKPNSALRKVARVRLTSG
FEITAYIPGIGHNLQEHSVVLVRGGRVKDLPGVRYHIVRGTLDAVGVKDRQQGRSKYGVK
KPK
>Q09WZ5 | RR18_MORIN
MDKSKRLFLKSKRSFRRRLPPIQSGDRIDYRNMSLISRFISEQGKILSRRVNRLTLKQQR
LITIAIKQARILCLLPFLNNEKQFERSESTPRTTGLRIKNK
>Q09WZ6 | RK33_MORIN
MAKGKDARVTIILECTSCLRNRVNKESRGISRYITQKNRHNTPSRLELRKFCPSCYKHTI
HGELKK
>Q09X09 | RBL_MORIN
MSPQTETKASVGFKAGVKDYKLTYYTPEYEVKDTDILAAFRVTPQPGVPPEEAGAAVAAE
SSTGTWTTVWTDGLTSLDRYKGRCYNIEPVAGEESQFIAYVAYPLDLFEEGSVTNMFTSI
VGNVFGFKALRALRLEDLRIPNAYIKTFQGPPHGIQVERDKLNKYGRPLLGCTIKPKLGL
SAKNYGRAVYECLRGGLDFTKDDENVNSQPFMRWRDRFLFCAEAIYKSQAETGEIKGHYL
NATAGTCEEMMKRAVFARELGVPIVMHDYLTGGFTANTTLAHYCRDNGLLLHIHRAMHAV
IDRQKNHGMHFRVLAKALRMSGGDHIHAGTVVGKLEGEREITLGFVDLLRDDFIEKDRSR
GIYFTQDWVSLPGVLPVASGGIHVWHMPALTEIFGDDSVLQFGGGTLGHPWGNAPGAVAN
RVALEACVKARNEGRDLAVEGNEIIREASKWSPELAAACEVWKEIKFEFEAMDTL
>Q09X19 | RR14_MORIN
MARKSLIQREKKRQKLEQKYHLIRRSSKKEISKVPSLNEKWKIHGKLQSLPRNSAPIRLH
RRCFSTGRPRANYRDFGLSGHILREMVQACLLPGATRSSW
>Q09X25 | RPOB_MORIN
MLEGGNEEISTIPGFNQIQFEGFCRFIDQGLTEELYKFPKIEDTDQEIEFQLFVETYQLV
EPLIKERDAVYESLTYSSELYVSAGLIWKTSRDMQEQTIFIGNIPLMNSLGTSIVNGIYR
IVINQILQSPGIYYRSELDHNGISVYTGTIISDWGGRLELEIDRKARIWARVSRKQKISI
LVLLSAMGLNLREILENVCYPEIFLSFLKDKEKKKIKSKENAILEFYQQFACVGGDPVFS
ESLCKELQKKFFQQRCELGRIGRRNMNRRLNLDIPQNTTFLLPRDILAAADHLIGMKFGM
GILDDMNHLKNKRIRSVADLLQDQFGLALVRLENMVRGTMCGAIRHKLIPTPQNLVTSTT
LTTTYESFFGLHPLSQVLDRTNPLTQIVHGRKASYLGPGGLTGRTASFRIRDIHPSHYGR
ICPIDTSEGINVGLIGSLAIHARIGHWGSLESPFYEISERSKKVRMLYLSPSIDEYYMVA
AGNSLALNRGSQEEQVVPARYRQEFLTIEWEQVHLRSIFPFQYFSIGASLIPFIEHNDAN
RALMSSNMQRQAVPLSRSEKCIVGTGLECQVALDSGVPTIAEHQGKIIYTDTEKIILSGN
RDTLSIPLVIYQRSNKNTCMHQKPQVSRGKCIKKGQILADGAATVGGELALGKNVLVAYM
PWEGYNSEDAVLINERLVYEDIYTSFHIRKYEIHTHVTSHGPERITNEIPHLEAHLLRNL
DKNGIVMLGSWVETGDILVGKLTPQMAKESSYAPEDRLLRAILGIQISISKETCLKLPIG
GRGRVIDVRWIQKKGGSSYNPETIRVYISQKREIKVGDKVAGRHGNKGIVSKILPRQDMP
YLQDGRPVDMVFNPLGVPSRMNVGQIFECSLGLAGGLLDRHYRIAPFDERYEQEASRKLV
FSELYEASKQTANPWVFEPEYPGKSRIFDGRTGDPFEQPVIIGKPYILKLIHQVDDKIHG
RCSGHYALVTQQPLRGRAKQGGQRVGEMEVWALEGFGVAHILQEMLTYKSDHIRARQEVL
GTTMVGGPIPKPEDAPESFRLLVRELRSLALELNHFLVSEKNFQINRKDV
>Q09X26 | RPOC1_MORIN

MIDRYKHQQLRIGSVSPQQISAWAKKILPNGEIIGEVTKPYTFHYKTNKPEKDGLFCERI
FGPIKSGICACGNYRVIGNDKEDPKFCEQCGVEFVDSRIRRYQMGYIKLACPVTHVWYLK
RLPSYIANLLDKPLKELEGLVYCDFSFARPIAKKPTFLRLRGSFEYEIQSWKYSIPLFFT
TQGFDAFRNREISTGAGAIREQLADLDLPIIIDYSLVEWKELGEERPTVNEWEDRKVGRR
KDFLVRRMELAKHFIRTNIEPEWMVLCLLPVLPPELRPIIQIDGGKLMSSDINELYRRVI
YRNNTLIDLLTTSRSTPGELVMCQEKLVQEAVDTLFDNGIRGQPMRDGHNKVYKSFSDII
EGKEGRFRETLLGKRVDYSGRSVIVVGPSLSLHRCGLPREIAIELFQTFVIRGLIRQHFA
SNIGVAKSKIREKEPVVWEILQDVMQGHPVLLNRAPTLHRLGIQAFQPILVEGHAICLHP
LVCKGFNADFDGDQMAVHVPLSLEAQAEARLLMFSHTNLLSPAIGDPISVPTQDMLIGLY
VLTSGNRRGICANRYNPCNYRNYQNERIDDNKYKYTKTQEKEPFFCNSYDAIGAYRQKRI
HLDSPLWLRWRLDQRVITSREAPIEVHYQSLGTYHEIYGHLLIVRSIKKEILCIYLRTTV
GHISLYREIEEAIQGFFRACSYGT
>Q0DDE3 | SSY23_ORYSJ
MSSAVVASSTTFLVALASSASRGGPRRGRVVGVAAPPALLYDGRAGRLALRAPPPPRPRP
RRRDAGVVRRADDGENEAAVERAGEDDDEEEEFSSGAWQPPRSRRGGVGKVLKRRGTVPP
VGRYGSGGDAARVRGAAAPAPAPTQDAASSKNGALLSGRDDDTPASRNGSVVTGADKPAA
ATPPVTITKLPAPDSPVILPSVDKPQPEFVIPDATAPAPPPPGSNPRSSAPLPKPDNSEF
AEDKSAKVVESAPKPKATRSSPIPAVEEETWDFKKYFDLNEPDAAEDGDDDDDWADSDAS
DSEIDQDDDSGPLAGENVMNVIVVAAECSPWCKTGGLGDVAGALPKALARRGHRVMVVVP
RYGDYAEAQDVGIRKYYKAAGQDLEVKYFHAFIDGVDFVFIDAPLFRHRQDDIYGGNRQE
IMKRMILFCKAAVEVPWHVPCGGVPYGDGNLVFLANDWHTALLPVYLKAYYRDNGMMQYT
RSVLVIHNIAYQGRGPVDEFPYMELPEHYLDHFKLYDPVGGEHANIFGAGLKMADRVVTV
SPGYLWELKTTEGGWGLHDIIRENDWKMNGIVNGIDYREWNPEVDVHLQSDGYANYTVAS
LDSSKPRCKAALQRELGLEVRDDVPLIGFIGRLDGQKGVDIIGDAMPWIAGQDVQLVLLG
SGRRDLEVMLQRFEAQHNSKVRGWVGFSVKMAHRITAGADVLVMPSRFEPCGLNQLYAMA
YGTVPVVHAVGGLRDTMSAFDPFEDTGLGWTFDRAEPHKLIEALGHCLETYRKYKESWRG
LQVRGMSQDLSWDHAAELYEEVLVKAKYQW
>Q0DEC8 | SSY1_ORYSJ
MATAAGMGIGAACLVAPQVRPGRRLRLQRVRRRCVAELSRDGGSAQRPLAPAPLVKQPVL
PTFLVPTSTPPAPTQSPAPAPTPPPLPDSGVGEIEPDLEGLTEDSIDKTIFVASEQESEI
MDVKEQAQAKVTRSVVFVTGEASPYAKSGGLGDVCGSLPIALALRGHRVMVVMPRYMNGA
LNKNFANAFYTEKHIKIPCFGGEHEVTFFHEYRDSVDWVFVDHPSYHRPGNLYGDNFGAF
GDNQFRYTLLCYAACEAPLILELGGYIYGQKCMFVVNDWHASLVPVLLAAKYRPYGVYRD
ARSVLVIHNLAHQGVEPASTYPDLGLPPEWYGALEWVFPEWARRHALDKGEAVNFLKGAV
VTADRIVTVSQGYSWEVTTAEGGQGLNELLSSRKSVLNGIVNGIDINDWNPSTDKFLPYH
YSVDDLSGKAKCKAELQKELGLPIRPDVPLIGFIGRLDYQKGIDLIKLAIPDLMRDNIQF
VMLGSGDPGFEGWMRSTESGYRDKFRGWVGFSVPVSHRITAGCDILLMPSRFEPCGLNQL
YAMQYGTVPVVHGTGGLRDTVENFNPFAEKGEQGTGWAFSPLTIEKMLWALRMAISTYRE
HKSSWEGLMKRGMSSDFTWDHAASQYEQIFEWAFMDQPYVM
>Q0DUI8 | CRTI_ORYSJ
MDTGCLSSMNITGTSQARSFAGQLPTHRCFASSSIQALKSSQHVSFGVKSLVLRNKGKRF
RRRLGALQVVCQDFPRPPLENTINFLEAGQLSSFFRNSEQPTKPLQVVIAGAGLAGLSTA
KYLADAGHKPILLEARDVLGGKIAAWKDEDGDWYETGLHIFFGAYPNIQNLFGELGINDR
LQWKEHSMIFAMPNKPGEFSRFDFPETLPAPLNGIWAILRNNEMLTWPEKVKFALGLLPA

MVGGQAYVEAQDGFTVSEWMKKQGVPDRVNDEVFIAMSKALNFINPDELSMQCILIALNR
FLQEKHGSKMAFLDGNPPERLCMPIVDHVRSLGGEVRLNSRIQKIELNPDGTVKHFALTD
GTQITGDAYVFATPVDILKLLVPQEWKEISYFKKLEKLVGVPVINVHIWFDRKLKNTYDH
LLFSRSSLLSVYADMSVTCKEYYDPNRSMLELVFAPAEEWVGRSDTEIIEATMQELAKLF
PDEIAADQSKAKILKYHVVKTPRSVYKTIPDCEPCRPLQRSPIEGFYLAGDYTKQKYLAS
MEGAVLSGKLCAQSVVEDYKMLSRRSLKSLQSEVPVAS
>Q0G9G9 | NU4C_LIRTU
MSYFPWLTIIVVLPIFAGSSIFFFPHRGNKVVRWYTICICLLELLLTTYAFCYHFQLDDP
LIQLEEDYKWINIFDFHWRLGIDGLSIGPILLTGFITTLATLAARPVTRDSRLFHFLMLA
MYSGQIGSFSSRDLLLFFIMWELELIPVYLLLSMWGGKKRLYSATKFILYTAGGSIFLLM
GVPGMGLYGSNEPILNFETSANQSYPLALEIIFYFGFLIAYAVKSPIIPLHTWLPDTHGE
AHYSTCMLLAGILLKMGAYGLVRINMELLPHAHSIFSPWLMIVGTIQIIYAASTSSGQSN
LKKRIAYSSVSHMGFTIIGIGSITDTGLNGAILQIISHGFIGAALFFLAGTSYDRIRLVY
LDEMGGIAIPMPKIFTMFSSFSMASLALPGMSGFVAESVVFWGIITSPKYLLMPKILITF
VMAIGMILTPIYSLSMSRQMFYGYKLFNVPNSYFLDSGPRELFVSICIFLPVIGIGIYPD
FVLSLSVDKVEAILANYFYK
>Q0G9H1 | RK32_LIRTU
MAVPKKRTSMSKKRIRRNIWKKKGYWAAVKALSLAKSISTGHSKSFFVRQTSNKALE
>Q0G9H9 | RR19_LIRTU
MTRSLKKNPFVANNFLGKIEKLNMREEKEIIITWSRASTIIPTMIGHTIAIHNGKDHLPI
YITDRMVGHKLGEFAPTLTFKGHARNDNRSRR
>Q0G9I3 | RR8_LIRTU
MGRDTIADILTSIRNADMDKKGTVRIASTNMAESVVKILLREGFIENVRKHRENNKYFLV
STLRHRRNRKGTYRNILKRISRPGLRIYSNYQRIPRILGGMGIVILSTSRGIMTDREARL
EGIGGEVLCYIW
>Q0G9I4 | IF1C_LIRTU
MKEQKLIHEGLITESLPNGMFRVRLDNEDLILGYVSGRIRRSFIRILPGDRVKIEVSRYD
STRGRIIYRLRNKDSND
>Q0G9I7 | RPOA_LIRTU
MVREEVAVSTRTLQWKCVESRTDSKRLYYGRFILAPLMKGQADTIGIAMRRALLGEIEGT
CITRVKSEKVPNEYYTIVGIEESVHEILMNLKEIVLRSHLYGTRDASICVRGPRYVTAQD
IIPPPSVEIVDTTQHIASLTEPIDLCIELQIERDRGYRMKTPNNYQDGSYPIDAVSMPVR
NANHSIHSYGNRNEKQEILFLEIWTNGSLTPKEALHEASRNLIDLFIPFLHAEEQDIVLE
DNPNRFTVPLFTFHDRLANIRKNKKGIALKCIFIDQSELPPRTYNCLKRSNIHTLLDLLS
NSQEDLMRIEHFRIEDVKQILDILQKHFTIDLPKNKF
>Q0G9M1 | RR14_LIRTU
MARKSLIQRERKRQKLEQKYHLIRRSSKKEISKVSSLSDKWEIHGKLQSPPRNSAPTRLH
RRCFSTGRPRANYRDFGLSGHILRERVHACLLPGATRSSW
>Q0G9M9 | RPOC2_LIRTU
MEVLMAERADLVFHNKVIDATAMKRLISRLIDHFGMAYTSHILDQVKTLGFQQATATSIS
LGIDDLLTIPSKGWLVQDAEQQSLILEKHHHYGNVHAVEKLRQSIEIWYATSEYLRQEMH
PNFRMTDPSNPVHIMSFSGARGNASQVHQLVGMRGLMSDPQGQMIDLPIQSNLREGLSLT
EYIISCYGARKGVVDTAVRTSDAGYLTRRLVEVVQHIVVRRTDCGTIRGISVSPRNGMTE
KILIQTLIGRVLADDIYMGLRCIATRNQDIGIGLVNRFITFRAQSIYIRTPFICRSTSWI

CRLCYGRSPTHGDLVELGEAVGIIAGQSIGEPGTQLTLRTFHTGGVFTGGTAEHVRAPSN
GKIKFNECLVHPTRTRHGHPAFLCYIDLYVTIESQDIIHNVNIPPKSFLLVQNDQYVESE
QVIAEIRAGTSTFNFKERVRKHIYSDSEGEMHWSTDVYHAPEYRYGNVHLLPKTSHLWIL
SGGPCRSSIVPFSLHKDQDQMNVHSLSVERRYISDLSVTNDRVRHKLFSSDPSGKKKERI
LDYSGPDRIVSNGHWNFLYPAILHENSDLLAKRRRNRFIIPFQYDQEREKELMPRSGISI
EIPINGILRRDTILAYFDDPRYRRSSSGITKYGTIEVDSIVKKEDLIEYRGAKEFRPKYQ
MKVDRFFFIPEEVHILPGSSPIMVRNNSIIGVDTRIALNTRSRVGGLVRVERKKKKIELK
IFSGDIHFPGETDKISRHSGILIPPGTGKKNSKESKKWKNWIYVQRITPTKKKYFVSVRP
VVTYEIADGINLGTLFPQDLLQERDNVQLRVVNYILYGNGKPIRGIYHTSIQLVRTCLVL
NWDQDRNGSIEEVHASFVEVGTNDLIRDFIRIDLVKSPISYIGKRDDTTGSGLIPDNESD
RTNINTFYSKTRIQSLTQHQGTIRTFLNRNKECQSFLILSSSDCSRIGPFNGSKSHKVTK
ESIKEDPMIPIRNSLGPLGTVPKIANFYSSYYLITHNQILLNKYLLLDNLKQTFQVLKYY
LMDENGRIYNPNLHSNIIFNPFDLNWCFLRHDYCEETSTIISLGQFICENVCISKYGPHI
KSGQVLIVHVDSLVIRSAKPHLATPGATVHGHYGEILSEGDTLVTFIYEKSRSGDITQGL
PKVEQVLEVRSIDSISMNLEKRIEGWNEHITRILGIPWGFLIGAELTIAQSRISLVNKIQ
KVYRSQGVQIHNRHIEIIVRQITSKVLVSEDGMSNVFSPGELIGLLRAERTGRALEEGIC
YRAILLGITRASLNTQSFISEASFQETARVLAKAALRGRIDWLKGLKENVVLGGMIPVGT
GFKGLVHRSRQHNNIPLEIKKKNLFEGEMRDILFHHRELLSSCIPKNFHDTSEQSFTGFN
DS
>Q0G9P9 | RK2_DAUCA
MAIHLYKTSTPSTRNRTVDSRVKSNPRNNLIYGQHRCGKGRNARGIITAGHRGGGHKRLY
RKIDFRRNEKDIYGRIVTIEYDPNRNAYICLIHYGDGEKRYILHPRGAIIGDTIVSGTEV
PIKMGNALPLTDMPLGTAIHNIEITRGRGGQLARAAGAVAKLIAKEGKSATLKLPSGEVR
LISKNCSATVGQVGNVGVNQKSLGRAGSKRWLGKRPVVRGVVMNPVDHPHGGGEGRAPIG
RKKPTTPWGYPALGRRSRKRNKYSDNLILRRRSK
>Q0G9Q0 | RK23_DAUCA
MDGIKYAVFTDKSIRLLGKNQYTSNVESGSTRTEIKHWVELFFGVKVIAMNSHRLPGKGR
RMGPIMGQTMHYRRMIITLQPGYSIPPLRKKRT
>Q0G9R4 | RK32_DAUCA
MAVPKKRTSISKKRIRKNIWKGKGSWAALKALSLGKSLSTGNSKSFFVRQKKNKS
>Q0G9S2 | RK22_DAUCA
MLKKLTKIKTEVYALGQHISMSAHKARRVVDQIRGRSYEETLMILELMPYRACYPILKLV
YSAAANANYNMDSNESNLVISKAEVSEGTATKKLKPRARGRSFTIKRPTCHIAIVVKDIS
LDEYQYLGVDFIDSFRCSKKLQSKKKYTAMSYHDMYTNGGIWDKK
>Q0G9S4 | RK16_DAUCA
MLSPKRTRFRKQHRGRMKGISYRGNHISFGKYALQALEPAWITSRQIEAGRRAMTRNARR
GGKIWVRIFPDKPVTVRPAETRMGSGKGSPEYWVAVVKPGRILYEMGGVTENIARRAISI
ASSKMPIRTQFIISG
>Q0G9S6 | RR8_DAUCA
MGRDIIADIITSIRNADMDRKRAVRIASTNITENIVKILLREGFIENVRKHKETKKDFLV
LTLRHRRNRKRSYRNFLNLKRISRPGLRIYSNYQRIPRILGGMGIVIISTSRGIMTDREA
RLERIGGEVLCYIW
>Q0G9S9 | RR11_DAUCA
MAKTIPRIGSRKNGRIGSRKNTRRIPKGVIHVQASFNNTIVTVTDVRGRVVSWSSAGTCG

FKGTRRGTPFAAQTAAGNAIRTVVDQGMQRAEVMIKGPGLGRDAALRAIRRSGILLTFVR
DVTPMPHNGCRPPKKRRV
>Q0G9U0 | RR18_DAUCA
MDKSKRPFLKSKRSFRRRLPPIQSGDRIDYRNMSLISRFISEQGKILSRRVNRLTLKQQR
LITIAIKQARILSLLPFLNNEKQFERTESTTRTAGLRARNK
>Q0G9U1 | RK33_DAUCA
MAKGKDVRITVILECTGCVRNGVNKVSTGISRYITEKNRHNTPNRLELRKFCPFCYKHTM
HGEIKK
>Q0G9W0 | RR4_DAUCA
MSRYRGPRFKKIRRLGALPGLTNKRPRAGSDLRNQSRSGKKSQYRIRLEEKQKLRFHYGL
TERQLLKYVRIAGKAKGSTGQVLLQLLEMRLDNILFRLGMATTIPGARQLVNHRHILVNG
RIVDIPSYRCKPRDIITARDEQNSRALIQNSFNSPSQDEMPKHLTLQPFQYKGLVNQIID
SKWVGLKINELLVVEYYSRQT
>Q0P3J3 | RBL_OSTTA
MAPQTETKTGTGFQAGVKDYRLTYYTPDYQVKETDILAAFRMTPQPGVPAEECGAAVAAE
SSTGTWTTVWTDGLTQLDRYKGRCYDLEPVPGEDNQFIAYVAYPLDLFEEGSVTNLFTSI
VGNVFGFKALRALRLEDLRIPVAYCKTFQGAPHGIQTERDKLNKYGRGLLGCTIKPKLGL
SAKNYGRAVYECLRGGLDFTKDDENVNSQPFMRWRDRFLFVAEAIYKSQAETGEIKGHYL
NATAGNVDQMLKRAQVAKELGMPIIMHDYLTAGFTANTTLATYCREEGLLLHIHRAMHAV
IDRQRNHGIHFRVLAKALRLSGGDHLHSGTVVGKLEGERNVTLGFVDLMRDAYVEKDRDR
GIYFSQDWASLPGVMPVASGGIHVWHMPALVEIFGDDACLQFGGGTLGHPWGNAPGASAN
RVALEACTQARNEGRDLAREGGDVIRAACKWSPELAAACEVWKEIKFEFDTVDTL
>Q0P3L1 | RR11_OSTTA
MATKQRKVTQKKSRRKVQRGVVHILATFNNTLVTISDRSGLVIASSSAGACGFRGARKGT
PFAAQTASEEAVRKALDYGLKYVDVMVKGPGAGREMAIRALQQLGLGVTLIRDVTPLPHN
GCRPPKKRRV
>Q0P3L3 | RR8_OSTTA
MVNDTISDMLTRLRNAYLADKEQTSLKATRVVKDIAQVLVQEGFLGPIEQEENGFFTVNL
KRGVKQFERVSTPGVRVYANHKQLQPVLNGMGVAVISTSQGIMTDRKARALGIGGEVLCK
IW
>Q0P3L4 | RK5_OSTTA
MIQRLQNLYLTKVQTTLTEQFQYKNTHEIPRLEKIVINRGVGSASQNTKLLESLADELTI
LAGQRPIVKRAKKAIAGFGVREDMPIGLTVTLRGERMYAFYDRLVNLALPRIRDFQGISP
KNFDGHGNYTLGLTEQLMFPEVSYEQIDQVCGMDISIVTTASTDREGHGLLKELGMPFKA
GSVN
>Q0P3L6 | RK16_OSTTA
MLSPKRTKYRKPHRGNRKGQALRGNKISFGDFALQALEPGWITSRQIEAGRRAMSRYAKR
GGKLWIRMFPDRSITARAAETRMGAGKGAPDYWVCIVKPGKILYELAGVPETIARASMRI
AAYKMPVKTKFLVRDEYVAPQ
>Q0P3L8 | RR19_OSTTA
MARSLKKGPFVADKLMKKIELLHSKGEKTVIQTWSRSSTITPIMIGHTIAVHNGREHIPV
FVTDQMVGHKLGEFSPTRTFRGHVKSDKKSKR
>Q0P3L9 | RK2_OSTTA
MALRFYKAYTPGTRNRSVADCSGLTKTRPEKSLTRSMHRAKGRNNRGVITCRHRGGGHKR

LYRQIDFRRNKYDMAATVKTIEYDPNRNARIALVEYQDGEKRYILHPNGLEIGDSIIASE
TAVNAVGNSLPLMAMPLGVQVHNIEMHPKKGGQLVRSAGAVAQLVAKEGEYVTLRLPSGE
VRLISNKCWATIGQVGNMEAMNLSLGKAGRSRWLGRRPTVRGSVMNAVDHPHGGGEGRCP
VGHAQPRTPWGKPALGVKTRTRKKYSDTLILRRRKVS
>Q0P3M5 | RPOC1_OSTTA
MSIEYVQLQLASPSEIKRWSQRILPTGEVVGEISKADTINYRTFKPERGGLFCERIFGST
SNGECSCGKTKRRKLIVPVNFNRLTLQKRNAELEATQAIIEVCPSCGVEPTNSKVRRYRM
GCISLKKPVAHMWYFRNSPNVLAALLNMRSQEIDETIHFQTYTPSKYGTQNYCLHGGVQW
YVNHWEPMHPYFAGDIDVSMDKAASWNSKSTFMPSQIKTIENCGGEALQELLTRLDLVFL
QRMLSRKLKNTIEKKKLAAKSLRKRHKRRKTAKLLGRTDQKNYGITIKVREYSRRLEFIR
SLARKGLRPEWMILSIFPVLPPDLRPMIQMSSGRFATSDLNDLYRRLIYRKIRFEKFLTL
FDEEFLPDLLIRHDLCLLQEAADSIIDNGRLDKPAQRPNRKPFKSLTSIIEGKHGRFRQN
LLGKRVDYSGRSVIVVGPKLRLHQCGLPREMALELFQPFVIRALLEESGVKNIRAAKNLL
QRRSQMVWDILDTVVLGHPVLLNRAPTLHRLGIQAFEPILLSGRAIQLHPLVCPAFNADF
DGDQMAVHVPLGLEAQAEARLLMLATHNWLSPATGEPSILPSQDMILGFYYLTTLKPTVS
LKRPVEQPMAVRGRNLIQSNSIFNNFESVLHAYETSKVDLHEIIWLRWSSYIQTTNSEPL
QITVNKTGHVTTVYDSFVMQSGATNGPDNSMLSVQMKSRDTSSISKVYNCLTSLNAAAFY
ILTTPGRVLFNNLIYENLFL
>Q0P3M9 | RR12_OSTTA
MPTIQQLIRSQRIKCETKTKSPALKACPQRRGICTRVYTTTPKKPNSALRKVARVRLTTG
IEVTAYIPGIGHNLQEHSVVLVRGGRVKDLPGVRYHIVRGSLDTAGVKDRLQSRSKYGVK
RPKS
>Q0P3N0 | RR18_OSTTA
MNFTRQRTSPIHAGEIISYKNVDLLRRFITEQGKILPRRLTGVTAKEQRQLAKAIKQARI
LGFLQFLYKE
>Q0P3P4 | CLPP_OSTTA
MPIGVPKVAYRLPGESTPQWVDLYNRLYRERVLFLGSGLDDELANQLNGIMLYLSAEDAS
RSLFLYINSPGGSVTAGLSVFDIMNYVQASVTTIGIGFAASMASFILAGGERGSRIALPH
CRVMIHQPQGGMEGQASEVVLEKEEIVRLRRLIGRLYVDLTGQPLSTIANDLDRDKYLSA
REAREYGLVDLVATTETATV
>Q0PUX0 | RK23_PRUPE
MDGIKYAVFTDKSIRLLGKNQYTSNVESGSTRTEIKHWFELFFGVKVIAMNSHRLPGRGR
RMGPIMGHTMHYRRMIITLQPGYSIPPLRKKRT
>Q0ZIV5 | RK23_VITVI
MDGIKYAVFTDKSIRLLGKNQYTSNVESGSTRTEIKHWVELFFGVKVIAMNSHRLPGKGR
RMGPIMGHTMHYRRMIITLQPGYSIPPLRKKRT
>Q0ZIY6 | RR11_VITVI
MAKPIPRIGSRRNGRIGSRKSARRIPKGVIHVQASFNNTIVTVTDVRGRVVSWSSAGTCG
FRGTRRGTPFAAQTAAGNAIRTVVDQGMQRAEVMIKGPGLGRDAALRAIRRSGILLSFVR
DVTPMPHNGCRPPKKRRV
>Q0ZIY7 | RPOA_VITVI
MVREKVTVSTRTLQWKCVESRVDNKRLCYGRFILSPLIKGQADTIGIAMRRALLGEIEGT
CITRAKSEKIPHEYSTIVGIQESVHEILMNLKEIVLRSNLYGTRGAFICARGPGYVTAQD
IISPPSVEIVDNTQHIARLMEPIDLCIGLQIERNRGYSIKRPNNFQDGSYPIDAVFMPVR

NANHSIHSYGNGNERQEILFLEIWTNGSLTPKEALHEASRNLIDLFIPFLHAEEETLHLE
YNQHKSTLPPLTFHDRLAKLRKNKKEIELKYIFIDQLELPPRIYNCLKRSNIHTLLDLLN
KSQKDLMKIEDFRIEDVKQLLGILEKHFTID
>Q0ZIZ4 | CLPP_VITVI
MPIGVPKVPFRNPGEDDISWIDVYNRLYRERLLFLGQEVESEISNQLIGLMIYLSIEDEN
KDLYFFINSPGGWVLPGIAIYDTMQFVPPEVHTICLGLAASMGSFILVGGTITKRLAFPH
ARVMIHQPAAAFYEAQAGEFVMEAEELLKLREIITKVYVQRTGKPLWVVSEDLERDVFMS
ATEAQTHGIVDLVAVQ
>Q0ZIZ5 | RR12_VITVI
MPTIKQLIRNTRQPIRNVTKSPALRGCPQRRGTCTRVYTITPKKPNSALRKVARVRLTSG
FEITAYIPGIGHNSQEHSVVLVRGGRVKDLPGVRYHIVRGTLDAVGVKDRQQGRSKYGVK
KPK
>Q0ZIZ8 | RR18_VITVI
MDKSKRPFLKSKRSFRRRLPPIQSGDRIDYRNMSLISRFISEQGKILSRRVNRLTLKQQR
LITIAIKQARILSSLPFLNNEKQFERTESTARTTGLRTRNK
>Q0ZJ22 | RR14_VITVI
MARKSLIQRERKRQKLEQKYHLIRRSSKKEISKVPSLSDKWEIHGKLQSSPRNSAPTRLH
RRCFLTGRPGANYRYFGLSGHILREMVHACLLPGATRSSW
>Q0ZJ28 | RPOB_VITVI
MFRGGNEGMSTIPGFNQIQFEGFCRFIDQGLTEELYKFPKIEDTDQEIEFQLFVETYQLV
EPLIKERDAVYESLTYSSELYVSAGLIWKTSRDMQEQTIFIGNIPLMNSLGTSIVNGLYR
IVINQILQSPGIYYRSELDHNGISVYTGTIVSDWGGRLELEIDRKARIWARVSRKQKISI
LVLSSAMGSNLREILENVCYPEIFLSFLNDKEKKKIGSKESAILEFYQQFACVGGDPVFS
ESLCKELQKKFFQQRCELGRIGRRNINRRLNLDIPQNNTFLLPQDILAAADHLIGMKFGM
GILDDMNHLKNKRIRSVADLLQDQFGLALIRLENVVRGTICGAIRHKLIPTPQNLVTSTP
VTTTYESFFGLHPLSQVLDRTNPLTQIVHGRKSSYLGPGGLTGRTASFRIRDIHPSHYGR
ICPIDTSEGINVGLIGSLAIHARIGHWGSLESPFYEISERSKKVRMLYLSPSRDEYYMVA
AGNSLALNQGIQEEQVVPARYRQEFLTIAWEQVHLRSIFPFQYFSIGASLIPFIEHNDAN
RALMSSNMQRQAVPLSRSEKCIVGTGLERQAALDSGVPVIAEHEGKIVYTDTDKIILSGN
GDTLSIPLVMYQRSNKNTCMHQKAQVRRGKCIKKGQILADGAATVGGELALGKNVLVAYM
PWEGYNSEDAVLISERLVYGDIYTSFHIRKYEIQTHVTSQGPERITNKIPHLEAHLLRNL
DKNGIVILGSWVETGDILVGKLTPQMAKESSYAPEDRLLRAILGIQVSTSKETCLKLPIG
GRGRVIDVRWIQKKGGSSYNPETIRVYISQKREIKVGDKVAGRHGNKGIISKILPRQDMP
YLQDGRPVDMVFNPLGVPSRMNVGQIFECSLGLAGGLLDRHYRIAPFDEKYEQEASRKLV
FSELYEASKQTANPWVFEPEYPGKSRIFDGRTGDPFEQPVIIGKPYILKLIHQVDDKIHG
RSSGHYALVTQQPLRGRAKQGGQRVGEMEVWALEGFGVAHILQEMLTYKSDHIRARQEVL
GTTIIGGTIPNPEDAPESFRLLVRELRSLALELNHFLVSEKNFQINRKEA
>Q14F95 | RK2A_POPAL
MAIHLYKTSTPSTRNGAVDSQVKSNTRNNLIYGQHRCSKGRNARGIITARHRGGGHKRLY
RKIDFRRNEKYIYGRIVTIEYDPNRNAYICLIHYGDGEKRYILHPRGAIIGDTIISGTEV
PIKMGNALPLSTDMPLGTAIHNIEITLGRGGQLARAAGAVAKLIAKEGKSATLKLPSGEV
RLISKNCSATVGQVGNAGVNQKSLGRAGSKCWLGKRPVVRGVVMNPVDHPHGGGEGRAPI
GRKKPATPWGYPALGRRSRKRNKYSDNLILRRRSK
>Q14FA1 | RR15_POPAL

MVKSSFISIISQEDKKENKGSVEFQIVSFTNKIRRLTSHLELHRKDYLSQRGLRKILGKR
QRLLSYLAKKNGVRYKELISQLNIRESKTR
>Q14FA8 | NU4C_POPAL
MNSFPWLTIFVGLPISAGFLIFVFPHRGNKVIRWYTIFICVLELLLMTYAFSYYFQLDDP
LIQLTESYKWINFFDFYWRLGIDGLSLGPILLTGFITTLATLAARPITRDSRLFHFLMLA
MYSGQIGLFSSQNLLLFFIMWELELIPVYLLLAMWGGKQRLYSATKFILYTAGSSVFLLM
GALGIAFYASNEPTFNFETSANQSYPVTLEILFYIGFLIAFAVKSPIIPLHTWLPDTHGE
AHYSTCMLLAGILLKMGAYGLVRINMELLSHAHSIFSSWLIIVGAIQIIYAASTSSGQKN
LKKRIAYSSVSHMGFTIIGIYSISDMGLNGAILQIISHGFIGAALFFLSGTSYDRIRLVY
LDEMGGMATLMPKIFMMFSILSLASLALPGMSGFFAELVVFFGIITAKKFFLMPKILITF
VTAVGTILTPIYLLSMLRQMFYGYKLFNATNSYFFDSGPRELFVSIAILLPVISIGIYPD
FVFSLSVDKVEAILSNYFYS
>Q14FB1 | RR12_POPAL
MPTIKQLIRNTRQPIRNVTKSPALGGCPQRRGTCTRVYTITPKKPNSALRKVARVRLTSG
FEITAYIPGIGHNSQEHSVVLVRGGRVKDLPGVRYHIVRGTLDAVGVKDRQQGRSKYGVK
KPK
>Q14FB6 | RK2B_POPAL
MAIHLYKTSTPSTRNGAVDSQVKSNTRNNLIYGQHRCSKGRNARGIITARHRGGGHKRLY
RKIDFRRNEKYIYGRIVTIEYDPNRNAYICLIHYGDGEKRYILHPRGAIIGDTIISGTEV
PIKMGNALPLTDMPLGTAIHNIEITLGRGGQLARAAGAVAKLIAKEGKSATLKLPSGEVR
LISKNCSATVGQVGNAGVNQKSLGRAGSKCWLGKRPVVRGVVMNPVDHPHGGGEGRAPIG
RKKPATPWGYPALGRRSRKRNKYSDNLILRRRSK
>Q14FC4 | RR11_POPAL
MAKLLPRIGSRKNGRISSRKNARKIPKGVIHVQASFNNTIVTVTDVRGRVISWSSAGACG
FRGTRRGTPFAAQTAAGNAIRTVVDQGMQRAEVMIKGPGLGRDAALRAIRRSGILLSFVR
DVTPMPHNGCRPPKKRRV
>Q14FD2 | CLPP_POPAL
MPIGVPKVPFRNPGEDSSNWIDVYNRLYRERLLFLGQDIDSEISNQLIGLMVYLSTESEI
KDLYLFINSPGGWVIPGIAIYDTMQFVRPDVQTVCMGLAASMGSFILVGGKITKRLAFPH
ARVMIHQPFAAFYEAQIGEFVLEAEELLKLREILTRVYAQRTGKPLWVVSEDMERDVFMS
AAEAQVHGIVDLVAVA
>Q14FE9 | RBL_POPAL
MSPQTETKTGVGFKAGVKEYKLNYYTPEYETKDTDILAAFRVTPQPGVPPEEAGAAVAAE
SSTGTWTTVWTDGLTSLDRYKGRCYDIEPVAGEENQFIAYVAYPLDLFEEGSVTNMFTSI
VGNVFGFKALRALRLEDLRIPPAYTKTFQGPPHGIQVERDKLNKYGRPLLGCTIKPKLGL
SAKNYGRAVYECLRGGLDFTKDDENVNSQPFMRWRDRFLFCAEALYKAQTETGEIKGHYL
NATAGTCEEMMKRAIFARELGVPIVMHDYLTGGFTANTSLAHYCRDNGLLLHIHRAMHAV
IDRQKNHGIHFRVLAKALRMSGGDHIHSGTVVGKLEGERDITLGFVDLLRDDFVEKDRSR
GIYFTQDWVSLPGVLPVASGGIHVWHMPALTEIFGDDSVLQFGGGTLGHPWGNAPGAVAN
RVALEACVQARNEGRDLAREGNEIIREASKWSPELAAACEVWKEIKFEFEAMDTL
>Q14FF5 | RR4_POPAL
MSRYRGPRFKKIRRLGALPGLTSKRPRAGSDLRNQSRAGKKSQYRIRLEEKQKLRFHYGL
TERQLLKYVRIAAKAKGSTGQVLLQLLEMRLDNILFRLGMASTIPRARQLVNHRHILVNG
RIVDIPSYRCKPRDIITAKDEQKSRVMIQNSLDSFPQEELPKHLTLHPFQYKGLVNHIID

SKWIGLKINELLVVEYYSRQT
>Q14FG5 | RPOB_POPAL
MLGDGNGGMSTIPGFNQIQFEGFCRFIDQGLAEELYKFPKIEDRDQEIEFQLFVETYQLV
EPSIKERDAVYESLTYSSELYVSGGLIWKNSRDMQEQTIFIGNIPLMNSLGTSIVNGIYR
IVINQILQSPGIYYRSELNHNGISVYTGTIISDWGGRVELEIDKKARIWARVSRKQKISI
LVLSSAMGLNLREILENVCYPEIFLSFLSDKEKKKIGSRENAILEFYQQFTCVGGGPVFS
ESLCKELQKKFFQQRCELGRIGRLNMNQRLNLDIPHNNTFLLPRDILAAADHLIGMKFGM
GTLDDMNHLKNKRIRSVADLLQDQFGLALIRLENVVRGTICGAIRHKLIPTPQNLVTSTP
LTTTYESFFGLHPLSQVLDRTNPLTQIVHGRKSSYLGPGGLTGRTASFRIRDIHPSHYGR
ICPIDTSEGINVGLIGSLTIHAKIGHLGSLESPFYEISARSKKVRMLYLSPNRDEYYMIA
AGNCLALNRGAREEQVVPARYRQEFLTIAWEQVRLRSFFPFQYFSIGASLIPFIEHNDAN
RALMSSNMQRQAVPLARSEKCIVGTGLERQVALDSGVPAIAEHEGKIIYTDIDKIILSGN
GYTVSIPLVMYQRSNKNTCMHQKTQVQRGKCIKRGQVLADGAATVGGELALGKNILVAYM
PWEGYNFEDAVLISERLVYEDVYTSFHIRKYEIQTHVTSQGPERITNEIPHLEAHLLRNL
DKNGIVMLGSWVETGDILIGKLTPQLAKESSYAPEDRLLRAILGIQVSTSKETCLKLPTG
GRGRVIDVRWIQKKGGSSYNPETIRVYILQKREIKVGDKVAGRHGNKGIISKILPRQDMP
YLQDGGPVDMVFNPLGVPSRMNVGQIFECSLGLAGSLLARHYRVAPFDERYEQEASRKLV
FSELYEAGKQTANPWVFEPECPGKSRIFDGRTGDPFEQPVIIGKPYILKLIHQVADKIHG
RSSGHYALVTQQPLRGRAKQGGQRVGEMEVWALEGFGVSHILQEMLTYKSDHIRARQEVL
GTTISGRTIPKPEDAPESFRLLVRELRSLALELKHFLISEKNFQINRKEV
>Q14FG6 | RPOC1_POPAL
MIDRYKHQQLRIGSVSPQQISAWTNKILPNGEIVGEVTKPYTFHYKTNKPEKDGLFCERI
FGPIKSGICACGNYRVIGDEKEEPKFCEQCGVEFVDSRIRRYQMGYIKLACPVTHVWYLK
RLPSYIANLLDKPLKQLEGLVYCDFSFARPIAKKPTFLRLRGSFEYEIQSWKYSIPLFLT
TQGFDTFRNREISTGAGAIRELLADLDLRIILDYSSLEWKELGEEGPTGNEWEDRKVGRR
KDFLVRRVELAKHFIRTNIEPEWMVLCLLPVLPPELRPIIQIDGGKLMSSDINELYRRVI
YRNNTLTDLLTTSRSTPGELVMCQEKLVQEAVDTLLDNGIRGQPMRDGHNKVYKSFSDVI
EGKEGRFRETMLGKRVDYSGRSVIVVGPSLSLHRCGLPREIAIELFQTFVIRGLIRQHLA
SNIGVAKSKIREKEPIVWGILQEVMRGHPILLNRAPTLHRLGIQAFQPILVEGRAICLHP
LVRKGFNADFDGDQMAVHVPLSLEAQAEARLLMFSHMNLLSPAIGDPISVPTQDMLMGLY
VLTSGNRRGICANRYNPFNCRNFQNEKIDANANKDKYIKEPFFCNSYDAIGAYRQKRINL
ESPLWLRWQLDQGLIASREAPIEGHFGSLGTYHEIYGHYLIVRNIKKKILSIYIRTTVGH
IYLYREIEEAIQGFCQACSDGT
>Q14FH4 | PSBK_POPAL
MLNILNLICICLNFALYSSSFFFTKLPEAYAFLNPIVDVMPVIPLFFFLLAFVWQAAVSF
R
>Q19V92 | CHLB_CHLAT
MKLAYWMYAGPAHIGTLRVASSFKNVHAIMHAPLGDDYFNVMRSMLERERDFTPVTASIV
DRHVLARGSQNKVVENITRKDKEERPDLIVLTPTCTSSILQEDLQNFVDRASMDSESDVI
LADVNHYRVNELQAADRTLEQVVRFYIEKSKKQGDLNLTKTEKPSANILGIFTLGFHNQH
DCRELKRLLQELGIEINEVIPEGGSVNNLKNLPRAWFNLVPYREVGLMTAIYLEKEFGMP
YVSTTPMGVVDTATCIREIEKILNSFDKDVVVDFESYIDKQTRFVSQAAWFSRSIDCQNL
TGKKAVVFGDATHAASMTKILAREMGINVACAGTYCKHDADWFKEQVQGYCDEVLITDDH
TEVGDLIARIEPSAIFGTQMERHIGKRLNIPCGVISAPVHIQNFPLGYRPFLGYEGTNQI

ADLVYNSFTLGMEDHLLEIFGGHDTKEVITKSLSTEEGLTWTSDAQAELSKIPGFVRGKI
KRNTEKFARENNISEINIEVMYAAKESLNA
>Q19VA6 | RR4_CHLAT
MSRYRGPRLKIVRRLGDLPGFTNKIQKRQASTQNKGKKTAKKRLSQYSIRLQEKQKLRYN
YGVTERQLLLYVRKARRIKGSTGQILLQQLEMRLDNIIYRLGMAPTIANARQLVNHGHIV
VNDRIVTIPSYRCKPKDIISVRNNSTSRNVITNNLSFSDSRLPPHLSFKKESLVALVNGL
VDRQWIPLNINELLVVEYYSRMA
>Q19VB3 | RR8_CHLAT
MVNDTVADMITRIRNANLVRQTNVQVIASNTTNSIASILKDEGFVEKIQTIETDSGLSSL
LITLKYQGKRRKPYITALKRISKPGLRVYANSREIPRVLGGIGIAIISTSKGIMTDRRAR
HEKVGGEILCYVW
>Q19VB6 | RR11_CHLAT
MAKKIRKIGIRKGKRKIPKGVVHVQATFNNTIVTITDIKGEVISWSSAGSCGFKGTKKGT
PFAAQTAAENAVRQAIEQGMKEAEITVSGPGSGRETAIRAIRTAGLGITVLKDVTPIPHN
GCRPPKKRRV
>Q19VC3 | CLPP_CHLAT
MPIGIPKVAYRIPGESVSTYVDVYNRLYRERILFLGEDLDDEVANQLIGVMVFLNSEDDT
KGIFFYINSPGGSMNSGLGVYDMIQHINVDVTTICMGLAASMASFILAGGTPGQRLMFPH
ARVMLHQPMGGNGGKAKYMVEESVEVKRLRELIAHLYAKRTGQPIERIRIDMNRDNFMRP
RAAKEYGLIDHMITHITELDELEKDASDLRRFGGVDLTKLNKQGS
>Q1ACE0 | CHLL_CHAVU
MKIAVYGKGGIGKSTTSCNISIALARRGKKVLQIGCDPKHDSTFTLTGFLIPTIIDTLQS
KDYHYEDVWPEDVIYKGYGEVNCVEAGGPPAGAGCGGYVVGETVKLLKELNAFYEYDVIL
FDVLGDVVCGGFAAPLNYADYCIIITDNGFDALFAANRIAASVREKARTHPLRLAGLVGN
RTSKRDLIDKYVEACPMPVLEVLPLIEDIRVSRVKGKTLFEMAETDSSLEYVCDYYLNIA
DQILSQPEGIVPKEIPDRELFTLLSDFYLNININSNNLEKNESSFLII
>Q1ACF1 | RK32_CHAVU
MAVPKKRTSKSKTKIRKTTWKKEACESAIKAFSLAKSILSQRSKSFYYSKKNIILPSSFE
SQSKEEE
>Q1ACF2 | RK21_CHAVU
MNTNTYAIIQMGGQQLQVQTGRFYDTCDFSILKPDTKIIIYKVTMIHLNSQLVIGKPWLH
NAMIKGRILHSYRDNKVTIYRMHSKKKMRRKQGHRQNITRFIVDGIFLDGKNI
>Q1ACF5 | RK23_CHAVU
MDKLKKPILTEKSISLLEKRQYTFELDKNITKSEAKKMIETHFQVKVIHMNSYYLPINKK
IRKNIGKLKRKKRMIFTLKVGDSIPFSSINMN
>Q1ACF7 | RR19_CHAVU
MTRSLKKGPFIANHLLKKVHILNLEAQKKVILTWSRGSTIIPSMIGHTIAIHNGREHIPI
FITDQMVGHKLGEFSATRTFRGHAKNDKKSRR
>Q1ACF8 | RK22_CHAVU
MNQKQEDFSPSVKASLKSLNISFHKTRKVVNQIRGCSYEKALMILEFMPYRACKPITQLI
SSAASNANHNLGLKKKNLFISEAKVDEGTTIKRFQPRAQGRAYSIHKPTCHITIGMKSKN
KIQ
>Q1ACF9 | RR3_CHAVU
MGQKIHPLGFRLGFIQEHYSHWFAKANSYSELLQEDKEIRSCIYNHIRNHVRNSSNYGGI

ARINIYRRTDLVQLQIHTGFPALLIENRIRGLEHLRRDIQEKIDHKKRKLRMTLVEVLKP
YTEPTILAEYIALQLENRVAFRRVMKKAIQLAKISDIKGIKIQIAGRLNGAEIARNEWIQ
KGRLPLQMLRANIHYCHYPAHTIYGVLGIKVWIFREKII
>Q1ACG3 | RR8_CHAVU
MSKDTISNMITSIRNGNLSKAKIVQIPATQTARSIAKILLDEGFIENFREVVISDKNYSL
LTLTLKYQGRTREPYITTLRRISKPGLRSYSSYQDIPKVMGGMGIVILSTSQGIITDRQA
RQRRIGGEVLCYIW
>Q1ACG7 | RPOA_CHAVU
MIQNVTDDKIQWKCLESKIESQRIHYGRFAIAPLKKGQANTLGITLRRTLLSDLDGICIT
SVKIDNIKHEYCTLTGVRESIQDILLNLKEIVFKGVCDKTQKGFIFVKGPKKITASDIQI
EPCIEILDSNQIIAHLTEPIDFKVELTIEKSMGFRLQNATQISKDSFSIDAIFMPIRNVN
YSIHPVEKAGDWKSELLILEIWTNGSITPKEAFHQASEKIMNIFLSLSNSSDNQIQEKST
KNFEEKEEFIFQNIDGFKQDKETKVQESLGWKKISINQLELSARAYNCLKNEKISTLFDL
LNYSQEDLLKIKNFGKRSFEQVVNALEKHFDMKLSKDSSKKFYEQLKNLELINNKDIS
>Q1ACH6 | CLPP_CHAVU
MPIGVPKVPFRLPGEEDAVWVDIYNRLYRERLLFLGQRVDDEIANQLIGIMMYLNGEDDS
KEMFMYINSPGGSVVAGIAVFDAMQYVGPDVTTICMGMAASMGSFILMGGEMTKRIALPH
ARVMIHQPASSYYDGQAGECLMEAEEVLKLRDCIAKVYMNRTGQPGWVIYRDMDRDIFMS
ALEAKSYGIVDLVGEDPTATPSVWFRWPSLLDSKDSNEK
>Q1ACH9 | RR18_CHAVU
MKKKPIQKQRSRRRLPPIKSGEKIDYKNLDLLRRFLSEQGKILSKRVNRLTSKQQRTMTI
AIKRARILALIPFLNNEN
>Q1ACL0 | ODPB_CHAVU
MSEKLLYEALNEGIHEEIERDPKVFVIGEDIGHYGGSYKVTKGLFEKYGNLRILDTPIAE
NSFTGIAIGAAMTGLRPIIEGMNMGFLLLAFNQIANNAGMLHYTSGGNFTTPLVVRGPGG
VGRQLGAEHSQRLESYFQSVPGLQMVACSTPYNAKGLIKSAIRSQNPIIFFEHVLLYNIK
ENIPQKEYLVPLEKAELVRSGNQITILTYSRMRYHVLQAAKTLIEKGYDPEIIDIISLKP
LDMGTISTSLRKTHKVLIVEECMKTGGIGTTLKSAILESLFDFLDTPIMSLSSQDVPTPY
NGFLEDLTVIQPSQIVEAAEKIILYS
>Q1ACL1 | RR16_CHAVU
MVKLRLKRYGKKQQPTYRIVAIESSFRREGRAFKEVGIYIPKYNKTQLNVPAIIELLKNG
AQPTSTVKNILLRAQIVV
>Q1ACM7 | RR4_CHAVU
MSRYRGPRLKIVRRLGPLPGFTQKLYKSKSQYLGSSTTSSSSNKKISQYNIRLLEKQKLR
FYYGLTERQLLKYVTIARNAKGPTGQVLLQLLEMRLDNIVFRLGMAPTIPAARQLVNHRH
ILVNDFTVNIPSYSCKLGDKISVQKRFESKTNIFANSTQSASLKVPSHLTLNPAKNEGLV
HQIVDREFIGAKINELLVVEYYSRQV
>Q1ACN3 | RR2_CHAVU
MNHLYSKTNLEEMLEAGVHFGHQSHKWNPRMAPYIFSERKGVHIIDLIQTSNLLHEACDF
LANAASNKKEFLLVGTKFQASDLIKSEALKSRCHYVNQRWLGGMLTNWMTIETRIQRLKN
LETQEKLGAFHHLPKKDAAHLKRQLNQLQKYLGGMKYMKNLPDVVIIVDQQKEIIAVQEC
RVLGIPIIALVDTNCDPEIVDIPIPANDDARSSIRWILSKLTAAIRDNRF
>Q1ACN6 | RPOB_CHAVU
MKFEKNTDIFILPDFIRIQLESFHRFIEKGLLEEFQRFPVIRDSSGELSYFLNGKRYKLQ

EPSFNEQEAIYQASTYSSELYVPAQITHTKSEKTKSEIILIGNIPIMTARGNFIINGASR
VIVNQILRSPGLYYGSELDAQANITYSCTFISEWGGRLKLEMDNKRRIWVRLSKKRKIPV
LLLLLAMGLNWKDLEKLISRPSLLLSYLSKEKRTPYSPEEAIIELYRHSCSVSGEISFSE
NIRKELYHKFFKIRCEIGSIGRLNINKKLNLDIPEEERFLVPQDLIAAINSLIEKSFKIV
NLDDIDHLKNRRVHLIADMLQSQVRIALNRLKRMIAEVMRGINKRKNLPNPQSLVTPKPL
VATFQEFFGSHPLCQFMDQTNPLAEITHKRRISSLGPGGLTQKTATFNVRDIHPSHYGKI
CPIETPEGQNAGLVSSLAILANINQNGFIESPFLKLHKNEYKQLTGSEHLCSEQEEYYRI
TTGEHSRETAEKKGKPTPIRYRQEFSATAFHFLHFRDILPIQYFSIATSLIPFLEHDDAN
RALMGSNMQRQAVPLLRPEKPIVGTGLEGQVALDSAALVLSKIEGKVDFVDGRNINIRSN
IVINEFFSFQLINYERSNQNTCLHQKPIVKKDDYVKKGELLADASSTLGGEISLGNNILV
AYMPWEGYNFEDAVLINERLIYEDIYTSIHIEKYEIEVNMTSLGPEKITKKIPHLSEYLL
RNLDVNGLILLGSWVKTGDVLVGKLTPREPGESLRLPEGRLLHAIFGIKASSFFETCLKV
APGAEGRVIDVRWVESANKLTQKYIKTVNIYILQKRKIQVGDKIAGRHGNKGVISRILPR
EDMPYIQDGTPIDMVLSPLGVPSRMNLGQIFECLLGLAGESLKKHYRIMPFDERHEREAS
RKLAFSELYQAKKFTGHSWLFEPDTPGKSQLFDGRTGQTFENAITVGKAYILKLIHQVDD
KIHARSTGPYSMVTQQPLGGKARRGGQRMGEMEVWALEGFGAAYTLQELLTRKSDDMKGR
NEALGAIVTGNLIPKPNTTPESFKLLMRELRCLCLNIHHCSLFQENLSMKNIEF
>Q1ACP2 | RR7_CHAVU
MSRRKIPIKRSPKPDSIYRSRLVNMMVNRILKNGKKSLASHILYNAMKEIKHKTKKNPSL
ILRQAVVRATPKITIKARRIGGSNYQVPVEIKPEKGHSLAIRWLLTASRKRTGKNMVFKL
ANELLDAAKDTGGAIRKKEETHRMAEANRAFAHFRY
>Q1KVR1 | RK14_SCEOB
MIQPQSYLNVADNSGARKLMCIRVLGGGRQTATIGDVIIAVVKDALPNMPLKKSDVVRAV
IVRTSKGVRRENGMMLCFDDNAAVVINKEGNPRGTRVFGPIARELRDRNFTKIVSLAPEV
V
>Q1KVR2 | RK5_SCEOB
MTQRLKKLYTEKIVPKFYKDFQYKNIHEVPSLKKIVINRGIGDASQNAKILETFLKELSI
IAGQKGVITRSKKSIAGFKIRDKMPVGVSVTLRGDRMYGFLDRLIHLALPRVRDFQGINP
KSFDKNGNYSLGLEEQLMFPEIEYDKIDQIRGMDISIVTTAKNQEEGLALLKEFGLPFQS

>Q1KVR6 | RR9_SCEOB
MQTEKKEILATAVGRRKEAVAQVQLLRGTGEFIINNKSANIYLNNNSCSLLALKAPFNVL
QNLEILTESNQIDTIVKVQGGGLIGQAEAIKLGVARALCQIEVSGSETTDLIRKSFKDQG
YLTIDARVKERRKYGLKKARKASQYHKR
>Q1KVR9 | CHLL_SCEOB
MKLAVYGKGGIGKSTTSCNISIALAKRGKKVLQIGCDPKSDSTFTLTGFLIPTIIDTLQA
KDYHYEDVWPEDVIYQGYAGVDCVEAGGPPAGAGCGGYVVGETVKLLKEFNAFYEYDVIL
FDVLGDVVCGGFAAPLNYADYCIIVTDNGFDALFAANRITASVREKARTHPLRLAGLIGN
RTKKRDLIEKYVETCPMPILEVLPLIEDIRVSRVKGKTLFEMTESEPTLQFVCDFYLNIA
DQLLTQPEGVIPRELGDRELFNLLSNFYLNSSNSTNTTLKNETNLFDLV
>Q1KVT1 | RR18_SCEOB
MNNQSFNNFSQVNSNSSFFADKPKNLQNTNLEMTNGTNPPSSFSKQTPQKRQSFGTNTNF
SKGNSSRGSTSNKRKVLSVSQILARVNQKKQRKLEQKKRKKPLKPIIPPKSFILLFKDKP
EKYVYNRRIIDYKHCGLLQRYIGLGGKILPRRQTKLTAKQQRYVAKTIKSARIMGLLPFV

TKERSFFR
>Q1KVT5 | RR4_SCEOB
MSRYIGPRLRIIRRIGKLRGFTRKKPFRRSFRGRGALQGKVIPPGQHGLTKLFKSRPFDS
NESDYLIRLKVKQRLRYNYGITEKQLVKYVRQAKKMKESTGQVLLQLLEMRLDNIVFRLN
MAPTICAARQLISHGHIHVNSKKVNIASYMCKPKDVISVSMKQSSLKLVNRNLQEYSQKM
SAYKKRLERTLAYVLFQRNISPNMANALEYINQGKVQVNNRKVLLPNYLCHSKDMISVKT
DKGIRKFQFSE
>Q1KVT6 | RK23_SCEOB
MMADFIKYPVTTEKSYLSMFKNKQYTFDVDLRLTKPQIKKLFETLFGVNVIGINTHRPPR
KKVRAGLSTGYRPAYKRVILTLKEGQSIQF
>Q1KVT9 | RR14_SCEOB
MANKAMIQRELKRQNLVMKFAKKRAILKQQIQQASSLKEKLALHRKLQQLPRNSSPVRLH
NRCLVTGRPKGYFRDFGLSRHVLREMAHEGLLPGVRKASW
>Q1KVV0 | RBL_SCEOB
MVPQTETRAGAGFKAGVKDYRLTYYTPDYVVKDTDILAAFRMTPQPGVPPEECGAAVAAE
SSTGTWTTVWTDGLTTLDRYKGRCYDIEPVPGEDNQYIAYVAYPIDLFEEGSVTNLFTSI
VGNVFGFKALRALRLEDLRIPPAYVKTFQGPPHGIQVERDKLNKYGRGLLGCTIKPKLGL
SAKNYGRAVYECLRGGLDFTKDDENVNSQPFMRWRDRFLFVAEATYKAQSETGEIKGHYL
NATAATCEEMLKRAQCAKELGVPIIMHDYLTGGFTANTSLSSYCRDHGLLLHIHRAMHAV
IDRQRNHGIHFRVLAKALRLSGGDHLHSGTVVGKLEGEREVTLGFVDLMRDDYIEKDRSR
GIYFTQDWCSMGGTMPVASGGIHVWHMPALVEIFGDDACLQFGGGTLGHPWGNAPGAVAN
RVALEACTQARNEGRDLAREGGDVIRSACKWSPELAAACEVWKEIKFEFETIDKL
>Q1KVX0 | RR12_SCEOB
MPTIQQLIRSARKKVSKKTKAPALKSCAQRRGICLRVYTVTPKKPNSALRKVARVRLTSG
FEVTAYIPGIGHNLQEHAVVLVRGGRVKDLPGVRYHIVRGTLDTAGVKNRVQSRSKYGVK
LAATKAAAKK
>Q1KVX4 | RPOB1_SCEOB
MYNSNSYIPDLVEIQRQGFFYLLEKGIIEEISKRNPITCFEKQIEIFFYPQYYRLTKPFY
SIQQAIFYKKSYVSKFYIPVQLTDRKTKRIFLKWILLAHFPLMTNRGHFLLNGSARVIIN
QLVRSPGIYFRENMHEIFSNKWSEKPSTTFRRFYADIICLKGTWLRIECDKDFSMWAKMK
KGPKIPLLWFLLGMGLSEKYILNSVYKPMNLLQSFTKEMEKMQKSKSSKELKYPYISSTK
QAWEQIQKLFKLKKTLRKFSFLKKNEEKPKTFIFGQSSTKIEAKVVFSDFAAFNNSVKRE
NTVFASNDDTFLVQTSFETKKKKNTRKKENFQKNKTQKTTKLSLQNKEKRVASKLEAYEL
GRKWFANKFMNPRTYDLGKQGRWLINKKLGLTIPLEQTTLTALDVLTATDSLMKIEDGFF
EIDDIDHLKNKRVRTAGEALQEIFSIGLIRLEKSIRLKLNGNGQQSFFQKTSFFNFDSLM
STRPINGAFREFFGTHPLSQFMDQINPLAEITHKRRLSSLGPGGVSRDTATLAIRGIHPS
HYGRICPIETPEGKNTGLVNSITTYSKISFHGYLESPFYKVYKGQVQRNLGVFYLSPDKD
DHFQTATPDLNLTPLGFLPKKPIPVRIGKRFVRMKTHKLALMGVSPLQMISVATSFIPFL
EHDDANRALMGSNMQRQAVPLIRPERPLIGTGLEARVVSDSGHAILAKTSGYIVYSSGSK
IYLYTI
>Q1KVX7 | RR3_SCEOB
MGQKIHPLGFRVGITKKHQSVWFARFHKHQYAQSVLEDRFLRKNLLKLLPQLFQKKLNNS
PNMPKISHIQIVRGLIPYEIGIQIHAQNCHMIKSAFEGLEVQPNFVHNLLKNHLILEKNS
VKKTSLFKNAGENIELSLTAESTNDFDSTEKKRGGLNRKGEQSLNFKNSTKKTMKSNVEK

RKKLFARFHQRFLGKFIVVKNGKKITKKFDSKKQLSFKNNLLSFFKKNLQERRTFRKNKF
QTSFSKSSGFSKNQKNRETISLKQKAFLNFVDSKVSKANLSKPCTKFVNFYMSQTNLSFL
NALKAEMNYWNQYFENSKTEEIQKFGSFRYLPLGSQKKWNLLRFKRFEKLPLPLLLKLFK
ALQQKALKKLERLRKEYLVFGKFSKTKTFSYYQRVRFLQNLKKFISLRKNQNSLNFSQST
FENSAQKTTNQKFALKVASLTEKSFQKKFSKIHEEKNTIKFIDHLQALVQQHRSKNLFLY
LATISESRQNLKKIQQFTKQQSDFLFGISPKTLVGLTNEEKQEILKNKVSKTFFKISQKN
ASQKKNFQDIFVEQLQKQRTICEKNIQLTPKISIQFYSVKAQDFQTRASAVADSIVDDLE
KRKAFRGVIKKAKEDLMRTPKVKGVKIQVSGRLNGAEIARSEWVRAGRVPLQTLRANIDY
CYKTAQTIYGIIGVKVWIYKGYTKTRKPTANSPSVLLDLL
>Q1KXP8 | RR7_HELAN
MSRRGTAEEKTAKSDPIYRNRLVNMLVNRILKHGKKSLAYQIIYRAVKKIQQKTETNPLS
VLRQAIHGVTPGIAVKARRVGGSTHQVPIEIGSTQGKALAIRWLLAASRKRPGRNMAFKL
SSELVDAAKGSGDAIRKREETHRMAEANRAFAHFR
>Q1KXQ3 | NU4C_HELAN
MNKFPWLTIIVVLPIFAGSLIFFLPHKGNRVIRWYTICICMLELLLTTYAFCYHFQLDDP
LIQLVEDYKWINFFDFRWKLGIDGLSLGPVLLTGFITTLATLAAWPVTRDSRLFHFLMLA
MYSGQIGSFSSRDLLLFFIMWELELIPVYLLLSMWGGKKRLYSATKFILYTAGGSIFLLM
GVLGVGLYGSNEPTLNFETSVNQSYPVALEIIFYIGFLIAFAVKLPILPLHTWLPDTHGE
AHYSTCMLLAGILLKMGAYGLIRINMELLPHAHSIFSPWLMVVGTIQIIYAASTSLGQRN
LKKRIAYSSVSHMGFILIGIASITDTGLNGAILQIISHGFIGAALFFLAGTSYDRIRLVY
LDEMGGVAIPMPKIFTMFSSFSMASLALPGMSGFVAEVLVFLGIITSQKYLLMPKIAITF
VMAIGMILTPIYLLSMSRQIFYGYKLFNTPNSYVFDSGPRELFVSISIFIPVIGIGMYPD
FVLSLSVDKVEGILSNYFYR
>Q1KXR8 | RR19_HELAN
MTRSLKKNPFVANHLLKKINKLNTKEEKEIIITWSRASTIIPIMVGHTIAIHNGKEHLPI
YITDRMVGHKLGEFAPTLNFRGHAKSDNRSRR
>Q1KXR9 | RK22_HELAN
MLNKRTTEVYALGQHISMSAHKARRVIDQIRGRSYEETLMILELMPYRACYPIFKLVYSA
AANASSNMGSNEANLVISKAEVNKGTIMKRLKPRARGRSFAIQKPTCHITIVMKDISLDE
YIDTDSIAWSQNKKKDTTMSYYDMYSNGGTWDKK
>Q1KXS0 | RR3_HELAN
MGQKINPIGFRLGTTQGHHSLWFAQPKNYSEGLQEDQKIRNYIKNYVQKNMKTSSGVEGI
ARIEIQKRIDLIQIIIYMGFPKILIESRPRGIEELQMNLQKEFNSVNRKLNIAITRIEKP
YGNPNILAEFIAGQLKNRVSFRKAMKKAIELTEQADTKGIQVQIAGRIDGKEIARVEWIR
EGRVPLQTIRAKIDYCSYTVRTIYGVLGIKIWIFIEGE
>Q1KXS3 | RR8_HELAN
MSSDTIADIITSIRNADMYRKSVVRVASTNISQSIVKILLREGFIENVRKHRENNKDFLV
LTLRHRRNRKRPYRNLLNLKRISRPGLRIYSNYQRIPRILGGMGVVILSTSRGIMTDREA
RLERIGGEILCYIW
>Q1KXS6 | RR11_HELAN
MAKAIPKKGSRGRISSRKSIRKIPKGVIHIQASFNNTIVTVTDVRGRVVSWSSAGTCGFQ
GTRRGTPFAAQTAAANAIRAVVDQGMQRAEVMIKGPGLGRDAALRAIRRSGILLTFVRDV
TPMPHNGCRPPKKRRV
>Q1KXT6 | RK20_HELAN

MTRIRRGYIARRRRTKIRLFASSFRGAHSRLTRTITQQKIRALVSAHRDRDRQKINFRRL
WITRINAAIRERGVCYSYSRFINDLYKRQLLLNRKILAQIAISNRNCLYMISNEIIKEVS
WKESTG
>Q1KXT7 | RR18_HELAN
MDKSKRTFLKSKRSFRKRLPPIQSGDRIDYKNMSLISRFISEQGKILSRRVNRLTLKQQR
LITIAIKQARILSLLPFLNNEKQFERTESTTRTPSLRARKR
>Q1KXW1 | RR14_HELAN
MAKKSLIQREKKRQKLEQKYHLIRRSSKEEISKVRSLSDKWEIYGKLQSPPRNSAPTRLH
RRCFSTGRPRANYRDFGLSGHILREMVHACLLPGATRSSW
>Q1KXX7 | RR16_HELAN
MVKLRLKRCGRKQRIVAIDVRSRREGRDLRKVGFYDPIKNQTYLNVPAILYFLEKGAQPT
GTVQDILKKAEVFKELGPNQTKFN
>Q20EV0 | RR14_OLTVI
MAKKALIQRELKRQKCVAQFAEKRKALKQAIKEASSLQEKLNLHRKLQQLPRNSSAVRLH
NRCRVTGRPKGYFRDFGLSRHVLREMAHDCVLPGVTKSSF
>Q20EV1 | RK20_OLTVI
MTRVKRGYVARKRRKKVLSLTKGFRGSSSVLFRSANQRNMKALKYAYRDRRKMKREFRKL
WITRINAATRMSNMTYSTFIHKLKKANIVLNRKLLAQLAVRDQQVFQQLFSYIEGV
>Q20EY2 | CHLB_OLTVI
MKLAYWMYSGPAHIGTLRVASSFKNVHAIMHAPLGDDYFNVMRSMLQRERDFTPVTASIV
DRHVLARGSQEKVVDNITRKDKEDNPDLILLTPTCTSSILQEDLQNFVNQAELDLETDVI
LADVNHYRVNELQAADRTLEQITRHYIEKAKRQGQLDIQKSEKPTANILGVFTLGFHNQH
DCRELKRLLNDLGIEVNEIIPEGAFVESLKNLPKAWFNLIPYREVGLMTAQYLKKEFNMP
YISTTPMGIVDTATCVREICKTVASLRLSFGVDTPQKQLKETNISSTKVDLVLKVEENRE
AKPLNTSMEVLGESKLRFGPLTNFDVEKYIDQQTRFISQAAWFSRSVDCQNLTGKKAIVF
GDATHAASMTKILTREMGLYVLCAGTYCKHDADWFREQVQGFCDSVLITDDHTEIGNIIA
QYEPDCIFGTQMERHVGKRLDIPCGVISAPVHIQNFPLGYRPFLGYEGINQIADLVYNSF
SLGMEEHLLEIFGGHDTKEVISDSLSTETGLEWTSEGLTELNKVPGFVRNKVKRNVEKFA
QQQNISKISADVMYAAKEA
>Q20F02 | RPOA_OLTVI
MKHILLSCVESRIEHNRSFYSRFQLGPFDLGQGLTVGNAFRRTLLSELSGVGITLIEIDG
VCHEYSTLPGVRESVLDILLNLKQLVLTSDTTFTVPQVGYLNFTGPGVVTAKDLKLPVSV
YCVDPDQPIATLSADATLNFKFLVCQGKNYIIQTPVDKFHEYQKKILNSKLNLPLSSRTT
LNSTFERPNQDSLVGTPLQEGVGKTKQNKRLNAHFQRQLKQLNRLKETSFKAYKTKVGEK
TISDEFQTNSVNHQNLTTFDLREKKLTDVTTSLQHGEEATLLKEEGLNKLDNSSPQLENK
LKGGRDFSGGSQLASQALPVDTLFMPIKKVNYMIDIDPAEPDRESVVLEIWTNGSIHPRQ
AVHEAAKQLIHLFSPFLQTHLVPTTLQSTPYSKAASKTQEFSLNETSTEKVKDELGKVAF
GSENNTAALNQQKNLVTAIQTLDIANLNFSLRTFTFLKKENINTVSELVEFWTRQKTEAK
DSIQSNVQSSVLLEISNNLKNFGVLPTS
>Q20F07 | RK5_OLTVI
MAQRFKELYLQTIVPKLKDEFGYKNSHQVPEIKKIVINRGLGDASQNAKILDSSLNELSI
IAGQRGVVTRSKKAIAGFKLRDKMAVGISVTLRGDRMYSFLDRLINLALPRIRDFQGISP
KSFDGCGNYSLGLEEQLMFPEIDYDKIDQVRGMDISIVTTCSNNEEGVALLKNFGMPFKA

>Q20F08 | RK14_OLTVI
MIQPQTYVNVADNSGARKLMCIRVLDASFKQSANIGDTIIAVVKEAIPNMPLKKSDIVRA
VIVRSAKGIRRPDGTIIRFDDNAAVVINKEGNPRGTRVFGPVARELREREFTKIVSLAPE
VL
>Q20F09 | RK16_OLTVI
MLSPKRTKYRRYHRGRMKGKAMRGNKLTYGDFALQSLEPGWLTSRQIEAGRRVLTRYVRR
TGKLWIRIFPDKPITMRPADTRMGKGKGAPEFWVAVVKPGTMIYEITGVTEPIARSAMRI
TAYKMPVKTQFVAR
>Q20F10 | RR3_OLTVI
MGQKVHPLGFRLGVTQKHRSQWFAKPQEYSQLVLEDHFLREELNKRFPNSGIIDLFIQRK
VDRTQVEVEIRTTRPGLLVGRKRQNCDKLKQLRDELQVELNKFRAKNGVSPTAKVALQVT
TVQNKSSEAIVIAEFLVNQLERRIAFRRALRRVLSIIQRDRVQGAKIQVSGRLNGAEIAR
SEWIRTGRVPLQTLRADVDYCHREAKTIYGILGIKVWTFKGEIKPVRKAVEVPVA
>Q20F12 | RK2_OLTVI
MGIRFYKANTPGTRNRSVSDFSEITHSKPEKSLTFNVQRAKGRNNRGVITSRHRGGGHKR
LYRLIDFKRNKIGIAAKVATIEYDPNRNARIALLHYQDGEKRYILHPRGLNVGSSIISSL
NAPVLTGNALPLKNIPLGGEVHNVELQPGAGGQLARAAGAVAQIVAKEGNLVTLRLPSGE
VRLVSNECWATVGQVGNTDAINMVVGKAGRKRWLGKRPHVRGVVMNPCDHPHGGGEGRSP
IGRPRPVSPWGKPALGQRTRKGHKYSDQMILRRRK
>Q27RZ0 | RR7_SOLTU
MSRRGTAEKKTAKSDPIYRNRLVNMLVNRILKHGKKSLAYQIIYRAVKKIQQKTETNPLS
VLRQAIRGVTPDITVKARRVGGSTHQVPIEIGSTQGKALAIRWLLAASRKRPGRNMAFKL
SSELVDAAKGSGDAIRKKEETHRMAEANRAFAHFR
>Q27S25 | RR12_SOLTU
MPTIKQLIRNTRQPIRNVTKSPALRGCPQRRGTCTRVYTITPKKPNSALRKVARVRLTSG
FEITAYIPGIGHNSQEHSVVLVRGGRVKDLPGVRYHIVRGTLDAVGVKDRQQGRSKYGVK
KPK
>Q2L8Z0 | PSBK_GOSHI
MLNIFNLICICFNSALFSSSFLFAKLPEAYAFLNPIVDFMPVIPVLFFLLAFVWQAAVSF
R
>Q2L8Z5 | RPOC2_GOSHI
MAERANLVFHNKVIDGTAIKRLISRLIDHFGMAYTSHILDQVKALGFQQATATSISLGID
DLLTIPSKGWLVQDAEQQSLILEKHHHFGNVHAVEKLRQSIEIWYATSEYLRQEMNPNFR
MTDPFNPVHIMSFSGARGNASQVHQLVGMRGLMSDPQGQMIDLPIQSNLREGLSLTEYII
SCYGARKGVVDTAVRTSDAGYLTRRLVEVVQHIVVRRTDCGTTRGISVSPQKRTLPERIF
IQTLIGRVLADDIYMGPRCIAIRNQDIGLGLVDRFRAFRTQPISIRTPFTCRSTSWICRL
CYGRSPTHGDLVELGEAVGIIAGQSIGEPGTQLTLRTFHTGGVFTGGTAEHVRAPFNGKI
KFNEDLVHPTRTRHGHPAFLCYRDLYVIIESEDIIHKVAIPPKSFLLVQNDQYVESEQVI
AEIRAGTYTLNLKERVRKHIYSDSEGEMHWSTDVYHSPEFTYSNVHLLPKTSHLWILSGG
SYKFSVVPFSLHKDQDQISIHYLSAERRYISRFSVNNDQVRHNLFSSDFSDKKEERIYDY
SELNRIIGTGHCDFIYSAILHENADLLAKRRRNRFIIPFQLIQDQEKELMLHSHSGISME
IPINGIFRRKSILAFFDDPRYRRKSSGITKYGTLGAHSIVKREDVIEYRGVKKVKPKYQM
KVDRFFFIPEEVHILSESSSIMVRNNSIIGVDTPITLNTRSQVGGLVRVERKKKRIELKI
FSGNIYFPGERDKISRHSGILIPPGTGKTNSKESKKLKNWIYVQRITPTKKKYFVLVRPV

TPYEIPDGLNLATLFPQDPFQEKDNMQLRAVNYILYGNGKPTRRISDTSIQLVRTCLVLS
WDQDNKSSFAEEVCASFVEVRTNGLIRDFLRIDLVKSHIFYIRKRNDPSGSELISDNRSD
RTNKNPFYSIYSNARIQQSFSQNHGTIHTLLNRNKESQSLIILSASNCFRMGPFNDVKYH
NVIKQSIKKDPLIPIKNLLGPLGTAPKIANFYSSFYPLITHNQTSVAKYFELDNLKQAFQ
VLNYYLIAENGRIYNFDPCRNIFLNAVNLNWYFPHHHYHHNYCEETSTIISLGQFICENV
CIAKSGPRLKSGQVFIVQADSIVIRSAKPYLATPGATVHGHYGETLYEGDTLVTFIYEKS
RSGDITQGLPKVEQVLEVRSIDSISMNLEKRIEGWNECITRILGIPWGFVIGAELTIVQS
RLSLVNKIQKVYRSQGVQIHNRHIEIIVRQITSKVLVSEDGMSNVFLPGELIGLLRAERT
GRALEEAICYRAVLLGITRASLNTQSFISEASFQETARVLAKAALRGRIDWLKGLKENVV
LGGMIPAGTGFKGLVHRSRQHNNILLETKKKNFFGGEMRDIFFHHRELFDSCISNNLHDT
SGRSFIGIEFNDS

>Q2L8Z9 | RPOB_GOSHI

MLGDENGEMSTIPGLNQIQFEGFCGFMDRGLTEELYKFPKIEDTEQEIEFQLFVETYQLV
EPLIKERDAVYESLTYSSELYVSAGLIWKTSKDMQEQTIFIGNIPLMNSLGTSIVNGIYR
IVINQILQSPGIYYRSELDHNGISVYTGTIISDWGGRLELEIDRKARIWARVSRKQKISI
LVLSSAMGSNLREILENVCYPEIFLSFLTDKEKKKIGSKENAILEFYQQFSCVGGDPVFS
ESLCKELQKKFFQQRCELGRIGRRNMNQRLNLNIPQNNTFLLPRDILAAADRLIGMKFGM
GPLDDMNHLKNKRIRSVADLLQDQFGLALVRLENVVRGTICGAIRHKLIPTPQNLVTSTP
LTTTYESFFGLHPLSQVLDRTNPLTQIVHGRKLSYLGPGGLTGRTANFRIRDIHPSHYGR
ICPIDTSEGINVGLIGSLAIHARIGHWGSLESPFYKIFERSKKAQMLYLSPSRDEYYMVA
AGNSLALNQGIQEEQVVPARYRQEFLTIAWEQVHLRSIFPFQYFSIGASLIPFIEHNDAN
RALMSSNMQRQAVPLSRSEKCIVGTGLERQVALDSGVPAIADHEGKIISTDTDKIILSGN
GDALGIPLVMYQRSNKNTCMHQTARVRRGKCIKKGQILADGAATVGGELALGKNVLVAYM
PWEGYNFEDAVLISERLVYEDIYTSFHIRKYEIQTHVTSQGPERITNEIPHLEAHLLRNL
DKNGIVMLGSWVETGDILVGKLTPQVAKESSYAPEDRLLRAILGIQVSTSKETCLKLPIG
GRGRVIDVRWVQKKGGSSYNPETIRVYISQKREIKVGDKVAGRHGNKGIISKILPRQDMP
YLQDGRPVDMVFNPLGVPSRMNVGQLFECSLGLAGSLLDRHYRIAPFDERYEQEASRKLV
FSELYQASKQTANPWVFEPEYPGKSRIFDGRTGGPFEQPVIIGKPYILKLIHQVDDKIHG
RSSGHYALVTQQPLRGRSKQGGQRVGEMEVWALEGFGVAHILQEMLTYKSDHIRARQEVL
GTTIIGGTIPKPEDAPESFRLLVRELRSLALELNHFLVSEKNFQINRKEA

>Q2L928 | RR18_GOSHI

MDKSKRLFLKSKRSFRRRLPPIQSGDRIDYRNMSLISRFISEQGKILSRRVNRLTLKQQR
LITIAIKQARILSSLPFLNNEKQFERSESTTRTTALRTRNK

>Q2L940 | RK16_GOSHI

MLSPKRNRFRKQHRGRMKGISYRGNRICFGRYALQALEPAWITSRQIEAGRRAMTRNVRR
GGKIWVRIFPDKPVTVRPTETRMGSGKGSPEYWVAVVKPGRILYEMSGVAENIARKAISI
AASKMPIKTQFIISG

>Q2L941 | RK14_GOSHI

MIQPQTHLNVADNSGARELMCIRVIGASNRRYAHIGDVIVAVIKEAVPNTPLERSEVIRA
VIVRTRKELKRDNGMIIRYDDNAAVVIDQEGNPKGTRIFGAIARELRQLNFTKIVSLAPE
VL

>Q2L942 | RR8_GOSHI

MGKDTIADIITSIRNADMNRKGTIQIGSTNITENIVKILLREGFIDNVRKHRERNKYFLV
LTLRHRRNRKGPHRTILNLRRISRPGLRIYSNYQQIPRILGGMGIVILSTSRGIMTDREA

RLEGIGGEILCSIW
>Q2L945 | RR19_GOSHI
MARSLKKNPFVANHLLKKIERLNTKAEKEIIITWSRASTIIPTMIGHTIAIHNGKEHLPI
YITDRMVGHKLGEFAPTINFRGHAKNDNKSRR
>Q2L946 | RR3_GOSHI
MGQKINPLGFRLGTTQSHHSLWFAQPKKYSEGLQEDKKIRDCIKNYVQKNTRLSSGVEGI
ARIEIQKRLDLIQVIIYMGFPKLLIEDKPRKLEELQMNVQKELNCMNRKLNIAITRIGNP
YGHPNILAEFIAGQLKNRVSFRKAMKKAIELTEQADTKGIQIQIAGRIDGKEIARVEWIR
EGRVPLQTIGAKIEYCSYRVRTIYGVLGIKIWIFIDEE
>Q2MI43 | RR15_SOLLC
MVKNSVISVISQEEKKGSVEFQVFNFTNKIRRLTSHLELHKKDYLSQRGLKKILGKRQRL
LAYLAKKNRVRYKELINRLDIRETKTR
>Q2MI44 | NDHH_SOLLC
MTAPTTRKDLMIVNMGPQHPSMHGVLRLIVTLDGEDVVDCEPILGYLHRGMEKIAENRTI
IQYLPYVTRWDYLATMFTEAITINGPEQLGNIQVPKRASYIRVIMLELSRIASHLLWLGP
FMADIGAQTPFFYIFRERELIYDLFEAATGMRMMHNYFRIGGVAADLPYGWIDKCLDFCD
YFLTGVAEYQKLITRNPIFLERVEGVGIIGRDEALNWGLSGPMLRASGIEWDLRKVDHYE
SYDEFDWQVQWQREGDSLARYLVRIGEMTESIKIIQQALEGIPGGPYENLEMRRFDRLKD
PEWNDFEYRFISKKPSPTFELSKQELYVRVEAPKGELGIFLIGDQSVFPWRWKIRPPGFI
NLQILPQLVKRMKLADIMTILGSIDIIMGEVDR
>Q2MI51 | CCSA_SOLLC
MIFSTLEHILTHISFSIVSIVITIHLITFLVDEIVKLYDSSEKGIIVTFFCITGLLVTRW
VSSGHFPLSDLYESLIFLSWSFSLIHIIPYFKKNVLILSKITGPSAILTQGFATSGILTE
IHQSGILVPALQSEWLIMHVSMMILGYAALLCGSLLSVALLVITFRKNRKLFSKSNVFLN
ESFFLGENVVENTSFFCTKNYYRSQLIQQLDYWSYRVISLGFTFLTIGILSGAVWANEAW
GSYWNWDPKETWAFITWIVFAIYLHTRTNRNLRGPNSAIVASIGFLIIWICYFGVNLLGI
GLHSYGSFPSTFN
>Q2MI58 | RK23_SOLLC
MDGIKYAVFTDKSIRLLGKNQYTSNVESGSTRTEIKHWVELFFGVKVIAMNSHRLPGKSR
RMGPIMGHTMHYRRMIITLQPGYSIPPLRKKRT
>Q2MI60 | RR19_SOLLC
MTRSLKKNPFVANHLLKKIDKLNTKAEKEIIVTWSRASTIIPTMIGHTIAIHNGKEHLPI
YITDSMVGHKLGEFAPTLNFRGHAKSDNRSRR
>Q2MI61 | RK22_SOLLC
MLKKKKTEVYALGEHISMSADKARRVIDQIRGRSYEETLMILELMPYRACYPILKLVYSA
AANASYNMGSSETNLVISKAEVNEGTTVKKLKPRARGRSFPIKRSTCHITIVMKDISLDD
EYGEMSSLKKTRWKKKSTAMTYRDMYNSGGLWDKK
>Q2MI62 | RR3_SOLLC
MGQKINPLGFRLGTTQSHHSLWFSQPKNYSEGLQEDKKIRDCIKNYVQKNMRTSSGIEGI
ARIEIQKRIDLIQVIIFMGFPKLLIESRPRGIEELQMTLQKEFNCVNRKLNIAVTRIAKP
YGNPNILAEFIAGQLKNRVSFRKAMKKAIELTEQADTKGIQIQIAGRIDGKEIARVEWIR
EGRVPLQTIRAKIDYCSYTVRTIYGILGIKIWIFLDEE
>Q2MI64 | RK14_SOLLC
MIQPQTHLNVADNSGARELMCIRIIGASNRRYAHIGDVIVAVIKEAVPNMPLERSEVVRA

VIVRTCKELKRDNGMIIRYDDNAAVVIDQEGNPKGTRIFGAIARELRELNFTKIVSLAPE
VL
>Q2MI68 | RR11_SOLLC
MAKAIPKISSRRNGRISSRKGARRIPKGVIHVQASFNNTIVTVTDVRGRVVSWSSAGTSG
FKGTRRGTPFAAQTAAANAIRTVVDQGMQRAEVMIKGPGLGRDAALRAIRRSGILLTFVR
DVTPMPHNGCRPPKKRRV
>Q2MI95 | NU3C_SOLLC
MFLLYEYDFFWAFLIISILVPILAFFISGVLAPISKGPEKLSTYESGIEPMGDAWLQFRI
RYYMFALVFVVFDVETVFLYPWAMSFDVLGVSVFIEAFIFVLILIIGLVYAWRKGALEWS

>Q2MI96 | NDHK_SOLLC
MVLAPEYSDNKKKNGKNKIETVMNSIQFPLLDRTAPNSVISTTLNDLSNWSRLSSLWPLL
YGTSCCFIEFASLIGSRFDFDRYGLVPRSSPRQSDLILTAGTVTMKMAPSLVRLYEQMPE
PKYVIAMGACTITGGMFSTDSYSTVRGVDKLIPVDVYLPGCPPKPEAVIDAITKLRKKIS
RELYEDRIRSQRANRCFTTNHKFHVRRSIHTGNYDQRVLYQPPSTSEIPTEIFFKYKNSV
SSAELVN
>Q2MIA8 | RPOB_SOLLC
MLGDGNEGISTIPGFNQIQFEGFCRFIDQGLTEELYKFPKIEDTDQEIEFQLFVETYQLV
EPLIKERDAVYESLTYSSELYVSAGLIWKNSRDMQEQTIFIGNIPLMNSLGTSIVNGIYR
IVINQILQSPGIYYRSELDHNGISVYTGTIISDWGGRSELEIDRKARIWARVSRKQKISI
LVLSSAMGLNLREILENVCYPEIFLSFLNDKERKKIGSKENSILEFYQQFACVGGDPVFS
ESLCKELQKKFFQQRCELGRIGRRNMNRKLNLDIPQNNTFLLPRDILAAADHLIGLKFGM
GALDDMNHLKNKRIRSVADLLQDQFGLALVRLENVVRGTICGAIRHKLIPTPQNLVTSPP
LTTTYESFFGLHPLSQVLDRTNPLTQIVHGRKLSYLGPGGLTGRTASFRIRDIHPSHYGR
ICPIDTSEGINVGLIGSLSIHARIGHWGSLESPFYEISERSTGVRMLYLSPGSDEYYMVA
AGNSLALNRDIQEEQVVPARYRQEFLTIAWEQVHLRSIFPFQYFSIGASLIPFIEHNDAN
RALMSSNMQRQAVPLSRSEKCIVGTGLERQAALDSGALAIAEREGRIVYTNTHKILLAGN
GDILSIPLVIYQRSNKNTCMHQKFRVPRGKCIKKGQILADGAATVGGELALGKNVLVAYM
PWEGYNSEDAVLISERLVYEDIYTSFHIRKYEIHTHVTSQGPEKVTNEIPHLEAHLLRNL
DKKGIVMLGSWVETGDILVGKLTPQVVKESSYAPEDRLLRAILGIQVSTSKETCLKLPIG
GRGRVIDVRWIQKRGGSSYNPETIRVYISQKREIKVGDKVAGRHGNKGIISKILPRQDMP
YLQDGRSVDMVFNPLGVPSRMNVGQIFECSLGLAGSLLDRHYRIAPFDERYEQEASRKLV
FSELYEASKQTANPWVFEPEYPGKSRIFDGRTGNPFEQPVIIGKPYILKLIHQVDDKIHG
RSSGHYALVTQQPLRGRAKQGGQRVGEMEVWALEGFGVAHILQEMLTYKSDHIRARQEVL
GTTIIGGTIPNPEDAPESFRLLVRELRSLALELNHFLVSEKNFQINRKEA
>Q2MIB0 | RPOC2_SOLLC
MEVLMAERANLVFHNKAIDGTAMKRLISRLIEHFGMAYTSHILDQVKTLGFQQATATSIS
LGIDDLLTIPSKGWLVQDAEQQSLILEKHHQYGNVHAVEKLRQSIEIWYATSEYLRQEMN
PNFRMTDPFNPVHIMSFSGARGNASQVHQLVGMRGLMSDPQGQMIDLPIQSNLREGLSLT
EYIISCYGARKGVVDTAVRTSDAGYLTRRLVEVVQHIVVRRTDCGTARGISVSPRNGIMP
ERIFSQTLIGRVLADDIYMGSRCIATRNQAIGIGLVNRFITFRAQPISIRTPFTCRSTSW
ICRLCYGRSPTHGDLVELGEAVGIIAGQSIGEPGTQLTLRTFHTGGVFTGGTAEHVRAPS
NGKIKFNEDLVHPTRTRHGHPAFLCSIDLYVTIESEDILHNVNIPPKSLLLVQNDQYVES
EQVIAEIRAGISTLNFKEKVRKHIYSDSDGEMHWSTDVYHAPEFTYGNVHLLPKTSHLWI

LLGGPCRSSLVYLSIHKDQDQMNAHSLSGKRRYTSNLSVTNDQARQKLFSSDFYGQKEDR
IPDYSDLNRIICTGQYNLVYSPILHGNSALLSKRRRNKFIIPLHSIQELENELMPCSGIS
IEIPVNGIFRRNSILAYFDDPRYRRKSSGIIKYGTIETHSVIKKEDLIEYRGVKEFRPKY
QMKVDRFFFIPEEVHILPGSSSLMVRNNSIVGVDTQITLNLRSRVGGLVRVERKKKRIEL
KIFSGDIHFPGETDKISRHTGVLIPPGTGKRNSKEYKKVQNWIYVQRITPSKKRFFVLVR
PVVTYEITDGINLGTLFPPDPLQERDNVQLRIVNYILYGNGKPIRGISDTSIQLVRTCLV
LNWNQDKKSSSCEEARASFVEIRTNGLIRHFLKINLVKSPISYIGKRNDPSGSGLLSDNG
SDCTNINPFSAIYSYSKAKIQQSLNQPQGTIHTLLNRNKECQSLIILSAANCSRMEPFKD
VKYHSVIKESIKKDPLIPIRNSLGPLGTCLPIENFYSSYHLITHNQILVTKYLQLDNLKQ
TFQVIKLKYYLMDENGKIFNPDPCRNIILNPFNLNWSFLHHYYCAETSKIISLGQFICEN
VCIAKNGPPLKSGQVILVQVDSIVIRSAKPYLATPGATVHGHYGETLYEGDTLVTFIYEK
SRSGDITQGLPKVEQVLEVRSIDSISMNLEKRVEGWNKCIPRILGIPWGFLIGAELTIAQ
SRISLVNKIQQVYRSQGVQIHNRHIEIIVRQITSKVLISEDGMSNVFSPGELIGLLRAER
MGRALEEAICYRVVLLGITRASLNTQSFISEASFQETARVLAKAALRGRIDWLKGLKENV
VLGGVIPVGTGFKGLVHPSKQHNNIPLETKKTNLFEGEMRDILFHHRKLFDSCLSKKFHD
IPEQSFIGFNDS
>Q2MIB1 | RR2_SOLLC
MTRRYWNINLEEMMEAGVHFGHGTRKWNPKMAPYISAKRKGIHITNLTRTARFLSEACDL
VFDAASRGKQFLIVGTKNKAADSVEWAAIRARCHYVNKKWLGGMLTNWSTTETRLHKFRD
LRMEQKTGRLNRLPKRDAAMLKRQLSRLQTYLGGIKYMTGVPDIVIIVDQHEEYTALREC
ITLGIPTICLTDTNCDPDLADISIPANDDAISSIRLILNKLVFAICEGRSSYIRNP
>Q2MIB9 | MATK_SOLLC
MEEIHRYLQPDSSQQHNFLYPLIFQEYIYALAQDHGLNRNRSILLENSGYNNKFSFLIVK
RLITRMDQQNHLIISTNDSNKNPFLGCNKSLYSQMISEGFACIVEIPFSIRLISSLSSFE
GKKIFKSHNLRSIHSTFPFLEDNFSHLNYVLDILIPYPVHLEILVQTLRYWVKDASSLHL
LRFFLHEYCNLNSLITSKKPGYSFSKKNQRFFFFLYNSYVYECESTFVFLRNQSSHLRST
SFGALLERIYFYGKIERLVEAFAKDFQVTLWLFKDPVMHYVRYEGKSILASKGTFPWMNK
WKFYLVNFWQCHFSMYFNTGRIHINQLSNHSRDFMGYLSSVRLNHSMVRSQMLENSFLIN
NPIKKFDTLVPIIPLIGSLAKAHFCTGLGHPISKPVWSDLSDSDIIDRFGRICRNLFHYY
SGSSKKKTLYRIKYILRLSCARTLARKHKSTVRTFLKRSGSELLEEFLTSEEEVLSLTFP
RASSSLWGVYRSRIWYLDIFCINDLANSQ
>Q2MID0 | RR15_SOLBU
MVKNSVISVIFQEEKKGSVEFQVFNFTNKIRRLTSHLELHKKDYLSQRGLKKILGKRQRL
LAYLAKKNRVRYKELINQLDIRETKTH
>Q2MID9 | RK32_SOLBU
MAVPKKRTSTSKKRIRKNIWKRKGYWVALKAFSLAKSLSTGNSKSFFVRQTKINK
>Q2MIE3 | YCF15_SOLBU
METLVSSIFWTLAPWKNMLLLKHGRIEILDQNTMYGWYELPKQEFLNSKQPVQIFTTKKY
WILFRIGPERRRKAGMPTGVYYIEFTR
>Q2MIE6 | RK2_SOLBU
MAIHLYKTSTPSTRNGTVDSQVKSNPRNNLIYGQRRCGKGRNARGIITARHRGGGHKRLY
RKIDFRRNEKDIYGRIVTIEYDPNRNAYICLIHYGDGEKRYILHPRGAIIGDTIVSGTEV
PIKMGNALPLTDMPLGTAIHNIEITLGKGGQLARAAGAVAKLIAKEGKSATLKLPSGEVR
LISKNCSATVGQVGNVGVNQKSLGRAGSKRWLGKRPVVRGVVMNPVDHPHGGGEGRAPIG

RKKPTTPWGYPALGRRSRKRNKYSDNLILRRRSK
>Q2MIE8 | RK22_SOLBU
MLKKKKTEVYALGEHISMSADKARRVIDQIRGRSYEETLMILELMPYRACYPILKLVYSA
AANASYNMGSSETNLVISKAEVNEGTTVKKLKPRARGRSFPIKRSTCHITIVMKDISLDD
EYGEMSSLKKTRWKKKSTAMTYRDMYNSGGLWDKK
>Q2MIF1 | RK14_SOLBU
MIQPQTHLNVADNSGARELMCIRIIGASNRRYAHIGDVIVAVIKEAVPNMPLERSEVVRA
VIVRTCKELKRDNGMIIRYDDNAAVVIDQEGNPKGTRIFGAIARELRELNFTKIVSLAPE
VL
>Q2MIF6 | RPOA_SOLBU
MVREKVTVSTRTLQWKCVESRTDSKRLYYGRFILSPLMKGQADTIGIAMRRALLGEIEGT
CITRVKSEKVPHEYSTITGIQESVHEILMNLKEIVLRSNLYGTSDASICVKGPGYVTAQD
IILPPYVEIVDNTQHIASLTEPIDFCIGLQIERNRGYLIKTPHNFQDGSYPIDAVFMPVR
NANHSIHSYGNGNEKQEILFIEIWTNGSLTPKEALHDASRNLIDLFIPFLHMEEDNLYLQ
DNQHTVPLSPFTFHDKLAKLIKNKKKIALKSIFIDQSELSSRIYNCLKMSNIYTLLDLLN
NSQEDLMKIEHFRSEDIKQILGILEKYFVIDLAKNKF
>Q2MIH9 | RBL_SOLBU
MSPQTETKASVGFKAGVKEYKLTYYTPEYQTKDTDILAAFRVTPQPGVPPEEAGAAVAAE
SSTGTWTTVWTDGLTSLDRYKGRCYRIERVVGEKDQYIAYVAYPLDLFEEGSVTNMFTSI
VGNVFGFKALRALRLEDLRIPTAYVKTFQGPPHGIQVERDKLNKYGRPLLGCTIKPKLGL
SAKNYGRAVYECLRGGLDFTKDDENVNSQPFMRWRDRFLFCAEALFKAQTETGEIKGHYL
NATAGTCEEMMKRAVFARELGVPIVMHDYLTGGFTANTTLAHYCRDNGLLLHIHRAMHAV
IDRQKNHGIHFRVLAKALRMSGGDHIHSGTVVGKLEGERDITLGFVDLLRDDFIEQDRSR
GIYFTQDWVSLPGVLPVASGGIHVWHMPALTEIFGDDSVLQFGGGTLGHPWGNAPGAVAN
RVALEACVKARNEGRDLAREGNEIIREASKWSPELAAACEVWKEIVFNFAAVDVLDK
>Q2MIJ7 | RPOC2_SOLBU
MEVLMAERANLVFHNKAIDGTAMKRLISRLIEHFGMAYTSHILDQVKTLGFQQATATSIS
LGIDDLLTIPSKGWLVQDAEQQSLILEKHHHYGNVHAVEKLRQSIEIWYATSEYLRQEMN
PNFRMTDPFNPVHIMSFSGARGNASQVHQLVGMRGLMSDPQGQMIDLPIQSNLREGLSLT
EYIISCYGARKGVVDTAVRTSDAGYLTRRLVEVVQHIVVRRTDCGTARGISVSPRNGIMP
ERIFSQTLIGRVLADDIYMGSRCIATRNQAIGIGLVNRFITFRAQPISIRTPFTCRSTSW
ICRLCYGRSPTHGDLVELGEAVGIIAGQSIGEPGTQLTLRTFHTGGVFTGGTAEHVRAPS
NGKIKFNEDLVHPTRTRHGHPAFLCSIDLYVTIESEDILHNVNIPPKSLLLVQNDQYVES
EQVIAEIRAGISTLNFKEKVRKHIYSDSDGEMHWSTDVYHAPEFTYGNVHLLPKTSHLWI
LLGGPCRSSLVYLSIHKDQDQMNAHSLSGKPRYTSNLSVTNDQARQKLFSSDFYGQKEDR
IPDYSDLNRIICTGQYNLVYSPILHGNSDLLSKRRRNKFIIPLHSIQELENELMPCSGIS
IEIPVNGIFRRNSILAYFDDPRYRRKSSGIIKYGTIETHSVIKKEDLIEYRGVKEFRPKY
QMKVDRFFFIPEEVHILPGSSSIMVRNNSIVGVDTQITLNLRSRVGGLVRVERKKKRIEL
KIFSGDIHFPGETDKISRHTGVLIPPGTGKRNSKEYKKVKNWIYVQRITPSKKRFFVLVR
PVVTYEITDGINLGTLFPPDPLQERDNVQLRIVNYILYGNGKPIRGISDTSIQLVRTCLV
LNWNQDKKSSSCEEARASFVEIRTNGLIRHFLRINLVKSPISYIGKRNDPSGSGLLSDNG
SDCTNINPFSAIYSYSKAKIQQSLNQPQGTIHTLLNRNKECQSLIILSAANCSRMEPFKD
VKYHSVIKESIKKDPLIPIRNSLGPLGTCLPIENFYSSYHLITHNQILVTKYLQLDNLKQ
TFQVIKLKYYLMDENGKIFNPDPCRNIILNPFNLNWSFLHHNYCAETSKIISLGQFICEN

VCIAKNGPPLKSGQVILVQVDSIVIRSAKPYLATPGATVHGHYGETLYEGDTLVTFIYEK
SRSGDITQGLPKVEQVLEVRSIDSISMNLEKRVESWNKCIPRILGIPWGFLIGAELTIAQ
SRISLVNKIQQVYRSQGVQIHNRHIEIIVRQITSKVLISEDGMSNVFSPGELIGLLRAER
MGRALEEAICYRVVLLGITRASLNTQSFISEASFQETARVLAKAALRGRIDWLKGLKENV
VLGGVIPVGTGFKGLVHPSKQHNNIPLETKKTNLFEGEMRDILFHHRKLFDSCLSKKFHD
IPEQSFIGFNDS
>Q2MIK8 | RR12_SOLBU
MPTIKQLIRNTRQPIRNVTKSPALRGCPQRRGTCTRVYTITPKKPNSALRKVARVRLTSG
FEITAYIPGIGHNSQEHSVVLVRGGRVKDLPGVRYHIVRGTLDAVGVKDRQQGRSKYGVK
KPK
>Q2PMN9 | RR15_SOYBN
MVKNSIIPVISQEKKEKNPGSVEFQIFKFTDRIRRLTSHFELHRKDYLSQRGLRKILGKR
QRLLSYLSKKDRIRYKKLINQFDIRESQIR
>Q2PMP7 | RR3_SOYBN
MGQKINPLGFRLGTTQSHDSIWFAQPTNYSENIQEDKKIRDCIKNYIQKNIRISSGVEGI
GQIKIQKRIDLIQVIIYMGFPKLLIEGKSQKIEELQTNMQKKLNCVNRKLNIAIVKVANA
YKHPNIIAEFIAGQLKNRVSFRKAMKKAIELTEQAGTKGVQVQIAGRIDGKEIARVEWIR
EGRVPLQTIRAKIDYCCYTVRTIYGVLGIKVWIFSK
>Q2PMP8 | RK16_SOYBN
MLSPQRTRFRKQHRGRMKGISYRGNHICFGRYALQALEPAWITSRQIEAGRRAMSRNVRR
GGQIWVRIFPDKPVTVRPTETRMGSGKGSPEYWVAVVKPGKILYEMGGVPENIARKAISI
ASSKMPIRTQFIISG
>Q2PMQ2 | RR11_SOYBN
MAKPISKKGSRKNVRIGSRKHTRKIPKGVIHVQASFNNTIVTVTDVRGRVISWSSAGTCG
FKGTRRGTPFAAQTAAGNAIRTVSDQGMQRAEVMIKGPGLGRDAALRAIRRSGILLNFIR
DVTPMPHNGCRSPKKRRV
>Q2PMR3 | RK33_SOYBN
MAKGKDIRVIVILECTGCDKKSVNKESTGISRYITKKNRQNTPSRLELRKFCPRCCKHTI
HAEIKK
>Q2PMS6 | PSBK_SOYBN
MLNIFNLVCICIHSVLYSSSFFSAKLPEAYAFLNPIVDIMPVIPLLFFLLAFVWQAAVSF
R
>Q2PMU5 | RR4_SOYBN
MSRYRGPRFKKIRRLGSLPGLTSKRPTVKSELRNQSRSSKKSQYRIRLEEKQKLRFHYGL
TERQLLKYVRIAGKAKGSTGQVLLQLLEMRLDNILFRLGMAATIPQARQLINHRHVLVNG
HIVDIPSYRCKPQDIITAKDEQKSKTLIQNYLDSAPREKLPNHLTLHPFQYKGLINQIID
NKWVGLKINELLVVEYYSRQT
>Q2QD42 | RK32_CUCSA
MAVPKKRTSISKKRIRKNIWKSKGRRAALKAFSLAKSLSTGNSKSFWGDKSNKY
>Q2TGZ2 | RBL_DUNTE
MVPQTETKTGAGFKAGVKDYRLTYYTPDYVVSETDILAAFRMTPQPGVPPEECDAAVAAE
SSTGTWTTVWTDGLTSLDKYKGRCYDLEPVPGEENQYIAYVAYPIDLFEEGSVTNLFTSI
VGNVFGFKALRALRLEDLRISPAYVKTFVGPPHGIQVERDKLNKYGRGLLGCTIKPKLGL
SAKNYGRAVYECLRGGLDFTKDDENVNSQPFMRWRDRFLFVAEAIYKSQAETGEIKGHYL

NATAGTAEGMLQRAQCAKELGVPIIMHDYLTGGFTANTSLAHYCRDHGLLLHIHRAMHAV
IDRQRNHGIHFRVLAKTLRMSGGDHLHSGTVVGKLEGEREVTLGFVDLMRDNFVEKDRSR
GIYFTQDWCSMPGVMPVASGGIHVWHMPALVEIFGDDACLQFGGGTLGHPWGNAPGAVAN
RVALEACTQARNEGRDLAREGGNVIRSACKWSPELAAACEVWKEIKFEFDTIDKL
>Q2VEB7 | RK2_SOLTU
MAIHLYKTSTPSTRNGTVDSQVKSNPRNNLIYGQRRCGKGRNARGIITARHRGGGHKRLY
RKIDFRRNEKDIYGRIVTIEYDPNRNAYICLIHYGDGEKRYILHPRGAIIGDTIVSGTEV
PIKMGNALPLTDMPLGTAIHNIEITLGKGGQLARAAGAVAKLIAKEGKSATLKLPSGEVR
LISKNCSATVGQVGNVGVNQKSLGRAGSKRWLGKRPVVRGVVMNPVDHPHGGGEGRAPIG
RKKPTTPWGYPALGRRSRKRNKYSDNLILRRRSK
>Q2VED0 | NU4C_SOLTU
MNYFPWLTIIVVFPIFAGSLIFFLPHKGNRVIRWYTICICILELLLTTYAFCYHFQSDDP
LIQLVEDYKWIDFFDFHWRLGIDGLSIGPILLTGFITTLATLAAWPVTRDSRLFHFLMLA
MYSGQIGSFSSRDLLLFFIMWELELIPVYLLLAMWGGKKRLYSATKFILYTAGGSVFLLM
GVLGVALYGSNEPTLNFETSVNQSYPVVLEIIFYIGFFIAFAVKSPIIPLHTWLPDTHGE
AHYSTCMLLAGILLKMGAYGLIRINMELLPHAHSIFSPWLMIIGTIQIIYAASTSLGQRN
LKKRIAYPSVSHMGFIIIGISSLTDTGLNGALLQIISHGFIGAALFFLAGTTYDRIRLVY
LDEMGGIAIPMPKMFTMFSSFSMASLALPGMSGFVAELIVFFGIITGQKYLLIPKLLITF
VMAIGIILTPIYSLSMPRQMFYGYKLFNAPKDSFFDSGPRELFLSISIFLPVIGIGIYPD
FVLSLAVDKVEVILSNFFYR
>Q2VED6 | YCF15_SOLTU
METLVSSIFWTLAPWKNMLLLKHGRIEILDQNTMYGWYELPKQEFLNSKQPVQIFTTKKY
WILFRIGPERRRKAGMPTGVYYIEFTR
>Q2VED8 | RK23_SOLTU
MDGIKYAVFTDKSIRLLGKNQYTSNVESGSTRTEIKHWVELFFGVKVIAMNSHRLPGKSR
RMGPIMGHTMHYRRMIITLQPGYSIPPLRKKRT
>Q2VEE3 | RK16_SOLTU
MLSPKRTRFRKQHRGRMKGISSRGNHISFGKYALQALEPAWITSRQIEAGRRAMTRNARR
GGKIWVRIFPDKPVTLRPAETRMGSGKGSPEYWVAVVKPGRILYEMGGVTENIARRAISL
AASKMPKRTQFIIS
>Q2VEE4 | RK14_SOLTU
MIQPQTHLNVADNSGARELMCIRIIGASNRRYAHIGDVIVAVIKEAVPNMPLERSEVVRA
VIVRTCKELKRDNGMIIRYDDNAAVVIDQEGNPKGTRIFGAIARELRELNFTKIVSLAPE
VL
>Q2VEH3 | NDHK_SOLTU
MVLAPEYSDNKKKNGKNKIETVMNSIQFPLLDRTAPNSVISTTLNDLSNWSRLSSLWPLL
YGTSCCFIEFASLIGSRFDFDRYGLVPRSSPRQSDLILTAGTVTMKMAPSLVRLYEQMPE
PKYVIAMGACTITGGMFSTDSYSTVRGVDKLIPVDVYLPGCPPKPEAVIDAITKLRKKIS
RELYEDRIRSQRANRCFTTNHKFHVRRSIHTGNYDQRVLYQPPSTSEIPTEIFFKYKNSV
SSAELVN
>Q2VEH5 | RR4_SOLTU
MSRYRGPRFKKIRRLGALPGLTNKKPRTGSDLRNQSRSGKKSQYRIRLEEKQKLRFHYGL
TERQLLKYVRIARKAKGSTGQVLLQLLEMRLDNILFRLGMASTIPAARQLVNHRHILVNG
HIVDIPSYRCKPRDIITAKDEQKSRALIQISLDSSPHEELPNHLTLQPFQYKGLVNQIID

SKWVGLKINELLVVEYYSRQT
>Q2VEJ3 | PSBK_SOLTU
MLNTFSLIGICLNSTLYSSSFFFGKLPEAYAFLNPIVDIMPVIPLFFFLLAFVWQAAVSF
R
>Q2WGF1 | RR8_SELUN
MGNDAIDDVTTAPRNASSRGAETVRVSSTDITISIGRVLVEEGFSGNPREHRQGTNRFPV
STSKYQGRTRKARITTRRRVSKPGLRIYPGYKDIPEVSGGIGTATPPTPGGTTTDREARQ
KRIGGEAPRHVWR
>Q31795 | RBL_ANTFO
MSPQTETKAGVGFKAGVKDYRLTYYTPDYETKDTDILAAFRMTPQPGVPPEEAGAAVAAE
SSTGTWTTVWTDGLTSLDRYKGRCYDIEPVAGEENQYIAYVAYPLDLFEEGSVTNMFTSI
VGNVFGFKALRALRLEDLRIPPAYSKTFQGPPHGIQVERDKLNKYGRPLLGCTIKPKLGL
SAKNYGRAVYECLRGGLDFTKDDENVNSQPFMRWRDRFLFVAEAIFKSQAETGEIKGHYL
NATAGTCEEMMKRAHFARELGMPIVMHDYLTGGFTANTTLARYCRDNGLLLHIHRAMHAV
IDRQRNHGIHFRVLAKALRMSGGDHIHSGTVVGKLEGEREVTLGFVDLLRDDYIEKDRSR
GIYFTQDWVSMPGVLPVASGGIHVWHMSALTEIFGDDSVLQFGGGTLGHPWGNAPGAVAN
RVALEACVQARNEGRDLAREGNDIIREASKWSPELAAACEVWKEIKFVFETIDTL
>Q31796 | ACCD_ANTFO
MSLMNWFEDRRKFSGLIGAFIEKATKGYILSERRKDRHIKIDTTKGLWTRCDNCENMLYI
RFLRQNKRICEECGYHLQMSSTERIESLIDRGTWHPMDEDMVARDALKFSDEDSYKNRVL
FYQKRTGLTDAIQTGIGKLNGIPIALGVMDFQFMGGSMGSVVGEKITRLIEYGTRESMPV
IIVCSSGGARMQEGTLSLMQMAKISAVLQIHQAQKKLLYIAILTYPTTGGVTASFGMLGD
VIIAEPKAYIAFAGKRVIEQTLRQKIPDGSQVAESLFDHGLLDLIVPRNLLRGVLSEIFE
LYSSAPCRRSNNS
>Q31886 | RBL_BOUGL
MSPQTETKASVGFKAGVKEYKLTYYTPEYETKDTDILAAFRVTPQPGVPPEEAGAAVAAE
SSTGTWTTVWTDGLTSLDRYKGRCYHIEPVPGEEDQFIAYVAYPLDLFEEGSVTNMFTSI
VGNVFGFKALRALRLEDLRIPIAYVKTFEGPPHGIQVERDKLNKYGRPLLGCTIKPKLGL
SAKNYGRACYECLRGGLDFTKDDENVNSQPFMRWRDRFLFCAEAIYKAQAETGEIKGHYL
NATAGNCEEMIKRAVFARELGVPIVMHDYLTGGFTANTSLSHYCRDNGLLLHIHRAMHAV
IDRQKNHGMHFRVLAKALRMSGGDHIHSGTVVGKLEGERDITLGFVDLLRDDYIEKDRSR
GIYFTQDWVSMPGVLPVRSGGIHVWHMPALTDIFGDDSVLQFGGGTLGHPWGNAPGAVAN
RVALEACVKARNEGRNLASEGNEIIREAAKWSPELAAACEVWKEIKFIFAAVDTL
>Q32616 | YCF66_MARPO
MNHMELGPSTILGVGLIIIGLFLYALKLREPYVSRDYDFFFSCIGLLCGGILFFQGWRLD
PILLLSQILLSGTTIFFIAESLYLRKNLNFVKSKKKYINLAKKNIYKYIYENFKLKKKWN
ELNYTRHIFYKKKKH
>Q32701 | RBL_NOTSU
MSPQTETKASVGFKAGVKDYKLTYYTPDYETKDTDILAAFRVTPQPGVPPEEAGAAVRAE
SSTGTWTTVWTDGLTSLDRYKGRCYHIEPVAGEENQYIAYVAYPLDLFEEGSVTNMFTSI
VGNVFGFKALRALRLEGLRIPPAYSKTFQGPPHGIQVERDKLNKYGRPLLGCTIKPKLGL
SAKNYGRAVYECLRGGLDFTKDDENVNSQPFMRWRDRFVFCAEAIDKAQAETGEIKGHYL
NATAGTCEEMIKRAVFARELGVPIVMHDYLTGGFTANTSLAHYCRDNGLLLHIHRAMHAV
IDRQKNHGIHFRVLAKALRMSGGDHIHAGTVVGKLEGERGITLGFVDLLRDDFIEKDRSR

GIYFTQDWVSLPGVLPVASGGIHVWHMPALTEIFGDDSVLQFGGGTLGHPWGNAPGAVAN
RVALEACVQARNEGGDLAREGNEIIREASKWSPELAAACEVWKEIKFEFQAMDTL
>Q32RF8 | RK21_ZYGCR
MKTYAIIEAGGEQLQVEPGRFYNIRHLSLRGVNFWGQNTKLLLYRVLMIRHQSATVLGNP
WVQNATVKGRILDARRDDKLVIYKMRAKKKTRRKRGHRQGLTRFVVDAICLNGKVLLD
>Q32RG2 | RPOC2_ZYGCR
MAELKNRIFYNQVMDKSAIKQLIIRLVACLGRVCTAHILDQLKTLGFQYSTQTGISLGID
DLLASPLKNWILQDAENEANVSQEYCRHGYIHAVERLRQIVETWHTTSEFLKREMTVSFN
ILDGFNPVHMMSFSGARGNVSQVHQLVGMRGLISDPQGNIIDLPIQSNLREGLSLTEYII
SCYGARKGVVDTAIRTADAGYLTRRLVEVAQHVVIRNVDCNTYEGIILRSIRTRQGNAYL
TQENRIIGRVLARPLYFGKRCIAVRNQDIGPDLAAKLSIISPQAILVRSPITCKTTDWVC
QLCYGWGVNQGKMVSLGEAIGVVAGQSIGEPGTQLTLRTFHTGGVFTGDIANHLRAPFNG
IAHFETHNCKPTRNRHGRLVWKCLQDLTITVIGQGKKHSLNVPSQSLLLINNNQYVESKQ
VIAEVRASIAPIKEKVQRNIYSYLQGEVVHTRSALRLSNAFSGTILLIHHNPNTGHLWIW
SGKLYQLSGQQASSIYTSEDFIQTNITVANKRYLKFDTHKISNKKVLKSVLIGKFTRFKQ
VKSIQTGTVRSVLIQKPDTLRMVVLSSVDQLEITMHKTPYIMEPLLTQKISNNILNLTSY
PKSSTLISKQKNTVSSFASIHIKRPTIGFQFQLPIVPKIRCYSLGLLGKLQRPLQYNLVL
SSPTQPIFIDRYYNTFTNWCYLNEHGNSYNLYGLQMIVLDKKLYNNNSISGLFNTYRNIP
KIGQLICKGTFVQQTTQLSESGKIVSIFKHKIVLRLSQPYLLAPGTFIHPDCYDVINRGD
IVITMMYEQLRTTDIIQGLPKAEQLLEARSFNEVVLKLENDFVMLTERIARQLRSLSRSY
MLSTKESTKHSQIDLVNRIQTVYLSQGVRIVDKHIEIIVRQMSSKVMLVENGDPLAVSSG
GLICLPLPPIGSFLPENWIEIPLSYVIDNHTFLPRELVELTRAQKINYVIQNPVAYKPVL
LGITKASLNTNSFISEASFQQTARVLSKSAIKNRVDWIRGLQENVLFGRMIPAGTGCREI
SSQLQYGNIFQKKWAQHTKWKLFVNSFGLNLSCLQIYLHFQAYCLESTNEQPQSNLHVHN
SRIFPFTTICSILKS
>Q32RK7 | CHLL_ZYGCR
MKIAVYGKGGIGKSTTSCNISIALARRGKRVLQIGCDPKHDSTFTLTGFLIPTIIDTLQS
KDYHYEDVWPEDVIYKGYGGVDCVEAGGPPAGAGCGGYVVGETVKLLKELNAFYEYDVIL
FDVLGDVVCGGFAAPLNYADYCIIITDNGFDALFAANRIAASVREKARTHPLRLAGLVGN
RTSKRDLIDKYVEACPMPVLEVLPLIEDIRVSRVKGKTLFEMAESEPSLNYVCEFYLNIA
DQILSQPEGVVPKEVPDRELFSLLSDFYLNPSSSRSDMQLEDNSLDFVMV
>Q32RM2 | ODPB_ZYGCR
MAEVLLFEALRQGLQEEMDRDPRVMVMGEDVGHYGGSYKVTKGFAERYGDLRLLDTPIAE
NSFTGMAIGAAMTGLRPVVEGMNMGFLLLAFNQIANNAGMLHYTSGGNFTIPIVIRGPGG
VGRQLGAEHSQRLESYFQSVPGLQMVACSTPYNAKGLIKSAIRSDNPIIILFEHVLLYNLK
EDLAEEEYLVCLEKAEVVRPGNDITILTYSRMRHNVLQATKSLVYKGYDPEIIDIVSLKP
FDLGTIGASVCKTHKVLIVEECMRTGGIGATLRAAIMEHFFDYLDAPILCLSSQDVPTPY
SSPLEELTVIQPNQIIQVVEQLCEN
>Q32RM8 | RPOA_ZYGCR
MNLHRISSEPKCKCLNHEIQNARLHYGRFAVYPLFPGQAITIGTAIRRALLGEVHSTCIT
SAYVVGASHEYSTLKGIRESIHDILLNLKDIVFKSDTLERQEGILLFNGPGIVTAQHIKL
PPVVQVVDNTQYIARLEMPTSIEIHITLENTKTCTSLNTMPTTKGRFILDAALKPVRNMN
YSIHSLGEGDMRQEMLVLEVWTNGSLTPQEVISQASQNLNDLLKPLLKVERYFQAKQNIH
KKRMFTPTQSENLNMDQIEMIGGNKLIDQPLLPADSHLNSSLSLNIDNLVHLEGISIDDL

QISVRASNCLKKVGIYTIRQLLSYTQQDLFQIKNLGKKSVEQIVIAIRKSYGHILG
>Q32RN4 | RK14_ZYGCR
MIQPQSYLNVADNSGARKLMCIRVLGSSNRKYAHIGDMVIAVVKETVPNMPLKKSEIVRA
VIVRTRKGLKRDNGMVLRFDDNAAVVINQEGNPRGTRVFGPVARELRDLNFTKIVSLAPE
VL
>Q32RN7 | RR19_ZYGCR
MTRSLKKGPFVADHLLNKIENLNAKEEKKVIITWSRASTIVPAMIGHTIAVHNGREHLPV
FITELMIRHKLGEFASTRTFRSHLKKSDKKSRR
>Q32RN8 | RK2_ZYGCR
MGIRSYKAYTPGTRNRSVSQFEEIVQSKPEKQLTSGQHRKKGRNNRGVITSRHRGGGHKR
LYRHIDFKRDKYGMLGRIVSIEYDPNRNARICLISYQDGEKRYILHPRGISIGDQILSDT
HAPISPGNALPLTNMPLGTTIHNIELQPGKGGQVARAAGAIAKIIAKEGKFATLRLPSGE
VRLVLQQCLATIGQVGNVDANNQSIGKAGSKRWLGRRPTVRGVVMNAADHPHGGGEGRAP
IGRKRPLTPWGRPTLGRKSRSRHKYSEALILRRRKNA
>Q32RP7 | RR7_ZYGCR
MSRRGTAEKRPAMPDPIYQNRLVNMLVNRIVKNGKKSLAYSILYKAMKDIKQSTQKNPLS
VIRQAVRRTTPNVAVKARRRGGSTYQVPVEIKSAQGRALAIRWILNAAKKRNGRSMAFKL
SSEIIDAARQTGNAIRKREETHRMAEANRAFAHFR
>Q32RP8 | RR16_ZYGCR
MVKLRLKRYGRKQQPTYRIIAIDVKSRRQGRALKEVGFYDPRKDQTHLDVATIITFIQQG
AQPTDTVSHILNRAGVFEQIHAMSIHE
>Q32RP9 | CHLB_ZYGCR
MKLAYWMYAGPAHIGTLRVASSFRNVHAIMHAPLGDDYFNVMRSMLERERDFTPVTASIV
DRHVLARGSQEKVVENITRKDKEERPDLIVLTPTCTSSILQEDLQNFVDRASIASNSDVI
LADVNHYRVNELQAADRTLEQVVRYYLDKARRQGTLSQSITEKPSVNIIGMFTLGFHNQH
DCKELKRLLQDLGIQVNEVIPEGGSVENLRNLPKAWLNLVPYREVGLMTALYLEKEFGMP
YVATTPMGIVGTAEFVRQIQSHINKWAPMFLGKLVDYEPYIDQQTRFISQAAWFSRSIDC
QNLTGKKVVVFGDTTHAASMTKILAREMGIHVVCAGTYCKHDADWFKEQVQGYCDEILVT
DDHTQVGDMIARIEPAAIFGSQMERHIGKRLDIPCGVISAPVHIQNFPLGYRPFLGYEGT
NQIADLVYNSFTLGMEDHLLEMFGGHDTKEVITKSLSTDTDFTWDSESQLELTKIPGFVR
AKIKRNTEKFARQNGVAKITVEVMYAAKEALNA
>Q32RQ1 | RBL_ZYGCR
MSPQTETKAGVGFKAGVKDYRLTYYTPDYETKETDILAAFRMTPQAGVPPEEAGAAVAAE
SSTGTWTTVWTDGLTSLDRYKGRCYDIEPVPGEENQYIVYVAYPLDLFEEGSVTNLFTSI
VGNVFGFKALRALRLEDLRIPPAYSKTFQGPPHGIQVERDKINKYGRPLLGCTIKPKLGL
SAKNYGRAVYEVLRGGLDFTKDDENVTSQPFMRWRDRFLFCAEAIYKAQAETGEIKGHYL
NATAGTCEEMLKRAEYAKELGVPIVMHDYLTGGFTANTSLAHYCRDNGLLLHIHRAMHAV
IDRQKNHGIHFRVLAKALRMSGGDHIHSGTVVGKLEGEREVTLGFVDLLRDDYIEKDRSR
GIYFTQDWVSMPGVLPVASGGIHVWHMPALTEIFGDDSVLQFGGGTLGHPWGNAPGAVAN
RVALEACVQARNEGRDLAREGNDVIREATKWSPELAAACEVWKEIKFEFDTIDTL
>Q32RQ9 | RR12_ZYGCR
MPTIQQLIRNTRQPTQNRTKSPALKACPQRRGVCTRVYTTTPKKPNSALRKVARVRLTSG
FEVTAYIPGIGHNLQEHSVVLIRGGRVKDLPGVRYHIIRGTLDAVGVKDRHQGRSKYGVK
RPK

>Q32RS1 | RR16_STAPU
MVKLRLKRYGRKQQPTYRIIAIDVRCRRDGKALKEVGFYDPLKDKTQLNVPAILTLLQHG
AQPTDTVSHILQKAGVFEK
>Q32RS5 | RR14_STAPU
MAKKSMIERDKKRKFLSAKYSIQRQQLKEQINQSLSLDEKAHLYRKLQSLPRNSAPTRIT
RRCFVTGRPKAVYRDFGLSRHVLREMAHACLLPGVIKASW
>Q32RT8 | RK33_STAPU
MGKNKDVRITITLECTNCPQNKLKRYPGISRYTTQKNRRNTPNRLELKKFCPHCSLHTLH
KEIKK
>Q32RT9 | RK20_STAPU
MTRVKRGYVAVRRRNRVLERAAGFRGAHSRLFRPAKQQVMKALVASKRDRDKRKRDFRKL
WITRINAAVRKQGMSYSRFIHQLYQSKILINRKMIAQIAVLDENGFTSIVNNI
>Q32RU8 | RR11_STAPU
MARPSKKISLRKGKKRAPKGVIHVQASFNNTIVTVTDVRGQTMSWSSAGSCGFKGAKKST
PFAAQTAAENAISLLIDQGMRQAEVMISGPGPGRDTALRAIKNSGLVISFVRDVTPMPHN
GCRPSSQRRV
>Q32RV2 | RK14_STAPU
MIQPQSYLNVADNSGARRLMCIRVLGSSNRKYANIGDMIIAVVKEAVPNMPLKKSEVVRA
VIVRTCKGIKRNNGMILRFDDNAAVVVNQEGNPRGTRVFGPVARELRDFNFTKIVSLAPE
VL
>Q32RV4 | RR3_STAPU
MGQKIHPLGFRLGVTQSHLSNWCVQPSQYSELLQEDEKLRGCIKEYVRKYVRTAENYGGI
DRIEIQRKTSAIKVEIHTGFPKLFVEKLGPRLKEIRREMQNTLQKSKHLKFIISLAEVEK
PYAKATILAEYIAVQVERRVAFRKTIKKAIQLAKEQGNVKGIKIQIAGRLNGAEIARSEW
AREGRVPLQTLRAQIDYCHYPAHTIYGVLGIRVWIFQEK
>Q32RV8 | RK23_STAPU
MIDYIKSPVLKFKLIRLLEKNQYTFDVDPKATKTDVKYWVENFFGVKVIGMNSHRPPRKM
KRMGPTIGYPVRYKRMIVTLRAGDSIPLF
>Q32RW6 | PSBK_STAPU
MFNAYLDTVLDLSANGTVILAKLPEAYAIFDPIVDVMPIIPVFFLLLAFVWQAAVSFR
>Q32RY2 | RPOC2_STAPU
MARLTAEFGDRIYYNQVMDRGAMRQLIGRLIIYLGSNCTSQILDQLKTLGFRHATQAGIS
LGVDDLITTPSKTWLIRDAEYQAYTSEEQYRRGSIHAVEKLRHVVETWHTTSEYLKREMT
SHFRITDPLNPVHIMSFSGARGNVSQVHQLVGMRGLMANPQGNIIDLPIQSNLREGLSLT
EYIISCYGARKGVVDTAVRTADAGYLTRRLVEVVQHVVIRNPDCETNQGIRLHSIRDRKT
TRDAGGNSLLSLQERLIGRVLARNVFLGKRCIAIKNQDLAPDLVNSLVNFPKALLSPGSE
GGHCEILVRSPLTCKSMPRACQKCYGWDLSCGNLVEIGAAIGIVAGQSIGEPGTQLTLRT
FHTGGVFTGGVAEHVRAPFNGIVHFSISSSTHKTKTIQPTRNRHGRPAWTCPEAFSVIIE
DRIGKKKVLNVPQYSLLLVKNHQYVQSRQIIAEVRASIAPLKEKIEKNIYSHLQGEVFYN
PIGLRASCLKQRHFLHFLCSASRMNLWIEFASIRSYLSPLVEKTSYIWIFSGRLNKFFNA
TLNLSFFYQTQDYIQRDLPIGIQNHVFNYSKVFLLSIYNRQRSVTFSVSRRDIRLGRKKK
FTDYIPLSRISTDKIFSKHTGSIHTISEQNEWILTLSFVDQFSKKMFSSVQTPGTSKVSE
KSEEQIIDFQEQLILNYPLSKTTIFLKEPSILAKNYSIAEKDSCIGLLGEFFIFLNELSI
VSVRTFISTSQINLFLKKNVFSLLCITHGNTFKGKEKIFFINDSKNIYNIKNWLISVNFL

IWCHFSNRILNKNQTLATIFLSNRSLRNEKDLQVKEFKIGQSICPSRDLGEKIFEPFTHS
ILPESGHLLSLNEGNLLIRLAKLYLIPTGGIAHTFHKQTINEGDTLVTLAYERLKASDII
QGLPKAEQLLEARVGNQVVINLYMRFEILVARIKEQLRNYIGSLTQMDLMLSQISVNRAS
KALEYSQLETVDQVQKVYLSQGVYISDKHFEVIVRQMSSKIAIVENTDFTAFSPKAVIRT
AFPYDVLGVFFPGEILDISKAQKMNRVLYKPLPCKPVLLGITQASLNANSFLSEASFERT
ITVLSRSALQGRMDWLKGLKENVLLSKMIPAGTGLKQAPVLLSPNGTGTLFSNNEQNIFQ
KKNVTSLSKSKGQKVQIRKIFSFQKSPQRLSFLYQETSSSNIRRKIQPTVCLCKPFITKR
NWLHISLKQLTEQSLNYETKKFCLQELTNNFIPRKKENIFYLKKKTIKTT
>Q32RZ7 | CHLL_STAPU
MKIAVYGKGGIGKSTTSCNISIALARRGKRVLQIGCDPKHDSTFTLTGFLIPTIIDTLQS
KDYHYEDVWPEDVIYKGYGGVDCVEAGGPPAGAGCGGYVVGETVKLLKELNAFYEYDVIL
FDVLGDVVCGGFAAPLNYADYCIIITDNGFDALFAANRIAASVREKARTHPLRLAGLVGN
RTSKRDLIDKYVEACPMPVLEVLPLIEDIRVSRVKGKTLFEMAESDPSQNYVCDFYLNIA
DQILSKSEGVVPREVPDRELFSLLSDFYLNQPSSSTNNSETNNLDFLMV
>Q32S07 | RK21_STAPU
MNTYAIIEAGGEQLQVQPGRFYDIRLNVPVTEFWENRKIVFSRVLMIRSESNTFLGKPWL
EHATVNGRIFHPRRGNKLIIYKMRPKKHTSKKNGHRQTFMRLIIDSIFFNNQNL
>Q332R5 | RK2_LACSA
MAIHLYKTSTPSTRNGAVDSKVKSNPRNNLIYGQHHCGKGRNARGIITAGHRGGGHKRLY
RKIDFRRNEKDIYGRIVTIEYDPNRNAYICLIHYRDGEKRYILHPRGAIIGDTIVSGTEV
PIKMGNALPLTDMPLGTAIHNIEITLGKGGQLARAAGAVAKLIAKEGKSATLKLPSGEVR
LISKNCSATVGQVGNVGVNQKSLGRAGSKRWLGKRPVVRGVVMNPVDHPHGGGEGRAPIG
RKQPTTPWGYPALGKRSRKRNKYSDNLILRRRSK
>Q332R9 | RR7_LACSA
MSRRGTAEEKTAKSDPIYRNRLVNMLVNRILKHGKKSLAYQIIYRAVKKIQQKTETNPLS
VLRQAIHGVTPGIAVKARRVGGSTQQVPIEIGSTQGKALAIRWLLAASRKRPGRNMAFKL
SSELVDAAKGSGDAIRKREETHKMAESNRAFAHFR
>Q332S0 | RR15_LACSA
MVKNSFISIIFQEQEENKENRGSVEFQVVSFTNKIRKLTSHLELHKKDYLSQRGLRKILG
KRQRLLAYLSKKNRARYKELIGQLNIRERKTR
>Q332S9 | RK32_LACSA
MAVPKKRTSISKKRIRKNIWKRKGYWAALKALSLGKSLSTGNSKSFFVRQTNKS
>Q332T6 | RR19_LACSA
MTRSLKKNPFVANNLLKKINKLNTKAEKEIIITWSRASTIIPIMVGHTIAIHNGKEHLPI
YITDRMVGHKLGEFAPTLNFRGHAKSDNRSRR
>Q332U5 | RPOA_LACSA
MVREKITVSTRTLQWKCVESAADSKRLLYGRFILSPLMKGQADTIGIAMRRALLGEIEGT
CITRAKSEKISHEYSTIMGIQESVHEILMNLKEIVLRSNLYGTCEASICVRGPGYVTAQD
IILPPYVEILDNTQHIASLTEPIELVIGLQIEKNRGYLIKAPNTFQDGSYPIDPVFMPVR
NANHSIHSYENGNKEILFLEIWTNGSLTPKEALYEASRNLIDLLIPFLHTKEENLNLEGN
QHMVPLPPFTFYDKLAKLTKNKKKMALKSIFIDQSELPPRIYNCLKRSNIYTLLDLLNNS
QEDLMKMEHFRIEDVKQILGILEKNFVIDLPKNKF
>Q332V2 | CLPP_LACSA
MPIGVPKVPFRSPGEEDASWVDIYNRLYRERLLFLGQEVDSEISNQLIGLMIYLSIEDDT

KDLYLFINSPGGWVIPGVALYDTMQFVQPDVHTICMGSAASMGSFILVGGEITKRLAFPH
ARVMIHQPAGSFSEVATGEFILEVGELLKLRETLTRVYVQRTGKPLWVVSEDMERDVFMS
ATEAQAYGIVDLVAVE
>Q332V5 | RK20_LACSA
MTRIRRGYIARRRRTKIRLFASSFRGAHSRLTRTITQQKIRALVSAHRDRDKQKINFRRL
WITRINAAIRERGVCYSYSRLINGLYKRQLLLNRKILAQIAISNRNCLYMISNEIIKEVG
WKESTG
>Q332V7 | RK33_LACSA
MAKGKDVRIPVLLECTACVRNGVNVNKASTGISRYITQKNRHNTPNRLELRKFCPYCYKH
TIHGEVKK
>Q332X6 | RR4_LACSA
MSRYRGPRFKKIRRLGALPGLTNKRPRAGSDLRNQSRSGKKSQYRIRLEEKQKLRFHYGL
TERQLLKYVRIAGKAKGSTGQVLLQLLEMRLDNILFRLGMAPTIPGARQLVNHRHILVNG
RIVDIPSYRCKPRDTIAARDEQKSKVLIQNSLDSSPHEELPNHLTLQPFQYKGLVNQIID
SKWVGLKINELLVVEYYSRQT
>Q332Z6 | RR16_LACSA
MVKLRLKRCGRKQRAVYRIVAIDVRSRREGRDLRKVGFYDPIKNQTYLNVPAILYFLEKG
AQPTGTVQDILKKAEVFKELCPNQTKFN
>Q33301 | YCF70_MAIZE
MVLYALFYVFLVLFIFFDSFKQESNKLELSGKEERKLGNGEDRLTSDSYLLFFLSYYSLL
LLSSDRRSL
>Q33BX4 | RK32_NICTO
MAVPKKRTSTSKKRIHKNIWKRKGYSIALKAFSLAKSLSTGNSKSFFVRQTKINK
>Q33BY9 | RK23_NICTO
MDGIKYAVFTDKSIRLLGKNQYTSNVESGSTRTEIKHWVELFFGVKVIAMNSHRLPGKSR
RMGPIMGHTMHYRRMIITLQPGYSIPPLRKKRT
>Q33BZ2 | RK22_NICTO
MLKKKKTEVYALGEHISMSADKARRVIDQIRGRSYEETLMILELMPYRACYPILKLVYSA
AANASYNMGSSEANLVISKAEVNGGTTVKKLKPRARGRSFPIKRSTCHITIVMKDISLDD
EYVEMYSLKKTRWKKKPTAMPYRDMYNSGGLWDKK
>Q33BZ5 | RK16_NICTO
MLSPKRTRFRKQHRGRMKGISHRGNHISFGKYALQALEPAWITSRQIEAGRRAMTRNARR
GGKIWVRIFPDKPVTLRPAETRMGSGKGSPEYWVAVVKPGRILYEMGGVTENIARRAISL
AASKMPIQTQFIIS
>Q33BZ6 | RK14_NICTO
MIQPQTHLNVADNSGARELMCIRIIGASNRRYAHIGDVIVAVIKEAIPNMPLERSEVVRA
VIVRTCKELKRDNGIIIRYDDNAAVVIDQEGNPKGTRIFGAIARELRELNFTKIVSLAPE
VL
>Q33C07 | CLPP_NICTO
MPIGVPKVPFRSPGEEDASWVDVYNRLYRERLLFLGQEVDSEISNQLIGLMVYLSIEDET
KDLYLFINSPGGWVIPGVAIYDTMQFVRPDVHTICMGLAASMGSFILVGGEITKRLAFPH
ARVMIHQPASSFYEAQTGEFVLEAEELLKLRETLTRVYVQRTGKPLWVVSEDMERDVFMS
ATEAQAYGIVDLVAVE
>Q33C11 | RK33_NICTO

MAKGKDVRVTVILECTSCVRNSVDKVSRGISRYITQKNRHNTPNRLELKKFCPYCYKHTI
HGEIKK
>Q33C26 | RBL_NICTO
MSPQTETKASVGFKAGVKEYKLTYYTPEYQTKDTDILAAFRVTPQPGVPPEEAGAAVAAE
SSTGTWTTVWTDGLTSLDRYKGRCYRIERVVGEKDQYIAYVAYPLDLFEEGSVTNMFTSI
VGNVFGFKALRALRLEDLRIPPAYVKTFQGPPHGIQVERDKLNKYGRPLLGCTIKPKLGL
SAKNYGRAVYECLRGGLDFTKDDENVNSQPFMRWRDRFLFCAEALYKAQAETGEIKGHYL
NATAGTCEEMIKRAVFARELGVPIVMHDYLTGGFTANTSLAHYCRDNGLLLHIHRAMHAV
IDRQKNHGIHFRVLAKALRMSGGDHIHSGTVVGKLEGERDITLGFVDLLRDDFVEQDRSR
GIYFTQDWVSLPGVLPVASGGIHVWHMPALTEIFGDDSVLQFGGGTLGHPWGNAPGAVAN
RVALEACVQARNEGRDLAQEGNEIIREACKWSPELAAACEVWKEIVFNFAAVDVLDK
>Q33C48 | RPOC2_NICTO
MEVLMAERANLVFHNKAIDGTAMKRLISRLIDHFGMAYTSHILDQVKTLGFQQATATSIS
LGIDDLLTIPSKGWLVQDAEQQSLILEKHHHYGNVHAVEKLRQSIEIWYATSEYLRQEMN
PNFRMTDPFNPVHIMSFSGARGNASQVHQLVGMRGLMSDPQGQMIDLPIQSNLREGLSLT
EYIISCYGARKGVVDTAVRTSDAGYLTRRLVEVVQHIVVRRTDCGTARGISVSPRNGMMP
ERFFIQTLIGRVLADDIYMGPRCIATRNQDIGIGLVNRFITFRAQPISIRTPFTCRSTSW
ICRLCYGRSPTHGDLVELGEAVGIIAGQSIGEPGTQLTLRTFHTGGVFTGGTAEHVRAPS
NGKIKFNEDLVHPTRTRHGHPAFLCSIDLYVTIESEDILHNVNIPPKSLLLVQNDQYVES
EQVIAEIRAGISTLNFKEKVRKHIYSDSDGEMHWSTDVYHAPEFTYGNVHLLPKTSHLWI
LLGGPCRSSLVYLSIHKDQDQMNAHFLSGKRRYTSNLSVTNDQARQKLFSSDFSVKKEDR
IPDYSDLNRIICAGQYNLVYSPILHENSDLLSKRRRNKFIIPLHSIQELENELMPCSGIS
IEIPVNGIFRRNSILAYFDDPRYRRKSSGIIKYGTVETHSVIKKEDLLEYRGVKEFSPKY
QMKVDLFFFIPEEVHILPGSSSIMVRNNSIVGVDTQITLNLRSRVGGLVRVERKKKQIEL
KIFSGDIHFPGETDKISRHTGVLIPPGTGKRNSKESKKVKNWIYVQRITPSKKRFFVLVR
PVVTYEITDGINLATLFPPDPLQERDNVQLRIVNYILYGNGKPIRGISDTSIQLVRTCLV
LNWNQDKKSSSCEEARASFVEIRTNGLIRHFLRINLVKSPISYIGKRNDPSGSGLLSDNG
SDCTNINPFSSIYSYSKAKIQQSLNQPQGTIHTLLNRNKECQSLIILSAANCSRMGPFKD
VKYHSVIKESIKKDPLIPIRNSLGPLGTSLPIENFYSSYHLITHNQILVTNYLQLDNLKQ
TFQVIKFHYYLMDENGKIFNPDPCRNIILNPFNLNWYFLHHNYCEETSKIISLGQFICEN
VCIAKNGPPLKSGQVILVQVDSIVIRSAKPYLATPGATVHGHYGETLYEGDTLVTFIYEK
SRSGDITQGLPKVEQVLEVRSIDSISMNLEKRIEGWNKCITRILGIPWGFLIGAELTIAQ
SRISLVNKIQQVYRSQGVQIHNRHIEIIVRQITSKVLVSEDGMSNVFSPGELIGLLRAER
MGRALEEAICYRVVLLGITRASLNTQSFISEASFQETARVLAKAALRGRIDWLKGLKENV
VLGGVIPVGTGFKGLVHPSKQHNNIPLETKKKNLFEGEMRDILFHHRKLFDSCLSKNFHD
IPEQSFIGFNDS
>Q36996 | YCF72_ORYSJ
MGAFPSPPPWGWSTGFITTPLTTGRLPSQHLDPALPKLFWFTPTLPTCPTVAKQFWDTKR
TSPDGNLKVADLPSFAISFATAPAALANCPPLPRVISMLCMAVPKGISVEVDSSFLSKNP
FPNCTSFFQSIRLSRCI
>Q37082 | YCF72_MAIZE
MGAFPSPPPWGWSTGFITTPLTTGRLPSQHLDPALPKLFWFTPTLPTCPTVAKQFWDTKR
TSPDGNLKVANLPSFAISFATAPAALANCPPLPRVISMLCMAVPKGISVEVDSSFFSKNP
FPNCTSFFQSIRLSRCI

>Q38933 | LCYB_ARATH
MDTLLKTPNKLDFFIPQFHGFERLCSNNPYHSRVRLGVKKRAIKIVSSVVSGSAALLDLV
PETKKENLDFELPLYDTSKSQVVDLAIVGGGPAGLAVAQQVSEAGLSVCSIDPSPKLIWP
NNYGVWVDEFEAMDLLDCLDTTWSGAVVYVDEGVKKDLSRPYGRVNRKQLKSKMLQKCIT
NGVKFHQSKVTNVVHEEANSTVVCSDGVKIQASVVLDATGFSRCLVQYDKPYNPGYQVAY
GIVAEVDGHPFDVDKMVFMDWRDKHLDSYPELKERNSKIPTFLYAMPFSSNRIFLEETSL
VARPGLRMEDIQERMAARLKHLGINVKRIEEDERCVIPMGGPLPVLPQRVVGIGGTAGMV
HPSTGYMVARTLAAAPIVANAIVRYLGSPSSNSLRGDQLSAEVWRDLWPIERRRQREFFC
FGMDILLKLDLDATRRFFDAFFDLQPHYWHGFLSSRLFLPELLVFGLSLFSHASNTSRLE
IMTKGTVPLAKMINNLVQDRD
>Q39161 | NIR_ARATH
MTSFSLTFTSPLLPSSSTKPKRSVLVAAAQTTAPAESTASVDADRLEPRVELKDGFFILK
EKFRKGINPQEKVKIEREPMKLFMENGIEELAKKSMEELDSEKSSKDDIDVRLKWLGLFH
RRKHQYGKFMMRLKLPNGVTTSAQTRYLASVIRKYGEDGCADVTTRQNWQIRGVVLPDVP
EILKGLASVGLTSLQSGMDNVRNPVGNPIAGIDPEEIVDTRPYTNLLSQFITANSQGNPD
FTNLPRKWNVCVVGTHDLYEHPHINDLAYMPANKDGRFGFNLLVGGFFSPKRCEEAIPLD
AWVPADDVLPLCKAVLEAYRDLGTRGNRQKTRMMWLIDELGVEGFRTEVEKRMPNGKLER
GSSEDLVNKQWERRDYFGVNPQKQEGLSFVGLHVPVGRLQADDMDELARLADTYGSGELR
LTVEQNIIIPNVETSKTEALLQEPFLKNRFSPEPSILMKGLVACTGSQFCGQAIIETKLR
ALKVTEEVERLVSVPRPIRMHWTGCPNTCGQVQVADIGFMGCLTRGEEGKPVEGADVYVG
GRIGSDSHIGEIYKKGVRVTELVPLVAEILIKEFGAVPREREENED
>Q39473 | FATB_CINCA
MATTSLASAFCSMKAVMLARDGRGMKPRSSDLQLRAGNAQTSLKMINGTKFSYTESLKKL
PDWSMLFAVITTIFSAAEKQWTNLEWKPKPNPPQLLDDHFGPHGLVFRRTFAIRSYEVGP
DRSTSIVAVMNHLQEAALNHAKSVGILGDGFGTTLEMSKRDLIWVVKRTHVAVERYPAWG
DTVEVECWVGASGNNGRRHDFLVRDCKTGEILTRCTSLSVMMNTRTRRLSKIPEEVRGEI
GPAFIDNVAVKDEEIKKPQKLNDSTADYIQGGLTPRWNDLDINQHVNNIKYVDWILETVP
DSIFESHHISSFTIEYRRECTMDSVLQSLTTVSGGSSEAGLVCEHLLQLEGGSEVLRAKT
EWRPKLTDSFRGISVIPAESSV
>Q39617 | POR_CHLRE
MALTMSAKSVSARAQVSSKAQAAPAVAVSGRTSSRVMPAPALAARSSVARTPLVVCAATA
TAPSPSLADKFKPNAIARVPATQQKQTAIITGASSGLGLNAAKALAATGEWHVVMACRDF
LKAEQAAKKVGMPAGSYSILHLDLSSLESVRQFVQNFKASGRRLDALVCNAAVYLPTAKE
PRFTADGFELSVGTNHLGHFLLTNLLLDDLKNAPNKQPRCIIVGSITGNTNTLAGNVPPK
ANLGDLSGLAAGVPAANPMMDGQEFNGAKAYKDSKVACMMTVRQMHQRFHDATGITFASL
YPGCIAETGLFREHVPLFKTLFPPFQKYITKGYVSEEEAGRRLAAVISDPKLNKSGAYWS
WSSTTGSFDNQVSEEVADDSKASKLWDISAKLVGLSA
>Q39743 | RBS1_FLAPR
MASIPATVATVAQANMVAPFTGLKANAAFPVTKKVNDFSTLPSNGGRVQCMKVWPPLGKK
KYETLSYLPDLTEVQLAKEVDYLLRNKWVPCLEFELEHGFVYRENARSPGYYDGRYWTMW
KLPMFGCTDSAQVMKELQECKKEYPQAWIRIIGFDNVRQVQCISFIASKPGGF
>Q39748 | RBS6_FLAPR
MASIPATVAAVAQANMVAPFTGLKANAAFPVTKKVNDFSTLPSNGGRVQCMKVWPPLGKK
KYXTLSYLPNLTEAQLAKEVDYLLRNKWVPCLEFELEHGFVYRENARSPGYYDGRYWTMW

KLPMFGCTDSVQVMKELQECKKEYPQAWIRIIGFDNVRQVQCISFIASKPDXF
>Q3BAH4 | RR7_PHAAO
MSRRGTAEEKTAKSDPIYRNRLVNMLVNRILKNGKKSLAYQIIYRAVKKIQQKTETNPLS
VLRQAIRGVTPDIAVKARRVGGSTHQVPIEIGSTQGKALAIRWLLGASRKRPGRNMDFRL
SSELVDAAKGSGDAIRKKEETHRMAEANRAFAHFR
>Q3BAJ9 | RK16_PHAAO
MLSPKRTRFRKQHRGRMKGISCRGNHICFGRYALQALEPAWITARQIEAGRRAMTRYARR
GGKIWVRIFPDKPVTVRPTETRMGSGKGSPEYWVSVVKPGRIIYEMSGVSEAVAKAAMEI
AACKMPIRTQFVISG
>Q3BAK4 | RR11_PHAAO
MTKPIAKISLRKNARIGSNKNERRIPKGVIHVQASFNNTIVTVTDVRGRVVSWASAGTSG
FRGTRKGTPYAAQAAAFNAIRTLVDQGMQRAEVMIKGPGLGRDAALRAIRRNGILLSFVR
DVTPMPHNGCRPPKKRRV
>Q3BAL9 | RK33_PHAAO
MAKGKDIRIIVILECTCCVRKGVNKELLGISRYITQKNRHNTPNRLEFRKFCRYCQKYTI
HGEIKK
>Q3BAN8 | RR4_PHAAO
MSRYRGPRFKKIRRLGVLPGLTSKRPRSRSDLQTQLRFGKRSQYRIRLEEKQKLRFHYGL
TERQLLKYVHIAGKAKGSTGQVLLQLLEMRLDNILFRLGMASTIPGARQLVNHRHILVNG
RIVDIPSYRCKPLDIITTKDKERSKALIQNYLVSSPRGELPNHLTIDSLQYKGFVNQIID
SKWIGLKINELLVVEYYSRQT
>Q3BAP8 | RPOB_PHAAO
MLQNGNEGMSTIPGFSQIQFEGFCRFINQGLTEEFHKFQKIEDRDQEIEFQLFVETYQLV
EPLITERDAVYESLTYSSELYVPAVLIWKTGRNMQEQTVFIGNIPIMNSFGTSIVNGIYR
IVINQIVQSPGIYYRSELEHNGVSVYTSTIISDWGGRSELEIDRKARIWARVSRKQKISI
LVLSSAMGSNIREILDNVCYPEIFLSFPNDKEKKKIGSKENAILEFYQQFACVGGDPVFS
ESLCKELQKKFFQQRCELGKIGRQNLNRRLNLDIPQNNTFLLPRDLLAAVDHLIGMKLGM
GTLDDMSHLKNKRIRSVADLLQDQFGLALVRLENTVRGTICGAIRHKLIPTPQNLVTSTS
LTTTYESFFGLHPLSQVLDRTNPLTQIVHGRKWSYLGPGGLTGRTASFRIRDIHPSHYGR
ICPIDTSEGINVGLMGSLAIHVKVGYWGSIESPFYGLSERSKEAQMVYLSPNRDEYYMVA
AGNSLALNRDIQEQQVVPARYRQEFLTIAWEEIHLRSIFPFQYFSIGASLIPFIEHNDAN
RALMSSNMQRQAVPLSRSEKCIVGTGLEGQTALDSGVSVIAECKGKIIYTDTQKIFLSSN
GDTLSIPLVMYQRSNKNTCMNQKTQVQRGKYIKKGQILAGGAATAGGELALGKNVLVAYM
PWEGYNFEDAVLISERLVYKDIYTSFHIRKYEIQTHVTSQGPERITKEIPHLEARLLRNL
DRNGIVMLGSWIETGDILVGKLTPQTASESSYAPEDRLLRAILGIQVSTSKETSLRLPIG
GRGRVIDVRWIHRRGVSNSNPERIRVYISQKREIKVGDKVAGRHGNKGIISKILSRQDMP
YLQDGTPVDMVFNPLGVPSRMNVGQIFECSLGLAGDLLKKHYRIGPFDERYEQEASRKLV
FSELYEASKKTKNPWVFEPEYPGKSRIFDGRTGDLFEQPVLIGKSYILKLIHQVDDKIHG
RSSGHYALVTQQPLRGRAKQGGQRVGEMEVWALEGFGVAHILQEMLTYKSDHIRARQEVL
GAMIIGATVPNPESAPESFRLLVRELRSLSLELNHFLVSEKNFQMDRKEA
>Q3BAQ1 | RPOC1_PHAAO
MIDQYKHQQLRIGSVSPQQIRAWAKKILPNGEIVGEVTKPYTFHYKTNKPEKDGLFCERI
SGPIKSGICACGNYRGIGTEKEDPKFCEECGVEFVDSRIRRYQMGYIKLTCPVTHVWYLK
RLPSYIANLLDKPLRELEGLVYCDFSFARSIAKKPTFLRLRGSFEYEIQSWQYSIPLFFT

TQGFETFRNREISTGAGAIREQLADSDLRIITDNSLLEWKELGDEESAGNEWEEKKIRRR
KDFLVRRIELAKHFLRTNVDPEWMVLCLLPVLPPELRPIIQIDGGKLMSSDINELYRRVI
YRNNTLTDLLATSRSTPGELVMCQEKLVQEAVDTLFDNGIRGQPMRDGHNKVYKSFSDVI
EGKEGRFRETLLGKRVDYSGRSVIVVGPLLSLHQCGLPREIAIELFQAFVIRGLIRQDVA
SNTGIAKSKIREKEPIVWEILQEVMQGHPVLLNRAPTLHRLGIQAFQPILVEGRAICLHP
LVCKGFNADFDGDQMAVHVPLSLEAQAEARLLMFSHMNLLSPAIGDPVSVPTQDMLIGLY
VLTIGNPRGICANRYNQSNSNCRNYKKEKVYKNDFKYTKELYFSSSYDALGAYRQKRIHL
DSPLWLRWRLDQRVVGSREVPIEIQYESFGNYNEIYKHYQIIGSVKIEICCIYIRTTAGH
ISFYREIEEAIQGFWRAYS
>Q3C1G7 | RPOB_NICSY
MLGDGNEGISTIPGFNQIQFEGFCRFIDQGLTEELYKFPKIEDTDQEIEFQLFVETYQLV
EPLIKERDAVYESLTYSSELYVSAGLIWKNSRDMQEQTIFIGNIPLMNSLGTSIVNGIYR
IVINQILQSPGIYYRSELDHNGISVYTGTIISDWGGRSELEIDRKARIWARVSRKQKISI
LVLSSAMGLNLREILENVCYPEIFLSFLSDKERKKIGSKENAILEFYQQFACVGGDPVFS
ESLCKELQKKFFQQRCELGRIGRRNMNRRLNLDIPQNNTFLLPRDILAAADHLIGLKFGM
GALDDMNHLKNKRIRSVADLLQDQFGLALVRLENVVRGTICGAIRHKLIPTPQNLVTSTP
LTTTYESFFGLHPLSQVLDRTNPLTQIVHGRKLSYLGPGGLTGRTASFRIRDIHPSHYGR
ICPIDTSEGINVGLIGSLAIHARIGHWGSLESPFYEISERSTGVRMLYLSPGRDEYYMVA
AGNSLALNQDIQEEQVVPARYRQEFLTIAWEQVHLRSIFPFQYFSIGASLIPFIEHNDAN
RALMSSNMQRQAVPLSRSEKCIVGTGLERQAALDSGALAIAEREGRVVYTNTDKILLAGN
GDILSIPLVIYQRSNKNTCMHQKLQVPRGKCIKKGQILADGAATVGGELALGKNVLVAYM
PWEGYNSEDAVLISERLVYEDIYTSFHIRKYEIQTHVTSQGPEKVTNEIPHLEAHLLRNL
DKNGIVMLGSWVETGDILVGKLTPQVVKESSYAPEDRLLRAILGIQVSTSKETCLKLPIG
GRGRVIDVRWIQKRGGSSYNPETIRVYILQKREIKVGDKVAGRHGNKGIISKILPRQDMP
YLQDGRSVDMVFNPLGVPSRMNVGQIFECSLGLAGSLLDRHYRIAPFDERYEQEASRKLV
FSELYEASKQTANPWVFEPEYPGKSRIFDGRTGNPFEQPVIIGKPYILKLIHQVDDKIHG
RSSGHYALVTQQPLRGRAKQGGQRVGEMEVWALEGFGVAHILQEMLTYKSDHIRARQEVL
GTTIIGGTIPNPEDAPESFRLLVRELRSLALELNHFLVSEKNFQINRKEA
>Q3C1J4 | RBL_NICSY
MSPQTETKASVGFKAGVKEYKLTYYTPEYQTKDTDILAAFRVTPQPGVPPEEAGAAVAAE
SSTGTWTTVWTDGLTSLDRYKGRCYRIERVVGEKDQYIAYVAYPLDLFEEGSVTNMFTSI
VGNVFGFKALRALRLEDLRIPPAYVKTFQGPPHGIQVERDKLNKYGRPLLGCTIKPKLGL
SAKNYGRAVYECLRGGLDFTKDDENVNSQPFMRWRDRFLFCAEALYKAQAETGEIKGHYL
NATAGTCEEMIKRAVFARELGVPIVMHDYLTGGFTANTSLAHYCRDNGLLLHIHRAMHAV
IDRQKNHGIHFRVLAKALRMSGGDHIHSGTVVGKLEGERDITLGFVDLLRDDFVEQDRSR
GIYFTQDWVSLPGVLPVASGGIHVWHMPALTEIFGDDSVLQFGGGTLGHPWGNAPGAVAN
RVALEACVKARNEGRDLAQEGNEIIREACKWSPELAAACEVWKEIVFNFAAVDVLDK
>Q3C1K6 | RK20_NICSY
MTRIKRGYIARRRRTKIRLFASSFRGAHSRLTRTITQQKIRALVSAHRDRDRKKRDFRRL
WITRINAVIRERGVSYSYSRLIHDLYKRQLLLNRKILAQIAISNRNCLYMISNEIIKEVD
WKESTRII
>Q3C1K7 | RR18_NICSY
MDKSKRPFLKFKRSFRRRLPPIQSGDRIDYRNMSLISRFISEQGKILSRRVNRLTLKQQR
LITLAIKQARILSLLPFLNNEKQFERTESTARTTGFKARNK

>Q3C1L5 | RK22_NICSY
MLKKKKTEVYALGEHISMSADKARRVINQIRGRSYEETLMILELMPYRACYPILKLIYSA
AANASYNMGSSEANLVISKAEVNGGTTVKKLKPRARGRSFPIKRSTCHITIVMKDISLDD
EYVEMYSLKKTRWKKKSTAMPYRDMYNSGGLWDKK
>Q3C1L8 | RK16_NICSY
MLSPKRTRFRKQHRGRMKGISHRGNHISFGKYALQALEPAWITSRQIEAGRRAMTRNARR
GGKIWVRIFPDKPVTLRPAETRMGSGKGSPEYWVAVVKPGRILYEMGGVTENIARRAISL
AASKMPIRTQFIIS
>Q3C1L9 | RK14_NICSY
MIQPQTHLNVADNSGARELMCIRIIGASNRRYAHIGDVIVAVIKEAVPNMPLERSEVVRA
VIVRTCKELKRDNGMIIRYDDNAAVVIDQEGNPKGTRIFGAIARELRELNFTKIVSLAPE
VL
>Q3C1M0 | RR8_NICSY
MGRDTIAEIITSIRNADMDRKRVVRIASTNITENIVQILLREGFIENVRKHREKNKYFLV
LTLRHRRNRKRPYRNILNLKRISRPGLRIYSNYQRIPRILGGMGIVILSTSRGIMTDREA
RLEGIGGEILCYIW
>Q3C1M2 | RR11_NICSY
MAKAIPKISSRRNGRIGSRKGARRIPKGVIHVQASFNNTIVTVTDVRGRVVSWSSAGTSG
FKGTRRGTPFAAQTAAANAIRTVVDQGMQRAEVMIKGPGLGRDAALRAIRRSGILLTFVR
DVTPMPHNGCRPPKKRRV
>Q3C1N5 | RK23_NICSY
MDGIKYAVFTDKSIRLLGKNQYTSNVESGSTRTEIKHWVELFFGVKVIAMNSHRLPGKSR
RMGPIMGHTMHYRRMIITLQPGYSIPPLRKKRT
>Q3C1N7 | RR19_NICSY
MTRSLKKNPFVANHLLKKIDKLNTKAEKEIIVTWSRASTIIPTMIGHTIAIHNGKEHLPI
YITDSMVGHKLGEFAPTLNFRGHAKSDNRSRR
>Q3C1Q4 | NU4C_NICSY
MNYFPWLTIIVVFPIFAGSLIFFLPHKGNRVIRWYTICICILELLLTTYAFCYHFQSDDP
LIQLVEDYKWINFFDFHWRLGIDGLSIGPILLTGFITTLATLAAWPVTRDSRLFHFLMLA
MYSGQIGSFSSRDLLLFFIMWELELIPVYLLLCMWGGKKRLYSATKFILYTAGGSVFLLM
GVLGLALYGSNEPTLNFETSVNQSYPVVLEIIFYIGFFIAFAVKSPIIPLHTWLPDTHGE
AHYSTCMLLAGILLKMGAYGLIRINMELLPHAHSIFSPWLMIIGTIQIIYAALTSLGQRN
LKKRIAYSSVSHMGFIIIGISSLTDTGLNGALLQIISHGFIGAALFFLAGTTYDRIRLVY
LDEMGGIAIPMPKMFTMFSSFSMASLALPGMSGFVAELIVFFGIITGQKYLLIPKILITF
VMAIGMILTPIYSLSMSRQMFYGYKLFNAPKDSFFDSGPRELFLSISIFLPVIGIGIYPD
FVLSLAVDKVEVILSNFFYR
>Q3MKB3 | MATK_PERAC
MEEFQRYIELDRSWQHNFFYPLIFQEYIYGFAYDHGLNKSILLENAGDKKYSLLIVKRLI
TRMYQQNHLILSANHSNQNDFFGHKHKKNLYYQIISEGFAVIVEIPFSLLLISSLGAKEK
KIVKSHNLRSIHSIFPFFEDKFLHLNYVLEILIPYPIHLEILVQTLRYWVKDASSLHLLR
FFLYEYRNWNSLITTQKSISIFSKRNQRLFLFLYNFHVCEYESIFVFLCNQSSHLRSTSF
GALLERIYFYGKLEYLVKVFTFTKDFRVILWLFKDPFLHYVRYRGKSILASKGTSLLMHK
WKYYLINFWQCHFSLWSQPRRIYINRLSKHSLDFMDFFSSVRLNSSVVRSQMVENSFLID
NPIKKFDTIVRIIPLVGSLAKAKFCNVLGHPISKSVWTDLLDSDIIDRFGRICRNLSHYY

SGSSRKKSLYRIKYILRLSCARTLARKHKSTVRAFLKRLGSEFLEEFFTEEEKVLSLILP
RDSSISRGLYRGPFWYLDIFCIHDLANDE
>Q3V4X1 | RK2_ACOCL
MAIHLYKTSTSSTRNGAVDSQVKSNPRNNLIYGQHHCGKGRNARGIITAGHRGGGHKRLY
RKIDFRRNKKDISGRIVTIEYDPNRNAYICLIHYGDGEKRYILHPRGAIIGDTIVSGTEV
PISMGNALPLTDMPLGTAIHNIEITLGKGGQLARAAGAVAKLIAKEGKSATLRLPSGEVR
LISKNCSATVGQVGNVGANQQSLGRAGSKCWLGKRPVVRGVVMNPVDHPHGGGEGRAPIG
RKKPTTPWGYPALGRRSRKRNKYSDRFILRRRK
>Q3V4Z6 | RK16_ACOCL
MLSPKRTRFRKQHRGRMKGMSYRGSHICFGRYALQALEPSWITSRQIEAGRRAMTRYARR
GGKIWVRIFPDKPVTIRPAETRMGSGKGSPEYWVSVVKPGRILYEMGGVSETVARAAIEI
AASKMPIRTQFIIAG
>Q3V4Z8 | RR8_ACOCL
MGKDTIADIITSIRNAQMAKKGTVRIASTNLTENVVKILLREGFIENVRKHRESNKDFLV
LTLRHRRTRKGIYRTILKRISRPGLRIYSNYQGIPKILGGIGIVIVSTSRGIMTDREARL
QGIGGEMLCYIC
>Q3V502 | RPOA_ACOCL
MVREEVVGSTRALQWKCVESRADSKRLYYGRFILSPLLKGQADTIGIAMRRALLGEIEGT
CITRAKSEKVPHEYSTIAGIEESVHEILMNLKEIVLRSNLYGTRDASICVRGPRCVTAQD
IISPPSVEVVDTTQHIASLTEPIDLCIGLQIQRDRGYRMKTTKNSQDGSYPIDAVSMPVR
NANHSIHSYGNGNQKQEILFLEIWTNGSLTPKEALHEASRNLIDLFIPFLHAEEEDINFE
ENQNRFTVPPFTFPDRLANLKKNKKEIALKCIFIDQSELPPRTYNCLKRSNIHTLLDLLS
NSQEDLMKIEYFRIEDVKQILDTLQKHFAIDLPKNKF
>Q3V532 | RR4_ACOCL
MSRYRGPRLKKIRRLGALPGLTSKGTRPGSDLRNQFRSGKRSQYRIRLEEKQKLRFHYGL
TERQLLRYVHIAGKAKGSTGQVLLQLLEMRLDNILFRLGMAPTIPGARQLVNHRHILVNG
RIVDIPSYRCKPRDIITTKDKQRSKVLIQNNMDSSTREELPKHLTLDSFQHKGLVNQIID
SKWVGLKINELLVVEYYSRQT
>Q3V542 | RPOB_ACOCL
MPRDGNEGMFTIPGFSQIQFEGFCRFIDQGLMEEFHKFPKIEDTDQEIEFQLFVERYQLV
EPLIKERDAVYESLTYSSELYVPAGLIWKTGRDMQEQTIFIGNIPLMNSLGTFIVNGIYR
IVINQILQSPGIYYRSELDHNGISVYTSTIISDWGGRSELEIDRKSRIWARVSRKQKISI
LVLSSAMGSNLREILDNVCYPEIFLSFLNDREKKKIGSKENAILEFYQQFACVGGDPVFS
ESLCKELQKKFFQQRCELGRIGRRNMNRRLNLDIPQSNTFLLPRDVLAAADHLIGMKFGM
GTLDDMNHLKNKRIRSVADLLQDQFGLALVRLENAVRGTICGAIRHKLILTPQNLVSSTS
LTTTYESFFGLHPLSQVLDRTNPLTQIVHGRKLSYLGPGGLTGRTASFRIRDIHPSHYGR
ICPIDTSEGINVGLIGSLAIHARIGHWGSIESPFYEVYQRSKETKMVFLSPSRDEYYTVA
TGNSLALNRGGIQEEQIVPARYRQEFLTIAWEQIHLRSIFPFQYFSIGASLIPFIEHNDA
NRALMSSNMQRQAVPLSRSEKCIVGTGLERQAALDSGVSAIAECEGKIIYTDTHKIVLSG
HGDTISIPLVMYQRSNKNTCMHQNPQVRRGKCIKKGQILADGAATVGGELALGKNVLVAY
MPWEGYNFEDAVLISERLVYEDIYTSFHIRKYEIQTHVTSQGPERITHEIPHLEAHLLRN
LDRNGIVALGSWVETGDILVGKLTPQTANESSYAPEDRLLRAILGIQVSTAKETCLKLPI
GGRGRVIDVRWIQKKGGSSYNPETIRVYISQKREIKVGDKVAGRHGNKGIISKILSRQDM
PYLQDGTPVDMVFNPLGVPSRMNVGQIFECSLGLAGDLLDRHYRIAPFDERYEQEASRKL

VFSELYEASKQTANPWVFEPEYPGKSRIFDGRTGDPFEQPVLIGKSYILKLIHQVDDKIH
GRSSGHYALVTQQPLRGRAKQGGQRVGEMEVWALEGFGVAHILQEMLTYKSDHIRARQEV
LGTTIIGGTIPTPEDAPESFRLLVRELRSLALELNHFLVSEKNFQINRKEA
>Q3V543 | RPOC1_ACOCL
MIDRYKHQQLQIGSVSPQQISAWANKILPNGEIVGEVTKPYTFHYKTNKPEKDGLFCERI
FGPIKSGICACGNYRVIGAEKEDPKFCEQCGVEFIDSRIRRYQMGYIKLACPVTHVWYLK
RLPSYIANLLDKPLKELEGLVYCDFSFARPIAKKPTFLRLRGLFEYEIQSWKYSIPLFFT
TQGFDTFRNREISTGAGAIREQLADLDLRIVIDNSSVEWKDLGDEGSTGNEWEDRKIGRR
KDFLVRRMELAKHFIRTNVEPERMVLCLLPVLPPELRPIIQIDGGKLMSSDINELYRRVI
YRNNTLTDLLKTSRSTPGELVMCQEKLVQEAVDTLLDNGIRGQPMRDGHNKVYKSFSDVI
EGKEGRFRETLLGKRVDYSGRSVIVVGPSLSLHRCGLPREIAIELFQTFVIRGLIRQHLA
SNIGIAKSKIREKEPIVWEILQEVMQGHPVLLNRAPTLHRLGIQAFQPVLVEGRAICLHP
LVCKGFNADFDGDQMAVHVPLSLEAQAEARLLMFSHMNLLSPAIGDPISVPTQDMLIGLY
VLTMGNRRGIFVNRYNPCNRRNYQNKTVDNNNYKHTKEKKPYFLSSYDALGAYQQKRINL
HSPLWLRWRLDQRVIGSREVPIEVQYESLGTYQEIYGHYLIVRSVKKEILCIYIRTTVGH
ISFYREIEESVQGFCRAYSYGT
>Q3ZJ12 | CLPP_PSEAK
MPIGVPKVPYRLAGNEVAEWVDLYNRLYRERVLFLCQDLDDELANQLIGIMLYLNAEDKS
KGIYVYINSPGGSVTCGVGVFDAMNYIKSDVTTICVGTAASMASFVLAGGRKGKRLALPH
SRIMIHQPEGGSQGQASVVLSESEEVLRIRDEVAQIYSERTGQTLERISRDMNRDQFMSA
REAKDYGLVDQIASSVSQ
>Q3ZJ37 | RK20_PSEAK
MTRIKRGFVARKRRKKVLNLTKGFRGSSSVLFRPANQRKMKALRFSYRDRRQRKRDLRSL
WITRINSASRLYNLNYSQFVYKLKQLNIHINRKWLAQLAIRDYKVFDELIKTVF
>Q3ZJ38 | RR18_PSEAK
MRKYNPRKIKNKVNIPIVLHKANSTIPFVQGTVDYKNVALLRKYISAEGKILSRRLTRLT
SKQQRHISTAIKTARIAGLLPFINQ
>Q3ZJ47 | RR12_PSEAK
MPTIQQLIHSAREKITNKTKSPALKACPQRRGVCTRVYTTTPKKPNSALRKVARIRLTTG
FEVTAYIPGVGHTLQEHSVVLVRGGRVKDLPGVRYHIVRGTLDTAGVKGRLKSRSKYGVK
KPKKK
>Q3ZJ78 | RR11_PSEAK
MAKQIRKTNKKVKMTKLPKGVVHIQSTFNNTIVTITNLKGEVISWSSAGAVGFKGARKST
PFAAKTAAQTAARQSMDQGLKQAKVLVKGAGPGRETAIRGLIDSGLQITLIRDITAIPHN
GCRPPKKRRV
>Q3ZJ83 | RK14_PSEAK
MIRPQTILNVADNSGAKKLMCIRVLGGSYKQSANIGDIIIAVVKQATPNMPLKKSDKVRA
VVVRTAQGVQRENGTFIRFDDNAAVVINKDDNPRGTRVFGPVARELRDRKFTKIVSLAPE
VL
>Q3ZJ84 | RK16_PSEAK
MLSPKRTKFRRHHRGRMKGKANRGNSIVYGDFGLQALEPGWIKSRQIESGRRVLTRYVRR
NGKLWVCLFPDKPVTMRAAESRMGSGKGMPEYWVAVVKPGKILYELTGLTETVARKALRI
TGHKMPVKTQIVINKKF
>Q3ZJ86 | RR19_PSEAK

MSRSLKKGPFVANHLLKKVQKLNDLDEKQVIKTWSRSSVIIPIMIGHTISIHNGKEHIPL
YITDLMVGHKLGEFAPTRTFKGHIKKDKKSKR
>Q3ZJ88 | RK23_PSEAK
MIDLVKYPVSTEKSYRLIEKNQYTFDVDVRLTKPQIRKVFENLFDIKVLAVNTHLLPTKK
KRLGLNLGFKTRYKRAIITIKANQTIPIFENSDS
>Q40406 | CRTI_NARPS
MSIVGLVSVVCPSGGIKKRYFSKGLDNFQGFRSSECLGIQLQVPVPFYSGIRQSPRATSL
QVVCKDCPRPELEGAVNFLEAAQLSASFRSSPRPEKGLEVVVVGAGLAGLSTAKYLADAG
HKPILLESRDVLGGKIAAWKDKDGDWYETGLHIFFGAYPNVQNLFGELGINDRLQWKEHS
MIFAMPNKPGEFSRFDFPEVLPAPLNGIWAILRNNEMLTWPEKVRFAIGLLPAMVGGQAY
VEAQDGLTVTEWMRRQGVPDRVNDEVFIAMSKALNFINPDELSMQCILIALNRFLQEKHG
SKMAFLDGNPPERLCMPIVDHIQSLGGRAQLNSRLQKIELNPDGTVKHFVLGNGNIITGD
AYVVAAPVDILKLLLPQEWREIPYFQKLDKLVGVPVINVHIWFDRKLKNTYDHLLFTRSP
LLSVYADMSVTCKEYYDPNRSMLELVFAPAEEWISRSDSEIIERTMKELAKLFPDEIAAD
QSKAKILKYHVVKTPRSVYKTIPDCEPCRPLQRSPIEGFYLAGDYTNQKYLASMEGAVLS
GKLCAQSIVQDYELLVRRSKKASTAEMTVV
>Q40677 | ALFC_ORYSJ
MASATLLKSSFLPKKSEWGATRQAAAPKPVTVSMVVRAGAYDDELVKTAKTIASPGRGIL
AMDESNATCGKRLASIGLENTEANRQAYRTLLVTAPGLGQYISGAILFEETLYQSTVDGK
KIVDILTEQKIVPGIKVDKGLVPLAGSNNESWCQGLDGLASREAAYYQQGARFAKWRTVV
SIPNGPSELAVKEAAWGLARYAAISQDNGLVPIVEPEILLDGEHGIDRTFEVAQKVWAET
FFYMAENNVMFEGILLKPSMVTPGAECKDRATPEQVSDYTLKLLHRRIPPAVPGIMFLSG
GQSEVEATQNLNAMNQGPNPWHVSFSYARALQNTCLKTWGGQPENVKAAQDALLLRAKAN
SLAQLGKYTSDGEAAEAKEGMFVKNYVY
>Q41578 | PORA_WHEAT
MALQLLPSTLSVPKKGSSMGAAAVKDTAAFLGVSSKAKKASLAVRTQVATAPSSVTTSPG
SATAKPSGKKTLRQGVVVITGASSGLGLAAAKALAETGKWHVVMACRDFLKASKAAKAAG
MADGSYTVMHLDLASLDSVRQFVDAFRRAEMPLDVLVCNAAIYRPTARTPTFTADGHEMS
VGVNHLGHFLLARLLMEDLQKSDYPSRRMVIVGSITGNSNTLAGNVPPKASLGDLRGLAG
GLSGASGSAMIDGDESFDGAKAYKDSKVCNMLTMQEFHRRYHEETGITFSSLYPGCIATT
GLFREHIPLFRTLFPPFQKFVTKGFVSEAESGKRLAQVVAEPSLTKSGVYWSWNKDSASF
ENQLSQEASDPEKARKVWELSEKLVGLA
>Q42796 | F16P1_SOYBN
MAAATASTQLIFSKPCSPSRLCPFQLCVFDTKQVLSSGRRRHVGGSGVRCMAVGEAATTG
TKKRSGYELQTLTSWLLKQEQAGVIDAELTIVLSSISMACKQIASLVQRANISNLTGVQG
AVNVQGEDQKKLDVVSNEVFSNCLRSSGRTGIIASEEEDVPVAVEESYSGNYIVVFDPLD
GSSNIDAAASTGSNFWIYSPNDECLADIDDDPTLDTTEQRCIVNVCQPGSNLLAAGYCMY
SSSIIFVLTLGNGVFVFTLDPMYGEFVLTQENLQIPRAGKIYAFNEGNYQLWDEKLKKYI
DDLKDPGQSGKPYSARYIGSLVGDFHRTLLYGGIYGYPRDKKSKNGKLRLLYECAPINFI
VEQAGGKGTDGLQVLRLQGTEIHQRVPLYIGEEVEKVEKYLA
>Q42822 | RBS_GLYTO
MASSMISSPAVTTVNRAGAGTVAPFTGLKSMAGFPTRKTNNDIASIASNGGRVQCMQVWP
TTGKKKFETLSYLPDLDDAQLAKEVEYLLRKGWIPCLEFELEHGFVYRENHRSPGYYDGR
YWTMWKLPVFGCTDASQVLKELQEAKTAYPNGFIRIIGFDNVRQVQCISFIAYKPPSF

>Q42843 | HEM11_HORVU
MAGATSATAAAGAFAAAKARGPAAACPWLVAAGGRRRSGVVRCDAGGDAQAASKAASITA
LEQFKISADRYMKEKSSIAVIGLSVHTAPVEMREKLAVAEELWPRAISELTSLNHIEEAA
VLSTCNRMEIYVVALSWNRGIREVVDWMSKKSGIPASELREHLFMLRDSDATRHLFEVSA
GLDSLVLGEGQILAQVKQVVRNGQNSGGLGKNIDRMFKDAITAGKRVRCETNISAGAVSV
SSAAVELAMMKLPKSECLSARMLLIGAGKMGKLVVKHLIAKGCKKVVVVNRSVERVDAIR
EEMKDIEIVYRPLTEMYEAAADADVVFTSTASESLLFTKEHAEVLPPISLAMGGVRLFVD
ISVPRNVGACLSEVEHARVYNVDDLKEVVEANKEDRVRKAMEAQTIITQELKRFEAWRDS
LETVPTIKKLRSYADRIRASELEKCLQKIGEDNLNKKMRRSIEELSTGIVNKLLHGPLQH
LRCDGSDSRTLDETLENMHALNRMFSLDTEKAVLEQKIKAKVEKTQS
>Q42850 | PORB_HORVU
MALQAATSFLPSALSARKEGAAKDSAFFGVRLADGLKLDATSLGLRTKRVNTSSVAIRAQ
AAAVSAPTATPASPAGKKTVRTGNAIITGASSGLGLATAKALAESGKWHVIMACRDYLKT
ARAARAAGMPKGSYTIVHLDLASLDSVRQFVKNVRQLDMPIDVVVCNAAVYQPTAKEPSF
TADGFEMSVGVNHLGHFLLARELLEDLKASDYPSKRLIIVGSITGNTNTLAGNVPPKANL
GDLRGLAAGLNGVGSAAMIDGAEFDGAKAYKDSKVCNMLTMQEFHRRYHEETGVTFASLY
PGCIATTGLFREHIPLFRLLFPPFQKYITKGYVSEEEAGKRLAQVVSEPSLTKSGVYWSW
NKNSASFENQLSEEASDTEKARKVWELSEKLVGLA
>Q42876 | AMPL2_SOLLC
MNGVLCSSSSSFHSYPSIFTKFQSSPIWSFSISVTPLCSRRAKRMAHSIARDTLGLTHTN
QSDAPKISFAAKEIDLVEWKGDILTVGATEKDLARDGNSKFQNPLLQKLDSKLSGLLSEA
SSEEDFSGKAGQSTILRLPGLGSKRIALVGLGSPTSSTAAYRCLGEAAAAAAKSAQASNI
AIALASTDGLSAELKLSSASAITTGAVLGTFEDNRFKSESKKPTLKSLDILGLGTGPEIE
KKIKYAADVCAGVILGRELVNAPANVLTPAVLAEEAKKIASTYSDVFSANILDVEQCKEL
KMGSYLRVAAASANPAHFIHLCYKPSSGEIKKKIALVGKGLTFDSGGYNIKTGPGCSIEL
MKFDMGGAAAVLGAAKALGQIKPAGVEVHFIVAACENMISGTGMRPGDIITASNGKTIEV
NNTDAEGRLTLSVGISCNQGVEKIVDLATLTGACVVALGPSIAGIFTPSDDLAKEVVAAS
EVSGEKLWRLPMEDSYWDSMKSGVADMVNTGGRPGGAITAALFLKQFVNEKVQWMHIDLA
GPVWSDKKKNATGFGVSTLVEWVLKNSTN
>Q42967 | DCUP_TOBAC
MMSCNYSFSSISPSSKSAFTSPSNFNLNPRLICCSAGGTVAEPKAINATQPLLLDAVRGK
EVERPPVWLMRQAGRYMKSYQLLCEKYPLFRDRSENVDLVVEISLQPWKVFRPDGVILFS
DILTPLSGMNIPFDIIKGKGPVIFDPLRTAADVEKVREFIPEKSVPYVGEALTILRKEVN
NQAAVLGFVGAPFTLASYVVEGGSSKNFTKIKRLAFAEPKVLHALLQKFATSMAKYIRYQ
ADSGAQAVQIFDSWATELSPVDFEEFSLPYLKQIVDSVKLTHPNLPLILYASGSGGLLER
LPLTGVDVVSLDWTVDMADGRRRLGPNVAIQGNVDPGVLFGSKEFITNRINDTVKKAGKG
KHILNLGHGIKVGTPEENFAHFFEIAKGLRY
>Q43036 | RBL_PLUAU
MSPQTETKAGVGFKAGVKEYKLTYYTPDYEPHDHDILAAFRVTPQPGGPPEEAGAAVAAE
SSTGTWTPVWTDGLTWFDRYKGRSYHIDAVPGGENPFIAYVAYPLDLFEEGSVANMFSSI
VGNVFGFKALRALRLEDLRIPPAYTKTFQGPPHGIQVERDKLNKYGRPLLGCTIKPKLGL
SAKNYGRAVYECLRGGLDFTKDDENVNSQPFMRWRDRFLFCAEALFKAQSETVEIKGHYS
NATAGTCEEMIKRAVFADELGVPIVMHDYLTGGFTANTSLAHYCRDNGLLLHIHRAMHAV
IDRQKNHGIHFRVLAKALRLSGGDHIHSGTVVGKLEGERDITLGFVDLLRDDFVEKDRSR

GIYFTQPWVSLPGVIPVASGGIHVWHMPALTEIFGDDSVLQFGGGTLGTPWGNAPGAVAN
RVALEACVQARNEGRDLAREGNEIIRQAAKWSPELAAACEVWKEIEFEYEAVDTL
>Q43058 | HEM2_PHYPA
MVGVMMAAAATPGCRVSQALTACSSHEGLRRVAPVFGPGVVSVSAPCKLPRKLNVQAVAE
PIAKSSPRTIEECEANVVAGNAPAAPPVPAKPSAPEGTPAISPLVMPARPRRNRRSPALR
AAFQETTISPANFILPLFVHEGEQNAPIGAMPGCQRLGWRHGLIDEVYKARDVGVNSVVL
FPKVPDALKSSTGDEAYNPDGLVPRCIRLLKAIPDLVIYTDVALDPYSSDGHDGIVREDG
LIMNDETVHQLCKQAVAQAQAGADVVSPSDMMDGRVGAIRKALDLAGHQDVSIIAYTAKY
ASAFYGPSREALDSNPRFGDKKTYQMNPANYREALIETRMDEAEGADILMVKPAMPYLDV
IRLLRDNTALPISAYQVSGEYSMIRAGCRGMLDEKKAVLESLLSIRRAGADVILTYFAIQ
AAQWLCAERV
>Q43086 | PYRB1_PEA
MTVASMLSSNSMNVGVSNPKMSSKTSACCLLNRPWPSSCSMSISSCGQFGVSEKSKLLCG
AGALQVESAPLFSVGQKFQLDDVIEAQQFDRETLSAIFEVARSMENIRGNSSGSQMLKGY
LMATLFYEPSTRTRLSFESAMKRLGGDVLTTENAREFSSAAKGETLEDTIRTVEGYSDII
VLRHFESGAARRAAATANIPVINAGDGPGQHPSQALLDVYTIEREIGKLDGIKVGLVGDL
ANGRTVRSLAYLLAKYRDVKLYFVSPNVVKMKDDIKEYLTSKGVEWEESSDLMEVASKCD
VVYQTRIQKERFGEKLNLYEEARGKYIVNQDVLKVMQNHAVVMHPLPKLDEIEADVDNDP
RAAYFRQAKNGLYIRMALLKVLLLGW
>Q43087 | PYRB2_PEA
MTASSSLFSCSMHMEVLTPKISKWPKNFVSCHSKISYVETNYLKSTCYPISRFFCINNLK
KTRQRDGIHCFSEGQKFQLDDVVEAQQFDRDILNAIFEVARDMEKIERNSPESQILKGYL
MATLFYEPSTRTRLSFESAMRRLGGEVLTTENAREFSSAAKGETLEDTIRTVEGYSDLIV
LRHFESGAARRAATIAGIPIVNAGDGPGQHPSQALLDVYTIEREIGKLDGIKVGLVGDLA
NGRTVRSLTYLLAKYKDVKIYFVSPEVVKMKDDIKDYLTSKGVDWEESSDLVEVASECDV
VYQTRIQKERFGERLDLYEKARGKFIVNQNILNAMQRHAVIMHPLPRLDEITVDVDADPR
AAYFRQAKYGLYIRMALLKLLLVGW
>Q43092 | SSG1_PEA
MATITGSSMPTRTACFNYQGRSAESKLNLPQIHFNNNQAFPVLGLRSLNKLHVRTARATS
GSSDTSEKSLGKIVCGMSLVFVGAEVGPWSKTGGLGDVLGGLPPVLAGNGHRVMTVSPRY
DQYKDAWDTNVLVEVKVGDKIETVRFFHCYKRGVDRVFVDHPLFLERVWGKTGSKLYGPK
TGIDYRDNQLRFSLLCQAALEAPRVLNLNSSKYFSGPYGEDVIFVANDWHSALIPCYLKS
MYKSRGLYKNAKVAFCIHNIAYQGRNAFSDFSLLNLPDEFRSSFDFIDGYNKPCEGKKIN
WMKAGILESDQVFTVSPHYAKELISGEDRGVELDNIIRSTGIIGIVNGMDNREWSPQTDR
YIDVHYNETTVTEAKPLLKGTLQAEIGLPVDSSIPLIGFIGRLEEQKGSDILVEAIAKFA
DENVQIVVLGTGKKIMEKQIEVLEEKYPGKAIGITKFNSPLAHKIIAGADFIVIPSRFEP
CGLVQLHAMPYGTVPIVSSTGGLVDTVKEGYTGFHAGPFDVECEDVDPDDVDKLAATVKR
ALKTYGTQAMKQIILNCMAQNFSWKKPAKLWEKALLNLEVTGNVAGIDGDEIAPLAKENV
ATP
>Q43467 | EFTU1_SOYBN
MAVSSATASSKLILLPHASSSSSLNSTPFRSSTTNTHKLTPLSSSFLHPTTVLRRTPSST
TTPRRTFTVRAARGKFERKKPHVNIGTIGHVDHGKTTLTAALTMALAALGNSAPKKYDEI
DAAPEERARGITINTATVEYETENRHYAHVDCPGHADYVKNMITGAAQMDGAILVVSGAD
GPMPQTKEHIILAKQVGVPNMVVFLNKQDQVDDEELLQLVEIEVRDLLSSYEFPGDDTPI

VSGSALLALEALMANPAIKRGDNEWVDKIFQLMDEVDNYIPIPQRQTDLPFLLAVEDVFS
ITGRGTVATGRVERGTIKVGETVDLVGLRETRNTTVTGVEMFQKILDEALAGDNVGLLLR
GVQKTDIQRGMVLAKPGTITPHTKFSAIVYVLKKEEGGRHSPFFAGYRPQFYMRTTDVTG
KVTSIMNDKDEESTMVLPGDRVKMVVELIVPVACEQGMRFAIREGGKTVGAGVIQSIIE
>Q43503 | LCYB_SOLLC
MDTLLKTPNNLEFLNPHHGFAVKASTFRSEKHHNFGSRKFCETLGRSVCVKGSSSALLEL
VPETKKENLDFELPMYDPSKGVVVDLAVVGGGPAGLAVAQQVSEAGLSVCSIDPNPKLIW
PNNYGVWVDEFEAMDLLDCLDATWSGAAVYIDDNTAKDLHRPYGRVNRKQLKSKMMQKCI
MNGVKFHQAKVIKVIHEESKSMLICNDGITIQATVVLDATGFSRSLVQYDKPYNPGYQVA
YGILAEVEEHPFDVNKMVFMDWRDSHLKNNTDLKERNSRIPTFLYAMPFSSNRIFLEETS
LVARPGLRIDDIQERMVARLNHLGIKVKSIEEDEHCLIPMGGPLPVLPQRVVGIGGTAGM
VHPSTGYMVARTLAAAPVVANAIIQYLGSERSHSGNELSTAVWKDLWPIERRRQREFFCF
GMDILLKLDLPATRRFFDAFFDLEPRYWHGFLSSRLFLPELIVFGLSLFSHASNTSRFEI
MTKGTVPLVNMINNLLQDKE
>Q43727 | G6PD1_ARATH
MATHSMIIPSPSSSSSSLATAASPFKETLPLFSRSLTFPRKSLFSQVRLRFFAEKHSQLD
TSNGCATNFASLQDSGDQLTEEHVTKGESTLSITVVGASGDLAKKKIFPALFALFYEGCL
PQDFSVFGYARTKLTHEELRDMISSTLTCRIDQREKCGDKMEQFLKRCFYHSGQYNSEED
FAELNKKLKEKEAGKISNRLYYLSIPPNIFVDVVRCASLRASSENGWTRVIVEKPFGRDS
ESSGELTRCLKQYLTEEQIFRIDHYLGKELVENLSVLRFSNLVFEPLWSRNYIRNVQLIF
SEDFGTEGRGGYFDQYGIIRDIMQNHLLQILALFAMETPVSLDAEDIRSEKVKVLRSMKP
LRLEDVVVGQYKGHNKGGKTYPGYTDDPTVPNHSLTPTFAAAAMFINNARWDGVPFLMKA
GKALHTRGAEIRVQFRHVPGNLYKKSFATNLDNATNELVIRVQPDEGIYLRINNKVPGLG
MRLDRSDLNLLYRSRYPREIPDAYERLLLDAIEGERRLFIRSDELDAAWDLFTPALKELE
EKKIIPELYPYGSRGPVGAHYLASKYNVRWGDLGEA
>Q43784 | SSG1_MANES
MATVIAAHFVSRSSHLSIHALETKANNLSHTGPWTQTITPNGLRSLNTMDKLQMKTQSKA
VKKVSATGNGRPAAKIICGHGMNLIFVGAEVGPWSKTGGLGDVLGGLPPAMAARGHRVMT
VSPRYDQYKDAWDTSVSVEIKIGDRIETVRFFHSYKRGVDRVFVDHPMFLEKVWGKTGSK
IYGPRAGLDYQDNQLRFSLLCLAALEAPRVLNLNSSKNFSGPYGEEVAFIANDWHTALLP
CYLKAIYQPMGIYKHAKVAFCIHNIAYQGRFAFSDFPRLNLPDKFKSSFDFIDGYEKPVK
GRKINWMKAGILESDRVLTVSPYYAQEVISGVERGVELDNFIRKTGIAGIINGMDVQEWN
PVTDKYIDIHYDATTVMDAKPLLKEALQAEVGLPVDRNVPLIGFIGRLEEQKGSDIFVAA
ISQLVEHNVQIVILGTGKKKFEKQIEHLEVLYPDKARGVAKFNVPLAHMITAGADFMLVP
SRFEPCGLIQLHAMRYGTVPIVASTGGLVDTVKEGYTGFQMGALHVECDKIDSADVAAIV
KTVARALGTYATAALREMILNCMAQDLSWKGPARMWEKMLLDLEVTGSEPGTEGEEIAPL
AKENVPTP
>Q49KT5 | RK23_EUCGG
MDGIKYAVFTDKSIRLLGKNQYTFNVESGSTRTEIKHWVELFFGVKVKAMNSHRLPGKGR
RMGPILGHTMHYRRMIITLQPGYSIPPLRKKRT
>Q49KU1 | RR15_EUCGG
MVKNSFISVISQEEEKEENRGSIEFQVFRFTNKIRRLTSHLELHRKDYLSQRGLRKILGK
RQRLLSYLSKKNRIRYKELISQLDIRESKTR
>Q49KV0 | RK32_EUCGG

MAVPKKSTSISKKRIRKNIWKKKVYRAALKAFSLAKSLSTGNSKSFFCTTIK
>Q49KV9 | RK22_EUCGG
MRKKRNKNLYGEVYALGQHISMSAHKARRVIDQIRGRSYEETLMILELMPYRACYPIFKL
VYSAAANASHNMGFNEASLVISKAEVNEGTTMKKLKPRARGRSYAIKRPTCHIRIVLKDK
SFYEEENFFCLKQSEWKKKKKYTDMTYHYMDKGGGLWDKK
>Q49KW0 | RR3_EUCGG
MGQKINPLGFRLGTTQGHHSIWFAQAKNYSDGLQEDQKIRNCIKNYLQKNMRISSGVEGI
ARIEIRKRIDLIQVIIYMGFPKLLLEGKTRRIEELQMNVQKELNCVNRKLNIAITRITNP
YGHPNILAEFIAGQLKNRVSFRKAIKKAIELTEQADTKGIQVQIAGRLDGKEIARVEWMR
EGRVPLQTIRAKIDYCSYGVRTVYGVLGIKIWIFWE
>Q49KW2 | RK14_EUCGG
MIQPQSHLNVADNSGARELMCIRIIGASNRRYAHIGDIIVAVIKEALPNTPLERSEVIRA
VIVRTCKELKRDNGMIIRYDDNAAVVIDQEGNPKGTRVFGAIARELRQLNFTKIVSLAPE
VL
>Q49KW5 | RR11_EUCGG
MAKSIPRTGSRRNVRSGSRKSTRRIPKGVIHVQASFNNTIVTVTDVRGRVISWSSAGTCG
FNGTRRGTPFAAQTAAGNAIRTVVDQGMQRAEVMIKGPGLGRDAALRAIRRSGILLSFVR
DVTPMPHNGCRPPKKRRV
>Q49KX3 | CLPP_EUCGG
MPIGVPKVPFRSPGEEDASWVDVYNRLYRERLLFLGQEVDSEISNQLIGLMVYLSIENDN
KDLYLFINSPGGWVIPGVAIYDTMQFVQPDVHTICMGLAASMGSFVLVGGEITKRLAFPH
ARVMIHQPASSFYEAQTGEFILEAEELLKLRETITRVYVQRTGKPLWVVSEDMERDVFMS
ATEAQAHGIVDLVAIE
>Q49KX4 | RR12_EUCGG
MPTIKQLIRNTRQPIRNVTKSPALGGCPQRRGTCTRVYTITPKKPNSALRKVARVRLTSG
FEITAYIPGIGHNSQEHSVVLVRGGRVKDLPGVRYHIVRGTLDAVGVKDRQQGRSQYGVK
KPK
>Q49KX7 | RR18_EUCGG
MEKSKRLFLKSKRSFRRRLPPIQSGDRIDYRNMSLISRFISEQGKILSRRVNRLTLKQQR
LITIAIKQARILSLLPFLNNEKQFERSESTAGATGLRTINK
>Q49KZ0 | RBL_EUCGG
MSPQTETKASVGFKAGVKDYKLTYYTPDYETKDTDILAAFRVTPQPGVPPEEAGAAVAAE
SSTGTWTTVWTDGLTSLDRYKGRCYHIEPVAGDENQYICYVAYPLDLFEEGSVTNMFTSI
VGNVFGFKALRALRLEDLRIPPSYTKTFQGPPHGIQVERDKLNKYGRPLLGCTIKPKLGL
SAKNYGRAVYECLRGGLDFTKDDENVNSQPFMRWRDRFLFCAEAIFKSQAETGEIKGHYL
NATAGTCEEMMKRAVFARELGVPIVMHDYLTGGFTANTSLAHYCRDNGLLLHIHRAMHAV
IDRQKNHGIHFRVLAKALRMSGGDHIHAGTVVGKLEGERDITLGFVDLLRDDFIEKDRSR
GIYFTQDWVSLPGVLPVASGGIHVWHMPALTEIFGDDSVLQFGGGTLGHPWGNAPGAVAN
RVALEACVQARNEGRDLAREGNEIIREASKWSPELAAACEVWKEIKFEFEAMDTL
>Q49L07 | RPOC1_EUCGG
MIDRYKHQQLRIGSVSPQQISAWAKKILPNGEIVGEVTKPYTFHYKTNKPEKDGLFCERI
FGPIKSGICACGNYRVIGDEKEDPKFCEQCGVEFVDSRIRRYQMGYIKLACPVTHVWYLK
RLPSYIANILDKPLKELEGLVYCDFSFARPIAKKPTFLRLRGLFEYEIQSWKYSIPLFFT
TQGFDTFRNREISTGAGAIREQLADLDLRIIIDYSLVEWKELGEERPTGNEWEDRKIGRR

KDFLVRRMELAKHFLRTNIEPEWMVLCLLPVLPPELRPIIQIDGGKLMSSDINELYRRVI
YRNNTLTDLLTTSRSTPGELVMCQEKLVQEAVDTLLDNGIRGQPMRDGHNKVYKSFSDVI
EGKEGRFRETLLGKRVDYSGRSVIVVGPSLPLHRCGLPREIAIELFQTFVIRGLIRQHLA
SNIGVAKSKIREKEPIVWEILQEVMQGHPVLLNRAPTLHRLGIQAFQPILVEGRAICLHP
LVRKGFNADFDGDQMAVHVPLSLEAQAEARLLMFSHMNLLSPAIGDPISVPTQDMLIGLY
VLTSGNRRGICTNRYNPWNRRNYQNERTYDNNYKYRKKKEPFFCNSYDAIGAYRQKRINL
DSPLWLRWRLDQRIIASREVPIEIHYESLGTYHEIYGHYLIVRSVKKEIICIYIRTTVGH
ISLFREIEEAIQGFCRACSFGT
>Q49U13 | MATK_EQUAR
MKITDKKLLLFENISKKVTYQQRFLYPLLFHKEFYVVTSDSFIDKSNMNLSKEFVLNDRY
SFLVIRRLINRIRNLNNSKKFIQTSLSDKSICSKLDFYLLKALNMVLETFLLIQSEQTKK
RVNWKSYKSIFSTCSFVEEKFIFSNQILDLKIPHFIHPESFIRILRQQIKDASFLHLTRF
FVHEYKERSKNEKLIYSFNKRNKFVTFSWNFFYIFELEFFLTSLLTRFINNLVLSNLFDQ
INLLEKINNNNKSQFFLSEKRIYNQNSCIHYVRYQNHCIMASEGFYFHDTNWIYYILNIW
QFFMHLWIQPFRFSTKHFQKQSFFFLGYQFGRESKLLKVRSISLDKSPTIYSRLKKNLLK
TQIVYPIDFLAKEGFCDISGYPISRSTWTTSTDEEILLNFNKIWKSFYFYYGGLIKKDIL
YRIKYILRFSCAKTLARKHKSTTRVVWKKIVSDLSLSSSLRRKKNFHFVFFQGSHG
>Q4FG71 | RR3_RANMC
MGQKINPLGFRLGTTQSHHSVWFAQPKNYSGGLQEDKKIKDCIKNYVQKNRKISSGVEGI
ARIKIKKRIDLIQVIIYMGFPKFLEGNPQGIEELQSNIQKEFNSVNQKLNIAITRIAKPY
GQPNILAEFIAGQLKNRVSFRKAMKKAIELTEQADTKGIQIQIAGRIDGKEIARVEWIRE
GRVPLQTIRAKIDYCSYTVRTIYGALGIKIWIFAGEE
>Q4FG72 | RR3_NUPAD
MGQKINPLGFRLGTTQSHRSFWFAQPKNYSKGLQEDEKIRDCIKNYVQKHMRISSGFEGI
ARIDIKKRIDLIQVIIHIGFANMLIEGRARGIEELQTNVQKSFHSVNRRLNIAIARVPRP
YGQPNILAEYIALQLKNRVSFRKAMKKAIELAEQADAKGIQVQIAGRLNGNEIARVEWIR
EGRVPLQTIRVKIDHCSYPVRTIYGVLGIKIWIFLDEE
>Q4FG73 | RR3_GINBI
MGQRINPLGFRLGVTQNHRSHWFAQKKYYSEDLQEDEEIRNCIENYVRRHMKNYSNYGGI
ARVEIRRKIDLIQVEIHIGFPNLLIEDRGRGIEQLRTDVRNMLNSANRKLNISIAKVAKP
YGVPNILAEYIALQLEDRVSFRKTVKKAIELAEQADIRGIQIQIAGRLDGNEIARVEWAR
GGRVPLQTIRARIDHCYYPAKTIYGVLGIKIWIFGDEE
>Q4H186 | MATK_PILTE
MEELQGYFEKDRSRQQPFLYPLLFQEYIYALAHDRGLNRNGSIFYEPLEVFGYDSKSSLA
LVKRLITRIYQQHFFLSSVNDSNQNQFVGHHHTNFFYSRFYSQMISEGFAIIVEIPFSLQ
LVSYLKEKEIPKSHNLRSIHSIFPFLEDKLLHFNYVSDILIPHPIHMEILVQILQCWIQD
VPLLHFLRFFLHEYHNWNSFFITQNKSIYLFSKETKRLFRFLYNSYVYECEFVFVFLRKY
SSYLRFTSFRTFLERRYFYGKMEHLQTEHLIIVCCDYFNGTLWSFKDPFMHYARCQGKAI
LVSKGTHLLMKKWKYNFVNLWQYYFHFWYQSYRIHINQLSKHSFHFLGYLSSLLKNSSTV
RNQMLDNSFLIDTLTTKFDTAVPVIFLIVSLSKAQFCTVSGHPISKPIWTDLSDSGIIER
FGRICRNLSHYHSGSSKKQGLYRIKYILRLSCARTLARKHKSTVRTFLQRLGSRLLEEFF
TEGEQDLSLILPKAIPFPFQGSHRERIWYLDIIRINDLVNRL
>Q4H195 | MATK_LAPNE
MEELQGYFEKDRSRQQPFLYPLLFQEYIYALAHDRGLNRNGSIFYEPLEVFGYDSKSSLA

LVKRLITRIYQQHFFLSSVNDSNQNRFVGHHHTNFFYSRFYSQMISEGFAIIVEIPFSLQ
LVSPLKEKEIPKSHNLRSIHFNFPFLEDQLLHFNYVSDILIPHPIHMEILVQILQCWIQD
VPLLHFLRFFLHEYHNWHSFFITQNKSIYLFSKETKRLFRFLYNSYVSECEFVFVFLRKH
SSYLRFTSFRTFLERRYFYGKMEHLQTEHLIIVCCDYFNRTLWSFKDPFMHYARCQGKAI
LVSKGTHLLMKKWKYNFVNLWQYYFHFWYQSYRIHINQLSNHSFYFLGYLSSLLKNSSTV
RNQMLDNSFLIDTLTTKFDTAVPVIFLIVSLSKAQFCTVSGHPISKPIWTDLSDSGIIER
FGRICRNLSHYHSGSSKKQGLYRIKYILRLSCARTLARKHKSTVRTFMQRLGSRLLEEFF
TEGEQDLSLILPKAIPFPFSGSHRERIWYLDIIRINDLVNRS
>Q4H1A1 | MATK_BELCH
MEELQGYLEKDRSRQQPLLYPLLFQEYIYALAHDRGLKGSLFYEPTEVFGYDSKFSLALV
KRLIIRIYQQNFFLSVVNDSNKNRFVSHHHNNFCYSHFYSQMISEGFAILVEIPFSLRLV
SYFEKKEIPKSHNLRSIHSFFPFLEDKLLHSDYVSDIIIPHPIHMEILVQILQCWIQDVP
LLHFLRFFLHKYHNWNSFLITPKKSIYVFSKENKRLFRFLYNSYVSECEFLLVFLRKQSS
YLRLTSFGTFLERRHFYVKMEHLQMQHLILIVVCRDYFQGTLWSFKDPFMHYVRCQGKAV
LASKGTHFLMKKWKYNFVNLWQYYFHFWYQSYRIHINQLSNYSFYFMGYLSSLLKNSSTV
RNQMLENSFLIDTVTNKFETIVPVIFLIGSLSKAQFCTVSGHPISKPIWADLSDSEIIER
FGRMCRNLSHYHSGSSKKQGLYRIKYILRLSCARTLAGKHKSTVRTFLRRLGSGLLEEFF
TEEEQVLSLILPKTIPFTFYGSKKERIWYLDIIRINDXVNHE
>Q4LAM6 | MATK_MALDO
MEEFQGYLELDRYQQHDFLYPLIFREYIYALAHDHGLNRSILLDNVGYDTKYSLLIIKRL
ISRMYQQNHLIISANDSNQNKFFGYNKNLYSQMMSEGFAVIVEIPFSRRLVSSLEATEIV
KSYNLRSIHSIFPFLEDKFPHLNYVSDVLIPYPIHLEILVQTLRYWVKDPSSLHLLRLFL
HEYSNWNSLITPKKIIFSKSNPRLFLLLYNSHVCEYESILLFLRNQSSHLRLTSSGIFFE
RIHFYEKKKDPVEEVFVNDFPAAILWFFKDPFMHYVRYQGKSILSSKDTPLLMNKWKYYL
VNLWQCHSYVWSQPGRIYINQLSKHSLDFLGYFSSMRPNLSVVRGQMLENSFIMDNAMKK
LDTLVPIIPLIGSLAKVKFCNALGHPISKSTWADSSDFDIIDRFLHICRNLSHYYSGSSR
KKSLYRIKYILRLSCVKTLARKHKSTVRTFLKRLGYKIIGRILYGRRTDSFFNLPKSFLY
FEEVL
>Q4VZH3 | RR4_CUCSA
MSRYRRPRFKKIRRLGALPGLTSKRPKTGNDLKNQSRSGKKSQYRIRLEEKQKLRFHYGL
TERQLLKYVRIAGKAKGSTGQVLLQLLEMRLDNILFRLGMASTIPQARQLVNHRHILVNG
SIVDIPSYRCKPRDIITAKDEKKSRTLIQNYLDSSPPQELPKHLTLQPLQYKGLVNQIID
SKWVSLKINELLVVEYYSRQT
>Q4VZK2 | RR11_CUCSA
MAKPIPRIGSRRNGRISSRKSTRRIPKGVIHVQASFNNTIVTVTDVRGRVISWSSAGTCG
FKGTRRGTPFAAQTAAGNAIRGVVDQGMQRAEVMIKGPGLGRDAALRAIRRSGILLSFIR
DVTPMPHNGCRPPKKRRV
>Q4VZK3 | RPOA_CUCSA
MVREKIRVSTRTLKWKCVESRADSKRLYYGRFILSPLMKGQGDTIGIAMRKALLGEIEGT
CITRAKSEKIPHEYSTIVGIQESVHEILMNLKEIVLRSNLYGTRDASICVKGPGCVTAQD
IILPPSVEIVDNTQHIANLMEPINLCIELKIERNRGYHIQTPNNFQDASYPMDAIFMPVR
NVNHSIHSYVNGNEKQEILFLEIWTNGSLTPKEALHEASRNLIDLFIPFLHAEEEKENFH
LKNNKKKVTLPLFTFHEKLAKLRKKKKEIALKYIFIDQSELPPRIYNCLKRCNIHTLFDL
LNNSPDELMKIKHFRIEDVKHILDILEMEKNFA

>Q4VZK5 | RK2_CUCSA
MAIHLYKTSTPSTRNGAVDSQVKSNPRNNLIYGQHRCGKGRNARGIITAGHRGGGHKRLY
RKIDFRRNEKDIYGRIVTIEYDPNRNAYICLIHYGDGEKRYILHPRGAIIGDTIVSGTEV
PIKMGNALPLTDMPLGTAIHNIEITLGKGGQLARAAGAVAKLIAKEGKSATLKLPSGEVR
LISKNCSATVGQVGNAGVNQKSLGRAGSKCWLGKRPVVRGVVMNPVDHPHGGGEGRAPIG
RKKPATPWGYPALGRRSRKRNKYSDNLILRRRSK
>Q4VZL1 | RR15_CUCSA
MVKNSFSSVISQEEKKENGGSVEFQVVSFTNKIRRLTSHLELHKKDYLSQRGLRKILGKR
QRLLSYLSKKNKMRYKELINQLDIRESKTQ
>Q4VZM8 | RR19_CUCSA
MARSLKKNPFVGNHLLKKINKLNTKGEKEIIVTWSRASTIIPTMIGHTIAVHNGKDHLPV
YITDRMVGHKLGEFAPTRNFRGHVKNDNRSRR
>Q4VZM9 | RK22_CUCSA
MIKTKKNRYEVYALGQHICMSPHKARRVIDQIRGRSYEETMMILELMPYRACYPILKLVY
SAAANASHNMGFNERDLVISKAEVNQGTTVKKLKPRAQGRSYPIKRPTCSITIVLKNTSV
NEEEFKRYQYTINQPGGLITKEKYTDMRCYDMYNNGGLWDKK
>Q4VZP1 | RPOB_CUCSA
MLGGGNERMSTIPAFNQIQFEGFCRFIDHGLTEELSKFPKIEDTDQEIEFQLFVETYKLV
EPLIKERDAVYESLTYSSELYVSAGLIWKTRRDMQEQTIFIGNIPLMNSLGTSIVNGLYR
IVISQILQSPGIYYRSELDHNGISVYTGTIISDWGGRLELEIDRKARIWARVSRKQKISI
LVLSSAMGSNLREILENVCYPEIFLSFLNDKEKKKIGSKENAILEFYQQFSCVGGDPVFS
ESLCKELQKKFFQQRCELGRIGRRNLNQRLNLDIPENNTFLLQRDILAAADHLIGLKFGM
GTLDDMNHLKNKRIRSVADLLQDQFGLALVRLENMVRGTICGAIRHKLIPTPQNLVTSTP
LTTTFESFFGLHPLSQVLDRTNPLTQIVHGRKLSYLGPGGLTGRTASFRIRDIHPSHYGR
ICPIDTSEGINVGLIGSLAIHARIGHWGSLETPFYEISERSKKVRMLYLSPSRDEYYMVA
TGNSLALNPGIQEEQIVPARYRQEFLTIEWEQVHLRSIFPFQYFSIGASLIPFIEHNDAN
RALMSSNMQRQAVPLSRSEKCIVGTGLERQVARDSGVAAIAEHGGKIIYTDTDKIIFSGN
GYTRRIPLVMYQRSNKNTCMQQKSQVHQGKCIKKGQILADGAATVGGELALGKNVLVAYM
PWEGYNFEDAVLISERLIYEDIYTSFHIRKYEIQTHVTSHGPERITNEIPHLEARLLCNL
DKNGIVMLGSWVETGDILVGKLTPQMAKESSYAPEDRLLRAILGIQVSTSKETCLKLPIG
GRGRVIDVRWIQKKGGSSYNPEIIRVYISQKREIKVGDKVAGRHGNKGIVSKILPREDMP
YLQNGRPVDMVFNPLGVPSRMNVGQIFECSLGLAGSLLDRHYRIVPFDERYEQEASRKLV
FSELYEASKQTASPWVFEPEYPGKSRIFDGRTGNPFEQPVIIGKPYILKLIHQVDDKIHG
RSSGHYALVTQQPLRGRAKQGGQRVGEMEVWALEGFGVAHILQEMLTYKSDHIRARQEVL
GTTIIGGTIPKPEDTPESFRLLVRELRSLALELNHFLVSEKNFQINRKEA
>Q4VZP2 | RPOC1_CUCSA
MIDRYKHQQLRIGLVSPQQISAWANKTLPNGEIVGEVTKPYTFHYKTNKPEKDGLFCERI
FGPIKSGICACGNYRVIGDKKEDSKFCEQCGVEFVDSRIRRYQMGYIKLACPVTHVWYLK
RLPSYIANLLDKPLKELEGLVYCDFSFARPIAKKPTFLRLRGSFEYEIQSWKYSIPLFFT
TQGFDTFRNREISTGAGAIREQLADLDLRLIIDYSLVEWKELGEEGPAGNEWEDRKVGRR
KDFLVRRMELAKHFIRTNIEPEWMVLCLLPVLPPELRPIIQIDGGKLMSSDINELYRRVI
YRNNTLIDLLTTSRSTPGELVMCQEKLVQEAVDTLLDNGIRGQPMRDGHNKVYKSFSDVI
EGKEGRFRETLLGKRVDYSGRSVIVVGPSLSLHRCGLPREIAIELFQTFLIRGLIRQHFA
SNIGVAKSKIREKEPIVWEILQEVMQGHPVLLNRAPTLHRLGIQAFQPILVEGRAICLHP

LVCKGFNADFDGDQMAVHVPLSLEAQAEARLLMFSHMNLLSSAIGDPISVPTQDMLIGLY
VLTSGNRRGICANRYNPSNRKNHKNEKIYNNNYKYTKEPFFCNSYDAIGAYRQKRINLDS
PLWLRWRLDQRVIASREAPIEVHYESLGTHHEIYGYYLIVKSIKKEILCIYIRTTVGHIS
LYREIEEAIQGFCRACSYGT
>Q4VZP3 | RPOC2_CUCSA
MEVLMAERADLVFHNKVIDGTAIKRLISRLIDHFGMAYTSHILDQLKTLGFQQATATSIS
LGIDDLLTIPSKGWLVQDAEQQSLILEKHHHYGNVHAVEKLRQSIEIWYATSEYLRQEMN
PNFRMTDPFNPVHIMSFSGARGNASQVHQLVGMRGLMSDPQGQMIDLPIQSNLREGLSLT
EYIISCYGARKGVVDTAVRTSDAGYLTRRLVEVVQHIVVRRTDCGTIRGILVSPGNRMIP
ERIFIQTLIGRVLADDIYMGPRCIGVRNQDIGIGLINRFITFQTQPISIRTPFTCRSTSW
ICRLCYGRSPTHGDLVELGEAVGIIAGQSIGEPGTQLTLRTFHTGGVFTGGTAEHIRAPS
NGKIKFNEDLVHPTRTRHGHPAFLCYIDLYVTIESEDIIHNVTIPPKSLLLVQNDQYVES
EQVIAEIRAGTYTLNLKERVRKHIYSDSEGEMHWSTDVYHAPEFTYSNVHLLPKTSHLWI
LSGGSCGCSLVPFSLYKDQDQINVHSLCVERRYISSLSVNNDKVGQKFYGPDLSGKNENG
IPDYSELNPILCTGQSNLTYPAIFHGNSDLLAKRRRNGFIIQFESLQEREKELRPPSGIS
IEIPINGIFRRNSILAFFDDPQYRRNSSGITKYGTIGVHSILKKEDLIEYRGVKDFKPKY
QMQMKVDRFFFIPEEVHILPESSSIMVRNNSIIGVATRLTLSIRSRVGGLVRVEKKKKRI
ELKIFSGDIHFPGEMDKISRHNGILIPPERVKKNSKKSKKSKNWIYVQWITPTKKKYFVF
VRPVIIYELADGINLVKLFPQDLLQERDNLELRVANYILYGNGKPIRGISGTSIQLVRTC
LLLNWDRDKKSSSIEDARASFVEVSTNGLVRNFLRIDLGKSDTAYIRKRKDPSGSGLIFN
NESDRTNINPFFSIYSKTRVPQSPSQNQGTIRTLLNRNKERQSLIILSASNCLQIDLFND
VKDYNVIKESSKKDPLISIRNSLGPLGAAPQIVNFYSFYYLITHNPISLTKYLQLDNLKQ
IFQVLKYYLMDENGGILNSDPCINIVFNTFNLNWHFLHDNYHNNYCEETPTRISLGQFFF
ENVCIAKNRPHLKSGQIIIVQVDSLVIRSAKPYLATSGATVHRHYGEILYEGDTLVTFIY
EKSRSGDITQGLPKVEQVLEVRSIDSISMSLEKRIEGWNERITRILGIPWGFLIGAELTI
VQSRISLVNKIQKVYRSQGVEIHNRHIEIIVRQITSKVLVSEDGMSNVFSPGELIGLLRA
ERTGRALEEAICYRAVLLGITKASLNTQSFISEASFQETARVLAKAALRGRIDWLRGLKE
NVVLGGMIPVGTGFRELAHRSRQHNNIPLETPPKKIFEGEMRDILFHHKELFDFFISTNL
HDTSEQEFLGFNDS
>Q507P6 | MATK_RANGL
MEELQRYLKMDRSRERDFLYPLLFQEYIYALAHDFGLTKSIPYESMQILSYDNKYSSLIV
KRLIIRMYQQKHLIILDNDSNQKNFLGHNKNLYSQMISEGFAVIVEIPFALRLVSSYQGK
EIEKSINLRSIHSTFPFLEDKFVHLNHVLDILIPYPIHLELLVQNLRCWIQDASFLHLLR
FFLYEYHNWNSLTTQKTKQNPLFFKENRRFFLFLYNFHVYESESIFLFLRKKSYHLRSTS
SIAFLDRTHFYGKIEHFQVVFRNYFHTILWLFKDPFMHYFRYQGKSIMSSKGTPLLMKKW
KYYLVNLWECHFYFWSQPDRIHINQLSNHFLDFLGYLSSVRPNPSVVRNQMLENAFIIDI
AINKLDTIVPIIPLIGSLAKAKFCNLSGQPVSKPAWTDSPDSDIIDRFGRICRNVSHYYS
GSSKKKTLYRIKYILRLSCARTLARKHKSTVRSFLKRLGSEFLEEFLIEEEQVLSFILPK
ISSSSQRLSKERVWYFDIIRINDLMDLS
>Q507Q9 | MATK_RANLI
MEELQRYLKMDRSRERDFLYPLIFQEYIYVLAHDFGLTKSIPYESMQILSYDNKYSSLIV
KRLIIRMYQQKHFLILDNDSNQKKFLGHNKNLYSQMISEGFAVIVEIPFALRLVSSYQGK
EIKKSINLRSIHSTFPFLEDKFVHLNHVLDILIPYPIHLELLVQNLRCWIQDASFLHLLR
FFLYEYHNWNSLTTQKTKQNSLFLKENRRFFLFLYNFHVYESESIFLFLRKKSYHLRSTS

SIAFLDRRRFYGKIEHFKVVFHNDFHTILWLFKDPFMHYFRYQGKSIMSSKGTLLLMKKW
KYYLVNLWECHFDFWSQPNRIHINQLSNRFLDFLGYLSGVRPNPSVVRNQMLENAFIIDI
AINKLDTIVPIIPLIGSLAKAKFCNLSGQPVSKPAWTDSPDSDIMDRFGRICRNVSHYYS
GSSKKKTLYRIKYIFRLSCARTLARKHKSTVRSFLKRLGSEFLEEFLIEEEEVLSFILPK
ISSSSQRLSKERIWYFDIIRINDLMDLS
>Q507T3 | MATK_RANRE
MEELQRYLKMDRSRERDFLYPLLFQEYIYALAHDFGLTKSIPYESMQILSYDNKYSSLIV
KRLIIRMYQQKHLIILDNDSKNKNFLGHNKNLYSQMISEGFAVIVEIPFALRLVSSYQGK
EIEKSINLGSIHSTFPFLEDKFVHLNHVLNILIPYPIHFELIVQNLRCWIQDASFLHLLR
FFLYEYHNWNSFTTQKMKQNSLFLKENRRFFLFLYNFHVYESESIFLFLRKKSYHLRSTS
SIAFLDRTHFFGKIEHLKVVFRNDFHTMLWLFKDPFMHYFRYQGKSIMSSKGTPLLMKKW
KYYLVNLWECHFYFWSQPNRIHINQLSNIFLNFLGYLSSVRPNPSVVRNQMLENAFIIDI
SINKLNTLVPIIPLIGSLAKAKFCNLSGQPISKPAWTDSLDSDIIDRFGRICRNVSHYYS
GSSKKKTLYRIKYILRLSCARTLARKHKSSVRSFLKRLGSEFLEEFLIEEEQVLSFILPK
ISSSSQRLSKERIWYFDIIRINDLMDL
>Q52TG6 | MATK_TYPAN
MKQLHVQRYLEKVRSRKQHFLYPLLFKEYIYAFAHDYGLNGSIFYEPAEIIGNDNKSSSV
LVKRLIIRMYQQNYLINSINHSNQNRFIGHNNYFYSHFFSLMISESFAVIMEIPFSLRLV
SSPEEKEIPQFQNLRSIHSIFPFLEDKLSHLNYVSDILIPHPIHFEILVQILQCRIQDVP
SLHLLRFFLHEYHNWNSLITSKKSIYVFSKENKRLFRLLYNFYVFECEFVFVFLRKQSSY
LRLTSFGTFLERIHFYGKIEHLLVVYRNYFNKTLWFFTDPFMHYVRYQGKAILASKGTHL
FMKKWKCYLVNFWQYYFHFWSQPHRIHINQLSNYSFHFLGYLSSVLRNLLVVRNQMLENS
YLIDTVTKKFDTIVPVIPLIGSLSKAKFCTLLGHPISKPIWTDLSDCDIIDRFGRICRNL
SHYYSGSSKKRSLYRIKYILRFSCARTLARKHKSTVRTFLQRLGSVLLEEFFTEEEQVLS
LIFPKTTPFSLHGSHRERIWYLDIIRINDLVNH
>Q56B59 | MATK_AESPA
MKEFKIYLELDGSQQHNFLYPLLFREYIYALAHDYGLNKSTISLETVGYDNKSSSLSVKR
LITRTYQRLQRIHLSIYANDSNPNHFIGHNNNLYSQILSEGFAVIVEIPFSLRLVSFLEE
KAKEKEMAKSHNFQSIHSIFPFFEDNFSHLNYVLDVLIPHPIRPEILVQTFRSWVKDASS
LHLLRFFLHEYFNWDSLITPKESISIFLTSNPRFFLFLYNSHGYEYESIFFFLRNQSSHL
RSTSSGLLLEQIYFYGKVEDLVEVFANDFQDILWLFKDPIMHYVRYQGKCILASKDTPLL
MNKWKYYLVNLWQWHFRVWFQLGRVHINHLYKDYINFLGYLSIGRLNTLVVRSQMLENAF
LIDNAMKKFETTLPIIPLIGSLTKARFCNPLGHPISKLTWADSSDSHIIDRFVRICRNLS
HYHSGSSKKKSLYRIKYILRVSCFKSLVRKHKSTVRVFLKRLGLEFLEEFLTEEEHVLSV
IFPRALFISRRLYKGRVWYLDILCINDLVNHDKFEILSN
>Q56P11 | RPOC2_LACSA
MEVLMAERPTQVFHNKVIDGTAMKRLISRFIDHYGIGYTSHILDQVKTLGFRQATAASIS
LGIDDLLTIPSKRWLVQDAEQQSFILEKHHHYGNVHAVEKLRQSIEIWYATSEYLRQEMN
PNFRMTDPFNPVHIMSFSGARGNASQVHQLVGMRGLMSDPQGQMIDLPIQSNLREGLSLT
EYIISCYGARKGVVDTAIRTSDAGYLTRRLVEVVQHIVVRRTDCGTVRGISVSPRNGMMT
DRIFIQTLIGRVLADDIYIGSRCIATRNQDIGVGLVSRFITFRAQPISIRTPFTCRSTSW
ICQLCYGRSPAHDDLVELGEAVGIIAGQSIGEPGTQLTLRTFHTGGVFTGGTAEHVRAPS
NGKIKFNEDLVHPTRTRHGHPAFLCSRDLYVTIESEDIIHNVCIPPKSFLLVQNDQYVES
EQVIAEIRARTSTLNLKEKVRKHIYSDSEGEMHWNTDVYHAPEFTYGNIHLLPKTSHLWI

LLGEPWRYSLGPCSIHKDQDQMNAYSLSVKPRYIANPSVTNNQVRHKFFSSYFSGKNQKG
DRIPDCSELNRMTCTDHSNLRYPAILDGNSDLLAKRRRNRFIIPLESIQEGENQLIPSSG
ISMEIPRNGILRRNSILAYFDDPRYIRKSSGLTKYETRELNSIVNEENLIEYRGVKVFWP
KYQKEVNPFFFIPVEVHILSESSSIMVRHNSIIGADTQITFNRRSRVGGLVRVKKKAEKM
KLIIFSGDIHFPGKTNKAFRLIPPGGGKPNSKEYKKLKNWLYIQRMKLSRYEKKYFVLVQ
PVVPYKKTDGINLGRLFPPDLLQESDNLQLRVVNYILYYDPILEIWDTSIQLVRTSLVLN
WDQDKKIEKACASFVEIRTNGLLRYFLRIDLAKSPISYTGKRNDLSGSGLISENGSDRAN
VNPFSSIYSYSKSRIKESLNPNQGTIHTLLNRNKESQSLIILSSSNCFRIGPFNDVKSPN
VIKESIKKNPLIPIRNSLGPLGTGFPIYNFDLFSHLITHNQILVTNYLQLDNFKQIFQIL
KYYLLDENGQIYNPYSCSNIILNPFHLNWYFLHYNYCEETSPIVSLGQFLCENVCIAKKG
PHLKSGQVLIVQVDSVVIRSAKPYLATPGATVHGHYGEILYEGDTLVTFIYEKSRSGDIT
QGLPKVEQVLEVRSIDSISMNLEKRIEGWNKSITRILGIPWAFLIGAELTIVQSRISLVN
KVQKVYRSQGVQIHNRHIEIIVRQITSKVLVSEDEMSNVFSPGELIGLLRAERMGRALEE
AICYQAVLLGITRASMNTQSFISEASFQETARVLAKAALLGRIDWLKGLKENVVLGGMIP
VGSGFKTPSSEPNNIPNNIAFELQKKNLLEGEMKDILFYHRKLFDSCLSNNFHDTQEQSF
F
>Q56P13 | RPOB_LACSA
MSTIPGFNQIQFEGFCRFIDQGLTEELSKFPKIEDTNQEIDFELFLERYQLVEPSIKERD
AVYESLTYSSELYVSARLIWKNDRRRYIQEQTILIGKIPLMTSLGAFIVNGIYRIVINQI
LQSPGIYYQSELNDNGISVYTGTIISDWGGRLELEIDRKTRIWVRVSRQQKLSILVLLSA
MGLNIREILENVCYPELFLSFLNDKKQIGSKENAILEFYQQFACVEGDPVFSESLSKDLQ
KKFFQQRCELGGIGRRNMNRRLNLDIPQNNTFLLPRDILAAADRLIRIKFGMGTLDDMNH
LQNKRIRSVADLLQEQFGLALVRLENMARGNIYAALKHNWTPTPQNLVNSTPLTDTYKVF
FRLHPLSQVLDRTNPLTQIVHGRKLSYLGPGGLTARTATFPIRDIHPSHYGRICPIDTSE
GINVGLIGSLAIHARIGRWGSLESPFYKISERSKGARMLYLSPGRDEYYMVAAGNSLALN
QGIQEEQVVPARYRQEFLTIAWEQVHLRSIFSFQYFSIGASLIPFIEHNDANRALMSSNM
QRQAVPLSQSEKCIVGTGLEGQAALDSGALAIAEHEGEIIYTDTDKILLSGNGDTLRIPL
VMYQRSNKNTCMHQKPQVQRGKCIKKGQILAYGAATVGGELALGKNVLVAYMPWEGYNFE
DAVLISERLVYEDIYTSFHIRKYEIQINQGSERVTNEIPHLEVHLLRNLDKNGIVMLGSW
VETGDILVGKLTPQMVKESSYAPEDRLLRTILGMRVYTSKETCLKLPIGGRGRVIDVRWV
QSSKTDETEKTESIRVYILQKREIKVGDKVAGRHGNKGIISKILPRQDMPYLQDGRPVDM
VFNPLGVPSRMNVGQIFESSLGLAGGLLDRHYRIAPFDERYEQEASRKLVFSELYEASKQ
TVNPWIFEPESPGKSRIFDGRTGDPFEQPVIIGKPYILKLIHQVDDKIHGRSSGRYSRLT
QQPLKGRAKKGGQRVGEMEVWALEGFGVAYILQEMLTYKSDHIRARQEVLGTIIFGGRIP
TPEDAPESFRLFVRELRSLALELNHFLVSEKTFQLNRKEA
>Q589A4 | RR7_SILLA
MSRRGTVEEKTAKSDPIYRNRLVNMLVNRILKHGKKSLAYQIIYRAVKKIQQKTETNPLS
VLRQAIRGVTPDIAVKARRVGGSTHQVPIEIGSTQGKALAIRWLLGAARKRPGRNMAFKL
SSELVDAAKGSGDAIRKKEETHRMAEANRAFAHFR
>Q589B1 | PSBK_SILLA
MLNIFSLIGLNSALYSSSCFFAKLPEAYAFLSPIVDFMPVIPLLFFLLAFVWQAAVSFR
>Q589B8 | RPOC2_SILLA
MAERANLVFHNKSIDGTAMKRLISRLIDHFGMAYTAHILDQVKTLGFQQATATSISLGID
DLLTIPSKGWLVQDAEQQSLILEKHHHYGNVHAVEKLRQSIEIWYSTSEYLRQEMNPNFR

MTDPYNPVHIMSFSGARGNVSQVHQLVGMRGLMSDPQGQMIDLPIQSNLREGLSLTEYII
SCYGARKGVVDTAVRTSDAGYLTRRLVEVVQHIVVRRTDCGTIRGISVSPRNGRMPERIF
TQILIGRVLADDIYLGSRCIATRNQDIGVGLVNRFITFRTQPIAIRTPFTCRSTSWICRL
CYGRSPTHGDLVELGEAVGIIAGQSIGEPGTQLTLRTFHTGGVFTGGTAEHVRAPYNGKI
KFNEGLVHPTRTRHGHPAFLCYIDLYVTIESEDSLHNVNIPPKSFLLVQNDQYVESEQVI
AEIRAGASTLNFKEKVRKHIYSDSEGEMHWSTDVYHAPEFTYGNVHLLPKASRLWILSGR
PYRSSVVSFSLHKDQDQTTGNSLSFEQKIYNLSMTNDQVQNKFLDPYIKKSSKKEDRSPN
YSELNGMSHCNHIYPAKNSDLLAKRRKNRLIIPFQFQLSQEREKELMSLSNGISIEIPIN
GIFRRNSIFAYFNDPRYRTKSSGITKYETIETHSSVKKEDLIEYRGVKEFRPKYQMKVDR
FFFISQEVHILPGSSSIMVRNNSIIGVDTQITLNTRSRVGGVVRVERKKKKIELKIFSGD
IYFPGETDKISRHSGILIPPGKTNYKESKNIKNWLYVQRITPTKKKYFVLVRPVVTYEIT
DGINLATLLPQDLLQERGNVQLRVFNYILYGNGKVTRGIYDTSIQLVRTCLVLNWNQDKK
DSSIEEARASFVEVRTNGIIRYFLKIGLMNRALSYIGKRNNPPLFSDDGLEYTNMNPFFS
IYSKPKLQQAFNPNQGTVRMLVGKNKECQSFIILSSSNCFRMGPFTLNGVKYPKESIKKD
RLILIKNSFGPLGTVLNFVNFFFFYHLITHNQILVNNYLQLDNLKQTCQVLKYQYYLMDE
NGIIYNPDFCSNIILNPFNLHWDFLHYNFCDETSTKMRLGQFICENICIKKNGLHLKPGQ
IIIVQFDSVVIRSAKPCLATPGATVHGHYGEIIYEGDTLVTFIYEKSRSGDITQGLPKVE
QVLEVRSVESISMNLEKRINGWNERIKKILGIPWGFLVGAELTIAQSRISLVNKIQKVYR
SQGVQIHDRHIEIIVRQITSKVLVSEDGMSNIFLPGELIGLFRAERTGRALEEAICYRTI
LLGITRASLNTQSFISEASFQETARVLAKAALRGRIDWLKGLKENVVLGGMIPVGTGFKG
FVHRSNQHKSISIPLKIKNKNRLEGEMRDILFYHRELLDSCFLKNLGDRSKQQ
>Q589B9 | RPOC1_SILLA
MIDQYKHQQLRIGLVSPKQISAWATKILPNREIVGEVTKPYTFHYKTNKPEKDGLFCERI
FGPIKSGICACGNYRVIRNEKEDPKFCEQCGVEFVDSRIRRYQMGYIKLACPVTHVWYLK
RLPSYIANFLDKPLKELEGLVYCDYPNFSFARPIAKKPTFLRLRGLFEYEIQSWKYSIPL
FFTTQGFDAFRNREISTGAGAIREQLADLDLRTIIDYSFAEWKELGEEDPTGNEWEDRKV
GRRKDFLVRRMELAKHFIRTNIEPEWMVLCLLPVLPPELRPIIQIDGGKLMSSDINELYR
RVIYRNNTLTDLLTTSRSTPGELVMCQEKLVQEAVDTLLDNGIRGQPMRDGHNKVYKSFS
DVIEGKEGRFRETLLGKRVDYSGRSVIVVGPSLSLHRCGLPREIAIELFQTFIIRGLIRQ
HLASNIGVAKSKIREKEPIIWKILQEVMQGHPILLNRAPTLHRLGIQAFQPILVEGRAIC
LHPLVCKGFNADFDGDQMAVHVPLSLEAQTEARLLMFSHMNLLSPAIGDPISVPTQDMLI
GLYILTSGTRRGICANRYNPWNRKNDQNERIDDKNYKYMEEPFFCNSYDAIGAYCQKRIN
LDSPLWLRWRLDQRIIASREAPVEVHYESLGTYHEIYAHYLIIRNIKKEIIFVYIRTTVG
HIYLYREIEEAIQGFSRACSYGKTVLK
>Q589C0 | RPOB_SILLA
MLREGNELMSTIPGFNQIQFEGFCQFIDQGLPEELYKFPKIEDTDQEIEFQLFVETYQLV
EPVIKEKDAVYKSLTYSSELYVSAGLIWKTGREIQEQTILIGNIPLMNSLGTFLVNGIYR
IVINQILQSPGIYYRSELDHNGISVYTGTIISDWGGRSELEIDRKARIWARVSRKQKISI
LVLSSAMGSNLKEILDNVCYPEIFLSFLNDKDKKNFGSKENAILEFYQQFACVGGDPVFS
ESLCKELQKKFFQQKCELGRIGRRNMNRRLNLDIPQNNTFLLPRDILAATDHLIGMKFGM
GTLDDMNHLKNKRIRSVADLLQDQFGLALVRLENVVRGTICGAIRHKLIPTPQNLVTSTP
LTTTYESFFGLHPLSQVLDRTNPLTQIVHGRKLSYLGPGGLTGRTASFRIRDIHPSHYGR
ICPIDTSEGINVGLIGSLAIHARIGLLGSLESPFYKISERSAMVQMLFLSPSIDEYYMVS
TGNSLALNQGIQEEQVVPARYRQEFLTIAWEQVHLRSIFPFQYFSIGASLIPFIEHNDAN

RALMSSNMQRQAVPLSQSEKCIVGTGLERQAALDSGILAIAEHEGKILYTDTDKIIFSGN
GDIQSIPLVMYQRSNKNTCMHQNPRIPRGKCIKKGQILADGAATVGGELALGKNILVAYM
PWEGYNFEDAVLISERLVYEDIYTSFHIRKYDIQTYVTSQGPERVTSEIPHLEAHLLRNL
DKNGIVRLGSWVETGDILVGKLTPQMAKESSYAPEDRLLRAILGIQVSTSKETCLKLPIG
GRGRVIDVRWIQKKGGSSYNPETIHVYILQKREIKVGDKVAGRHGNKGIISKILPRQDMP
YLQDGRPVDMVFNPLGVPSRMNVGQIFECSLGLAGGLLDRHYRIAPFDERYEQEASRKLV
FSELYQASKQTSEPWIFEPEYPGKSRIFDGRTGDLFEQPVIIGNPYILKLIHQVDDKIHG
RSSGHYALVTQQPLRGRAKQGGQRVGEMEVWALEGFGVAHILQEMLTYKSDHIKARQDVL
GTTIIGGTIPNPEDAPESFRLLIRELRSLALELNHFLVSEKTFQINRMEA
>Q5D1B9 | MATK_OXYPI
MKEYQVLLERDRSRQQDFLYPLIFREYVYGLAYSHDFNRSTFVENVGYDKKYSLLIVKRL
ITRMYQQNHLIISANDSKKNPFLGYNKNFYSQIISEGFAIVVEIPFFLQFSSSLEEADIV
KSYKNLRSIHSVFPFLEDKFPYLNYVSDIRIPYPIHLEILVQILRYWVKDAPFFHLLRLF
LYNFCNRNSFLTPKKSISTFSKSNPRLFLFLYNFYVCEYESIFLFLRKKSSHLRLKSFSV
FFERIFFYAKREHLVEVFAKDFSSTVTFFKDPLFHYVRYQGKSILASKNAPLLMNKWKHY
FIHLWECFFDVWSQPGTIHIKQLSEHSFYLLGYFSNVRLNRSVVRSQMLQNTFLIEIVSK
KLDTIVPIIPIIRSLAKAKFCNVLGHPISKAVWADSSDFDIIERFLRICRNLSHYYNGSS
KKKSLYRIKYILRLSCIKTLACKHKSTVRAFLKRSGSEELLEEFFTEEEEILSLIFPRAS
CTLQKLHGNRIWYLDILFSNDLVNHE
>Q5D1C1 | MATK_ASTSI
MKESQVFLERDRSRQQDFLYPLIFREYVYGLAYSHDLNRSTFVENVGYDNDNKYSLLIVK
RLITRMYQQNHLIISANDSNKNPFLGYNKNFYSKIISEGFAIVVEIPFFLQFSSSLEEAE
IIKSYKNLRSIHSIFPFLEDKFPHLNYVSDIRIPYPIHLEILVQILRYWVKDAPFFHLLR
LFLYNFCNRNSWITPKKSISTFSKSNPRLFLFLYNFYVCEYESIFLFLRKKSSHLRFKSF
SVFFERIFFYAKREHLVEVFAKDFSSTLTLFKDPLIHYVRYQGKSILASKNAPLLMNKWK
HYFIHLWECFFDVWSQPGTIHIKQLSEHSFYLLGYFSNVRLNRSVVRSQMLQNTFLIEIV
SKKLDIIVPIIPIIRSLAKAKFCNVLGHPISKAVWADSSDFDIIDRFLRICRNLSHYYNG
SSKKKSLYRIKYILRLSCIKTLACKHKSTVRAFLKRSGSEELLEEFFTEEEEILSLIFPR
ASATLHKFNGNRIWYLDILLSNDLVNHE
>Q5F4M7 | MATK_CALOC
MEELQGYLEIDGFRQHHFLYPLLLQEYIYALAHDHGLNGSILSEPMENLSHDNKSSSLIV
KRLITRMHQQNHFIISVNDSNQKGFVGHNKNFHSQKISEGFAVIVEIPFSLQLVSSLEEK
EIAKFHNSRSIHSIFPFFEDKLSHLNHVSDILIPYPIHLEILVQTLRCWIQDAPSLHLLR
FFLHEYWNSNSLITPKKSISFFSKENQRLFLFLYNSHVYECESVFIFLRKQSSHLRSTSF
GSFLERTHFYGKIEHLVVVLGNDFPKTLWLFKDPFVHYVRYQGKSILASRGTQFLIKKWK
YHLVNFWQCHFYLWSQPDRIHLNQLCNHSFYFLGYLSSVQLNSSVVRSQMLENAFLMDTA
IKKFETIVPIIPLIGSLAKAKFCNGSGHPISKPFRTDLSDSEILNRFGRICKNLSHYHSG
SSKKQSLYRIKFILRLSCARTLSRKHKSTVRAFLKRLGSELLEEFLTEEEQVLSLIFPRT
PSHRPHRERIWYLDIICINDLANHE
>Q5GA98 | MATK_MIMGU
MEESQRYLQLERSQQHDFLYPLIFQEYIYAFAYDRGFSRSSLSENPSYNNKSSLLIVKRL
ITRMYQQNHFIIYYPNDSNQNTFWARRNNLYSPIISEGFAFIVEIPFSLGLIYCLEGKNK
KIVKSQNLRSIHSIFPFLEDNFSHLNFVLDILIPQPVHVEILVQTLRCRVKDASSLHLLR
FFLNEYCNSKSLITPQKASSSFSKKNKRLFLFLYNSHLCEYESIFVFLRTKSSHLRSTSL

GVLLERIYFYGKIERVVNIFVKVKDFQANLRLVKEPCMHYIRYQRKSILASKGTSLFMNK
WKSYLVAFWQWHFSQWFHPRRIYINHISNHSLEFLGYLSNVRMNPSVVRSQIIENSFLIN
NAIKKVDTLIPIIPLIAAXXKAKFXNVLGHPIXKSVRADLSDFNIIDRFGRICRNLSHYY
SGSSKKKSLYRIKYILRLSCARTLARKHKSTVRTFLKRLGSEFLEEFLMSEEDVLFFTFP
KASSTLWGVYRSRIWYLDIISINDLANHKSKL
>Q5GAA8 | MATK_CRAPL
MEEIQRYLQLKRSQQHDFLYPLIFQEYIYAFAHDRDFGRSILSENLGSKSKSSLLVVKRL
ISRMYQQNRFILSLNDSNQNPFWTCNNNFDSQIISEGFALIGEIPFSLRFKSCLEEKNKV
NSQSLRSIHSIFPFLEDNFSHLNYILDILITHPVHVEILVQNLRYWLKDASSLHLLRFLL
NDYWNSLITPKKASSSFSKKNQRLFLFLYNSHVCEYEYFFVFIRKQSSHLLSTSYGVFLE
RIYCYEKVERLXNVFIKVKDLQANLWLVKEPCMHYVRYQRKFILASKGTSVLMNKWKCYL
VTFWQWHFSLWFHPRRIYINQLSNHSLEFLGYLSSVRMNPSVVRSQILENSFLINNGIKK
LETLVPIFLLITSLAKAKFCNVLGHPISKPVWADLSDSNIIDRFGRISRNLSHYYSGSSK
KKSLYRVKYILRLSCARTLARKHKSTVRAFLKRLGSELLEEFLMSEEDILSLTFPKPSST
LRGVYRSRIWYLDIIWINDLANYKSKI
>Q5GAA9 | MATK_ARTFM
MEEIQRYLQLERSQQHDFLYPLIFQEYIYAFAHDRDFGRSILSENLGYDSKSSLLVVKRL
ISRMYQQNRFILSLDDSNQNPFWTCNNNFYSQTVSEGFAFIVEIPFSLRFKSCLEEKKIV
NSQNLRSIHSIFPFLEDNFAHLNYILDILIPHPVHVEILVQNLRHWLKDASSLHLLRFLL
NEYWNWNSLITPKKASSSFSKKNQRLFLLLYNSHVCEYEYFFVFIRNQSSHLRSTSSGVF
LERIYFYEKIERLVNIFIKVKDLQANLWLVKEPCMHYVRYQRKSILASKGTSVLMNKWKC
YLVTFWQWHFSLWFHPRRIYINQLSNHSLEFLGYLSSVRMNPSVVRSQILENSFLINNAI
KKLETLVPIFLLIASLAKAKFCNVLGHPISKPVWADLSDSNIIDRFGRISKNLSHYYSGS
SKKKSLYRVKYILRLSCARTLARKHKSTVRAFLKRLGSELLEEFLMTEEDILSLTFPKPS
SALRGVYRSRIWYLDIIWINDLANYK
>Q5GAB0 | MATK_OTACA
MEEIQRYFQLERSHQHDFLYPLIFQEYIYTFAHDRGCNRSILSENPCYENKSSLLIVKRL
ITRMCQQNHFLISSNDFNQNPFWGRNKNFDFQIISEGFALIVEIPFSLRLRASLEEKKIV
TFQNLRSIQAIFPFLEDSFSHLNFVLDISIPHSIHLEILVQTLRYWVKDASSLHLLRFFL
NEYCNSNSLITPKKVSSSFSKRNQRFFLFLYNSHVCEYESIFVFLRKQSSHLRSTSSGLL
LERIYFYAKMERLVNLFVKVKDFQVNLWLVKEPCMHYVRYQRKSILASKGVSLLINKWKS
YLVAFWQWHFSMWFHPRRISINQLSNHSLEFLGYLSSVRMNPSVVRSQILENSFLINNAL
KKFDTLVPINPLIASLAKAKFCNVLGHPISKPVWADFSDLNIIDRFGHISRNISHYYSGS
SXKNSLYRIKYILRLSCARTLARKHKSTVRVFLKRLGSELLEEFLMSEEDVLSLTFPKAS
STFWGVYRSRIWYLDIISINDLANHKSKF
>Q5GF59 | MATK_IRITE
MEELQGYLEKDRSRQQPFLYPLLFQEYIYALAHNRGLKGSLFYEPTEVFGYDSKSSLALV
KRLIIRIYQQNDFLSVVNDSNKNRFVSHHRDNFCYSHFYSQMISEGFAILVEIPFSPRLV
SYFEKKEIPKSHNLRSIHSIFPFLEDKLLHLNYVSDILIPHPIHMEILVQILQCWIQDVP
LLHFLRFFLHKYHNWNSFLITPKKSIFVFSKENKRLFRFLYNSYVSECEFLLVFLRKQSS
YLRLTSFGFFLERRHFYVKIERLQMQHLILIVVCRDFFQGTLWSFKDPFMHYVRCQGKAV
LASKGTHLLMKKWKYNFVNLWQYYFNFWYQSYRIHINQLSNYSFYFLGYLSSLLKNSSTV
RNQMLENSFLIDTVTNKLETLVPVIFLIGSLXKVQFCTVSGHPISKPIWADLSDSEIIER
FGRMCRNLSHYHSGSSKKQGLYRIKYILRLSCARTLARKHKSTGRTFLRRLGSGLLEEFF

TEEEQVLSLILPKKIPFTFYGSHKERIWYLDIIRINDLVNHS
>Q5GF70 | MATK_IRIOR
MEELQGYLEKDGSRQQPFLYPLLFQEYIYALAHDRGFKGSLFYEPAEVFGYDSKSSLALV
KRLIIRIYQQNDFLSAVHDSNKNQFVSHHHKNLGYSHFYSQMISEGFAILVEIPFSLRLV
SYFEKKEIPKSHNLRSIHSIFPFLEDKLLHLNYVSDILIPHPIHMEILVQILQCWIQDVP
LLHFLLFFLHKYHNWNSFLITPKKSIYVFSKENKRLFRFLYNSYVSECEFLLVFLRKQSS
YLRLTSFGPFLERRHFYVKMEHLQMQHLILIVVCRDYFQGTLWSYXXXXXXXXXXXXXVI
LASKGTHLLMKKWKYNFVNLWQYYFHFWYQSYRIHINQLSNHSFYFLGYLSSLPRNSSTV
RNQMLENSFIIDTVTKKFETIVPVIFLIGSLSKAQFCTVSGHPISKPIWADLSDSEIIER
FGRMCRNLSHYHSGSSKKQGLHRIKYILRLSCARTLARKHKSTVRTFLRRLGSGLLEEFF
TEEEQVLSFILPKTIPFTFYGSHKERIWYLDIIRINDLVNHS
>Q5GGS4 | MATK_CHIAL
MEEIQRYLQLDRSQQHDFLYPLIFQEYIYALAHHHSLNRSILLENPDYDNQLSFLIVKRL
ITRMYQQNHFIIFANDSNQNPFFGRNNNLYSQTISEGFSFIVEIPFYIRLIPSQAGKGIL
KSYNLRSIHSLFPFLENNFSHLNSVLDILIPRSVHLEILVQNLRYWVKDVSSLHLLRFLF
RESWNCNPLIATKKRGFGFEPKRSQRLLFFLYNSHVCEYESIFVFLRNQSSHLRSTSFGV
FLERIYFYGKIERLVEVFAKDFRASLWLFKDPFMHYVRYQGKSILVSKGTPLLMNKWKYY
LVNFWQSYFDLWVHSGRVYINQLPNHSLNFIGYLSSVRLNPSMVRSQMLENSFLINNAIK
KLDTLVPIVPLIGSLAKAKFCNLLGHPISKPAWAGLSDSDIIDRFGQICRNLSHYHSGSS
KKKSLYRIKYILRLSCAKTLARKHKSTVRAFLKRLGSEFLEEFLTLEEEVLSLTFPRASS
TFRGEYRSRIWYLDIIYINDLTNFQ
>Q5GIQ4 | MATK_SYZSA
MEEFQGYFELDRSRQHDFLYPLLFREYIYALAHDHGLNRSILFKNAGYDKKSSSIVVKRL
ITRMYQQNPLIFSANDSIQNPFFGHNKNLYSQIISEGFAVIVEIPFSLRLVSSLERKEIA
KSHNLRSIHSIFPFLEDKFSHLDYVSDVLIPYHIHLEILVQTLRYWVKDASSLHLLRFFL
HEYWNSLITPKKHITLFSKGNPRLFLFLYNSHICEYESIFLFLRNQSSHLRSTSSGIFFE
RIYFYVKIEHFAKVFFDNDFQCILWFFKDPFMHYVRYQGKSILASKDTPLLMNKWKYYLV
TLWQYHFYAWFQPVRIDINQLCKYSLDFLGYRSSVRLNSSVVRSQMLENSFLINNAMKKF
ETIVPIIPLIGSLSKANFCNTLGHPISKPTRADSSDSDIIDRFLRICRNLSHYHSGSSKK
KSLYRVKYILRLSCVKTLARKHKRTVRTFFKRLGSEFLEEFLTEEEVVLSLIFPRTYSTS
RRLYRGPIWYLDITSINDLVNYENE
>Q5GIR6 | MATK_BACCT
MEEFQGYLELDRYRQHDFLYPLLFREYIYALAHDHGLNKSILSENAGYGNKSSSIIVKRL
ITRMYQQNPLIFSANDSIQNPFFGHNKNLYSQIISEGFAVIVEIPFSLRLVSFLERKEIA
KSKNLXSIHSIFPFLEDKFSHLDYVSDVLIPYHIHLEILVQTLRYWVKDASSLHLLRFFL
HEYWNTLITPKKYITLFSKGNPRLFLFLYNSHICEYESIFLFLRNKSSHLRSTSSGIFFE
RIYFYVKIEHFVKVFFDNDFQCILWFFKDPFMHYVRYQGKSILASKDTPLLMNKWKYYLV
NLWQYHFYAWFQPGRININQLCKYSLDFLGYRSSVRLNSSVVRSQMLENLFLINNAMKKF
ETIVPIIPLIGSLSKANFCNTLGHPISKPTRSDSSDSDIINRFLRICRNLSHYHSGSSKK
KSLYRVKYILRLSCVKTLARKHKITIRTFFKKSGSEFLEEFLTEEEVVLSLIFPRTYSSS
RRLYREQSWYLDITSINDLVNYE
>Q5J2U8 | MATK_DROLU
MKIDEFQGYLELDRSWQDSFLYPLSFQEYIYALAHDHGLNRTILLENAGYENKYNFLIVK
RLIIRMYRQNQFILSTNDFQQNIFFASPQKIYSQVIAEVFSVIVEIPFSLRLLFSLEGKQ

IRKSHNLRSIHSIFPFLEDQFSHLNYVLDIRIPHPVHLEILVQTLRYWVKDAPSLHLLRF
FLYESHNWNSFFSLKKNISFLKKRNQRFFLFLYNSHVCEYESIFFFICNQSSHLQSTFYG
SLIERIHFYGKVEHLVKVFTKNFQVILWFFQDPLMHYVRYQGKSILASKATSLLINKWKY
YLVNFWQCYFSVWSQPKRIYINQLSKNSLDFTGFLSSVCLNTSVVRSQMLENSFIMDNAI
NKFDTIVPSIPLIGSLSKAKFCNVLGHPISKPVWTDLSDSEIIDRFGXICRSISHYYSGS
SKKTSLYRIKYILRLSCARTLARKHKSTVRSFLKRLGSEFLEEFFTEEEKVLSFLLPRDY
SISQRLYRGRIWYLDIFCIHDFANHEWLVMRLSK
>Q5J2V8 | MATK_RIVHU
MEKFQRYIELDRFWQHNFFYPLIFQEYIYAIAYNHGLNKSILLGNAVDKKYSLLIVKRLI
TRMYQQNHLILSPNASNQNSFWGYMKNLYSQMISEGFAVIVEIPFSLLLIYSLEEKEIIK
SHNLRSIHSIFPFLEDKFLHLNYVLDILIPYPIHIEILVQTLRYWLKDASSLHLLRFFLY
EYHNGNNLITSKKSISIFSKRNQRLFLFLYNFYMCEYESVLVFLRNRSSHLRSTSFGVFL
ERIYFHGKLEYLVKNLTKNFWVILWLFKEPFTHYVRYQGKSILASKSTSLLMHKWKYYLI
NFYQCNFSVWPQPRRIYINGLSNHSLDFMGFLSSVRLNSSVVRSQMLENSFLIEKTIKKF
DTIVPIIPLVGSLAKAQFCNVLGHPISKSVWTDLADSDIIDRFGRICRNIFHYYSGSSRK
KSLYRIKYILRLSCARTLACKHKSTVRAFLKRLGSEFLEEFFTEEEKVLSLILPRDSSTS
RGLYRGRIWYLDIICIHNFADDE
>Q5J2W0 | MATK_SILOT
MEEFQGYIEREGSWQHNFLYPLIFQEYLFRFAYGHGLNQSILLETSGNRKYSLLIVKRLI
TRMDQQNHLIPSANDSNQNDFWGHKHNFYSQMISEGFSGIVEIPFYRLLIASLEKQKKVK
SHNFRSIHSIFPFLEDKFLPLNSVLDILLPYPAHLEILVQILRDWVRDASSLHLLRFFLY
EDHNNNCNSLFTPKNSISFFFFRNQRFFVFLYNFHVCEYESIFFFLCNQSSHLRSTSYRA
LLERTFFYGKLDYLVNLFTKDFAVILWLFKDPSPHSVRYKGKFILASKGTFFLMHKWKFY
LIHFWQCHFSVWSHPRRIYLNRLSTHCLDFIGFLSSVQLTSSVVRSQMLENAFLIDNTIK
RFDPKIPISTLFGSLAKAQFCNRLGHPISKSVWIDLSDSDIIDRFGRICRNLSHYYSGSS
RKKSLYRLKYILQISCARTLARKHKSAARAFLKRLGSEFLEEFFTEHEKVLSLILPKNGS
NSRGFYRGSIWYLDIICIHNLANDE
>Q5J2Z9 | MATK_AMACA
MEKLQGHRELDRSWQHNFFYPLIFQEYIYVFAYDHALNKLILLENAIDKKYSLLIVKRLI
TRMYQQNHFILSVNDSNQNEIFGHKHKKNLYSQMITEGFAVIVEIPFSLLLISSLEGKEI
VESQNLRSIHSIFPFLEDKFLHLNYVLDILIPYPAHLEILVQTLRYWLKDASSLHLLRYF
LYEYRNWNSLIRPKESISPFSKRNRRFFLFLYNLLVYEYESIFVILRKQSSYLRSTSFGA
LLERIHFYGKIKYLVKVKVKVFGVILWLFKEPFLHYVRYQGKCLLASKGTSFLMYKWKYY
FIAFWQCHFSVWSQPRRIYINQLSNYSLDFMGFISNVGLNSSVIRSQMLENSFLVDNIIK
KFDTIVPIIPLVGSLAKAKFCNGLRHPISKSGWTDLSDADIIDRFGRICRNLSHYYSGSS
RKKSLYRIKYILRLSCARTLSRKHKSTVRAFLKRLGSEFLEEFFTEEDKVLSLILPRDSS
SSGFYRGRVWYLDIICIHNLANDE
>Q5J300 | MATK_AMAGR
MEKLQGHRELDRSWQHNFFYPLIFQEYIYVFAYDHALNKLILLENAIAKKSSLLIVKRLI
TRMYQQNHFILSVNDSNQNEIFGHKHKKNLYSQMITEGFAVIVEIPFSLLLISSLEGKEI
VESQNLRSIHSIFPFLEDKFLHLNYVLDILIPYPAHLEILVQTLRYWLKDASSLHLLRYF
LYEYRNWNSLIRPKESISPFSKRNRRLFLFLYNLLVYEYESLFVILRKQSSYLRSTSFGA
LLERIHFYGKLKYLVKVKVKVFGVILWLFKEPFLHYVRYQGKCLLASKGTSFLMYKWKYY
FIAFWQCHFSVWSQPRRIYINQLSNYSLDFMGFISNVGLNSSVIRSQMLENSFLVDNIIK

KFDTIVPIIPLVGSLAKAKFCNGLGHPISKSVWTDLSDADIIDRFGRICRNLSHYYSGSS
RKKSLYRIKYILRLSCARTLSRKHKSTVRAFLKRLGSEFLEEFFTEEEKVLSLILPRDSS
TLSGFYRGRVWYLDIICIHNLANDE
>Q5QA83 | CLPP_ACOGR
MPIGVPKVLFGSPGEEDAAWVDIYNRLHRERLLFLGQELDSEISNQLVGLMVYLSIEDKT
RDFFLFINSPGGWVIPGIGLYDTMQFVPPDVHTICMGLAASMGSFILVGGEITKRLAFPH
ARVMIHQPASSFYEAQAGEFVLEAEELLKLRETLTRVYVQRTGKPLWVVSEDMERDVFLS
ATEAQAHGIVDLVGDENMGDLV
>Q5SCW6 | RR16_HUPLU
MVKLRLKQYGGKQQATYRIVAIDARSRGXGRAIQEVGSYDPIKDQTQXNMPTIVYFIERG
AQPTETVKDILQKAEIFAQLRAKS
>Q5SCX1 | PSBK_HUPLU
MLKFYLENVFHLIFFAKLPEAYAVSDPIVNVMPIIPLFFFLLAFVWQASVSFR
>Q5SCY6 | RR7_HUPLU
MSRRDDTKADPIYRNRLVNMLVNRILKHGKKSLAYRILYNAMMNIRRETKYNPLFILRQA
IRQVTPNVTIETRRVGGSTYRVPTEIESTQGKALAIRWLLEASRKRPGRNIASKSSSELM
DAAENSGNAIRKREETHRMAEANRAFAHLR
>Q5SD08 | RR4_HUPLU
MSRYRGPRLKIIRRLGELPGLTRKTPKLKPDYINKSTPNKKVSQYCIRLEEKQKLRFHHG
LAEQQLLKYVRIARRAKGSTGQVLLQLLEMRLDNIIFRLGMAPTIPGARQLVNHKHILVN
NRAINVPSYRCKPKDIITTRDRPESRARITKNYEFSQTYKIPNHLTLHSLQSVGLVNKIV
DRESIDLNINELLVVEYYSRKA
>Q5SD15 | IF1C_HUPLU
MKKQKLIDMEGVVTESLPNTMFRVCLDNGCQVLTHVSGRMRRNYIRILPGDRVKVESSPY
DLTKGRITYRFHTKSSND
>Q5SD16 | RR8_HUPLU
MGNDTIANMITSVRNANLGKEETVRVPATNINRSIGKILLQEGFVKNLGELREGTNHFMI
LTLKYRGRKREPYITTLRRISKPGLRLYYNYKKIPKVLGGMGVVIPSTSCGTMTDREARQ
KQIGGEILCYVW
>Q5SD18 | RK16_HUPLU
MLSPKRTKFRKQHRGRLGGVSTRGNRICFGRFALQALEPAWITSRQIEAGRRAITRYARR
GGKIWIRIFPDKPITVRPAETRMGSGKGSPEYWVSVVKPGRILYEVGGIPETIARAAMKI
AAYKMPVRTQIIVATPIEMQNQ
>Q5SD19 | RR3_HUPLU
MGQKINPLGFRLGITKNHNSHWFAKPNNYSEFLQEDRKVRNCIDNYVHRHVKNSSNYGGV
ARVEIQRKTDLILVEIHTGFPALLVESHGQGIEQIRKDVQQNLLDSKIRRVHVTSTEIAK
PYGEPNILAEYIAIQLENRVAFRRTIRKAIELAKETDIKGIKIQIAGRLNGTEIARVEWA
REGRVPLQTLRAQIDYCYYPAQTIYGVLGIKIWIFRDEE
>Q5SD20 | RK22_HUPLU
MKIGNDSNKEVKIFVKNIHMSAYKLRRVVNQIRGHSYGEAVMILEFMPYRACYPVLKLVS
NAAENANHKMGLRKADLFVSEVKVDAGSFAKRLRLRAQGRNYPIHKPTCHITIILKKKSV
YKEEL
>Q5SD26 | RR11_HUPLU
MPRLIKKVGSLRRGKRGVPKGVIHIQASFNNTIVTVTDVRGQAVSWSSAGACGSRGTKKS

TPFAAQTAAENAIRMLIDQGGMEQAEIIMKGPGPGRDTASRAIRKSGVVLSFVRDITPMP
HNGCRPPKKRRV
>Q5SD27 | RR12_HUPLU
MPTIQQLIRNQRQPVENRTKSPALQGCPQRRGVCTRVYTTTPKKPNSALRKVARVRLTSG
FEITAYIPGIGHNSQEHSVVLVRGGRVKDLPGVRYHIVRGTLDAVGVKNRQQGRSKYGVK
KPK
>Q5SD28 | CLPP_HUPLU
MPIGVPKVPFRLPGDEDAVWIDVYNRLFRERLLFLGQQVDDELANQLICIMIYPNGEDDS
RDMYLYVNSPGGAVFAGISIYDAMQFVVPDVQTICIGLAASMGSFVLAGGEITKRIALPH
ARVMIHQPASSYYEGQVGECIMEAEEVLKIRDCITKVYAQRTGKPLWVVSEDTERDVFTS
AEEAKVYGVIDPVAVETSSNSPISKLTKS
>Q5SD37 | RR18_HUPLU
MNKSKRSFRRRLPPIRSGETINYRNISLLRRFISEQGKILSRRMNRLTSKQQRLITVAIK
RARISAPSPFLNNESRSSTNY
>Q5VH50 | MATK_BATMA
MEEFQVYLELHKAHNFLYPLFFQEYIYALAHNHRLNRLNRSSLFQNGDYDNKFSSLIVKR
LIFRMYQQNNFIISTKNLNQNTFFSHNKNLYYQIISVVLAVIVEIPFSIRFISSLEGKEL
VKSHNLQSIHSIFPFLEDKFSHLNYVLNVLIPHPIHLEILLQTLRYWIKDASSLHLLRFY
LYEYSNLKNFFIPKKSTLNPRFFLFLYNSYVYQYESILFFLRKQSSHLQSNSFGILIERI
YFYGKIESLLKVFIKKFQDILWLSKDPFMHYVRYRGKSILASKHGPLLISKWKYYFVNLW
QCHFYVWSQSKRVHIKQLSKDYLNFLGYLSSLRLNFLVVRSQMLENSYLIDNVIKKFHTK
IPMSSIIASLAKARFCNVLGHPISKSTWTDSSDSEILDRFVRIYRNLAHYYSGSSKKKNL
YRIKYILRVSCVKTLARKHKSTVRAFLKRLGSDFLEEFLLEEEQVLSLMFSRVFSVSQRF
YRVRIWYLDIFYINDFVNH
>Q5YJV9 | MATK_LUPCO
MEEYQDQVYLELDISRQQHFLYPLIFREYIYGLVYGHDFNGSIFSENVDYDNKSSLLIVK
RLITRMDQQNHLIISANDSKKKKFLSYNKNLYSQIISEGFAIVVEIPLSLQLNSSSEESK
IIKYYKNLRSIHSIFPFFEDKLTYLNYVSDARIPYPIHLEILVQVFRYWAKDAPFFHLLR
LFFYEYCNWNNLITPKKSISTFSKSNLRVFLFLYNFYVCEYESIFLFLRNKSSHLRLTSF
SVLFERIYFYGKIEHFLEVFAKDFSSTLSFFKELFIHYVRYQEKYILASKNASLLMNKWK
NYLIRLWQYHFDVWSQPRTIQINQFSEGSFHLLGYFSNVRLNRSAVRSQMLENSFLIEIV
MKKLETIVPIIPLIRSLAKAKFCNVLGHPISKPVWADSSDFHIIDRFLRICRNLSHYYNG
SSKKKSLYRVKYILRLSCIKTLARKHKSTVRAFLKRLGSEKLLEEFFTEEEEILSLVFQR
ASSTLQGFYRGRVWYLDIIFSNDLVNHE
>Q5YJX3 | MATK_GYMDI
MKEFQVYLELDRSLQHDFLYPLIFREYIYALAYDHGLNNSILVENLGYDNKSSLLIVKRL
ITRMYQQKHLILSANDSNKNQFWGYNKNLYSQIISEGFAVSVEIPFSLQLISSLEEAEIV
KSYNLRSIHSIFPFFEEKFPYLNYVSDVRIPYPIHLEILVQTLRYWVKDASSFHLLRLFL
YEYCNWNSLITPKKWISTFSKSNPRLFLFLYNFYVCEYESILIFLRNKSSYLRLTSSGVL
FERIYFYAKIEHRVEVFDKDFPSTLWFFKDPFIHYVRYQGKSILSSKNTPFFMNKWKYYL
IHLWQCHFYVWSQPGKIHINQLSEHSFYFLGYFSNVRLNPSVVRSQMLENSFIIENVMKK
LDTIIPIIPLIRSLAKAKFCNVLGHPISKPVWADSSDFYIIDRFLRICRNLSHYYNGSSN
KKSLYRIKYILRLSCIKTLARKHKSTVRVFLKRLGSKFLEEFFTEEEEILSLILPRASFT
LQRLYRGRIWYLDIFYFHQ

>Q5YJY5 | MATK_PARAU
MEEFQAYLELDRSRQHDFLYPLIFREYIYALAYDHGLNSSILLENLGYENKYSLLIVKRL
ITRMYQQNHLIISANDSNKNPFWGYNKNLYSQIISEGFAVSVEIPFSLQLISSLEEAEII
RSYNLRSIHSIFPFFEEKFPYLNYVSDVRIPYPIHLEILVQTLRYWVKDASFFHLLRLFL
YEYCNWNSLITQKKWISTFSKRNPRFFLFLYNFYVCEYESIFLFLRNKSSYLRLTSSGVL
FERIYFYAKIEHFVEVFDKDFPSTLWFFKDPFIHYVRYQGKSILASKNTPFLMKKWKYYL
IHLWQCHFFVWSQPGKIHINQLSEHSFYFLGYFSNVRLNPSVVRSQMLENSFIIENVMKK
LDTIIPIIPIIRSLAKARFCNVLGHPISKPVWADSSDFDIIDRFLLICKNLSHYYNGSSK
KKNLYRIKYILRLSCIKTLARKHKSTVRVFLKRLGSELLEEFFTEEEEILSLIFPRASST
FQRLYRGRIWYLDIFYFHQ
>Q5YK03 | MATK_VICFA
MKEYKVYLERARSRQQHFLYPLIFREYIYGLAYSHNLNRSIFVENIGYDKKYSLLIVKRL
ITRMYQQNHLIIWANDSNKNPFWGYKNNYYSQIISEGFAIVVEIPFFLQLSSSLEEEEII
KSYKNLRSIHSIFPFLEDKLTYLNYVSDIRIPYPIHLEILVQILRYWVKDAPFFHFLRLF
LWNWNSFITTKKSISTFSKSHRRFFLFLYNFYVCEYESIFVFLRNKSSHLRLKSFSVFFE
RIFFYAKREHLVKVFAKDFSYTLTFFKDPNIHYVRYQGKCILASKNAPFLMNKWKHFFIH
LWQCFFDVWSQPRMININPLSEHSFQLLGYFSNVRLNRSVVRSQMLQNTFLIEIVIKKLD
IIVPIIPLIRSLAKAKFCNVLGQPISKPVWADSSDFDIIDRFLGICRNLSHYYNGSSKKK
SLYRIKYILRLSCIKTLACKHKSTVRAFLKRSGSEEFLQEFFTEEEEILAFLFPRDSSTL
QRLHRNRIWYLDILFSNDLVHDE
>Q5YK05 | MATK_CICAR
MKEYQVYLERARSRQQDFLYPLIFREYIYGLAYSQNFKRSSFVENLGYDSKYSLLIVKRL
ISRMYQQNHLIISANDSNKNPFWGYNKNFNSQIISEGFAIVVEIPFFLQSGSSLKESEII
KSYKNLRSIHSVFPFLEDKFTYLNYVSDIRIPYPIHLEILVQILRYWVKDAPFFHLLRLF
VYNFCNWNSFITTKKSIYTFSKSNPRLFLFLYNFYVWEYESIFLFLRNKSSHLRLKSFSV
FFERIFFYAKREHLVEVFAKDFPYTLKFFKDPLIHYVRYQGKSILASRNAPILMNKWKHY
FLHLWQCFFDVWSQPGTIKINQLSQHSFQLLGYFSNVRLNRSVVRSHMLQNTFLIEIVRK
KLDIIVPIIPLIRSLAKGKFCNVLGHPISKPVWADSSDLDIIDRFLRICRNLSHYYNGSS
KKKSLYQIKYILRLSCIKTLACKHKSTVRAFLKRSGSEELLEEFFTEEEEILSFIFPRDS
STLQRLHRNRINRIWYLDILFSNDLVNHE
>Q5YK53 | MATK_GLETR
MKEFQVYLELDRSLQHDFLYPLIFREYIYALAYDHGLNSSILVENLGYDNKSSLLIVKRL
ITRMYQQNHLILSANDSNKNQFWGYNKNLYSQIISEGFAVSVEIPFSLQLISSLEEAEIV
KSYNLRSIHSIFPFFEEKFPYLNYVSDVRIPYPIHLEILVQTLRYWVKDASSFHLLRLFL
YEYCNWNSLITPKKWISTFSKSNPRLFLFLYNFYVCEYESILIFLRNKSSYLRLTSSGVL
FERIYFYAKIEHRVEVFDKDFPSTLWFFKDPFIHYVRYQGKSILSSRNTPFFMNKWKYYL
IHLWQCHFYVWSQPGKIHINQLSEHSFYFLGYFSNVRLNPSVVRSQMLENSFIIENVMKK
LDTTIPIIPLIRSLAKAKFCNVLGHPISKPVWADSSDFDIIDRFLRICRNLSHYYNGSSN
KKSLYRIKYILRLSCIKTLARKHKSTVRVFLKRLGSKFLEEFFTEEEEILSLILPRASFT
LQRLYRGRIWYLDIFYFHH
>Q5YLB5 | GYRA_NICBE
MKLHTLNPQTPLTQSKPMAFSTGITPSRFSGLRKTSSELRFLSSVTPPPRKQLRPVSARR
KEEEVGDEGNGSVILRDRGENEDRNGGERVVLTELHKEATEAYMSYAMSVLLGRALPDVR
DGLKPVHRRILYAMHELGLSSKKPYKKCARVVGEVLGKFHPHGDTAVYDSLVRMAQDFSL

RSPLIRGHGNFGSIDADPPAAMRYTECRLEALTESMLLADLEQNTVDFVPNFDNSQKEPS
LLPARVPNLLLNGASGIAVGMATNIPPHNLGELVDALSALIHNPEATLQELLEYMPGPDF
PTGGIIMGNIGILEAFRTGRGRVVIRGKTDIELLDSKTKRAAIIIQEIPYQTNKASLVEK
IADLVENKILEGVSDIRDESDRSGMRIVIELKRGSDPAIVLNNLYRLTALQSSFSCNMVG
ILNGQPKLMGLKELLQAFLDFRCSVVERRARFKLSQAQERNHIVEGIIVGLDNLDEVINT
IRKASSNALAAASLRKEFELSEKQAEAILDISLRRLTALERNKFVEEGKSLRTQISKLEE
LLSSKKQILQLIEEEAIEIKNKFFNPRRSMLEDTDSGDLEDIDVIPNEEMLLAISEKGYV
KRMKPDTFNLQNRGTIGKSVGKLRVNDAMSDFLVCRAHDKVLYFSDKGTVYSSPAYKIPE
CSRTAAGTPLVQILSLSDGERITSIIPVSEFAADQYLVMLTVNGYIKKVSLNYFASIRCT
GIIAIQLVPDDELKWVKCCSNNDFVAMASQNGMVILTPCANIRALGRNTRGSVAMRLKEG
DKVASMDIIPDALQKELDKTLEVQQRQYRSMKGPWLLFVSESGYGKRVPVSRFRTSPLNR
VGLFGYKFSSEDCLAAVFVVGFSLGEDGESDEQVVLVSQSGTVNRIKVRDISIQSRYARG
VILMRLEHAGKIQSASLISAADADPEDEDATAVAA
>Q646L1 | MATK_PLETI
MEEYQVYLELDGYQQYDFLYPLLFREYIYALAHDYALNRSRISLENGTYDNKSSSLSVKR
LITRMYQRIPLSISTNDSNQNQFFGHNNNLYSQMISEGFAVIVEIPFSLRLVSFLEGKEI
EKSHNFQSIHSIFPFFEDKFSHLNYVLDVLIPHPIRPEILVQTLRYWVKDAPFLHLLRFF
LHEYFNWNSLITRKKSISIFSKSNPRLFLFLYNSHVCEYESILLFLRNQSSHLRSTSSGI
FLERIYFYRKVEHLVQVLANDFQDILWLFKDPFMHYVRYQGKSILASKDRPLVMNKWKYY
LIHLWQWHFHMWSEPGRVHIKHFYKHSINCLGYLFSVRLNPLVVRSQMLENSFIIDNAMK
KFDTTVPIIPLIGSLTKARFCNTLXHPISKPTRADSSDSHIIDRFVRICRNLSHYHSGSS
KKKSLYRIKYILRLSCVKSLVRKHKNTVRAFLKRLGSEFVEEFLTEEEHVLSLIFPRASS
TSRRLYRGRIWYLDIFCINDLVNHE
>Q67BD8 | MATK_BETPA
MEEFQGYLELDRFRQHDFLYPLIFREYIYALAHDHGLNRVILLENVTYDNKSSLLIVKRL
ITRMYQQNHLMISANDSNQNKFLGYNKNLYSQIISEGFAIIAEIPFSLRLIFSLEGSQIV
RSYNLRSIHSIFPFLEDKFPHLNYVADVLIPYPIHLEILVQTLRYRVKDASSLHLLRFFL
HEYSNGNILIIILNKSISIFSKSNSRLLLFLYNSYICEYESVFLFLRNQSNHLRLTSSGVL
FERIYLHRKMEDLVEVFVNDFQGILCFLKDPFIHYVRYQGKSILASKDTPLLMNKWKYYL
VSLWQCHFFVWSRPGRIYINQLSKHSLDFLGYFSSVPLNPSMVRSQMLENSFIINNAPKK
LDTIVTIIPLIGSLAKAKFCNALGHPISKPTWADLSDFDIINRFVRICKNLSHYYSGSSK
KKGMYRIKYILRLSCVKTLARKHKSTIRAFLKRLGSELFEEFFTEEEEFLSLIFPRTSFT
LRRLYRGRVWYLDIICMNGLANHE
>Q67IA4 | RR7_YUCGL
MSRRGTAEEKTAKSDPIYRNRLVNMLVNRILKHGKKSLAYQILYRAVKKIQQKTETNPLS
VLRQAIRGVTPDIAVKARRVGGSTHQVPIEIGSTQGKALAIRWLLGASRKRPGRNMAFKL
SSELVDAAKGSGDAIRKKEETHRMAEANRAFAHFR
>Q67IB0 | RR7_MUSCM
MSRRGTAEEKTAKSDPIYRNRLVNMLVNRILKHGKKSLAYQIIYRAVKKIQQKTETNPLS
VLRQAIRGVTPDIAVKARRVGGSTHQVPIEIGSTQGKALAIRWLLGASRKRPGRNMAFKL
SSELVDAAKGSGDAIRKKEETHRMAEANRAFAHFR
>Q67IB6 | RR7_MAIRA
MSRRGTAEEKTAKSDPIYRNRLVNMLVNRILKHGKKSLAYQIIYRAVKKIQQKTETNPLS
VLRQAIRGVTPDIAVKARRVGGSTHQVPIEIGSTQGKALAIRWLLGASRKRPGRNMAFKL

SSELVDAAKGSGDAIRKKEETHRMAEANRAFAHFR
>Q67ID1 | RR7_ALLTE
MSRRGTAEEKTAKSDPIYRNRLVNMLVNRILKHGKKSLAYQIIYQAVKKMQQKTETNPLS
VLRQAIRGVTPDIAVKARRVGGSTHQVPIEIGSTQGKALAIRWLLGASRKRPGRNMAFKL
SSELVDAAKGSGDAIRKKEETHRMAEANRAFAHFR
>Q67IE3 | RR7_PHOTN
MSRRGTAEEKTAKSDPIYRNRLVNMLVNRILKHGKKSLAYQIIYRAVKKIQQKTETNPLS
VLRQAIRGVTPDIAVKARRVGGSTHQVPIEIGSTQGKALAIRWLLGASRKRPGRNMAFKL
SSELVDAAKGSGDAIRKKEETHRMAEANRAFAHFR
>Q67II8 | RR7_TYPAN
MSRRGTAEEKTAKSDPIYRNRLVNMLVNRIMKHGKKSLAYQIIYRAVKKIQQKTETNPLS
VLRQAIRGVTPDIAVKARRVGGSTHQVPIEIGSTQGKALAIRWLLGASRKRPGRNMAFKL
SSELVDAAKGSGDAIRKKEETHRMAEANRAFAHFR
>Q68RU3 | RK23_PANGI
MDGIKYAVFTDKSIRLLGKNQYTSNVESGSTRTEIKHWVELFFGVKVIAMNSHRLPGRGR
RMGPIMGQTMHYRRMIITLQPGYSIPPLRKKRT
>Q68RU7 | RR7_PANGI
MSRRGTAEEKTAKSDPIYRNRLVNMLVNRILKHGKKSLAYQIIYRAVKKIQQKTETNPLS
VLRQAIRGVTPDIAVKARRVGGSTHQVPIEIGSTQGKALAIRWLLAASRKRPGRNMAFKL
SSELVDAAKGSGDAIRKKEETHRMAEANRAFAHFR
>Q68RV6 | NU4C_PANGI
MNFFPWLTIIVVLPIFAGSVIFFLPHRGNRVIRWYTICICILELLLTTYAFCYHFQFDDP
LIQLVEDYKWINFFAFHWRLGIDGLSIGPILLTGFITTLATLAARPVTRDSRLFHFLMLA
MYSGQIGSFSSRDLLLFFIMWEFELIPVYLLLSIWGGKRRLYSATNFILYTAGGSIFLLM
GVLGVGLYGSNEPTLNFETSVNQSYPVALEIIFYIGFFIAFAVKSPIIPLHTWLPDTHGE
AHYSTCMLLAGILLKMGAYGLIRINMELLPHAHSIFSPWLVIVGTIQIIYAASTSPGQRN
LKKRIAYSSVSHMGFILIGIGSITDTGLNGAILQIISHGFIGAALFFLAGTSYDRIRLVY
LDEMGGIAIPMPKIFTMFTSFSMASLALPGMSGFVAELIVFFGIITSQKYLLMPKILITF
VMAIGMILTPIYSLSMLRQMFYGYKLFNAPNSYVFDSGPRELFVSISIFLPVIGIGMYPD
FVLSLSVDKVEVILSNFFYR
>Q68RW9 | RK16_PANGI
MLSPKRTRFRKQHRGRMKGISYRGNHISFGKYALQALEPAWITSRQIEAGRRAMTRNARR
GGKIWVRIFPDKPVTVRPAETRMGSGKGSPEYWVAVVKPGRILYEMGGVTENIARKAISI
ASSKMPIRTQFIISA
>Q68RX1 | RR8_PANGI
MGRDTIADIITSIRNADMDKKRVVRIASTNITENVVKILLREGFIENVRKHQENKKDFLV
LTLRHRRNRKRPYRNILNLKRISRPGLRIYSNYQRIPRILGGIGIVILSTSRGIMTDREA
RLERIGGEILCYIW
>Q68RX2 | IF1C_PANGI
MKEQKWIHEGLITESLPNGMFRIRLDNEDMILGYVSGKIRRSFIRILPGDRVKIEVSRYD
STRGRIIYRLRNKNSKD
>Q68RX4 | RR11_PANGI
MAKAIPRSGSRRSGRIGSRKSTRRIPKGVIHVQASFNNTIVTVTDVRGRVVSWSSAGTCG
FKGTRRGTPFAAQTAAGNAIRTVVDQGMQRAEVMIKGPGLGRDAALRAIRRSGILLTFVR

DVTPMPHNGCRPPKKRRV
>Q68RY4 | RR18_PANGI
MDKSKRPFLKSKRSFRRRLPPIQSGDRIDYRNMSLISRFISEQGKILSRRVNRLTLKQQR
LITIAIKQARILSLLPFLNNDKQFERTESTARTAGLRARKK
>Q68S04 | RR4_PANGI
MSRYRGPRFKKIRRLGALPGLTNKRPRAGSDLRNQSRSGKKSQYRIRLEEKQKLRFHYGL
TERQLLKYVRIAGKAKGSTGQVLLQLLEMRLDNILFRLGMAPTIPGARQLVNHRHILVNG
RIVDIASYRCKPRDIITARDEENSRTLIQNSLDSSSHDELPKHLTLHPFQYKGLVNQIID
SKWVGLKINELLVVEYYSRQT
>Q6BDG1 | MATK_KETDA
MDEFHRYGKEDSSWQQCFLYPLFFQEDLYAISHDHYLDGSSSSESMEHFSSNDQFSFLTV
KRLIGQIRQQNHSIVLFLNCDSNPLVDRNKSSYSESVLEGLTLVLEVPFSIRSKYSVEGM
NEWKSFRSIHSIFPFLEEKFPHSNYILDTRIPYSIHPEILVRTFRRWIRDAPSLHPLRSV
LYKYRNSPENFKRSIIVAPRVNTRFLLFLWNHYVYECESILVPLLKQSSHSRSSSHGSFP
ERTHFDRKIKNIIRISRRNSLKSIWSLKDPRIHYVRYGERSIIAIKGTHLLVKKCRYHLL
IFWQCYFHLWSEPYRVCSHQLSKNCSSFLGYFLRVRMKSLLVRTKMLDELFITDLITDEF
YPIVPIVPIIGLLAREKFCDISGRPISKLYWTSLTDDDILDRFDRIWKNLFHYYSGSFGR
DGLYRIKYILSLSCAKTLACKHKSTIRVVRKELGPELFKKSFSKEREFDFPAFSSKAAAR
SQRERIWHSDIPQINPLANSWQKIQDLKSENGKLNLFDQ
>Q6BDG8 | MATK_PINYU
MDEFHRCGKEDSFWQPCFLYPLFFQEDLYAISHDHYLDVSSSSRPMEHLSSNDQLSFLTV
KRLIGQIRQQNHSIVLFVNCDPNPLADRKKSFYSESVLEALTLVLEVPFSIWSKSSVEGM
NECKSFRSIHSIFPFLEDKFPHSNSILDARIPYSIHPEILVRTFRRWIRDAPSLHPLRSV
LYDYRNSPENLQRSIIVVPRVNTRFFLFLLNYYVWECESILFSRLKRSSHSRSLAHGSFP
QRTHFHRKIKHIIIFSRRNSLKSIWSLKDPKIHYVRYGERPIIAIKGADLLVKKCRYYLL
IFRQFYFHLWSEPYRVCSHQLSKNCSSSPGYFLRVRMNPLLVRTKTLDELFIPVLITNEM
DPIVPIVPIIGLLATEKFCDISGRPISKLSWTSLTDDDILDRFDQIWRNLFHYYSGSFDR
DGLYRIKYILLLSCAKTLACKHKSTIRVVRKELGPELFKKSFSKEREFDSLPFSSKAAAR
SQRERIWHSDIPQINPLANSWQKIQDLKIENLFDQ
>Q6BDH3 | MATK_PINPU
MDEFHRYGKEDNSRQQCFLYPLFFQEDLYAISHDHYLDGSSSSEPMEHLSSNDQFSFLTV
KRLIGQIRQQNHSIVLFVNCAPNPLADCKKSSYSESVLEGLTLVLEVPFSIRSKYSVEGM
NEWKSFRSIHSIFPFLEDKFPHSNYISDARIPYSIHPEILVRTFRRLIRDAPSLHPLRSV
LYEYRNSPENLQRSIIVVPRVNTRFFLFLWNYYVYECESILFSLLKRSSHSRSVAHRPFP
QRTHFHRKIKHIIIFSRRNSLKSIWLLKDPKINYVRYGERSIIAIKGTHLLVKKCRYYLL
LFRQCYFHLWSEPYRVCSHQLSKNCSSSPGYFLRVRMNPLFVRTKMLDELFIADLITNEF
DPIVPIVPILGLLAREKFCDVSGRPISKLSWTNLTDDDILNRFDQIWRNLFHYYSGSFGR
DGLYRIKYILSLSCAKTLACKHKSTIRVVRKELGPELFQKSFSKEREFDSLPFSSKAAAR
SQRERIWHSDIPQINPLVNSWQKIQDLKIENLFDQ
>Q6BDJ4 | MATK_PINCE
MDEFHRYGKEDNSRQQCFLYPLFFQEDLYAISHDHYLDGSSSSEPMEHLSSNDQFSFLTV
KRLIGQIRQQNHSIVLFVNCAPNPLADCKKSSYSESVLEGLTLVLEVPFSIRSKYSVEGM
NEWKSFRSIHSIFPFLEDKFPHSNYISDARIPYSIHPEILVRTFRRLIRDAPSLHPLRSV
LYEYRNSPENLQRSIIVVPRVNTRFFLFLWNYYVYECESILFSLLKRSSHSRSLAHRPFP

HGTHFHRKIKHIIIFSRRNSLKSIWLLKDPKINYVRYGERSIIAIKGTHLLVKKCRYYLL
LFRQCYFHLWSEPYRVCSHQLSKNCSSSLGYFLRVRMNPLFVRTKMLDELFIADLITNEF
DPIVPIVPILGLLAREKFCDVSGRPISKLSWTNLTDDDILNRFDQIWRNLFHYYSGSFGR
DGLYRIKYILSLSCAKTLACKHKSTIRVVRKELGPELLSKIVFKRTRFDSLPFSSKAAAR
SQRERIWHSDIPQINPLVNSWQKIQDLKIENLFDQ
>Q6EIH3 | MATK_TILAM
MEEFQVYLELNRSRRHDFLYPLIFREYIYALAHDHGLNKSMIFLENQGYGNKFSSLIVKR
LIIRMDQQNHLIISANDXNQNPFFGHNNNLYSQMISAGFAVIVEIPFSLRLVSYSQGEEV
AKSHNLQSIHSIFPFLEDKFSHLNYVLDVLIPHPIHLEILVQALRYWVKDASSLHLLRFS
LYEYCNLKSFITPKKSISIFNPRLFLFLYNSHVCEYESIFLFLRNQSSHLRSTSSGVFLE
RIYFYGKIEYLVEVFYNDFQNNLWLFKDPFIHFIRYQGKAILASKDTSLLMNKWKYYFVD
LWQYYFYMWSQSGRVRINQLSKYSLDFLGYLSSVRLNPSVVRSQMLENSFIIDNAMKKLD
TRIPIISLIGSLSKAKFCNTLGHPISKPTWADSSDSDIIDRFVRICRNLSHYHSGSSKKK
SLYRIKYILRLSCVKTLARKHKSTVRAFLKRLGSEFLEEFFTEEEHVFSLIFPKVFFTSR
KLYRGRIWYLDIICINALVNHE
>Q6EIJ4 | MATK_PACAQ
MEEFQVYLELNRSRRHDFLYPLIFREYIYALAHDHGLNKSMIFLENQGYGNKFSSLIVKR
LIIRMDQQNHLIISANDSNQNPFFGHKKNLYSQMISAGFAVIVEIPFSLRLVSYSQGEEV
AKSHNLQSIHSIFPFLEDKFSHLNYVLDVLIPYPIHLEILVQALRYWVKDASSLHLLRFS
LYEYCNLKSFITPKKSISIFNPRLFFFLYNSHACEYESIFLFLRNQSSHLRSTSSGVFLE
RIYFYGKIDYLVEVFSNDFQNNLWLFKDPFTHFIRYQGKAILASKDTSLLMNKWKYYFVD
LWQYYFYMWSQSGRFRINQLSKYSLDFLGYLSSVRLNPSVVRSQMLENSFIIDNAMKKLD
TRIPIISLIGSLSKAKFCNTLGHPISKPTWADSSDSDIIDRFVRICRNLSHYHSGSSKKK
SLYRIKYILRFSCVKTLARKHKSTVRAFLKRLGSEFLEEFFTETEEEHFFSLIFPRVFFT
SRKLYRGRIWYLDIICINALVNHE
>Q6EIJ6 | MATK_ADADI
MEEFQVYLELNRSRRHDFLYPLIFREYIYALAHDHGLNKSMIFLENQGYVNKFSSLIVKR
LIIRMDQQNHLIISANDSNQNPFFGHNNNLYSQMISAGFAVIVEIPFSLRLVSYSQGEEV
AKSHNLQSIHSIFPFLEDKFSHLNYVLDVLIPHPIHLEILVQALRYWVKDASSLHLLRFS
LYEYCNLKSFITPKKSISIFNPRLFFFLYNSHACEYESIFLFLRNQSSHLRSTSSGVFLE
RIYFYGKIEYLLEVFYNDFQNNLWLFKDPFIHFIRYQGKAILASKDTSLLMNKWKYYFVD
LWQYHFYMWSQSGRVRINQLSKYSLDFLGYLSSVRLNPSVVRSQMLENSFIIDNAMKKLD
TRIPIISLIGSLSKAKFCNTLGHPISKPTWADSSDSDIIDRFVRICRNLSHYHSGSSKKK
SLYRIKYILRFSCVKTLARKHKNTVRAFLKRLGSEFLEEFFTETEEEHVFSLIFPRVFFT
SRKLYRGRIWYLDIICINALVNHE
>Q6EM84 | RR7_NELLU
MSRRGTAEEKTAKSDPIYRNRLVNMLVNRIMKHGKKSLAYQIIYRAVKKIQQKTETNPLS
VLRQAIRGVTPDIAVKARRVGGSTHQVPIEIGSTQGKALAIRWLLGASRKRPGRNMAFKL
SSELVDAAKGSGDAIRKKEETHRMAEANRAFAHFR
>Q6EMB0 | RR7_ARIMA
MSRRGTAEEKTAKSDPIYRNRLVNMLVNRILKHGKKSLAYQIIYRAVKKIQQKTETNPLS
VLRQAIRGVTPDIAVKARRVGGSTHQVPIEIGSTQGKALAIRWLLGASRKRPGRNMAFKL
SSELVDAAKGSGDAIRKKEETHKMAXANRAFAHFR
>Q6ENA1 | NDHH_ORYNI

MSLPLTRKDLMIVNMGPQHPSMHGVLRLIVTLDGEDVIDCEPILGYLHRGMEKIAENRTI
IQYLPYVTRWDYLATMFTEAITVNAPEFLENIQIPQRASYIRVIMLELSRIASHLLWLGP
FMADLGAQTPFFYIFRERELIYDLFEAATGMRMMHNYFRIGGVAADLPYGWIDKCLDFCD
YFLRGVIEYQQLITQNPIFLERVEGVGFISGEEAVNWGLSGPMLRASGIQWDLRKVDLYE
SYNQFDWKVQWQKEGDSLARYLVRIGEMRESIKIIQQAVEKIPGGPYENLEVRRFKKAKN
SEWNDFEYRFLGKKPSPNFELSKQELYARVEAPKGELGIYLVGDDSLFPWRWKIRPPGFI
NLQILPQLVKKMKLADIMTILGSIDIIMGEVDR
>Q6ENA4 | NU6C_ORYNI
MDLPGPIHEILVLFGGFVLLLGGLGVVLLTNPTFSAFSLGLVLVCISLFYILLNSYFVAV
AQLLIYVGAINVLIIFAVMFVNGSEWSKDKNFWTIGDGFTSLVCITIPFSLMTTIPDTSW
YGILWTTRSNQIVEQGLINNVQQIGIHLATDFYLPFELISIILLVSLIGAITMARQ
>Q6ENA5 | NU4LC_ORYNI
MMFEHVLFLSVYLFSIGIYGLITSRNMVRALICLELILNSINLNLVTFSDLFDSRQLKGD
IFAIFVIALAAAEAAIGLSILSSIHRNRKSTRINQSNFLNN
>Q6ENA7 | NU4C_ORYNI
MSSFPWLTILVVLPIFAGSLIFFLPHRGNKIVRWYTMSICLLEFLLMTYAFCYHFQLEDP
LIQLKEDSKWIDVFNFHWRLGIDGLSLGSILLTGFMTTLATLAAWPVTRNSRLFYFLMLA
MYSGQIGLFSSRDLLLFFIMWELELIPVYLLLSMWGGKRRLYSATKFILYTAGGSIFFLI
GVLGMGLYGSNEPRLDLERLINQSYPATLEILFYFGFLIAYAVKLPIIPLHTWLPDTHGE
AHYSTCMLLAGILLKMGAYGLIRINMELLPHAHYLFSPWLVIIGAMQIIYAASTSLGQRN
FKKRIAYSSVSHMGFIIIGIGSITNIGLNGAILQILSHGFIGATLFFLAGTACDRMRLVY
LEELGGVSIPMPKIFTMFSSFSMASLALPGMSGFVAELVVFFGLITSPKFLLMPKMLITF
VMAIGMILTPIYLLSMLRQMFYGYKLFHVPNENFEDSGPRELFLLICIFLPVIGIGIYPD
FVLSLSVDRVEALLSNYYPK
>Q6ENA9 | RK32_ORYNI
MAVPKKRTSMSKKRIRKNLWKKKTYFSIVQSYSLAKSRSFSGVSEHPKPKGFSRQQTNK
>Q6ENB2 | RR15_ORYNI
MKKKGGRKIFGFMVKEEKEENWGSVEFQVFSFTNKIRRLASHLELHKKDFSSERGLRRLL
GKRQRLLAYLAKKNRVRYKKLISQLDIRER
>Q6ENC9 | YCF72_ORYNI
MGAFPSPPPWGWSTGFITTPLTTGRLPSQHLDPALPKLFWFTPTLPTCPTVAKQFWDTKR
TSPDGNLKVADLPSFAISFATAPAALANCPPLPRVISMLCMAVPKGISVEVDSSFLSKNP
FPNCTSFFQSIRLSRCI
>Q6END5 | RR3_ORYNI
MGQKINPLGFRLGTTQNHHSFWFAQPKNYSEGLQEDKKIRNCIKNYIQKNRKKGSNRKIE
ADSSFEVITHNKKMDSGSSSEVITHIEIQKEIDTIHVIIHIGFPNLLKKKGAIEELEKDL
QKEVNSVNQRLNIGIEKVKEPYRQPNILAEYIAFQLKNRVSFRKAMKKAIELTKKTDIKG
VKVKIAGRLAGKEIARAECIKKGRLPLQTIRAKIDYCCYPIRTIYGVLGVKIWIFVDEE
>Q6END9 | IF1C_ORYNI
MTEKKNRREKKNPREAKITFEGLVMEALPNGMFRVRLENDTIILGYISGKIRSSSIRILM
GDRVKIEVSRYDSSKGRIIYRLPHKDSKRTEDSKDTEDLKDTKDSKG
>Q6ENE2 | RPOA_ORYNI
MVREEVAGSTQTLQWKCVESRVDSKRLYYGRFILSPLRKGQADTVGIALRRALLGETEGT
CITHAKFGSVPHEYSTIAGIEESVQEILLNLKEIVLRSNLYGVRTASICVKGPRYITAQD

IILPPSVEIVDTAQPIANLTEPTDFRIELRIKRDRGYHTEVRKNTQDGSYPIDAVSMPVR
NVNYSIFACGNGNAKYEILFLEIWTNGSLTPKEALYEASRNLIDLFLPFLHTEEEGTRFQ
ENKNRFTSPLLSFQKRLTNLKKNKKRIPLNCIFIDQLELPSRTYNCLKRANIHTLLDLLS
KTEEDLMRIDSFRMQDGKQIWDTLEKHLPMDLPKNKF
>Q6ENG9 | NU3C_ORYNI
MFLLHEYDIFWAFLIIASLIPILAFWISALLAPVREGPEKLSSYESGIEPMGGAWLQFRI
RYYMFALVFVVFDVETVFLYPWAMSFDVLGISVFIEAFIFVLILVVGLVYAWRKGALEWS

>Q6ENH0 | NDHK_ORYNI
MVLTEYSDKKKKEGKDSIKTVMSLIEFPLLDQTSSNSVISTTLKDLSNWSRLSSLWPLLY
GTSCCFIEFASLIGSRFDFDRYGLVPRSSPRQADLILTAGTVTMKMAPSLVRLYEQMPEP
KYVIAMGACTITGGMFSTDSYSTVRGVDKLIPVDVYLPGCPPKPEAVIDALTKLRKKISR
EIVEDRTLSQKKNRCFTTSHKLYVRRSTNTGTYEQELLYQSPSTLDISSETFFKSKSPVS
SYKLVN
>Q6ENH1 | NDHJ_ORYNI
MQQGWLSNWLVKHEVVHRSLGFDHRGIETLQIKAEDWDSIAVILYVYGYNYLRSQCAYDV
APGGSLASVYHLTRIQYGIDNPEEVCIKVFAQKDNPRIPSVFWIWRSSDFQERESFDMVG
ISYDNHPRLKRILMPESWIGWPLRKDYITPNFYEIQDAH
>Q6ENH2 | RR4_ORYNI
MSRYRGPRFKKIRRLGALPGLTRKTPKSGSNLKKKFHSGKKEQYRIRLQEKQKLRFHYGL
TERQLLRYVHIAGKAKSSTGQVLLQLLEMRLDNILFRLGMASTIPEARQLVNHRHILVNG
RIVDIPSFRCKPRDIITTKDNQRSKRLVQNSIASSDPGKLPKHLTIDTLQYKGLVKKILD
RKWVGLKINELLVVEYYSRQT
>Q6ENH6 | RR14_ORYNI
MAKKSLIQRERKRQKLEQKYHLIRRSSKKKIRSKVYPLSLSEKTKMREKLQSLPRNSAPT
RLHRRCFLTGRPRANYRDFGLSGHILREMVYACLLPGATRSSW
>Q6ENI1 | RR2_ORYNI
MTRRYWNINLKEMIEAGVHFGHGIKKWNPKMAPYISAKRKGTHITNLARTTRFLSEACDL
VFDAASQGKSFLIVGTKKRAADLVASAAIRARCHYVNKKWFSGMLTNWSITKTRLSQFRD
LRAEEKMEKFHHLPKRDVAILKRKLSTLLRYLGGIKYMTRLPDIVIVLDQQKEYIALREC
AILGIPTISLADTNCDPDLANISIPANDDTMTSIRLILNKLVFAICEGRSLYIRNH
>Q6ENJ5 | RR16_ORYNI
MLKLRLKRCGRKQRAVYRIVAIDVRSRREGRDLRKVGFYDPIKNQTCLNVPAILYFLEKG
AQPTRTVSDILRKAEFFKEKERTLS
>Q6ENJ6 | MATK_ORYNI
MEKFEGYSEKLKFPRQYFVYPLLFQEYIYVFAHDYGLNGSELVEIIGSNNKKFSSLLVKR
LMIRMYQQNFWINLVNHPNQDRLLDYNNFFYSEFYSQILSEGFAIVVEIPFSLREQSCPE
EKEIPKFQNLRSIHSIFPFLEDKFLHLHYLAHIEIPYPIHLDILLQLLQYRIQDVPSLHL
LRFFLNYYSNWNSFITSMKSIFILKKENKRLFRFLYNSYVSEYEFFLLFLRKQSSCLRLT
SSGTFLERIIFSRKMEHFGLMYPAFFRKTIWFVMDPLMHYVRYQGKAILASKGTLLLKKK
WKCYLVRLWQYSFSFWTQPQRIHLNQLENSCFDFLGYFSSVPINSLLVRNQMLENSFLID
TQMKKFDTKVPVTPLIGSLAKAQFCTGSGHPISKPIWTDLSDWDILDRFGRICRNLFHYH
SGSSKKKTLYRLKYILRLSCARTLARKHKSTVRAFMQWLGSVFLEEFFTEEEQVFSLMFA
KTTYFSFRGSHSERIWYLDILRINDLVNPLN

>Q6ENP4 | NU6C_SACOF
MDLPGPIHEILVLFGGFVLLLGGLGVVLLTNPIYSAFSLGLVLVCISLFYFLLNSYFVAV
AQLLIYVGAINVLIIFAVMFVNGSEWSKDKNYWTIGDGFTLLLCITIPFSLMTTIPDTSW
YGILWTTRSNQIVEQGLINNVQQIGIHLATDFYLPFELISLILLVSLIGAITMARQ
>Q6ENP5 | NU4LC_SACOF
MMFEHVLFLSVYLFSIGIYGLITSRNMVRALICLELILNSINLNLVTFSDLFDSRQLKGD
IFAIFVIALAAAEAAIGLSILSSIHRNRKSTRINQSNFLNN
>Q6ENQ1 | RR15_SACOF
MVKEEKQENRGSVEFQVFSFTNKIRRLASHLELHKKDFSSERGLRRLLGKRQRLLAYLAK
KNRVRYKKLISQLDIREK
>Q6ENQ5 | YCF68_SACOF
MAYSSCLNRSLKPNKLLLRRIDGAIQVRSHVDRTFYSLVGSGRSGGGPPRLLSSRESIHP
LSVYGELSLEHRLRFVLNGKMEHLTTHLHRPRTTRSPLSFWGDGGIVPFEPFFHAFPGGL
EKAVINRTSLILPS
>Q6ENR2 | YCF73_SACOF
MTKDETLLVFTLVVSSVSIFLFGILLFMVLISATRDFRERTKSKLVKIMIWAGIVVITFA
IAVRIYPIFIFLLKERIKPLVEALYDKLPWIWEVSLSRYWDRLIDFLDRYLWACAQRIQT
GIRKQKGEFVVTFSCRVKKRLYARAIEVGIHLSLLSNLFWILKTTLAVGYRLL
>Q6ENR3 | YCF15_SACOF
MLIVLFRSKDIRGGRFVRPILIFRTKRSWILFRIGPERRREAEMPTDLCLFSNSPDPIVP
VFGTSSAKVTEWVSHQSNPFDKSGVILDIIFYIYRNIIE
>Q6ENS0 | YCF72_SACOF
MGAFPSPPPWGWSTGFITTPLTTGRLPSQHLDPALPKLFWFTPTLPTCPTVAKQFWDTKR
TSPDGNLKVANLPSFAISFATAPAALANCPPLPRVISMLCMAVPKGISVEVDSSFLSKNP
FPNCTSFFQSIRLSRCI
>Q6ENS1 | RK2_SACOF
MAKHLYKTPIPSTRKGTVDRQVKSNPRNKLIHGRHRCGKGRNARGIITARHRGGGHKRLY
RKIDFRRNQKDISGRIVTIEYDPNRNAYICLIHYGDGEKRYILHPRGAIIGDTIVSGTKV
PISMGNALPLTDMPLGTAIHNIEITRGRGGQLARAAGAVAKLIAKEGKLATLRLPSGEVR
LVSQNCLATVGQVGNVGVNQKSLGRAGSKCWLGKRPVVRGVVMNPVDHPHGGGEGKAPIG
RKKPTTPWGYPALGRRTRKRKKYSDSFILRRRK
>Q6ENS3 | RR19_SACOF
MTRKKTNPFVARHLLAKIEKVNMKEEKEIIVTWSRASSILPAMVGHTIAIHNGKEHIPIY
ITNPMVGRKLGEFVPTRHFTSYESTRKDTKSRR
>Q6ENS5 | RR3_SACOF
MGQKINPLGFRLGTTQNHHSFWFAQPKNYSEGLQEDKKIRNCIKNYIQKNRKKGSNRKME
SDSSSEVITHIEIQKEIDTIHVIIHIGFPNLLKKKGAIEELEKDLQKEVNSVNQRLNIAI
EKVKEPYRQPNILAEYIAFQLKNRVSFRKAMKKAIELTKKADIKGIKIQIAGRLAGKEIA
RAECIKKGRLPLQTIRAKIDYCCYPIRTIYGVLGVKIWIFVEEE
>Q6ENS7 | RK14_SACOF
MIQPQTLLNVADNSGARKLMCIRVIGAAGNQRYARIGDVIIAVIKDAVPQMPLERSEVIR
AVIVRTRKEFKGDDGIIIRYDDNAAVIIDQKGNPKGTRVFGAVAEELRELNYTKIVSLAP
EVL
>Q6ENU0 | RR12_SACOF

MPTVKQLIRNARQPIRNARKSAALKGCPQRRGTCARVYTINPKKPNSALRKVARVRLTSG
FEITAYIPGIGHNLQEHSVVLVRGGRVKDLPGVRYRIIRGTLDAVAVKNRQQGRSKYGAK
KPKK
>Q6ENU3 | RK33_SACOF
MAKGKDVRIRVILECISCVRKGTNKESTGISRYSTQKNRHNTPGQLELRKFCRYCRKHTT
HNEIKK
>Q6ENW5 | RR14_SACOF
MAKKSLIQREKKRHKLEQKYHLIRRSSKKKIRSKVSPLSLSEKTKMQEKLQSLPRNSAPT
RLHRRCFLTGRPRANYRDFGLSGHILREMVYACLLPGATRSSW
>Q6ENX2 | RPOC1_SACOF
MIDQYKHKQLQIGLVSPQQIKAWAKKILPNGEVVGEVTRPSTFHYKTDKPEKDGLFCERI
FGPIKSGICACGNSRASVAENEDERFCQKCGVEFVDSRIRRYQMGYIKLACPVTHVWYLK
GLPSYIANLLDKPLKKLEGLVYGDFSFARPSAKKPTFLRLRGLFEDEISSCNHSISPFFS
TPGFATFRNREIATGAGAIREQLADLDLRIIIENSLVEWKELEDEGYSGDEWEDRKRRIR
KVFLIRRMQLAKHFIQTNVEPEWMVLCLLPVLPPELRPIVYRSGDKVVTSDINELYKRVI
RRNNNLAYLLKRSELAPADLVMCQEKLVQEAVDTLLDSGSRGQPMRDGHNKVYKSLSDVI
EGKEGRFRETLLGKRVDYSGRSVIVVGPSLSLHQCGLPLEIAIKLFQLFVIRDLITKRAT
SNVRIAKRKIWEKEPIVWEILQEVMRGHPVLLNRAPTLHRLGIQAFQPTLVEGRTICLHP
LVCKGFNADFDGDQMAVHLPLSLEAQAEARLLMFSHMNLLSPAIGDPICVPTQDMLIGLY
VLTIGNRRGICANRYNSCGNSPNKKINYNNNNYYKYTKDKEPHFSSSYDALGAYRQKRIG
LNSPLWLRWKLDQRIVGSREVPIEVQYESFGTYHEIYAHYLVVGNRKKEIRSIYIRTTLG
HISFYREIEEAVQGFSRAYSYTI
>Q6ENX3 | RPOB_SACOF
MLRNGNEGMSTIPGFSQIQFEGFCRFINQGLAEELEKFPTIKDPDHEIAFQLFAKGYQLL
EPSIKERNAVYESLTYSSELYVSARLIFGFDVQKQTISIGNIPIMNSLGTFIINGIYRIV
INQILLSPGIYYRSELDHKGISICTGTIISDWGGRSELAIDKKERIWARVSRKQKISILV
LSSAMGSNLREILDNVSYPEIFLSFPNAKEKKRIESKEKAILEFYQQFACVGGDLVFSES
LCEELQKKFFQQKCELGRVGRRNMNRRLNLDIPQNNTFLLPRDVLAATDHLIGMKFGTGI
LDDDDMNHLKNKRIRSVADLLQDQFGLALGRLQHAVQKTIRRVFIRQSKPTPQTLVTPTS
TSILLITTYETFFGTYPLAQVFDQTNPLTQTVHGRKVSCLGPGGLTGRTASFRSRDIHPS
HYGRICPIDTSEGINVGLTGSLAIHARIDHWWGSIESPFYEISEKAKEKKERQVVYLSPN
RDEYYMIAAGNSLSLNQGIQEEQVVPARYRQEFLTIAWEQIHVRSIFPFQYFSIGGSLIP
FIEHNDANRALMSSNMQRQAVPLSRSEKCIVGTGLERQTALDSRVSVIAEREGKIISSDS
HKILLSSSGKTISIPLVAHRRSNKNTCMHQKPRVPRGKSIKKGQILAEGAATVGGELALG
KNVLVAYMPWEGYNFEDAVLISERLVYEDIYTSFHIRKYEIQTDTTSQGSAEKITKQIPH
LEEHLLRNLDRNGVVRLGSWVETGDILVGKLTPQIASESSYIAEAGLLRAIFGLEVSTSK
ETSLKLPIGGRGRVIDVKWIQRDPFDIMVRVYILQKREIKVGDKVAGRHGNKGIISKILP
RQDMPYLQDGTPVDMVFNPLGVPSRMNVGQIFESSLGLAGDLLKKHYRIAPFDERYEQEA
SRKLVFSELYEASKQTKNPWVFEPEYPGKSRIFDGRTGDPFEQPVLIGKSYILKLIHQVD
EKIHGRSTGPYSLVTQQPVRGRAKQGGQRIGEMEVWALEGFGVAHILQEILTYKSDHLIA
RQEILNATIWGKRVPNHEDPPESFRVLVRELRSLALELNHFLVSEKNFRVNREDV
>Q6ENX8 | MATK_SACOF
MQKFEGYSEKQKSRQHYFVYPLLFQEYIYAFAHDYGLNGSEPVEIFGCNNKKFSSILVKR
LIIRMYQQNFLINSVNYPNQDRLFDHRNYFYSEFYSQILSEGFGIVVEIPLSLGQLSCPE

EKEIPKFQNLQSIHSIFPFLEDKFLHLHYLSHIEIPYPIHLEILVQLLEYRIQDVPSLHL
LRFFLHYYSNWNSLITSMKSIFLLKKENQRLFRFLYNSYVSEYEFFLLFLRKQSSCLRLT
SSGTFLERIIFSGKMEHFGVMYPGFFRKTIWFFMDPLMHYVRYQGKAILASKGTLLLKKK
WKSYLVNFSQYFFYFWTQPQRIRLNQLTNSCFDFLGYLSSVPINTLLVRNQMLENSFLID
TRMKKFNTTVPATPLVGSLSKAQFCTGSGHPISKPVWTDLSDWDILDRFGRICRNLFHYH
SGSSKKQTLYRLKYILRLSCARTLARKHKSTVRTFMQRLGSVFLEEFFTEEEQVFSLMFT
KTIHFSFHGSQSERIWYLDIIRINDLVNPLTLN
>Q6ENY4 | RR16_SACOF
MVKLRLKRCGRKQQAIYRIVAIDVRSRREGRDLRKVGFYDPIKNQTCLNVPAILYFLEKG
AQPTRTVYDILRKAEFFKDKERTLS
>Q6ENY5 | PSBK_SACOF
MPNILSLTCICFNSVLCPTSFFFAKLPEAYAIFNPIVDVMPVIPVLFFLLAFVWQAAVSF
R
>Q6EVY6 | RK23_NYMAL
MDGIKYAVFTEKSIRLLGNNQYTSNVESGSTRTEIKHWIELFFGVKVVAMNSHRLPGKGR
RMGPIMRHTMHYRRMIITLQPGYSIPPLIEKRT
>Q6EVZ0 | RR7_NYMAL
MSRRGTAEEKTAKSDPIYRNRLVNMLVNRILKHGKKSLAYQIIYRAVKKIQQKTETNPLS
VLRQAIRGVTPNIAVKARRVGGSTHQVPIEIGSTQGKALAIRWLLGASRKRPGRNMAFKL
SSELVDAARGSGDAIRKKEETHRMAEANRAFAHFR
>Q6EW11 | RR19_NYMAL
MARSSKKNPFVANHLLSKIEKLNKAEERSIIVTWSRASTIIFAMVGHTIAVHNGKEHLPI
YITERMVGHKLGEFAPTLTFRGHARNDNRSRR
>Q6EW12 | RK22_NYMAL
MIKKTSGTEIRALARHIGMSAQKARRVIDQIRGCSYEQTLMILELMPYRACYPIFKLVYS
AAANASHNRGLKEADLFISKAEVNEGVIVKRLKPRARGRSYPIKRPTCHITIVLSERPNF
NFKNI
>Q6EW13 | RR3_NYMAL
MGQKINPLGFRLGTTQSHRSFWFAQPKNYSKGLQEDEKIRDCIKNYVQKHMRISSGFEGI
ARIDIKKRIDLIQVIIHIGFANMLMEGRARGIEELQTNVQKSFHSVNRRLNIAIARVARP
YGQPNILAEYIALQLKNRVSFRKAMKKAIELAEQADAKGIQVQIAGRLNGNEIARVEWIR
EGRVPLQTIRVKIDHCSYPVRTIYGVLGIKIWIFLDEE
>Q6EW15 | RK14_NYMAL
MIQPQTLLNVADNSGARKLMCIRIIGASNYRYAHIGDVIVAVIKEAVPNMPLERSEVIRA
VIVRTCKELKRDNGMIIRYDDNAAVVIDQEGNPKGTRVFGSIARELRQLNFTKIVSLAPE
VL
>Q6EW19 | RR11_NYMAL
MTKPIPKIGSRRNSRISSRKSGCRTPIGVIHVQASFNNTIVTVTDPQGRVVSWSSAGTCG
FKGTRRGTPFAAQTAAGNAIRAVVDQGMQRAEVMIKGPGLGRDAALRAIRRSGILLNFVR
DVTPMPHNGCRPPKKRRV
>Q6EW28 | RR12_NYMAL
MPTIKQLIRNTRQPMRNVTKSPALRGCPQRRGTCTRVYTITPKKPNSALRKVARVRLTSG
FEITAYIPGIGHNLQEHSVVLVRGGRVKDLPGVRYHIVRGTLDAVGVKDRQQGRSKYGVK
KPK

>Q6EW30 | RK20_NYMAL
MTRVRRGYIARRRRRKIRLFASSFRGAHSRLTRTATQQKIRALVSSHRGRGRQKRDFRRL
WITRINAVTRENGVPYSRLIHDLRKKQLLLNRKILAQIAISNRNCLYMISNDIIK
>Q6EW50 | RR14_NYMAL
MARKSLIQREKKRQKLEEKYHLIRRSSKKEISKVSSLDEKWEIHVKLQSPPRNSAPIRLH
RRCFLTGRPRANYRDFGLSGHVLREMVHACLLPGATRSSW
>Q6EW56 | RPOB_NYMAL
MLRDGGDEGMFTIPGFSQIQFEGFCRFIDQGLMEELHQFPKIEDTDQEIEFQLFGESYQL
VEPLIKERDAVYESITYSSELYVPAGLIWRTGRNMQEQTVLLGNIPLMNSLGTSIVNGIY
RIVINQILQSPGIYYSTGLDHNGISVYTGTIISDWGGRSELEIDRKERIWARVSRKQKIS
ILVLSSAMGSSLREILDNVCYPEIFLSFPNEKEKKKIGSKENAILEFYQKFACVGGDPVF
SESLCKELQKKFFQQRCELGRIGRRNMNQRLNLDIPPNNTFLLPRDVLAAADHLIGMKFG
MGTLDDMNHLKNKRIRSVADLLQDQFGLALVRLENVVRGTICGAIRHKLIPTPRNLVTST
PLTTTYESFFGLHPLSQVLDRTNPLTQIVHGRKSSYLGPGGLTGRTASFRIRDIHPSHYG
RICPIDTSEGINVGLIGSLAIHARVGDWGSIETPFYEISERSKEEQMVYLSPSRDEYYMV
AAGNSLALTRGIQEEEVGPARYRQEFLTIAWEQIHLRNIYPFQYFSIGASLIPFIEHNDA
NRALMSSNMQRQAVPLSQSEKCIVGTGLERQAALDSGGSAIAEREGKIIYTDAEKIVLSG
NGDTISIPLVMYQRSNKNTWMHQKPQVHRGKYLKKGQILADGAATVGGELALGKNVSVAY
MPWEGYNSEDAVLISERLVYDDIYTSFHIRKYEIQTHVTSQGPERITNEIPHLEPYLLRN
LDRNGIVMLGSWVETGDVLVGKLTPQTAKESSYAPEDRLLRAILGIQVSTAKETCLKLPI
GGRGRVIDVRWGQKKGGSIYNPEMIRVYISQKRKIKVGDKVAGRHGNKGIISKILPRQDM
PYLQDGTPVDMVFNPLGVPSRMNVGQMFECSLGLAGDLLGRHYRITPFDERYEQEASRKL
VFSELYEASKQTANPWVFEPEYPGKSRIFDGRTGDPFEQPVIIGKSYMLKLIHQVDDKIH
GRSSGHYALVTQQPLRGRAKQGGQRVGEMEVWALEGFGVAHILQEMLTYKSDHIRARQEV
LGTTIVGGTIPNPEGAPESFRLLVRELRSLSLELNHFLVSEKNFQINRKEV
>Q6EW66 | RR16_NYMAL
MVKLRLKRCGRRQRATYRIIAIDVRSRREGRDLRKVGFYDPIKNQTYLNVPAILYFLEKG
AQPTGTVHDISKKAEVFKELRVNQTK
>Q6EW67 | MATK_NYMAL
MEKLQYELQGYLEIDRYRKQRFLYPLLFREYIYALAHDHGLNSSIFYEPTENLGYDNDNK
SSSLIVKRLITRLHQQNHLTISVNDSRFVGPNRSFYSQTIPEGFAGIMEIPFSVRLVSSL
ERERIAKYHNLRSIHSIFPFLEDKLSHLYYVSDILIPYPIHLEILLQTLRTRIRDAPSLH
LLRCFLHEHHNWNSLITSNKSISIFSKENQRLFLFLYNSHVYECESVLVFLRKQSSHLRS
ISSLAFLERTHFYGKIKHLVVTPRNDSQRTLPLWFFKEPLMHYVRYQGKSIMASRCTNLL
MKKWKYYLVNFWQCHFHLWSQPGRIHINELSNHSFYFLGYLSGVRLTPWVIRSQMLENSF
MIDTAIKRFDTIVPIFPLIGSLVKAKFCNVSGYPISKSVWADSSDSDIIARFGWICRNLS
HYHSGSSKKHSLCRIKYILRLSCARTLARKHKSTVRAICKRLGSKLLEEFLTEEHEIVSF
IFRRTRLRSERIWYLDIIRIHGLVPHS
>Q6EW71 | RBL_NYMAL
MSPKTETKASVGFKAGVKDYRLTYYTPEYETLATDILAAFRVTPQPGVPPEEAGAAVAAE
SSTGTWTTVWTDGLTSLDRYKGRCYHIEPVAGEENQYIAYVAYPLDLFEEGSVTNMFTSI
VGNVFGFKALRALRLEDLRIPPAYSKTFQGPPHGIQVERDKLNKYGRPLLGCTIKPKLGL
SAKNYGRAVYECLRGGLDFTKDDENVNSQPFMRWRDRFLFCAEAIYKAQAETGEIKGHYL
NATAGTSEEMMKRAVCARELGVPIVMHDYLTGGFTANTSLAHYCRDNGLLLHIHRAMHAV

IDRQRNHGIHFRVLAKALRMSGGDHIHAGTVVGKLEGERDVTLGFVDLLRDDFIEKDRSR
GIYFTQDWVSMPGVLPVASGGIHVWHMPALTEIFGDDSVLQFGGGTLGHPWGNAPGAVAN
RVALEACVQARNEGRDLAREGNEVIREASKWSPELAAACEVWKEIKFEFEAMDVL
>Q6H9K4 | RR4_EQUSY
MSRYRGPRLRIIRRLRNLPGLTNKLVESKKNQASGSDQSNQKKVSQYCIRLEAKQRLRFN
YGLTERQLLNYVRIARCAKGSTGQILLQLLEMRLDNILFRLGVVPTIPSARQLINHRHIL
VNNRIVDIPSFHCKPKDIITIGAPKTYQSIITKRIEAFAKDQIPDHLTLSLSEPKKPKGF
VNYLINRESIGLKINELLVVEYYSRKA
>Q6H9K7 | RR4_EQUPT
MSRYRGPRLRIIRRLRNLPGLTNKLVESKKNQVSGSDQSNQKKVSQYCIRLEAKQRLRFN
YGLTERQLLNYVRIARCAKGSTGQILLQLLEMRLDNILFRLGVVPTIPSARQLINHRHIL
VNNRIVDIPSFHCKPKDIITIGAPKTYQSIITKRIESFAKDQIPDHLTLSLSEPKKPKGF
VNYLINRESIGLKINELLVVEYYSRKA
>Q6H9L0 | RR4_EQULA
MSRYRGPRLRIIRRLQNLPGLTNKLVESKKNKVSGSDQSIQKKVSQYGIRLEAKQRLRFN
YGLTERQLLNYVRIARGAKGSTGQILLQLLEMRLDNILFRLGFVPTIPSARQLINHRHIL
VNNRIVDVPSFHCKPKDIITIGAPKTYQSILSKRLESFAKDQIPEHLTLSLSEPKKPKGF
VNYLINRESIGLKINELLVVEYYSRKA
>Q6H9L1 | RR4_EQUHY
MSRYRGPRLRIIRRLQNLPGLTNKLVESKKKKVSGSDQSIQKKVSQYGIRLEAKQRLRFN
YGLTERQLLNYVRIARCAKGSTGQILLQLLEMRLDNILFRLGFVPTIPSARQLINHRHIL
VNNRIVDVPSFHCKPKDIITIEAPKTYQSILSKRLESFAKDQIPEHLTLSLSEPKKPKGF
VNYLINRESIGLKINELLVVEYYSRKA
>Q6H9L3 | RR4_EQUFL
MSRYRGPRLRIIRRLRNLPGLTNKLVESKKNQASGNDQSNQKKVSQYCIRLEAKQRLRFN
YGLTERQLLNYVRIARCAKGSTGQILLQLLEMRLDNILFRLGVVPTIPSARQLINHRHIL
VNNRIVDIPSFHCKPKDIITIGAPKTYQSIITKRIEAFAKDQIPDHLTLSLSEQKKPKGF
VNYLINRESIGLKINELLVVEYYSRKA
>Q6KGV8 | RR7_SPAWA
MSRRGTAEKKTAKSDPIYRNRLVNMLVNRILKHGKKSLAYQIIYRAVKKIQQKTETNPLS
VLRQAIRGVTPDIAVKARRVGGSTHQVPIEIGSTQGKALAIRWLLGASRKRPGRNMAFKL
SSELVDAAKGSGDAIRKKEETHKMAEANRAFAHFR
>Q6KGW1 | RR7_SCIVE
MSRQTPARKRRKKYSEKSDAIYHNRLVNMLVNRILKNGKKALAYKIFYRSMKTIYENTNI
NPLLIIRQAIQTLTPKVMVKARRVTGTTHQIPIDVKPKQGTILAIRWLLESSRKRSGQTM
HDKLSHEIMDAARNKGHAIRKKEETHKMAESNRAXAHYR
>Q6KGW4 | RR7_SCHCH
MSRRGTAEEKTAKSDPIYRNRLVNMLVNRILKHGKKSLAYQIIYRAVKKIQQKTEANPLS
VLRQAIRGVTPDIAVKARRVGGSTHQVPIEIGSTQGKALAIRWLLGASRKRPGRNMAFKL
SSELVDAAKGSGDAIRKKEETHRMAEANRAFAHFR
>Q6KGW7 | RR7_SAGLA
MSRRGTAKGKTAKYDPIYRNRLVNMLVNRILKHGKKALAYKILYGAVKKIQQNTKTNPLS
ILRQAIRGVTPDIAVKARRKSGSTRQVPIEIGSTQGKTLAIRWLLGASRKRPGQNMAFKL
SSELVDAAKGRGGAIRKKEETIKMAEANRAFAHFR

>Q6KGX6 | RR7_MAGST
MSRRGTAEEKTAKSDPIYRNRLVNMLVNRILKHGKKSLAYQILYRAVKKIQQKTETNPLS
VLRQAIRGVTPDIAVKARRVGGSTHQVPIEIGSTQGKALAIRWLLGASRKRPGRNMAFKL
SSELVDAAKGSGDAIRKKEETHRMAEANRAFAHFR
>Q6KGX9 | RR7_LILSU
MSRRGTAEEKTAKSDPIYRNRLVNMLVNRILKHGKKSLAYQIIYRALKKIQQKTETNPLS
VLRQAIRGVTPNIAVKARRVGGSTHQVPIEIGSTQGKALAIRWLLGASRKRPGRNMAFKL
SSELVDAAKGGGDAIRKKEETHRMAEANRAFAHFR
>Q6L380 | RK33_SACHY
MAKGKDVRIRVILECISCVRKGTNKESTGISRYSTQKNRHNTPGQLELRKFCRYCRKHTT
HNEIKK
>Q6L394 | NU3C_SACHY
MFLLHEYDIFWTFLIIASLIPILVFWISGLLAPVSEGPEKLSSYESGIEPMGGAWLQFRI
RYYMFALVFVVFDVETVFLYPWAMSFDVLGVSVFIEAFIFVLILVVGLVYAWRKGALEWS

>Q6L397 | RR4_SACHY
MSRYRGPRLKKIRRLGALPGLTRKTPKSGSNQKKKFHSGKKEQYRIRLQEKQKLRFHYGL
TERQLLRYVHIAGKAKRSTGQVLLQLLEMRLDNILFRLGMASTIPGARQLVNHRHILVNG
RIVDIPSFRCKPRDIITTKDNQRSKRLVQNYIASSDPGKLPKHLTVDTLQYKGLVKKILD
RKWVGLKINELLVVEYYSRQT
>Q6L3A4 | RR2_SACHY
MTRRYWNINLKEMIEAGVHFGHGIKKWNPKMAPYISAKRKGTHITNLARTARFLSEACDL
VFDAASQGKSFLIVGTKKRAADLVASAAIRSRCHYVNKKWFSGMLTNWSITKTRLSQFRD
LRAEEKMGKFHHLPKRDAAILKRKLSTLQRYLGGIKYMTRLPDIVIVLDQQKEYIALREC
AILGIPTISLVDTNCDPDLANISIPANDDTMTSIRLILNKLVFAISEGRSLYIRNR
>Q6L3A7 | RPOB_SACHY
MLRNGNEGMSTIPGFSQIQFEGFCRFINQGLAEELEKFPTIKDPDHEIAFQLFAKGYQLL
EPSIKERNAVYESLTYSSELYVSARLIFGFDVQKQTISIGNIPIMNSLGTFIINGIYRIV
INQILLSPGIYYRSELDHKGISICTGTIISDWGGRLELAIDKKERIWARVSRKQKISILV
LLSAMGLNLREILDNVSYPEIFLSFLNAKEKKRIESKEKAILEFYQQFACVGGDLVFSES
LCEELQKKFFQQKCELGRVGRRNMNRRLNLDIPQNNTFLLPRDVLAATDHLIGMKFGTGI
LDDDDMNHLKNKRIRSVADLLQDQFGLALGRLQHAVQKTIRRVFIRQSKPTPQTLVTPTS
TSILLITTYETFFGTYPLAQVFDQTNPLTQTVHGRKVSCLGPGGLTGRTASFRSRDIHPS
HYGRICPIDTSEGINVGLTGSLAIHARIDHWWGSIESPFYEISEKAKEKKERQVVYLSPN
RDEYYMIAAGNSLSLNQGIQEEQVVPARYRQEFLTIAWEQIHVRSIFPFQYFSIGGSLIP
FIEHNDANRALMSSNMQRQAVPLSRSEKCIVGTGLERQTALDSRVSVIAEREGKIISSDS
HKILLSSSGKTISIPLVAHRRSNKNTCMHQKPRVPRGKSIKKGQILAEGAATVGGELALG
KNVLVAYMPWEGYNFEDAVLISERLVYEDIYTSFHIRKYEIQTDTTSQGSAEKITKQIPH
LEEHLLRNLDRNGVVRLGSWVETGDILVGKLTPQIASESSYIAEAGLLRAIFGLEVSTSK
ETSLKLPIGGRGRVIDVKWIQRDPFDIMVRVYILQKREIKVGDKVAGRHGNKGIISKILP
RQDMPYLQDGTPVDMVFNPLGVPSRMNVGQIFESSLGLAGDLLKKHYRIAPFDERYEQEA
SRKLVFSELYEASKQTKNPWVFEPEYPGKSRIFDGRTGDPFEQPVLIGKSYILKLIHQVD
EKIHGRSTGPYSLVTQQPVRGRAKQGGQRIGEMEVWALEGFGVAHILQEILTYKSDHLIA
RQEILNATIWGKRVPNHEDPPESFRVLVRELRSLALELNHFLVSEKNFRVNREDV

>Q6L3B3 | PSBK_SACHY
MPNILSLTCICFNSVLCPTSFFFAKLPEAYAIFNPIVDVMPVIPVLFFLLAFVWQAAVSF
R
>Q6L3B4 | RR16_SACHY
MVKLRLKRCGRKQQAIYRIVAIDVRSRREGRDLRKVGFYDPIKNQTCLNVPAILYFLEKG
AQPTRTVYDILRKAEFFKDKERTLS
>Q6L3B6 | RR19_SACHY
MTRKKTNPFVARHLLAKIEKVNMKEEKEIIVTWSRASSILPAMVGHTIAIHNGKEHIPIY
ITNPMVGRKLGEFVPTRHFTSYESTRKDTKSRR
>Q6L3B9 | RK2_SACHY
MAKHLYKTPIPSTRKGTVDRQVKSNPRNKLIHGRHRCGKGRNARGIITARHRGGGHKRLY
RKIDFRRNQKDISGRIVTIEYDPNRNAYICLIHYGDGEKRYILHPRGAIIGDTIVSGTKV
PISMGNALPLTDMPLGTAIHNIEITRGRGGQLARAAGAVAKLIAKEGKLATLRLPSGEVR
LVSQNCLATVGQVGNVGVNQKSLGRAGSKCWLGKRPVVRGVVMNPVDHPHGGGEGKAPIG
RKKPTTPWGYPALGRRTRKRKKYSDSFILRRRK
>Q6L3D3 | RR15_SACHY
MVKEEKQENRGSVEFQVFSFTNKIRRLASHLELHKKDFSSERGLRRLLGKRQRLLAYLAK
KNRVRYKKLISQLDIREK
>Q6L3D7 | NU6C_SACHY
MDLPGPIHEILVLFGGFVLLLGGLGVVLLTNPIYSAFSLGLVLVCISLFYFLLNSYFVAV
AQLLIYVGAINVLIIFAVMFVNGSEWSKDKNYWTIGDGFTLLLCITIPFSLMTTIPDTSW
YGILWTTRSNQIVEQGLINNVQQIGIHLATDFYLPFELISLILLVSLIGAITMARQ
>Q6L3E0 | NU4C_SACHY
MSYFPWLTILVVLPIFAGSLIFFLPHKGNKIVRWYTIAICLLEFLIMTYAFCYHFQLEDP
LIQLKEDSKWIDVFDFHWRLGIDGLSLGSILLTGFITTLATLAAWPVTRNSQLFYFLMLA
MYSGQIGLFSSRDLLLFFIMWELELIPVYLLLSMWGGKRRLYSATKFILYTAGGSIFFLI
GVLGMGLYGSNEPGLDLERLINQSYPTTLEILLYFGFLIAYAVKLPIIPLHTWLPDTHGE
AHYSTCMLLAGILLKMGAYGLIRVNMELLPHAHYLFSPWLVIIGAVQIIYAALTSLGQRN
FKKRIAYSSVSHMGFIIIGIGSITNIGLNGAILQILSHGFIGATLFFLAGTACDRMRLVY
LEELGGISIPMPKIFTMFSSFSMASLALPGMSGFVAELVVFFGLITSPKFMLMPKMLITF
VMAIGMILTPIYLLSMLRQMFYGYKLFHVPNKNFVDSGPRELFLLICIFLPVIGIGIYPD
FVLSLSVDRVEVLLSNYYTK
>Q6L3F1 | YCF73_SACHY
MTKDETLLVFTLVVSSVSIFLFGILLFMVLISATRDFRERTKSKLVKIMIWAGIVVITFA
IAVRIYPIFIFLLKERIKPLVEALYDKLPWIWEVSLSRYWDRLIDFLDRYLWACAQRIQT
GIRKQKGEFVVTFSCRVKKRLYARAIEVGIHLSLLSNLFWILKTTLAVGYRLL
>Q6LA06 | MATK_TYPLA
MKQLHVQKYLEKVRSRKQHFLYPLLFKEYIYAFAHDYGLNGSIFYEPTEILGNDNKSSSV
LVKRLIIRMYQQNYLINSTNHSNQNRFLGHNNYFYSRFFSQMISESFAVIMEILFSLRLV
SSSEKKEIPQFHNLRSIHSIFPFLEDKLSHLNSISDILIPHPIHLEILVQILQCRIQDVP
SLHLLRFFLHEYHNWNSLITSKKSIYVFSKENKRLFRLLYNFYVFECEFVFVFLRKQSSY
LQLTSFGTFLERIHFYGKIEHLLVVYRNFFNKTLWFFTDPFMHYVRYQGKAILASKGTHL
FMKKWKCYLVNFWQYYFHFWSQPHRIHINQLSNYSFHFLGYLSSLLRNPLVVRNQMLENS
YLIDTVMTKFDTIXPVDPLIGSLSKAKFXTLLGHPISKPIWTDLSDCDIIDRFGRICRXL

SHYYSGSSKKRTLYRIKYILRFSCARTLARXHKSTVRTFMQRLGSVLLEEFFTEEEQVLS
LIFPKTTPFSLHGSHRERIWYLDIIRINDLVNH
>Q6LA20 | MATK_DIAEN
MEELQGYLEKGESRQQHFLYPLLFQEYIYALAHDYSLNSSIFYEPMEIFGYDNKSSLALV
KRLIIRIYQQNSLISSVNDSNQNRLLWHNHFFYSHFYSQMISESFGILVEIPFSLRLVSY
FEEIEISKYXNLRSIHSIFPFLEDKLSHLNYVSDILIPHPIHMEILVQILQCWIQDVPFL
HFLQFFLREYHNWNSLLITQKKSIYVFSKENKRLFRLLYNSYAFECEFLFXFIRXQSYYL
RXISYGTFLERTHFYGKIEHLQIEHFIFIVVCRNYFHRTLWLFKDSFMHYVRYEGKAILA
SKGTGTPLLMKKWKYLFFHFWQYYFHFWSQPYRIHISPLSKHSFYFLGYLSSLLRNFLAV
RNQMLDNSFLTDTIIKKLDTTVPVILLIGSLSKAKFCTVSGHPISKPIWADLPDSDILDQ
FGRICKNLSHYHSGSSKKRDLYRIKYILRLSCARTLARKHKSTVRTFLRRLGSGLLEEFF
TEEEQVLSLIFPKTTPFILHGSHRERIWYLDIIRINDLVNYS
>Q6PP78 | MATK_VIGUN
MEQYQAYLELRRSRYQDILYPLFFRESIYGLAYAHESFFIENVDYNNKFSLLIVKRLSTR
MYQQTHFILFVNDSKKNTFVGYNYHFFSQIILEGFGIVVEILFSLQLFSSSLRGLEIVKS
YKNFQSIHSIFPFFEDQLIYLNHKSDIRIPYPIHLEILVQILRYSIKDVSFFHLIRLFFY
YYYNWNSLFPPKKWIFTFFSKRNRRIFLFLYNLYVWEYESIFLFLRNKSSQLQLKHFRVF
FERIFFYEKIKHLVEVSTKNCSYTFFFFKDTFIHYVRYQGKSILVLKNTPFLINKWKYYF
IYLWQCHFDIWAGLETIYINELSQYSFHFLGYFLSIPLNLSVVRSQMLQNSFLIKIVIKK
LDTIVPIIPLMRSLAKTKFCNVMGHPISKPVWANLSDFDIIDRFLRICRNFSHYYNGSAK
KKSFYQIKYILRFSCIKTLARKHKSTVRIYLKKLSSEKLLEEFFTEEDLFSLIFPRTSFT
LRRFYRGRIWYLDILLRNDFVNYL
>Q6PSB9 | MATK_VIGMU
MEQYKAYLELHRSRYQDILYPLFFRESIYGLAYRHESFFIENVDYNNNFSLLIVKRLSTR
MYQQTHFILFVNDSKKNTFVGYNYHFYSQIILEGFGIVVEILFSLQLFSSSFRGLEIVKS
YTNLQSIHSIFPFFEDKLIYLNHKSDIRIPYPIHLEILVQILRYSIKDVSFFHLIRLFFY
YYSNWNSLFPPKKWIFTFFSKRNRRIFLFLYNLYVWEYESIFLFLRNKSSQLQLKHFRVF
FERIFFYEKIKHLVKVSTKNCSYTLFFFKDTFIHYVRYQGKSILVLKNTPFLINKWKYYF
IYLWQCHFDIWAGLETIYINELSQYSFHFLGYFLSIPLNLSVVRSQMLQNSFLIQIVIKK
LDTIVPIIPLMRSLAKTKFCNVMGHPISKPVWANLSDFDILDRFLRICRNFSHYYNGSAK
KKSFYQIKYILRFSCIKTLARKHKSTVRIYLKKLSSEKLLEEFFTEEDLFSLIFPRTSLT
LRRFYRGRIWYLDILFRNDFVNYL
>Q6PSE2 | MATK_AMPBR
MEEYRAYLELHRSRHQDTLYPLFFREYIYGLACGHGSILVESVGYNNKFSLLIVKRLITR
MYQQTHFIIFANDSNKNPFRGYNNHFYSQIILEGFVVVVEIRFSLQLFISSLRELEIIKS
YNNLRSIHSIFPFFEDKLIYLNLESDIRIPYPIHLEILVQILRYWIKDVSFFHLLRLFFS
YYYNRNNLFTPKKWISTFFSKSNPSFFLFLYNLYVQEYESIFIFLRNKSSQLRLKYFRVF
FERIFFYEKIEHLVEVSVKDCSYTFSFFKDTFIHYVRYQGKSILVSKNTPLFINKWKYYF
IYLWQCHFDIWSRPGTIHINQLSRHSFHFLGYFLSIRLNFSVVRSQMLQNSFLIKIVMKK
LDTIVPIISLIRSLAKAKFCNVFGHPISKPVWANLSDFDIIDRFLRICRNFYHYYNGSAK
KKSLYQIRYILRLSCIKTLARKHKSTARTFLKRLGSEKLLEEFFTEEEDIFSLIFPIPKT
SFTVQRLYRGRIWYLDILFRNDFVNHL
>Q6QHD9 | MATK_BRACM
MEKFQGYLEFDGARQQSFLYPLFFRDYIYVLAYDHGLNRLNRNRPIFLENADYDKKYSSL

IVKRLILRMYEQNRLIIPTKDLNKNLGHTNNFYYQMISVLFAVIVEIPFSLRLGSSIEGK
NVKKSYNLQSLHSIFPFLEDKLSHFNYVLDVLIPYPIHLEILVQTLRYRVKDASSLHFFR
FCLYEYCNWKNFDSKKKSILNPRFLLFLYNSHVCEYESIFFFLRKQSSHLRSTSYDVFFE
RILFYGKIQHFFKVFVNNFSALLGLLKDPFLHYVRYHGKYILATKDTPLLMNKWKYYFVN
LWQCYFSVWFQSQKVNINQLSKDNLEFLGYLSSLRLNPLVVRSQMLENSFLIDNVRIKLD
SNIPISSIIGSLAKDKFCNVLGHPISKATWTDSSDSDILNRFVRICRNISHYYSGSSNKK
NLYRIKYILRLCCVKTLARKHKSTVRAFLKRLGSGLLEEFLTGEDQVLSLIFPRSDYASK
RLYRVRVWYLDILYLNDLVNHE
>Q6QUL7 | MATK_CALFL
MEELQGYLEIDGFRQHHFLYPLLLQEYIYALAHDHGLNGSILSEHMENLSHDNKSSSLIV
KRLITRMHQQNHFIISVNDSNQKGFVGHNKNFHSQKISEGFAVIVEIPFSLQLVSSLEEK
EIAKFHNSRSIHSIFPFFEDKLSHLNHVSDILIPYPIHLEILVQTLRCWIQDAPSLHLLR
FFLHEYWNSNSLITPKKSISFFSKENQRLFLFLYNSHVYECESVFIFLRKQSSHLRSTSF
GSFLERTHFYGKIEHLVVVLGNDFPKTLWLFKDPFVHYVRYQGKSILASRGTQFLIKKWK
YHLVNFWQCHFYLWSQPDRIHLNQLCNHSFYFLGYLSSVQLNSSVVRSQMLENAFRMDTA
IKKFETIVPIIPLIGSLAKAKFCNGSGHPISKPFRTDLSDSEIINRFGRICKNLSHYHSG
SSKKQSLYRIKFILRLSCARTLSRKHKSTVRAFLKRLGSELLEEFLTEEEQVLSLLFPRT
PSHRPHRERIWYLDIICINDLANHE
>Q6TND0 | MATK_DALWR
MEEYKVYLELDRSRQQDFLYPFIFQESIYGLVYGHDLNGSILVENVDYDNKSSLLIVKRL
ITRMYQQNHLIVSANDFNKNQQFWGYNKNLYSQIISEAFAIVVEIPFYSQLRSSLEGLEG
AEVIKSYNKLRSIHAIFPFFEDKFTYLNYVSDVQIPYPIHLEILVQILRYWVKDPPLFHL
LRSFLYQYCNWNSFINPKKSISSFSKSNPRFFFFLYNFYVCEYESIFLFLRKKSSHLRLT
SFSVLFERIYFYAKIEHLVEVFPKDFLSTLSLFKDPLIHYLRYQGKSILASKNAPLLMNK
WKYYLISLWQCYFNVWSQPGTIYINQLSDHSFHFFWGGYFSNVRLNLSVVRSQMLENSFL
IEIVMKKLDTIVPILPIIRSLAKAKFCNVLGHPISKPVWADLSDFGIIDRFLRIRRNISH
YYNGSSKKKSLYRIKYILRLSCIKTLVRKHKSTVRAFLKRLGSEELLKEFFTEEEDILSL
IFPRASSTLQRLYGGRIWYLDIIFSNDLVNHS
>Q6VQA9 | CHLL_CHLPR
MKLAVYGKGGIGKSTTSCNISIALARRGKKVLQIGCDPKHDSTFTLTGFLIPTIIDTLQA
KDYHYEDVWPEDVIYQGYGEVDSVEAGGPPAGAGCGGYVVGETVKLLKELNAFYEYDVIL
FDVLGDVVCGGFAAPLNYADYCLIVTDNGFDALFAANRIVASVREKSKTHPLRLAGLIGN
RTSKRDLIDKYVEVCPMPVIEVLPLIEDIRVSRVKGKTVFEMAETDQKLNYICDFYLNIA
DQLLASPEGVIPLELEDRELFTLLSTFYLTVTPQNQGETQSTISTPLTSNSASELDFILV

>Q6YLW2 | RPOA_SECCE
MVREEVAGSTQTLQWKCVESRVDSKRLYYGRFILSPLRKGQADTVGIALRRALLGEIEGT
CITRAKFGSVPHEYSTIAGIEESVQEILLNLKEIVLRSNLYGVRDASICVKGPRYITAQD
IILPPSVEIVDTAQPIANLTEPIDFCIDLQIKRDRGYQTELRKNYQDGSYPIDAVSMPVR
NVNYSIFSCGNGNEKHEILFLEIWTNGSLTPKEALYEASRNLIDLFLPFLHAEEEGASFE
ENKNRFTPPLFTFQKRLTNLKKNKKGIPLNCIFIDQLELTSRTYNCLKRANIHTLLDLLS
KTEEDLLRIDSFRMEDRKHIWDTLEKHLPIDLLKNKLSF
>Q6YP18 | MATK_PICEN
MDEFHRYGKEDSSWQQCFLYPLFFQEDLYAISHDHYLDGSSSSEPMEHLSSNDQFSFLTV

KRLIGQIRQQNHSIVLFVNCDPNPLVDRKKSSYSESVLEGLTLVLEVPFSIRSKYSVEGM
NEWKSFRSIHSIFPFLEDKFPHSNYVSDTRIPYSIHPEILVRTFRRWIGDAPSLHPLRSI
LYEYRNSSESLQRSIIVVPKVNTRFFLFLWNNYVYECESILVSLLKRSSHSRSLSHGSFP
QRTHFHRKIKNIFLFSRRNSFQSIWSLKDPNIHYVRYGERSIIAIKGTNLLVKKYRYYLP
IFRQCYFHLWNEPYRVCSHQLSKNCSSSLGYFLRFRMKPLLVKTKMLDELFIADLITDEF
DPIVPIVPIIGLLSREKFCDISGRPISKLSWTSLTDDDILDRFDRIWRNLFHYYSGSFGR
DGLYRIKYILSLSCAKTLACKHKSTIRVVRKELGPELFKKSFSKERELDSPPFSSKAAAR
SQRERIWHSDIPQINPLAHSWQKIQDLKIENLFDQ
>Q6YXJ8 | RR11_PHYPA
MAKLIKKISLRKGKRRIPKGVIHIQASFNNTIVTVTDIRGQVVFWSSAGACGFKGAKKST
PFAAQTAAENAIRVLIDQGMKQAEVMISGPGPGRDTALRAIRRSGVILNFVRDVTPMPHN
GCRPPKKRRV
>Q6YXK5 | RK23_PHYPA
MDEIKYPVLTEKTIRLLEKNQYTFDVNKKSTKPQIKKWIENFFNVKVKAINSHIPPEKKK
RIGPIIGHSVRYKRMIITLKTGYSIPLFSNK
>Q6YXK6 | RR19_PHYPA
MTRSLKKGPFVADHLLKKIEDLNLKKEKKIIITWSRASTIVPTMIGHTIAVYNGQEHLPI
YITDRMIGHKLGEFAPTRNFRGHTKSDKKSRR
>Q6YXL2 | IF1C_PHYPA
MKKQNLIDMEGVVTESLPNAMFRVCLDNGCEVLTHISGKIRRNYIRILPGDRVKVELTPY
DLTKGRITYRLRAKSSNS
>Q6YXM4 | RR18_PHYPA
MKQAINKSKRSSRRRLPPIRSGEIIDYKNINLLRRFISEQGKILSRRMNRLTSKQQRLMT
IAIKRARVLALLPFLNNEN
>Q6YXM5 | RK20_PHYPA
MTRVKRGYVARKRRKNIFTLTSGFQGAHSKLFRTANQQGMRALASSYRDRNRRKRDLRRL
WITRINAAARNNGISYNKLIQNLYQNQILLNRKMLAQIALLDTNCFSTIMKKINE
>Q6YXP3 | RR4_PHYPA
MSRYRGPRVRIIRRLGVLPGLTNKTPQLKSSSANQSTAKKISQYRIRLEEKQKLRFHYGI
TERQLLNYVRIARKAKGSTGQILLQLLEMRLDNIVFRLGMAPTIPGARQLVNHRHVLVND
CIVDIPSYRCKPEDSITVKNRQKSQAIITKNIDFSQKSKVPNHLTFDSTQKKGLVNQILD
RESIGLKINELLVVEYYSRQA
>Q6YXP6 | RR15_PHYPA
MSKKLFIGSSLLSKEQTGSVEFQISHLTNRVLKLTDHLKFHDKDYSSQRGLLKILGKRKR
LLSYLSKTNLTSYETLINKLNIRKLKNR
>Q6YXQ3 | NU4C_PHYPA
MSNFPWLTTIVLLPVFAGCVIPFFPNKGNNLIRWYTLGVCLLEFLLITYVFCYYFKFDDP
IIQLKEDYNWIDFLDFHWRLGIDGLSIGLILLTGFITTLATLAAWPVTRNPRLFYFLMLA
MYSGQVGLFASQDILLFFFMWELELIPVYLLLCIWGGKRRLYATTKFILYTAGGSIFILM
GALTMGFYGSNQLTLDFQNLSNKSYPLELEIILYLGFFIAYAVKLPIFPLHTWLPDTHGE
AHYSTCMLLAGILLKMGGYGLIRINMELLPHAHSIFAPWIVAVGAIQIVYAALISFSQRN
LKRRIAYSSISHMGFVLIGIGSMTDVGLNGAILQMVSHGLIGAALFFLAGITYDRTRTLF
LDQMGGTAIYMPKIFTMFSSFSMASLALPGMSGFVAEFLVFLGIVVSNKYSFNFKILVTI
IEAIGIILTPIYLLSMLRQMFYGYKFSKFTTFSNSNMDAGPREIFILVCLIFPIVGIGLY

PNSVLSLWNSKVSFILSKFIV
>Q6YXQ4 | RK32_PHYPA
MAVPKKRTSKSKKKIRETVWREKANQARIKAFSLAQSILTGRSKSFYYTTTEKDSNLSE
>Q6YXQ5 | RK21_PHYPA
MYAIIETGGEQLRVEPGRFYDIRHFASLKSKNLSSNTKILIYRVLMIRNETTINIGQPWL
KNAVIKGRILHSHLENKITVYKMNSKKKTRRKFGHRQNSARFVVDSICLDGKDL
>Q70CZ6 | MATK_CESEL
MEEIQRYLQPDRSQQHNFLYPLIFQEYIYALAHDHGLNINRSILLENPGYNNQLSLLIVK
RLITRMYPQNHFFICTNDSNQNPFLGCNKSLYSQMISEGFAFILEIPFSLQLISSSSLSL
EGKNVFKSHNLRSIHSTFPFLEDNFSHLNYVLDILIPYPVHLEILXQTLRYWVKDASSLH
LLRFFLHEYWNLNSLITPKKPGYSFSKKKKRFFFFLYNSYVYECESTFVFLRNQSYHLRS
TSFGALLERIYFYGKIECLVEVFAKDFQXTLWLFKDPXMHYVRYQGKSILASKGTFLLMN
KWKFYLVNFWQCHFSXCFETGRIHINQLSNHSRDFLGYLSSVRLNPSMVRSQILENSFLI
NNAIKKFDTLVPIIPLIGSLAKANFCTVLGHPISKPVWSDLSDSDIIDRFGRICRKLFHY
YSGSSKKKTLYRIKYILRLSCGRTLARKHKSTVRAFLKRSGSELLEEFLTSEEQVLSLTF
RRASSSLWGVYRNRIWYLDIFSINDLANYQ
>Q70D04 | MATK_MANOF
MEEIQRYLQPDSSSSQQHNFLYPLIFQEYIYALAHDHGLNRNRSILLENPGYNNKFSFLI
VKRLITRMYQQNHFRISTNDSNKNPFLGCNKSLYSQMISEGFAFIVEIPFSLRLISSLSS
FEGKKIFKSHNLRSIHSTFPFLEDNFAHLNYVLDILIPYPVHLEILVQTLRYWVKDASSL
HLLRFFLHEYWNLNSLITSKKPGYSFSKTNQRFFFFLYNSYVYESESTFVFLRNQSSHLR
STSFGALLERIYFYGKIERLVEVFAKDFQVTLWLFKDPFMHYVRYQGKSILASKGTFLLM
NKWKFYLVNFWQCNFSLCFPTGRIHINQLSNHSRDFMGYLSSVRLNPSMVRSQMLENAFL
INNGIKKFDTLVPIIPLIGSLAKANFCTVLGHPISKPVWSDLSDSDIIDRFGRICRNLFH
YYSGSSKKKTLYRIKYILRLSCARTLARKHKSTVRTFLKRSGSELLEEFLTSEEQVLSLT
FPRASSSLWGVYRSRIWYLDIFCINDLANYQ
>Q70D38 | MATK_NICAC
MEEIQRYLQPDRSQQHNFLYPLIFQEYIYALAHDHGLNRNRSILLENPGYNNKLSFLIVK
RLITRMYQQNHFLISTNDSNKNSFLGCNKSLYSQMISEGFAFIVEIPFSLRLISSLSSFE
GKKIFKSHNLRSIHSTFPFLEDNFSHLNYVLDILIPYPVHLEILVQTLRYWVKDASSLHL
LRFFLHEYWNLNSLITSKKPGYSFSKKNQRFFFFLYNSYVYECESTFVFLRNQSSHLRST
SFGALLERIYFYGKIERLVEVFAKDFQVTLWLFKDPFMHYVRYQGKSILASKGTFLLMNK
WKFYLVNFWQCHFYLCFHTGRIHINQLSNHSRDFMGYLSSVRLNPSMVRSQMLENSFLIN
NAIKKFDTLVPIIPLIGSLAKGNFCTVLGHPISKPVWSDLSDSDIIDRFGRICRNLFHYY
SGSSKKKTLYRIKYILRLSCARTLARKHKSTVRTFLKRSGSELLEEFLTSEEQVLSLTFP
RASSSLWGVYRSRIWYLDIFCINDLANYQ
>Q70D40 | MATK_NICTO
MEEIQRYLQPDRSQQHNFLYPLIFQEYIYALAHDHGLNRNRSILLENPGYNNKLSFLIVK
RLITRMYQQNHFLISTNDSNKNEFLGCNKSLYSQMISEGFAFIVEIPFSLRLISSLSSFE
GKKIFKSHNLRSIHSTFPFLEDNFSHLNYVLDILIPYPVHLEILVQTLRYWVKDASSLHL
LRFFLHEYWNLNSLITSKKPGYSFSKKKQRFFFFLYNSYVYECESTFVFLRNQSSHLRST
SFGALLERIYFYGKIERLIEVFAKDFQVTLCLFKDPFMHYVRYQGKSILASKGTFLLMNK
WKFYLVNFWQCHFSLCFHTGRIHINQLSNHSRDFMGYLSSVRLNSSMVRSQMLENSFLIN
NAIKKFDTLVPIIPLIGSLAKANFCTVLGHPISKPVWSDLSDSDIIDRFGRICKNLFHYY

SGSSKKKTLYRIKYILRLSCARTLARKHKSTVRAFLKRSGSELLEEFLTSEEQVLSLTFP
RASSNLWGVYRSRIWYLDIFCINDLANYQ
>Q70XV7 | RR15_AMBTC
MVINSFISSISKEEKKGSVEFQVFCFTDKIRKLTSHLELHKKDFLSQRGMRKILGKRQRM
LAYLSNKNKVRYKKLIGQLNIREPKTR
>Q70XX2 | RR3_AMBTC
MGQKINPLGFRLGTTQSHRSLWFAQPKDYSRNLQEDEKIRDCIKNYVQKHMRISSGFQGI
ARLGIQKRIDLIQVTIYIGSSNLLIEGPTRGIEELRTDVQKKLNSMNRRLNITITRIARP
YEQPNILAEYIALQLKNRVSFRKAMKKAIELAEQANTKGIRVQIAGRLNGKEIARVEWIR
EGRVPLHTIRAKIDYCSYMVQTIYGVLGIKIWIFVDEE
>Q70XX3 | RK16_AMBTC
MLSPKRTRFRKQHRGRMKGVSSRGNHICFGRYALQALEPAWITSRQIEAGRRAMTRYARR
GGKIWVRVFPDKPITVRPAETRMGSGKGSPEYWVSVIKPGRILHEMGGVPETVARAAMEI
AACKMPIRTQFISAK
>Q70XX6 | RR11_AMBTC
MKKPIPRIGSRRNGRIGSRKNGRRIPKGVIHVQASFNNTIVTVTDVRGRVVSWCSAGTCG
FRGTRRGTPFAAQTAATNAIRTVVDQGMQRAEVMIKGPGLGRDAALRAIRRSGVLLSFVR
DVTPMPHNGCRPPKKRRV
>Q70XY2 | CLPP_AMBTC
MPVGVPKVPFRSPGEEDATWVDIYNRLHRERLLFLGQEVEGEISNQIVGLMVYLSIEDDT
RDLYLFINSPGGWVIPGIAIYDAMQFVPPDVHTICMGLAASMGSFVLAGGEITKRLAFPH
ARVMIHQPASSFYEAPAGEFILEAGELLKLRETITSVYVQRTGNPLWVVSEDMERDVFMS
ATEAQAHGIVDLIAVENTWDFP
>Q70XY3 | RK20_AMBTC
MTRVRRGYIARRRRTKIRLFASTFRGAHSRLTRTATQQKMRALVSIYRDRGRQKREFRRL
WIARINAATHENGVSYSRLINDLYKRQLLLNRKIPAQIARYNLNSLYMISNEIIKEKRD
>Q70XY4 | RR18_AMBTC
MDKSKQPFRKSKRSFRRRLPPIGSGDRIDYRNMSLIGRFISEQGKILSRRVNRLTLKQQR
LITIAIKQARILSPLPFLNNEKQFERTESLPRITGTRTIKK
>Q717X5 | MATK_LACFR
MEELQSYLKIDRSRQKHFLYPLLFQEYIYALTHDHGLNGSIFYESIENLGYDKKSSSLIV
KRLIIRMHQQNFLILFVNDFRQNQNNKNLDSQIISDAFAVIVEIPFSLQLVSCLEDKKIA
KYQNLRSIHSIFPFLEDKLTHLNYVLNILIPYPIHLEILVQTLRRWIQDAPSLHLLRFFL
HESGNSNSFISPKKSISFFSNGNFRFFLFLYNSHVYECESLFVFLRKQCSYLRSISSGAF
LERTYFYGKIEYLVIVLCNDFQRALCLFKDPFMHYVRYRGKSILASKATYLMIKKWKYHL
VNLWQCHFHFWSQPGRIHIDQLYNHSLHFMGYLSSVRITPSVVRSKMLDNSYLIDNSIRK
FETVVPIISLICSLAKAKFCNGSGHPISKPARSDSSDSDIIDRFGRICINLSHYYSGSSK
KRSLYRIKYILRLSCARTLARKHKSTVRTFLKRFGSFFLEEFFTEAEEVLSLIFPRTPFS
SHRFHKDWIWYLDIIRIHDLANHE
>Q71L19 | RR7_STAER
MSRRSTAEKETAKSDPIYRNRLVNMLVNRILRHGKKSLAYRIIYRAMKNIQQKTEKNPLS
VLRQAIRGVTPNVTVKTRRVGGSTYQVPIEIRSTQAKALAIRWLLGASRKRPPGRNMAFK
LSYELMDAARENGNAIRKKEETHRMAEANRAFAHFR
>Q71L39 | RR7_BOWSE

MSRRSTAEKETAKSDPIYRNRLVNMLVNRILRHGKKSLAYRILYRAMKNIQQKTEKNPLS
VLRQAIRGVTPNVTVKARRVGGSTYQVPIEIRSTQGKALAIRWSLGASRKRPPGRNMAFK
LSYELMDAARENGNAIRKKEEAHRMAEANRAFAHFR
>Q71T50 | RR7_HYDCA
MSRRGTAEEKTAKSDPIYRNRLVNMLVNRILKHGKKSLAYQIIYRALKKIQQKTETNPLS
VLRQAICGVTPDIAVKARRVGGSTHQVPIEIGSTQGKALAIRWLLWASRKRPGRNMAFKL
SSELVDAAKGSGDAIRKKEETHRMAEANRAFAHFR
>Q75VA5 | MATK_QUESU
MEEFQGYLELDRFRQHDFLYPFIFREYSYALAHGHGLNRYMLLENIGYDNKSSLLIVKRL
ITTMYQQNYLIISANDSKKNPFFGYNKNLHSKILSEGFAIIVEIPFYLRLISSLEGAEIV
RFYNLRSIHSIFPFLEEKFPHLNYSADILIPYPAHLEILVQTLRYRVKDASYLHLLRFFL
HEYSNCNSLIITNKSISIFSKSNPRFFLFLYNSYLCEYESIFLFLRNQSSHLRLTSSGVL
FERLCLYRKIEHFAEVFANDFPVIPCFLKDPFMHYVRYQGKSILASKDTPLLMNKWKSYL
VNLWQCHFDVWSHAASIRINQLSKHSLDFLSYFSSVRRNPAVVRNQMLENSFLLNNAPNK
LDTIVPIIPLIGSLAKAKFCNAVGHPISKLTRADLSDFEIINRFLHICRNLSHYYSGSSK
KKNMYRIKYILRLSCVKTLARKHKSTARAFLKRVDSEFFQEFFTEEGGFISLIFPRASFA
LRRLYSGRVWYLDIIFINGLSNHE
>Q75VB1 | MATK_QUELY
MEEFQGYLELDIFRQHDFLYPLIFREYSYALAHGHGLNRYMLLENIGYDNKSSLLIVKRL
ITTMYQQNYLIISANDSKQNPFFGYNKNLHSKILSEGFAIIVEIPFYLRLISSLEGAEIV
RFYNLRSIHSIFPFLEEKFPHLNYSADILIPYPAHLEILVQTLRYRVKDASYLHLLRFFL
HEYSNCNSLIITNKSISIFSKSNPRFFLFLYNSYICEYESIFLFLRNQSSHLRLTSSGVL
FERLCLYRKIEHFAEVFANDFPVIPCFLKDPFMHYVRYQGKSILASKDTPLLMNKWKSYL
VNLWQCHFDVWSHAASIRINQLSKHSLDFLSYFSSVRRNPAVVRNQMLENSFLLNNAPNK
LDTIVPIIPLIGSLAKAKFCNAVGHPISKLTRADLSDFEIINRFLHICRNLSHYYSGSSK
KKNMYRIKYILRLSCVKTLARKHKSTARAFLKRVDSEFFQEFFTEEGGFISLIFPRASFA
LRRLYSGRVWYLDIIFINGLSNHE
>Q75VB2 | MATK_QUEIL
MEEYQGYLELDRFRQHDFLYPLIFREYSYALAHGHGLNRYMLLENIGYDNKSSLLIVKRL
ITTMYQQNYLIISANDSKQNPFFGYNKNLHSKILSEGFAIIVEIPFYLRLISSLEGAEIV
RFYNLRSIHSIFPFLEEKFPHLNYSADILIPYPAHLEILVQTLRYRVKDASYLHLLRFFL
HEYSNCNSLIITNKSLSIFSKSNPRFFLFLYNSYICEYESIFLFLRNQSSHLRLTSSGIL
FERLCLYRKIEHFAEVFANDFTGIPCFLKDPFMHYVRYQGKSILASKDTPLLMNKCKSYL
VNLWQCHFDVWSHAASIRINQLSKHSLDFLSYLSSVRRNPAVVRNQMLENSFLLNNAPNK
LDTIVPIIPLIGSLAKAKFCNAVGHPISKLTRADLSDFEIINRFLHICRNLSHYYSGSSK
KKNMYRIKYILRLSCVKTLARKHKSTARAFLKRVDSEFFQEFFTEEGGFISLIFPRASFA
LRRLYSGRVWYLDIIFINGLSNHE
>Q75VB5 | MATK_QUECE
MEEFQGYLELDRFRQHDFLYPFIFREYSYALAHGHGLNRYMLLENIGYDNKSSLLIVKRL
ITTMYQQNYLIISANDSKQNPFFGYNKNLHSKILSEGFAIIVEIPFYLRLISSLEGAEIV
RFYNLRSIHSIFPFLEEKFPHLNYSADILIPYPAHLEILVQTLRYRVKDASYLHLLRFFL
HEYSNCNSLIITNKSISIFSKSNPRFFLFLYNSYLCEYESIFLFLRNQSSHLRLTSSGVL
FERLCLYRKIEHFAEVFANDFPVIPCFLKDPFMHYVRYQGKSILASKDTPLLMNKWKSYL
VNLWQCHFDVWSHAASIRINQLSKHSLDFLSYFSSVRRNPAVVRNQMLENSFLLNNAPNK

LDTIVPIIPLIGSLAKAKFCNAVGHPISKLTRADLSDFEIINRFLHICRNLSHYYSGSSK
KKNMYRIKYILRLSCVKTLARKHKSTARAFLKRVDSEFFQEFFTEEGGFISLIFPRASFA
LRRLYSGRVWYLDIIFINGLSNHE
>Q76IH4 | MATK_PINSY
MDEFHRCGKEDSFWQQCFLYPLFFQEDLYAISHDHYLDVSSSSRPMEHLSSNDQLSFLTV
KRLIGQIRQQNHSIVLFVNCDPNPLADRKKSFYSESVLEALTLVLEVPFSIWSKSSVEGM
NECKSFRSIHSIFPFLEDKFPHSNSILDARIPYSIHPEILVRTFRRWIRDAPSLHPLRSV
LYDYRNSPENLQRSIIVVPRVNTRFFLFLLNYYVCECESILFSRLKRSSHSRSLSHGSFP
QRTHFHRKIKHIIIFSRRNSLKSIWSLKDPKIHYVRYGERPIIAIKGADLLVKKCRYYLL
IFRQFYFHLWSEPYRVCSHQLSKNCSSSPGYFLRVRMNPLLVRTKTLDELFIPVLITNEM
DPIVPIVPIIGLLATEKFCDISGRPISKLSWTSLTDDDILDRFDQIWRNLFHYYSGSFDR
DGLYRIKYILLLSCAKTLACKHKSTIRVVRKELGPELFKKSFSKEREFDSLPFSSKAAAR
SQRERIWHSDIPQINPLANSWQKIQDLKIENLFDQ
>Q76IH5 | MATK_PINLU
MDEFHRCGKEDSFWQQCFLYPLFFQEDLYAISHDHYLDVSSSSRPMEHLSSNDQLSFLTV
KRLIGQIRQQNHSIVLFVNCDPNPLADRKKSFYSESVLEALTLVLEVPFSIWSKSSVEGM
NECKSFRSIHSIFPFLEDKFPHSNSILDARIPYSIHPEILVRTFRRWIRDAPSLHPLRSV
LYDYRNSPENLQRSIIVVPRVNTRFFLFLLNYYVCECESILFSRLKRSSHSRSLSHGSFP
QRTHFHRKIKHIIIFSRRNSLKSIWSLKDPKIHYVRYGERPIIAIKGADLLVKKCRYYLL
IFRQFYFHLWSEPYRVCSHQLSKNCSSSPGYFLRVRMNPLLVRTKTLDELFIPVLITNEM
DPIVPIVPIIGLLATEKFCDISGRPISKLSWTSLTDDDILDRFDQIWRNLFHYYSGSFDR
DGLYRIKYILLLSCAKTLACKHKSTIRVVRKELGPELFKKSFSKEREFDSLPFSSKAAAR
SQRERIWHSDIPQINPLANSWQKIQDLKIENLFDQ
>Q76LM0 | MATK_HORVD
MEKFEGYSEKQKSRQQYFVYPLLFQEYIYAFAHDYGLNGSEPVEIVSWNNKKFSSLLVKR
LIIRMYQQNFLDNSVNHPNQDRLLDYKIFFYSEFYSQILSEGFAIVVEIPFSLRELSCPK
EKEIPKFQNLRSIHSIFPFLEDKFLHLDYLSHIEIPYPIHLEILVQLLQYRIQDVPSLHL
LRFFLNYYSNWNSFITSMKSILFFQKENKRLVKFLYNSYVSEYEFFLLFLRKQSSCLPLA
YSGTFLERIHFSRKMEHFGIMYPGFSRKTLWFFMDPLIHYVRYQGKAILASKGSFFLKKK
WKCYLINFWQYYFFFWTQPRRIHINQLANSCFDFMGYLSSVPKSPLLVRNQMLENSFLID
TRMKKFDTIVPATLLIGYLSKAQFCTGSGHPISKPIWTDLSDWDILDRFGRICRNLFHYH
SGSSKKRTLYRLKYILRLSCARTLARKHKSTVRTFMQRLGSAFLEEFFTEEEQVFSLMFT
KTTLFSFSGSHTERIWYLDIIGINDLVNPLN
>Q7FNS1 | RR19_ATRBE
MTRSLKKNPFVANHLLKKIDKLNTKAEKEIIVTWSRASTIIPTMIGHTIAIHNGKEHLPI
YITDSMVGHKLGEFAPTLNFRGHAKSDNRSRR
>Q7FNS6 | RK33_ATRBE
MAKGKDVRVTVILECTSCVRNSVDKVSRGISRYITQKNRHNTPNRLELKKFCPYCYKHTI
HGEIKK
>Q7GED0 | RPOA_TAECM
MVREEVAGSTQTLQWKCVESRVDSKRLYYGRFILSPLRKGQADTVGIALRRALLGEIEGT
CITRAKFGSVPHEYSTIAGIEESVQEILLNLKEIVLRSNLYGVRDASICVKGPRYITAQD
IILPPSVEIVDTAQPIANLTEPIDFCIDLQIKRDRGYQTELRKNYQDGSYPIDAVSMPVR
NVNYSIFSCGNGNEKHEILFLEIWTNGSLTPKEALYEASRNLIDLFLPFLHAEEEGASFE

ENKNRFTPPLFTFQKRLTNLKKNKKGIPLNCIFIDQLELTSRTYNCLKRANIHTLLDLLS
KTEEDLLRIDSFRMEDRKHIWDTLEKHLPIDLLKNKLSF
>Q7GED9 | RPOA_AEGCM
MVREEVAGSTQTLQWKCVESRVDSKRLYYGRFILSPLRKGQADTVGIALRRALLGEIEGT
CITRAKFGSVPHEYSTIAGIEESVQEILLNLKEIVLRSNLYGVRDASICVKGPRYITAQD
IILPPSVEIVDTAQPIANLTEPIDFCIDLQIKRDRGYQTELRKNYQDGSYPIDAVSMPVR
NVNYSIFSCGNGNEKHEILFLEIWTNGSLTPKEALYEASRNLIDLFLPFLHAEEEGASFE
ENKNRFTPPLFTFQKRLTNLKKNKKGIPLNCIFIDQLELTSRTYNCLKRANIHTLLDLLS
KTEEDLLRIDSFRMEDRKHIWDTLEKHLPIDLLKNKLSF
>Q7GUC7 | RK14_PINKO
MIQSQTYLNIADNSGARKIMCIRVLGASNRKCAHIGDVIIAIIKEAVPNMPLEKSEVVRA
VVIRTCKEFERDNGMMIRSDDNAAVVIDQEGNPKGTRVFGPVAQELRQLNFTKIVSLAPE
VL
>Q7H6F6 | RPOA_PSAJU
MVREEVAGSTQTLQWKCVESRVDSKRLYYGRFILSPLRKGQADTVGIALRRALLGEIEGT
CITRAKFGSVPHEYSTIAGIEESVQEILLNLKEIVLRSNLYGVRDASICVKGPRYITAQD
IILPPSVEIVDTAQPIANLTEPIDFCIDLQIKRDRGYQTELRKNYQDGSYPIDAVSMPVR
NVNYSIFSCGNGNEKHEILFLEIWTNGSLTPKEALYEASRNLIDLFLPFLHAEEEGTSFE
ENKNRFTPPLFTFQKRLTNLKKNKKGIPLNCIFIDQLELTSRTYNCLKRANIHTLLDLLS
KTEEDLMRIDSFRMEDRKHIWDTLEKHLPIDLLKNKLSF
>Q7H6J1 | RPOA_HORJU
MVREEVAGSTQTLQWKCVESRVDSKRLYYGRFILSPLRKGQADTVGIALRRALLGEIEGT
CITRAKFGSVPHEYSTIAGIEESVQEILLNLKEIVLRSNLYGVRDASICVKGPRYITAQD
IILPPSVETVDTAQPIANLTEPIDFCIDLQIKRDRGYQTELRKNYQDGSYPIDAVSMPVR
NVNYSIFSCGNGNEKHEILFLEIWTNGSLTPKEALYEASRNLIDLFLPFLHAEEEGTSFE
ENKNRFTPPLFTFQKRLTNLKKNKKGIPLNCIFIDQLELTSRTYNCLKRANIHTLLDLLS
KTEEDLMRIDSFRMEDRKHIWDTLEKHLPIDLLKNKLSF
>Q7H6J2 | RPOA_HORBU
MVREEVAGSTQTLQWKCVESRVDSKRLYYGRFILSPLRKGQADTVGIALRRALLGEIEGT
CITRAKFGSVPHEYSTIAGIEESVQEILLNLKEIVLRSNLYGVRDASICVKGPRYITAQD
IILPPSVETVDTAQPIANLTEPIDFCIDLQIKRDRGYQTELRKNYQDGSYPIDAVSMPVR
NVNYSIFSCGNGNEKHEILFLEIWTNGSLTPKEALYEASRNLIDLFLPFLHAEEEGTSFE
ENKNRFTPPLFTFQKRLTNLKKNKKGIPLNCIFIDQLELTSRTYNCLKRANIHTLLDLLS
KTEEDLMRIDSFRMEDRKHIWDTLEKHLPIDLLKNKLSF
>Q7HEK3 | MATK_PICPU
MDEFHRYGKEDSSWQQCFLYPLFFQEDLYAISHDHYLDGSSSSEPMEHLSSNDQFSFLTV
KRLIGQIRQQNHSIVLFVNCDPNPLVDRKKSSYSESVLEGLTLVLEVPFSIRSKYSVEGM
NEWKSFRSIHSIFPFLEDKFPHSNYVSDTRIPYSIHPEILVRTFRRWIGDAPSLHPLRSI
LYEYRNSSESLQRSIIVVPKVNTRFFLFLWNNYVYECESILVSLLKRSSHSRSLSHGSFP
QRTHFHRKIKNIFLFSRRNSFQSIWSLKDPNIHYVRYGERSIIAIKGTHLLVKKYRYYLP
IFRQCYFHLWNEPYRVCSHQLSKNCSSSLGYFLRVRMKPLLVKTKMLDELFIADLITDEF
DPIVPIVPIIGLLSREKFCDISGRPISKLSWTSLTDDDILDRFDRIWRNLFHYYSGSFGR
DGLYRIKYILSLSCAKTLACKHKSTIRVVRKELGPELFKKSFSKERELDSPPFSSKAAAR
SQRERIWHSDIPQINPLAHSWQKIQDLKIENLFDQ

>Q7HKI2 | MATK_ACEPL
MKEYQIHLELDRSQQHNFLYPLLFREYIYALAHDHGLNRSTIPLENGGYDNKSSSLSVKR
LISRTYQRIHLSIYAKDSNPNQFIGHNNKFYSQMISEGFSVIVEIPFSLRLVAFLEGKEM
AKSQNFQSIHSIFPFFENNFSHLHYVLDVLIPYPIRPEILVRTFRYWVKDASSLHLLRFF
LHEYFNWNSLITPKKSNSIFSTRNPRFFLFLYNSHVYEYESIFFFLRNQSSHLRSTSSGP
LFERISFYGKVEDLVQVFVNDFQDNLWLFKHPIMHYVRYQGKSVLASKDMPLLMNKWKYY
LVNLWQWHFHVWSQPGRIHINHLYKDYIDFLGYLSRGRLNTLVVRSQMLENAFLIDNAMK
QFETTVPIIPLIGSLTTARFCNSLGHPISKPTWADSSDSYIIDRFMRICRNLSHYHSGSS
KKKSLYRIKYILRVSCVKSLVRKHKSTVRVFLKRLGSEFLEEFFTEEEHVLSLIFPRALF
TSRRLYRGRVWYLDIICINDLVNHDKLEIVPN
>Q7HKI3 | MATK_ACEOP
MKEYQIHLELDRSQQHNFLYPLLFREYIYALAHDHGLNRSTIPLENGGYDNKSSSLSVKR
LISRTYQRIHLSIYAKDSNPNHFIGHNNKFYSQMISEGFSVIVEIPFSLRLVAFLEGKEK
EMAKSHNFQSIHSIFPFFENNFSHLHYVLDVLIPYPIRPEILVRTFRYWVKDASSFHLLR
FFLHEYFNLNSLITPKKSNSIFSTSNPRFFLFLYNSHVYEYESIFFFLRNQSSHLRSTSS
GPLFERISFYGKVEDLVQVFVNDFQDYLWLFKHPIMHYVRYQGKSVLASKDMPLLMNKWK
YYLVNLWQWHFHVWSQPGRIHINHLYKDYIDFLGYLSRGRLNTLVVRSQMLENAFLIDNA
MKQFETTVPIIPLIGSLTTARFCNSLGHPISKPTWADSSDSYIIDRFMRICRNLSHYHSG
SSKKKSLYRIKYILRVSCVKSLVRKHKSTVRVFLKRLGSEFLEEFFTEEEHVLSLIFPRA
LFTSRRLYRGRVWYFDIICINDLVNHDKLEIVPN
>Q7HUK6 | MATK_MENLI
MEEFQRYFELDRYQQHDFLYPLIFQEYIYALAHDHGLNRSILLENAGYDKKSSLLIVKRL
ITRMYQQNHLIISANDSNQNPFLGHNKNLYSQMISEGFAVIVEIPFSLRLRSSLEGKEIV
KSHNLQSIHSIFPFLEDKFLHLNYVLDILIPYPIHLEILVQTLRHWVKDASSLHLLRFFI
HEYRNWNSLITPKRPSAHFSKRNQRLFLFLYNSHVCEYESIFIFLLTQSSHLRSTSSGVL
LERIYFYGKLEHSVEVCAKDFKAILWLFKDPFIHYLRYQGKSILTSKGTSLLMNKWKYYL
LNCWQCHFYVWSQPRRIYINQLSNHSLDFLGYLSSVRLNPSMVRSQMLENSFLIDNAIKK
FDTIVPIIPMIGSLAKAKFCNVLGHPISKPVWADLSDSDIIDRFGRICRNLSHYHSGSSK
KKTLYRIKYILRLSCARTLARKHKSTVRAFLKRVGSELLEEFFTEEEQVLSLTFPRAFST
STSRGLYRRRIWYLDIICINDLANHE
>Q7ICQ5 | IF1C_FOUSP
MKEQKWIHEGLITESLPNGMFRVRLDNEDLILGYVSGKIRRSFIRILPGDRVKIEVSRYD
STRGRIIYRLRNKDSKD
>Q7IS11 | MATK_TAXCU
MDEFQRYGNKHKSWQQCFLYPLFFREDLYTIAHDLYLDKSSSSEPTELSISNFFSFPTVK
RLIRRIRQQNDSNSIGLFRNCDPNRFINRNRNSYSELVLEGLTVILEVSLAMQSKHFIEG
MDGWKSIRSIHCIFTLMEDKFPYSNYVSDIRVPYSIHPEILVRTFRRWIRDAPSLHLLRS
ILHEWRNSFSAENLQKALVPPRENRRFSLFLWNSYVYECESFLVSLLKQFYHSRSLLYGS
FPDRTHFDKKIKHIVIFPVKISTKRIWLLKYPFIYYVRYGERSLIALKGTHLQVKRCRYH
LFNFWQYYFHLWSQPYRVCILELSKIYFYFLGHFLSFKMKTLVVRTKMLDDLLISDIIAN
EFNPIAPIRSILLYLTKERFCDISGQPISRLSWTNLSDDDILDRFDRMCRNIFHYYSGSI
NKDGLYYIKYILLLPCAKTLACKHKSTIRVVREESGSELFTKSFSKEREFIYSSFSKTCS
QRERNWNSDIIQINILVNYWQKIHNKQIEK
>Q7IZ70 | MATK_RORPA

MXXXXGYLEFDGARQQSFLYPLFFREYIYVLAYDHGLNRLNRNRSIFFENVDYEKKYSSL
IVKRLILRMYEQNRLIIPSKDLNQNHFFGHTSLFYYQMISVLFAVIVEIPFSLRLGSSFE
GKQFKKSYNLQSIHSIFPFLEDKLSHFNYVLDVVIPYPIHLEILVQTLRYRVKDASSLHF
FRFCLYEYCNWKDFSIKKKSILNPRFFLFLYNSHVCEYESIFFFLRKRSSHLRSTSYEVL
FERILFYGKIQHFLKVFINSFPAILGLLKDPFIHYVRYHGRCILATKDTPLLMNKWKYYF
VNLCQCYFSVWFQSQKVNINQLSKDNLEFLGYLSSLRLNPLVVRSQMLENSFLIDNVRIK
LDSKIPISSIIGSLAKDKFCNVLGHPISKAVWTDSSDSDILNRFVRISRNISHYYSGSSN
KKNLYRIKYILRLCCVKTLARKHKSTVRAFLKRLGSGLLEEFLTGEDQVLSLIFPRSYYA
SKRLYRVRIWYLDILYLNDLVNHE
>Q7J1C8 | MATK_LIRTU
MEELQGYLEIDRSRQQHFLYPLLFQEYIYALAHDHGLNGSIFYEPMENLGYDNKSSSLIV
KRLITRMHQQNHLIISVNDSNENGFVGHNKSFYSQMVSEGFAVIMEIPFSLRLVSSLEEK
EIAKSHNLRSIHSIFPFFEDKLSHLNHVSDILIPHPIHLEILVQTLHCWIQDAPSLHLLR
FFLHEYRNSNSLITPKKSISLFSKENQRFFLFLYNSHVYECESVLVFLRKQSSHLRSTSS
GTFLERTHFYGKIEHLVVVLRNDFQKTLWLFKDPFMHYVRYQGKYILASKGTHLLMKKWK
SHLVNFWQCHFYLWSRPDRIHINQLYNHSFYFLGYLSSVRLNTSAVRIQMLENSFLIDTS
INKFETLVPIISLIGSVAKAKFCNVSGHPISKSVRADSSDSDIINRFGRIYRNLSHYHSG
SSKKQTLYRIKYILRLSCARTLARKHKSTVRAFLKRLGSEFLEEFLTEEEQVLSLIFQRN
SFPSYRSHRERIWYLDIIRINDLANHS
>Q7J5Z8 | MATK_PAEPE
MEKSQGYLELDKSWRHDFLYPLIFQEYIYALAHEQGLNRSILLENTDHDNKYSSLIVKRL
ITLIHQQNHFLIFDNDSNQNPFWKHNNNLYSQTISEGFVIIVEIPFSPRFVDSLEEKKKI
LKSNNLRSIHSIFPFLEDQILHLNFVANILIPYPIHLEIVVQSLRYRVKDASSLHLLRFF
LFTLNKSISSFSKRNQRFFLFLYNSHVYEYESTFLFLRNKTSHLRSTSSGAFLERIFFYG
KIKHLIEVFANDFQAILWLFKDPFMHYVRYQGKSILASKRTSLRMNKWKYYLVNFWQCQF
YVWSQPGRVSINQLSNHSLDFLGYLSSVRRNPLAVRSQMLENSFLTDNAIKKFDIIVLLI
SLIGSLAKAKFCNVLGHPLSKPARADSSDSDIIERFVRICRNLSHYHSGSSKKKSLYRIK
YILRLSCARTLARKHKTTVRSFLKRLGSELLEEFLTEDGQVISLIFPRTSSTSWRLYRGG
IWYLDITCINDLANHE
>Q7J5Z9 | MATK_PAEOF
MEKSQGYLELDKSWRHDFLYPLIFQEYIYALAHEQGLNRSILVENTDHDNKYSSLIVKRL
ITLIYQQNHFLIFDNDSNQNPFWKHNNNLYSQTISEGFVIIVEIPFSPRFVDSLEEKKKI
LKSNNLRSIHSIFPFLEDQILHLNFVANILIPYPIHLEIVVQSLRYRVKDASSLHLLRFF
LFTLNKSISSFSKRNQRFFLFLYNSHVYEYESTFLFLRNKTSHLRSTSSGAFLERIFFYG
KIKHLIEVFANDFQAILWLFKDPFMHYVRYQGKSILASKRTSLRMNKWKYYLVNFWQCQF
YVWSQPGRVSINQLSNHSLDFLGYLSSVRRNPLAVRSQMLENSFLTDNAIKKFDIIVLLI
SLIGSLAKAKFCNVLGHPLSKPARADSSDSDIIERFVRICRNLSHYHSGSSKKKSLYRIK
YILRLSCARTLARKHKTTVRSFLKRLGSELLEEFLTEDGQVISLIFPRTSSTSWRLYRGG
IWYLDITCINDLANHE
>Q7JEV7 | MATK_TRIGN
MEELQLQGYLEKDGSRQQNFLYPLIFQEYIYTLAHDHGLNSSIFDEPMEIVGLGYDNKSS
SVLVKRLITRMYQQNSLIYSMNDFNQNRFVGHNNSFYSNFDSKMVSEGFAVIVEIPFSLR
LVPSSEEIPKSQNLRSIHSIFPFLEDKLSYLNYVLDILIPYPIHLEILVQILQCWIQDVP
SLHFLRLFLHEFHNWNNLITPTKSISVFSKENKRLFRILYNSYVSEYEFVFVFLRKQSYY

LRSTSSRAFLERTHFYVKIEHLIDVCHNHFQKILWFFKDSFMHYVRYKGKAILASRGTYL
LIKKWKCYLVNFWQYHFHFWSKPYRIHINPFSNYSFYFLGYISSVRINPSAVKNQMLENF
YLVDTLTQKFDTIVPVIPLIGSLSKAKFCTILGHPISKPIWAELSDSDIIDRFGRICRNL
SHYHSGSSKKQSLYRIKYILRLSCARTLARKHKSTVRNLLQRLGSGLLEEFFTEEEQVIS
PIFPKTTLFPLHGSHKERIWYLNIIRINDLANYLDWS
>Q7JEV9 | MATK_TRIFE
MEELQLQGYLEKDGSRQQNFLYPLIFQEYIYTLAHDHGLNSSIFYEPMEIVGLGYDNKSS
SVLVKRLITQMYQQNSLIYSMNDFNQNRFVGHNNSFYSNFYSQMVSEGFAVIVEIPFSLR
LVPSSEEIQIPKSQNLRSIHSIFPFLEDKLSHLNYVLDILIPYPIHLEILVQILQCWIQD
VPSLHFLRFFLHEFHNWNNLNLITPTKSISVFSKENKRLFWILYNSYVSEYEFLFVFLRK
QSYYLRSTSSRAFLERTHFYVKIEHLIDVCHNHFQKILWFFKDSFMHYVRYKGKAILASR
GTYLLIKKWKCYLVNFWQYNFHFWSKPYRIHINPFSNYSFYFLGYISSVLINPSAVKNQM
LENFYLVDTLTQKFDTIVPVIPLIGSLSKAKFCTILGHPISKPIWAELSDSDIMDRFGRI
CRNLSHYHSGSSKKQSLYRIKYILRLSCARTLARKHKSTVRNLLQRLGSGLLEEFFTEEE
QVISPIFPKTTLFPLHGSHRERIWYLDIIRINDLANYLDWS
>Q7JEW0 | MATK_TRIEE
MEELQLQGYLEKDGSRQQNFLYPLIFQEYIYTLAHDHGLNSSIFYEPMEIVGLGYDNKSS
SVLVKRLITQMYQQNSLIYSMNDFNQNRFVGHNNSFYSNFYSQMVSEGFAVIVEIPFSLR
LVPSSEEIQIPKSQNLRSIHSIFPFLEDKLSHLNYVLDILIPYPIHLEILVQILQCWIQD
VPSLHFLRFFLHEFHNWNNLNLITPTKSISVFSKENKRLFWILYNSYVSEYEFLFVFLRK
QSYYLRSTSSRAFLERTHFYVKIEHLIDVCHNHFQKILWFFKDSFMHYVRYKGKAILASR
GTYLLIKKWKCYLVNFWQYNFHFWSKPYRIHINPFSNYSFYFLGYISSVLINPSAVKNQM
LENFYLVDTLTQKFDTIVPVIPLIGSLSKAKFCTILGHPISKPIWAELSDSDIMDRFGRI
CRNLSHYHSGSSKKQSLYRIKYILRLSCARTLARKHKSTVRNLLQRLGSGLLEEFFTEEE
QVISPIFPKTTLFPLHGSHRERIWYLDIIRINDLANYLDWS
>Q7JEW7 | MATK_TRICT
MEELQLQGYLEKDGSRQQNFLYPLIFQEYIYTLAHDHGLNSSIFYEPMEIVGLGYDNKSS
SVLVKRLITRMYQQNSLIYSMNDFNQNRFVGHNNSFYSNFDSQMVSEGFAVIVEIPFSLR
LVPSSEEIQIPKSQNLRSIHSIFPFLEDKLSHLNYVLDILIPYPIHLEILVQILQCWIQD
VPSLHFLRFFLHEFHNWNNLNLITPTKSISVFSKENKRLFWILYNSYVSEYEFLFVFLRK
QSYYLRSTSSRAFLERTHFYVKIEHLIDVCHNHFQKILWFFKDSFMHYVRYKGKAILASR
GTYLLIKKWKCYLVNFWQYNFHFWSKPYRIHINPFSNYSFYFLGYISSVLINPSAVKNQM
LENFYLVDTLTQKFDTIVPVIPLIGSLSKAKFCTILGHPISKPIWAELSDSDIMDRFGRI
CRNLSHYHSGSSKKQSLYRIKYILRLSCARTLARKHKSTVRNLLQRLGSGLLEEFFTEEE
QVISPIFPKTTLFPLHGSHRERIWYLDIIRINDLANYLDWS
>Q7XE48 | SSY21_ORYSJ
MAAAAVSSLLAPSGSCYSPGCHSCWGPGPGGGRRLPSPRRRPITAAARPTWAVPRRSRLE
WGRVEAQNSGARTSCRAALQWLSSTARSHVNVGYGSPLVFPGLTKPGSSRCLCVVGMVGN
AGNQVGDDSDDGIKVTNEKLRAVIRKSKEVLEIHRNLLEKISASERKKITSIIEDSSIYN
EQDPFGQRDSSFYHLDEVPDDDEFSYDLQMYLDRRPDQSEVVATQDYEAQLSQISEMGQS
VAEGTSDDPSASAAVDLINIILVAAECAPWSKTGGLGDVAGALPKALARRGHRVMVVVPM
YKNYAEPQQLGEPRRYQVAGQDMEVIYYHAYIDGVDFVFIDNPIFHHVENDIYGGDRTDI
LKRMVLLCKAAIEVPWYVPCGGYCYGDGNLVFLANDWHTALLPVYLKAYYHDNGFMIYAR
SVLVIHNIAHQGRGPLDDFSYLDLPVDYMDLFKLYDPFGGDHLNIFAAGIKAADRLLTVS

HGYAWELKTAEGGWGLHGIINESDWKFQGIVNGIDTTDWNPRCDIHLKSDGYTNYSLETV
QAGKQQCKAALQKELGLPVRGDVPVIAFIGRLDHQKGVDLIAEAMPWIAGQDVQLIMLGT
GRQDLEDTLRRLESQHYDRVRGWVGFSIRLAHRMTAGADILLMPSRFEPCGLNQLYAMMY
GTVPVVHAVGGLRDTVEHYNPYEESGLGWTFEKAEANRLIDALGHCLNTYRNYRTSWEGL
QKRGMMQDLSWDNAAKLYEEVLLAAKYQW
>Q7YJG1 | MATK_ANTLI
MEELQGYLEKDKSWQQHLLYPLLFQEYIYALAHDHSLSGSIFYEPVEVFGYDKKSSLALV
KRLNTRIYQQNFLISSVNGSNQKRLVGXNNFFXSLFXSQMIIESFAIFXEILFLRALVSF
FGEKKVLKYPNLPSIHSIFPFLEXKLPHLNYVSDILIPHPIHMXILVQILQCWIQDVPFL
HSLRFFLHEYHNWNSLLITHKKSICLFSKENKRLFRFLYNSYVSEFEFLLVFFRKQSSYL
RLRSSGTFLERTHFYGKIEHFQIXHFAFYSSMSXLFPVVXLKXPFMHYVRYQGKALLASK
GTHLVMKKWKYHFVTLWQYYFHFWSQLYRIRINXLSNYSFYFLGYLSSVLINFSAVRNQM
LENSFLIDIITKKFDSIIPVILLIESLSKAQFCTVSGHPISKPIWADLSDSDILDRFGWI
CRNLSHYHSGSSKKQDLYRIKYILRLSCARTLARKHKSTVRTFLRRLGPGLLEEFFTEEE
RVLSLIFPKTISFILHGSHKERIWYLDIIRMNDLVNYSXLX
>Q7YJG2 | MATK_KNIUV
MEELQRYLEKGRSRQQHFLYPLLFQEYIYALAHDHGLNSSIFYEPVEIISYDNKSSLALV
KRLITRIYQQNYLISSVNDSNQNQFLGHNAFFYSHFFSQIISESFAIIVEIPFSLRLVSF
FEEKEIPIYHNLRSIHSIFPFLEGKLSHLNYVSDILIPHPIHMEILVQTLQCRVQDVPFL
HLLRFFLHEYHNWNSLLITQNKSIYFFSKENKRIFRLLYNSYVFECEFLLVFFRKQSYYL
RLTSSGTFLERTHFYRKIEHLPIEHFFVVCRNYFHRTRWFFKNPFMHYVRYQRKAILASR
GTHFLMKKWKYHFVNFWQYYFHFWSRPYRIHINHLSNYSFYFLGYLSSLLINSSAVRNQM
LENSFLIDTVTNKFDTIVPVILLIRSLSKAKFCTVSGHPISKPIWADLSDSDIIDRFGRI
CRNLSHYHSGSSKKQDLYRIKYILRLSCARTLARKHKSTVRTFLRRLGSGLLEEFFTEEE
QVLSLIFPKRTPFTLHGSHRERIWYLDIICINDLVNHSXLS
>Q7YJT3 | NU4C_CALFE
MSYFPWLTVIVVLPISAGSSIFFVPYRGNKNKVIRWYTICICLLEILLTTYAFCYHFQSD
DPLIQLEEAYKWIHIFDFHWRPGIDGLSIGPILLTGFITTLATLAARPVTRDSRLFHFLM
LAMYSGQVGSFSSRDLLLFFLMWELELIPVYLLLSMWGGKKRMYSATKFILYTAGGSIFL
LMGVSGMGLYGSNEPTLNFETLANQSYPLGLEIIFYFGFLIAYAVKSPIIPLHTWLPDTH
GEAHYSTCMLLAGILLKMGAYGLVRINMELLPHAHSIFSPWLVIVGAIQIIYAASTSFGQ
RNFKKRIAYSSVSHMGFTLIGIGSITDTGLNGAILQIISHGFIGAALFFLAGTSYDRIRL
VYLDEMGGIAIPMPKIFTMFSSFSMASLALPGMSGFVAELVVFFGIITSSKYLLMPKIVI
SFVMAIGMILTPIYSLSMLRRMFYGYKLFNVPDFYFLDSGPRELFVSICIFLPVVGIGIY
PDFVLSLSVDRVEAILSNYFHK
>Q7YJT7 | RK2_CALFE
MAIHLYKTSTPSTRNGAVDGQVKSNPRNNLIYGQHHCSKGRNARGIITARHRGGGHKRLY
RKIDFRRNEKDISGRIVTIEYDPNRNAYICLIHYGDGEKRYILHPRGAIIGDTIVSGTEV
PISMGNALPLTDMPLGTAIHNIEITLGKGGQLARAAGAVAKLIAKEGKSATLRLPSGEVR
LISKNCSATVGQVGNVGVNQKSLGRAGSKRWLGKRPVVRGVVMNPVDHPHGGGEGRAPIG
RKKPTTPWGYPALGRRSRKRNKYSDSFILRRRK
>Q7YJU1 | RK16_CALFE
MLSPKRTRFRKQHRGRMKGISYRGNHICFGRYALQALEPSWITSRQIEAGRRAMTRYARR
GGKIWVRIFPDKPVTVRPTETRMGSGKGSPEYWVSVVKPGRILYEMGGVSETVARAAISI

AACKMPIRTQFIIAR
>Q7YJU2 | RK14_CALFE
MIQPQTHLNVADNSGARELMCIRIIGASNRRYAHIGDVIVAVIKEAVPNMPLERSEVIRA
VIVRTCKELKRDNGMIIRYDDNAAVVIDQEGNPKGTRVFGAIARELRKLNFTKIVSLAPE
VL
>Q7YJU6 | RPOA_CALFE
MVREEVAVSTRTLQWKCVESRTDSNRLYYGRFVLSPLMKGQADTIGIAMRKALLGEIEGT
CITRAKSEKVPHEYSTIVGIEESVHEILMNLKEIVLKSNLYGTRDASICVRGPKYVTAQD
IISPPSVEIVDTTQHIASLTEPINLCIELQIERDRGYRMKAPNNYQDGSYPIDAVSMPVR
NANHSIHSYGSGNEKQEILFLEIWTNGSLTPKEALHEASRNLIDLFIPFLHAEEQDIHLN
LEDNQNRFTVSPFTLHDRLGNIRKNKKEIALKCIFIDQSELPTRTYNCLKRSNIHTLLDL
LNNSQEDLMRIEHFRIEDIKQILDILQKHFGIDLPKNKRF
>Q7YJV4 | RR18_CALFE
MDKSKRPFRKSKRSFRRRLPPIGSGDRIDYRNMSLISQFISEQGKILSRRVNRLTLKQQR
LITIAIKQARILSSLPFLNNEKQFERTESIPRTTGPRTRK
>Q7YJX8 | RPOB_CALFE
MPTIPGFSQIQFEGFCRFIDQGLTEELHKFPKIEDTDQEIEFQLFVETYQLVEPLIKERD
AVYESLTYSSELYVSAGLIWKTGRDMQEQTIFIGNIPLMNSLGNSIVSGIYRIVINQILQ
SPGIYYRSELDHNGISVYTGTIISDWGGRPELEIDRKARIWARVSRKQKISILVPSSAMG
SNLREILDNVCYPEIFLSFPNDKEKKKIGSRENAILEFYKQFACVGGDPVFSESLCKELQ
KKFFQQRCELGRIGRRNMNRRLNIDIPQNNTFLLPRDVLAVVDHLIGMKFGMGTLDDMNH
LKNKRIRSVADLLQDQFGLALVRLENAVRGTICGAIRHKLIPTPHNLVTSTPLTTTYESF
FGLHPLSQVLDRTNPLTQIVHGRKSSYLGPGGLTGRTASFRIRDIHPSHYGRICPIDTSE
GINVGLIGSLAIHARIGHWGSIESPFYEISEKSKEIVYLPPSRDEYYMVAAGNSLALNRG
IQEEQVVPARYRQEFLTIAWEQIHLRSIFPFQYFSIGASLIPFIEHNDANRALMSSNMQR
QAVPLSRSEKCIVGTGLEGQAALDSGVSAIAEHEGKIIYTDTDKIVLSGNRDTISIPLVM
YQRSNKNTCMHQKPRVPRGKCIKKGQILADGAATVGGELALGKNVLVAHMPWEGYNSEDA
VLISERLIYGDIYTSFHIRKYEIQTHVTSQGPERITNEIPHLEAHLLRNLDKNGIVMLGS
WIERGDILVGKLTPQAAKESSYAPEDRLLRAILGIQVSTAKETCLKLPIGGRGRVIDVRW
IQKKGGSSYNPETIRVSILQKREIKVGDKVAGRHGNKGIVSKILPRQDMPYLQDGTPVDM
VFNPLGVPSRMNVGQMFECSLGLAGYLLDKHYRIAPFDERYEQEASRKLVFPELYSASKQ
TVNPWVFEPEYPGKSRIFDGRTGDPFEQPVIIGKSYILKLIHQVDDKIHGRSSGHYALVT
QQPLRGRAKQGGQRVGEMEVWALEGFGVAHISQEMLTYKSDHIRARQEVLGATIIGGTIP
KPEDAPESFRLLVRELRSLALELNHFLVSEKNFQINRKEA
>Q7YJY5 | PSBK_CALFE
MLNIFSLICLNSALHSSSFFFAKLPEAYAFFNPIVDVMPVIPVLFFLLALVWQAAVSFR
>Q7YKM5 | MATK_PROLU
MEEIQRYLQLERSQQHDFLYPLIFQEYIYTFAHDHGFSRSIWSKNRGYDNKSSLLIVKRL
ITRMYQQNHFLISLNDSNQNPFWARNKNLYSQIISEGFAFIVEIPFSIRLISCLEGKKIV
KSQNLRSIHSIFPFLEXNFSHLNFVLDILIPHPVHVEILDASSLHLLRFFLNEYCNWNSL
ITPKKASSSFSKINQRLFLFLYNSHVCEYESIFVFLRNQSSHLRSTSSGVLLERIYFYGK
IEHLVNVFVTVKDFQANLWLVKEPCMHYIRYQRKSILASKGTSLFMNKWKCYLVTFWQWH
FSLWFHPSRIYINQLSNHSLEFLGYLSSVRMNPSVVRSQILENAFLINNAIKKFDTLVPI
IPLIASLAKAKFCNVLGHPISKPVRADLSDSNIIDRFGCICRNLSHYHSGSSKKKSLYRI

KYILRLSCARTLARKHKSTVRAFLKRLGSELLEQFLMSEEDVLFXTFXKASSTLRGVNNS
RIWYVDIISINDLAXHKSKF
>Q7YKQ2 | MATK_IBILU
MEEIQRYLQLERSQQHDFLYPLIFQEYIYTFAHDRGFSRSILSENPGYDNKSSLLIVKRL
ITRMYQQNHFIISPNDSNQNPFWARNKNLYSQIISEGFAFIVEIPFSIRLISCLEGKKIV
KSQNLRSIHSIFPFLEDNFSHLNFVLDILIPHSVHVEILVQTLRYWVKDASSLHLLRFFL
NEYCNWNSLITPKKASSSFSKRNQRLFLFLYNSHVCEYESIFVFLRNQSSHLRSTSSGVL
LERIYFYRKIERLVNVFVKVKDFQANLWFVKEPCMHYIRYQRKSILASKGTSLFMNKWKC
YFVTFWQWHFSLWFHPSRIYINQLSNHSLEFLGYLSSVRINPSVVRSQILENAFLINNAI
KKFDTLVPIIPLIASLAKAKFCNVLGHPVSKPGRADLSDSNIIDRFGCICRNLSHYHSGS
SKKKSLYRIKYILRLSCARTLARKHKSTVRAFLKRLGSEFLEQFLMSEEDVLFLTFQKAS
STLRGVYRSRIWYLDIISINDLANHKSKF
>Q7YKQ9 | MATK_AVIGE
MEEIKRYLQRNRSHHQDFLYPLIFQEYIYAFAHYRGFSQSILSENPGYDNKSSLLLVKRL
ITRMYQQNHFLIFPNDSNQNPFFARNKNLYSQIIPEGFAFIVEIPFSLRLISCLEGKKIV
KSQNLRSIHSIFPFLENNFSHFHFVLDVLIPHPVHVEILVQTLRYWLKDASSLHLLRFFL
NEYCNWNSCILPKKAGSSFSKRTQRLFLFVYNSHVWEYESIFVFLRNQSAHLRSISSGVL
LERIYFYGKMERLVNVFVKVKDFRSNPGLIKEPCMHYIRYQRKSILASKGTSLFMNKWKC
YLVTLWQWHFSLWFHPRRIYINQLANHSLEFLGYLSSVRMNPSVIRSQILENAFLINNAI
KKFDTLVPIIPLIMSLAKAQFCTVLGHPISKPVWADLSDSNIIDRFRRISRNLSHYHSGS
STKKSLYRIKYILRLSCVRTLARKHKRTVCVFLKRSGSELLEEFLMSEEGVPFFTFQKGF
STLRGIYRSRVWYLDIFSINDLANHK
>Q7YKX3 | RR11_CHLRE
MAKQTRKLTTNKTKKKIFRGVVHIQAGHHNTIVTITNIRGEVLCWSSAGACGFRGKRKST
SFAAKKAAETVARKSRDFTMKAAKILVTGPGQGRESAIREIFKAGIKVSVIREKTGIPHN
GCRPPKKRSV
>Q7YM14 | MATK_QUERU
MEEFQGYLELDRFQQHDFLYPLIFREYSYALAHGHGLNRYMLLENIGYDNKSSLLIVKRL
ITTMYQQNYLKISANDSKQNPFFGYNKNLHSKILSEGFAIIVEIPFYLRLISSLEGAEIV
RFYNLRSIHSIFPFLEEKFPHLNYSADILIPYPAHLEILVQTLRYRVKDASYLHLLRFFL
HEYSNCNSLIITNKSISIFSKSNPRFFLFLYNSYICEYESIFLFLRNQPSHLRLTSSGVL
FERLCLYRKIEHFAEVFSNDFPVIPCFLKDPFMHYVRYQGKSILASKDTPLLMNKWKSYL
VNLWQCHFDVWSHAASIRINQLSKHSLDFLSYFSSVRRNPAVVRNQMLENSFLLNNAPNK
LDTMVPIIPLIGSLAKAKFCNAVGHPISKLTRADLSDFEIINRFLHICRNLSHYYSGSSK
KKNMYRIKYILRLSCVKTLARKHKSTARAFLKRVDLEFFQEFFTEEGGFISLIFPRASFA
LRRLYSGRVWYLDIIFINGLSNHE
>Q7YM28 | MATK_GALBE
MEELQGYLEIDRSRQQHFLYPLLFQEYIYALAHDHGLNGSIFYEPMENLGYDNKSSSLIA
KRLITRMHQQNHLIISISVNDSKQNRFVGHNKNLYSQMVSEGFAVIVEIPFSLRLVSSLE
EKEIEKSHNLRSIHSIFPFFEDKLSHLNHVSDILIPHPIHLEILVQTLRCWVQDAPSLHL
LRFFLHEYGNSNSLITPKKSISFFSKENQRFFLFLYNSHVYECESVFVFLRKPFSHLRST
SFGAFLERIHFYGKTEHLVVVPRNYFQKTLWLFKDPFMHYARYQGKSILASKGTHLLMKK
WKSHLVHFWQYHFYLWSQPDRIHINQLCNHSFYFLGYFSSVRLNLSVVRSEMLENSFLID
TSIKKFETLAPIIPLIGSLAKAKFCNVSGHPISKSVWANSSDSDILNRFGRIYRNLSHYH

SGSSKKQILYRIKYILRLSCARTLARKHKSTVRTFLKRLGSEFLEEFLTEEEQVLSLIFP
RTSFPFYRSHRERIWYLDIIRINDLANHE
>Q7YM30 | MATK_CANOD
MEELQRHFEIDGSRQQRFIYPFLFQEYIYALAHYYALNGSIFYETVENLGYDKKSSSLIV
KRLITRMHRQNRLIISINDSNQNRFIGHNKNLYTQTVSEGFAVIMEIPFSLRLVFSLEEK
EISKSHNLRSIHSLFPFFEDKLSHLNHVSHILIPHPAHLEILVQILHCWIQDAPSLHLLR
FFLHDYHNSKSINAQKKSIFVCSKENRRFFSFIYNFHVYESELMFVFLRKQSSHLRSTSF
GTVLERTHFYGKIEHLVVVLRNDFQKTLWLFKDPFMHYVRYQGKSILASKGTHLRMKKWK
SYLVLFWQSHFYLWSQPERIRINQLYNHSFYFLGYLSGVRRNPSVVRSQMLENSFLIETS
IKKFETLVPITPLIGSLAKAKFCNVSGHPISKPSWADLSDSDIINRFGRIYRNFSHYHSG
SSKKQTLYQIKYILRLSCARTLARKHKSTVRAFLKRLGSKFLEEFLTEEEQVLSLIFPRT
PSPSYRSHRERIWYLDIIYINILTNHV
>Q7YMG2 | MATK_PINUN
MDEFHRCGKEDSFWQQCFLYPLFFQEDLYAISHDHYLDVSSSSRPMEHLSSNDQLSFLTV
KRLIGQIRQQNHSIVLFVNCDPNPLADRKKSFYSESVLEALTLVLEVPFSIWSKSSVEGM
NECKSFRSIHSIFPFLEDKFPHSNSILDARIPYSIHPEILVRTFRRWIRDAPSLHPLRSV
LYDYRNSPENLQRSIIVVPRVNTRFFLFLLNYYVCECESILFSRLKRSSHSRSLSHGSFP
QRTHFHRSIKHIIIFSRRNSLKSIWSLKDPKIHYVRYGERPIIAIKGADLLVKKCRYYLL
IFRQFYFHLWSEPYRVCSHQLSKNCSSSPGYFLRVRMNPLLVRTKTLDELFIPVLITNEM
DPIVPIVPIIGLLATEKFCDISGRPISKLSWTSLTDDDILDRFDQIWRNLFHYYSGSFDR
DGLYRIKYILLLSCAKTLACKHKSTIRVVRKELGPELFKKSFSKEREFDSLPFSSKAAAR
SQRERIWHSDIPQINPLANSWQKIQDLKIENLFDQ
>Q7YMV6 | MATK_CLELI
MEELQGYLKIDRSRERDFLYPLLFQEYIYALAHDHGLNKSILYEPMENLGYDKKYSLIIV
KRLITRMYQQKHLIIFTNDSNPNFFFGHNKNLDSQMISEGVAVIVELPFSLRLVSSPESK
EIDKSMTTLRSIHSIFPFLEDKLLHLNHVLDILIPYPIHLELLVQTLRSWIQDAPFLHLL
RFFLYKYHNWNSLITQKTKMILFFSKENQRFFLFLYNFHVYESESIFVFLRKQSYHLRST
SSRAFLDRTHFYRKIEHFLVDFRNDFHTILWLFKDPFIQYFRFQGKSILSSKGTPLLMKK
WKYYLVNLWECHFYFWSQPDRIHINQLSNHFIDFLGYLSSVRPTPSAVRSQMLEKSFIID
IVIKKFDTIVPIIPMIGSLAKAKFCNFSGHPISKPAWADSSDSDIIDRFGRICRNLSHYY
SGSSKKKSLYRIKYILRLSCARTLARKHKSTVRSFLKRLGSEFLEEFLMEEEQVLSFILP
RISYFSKRLYKERIWYFDIIRINDLTNLS
>Q7YMV7 | MATK_CLELA
MEELQGYLKIDRSRERDFLYPLLFQEYIYALAHDHGLNKSILYEPMENLGYDKKYSLIIV
KRLITRMYQQKHLIIFTNDSNTNFFFGHNKNLDSQMISEGVAVIVELPFSLRLVSSPESK
EIDKSMTTLRSIHSIFPFLEDKLLHLNHVLDILIPYPIHLELLVQTLRSWIQDAPFLHLL
RFFLYKYHNWNSLITQKTKMILFFSKENQRFFLFLYNFHVYESESIFVFLRKQSYHLRST
SSRAFLDRTHFYRKIEHFLVDFRNDFHTILWLFKDPFIQYFRFQGKSILSSKGTPLLMKK
WKYYLVNLWECHFYFWSQPDRIHINQLSNHFIDFLGYLSSVRPTPSAVRSQMLEKSFIID
IVIKKFDTIVPIIPLIGSLAKAKFCNFSGHPISKPAWADSSDSDIIDRFGRICRNLSHYY
SGSSKKKSLYRIKYILRLSCARTLARKHKSTVRSFLKRLGSEFLEEFLMEEEQVLSFILP
RISYFSKRLYKERIWYFDIIRINDLTNLS
>Q859W3 | MATK_FAGTA
MEEFQGYLELYRSWQDNFLYPLILQESIYALVHNQDLGLNGSILLSKKKGYDTKYSLLVV

KRLVIRMYQQNFVILSLNDSNKNEFFVPNKNLYSQRISEGFAVLAEIPFSMRVMSSLKGK
ERKQYQNLRSIHSIFPFLEDKFSRLNHVLDILIPHPVHTKILVQTIRYCVKDISCLHLLQ
LLLYEYCNNGITLKGSVSNLSKNKNQRFLLFLYNSYVCECESIFVFLRNQSSHLRSTSYG
AFLARVYFYLKLEHFLKVFTKHFRVILQFFKDPFMHYVRYQGKWILASRGTFLLMTKLKY
YFVNFWQCNFYLWLQTRRIYINKSLNQPIDFIGFLLSVRLNPSVVRSQMLENSFLIYNGI
KKFETLVPTMSLIGSLAKAKFCNVLGHPISKPAWADLSDSDIIRRFGRMCRNLSHYYSGS
SKKGGLYRIKYILRLSCARTLARKHKSTVRAFMKRLGSEFFEEFFFKEEKVISLILSRDS
SISRRLYRGPIWYFDIFCIHDLASHND
>Q85AI6 | RR7_ADICA
MNEIRNIESDPIYRNRLANMLVDRILKNGKKSLAYQIFYQAMKRIRQKTNRNPLSVLRQA
VRGVTPDVVTETKRVGGSTYRVPIEVVPAKGKALAIRWLLIACRKRSGRSMALRLSDELI
DAARNSGSAIRKKEETHKVAEANKAFAHFR
>Q85AM2 | RR8_ANTFO
MGNDIIANMITAIRNANLGRAETVEVPATNLTRNIAKILLREGFIESFSEHQENKNSFLI
FILKYRGKKRKPYITTLRRISKPGLRIYSNYQEIPRVLGGTGIVILSTSRGIITDREARQ
KQVGGEILCYVW
>Q85AW7 | RR2_ANTFO
MKQKYWNINLEEMMEAGIHFGHQAAKWNPKMKPYIFTERRGIHILNVTKTARLLSEACDF
VANAASKGKQFLIVGTKYQAADLVASAALKARCHYVNQKWLGGMLTNWSTIEKRLRKFED
LQTKKNMGALDELPKKEAANLERQLAQLQKYLGGIRYMTNLPDIVIIVDQQKELTAIREC
INLNIPTICLVDTDCDPDLTDISIPANDDARASIRWVLDKLTSAIQEGRCNSTKI
>Q85BB6 | PSBK_ANTFO
MFYIHLENTFDLSSTILVKLPEAYAIFDPIVDVMPIIPLFFFLLAFVWQASVSFR
>Q85BJ7 | RK23_ANTFO
MDKVKYPVLTEKTIRLLERNQYCFDVDLKASKTEIKQWIQDFFKVKVVAMNSHRPPKKKK
RIGPVLGYPVRYKRMIITLESNYSIPLFLNK
>Q85BR7 | MATK_HORML
MEKFEGYSEKHKSRQQYFVYPLLFQEYIYAFAHDYGLNGSEPVEIVSCNNKKFSSLLVKR
LIIRMYQQNFLDNSVNHPNQDRLLDYKNYFYSEFYSQILSEGFAIVVEIPFSLRELSCPK
EKEIPKFQNLRSIHSIFPFLEDKFLHLDYLSHIEIPYPIHLEILVQLLQYRIQDVPSLHL
LRFFLNYYSNWNSFITSMKSIFFFQKENKRLFKFLYNSYVSEYEFFLLFLRKQSSCLPLA
SSGTFLERIHFSRKMEHFGIMYPGFSRKTLWFFMDPLMHYVRYQGKAILASKGSFFLKKK
WKCYLINFWQYYFFFWTQPRRIHINQLANSCFDFMGYLSSVPKSPLLVRNQMLENSFLID
TRMKKFDTIVPATLLIGYLSKAQFCTGSGHPISKPIWTDLSDWDILDRFGRICRNLFHYH
SGSSKKRTLYRLKYILRLSCARTLARKHKSTVRTFMQRLGSAFLEEFFTEEEQVFSLMFT
KTTLFSFSGSHTERIWYLDIIRINDLVNPLN
>Q85C28 | RK32_ANTFO
MAVPKKRTSKSKRKIRKTVWREKANKAAKKAFSLARLILSGRSKSFCYTVNNKSSESSES
TSIDESDDS
>Q85C71 | RPOC2_ANTFO
MAESAKLLFYNKVIDGTAIKQFIGRLVAHFGITYTAHILDQLKTLGFQQATYAATSLGID
DLLTAPSKGWLIQDAEHHSYTLEKHHRYGNVHAVEKLRQLIETWYATSEYLKQEMNPNFR
MTDPLNPVHMMSFSGARGSASQVHQLVGMRGLVSDPQGQIIDLPIKSNFREGLSLTEYII
SCYGARKGVVDTAVRTSDAGYLTRRLVEVVQHIVVRKTDCNTFRGISLNSTEDKKKNLQI

FTQQKLVGRVLADNLYINARCLAIRNQDINTNLIEKLITLKTPLISIRSPLTCKSMLWIC
QLCYGWSLTHYGDLVELGEAVGIIAGQSIGEPGTQLTLRTFHTGGVFTGDIAEHVRTPFN
GTIEFDTDLVYPTRTRHGHPAWVCRTDLAVTIKSQNKIHNLIIPSQSLLLIQNNQYVESK
QLIAEVHAKVSPFKEKVQKYIYCNIEGEMHWSKRVRHASKYLHSNVHLIFNTGHLWILSG
SSHEDKTSSMFFKNQDQINTKFFISPRKILFMKKTNQVNSKNLDPYLEKEMETLNYSNLY
LGILNKSRNFVYPSIILHDYEVKRIYDEKKGENFLLLEERHGEKKTQIFRRFVLNIPKSG
ILENKDIFAISNDPGYGIQSPGIIKYGTIKVNPIKGKGETFKNRETKILRLRPRYQVIEP
GNFFPIPEEVHILYESFPPILVRNDSLIKKNTQITSDIRSQVGGLVRIRRKMNDSYEVKV
LPGRVYHPEERRNISKQNDILVPPGEEISNKFQSENWLYLEWITPPKEKPFIFIRPAIEF
IVPEETDLAETFTLNSQKKQEILKVKKIRYLLYEDGEEVEVTNKTGIQLIQTGLVLDWKE
DSSIKKVYASLTEIETNGLSKKFLQISLIEHPIFEIEKKKNNVNLKYLFTNEINYSSHSF
NYENGLFNGYRGIIRISSNENQEDKSLPILSPFDFVQIFLSKNSEEYTPEMKDEGNFNLD
TNSIFYAKSFVKKFNQNSITSSQNSSGTFINKEFYNHNNINQEFNSRKIIRNFFLSIERN
FMEILLLRKLGLLGNSHSLPSPFQFSCWANTHNQPIINRYSILENLRDVFQVPKWYFFDE
NKKTHKLDLSKNSIYHLLTWSFSIALSYEEGAIHLVGLGQFICENISISSKYQTISESGQ
IIAIHPEFLVVRLAKPSLATGGATIHSHYGQIIKKGDVLITLVYERLKSGDIIQGLPKVE
QLLEARPINSTSINLENGFENWNRDMTKSLGSLWGFLLSAKTTMNQSQINLVDQIQEVYQ
SQGVYISDKHIEIVVRQMTSKVLTLEDGMANGFLPGELIESSRAQRMNRVLEESVLYKPI
LLGITKASLNTQSFISEASFQETTRVLAKAALRGRIDWLKGLKENVIFGGVISAGTGCQE
VVWQVILEKRKETYSKRKKNKLFSGRVRDVFSYYQRILFFPTMKIIHKTLKKPLSEINLD
PNYRK

>Q85CS9 | RR3_ANTFO

MGQKINPLGFRLGVTQNHCSHWFAKPNNYYKLLEEDEKMRNCIYNYVRKHIKSSSNYGGI
ARIEIERKTDLIQIEIYTGFPALLVESRGRGIEELKADVQTVLNLGNRKLRMALTEVEEP
YTEPNILAEYIALQLENRVAFRRTMKKAIELTRKTSVKGIKIQIAGRLNGAEIARVEWAR
EGRVPLQTIRAQINYCYYPAQTIYGVLGIKVWIFQTEE

>Q85CT4 | RK14_ANTFO

MIQPQTYLNVADNSGARKLMCIRILGASNRKYGNIGDIIIAVTKEAVPNMPLKKSEVVRA
VIVRTCKEVRRDNGMTIRFDDNVAATTNQEGNPKGTRVFGPVARESRGYNFTKIIPLAPE
VL

>Q85FG6 | CHLN_ADICA

MKTPETLAFECETGNYHTFCPISCVAWLYQKIEDSFFLVVGTKTCGYFLQNALGVMIFAE
PRYAMAELEEGDISAQLNDQKELEKICLQIRDDRNPSVIIWIGTCTTEIIKMDLEGIAPK
LEKQIGIPIIVARANGLDYAFTQGEDTVLAAMAHRCPEYKHCTTNLKNRTDEMARIDNHT
TNKSPFEASKLTRFNLVLFGSLSSSIASQLNLELKRQSISVSGWLPSQKYEELPGLGEGI
YVCGVNPFLSRTATTLMRRRKCKLVGAPFPIGPDGTRAWIEKICSVFDIKSYGLENRESE
IWENVKDYIQVINGKSVFFMGDNLLEISLARFLIRCGMIVYEVGIPYMDRRYQAAELLFL
QHTCRKMKTPLPRIVEKPDNYGQIQRMHELKPHLAITGMAHANPLEARNIDTKWSVEFTF
AQIHGFSSVRDILELVTRPLRRRGVITPGFRSLSKQTTGD

>Q85FH3 | NU4LC_ADICA

MFEQGLILSAYLLCVGFFGLITSRNMVRALMSLELIFNAITLNFITLSNLFDNRETGEIF
TLFVIAVAAAEAATGLAIALSIHRNRRSTRIDQSNLLKW

>Q85FH6 | CCSA_ADICA

MIYTGIEYIFVNISFVMFFFVTLLNLINLFYKIDKIDHFSKNSMTIAFFCTTGFLITRYL

QTRHLPLGNLYESLMFLSWGFSLLYLILEVRDQIGLSHAVLAPGAMLIHAFATLSLPQQM
RSPTLLVPALQSQWLMMHVSAMLISYITLLCGSLLAITLLSLFYGKVGSVTLEHKFEKQS
FFFLMNPRKNLWKQTGAENYSYFLISNSRKCQLINCLDKWACQTISLGFSLLTIGILSGA
VWANEAWGSYWSWDPKETWALVTWLVYAIYLHTKVDKRKMGEGPAMTASMGFFLVWICFL
GVNLLGVGLHNYGWLA
>Q85FI2 | RR19_ADICA
MARSSKRGPFVANHLIREIRNLNIQKKRKLITTWSRASAIVPIMIGHTIAVHNGREHLPI
YVTDRMVGHKLGEFVPTRNFRGHAKSDKGSRR
>Q85FI4 | RR3_ADICA
MGRKIHPLGFRLGVSQKHYSYWFAQKKDYPKFLEEDRKIRNLVEKYIQKHVKSVSNYGGV
GHIEIQRKTDLIQINIYTGFPDLLIEEQSLGISQMKQDLRNLLGLESQNLRVTLTGVIQP
YGEPKILAEHVASQLKNRVPFRRTMKKTIEMAGRTNGGGIKIQIAGRLNGSEMARVEWAR
EGRVPLQTVEANISYCYHPAQTIYGVLGIKIWVFRDT
>Q85FI6 | RK14_ADICA
MIQIQSYLDVADNSGARKLMCIRVLGTGNRKYADIGDIIIAVVKEAVPNMFLKRSEVVRA
VVVRTRRGLKRRNGMVLEFDDNAAVIINQEGNPRGTRIFGPVARELRECNFARVVSLAPE
VL
>Q85FJ0 | RR11_ADICA
MSNKSRKIRSRGGKRGVQKGVIHIQASFNNTIITVTDVRGQVIIWSSAGACGFKGTRKST
PFAAQAAAENAIKASVDRGMKQAEVMMSGPGPGRDTALRAIRRSGIILSFIRDVTPMPYN
GCRPPRKRRV
>Q85FL5 | NU3C_ADICA
MFLSHQYDSFWIFLLVCISIPLLAFSITRFAAPPREGPEKSTSYESGIEPKGDTWIRFQI
RYYMFALVFTVFDVETVFLYPWATSFEELGLFAFVEVIVFIFILIVGLVYAWRKGALEWC

>Q85FM7 | RPOB_ADICA
MPVLPELAQIQFKGFSRFVHQKLLKELENFPKIEDTDKEVEFRSIGNQYQLTEPSLEERD
AAYQCITYSADLYVPAQLICKEDQIVQEEDVRLGSIPWMNSGGTFIINGIARVLVSQILR
SPGIYYNRESDQRGILAYTSTVISDQGGRFKLEIDGRNRIWVRISKKKKISIFILLVAMG
LCKRDILQNSRYPRIFSNMFKRQKESIESPEDAIVELYKHLYSATDADVFFSESIFRELQ
KRFFQHRCELGRIGRRNLNIKLTLDVPETESFLLPQDILAAADHLVEISYGEGKLDDIDH
LKNRRVRSVADLLQEQLKLALNRLENLIRQNLSRAVRRKRAITPRGLVTPVPVIAAFKEF
FGSHPLSQFLDQTNPLSEMVHKRRLSSVGPGGLTRRTASFQARDIHFSHYGRICPIETSE
GMNAGLISSLAIQAGVNTSGSLGSPYLEMSDSSGEEQLVNLSPAEDDYYRVASENLLLSE
WRGSEKEIIPVRYQQEFLSVLWEQVDFRSIHPLHHFSIGASLIPFIEHNDANRALMGSTM
QRQAVPLIKPERCIVGTGLESQVALDSGSVAISEQDGKVCYIDGNRIELSLIHEAKKNKL
AQPTTIEELKIFERSNNNTCMHQKPAVSLGELLRGGEIVADGAATVRGELALGKNILVAY
MPWEGYNFEDAVLISERLIYEDIFTSLHIEKHEVEICATNQGPERVTKQISQLDGYLLRH
LDDDGLVELGSWVEAGDVLVGKLTPQEGASSSRVPEGRLLQAIFGIQLVNARESCLRVPI
GGRGRVIDVRWVYPEDDATNDLEVIHIYILQRRKIQVGDKIAGRHGNKGIVSKIVPRQDM
PYLQNGTPVDMILSPLGVPSRMNVGQIFECLLGLAGEFLETHYRVVPFDERYEREASRKL
VFAELHEAGARTNNPWLFEPSHPGKSRLIDGRTGDPFGQSITTGKAYIMKLIHQVDDKIH
ARSSGPYALVTQQPLKGKSRRGGQRIGEMEVWALEGFGVSYTLQEMLTTKSDHIQARYKA
LSAIMTGKPVCKPKTVPESFRLLVRELRCMGLNLERNFISEEDLGREFENI

>Q85FN0 | RR2_ADICA
MQQKNWTIDLEGMMAAGIHFGHQTQRWNPKMSPYIFTERKGVHILDLTQTARLLSEACDL
VFDAAVEGKEFLMVGTKNQVTDLIVSAALKSQCHYVNEKWLAGTLTNWVTTETRLRRFQH
LQTGGDLGEFDRLPKREAAILKGQLVRLKKCLGGIQYMSDLPDIAIITDQHEQSIALKEC
SILGIPTICIVDTDCDPDLVDVPIPGNDDARSSIRWILDKLALAISEGRSNIKVT
>Q85FN7 | PSBK_ADICA
MTVSYSIYLENSLHFGDALLAKLPEAYAIFDPIVDVMPVIPVFFLLLAFVWQAAVSFR
>Q85FN9 | RR16_ADICA
MVKLRPKQCGRKQRTYRIVAIESQSRQEGKVIKEVEFYNPRREETQLDILAITTLCGSGV
KLTETVCNIFRRATFKIT
>Q85UZ8 | RR18_LARLA
MKQTMDKPKRSFRRHLTPIRRHLSPIGSGDRIDYKNMSLISRFISEQGKILSGRVNRLTS
KQQRLMTNAIKRARILSLLPFLYNEN
>Q85V01 | RR18_LARDC
MKQTMDKPKRSFRRHLTPIRRHLSPIGSGDRIDYKNMSLISRFISEQGKILSGRVNRLTS
KQQRLMTNAIKRARILSLLPFLYNEN
>Q85WB7 | GSA_BRANA
MSATLTGSGTALGFSCSSKISKRVSSSPSTRCSIKMSVSVDEKKKSFTLQKSEEAFNAAK
NLMPGGVNSPVRAFKSVGGQPVLIDSVKGSKMWDIDGNEYIDYVGSWGPAIIGHADDEVL
AALAETMKKGTSFGAPCLLENVLAEMVISAVPSIEMVRFVNSGTEACMGVLRLARAFTNK
EKFIKFEGCYHGHANAFLVKAGSGVATLGLPDSPGVPKAATSDTLTAPYNDIEAVAKLFE
AHKGEISAVILEPVVGNSGFITPTPEFINGLRQLTKDNGALLIFDEVMTGFRLAYGGAQE
YFGITPDLTTLGKIIGGGLPVGAYGGRRDIMEMVAPAGPMYQAGTLSGNPLAMTAGIHTL
KRLKQPGTYEYLDKITKELTNGILEAGKKTGHPMCGGYISGMFGFFFAEGPVYNFADAKK
SDTEKFGKFFRGMLEEGVYFAPSQFEAGFTSLAHTSEDIQFTISAAERVLGRI
>Q85WL0 | MATK_ERYRE
MEELQGYFKKARSLQQHFLYPLLLQEFIYTLAYDDGLKGSIFYEPIEFFGYDNKSSLVLI
KRLITRMYQQNFLIYSVNDSNQNGLRGHINYFYSHFFYSHIVSEGFSVIVEIPFSLRLVS
SPKEKEIPKSQNLRSIHSIFPFLEDKLSHLNNVSDILIPHPIHLEILVPILQYWIQDVPS
LHLLRFFLHKYHNLNSFIQNNKTIYVFSKENKRLFWFLYNSYVSECEFLLVFLRKQSCYL
RSTSSVAFLERSHFYGKMEHIIIVCCNNFQKTLWPFKDPFMHYVRYQGKAILASRGAHLL
MKKWRYYLVNFWQYYFHFWSQPYRMHINPLLNYSFYFLGYLSSVLINPYAVKNKMLENSF
LIDTVFKKFDTIIPIIPLIGSLSKAKFCTVSGHPISKPVWGDLSDFDIIDRFGRICRNLS
HYHSGSSKKQSLYRIKYILRLSCARTLARKHKSTARALLQRLGSGLLEEFFTEEEQVLSF
IFPKTTPFPLHGSHKERIWSLDIIRVNDLVNQII
>Q85WS8 | RPOC1_PINKO
MIEQNKHQQLRIGLASPEQICAWSEKILPNGEIVGQVTKPYTLHYETNKPERDGSFCERI
FGPIKSRVCACGNSPGIGNEKIDSKFCTQCGVEFVDSRIRRYQMGYIKLACPVVHVWYLK
RLPSYIANLLAKTRKELEGPVYCDLFLARPIAKKPTLLRSRGTFNYEIQSWKDIIPHYLS
ARPHYLFARGSGTFKEREIATGGDAIGEQLTGLDLQMIIDRSHMEWKNLVELKWNRLEEN
QESTVDRWEDEKIRRRKDFLVGRIKLAKHFLRTNIEPKWMVLCLLPVLPPEPRPIVQLGE
GGLITSSDLNELYRRVINRNNTLTNLLARSGSESFVICQKKLIQEAVDALLDNGICGQPM
RDSHDRPYKSFSDVIEGKEGRFRENLLGKRVDYSGRSVIVVGPFLSLYQCGLPSEIAIEL
FQAFVIRSLIGRHIAPNLRAAKSMIRDKGPIVWEVLQEVMQGHPVLLNRAPTLHKLGIQA

FQPILVEGRAIRLHPSVCGGFNADFDGDQMAVHVPLSLEARAEARLLMFSETNLLSPAIG
DPISIPTQDMLLGLYISTVENSQGIYGNRYHPYHSEKKSFSCKKPSFYSYDDVLRAYRQK
RIDLYSPLWLRWGELDLRIITSVNQEAPIEVQYESLGTFHEIHEHYRIRKGRMGEILNIY
IRTTVGRTRFNREMEEAIQGFACFEHPKKSLPALRI
>Q85WT6 | CHLL_PINKO
MKIAVYGKGGIGKSTTSCNISVALARRGQKVLQIGCDPKHDSTFTLTGFLIPTIIDTLQS
KDYHYEDIWPEDVIHKGYGGVDCVEAGGPPAGAGCGGYVVGETVKLLKELNAFYEYDIIL
FDVLGDVVCGGFAAPLNYADYCVIITDNGFDALFAANRITASIREKARTHPLRLAGLVGN
RTSRRDLINKYVEACPMPVIEVLPIIEDIRVSRVKGKTLFEMVGSEPSLNYVCNYYLGIA
DQILSQPEGIVPKEIPDRELFSLLSDLYLNPIGGGGQKKKIQENFLGFTRI
>Q85WU8 | RR15_PINKO
MIKNLSISSSLIPDKQRGSVESQVFFLTNRVLRLTQHLQLHGRDYSSQRGLWLILSKRKQ
LLVYLSKRDKLRYDDLIGQLSIRRLKTR
>Q85WV0 | RK32_PINKO
MAVPKKRTSRSKKKIRKNVRKGKKHIGPAIKAFSLAKSISTGHSKSFYCIVNDDSSGSSE
SKLTAFDLDDP
>Q85WW7 | RR14_PINKO
MARKSLIQREKKRQALERKYHLIRQSLEEKSKVSSLDDKWEIHRKLQSSPRNSAPTRLHR
RCSSTGRPRANYRDFGLSGHILREMAHACLLPGITKSSW
>Q85WX8 | RR4_PINKO
MSRYRGPRLKIIRRLKTLPGLTSKRPKNRKDSMNMNRSSSRKISQYRIRLEEKQKLRFHY
GLTERQLLKYVRVSRRAKGSTGQVLLQLLEMRLDNIIFRLGMAPTIPGARQLVNHGHIRV
NDHMVDIPSYPCKPQDVITIRDQPRLRAIIKKNIDLFQRDKLPNHLTFHPLQYKGFINQI
IDSKWISLKINELLVVEYYSRQA
>Q85WY6 | RK23_PINKO
MDEVKYPVLTEKSIRLLERNQYTFNVDLQSNKTKIKNWIENFFDVKVIAMNSYRLPEKGG
KRGSMIVHPIRCKRMIITLRTGDSIPLFSEQ
>Q85WY8 | RR19_PINKO
MARSLKKNPFVANHLLRKIKNLNIKKEKKIIVTWSRASVIVPAMIGHTIAVHNGREHLPI
YVTDRMVDHKLGEFAPTLLFQGHARNDKKSRR
>Q85WY9 | RK22_PINKO
MESMGNNSPGPEIRALARNIRMSAHKARRVINQIRGRSYGQALMILELMPYGACYPISQL
IHSAAANANHNMGLNKANLLVGRVEVNEGAVLKRIQPRAQGRGYPIQKPTCHITIVSEEI
SRSNDPIMSIESRKKGYVWRRK
>Q85WZ0 | RR3_PINKO
MAQKINPLGFRLGVTQNDRSHWFAQQRNYSKDLREDQKIRTCIENYVRTHIKSSSNYGGI
ARVEIRRKIDLIQVKIYIGFPNLLLIEGRGQGIEKLRNDVLNMLDSADRKLHIAIEKVAK
PYRKPNILAEYIALQLEKRVPFRKTMKKAIELAEREEVEGIQIQIAGRLDGKEIARVEWD
RGGRVPLQTIRARIDYCYYPVQTIYGVLGIKIWILEE
>Q85WZ6 | RR8_PINKO
MGNDTIANLITSIRNADMVEKGTVRVTATNITKNIGRILLREGFIEDVREHQEGQKSFLI
STSKYRRRKKRTYMTTSKRTSKPGLRIYSNYREIPKVLGGMGIVILSTSQGILTDREARQ
KKIGGEILCYVW
>Q85WZ7 | IF1C_PINKO

MKKQNLIHAEGLVTESLPNGMFRVLTDNGCQILTHISGRIRRNSVRILPGDRVKVELSAY
DLTKGRIIYRLSNKS
>Q85X25 | ACCD_PINKO
MSIKEWFEDRRKITGLLKNSVERDSKDANETEKNKNLSIDYAKINRLWAQCDNCESLLYI
RFLRENQSVCKECGYYLQMNSSDRIELPIDRDTWHPMDEDMYTLDVLQFHSENEPSHSDH
LDSEDESYKDHISFYQIETGLTDAIQTGIGQLNGIPIALGVMDFKFMGGSMGSVVGEKIT
RLIERATAESLPVIMVCASGGARMQEGSFSLMQMAKIASALYIHQKDKKLLYISILTSPT
TGGVTASFGMLGDIIIAEPKAYIAFAGKRVIEQTLGQKVIEDFQVTEHLFGHGLFDLIVS
RNLLKGVLSELFRLYGLPRK
>Q85X50 | MATK_PINKO
MDEFHRYGKEDNSRQQCFLYPLFFQEDLYAISHDHYLDGSSSSEPMEHLSSNDQFSFLTV
KRLIGQIRQQNHSIVLFVNCAPNPLADCKKSSYSESVLEGLTLVLEVPFSIRSKYSVEGM
NEWKSFRSIHSIFPFLEDKFPHSNYISDARIPYSIHPEILVRTFRRLIRDAPSLHPLRSV
LYEYRNSPENLQRSIIVVPRVNTRFFLFLWNYYVYECESILFSLLKRSSHSRSLSHRPFP
QRTHFHRKIKHIIIFSRRNSLKSIWLLKDPKINYVRYGERSIIAIKGTHLLVKKCRYYLL
LFRQCYFHLWSEPYRVCSHQLSKNCSSSPGYFLRVRMNPLFVRTKMLDELFIADLITNEF
DPIVPIVPILGLLAREKFCDVSGRPISKLSWTNLTDDDILNRFDQIWRNLFHYYSGSFGR
DGLYRIKYILSLSCAKTLACKHKSTIRVVRKELGPELFQKSFSKEREFDSLPFSSKAAAR
SQRERIWHSDIPQINPLVNSWQKIQDLKIENLFDQ
>Q85X62 | RPOC2_PINKO
MKIWRFLLMKKQTRLPFDNLPFYNKVMDKTAIKKLISRLIDHFGMTYTSHILDQLKTSGF
KQATDTAISLGIDDLLTAPSKGWLVQDAEQQGSVSEKQNHYGNLHAVEKLRQSIEIWYAT
SEYLRKEMNTNFSMTDPLNPVHVMSFSGARGNTSQVHQLVGMRGLMSDPQGQIIDLPIRR
NLREGLSLTEYIISCYGARKGVVDTAVRTADAGYLTRRLVEVVQQIVVRRTDCGTVQGIF
VSPIRGRERDINEVVVRTQILIGRVLADDVYINRRCIATRNQDIGVGLANQLRNIRPRPI
YIRTPFTCKSISRICQLCYGRSTTHSHLIELGEAVGIIAGQSIGEPGTQLTLRTFHTGGV
FTGDIAEHIRAPFNGKIEFNENLVYPTRTRNGHPAYLCHNNLSITIDGQNQVQNLTIPPQ
SLLLVQNDQYVESEQIIAEVRARTSSFKEKVRKNIYSDLEGEMHWSTNVCHAPEYVHGNV
HSILRTGYLWILSGGIYGSGVVPFPFHKYQDQVDVQPFVAKHTDSYVDQVEHRSGDSNCY
GKEEQIFSYSETETDRTISNEHRDSIYVTFSPKNYNMKGKKQMNRFIVSLQCDKEWGKRI
IPCPDAILRIPKSGILQINSIFGYSNVEHGIPDGPNMTTPFSLDLSREGDNLQIQISNSI
LYEDGERIQVMSDTSIPLVRTCLGFDWEQIDSIESEAYVSLISVRTNKIVNNMVQISLMK
YPPFFMGRRDNKASSNLMFHNNLDHTNLFSSNGASQLISKHQGTICSLSNGEEDSGSFMV
LSPSDCFRIVLFNDSKCYDTGNKSNRKDPMRKIIEFSGLLGHLHSITSRFPSSQFITDKK
VLSKKHSIFHNYFMDENMRISHFDPCRNIISNLLGPNWCSSSSEFCKKTFPVVSLGQLIP
ESVWISEDEPLPESGQIIAVDEESLVIRSAKPYLATRKATVHGHYGEILDKGDTLITLIY
ERLKSSDIIQGLPKVEQLSEARLNNSISMNLKESFENWTGDMTRFLGSLWGLFISARITM
EQSQIHLVNQIQKVYRSQGVRIGDKHIEIIVRQMTSKVLISEDGTANVFSPGELIGLSRA
QRMDRALEETIYYQTMLLGITRASLNTQSFISEASFQETARVLAKAALQGRIDWLKGLKE
NVILGGMIPAGTGQHIHRSGKRNGIDPRIGNRNLFSNKVKDILFHHDKVSFFSIQENYHN
ILKQPLKES
>Q85X72 | PSBK_PINKO
MPVIFNICLDDAFIHSNNPFFGKLPEAYAIFDPIVDVMPIIPVLSFLLAFVWQAAVSFR
>Q85XY8 | MATK_MIMPU

MKEYQVYLERDRSRQQDFLYPLIFREYVYGLAYSHDFNRSTFVENVGYDNKYSLLIVKRL
ITRMYQQNHLIISANDSKKNPFLGYNKNFYSQIISEGFAIIVEIPFFLQFSSSLEAAEIV
KSYKNLRSIHSIFPFLEDKFPYLNYVSDIRIPYPIHLEILVQILRYWVKDAPFFHLLRLF
LYNFCNRNSFLTPKKSISTFSKSNPRLFLFLYNFYVCEYESIFLFLRKKSSHLRLKSFSV
FFERIFFYAKREHLVEVFAKDFSSTLTFFKDPLIHYVRYQGKSILASKNAPLLMNKWKHY
FIHLWECFFDVWSQPGTIHIKQLSEHSFYLLGYFSNVRLNRSVVRSQMVQNTFLIEIVSK
KLDIIVPIIPIIRSLAKAKFCNVLGHPISKAVWADSSDFDIIDRFLRICRNISHYYNGSS
KKKSLYRIKYILRLSCIKTLACKHKSTVRAFLKRSGSEELLEEFFTEEEEILSLIFPRAS
STLQKLHGNRIWYLDILFSNDLVNHE
>Q85ZU5 | MATK_HORJU
MEKFEGYSEKHKSRQQYFVYPLLFQEYIYAFAHDYGLNGSEPVEIVSCNNKKFSSLLVKR
LIIRMYQQNFLDNSVNNPNQDRLLDYKNYFYSEFYSQILSEGFAIVVEIPFSLRELSCPK
EKEIPKFQNLRSIHSIFPFLEDKFLHLDYLSHIEIPYPIHLEILVQLLQYRIQDVPSLHL
LRFFLNYYSNWNSFITSMKSIFYFQKENKRLFKFLYNSYVSEYEFFLLFLRKQSSCLPLA
SSGTFLERIHFSRKMEHFGIMYPGFSRKTLWFFMDPLMHYVRYQGKAILASKGSFFLKKK
WKCYLINFWQYYFFFWTQPRRIHINQLANSCFDFMGYLSSVPKSPLLVRNQMLENSFLID
TRMKKFDTIVPATLLIGYLSKAQFCTGSGHPISKPIWTDLSDWDILDRFGRICRNLFHYH
SGSSKKRTLYRLKYILRLSCARTLARKHKSTVRTFMQRLGSAFLEEFFTEEEQVFSLMFT
KTTLFSFCGSHTERIWYLDIIRINDLVNPLN
>Q8GVA0 | MATK_GALEW
MEELQGYLEKDRSRQQHFLYPLLFQEYIYALAHDYGLNGSIFYESAEVFGYDNKSSLALV
KRLITRIYQQKSLIYLVNDSKQNRFVGHTHNNFFYSRFYSQMISESFSIIVEIPFSLRLV
SYLKEKEIPKYHNLRSIHSIFPFLEDKLSHLNYVSAILIPHPIHMEILVQILQCWIQDVP
FLHLLRFFLHEYHNWNSFLITQKKSIFVFSKEKKRLFRFIYNFYVFECEFLFVFIRKQSS
YLRLTFFGTFFERTHFYGKIEHLQIEKLIVICRNDFHRTFWFFKDPFMHYVRYQGKAILA
SKGTHLLMTKWKYHFVNFWQYYFNFWSQPYRIQINQLSNYSFYFLGYLSSLLINSSAVRN
QMLENSFIIDTLTKKFDTIVPVILLIGSLSKAKFCTISGHPISKPIWANLSDSDILDRFG
RICRNLFHYHSGSSKKQGLYRIKYILRLSCARTLARKHKSTVRTFLRRLGSGLLEEFFTE
EEEVLSLMFPKTTSFTLHGSHRERIWFLDIIRINELVNRS
>Q8HQR0 | MATK_PINPR
MDEFHRYGKEDNSRQQCFLYPLFFQEDLYAISHDHYLDGSSSSEPMEHLSSNDQFSFLTV
KRLIGQIRQQNHSIVLFVNCAPNPLADCKKSSYSESVLEGLTLVLEVPFSIRSKYSGMNE
WKSFRSIHSIFPFLEDKFPHSNYISDARIPYSIHPEILVRTFRRLIRDAPSLHPLRSVLY
EYRNSPENLQRSIIVVPRVNTRFFLFLWNYYVYECESILFSLLKRSSHSRSLSHRPFPQR
THFHRKIKHIIIFSRRNSLKSIWLLKDPKIHYVRYGERSIIAIKGTHLLVKKCRYYLLLF
RQCYFHLWSEPYRVCSHQLSKNCSSSPGYFLRVRMNPLFVRTKMLDELFIADLITNEFDP
IVPIVPILGLLAREKFCDVSGRPISKLSWTNLTDDDILNRFDQIWRNLFHYYSGSFGRDG
LYRIKYILSLSCAKTLACKHKSTIRVVRKELGPELFQKSFSKEREFDSLPFSSKAAARSQ
RERIWHSDIPQINPLVNSWQKIQDLKIENLFDQ
>Q8HQS2 | MATK_PINRE
MDEFHRCGKEDSFWQQCFLYPLFFQEDLYAISHDHYLDVSSSSRPMEHLSSNDQLSFLTV
KRLIGQIRQQNHSIVLFVNCDPNPLADRKKSFYSESVLEALTLVLEVPFSIWSKSSVEGM
NECKSFRSIHSIFPFLEDKFPHSNSILDARIPYSIHPEILVRTFRRWIRDAPSLHPLRSV
LYDYRNSPENLQRSIIVVPRVNTRFFLFLLNYYVCECESILFSRLKRSSHSRSLSHGSFP

QRTHFHRKIKHIIIFSRRNSLKSIWSLKDPKIHYVRYGERPIIAIKGDDLLVKKCRYYLL
IFRQFYFHLWSEPYRVCSHQLSKNCSSSPGYFLRVRMNPLLVPTKTLDDFFIPILITNEM
DPIVPIVPIIGLLATEKFCDISGRPISKLSWTSLTDDDILDRFDQIWRNLFHYYSGSFDR
DGLYRIKYILLLSCAKTLACKHKSTIRVVRKELGPELFKKSFSKEREFDSLPFSSKAASQ
RERIWHSDIPQINPLANSWQKIQDLKIENLFDQ
>Q8HQT1 | MATK_PINAT
MDEFHRCGKEDSFWQQCFLYPLFFQEDLYAISHDHYLDVSSSSRPMEHLSSNDQLSFLTV
KRLIGQIRQQNHSIVLFVNCDPNPLADRKKSFYSESVLEALTLVLEVPFSIWSKYSVEGM
NESKSFRSIHSIFPFLEDKFPHSNSILDARIPYSIHPEILVRTFRRWIRDAPSLHPLRSV
LYEYRNSPDNLQRSIIVVPRVNTRFFLFLWNYYVCECESILFSRLKRSSHSRSLSHGSFP
QRTHFHRKIKHIIIFSRRNSLKSIWSLKDPKIHYVRYGERPIIAIKGAHLLVKKCRYYLL
IFRQFYFHLWSEPYRVCSHQLSKNCSSSLGYFLRVRMNPILVRTKMLDELFIADLITDEI
DPIVPIVPIIGLLATEKFCDISGRPISKLSWTSLTDDDILDRFDQIWRNLFHYYSGSFDR
DGLYRIKYILSLSCAKTLACKHKSTIRVVRKELGPELFKKSFSKEREFYSLRFSSKAAAR
SQRERIWHSDIPQINPLANSWQKIQDLKIENLFDQ
>Q8HQT2 | MATK_PINPN
MDEFHRCGKEDSFWQQCFLYPLFFQEDLYAISHDHYLDVSSSSRPMEHLSSNDQLSFLTV
KRLIGQIRQQNHSIVLFVNCDPNPLADRKKSFYSESVLEALTLVLEVPFSIWSKSSVEGM
NESKSFRSIHSIFPFLEDKFPHSNSILDARIPYSIHPEILVRTFRRWIRDAPSLHPLRSV
LYEYRNSPDNLQRSIIVVPRVNTRLFLFLWNYYVCECESILFSRLKRSSHSRSLSHGSFP
HRTHFHRKIKHIIIFSRRNSLKSIWSLKDPKIHYVRYGERPIIAIKGAHLLVKKCRYYLL
IFRQFYFHLWSEPYRVCSHQLSKNCSSSPGYFLRVRMNPILVRTKMLDELFIADLITDEI
DPIVPIVPIIGLLATEKFCDISGRPISKLSWTSLTDDDILDRFDQIWRNLFHYYSGSFDR
DGLYRIKYILSLSCAKTLACKHKSTIRVVRKELGPELFKKSFSKEREFYSLRFSSKAAAR
SQRERIWHSDIPQINPLANSWQKIQDLKIENLFDQ
>Q8HQT5 | MATK_PINTA
MDEFHRCGKEDSFWQQCFLYPLFFQEDLYAISHDHYLDVSSSSRPMEHLSSNDQLSFLTV
KRLIGQIRQQNHSIVLFVNCDPNPLADRKKSFYSESVLEALTLVLEVPFSIWSKSSVEGM
NESKSFRSIHSIFPFLEDKFPHSNSILDARIPYSIHPEILVRTFRRWIRDAPSLHPLRSV
LYEYRNSPDNLQRSIIVVPRVNTRFFLFLWNYYVCECESILFSRLKRSSHSRSLSHGSFP
HRTHFHRKIKHIIIFSRRNSLKSIWSLKDPKIHYVRYGERPIIAIKGAHLLVKKCRYYLL
IFRQFYFHLWSEPYRVCSHQLSKNCSSSPGYFLRVRMNPILVRTKMLDELFIADLITDEI
DPIVPIVPIIGLLATEKFCDISGRPISKLSWTSLTDDDILDRFDQIWRNLFHYYSGSFDR
DGLYRIKYILSLSCAKTLACKHKSTIRVVRKELGPELFKKSFSKEREFYSLRFSSKAAAR
SQRERIWHSDIPQINPLANSWQKIQDLKIENLFDQ
>Q8HQT6 | MATK_PINEG
MDEFHRCGKEDSFWQQCFLYPLFFQEDLYAISHDHYLDVSSSSRPMEHLSSNDQLSFLTV
KRLIGQIRQQNHSIVLFVNCDPNPLADRKKSFYSESVLEALTLVLEVPFSIWSKYSVEGM
NESKSFRSIHSIFPFLEDKFPHSNSILDARIPYSIHPEILVRTFRRWIRDAPSLHPLRSV
LYEYRNSPDNLQRSIIVVPRVNTRFFLFLWNYYVCECESILFSRLKRSSHSRSLSHGSFP
QRTHFHRKIKHIIIFSRRNSLKSIWSLKDPKIHYVRYGERPIIAIKGAHLLVKKCRYYLL
IFRQFYFHLWSEPYRVCSHQLSKNCSSSPGYFLRVRMNPILVRTKMLDELFIADLITDEI
DPIVPIVPIIGLLATEKFCDISGRPISKLSWTSLTDDDILDRFDQIWRNLFHYYSGSFDR
DGLYRIKYILSLSCAKTLACKHKSTIRVVRKELGPELFKKSFSKEREFYSLRFSSKAAAR

SQRERIWHSDIPQINPLANSWQKIQDLKIENLFDQ
>Q8HTL2 | RK2_CHLRE
MGIRFLQAYTPGTRNRSVSDFSELTDKNSTPEKALTVSLHRAKGRNNRGIITCRHRGGGH
KRLYRQIDFRRDKIGVTAKVVRIEYDPNRNARIALLRYEDGEKRYIIHPRGLNIGDIIQS
DLNAPILIGNSLPLRNIPLGAEVHNVEFQPGSGGQLARSAGAMVEILAKEGNFVTIRLPS
KEIRLVSKNCWATVGQVGNIEAYNLTIGKAGRTRWLGKRPTVRGSVMNPVDHPHGGGEGR
APIGRSRPVTPWGRPALGQLTRKPKKYSNTLIVKKRKK
>Q8HTL3 | RK23_CHLRE
MLDLVKYPVITQKTYIALFKDRQYTFDVDLRLTKPQIKKVFETLFNVDVISVNTHIPPRQ
KIRVGLAQGYRPRYKRAIITLKEGQSINYSLKNDN
>Q8HTL6 | RPOB1_CHLRE
MLNISTNMMINQTLNFDSNQESQKNKTERISKTKLTRELITKTDHDIYVDDISFLQREDN
VNVSQIDLTARGVLNPEDQGVLHKSTEFGVFNKNKTGLPASVDLKIKQNSFFSVKQPDFN
KYLISDFVEIQRNSFFTLLEKGIIEEFSKRNPITNSKKTMEIFFYPDYYQLTPPEYSPSQ
AIIKSKSYTSKLYIPVQLTDKSKNIIKLKWVYIGDIPLMTKRGHFILNGCARVIVNQMVR
SPGIYYQKKIYENFSNKWSEKPENTFTRYFADLICNRGTWLRIEMDKYNKIWAQMKRVPK
IPIMWFLIAVGLTDKIVLKTVMDSKILLYNLDEDPLNPRKKPLPYVKTPPAAWSAIYNIV
FAKKIKAQEKAKKMLELTEGNPSSKSQTKNKTSASKKSKTLNVANTKGKTPAENIKTLSE
LIDLEKALFLKSEQGRKWIFNKFMNPRTYDLGKVGRVNFNRKLKLSISQDITTLTPQDLL
AATNNLILVSKGLRELDDIDHLKNRRVRTSGELIQIQIGVGLVRLEKTIREKMTYASGLS
SLPSQKFAFRSSKQRNQSPVGDAENATQLTIGNLINTKPFNGALREFFGTSPLSQFMDQI
NPLAELTHKRRLSSMGPGGVTRDSATLAIRGIHPSHYGRICPVETPEGKNTGLVNSLTAY
ARVNAAGYIETPFYRVYKGQVQKKTGLYFFSAKQEEKIKLGAPDLYTSEIGFLPKASIPV
RIVEDFTKISRNEIQYVGVAPIQMISIATSLIPFFEHDDANRALMGSNMQRQAVPILKPQ
RPIVGTGLEARAVSDSGHVITAKSSGIVMYTSSKEIIIYSLQ
>Q8HUG7 | MATK_CORVR
MEEYQLYLELDRSRQQDFLYPLVFHEYVYGLAYSHDLNRSIFVENIGYDNKYSLLIVKRL
ITRMYRQNHLIISANDSNKNRFLRYNKNFDSQIISGGFAIVVEILFSLQLSSSLEEAEII
KSYKNLRSIHSIFPFFEDKVTYLNYISDIQVPYPIHLEILVQILRYWVKDAPFFHLLRLF
LYDYCNWNSIIIPKKSIYTFSKNNTRFFFFLYNFYVCEYESIFFFLRTQSSHLRLKSFRF
FFERIFFYAKKGHLVEVFVKDFFSTLTFFKDPFIHYVRYQGKSILASKNLPILMNKWKYY
FIHLWQCYFDVWSQPGTIHINQLSEYSFHFLGYFLKGGLKHSVVRGQMLQKGFLIKIIIK
KLDIIVPIIPIIRLLAKAKFCNVLGNPLSKPSWADLSDFDIIARFLRICRNLSHYYNGSS
KKKSLYRIKYILRLSCIKTLACKHKSTVRAFLKRLGSEELLEEFFTEEEEILSLIFPRTP
STLRRLHRNRIWYLDIFFSNDNDLINHD
>Q8HUG9 | RPC1A_CHLRE
MHYKKYAELLKNGTYFSHIHSPTLLYPKGKVMYAQYGNSPLQGLNSQVTNLNLIRKVVAA
SAEMDSFKKLPNLQNNKLPLNNLKARLNTKAIYISNEEAQLTVSKIGTPNAIETDVPGTI
KNAPSNTLLTHRSFKFFVNKIYAKPLISLKKTKETKPLLLGKTGTTLTQKGLNPFQSLFL
NTKSSSPSTARSFGTKNIVNTLQIKKIVHKFENSYSKLTEINLITINLASANRIRQWAEK
TLPNGKVVGEVINPETIHYKTLKPIKGGLFCERIFGPLKDHECACGKKFNIKNYLTKTVN
NSSIQPIAESRQTQPNTQLSLNLQKRYFCRICDVEYTYSIIRRTQLGYIQLASPTTHVWF
VKGIPSYISILLDMKKKHLQGITYNTETLTLENSFRGRQLLPVSPSSIFESWQKIMKKQY
PEKYNLTNTMIKIKSTNTLPLRVSQPNSNTSYSYMPNIYIPEGEGEEKTKTKLKKTTPLN

AIAQKGVKYKKPKTKKALYKQYLKNYYARKPKNSNQAEAVSFGVNKVQLPLTAFQQRNKV
YFYKSEKRNWPVLTTVAKMQYIVSKKGWFKLIQYVIKSAESYGAATPTKMDGLQKMSLLT
KQA
>Q8HUH2 | RPOA_CHLRE
MTIYPNLKKIMTKTQSTDFFIACKESRIENNTNFYGCFYLGPFDESLSQTLANDLRRTLL
SELTGLAITSIEIEGVLHKFSTLTGMKEPVLDLICNLQNIVLRKETISSTNMNYRATKKT
YIGFLSVNGPRVIKAADLKLPAGLQCVDPNQYIATLAEDGFLNMKFNINEGKNYIKQKPY
NLDVTTLKKRNILLQNFKNKIGLTALKNKQLMSTTMEGKPLLSNSDNLRFKKSFKRMLTS
PNQSLKNKTSLGHDTVSNPIPLDAVFMPVTKINCIIEENNVYSDFSTDPSLEFSTHLVPS
LTTQTNLRQESTHKFVNKANSLLQENNLFRSEKVYLPNIYIPEGEGDALSLKGVSPYSDF
KTFLSTLNYNSLYQTSLFLNQSLGQNKLLPWQANTLFFDVTNFADSQSNDVNMNMDLAGD
KTSVQKIHSTGISNTDAQLNKLSLTKIKSFLGSSDKLQNKTYQSFLQPKYASSNLRTLLT
QRSLQKNQSVFMHSFLDDQAKHKELTSNTLRANKFQVMKTVVSIPPKTFKTNPINTFHNQ
SLTFEYARKF
>Q8HVA1 | MATK_ALPCA
MEELQGYLEEYRSRQQQFLYPLLFQEYIYVFAYDHGLNSSIFYEPQNSLGYDNKFSSVLV
KRLIIRMYQKNYWIYSVNDIYQNIFVGHNNYFYFHFFSQILSEGFAVIVEIPFSLQLISS
LEEKEIPKSHNLQSSHSIFPFLEDKLLHLNYLSDILIPYPAHMEILVQMLQSWIQDALSL
HLLQFLLHEYYNWNSLIIPNKSIYVFSKDNKRLFCFLYNLYIYEYEFLLVFPCKQSSFLR
LISSGVLLERIHFYVKIEHLGVCRIFCQKTLWIFKDPFIHYIRYQGKSILGSRGTHFLMK
KWKYHLVHFWQYYFHFWSQPYRIDTKKLSNYSFYFLGYFSSVQMNSSMVRNQMLENSFLM
DTLTKKLDTRIPIIPLIRSLSKAQFCTVSGYPISKPIWTDLADCDIINRFGRICRKLSHY
HSGSSKKQSLYRMKYILRLSCARTLARKHKSSARSFLQRLSSGLLEEFFTEEEQVISLIF
PKRTSFYLYGSYRERIWYLDIIRINDLVNSLLVTT
>Q8L803 | RK9_WHEAT
MASPSCASTLPWTAAAFSYPRRLQTRRAPSLVIVAQGRVKKYRQVILKDDIDEISGKKGD
TMKVRAGFYRNFLLPKGKATLLTPDVLKEMQLEQERIEAEKKRVKEEAQQLARVFETIGA
FKVPRKGGKGKQIFGSVTAQDLVDIIKSQLNRDVDKRLVEVPEIREVGEYVAEIKLHPDV
TAKVRLTVYTK
>Q8LB10 | CLPR4_ARATH
MEVAAATATSFTTLRARTSAIIPSSTRNLRSKPRFSSSSSLRASLSNGFLSPYTGGSISS
DLCGAKLRAESLNPLNFSSSKPKRGVVTMVIPFSKGSAHEQPPPDLASYLFKNRIVYLGM
SLVPSVTELILAEFLYLQYEDEEKPIYLYINSTGTTKNGEKLGYDTEAFAIYDVMGYVKP
PIFTLCVGNAWGEAALLLTAGAKGNRSALPSSTIMIKQPIARFQGQATDVEIARKEIKHI
KTEMVKLYSKHIGKSPEQIEADMKRPKYFSPTEAVEYGIIDKVVYNERGSQDRGVVSDLK
KAQLI
>Q8LU58 | CHLL_CHAGL
MKIAVYGKGGIGKSTTSCNISIALARRGKRVLQIGCDPKHDSTFTLTGFLIPTIIDTLQS
KDYHYEDVWPEDVIYKGYGGVDCVEAGGPPAGAGCGGYVVGETVKLLKELNAFYEYDVIL
FDVLGDVVCGGFAAPLNYADYCIIITDNGFDALFAANRIAASVREKARTHPLRLAGLVGN
RTSKRDLIDKYVEACPMPVLEVLPLIEDIRVSRVKGKTLFEMAESQESLNYVCDFYLNIA
DQILSCPEGVVPKEVPDRELFSLLSDFYLNPTLSEKENTLSPSSLDFMMV
>Q8LW11 | RK23_PHAAN
MNGIKYAVFTDKSIRLLGKNQYTFNVESGSTRTEIKHWVELFFDVKVIAMNSHRLPVKGR

RVRPIMGHTMHYRRMIITLQPGYSIPPLRKKRT
>Q8M930 | MATK_PAUTO
MEEIQRYLQLERSQQHDFLYPLIFQEYIYAFAHDPGFSRSILSENPGYDNKSSLLIVKRL
ITRMYQQNHFIISPNDSNQNPFWARNKNLYSQIISEGFAFIVEIPFSLRLISCLEGKKKK
IVKYQNLRSIHSIFPFLEDNLSHFNFVLDILIPHPVHVEILVQTLRYWVKDASSLHLLRF
FLNEYYNWNSLITPKKASSSFSKRNQRLFLFLYNSHVCEYESIFVFLRNQSSHLRSTFSG
ILFERIYFYGKIERLVNVFVKVKDFQANLWLVKEPCMHYIRYQRKSILASKGTSFFMNKW
KCYLVTFWQWHFSLWFHPRRIYINQLSNHSLEFLGYLSSVRMNPSVVRSQILENSFLINN
AIKKVDTLVPIIPLIASLAKAKFCNVLGHPISKPVRADLSDSNIIDRFGRIYRNLSHYHS
GSSKKKSLYRIKYILRLSCARTLARKHKSTVRTFLKRLGSELLEEFLMSEEDVLFLTFPK
ASSTLQGVYRSRIWYLDIISINDLANHKSKF
>Q8M986 | MATK_PLAAE
MEEIQRDLQLERSRQQDFLYPLIFQEYIYAFAHDRSLSRSFLSENPDSENQVYENKSSLL
IVKRLITRMHKQNHFLISTNDSKKNLFLGRNKDLDSLILLEGFAFIVEIPYSLRLISSLE
GKRKKIEKSQTLRSIHSIFPFLEDNFSHLNFVLDILIPYPVHAEILVQTLRYWVKDAPSL
HFLRXXLNDYWSLSTPKKAGLKRNQRFFLFLYNSHVCEYESIFVFLRNQSSHLQSLSFGV
LLERIYFYGKIECLGSVFLKVTDCQANLWLVKEPCMHYVRYQRKCILSSKGTSLFMNKWK
CYLATFWQWHFSLWFHPRRISTNPLYNHLLEFVGYLSSARMHPAMVRSQILENSFLINNA
IKKVDALIPIMPMVTSLAKAQFCNLLGHPTSKPVWADLSDSNIINRFGHICRNISHYYSG
SSKKKSLYRIKYILRLSCARTLARKHKSTVRTFLKTAGSGFLEEFLMSEEDLLCWTFPKA
SSALWGVYKSRIWHLDIIWINDLANHKKNLRPWK
>Q8M9L8 | MATK_IMPCA
MEEFKGYLELDRSQQQDFLYSLSFQESIYTLAHDHGLNRLILNADSDKKYSLLIVKCLIT
QMYQQNLLIFSANEPKQNLFFGHNTDLYCRILFEAFTVILEIPFSLRERPFIERKEMVQF
LNLRSIHSIFPFLEDKFSHSNYVVDILLPHSIHLEILVQTLRYWVKDASSLHLLRFFLHQ
YHNWNSFIIPKQSRFFFXKKKKBQRLFFFLYNSKVCEYESIFIFLRNQSVHLRSISSEAF
LERIYFYEKMEYFLEVYAKDFQAFFWLFKDLFMHYVRYQGKCILVSKGTSFLMSKWKYYL
VNLWQSCFYMWSQPGRVQINQFSNYSLDFLGYLSSVRRNPSMTWSQMLENSFLISLVIKK
FDTRVPTIPLIRSLSKAKFSNILGYPISKASWADFSDSNIIDRFGRIYRNLSHYHSGSPK
KTSLYKVKYILRLSCVRTLARKHKSKVRSFLKRFGSGLLEEFFMEEEQVLTLNLQKISFT
LRRFYGRQIWYLDIFCINDLAKSE
>Q8M9U4 | RK21_CHAGL
MSTYAIIDLGGKQLRVEPGRFYDAHLFSSFKSLLSESNTKIIIFRVLMISHGTEVQFGYP
WLKNASVKARILHKKQNDKMLIYKMRSKKKTRKKFGHRQKIARFIVDAIQYNGQTFTTNL
K
>Q8M9U6 | RK23_CHAGL
MIDKVKSPLLTEKSIRLLQKNQYTFQVNSDVNKTEFKKWIEIFFKVKVMSINSCRPPRKK
KRIGLISGYTVRYKKIIVTLKSGDSIPLFSI
>Q8M9U7 | RK2_CHAGL
MGIRLYKAYTPGTRNRSISDFKELKNNKPEKSLTSSYHKKQGRNNRGIITIRHRGGGHKK
LYRQIDFERNKFNIPGQVKSIEYDPNRNTRISLIHYEDGEKRYILAPRGLNIGDKIISSE
EADITIGNTLPLTKIPLGTAIHNIEIKPGKGGQLVRSAGTVAQLIAKEGLVATIRLPSGE
VRTIGKNCLATIGQLGNVDSNNRSFGKAGSKRWLGKKPTVRGVVMNPVDHPHGGGEGRAP
IGRKRPLTPWGRPALGKKSRKNHKYSDAFIIRRRK

>Q8M9U9 | RK22_CHAGL
MINNTNSNISIKAVGKKIRISPHKMRKVIDQIRGRSYEQALMILEFMPYKACNYVLKLLS
SVAANANHNFGLNKSDLFIEKIIADGGPKLKRFQPRAQGRGYPILKPTCNLMIVVKVKS
>Q8M9V1 | RK16_CHAGL
MLSPKRTKFRRHHRGRMKGISSRGNFIAFGKFALQALEPSWITSRQIEAGRRAMTRYARR
GGKIWIRIFPDKPVTMRAAETRMGSGKGSPEFWVAVVKPGRILYEMSGVSESIAKSAMRI
AAFKMPIKTQFLVKKDKV
>Q8M9V2 | RK14_CHAGL
MIQPQSYLNVADNSGARKIMCIRILGGSNKKYGHIGDTIIGVVKEAIPNMTLKKSEVVRA
VIVRTCKELKRKNGTILRFDENAAVIINQEGNPRGTRIFGPVARELREANFTKIVSLAPE
VL
>Q8M9V4 | IF1C_CHAGL
MKKQNLIEMEGIVTESLPNAMFRVSLDNGCQVLAHISGKIRRNYIRILPGDKVKVELSPY
DLTKGRITYRLRMKTTNG
>Q8M9V6 | RR11_CHAGL
MPKPSKKINLRKIKKKVPKGVIHIQASFNNTIVTITDVRGQVISWSSAGACGFKGAKKST
PFAAQTAAEKALRPLIDQGMRQAEVMISGPGRGRDTALRIIRKSGITLNFVRDVTPIPHN
GCRPPSKRRV
>Q8M9V7 | RPOA_CHAGL
MADQTISNVLNQPQWRCLESKIEKSTLHYGRFIISPLWKGQANTIGLALRRTLLSEVEGT
CITSVKIKNAIHEYSSLSGVQESVHDILINLKKIILSSSFSGIFEGFLSVVGPKTVLASD
LNLPSFIKIVNPDQYIATVNKPINFNLQIYIQKGKGYSLKNPISIKQGFFLVDPVFIPIR
SVNYSIHSFEDEKATKEFLIVEVWTNGSIEPKEAIKEASYNLVNLFTPLVSQEYQQLKNQ
LFKEDEKNKELNFIETNSNAISDNENSYNLYNQIFLDQLELSSRAYNSLKKNNINTISDL
LKYSYEDLLKIKNFGKKSADQVIEQLKKRFKIQL
>Q8M9Y6 | RK33_CHAGL
MAKGKDVRLVITLECTNCSQNPNKRFSGISRYTTSKNRRNTTNRLELKKFCPQCSVHTIH
KEIKK
>Q8M9Y7 | RR18_CHAGL
MKDLVNRSKRMSRRRLAPIRAGESIDYKNVGLLRRFISEQGKILSRRVNRLTAKQQRNMT
KAIKRARILALLPFLNNEN
>Q8MA11 | RPOC1_CHAGL
MFDKQKNRYIRVELASPDQIRNWAERTLPNGEIVGKVTKPYTLHYNSHKPEKDGLFCERI
FGPIKSGICACGKYHGFVKNLPEVKFCKQCGVEFTDSKVRRYRMGYIQLAYPATHIWYLK
RLPSHIATLLKMSLKEVESLVYCDLFLPRPIIKKPNLLKVKKLFNYDDQLWKETLPRFFS
TRSFESFQNREMATGGDAIHKRLSSLNLQKLIFQSYNEWEKLSLQRSTGNSYEDKKIQRM
KDIIIRRIKLAKQFFENNIKPEWMVLSVLPVLPPELRPMIEINQGELITSDLNELYRRVI
YRNNTLIGFLNKSNSTPAGLIIYQKRLVQEAVDALIDNRIGNQIMKDRNNRPYKSFSEII
EGKEGRFRQDLLGKRVDYSGRSVIVVGPSLSLHQCGIPKDMAIELFQPFIIRDLINCQLA
PNLRAARSMIQNQEPIIFKILEKIIKNHPILLNRAPTLHRLGIQAFQPILVEGLAIRLHP
LVCGGFNADFDGDQMAVHIPLSLEAQAEARFLMLANSNLLSPANGEPITVPSQDMLLGLY
VLTMSCKQGIYIYNEQKLKKKIPYFCNYIDVITAYEKKQINLHDPIWLKWYGNLRGTSST
KKQKLFEIQCESRGTRRKIFENWHFCENIRNRKFTIYLFTTPGRIIFNQLLEQSIQATVR
I

>Q8MA20 | MATK_DIOKA
MEEFKRYLELDRSQQHDFVYPLIFQEYIYALAHDHGLNRSIFLENTGYDNKSSLLIVKRL
ITQMYQRNHLIFCANDSNQNPFFGHNTNVYSQMLLEGFAVLVEIPFSLRLISSLKGKEIV
KSHNLRSIHSIFPFLEDKFSHLNYVLDILIPHSIHLEVLVQTLRYWVKDVSSLHLLRFFL
HEYRNWNSLITPKKSSFSFSKRNQRLFLFLYNSHVCEYESIFVFLRNQSSYLRSISSGTF
LERIYFYGKIEHFVEVFTKDFKAILWLFKDPFMHYVRYQGKSLLASKGTSLLMNKWKYYL
VNFWQCYFYMWSQPGRIHRNQLSNHSLDLLGYLSSVRLNPSIVRSQMLENSFLIGNAIKK
FDTIVPIIPLIGSLSKAKFCNVLGHPISKPVWSDLSDSDIIDRFGRIYRNLSHYYSGSSK
KMSLYRIKYILRLSXARTLARKHKSTVRAFLKRLGSELLEEFFTEEEQVFSLTFPKASFT
SRGLYRRRIWYLDIIXINDLANH
>Q8MA31 | MATK_CAMSI
MEEFKRYLELDRSQQHDFVYPLIFQEYIYALAHDHGLTRSIFLENIGYDNKFSLLIVKHL
IIQMYQQNHFLFSANDSNQNPFFGHNTNLYSQMILEGFVAVVEIPFSLFSLEGKEIVKSQ
NLRSIHSIFPFLEDKFSHLNYVLDILIPHSIHLEISVQTLRYWVKDASSLYLLRFFLHMY
WNWNSLITPKKSSFYFSKRNQRLFLFLYNFHICEYESIFVFLRKQSSHLRSISSGTFLER
RYFYGKIEHFLEVFTKDFQVILWLFKDPFIHYVRYQGKYILASKGTSLLMNKWKSYLVNF
WQCYFYMWSQPGRIHINQLSKHSPDFLGYLSSVRLNPSMVRSQMLENSFLIGNAIKRFDT
IVPIIPMIGSLSKAKFCNVLGHPISKPVWADLSNSDIIDRFGRIYRNLSHYHSGSSKKTS
LYRIKYILRLSCARTLARKHKSTVRAFLKRLGSELLEEFFMGEEQVFSLTFPSSTSQGLY
RRRIWYLDIVCINDLASHE
>Q8MA72 | MATK_BYBLI
MEEIQIYLQLERSQQHDFLYPLIFQEYIYAFAHDRGFNRSISSENLGYDNKFSFLIVKRL
ISRMYQQNHFFISLNDSNKNLFCARNKNFDSQIISEVFACIVEIPFSIPYINLEWKKKKI
VKPQNLRSIHSTFPFLEDNFSHLNLLVDILIPYPIHAEILVQTLRYWIKDASSLHLLRFF
LHEYCILNSFIIPKKASSSFSNFSKRNPKLFLFLYNSHVCEYESVFLFLRNQSSHLRSTS
SGVLLERIYFYRKIERLVNTFVKLKYFQPNLWFVKEPYIHYVSYQKIASLASKGTSLLMK
KWKCYFVTFWQWYFSLWFHPKRIYIKQLSNQSFYFLGYLSSVRMNFSVVRSQILGKSFLI
NNVIKKFDTLVPTIPMIASLAKAKFCNVLGHPISKPVWADLSDSHIIDRFWRICRNISHY
HSGSSKKKSLYRIKYILRLSCARTLARKHKSTVRTFLKKLGSELLKEFFRSEEDVFSLTF
HKASPAFWEVYRSRIWYLDIICLNDLGNPKSQF
>Q8MAI8 | RPOA_ELYHY
MVREEVAGSTQTLQWKCVESRVDSKRLYYGRFILSPLRKGQADTVGIALRRALLGEIEGT
CITRAKFGSVPHEYSTIAGIEESVQEILLNLKEIVLRSNLYGVRDASICVKGPRYITAQD
IILPPSVEIVDTAQPIANLTEPIDFCIDLQIKRDRGYQTELRKNYQDGSYPIDAESMPVR
NVNYSIFSCGNGNEKHEILFLEIWTNGSLTPKEALYEASRNLIDLFLPFLHAEEEGTSFE
ENKNRFTPPLFTFQKRLTNLKKNKKGIPLNCIFIDQLELTSRTYNCLKRANIHTLLDLLS
KTEEDLLRIDSFRMEDRKHIWDTLEKHLPIDLLKNKLSF
>Q8MAJ0 | RPOA_AEGUN
MVREEVAGSTQTLQWKCVESRVDSKRLYYGRFILSPLRKGQADTVGIALRRALLGEIEGA
CITRAKFGSVPHEYSTIAGIEESVQEILLNLKEIVLRSNLYGVRDASICVKGPRYITAQD
IILPPSVEIVDTAQPIANLTEPIDFCIDLQIKRDRGYQTELRKNYQDGSYPIDAVSMPVR
NVNYSIFSCGNGNEKHEILFLEIWTNGSLTPKEALYEASRNLIDLFLPFLHAEEEGASFE
ENKNRFTPPLFTFQKRLTNLKKNKKGIPLNCIFIDQLELTSRTYNCLKRANIHTLLDLLS
KTEEDLLRIDSFRMEDRKHIWDTLEKHLPIDLLKNKLSF

>Q8MC55 | MATK_PICSI
MDEFHRYGKEDSSWQQCFLYPLFFQEDLYAISHDHYLDGSSSSEPMEHLSSNDQFSFLTV
KRLIGQIRQQNHSIVLFVNCDPNPLVDRKKSSYSESVLEGLTLVLEVPFSIRSKYSVEGM
NEWKSFRSIHSIFPFLEDKFPHSNYVSDTRIPYSIHPEILVRTFRRWIGDAPSLHPLRSI
LYEYRNSSESLQRSIIVVPKVNTRFFLFLWNNYVYECESILVSLLKRSSHSRSLSHGSFP
QRTHFHRKIKNIFLFSRRNSLQSIWSLKDPNIHYVRYGERSIIAIKGTHLLVKKYRYYLP
IFRQCYFHLWNEPYRVCSHQLSKNCSSSLGYFMRVRMKPLLVKTKMLDELFIADLITDEF
DPIVPIVPIIGLLSREKFCDISGRPISKLSWTSLTDDDILDRFDRIWRNLFHYYSGSFGR
DGLYRIKYILSLSCAKTLACKHKSTIRVVRKELGPELFKKSFSKERELDSPPFSSKAAAR
SQRERIWHSDIPQINPLAHSWQKIQDLKIENLFDQ
>Q8MC99 | RPOA_PHAAN
MVQEKLRFSTRTLQWKCVESRIESKRLYYGRFILSPLMKGQADTIGIAIRRILLGEIEGT
CITRVKSEKIPHEYSTIIGIEESVHEIFMNLKEIVLKSNMYGTQDASISFKGPGYITAQD
IILPPSVEIVDNRQHIANVTEPVNLCIELKIERNRGYRIKTLKNFQDGSYDIDARFMPVR
NVNYSIHSYVNGNEKQEILFLEIWTNGSLTPKEALYEASQNLIDLFIPFLHAEEENFNLE
KKKHKVTLPLFTFHDILVKDKLRKNKKEIALKSIFIDQLELPPRIYNCLKRSNIHTLLEL
LNNSQEXLLKIEHFRVEDGKSILDILKIQKYFT
>Q8MCA4 | RK16_PHAAN
MLSNPQRTRFRKQHRGRMKGISYRGNHICFGRYALQALEPAWITSRQIEAGRRAMSRNVR
RGGQIWVRIFPDKPVTVRPTETRMGSGKGFPEYWVAVVKPGKILYEMGGVPENIARKAIS
IASSKMPIRTQFIISG
>Q8MCA5 | RR3_PHAAN
MGQKINPLGFRLGTTQSHDSIWFAQPTKYSENIQEDKKIRDWIKNYIQKNRRISSGVEGI
GEIKIQKRIDLIQVIIYMGFPKLLIEGKPQKIEELQTNMHKKLNCVNRKLNIAIVKVTNA
YKHPNILAEFIAGQLKNRVSFRKAMKKAIELTEQAGTKGVQVQIAGRIDGKEIARVEWIR
EGRVPLQTIRAKIEYCCYTVRTIYGVLGIKVWIFSK
>Q8MCK8 | MATK_TRIFG
MKEYQVYLERARSRQQDFLYPLIFREYIYGLAYSHNFNRSIFVENLGYDNKYSLLIVKRL
ITRMYQQNHLIISANDSNKNPFFGYNKNFYSQIISEGFAIVVEIPLFPELSSSLEEAEII
KSYKNVRSIHSIFPFLEDKFTHLNYVSDIRIPYPIHLEILVQILRYWVKDAPFFHLLRLF
LYNFCNWNSFITTKESISTFSKRNPRFFLFLYNFYVCEYESIFLFLRNKSSHLRLKSFSV
FFERIFFYAKREHLVEVFAKDFSYPLTFFKDPLIHYVRYQGKCILASKNAPFLMNKWKHY
FIHLWQGFFDVWSQPRTININQLSEHSFQLLGYFLNVRLNRSVVRSQMLQNTFLIEIVSK
KLDIIVPIMPLIRSLAKAKFCNVLGPPISKPVWADSSDFDIIDRFLRICRNLSHYYNGSS
KKKSLYQIKYILRLSCIKTLACKHKSTVRAFLKRSGSEELLEEFFTEEEEILSLIFPRDS
STLHRLNRNRIWYLDILFSNDLVNDE
>Q8MCM0 | MATK_TRISU
MKEYQVYLERARSRQQDFLYPLIFREYIYGLAYSHNWNRSIFVENGGYDNKYSLLNVKRL
ITRMYQQNHLIISANDSNKNPFLGYNKNFYSKIISEGFAIVVEIPLFLQLSSSLEEAEII
KSYKNVRSIHSIFPFLEDKFTYLNYVSDIRIPYPIHLEILVQILRYWVKDAPFFHLLRLF
LYHFCNWNRFITTKKSISTFSKSNPRLFLFLYNFYVCEYESIFLFLRNKSSHLRFKSFSV
FLERIFFYAKREHLVEVFSKDFSYPLPFFKDPNIHYVRYQGKCILASKNVPFLMNKWKHY
FIHLWQCFFDVWSQPRTININQLSEHSFQLLGYFSNVRLNRSVVRSQMLQNTFLIEIVSK
KLDIIVPIIPLIRSLAKAKFCNVLGHPISKPVWADSSDFDIMERFLRICRNLFHYYNGSS

KKKSLYRIKYILRLSCIKTLACKHKSTTRAFLKRSGSEELLEEFFTEEEEILSLIFPRDS
FTLRRFYRNRIWYLDILFRNDLVNDE
>Q8MCM2 | MATK_TRISO
MKEYRVYLERARSRQQDFLYPLIFREYIYGLAYSHNFNRSIFVENGGYDNKYSLLNVKRL
ITRMYQQNHLIISANDSNKNPFWGYNKNFYSQIISEGFAIVVEIPFFLQLSSSLEEAEII
KSYKNVRSIHSIFPFLEDKFTYLNYVSDIRIPYPIHLEILVQILRYWVKDVPFFHLLRLF
LYDFCNWNCFIPSKKSISTFSKSNPRLFLFLYNFYVCEYESIFLFLRNKSSHLRLKSFSV
FFERIFFYAKREHLVEVFSKDFSYTLPFFKDPNIHYVRYQGKCILASKNVPFLMNKWKHY
FIYLWQCFFDVWSQPRTININQLSEHSFQLLGYFSNVRLNRSVVRSQMLQNTFLIEIVSK
KLDIIVPIIPLIRSLAKAKFCNVLGHPISKPVWADSSDFDIIERFLRICRNLSHYYNGSS
KKKSLYRIKYILRLSCIKTLACKHKSTVRAFLKRSGSEELLEEFFTEEEEILSLIFPRDS
FTLHRFHRNRIWYLDIIFSNDLVNDE
>Q8MCM7 | MATK_TRIMI
MKEYRVYLERARSRQQDFLYPLIFREYIYGLAYSHNFNKSIFVENGGYDNKYSLLNVKRL
ITRMYQQNHLIISANDSNKNPFLGYNNNFYSQIISEGFAIVVEIPFFLQLSSSLEEAEII
KSYKNLRSIHSVFPFLEDKFTYLNYVSDIRIPYPIHLEILVQILRYWVKDVPFFHLLRVF
LYHFCNWNCFIPTKKSISTFSKSNPRLFLFLYNFYVCEYESIFLFLRNKSYHLRLKSFSV
FFERIFFYAKREHLVEVFSKDFSYTLPFFKDPNIHYVRYQGKCILASKNVPFLMNKWKYY
FIHLWQCFFDVWSQPRTININQLSEHSFQLLGYFSNVRLNRSVVRSQMLENTFLIEIVSK
KLDIIVPIIPLIRSLAKAKFCNVLGHPISKPVWADSSDFDIIERFLRICRNLSHYYNGSS
KKKSLYRIKYILRLSCIKTLACKHKSTVRAFLKRSGSEELLEEFFTEEEEILSLIFPRDS
FTLHRFHRNRIWYLDILFSNDLVNDE
>Q8MCN2 | MATK_TRIGC
MKEYRVYLERARSRQQDFLYPLIFREYIYGLAYSHNFNRSIFVENGGYDNKYSLLNVKRL
ITRMYQQNHLIISANDSNKNPFLGYNKNFYSQIISEGFAIVVEIPFFLQLSSSLEEAEII
KSYKNLRSIHSVFPFLEDKFTYLNYVSDIRIPYPIHLEILVQILRYWVKDVPFFHLLRLF
LYHFCNWNCFIPTKKSISTFSKSNPRLFLFLYNFYVCEYESIFLFLRNKSYHLRLKSFSV
FFERIFFYAKREHLVEVFSKDFSYTLPFFKDPNIHYVRYQGKCILASKNVPFLMNKWKYY
FIHLWQCFFDVWSQPRTININQLSEHSFQLLGYFSNVRLNRSVVRSQMLQNTFLIEIVSK
KLDIIVPIIPLIRSLAKAKFCNVLGHPISKPVWADSSDFDIIERFLRICRNLSHYYNGSS
KKKSLYRIKYILRLSCIKTLACKHKSTVRAFLKRSGSEELLEEFFTEEEEILSLIFPRDS
FTLHRFHRNRIWYLDILFNNDLVNDE
>Q8MCN8 | MATK_TRIRS
MKEYRVYLERARSRQQDFLYPLIFREYIYGLAYSHNFNRSIFVENGGYDNKYSLLNVKRL
ITRMYQQNHLIISTNDSNKNPFLGYNKNFYSQIISEGFAIVVEIPFFLQLSSSLEEAEII
KSYKNVRSIHSIFPFLEDKFTYLNYVSDIRIPYPIHLEILVQILRYWVKDVPLFHLLRLF
LYDFCNWNCFTPTKKSISTFSKSNPRLFLFLYNFYVCEYESIFLFLRNKSSHLRLKSFSV
FFERIFFYAKREHLVEVFSKDFSYTLPFFKDPNIHYVRYQGKCILASKNVPFLMNKWKHY
FIHLWQCFFDVWSQPRTIDINQLSEHSFQLLGYFSNVRLNRSVVRSQMLENTFLIEIVSK
KLDIIVPIIPLIRSLAKAKFCNVLGHPISKPVWADSSDFDIIERFLRICRNLSHYYNGSS
KKKSLYRIKYILRLSCIKTLACKHKSTVRAFLKRSGSEELLEEFFTEEEEILSLIFPRDS
FTLHRFHRNRIWYLDILFSNDLVNDE
>Q8MCR5 | MATK_LENER
MKESQVYLERARSRQQHFLYSLIFREYIYGLAYSHNLNRSLFVENVGYDNKYSLLIVKRL

ITRMYQQNHLIISANDSNKNSFWGYNNNYYSQIISEGFSIVVEIPFFLQLSSSLEEAEII
KYYKNFRSIHSIFPFLEDKFTYLNYVSDIRIPYPIHLEILVQILRYWVKDAPFFHLLRLF
LCNWNSFITTKNKKSISTFSKINPRFFLFLYNFYVCEYESIFVFLRNQSSHLPLKSFRVF
FERIFFYAKREHLVKLFAKDFLYTLTLTFFKDPNIHYVRYQGKCILASKNAPFLMDKWKH
YFIHLWQCFFDVWSQPRTININPLSEHSFKLLGYFSNVRLNRSVVRSQMLQNTFLIEIVI
KKIDIIVPILPLIRSLAKAKFCNVLGQPISKPVWADSSDFDIIDRFLRISRNLSHYYKGS
SKKKSLYRIKYILRLSCIKTLACKHKSTVRAFLKRSGSEEFLQEFFTEEEEILSLIFPRD
SSTLERLSRNRIWYLDILFSNDLVHDE
>Q8MCR7 | MATK_LATVE
MKEYQVYLERARSRQQDFLYPLLFREYIYGLAYSHNLNRSIFLENVGYDNKYSLLIVKRL
ITRMYQQNHLIISANDSTKNPFWGYNKNLDSQIISEGFAIVVEIPFLRQLSSSLEEAEIL
QSYQNWRSIHSIFPFLEDKLTYLNYVSDIRIPYPIHLEILVQILRYWVKDAPFFHLLRLF
LYNFSNWNSFLTTKKSISTFSKRNPRLFLFLHNFYVCEYEYIFVFLRTKSSHLRLKSFSV
FFERIFFDAKREHLVKVFSKDFSYTLTFFKDPNIHYVRYQGKCILASKNVPFLMNKWKHY
FIHLWQCFFDVWPQPRMININPLSEHSFQLLGYFLNVRLNRSVVRSQMLQNTFLIEIGIK
KLDIIVPILPLIRSLAKAKFXBILGEPISKPVWADSSDFDIIDRFLRICRNLSHYYNGSS
KKKSLYRIKYILRLSCIKTLACKHKSTVRAFLKRSGSEELLQEFFTEEXXILSLIFPXBS
STLQRNRIWYLDILFSNDLVHDE
>Q8MCR9 | MATK_LATSA
MKEYKVYLERARSRQQDFLYPLLFREYIYGLAYSHNLNRSIFLENVGYDNKYSLLIVKRL
ITRMYQQNHLIISANDSNKNRFWGYNKNLDSQIISEGFAIVVEIPFLRQLSSSLEEAEIL
QSYKNLRSIHSIFPFLEDKFTYLNYVSDIRIPYPIHLEILVQILRYWVKDAPFFHLLRLF
LYNFCNWNSFITTRKWISTFSKSNPRLFLFLHNFYVCEYESIFVFLRTKSSHLRFKSFSV
FFERIFFYAKREHLEKVFYKDFSYPLTFFKDLNIHYVRYQGKCILASKNAPFWMNKWKHY
FIHLWQCFFDVWSQPRMININPLSEHSFQLLGYFLNVRLNRSVVRSQMLQNTFLIEIVIQ
NLDIIVPIIPLIRSLANAKFCNILGEPISKPVWADSSDFDIIDRFLRICRNLSHYYNGSS
KKKSLYRIKYILRLSCIKTLACKHKSTVRAFLKRSGSEELLQEFFTEEEEILSLIFPRDS
STLQRLHRNRIWYLDILFSNDLVHDE
>Q8MD24 | MATK_LOACH
MEEFQRYFELNRYQQHNFLYPLIFQEYIAALAHDHGLNRSILLENTGYGNKFSLLSVKRL
ITQMYQQNHLIISANDSSQNQFFGHNKNLYSQMISEGFAVIVEIPFSLRLRSSLEGKEIV
KSQNLQSIHSIFPFLEDKFLHLNYVLDILIPYPIHLEILVQTLRHWVKDASSLHLLRFFL
HEYRNWNSLITPKKTSSSFSKRNQRLFFFLYNSHVCEYESIFIFILSQSSHLRSTSSGAL
LERIYFYGKIEHSVEVFAKDFPSILCLLKDPFIHYLRYQGKSILASKGTPLLMNKWKYYL
VNSWQCHFYVWSQSRRIYINQLSNHFLDFLGYLSSVRLNPSMVRSQMLENSFLIDNAIKK
FETIVPIIPLIGSLAKAKFCNVLGHPISKPVWADLSDSDIIDRFGRICRNLSHYHSGSSK
KKSLYRIKYILRLSCARTLARKHKSTVRAFLKMKRVGSELLEKFFTEEEQVFSLTFPRVS
STSTSRGLYRRRIWYLDIICINDLANHE
>Q8MEC7 | MATK_FILMA
MEELKGYLEKSRSKQQHFLYPLLFQEYIFVLAHDHGLNVNGSIFYEPAEISGYDKKFSSL
LVKRLITRMYQQNYLINSVNDSNQNRFVGHNKNFDSQMISEGFAVIVEIPFSLRLVSSLE
EKKEIQKSQNLRSIHSIFPFFEDKLSHLICVLDILIPYPVHLEILVQILQCWIQDVPSLH
LLRFFFHEYNNWSNLITPKKSNYYGFSKENPRLFLFLYNSYVVECESIFVFLRKQSSYLR
STSSGTFLERTHFHEKIEQHLVVLCCNDFQKTLWLFKDPFMHYVRYQGKSILASKGTRFL

MKKWKSYFVNFWQCHFHFWSQSCRIHINQFPNFSLHFLGYLSSVPINPSAVKSQMLENSF
LIDTVTKKFETLVPIISMIGSLSKAKFCNVSGNPISKPVWADLSDSDIIDRFGRICRNLS
HYYSGSSKKQSLYRIKYILRLSCARTLARKHKSTVRAFLQRLGSEFLEEFFTEEEKVLSL
ILPRISYPLHKLYRERIWYLDIIRINDLVNHL
>Q8MEC8 | MATK_ARITO
MEELRGYLEKSRSIQQHFLYPLLFQEYIYALAHNHGLNVNGSIFYEPPEISGYDKKFSSL
LVKRLITRMYQQNYLINSFNDSNQNGFVGHNKNFYSQMISEGFAVIVEIPFSLRLVSSLE
EKKEIPKSQNLRSIHSIFPFFEDKLSHLNCISDILIPYPVHLEILVQIIQCWIQDVPSLH
LLRFFFHEFYNWNNLITPKKSNYYGFSKENPRLFLFLYNSYVVECESILVFLRKQSSYLR
STSSGTFLERTHFYEKIEQHLVVLCCNDFKKTLWLFKDPFIHYVRYQGKSILASKGTHLL
MKKWKSYFVNFWQCHFHFWSQPSRIHISQFSNFSFYFLGYLSSVPLNPLAVKSQMLESSF
LIDTVTKKCETIVPIIPMIGSLSKAKFCNVSGNPISKPVWADLSDSDIIDRFGRICRNLS
HYYSGSSKKQSLYRIKYILRLSCARTVARKHKSTVRAFLQRLGSEFLEEFFMEEEKVLSL
ILPRISYPLHKLYRERIWYLDIIRINDLANHL
>Q8MEC9 | MATK_ANCDI
MEEFKGYLEKSRSKQQHFLYPFLFQEYIYALAHDHGLNVNGSIFYEPAEISGYDNKFSSL
LVKRLITRMYQQNYLINSVNDSNQNRFVGHNKNFYSQMISEGFAAIVEIPFSLRLASSLE
EKKEIPKSQNLRSIHSIFPFFEDKLSHLNYVSDILIPYPVHLEILVQILQCWIQDVPSLH
LLRFFFHEYHNWNNLITQKKSSYGFSKENPRLFWFLYNSYVVECESILVFLRKQSSYLRS
TSSGPFLERTHFYEKIGQHLIVLCCNDFQKTLWLFKDPFMHYVRYQGKSILASKRTHLLM
KKWKSYFVNFWQCHFHFWSQPCRIHINQFSNFSFYFLGYLSNVPINPSAVKSQMLENSFL
IDTVTKKFETIVPIIPMIGSLSKAKFCNVSGNPISKPVWADLSDSDIIDRFGRICRNLSH
YYSGSSKKQSLYRIKYILRLSCARTLARKHKSTVRAFLQRLGSEFLEEFFTEEEKVLSLI
LPRISYPLHKLYRERIWYLDIIRINDLVNHL
>Q8MED5 | MATK_AMOTI
MEELKGYLEKSRSKQQHFLYPLLFQEYIYALAHDHGLNVNGSFFYEPAEISGYDKKFSSL
LAKRLITRMYQQNYLINSVNDSNQNRFVGHNRNFYSQMISEGFAVIVEIXFSLRLVSSLE
EKKEIPKSQNLRSIHSIFPFFEDKLSHLNCVSDILIPYPVHLEILVQILQCWIQDVPSLH
LLRFFFHEYHNWNNLITPKKSNYYGFSKENPRLFLFLYNSYVVECESILVFLRKQSSYLR
STSSGTFLERAHFYEKIEQHLVVLCCNDFQKTLWLFKDPFMHYVRYQGKSILASKGTHLL
MKKWKSYFVNFWQCHFHFWSQPCRIHINQFSNFSFYFLGYLSSVPINPSAVKSQMLENSF
LVDTATKKFETIVPIIPMIGALSKAKFCNVSGNPISKPVWADLSDSDIIDRFGRTCRNLS
HYYSGSSKKQSLYRIKYILRLSCARTLARKHKSTVRAFLQRLGSEFLEEFFTEEEKALSL
ILPRISYPLHKLYRERIWYLDIIRINDLVNHL
>Q8MEE6 | MATK_AMOPO
MEELKGYLKKSRSKQQHFLYPLLFQEYIYAFAHDHGLNVNGSFFYEPAEISGYDKKFSSL
LVKRLITRMYQQNYLINSVNDSNQNRFVGHNKNLYSQMISEVFAVIVEIPFSLRLVSFLA
EKKEIPKSQNLRTIHSIFPFFEDKLSHLNCVSDILIPYPVHLEILVQILQCRIQDVPSLH
LLRFFFHEYHNWNNLITPKKSNYYGFSKENPRLFLFLYNSYVVECESIFVFLRKQSSYLR
STSSGTFLERAHFYEKIEQHLVVLCCNDFQKTLWLFKDPFMHYVRYQGKSILASKGTHLL
MKKWKSYFVNFWQCHFHFWSQPCRIHINQFSKFSFYFLGYLSSVPINPSAVKSQMLENSF
LVDTVTKKFETIVPIIPMIGALSKAKFCNVSGNPISKPVWADLSDSDIIDRFGRTCRNLS
HYYSGSSKKQSLYRIKYILRLSCARTLARKHKSTVRAFLQRLGSEFLEEFFTEEEKALSL
ILPRISYPLDKLYRERIWYLDIIRINDLVNHL

>Q8MEH7 | MATK_AMOBY
MEELKGYLEKSRSKQQHFLYPLLFQEYIYVLAHDHGLNVNGSIFYEPAEISGYDKNFSSL
LVKRLITRIYQQNYLINSVNDSNQNGFVGHNKNFYSQMISEGFAVIVEIPFSLRLVSSLE
GKKEIPKSQNLRSIHSIFPFFEDKLSHLNCVSDILIPYPVHLEILVQILQCWIQDVPSLH
LLRFFFHEYQNWNNLITPKKSNYYGFSKENPRLFLFLYNSYVVECESILVFLRKQSSYLR
STSSGTFLERAHFYEKIEQHLVVLCCNDFQKTLWLCKDPFMHYVRYQGKSILSSKGTHLL
MKKWKSYFVNFWQCHFHFWSQPCRIHINQFSNFSFYFLGYLSSVPINPSAVKSQMLENFF
LVDTVTKKFETIVPIIPMIGALSKAKFCNVSGNPISKPVWADLSDSDIIDRFGRTCRNLS
HYYSGSSKKQSLYRIKYILRLSCARTLARKHKSTVRAFLQRLGSEFLEEFFTEEEKALSL
ILPRISYPLHKLYRERIWYLDIIRINDLVNHL
>Q8MEW7 | MATK_CEDDE
MDEFHRYGKEDSSWQQCFLYPLFFQEDLYAISHDHYLDGSSSSEPMEHFSFNDQLSFLTV
KRLIGRIREQNHSIGLFVNCDPNPLVDRNKSSYFESVLEGLTLVLEVPFSTRSKYSVQGI
KEWKSFRSIHSIFPFLEEKFPHSNYILDTRIPYSIHPEFLVRTFRRWIQDAPSLHPLRSV
LYEYRNSPENLQRSIIVAPRVNTRFFLFLWNHYVYECESILVPLLKRSFQSRSSSHGSFP
ERTLFDRKIKHIIRISHRNSLKSIWSLKDPKIHYVRYGERFIIAIKGTHLLVKKCRYYLP
IFRQCYFHLWSEPYRVCSHQLSKNCSSFPGYSLRVRMKPLLVRTKMLGELFITDLITDEF
YPIVPIVSIIGLLAREKFCDISGRPISKLAWTSLTDDDILDRFDRIWRNFFHYYSGSFGR
DGLYRIKYILSLSCAKTLACKHKSTIRVVRKELGPELFKKSFSKEREFDSPAFSSKAVAR
SQRERIWHSDIPQINPLANSWQKIQDLKIKNLFDQ
>Q8MEW9 | MATK_WELMI
MGNFYRKNIEILGQQRFLYPLLFQDEFYAIAQNLFSNPRASVEVREEICKNCNSFSFFTV
KRLIFKIRKRNVFLESKNKNSISLALIAEGLTLGLDVFFSAQWKRFVDGEGGTEQLSFQS
IHSIFAYLDETTPYSFSSLGIRIPSYVHPELFIRMFNCLCWIDDVCFLHLLSSMLCFLKH
LTILDKFIFFNSKGFIRLVLFLWNIFVSKGESSKISLWKQKCYRAKFKSFGSFAEQTHFH
RKMKLKNPKIKAHEKFSEVFFFSHYSRFGEKSVLIGTPILIKKYRYFFCHFWQTSFFFSE
TYGLFVHEFSRKNISLIGYSFYFQNHRTFFRIKMFYDFFFTELVNNEFHPKFGIISIMKF
LSIEGFCDIMGRPISKLSWTCFTDDDIFDKCDRFWKILYYYYCGAKNKAYLDRIKYILLL
SCFKTIAFKHKSTIRVVRKEFDFELRKKFFPKEIEFERDFLCSRFAQKFKKWLLKINLAT
XRFWLLNILKVHFLTKSWHKDQDALDFCLIVEKNNILPMLNNFL
>Q8MEX3 | MATK_EPHSI
MCYFISKKKGLGGFFLKKNENIRWQQHFLYPLLFHNDFYVIDPNLLFNSSPSFEKIEKLP
NSFRFLNVKRSIKLLHQQNYLVYNTRSSCFNFIGKTFFFCFDIFVSTWWKHFFGFKVTFK
EINQQVNFQSVFSLFLFLEEVFMFSLSFSNIRIPSSIHSELLIRRFKFSIQDVSFLHFLS
FILFSKQFKFVNNSIIFPRGNVIFCFFLGNILLSIFEDFFTLRWKSCFHEKSLSYGLFSE
QKHFQQKWNFLTRKPKKKDTIRLLKDFFFHYIRYGEKLILLGTTILVKKCEFFFLNFWQT
YLFVLSEPSSFFLKQISSQNIFFLAYYLEYPTNSFLLRLNILDYFLSTDFVGRELNSKLS
AVFVIQFLSKEGLCDIMGNPKSKLAWLSFTDNSILDKYDHFCRNVDSFYSEAINKRFLDR
VKDILFLSCIKTLACKHKSTIRIVRKELGFELRKIFVRKQVEFKNKKLLYFCFHKQFRKL
LFKIDLVTERLWFLDILEVNNFTKFWVKQQNALDFFFIFDQNIWFMLDQFL
>Q8MEX4 | MATK_ZAMFL
MDKFQRDGKEYTSRQRRFLYPFLFKEDLYAIAIDHYFNRSSSFEPMENSSSNDRFSFLTV
KRSISRMRQQNGSIVPFVNCNQKKLVGYNRSFYSELVLGGLTAVPEVPFSIRSKHSLEGM
NEWTSFRSINSIFPLMEDKIPHSNFILDIRIPHLTHPEILVRTFRRWIQDAPFLHSLRFV

LHEHRNLIISSNLDQLILIASKENTRLSLFLWNSYAYECESLLVPLWKRFSHSRSLPYES
FVERTPFCRKIEHIIIFSHKYLKKSLWFLKDPSIHYVKYRERSIIALRGTYLLVKKWRYH
LTNFWQCHFHLWLQPYRIYIDELSNNCFSFLGYLLSVKMNTSVVRIKMLDDSFITDLITK
EFDPIAPTTLLIGSLAKEKFCDISGHPISRLAWTGLTDDDILDRFDRIWRNIFHYYSGSS
KKDGLYRMKYILRLPCAKTLACKHKSAIRVVRERFGSELFTKSFPKERESIFLSFSKTRS
QRERIWHSDIIQRNPLVNSWWKKHNFQIEPLFDR
>Q8MEX7 | MATK_MACCO
MDKLQRDGKEDTSRQRRFLYPLLFQEDFYTIAYDHCSNRSSSFEPMGNSSSNDRFSFLTV
KRSISRIRQQNGSIVPFVNCDQNKLVGHNKSFYSELVLGGLTAVSEVPFSIRSKHSLEGM
NEWASFRSINSIFPLMEDKIPHSNFILDIRIPHLTHPEILVRTFRRWIQDAPFLHSLRSV
LHEHRNLIISSNLDQLILIASKKNTRLSLFLWNYYAYECESLLVPLWKRFSHSRSLPYES
FIERTPFYRKIEHIVIFYHKYLKKSLWFLKDPSIHYVKYRERSIIALRGTYLLVKKWRYH
LTNFWQCHFHLWLQPYRIYIDELSNNCFSFLGYLLSVKMKTSVVRIKMLDDSFITDLITK
EFDPIAPTTLLIGSLVKEKFCDISGHPFSRLAWTGLTDDDILDRFDRIWRNIFHYHSGSS
KKDGLYRMKYILRLPCAKTLACKHKSAIRAVRERFGSELFTKSFPKERESIFLSFSKTRS
QRERIWYSDIIQRNPFVNS
>Q8MEX8 | MATK_LEPPR
MDKFQRDGKEDTSRQWRFLYPLLFQEDLYTIAYDHYSNRSSSLEPMGNSSSNDRFSFLTV
KRSISRIRQQNGSIVPFVNCDQNKLVGHNKSFYSELVLGGLTAIPGVPFSIRSKHPLEEX
NEWTSFRSIHSIFPLMEDKIPHSNFILDIRIPHLTHPEILVRTFRRWIQDAPSLHSLRSV
LHEHRNLIISSNLDQLILIASKENTRLSLFLWNYYAYECESLLVPLWKRFSHSRSLPYES
FIERTPFYRKIEHIAIFYHKYLKKSLWFLKDPSIHYVKYRERSIIALRGTYLLVKKWRYH
LTNFWQCHFHLWLQPYRIYIDELSNNGFSFLGYLLSAKMXPPVVKNKTVDDPFIPVLIXK
GFDPAAPVXFLIGSLVKEKFCDISGHPFSRLAWTGLTDDDILDRFDRIWRNIFHYHSGSS
KKDGLYHMKYILRLPCAKTLACKHKSAIRVVRERFGSELFTKSFPKERESIFLPFSKTRS
QRERIWHSDIIQRNPSVNPWWKEHNLRIEPLFDR
>Q8MEY0 | MATK_ENCAL
MDKLQRDGKEDTPRQRRFLYPLLFQEDLYAIAYDHYFNRSSSFEPMENSSSNDRFSFLTV
KRLISRIRQQNGSIVPFVNCDQTKLVGHNRSFYSELVLGGLTAVPEVPLSIRSKHSLERM
NEWTSFRSIHSIFPLMEDKIPHSNFILDIRIPHLTHPEILVRTFRRWIQDAPSLHSLRSV
LHEHRNLIISSNLDQLILIASKENTRLSLFLWNYYAYECESLLVPLWKRFFYSRSLPYES
FIERTPFYRKIEHIVIFYHKYLKKSLWFLKDPSIHYVKHRERSIIALRGTYLLAKKWRYH
ITKFWQCHFHLWPQPYRIYIDELSNNCFSFLGYLLSVKMKTSVVRIKMPDDSFITDLITK
EFDPIAPTTLLIGSLAKEKFCDISGHPISRLAWTGLTDDDILDRFDRIWRNIFHYHSGSS
KKDGLYRMKYILRLPCAKTLACKHKSAIRVVRERFGSELFTKSSPKERESIFLSFSKTRS
QRERIWHSDIIQINPLINSCRKKHNLQIEPLFDR
>Q8MEY1 | MATK_DIOSP
MDKLQRDGKEDTPRQRRFLYPLLFQEDLYAIAYDHYFNRSSSFEPMENSSSNDRFSFLTV
KRLISRIRQQNGSIVPFVNCDQTKLVGHNRSFYSELVLGGLTAVPEVPLSIRSEHSLERM
NEWTSFRSIHSILPLMEDKIPHSNFILDIRIPHLTHPEILVRTFRRWIQDAPSLHSLRSV
LHEHRNLIISSNLDQLILIASKENTRLSLFLWNYYAYECESLLVPLWKRFFYSRSLPYES
FIERTPFYRKIEHIVIFYHKYLKKSLWFLKDPSIHYVKHRERSIIALRGTYLLAKKWRYH
ITKFWQCHFHLWPQPYRIYIDELSNNCFSFLGYLLSVKMKTSVVRIKMPDDSFITDLITK
EFDPIAPTTLLIGSLAKEKFCDISGHPISRLAWTGLTDDDILDRFDRIWRNIFHYHSGSS

KKDGLYRMKYILRLPCAKTLACKHKSAIRVVRERFGSELFTKSSPKERESIFLSFSKTRS
QRERIWHSDIIQINPLINSCRKKHNLQIEPLFDR
>Q8RU60 | RBL_ATRBE
MSPQTETKASVGFKAGVKEYKLTYYTPEYQTKDTDILAAFRVTPQPGVPPEEAGAAVAAE
SSTGTWTTVWTDGLTSLDRYKGRCYRIERVVGEKDQYIAYVAYPLDLFEEGSVTNMFTSI
VGNVFGFKALRALRLEDLRIPPAYVKTFQGPPHGIQVERDKLNKYGRPLLGCTIKPKLGL
SAKNYGRAVYECLRGGLDFTKDDENVNSQPFMRWRDRFLFCAEALYKAQAETGEIKGHYL
NATAGTCEEMIKRAVFARELGVPIVMHDYLTGGFTANTSLAHYCRDNGLLLHIHRAMHAV
IDRQKNHGIHFRVLAKALRMSGGDHIHSGTVVGKLEGERDITLGFVDLLRDDFVEQDRSR
GIYFTQDWVSLPGVLPVASGGIHVWHMPALTEIFGDDSVLQFGGGTLGHPWGNPPGAVAN
RVALEACVKARNEGRDLAQEGNEIIREACKWSPELAAACEVWKEIVFNFAAVDVLDK
>Q8RXX5 | RK192_ARATH
MATSSHLLPQALHMIPRTPSFSSKNLGVSSILPRASSVNSRLSVSRVFLNHSSSNFGFAI
DSKKRKEFIAKAEESTEGETEAVVENAVETEAEGEGEATVAAEEAKPPWKTRVKLGDIMG
LLNKKAIEVAETVRPVPGLRTGDIVEIKLEVPENKRRLSIYKGIVMSRQNAGIHTTIRIR
RIIAGIGVEIVFPIYSPNIKEIKVVSHRKVRRARLYYLRDKLPRLSTFK
>Q8S8U0 | RK2B_ATRBE
MAIHLYKTSTPSTRNGTVDSQVKSNPRNNLIYGQRRCGKGRNARGIITARHRGGGHKRLY
RKIDFRRNEKDIYGRIVTIEYDPNRNAYICLIHYGDGEKRYILHPRGAIIGDTIVSGTEV
PIKMGNALPLTDMPLGTAIHNIEITLGKGGQLARAAGAVPKLIAKEGKSATLKLPSGEVR
LISKNCSATVGQVGNVGVNQKSLGRAGSKRWLGKRPVVRGVVMNPVNHPHGGGEGRAPIG
RKKPTTPWGYPALGRRSRKRNKYSDNLILRRRSK
>Q8S8U3 | RR15_ATRBE
MVKNSVISIIFQEEKRGSVEFQVFNFTNKIRRLTSHLELHKKDYLSQRGLKKILGKRQRL
LAYLAKKNRVRYKELINQLDIRETKTR
>Q8S8U7 | NU4C_ATRBE
MNYFPWLTIIVVFPIFAGSLIFFLPHKGNRVIRWYTICICILELLLTTYAFCYHFQLDDP
LIQLVEDYKWIDFFDFHWRLGIDGLSIGPILLTGFITTLATLAAWPITRDSRLFHFIMLA
MYSGQIGSFSSRDLLLFFIMWELELIPVYLLLAMWGGKKRLYSATKFILYTAGGSVFLLM
GVLGVALYGSNEPTLNFETLVNQSYPVVLEIIFYIGFFIAFAVKLPIIPLHTWLPDTHGE
AHYSTCMLLAGILLKMGAYGLIRINMELLPHAHSIFSPWLMIIGTIQIIYAASTSLGQRN
LKKRIAYSSVSHMGFIIIGISSLTDTGLNGALLQIISHGFIGAALFFLAGTTYDRIRLVY
LDEMGGIAIPMPKMFTMFSSFSMASLALPGMSGFVAELIVFFGIITGQKYVLIPKILITF
VMAIGMILTPIYSLSMSRQMFYGYKLFNAPKDSFFDSGPRELFLSISIFLPVIGIGIYPD
FVLSLAVDKVEVILSNFFYR
>Q8S8U9 | RK32_ATRBE
MAVPKKRTSTSKKRIRKNIWKRKGYWVALKAFSLAKSLSTGNSKSFFVRQTKINK
>Q8S8V4 | RK22_ATRBE
MLKKKKTEVYALGEHISMSADKARRVIDQIRGRSYEETLMILELMPYRACYPILKLVYSA
AANASYNMGSSEANLVISKAEVNGGTTVKKLKPRARGRSFPIKRSTCHITIVMKDISLND
EYVKMNSLKKTRWKKKSTAMTYHDMYNSGGLWDKK
>Q8S8V6 | RK16_ATRBE
MLSPKRTRFRKQHRGRMKGISYRGNHISFGKYALQALEPAWITSRQIEAGRRAMTRNARR
GGKIWVRIFPDKPVTLRPAETRMGSGKGSPEYWVAVVKPGRILYEMGGVTENIARRAISL

AASKMPIRTQFIIS
>Q8S8V7 | RK14_ATRBE
MIQPQTHLNVADNSGARELMCIRIIGASNRRYAHIGDVIVAVIKEAVPNMPLERSEIVRA
VIVRTCKELKRDNGMIIRYDDNAAVVIDQEGNPKGTRIFGAIARELRELNFTKIVSLAPE
VL
>Q8S8W2 | CLPP_ATRBE
MPIGVPKVPFRSPGEEDASWVDVYNRLYRERLLFLGQGINSEISNQLIGLMVYLSIEDET
KELYLFINSPGGWVIPGIAIYDTMQFVRPDVHTVCMGLAASMGSFILVGGEITKRLAFPH
ARVMMHQPASGYYEAQTGEFVLEAEELLKLRETLTRVYVQRTGKPLWVVSEDMEKDVFMS
ATEAQAYGIVDLVAVE
>Q8S8Y1 | RPOC2_ATRBE
MEVLMAERANLVFHNKAIDGTAMKRLISRLIEHFGMAYTSHILDQVKTLGFQQATATSIS
LGIDDLLTIPSKGWLVQDAEQQSLILEKHHHYGNVHAVEKLRQSIEIWYATSEYLRQEMN
PNFRMTDPFNPVHIMSFSGARGNASQVHQLVGMRGLMSDPQGQMIDLPIQSNLREGLSLT
EYIISCYGARKGVVDTAVRTSDAGYLTRRLVEVVQHIVVRRTDCGTARGISVSPRNGMMP
ERIFSQTLIGRVLADDIYMGPRCIATRNQDIGIGLVNRFITFRAQPISIRTPFTCRSTSW
ICRLCYGRSPTHGDLVELGEAVGIIAGQSIGEPGTQLTLRTFHTGGVFTGGTAEHVRAPS
NGKIKFNEDLVHPTRTRHGHPAFLCSIDLYVTIESEDILHNVNIPPKSLLLVQNDQYVES
EQVIAEIRAGISTLNFKEKVRKHIYSDSDGEMHWSTDVYHAPEFTYGNVHLLPKTSHLWI
LLGGPCKSSLVYLSIHKDQDQMNAHSLSGKQRYTSNLSVTNDQARQKLFSSGFSGKKEDR
IPDYSDLNRIICTGQYNLVYSPILHENSDLLSKRRRNKFIIPLHSIQELENELMPCSGIS
IEIPVNGIFRRNSILAYFDDPRYRRKSSGIIKYGTIETHSVIKKEDLIEYRGVKEFRPKY
QMKVDRFFFIPEEVHILPGSSSIMVRNNSIVGVDTQITLNLRSRVGGLVRVERKKKRIEL
KIFSGDIHFPGETDKISRHTGLLIPPGTGKINSKESKKVKKWIYVQRITPSKKKFFVLVR
PVVTYEITDGINFETLFPPDPLQERDNVQLRIVNYILYGNGKPIRGISDTSIQLVRTCLV
LNWNQDKKSSSCEEARASFVEIRTNGLIRHFLRINLVKSPISYIGKRNDPSGSGLLSDNG
SDCTNINPFSSIYSYSKAKIQQSLNQPQGTIHTLLNRNKECQSLIILSSANCSRMGTLKY
HSVIKDSIKKDPLIPIRNSLGPLGTCLPIENFYSSYRLITHNQILVTNYLQLDNLKQTFQ
VIKFKYYLMDENGKIFNPDPCRNIILNPFNLNWYFLHHNYCEETSKIISLGQFICENVCI
AKNGPPLKSGQVILVQVDSIVIRSAKPYLATPGATVHGHYGETLYEGDTLVTFIYEKSRS
GDITQGLPKVEQVLEVRSIDSISMNLEKRVEGWNKCITRILGIPWGFLIGAELTIAQSRI
SLVNKIQQVYRSQGVQIHNRHIEIIVRQITSKVLVSEDGMSNVFSPGELIGLLRAERMGR
ALEEAICYRVVLLGITRASLNTQSFISEASFQETARVLAKAALRGRIDWLKGLKENVVLG
GVIPVGTGFKGLVHPSKQHNNIPLETKKKNLFEGEMRDILFHHRKLFDSCLSKKFHDIPE
QSFIGFNDS
>Q8S8Y4 | PSBK_ATRBE
MLNTFSLIGICLNSTLYSSSFFFGKLPEAYAFLNPIVDIMPVIPLFFFLLAFVWQAAVSF
R
>Q8S8Y5 | RR16_ATRBE
MVKLRLKRCGRKQRAVYRIVAIDVRSRREGKDLRKVGFYDPIKNQTYLNVPAILYFLEKG
AQPTGTVQDILKKAEVFKELRPNQPKFN
>Q8S8Y6 | MATK_ATRBE
MEEIQRYLQPDSSQQHNFLYPLIFQEYIYALAHDHGLNINRSILLENPGYNNQFSLLIVK
RLITRMYQQNHFLISTNDSNKNTFLGCNKSLYSQMISEGFAFIVEIPFSLRLISSLSSFE

GKKILKSHNLRSIHSTFPFLEDNFSHLNYVLDILIPYPVHLEILVQTLRYWVKDASSLHL
LRFFLHEYWNLNSLITSKKPGYSFSKKNKRFFFFLYNSYVYECESTFVFLRNQSSHLRPT
SFGALLERIYFYGKIERLVEVFAKDLQVTLWLFKDPFMHYVRYQGKSILASKGTFLLINK
WKFYLVNFWQCHFSLCFHTGRIHINQLSNHSRDFMGYLSSVRLNPSMVRSQMLENSFIIN
NAIKKFDTLVPIIPLIGSLAKENFCTVLGHPISKPVWSDLSDSDIIDRFGRICRNLFHYY
SGSSKKKTLYRIKYILRLSCARTLARKHKSTVRTFLKRSGSELLEEFLTSEEQVLSLTFP
RASSSLWGVYRSRIWYLDIFCINDLANYQ
>Q8SE90 | MATK_ACEPS
MFQMKEYQIHLELDRSQQHNFLYPLLFREYIYALAHDHGLNRSTIPLENGGYDNKSSSLS
VKRLISRTYQRIHLSIYAKDSNPNQFIGHNNQFYSQMISEGFSVIVEIPFSLRLVAFLEG
KEKEMAKSHNFQSIHSIFPFFENNFSHLHYVLDVLIPYPIRPEILVRTFRYWVKDASSLH
LLRFFLHEYFNWNSLITPKKSNSIFSTSKPRFFLFLYNSHVYEYESIFFFLRNQSSHLRS
TSSGLLFERISFYGKVEDLVQVFVNDFQDNLWLFKHPIMHYVRYQGKSVLASKDMPLLMN
KWKYYLVNLWQWHFHVWSQPGRIHINHLYKDYIYFLGYLSRGRLNTLVVRSQMLENAFLI
DNAMKQFETTVPIIPLIGSLTTARFCNSLGHPISKPTWADSSDSYIIDRFMRICRNLSHY
HSGSSKKKSLYRIKYILRVSCVKSLVRKHKSTVRVFLKRLGSEFLEEFFTEEEHVLSLIF
PRAVFPSRRLYRGRVWYFDIICINDLVNHDKFEIFPN
>Q8SKV2 | MATK_ALPZE
MEELQGYLEEYRSRQQQFLYPLLFQEYIYVFAYDHGLNSSIFYEPQNSLGYDNKFSSVLV
KRLIIRMYQKNYLIYSVNDIYQNIFVGHNNYFYFHFFSQILSEGFAVIVEIPFSLQLISS
LEEKEIPKSHNLQSSHSIFPFLEDKLLHLNYLSDILIPYPAHMEILVQMLQSWIQDALSL
HLLQFLLHEYYNWNSLIIPNKSIYVFSKDNKRLFCFLYNLYIYEYEFLLVFPCKQSSFLR
LISSGVLLERIHFYVKIEHLGVCRIFCQKTLWIFKDPFIHYIRYQGKSILGSRGTHFLMK
KWKYHLVHFWQYYFHFWSQPYRIDTKKLSNYSFYFLGYFSSVQMNSSMVRNQMLENSFLM
DTLTKKLDTRIPIIPLIRSLSKAQFCTVSGYPISKPIWTDLADCDIINRFGRICRKLSHY
HSGSSKKQSLYRMKYILRLSCARTLARKHKSSARSFLQRLSSGLLEEFFTEEEQVISLIF
PKRTSFYLYGSYRERIWYLDIIRINDLVNSLLVTT
>Q8SM04 | MATK_PENHE
MEEIQRYLQLKRSQQHNFLYPLIFQEYIYAFAHDRSFSRSILSEKTGYEKKSSLLIVKRV
ITRMYQQNPFIIFLNDSTQNKFLGHNNNFYSQLISEGFAFIVEIPFFLRLISSREGKKKK
IVKSQNLRSIHSIFPFLEDSFSHLNFLLDILIPHPVHVEILVQTLRYWVKDASSLHLIRF
LLNEYCNSNCVFIPKKASSSFSNSKRNQRLFLFLYNSHVCEYESIFFFLRNQSSRLRSTS
SRVLLERIYFYGKIEHLVNVVVKVKDFQANLWLVKEPCMHYVRYQRKAILASKGTSLFMN
KWKCFLVTFWQWHFSLWFHPRRIYINQLSKNFLECLGYLSSVQMNPSVVRSQILENSFLI
NNAIKKFDTLVPIIPLIASLANTKFCNVLGHPISKPVWADLSDSHIIDRFGRICRNLSHY
HSGSSKKKSLYRIKYILRLSCARTLARKHKSTVRAFFKRLGSELLEEFLMSEEDVLFLTF
PKASSTLRGVSKSRIWYLDILCISDLVNYK
>Q8SM20 | MATK_ANTMA
MEEIQRYLQLDRSQQHDFLYPLIFQEYIYAFAHDRGFGRSILSENPGYDKKSSLLIVKRL
ITRMYQQNHFIGSTNDSNQNPFLGRNKNLYYQIISEGFAFIVEIPFSLRLISSREGKNKK
IVKSQNLRSIHSIFPFLEDNFSHLNFVLDILIPYPVHVEVLVQTLRYWVKDASSLHLVRF
FLNEYCNWNSLILPKKASFSCSKINQRLFLFLYNSHVCEYESIFVFLRNQSSHLLSTSSG
VLLERIYFYGKIECLVNVFVKVKDFQANLWLVKEPCMHYVRYQRKSILASKGTFLLIKKW
KCYLMTFWQWHFLLWFQPRRIYINQLSNHFLEFLGYLSNVRMNPAVVRSQILENSFLMNN

SIKKFDTLVPISPLIASLAKAKFCNVLGHPISKPVRADLSDSNIIDRFGHICRNLSHYHS
GSFKKKSLYRIKYILRLSCARTLARKHKSTVRAFLKRLGSDLLEEFLMSEEDVLSLTFPK
ASSTLRGGYKSRIWYLDIIYINDLANYK
>Q8SM90 | MATK_ACECA
MKEYQIHLELDRSQQNNFLYPLLFREYIYALAHDHGLNRSTIPLENGGYDNKSSSLSVKR
LISRTYQRIHLSIYAKDSNPNQFIGHNNKFYSQMISEGFSVIVEIPFSLRLVAFLEGKEM
AKSQNFQSIHSIFPFFENNFSHLHYVLDVLIPYPIRPEILVRTFRYWVKDASSLHLLRFF
LHEYFNWNSLITPKKSNSIFSTRNPRFFLFLYNSHVYEYESIFFFLRNQSSHLRSTSSGL
LFERISFYGKVEDLVQVFVNDFQDNLWLFKHPIMHYVRYQGKSVLASKDMPLLMNKWKYY
LVNLWQWHFHVWSQPGRIHINHLYKDYIDFLGYLSRGRLNTLVVRSQMLENAFLIDNAMK
QFETTVPIIPLIGSLTTARFCNSLGHPISKPTWADSSDSYIIDRFMRICRNLSHYHSGSS
KKKSLYRIKYILRVSCVKSLVRKHKSTVRVFLKRLGSEFLEEFFTEEEHVLSLIFPRALF
TSRRLYRGRVWYLDIICINDLVNHDKLEIVPN
>Q8W463 | RK191_ARATH
MAMSFHRLPQALHMIPRNPTQSSKNLGFSSFLSCAPSMDSRISVSRLSLNHPGSKFGFSL
DTRVRNEFIVRAEEGNTEAESEEFVAEIADTEGNVEEVVEAKPTRKPRIKLGDVMGILNQ
KAIEVAEKVRPVPEIRTGDIVEIKLEVPENKRRLSIYKGIVMSRQNAGIHTTIRIRRIIA
GIGVEIVFPIYSPNIKEIKVVSHRKVRRARLYYLRDKLPRLSTFK
>Q8W7A1 | MATK_CALPE
MEEFQGYLELDRSRQHDFLYPXLFREYIYALAHDHVLLNRSIFFENAGYYNKSSSIIVKR
LITRMYQQNXLIFSANDSIQNPFFGHNNNLYSQIFSEGFAVIVEIPFSLRLVSSLERKEI
AKSHNLRSIHSIFPFLEDKFSHLDYVLDVLMPYHIHLEILVQTLRYWVKDASSLHLLRFF
LHEYWNSLITPKKHIPIFSKGNPRLFLFLYNSHMCEYESILLFLRNQSSHLRSTSSGIFF
ERIYFYVKIEHFAKXFFDNDFQCILWFFKDPFMHYVKYQGKSILASKDTPLFMNKWKYYL
VNLWQYHFYAWFQPGRININQLCNYSIDFLGYRSSVRLNSSVVRSQMLENSFIINNAIKK
FETIVPIIPLIGSLSKANFCNTLGHPISKPTRADSSDSDIIDRFLRICRNLSHYHSGSSK
KKSLYRVKYILRLSCVKTLARKHKRTVRTFLKRLGSEFLEEFLTEEEVVLSLIFPRTYST
SRRLYRGQIWYLDITSINDLVNYE
>Q8W8E6 | MATK_FAGCR
MEEFQRYLELDRFRQHDFLYPLLFREYIYALAHDHGFNSYMLLENIGYDNKSSLLIVKRL
ITRMYQHNYLMISANDSNQNQFFGYNKNLHSQIISEGFAVIVEIPFSLRLISSFEGAEIV
ISYKLRSIHSIFPFLEDKLPHLNYTTDVRIPYPIHLEILIQTLRSRVKDASYLHLLRFFL
HQYSNWNILIIITTKSISIFSKSNPRFFLFLYNSHICQYESIFLFLGNQSSHLRLISSGVL
FERLYLHKKIEHFAEVFANDFPVIPCFLKDPFMHYVRYQGKSILASKDTPLLMNKWKYYL
VNLWQCHFYVWSHPGRIHINQLSKHSLDFWGYFSSVRLNPSVVRSQMLENSFLINNAPKK
LDIIVPIIPLIGSLAKARFCNALGHPISKLTRADLSDFEILNRFLRICRNLSHYYSGSSK
KKSMYRIKYILRLSCVKTLARKHKSTARAFLKKVGSEFVQEFFTEEEEFLSLIFPRTSFT
LRRLYRGRVWYLDIIFINGLANHE
>Q8W8V0 | RR7_PSINU
MSRQSETKKRTAKSDPIYRNRSVSMFINHILKDGEKSLAHKILYRAMKQIKRKTKKNPLS
VLRQAVYRVTPNVAVKSRRVGGSNYQVPVEVKPARGKALAIRWIVGASRNRSGRSMASKS
SYELIDAARNTGSAIRKKEETHKMAEANKAFAHLR
>Q8WHL6 | MATK_LEMGI
MEEFKGYLQKGGFKQQHFLYPLLFQEYIYGLAHDQGLNVNASTFNEPPEISGYGNKYSSL

LVKRLIIRIYQQNSFLYSVNNSKQNRFVGHNKNYYYKMICEGFSIVVEIPFSLRLVSSIK
ETKEIMKFQNLRSIHSLFPFLEDKFSHLNSVSDIVIPYPIHLEILLQILQCWIQDVPTLH
LLRLIFYDYHNRSNSITPNKSSYGFSKDNPRLYRFLYNSYVVECESIFYFLRKSSSYLRS
TSFRPLLERTHFYGKMKHIGVTCCNDFQKTLWLFKDPFMHYVRYQGKCIIASKGTHLLMK
KWKSYFVNLWQCHFHFWSQPSRIHINQFPHFSFYFLGYLSSVPINLSSAKSQMLENAFLI
DTFTPKFETMISIIPMIGSLAKAKFCNLSGNPISKPAWAELSDSDIIDRFGRIYRNLSHY
YSGSSKKQSLYRIKYILRLSCARTLARKHKSTVRAFLQRLGSEFFEEFFMEEEKVLSLIL
PRTYYPLHQLSREPIWYLDIIRINDLVNHFDL
>Q8WHM4 | MATK_LEMMN
MEDFKGYLQKGGFKQQHLLYPLLFQEYIYALAHDQGLNVNASTFNEPPEISGYDKKYSSL
LVKRLINRLYQQNTFIHSVNNSKDNRFVGHNKNFYYQMISEAFAIVVEIPFSRRLVSSLK
EKKEIPKYQNLRSIHSLFSFLEDKFSHLNYVSDILVPYPVHLEILVQILQCWIQDVPTLH
LLRLLFQDYHNGNNRITPNKSTYGFSKDNPRLYRFLYNSYVVEYEAIFVFLRKSSSYLRS
TSFGPLLERTHFYRKMKHIGVTCCNDFQKTLWLFKDALMHYVRYQGKSIMASKGNHLLMK
KWKSYFVNLWQCHFHFWSQPSRIHINQFPHFSFYFLGYLSSVPINPSSAKSQMLENSFLI
DTVTPKFETMISIIPMIGSLAKAKFCNLSGNPLSKPVWAELSDSDIIDRFGRIYRNLSHY
YSGSSKKQTLYRIKYILRLSCARTLARKHKSTVRAFLQRLGSEFFEEFFIEEDKILSLIL
PTTSYSLQQLSRESVWFLDIIRINDLVNHLDL
>Q8WHM8 | MATK_PISST
MEEFKGYFEKSGSIQQHFLYPLLFQEYIYALAHNHGLNVNGSIFYELPEISGYDKKFSSL
LVKRLITRVYQQNYLINSVNHSNPNRFVGHNKNFYSQMISEGFAVIVEIPFSLRLVSSLE
EKKEIPKSQNLRSIHSIFPFFEDNFSHLNWISDILIPYPDHLEMLVQILQCWIQDVPSLH
LLRIFFHEYHNWGNPITSRKSNYYGLSKENPRLFWSLYNPYVVKCESIFNFLRKQSSYLR
STFYGTILERTHFYGKMKPFGVTCANDFQKTLWLFKDPFMHYVRYQGKSILVSKGTHLLM
KKWKSYFVNFWQCHFRFWSQPGRIHISQFSKFSFYFLGYLSSVPINPSAVKSQMLESSFL
IDIVTKKFETIVPIIPIIGSLSKAKFCNVSGNPISKPVWADLSDSDIIDRFGRICRNLSH
YYSGSSKKKSLYRIKYILRLSCARTLARKHKSTVRAFLQRLGSEFLEEFFTEEEKVLSLI
LPRISYPLHKLYRERIWYLDIIRINNLTNHL
>Q8WHX3 | NDHH_PSINU
MAMLLKNKDQMIVSMGPHHPSMHGVLRLIVTLDGENVADCEPVLGYLHRGMEKIAENRTI
IQYLPYVTRWDYLATMFTEAITVNAPEKLANIRIPKRASYIRVIMLELSRIASHLLWLGP
FMADIGAQTPFFYILREREMIYDLFEAATGMRMMHNYFRIGGVAVDLPYGWIDKCLDFCD
YFSPKISEYEKLIVHNPIFLERVKGVGFISREEAINWGLSGPMLRASGVQWDLRKVDHYE
CYDEVDWQIQWQKEGDSLARYLVRIGEMRESVKILQQALRIIPGGPYENLEARRLHQSQN
LEWNDFDYQFMGKKSSPTFKLLKQEHYVRIEAPKGELGIFLIGNDSVFPWRWKIRPPGFI
NLQILPQLVRGMKLADIMPILGSIDIIMGEIDR
>Q8WHY2 | RR19_PSINU
MTRSLKKVPFVAHHLWKKIESLNIKKEKRVIITWSRASTIVPGMIGHTIAVYNGREHLPI
YVTDRMVGHKLGEFVLTRTFRGHARKDKKSRR
>Q8WHY3 | RK22_PSINU
MEKKKEVRALAKYVKMSAHKVRRVINQIRGRSYEEALISLEFLPYRACYPLLQLVSSAAA
NANNNLGLKKDTLLISEAKVDEASVSKRFQPRAQGRAYSIQKDTCHITIKIRERSK
>Q8WHZ0 | RR11_PSINU
MPKPIKRLSSHKKKRVIFKGIIQIKASFNNTIVTVTNSQGQVITWSSAGACGFKGTKRST

PFAAQIATENAIRTLISQGMKQAEVMISGPGPGRDTALRTIRKSGLVLHFVRDVTPLPHN
GCRPPKRRRV
>Q8WHZ7 | CLPP_PSINU
MPIGVPKVPFRLPGEEDAGWVDVYNRLYRERLLFLGQQVDDEIANQLIGIMMYLNGEDEN
RDMYSYINSPGGAVLPGISVYDAMQFVVPDVHTICMGLAASMGSFILTGGEITKRIALPH
ARVMIHQPASSYYDGQAGECIMEAEEILKLRDCITRVYAQRTEKPLWVISEDMERDIFMS
AKEARAYGIVDLIALEND
>Q8WHZ9 | RR18_PSINU
MKKSERSSRKRLSATVTSDLIDYKNIDLLRRFISERGKILSRRMTKLTSKQQRSVTVSIK
RARILALLPFINRDS
>Q8WI25 | RPOC1_PSINU
MIHKNNYQQLRIGLASPEQIRAWAERILPTGEIVGKVTQPYTLHYKTHKPERDGLFCERI
FGPIKSGFCACGNYQAVDNGKEFSSFCKQCGVEFTESRVRRYQMGYIELACPVTHVWYLK
RLPSYIANLLAKPLNDLESLVYCDITYPNLYLARPIAEKPTLLQLKGFFKYEDQSWRYLF
PRFFSTRGFEAFKSREIATGGDAVRKELASLNLKTVMECTYLEWKDLAKQKPTGNEWEDR
GIQRIKDFMVRRIRLVKHFLYTDVKPEWMVLSLLPVLPPELRPMIEVSGGLLLTSDLNEL
YRKVIYRNNTLIDFLLRSEFTPEGLIVCQKRLVQEAVDALIDNGIRGQPIRDSHNRPYKS
FSDIIEGKEGRFRENLLGKRVDYSGRSVIVVGPSLPLHRCGLPREMAIELFQAFVIRGLI
GRYLAQNLRDAKNMIQNKEPIIWKILRDVMQGHPVLLNRAPTLHRLGIQAFQPILIEGRA
IRLHPLVCGGFNADFDGDQMAVHVPLSLEAQAEARFLMLSHTNLLSPATGDPIAVPTQDM
LLGLHILTIEDSQGIYGVRYFPYSKRNCTSFFKIPYFNSYDDVLRAKEQRQIHFYSPIWL
RWKGFLRVITSINREFPIEIQYESSNISVHIYENYQIRKDKKGNRLSLYIRTTAGRVLFN
QQIEEAIQGTLKAF
>Q8WIJ9 | MATK_VACVI
MEEFKRYLELDRSQQHDFIYPLIFQEYIYALAHDRGLNRSIVFENRDYDNKSSLLIRKRL
ITXXXXXXXXXXXXXXXXKNKFLGYNTNFDSQMIFEGFAVVVEIPFYLQLLSSLEGKEIV
KSHNLRSLHSIFPXXXXXXXXXXXXXXXXXXXXXXMEILVRTLRYWVKDPSSLHLLRFFL
HEYPNRNSLITPKKYSFSFSKRNQKFFLFLYNFHVCEYESIFVFLRNQSSHLCSISFETF
LERILFYKKIELEVFVKDFKGILWVFKDPFLHYVRYRGKSILASNGSSLLMNKWKYYLVN
FWECYFSIWAQPRRIHINQLSNNSFDFLGYLSSVRLKPSMVRTQMIENSFLIENASKKFD
TLVPITPMIASLSKAKFCNVLGHPMNKXVWDGLSDSDIIERIGRLYXNLSHYYSGSLKKM
NLXRIKFILRILLCGTLASKHKGTLRSVLNRLGVGLLVEFITEEEQVFYLTFQKASSTSR
KLYQRRIWYLDIFCVNDTANHE
>Q8WIT2 | MATK_STEPS
MEEFKRYLELDRSQQHDFVYPLIFQEYIYALAHDHGLNRSILLENIGYDNKSSLLIVKRL
ITHLITQMYQQNHFLFSANDSNQNPFLGHNTNLYFQMILEGFAVVVEIPFSLRLRFSLEG
KEIVKSQNLRSIHSIFPFLEDKFSHLNYVLDILIPHSIHLEILVQTLRYWVKDASSLYLL
RFFLHEYRNWNSLITPKKSSFSFSKRNQRLFLFLYNFHICEYESIFVFLRNQSSHLRSIS
SGTFLERRYFYEKIEHFVEVFTKDFQAILWFFKDPFIHYVRYQGKSILASKGTSLLMNKW
RYYLVNFWQCYFYIWSQPGRIHINQLSKHSLDFLGYLSSVRLNPSMVRSQMLENSFLIGN
AIKKFDTIVPIIPMIGSLSKAKFCNVLGHPISKPVWADLSDSDIIDRFGRIYRNLSHYHS
GSSKKTSLYRIKYILRLSCARTLARKHKSTVRAFLKRLGSELLEEFFTGEEQVFSLTFPK
ASSTSRGLYRRRIWYLDIICINDLANHE
>Q8WIV4 | MATK_CYRRA

MEEFKRYLELDRSQQHDFIYPLIFQESIYALAHDHGLNRSLLLENEGYDNKSSLLIVKRL
ITHLITQMYQQNRFIFSANDSNQNHFLGHNTNLYSQIILEGFAVVVEIPFSLRLISSLEG
KEIVKSHNLRSIHSIFPFLEDKISHLNYVLDILIPHSIHLEILVQTLRYWVKDASSLHLL
RFFLHEYRNWNSLITPNKSSFSFSKRNQRLFLFLYNFHVCEYESIFVFLRNQSSHLRSIS
SGTFLERIYFYRKIEHFVFVEVFTKDFQTVLWLFKDPFLHYVRYQGKSLLAAKGASLLMN
KWKYYFVNFWQCYFSMWSQPRRIHINPLSNHSLDFLGYLSSMRLNPSMVRSQMLENSVLL
GNAIKKFDTTVPITPLIGSLSKAKFCNVLGHPLSKPAWADLSDSDIIDRFGCIYRNLSHY
HSGSLKKKSLYQIKYILRLSCARTLARKHKSTVRAFLKRLGVELLEEFFTEEEQVFYLTF
PKASSPSRGLYRIYRKRVWYLDIICINDLANHS
>Q8WIV8 | MATK_CAMSS
MEEFKRYLELDRSQQHDFVYPLIFQEYIYALAHDHGLTRSIFLENIGYDNKFSLLIVKHL
ITQMYQQNHFLFSANDSNQNLFFGHNTNLYSQMILEGFVAVVEIPFSLFSLEGKEIVKSQ
NLRSIHSIFPFLEDKFSHLNYVLDILIPHSIHLEISVQTLRYWVKDASSLYLLRFFLHMY
WNWNSLITPKKSSFDFSKRNQRLFLFLYNFHICEYESIFVFLRKQSSHLRSISSGTFLER
RYFYGKIEHFLEVFTKDFQVILWLFKDPFIHYVRYQGKYILASKGTSLLMNKWKSYLVNF
WQCYFYMWSQPGRIHINQLSKHSPDFLGYLSSVRLNPSMVRSQMLENSFLIGNAIKKFDT
IVPIIPMIGSLSKAKFCNVLGHPISKPVWADLSDSDIIDRFGRIYRNLSHYHSGSSKKTS
LYRIKYIXXXXXXXTLARKHKITVRAFLKRLGSELLEEFFTGEEQVFSLTFPSSTSQGLY
RRRIWYLDIVCINDLASHE
>Q8WJ31 | MATK_CHOSU
MEEFQRYLELDRSRQHDFLYPLLFREYIYALAHDHGLNKSILSENAGYGNKSSSIIVKRL
ITRMYQQNPLIFSANDSIQNQFFGHNKNLYSQIISEGFAVIVEIPFSLRLVSFLERKEIA
KSQNLQSIHSIFPFLEDKFSHLDYVSXVLIPYHIHLEILVQTLRYWVKDASSLHLLRFFL
HEYWNTLITPKKYITLFSKGNPRLFLFLYNSHICEYESIFLFLRNKSSHLRSTSSGIFFE
RIYFYVKIKHFVKVFFDNDFQCILWFFKDPFMHYVKYQGKSILASKXTPLLMNKWKYYLV
NLWQYHFYAWFQPGRININQLCXYSLDFLGYRSSVRLNSSVVXXQMLKNLFLINNAMKXF
ETIVPIIPLIGSLSKANFCNTLGHPISKPTRSDSSDSDIINRFLRICRNLSHYHSGSSKK
KSLYRVKYILRLSCVKTLARKHKITIRTFLKKSGSEFLEKFLTEEEVVLSLIFPRTYSTS
RRLYXZQSWYLDITSINDLVNYE
>Q8WJ37 | MATK_ANEAN
MEEFQGYFELDRSRQHDFLYPLLFREYIYALAHDHGLNRSILFENAGYDKKSSSIIVKRL
ITRMYQQNPLIFSANDSIQNPFFGHNKNLYSQIISEGFAVIVEIPFSLRLVSSLERKEIA
KSHNLRSIHSIFPFLEDKFSHLDYVSDVLIPYHIHLEISVQTLRYWVKDASSLHLLRFFL
HEYWNSLITPKKHITLFSKGNPRLFLFLYNSHICEYESIFLFLRNQSSHLRSTSSGIFFE
RIYFYVKIEHFAKVFFDNDFQCILWFFKDPFMHYVRYQGKSILALKDTPLLMNKWKYYLV
TLWQYHFYAWFQPGRIDINQLCKYSLDFLGYRSSVRLNSSVVRSQILENSFLINNAMKKF
ETIVPIIPLIGSLSKANFCNTLGHPISKPTRADSSDSDIIDRFLRICRNLSHYHSGSSKK
KSLYRVKYILRLSCVKTLARKHKRTVRTFLKRLGSEFLEEFLTEEEVVLSLIFPRTYSTS
RRLYRGQIWYFDITSINDLVNYE
>Q8WJP1 | MATK_PRUPE
MEEFQGYLELDRYQQHDFLYPLIFREYIYALAHDHGLNRSILLDNVGYDNKSSLLIIKRL
ISRMYKHNHFFISANASNQKKKLGYNKNLYSQKISEGFTVIVEISFSLRLVSSLEATETV
KSYNLRSIHSIFPFLEDKFPHLNYVSDVLIPYPIHLEILVQTLRYWVKDASSLHLLRLFL
HEYYNWNSLITSNNFFFSKSNPRLFLLLYNSHVCEYEFILLFLRNQSSHLQLTSSGIFFE

RIHFYEKIKYPVEKVFANDFPASILWFFKDPFMHYVRYQGKSILASKDTPLLMNKWKYYL
VNLWQCHSYVWSQPGRIYINKLSKHSLDFLGYFSSIRPNLSVVRSQMLENSFITDNAMKK
LDTLVPIIPLIGSLAKVKFCNALGHPISKSTWADSSDFDIIDRFLRICRNLSHYYSGSSR
KKSLYRIKYILRLSCLKTLARKHKSTVRTFLKRLGSKLLEEFFTEEEQILSLVFPRASYT
FTFKKLYRGRIWYLDIFCINDLINYE
>Q8WJP3 | MATK_PRUDU
MEEFQGYLELDRYQQHDFLYPLIFREYIYALAHDHGLNRSILLDNVGYDNKSSLLIIKRL
ISRMYKQNHFFISANDSNQKKKLGYNKNLYSQKISEGFTVIVEISFSLQLVSSLEATKTV
KSYNLRSIHSIFPFLEDKFPHLNYVSDVLIPYPIHLEILVQTLRYWVKDASSLHLLRLFL
HEYYNWNSLITSNNFFFSKSNPRLFLLLYNSHVCEYEFILLFLRNQSSHLQLTSSGIFFE
RIHFYEKIKYPVEEVFANDFPASILWFFKDPFMHYVRYQGKSILASKDTPLLMNKWKYYL
VNLWQCHSYVWSQPGRIYINKLSKHSLDFLGYFSSIRPNLSVVRSQMLENSFITDNAMKK
LDTLVPIIPLIGSLAKVKFCNALGHPISKSTWADSSDFDIIDRFLRICRNLSHYYSGSSR
KKSLYRIKYILRLSCLKTLARKHKSTVRTFLKRLGSKLLEEFFTEEEQILSLVFPRASYT
FTFKKFYRGRIWYLDIFCINDLINYE
>Q8WJR4 | MATK_ARUDI
MEEFQGYLEIDGYQQHDFLYPLIFREYIYALAHGLNRSILLDNVGYDNKSSLLIIKRLIS
RMYQQNHLIISANYYKQNKFFGYNKNLYSQIISEGFAVIVEIPFSLRSVSSLEATEKEII
KSYNLRSLHSLFPFLEDKFPHLNYVSDVLIPYPIHLEILVQTLRYWVKDSSSLHLLRLFL
HEYYNWNSLITPNKFIFSKSNQRLFLLLYNSHVCEYESILLFLRNQSSHLPLTSYGIFFE
KIHFYEKIKYPGDEVFSNDFMVSILWFFKDPFMHYVRYQGKSILASKDTPLMMNKWKYYL
VNLWQCHYYVWSQPGRIYINQLSKHSLYFLGYFASMQPNLSVVRSQMLENSFIMDNAMKK
LDTLVPIIPLIVSLAKVKFCNALGHPISKSTWTDSSDFDIIDRFVRICRNISHYYSGSSR
KKSLYRIKYILRLSCVKTLARKHKSTVRTFLKRLGSKLLEEFFTEEELIRSLIFPRTSYS
LKKFYRGRIWYFDIFCINDLVNHE
>Q8WJR5 | MATK_ADEFA
MEEFQGYLELDKYQQHDFLYPLIFREYIYALAYDHGLNRSILLDNVGYENKYSLLIIKRL
ISRMYKQNNFIISANDSNQNKFLGYNKNLYSQMISEGFAVIVEIPFSLRLVSSLEATEIV
KSYNLRSIHSIFPFLEDKFSHLNYVSDVLIPYPIHLEILVQTLRYWVKDPSSLHLLRLFL
HEYYNLNSLITPNKFIFSKSNPRLFLLLYNSHVCEYESILLFIRNQSSHLRLTSSGIFFE
RIHFYEKRKYPGEEVFSNDFPSAILWFFKDPFMHYVRYQGKSILASKDSALLMNKWKYYL
VNLWQCHSYVWSQPGRICINQLSKHSIYFLGYFSSMRPNLSVVRSQMLENSFIMDNAMKK
FDTLVPIIPLIRSLAKVKFCNTLGHPISKSAWADSSDFDIIDRFVRICRNLSHYYSGSSR
KKSLYRIKYILRLSCVKTLARKHKSTVRTFLKRLGSKLLEEFFTEEQQILSLIFPRASYT
LKKFYRGRIWYLDIFCINDLANHE
>Q8WJV2 | MATK_DRACH
MYLNGRITRIFRLKKNSFRQQNFLYPLLLQEYIYSLAHYNSFNSLISYEPVEIIGYDNKS
SLVLVKRLITRMYQQKSLISSLNDSNQNEFWGHKNSFSSHFSSQMVSEGFGVILEIPFLS
RLVSSLEEKRIPKYQNLRSIHSIFPFLEDKLSHLNYVSDLLIPHPIHLEILVQILQCWIK
DVPSLHLLRLFFHEYHNLNSLITSKKAIDVFSKRKKRFFWFLHNSYVYECEYLFLFLRKQ
SSYLRSISSGVFLERTQFYGKIEYLIIRCCNSFQKILWVLKDTFIHYVRYQGKAILASER
TLILMNGWKFHLVNFWQSYFHFWVQPYRIHINQLPNYSFSFLGYFSSVRKNPLVVRNQML
ENSFLINTLNNKLDTIVPAISLIGSLSKAQFCTVLGHLISKPIWTDLSDSAIRDRFCRIC
RNLCRYHTGSSKKQVLDRIKYILRLSCARTLARKHKSTVRTFMRRLGSGFLKEFFLEEEQ

SPSLIFLQKISFILHGLHRERIWYLDIIRINDLVDHS
>Q8WKE7 | MATK_FAGJA
MEEFQRYLELDRFRQHDFLYPLLFREYIYALAHDHGFNSYMLLENIGYDNKSSLLIVKRL
ITRMYQHNYLMISANDSNQNPFFGYNKNLHSQIISEGFAVIVEIPFSLRLISSFEGAEIV
ISYKLRSIHSIFPFLEDKLPHLNYTTDVRIPYPIHLEILIQTLRSRVKDASYLHLLRFFL
HQYSNWNILIITTKSISIFSKSNPRFFLFLYNSHICQYESIFLFLGNQSSHLRLISSGVL
FERLYLHKKIEHFAEVFANDFPVIPCFLKDPFMHYVRYQGKSILASKDTPLLMNKWKYYL
VNLWQCHFYVWSHPGRIHINQLSKHSLDFWGYFSSVRLNPSVVRSQMLENSFLINNAPKK
LDIIVPIIPLIGSLAKARFCNALGHPISKLTRADLSDFEILNRFLRICRNLSHYYSGSSK
KKSMYRIKYILRLSCVKTLARKHKSTARAFLKKVGSEFVQEFFTEEEEFLSLIFPRTSFT
LRRLYRGRVWYLDIIFINGLANHE
>Q8WKL8 | MATK_POPNI
MKIEKSQRNLEIDRSRKNDFLYPLIFREYIYTFAHDRDLNRSILLENVSYDNKYSLLIVK
RLITRMYQQNHLIISANDSNQNTFFRYNKNLYFQMISEGFAVIVEIPFSLRLVSSLERSE
IVKSHNLRSIHSIFPFLEGKFPHLNYLSEGLIPYPIHLEKLVQILRYWVKDPSSLHLLRL
FLHEYWNLNSLIIPKKSISFFVKKNQRFFLFLYNSHVYEYESVFFFLCKQSFHFRLTFYQ
VFLERIDFYGKIEHFVEVFTKDWGDSLCLLKDPFIHYIRYQGKSIFVSKDTPLLMKKWKY
YLVNLCQCHFDVCFQPQKIHINPFSLYKHSFALLGYLSSSSVRLNLSVVRSQMLENAFLM
DNIMNKLDTTVSIIPLIGSLAKMKFCNAVGHPISKPTWADFSDSDIIDRFVRICRNLSHY
YSGSSRKKSLYRIKYILRLSCVKTLARKHKSTVRIFLKRLGSELLEEFFTEEEQIIFLIF
PRASSISQKLYRGRVWYLDIICINELSNHE
>Q8WKN0 | MATK_ACEPM
MKEYQIHLELDRSQQHNFLYPLLFREYIYTLAHDHGLNRSTIPLENGGYDNKSSSLSVKR
LISRTYQRIHLSIYAKDSNPNQFIGHNNQFYSQMISEGFSVIVEIPFSLRLVAFLEGKEK
EMAKSHNFQSIHSIFPFFENNFSHLHYVLDVLIPYPIRPEILVRAFRYWVKDASSLHLLR
FFLHEYFNWNSLITPKKSNSIFSTSNPRFFLFLYNSHVYEYESIFFFLRNQSSHLRSTSS
GLLFERISFYGKVEDLVQVFVNDFQDNLWLFKHPIMHYVRYQGKSVLASKDMPLLMNKWK
YYLVNLWQWHFHVWSQPGRIHINHLYKDYINFLGYLSRGRLNTLVVRSQMLENAFLIDNA
MKQFETTVPIIPLIGSLTMARFCNSLGHPISKPTWADSSDSYIIDRFMRICRKLSHYHSG
SSKKKSLYRIKYILRVSCVKSLVRKHKSTVRVFLKRLGSEFLEEFFTEEEHVLSLIFPRA
LFTSRRLYRGRVWYLDIICINDFVNHDKFEIVPN
>Q8WKP4 | RK14_CUCSA
MIQPQTLLNVADNSGARELMCIRIIGASNRRYAHIGDVIVAVIKKAVPNTPLERSEVIRA
VIVRTCKELKRENGMIIRYDDNAAVVIDQEGNPKGTRIFGAIARELRQLNFTKIISLAPE
VL
>Q94KR7 | IF1C_SOYBN
MFTSLHTPILHPRYCHHPTPSCTQFSPLALPPFHRTLSFLAPPPLLPAAPALSAASAAKP
DKSGEQKWVHEGLIMESLPNGMFRVRLDNEDLILGYISGKIRKNYVRILPGDRVKVEVTR
YDSSKGRIVYRLRSSTPS
>Q94PL3 | IF1C_MONCA
MKEQKWIHEGLITESLPNGMFRVRLDNEDLILGYVSGKIRRSFIRILPGDRVKIEVSRYD
STRGRIIYRLRNKDSKD
>Q95BX6 | MATK_ULMPA
MAKFQGYLELDRFWKHDFLYPLIFREYIYAFAHDRGLNKSSLLENVGYDNKSSLLIVKRL

ITRMYQQNHLILVAHDSNQNNVFWSNKNLYSQLISEGFAVIVEIPFSIRLVSSLKGTEIV
KYSNLRSIHSIFPFLEDKFPYLNSVSDVLIPYPIHLEILVQILRYWVKDASSLHLLRLFL
HDYYNWNSLIISNKSISIFFKSNSRFFLFLYNSHVCEYESILLFIRNQSCHLRLTSSGSF
FERIYFYEKIKHPIEEVFSNDFPAIPLFFQDPFMHYVRYQGKSILVSKDTPLLMNKWKYY
LVHLWQCHFYVWSEPGRIHINQLSKHSLFFLGYLSSMRLNLSVVRSQMLENSFLMDNAMK
KIDTLVPISPLIGSLAKMKFCNALGHPISKSTWADSSDLDIIDRFVRIWRNLFHYYSGSS
KKKSLYRIKYILRVSCVKTLARKHKSTVRAFLKRLGSELLEEFFMEEEEVLSFIFPRTYY
TLRRLYRGRIWYLDIFCINDFVNHE
>Q95DP2 | MATK_NICBE
MEEIQRYLQPDRSQQHNFLYPLIFQEYIYALAHDHGLNRNKSILLENPGYNNKFSFLIVK
RLITRMYQQNHFLISTNDSNKNSFLGCNKSLYSQMISEGFAFIVEIPFSLRLISSLSSFE
GKKVFKSHNLRSIHSTFPFLEDNFSHLNYVLDILIPYPVHLEILVQTLRYWVKDASSLHL
LRFFLHEYWNLNSLITSKKPGYSFSKKNQRFFFFLYNSYVYECESTFVFLRNQSSHLRST
SFGALLERIYFYGKIERLVEVFAKDFQVTLWLFKDPFMHYVRYQGKSILASKGTFLLMNK
WKFYLVNFWQCHFSLCFHTGRIHINQLSNHSRNFMGYLSSVRLNPSMVRSQMLENSFLIN
NAIKKFDTLVPIIPLIGSLAKANFCTVLGHPISKPVWSDLSDSDIIDRFGRICRNLFHYY
SGSSKKKTLYRIKYILRLSCARTLARKHKSTVRTFLKRSGSELLEEFLTSEEQVLSLTFP
RASSSLWGVYRSRIWYLDIFCINDLANYQ
>Q95DR2 | MATK_NICPA
MEEIQRYLQPDRSQQHNFLYPLIFQEYIYALAHDHGLNRNRSVLLENPGYNNKFSLLIVK
RLITRMYQQNNFLISTNDSNKNEFLGCNKSLYSQMISEGFAFIVEIPFSLRLISSLSSFE
GKKIFKSHNLRSIHSTFPFLEDNFSHLNYVLDILIPYPVHLEILVQTLRYWVKDASSLHL
LRFFLHEFWNLNSLITSKKPGYSFSKKNQRFFFFLYNSYVYECESTFVFLRNQSSRLRST
SFGALLERINFYGKMERLVEVFTKDLQVTLWLFKDPFMHYVRYQEKSILASKGTFLLMNK
WKFYLVNFWQCHFSLCFHTGRIHINQLSNHSRDFMGYLSSVRLNPSMLRSQMLENSFLIN
NAIKKFDTLVPIIPLIGSLAKANFCTVLGHPISKPVWSDLSDSDIIDRFGRICRNLFHYY
SGSSKKKTLYRIKYILRLSCARTLARKHKSTVRTFLKRSGSELLEEFLTSEEQVLSLTFP
RASSSLWGVYRSRIWYLDIFCINDLANYQ
>Q95E84 | MATK_SCHTR
MEEFQRYIELDRSWQHNFVYPLIFQEYIYGFAYDHGLNKYILLENAGDKKYSLLIVKRLI
NRMYQQTHWILFANHSNQNDFFGHKHKKNLYYQIISEGFAVIVEIPFSPLLISSLEAKEK
KIVKSHNLRSIHSIFPFFEDKFLHLNYVLEILIPYPIHLEILVQTLRYWVKDASSLHLLR
FFLYEYRNWNSLITPQKSISIFSKRNQRLFLFLYNFHVCEYESIFVFLCNQSSHLRSTSF
GALLERNYFYGKLEYLVKVFTFTKDFCVILWLLKDPFLHYVRYRGKSILASKGTPLLMHK
WKYFLFNFWQCHFSLWSPPRRIYINRLSKHSLDFMGFFSSVRLNSSVVRSQMVENSFLID
NPIKKFDTIVRIIPLVGSLAKAKFCNVLGHPISKSVWTDLLDSDIMDRFGRICRNLSHYY
SGSSRKKSLYRIKYILRLSCARTLARKHKSTVRAFLKRLGSEFLEEFFTEEEKVLSLILP
RDSSISRGLYRGPFWYLDIICIHDLANDE
>Q95EA5 | MATK_BROHE
MEEFQRYIELDRSWQHNFFYPLIFQEYIYGFAYDHGLNKSILLENAGDKKYSLLIVKRLI
NRMYQQTHLIISANHSNQNDFFGHKHKKNLYYQIISEGFAVIVEIPFSLLLISSLEAKEK
KIVKSHNLRSIHSIFPFFEDKFLHLNYVLEILIPYPIHLEILVQTLRYWVKDASSLHLLR
FFLYEYRNWNSLITPQKSISIFSNQRLFLFLYNFYVCEYESIFVFLCNQSSHLRSTSFGA
LLERNYFYGKLEYLVKVKTNTKDFCVILWLFKDPFLHYVRYRGKSILASKGTSLLMHKWK

YYLFNFWQCHFSLWSPPRRIYINRLSKHSLDFMGFFSSVRLNSSVVRSQMVENSFLIDNP
IKKFDTIVRIIPLVGSLAKAKFCNVLGHPISKSVWTDLLDSDIIDRFGRICRNLSHYYSG
SSRKKSLYRIKYILRLSCARTLARKHKSTVRAFLKRLGSEFLEEFFTEEEKVLSLILPRD
SSISRGLYRGPFWYLDIICIHDLANDE
>Q95ED6 | MATK_MAIPO
MEEFQRYIELDRSWQYNFFYPLIFQEYIYGFAYDHGLNKSILLENAGDKKYSLLIVKRLI
TRMYQQNHLSLSANHSNQNDFFGHKHKKNLYYQIISEGFAVIVEIPFSLLLISYLEAKEK
KIVKSHNLQSIHSIFPFFEDKFLHLNFLLEILIPYPIHLEILVQTLRYWVKDASSLHLLR
FFLYEYRNWNSLITPQKSISFFSKRNQRLFLFLYNFYVCEYESIFLFLCNQSSHLRSTSF
GALLERIYFYGKLEYLVKVKTFTNTQDFRVILWLFKDPFLHYVRYRGKSILASKGTSLLM
HKWKYYLINFWQCHFSLWSQPRRIYINRLSKHSLDFMGFFSSVRLNSSVVRSQMVENSFL
IDNPIKKFDTIVRIIPLVGSLAKAKFCTVLGHPISKSVWTDLLDSDIIDRFGRICRNLSH
YYSGSSRKKSLYRIKYILRLSCARTLARKHKSTVRAFLKRLGSEFLEEFFTEEEKVLSLI
LPRDSSISRGLYRGPFWYLDIICIHDLANYE
>Q95ED9 | MATK_OPUQU
MEEFQRYIELDRSWQHNFFYPLIFQEYIYGFAYDHGLNKSILLENAGHKKYSLLIVKRLI
TRMYQQNHLILSANHSNQNDFFGHKHKKNLYYQIISEGFAVIVEIPFSLLLISSLGAKEK
KIVKSHNLRSIHSIFPFFEDKFLHLNYVLKILIPYPIHLEILVQTLRYWVKDASSLHLLR
FFLYEYRNWNSLITPQKYISIFSKKNQRLFLFLYNFHVCEYESIFVFLCNQSSHLRSTSF
GALLERIYFYGKLEYLVKVKTFTKDFRLILWLFKDPFLHYVRYRGKSILASKGTSLLMYK
WKYYLINFWQCHFSLWSQPRRIYINRLSKHSLDFMSFFSSVRLNSSVVRSQMVENSFLID
NPIKKFDTVVRIIPLVGSLAKAKFCNVLGHPVSKSAWTDLLDSDIIDRFGRICRNLSHYY
SGSSRKKSLYRIKYILRLSCARTLARKHKSTVRAFLKRLGSEFLEEFFTEEEKVLSLILP
RNFSISRGLYRGPLWYLDIICIHDLANDE
>Q95EE5 | MATK_GRABR
MKEFQRYIELDRSWQHNFFYPLIFQEYIYGFAYDPDLKKSILLENAGDKKYSLLIVKRLI
TRMYQQQNHLILFANHSNQNDFWGHKHTNNLYYQIISEVFAFIVEIPFSLLLISSLESKK
RKIVKSHNLRSIHSIFPFFEDKFLHLNYVLEILIPYPIHLEILVQTLRYWMKDASSLHLL
RFFLYEYRNWNSLITSQKSISIFSKRNKRLFLFLYNFHVCEYESIFVFFCNQSSHLRSTS
FGVLLERIYFYGKLEYLVKVFPFTKDFRLILWLFKEPFPHYVRYRGKSILASKGTSLLMH
KWKYYLINFWQCYFSLWSQPTRIYINQLSKHSLDFMGFFSSVQLNSSVVRSQMVENSFLI
ENTMKKFDTIVRIIPLVGSLAKANFCNVLGHPISKSVWTDLLDSDIIDRFGCICRNLSHY
YSGSSRKKTLYRIKYILRLSCARTLARKHKSTVRAFLKRLGSEFLEEFFTEEEKVLSLIL
PRDSSIARGFYRGPIWYLDIICIHDLANDE
>Q95EF3 | MATK_KOKDR
MEQFQVYLELNRSRRHDFLYPLIFRESIYALAHGHGLNKSMIFFENQGYGNKFSSLIVKR
LILRMDQQKRLISSANDSNQNPVFGHNNNLYSQMVAAGFAVIVEIPFSLRLISYSQGAEV
AKSHNLQSIHSIFPFLEDKLSHLNYVLEALIPHPIHLEILVQALRYWVKDASSLHLLRFS
LYEYCNLKSFITPKKSISIFNPRLFLFLYNSHACEYEFIFLFLRNQSSHLRSTSSGVFLE
RIFFYGKIKYLGEVFYNDFQNNLWLFKDPFIHFIRYQGKSILASKDTSLLINKWKYYFVD
LWQYYFYLWSQSGRVRINQLSKYSLDFLGYLSSVRLNPSVVRSQMLENSFLIDNAMKTLD
TRIPIISLIGSLSKAKFCNTLGHPISKPTWADSPDSDIIDRFVRICRNLSHYHSGSSKKK
SLYRIKYILRFSCVKTLARKHKSTVRAFLKKLGSEFLEEFFTETEEEHAFSLIFPRGFFA
LRKFDRGRIWYLDIICIDALVNHS

>Q95EF8 | MATK_GOSGO
MEEFQVYLELNRSRRHDFLYPLIFREYIYALAHEHGLNKSMIFFENQGYGNKFSSLIVKR
LILRMDQQNRLISSANDSNQNPVFGHNNNLYSQMIAAGFAVIVEIPFSLRLISYSQGAEA
AKSHNFQSIHSIFPFLEDKFSHLNYVLEALIPHPIHLEILVQALRYWVKDASSLHLLRFS
LYEYCNLKSFITPKKSISIFNPRLFLFLYNSHTCEYESIFLFLRNQSSHLRSTSSGVFLE
RIFFYGKIKYLGEVFYNDFQNNLWLFKDPFIHFIRYQGKSILASKDTSLLINKWKYYFVD
LWQYYFYLWSQSGRVRINQLSKYSLDFLGYLSSVRLNPSVVRSQMLENSFLIDNAVKTLD
TRIPIISIIGSLSKAKFCNTLGHPISKPTWADSPDSDIIDRFVRISRNLSHYHSGSSKKK
SLYRIKYILRFSCVKTLARKHKSTVRAFLKKLGSEFLEEFFTETEEEHVFSLIFPRGFFA
LRKVYRGRIWYLDIICINALVNHS
>Q95GM3 | IF1C_BOROF
MKEEKWIHEGLITESLPNGMFRVRLDNEDLVLGYVSGKIRRSFIRILPGDRVKIEVSRYD
STRGRIIYRLRNKDSKD
>Q95GM7 | IF1C_CORMA
MKEQKWINEGLITESLPNGMFRVRLDNEDLSLGYVSGKIRRSFIRVLPGDRVKIEVSRYD
STRGRIIYRLRNKDSKD
>Q95GN5 | IF1C_ASACA
MKEQKLIHEGLIIESLPNGMFRVRLDNEDLILGYVSGRIRRSFIRILPGDRVKIEVSRYD
STRGRIIYRLRNKDSND
>Q95GN7 | IF1C_ILLPA
MKEQKWIHEGLITESLPNGMFRVRLDNVDNEDLILGYVSGRIRRSFIRILPGXRVKIEVS
RXDSTRGRIIYRLRNKDSNB
>Q95GT2 | MATK_NEPAL
MEELRGYLELDRSWQRDFLYTLILQEYIYSLAHDHGFNRTIFLENAGYEKKYSFLIVKRL
ITRMYQQNHLILSANDSNQNEFLGQKKNLYSQMISEGFAFIVEIPFSLQLLFSLEGKEIX
XXXXXRSIHSIFPFLEDKFSHLNYVLDILIPHPVHLEILVQTIRYWTKDASSLHLLRFFL
YEYRNWNSRISIKQYISFFSNRNQRLFLFLYNSHVCEYESIFIFLRNQPFHLRSTFSGAF
LERILFYEKMEHLVKVFTKNFQVILWFFKDTFIHYVRYQGKSFVASKGTSLLMIKWKYYL
VNFWQCYFSVWSQPRRIYINQLSNHSLDFMXFLSSVRLNPSVVRIQMFEKSFIIDNAINT
FDTLVPNIHMSGSFAKEKFCNIFGHPISKPVWADLSDSDIIDRFGRICRSLSHYYSGSSR
KKSLYRIKYILRLSCARTLARKHKSTVRTFLKRLGSEFLEEFFMEEEKVLSLILPRDSYT
SQRFYRGRIWYLDIFCIHDLANHE
>Q95GT7 | MATK_NEPDI
MEELRGYLELDRSWQRDFLYTLILQEYIYSLAHDHGFNRTIFFENAGYEKKYSFLIVKRL
ITRMYQQNHLILSANDSNQNEFLGQKKNLYSQMISEGFAFIVEIPFSLQLLFSLEGKEIV
KSRNLRSIHSIFPFLEDKFSHLNYVLDILIPHPVHLEILVQTIRYWTKDASSLHLLRFCL
YEYRNWNSRISRKQYISFFSNRNQRLFLFLYNSHVCEYESIFIFLRNQPSHLRSTFSGAF
LERIYFYEKIEHLVKVFTKNFQVILWFFKDTFMHYVRYQGKSFLASKGTSLLMIKWKYYL
VNFWQCSFSVWSQPRRIYINXLXNHXLDFMXFLSSVRLNPSVVRIQMLEKSFIIDNAINT
FDTRVPNIPMIGSFAKAKFCNIFGHPISKPVWAYLSDSDIIDRFGRICRSLSHYYSGSSR
KKSLYRIKYILRLSCARTLARKHKSTVRTFLKRLGSEFLEEFFMEEEKVLSLILPRDSYT
SQRLYRGRIWYLDIFCIHDLANHE
>Q95H44 | NU6C_WHEAT
MDLPGPIHEILMLFGGFVLLLGGLGVVLLTNPIYSAFSLGLVLVCISLFYFLLNSYFVAV

AQLLIYVGAINVLIIFAVMFVNGSEWSKDKNYWTIGDGFTSLVCITIVFSLMTTIPDTSW
YGILWTTRSNQIVEQGLINNVQQIGIHLATDFYLPFELISIILLVSLIGAITMARQ
>Q95H50 | RK16_WHEAT
MLSPKRTRFRKQHRGRMKGKSCRGNRICFGRYALQALEPAWITARQIEAGRRAITRYARR
GGKIWVRIFPDKPVTLRPTETRMGSGKGSPEYWVSVVKPGRILYEMGGVSETVARAAISI
AASKMPIRSQFIRLEI
>Q95H51 | RK14_WHEAT
MIQPQTLLNVADNSGARKLMCIRVIGAAGNQRYARIGDVIVAVIKDALPQMPLERSEVIR
AVIVRTCKEFKCEDGIIIRYDDNAAVIIDQKGNPKGTRVFGAIAEELRGLNFTKIVSLAP
EVL
>Q95H56 | RK33_WHEAT
MAKGKDVRIRVILECISCVRKGANEESTGISRYSTQKNRHNTPGQLEFKKFCRYCRKHTT
HHEIKK
>Q95H60 | NDHJ_WHEAT
MQQGWLSNWLVKHEVVHRSLGFDHRGIETLQIKAGDWDSIAVILYVYGYNYLRSQCAYDV
APGGSLASVYHLTRIQYGIDNPEEVCIKVFAQKDNPRIPSVFWIWRSADFQERESYDMVG
ISYDNHPRLKRILMPESWIGWPLRKDYITPNFYEIQDAH
>Q95H61 | RR4_WHEAT
MSRYRGPRLKKIRRLGALPGLTRKTPKSGSNLKKKFNSGKKEQYRIRLQEKQKLRFHYGL
TERQLLRYVHIAGKAKRSTGQVLLQLLEMRLDNILFRLGMASTIPGARQLVNHRHILVNG
RIVNIPSFRCKPRDIITTKDNQRSKGLVQNYIASSDPGKLPKHLAIDTLEYKGLVNKILD
RKWVGLKINELLVVEYYSRQT
>Q95H62 | RR14_WHEAT
MAKKSLIQREKKRQKLEQKYHLIRQSLKKKIRSKVSPLSLSEKTKMREKLQSLPRNSAPT
RLHRRCFLTGRPRANYRHFGLSGHVLREMVYECLLPGATRSSW
>Q9AWA5 | GWD1_SOLTU
MSNSLGNNLLYQGFLTSTVLEHKSRISPPCVGGNSLFQQQVISKSPLSTEFRGNRLKVQK
KKIPMEKKRAFSSSPHAVLTTDTSSELAEKFSLGGNIELQVDVRPPTSGDVSFVDFQVTN
GSDKLFLHWGAVKFGKETWSLPNDRPDGTKVYKNKALRTPFVKSGSNSILRLEIRDTAIE
AIEFLIYDEAHDKWIKNNGGNFRVKLSRKEIRGPDVSVPEELVQIQSYLRWERKGKQNYP
PEKEKEEYEAARTVLQEEIARGASIQDIRARLTKTNDKSQSKEEPLHVTKSDIPDDLAQA
QAYIRWEKAGKPNYPPEKQIEELEEARRELQLELEKGITLDELRKTITKGEIKTKVEKHL
KRSSFAVERIQRKKRDFGHLINKYTSSPAVQVQKVLEEPPALSKIKLYAKEKEEQIDDPI
LNKKIFKVDDGELLVLVAKSSGKTKVHLATDLNQPITLHWALSKSPGEWMVPPSSILPPG
SIILDKAAETPFSASSSDGLTSKVQSLDIVIEDGNFVGMPFVLLSGEKWIKNQGSDFYVG
FSAASKLALKAAGDGSGTAKSLLDKIADMESEAQKSFMHRFNIAADLIEDATSAGELGFA
GILVWMRFMATRQLIWNKNYNVKPREISKAQDRLTDLLQNAFTSHPQYREILRMIMSTVG
RGGEGDVGQRIRDEILVIQRNNDCKGGMMQEWHQKLHNNTSPDDVVICQALIDYIKSDFD
LGVYWKTLNENGITKERLLSYDRAIHSEPNFRGDQKGGLLRDLGHYMRTLKAVHSGADLE
SAIANCMGYKTEGEGFMVGVQINPVSGLPSGFQDLLHFVLDHVEDKNVETLLERLLEARE
ELRPLLLKPNNRLKDLLFLDIALDSTVRTAVERGYEELNNANPEKIMYFISLVLENLALS
VDDNEDLVYCLKGWNQALSMSNGGDNHWALFAKAVLDRTRLALASKAEWYHHLLQPSAEY
LGSILGVDQWALNIFTEEIIRAGSAASLSSLLNRLDPVLRKTANLGSWQIISPVEAVGYV
VVVDELLSVQNEIYEKPTILVAKSVKGEEEIPDGAVALITPDMPDVLSHVSVRARNGKVC

FATCFDPNILADLQAKEGRILLLKPTPSDIIYSEVNEIELQSSSNLVEAETSATLRLVKK
QFGGCYAISADEFTSEMVGAKSRNIAYLKGKVPSSVGIPTSVALPFGVFEKVLSDDINQG
VAKELQILMKKLSEGDFSALGEIRTTVLDLSAPAQLVKELKEKMQGSGMPWPGDEGPKRW
EQAWMAIKKVWASKWNERAYFSTRKVKLDHDYLCMAVLVQEIINADYAFVIHTTNPSSGD
DSEIYAEVVRGLGETLVGAYPGRALSFICKKKDLNSPQVLGYPSKPIGLFIKRSIIFRSD
SNGEDLEGYAGAGLYDSVPMDEEEKVVIDYSSDPLITDGNFRQTILSNIARAGHAIEELY
GSPQDIEGVVRDGKIYVVQTRPQM
>Q9BA08 | RR15_OENHO
MVKKAFISVISQEENRGSVEFQVVSFTNKIRRLTSHLEFHRKDFLSQRGLRKILGKRQRL
LSYLSKKDKVRYTELISQLDIRELTTR
>Q9BBN0 | RPOA_MARQU
MLKDGTSVSNEVIQWKCVEFRIESKRLHYGRFVISPFKKGQANTVGIAMRRALLGEVGGA
SITSARFEGVAHEYSTVAGIQETIHDILVNLKEIVLRSDSDGNQKAILSVTGPKRVTAGD
ISLPPSVKVIDDSQYIVTITQPISVNIELNIECDCGYRIESLNEYRDGEFPVDAVFMPVR
NVNYSVHPFGSGKEMREILFIEVWTNGSLTPTEAISKASKSSIDLLSPFLHTKHEDIPDF
ESNRDSSSLMKFSSRVDDVDKSEGDFFKNTFIDQLELPARAFNCLKRAEIHTISDLLSYS
RDDLLKLKSFGKKSVDQVSRALWERFATELPNEKPRVVGDE
>Q9BBN7 | RR15_LOTJA
MVKKSFIPVISQEKKEENPGSVEFQVFNFTNKIRRLTSHFELHRKDYLSQRGLRKILGKR
QRLLSYLSKKNKIRYKKLINLLDIRESKIR
>Q9BBN8 | NDHH_LOTJA
MNVPATRKDLMIVNMGPHHPSMHGVLRLIVTLDGEDVIDCEPILGYLHRGMEKIAENRTI
IQYLPYVTRWDYLATMFTEAITVNGPEQLGNIQVPKRASYIRVIMLELSRIASHLLWLGP
FMADIGAQTPFFYIFREREFIYDLFEAATGMRMMHNFFRIGGVAADLPHGWIDKCFDFCN
YFFTRVVEYQKLITRNPIFLERVEGVGVVGGEEVINWGLSGPMLRASGIQWDLRQVDNYE
CYEEFDWEVQWQKEGDSLARYLVRIGEMMESIKIIQQALEGIPGGPYENLEIRCFGREKE
PEWNDFEYRFIGKKPSPTFELPKQELYVRVEAPKGELGIFLIGDQNGFPWRWKIRPPGFI
NLQILPQLVKRMKLADIMTILGSIDIIMGEVDR
>Q9BBP3 | NU4C_LOTJA
MNYFPWLTLVVILPIAGGSLIFLFPHRGNKVIRWYTVCICLIDLLLTTYAFCYHFQLDDP
LIQLTESYKWINFFDFYWRFGIDGLSIGPILLTGFITTLATLAAQPITRECKLFYFLMLA
MYSGQIGPFSSRDILLFFIMWELELIPVYLLLAMWGGKKRLYSATKFILYTAGSSVFLLM
ATLGIGLYGSNEPTLNFETLNNQSYPLALEIIVYIGFLIAFAVKSPIIPLHTWLPDTHGE
AHYSTCMLLAGILLKMGAYGLVRINMELFSHAHSIFCPWLMILGSIQIIYAASASLGQRN
LKKRIAYSSVSHMGFLIIGIGSISDTGLNGAILQIISHGFIGAALFFLSGTSYDRLRLLY
LDEMGGMALPMPKIFTVFTILSMASLALPGMSGFFAELIVFWGIITSQKYFLIMKILITF
VTAIGMILTPIYSLSILRQMFYGYKFFNTPNSYFFDSGPRELFISISILIPIIGIGIYPD
FIFSFSVDKVEAVLSHF
>Q9BBP9 | RK16_LOTJA
MLSPKRTRFRKQHRGRMKGISHRGNQICFGRYALQALEPAWITSRQIEAGRRAMSRNVRR
GGQIWVRIFPDKPVTVRPTETRMGSGKGSPEYWVAVVKPGKIVYEMGGVAENIARKAISI
AASKMPIRTQFIISG
>Q9BBR0 | RK20_LOTJA
MTRIKRGYIARKRRTKIRLFTSSFRGAHSRLTRTISQQKIKALVSAHRDRNRKKREFRGL

WISRINAGIGDNDKKKNIYYSYSNFMYNLYKKQLLLNRKIVAQIAIFKGNCLFMIANEII
T
>Q9BBR1 | RR18_LOTJA
MDKSKRLFLKSKRFFRRRLPPIQSGDRIDYKNMSLISRFISEQGKILSRRVNRLTLKQQR
LITIAIKQARILSSLPFINNEKKQFEKSELTATRTTTVFKTKKR
>Q9BBR2 | RK33_LOTJA
MAKGKDIRIIVILECTGCDQKSVNKESSGISRYITQKNRHNTPSRLELIKFCPCCRKHMI
HAEIKK
>Q9BBS6 | RR2_LOTJA
MTKRYWNITFEEMMEAGVHFGHGTRKWNPKMAPYISMKRKGIHIINLTRTARFLSEACDL
VFDAASRGKRFLIVGTKKKAADLVARAAIRARCHYVNKKWLGGMLTNWYTTETRLRKFRE
LRTEQKTGKLNCLPKRDAAILKRQLSHLETYLGGIKYMTGLPDIVIIVDQQEEYTALREC
ITLGIPTICLIDTNCDPDLADISIPANDDAIASIRLILNKLVFAICEGHSSYIRNF
>Q9BBS7 | RPOC2_LOTJA
MAERANLVFHNKVIGGTAIKRIISRLIDHFGMAYTSHILDQVKTLGFRQATTTSISLGID
DLLTIPSKGWLVQDAEQQSLILEKHHHYGNVHAVEKLRQSIEIWYATSEYLRQEMNPNFS
MTDPFNPVHIMSFSGARGNASQVHQLVGMRGLMSDPQGQMIDLPIQSNLREGLSLTEYII
SCYGARKGVVDTAVRTSDAGYLTRRLVEVVQHIVVRRTDCGTVRGISVNTRNRMMSERIL
IQTLIGRVLADDIYIGSRCIVVRNQDIGIGLINRFINFQTQPIFIRTPFTCRNTSWICRL
CYGRSPIHGNLVELGEAVGIIAGQSIGEPGTQLTLRTFHTGGVFTGGTAEYVRSPSNGKI
KFNENSAYPTRTRHGHPAFLCYIDLYVTIESNDIMHNVIIPPKSFLLVQNDQYVKSEQII
AEIRAGTYTLNLKEKVRKHIFSDSEGEMHWSTNIYHVSEFAYSNVHILPKTSHLWILSGN
SHKSDTVSLSLLKDQDQMSTHSLPTAKRNTSNFLVSNNQVRLCPDHCHFMHPTISPDTSN
LLAKKRRNRFIIPFLFRSIRERNNELMPDISVEIPIDGIIHKNSILAYFDDPQYRTQSSG
IAKYKTIGIHSIFQKEDLIEYRGIREFKPKYQIKVDRFFFIPQEVHILSESSSIMVRNNS
IIGVNTPITLNKKSRVGGLVRVEKNKKKIELKIFSGDIHFPGEIDKISQHSAILIPPEMV
KKKNSKESKKKTNWRYIQWITTTKKKYFVLVRPVILYDIADSINLVKLFPQDLFKEWDNL
ELKVLNFILYGNGKSIRGILDTSIQLVRTCLVLNWNEDEKSSSIEEALASFVEVSTNGLI
RYFLRIDLVKSHISYIRKRNDPSSSGLISYNESDRININPFFSIYKENIQQSLSQKHGTI
RMLLNRNKENRSFIILSSSNCFQMGPFNNVKYHNGIKEEINQFKRNHKIPIKISLGPLGV
APQIANFFSFYHLITHNKISSIKKNLQLNKFKETFQVIKYYLMDENERIYKPDLYNNIIL
NPFHLNWDFIHPNYCEKTFPIISLGQFICENVCIVQTKNGPNLKSGQVITVQMDFVGIRL
ANPYLATPGATIHGHYGEMLYEGDILVTFIYEKSRSGDITQGLPKVEQVLEVRSIDSISM
NLEKRIDAWNERITGILGIPWRFLIGAELTIAQSRISLVNKIQRVYRSQGVHIHNRHIEI
IVRQITSKVLVSEDGMSNVFSPGELIGLLRAQRTGRALEESICYRTLLLGITKTSLNTQS
FISEASFQETARVLAKAALRGRIDWLKGLKENLVLGGIIPVGTGFKKIGDRSSARQDTKI
TLETIKIIRGRN
>Q9BBS8 | RPOC1_LOTJA
MIDQYKHQQLRIGSVSPQQISAWATKILPNGEIVGEVTKPYTFHYKTNKPEKDGLFCERI
FGPIKSGICTCGNYRVIGDKKDEPKFCEQCGVEFVDSRIRRYQMGYIKLACPVTHVWYLK
RLPSYIANLLDKPLKELEGLVYCDFSFARPVVKKPTFLRLRGLFEYEIQSWKYSIPLFFA
THGFDTFRNREISSGAGAIREQLVDLDLRIVIDSSLVEWKELGEERSTHNENEWEDRKVG
RRKNFLVRRMELAKHFIRTNIEPEWMVLCLLPVLPPELRPIIQIDGGKLMSSDINELYRR
VIYRNNTLIDLLTTSRSTPGELVMCQEKLVQEAVDTLLDNGIRGQPMKDGHNKVYKSFSD

IIEGKEGRFRETLLGKRVDYSGRSVIVVGPSLSLHRCGLPREIAIELFQTFLIRSLIRKH
FASNIGVAKSKIREKEPIVWEILQEVMRGHPILLNRAPTLHRLGIQAFQPILVEGRAICL
HPLVCKGFNADFDGDQMAIHVPLSLEAQAEARLLMFSHTNLLSPAIGDPIAVPTQDMLIG
LYILTSGNRRGICANRYNRSNWKNSKNEKIRDKKYMKKKEPFFSNSYDAIGAFRQKKINF
DSPFWLRWRLDKCIMSSREAPIEVHYESLGTYHDIYEHYLVIRSIKKQICCIYIRTTIGH
ISFYREIEEAIQGFCRGYSYGI
>Q9BBS9 | RPOB_LOTJA
MLGVGNEGMSTLPGLNQIQFEGFCRFIDRGLTEELFKFPKIEDTDQEIEFQLFVETYQLV
EPSIKEKDAVYESLTYSSELYVSAGLIWKNSKNIQEQTIFIGNIPLMNSLGTSIVNGIYR
IVINQILQSPGIYYQSELDHKGISVYTGTIISDWGGRLELEIDRKARIWARVSRKQKISI
LVLSSAMGSNLNEILENVCYPEIFLSFLNDKEEKKIGSKESAILEFYRQFACVGGDPVFP
ESLCRELQKKFFQQRCELGEIGRRNMNRRLNLDIPQNNTFLLPRDILTAADHLIGMKFRM
GTLDDMNHLKNKRIRSVADLLQDQFGLALVRLENMVRGTICGAIRYKLIPTPQNLVTSTP
LTTTYESFFGLHPLSQVLDRTNPLTQIVHGRKLSYLGPGGLTGRTASFRIRDIHSSHYGR
ICPIDTSEGINVGLIGSLAIHARIGRWGSIESPFFEISERSKRIHMLYLSPSRDEYYMIA
TGNYLALNQGNQEEQIVPARYRQEFLTIAWEQVHLRSIFSFQYFSIGASLIPFIEHNDAN
RALMSSNMQRQAVPLSQSEKCIVGTGLERQVALDSGTLAIAEHEGKIIYKDTNKIVLFGS
GETLSVPLVIYRRSNKNTCMHQKSQVQRGKCIKRGQILADGAATVGGELSLGKNVLVAYM
PWEGYNSEDAVLISDRLVYEDIYTSFHIRKYEIQTHVTSNGPERITNKIPHLEVHLLRNL
DKNGLVILGSWVEAGDILVGKLTPQMAKESSYAPEDRLLRAILGIQVSTSKETCLKLPIG
GRGRVIDVRWLHKKGGSGYNPETIHIYILQKREIKVGDKVAGRHGNKGIVSKILARQDMP
YLQDGRPVDMVFNPLGVPSRMNVGQIFECSLGLAGDVLDRHYRIAPFDERYEQEASRKLV
FSELYQASKQTSNPWIFEPEYPGKSRIFDGRTGTPFEQPVIIGNPYILKLIHQVDDKIHG
RSSGHYALVTQQPLKGRAKQGGQRVGEMEVWALEGFGVAHILQEMLTYKSDHIKARQEVL
GTTIIGGTISKPVDAPESFRLLVRELRSLALELNHFLVSEKNFRIHRKEV
>Q9BBT3 | RR14_LOTJA
MARKSLIQREKKRQKLEQKYHLIRRSSKKEISKVPSLSEKWKIHGKLESLPRNSAPTRLH
RRCFSTGRPRANYRDFGLSGHTLREMVHECLLPGATRSSW
>Q9BBT5 | RR4_LOTJA
MSRYRGPRFKKIRRLGALPGLTSKRPTVGSELRNQSRSTKKSQYRIRLEEKQKLRFHYGL
TERQLLKYVRIAGKAKGSTGQVLLQLLEMRLDNILFRLGMASTIPQARQLVNHRHVFVNG
HIVDIPSYRCKPQDIITAKDNKKSKTLIQNSLESAPREELPTHLTLQPFQYKGLVNQIID
SKWVGLKINELLVVEYYSRQT
>Q9BCJ9 | MATK_ROSMU
MEEFQGYLELYRSQQHDFLYPLIFREYIYALAHDRGLNRSVLLDNVGYDKKSSLLIIKRL
ISRMYQQNHFIISLNDSNQNKFFGYNKNLYSQMISEGFAVIVEIPFSLRLVSSLEETETV
KSYNLRSIHSIFPFFEDKFPHLNYASDVLIPYPIHLEILVQTLRYCVKDPSSLHLLRLFL
HEYYNWNTLITPKKSIFAKSNQRLFLLLYNSYVCEYESILLFLRNQSNHLRLTSSGILFE
RIRFYEKIKYPVEEIFANDFPATLWFFKDPFIQYVRYQGKSILASKDTPLLMNKWKYYLV
HFWQCHFYVWSQPGRIHINQLSKHSFDFLGYLSSIRPNISVVRSQLLENSFLMDNAMKKL
DTLFPIIPMIGSLAKVKFCNTSGHPISKSSWADSSDSDIIDRFVRIGGNLSHYYSGSSKK
KSLYRIKYILRLSCVKTLARKHKSTVRTFLKRLGPKLLDEFFTEEEQIFSLLFPRTSSTL
KRFYRGRIWYLDILCINDLVNHE
>Q9C5U8 | HISX_ARATH

MSLNLSRLSLLSSPRISISTHAPRKGYVCCSMKSYRLSELSSSQVDSLKSRPRIDFSSIF
ATVNPIIDAVRSNGDNAVKEYTERFDKVQLNKVVEDMSELSVPELDSNVKEAFDVAYDNI
YAFHLAQKSTEKSVENMKGVRCKRVSRSIGSVGLYVPGGTAVLPSTALMLAIPAQIAGCK
TVVLATPPSKDGSICKEVLYCAKRAGVTHILKAGGAQAIAAMAWGTDSCPKVEKIFGPGN
QYVTAAKMILQNSEAMVSIDMPAGPSEVLVIADEHASPVYIAADLLSQAEHGPDSQVVLV
VVGDSVDLNAIEEEIAKQCKSLPRGEFASKALSHSFTVFARDMIEAISFSNLYAPEHLII
NVKDAEKWEGLIENAGSVFIGPWTPESVGDYASGTNHVLPTYGYARMYSGVSLDSFLKFM
TVQSLTEEGLRNLGPYVATMAEIEGLDAHKRAVTLRLKDIEAKQLA
>Q9FKG3 | K6PF4_ARATH
MEASISFLGSTKPNISLFNPSSNVLPRRDFPLPALKLKKVSVLPRILHQKRLIRAQCSDG
FKPEEDDGFVLEDVPHLTKFLPDLPSYPNPLKESQAYAIVKRTFVSSEDVVAQNIVVQKG
SKRGVHFRRAGPRERVYFRSDEVKACIVTCGGLCPGINTVIREIVCGLNNMYGVNNILGI
QGGYRGFYSKNTMNLTPKVVNDIHKRGGTFLQTSRGGHDTAKIVDNIQDRGINQVYIIGG
GGTQKGAEKIYEEVERRGLQVAVSGIPKTIDNDIAVIDKSFGFDTAVEEAQRAINAAHVE
VESVENGVGIVKLMGRYSGFIAMIATLANRDVDCCLIPESPFFLEGKGGLFEFIEERLKE
NRHMVIVIAEGAGQDYVAQSMRASETKDASGNRLLLDVGLWLTQQIKDHFTNVRKMMINM
KYIDPTYMIRAIPSNASDNVYCTLLAQSAVHGAMAGYSGFTVGPVNSRHAYIPISQVTEV
TNTVKLTDRMWARLLASTNQPSFLTGEGALQNVIDMETQEKIDNMKISSI
>Q9FS90 | RR4_SPLSP
MSRYRGPRVRIIRRLGALPGLTNKTPQLKSSPINQSTSNKKISQYRIRLEEKQKLRFHYG
ITERQLLNYVRIARHAKGSTGEILLQLLEMRLDNIIFRLGMAPTIPGARQLVNHRHILVN
EHIVDIPSYRCKPQDFITIKDRQKSQAIISKNIEFYQKYKIPNHLIYNSLKKQGLVNQIL
DRESIGLKINELLVVEYYSRQA
>Q9FSD9 | RR4_PELNE
MSRYRGPRTKIIRRLGALPGLTSKILELESGYIGQSTPNKKVSQYRIRLEEKQKLRFHYG
LTERQLLKYVRIARKAKGSTGQILSQTLEMRLDNIIFRLGMSPTIPGARQLVNHRHILIN
DNTVDIPSYNCEPKDVITVNNRKESVIIKNMDSSRKPKVPNHLTFDSIRFRGSVNQTIDR
ECIDLKINELLVVEYYSRQV
>Q9FV46 | ZDS_TARER
MATSSTSSTTSLCFPATSAAGARTSFRTTDTFLRYRRSRQLTRLKVRKAVVRSDLDRDVS
DMRTNAPKGLFPPEPEHYRGPKLKVAIIGAGLAGMSTAVELLDQGHEVDIYESRTFIGGK
VGSFVDKQGNHIEMGLHVFFGCYNNLFRLLKKVGAEKNLLVKDHTHTFVNRGGELGELDF
RFPVGAPLHGINAFLTTNQLKTYDKARNAVALALSPVVRALVDPDGAMTQIRNLDNISFS
EWFMSKGGTRTSIQRMWDPVAYALGFIDCDNISARCMLTIFSLFATKTEASLLRMLKGSP
DVYLSGPIRDYIIEKGGRFHLRWGCREILYEKSANGETYVTGLAMSKATQKQIVKADVYI
AACDVPGIKRLLPSNWREWEFFDNIYKLVGVPVVTVQLRYNGWVTELQDVERSRQLRQAA
GLDNLLYTPDADFSCFADLALASPEDYYIEGQGSLLQCVLTPGDPYMPLPNEEIISRVSK
QVLALFPSSQGLEVTWSSVVKIGQSLYREGPGKDPFRPDQKTPVKNFFLAGSYTKQDYID
SMEGATLSGRQASAFICDAGEELAALRKQLAAIQSIDSIGVDEMSLV
>Q9FVC8 | DAPA2_ARATH
MAALKGYGLCSMDSALQFPCPKLFNSYKRRSSKWVSPKAAVVPNFHLPMRSLEVKNRTNT
DDIKALRVITAIKTPYLPDGRFDLEAYDDLVNIQIQNGAEGVIVGGTTGEGQLMSWDEHI
MLIGHTVNCFGGSIKVIGNTGSNSTREAIHATEQGFAVGMHAALHINPYYGKTSIEGLIA
HFQSVLHMGPTIIYNVPGRTGQDIPPRAIFKLSQNPNLAGVKECVGNKRVEEYTENGVVV

WSGNDDECHDSRWDYGATGVISVTSNLVPGLMRKLMFEGRNSSLNSKLLPLMAWLFHEPN
PIGINTALAQLGVSRPVFRLPYVPLPLSKRLEFVKLVKEIGREHFVGEKDVQALDDDDFI
LIGRY
>Q9G1H4 | MATK_RORAM
MXXXXGYLEFDGARQQSFLYPLFFREYIYVLAYDHGLNRLNRNRSIFFENVDYEKKYSSL
IVKRLILRMYEQNRLIIPSKDLNQNHFFGHTSLFYYQMISVLFAVIVEIPFSLRLGSSFE
GKQFKKSYNLQSIHSIFPFLEDKLSHFNYVLDVVIPYPIHLEILVQTLRYRVKDASSLHF
FRFCLYEYCNWKDFSIKKKSILNPRFFLFLYNSHVCEYESIFFFLRKRSSHLRSTSYEVL
FERILFYGKIQHFLKVFINSFPAILGLLKDPFIHYVRYHGRCILATKDTPLLMNKWKYYF
VNLCQCYFSVWFQSQKVNINQLSKDNLEFLGYLSSLRLNPLVVRSQMLENSFLIDNVRIK
LDSKIPISSIIGSLAKDKFCNVLGHPISKAVWTDSSDSDILNRFVRISRNISHYYSGSSN
KKNLYRIKYILRLCCVKTLARKHKSTVRAFLKRLGSGLLEEFLTGEDQVLSLIFPRSYYA
SKRLYRVRIWYLDILYLNDLVNHE
>Q9GE26 | RR7_AMBTC
MSRRGTAEEKTAKSDPIYRNRLVNMLVNRILKHGKKSLAYQIIYRALKKIQQKTETNPLS
VLRQAIRGVTPDIAVKARRVGGSTHQVPIEIGSTQGKALAIRWLLGASRKRPGRNMAFKL
SSELVDAAKGNGDAIRKKEETHRMAEANRAFAHFR
>Q9GF29 | MATK_SISIR
MXXXXGYLEFDGARQQSFLYPLFFREYIYVLAYDQGLNRLNRNRSIFLENADYDKKYSSL
IVKRLILRMYEQNRLIIPTKGLNKNLGHTNLFYYQMISVLFAVIVEIPFSLRLGSSFEGK
KLKKSYNLQSIHSIFPFLEDKFSHFNYVLDVLIPYPIHLEILVQTLRYRVKDASSLHFFR
FCLYEYCNWKNFYSKKKSILNPRFLLFLYNSHVCEYESIFFFLRKRSSHLRSTSYEVFFE
RILFYGKIQHFLKVFINNFPAILGLLKDPFLHYVRYHGKCILATKDTPLLMNKWKYYFVN
LWQCYFSVWFQSQKVNINKLSKDNLEFLGYLSSLRLNPLVVRSQMLENSFLIDNVRIKLD
SKIPISSIIGSLAKDKFCNVLGHPISKATWTDSSDSDILNRFVRICRNISHYYSGSSKKK
NLYRIKYILRLCCVKTLARKHKSTVRAFLKRLGSGLLEEFLTGEDQVLSLIFQRSDYASK
RLYRVRVWYLDILYLNDLVNHE
>Q9GF34 | MATK_MATIN
MXXXXGYLEFDGAQQQSFLYPLFFREYIYVLAYDHGLNRLNRNRSIFLENADYDKRYSSL
IVKRLILRMSEQNRLITSTKDFSQNPFLGHTHLFYYQMISVLFAVIVEIPFSLRLGSSFE
GKKLKKSYNLQSIHSIFPFLEDKLSHFNYVLDVLIPYPIHLEILVQTLRYRVKDASSLHF
FRFCLYEYCSWKNFDIKKKSIFNPRFFLFLYNSHVCEYESIFFFLRKRSSHLRSTSYEVL
FERIFFYGKIQHFFKVFVNNFPAILGLLKDPFIHYVRYRGKCILATKDTPLLMNKWKYYF
VNLWQCYFSIWFQPQKANINQLYKANLEFLGYMSSLRLNPLVVRSQMLENSFLIDNVRIK
LDSKIPISSIIGSLAKDKFCNVLGHPISKANWTDSSDSDILNRFVRICRNISHYYSGSSK
KKILYRIKYILRLCCVKTLARKHKSTVRAFLKRLGSGLLEEFLTGEDQVLSLIFPRSFYA
SKRFFRVRIWYLDILYLNDLVNHE
>Q9GF37 | MATK_ARAKO
MEKFQGYLEFDGARQQSFLYPLFFREYIYVLAYDHGLNRLNKNRSIFLENTDYDKKYSSL
IVKRLILRMYEQNRLIIPTKDLNQNSFLGHTSLFYYQMISVLFAVIVEIPFSLRLGSPFQ
GKQVKKSYNLQSIHSIFPFLEDKLAHFNYVLDVLIPYPIHLEILVQTLRYRVKDASSLHF
FRFCLYEYCNWKNFYKKKKSILNPRFFLFLYNSHVCEYESIFFFLRKRSSHLRSTSYEVL
FERILFYGKIQYFLKVFVNNFPAILGLLKDPFIHYVRYHGRCVLATKDTPLLMNKWKYYF
VNLWQCYFSVWFQSQKVNINQLSKDNLEFLGYLSSLRLNPLVVRSQMLENSFLIDNVRIK

LDSKIPISSIIGSLAKDKFCNVLGHPISKATWTDSSDSDILNRFVRICRNISHYYSGSSK
KKNLYRIKYILRLCCVKTLARKHKSTVRAFLKRLGSGLLEEFLTGEDQVLSLIFPRSYYA
AKRLYRVRIWYLDILYLNDLVNHE
>Q9GF40 | MATK_ARABL
MEKFQGYLEFDGARQQSFLYPLFFREYIYVLAYDHGLNRLNKNRSIFLENADYDKKYSSL
IVKRIILRMYEQNRLIIPTTDLHKNLGHTNLFYYQMISVLFAVIVEIPFSLRLGSSFEGK
QLKKSYNLQSIHSIFPFLEDKLSHFNYVLDVLIPYPIHLEVLVQTLRYRVKDASSLHFFR
FCLYEYCNWKNFDIQKKCILNPRFLLFLYNSHICEYESIFFFLRKRSSHLRSTAYEVFFE
RILFYGKIQNFLKVFVNNFPAMLGLFKDPFLHYVRYHGKSILATKDTPLLMNKWKFYFVN
LWQCYFSVWFQSQKVNINQLSKDNLEFLGYLSSLRLNPLVVRSQMLENSFLIDNIRIKLD
SKIPISSIIGSLAKDKFCNVLGHPISKATWTDSSDSDILNRFVRICRNISHYYSGSSKKK
NLYRINYILRLCCVKTLARKHKSTVRAFLKRLGSGLLEEFLTGEDQVLSLIFPRSYYASK
RLYRVRIWYLDILYLNDLVNNE
>Q9GF47 | MATK_OLIPU
MEKFQGYLEFDGARQQSFLYPLFFREYIYVLAYDHGLNRLNKNRSIFLENTDYDKKYSSL
IVKRLILRMYEQNRLIIPTKDLNQNSFLGHTSLFYYQMISVLFAVIVEIPFSLRLGSPFQ
GKQVKKSYNLQSIHSIFPFLEDKLAHFNYVLDVLIPYPIHLEILVQTLRYRVKDASSLHF
FRFCLYEYCNWKNFYKKKKSILNPRFFLFLYNSHVCEYESIFFFLRKRSSHLRSTSYEVL
FERILFYGKIQYFLKVFVNNFPAILGLLKDPFIHYVRYHGRCVLATKDTPLLMNKWKYYF
VNLWQCYFSVWFQSQKVNINQLSKDNLEFLGYLSSLRLNPLVVRSQMLENSFLIDNVRIK
LDSKIPISSIIGSLAKDKFCNVLGHPISKATWTDSSDSDILNRFVRICRNISHYYSGSSK
KKNLYRIKYILRLCCVKTLARKHKSTVRAFLKRLGSGLLEEFLTGEDQVLSLIFPRSYYA
AKRLYRVRIWYLDILYLNDLVNHE
>Q9GF55 | MATK_CARAN
MEKFQGYLEFDGARQQSFLYPLFFREYIYVLAYDHGLNRLNRNRSIFLENADYDKKYSSL
IVKRLILRMYEQNRLIIPTKDLNQNNFLGHTSLFYYQMISVLFAVIVEIPFSLRLGSSFE
GNLFKKSYNLQSIHSIFPFLEDKLSHFNYVLDVVIPYPIHLEILVQTLRYRVKDASSLHF
LRFCVYEYFNCKNFYIKKKSILNPRFFLFLYNSHVCEYESIFFFLRKRSSHLRSTSYEVL
FERIFFYGKIQHFFKVFVNNFPAILGLLKDPFIHYVRYHGRSILATKDTPLLMNKWKYYF
VNLWQCYFSVWFQSQKVHIKQLSKDNLEFLGYLSSLRLNPLVVRSQMLENSFLIDNVRIK
LDSKIPISSIIGSLAKDKFCNVLGHPISKVIWTHSSDSDILNRFVRICRNISHYYSGSSK
KKFLYRIKYILRLCCVKTLARKHKSTVRAFLKRLGSGLLEEFLTGEDQVLSLIFPISYYA
SKRLYRVRIWYLDILYLNDLANHE
>Q9GF61 | MATK_ARALP
MEKFQGYLEFDGARQQSFLYPLFFREYIYVLAYDHGLNRLNRNRSIFLENTDYDKKYSSL
IVKRLILRMYEQNRLIIPTKDLNQNSFLGHTSLFYYQMISVLFAVIVEIPFSLRLGSSFQ
GKQLKKSYNLQSIHSILPFLEDKLAHFNYVLDVLIPYPIHLEILVQILRYWVKDASSLHF
FRFCLYEYCNCKNFYIKKKSILNPRFFLFLYNSHVCEYESIFFFLRKRSSHLRSPSYEVL
FERIFFYGKIQHFFKVFVNNFPAILGLLKDPFIHYVRYHGRCILATKDTPLLMNKWKYFF
VNLWQCYFSVWFQSQKVNINQLSKDNLEFLGYLSSLRLNPLVVRSQMLENSFLIDNVRIK
LDSKIPISSIIGSLAKDKFCNVLGHPISKATWTDSSDFDILNRFVRICKNISHYYSGSSK
KKNLYRIKYILRLCCVKTLARKXKSTVCAFLKRLGSGLLEEFLTGEDQVLSLIFPRSYYA
SKRLYRVRIWYLDILYLNDLVNHE
>Q9GFK0 | RR7_TROAR

MSRRGTAEEKTAKSDPIYRNRLVTMLVNRILKHGKKSLAYQIIYRAVKKIQQKTETNPLS
VLRQAIRGVTPDIAVKARRVGGSTHQVPIEIGSTQGKALAIRWLLGASRKRPGRNMAFKL
SSELVDAAKGSGDAIRKKEETHRMAEANRAFAHFR
>Q9GFK8 | RR7_LACFR
MSRRGTAEEKTAKSDPIYRNRLVNMLVNRILKHGKKSLAYQILYRAVKKIQQKTETNPLS
VLRQAIRGVTPDIVVKARRVGGSTHQVPIEIGSTQGKALAIRWLLVASRKRPGRNMAFKL
SSELVDAAKGGGDAIRKKEETHKMAEANRAFAHFR
>Q9GFL3 | RR7_GINBI
MSRRSTAEKETAKSDPIYRNRFINMLVNRILKHGKKSLAYRILYRAMKKIQQKTGKNPLS
VSRQAIRGVTPDVTVKARRVGGSTYQVPIEIRSTQGKALAIRWLLGASRKRPGRNMAFKL
SYELMDAARENGNAIRKKEETHRMAEAXRAFAHFR
>Q9GFL8 | RR7_DIOBU
MSRRGTAEEKTAKSDPIYRNRLVNMLVNRILKHGKKSLAYQIIYRTVKKIQQKTETNPLS
VLRQAIRGVTPDIAVKARRVGGSTHQVPIEIGSTQGKALAIRWLLGASRKRPGRNMAFKL
SSELVDAAKGSGDAIRKKEETHRMAEANRAFAHFR
>Q9GFM9 | RR7_CABCA
MSRRGTAEEKTAKSDPIYRNRLVNMLVNRILKHGKKSLAYQIIYRAVKKIQQKTETNPLS
VLRQAIRGVTPNIAVKARRVGGSTHQVPIEIGSTQGKALAIRWLLGASRKRPGRNMAFKL
SSELVDAAKGSGDAIRKKEETHRMAEANRAFAHFR
>Q9GFN8 | MATK_RHISY
MEEYPRYLELDRSRKNDFLFPLIYREYIYRLSHDHGLNRSILLENEGHNNKFSLVIIKRL
ITRIYQQNHLIISANDSNQNPFLRYNKNLYLQMISEGFAVIVEILFCLQLVSSLEESEIV
KSHNLRSIHSLFPFLEDKFPHLNYVSDVLVPYPIHLEKLIQTLRYWVKDPSSLHLFRLVV
HEEWNWNSLIISKKSISIFPKSNPRFFFFLYNIYVYEYESIFFFLRNQSFYLRSTFSWVL
LERIYFYGKLEQFTDVFANDFPSVLCLFKDPFMHYVRYQGKLILASKYTPLLMKKWKYYL
VNLGQCHFYVWFQPEKIYINLLSKHSLDFLGYLSRYRLNPSVVRSQMFENSFIIDNAMKK
LDTIVPIIPLIGSLAQTNFCNEIGYPVSNPTRAANSSDSDIIARFLRLCRNLFHYYSGSS
KKKSLYRLKYILRLSCVKTLACKHKSTVRFFLKRLGSEFLEEFLTEEARVLSLIFPRVSY
ISRTLYRGKVWYLDIICINDLSNHK
>Q9GGE2 | RR14_CHLRE
MAKKSMIQRELKRQKLVMKYATKRAALKEQIKQTTFLKEKLSLHRKLQQLPRNSSAVRLH
NRCMITGRPKGYFRDFGLSRHVLREMAHQGLLPGVCKSSW
>Q9GHB1 | MATK_EICCR
MEELQSFLEKDRSWXQHFLYPLLFQEYIYVFAHDHGLNGSIFYEPVNFLGYDKKSSAVLV
KRLIIRMYQQNHLICSFNESNRNRFVGHNYYSYFYSLMISEGVSLIVEIPFSLQLKSSIE
EIHNLRSIHSIFPFLEDKLSXLNYLXDILIPHPIHMXILVQILQSRSPDAPSLHFLRLFL
HQYHNWNSLITPKKSISVISKENKRLFXXLYNSYISECEFLLVFLRKQSSYLPLTSSGVF
LERTYYYGKIQRILVWQNFFQKTLWVFKDPXMHYVRYQGKVILGSKGTNFLMKKWKFYFV
NLWQYYFHFWSQPCRIHINQLSNYSFYFLGYFSNVLKNPLSVRNQMLENSFLMDTLTKKF
DTLVPVIPLISSLSKAKFCTVSGHPISKPIWTDLSDCDIINRFGRICRNLSHYHSGSSKK
QSLYRIKYILRLSCARTLARKHKSTVRIFMQRVSSGLLEEFFTEEERVLSLIFPQTTFFS
LRGSQRERIWYLDIIRINDLVTNF
>Q9GHE1 | MATK_DISSE
MEXLQGYLEXDRSRQQYFLYPLLFQEYIYTLAHGHGLNGSIFDEPVEIFGFDNKSSSVLV

KRLITRMYQQNYLVYLVNDSNQNRSIGRNHFFYSQFFFQMVSEGFAAIVEIPFSLQLVFS
SXEXERPKSHNLRSIHSIFPFLEDKFSHLNYVSEILIPHPIHMETLVQILQCWVRDVPSL
HFLRFFLHEYDNWNSFITPNKSSYAFSKENKRFFWFFYNSYVFEFEFLLVFLRKQSYYLQ
STSFGXFLDRXHFYGKKERLISACCNYSQKTLRFFKDPFMHYIRYQGKAILASRDTHILM
KKWKCYLVNFWQYYFHFLSYPYRIQINQLKNHSFFFLGYLSSVLINPLAVKNKMLDHSFL
IDTVTKKFDTTIPVIPLIGSLSKAKFCTVSGYPSSKTIWADLSDSDIVGRFGRICRNLSH
YYSGSSKKQSLYRIKYXXRLSCARTLARKHKRTIRTLLQRLGSGFLEEFFTEEEQVLSLI
FSKTTIPFPLYRLHRERIWYLDIIRINDLTNHLDWP
>Q9GHE5 | MATK_ZANAE
MEELKGYLEKSQSKQQHFLYPLLFLEYIYALAHDHGLNVNGSIFYEPAEMSGYDNKFSSL
LVKRLITRMYQQNFLINSVNDSNQNRFVGHNKNFYSQMISEGFAVIVEIPFSLRLVSSLE
EKKEIPKSQNLRSIHSIFPFFEDKLSHLNYVSDILIPYPVHLKILVQILQCWIQDVPSLH
LLRFFFHEYHNWNNIITPKKSSYGFSKENPRLFRFLYNSYVVECESILVFLRKQSSYLRS
TSSGTFLERTHFYEKIEQQHLVVLCYNDFQKILWLFKDPFMHYVRYQGKSILASKGTHFL
MKKWKSYFVNFWQCHFHFWSQPRRIHINQFSKFSFYFLGYLSNVPINPSAVKSQMLENSF
LIDTVTKKFETIVPIVPMIGSLSKAKFCNVLGNPISKPVWADLLDSDIIDRFGRICRNLS
HYYSGSSKKQSLYRIKYILRLSCARTLARKHKSTVRAFMQRLGSEFLEEFFTEEEKVISL
ALPRISYPLHKLYRERIWYXDIIRINDLVNHL
>Q9GI85 | MATK_ADELA
MEEYQIYLEIDGSCQENFLYPLSFQEYIYGLAYGHDLNRKVSILVENVDSDKKYSLLIVK
RLITRMYQQNHLLLFANDSKKNLFLGYNKNFYSQIISDAFAVIVEIPFSRQFISSLEDAE
TIKSFNNLRSIHSIFSFFEDKFTYLNFVSDVRIPYPIHLEILVQTXXXXXXXXPFFHLLR
LFLYEYSNWNTFITQKKRISTLSKSNPRFYIFLYNFYVCEHESIFLFLRKNSSHLRLNSF
SLLFERIHFYAKLEHLVEVFAKDFSCTLAFFKDPMIHYVRYQGKSILASKNAPLLLNKWK
YYLIYLWQCHFDVWSQEGTIRIKQLLSEHSFHFFWGGYISNVRLNFSVVRSQMLENSFPI
EIGMKRLDTIVPIIPLIRSLAKAKFCNILGHPISKPVWTDSSDFDIIDRFLRICRNISHY
YKGSSKKKGLYRIKYILRLSCIKTLARKHKSTVRAFLKRLDSEELLEEFLTEEEEILSLI
FPRASSTLQSLYRDQIWYLDILFSHDLFNYE
>Q9LFV6 | RPOT2_ARATH
MSSAQTPLFLANQTKVFDHLIPLHKPFISSPNPVSQSFPMWRNIAKQAISRSAARLNVSS
QTRGLLVSSPESIFSKNLSFRFPVLGSPCHGKGFRCLSGITRREEFSKSERCLSGTLARG
YTSVAEEEVLSTDVEEEPEVDELLKEMKKEKKRESHRSWRMKKQDQFGMGRTKFQNLWRR
QVKIETEEWERAAAEYMELLTDMCEQKLAPNLPYVKSLFLGWFEPLRDAIAKDQELYRLG
KSKATYAHYLDQLPADKISVITMHKLMGHLMTGGDNGCVKVVHAACTVGDAIEQEIRICT
FLDKKKKGDDNEESGGVENETSMKEQDKLRKKVNELIKKQKLSAVRKILQSHDYTKPWIA
DVRAKVGSRLIELLVRTAYIQSPADQQDNDLPDVRPAFVHTFKVAKGSMNSGRKYGVIEC
DPLVRKGLEKSGRYAVMPYMPMLVPPLKWSGYDKGAYLFLTSYIMKTHGAKQQREALKSA
PKGQLQPVFEALDTLGSTKWRVNKRVLTVVDRIWSSGGCVADMVDRSDVPLPEKPDTEDE
GILKKWKWEVKSAKKVNSERHSQRCDTELKLSVARKMKDEEAFYYPHNMDFRGRAYPMPP
HLNHLGSDLCRGVLEFAEGRPMGISGLRWLKIHLANLYAGGVDKLSLDGRLAFTENHLDD
IFDSADRPLEGSRWWLQAEDPFQCLAVCISLTEALRSPSPETVLSHIPIHQDGSCNGLQH
YAALGRDTLGAEAVNLVAGEKPADVYSGIATRVLDIMRRDADRDPEVFPEALRARKLLNQ
VDRKLVKQTVMTSVYGVTYIGARDQIKRRLKERSDFGDEKEVFGAACYAAKVTLAAIDEM
FQAARAIMRWFGECAKIIASENETVRWTTPLGLPVVQPYHQMGTKLVKTSLQTLSLQHET

DQVIVRRQRTAFPPNFIHSLDGSHMMMTAVACKRAGVCFAGVHDSFWTHACDVDKLNIIL
REKFVELYSQPILENLLESFEQSFPHLDFPPLPERGDLDLKVVLDSPYFFN
>Q9LLC1 | BCCP2_ARATH
MASLSVPCVKICALNRRVGSLPGISTQRWQPQPNGISFPSDVSQNHSAFWRLRATTNEVV
SNSTPMTNGGYMNGKAKTNVPEPAELSEFMAKVSGLLKLVDSKDIVELELKQLDCEIVIR
KKEALQQAVPPAPVYHSMPPVMADFSMPPAQPVALPPSPTPTSTPATAKPTSAPSSSHPP
LKSPMAGTFYRSPGPGEPPFVKVGDKVQKGQIVCIIEAMKLMNEIEAEKSGTIMELLAED
GKPVSVDTPLFVIAP
>Q9LS02 | AOC2_ARATH
MASSAVSLQSISMTTLNNLSCNQQFHRSSLLGSSKSFQNLGISSNGSDFSYPSSFTAKKN
LTASRALSQNGNIENPRPSKVQELSVYEINELDRHSPKILKNAFSLMFGLGDLVPFTNKL
YTGDLKKRVGITAGLCVVIEHVPEKKGERFEATYSFYFGDYGHLSVQGPYLTYEDSFLAI
TGGAGIFEGAYGQVKLQQLVYPTKLFYTFYLKGLANDLPLELTGTPVPPSKDIEPAPEAK
ALEPSGVISNYTN
>Q9M3I5 | NDHH_SPIOL
MAVPTTRKDLMIVNMGPHHPSMHGVLRLIVTLDGEDVIDCEPIVGYLHRGMEKIAENRTI
IQYLPYVTRWDYLATMFTEAITVNGPEQLGNIQVPKRASYIRVIMLELSRIASHLLWLGP
FMADIGAQTPFFYILRERELIYDLFEAATGMRMMHNYFRIGGVAADLPYGWIDKCLDFCD
YFLIGLTEYQKLITRNPIFLERVENVGIIGGEEAINWGLSGPMLRASGIQWDLRKVDHYE
CYDEFDWEVQWQKEGDSLARYLIRIGEMAESVKIIQQALEGIPGGPYENLEIRRFNRIKY
PEWNDFEYRFISKKPSPAFELSKQELYVRVEAPKGELGIFLIGDQSVFPWRWKIRPPGFI
NLQILPQLVKKMKLADIMTILGSIDIIMGEVDR
>Q9M3K7 | RR18_SPIOL
MDKSKRPFIKSKRSFRRRLPPIQSGDRIDYRNMSLISRFISEQGKILSRRVNRLTLKQQR
LITSAIKQARILSLLPFLNNEKQFERTESTTRTANFRTKNK
>Q9M3M1 | NDHJ_SPIOL
MQGRLSAWLVKHGLVHRSLGFDYQGIETLQIKPEDWHSIAVILYVYGYNYLRSQCAYDVA
PGGLLASVYHLTRIEYGVDQPEEVCIKVFAPRRNPRIPSVFWVWKSADFQERESYDMFGI
SYDNHPRLKRILMPESWIGWPLRKDYIVPNFYEIQDAY
>Q9M3N0 | MATK_SPIOL
MEEFQRHIELDRSWQHNFLYPLIFQEYIYAFAYDHGLNKSILLENSGNSKYSLLLVKRLI
TRMYQQNHLILSANDSNQNVILGHKHKKNLYSQMITEGFAVIVEVPFSLLLISSLGEKEI
VKSHNLRSIHSIFPFLEDKLLHLNYVLDILITYPAHLEILVQTLRYWVKDASSLHLLRFF
LYEYRNLNSLITPKELISFLKKRNQRLFLFLYNLHVCEYESLFVFLCNQSSYLRPTSFGA
LIERIYFCGKLKYLVKVFTKDFGVILWIFREPFPHYVRYQGKSILASKGTSLLMHKWKYY
LIYFGQCHFSVWSQPKRLYINRLSNHSLDFMGFLSRVRLNSSVIRSQMLKNSFLIENISK
KFDTVVPIIPLVGSLAKAKFCNVLGHPISKSVWTDLSDSDILDRFGRICRNISHYYSGSS
RKKSLYRIKYILRLSCARTLSRKHKSTVRAFLKRLGSEFLEEFFTEEEKALSLILPRDSS
ISRGLYRGRIWYLDIICIHNLANDE
>Q9M401 | BCAT3_ARATH
MERAAILPSVNQNYLLCPSRAFSTRLHSSTRNLSPPSFASIKLQHSSSSVSSNGGISLTR
CNAVSSNSSSTLVTELADIDWDTVGFGLKPADYMYVMKCNIDGEFSKGELQRFGNIEISP
SAGVLNYGQGLFEGLKAYRKKDGNNILLFRPEENAKRMRNGAERMCMPAPTVEQFVEAVT
ETVLANKRWVPPPGKGSLYVRPLLMGTGAVLGLAPAPEYTFIIYVSPVGNYFKEGVAPIN

LIVENEFHRATPGGTGGVKTIGNYAAVLKAQSIAKAKGYSDVLYLDCIYKRYLEEVSSCN
IFIVKDNVISTPEIKGTILPGITRKSMIDVARTQGFQVEERNVTVDELLEADEVFCTGTA
VVVSPVGSVTYKGKRVSYGEGTFGTVSKQLYTVLTSLQMGLIEDNMKWTVNLS
>Q9M4B8 | RR4_JAMAU
MSRYRGPRVKIIRRLGALPGLTNKTFKSKSSFIDRSTSNKKVSQYRIRLEEKQKLRFHYG
LTERQLLKYVRIARKAKGSTGQVLLQLLEMRLDNTIFRFDMAPTIPGARQLVNHRHISVN
NGIVDIPSYNCEPGDTITIRNKQKSQSIITKNTNLFQKIKIPSHLTFDSTQLRGFVNQTV
NREWVNLKINELLVVEYYSRQV
>Q9MDA1 | RK23_OENHO
MDGIKYAVFTDKGVRLLGKNQYTSNVESGSTRTEIKHWVELFFGVKVQAMNSHRLPGKGR
RMGPIMGHTMHYRRMIITLQPGYSIPPLRKKRT
>Q9MDK3 | RR12_OENHO
MPTIKQLIRNTRQPIRNVTKSPALRGCPQRRGTCTRVYTINPKKPNSALRKVARVRLTSG
FEITAYIPGIGHNSQEHSVVLVRGGRVKDLPGVRYHIVRGTLDAVGVKDRQQGRSKYGVK
RPK
>Q9MSA7 | MATK_CEPFR
MDEFQRDGKKNRSWQQCFLYPLFFREDLYAIAHDHNLDRSSSSEPTEILNSNYFSFLTVK
RLIRRIRQQNDSIVLFGICDPNQFIDRNRNSYSESVLEGLTVVLEVSFAMRSKHFLEGMD
GWKSIRSIHSIFPLMEDKFPHSNYISDIRVPYSIHPEILVRTFRRWIRDAPFLDLLRSIL
HEWRNSFSAENLQKALVAPGENMRFPLFLWNSYIYECESFLVPLLKRFYTSRSLVYGSFP
DRTDFDRKIKHIFTFPVKISTKRIWWMKDSFIHYVRYGERSLIALKGTHLQVKKCRYHLF
HFWQCYFHLWSQSYRVSILELSRNYSYFLGFFIRFKMKSLVVRTKMIDSLLTTDLITNEL
NPIAPIRSILLFLAKERFCDISGRPISRLSWTSLSDDDILDRFDRIWINLFHYYSGSINK
DSLYHIKYILLLSCAKTLACKHKSTIRLVREEPGSELFTKSFSKEREFIYSSFSKTRSQR
ERIWNSDILQINPLANSYTINK
>Q9MSR8 | MATK_AMEAR
MDEFQREGNKHRFWQQCFLYQIFFGEDLNAMVHDHHLDRSSSFERTEILISNYFSFLTVK
RLIRRIRQQNDSTGLFGNCDPNQFIDRNRNSYSESVLEALTVILEVSFAMRSKHFLEGIN
GWKSIRSIHCIFPLMEDKFPYSNYISDIRVPYSIHPELLVRTFRRWIRDTPSLHLLRFIL
HSWKNSFSAENLQKAMVAPRENMRLSLFLWNSYVYECESFLVPLLKRFSHSQSLLYGSFP
NXNHFVRKIKHIVIFPXINISTXRIWLLKDPFIQYVRYGERSLIALKGTHLQVKKCRYHL
FHFWQYYFHLWSQPYRICILELSKNYSFFLGYFLSFKMKPLVVRTKMLNDLFITNLITNE
LNPIAPIRSILFFLAKERFCDISGQTISKLSWTSLSDDDILDRFDRICRNLFHYYSGSIN
PDGLYYIKYILLLPCAKTLACKHKSTIRVVREESGSELFTKSFSKEREFISSSFSKTRSQ
RERFWNSDIIQINPLSNSWQKIQNKQVEN
>Q9MST4 | MATK_METGY
MDEFQRNENKHRSWQQFFLYPLFFREDLYAIAHDHHLDRSGSSEPTEILVSHFFSFLTVK
RSIRRIRKQNNSISLLRNCDRNQFSECKKNXCSKSLLEGLTVVLEVSFAMRSKHFIEGMD
GWNSIRSIHCIFPLMEDKLTHSNYISDIRVPYSIHPEILVRIFRRWIRDTPSLHLLRSIL
HEWQNSFSRDNLQKAIITPRENTRFSLFLWNSYVHECESFLVPLVKRFFNSQSLLYGSFP
DRTHFDKKMKHIVILXXRQISTKKIWLLKDSFMHYVRYGERSLIALKGTHLEVKKWRYHL
FHFWQYYFHLWFQPYRIRSLELSKTYSSFLGYFLHVKMRPLVVRAKMLDNLFITDLITNE
LKLIAPIRSILFFLAKEKFCDISGWPISKLSWTSLSDDDILDRFDRIWINLFHYYSGSMN
QDGLYHIKYILLLSCAKTLACKHKTTIRVVREQLGSELFTKSFSKEREFISSSFSKNRLK

RERIWNSEISQINPLANFWQNMQNKQIEN
>Q9MSV4 | MATK_CHAOB
MDEFQRNSNKHRSWQQFFLYPLFFREDLYAIAHYHHLDRSGSSEPTEILVSNFLSFLTVK
RSIRRIRKQNNSISLLGNSDSNKLIEYNKNSSFQLILEGFTIXLEVXXXXRSKHFIKXMD
GWNSXRSIHCIXXFMEAKLPHSNYISDLRVPYSIHPEILVRIFRRWIRDVPSLHLLRSIL
HEWKNSFNRENLQKALITQRENTRFSLFLWNSYVYECESFLIPLIKRILNSQSLLYGSFP
DRTHFEKKIKDIVIFPPHKISTKKIWLLKDSFIHYVRYGERSLIALKGTHLQVKKCRYHL
FHFWQYYFHLWFQPYRICSLELSKTSFSFLGFFMHVKMRPLVVRAKMLDDLFITDLITNE
LNSTAPIRSILFSLAKEKFCDISGWPISKLSWTSLSDDDILDRFDRIWINLFHYYSGSIN
QDGLYHIKYILLLSCAKTLACKHKSTIRVIREQLGSELFTKSFSKEREFISSSSSKTRLQ
RERIWNLEIXQINPLANFWQKMQNKQIKN
>Q9MTH6 | NDHH_OENHO
MNVTTTRKDLMIVNMGPHHPSMHGVLRLILTLDGEDVIDCEPILGYLHRGMEKIAENRTV
IQYLPYVTRWDYLATMFTEAITINGPEQLGNIQVPKRASYIRIIMLELSRIASHLLWLGP
FMADIGAQTPFFYIFRERELVYDLFEAATGMRMMHNYFRIGGVAADLPYGWIDKCLDFCD
YFLTAVSEYQKLITRNPIFLERVEGVGIIGGEEAINWGLSGPMLRASGIEWDLRKVDRYE
CYGELDWEIRWQKEGDSLARYLVRMSEMTESIKIIQQALEGIPGGPYENLEIRCFDREKD
PEWDGFEYRFISKKPSPTFELPKQELYVRVEAPKGELGIFLIGDQSGFPWRWKIRPPGFI
NLQILPQLVKRMKLADIMTILGSIDIIMGEVDR
>Q9MTH9 | NU6C_OENHO
MDLPGPIHDFLLVFLGSGLIVGGLGVVLLTNPIFSAFSLGLVLVCISLFFSLSNSYFVAA
AQLLIYVGAINVLILFAVMFMNGSEYSKDLTLWTVGDGITSLVCTSIFISLITTILDTSW
YGIIWTTKSNQIIEQDLIGNSQQIGIHLSTDFFLPFELISIILLVSLIGAIAVARQ
>Q9MTI0 | NU4LC_OENHO
MILEHVLVLSAYLFSIGIYGLITSRNMVRALMCLELILNSVNLNFVTFSDFFDSRQLKGD
IFSIFIIAIAAAEAAIGLAIVSSIYRNRKSIRINQSNLLNK
>Q9MTI3 | RK32_OENHO
MAVPKKRTSISKKRIRKTIWKKKAYWASLKAFSLAKSLSTGNSKSFFYSKF
>Q9MTI6 | RK22_OENHO
MKKKKTYGEVYALGQYISMSAPKARRVIDQIRGRSYEETLMLLALMPYRACDPILKLVNS
AAANARHNMSFNEATLVISKAEVNEGTTVKKLKPRARGRSYPIRRPTCHIRIVLQDTSFD
EFEEDFFSLKKDAWEKK
>Q9MTJ2 | RR11_OENHO
MAKSIPSAGLRLRLRLRRNARRRSRKSTRKIPKGVIHVQASFHNTIVTVTDVRGRVISWS
SAGTCGFKSTRKGTPFAAQTAAGDAIRPVVDQGMQRAEVRIKGPGLGRDAALRAIRRSGI
RLSCIRDVTPLPHNGCRPPKKRRV
>Q9MTK1 | RR18_OENHO
MDKSKRLFLKSKRSFRRRLPPIQSGDRIDYRNISLISRFISQQGKILSRRVNRLTLKQQR
LITIAIKQARILSLLPFRPKAQRFKRSQSTARTVGLRTRNK
>Q9MTM2 | RR2_OENHO
MTRRYWNINLEEMMEAGVHFGHGIKKWNPRMAPYIYANRKGIHITNLTKTARFLAEACDL
VFDAASRGGQFLIVGTKKQAAALVARAAIKARCHYVNKKWLGGMLTNWSTTETRLHQFRD
LRTEQKTGRLNRLPKRDAAILKRQLSHLQTYLGGIKYMTGLPDILIIILDQQEEYTALREC
ITLGIPTICLIDTDCDPDLADLPIPANDDAMASIRLILNKLVFAICEGRSSSIRNP

>Q9MTM4 | RPOC1_OENHO
MIDRYKHQQLRIGSVSPQQISTWANKILPNGEIVGEVTKPYTFHYKTNKPERDGLFCERI
FGPIKSGICACGTYRVIGDKKEDPNFCEQCGVEFVDSRIRRYQMGYIKLACPATHVWYLK
RLPSYIANLLDKPLKELEGLVYCDFSFARPVAKKPTFLRLRGLFEYEIQSWKYSIPLFFT
TQGFDTFRNREISTGAGAIREQLAGLDLRVIIDYSLVEWKELGEERSTGNEWEDRKIGRR
KQFLVRRVELAKHFIRTNIEPEWMVLCLLPVLPPELRPIIQMDGGKLMSSDINELYRRVI
YRNNILADLLTTSRSTPGDLVMGQEKLVQEAVDTLLDNGIRSRPVRDGQNKVYKSFSDVI
EGKEGRFRETLLGKRVDYSGRSVIVVGPTLPLHRCGLPREIAIELFQTFLIRGLIRQHLA
SDIVGAKSQIREKEPIVWEILQQVMQGHPVLLNRAPTLHRLGIQAFQPILVEGRAICLHP
LVCKGFNADFDGDQMAVHVPLSLEAQTEARLLMFSHMNLLSPAMGDPISVPTQDMLIGLY
ILTSGNPRGICTNRYNPWNRSNYQNERISDNNWKKKEPFFCNSYDAIGAYRQKRIHLDSP
LWLRWRLDQRVIASREVPIEVQYESLGTYHEIYGHYIIVRSVKTEILWMYIRTTVGHISL
FREMEEAIQGFCRARWYLS
>Q9MTP4 | NDHK_OENHO
MNSIEFTLLARRTQNSVISTTSNDLSNWSRLSSLWPLLYGTSCCFIEFASLIGSRFDFDR
YGLVPRSSPRQADLILTAGTVTMKMAPSLVRLYEQMPEPKYVIAMGACTITGGMFSTDSY
STVRGVDKLIPVDVYLPGCPPKPEAIIDAITKLRKKISREIYEDRIRSQEENRCFTTNHK
FHVGPSMHTGNYDPGLLYQLPSTSEIASETFFKYKSSVSAHELVN
>Q9MTP5 | NU3C_OENHO
MFLLYEYDIFWAFLIISSVIPILAFRISGLLAPTSIGPEKLSSYESGIEPMGDAWLQFRI
RYYMFALVFVVFDVETIFLYPWALSFDILGVSVFIEALIFVLILVLGLVYAWRKGALEWS

>Q9MTQ1 | MATK_OENHO
MEEFPGYFELDRSRQHDFLYPLIFRESIYALAHDHGLNRNRSTLFENEVDYDKKYSLIIV
KRLITRMYQRNHLIISANGSVQNPFWGHNQNLYSKILSEGFAVIVEIPFSLRVLSSFERK
EKDIAKSPTLRSIHSIFPFLEDQFSHLDYLSHVLIPYPIHLEIAVQTLRYWVKDASSLHL
LRIFLHEYWNSFSTPKKHITLFLKGNSRFFLFLYNSYVCEYESIFLFIRNQSSHFQSTSS
GVFFERILFYVKIDHLVEVFVGTDFLDIRSFFKDPNMHYVRYQGKSILASKDTPLLMNKW
KYYLVNLWQYHFSVWSQPGRININQLGKYSLDFLGYFSNVQLKSSVVRNQTLENSFLINN
AMKKLETTVPILPLIGSLSRAKFCNALGHPISKPTRNDSSDSDIIDRFVRICRNLSHYHS
GSSKKKSLYRIKYILRLSCVKTLARKHKSSVRAFLKRLGSELGDEFLTEEGVVLAVIFPK
ASGRLYRGRIWYLDIPCINDRVGDAEGSIFTK
>Q9MUL3 | NU6C_MESVI
MSFSEQIQNLSLLLLEIGTIIGALGVVLLPNILYSGFLLGGVLICIAGIYLLLNAEFIAA
AQVLIYVGAINVIILFAIMLVNKIENLNPSNNQMMRNGLSSFICFSFFILLSNMIFDTQW
IDTVGVSTKYSISIIGNHIFSDFLLPFEIVSVLLLVTLVGAVFIARKEDASEIEISKISF
LNLPDPSKK
>Q9MUL4 | NU4LC_MESVI
MYIENFLLLASALFCIGIYGLLTSRNIVRVLMCLELCLNAININFIAFSNFIDYEKINGQ
VIAIFIMTIAAAEAAIGLALVLTIYRNRETVDIENFDLLKG
>Q9MUL8 | RK32_MESVI
MAAPKKRTSKSRKNMRKSTWKRQAATQAKKALSLAKSIATGKSTIRGVQSNLSDES
>Q9MUM3 | CCSA_MESVI
MNLIEIETYLANASFALLLITMLIYGMKAIFTKNNILQLFGTLGILFSNFLVALLLSIRW

FDSHHFPLSNMYESLMFLCWCFTFFHLLIEKYIQINFIGFITVPIAMLVNAFATFFLPLD
MQHSTPLVPALKSNWLIMHVTIMMASYAALILGSLLSIAFLFLTYNKQIELQGNSIGNIN
DEMNSYITIDIEFQKNESIELAKLIDNLSYRTIGIGFPLLTIGIISGAVWANDAWGSYWS
WDPKETWALITWIIFAIYLHTRITKGWQGRRPAIVAFIGFVIVWVCYLGVNLLGQGLHSY
GWFTK
>Q9MUM5 | MIND_MESVI
MIEQINKDGEKKNSTDTRTIVITSGKGGVGKTTTTANLGMSIARLGYKVALIDADVGLRN
LDLLLGLENRVIYTAMEVFEGECCLDQALIRDKRWSNLALLAISKTRQRYHLTRRNMEML
VDSIRLRNYNFILIDCPAGIDVGFVNAVAPAEEAVVVTTPEITSIRDADRVAGLLEASGI
YEVKLLVNRVRPDMIQKNDMLSVRDVQEMLGIPLLGAIPEDTNVIVSTNRGQPLVLNKKL
TLSGISFENAARRLVGRKEYLVNLETGNKGLLKRVQQFLTGSEENV
>Q9MUM8 | NU4C_MESVI
MNNFPWITSIVMLPILAGLLIPFIPDENGKNVRWYALGIGLLDFLLISYIFGYKYNIQDT
SLQLIDDYEWISSINFHWRLGIDGLSIPLILLTGFITTLAMLGAWPIQKNAKLFYFLMLA
MYSGQLGVFASQDLLLFFLMWELELIPIYILLIIWGGKKRLYAATKFILYTALGSIFILI
AAFGMAFYGENMSFDMQILGEKEYPINLEILFYICFLIAYAVKLPAFPVHTWLPDTHGEA
HYSTCMLLAGILLKMGGYALIRINMNMLPNAHIYFAPYLAIIGVINIIYAALTSFAQRNI
KRKIAYSSISHMGFVLIGISSFTDIGLSGAMLQMVSHGLIGASLFFLAGTTYDRTRTLIL
EDMGGIAKYMPKIFAMFTTCSLASLALPGMSGFVAELMVFLGFANSNAYSIEFRGIITFL
EAIGIIVTPIYLLSMLRQVFYGSENLKLLKVNNLIDASAREIFIISCLLVPVIGIGIYPR
ILTQIYDLKTNAIIEHLEIIRSNSQIM
>Q9MUN0 | RR4_MESVI
MSRYRGPRLKIVRKLGDLPGLTSKINKNLQLAEQNKGKKSTKTKLSQYGIRLQEKQKLKY
NYGVTEKQLLLYIRKARTIKGSTGQMLLQYLEMRLDNTVFRLGLAPTIAGARQLVNHGHI
MVNNRIVTIPSYKCKPKDILSIRNNNKSRNLVLNNLASPSVSKIPNHLLLKKDTLTATVN
GIVERKSIPLEINELLVVEYYSRQT
>Q9MUN2 | RRP3_MESVI
MNIFIKQNSSTVKRRYPLQKFVLKFLWLEKNLAVTVDQVTNRGNSPITEYFFWPRKDAWE
ELKDALANKPWISYDESIALLNQTTDVINYWQEDEKKPSLSEAQAKFPNCIFTDKFANCL
YEN
>Q9MUP2 | RR12_MESVI
MPTIQQLIRSERKRVYNKTKSPALKACPQRRGVCTRVYTTTPKKPNSALRKVARVRLTSG
FEVTAYIPGIGHNLQEHSVVLIRGGRVKDLPGVRYHIIRGILDTAGVKDRAQSRSKYGVK
RS
>Q9MUP5 | RK33_MESVI
MAKTKGIRISITLECTSCKNNNNKRSTGISRYMTQKNRRNTPNRLELKKFCSHCNQSTIH
KEIK
>Q9MUQ9 | NU3C_MESVI
MFILEGYDSFVVFFIVACLVPILALSGSKLIRPKLSGIEKKMTYESGIEPMGEAWVQFNI
RYYMFALIFVIFDVETLFLYPWAIVFKDLGITAFLETLIFLSILIIGLVYAWRKGALEWS

>Q9MUR6 | RR14_MESVI
MAKKSMIEREKKRQKLVNKYAVKRKELKEQIKTSVSFEERFKLQLELQKLPRNSSPTRLH
NRCSVTGRPKGYYRDFGLSRHVLREMAHECLLPGVTKSSW

>Q9MUR9 | CHLB_MESVI
MKLAYWMYAGPAHIGTLRVASSFKNVHAIMHAPLGDDYFNVMRSMLERERDFTPVTASIV
DRHVLARGSQNKVVDNITRKDKEERPDLIVLTPTCTSSILQEDLQNFVNRASMNSNSDVI
LADVNHYRVNELQAADRTLEQVVRFYIEKAKNNNDLDLIKTTKPSANIIGIFTLGFHNQH
DCRELKRLLQDLGIQINEVIPEGGSVSNLKKLPKAWFNLVPYREVGLMTAIYLEKEFKMP
YVSTTPMGVVDTGSCIKEIENIINSYTQDKISYDEYIDKQTRFVSQAAWFSRSIDCQNLT
GKKAVVFGDATHAASMTRILSKEMGIYVICAGTYCTHDADWFKEQVQGYCDEVLITDNHT
EVGDLIARVEPAAIFGTQMERHIGKRLNIPCGVISAPVHIQNFPLGYRPFLGYEGTNQIA
DLVYNSFTLGMEDHLLEIFGGHDTKEVITKGLSTDSELRWTSESENELRKIPGFVRGKIK
RNTEKFARQNNVTEITVEIMYAAKEAMNA
>Q9MUS7 | RPOC2_MESVI
MGRMKKIFFNQKSDLNSMKLFFCNRIMNKGEIRRIIAWFLFNHGTSRTAHMVDRLKILGF
YHATKAGISLSIDDLSIPPTKKWLLEDAEQEIAKTQKNYSIGKITAVERFQKVIDTWHST
SETLKNEVVQYFEQTNPLNPVYMMAFSGARGNLSQVSQLVGMRGLMSDPQGQIIDLPIRS
NFREGLTVTEYVISCYGARKGLVDTALRTADSGYLTRRLVDVAQDMIIRETDCGTKSGIL
LGPVKDEQKVVFSLQERLIGRVLAENIYSPKQKQYIAKKNQDISASLSDKICEIKRTNIL
VRSPLTCKSIRSVCQLCYGWSLAHGNLVDLGEAVGIIAAQSIGEPGTQLTMRTFHTGGVF
TGNIAKQIRSSIDGKIIYSLTNTIPMRTRHGEIALITQSNINICIQGKNKEFNIEVPINT
ILLVNNGSMVRAKQLIAEVSAPVNAQSVEIASKYVASDFSGEIHFENLLLQEKKSAYGHK
MNRAGQYGGLLWILSGQVYKFSLDVPLMFDTADYIHNGSCMAEHNIISQYGGILQTSENN
LTIDNSNSYLYVLNSSMTIINAYLSHIKNARSEAYILTDDLNQKFFMQVESNNVIKNNQI
IAKFVTKTYLTNTGGILKYSSEIEVDEYDIVTQSYPVRKGGIIMWIPEETHLINKDASLL
MVRDCEYVEAGTQIIKDLICHSSGLAQVSQTNDILREVVIKPGKIYRPIDFSQVLDKHQT
LIQAGEEICDGIIAEELVYLENVNLSSGSALLIRPVVQFLIPNSTELKKLEAQYQLKQNI
SIQPSIHICYKDGQRIHAWQSMEIAKIFLRLEIDSHLHKLNVTLNLIENKLLQIKMFEYL
MIDLNQIIDFQSYQNVTRLVRKFNNYIIPGTILAKTEIIAKDHGEVRQSNKNFEEQKSFL
IMNEHDQMTLFVKNAYEFFQLGDLVHKGKEIVPGILAPQSGQVIQLQANRVTLRLARPYL
VSSEAVLYVDDGDFVKSGDTLVMLIFEQSKTGDIVQGLPRIEELLEARRTKGLKPLPNNL
HDLLQSFFDEATEKYGIQKAAKKSFEKLQLFLVNEVQSVYKAQGVHISDKHIEVIVRQMT
SKVMIEEGGDTTLLPGELIELHRIENMNRNVSVHAKYKPIVLGITKASLNTESFISAASF
QETTRVLTKAAIEGKTDWLRGLKENVIIGRLIPAGTGFNVHDKMYSRDLSLSNSYNLAYS
SNSQHKRIEQKNAEYNFEDIIFD
>Q9MUT8 | RK23_MESVI
MIDIVKYPVLTEKATRLLENNQYTFDVDPKANKITIKALIEDFFNVKVLSVNTHRPPRKK
RRIGRSEGYRPNYKRVIVTLKTGDSIKLLPET
>Q9MUT9 | RK2_MESVI
MGIRLYKAYTPGTRNRSVLEFNDITKTNPEKSLTYHRHRSKGRNNRGIITIRHRGGGHKR
LCRLIDFTREKNIPATVASIEYDPNRNCRIALLYYKNGIKRYIIHPRGLSVGKEIVSSVE
APLSVGNSLPLNKIPLGTGIHNIELSPGQGGQLARAAGAVAQLIAKEGKFVTVRLPSGEV
RLILKECWATIGQVGNVDANNITIGKAGRTRWLGKRPVVRGVVMNPVDHPHGGGEGRSPI
GRPKPVSPWGKTALGAKTRKRKKYSDVLIIRRRKSA
>Q9MUU4 | RK14_MESVI
MIQAQSYLNVADNSGAKKIMCIRVLGGSQRKYAAIGDVIIGVVKDSVPNMSLKKSEVVRA
VVVRTCKGIRRENGMTIRFDDNAAVVINKDGNPKGTRVFGPVARELRDRNFTKIVSLAPE

VL
>Q9MUU7 | IF1C_MESVI
MERQNLIEMEGVITESLPNAMFRVHLDNGFNVLAHISGKIRRNYIRILPGDRVKIELTPY
DLTKGRITYRLRKR
>Q9MUV0 | RPOA_MESVI
MSGNDLFPSTIYCIESKIESPRNLYGRFLIEPLAIGQGITVGNTLRRILLGDIEGAAITS
VKIPGANNEFSILPGIRESVLEILLNLKEIVFRTKSLDVQKGYLSIQGPCVVKAANLQLP
TSIEVVDGGQYIATLSGNANLDMEFVINTGKGYQMADYAIKKNSYISSLPVDAIFMPIHK
VNYMVEQDHTSKFLTERVILEIWTNGSISPRDALDIGIKKVIDLFNPLHHCSSEYSTNKN
DFSTESKINDILVEELELSVRAYNCLKRAQIHTISDLLAYSQEDLLEIKNFGRRSAEEVI
EALEKKLNIYLPKEKY
>Q9MUV1 | RR9_MESVI
MSVQKMMPIINYLGTGRRKSAVARVRLVPGNGEVIINGLPGTNYLQFNGSYLSAVRSPLE
TLGLEDNYDIIVKAVGGGLTGQAEAIRLGVARALCTIDTSNRHPLKKEGFLTRDSRVKER
KKYGLKKARKAPQFSKR
>Q9MUZ3 | MATK_BROIN
MEKFEGYSEKHKSRQQYFVYPLLFQEYIYAFAHDYGLNGSEPVEIVSCNNKKFSSLLVKR
LIIRIYQQNFWDNSVNHPNQDRLLYYKNYFYSEFYSQILSEGFAILVEIPFSLRELSCPK
EKEIPKFQNLRSIHSIFPFLEDKFLHLDYLSHIEIPYPIHLEILVQLLQYRIQDVPSLHL
LRFFLNYYSNSNSFITSMKSIFIFKKENKRLFRFLYNSYVSEYEFFLLFLRKQSSCLPLA
SSGTFLERIHFSRKMEHFWIMYPGFSRKNLWFFMDPLIHYVRYQGKAILASKGTFFLKKK
WKCYHINLWQYYFRFWTQPRRIHINQLANSCFDFMGYLSSVPKSPLLVRNKMLENSFLID
TRMKKFDTIVPATLLIGYLSKAQFCTGSGHPISKPIWTDLSDWDILDRFGRICRNLFHYH
SGSSKKQTLYRLKYILRLSCARTLARKHKSTVRTFMQRLGSVFLEEFFTEEEQIFCLMFT
KTTLFSFSGSHTERIWYLDIIRINDLVNPLN
>Q9MUZ4 | MATK_PHLPR
MEKFEGYSEKHKSRQQYFVYPLLFQEYIYAFAHDYGLNDSEPVEIVSYNNKKFSSLLVKR
LIIRMYQQNFGINSVNHPNQDRLLDYKIGFYSEFYSQILSEGFAIVVEIPFSLRELPCPK
QKEIPKFQNLRSIHSIFPFLEDKFLHLDYLSHIEIPYPIHLEILVQLLQYRIQDVPSLHL
LRFFLNYYSNWNSFITSMKSFFLFKKENKRLFRFLYNSYVSEYEFFLLFLRKQSSCLPLA
SSGTFLERIHFSRKMEHFGIMYPGFFRKTLWFFMDPLMHYARYQGKAIFASKGTLFFNKK
WKWYLIHLWQYFFSFWTQPRRIHLNQLANSCFDFMGYLSSVPKSPLLVRNQMLENSFLID
TRMQKLDTIVPVTALIGYLSKAQFCTGSGHPISKPIWTDLSDWDILDRFGRICRNLFHYH
SGSSKKQTLYRLKYILRLSCARTLARKHKSTVRTFMQRLGSAFLEEFFTEEELVFSLMFT
KTTLFSFRGSHSERIWYFDIIRINDLVKPLN
>Q9MUZ5 | MATK_PHAAD
MEKFEGYSEKHKSRQQYFVYPLLFQEYIYAFAHDYGLNDSEPVEIVSSNNKKFSSLLVKR
LITRMYQQNFWINSVNHPNQDRLLDYKIGFYSEFYSQILPEGFAIVVEIPFSLREFSCPK
EKEIPKFQNLRSIHSIFPFLEDKFLHLDYLSHVEIPYPIHLEILVQLLQYRIQDVPSLHL
LRFFLNYYSNWNSFITSMKSIFLFKKENKRLFRFLYNSYLSEYEFFLLFLRKQSSCLPLA
SSGTFLERIIFSRKMEHLGIMYPGFFRKTIWFFMDPLMHYVRYQGKAILASKGTHFLNKK
WKWYLINLWQYFFSFWIQPRRIHLNQLANSCFDFLGYLSSVPKSTLLVRNQMLENLFLID
TRMKKLDTIVPATALIGYLSKAQFCTGSGHPISKPIWTDLSDWDILDRFGRICRNLFHYH
SGSSKKQTLYRLKYILRLSCARTLARKHKSTVRTFMQRLGSAFLEEFFTEEELVFSLMFT

KTTLFSFRGSHNERIWYFDIIRINDLVKPLN
>Q9MV46 | MATK_ABIHL
MDEFHRYGKEDSSWQQCFLYPLFFQEDLYAIYHDHYLDGSSSSESMEHLSSNDQFSFLTV
KRLIGQIRQQNNSIVFFLNCDPNPLVDRNKSFYYESVLEGLTLVLEVPFSIRSKYSVEGM
NEWKSFRSIHSIFPFLEDKFPHSNYILDTRIPYSIHPEILVRTFRRWIRDAPSLHPLRSV
LYKYRNSPDNFQKSIIIDPRVNTRFLLFLWNHYVYGCESILVPLLKRSFHPRSLSHGSFP
DQTHFDRKIKHIIRNYRRNSLKSIWSLKDPRIHYVRYAERSIIAIKGTHLLVKKCRYHLP
IFRQFYFHLWSEPYRVCSHQLSKNCSSSLGYFLRVRMKPLLVRTKMLDELFITDLITDEF
DPIVPIVPIIGLLAREKLCDISGRPISKLYWTSLTDDDILDRFDRIWKNIFHYYSGSLDR
DGLYRIKYILSLSCAKTLACKHKSTIRVVRKELGPELFKKDFSKEREFYFPAFSSKAAAR
SQRERIWHSDIPQINPLANSWQKIQDLKS
>Q9MV48 | MATK_PSEMZ
MDEFHRNGKEDSSWQQCFLYPLFFQEDLYAIYHDHYLDGSSSSEPMEHVSSNDQFSFLTV
KRLIGQIRQQNHSIFLFVNRDPNPLVDRNKSSYSESVLEGLTLVLEVPFSIRSKNSVEGI
NEWKSFRSIHSIFPFLEDKFPHSNYISDTRIPYSIHPEILVRTFRRWIRDAPSLHLLRSV
LYEYRNSSENLQRSLIVAPRVNTRFFLFLWNHYVYECESILVPLLKRSSHSRSLSHGSFP
ERTHFNRKIKHILIFSRRNSLKRIWSLKDPNIHYVRYGERSIIAIKGTHLLVKKCRYHLL
IFRQCYFHLWFEPYRVCSHQLSKNCSSSLGYFLRIRMKPLLVRTKMLDELFIADLITGEF
DPIVPIIPIIGLLAREKFCDISGRPISKLSWTSLTDDDILDRFDRIWRNIFHYYSGSFGR
DGLYRIKYILSLSCAKTLACKHKSTIRVVRKELGPELFKKSFSKEREFDSPPFSSKSGAR
SQRERIWHSDIPQINPLANSWQKIQDLKVENLFDQ
>Q9MV49 | MATK_TSUCA
MDEFHRYGKEDSSWQQCFLYPLFFQEDLYAISHDHYLDVSSSSEPMEHLSSNDKLSFLIV
KRLIGQIRKQNNSIFLFVNCDPNPLVNHNKSSYSESVLEGLTLVLEVPFSIRSKYSVEGI
NEWKSFRSIHSIFPFLEDKFPHSNYILDTRIPYSIHPEILVRTFRRWIRDAPSLHPLRSV
LYKYRNSPENLKRSIIVVPRVNTRFLLFLWNNYVYECESILVPLLKRSFHPRSSSYGSFP
ERTHFHRKVKHIIRNFRRNSLKSIWSLKDPKIHYVRYGERPIIAIRGTHLLVKKCRYHLP
IFRQCYFHLWSEPYRVCSHQLSKNCSSSLGYSLRVRMKPLLVRTKMLDKLFITDLITDEF
DPIVPIVPIIGLLAKEKFCDISGRPISKLSWTSLTDDDILDRFDRIWRNIFHYYSGSFGR
DGLYRIKYILSLSCAKTLACKHKSTIRVVRKELGPELFKKYFSKEREFDYPAFSSKAAAH
SQRERIWHSDIPQINPLANSWQKIQDLRIERKLI
>Q9MV51 | MATK_ABIFI
MDEFHRYGKEDSSWQQCFLYPLFFQEDLYAIYHDHYLDGSSSSESMEHLSSNDQFSFLTV
KRLIGQIRQQNNSIVFFLNCDPNPLVDRNKSFYYESVLEGLTLVLEVPFSIRSKYSVEGM
NEWKSFRSIHSIFPFLEDKFPHSNYILDTRIPYSIHPEILVRTFRRWIRDAPSLHPLRSV
LYKYRNSPDNLQKSIIIDPRVNTRFLLFLWNHYVYGCESILVPLLKRSFHPRSLSHGSFP
DQTHFDRKIKHIIRNYRRNSLKSIWSLKDPRIHYVRYAERSIIAIKGTHLLVKKCRYHLP
IFRQFYFHLWSEPYRVCSHQLSKNCSSSLGYFLRVRMKPLLVRTKMLDELFITDLITDEF
DPIVPIVPIIGLLAREKLCDISGRPISKLYWTSLTDDDILDRFDRIWKNIFHYYSGSLDR
DGLYRIKYILSLSCAKTLACKHKSTIRVVRKELGPELFKKYFSKEREFDFPAFSSKAAAR
SQRERIWHSDIPQINPLANSWQKIQDLKS
>Q9MVD8 | MATK_MICFI
MEELQGYLEIDRSRQQHFLYPLLFQEYIYALAHDHGLNGSIFYEPMENFGYDNKSSSLIV
KRLITRMHQQNHVILSVNDSNESIFVGHNKNFYFQMVSEGFAVIMEIPFSLRLVSSLEEK

EIAKSHNSRSIHSIFPFFEDKLSHLNHVSDILIPHPIHLEILVQTLHCWIQDAPSLHLLR
FFLHEYRNSNSLITPKKSISLFSKENQRFFLLLYNSHVYECESVLVFLRKQSSHLRSTSS
GTFLERTHFYGKIEHLVVVLRNDFQKTLWLFKDPFMHYVRYQGKSILASKGTHLLMKKWK
SHLVHFWQCHFYLWSLPDRIHINQLYNHFLYFLGYLSSVRLNTSVVGIQMLENSFLIDTS
INKFETLVPIIPLIGSVAKAKFCNVSGHPISKSVRADSSDSDIINRFGRIYRNLSHYHSG
SSKKQTLYRIKYILRLSCARTLARKHKSTVRAFLKRLGSEFLEEFLTEEEQVLSLIFQRT
SSPSYRSHRERIWYLDIIRINDLANHS
>Q9MVW4 | MATK_CRYJA
MGEFQRNENKHKSWQQFFLYPLFFREDLYAIAHDHHLDRSGSSEPTEILVSNFLSFLTVK
RSIRRMRKQTNSISLFGNSDPNKLIECNKNFYSKSILEGFTIVLEVSFAMRSKHFIEGMN
GWNSLRSIHCLFPLMEDKLPHSNYISDIRVPYSIHPEILVRIFRRWIRDAPSLHLLRSIL
HEWKNSFSRENLQKALITQIENTRFSLFLWNSYVYECESFLIPLIKRFFNSQSLLYGSFP
DRTHFDKKIKDIVLFPRKISTKKIWLLKDSFIHYVRYGERSLMALKGTHLQVKKCRYHLF
HFWQYYFHLWFQPYRICSLQLSKTSFSFLGYFLHVKMKPLVVRVKMLDDLFITDLITNEL
NPIAPIRAILFFLAKEKFCDISGWPISKLSWTSLSDDDILDRFDRIWINLFHYYSGSINQ
EGLYHIKYILLLSCAKTLACKHKSTIRVVREQLGSELFTKSFSKEKFISSSFSKTRLQRE
RIWNSEISQINPLANFWQKMQNKQIEN
>Q9S795 | BADH1_ARATH
MAIPMPTRQLFIDGEWREPILKKRIPIVNPATEEVIGDIPAATTEDVDVAVNAARRALSR
NKGKDWAKAPGAVRAKYLRAIAAKVNERKTDLAKLEALDCGKPLDEAVWDMDDVAGCFEF
YADLAEGLDAKQKAPVSLPMESFKSYVLKQPLGVVGLITPWNYPLLMAVWKVAPSLAAGC
TAILKPSELASVTCLELADICREVGLPPGVLNVLTGFGSEAGAPLASHPGVDKIAFTGSF
ATGSKVMTAAAQLVKPVSMELGGKSPLIVFDDVDLDKAAEWALFGCFWTNGQICSATSRL
LVHESIASEFIEKLVKWSKNIKISDPMEEGCRLGPVVSKGQYEKILKFISTAKSEGATIL
HGGSRPEHLEKGFFIEPTIITDVTTSMQIWREEVFGPVLCVKTFASEDEAIELANDSHYG
LGAAVISNDTERCDRISEAFEAGIVWINCSQPCFTQAPWGGVKRSGFGRELGEWGLDNYL
SVKQVTLYTSNDPWGWYKSPN
>Q9SA18 | AKH1_ARATH
MPVVSLAKVVTSPAVAGDLAVRVPFIYGKRLVSNRVSFGKLRRRSCIGQCVRSELQSPRV
LGSVTDLALDNSVENGHLPKGDSWAVHKFGGTCVGNSERIKDVAAVVVKDDSERKLVVVS
AMSKVTDMMYDLIHRAESRDDSYLSALSGVLEKHRATAVDLLDGDELSSFLARLNDDINN
LKAMLRAIYIAGHATESFSDFVVGHGELWSAQMLAAVVRKSGLDCTWMDARDVLVVIPTS
SNQVDPDFVESEKRLEKWFTQNSAKIIIATGFIASTPQNIPTTLKRDGSDFSAAIMSALF
RSHQLTIWTDVDGVYSADPRKVSEAVVLKTLSYQEAWEMSYFGANVLHPRTIIPVMKYDI
PIVIRNIFNLSAPGTMICRQIDDEDGFKLDAPVKGFATIDNLALVNVEGTGMAGVPGTAS
AIFSAVKEVGANVIMISQASSEHSVCFAVPEKEVKAVSEALNSRFRQALAGGRLSQIEII
PNCSILAAVGQKMASTPGVSATFFNALAKANINIRAIAQGCSEFNITVVVKREDCIRALR
AVHSRFYLSRTTLAVGIIGPGLIGGTLLDQIRDQAAVLKEEFKIDLRVIGITGSSKMLMS
ESGIDLSRWRELMKEEGEKADMEKFTQYVKGNHFIPNSVMVDCTADADIASCYYDWLLRG
IHVVTPNKKANSGPLDQYLKIRDLQRKSYTHYFYEATVGAGLPIISTLRGLLETGDKILR
IEGIFSGTLSYLFNNFVGTRSFSEVVAEAKQAGFTEPDPRDDLSGTDVARKVTILARESG
LKLDLEGLPVQNLVPKPLQACASAEEFMEKLPQFDEELSKQREEAEAAGEVLRYVGVVDA
VEKKGTVELKRYKKDHPFAQLSGADNIIAFTTKRYKEQPLIVRGPGAGAQVTAGGIFSDI
LRLAFYLGAPS

>Q9SAK2 | KSB_ARATH
MSINLRSSGCSSPISATLERRLDSEVQTRANNVSFEQTKEKIRKMLEKVELSVSAYDTSW
VAMVPSPSSQNAPLFPQCVKWLLDNQHEDGSWGLDNHDHQSLKKDVLSSTLASILALKKW
GIGERQINKGLQFIELNSALVTDETIQKPTGFDIIFPGMIKYARDLNLTIPLGSEVVDDM
IRKRDLDLKCDSEKFSKGREAYLAYVLEGTRNLKDWDLIVKYQRKNGSLFDSPATTAAF
TQFGNDGCLRYLCSLLQKFEAAVPSVYPFDQYARLSIIVTLESLGIDRDFKTEIKSILDE
TYRYWLRGDEEICLDLATCALAFRLLLAHGYDVSYDPLKPFAEESGFSDTLEGYVKNTFS
VLELFKAAQSYPHESALKKQCCWTKQYLEMELSSWVKTSVRDKYLKKEVEDALAFPSYAS
LERSDHRRKILNGSAVENTRVTKTSYRLHNICTSDILKLAVDDFNFCQSIHREEMERLDR
WIVENRLQELKFARQKLAYCYFSGAATLFSPELSDARISWAKGGVLTTVVDDFFDVGGSK
EELENLIHLVEKWDLNGVPEYSSEHVEIIFSVLRDTILETGDKAFTYQGRNVTHHIVKIW
LDLLKSMLREAEWSSDKSTPSLEDYMENAYISFALGPIVLPATYLIGPPLPEKTVDSHQY
NQLYKLVSTMGRLLNDIQGFKRESAEGKLNAVSLHMKHERDNRSKEVIIESMKGLAERKR
EELHKLVLEEKGSVVPRECKEAFLKMSKVLNLFYRKDDGFTSNDLMSLVKSVIYEPVSLQ
EESLT
>Q9SCY0 | PGMP_ARATH
MTSTYTRFDTVFLFSRFAGAKYSPLLPSPSFTLSTSGIHIRTKPNSRFHSIIASSSSSSV
VAGTDSIEIKSLPTKPIEGQKTGTSGLRKKVKVFMEDNYLANWIQALFNSLPLEDYKNAT
LVLGGDGRYFNKEASQIIIKIAAGNGVGQILVGKEGILSTPAVSAVIRKRKANGGFIMSA
SHNPGGPEYDWGIKFNYSSGQPAPETITDKIYGNTLSISEIKVAEIPDIDLSQVGVTKYG
NFSVEVIDPVSDYLELMEDVFDFDLIRGLLSRSDFGFMFDAMHAVTGAYAKPIFVDNLGA
KPDSISNGVPLEDFGHGHPDPNLTYAKDLVDVMYRDNGPDFGAASDGDGDRNMVLGNKFF
VTPSDSVAIIAANAQEAIPYFRAGPKGLARSMPTSGALDRVAEKLKLPFFEVPTGWKFFG
NLMDAGKLSICGEESFGTGSDHIREKDGIWAVLAWLSILAHRNKDTKPGDKLVSVADVVK
EYWATYGRNFFSRYDYEECESEGANKMIEYLREILSKSKAGDVYGNYVLQFADDFSYTDP
VDGSVASKQGVRFVFTDGSRIIFRLSGTGSAGATVRIYIEQFEPDVSKHDVDAQIALKPL
IDLALSVSKLKDFTGREKPTVIT
>Q9SIK1 | GLGL4_ARATH
MDSSYSFALGTSSSILPKLSFRNVENRFYGEKNNNNGLCKRFGSDLGSKKFRNQKFKHGV
VYAVATSDNPKKAMTVKTSMFERRKVDPQNVAAIILGGGNGAKLFPLTMRAATPAVPVGG
CYRLIDIPMSNCINSCINKIFVLTQFNSASLNRHLARTYFGNGINFGGGFVEVLAATQTP
GEAGKKWFQGTADAVRKFLWVFEDAKNRNIENILILSGDHLYRMNYMDFVQSHVDSNADI
TLSCAPVSESRASNFGLVKIDRGGRVIHFSEKPTGVDLKSMQTDTTMLGLSHQEATDSPY
IASMGVYCFKTEALLNLLTRQYPSSNDFGSEVIPAAIRDHDVQGYIFRDYWEDIGTIKTF
YEANLALVEERPKFEFYDPETPFYTSPRFLPPTKAEKCRMVDSIISHGCFLRECSVQRSI
IGERSRLDYGVELQDTLMLGADYYQTESEIASLLAEGKVPIGIGKDTKIRKCIIDKNAKI
GKNVIIMNKGDVQEADRPEEGFYIRSGITVIVEKATIQDGTVI
>Q9SM59 | PGMP_PEA
MAFCYRLDNFIISAFKPKHSNVPLSIHHSSSNFPSFKVQNFPFRVRYNSAIRATSSSSST
PTTIAEPNDIKINSIPTKPIEGQKTGTSGLRKKVKVFKQENYLANWIQALFNSLPPEDYK
NGLLVLGGDGRYFNKEAAQIIIKIAAGNGVGKILVGKEGILSTPAVSAVIRKREANGGFI
MSASHNPGGPEYDWGIKFNYSSGQPAPESITDKIYGNTLSISEIKIADIPDVDLSNVGVT
KFGSFSVEVIDPVSDYLELLETVFDFQLIKSLISRPDFRFTFDAMHAVAGAYATPIFVDK
LSASLDSISNGIPLEDFGHGHPDPNLTYAKDLVKIMYAENGPDFGAASDGDGDRNMILGT

SFFVTPSDSVAVIAANAKEAIPYFKDSIKGLARSMPTSGALDRVAEKLNLPFFEVPTGWK
FFGNLMDAGNLSICGEESFGTGSDHIREKDGIWAVLAWLSIIAHRNKDTKPGEKLVSVSD
VVKEHWATYGRNFFSRYDYEECESEGANKMIEYLRELLSKSKPGDKYGSYVLQFADDFTY
TDPVDGSVVSKQGVRFVFTDGSRIIYRLSGTGSAGATVRVYIEQFEPDVSKHDVDAQIAL
KPLIDLALSVSKLKDFTGREKPTVIT
>Q9SMG1 | RR4_FUNHY
MSRYRGPRVRIIRRLGVLPGLTNKTPQLKSSSPNQSAAKKISQYRIRLEEKQKLRFHYGI
TERQLLNYVRIARKAKGSTGQVLLQLLEMRLDNIVFRLGMAPTIPGARQLVNHRHVLVND
CIVDIPSYRCKPEDSITVKNRQKSQAIITKNIDFSQKAKVPNHLTFDSTQKKGLVNQILD
RESIGLKINELLVVEYYSRQA
>Q9SS38 | GYRBP_ARATH
MESLQESSTSKDYSSEHIQVLEGLDPVRKRPGMYIGSTGSRGLHHLVYEILDNAIDEAQA
GFASKIDVVLHSDDSVSISDNGRGIPTDLHPATGKSSLETVLTVLHAGGKFGGKSSGYSV
SGGLHGVGLSVVNALSEALEVIVRRDGMEFQQKYSRGKPVTTLTCHVLPPESRGTQGTCI
RFWPDKEGFALFTTAIQFDHNTIAGRIRELAFLNPKVTISLKKEDDDPERDVYSEYFYAG
GLTEYVSWLNTDKKPLHDVLGFRKEINGSTVDVSLQWCSDAYSDTMLGYANSIRTIDGGT
HIEGVKASLTRTLNSLAKKLKVIKEKDISLSGEHVREGLTCIVSVKVPNPEFEGQTKTRL
GNPEVRKIVDQSVQEYLTEYLELHPDVLESIISKSLNAYKAALAAKRARELVRSKSVLKS
SSLPGKLADCSSTDPAESEIFIVEGDSAGGSAKQGRDRRFQAILPLRGKILNIERKDEAA
MYKNEEIQNLILGLGLGVKGEDFNKENLRYHKIIILTDADVDGAHIRTLLLTFFFRYQRA
LFDAGCIYVGVPPLFKVERGKQAHYCYDDAALKKITASFPGNASYNIQRFKGLGEMMPAQ
LWETTMNPDTRILKQLVVDDAAETNMVFSSLMGARVDVRKELIKSAATRMNLENLDI
>Q9SYL9 | RK13_ARATH
MAVLCSSSTVILSSSSVKSSGSERKSPFLGFSLTAISKPSVRVGIYANSKRGLQVKCEAE
PTTTTSLVPANQRWMFDEEEANGPDIWNTTWYPKASDHVNTDKPWFVVDATDKILGRLAS
TIANHIRGKNLASYTPSVDMGAFVIVVNAEKVAVSGKKRNQKLYRRHSGRPGGMTVETFD
QLQQRIPERIVEHAVRGMLPKGRLGRALFNHLKVYKGPDHPHEAQKPLDLPIRDKRIQLQ
K
>Q9T390 | TILS_NEPOL
MKGGRVQVITKNNSIAKHQLGEYAVHIYVLLYYLSMREILREFEEACLLQGLVSPSTRLL
ISCSGGQDSVTLLFLLCQLQTNWTWRLGVVYCNHMWRYGSIETPAKLARICLLFGVSCSF
SVSGSRLQKEEEGRSWRLRVLCRMSSIHSWFYISTGHTASDRIETLLSNVLRGSSSSGMR
SINWYTSLDTQVGHRRISIPIIRPLLGISRLELRNYANRWKLPLCYDPSNQDQRIRRNRI
RHELLPYLRHWWNPQIDRLLAQTAEVTSWESSYYDLICTQICQQYEWIGDGGVRFPWRIF
HSIPTSLHSRILWIFLNRALVFLNPAHGFQGNFDILQFLLETKTCHCRHGSIYISKDVDW
LRMTLVK
>Q9T399 | CHLL_NEPOL
MKLAVYGKGGIGKSTSSCNISIALATRGKKVLQIGADPKHDSTFALTGFLIPTIMDTLQS
KDYHYEEIWPEDIIYQGYGGVDCVEAGGPPAGAGCGGFVVGETTKLLKELNSFYEYDVIL
FDVLGDVVCGGFPAPLNYADYCLIVTDNGFDALFAANRIAASVREKARTHPLRLAGLVGN
RTSKRDLIDKYVESCPMPVLEVLPLIEEIRVSRVKGKTLFEMTETDSSLNYVCQYYLNIA
DQLLAEPEGVVPKEVADRELFSLLSDFYHREASFTPQEGVVDSFLLV
>Q9T3P6 | MIND_NEPOL
MTMQDKEPSAPACRVIVITSGKGGVGKTTATANLGMCIARLGYRVALIDADIGLRNLDLL

LGLENRVVYTAMEVIEGQCRLEQALIRDKRWKNLSMLAMSKNRQRYNMTRKNMMMIVDSI
KERGYQYILIDCPAGIDAGFVNAIAPADEAILVTTPEITAIRDADRVAGLLEANDFYNVR
LVANRVRPEMIQQNDMMSVDDVQGMIGVPLLGAIPEDKNVIISTNRGEPLVCQKTITLAG
VAFEEAARRLVGLPSPSDSAPSRGWFAAIRRLWS
>Q9T467 | CHLB_NEPOL
MKLAYWMYAGPAHLGVLRVASSFKNVHAIMHAPLGDDYFNVMSSMLERDRDFTPVTASIV
DRHVLATGSQRKVVATIHRKDQEDEPDLILVTPTCTSSILQEDLGNYVARAAPYTKSEVL
LADVQHYRIHELQAQDRILEQVVRHIMDPERQKGTLDTTPTPTPSANLIGFFDLGFHHRD
DSRELRRLLHGLGIEINSVLPKGGSIPDLHQLAKAWFNIIPYREAGLMTAKYLEESFHIP
YIDRTPIGLIETRKFIGEIEAILQTRGVDRHEYYEKFIIHHETIVSQAAWFARSIDCQNL
LGKRVIVFGDCTHAATITKLLVRELGIHVVCAGTYCKYDEAWFREQVNGYVDEILITEDH
TQVADTISRLEPAAIFGTQMERHVGKRLDIPCGVISAPAHIQNFPLSFRPFLGYEGTNVV
ADLIYNTFRLGMEDHLLELFGGHDTKQIGESQMAESISNSKGCQWTPEAEKELAHIPRFV
RSKVKRATERFAQEHGYPVINLECIYLAKQSTSVDIETIQHLLFNDETEVQ
>Q9T4F2 | RBL_NEPOL
MAPQTETQAKAGFKAGVKDYRLTYYTPDYQVKDTDILAAFRMTPQPGVPPEECGAAVAAE
SSTGTWTTVWTDGLTSLDRYKGRCYDLEPVAGEDNQYIAYVAYPLDLFEEGSVTNLFTSI
VGNVFGFKALRALRLEDLRISVAYCKTFQGAPHGIQVERDKLNKYGRGLLGCTIKPKLGL
SAKNYGRAVYECLRGGLDFTKDDENVNSQPFMRWRDRFLFCAEAIYKAQAETGEIKGHYL
NATAGTSEEMLKRAVFAKELGVPIIMHDYLTGGFTANTSLAYYCRDNGLLLHIHRAMHAV
IDRQRNHGIHFRVLAKALRLSGGDHLHSGTVVGKLEGEREVTLGFVDLMRDAYVEKDRSR
GIYFTQDWASLPGVMPVASGGIHVWHMPALVEIFGDDACLQFGGGTLGHPWGNAPGAAAN
RVALEACTQARNEGRDLAREGGDVIRAACKWSPELAAACEVWKEIKFEFDTVDTL
>Q9T4F6 | CHLN_NEPOL
MLSDQVTTAELHPSAPLTECETGIYHTFCPISCVAWLYQRVEDSFFLVIGTKTCGYFLQN
ALGVMIFAEPRYAMAELEEGDISAQFNDYHELRRLCVQIQQDRHPSVIVWIGTCTTEIIK
MDLEGMAPTLQAEIGIPIVVARANGLDYAFTQGEDTVLAAMAQRCPDWTPDRTATQSTSS
PPLVIFGSVPATVVSQLTFELQRQKVEVTGWLPSARYADLPAVGEDVYACGVNPFLSRTA
ITLMRRRRCKMIGSPFPIGPDGTRAWIEKICHVFGLETTGLAERERQVWSQLEDYLSLVR
GKSAFLMGDNLLEISLARFLIRCGMIVHEVGIPYMDARFQAAELHLLMRTCEAAHVPMPR
IVTKPDNFYQVQRMRDVQPDLAITGMAHANPLEARGTGTKWSVELTFWQLHGFASAKDLL
ELVTRPLRRTH
>Q9TIB6 | MATK_ELEIN
MAKFEGYLEKQKSRQQYFVYPLLFQEYIYAFAHDYVLNGSEPVEIIGCNNKKFSSLLVKR
LIIRMYQQNFWINSVNHSNQDRLLDHSNHFFSEFYSQILSEGFAIVVEIPFSLGQLSCPE
EKEIPKFQNLRSIHSIFPFLEDKFLHLHYLSHIEIPYPIHFEILVRLLEXRIQDVPSLHL
LRFFLNYYSIWNSLITSMKSVFLLKKENKRLFRFLYNSYVSEYEFFLLFLPKQSSCLRLT
SSGTFLQRIHFSVKMEHFGVMYPGFSRKTIWFFMDPLMHYVRYQGKAIFASKGTFFLKKK
WKSYLVNFPQYPSSSWTQPQRIRLNQLTRSCFDFLGYFSSVPINTFLVRNQMLENFFLID
TRMRKFDTTAPATPLIGSLSKAQFCTGLGHPISKPIWTDLSDWDILDRFGRICRNLFHYH
SGSSKKRTLYRLKYILRLSCARTLARKHKSTVRTFMQRLGSVFLEEFFTEEEQVFSLMFA
KTTHFSFHGSHSERIWYLDIIRIDDLVNPLTLN
>Q9TK08 | MATK_STYHA
MKKYQRYLELDRSQQQNFLYPLIFREYIYGLAPSHDLNRNRYILSENVDYDKNSSLLIVK

RLITRIYQQNHLILFDNDSSKNQFWGYNKNLYSQIISEGFAIVMEIPFSRQLSSSLEEAG
IVKSFNNLESIHSIFSFFEDKFTYLKFFSDVRIPYPIHLEILVQTXXXXXXXXPLLHLLR
LFLYEYCNWTSLITQKKIIFTFSKSNPRFFLFLYNFYVCEHESIFLFLRKKSSHLRLNSF
SVFFERIYFYTKLEHLVEVFSKNFSSTLSFFKDPLIHYVRYQGKSIFASKNAPFLMNKWK
YYFIYFWQCYFDIWSQPRMIQINELFENSFHFFWGGYLSNVRLNFSVVRSQMLEDSFLIE
IVMKKLDTIVPIIPLIRSLAKAKFCNRLGHPISKPVWTDSSDFDIIDRYLRICRNLSHYY
NGSSKKSLYRIKYIIRLSCIKTLARKHKRTLRAFLKRLDSEELLEEFFTEEEEILSLIFP
RSSATLRRLYRSRIWYLDILFSNDTINVN
>Q9TKB6 | MATK_KUNPU
MEEFQGYLELDRSRQHYLLYPLLFREYIYALAHDHGLNRSILFESAGYDNKSSSIIVKRL
ITRMYQQNPLIFSAKDSIQNQFFGHNKNLYSQILSEGFAVIVEIPFSLRFLFLLERKEIA
KSYHLRSIHSIFSFLEDKFTHLDYVSDVLIPYHIHLEILVQTLRYWVKDASSLHLLRLFL
HDYWNSFITPKKHITFFFKGNPRLFLFLYNSHICEYEYIFLFLRNQSSHLRSTSSGIFFE
RIHFYVKIEHFVKVFFDNNFQCILWFLKDPFMHYVRYQGKFFMASKDTPLLMNKWKCYLV
NLWQYHFSVWFQPGRIDINRLCKYSLDFLGYRSSVRLNSSVVRSQMLENLFLINNAMKKF
ETIVPIIPLIGSLYKSNFCNTFGHPISKPTRTHSSDSDIIDRFLRICRNLSHYHSGSSKK
KSLYRVKYILRLSCVKTLARKHKRTVRTFVKRLGSEFLEEFLTEEEVFLSLIFPRTYSTS
RRLYRGQIWYLDITSINDLVNYE
>Q9TKD1 | MATK_AGOFL
MEEFQGYLDLNRSRQHYLLYPLLFREYIYALAHDHGLNRXIGFENAGYDNKSSSIIVKRL
ITRMYQQNPLIFSAKDSIQNQFFGHNKNLYSQILSEGFAVIVEIPFSLRFLFSLERKXIP
KSHNLRSIHSIFPFLEDKFTHLXYVSDVLIPYHIHFEILVQTLRYWVKDASSLHLLRLFL
REYWNSLITPKKHITFFFKGNTRLFLFXYNSHICEYEYIFLFLRNQSSHLRSTSSGIFFE
RIYFYVKIXHFVKXFFDNNFQCILWFFKDPFMHYVXYQGKFFIASKDTPLLMNKWKCYLV
NLWQYHFSVWFQPGRIDINQLCKYSFDFLGYRSSVRLNSSVVRSQMLENLFLINNAMKKF
ETIVPIIPLIGSLYKSNFCNTFGHPISKPTRIDSSDSDIIDRFLRICRNLSHYHSGSSKK
KSLYRVKYILRLSCVKTLARKHKRTVRTFVKRLGSEFLEKFLTEEEVVLSLVXPRTYSTS
RRLYRGQIWYLDITSINDLXNYE
>Q9TKD3 | MATK_THRSA
MEEFQGYLEVDRSQQHDLLYPLLFREYIYALAHDHGLNRSILFENAGYDNKSSSIIVKRL
ITRMYQQNRLIFSSKDSIQNQFIGHNKNLYSQIISEGFAVIVEIPFSLRLVFSLERKEMA
KSHNLRSIHSIFPFLEDKFTHLDYVSDVLIPYYIHLEILVQTLRYWVKDSSSLHLLRFFL
HEYCNSLITPKKHITFFSKGNPRLFLFLYNSHICEYEYIFLFLRNQSSHLRSTSSGIFFE
RIYFYVKIEHFFKVFFDNNFQCTLWFFKDPFMHYVRYQGKFFLASKDTSLRMNKWKYYLV
NLWQYHFYAWFKPGRIDINQLFKYSLDFLGYRSNVRLNSSVVRSQMLZNLFLINNAMKKF
ETIVPIIPLIGSLYKANFCNTFGHPISKPTRTDSSDSDIIDRFLRICRNLSHYHSGSSKK
KSLYRVKYILRLSCVXXXXXXXXXTVRTFVKRLGSEFLEEFLTEEEVVLSLIFPRTYSTS
RRLYRGHIWYLDITSINDLVNYE
>Q9TKI5 | MATK_MEDSA
MKEYQVYLERARSRQQDFLYPLIFREYIYGLAYSHNFNRSIFVENVGSDSKYSLLIVKRL
ITRMYQQNHLIISANDSNKNPFWGYNKNFYSQIISEGFAIVVEIPFFLEKSSSLEEAEII
KSYKNLRSIHSIFPFLEDKFTYLNYVSDIRIPYPIHLEILVQILRYWVKDAPFFHLLRLF
LYNFSNWNSFITTKNSISTFSKSNPRLFLFLYNFYVCEYESIFLFLRNKSSHLRLKSFSV
FFERIFFYAKREHLVEVFAKDFSYTLTFFKDPLIHYVRYQGKCILASKNSPFLMNKWKHY

FIHLWQGFFYVWSQPRTIHINQLSEHSFQLLGYFLNVRVNRSVVRSQMLQNTFLIEIFSK
KLDIIVPIIPLIRSLAKAKFCNVLGPLISKPVWADSSDFDIIDRFLXICRNLSHYYNGSS
KKKSLYRIKYILRLSCIKTLACKHKSTVRAFLKRSGSEELLEEFFTEEEEILSLIFPRDS
STLHRLNRNRIWYLDILFSNDLVNDE
>Q9TKP0 | MATK_CARAB
MKEYQVYLERDRSRQQDFLYPLIFREYIYGLAYSHDFNRSSFVENVGYDNKFSLLIVKRL
ITRMYQQNHLIISANDSNKNPFLGYNXNFYSQIISEGFAIVVEIPFFLQLSSSLEEAEIV
KSYQNLRSIHSIFPFLEDKFTYLNYVSDIRIPYPIHLEILVQILRYWVKDAPFFHLLRLF
LYNFCNWNSFSTPKSTFSKSNPRLFLFLYNFYVCEYESIFLFLRKKSSHLRLKSFSVFFE
RIFFYAKREHLVEVFAKDFSSTLTFFNDPLIHYVRYQGKSILASKNGPLVMNKWKHYCIH
LWQCFFDIWSQPGTIHINQLSEHSFHLLGYFSNVRLNRSVVRSQMLQNTFLIEIVSKKLD
IIVPIIPLIRSLAKAKFCNVLRDPISKPVWGDSSDFDIIERFLRICRNLSHYYNGSSKKK
SLYRIKYILRLSCIKTLACKHKTTVRAFLKRSGSEELLEEFFTEEEGILSLIFPRASSTL
QRLHRNRIWYLDILFSNDLVNHE
>Q9TKP8 | MATK_LOTPU
MEEYQLYLELDRYRQQDFLYPLVFHEYIYGLAYSHDLNRSIFVENIGYDKKYSLLIVKRL
ITRMYRQNHLIISAKDSNKNRFLRYNKNFYSQIISEGFAIVVEILFSLQLSGSLEEAEII
KSYKNLRSIHSIFPFFEDKVTYLNYISDIRVPYPINLEILVQILRYWVKDAPLFHLLRLF
LYDYCNWNSIIIPKKSIFLFSKNNPRFFFFLYNFYLCEFESIFFFLRTKSSHLRLKSFRV
FFERIFFYAKKGHLVEVFLKDFFSTLTFFKDPFIHYVRYQGKSILASKNLPILMNKWKYF
FIHLWQCYFDVWSQPRTIHINQLSEYSFHFLGYFLKVGLKHSVVRGQMLQNGFLIKILIK
KLDIIVPIIPIIRLLAKSKFCNVLGNPLSKPSWADLSDFEIIARFLRICRNLSHYYNGSS
KKKSLYRIKYILRLSCIKTLACKHKSSVRAFLKRLGSEELLEEFFTEEEEILSLIFPRTS
STLRRLHRNRIWYLDILFSNDNDIINHD
>Q9TKS4 | MATK_PHACN
MEKYQAYLELLRRSRYQDILYPLFFRESIYGLAYGHESFFIENIDYNNKFSLLIVKRLST
RMYQQTNFILFANDSNKKTFGGYNYNFDSQIILEGFGVVVEILFSLQLFISSLRGLESVK
SYKNLQSIHSIFPFFEDKLIYLNHKSDIRIPYPIHLEILVQXXXXXXXXXSFFHLIRFFF
YYYSNGNSLFPPKKGISTFFSKRNLRIFLFLYNLYVWEYESIFLFLRNKSSQLQLKHFRV
FFERIFFYEKIKHLVEISTKNCSYTLFFFKDTFIHYVRYQGKSILVLKNTPLLINKWKYY
FIYLWQCYFDIWAGPETIYINQLSQYSFHFLGYFLSIPQNLSVVRSQMLKNSFLIKIVIX
XXXXXXXXXXXXXXXXXXXXXXXXXXXXXXXXXXXXXXXXLDRFLRICRNFSHYYNGSA
KKKSFYQIKYILRFSCIKTLARKHKSTVRTFLKKLSSEKLLEEFFTEEDLFSLIFPRTSL
TLRRFSRGRIWYLDILFRNDFVNHLEFKIGSDTF
>Q9TKV3 | NU6C_NEPOL
MEIVQNFSSAALTTGILLGCLGVIFLPSIVYAAFLLGAVFFCLAGIYVLLHADFVAAAQV
LVYVGAINVLILFAIMLVNPQDAPPRALDSPPLIPGIACIGLLGVLVQMISTTSWLTPPW
TPEPNSLPVLGGHLFSDCLLAFEVMSLVLLVALVGAIVLARREPVERSS
>Q9TKV8 | NU4C_NEPOL
MTHFPWLSTIVLFPLLASLAIPWLPDRKGTTVRWYALGVGLIDFSLIAYMFGRYYDFEQT
SLQFVEDITWIDRLHLHWSLGVDGLSMPLVLLTGFITTLATLAAWPVTKNPRLFYFLMLA
MYTGQLGVFVVQDLLLFFLMWELELIPVYLLVSCWGGKKRLYAATKFILYTALGSIFILL
GALTMPFMGIQGVTFDMSTLAYREYSLPVEILLYTGFLIAYGVKLPAIPVHTWLPDTHGE
AHYSTCMLLAGILLKMGGYALIRINMNMLPHAHALFSPWLIGVGVVNIIYAALTSFAQRN

LKRKIAYSSVSHMGFVLIGIGSLSEAGLNGAMLQMISHGLIGASLFFLAGTTYDRTRTLV
LEEMGGLATFMPKTFALFTACSLASLALPGMSGFFAELLVFLGLVTSEAYSPTFRAIMTV
FEAIGILLTPIYLLSMLRQLFYGRSIGRPKALIDAGPREVFVVSCLLVPILGIGIYPKLA
TAIYVNTTDHVVQHVLSALR
>Q9TKX3 | CYSA_NEPOL
MSILIENISKTFGTFRALDHVNLEVKAGSLVALVGPSGSGKSTLLRMIAGLERADEGKIW
LAGRDATYAPIQKRHIGFVFQNYALFKHLNVAKNISFGLEVRQANPNQIRSRVRDLLQLI
QLEHMADRYPAQLSGGQRQRVALARALAVEPKVLLLDEPFGALDARVRRELRSWLRDLHQ
EMPVTTVFVTHDQQEAMEVAHEIVVFNQGRLEQVGSPQEIYDHPATPFVMGFMGHINHGV
DDVQQSSYFVRPNDVIIQLVPSTNSLSGKVVGMTYGDTSMKLDVDIPQQVPSSDWAPRGS
LWRIHLSRRDFNQFNSMLVGGIQIGSVLYVQPRRTEMVRGYSI
>Q9TKX4 | RK20_NEPOL
MTRVKRGYVARKRRNKILRANRSFRGTHSKLFRIANQQHMKALRYSYRDRACKKRDFRHL
WITRINAVVRSYGLNYSRFMHQLRLGNMVLNRKVLSQLASLDPASFNRLIRAT
>Q9TKX9 | NU3C_NEPOL
MMILEGYGSVLAFFVIASLIPVIALSASKLLRPRGGGPERRTTYESGIEPMGEAWIQFNI
RYYMFALVFVVFDVETLFLYPWAVTFQRLGLSAFFEVLIFIIVLLIGLVYAWRKGALEWS

>Q9TKZ4 | RK19_NEPOL
MTKEDKEKKEKIEYRPLPAIAVGDLVKVGLLILEGDKERVQTYEGTVISKHRAGLNSTIT
VRKTFQGVGIERVFCLYSPRMVFVEFIRHSKARRAKLYYLRNRVGKQSRLEERFDLVTNT
ILLDLLASESDPSNSRT
>Q9TKZ6 | RR7_NEPOL
MSRRNTAVKRSISSDPVYNSQLIHMMISHILKEGKKALAYRLMYDAMKRIEKTTQQDPIL
VVERAVRNATPTIEVKARRMGGSIYQVPLEVKPERGTALALRWILLAARNRTGRDMVAKL
SNELMDASNRIGNAVRKRDEMHRMAEANKAFAHIRV
>Q9TL04 | RPOC2_NEPOL
MNTQEKHQFEPVLPQAHDDPTLSKPPIFFNRTADKGMIKRLIAWFLVHYGLADTVSMIED
LKQVGFQYATRAGISLGIEDLRIPPTKPRLLQEAHREINRMEFRYQQGYVSLVERFQKVI
DTWNGTSELLKDDVVENFLATDPLNPVYMMAFSGARGNLSQVRQLVGMRGLMSNPQGEII
DLPIRSNFREGLTVTEYIISCYGARKGLVDTALRTANSGYLTRRLVDVAQDIVIRMTSCS
PSAYPLISFTKSGKEVVYPLEERLLGRVLAIGAFNQEGELISGPDTAISQEIAVQLMDCD
PKEILVRSVLLCKSKRGLCRLCYGWNLGTGTIVSIGEAVGIIAAQSIGEPGTQLTMRTFH
TGGVFAGGVTNEIRAPHAGLVHYPRPIHPKWVRTRHGQFGILISEKIEIIFEHESKKTIQ
VFDAGTVLTIHEGEKVHTNQLIGEIPAHGTLVTGWRSLKSGVDGEVRFDELELLKQRPRS
DRTSPVRLDPRVCNKAHLWILQGHVNTFEMPIQLVANIGDKVEKDSIVSTTKQVVSEEGR
IIYRYDEKRGKEQTIITHAFGSVQFDGVDAWLPTKSQGIKQSIRTTGGKLSISSQGMTLS
ATKIKQEGIGSFDWPALAEPSIEEDEELEEAYKKTKDPIIQPTPYPLQYCVLPEQKYVIQ
AQEGWIISVLGGQSVGASQSLATYVDSQWEVEQGQLSIRSIEYRVHDQSWIQVITPSLVE
IESGRGSYHLMAKAGWIIPISTKIRCKDYRLISGSILLGKWSLPISRFAVEQAASYPAIL
IRPIHTYPVYPTTIWDETINWQRGIASIGSNDWHPRCIAPTYQAPSYVESFKGRQTFTGE
EKQEGNKPVEITSKNRRKSAANSSHETRMTQNRTWPIVLADTRIQKGASPIQIEWTWPHA
KNPWISPNHGAIAGHIRFVNMTILDSSSISTKQELDHREAQVIPTTQTVSGVHRLTPLLE
KTVFSQDDGEIQRMLPFSRALVKPVRTNRSKTRRNASGKTQVKAQARSQAKARSVRLKLK

ETVKTRSQEKFTNEMKKQLAKSSEGNKGFQIRQALFPKKLIRLMVVLRPVDIMTFATYGA
RVCIPLGAMIYQGEELFEGASTPISGQLIEIRRDRVTLRIGQPYRVGWQSRLMVTRDQIV
KAEKVLAHLLYFKTKTGDIVQGLPKIEEILEARRKKGNEIIRSNLQDFVQQFYQDNLDEG
FSRKYASTRTMRAMQVLILRRVQLVYRSQGVQISDKHLEIISLRMTSRVLVEKAYGTGIL
PGEIIEKRRADMLNQGRTRIRYCPIVLGITKASLTTKGFISSASFQETTRVLTQAVLQGK
SDWLLGLKENVILGRLIPAGVGIYGHWVGPHEFNIKQMLKLWVLPPIITGQSTMRAMFHS
TTRYWQDLEAVMESPNKLYPVTPYKCYPDTQHLAGLSIWMPEFEFRRFETFTEEEMVAAE
SLFTAHPNCHARRINNKQLNSLI
>Q9TL17 | RK23_NEPOL
MIIDLVKRPVITEKATRILEKNQYTFDVELSLTKPKIKALIEKAFKVEVVSVNTHRPPRR
KRRLGTTQGYLPRYKRAIITLKRGFMIPLTPF
>Q9TL22 | RK14_NEPOL
MIQPQTILQVADNSGARQLMCIRVLGGRKRYASIGDVIIAVVKDAIPNMPVKKSDVVRAV
VVRTSKPVRRDTGMLIRFDDNAAVIVNQEGNPRGTRVFGPVARELRDRQFMKIISLAPEV
L
>Q9TL23 | RK5_NEPOL
MAQRLKTFYLNSVVPKLRQQFHHRNIHEVPSIKKIVINRGLGDASQNAKLLDGSLQELTT
IAGQRGVITRSKQAIAAFKIRQDMPVGISVTLRGERMYAFLDRLINLALPRIRDFQGMST
KSFDGQGNYSLGLNEQLMFPEINYDAIDQMRGMDISIITSATEDREGFALLQALGMPFRT
ATID
>Q9TL25 | IF1C_NEPOL
MNKQGLFQMEGLVIESLPNAMFRVRLQNGFVILAHISGKIRRNYIRIIVGDRVLCELTPY
DLTKGRIVYRQRSTPRP
>Q9TL29 | RR9_NEPOL
MDHTSSVGLRKTGRRKCAVAQVRIIEGTGQLTINQRPGVPYLQYNPAYLLAAQGALDVTE
LTNRYDTIVKTKGGGLTGQAEAIKLGLARALCSLHIKHRKALKPQGYLTRNPLRKERKKY
GLKKARKAPQFSKR
>Q9TLD8 | MATK_ANDPO
MXECKGYLELXRSXXHDFIYPLIFQEYIYALAHDRGLNRSIFFENAXYDNKSSLLIVKRL
ITHLIXQMYXQNHFLFYTNNFXPDPFGGYXTGVXSXMIFEGFGVVVEIPFYLRLLSFLEG
KERVKSHNLRSLHSIFPFLEXXXXXXXXXXXXXXXXXXXXXXXXXXXXXXXXXXXXXXXI
RFFLHEYPNRNSLITPKKYSFSFSKRNKKFFLFLYNFHVYEYESIFVFLRNQSSHLCSIS
FETFLERILFYKKIELEVFAKHFKAILWVFKDPFLHYVRYRGKFILASKGSSLLMNKWEY
YLVNFWKCYFYIWAQPRRIHIKQLSKNSLDFLGYLSNVRLKPSMVRSQMIENSFLIENAG
KKLDTLVPITSMIGSLSKAKFCNVLGHPMNKPVWGGLSDSXIMERIRAXIXKSSHYYSXS
LKKISLYXIKYILRLPGAKTLARKHKITVRSFLKRLGVGLLEEFFTEEEQVFYLTFPKAS
STSRKLYQRRVWYLDIFYINDTPNHE
>Q9TM55 | RR7_BETVU
MSRRGTVEEKTAKSDPIYRNRLVNMLVNRILKHGKKSLAYQILYRAVKKIQQKTETNPLS
VLRQAIRGVTPDIAVKARRVGGSTHQVPIEIGSTQGKALAIRWLLGAARKRPGRNMAFKL
SSELVDAAKGSGDAIRKKEETHRMAEANRAFAHFR
>Q9TMB2 | MATK_MEDVI
MEELQGYLKKNGSPQQHFLYPLLLQEYIYTLAHDHGLNSSIFYESIEFIGYDNKSSLVLV
KRLITRMYQQNLFIYSVNDSNQNRFGGHTNFFYSHFFYSQMVSESFSVIVEIPFSLRLVS

SLEEEEKEIPKSQNLRSIHSIFPFLEDKLFHLNNVSDILIPHPIHLEILVQTLQYWIQDV
PSLHLLRFFLHKYHNLNSFIQNNKTIYVFSKENKRLFWFLYNSYVSECEFLFVFIRKKSC
YLRSTSSGAFLERSHFYGKKEHIIVVCCNNFQKTLCPVKDLFMHYVRYQGKAILASRGTH
LLMKKWRYYLVNFWQYYFHFWSQPYRMQINPLLNYSFYFMGYLSSVLINPYAVKNQMLEN
SFLIDTDINKFDTIIPGIPLIGSLSKVKFCTVSGHPISKPGWADLSDFDIIDRFGRICRN
LSHYHSGSSKKQTLYRIKYILRLSCARTLARKHKSTARALLQRLGSGLVEEFFTEEEQFI
SFIFPKTTPFPLHGSHRERIWSLDIIRVNDLVNQII
>Q9TMC8 | MATK_GAGLU
MEEFQEYFKKDKSPQQHFLYPLLLQEYIYTLAYDDGLNGSIFYEPIEFMGYDNKSSLVLV
KRLIIRMYQQNFFIYLVNDSNQNRFRGHTNYFYSHFFYSQMVSEGFSVIVEIPFSLRLVS
SPEEKEIPKSQNLRSIHSIFPFLEDKLSHLNNVSXILIPHPIHLEILVQILQCXIQDVPS
LHLLRFFLHKSHNMNSFLKNNKTIHVFSKETKRLFWFLYNSYVYECEFLLVFLRKQSCYL
RSTSSGAFLERSYFYGKMEHIHIIIVYCNNFHKTLWSIKDPFMQYVRYQGKAILASRGAY
LMMNKWRYYLLNFWQYYLNFWSQPYRMHINPLLNYSFYFMGYLSSVLINPSAVKNQMLEN
SFIIDTGIHKFDTIIPVIPLIGSLSKAKFCTVSGHPISKPVWADLSDFDIIDRFGKICRN
LSHYYSGSSKKQSLYRIKYILRLSCARTLARKHKSTARALLQRLGSGLLEEFFMEEEQVL
SFIFPKTTLFPLHGSHRERIWSLDIIRVNDLVNQII
>Q9TN87 | MATK_RUSAC
MEELQGYLEKDRSRQQHFLYPLLFQEYIYALAHNHGVNGSIFYEPVEVFGYDNKSSLVLV
KRLITRIYQQNYLISLVNDSNQNRFVEYNHNNFFXSHFHSQMISESFAIIVEIPFSLRLV
SYFEEKEIPKYHNLRSIHSIFPFLEDKLSHLNYVSDILIPHPIHMEILVQILQCWIQDVP
FLHLLRFFLHEYHNLNSLLITQKKSIYVFSKENKRVFRFLYNFYVFEWEFLFVFIRKQSS
YLRLTSSGTFLERTHFYEKIEHLKIEHFVVVCRNFFQRTLWFCKDPFMHYVRYQGKAILA
SKGTHLLMKKWKYLFFNFWQYYFHVWSQPYRIHINQLSNYSFYFLGYLSSLLLHFSAVRN
QMLKNSFLIDTITKKFDTIVPVIFLIGSLAKAKFCTVSGHPISKPIWTDLSDSDILDRFG
RICRNLSHYHSGSSKKQSLYRIKYILRLSCARTLARKHKSTVRTFLRRSGSGLLEEFFTE
EEQVLSLIFPKTTPFILHGSHRERIWYLDIIRINDLVNHS
>Q9TN89 | MATK_BEARE
MEELQGYLEKDRSRQQHFLYPLLFQEYIYALAHNHGLNGSIFYEPVEVFGYDNKSSLVLV
KRLIIRIYQQNFWISLVNDSNQNRFVGYNHNNFFXSHFYSQMISESFAIIVEIPFSLRLV
SYFEEKEIPKYHNLRSIHSIFPFLEDKLSHLNYVSDILIPHPIHMEILVQILQCWIQDVP
FLHLLRFFLHEYHNLNSLLITQKKSIYVFSKENKRLFRFLYNSYVFEWEFLLVFIRKQSS
YLRLTSSGTFLERTHFYEKIEHLQIEHFVVVCRNYFHRTLWFCKDPFMHYVRYQGKAILA
SKGTHLLMKKCKYHFFNFWQYYFHVWSQPYRIHLNQLSNYSFYFLGYLSSLLLNFSAVRN
QMLENSFLIDTITKKFDTIVPVIFLIGSLAKAKFCTVSGHPISKPIWTDLSDSDILDRFG
RICRNLSHYHSGSSKKQGLYRIKYILRLSCARTLARKHKSTVRTFLRRLGSGLLEEFFTE
EEQVLSLIFPKATPFILHGSHRERIWYLDIICINDLVNHS
>Q9TNA1 | MATK_LIRPL
MEELQGYLEKDGSRQQHFLYPLLFQEYIYALAHNHGLNGSIFYEPVEVFGYDNKSSLVLV
KRLITRIYQQNFLISLVNDSNQNRFVGYNHNNFFFSHFHSQMISESFAIIVEIPFSLRLV
SYFEEKEIPKYHNLRSIHSIFTFLEDKLSHLNYVSDILIPHPIHMEILVQILQCWIQDVP
FLHLLRFFLHEYHNLNSLLITQKKSIYVFSKENKRLFRFLYNSYVFEWEFLFVFIRKQSS
YLRLTSSGTFLGRTHFYEKIEHLQIKHFVVVCRNYFHRTLWFCKDPFMHYVRYQGKAILA
SKGTHLLMKKWKYHFFNFWQYYFHVWTQPYRIHINQLSNYSFYFLGYLSSLLLNFSAVRN

QMLENSFLIDTITKKFDTXVPVIFLIGSLAKAKFCTVSGHPISEPIWTDLSDSDILDRFG
RICRNLSHYHSGSSKKQGLYRIKYILRLSCARTLARKHKSTVRTFLRRLGSGLLEEFFTE
EEQVLSLIFPKTTPFILHGSHRERIWYLDIIRINDLVNHS
>Q9TNB2 | MATK_MAIDI
MEELQGYLEKDRSRQQHFLYPLLFQEYIYALAHNHGLNGSIFYEPVEVFGYDNKSSLVLV
KRLITRIYQQNFLISLVNDSNQNRFVGYNHNNFFFSHFHSQMISESFAIIVEIPFSLRLV
SYFEEKEIPKYHNLRSIHSIFPFLEDKLSHLNYVSDILIPHPIHMEILVQILQCWIQDVP
FLHLLRFFLHEYHNLNSLLITQKKSIYVFSKENKRLFRFLYNSYVFEWEFLLVFIRKQSS
YLRLISSGTFLERIHFYEKMEHLQMEHFVVVCRNYFHRTLWFCKDSFMHYVRYQGKAILA
SKGTHLLMKKWKYHFFNFWQYYFHVWSQPYRIHINQLSNYSFYFLGYLSSLLLNLSAVRN
QMLENSFLIDTITKKFDTIVPVIFLIGSLAKAQFCTVSGHPISKPIWADLSDSDILDRFG
RICRNISHYHSGSSKKQGLYRIKYILRLSCARTLARKHKSTVRTFLRRLGSGLLEEFFTE
EEQVLSLIFPKTTPFILHRSHRERIWYLDIIRINDLVNHS
>Q9XGS0 | TRXM_BRANA
MAAFTCTSSPPISLRSEMMIASSKTVSLSTRQMFSVGGLRTRVSLSSVSKNSRASRLRRG
GIICEAQDTATGIPMVNDSTWESLVLKADEPVVVDFWAPWCGPCKMIDPIVNELAQQYTG
KIKFFKLNTDDSPATPGKYGVRSIPTIMIFVKGEKKDTIIGAVPKTTLATSIDKFLQ
>Q9XGX5 | RBS2_AMAHP
MASSMMSNAATAVAVAATSGGAQANMVAPFNGLKSIASFPVTRKSNDITSIASNGGRVQC
MQVWPPVGKKKFETLSYLPPLSDAQLLAQVQYLLNKGWIPCIEFELEHPFVYRENHRSPG
YQDGRYWTMWKLPMYGCTDPAQVLNEVEEAKKAYPTAFIRIIGFDNKRQVQCVSFIAYKP
ADSY
>Q9XPP8 | MATK_TRIUN
MEELQGYLEKDGSRQQNFLYPLIFQEYIYTLAHDHGLNSSIFYEPMEIVGLGYDNKSSSV
LVKRLITRMYQQNSLIYSMNDFNQNLFVGHNNSFYSHFYSQMVSEGFAVIVEIPFSLRLV
PSSEEIPKSQNLRSIHSIFPFLEDKLSHLNYVLDILIPYPIHLEILVQILQCWIQDVPSL
HFLRFFLHEFHNWNNFLTPTKSISVFSKENKRLFRILYNSYVSEYEFVFFFLRKQSYYLR
STSSGAFLERTHFYVKIEHLIDVCHNHFQKILWFFKDSFMHYVRYKGKAILASRGTYLLI
KKWKCYLVNFWQYYFHFWSKPYRIHINPFSNYSFYFLGYISSVLINPSAVKNQMLENFYL
VDTLTQKFDTIVPVIPLIGSLSKAKFCTILGHPISKPIWAELSDSDIIDRFGRICRNLSH
YHSGSSKKQSLYRIKYILRLSCARTLARKHKSTVRNLLQRLGSGLLEEFFTEEEQVISPI
FPKTTLFPLHGSHRERIWYLDIIRINDLANYLDWS
>Q9XPS8 | RPOC1_WHEAT
MIDQYKHQQLQIGLVSPQQIKAWANKNLPNGEVVGEVTRPSTFHYKTDKPEKDGLFCERI
FGPIKSGICACGNSRASGAENEDERFCQKCGVEFVDSRIRRYQMGYIKLACPVTHVWYLK
GLPSYIANLLDKPLKKLEGLVYGDFSFARPSTKKPTFLRLRGLFEEEIASCNHSISPFFS
TPGFATFRNREIATGAGAIREQLADLDLRIIIENSLVEWKELEDEGYSGDEWEDRKRRIR
KVFLIRRMQLAKHFIQTNVEPEWMVLCLLPVLPPELRPIVYRSGDKVVTSDINELYKRVI
RRNNNLAYLLKRSELAPADLVMCQEKLVQEAVDTLLDSGSRGQPTRDGHNKVYKSLSDVI
EGKEGRFRETLLGKRVDYSGRSVIVVGPSLSLHQCGLPLEIAIKLFQLFVIRDLITKRAT
SNVRIAKRKIWEKEPIVWEILQEVMRGHPVLLNRAPTLHRLGIQAFQPTLVEGRTISLHP
LVCKGFNADFDGDQMAVHLPLSLEAQAEARLLMFSHMNLLSPAIGDPICVPTQDMLIGLY
VLTIGKRRGICANRYNSCRNYPNLKVNYNNNNNSKYRKDKEPHFSSSYDALGAYRQKLIS
LDSPLWLRWNLDQRVIGSREVPIEVQYESLGTYHEIYAHYLIMGNRKKEIRSIYIRTTLG

HISFYREIEEAIQGFSQAYSYTT
>Q9XPS9 | RPOC2_WHEAT
MAERANLVFHNKEIDGTGMKRLISRLIDHFGMGYTSHILDQLKTLGFHQATTTSISLGIE
DLLTIPSKGWLVQDAEQQSFLLEKHYYYGAVHAVEKLRQSVEIWYATSEYLKQEMNSNFR
ITDPSNPVYLMSFSGARGNASQVHQLVGMRGLMSDPQGQMIDLPIQSNLREGLSLTEYII
SCYGARKGVVDTAVRTADAGYLTRRLVEVVQHIIVRRRDCGTIRGISVSPQNGMTEKLFV
QTLIGRVLADDIYIGSRCIAARNQDIGIGLVNRFITAFRAQPFRAQPIYIRTPFTCRSTS
WICQLCYGRSPTHSDLVELGEAVGIIAGQSIGEPGTQLTLRTFHTGGVFTGGTADLVRSP
SNGKIKFNENLVHPTRTRHGQPAFLCYIDLHVTIQSQDILYSVNIPSKSLILVQNDQYVK
SEQVIAEIRAGTSTLHFKERVQKHIYSESDGEMHWSTDVYHAPEYQYGNLRRLPKTSHLW
ILSVSMCRSSIASFSLHKDQDQMNTYGKKDREILDYSTSDRIMSNGHWNFIYPSIFQDNS
DLLAKKRRNRFVIPLQYHQEQEKELISCFGISIEIPLMGVLRRNTIFAYFDDPRYRKDKK
GSGIVKFRYRTLEEEYRTRAEDSEEEYETLEDEYRTREDEYEYETLEESKYGILEDEYEY
ETLEDEYGSPENEYGNPENEYRTLEKDSEEEYGSPESKYRTQEDEYGTIEEDSEDEYGSP
GESAEEKYGTLEEDSEEDSEDEYESPEEDSILKKEGLIEHRGTKEFSLKYQKEVDRFFFI
LQELHILPRSSSLKILDNSIIGVDTQLTKNTRSRLGGLVRVKRKKSHTELKIFSGDIHFP
EEADKILGGCLIPPERQKKDSKESKKRKNWVYVQRKKILKSKEKYFVSVRPTVAYEMDEG
RNLATLFPQDLLQEENNLQIRLVNFISHENSKLTQRIYHTNSQFVRTCLVVNWEQEEKEK
AGASLVEVRANDLIRDFLRIELVKSTISYTRKRYDRTSAGPIPHNRLDRANINSFYSKAK
IESLSQHPEAIGTLLNRNKEYHSLMILSASNCSRIGLFKNSKHPNAIKEWNPRIPIREIF
GPLGAIVASISHFSSSYYLLTHNKILLKKYLFVDNLKQTFQVLQELKYSLIDENKRISNF
DSNIMLDPFLLNCHFVHHDSWEETLAIIHLGQFICENVCLFKSHIKKSGQIFIVNMNSFV
IRAAKPYLATTGATVNGHYGEILYKGDRLVTFIYEKSRSSDITQGLPKVEQIFEARSIDS
LSPNLERRIEDWNERIPRILGVPWGFLIGAELTIAQSRISLVNKIQKVYRSQGVQIHNRH
IEIIIRQVTSKVRVSEDGMSNVFSPGELIGLLRAERAGRALDESIYYRAILLGITRASLN
TQSFISEASFQETARVLAKAALRGRIDWLKGLKENVVLGGIIPVGTGFQKFVHRSPQDKN
LYFEIKKKNLFASEMRDFLFLHTELVSSDSDVTNNFYET
>Q9XR19 | MATK_PARTT
MEELQLQGYLEKDGFRQQNFLYPLIFQEYIYTLAHDHGLNSSIFYEPMEIVGLGYDNKSS
SVLVKRLITRMYQQNSLIYSMNDFNQNRFVGHNNSFYSNFYSKMVSEGFAVIVEIPFSLR
LVPSSEEIPKSQNLRSIHSIFPFLEDKLSHLNYVLDILIPYPIHLEILVKILQCWIQDVP
SLHFLRFFLHEFHNWNNLITPTKSISVFSKENKRLFRILYNSYVSEYEFVFVFLRKQSYY
LRSTSSGAFLERTHFYVKIEHLIDVCHNHFQKILWFFKDSFMHYVRYKGKAILASRGTYL
LIKKWKCYLVNFWQYNLNFWSKPYRIHINPFSNYSFYFLGYISSVLINPSAVKNQMLENF
YLVDTLTQKFDTIVPVIPLIGSLSKAKFCTILGHPISKPIWAELSDSDIIDRFGRICRNL
SHYHSGSSKKQSLYRIKYILRLSCARTLARKHKSTVRNLLQRLGSGLLEEFFTEEEQVIS
PIFQKTTLFPLHGSHRERIWYLDITRINDLANYLDWS
>Q9ZP40 | PG1_PEA
MALLSSTLRAPLVFSKNPKPVSLSSLHSRIYLSPRSPRFPSLRFISAAGDTGDAEKPSSN
ISDEWGEGSEPETKPFTYFKLPDSDPPKDEDEWGKGAAAGAGSYNDAGNGTPTFAAEASP
EAEAEDGVDENLEGLKRSLVDTVYGTELGFRARSEVRAEVSEFVAQLEAANPTPAPVEEP
DLLNGNWVLLYTASSELLPLLAAGSLPLLKLDKISQTIDTDSFTVVNSTTLSSPFASFSF
SVSASFEVRSPTRIQVTFKEGSLQPPEIKSKIDLPENINIFGQQLSLGPLLQSLGPLENV
VANISRVISGQSPLKIPIPGERTSSWLITTYLDKDLRISRGDGGLFVLAREGSSLLDQ

(2) 275 mitochondrion proteins

>A2Y4S9 | CYC_ORYSI
MASFSEAPPGNPKAGEKIFKTKCAQCHTVDKGAGHKQGPNLNGLFGRQSGTTPGYSYSTA
NKNMAVIWEENTLYDYLLNPKKYIPGTKMVFPGLKKPQERADLISYLKEATS
>O04866 | ARGD_ALNGL
MTSLQYFSLNRPVFPATHLHRPGIRHLQVSACANVEVQAPSSVKKQGVSKEVMEAAGRVL
VGTYARVPVVLSRGKGCKLYDPEGREYLDLSAGIAVNVLGHADSDWLRAVTEQAATLTHV
SNVFYSIPQVELAKRLVASSFADRVFFSNSGTEANEAAIKFARKFQRFTRPDEKQPATEF
VSFSNSFHGRTMGSLALTSKENYRSPFEPVMPGVTFLEYGNIEAATQLIQRRKIAAVFVE
PIQGEGGVYSATKEFLYALRKACDDSGTLLVFDEVQCGLGRTGYLWAHEIYDVFPDIMTL
AKPLAGGLPIGAVLVTERVASAITYGDHGTTFAGGPLVCKAALTVLDKILRPGFLASVSK
KGHYFKEMLINKLGGNSHVREVRGVGLIVGIELDVSASPLVNACLNSGLLVLTAGKGNVV
RIVPPLIITEQELEKAAEILLQCLPALDRHG
>O05000 | NU2M_ARATH
MKAEFVRILPHMFNLFLAVFPEIFIINATFILLIHGVVFSTSKKYDYPPLASNVGWLGLL
SVLITLLLLAAGAPLLTIAHLFWNNLFRRDNFTYFCQIFLLLSTAGTISMCFDFFDQERF
DAFEFIVLILLSTCGMLFMISAYDLIAMYLAIELQSLCFYVIAASKRKSEFSTEAGLKYL
ILGAFSSGILLFGCSMIYGSTGATHFDQLAKILTGYEITGARSSGIFMGILFIAVGFLFK
ITAVPFHMWAPDIYEGSPTPVTAFLSIAPKISIFANILRVFIYGSYGATLQQIFFFCSIA
SMILGALAAMAQTKVKRLLAYSSIGHVGYICIGFSCGTIEGIQSLLIGIFIYALMTMDAF
AIVLALRQTRVKYIADLGALAKTNPILAITFSITMFSYAGIPPLAGFCSKFYLFFAALGC
GAYFLALVGVVTSVIGCFYYIRLVKRMFFDTPRTWILYEPMDRNKSLLLAMTSFFITLFL
LYPSPLFSVTHQMALSLYL
>O22642 | CYC_FRIAG
MASFSEAPPGDFKSGEKIFKTKCAQCHTVDKGAGHKQGPNLNGLFGRQSGTTAGYSYSAA
NKNKAVNWDENTLYDYLLNPKKYIPGTKMVFPGLKKPQDRADLIAYLKEATSS
>O23936 | GCST_FLATR
MRGGLWQVGQSITRRLGQSDKKTIVRRWYASEADLKKTVLYDFHVANGGKMVPFAGWSMP
IQYKDSIMESTINCRENGSLFDVSHMCGLSLKGKDCVAFLEKLVVADVAGLAPGTGSLTV
FTNEKGGAIDDSVITKVTDDHIYLVVNAGCRDKDLAHIEQHMKAFKAKGGDVSWHIHDER
SLLALQGPLAGSTLQHLTKDDLSKMYFGDFRIIDISGSKCFLTRTGYTGEDGFEISVPSE
NAVDLAKAILEKSEGKVRLTGLGARDSLRLEAGLCLYGNDMEQHITPVEAGLTWAIGKRR
RAEGGFLGAEVILKQIADGPAIRRVGLFSTGPPARSHSEIQNEQGENIGEVTSGGFSPCL
KKNIGMGYVKSGLHKPGTKLKIVIRGKTYEGSVTKMPFVPTKYYKPA
>O49066 | SODM_CAPAN
MALRNLMTKKPFAGILTFRQQLRCVQTFSLPDLSYDYGALEPAISGEIMQLHHQKHHQTY
ITNYNNALQQLHDAINKGDSPTVAKLQGAIKFNGGGHINHSVFWKNLAPTREGGGEPPKG
SLGSAIDTNFGSLEAVIQKMNAEGAALQGSGWVWLGLDKELKRLVIETTANQDPLVIKGP
NLVPLLGIDVWEHAYYLQYKNVKPDYLKNIWKVINWKYAAEVYEKECP
>O49850 | GCSP_FLAAN
MERARRLAMLGRLVSQTKHNPSISSPALCSPSRYVSSLSPYVCGGTNVRSDRNLNGFGSQ
VRTISVEALKPSDTFPRRHNSATPEEQTKMAEFVGFSNLDSLIDATVPKSIRLDSMKYSK
FDEGLTESQMIAHMQDLASKNKIFKSFIGMGYYNTSVPTVILRNIMENPGWYTQYTPYQA
EIAQGRLESLLNFQTMITDLTGLPMSNASLLDEGTAAAEAMAMCNNIQKGKKKTFIIASN

CHPQTIDICKTRADGFDLKVVTSDLKDFDYSSGDVCGVLVQYPGTEGELLDYSEFIKNAH
ANGVKVVMASDLLALTILKPPGELGADIVVGSAQRFGVPMGYGGPHAAFLATSQEYKRMM
PGRIIGVSVDSSGKPALRMAMQTREQHIRRDKATSNICTAQALLANMAAMYGVYHGPEGL
KTIAKRVHGLAGTFAAGLKKLGTVQVQDLPFFDTVKVTCADSKAIAEEACKHKMNLRIVD
KNTITVAFDETTTIEDVDTLFKVFALGKPVPFTAASIAPEVQDAIPSGLVRETPYLTHPI
FNMYHTEHELLRYISKLQSKDLSLCHSMIPLGSCTMKLNATTEMMPVTWPAFADIHPFAP
TEQAQGYQEMFKNLGDLLCTITGFDSFSLQPNAGAAGEYAGLMVIRAYHMARGDHHRNVC
IIPVSAHGTNPASAAMCGMKIITVGTDSKGNINIEELRKAAEANKENLSALMVTYPSTHG
VYEEGIDEICKIIHDNGGQVYMDGANMNAQVGLTSPGWIGADVCHLNLHKTFCIPHGGGG
PGMGPIGVKKHLAPYLPSHPVVPTGGIPAPEESQPLGTIAAAPWGSALILPISYTYIAMM
GSQGITNASKIAILNANYMAKRLENHYPILFRGVNGTVAHEFIVDLRPLKTTAGIEPEDV
AKRLIDYGFHGPTMSWPVPGTLMIEPTESESKAELDRFCDALISIRQEIAEIEKGNVDFN
NNVIKGAPHPPQLLMADKWTKPYSREYAAYPAPWLRAAKFWPTTCRVDNVYGDRNLICTL
QPPQEYEEKAEATA
>O49954 | GCSP_SOLTU
MERARKLANRAILKRLVSQSKQSRSNEIPSSSLYRPSRYVSSLSPYTFQARNNAKSFNTQ
QARSISVEALKPSDTFPRRHNSATPEEQTKMAEFCGFQSLDALIDATVPQSIRSESMKLP
KFDSGLTESQMIEHMQNLASKNKVFKSYIGMGYYNTYVPPVILRNLLENPAWYTQYTPYQ
AEISQGRLESLLNYQTMITDLTGLPMSNASLLDEGTAAAEAMAMCNNILKGKKKTFLIAS
NCHPQTIDICKTRADGFDLKVVTVDLKDIDYKSGDVCGVLVQYPGTEGEILDYGEFIKNA
HAHGVKVVMASDLLALTMLKPPGELGADIVVGSAQRFGVPMGYGGPHAAFLATSQEYKRM
MPGRIIGLSVDSTGKPALRMAMQTREQHIRRDKATSNICTAQALLANMAAMYAVYHGPEG
LKTIGQRVHGLAGTFSAGLKKLGTVEVQDLPFFDTVKVKCSDAKAIADVANKNDINLRIV
DNNTITVSFDETTTLEDVDDLFKVFALGKPVPFTAQSIAQEVENLIPSGLTRETPFLTHQ
IFNSYHTEHELLRYLHKLQSKDLSLCHSMIPLGSCTMKLNATTEMMPVTWPSFANIHPFA
PTEQAAGYQEMFDDLGALLCTITGFDSFSLQPNAGAAGEYAGLMVIRAYHMSRGDHHRNV
CIIPVSAHGTNPASAAMCGMKIVAVGTDAKGNINIEELRKAAEANKDNLAALMVTYPSTH
GVYEEGIDEICKIIHDNGGQVYMDGANMNAQVGLTSPGFIGADVCHLNLHKTFCIPHGGG
GPGMGPIGVKKHLAPYLPSHPVVPTGGIPSPDKSEPLGAISAAPWGSALILPISYTYIAM
MGSKGLTDASKIAILSANYMAKRLEKHYPVLFRGVNGTCAHEFIIDLRGFKNTAGIEPED
VAKRLIDYGFHGPTMSWPVPGTLMIEPTESESKAELDRFCDALISIREEIAQIEKGNVDI
NNNVLKGAPHPPSMLMADAWTKPYSREYAAYPAPWLRSAKFWPTTGRVDNVYGDRNLICT
LLPVSEMAEEKAATA
>O64966 | HMDH1_GOSHI
METHRRSSTNSIRSHKPARPIALEDDSTKASDALPLPLYLTNAVFFTLFFSAVYFLLCRW
REKIRSSTPLHVVTFSEIVAILASVASFIYLLGFFGIDFVQSLVLRPSADVWATEDDEVE
SEVLLRNEDARHVPCGQALDRSIRSLQPPEPIVTAEKVFDEMPVTVMTEEDEEIIRSVVC
GMTPSYSLESKLDDCKRAAAIRREALQRITGKSLSGLPLDGFDYESILGQCCEMPVGYEQ
IPVGIAGPLLLNGREYSVPMATTEGCLVASTNRGCKAIHLSGGATSVLLRDGMTRAPVVR
FGTAKRAADLKLYLEDPENFETLACVFNRSSRFARLQSIKCAIAGKNLYLRFSCFTGDAM
GMNMVSKGVQNVLDFLQTDFPDMDVIGISGNFCSDKKPAAVNWIEGRGKSVVCEAIINGD
VVTKVLKTSVESLVELNMLKNLTGSAMAGALGGFNAHASNIVTAVYIATGQDPAQNVESS
HCITMMEAVNGGKDLHVSVTMPSIEVGTVGGGTQLASQSACLNLLGVKGASKESPGANSI
LLATIVAGAVLAGELSLMSALAAGQLVKSHMKYNRSSKDVSKVSS

>O80433 | CISY_DAUCA
MVFFRSVSLLNKLRSRAVQQSNLSNTVRWFQVQTSASDLDLRSQLKELIPEQQERIKKLK
AEHGKVQLGNITVDMVLGGMRGMTGLLWETSLLDPEEGIRFRGLSIPECQKLLPGAKPGG
EPLPEGLLWLLLTGKVPTKEQVDALSAELRSRAAVPEHVYKTIDALPVTAHPMTQFATGV
MALQVQSEFQKAYEKGIHKTKYWEPTYEDSITLIAQLPVVAAYIYRRMYKNGQSISTDDS
LDYGANFAHMLGYDSPSMQELMRLYVTIHTDHEGGNVSAHTGHLVASALSDPYLSFAAAL
NGLAGPLHGLANQEVLLWIKSVVSECGENVTKEQLKDYIWKTLNSGKVVPGYGHGVLRNT
DPRYICQREFALKHLPDDPLFQLVSNLFEVVPPILTELGKVKNPWPNVDAHSGVLLNHYG
LTEARYYTVLFGVSRAIGICSQLVWDRALGLPLERPKSVTMEWLENHCKKSS
>O80988 | GCSP_ARATH
MERARRLAYRGIVKRLVNETKRHRNGESSLLPTTTVTPSRYVSSVSSFLHRRRDVSGSAF
TTSGRNQHQTRSISVDALKPSDTFPRRHNSATPDEQAQMANYCGFDNLNTLIDSTVPKSI
RLDSMKFSGIFDEGLTESQMIEHMSDLASKNKVFKSFIGMGYYNTHVPPVILRNIMENPA
WYTQYTPYQAEISQGRLESLLNYQTVITDLTGLPMSNASLLDEGTAAAEAMAMCNNILKG
KKKTFVIASNCHPQTIDVCKTRADGFDLKVVTVDIKDVDYSSGDVCGVLVQYPGTEGEVL
DYGEFVKNAHANGVKVVMATDLLALTMLKPPGEFGADIVVGSGQRFGVPMGYGGPHAAFL
ATSQEYKRMMPGRIIGVSVDSSGKQALRMAMQTREQHIRRDKATSNICTAQALLANMTAM
YAVYHGPEGLKSIAQRVHGLAGVFALGLKKLGTAQVQDLPFFDTVKVTCSDATAIFDVAA
KKEINLRLVDSNTITVAFDETTTLDDVDKLFEVFASGKPVQFTAESLAPEFNNAIPSSLT
RESPYLTHPIFNMYHTEHELLRYIHKLQNKDLSLCHSMIPLGSCTMKLNATTEMMPVTWP
SFTNMHPFAPVEQAQGYQEMFTNLGELLCTITGFDSFSLQPNAGAAGEYAGLMVIRAYHM
SRGDHHRNVCIIPVSAHGTNPASAAMCGMKIVAVGTDAKGNINIEELRNAAEANKDNLAA
LMVTYPSTHGVYEEGIDEICNIIHENGGQVYMDGANMNAQVGLTSPGFIGADVCHLNLHK
TFCIPHGGGGPGMGPIGVKQHLAPFLPSHPVIPTGGIPEPEQTSPLGTISAAPWGSALIL
PISYTYIAMMGSGGLTDASKIAILNANYMAKRLESHYPVLFRGVNGTVAHEFIIDLRGFK
NTAGIEPEDVAKRLMDYGFHGPTMSWPVPGTLMIEPTESESKAELDRFCDALISIREEIS
QIEKGNADPNNNVLKGAPHPPSLLMADTWKKPYSREYAAFPAPWLRSSKFWPTTGRVDNV
YGDRNLVCTLQPANEEQAAAAVSA
>O81235 | SODM_ARATH
MAIRCVASRKTLAGLKETSSRLLRIRGIQTFTLPDLPYDYGALEPAISGEIMQIHHQKHH
QAYVTNYNNALEQLDQAVNKGDASTVVKLQSAIKFNGGGHVNHSIFWKNLAPSSEGGGEP
PKGSLGSAIDAHFGSLEGLVKKMSAEGAAVQGSGWVWLGLDKELKKLVVDTTANQDPLVT
KGGSLVPLVGIDVWEHAYYLQYKNVRPEYLKNVWKVINWKYASEVYEKENN
>O81796 | IDH3_ARATH
MARRSVSIFNRLLANPPSPFTSLSRSITYMPRPGDGAPRTVTLIPGDGIGPLVTGAVEQV
MEAMHAPVHFERYEVLGNMRKVPEEVIESVKRNKVCLKGGLATPVGGGVSSLNMQLRKEL
DIFASLVNCINVPGLVTRHENVDIVVIRENTEGEYSGLEHEVVPGVVESLKVITKFCSER
IARYAFEYAYLNNRKKVTAVHKANIMKLADGLFLESCREVAKHYSGITYNEIIVDNCCMQ
LVAKPEQFDVMVTPNLYGNLIANTAAGIAGGTGVMPGGNVGAEHAIFEQGASAGNVGNDK
MVEQKKANPVALLLSSAMMLRHLRFPTFADRLETAVKQVIKEGKYRTKDLGGDCTTQEVV
DAVIAALE
>P00051 | CYC_CUCMA
ASFDEAPPGNSKAGEKIFKTKCAQCHTVDKGAGHKQGPNLNGLFGRQSGTTPGYSYSAAN
KNRAVIWEEKTLYDYLLNPKKYIPGTKMVFPGLKKPQDRADLIAYLKEATA

>P00052 | CYC_PHAAU
ASFDEAPPGNSKSGEKIFKTKCAQCHTVDKGAGHKQGPNLNGLFGRQSGTTAGYSYSTAN
KNMAVIWEEKTLYDYLLNPKKYIPGTKMVFPGLKKPQDRADLIAYLKESTA
>P00053 | CYC_CANSA
ASFBZAPPGBSKAGEKIFKTKCAECHTVGRGAGHKQGPNLNGLFGRQSGTTAGYSYSAAN
KNMAVTWZZKTLYDYLLNPKKYIPGTKMVFPGLKKPZBRADLIAYLKESTA
>P00054 | CYC_SESIN
ASFBZAPPGBVKSGEKIFKTKCAQCHTVDKGAGHKQGPNLNGLFGRQSGTTPGYSYSAAN
KNMAVIWGENTLYDYLLNPKKYIPGTKMVFPGLKKPQERADLIAYLKEATA
>P00056 | CYC_MAIZE
ASFSEAPPGNPKAGEKIFKTKCAQCHTVEKGAGHKQGPNLNGLFGRQSGTTAGYSYSAAN
KNKAVVWEENTLYDYLLNPXKYIPGTKMVFPGLXKPQERADLIAYLKEATA
>P00057 | CYC_RICCO
ASFBZAPPGBVKAGEKIFKTKCAQCHTVEKGAGHKQGPNLNGLFGRQSGTTAGYSYSAAN
KNMAVQWGENTLYDYLLNPKKYIPGTKMVFPGLKKPQDRADLIAYLKZATA
>P00058 | CYC_GOSBA
ASFQZAPPGBAKAGEKIFKTKCAQCHTVDKGAGHKQGPNLNGLFGRQSGTTAGYSYSAAN
KNMAVQWGENTLYDYLLNPKKYIPGTKMVFPGLKKPQDRADLIAYLKZSTA
>P00059 | CYC_ABUTH
ASFQZAPPGBAKAGEKIFKTKCAQCHTVEKGAGHKQGPNLNGLFGRQSGTTPGYSYSAAN
KNMAVNWGENTLYDYLLNPKKYIPGTKMVFPGLKKPQDRADLIAYLKZSTA
>P00060 | CYC_SOLLC
ASFNEAPPGNPKAGEKIFKTKCAQCHTVEKGAGHKEGPNLNGLFGRQSGTTAGYSYSAAN
KNMAVNWGENTLYDYLLNPKKYIPGTKMVFPGLKKPQERADLIAYLKEATA
>P00061 | CYC_SOLTU
ASFGEAPPGNPKAGEKIFKTKCAQCHTVDKGAGHKEGPNLNGLFGRQSGTTAGYSYSNAN
KNMAVTWGENTLYDYLLNPKKYIPGTKMVFPGLKKPQERADLIAYLKEATA
>P00062 | CYC_SAMNI
ASFAEAPPGNPKAGEKIFKTKCNQCHTVDKGAGHKQGPNLNGLFGRQSGTTAGYSYSAAN
KNMAVNWEEKTLYDYLLNPKKYIPGTKMVFPGLKKPQDRADLIAYLKQSTA
>P00063 | CYC_ACENE
ASFAEAPPGNPAAGEKIFKTKCAQCHTVDKGAGHKQGPNLNGLFGRQSGTTAGYSYSAAN
KNMAVNWGYNTLYDYLLNPKKYIPGTKMVFPGLKKPQDRADLIAYLKQSTAA
>P00064 | CYC_ALLPO
ATFSZAPPGBZKAGQKIFKLKCAQCHTVEKGAGHKQGPNLNGLFGRQSGTAAGYSYSAAN
KNMAVVWZZBTLYDYLLNPKKYIPGTKMVFPGLKKPQDRADLIAYLKESTA
>P00065 | CYC_ARUMA
ASFAEAPPGNPKAGEKIFKTKCAQCHTVEKGAGHKQGPNLNGLFGRQSGTTAGYSYSAAN
KNMAVIWEESTLYDYLLNPXKYIPGTKMVFPGLXKPQERADLIAYLKESTA
>P00066 | CYC_NIGDA
ASFBZAPAGBSASGEKIFKTKCAZCHTVBZGAGHKZGPNLHGLFGRQSGTVAGYSYSAAN
KNKAVNWEEKTLYDYLLNPKKYIPGTKMVFPGLKKPZZRABLLAYLKESTA
>P00067 | CYC_TROMA
ASFAEAPAGDNKAGDKIFKNKCAQCHTVDKGAGHKQGPNLNGLFGRQSGTTAGYSYSAAN

KNKAVLWZZATLYDYLLNPKKYIPGTKMVFPGLKKPQDRADLIAYLKESTA
>P00068 | CYC_WHEAT
ASFSEAPPGNPDAGAKIFKTKCAQCHTVDAGAGHKQGPNLHGLFGRQSGTTAGYSYSAAN
KNKAVEWEENTLYDYLLNPKKYIPGTKMVFPGLKKPQDRADLIAYLKKATSS
>P00069 | CYC_GUIAB
ASFAEAPAGDAKAGEKIFKTKCAZCHTVZKGAGHKQGPNLNGLFGRQSGTTAGYSYSAAN
KNKAVAWZZBSLYDYLLNPKKYIPGTKMVFPGLKKPZZRADLIAYLKASTA
>P00070 | CYC_HELAN
MASFAEAPAGNPTTGEKIFKTKCAQCHTVEKGAGHKQGPNLNGLFGRQSGTTAGYSYSAG
NKNKNKAVIWEENTLYDYLLNPKKYIPGTKMVFPGLKKPQERADLIAYLKTSTA
>P00071 | CYC_PASSA
ASFAEAPPGDKDVGGKIFKTKCAZCHTVZLGAGHKQGPNLNGLFGRQSGTTAGYSYSAAN
KNKAVLWABBTLYDYLLNPKKYIPGTKMVFPGLKKPQDRADLIAYLKHATA
>P00072 | CYC_FAGES
ATFSEAPPGNIKSGEKIFKTKCAQCHTVEKGAGHKQGPNLNGLFGRQSGTTAGYSYSAAN
KNKAVTWGEDTLYEYLLNPKKYIPGTKMVFPGLKKPQERADLIAYLKBSTZ
>P00073 | CYC_SPIOL
ATFSEAPPGNKDVGAKIFKTKCAQCHTVDLGAGHKQGPNLNGLFGRQSGTAASYSYSAAN
KNKAVIWSEDTLYEYLLNPKKYIPGTKMVFPGLKKPQDRADLIAYLKDSTQ
>P00074 | CYC_GINBI
ATFSEAPPGDPKAGEKIFKTKCAZCHTVZKGAGHKQGPNLHGLFGRQSGTTAGYSYSTGN
KNKAVNWGZZTLYEYLLNPKKYIPGTKMVFPGLKKPZZRADLISYLKQATSQE
>P00075 | CYC_ENTIN
STFABAPPGBPAKGAKIFKAKCAZCHTVBAGAGHKQGPNLNGAFGRTSGTAAGFSYSAAB
KBKTADWBZBTLYDYLLNPKKYIPGTKMVFAGLKKPZBRADLIAFLKDATA
>P00412 | COX2_MAIZE
MILRLLECRFFTIALCDAAEPWQLGFQDAATPMMQGIIDLHHDIFFFLILILVFVLWMLV
RALWHFNEQTNPIPQRIVHGTTIEIIWTIFPSVILLFIAIPSFALLYSMDGVLVDPAITI
KAIGHQWYWTYEYSDYNSSDEQSLTFDSYMIPEDDLELGQLRLLEVDNRVVVPAKTHLRM
IVTSADVLHSWAVPSLGVKCDAVPGRLNLTSILVQREGVYYGQCSEICGTNHAFMPIVVE
AVTLKDYADWVSNQLILQTN
>P00413 | COX2_WHEAT
MILRSLSCRFLTIALCDAAEPWQLGFQDAATPMMQGIIDLHHDIFFFLILILVFVLWMLV
RALWHFNEQTNPIPQRIVHGTTIEIIWTIFPSVILLFIAIPSFALLYSMDGVLVDPAITI
KAIGHQWYWTYEYSDYNSSDEQSLTFDSYTIPEDDPELGQSRLLEVDNRVVVPAKTHLRM
IVTPADVLHSWAVPSLGVKCDAVPGRLNLTSILVQREGVYYGQCSEICGTNHAFMPIVVE
AVTLKDYADWVSNQLILQTN
>P04373 | COX2_ORYSJ
MILRSLECRFLTIALCDAAEPWQLGSQDAATPMMQGIIDLHHDIFFFLILILVFVSRMLV
RALWHFNEQTNPIPQRIVHGTTIEIIRTIFPSVIPLFIAIPSFALLYSMDGVLVDPAITI
KAIGHQWYRSYEYSDYNSSDEQSLTFDSYTIPEDDPELGQSRLLEVDNRVVVPAKTHLRM
IVTPADVLHSWAVPSSGVKCDAVPGRSNLTSISVQREGVYYGQCSEICGTNHAFTPIVVE
AVTLKDYADWVSNQLILQTN
>P05488 | RT13_TOBAC

MLYISGARLVGDEQVRIASTKIDGIGPKKAIQVRYRLGISGNIKIKELTKYQIDQIEQMI
GQDHVVHWELKRGERADIERLISISCYRGIRHQDGSPLRGQRTHTNARTCRKLIRK
>P05490 | COX2_OENBE
MIVNECLFFTIALCDAAEPWQLGFQDAATPMMQGIIDLHHDILFFLILILVFVLWILVRA
LWHFYYKKNPIPQRIVHGTTIEILWTIFPSIILMFIAIPSFALLYSMDEVVVDPAMTLKA
IGHQWYWTYEYSDYNSSDEQSLTFDSYMIPEDDLELGQLRLLEVDNRVVVPVKTNLRLIV
TSADVLHSWAVPSLGVKCDAVPGRLNQISMLVQREGVYYGQCSEICGTNHAFMPIVIEAV
SATDYTNWVSNLFIPPTS
>P05491 | COX2_SOYBN
MKFEWLFLTIAPCDAAEPWQLGFQDAATPMMQGIIDLHHDIFFFLILILVFVSRILVRAL
WHFHYKKNPIPQRIVHGTTIEILRTIFPSIIPMFIAIPSFALLYSMDEVVVDPAITIKAI
GHQWYRTYEYSDYNSSDEQSLTFDSYTIPEDDLELGQSRLLEVDNRVVVPAKTHLRIIVT
PADVPHSWAVPSLGVKCDAVPGRLNQISISVQREGVYYGQCSEICGTNHAFTPIVVEAVP
SKDYGSRVFNQLIPQTTGEA
>P05492 | ATPAM_OENBI
MEFSPRAAELTTLLESRITNFYTNFQVDEIGRVISVGDGIARVYGLNEIQAGEMVEFASG
VKGIALNLENENVGIVVFGSDTAIKEGDLVKRTGSIVDVPAGKSLLGRVVDALGVPIDGR
GALGDHERRRVEVKVPGIIERKSVHEPMQTGLKAVDSLVPIGRGQRELIIGDRQTGKTAI
AIDTILNQKQMNSRATSESETLYCVYVAIGQKRSTVAQLVQILSEGNALEYSILVAATAS
DPAPLQFLAPYSGCAMGEYFRDNGMHALIIYDDLSKQAVAYRQMSLLLRRPPGREAFPGD
VFYLHSRLLERAAKRSDQTGAGSLTALPVIETQAGDVSAYIPTNVISITDGQICLETELF
YRGIRPAINVGLSVSRVGSAAQLKAMKQVCGSLKLELAQYREVAAFAQFGSDLDAATQAL
LNRGARLTEILKQPQYAPLPIEKQIIVIYAAVNGFCDRMPLDRISQYERAIPQSVKQELL
QSLVEKGGLNNERKIEPDAFLKENAKPYIKG
>P05493 | ATPAM_PEA
MEFSVRAAELTTLLESRITNFYTNFQVDEIGRVVSVGDGIARVYGLNEIQAGELVEFASG
VKGIALNLENENVGIVVFGSDTSIKEGDLVKRTGSIVDVPAGKAMLGRVVDALGVPIDGR
GALSDHERRRVEVKAPGIIERKSVHEPMQTGLKAVDSLVPIGRGQRELIIGDRQTGKTAI
AIDTILNQKQMNSRATSESETLYCVYVAIGQKRSTVAQLVQILSEANALEYSILVAATAS
DPAPLQFLAPYSGCAMGEYFRDNGMHALIIYDDLSKQAVAYRQMSLLLRRPPGREAFPGD
VFYLHSRLLERAAKRSDQTGAGSLTALPVIETQAGDVSAYIPTNVISITDGQICLETELF
YRGIRPAINVGLSVSRVGSAAQLKAMKQVCGSLKLELAQYREVAAFAQFGSDLDAATQAL
LNRGARLTEVLKQPQYAPLPIEKQILVIYAAVNGFCDRMPLDKIAQYERDILSTIKQELL
QSLKGGLTGERKIEPDAFLKEKALSLI
>P05494 | ATPAM_MAIZE
MEFSPRAAELTTLLESRMINFYTNLKVDEIGRVVSVGDGIARVYGLNEIQAGEMVEFASG
VKGIALNLENENVGIVVFGSDTAIKEGDLVKRTGSIVDVPAGKAMLGRVVDALGVPIDGK
GALSDHERRRVEVKAPGIIERKSVHEPMQTGLKAVDSLVPIGRGQRELIIGDRQTGKTAI
AIDTILNQKQMNSRGTNESETLYCVYVAIGQKRSTVAQLVQILSEANALEYSMLVAATAS
DPAPLQFLAPYSGCAMGEYFRDNGMHALIIYDDLSKQAVAYRQMSLLLRRPPGREAFPGD
VFYLHSRLLERAAKRSDQTGAGSLTALPVIETQAGDVSAYIPTNVISITDGQICLETELF
YRGIRPAINVGLSVSRVGSAAQLKAMKQVCGSSKLELAQYREVAAFAQFGSDLDAATQAL
LNRGARLTEVPKQPQYEPLPIEKQIVVIYAAVNGFCDRMPLDRISQYEKNILSTINPELL
KSFLEKGGLTNERKMEPDASLKESALNL

>P05495 | ATPAM_NICPL
MELSPRAAELTSLLESRISNFYTNFQVDEIGRVVSVGDGIARVYGLNEIQAGEMVEFASG
VKGIALNLENENVGIVVFGSDTAIKEGDLVKRTGSIVDVPAGKAMLGRVVDGLGVPIDGR
GALSDHERRRVEVKAPGIIERKSVHEPMQTGLKAVDSLVPIGRGQRELIIGDRQTGKTAI
AIDTILNQKQLNSRATSESETLYCVYVAIGQKRSTVAQLVQILSEANALEYSILVAATAS
DPAPLQFLAPYSGCAMGEYFRDNGMHALIIYDDLSKQAVAYRQMSLLLRRPPGREAFPGD
VFYLHSRLLERAAKRSDQTGAGSLTALPVIETQAGDVSAYIPTNVIPITDGQICLETELF
YRGIRPAINVGLSVSRVGSAAQLKTMKQVCGSSKLELAQYREVAALAQFGSDLDAATQAL
LNRGARLTEVPKQPQYAPLPIEKQILVIYAAVNGFCDRMPLDRISQYERAIPNSVKPELL
QSFLEKGGLTNERKMEPDTFLKESALAFI
>P05500 | ATP6_OENBE
MKRFYKTAFFSEIGSEEVSHFWADTMSSHSPLEQFSILPLIPMNIGNLYFSFTNSSLFML
LTLSLVLLLVNFVTKKGGGNLVPNAWQSLVELIYDFVLNLVNEQIGGLSGNVKQKFFPCI
LVTFTFLLFCNLQGMIPYSFTVTSHFLITLGLSFSIFIGITIVGFQRNGLHFLSFLLPAG
VPLPLAPFLVLLELISYCFRALSLGIRLFANMMAGHSLVKILSGFAWTMLCMNDLFYFIG
DLGPLFIVLALTGLELGVAILQAYVFTILICIYLNDAINLH
>P07506 | COX1_SOYBN
MTNPVRWLFSTNHKDIGTLYFIFGAIAGVMGTCFSVLIRMELARPGDQILGGNHQLYNVL
ITGHAFLMIFFMVMPAMIGGSGNWSVPILIGAPDMAFPRLNNISFWLLPPSLLLLLSSAL
VEVGSGTGWTVYPPLSGITSHSGGAVDSAISSLHLSGVSSILGSINFITTISNMRGPGMT
MHRSPLFVWSVPVTAFPLLLSLPVLAGAITMLLTDRNFNTTFSDPAGGGDPILYQHLFRF
FGHPEVYIPILPGSGIISHIVSTFSGKPVFGYLGMVYAMISIGVLGFLVWAHHMFTVGLD
VDTRAYFTAATMIIAVPTGIKIFSWIATMWGGSIQYKTPMLFAVGFIFLFTIGGLTGIVL
ANSGLDIALHDTYYVVAHFHYVLSMGAVFALFAGFHYWVGKIFGRTYPETLGQIHFWITF
FGVNLTLFPMHFLGLSGMPRRIPDYPDAYAGWNALSSFGSYISVVGIRRFFVVVTITSSS
GNNITRANIPWAVEQNSTTLEWLVQSPPAFHTFGELPAIKETKSYVK
>P07924 | RT13_WHEAT
MSYISGARSLPDEQVRIASTKMDGIGPKKAIQLRYRLGISGNIKMNELTKYQIDQIEQMI
AQDHVVHWELKRGERADIERLISISRYRGIRHQDGSPLRGQRTHTNARTARKQIRK
>P07925 | ATP6_MAIZE
MERNGEIVNNGSIIIPGGGGPVTESPLDQFGIHPILDLNIGKYYVSFTNLSLSMLLTLGL
VLLLVFVVTKKGGGKSVPNAFQSLVELIYDFVPNLVNEQIGGLSGNVKHKFFPCISVTFT
FSLFRNPQGMIPFSFTVTSHFLITLALSFSIFIGITIVGFQRHGLHFFSFLLPAGVPLPL
APFLVLLELISHCFRALSSGIRLFANMMAGHSSVKILSGFAWTMLFLNNIFYFLGDLGPL
FIVLALTGLELGVAISQAHVSTISICIYLNDATNLHQNESFHNCIKTRSQS
>P08681 | COX1_CHLRE
MRWLYSTSHKDIGLLYLVFAFFGGLLGTSLSMLIRYELALPGRGLLDGNGQLYNVIITGH
GIIMLLFMVMPALFGGFGNWLLPIMIGAPDMAFPRLNNISFWLNPPALALLLLSTLVEQG
PGTGWTAYPPLSVQHSGTSVDLAILSLHLNGLSSILGAVNMLVTVAGLRAPGMKLLHMPL
FVWAIALTAVLVILAVPVLAAALVMLLTDRNINTAYFCESGDLILYQHLFWFFGHPEVYI
LILPAFGIVSQVVSFFSQKPVFGLTGMICAMGAISLLGFIVWAHHMFTVGLDLDTVAYFT
SATMIIAVPTGMKIFSWMATIYSGRVWFTTPMWFAVGFICLFTLGGVTGVVLANAGVDML
VHDTYYVVAHFHYVLSMGAVFGIFAGVYFWGNLITGLGYHEGRAMVHFWLLFIGVNLTFF
PQHFLGLAGMPRRMFDYADCFAGWNAVSSFGASISFISVIVFATTFQEAVRTVPRTATTL

EWVLLATPAHHALSQVPVLRTASSH
>P08742 | COX1_MAIZE
MTNLVRWLFSTNHKDIGTLYFIFGAIAGVMGTCFSVLIRMELARPGDQILGGNHQLYNVL
ITAHAFLMIFFMVMPAMIGGFGNWFVPILIGAPDMAFPRLNNISFWLLPPSLLLLLSSAL
VEVGSGTGWTVYPPLSGITSHSGGAVDLAIFSLHLSGVSSILGSINFITTIFNMRGPGMT
MHRLPLFVWSVLVTAFLLLLSLPVLAGAITMLLTDRNFNTTFFDPAGGGDPILYQHLFWF
FGHPEVYILILPGFGIISHIVSTFSRKPVFGYLGMVYAMISIGVLGFLVWAHHMFTVGLD
VDTRAYFTAATMIIAVPTGIKIFSWIATMWGGSIQYKTPMLFAVGFIFLFTIGGLTGIVL
ANSGLDIALHDTYYVVAHFHYVLSMGAVFALFAGFYYWVGKIFGRTYPETLGQIHFWITF
FGVNLTFFPMHFLGLSGMPRRIPDYPDAYAGWNALSSFGSYISVVGIRRFFVVVAITSSS
GKNKRCAESPWAVEQNPTTLEWLVQSPPAFHTFGELPTIKETRNQSSC
>P08743 | COX1_OENBE
MTNPVRWLFSTNHKDIGTLYFIFGAIAGVMGTCFSVLIRMELARPGDQILGGNHQLYNVL
ITAHAFLMIFFMVMPAMIGGSGNWSVPILIGAPDMAFPRLNNISFWLLPPSLLLLLSSAL
VEVGSGTGWTVYPPLSGITSHSGGAVDSAISSLHLSGVSSILGSINFITTISNMRGLGMT
MHRSPLFVWSVLATAFPILLSLPVLAGAITMLLTDRNFNTTFSDPAGGGDPILYQHLFRF
FGHPEVYILILPGSGIISHIVSTFSGKPVFGYLGMVYAMISIGVLGFLVWAHHMFTVGLD
VDTRAYFTAATMIIAVPTGVKIFSWIATMWGGSIQYKTPMLFAVGSIFLFTVGGLAGIVP
ANSGLDIALHDTYYAGAHFHYVLSMGAVFALFAGFRYWVGKIFGRTYPETLGQIHFWITF
FGVNPTFFPMHFLGLSGMPRPIPDYPESYAGWNALSSFGSYISVVGIRCFFVVVTITSSS
GNNKRCAPSPWAVEKNSTTLEWMVQSPPAFHTFGELPATKETKSYVK
>P08744 | COX2_PEA
MKLEWLFLTIAPCDAAEPWQLGFQDAATPMMQGIIDLHHDIFFFLILILVFVSRILVRAL
WHFHYKKNPIPQRIVHGTTIEILRTIFPSIIPMFIAIPSFALLYSMDGVLVDPAMTIKAI
GHQWYRTYEYSDYNSSDEQSLTFDSYTIPEDDLELGQSGLLEVDNRVVVPAKTHLRIIVT
PADVPHSWAVPSLGVKCDAVPGRLNQISISVQREGVYYGQCSEICGTNHAFPIVVEAVPS
KDYGSRVSNQLIPQTGEA
>P08977 | RT13_MAIZE
MSYISGARSLPDEQVRIASTKMDGIGPKKAIQLRYRLGISGNIKIHELTKYQIDQIEQMI
AQDHVVHWELKRGERADIERLISISRYRGIRHQDGSPLRGQRTHTNARTARKQIRKGNER
RLPKEQATD
>P0C520 | ATPAM_ORYSA
MEFSPRAAELTTLLESRMTNFYTNFQVDEIGRVVSVGDGIARVYGLNEIQAGEMVEFASG
VKGIALNLENENVGIVVFGSDTAIKEGDLVKRTGSIVDVPAGKAMLGRVVDALGVPIDGK
GALSDHERRRVEVKAPGIIERKSVHEPMQTGLKAVDSLVPIGRGQRELIIGDRQTGKTAI
AIDTILNQKQMNSRGTNESETLYCVYVAIGQKRSTVAQLVQILSEANALEYSILVAATAS
DPAPLQFLAPYSGCAMGEYFRDNGMHALIIYDDLSKQAVAYRQMSLLLRRPPGREAFPGD
VFYLHSRLLERAAKRSDQTGAGSLTALPVIETQAGDVSAYIPTNVISITDGQICLETELF
YRGIRPAINVGLSVSRVGSAAQLKAMKQVCGSLKLELAQYREVAAFAQFGSDLDAATQAL
LNRGARLTEVSKQPQYEPLPIEKQIVVIYAAVNGFCDRMPLDRISQYEKAILSTINPELL
KSFNEKGGLTNERKIELDAFLKQTAKEIN
>P0C521 | ATPAM_ORYSI
MEFSPRAAELTTLLESRMTNFYTNFQVDEIGRVVSVGDGIARVYGLNEIQAGEMVEFASG
VKGIALNLENENVGIVVFGSDTAIKEGDLVKRTGSIVDVPAGKAMLGRVVDALGVPIDGK

GALSDHERRRVEVKAPGIIERKSVHEPMQTGLKAVDSLVPIGRGQRELIIGDRQTGKTAI
AIDTILNQKQMNSRGTNESETLYCVYVAIGQKRSTVAQLVQILSEANALEYSILVAATAS
DPAPLQFLAPYSGCAMGEYFRDNGMHALIIYDDLSKQAVAYRQMSLLLRRPPGREAFPGD
VFYLHSRLLERAAKRSDQTGAGSLTALPVIETQAGDVSAYIPTNVISITDGQICLETELF
YRGIRPAINVGLSVSRVGSAAQLKAMKQVCGSLKLELAQYREVAAFAQFGSDLDAATQAL
LNRGARLTEVSKQPQYEPLPIEKQIVVIYAAVNGFCDRMPLDRISQYEKAILSTINPELL
KSFNEKGGLTNERKIELDAFLKQTAKEIN
>P0C522 | ATPAM_ORYSJ
MEFSPRAAELTTLLESRMTNFYTNFQVDEIGRVVSVGDGIARVYGLNEIQAGEMVEFASG
VKGIALNLENENVGIVVFGSDTAIKEGDLVKRTGSIVDVPAGKAMLGRVVDALGVPIDGK
GALSDHERRRVEVKAPGIIERKSVHEPMQTGLKAVDSLVPIGRGQRELIIGDRQTGKTAI
AIDTILNQKQMNSRGTNESETLYCVYVAIGQKRSTVAQLVQILSEANALEYSILVAATAS
DPAPLQFLAPYSGCAMGEYFRDNGMHALIIYDDLSKQAVAYRQMSLLLRRPPGREAFPGD
VFYLHSRLLERAAKRSDQTGAGSLTALPVIETQAGDVSAYIPTNVISITDGQICLETELF
YRGIRPAINVGLSVSRVGSAAQLKAMKQVCGSLKLELAQYREVAAFAQFGSDLDAATQAL
LNRGARLTEVSKQPQYEPLPIEKQIVVIYAAVNGFCDRMPLDRISQYEKAILSTINPELL
KSFNEKGGLTNERKIELDAFLKQTAKEIN
>P12786 | COX1_PEA
MTNPVRWLFSTNHKDIGTLYFIFGAIAGVMGTCFSVLIRMELARPGDQILGGNHQLYNVL
ITAHAFFMIFFMVMPAMIGGSGNWSVPILIGAPDMAFPRLNNISFWLLPPSLLLLLSSAL
VEVGSGTGWTVYPPLSGITSHSGGAVDSAISSLHLSGVSSILGSINFLTTISNMRGPGMT
MHRSPLFVWSVPVTAFPLLLSLPVLAGAITMLLTDRNFNTTFSDPAGGGDPILYQHLFRF
FGHPEVYIPILPGSGIISHIVSTFSGKPVFGYLGMVYAMISIGVLGFLVWAHHMFTVGLD
VDTRAYFTAATMIIAVPTGIKIFSWIATMWGGSIQYKTPMLFAVGFIFLFTIGGLTGIVP
ANSGLDIALHDTYYVVAHFHYVLSMGAVFALFAGFHYWVGKIFGRTYPETLGKIHFWITF
FGVNLTLFPMHFLGLSGMPRRIPDYPDAYAGWNALSSFGSYISVVGIRRFFVVVTITSSS
GNNITRANIPWAVEQNSTTLEWLVQSPPAFHTFGELPAIKETKSYVK
>P12857 | ADT2_MAIZE
MADQANQPTVLHKLGGQFHLSSSFSEGVRARNICPSFSPYERRFATRNYMTQSLWGPSMS
VSGGINVPVMPTPLFANAPAEKGGKNFMIDFMMGGVSAAVSKTAAAPIERVKLLIQNQDE
MIKSGRLSEPYKGIADCFKRTIKDEGFSSLWRGNTANVIRYFPTQALNFAFKDYFKRLFN
FKKDRDGYWKWFAGNLASGGAAGASSLFFVYSLDYARTRLANDAKAAKGGGDRQFNGLVD
VYRKTLKSDGIAGLYRGFNISCVGIIVYRGLYFGLYDSIKPVVLTGSLQDNFFASFALGW
LITNGAGLASYPIDTVRRRMMMTSGEAVKYKSSLDAFQQILKKEGPKSLFKGAGANILRA
IAGAGVLSGYDQLQILFFGKKYGSGGA
>P12862 | ATPAM_WHEAT
MEFSPRAAELTTLLESRMTNFYTNFQVDEIGRVVSVGDGIARVYGLNEIQAGEMVEFASG
VKGIALNLENENVGIVVFGSDTAIKEGDLVKRTGSIVDVPAGKAMLGRVVDALGVPIDGK
GALSDHERRRVEVKAPGIIERKSVHEPMQTGLKAVDSLVPIGRGQRELIIGDRQTGKTAI
AIDTILNQKQMNSRGTNESETLYCVYVAIGQKRSTVAQLVQILSEANALEYSILVAATAS
DPAPLQFLAPYSGCAMGEYFRDNGMHALIIYDDLSKQAVAYRQMSLLLRRPPGREAFPGD
VFYLHSRLLERAAKRSDQTGAGSSTALPVIETQAGDVSAYIPTNVISITDGQICLETDVF
YRGIRPAINVGLSVSRVGSAAQLKAMKQVCGSSKLELAQYREVAAFAQFGSDLDAASQAL
LNRGARLTEVPKQPQYEPLPIEKQIVVIYAAVNGFCDRMPLDRISQYEKAILSTINPELQ

KSFLEKGGLTNERKMEPDASLKESTLPYL
>P14578 | COX1_ORYSJ
MTNLVRWLFSTNHKDIGTLYFIFGAIAGVMGTCFSVLIRMELARPGDQILGGNHQLYNVL
ITAHAFLMIFFMVMPAMIGGFGNWFVPILIGAPDMAFPRLNNISFWLLPPSLLLLLSSAL
VEVGSGTGWTVYPPLSGITSHSGGAVDLAIFSLHLSGVSSILGSINFITTIFNMRGPGMT
MHRLPLFVWSVLVTAFLLLLSLPVLAGAITMLLTDRNFNTTFFDPAGGGDPILYQHLFWF
FGHPEVYILILPGFGIISHIVSTFSRKPVFGYLGMVYAMISIGVLGFLVWAHHMFTVGLD
VDTRAYFTAATMIIAVPTGIKIFSWIATMWGGSIQYKTPMLFAVGFIFLFTIGGLTGIVL
ANSGLDIALHDTYYVVAHFHYVLSMGAVFALFAGFYYWVGKIFGRTYPETLGQIHFWITF
FGVNLTFFPMHFLGLSGMPRRIPDYPDAYAGWNALSSFGSYISVVGIRRFFVVVAITSSS
GKNKRCAESPWAVEQNPTTLEWLVQSPPAFHTFGELPAIKETKS
>P14875 | RT14_OENBE
MEKRNIRDHKRRLLATKYELRRKLYKAFCNDPALPSDMRDKHRYKLSKLPRNSSFARVRN
RCISTGRPRSVYEFFRISRIVFRGLASRGPLMGIKKSSW
>P15451 | CYC_CHLRE
MSTFAEAPAGDLARGEKIFKTKCAQCHVAEKGGGHKQGPNLGGLFGRVSGTAAGFAYSKA
NKEAAVTWGESTLYEYLLNPKKYMPGNKMVFAGLKKPEERADLIAYLKQATA
>P15758 | RT13_OENBE
MSYISGARLVADEQVRIASTKMDGIGPKKAIQVRSRLGGNIKRKELTKYQIDQIEQMRGQ
DHVVHWELKRGERADIERFISISCYRGIRHQDGLPLRGQRSHTNARTSRKRIRK
>P16048 | GCSH_PEA
MALRMWASSTANALKLSSSSRLHLSPTFSISRCFSNVLDGLKYAPSHEWVKHEGSVATIG
ITDHAQDHLGEVVFVELPEPGVSVTKGKGFGAVESVKATSDVNSPISGEVIEVNTGLTGK
PGLINSSPYEDGWMIKIKPTSPDELESLLGAKEYTKFCEEEDAAH
>P16265 | NU3M_MAIZE
MLEFAPICIYLVISLLVSLILLGVPFLFASNSSTYPEKLSAYECGFDPFGDARSRFDIRF
YLVSILFIIFDLEVTFFFPWAVSLNKIDLFGFWSMMAFLLILFIGSLYEWKRGALDWE
>P17614 | ATPBM_NICPL
MASRRLLASLLRQSAQRGGGLISRSLGNSIPKSASRASSRASPKGFLLNRAVQYATSAAA
PASQPSTPPKSGSEPSGKITDEFTGAGSIGKVCQVIGAVVDVRFDEGLPPILTALEVLDN
QIRLVLEVAQHLGENMVRTIAMDGTEGLVRGQRVLNTGSPITVPVGRATLGRIINVIGEA
IDERGPITTDHFLPIHREAPAFVEQATEQQILVTGIKVVDLLAPYQRGGKIGLFGGAGVG
KTVLIMELINNVAKAHGGFSVFAGVGERTREGNDLYREMIESGVIKLGEKQSESKCALVY
GQMNEPPGARARVGLTGLTVAEHFRDAEGQDVLLFIDNIFRFTQANSEVSALLGRIPSAV
GYQPTLATDLGGLQERITTTKKGSITSVQAIYVPADDLTDPAPATTFAHLDATTVLSRQI
SELGIYPAVDPLDSTSRMLSPHILGEDHYNTARGVQKVLQNYKNLQDIIAILGMDELSED
DKMTVARARKIQRFLSQPFHVAEVFTGAPGKYVDLKESINSFQGVLDGKYDDLSEQSFYM
VGGIDEVIAKAEKIAKESAA
>P18260 | ATPAM_HELAN
MEFSPRAAELTTLLESRISNFYTNFQVDEIGRVVSVGDGIARVYGLNEIQAGEMVEFASG
VKGIALNLENENVGIVVFGSDTAIKEGDLVKRTGSIVDVPAGKAMLGRVVDALGVPIDGR
GALSDHERRRVEVKAPGIIERKSVHEPMQTGLKAVDSLVPIGRGQRELIIGDRQTGKTAI
AIDTILNQKQMNSRSTSESETLYCVYVAIGQKRSTVAQLVQILSEANAMEYSILVAATAS
DPAPLQFLAPYSGCAMGEYFRDNGMHALIIYDDLSKQAVAYRQMSLLLRRPPGREAFPGD

VFYLHSRLLERAAKRSDQTGAGSLTALPVIETQAGDVSAYIPTNVIPITDGQICSETELF
YRGIRPAINVGLSVSRVGSAAQLKTMKQVCGSSKLELAQYREVAALAQFGSDLDAATQAL
LNRGARLTEVPKQPQYAPLPIEKQILVIYAAVNGFCDRMPLDRISQYERAILKSIKTELL
QSLLEKGGLTNERKMEPDTFLKECALPYTI
>P18630 | NU3M_OENBE
MLEFAPICISLVISLLLSLILLVVPFLFSSNSSTYPEKLSAYECGFDPFGDARSRFDIRF
YLVSILFIIFDLEVTFFFPWAVSFNKIDLFGFWSMMAFLLILTIGFLYEWKRGALDWE
>P20113 | NU4M_CHLRE
MFISVLLILFALCVTLIPEAHYHMVRVWSFVATIIPMWVVTWMWWNFDASGHGLQMLVIL
GRSHLAFGIDGVALSLMLLTTVLFPICMMLLRTVAGFMTFILLEVLVLSALCVLDLLGFY
ILFEASLILLFLLIGRAPYGSLEAAYKIVLYTMAGSLVLLPTLFMIYSECGTTNVLYMTC
AYNHQTVLGWGLLAVLAVKIPLMPVHLWLPEAHVAAPTAGSVLLAGVLLKLGGIGFLRFM
LPVVPEFCVSVFPLVSTLCLVSFLFSTLSTLRQIDLKKIVAYSSIAHMSMVTLAIFSQSE
FSAYSSSFLMIAHGLISPALFLIVGILYDRAHTKFILYFSGLGASMPIGSTLFFLFTLGN
LAFPLFPNFIAEVLCMVSIFAVHELLAYVFCVCQVLGAAYGFWAFNRVVHGLPRGPADVT
RTEFHTVLPLLIGAVWLGIKPMA
>P22201 | ATPAM_BRANA
MELSPRAAELTNLFESRIRNFYANFQVDEIGRVVSVGDGIAQVYGLNEIQAGEMVLFANG
VKGMALNLENENVGIVVFGGDTAIKEGDLVKRTGSIVDVPAGKAMLGRVVDAMGVPIDGR
GALSDHEQRRVEVKAPGILERKSVHEPMQTGLKAVDSLVPIGRGQRELLIGDRQTGKTTI
AIDTILNQKQINSRATSESETMYCVYVAIGQKRSTVGQLIQTLEEANALEYSILVAATAS
DPAPLQFLAPYSGCAMGEYFRDNGMHALIIYDDLSKQAVAYRQMSLLLRRPPGREAFPGD
VFYLHSRLLERAAKRSDQTGAGSLTALPVIETQAGDVSAYIPTNVISITDGQICLETELF
YRGIRPAINVGLSVSRVGSAAQLKAMKQVCGSSKLELAQYREVAAFAQFGSDLDAATQAL
LNRGARLTEVPKQPQYAPLPIEKQILVIYAAVNGFCDRMPLDRISQYEKAIPNSVKPELL
QALKGGLTNERKMEPDAFLKERALRLI
>P23209 | RT13_DAUCA
MLYISGARLVADKQVRIALTKMYGIGPKKAIQVCYRLGISGNIKIKELTKYQIDQMEQMI
GQDHVVHWELKRGERADIERFISISCYRGIRHQDGLPLRGQRTHTNARTCRKQIRK
>P24459 | ATPAM_PHAVU
MEFSSRAAELTTLLESRMTNFYTNFQVDEIGRVVSVGDGIARVYGLNEIQAGEMVEFASG
VKGIALNLENENVGIVVFGSDTAIKEGDLVKRTGSIVDVPAGKAMLGRVVDALGVPIDGR
GALSDHERRRVEVKAPGIIERKSVHEPMQTGLKAVDSLVPIGRGQRELIIGDRQTGKTAI
AIDTILNQKQMNSRATSESETLYCVYVAIGQKRSTVAQLVQILSEANALEYSILVAATAS
DPAPLQFLAPYSGCAMGEYFRDNGMHALIIYDDLSKQAVAYRQMSLLLRRPPGREAFPGD
VFYLHSRLLERAAKRSDQTGAGSLTALPVIETQAGDVSAYIPTNVISITDGQICLETELF
YRGIRPAINVGLSVSRVGSAAQLKAMKQACGSLKLELAQYREVAAFAQFGSDLDAATQAL
LNRGARLTEVLKQPQYAPLPIEKQILVIYAAVNGFCDRMPLDKIPQYERDILTTIKPELL
QSLKGGLTSERKIELEKFLKEKAKNYTL
>P24794 | COX1_BETVU
MTNLVRWLFSTNHKDIGTLYFIFGAIAGVMGTCFSVLIRMELARPGDQILGGNHQLYNVL
ITAHAFLMIFFMVMPAMIGGFGNWFVPILIGAPDMAFPRLNNISFWLLPPSLLLLLSSAL
VEVGSGTGWTVYPPLSGITSHSGGAVDLAIFSLHLSGVSSILGSINFITTIFNMRGPGMT
MHRLPLFVWSVLVTAFLLLLSLPVLAGAITMLLTDRNFNTTFFDPAGGGDPILYQHLFWF

FGHPEVYILILPGFGIISHIVSTFSGKPVFGYLGMVYAMISIGVLGFLVWAHHMFTVGLD
VDTRAYFTAATMIIAVPTGIKIFSWIATMWGGSIQYKTPMLFAVGFIFLFTVGGLTGIVL
ANSGLDIALHDTYYVVAHFHYVLSMGAVFALFAGFYYWVGKIFGRTYPETLGQIHFWITF
FGVNLTFFPMHFLGLSGMPRRIPDYPDAYAGWNALSSFGSYISVVGICCFFVVVTITLSS
GKNKRCAPSPWAVEENSTTLEWMVQSPPAFHTFGELPAIKETKS
>P25083 | ADT1_SOLTU
MADMNQHPTVFQKAANQLDLRSSLSQDVHARYGGVQPAIYQRHFAYGNYSNAGLQRGQAT
QDLSLITSNASPVFVQAPQEKGFAAFATDFLMGGVSAAVSKTAAAPIERVKLLIQNQDEM
LKAGRLSEPYKGIGECFGRTIKEEGFGSLWRGNTANVIRYFPTQALNFAFKDYFKRLFNF
KKDRDGYWKWFAGNLASGGAAGASSLFFVYSLDYARTRLANDRKASKKGGERQFNGLVDV
YKKTLKSDGIAGLYRGFNISCVGIIVYRGLYFGMYDSLKPVLLTGNLQDSFFASFGLGWL
ITNGAGLASYPIDTVRRRMMMTSGEAVKYKSSLDAFSQIVKNEGPKSLFKGAGANILRAV
AGAGVLAGYDKLQVLVLGKKFGSGGA
>P25855 | GCSH1_ARATH
MALRMWASSTANALKLSSSVSKSHLSPFSFSRCFSTVLEGLKYANSHEWVKHEGSVATIG
ITAHAQDHLGEVVFVELPEDNTSVSKEKSFGAVESVKATSEILSPISGEIIEVNKKLTES
PGLINSSPYEDGWMIKVKPSSPAELESLMGPKEYTKFCEEEDAAH
>P26846 | NU2M_MARPO
MFEHDFLALFPEIFLINATIILLIYGVVFSTSKKYDYPPLVRNVGWLGLLSVLITILLVA
VGSPLAVANLVYNNLIIDNFTYFCQIFLLLSTASTMVMCLDYFKQESLNAFESIVLILLS
TCSMLFMISAYDLIAMYLAIELQSLCFYVIAASKRDSEFSTEAGLKYFILGAFSSGILLF
GCSMIYGFTGVTNFEELAKIFTGYEITLFGAQSSGIFMGILFIAVGFLFKITAVPFHMWA
PDVYEGSPTIVTAFFSIAPKISILANMLRVFIYSFYDPTWQQLFFFCSIASMILGALAAM
AQNKVKRLLAYSSIGHVGYLLIGFSCGTIEGIQSLLIGIFIYVLMTVNVFAIVLALRQNR
FKYIADLGALAKTNPILAITLSITMFSYAGIPPLAGFCSKFYLFFAALGCGAYLLALIGV
VTSVISCFYYIRFVKIMYFDTPKTWVLYKPMDREKSLLLAITVFFITFFFLYPSPLFLVT
HQMALCLCL
>P26847 | NU3M_MARPO
MEFAPIFVYLVISLLLSLILIGVSFLFASSSSLAYPEKLSAYECGFDPFDDARSRFDIRF
YLVSILFIIFDLEVTFLFPWAVSLNKIGLFGFWSMMVFLFILTIGFVYEWKKGALDWE
>P26848 | NU4M_MARPO
MLQVLAPFYSNLSGLILLPLLGSLIILVIPNSRVRLIRGITIWTSLITFLYSLFFWIRFE
NDTAKFQFVETIRWLPYSNINFYIGIDGISLFFVILTTFLTPICILVGFYSVKSYKKEYM
IAFFICESFLIAVFCSLDLLIFYVFFESVLIPMFIIIGVWGSRQRKIKAAYQFFLYTLMG
SLFMLLAILFIFFQTGTTDLQILLTTEFSERRQILLWIAFFASFSVKVPMVPVHIWLPEA
HVEAPTAGSVILAGILLKLGTYGFLRFSIPMFPEATLYFTPFIYTLSVIAIIYTSLTTIR
QIDLKKIIAYSSVAHMNFVTIGMFSLNIQGIEGSILLMLSHGLVSSALFLCVGALYDRHK
TRIVKYYGGLVSTMPIFSTIFLFFTLANMSLPGTSSFIGEFLILVGAFQRNSLVATLAAL
GMILGAAYSLWLYNRVVFGNFKPNFILKFSDLNRREVLIFLPFIVGVIWMGVYPEVFLEC
MHTSVSNLVQHGKFD
>P26850 | NU6M_MARPO
MILFYVFVVLALVSGAMVIRAKNPVHSVLFLILVFCNTSGLLVLLGLDFFAMIFLVVYVG
AIAVLFLFVVMMLHIRIEEIHENVLRYLPVGGIIGLIFLLEIFLMVDNDYIPILPTKLSA
TYLTYTVYAGKIHSWTNLETLGNLLYTTYFFLFLVSSLILLVALIGAIVLTMHKTTKVKR

QDVFIQNAIDFQNTIKKVR
>P26853 | ATP6_MARPO
MACSPLEQFAIIQLIPIHIGNLYFSFTNSSLFMLLTISLVLLLVHFVTLNGGNLVPNAWQ
SFVEMIYDFVLNLVNEQISGASSVKQRFFPLIYVTFTFLLFCNLIGMIPYSFTVTSHFII
TLGLSFSLFIGITIVGFQTHGLHFFSILLPQGVPLPLAPFLVLLELISYCFRALSLGIRL
FANMMAGHSLVKILSGFAWTMLSMGGILYLGQLAPFFIVFALTGLELGVAILQAYVFTIL
LCIYLNDAINLH
>P26856 | COX1_MARPO
MNNFAQRWLFSTNHKDIGTLYLIFGAIAGVMGTCFSVLIRMELAQPGNQILGGNHQLYNV
LITAHAFLMIFFMVMPAMIGGFGNWFVPILIGSPDMAFPRLNNISFWLLPPSLLLLLSSA
LVEVGCGSGWTVYPPLSGITSHSGGSVDLAIFSLHLSGVSSILGSINFITTIFNMRAPGL
TMHRLPLFVWSVLVTAFLLLLSLPVLAGAITMLLTDRNFNTTFFDPAGGGDPILYQHLFW
FFGHPEVYILILPGFGIISHIVSTFSRKPVFGYLGMVYAMISIGVLGFIVWAHHMFTVGL
DVDTRAYFTAATMIIAVPTGIKIFSWIATMWGGSIQYKTPMLFAVGFIFLFTVGGLTGIV
LANSGVDIALHDTYYVVAHFHYVLSMGAVFALFAGFYYWIGKITGLQYPETLGQIHFWIT
FFGVNLTFFPMHFLGLAGMPRRIPDYPDAYAGWNAFSSFGSYVSVVGIFCFFVVVFLTLT
SENKCAPSPWAVEQNSTTLEWMVPSPPAFHTFEELPAIKESI
>P26857 | COX2_MARPO
MNLIWIFPIAFCDAAEPWQLGFQDPATPMMQGIIDLHNDIFFFLIVILIFVLWMLVRALW
HFHYKRNPIPERIVHGTTIEIIWTIFPSIILMFIAIPSFALLYSMDEVVDPAITIKAIGH
QWYWTYEYSDYNSSDEQSLTFDSYMIPEDDLELGQLRLLEVDNRVVVPAKTHLRMIITSA
DVLHSWAVPSLGVKCDAVPGRLNQTSIFIKREGVYYGQCSELCGTNHGFMPIVVEAVSLD
DYVSWVSNKLD
>P26859 | RM02_MARPO
MRNSCWKGKALKQLTFHLKRNSAGRNSSGRITVFHRGGGSKRLQRKIDFKRSTSSMGIVE
RIEYDPNRSSWIALVRWIEGVLRPGKRLAFSKANSRREKNMFFFGLLFSFSSLPRQAQRI
KYEKTRALRPCEQILESSWVLGTRDLRAKEVSLGPLGSFLGLPSIAVAGAKPAFFAFRMK
GPSSLTGRERLSPLRGENTFSQSEGQRWKTQSGAPRRKSLVLSWSQGPKARNGLMISAHD
IGKKDRRPEMAGPHTIPEHAPRALHAVGPSGSGRVLRTSEPFTYILASENLEVGNTVMNF
HGSKPSTLLNYHQPSQKANDPSGLRVEETAWDSQAWLHPRGDYASSENKYILDSYYQMVG
NCIPLAKIPIGTWVHNIERNPGQGAKLTRAAGTFAQIIQKVENTPQCIVRLPSGVDKLID
SRCRATIGIVSNLNHGKRKFNKAGQSRWLGRRPIVRGVAMNPVDHPHGGGEGRTKGGRPS
VSPWGKPTKGGFKTVVRKRRN
>P26860 | RM05_MARPO
MFSPNRNRLEFHYNQVIRPDLLLKINYENIMEVPRLCKIIVVPKAPSNFIKNVKLAMEIV
CGQKFIQTRSRGSTGKSFRFNKFVLNQESKRDTGYVTYLARSTLRGHIMYNFLEKLVTII
SFYDYPVKIQKNSIQLSMATSLLRLFPEIQDHFEIFEHIRGFDVTIVTSANTQDETVILW
SGFLQKEV
>P26861 | RM06_MARPO
MEAKFFCFLEIIGVGYKASTNAQGSILYLKLGFSHEIRLQVTPSVRVFCLKPNLICCTGM
DHQKVTQFAAIVKSCKPPEVYKGKGIQYRNEIIHKKQGKKK
>P26862 | RM16_MARPO
MLYPKRTKFRKYQKGRCKGCKADGTQLCFGKYGIKSCEAGRISYQAIEAARRAISRKFRR
NSKIWVRVFADIPITSKPAEVRMGKGKGNTKGWIARVLKGQILFEMDCVSLSNAQQAATL

AAHKLGLSIKFFKWS
>P26864 | RT02_MARPO
MYNSNLLVIQKLLSTNAYLGHRIPTSDFQGYLYGFRNEMAIIDLEKTLICLRRTCNLIGS
IISAKGHLLLVNTNPEYNKIIQQMAKKTNQSYINHKWIGGFLTNWKHMKKVKKHFQDFSA
HPNLKDAFTSSPFDYFPRFKKMQKCFEGIMTHNIPDCLVIINANQNSMAILEANQLQIPI
VALVDSNIPNRLHKLITYPVPVNDDSIKFVYLFCNLITKTVILSKRSQRPKVKVKRL
>P26865 | RT03_MARPO
MAQKVNPISVRLNLNRSSDSSWFSDYYYGKLLYQDLNFRDYFGSIRPPTGNTFGFRLGRC
IIHHFPKRTFIHVFFLDRLSQSRHQGLGAIPSVKLIRRINDNTVKQRNEVGIWPKKRYEY
HDRLPSIQKIDQLLRVSDWMADIHSTFQSIWPKDENDDRRASEERYAFSRFAPSILVAVR
AEKKKAIFGSEGDFFGFTGRAFLDYFVMQYFFNLKNQIQFDPMVNRSPVAQGVAKTSMIG
KAIPAKTEQGTQSGESICQPRSTLYFDAIIFLRYARFRKATSLSSRYYYLKKMQSLFSNQ
TKTNTLIQPVKIASVYQSASLIAQEISWKLEQKKSFRQICRSIFKQIKKCPYVKGIRIGC
SGRLNGAEIAKTECKKYGETSLHVFSDQIDYAKTQASTPYGILGVKVWVSYFLTQKKGTS
CAISKTYKIS
>P26866 | RT04_MARPO
MFASRFKVCRQILENVWQTKKLTLKQKFLISELQKQKKNKKQSDFSIQLQTIKKLSLFYG
NLPIKKMQRAKTHTYIDKKNSLLFNIEKRLDVILVRLNFCSTMFQARQLISHKNICVNYK
KVNIPGFQVSNGDLISIQENSLDFFKSNIRKNFQTNRIRRMKPNHLEVNYKTLKAVVLYE
PQQIQFPYKIDLDLLD
>P26869 | RT10_MARPO
MTAKICIVIKSFENQRSGLLLNTRKIGLPKKQTLYTVLRSPHIDKKSREQFEMRIHKQLL
VIETETHKLREKLNWLKLHDLLGVQVKIIFYYQTRLDKVCKS
>P26870 | RT11_MARPO
MQKKHGITNMQKKHCITYIQSTFGNTIITLTDYNGNTKTWSSSGSVGFKGSRRSTNYAAQ
ATAENAARVAIQLGFKFVEVRIKGLGYGKESSLRGLKLGGLIITKIRDVTPTPHNGCRPP
KKRRV
>P26871 | RT12_MARPO
MPTMNQLVRKGRESKRRTKRTRALNKCPQKQGVCLRVSTRSPKKPNSALRKIAKVRLTNR
NEIIAYIPGEGHNLQEHSVVMVRGGRVQDLPGVKYHCIRGVKDLQGIPGRRRGRSKYGTK
KPKDYI
>P26872 | RT13_MARPO
MSYILGTNLNSNKQVKIALTRIFGIGPKKAIQVCDQLGLSDTIKVNKLTKYQFDQILKII
SQNYLVDSELKRVIQRDIKRLISIGCYRGFRHNAGLPLRGQRTHTNAKTCRKLRYVSIRS

>P26873 | RT14_MARPO
MSNQIIRDHKRRLLVAKYELKRMHYKAICQDRNLPNKIRYEYFFKLSKLPRNSSKTRVRN
RCIFTGRPRSVYKLFRISRIVFRELASKGSLIGINKSCW
>P26874 | RT19_MARPO
MTRSIWKGPFVDTCLFKQKKIRWRIWSRRSCILPQFVGCYAQIYNGKGFVGLKITEEMVG
HKFGEFASTRKTSSLGKRALPSKTKIKPIKKVR
>P27062 | NU3M_PANGI
MSEFAPICIYLVISLLVSLIPLGVPFPFASNSSTYPDKLSAYECGFDPSGDARSRFDIRF
YLVSILFIIPDPEVTFSFPWAVPPNKIDPFGSWSMMAFLLILTIGSLYEWKRGASDRE

>P27070 | RT12_PANGI
MPTLNQLIRHGREEKRRTDRTRASDQCPQKQGVRPRVPTRTPKKPNSAPRKIAKVRLANR
HDIFPHIPGEGHNSQEHSMVLIRGGRVKDSPGVKSHCIRGVKDLLGIPDRRRGRSKYGAE
KPKSI
>P27080 | ADT_CHLRE
MAKEEKNFMVDFLAGGLSAAVSKTAAAPIERVKLLIQNQDEMIKQGRLASPYKGIGECFV
RTVREEGFGSLWRGNTANVIRYFPTQALNFAFKDKFKRMFGFNKDKEYWKWFAGNMASGG
AAGAVSLSFVYSLDYARTRLANDAKSAKKGGGDRQFNGLVDVYRKTIASDGIAGLYRGFN
ISCVGIVVYRGLYFGMYDSLKPVVLVGPLANNFLAAFLLGWGITIGAGLASYPIDTIRRR
MMMTSGSAVKYNSSFHCFQEIVKNEGMKSLFKGAGANILRAVAGAGVLAGYDQLQVILLG
KKYGSGEA
>P27084 | SODM_PEA
MAARTLLCRKTLSSVLRNDAKPIGAAIAAASTQSRGLHVFTLPDLAYDYGALEPVISGEI
MQIHHQKHHQTYITNYNKALEQLHDAVAKADTSTTVKLQNAIKFNGGGHINHSIFWKNLA
PVSEGGGEPPKESLGWAIDTNFGSLEALIQKINAEGAALQWLGLDKDLKRLVVETTQDPL
VTKGASLVPLLGIDVWEHAYYLQYKNVRPDYLKNIWKVINWKHASEVYEKESS
>P27527 | RT19_PETHY
MPRRSIWKGSFVDAFLLRMKKKRDLLFNRKIWSRRSSILPEFVDCFVRIYNGKTFVRCKI
TEGKVGHKFGEFAFTRKRRPSRTNIGPGRKRGKK
>P27572 | NU4M_WHEAT
MLEHFCECYFDLSGLILCPVLGSIILLFIPNSSIRLIRLIGLCVSLITFLYSLVLWIQFD
PSTAKFQFVESLRWLPYENIHLYMGIDGLSLFFVILTTFLIPICILVGWSGMRSFGKEYI
IAFLICEFLMIAVFCMLDLLLFYVFFESVLIPMFIIIGVWGSRQRKIKAAYQFFLYTLLG
SVFMLLAILLILLQTGTTDLQILLTTEFSERRQILLWIAFFASFAVKVPMVPVHIWLPEA
HVEAPTAGSVILAGILLKLGTYGFLRFSIPMFPEATLCFTPFIYTLSAIAIIYTSLTTLR
QIDLKKIIAYSSVAHMNLVTIGMFSLNIQGIGGSILLMLSHGLVSSALFLCVGVLYDRHK
TRLVRYYGGLVSTMPNFSTIFFFFTLANMSLPGTSSFIGEFLILVGAFQRNSLVATLRAL
GMILGAAYSLWLYNRVVSGNLKPDFLYKFSDLNGREVFIFLPFLVGVVWMGVYPKVFLDC
MHTSVSNLVQHGKFH
>P27754 | RT03_OENBE
MARKGNPISVRLDLNRSSDSSWFSDYYYGKSVYQDVNLRSYFGSIRPPTRLTFGFRLGRC
IILHFPKRTFIHFFLPRRPRRLKRGKKSRPGKEKARWWEFGKVGPIGCLHSNDDTEEERN
EVGGRGAGKRVESIRLDDREKQNEIRIWPKKKQGYGYHDRSPSIKKNLSKSLRVSGAFKH
PKYAGVLNDIAFLIENDDSFRKTKLFKFFFPKKSRSDGPTSHLLKRTPPAVRPSLNYSVM
QYYLNTKNQMHFDPLVVLNHFVAPGVAEPSTMGGANRQGRSNELRIRSRIAFFVESSTSE
KKSLAEDKKGLPHFIRQENDLRFAGRTKTTISLFPFFGATFFFPRDGVGVYKHLFFEDAR
EQLLGQLRIKCWKLMGKDKVMELIEKFIDLGGIGELIKGIEIMIEIILRNRRIPYGYNSY
LNEVKKMRSLLSNRTKTNTIIESVKIKSVYQSASPIAQDISFQPRNKRRSFRSIFSQIVK
DIPLVMKKGVEGIRICCSGRSKGAEIARTECGKYGKTSRNVFNQKIDYAPAEVSTRYGIL
GVKVWISYSK
>P27928 | RT03_MAIZE
MARKGNPISVRLDLNRSSDPSRFSDYYYGKSLYQDVNLRSYFSSIRPPTRLTFGFRLGRC
IILHFPKRTFIHFFLPRRPLRLKRRDKSRPGKDKGRWWAFGKVGPIGCLHSSEGTEEERN
EVRGRGAGKRVESIDREKQNEIRIWPKKMQRYGYHDRTPSRKKNFSKSLRVSGAFKHPKY

AGVVNDIAFLIENDDSFIKTKLFKFFFLPKKSRSDGPTSHLLKRTLPAVRPSLNYSVMQY
FFNTKNKMHFDPVVVLNHFVAPGVAEPSTMGGAKGGSLDKRIRSRIAFFVESSTSDKKCL
ARAKKRLIHFIRQANDLRFAGTTKTTISLFPFFGATFFFSRDGVGVYNNPFYEYAREQLL
GQLRIKCRNLMGKDKVMELIEKFIDLGRIGKLIKGIEMMIEIILRKRRIPYGYNSYLNEV
QKMRSFLSNRTNTNTLIESVKIKSVYQSASLIAQDISFQLRNNPISFRSIFSKIVKDIPL
IMPKGVEGIRICCSGRLGGAEIARTECGKYGKTSCNVFNQKIDYAPAEVSTRDGISGVKV
RISYSQNKKGRAISETYEI
>P28520 | RT12_ORYSJ
MPTKNQLIRHGREEKQRTDRTRASDQCPQKQGVCLRVSTRTPKKPNSALRKIAKVRLSNR
HDIFAHIPGEGHNSQEHSIVLVRGGRVKDSPGVKSHRIRGVKDLLGIPDRRKGRSKYGAE
RPKSK
>P29057 | HMDH1_HEVBR
MDTTGRLHHRKHATPVEDRSPTTPKASDALPLPLYLTNAVFFTLFFSVAYYLLHRWRDKI
RNSTPLHIVTLSEIVAIVSLIASFIYLLGFFGIDFVQSFIARASHDVWDLEDTDPNYLID
EDHRLVTCPPANISTKTTIIAAPTKLPTSEPLIAPLVSEEDEMIVNSVVDGKIPSYSLES
KLGDCKRAAAIRREALQRMTRRSLEGLPVEGFDYESILGQCCEMPVGYVQIPVGIAGPLL
LNGREYSVPMATTEGCLVASTNRGCKAIYLSGGATSVLLKDGMTRAPVVRFASATRAAEL
KFFLEDPDNFDTLAVVFNKSSRFARLQGIKCSIAGKNLYIRFSCSTGDAMGMNMVSKGVQ
NVLEFLQSDFSDMDVIGISGNFCSDKKPAAVNWIEGRGKSVVCEAIIKEEVVKKVLKTNV
ASLVELNMLKNLAGSAVAGALGGFNAHAGNIVSAIFIATGQDPAQNVESSHCITMMEAVN
DGKDLHISVTMPSIEVGTVGGGTQLASQSACLNLLGVKGANKESPGSNSRLLAAIVAGSV
LAGELSLMSAIAAGQLVKSHMKYNRSSKDMSKAAS
>P29185 | CH61_MAIZE
MYRAAASLASKARQAGNSLATRQVGSRLAWSRNYAAKDIKFGVEARALMLRGVEELADAV
KVTMGPKGRNVVIEQSFGAPKVTKDGVTVAKSIEFKDRVKNVGASLVKQVANATNDTAGD
GTTCATVLTKAIFTEGCKSVAAGMNAMDLRRGISMAVDAVVTNLKGMARMISTSEEIAQV
GTISANGEREIGELIAKAMEKVGKEGVITIADGNTLYNELEVVEGMKLDRGYISPYFITN
SKTQKCELEDPLILIHDKKVTNMHAVVKVLEMALKKQKPLLIVAEDVESEALGTLIINKL
RAGIKVCAVKAPGFGENRKANLQDLAILTGGEVITEELGMNLENFEPHMLGTCKKVTVSK
DDTVILDGAGDKKSIEERAEQIRSAIENSTSDYDKEKLQERLAKLSGGVAVLKIGGASEA
EVGEKKDRVTDALNATKAAVEEGIVPGGGVALLYASKELDKLQTANFDQKIGVQIIQNAL
KTPVHTIASNAGVEGAVVVGKLLEQENTDLGYDAAKGEYVDMVKTGIIDPLKVIRTALVD
AASVSSLMTTTESIIVEIPKEEAPAPAMGGGMGGMDY
>P29380 | CYC1_ARATH
MQVADISLQGDAKKGANLFKTRCAQCHTLKAGEGNKIGPELHGLFGRKTGSVAGYSYTDA
NKQKGIEWKDDTLFEYLENPKKYIPGTKMAFGGLKKPKDRNDLITFLEEETK
>P29677 | MPPA_SOLTU
MYRCASSRLSSLKARQGNRVLTRFSSSAAVATKPSGGLFSWITGDTSSSVTPLDFPLNDV
KLSPPLPDYVEPAKTQITTLANGLKVASEASVNPAASIGLYVDCGSIYETPASYGATHLL
ERMAFKSTLNRSHLRIVREIEAIGGNVTASASREHMIYTYDALKTYVPQMVEMLADCVRN
PAFLDWEVKEQLEKVKAEISEYSKNPQHLLLEAVHSAGYAGPYGNSLMATEATINRLNST
VLEEFVAENYTAPRMVLAASGVEHEEFLKVAEPLLSDLPKVATIEEPKPVYVGGDYRCQA
DAEMTHFALAFEVPGGWMSEKESMTLTVLQMLMGGGGSFSAGGPGKGMYSRLYLRVLNQY
PQIHAFSAFSSIYNNTGLFGIQGTTSSDFGPQAVDVAVKELIAVANPSEVDQVQLNRAKQ

ATKSAILMNLESRMVASEDIGRQLLTYGERNPVEHFLKAIDAVSAKDIASVVQKLISSPL
TMASYGDVLSLPSYDAVSSRFRSK
>P29685 | ATPBM_HEVBR
MASRRLLSSLLRSSSRRSVSKSPISNINPKLSSSSPSSKSRASPYGYLLTRAAEYATSAA
AAAPPQPPPAKPEGGKGGGKITDEFTGKGAIGQVCQVIGAVVDVRFDEGLPPILTSLEVL
DHSIRLVLEVAQHMGEGMVRTIAMDGTEGLVRGQRVLNTGSPITVPVGRANPWTYHEVIG
EPIDERGDIKTSHFLPIHREAPAFVDQATEQQILVTGIKVVDLLAPYQRGGKIGLFGGAG
VGKTVLIMELINNVAKAHGGFSVFAGVGERTREGNDLYREMIESGVIKLGDKQADSKCAL
VYGQMNEPPGARARVGLTGLTVAEHFRDAEGQDVLLFIDNIFRFTQANSEVSALLGRIPS
AVGYQPTLATDLGGLQERITTTKKGSITSVQAIYVPADDLTDPAPATTFAHLDATTVLSR
QISELGIYPAVDPLDSTSRMLSPHILGEEHYNTARGVQKVLQNYKNLQDIIAILGMDELS
EDDKLTVARARKIQRFLSQPFHVAEVFTGAPGKYVELKESITSFQGVLDGKYDDLPEQSF
YMVGGIDEVIAKADKIAKESAS
>P31023 | DLDH_PEA
MAMANLARRKGYSLLSSETLRYSFSLRSRAFASGSDENDVVIIGGGPGGYVAAIKAAQLG
FKTTCIEKRGALGGTCLNVGCIPSKALLHSSHMYHEAKHSFANHGVKVSNVEIDLAAMMG
QKDKAVSNLTRGIEGLFKKNKVTYVKGYGKFVSPSEISVDTIEGENTVVKGKHIIIATGS
DVKSLPGVTIDEKKIVSSTGALALSEIPKKLVVIGAGYIGLEMGSVWGRIGSEVTVVEFA
SEIVPTMDAEIRKQFQRSLEKQGMKFKLKTKVVGVDTSGDGVKLTVEPSAGGEQTIIEAD
VVLVSAGRTPFTSGLNLDKIGVETDKLGRILVNERFSTNVSGVYAIGDVIPGPMLAHKAE
EDGVACVEYLAGKVGHVDYDKVPGVVYTNPEVASVGKTEEQVKETGVEYRVGKFPFMANS
RAKAIDNAEGLVKIIAEKETDKILGVHIMAPNAGELIHEAAIALQYDASSEDIARVCHAH
PTMSEAIKEAAMATYDKPIHI
>P31167 | ADT1_ARATH
MVDQVQHPTIAQKAAGQFMRSSVSKDVQVGYQRPSMYQRHATYGNYSNAAFQFPPTSRML
ATTASPVFVQTPGEKGFTNFALDFLMGGVSAAVSKTAAAPIERVKLLIQNQDEMIKAGRL
SEPYKGIGDCFGRTIKDEGFGSLWRGNTANVIRYFPTQALNFAFKDYFKRLFNFKKDRDG
YWKWFAGNLASGGAAGASSLLFVYSLDYARTRLANDAKAAKKGGGGRQFDGLVDVYRKTL
KTDGIAGLYRGFNISCVGIIVYRGLYFGLYDSVKPVLLTGDLQDSFFASFALGWVITNGA
GLASYPIDTVRRRMMMTSGEAVKYKSSLDAFKQILKNEGAKSLFKGAGANILRAVAGAGV
LSGYDKLQLIVFGKKYGSGGA
>P31691 | ADT_ORYSJ
MAEQANQPTVLQKFGGQFHLGSSFSEGVRARNICPSVSSYDRRFTTRSYMTQGLVNGGIN
VPMMSSSPIFANAPAEKGGKNFMIDFLMGGVSAAVSKTAAAPIERVKLLIQNQDEMIKAG
RLSEPYKGIGDCFGRTIKDEGFASLWRGNTANVIRYFPTQALNFAFKDYFKRLFNFKKDK
DGYWKWFGGNLASGGAAGASSLFFVYSLDYARTRLANDAKAAKGGGERQFNGLVDVYRKT
LKSDGIAGLYRGFNISCVGIIVYRGLYFGMYDSLKPVVLTGSLQDNFFASFALGWLITNG
AGLASYPIDTVRRRMMMTSGEAVKYKSSMDAFSQILKNEGAKSLFKGAGANILRAIAGAG
VLSGYDQLQILFFGKKYGSGGA
>P31692 | ADT_CHLKE
MLSSALYQQAGLSGLLRASAMGPQTPFIASPKETQADPMAFVKDLLAGGTAGAISKTAVA
PIERVKLLLQTQDSNPMIKSGQVPRYTGIVNCFVRVSSEQGVASFWRGNLANVVRYFPTQ
AFNFAFKDTIKGLFPKYSPKTDFWRFFVVNLASGGLAGAGSLLIVYPLDFARTRLAADVG
SGKSREFTGLVDCLSKVVKRGGPMALYQGFGVSVQGIIVYRGAYFGLYDTAKGVLFKDER

TANFFAKWAVAQAVTAGAGVLSYPFDTVRRRLMMQSGGERQYNGTIDCWRKVAQQEGMKA
FFKGAWSNVLRGAGGAFVLVLYDEIKKFINPNAVSSASE
>P35017 | SODM_HEVBR
MALRSLVTRKNLPSAFKAATGLGQLRGLQTFSLPDLPYDYGALEPAISGEIMQLHHQKHH
QTYITNYNKALEQLNDAIEKGDSAAVVKLQSAIKFNGGGHVNHSIFWKNLAPVREGGGEL
PHGSLGWAIDADFGSLEKLIQLMNAEGAALQGSGWVWLALDKELKKLVVETTANQDPLVT
KGPTLVPLLGIDVWEHAYYLQYKNVRPDYLKNIWKVMNWKYASEVYAKECPSS
>P37399 | ATPBM_DAUCA
MASRRLLSSFLRSSTRRSLRPSFSNPRPSFLTSYCSSPASILRRYATAAPAKEPAASKPA
GTAGTGKGTITDEKTGAGAIGQVCQIIGAVVDVKFEEGLPPIMTALEVIDFEIRLVLEVA
PSLGENTVRTIAMDGTEGLVRGQKVLNTGAPITIPVGRATLGRIINVIGEPIDHRGEIKT
DQYLPIHREAPTFVDQATEQQILVTGIKVVDLLAPYQKGGKIGLFGGDWVGKTVLIMELI
NNVAKAHAVFAGVGERTREGNDLYKEMMESGVIKLGDQQAESKCALVYGQMNEPPGSRAR
VGLTGLTVAEHFRDAEGEDVLLFVDKRFRFTQANSEVSALLGRIPSAVGYQPTLATDLGG
LQERITTTKKGSITSVQAIYVPADDLTDPAPATTFAHLDATTVLSRQISELGIYPAVDPL
DSTSRMLTPESGEEHYNTARGVQKVLQNYKNLQDIIAILGMDELSEDDKLTVARARKIQR
FLSQPFHVAEIFTGAPGKYVELKECVTSFQGVLDGKYDDLPEQSFYMLGGIEEVIAKAEK
MAKENPQ
>P37841 | UCRI_SOLTU
MLRVAGRRLSSSAARSSSTFFTRSSFTVTDDSSPARSPSPSLTSSFLDQIRGFSSNSVSP
AHQLGLVSDLPATVAAIKNPSSKIVYDDSNHERYPPGDPSKRAFAYFVLTGGRFVYASSV
RLLILKFVLSMSASKDVLALASLEVDLSSIEPGSTVTVKWRGKPVFIRRRTDDDIKLANS
VDLGTLRDPQQDAERVKNPEWLVVVGVCTHLGCIPLPNAGDFGGWFCPCHGSHYDISGRI
RKGPAPYNLEVPTYSFLEENKLLIG
>P37900 | HSP7M_PEA
MAATLLRSLQRRNLSSSSVSAFRSLTGSTKTSYATHKLASLTRPFSSRPAGNDVIGIDLG
TTNSCVSVMEGKNPKVIENSEGARTTPSVVAFNQKSELLVGTPAKRQAVTNPTNTLFGTK
RLIGRRFDDAQTQKEMKMVPYKIVRAPNGDAWVEANGQQYSPSQIGAFVLTKIKETAEAY
LGKTISKAVVTVPAYFNDAQRQATKDAGRIAGLDVQRIINEPTAAALSYGMNNKEGLIAV
FDLGGGTFDVSILEISNGVFEVKATNGDTFLGGEDFDNALLDFLVSEFKRTESIDLAKDK
LALQRLREAAEKAKIELSSTSQTEINLPFISADASGAKHLNITLTRSKFEALVNNLIERT
KAPCKSCLKDANISIKDVDEVLLVGGMTRVPKVQQVVSEIFGKSPSKGVNPDEAVAMGAA
LQGGILRGDVKELLLLDVTPLSLGIETLGGIFTRLISRNTTIPTKKSQVFSTAADNQTQV
GIKVLQGEREMAADNKSLGEFDLVGIPPAPRGLPQIEVTFDIDANGIVTVSAKDKSTGKE
QQITIRSSGGLSDDEIDKMVKEAELHAQRDQERKALIDIRNSADTSIYSIEKSLAEYREK
IPAEVAKEIEDAVSDLRTAMAGENADDIKAKLDAANKAVSKIGQHMSGGSSGGPSEGGSQ
GGEQAPEAEYEEVKK
>P41978 | SODM2_MAIZE
MALRTLASKNALSFALGGAARPSAASARGVTTVALPDLSYDFGALEPVISGEIMRLHHQK
HHATYVVNYNKALEQLDAVVVKGDASAVVQLQGAIKFNGGGHFNHSIFWENLKPISEGGE
PPHGKLGWAIDEDFGSFEALVKRMNAEGAALQGSGWVWLALDKEPKKLSVETTANQDPLV
TKGASLVPLLGIDVWEHAYYLQYKNVRPDYLNNIWKVMNWKYAGEVYENVLA
>P41979 | SODM3_MAIZE
MALRTLASKNALSFALGGAARPSAESARGVTTVALPDLSYDFGALEPVISGEIMRLHHQK

NHATYVVNYNKALEQIDDVVVKGDDSAVVQLQGAIKFNGGGHVNHSIFWKNLKPISEGGG
EPPHGKLGWAIDEDFGSFEALVKRMNAEGAALQGSGWVWLALDKEAKKVSVETTANQDPL
VTKGASLVPLLGIDVWEHAYYLQYKNVRPDYLNNIWKVMNWKYAGEVYENVLA
>P41980 | SODM4_MAIZE
MALRTLASKNALSFALGGAARPSAASARGVTTVALPDLSYDFGALEPAISGEIMRLHHQK
HHATYVGNYNKALEQLDAAVAKGDASAVVQLQGAIKFNGGGHVNHSIFWKNLKPISEGGG
EPPHGKLGWAIDEDFGSFEALVKRMNAEGAALQGSGWVWLALDKEPKKLSVETTANQDPL
VTKGASLVPLLGIDVWEHAYYLQYKNVRPDYLNNIWKVMNWKYAGEVYENVLA
>P42027 | NDUS7_BRAOL
MAMITRNTATRLPLVLQSHRAAAVSHLHTSLPALSPATTPTSYTRPGPPSTSAPPPGLSK
TAEFVISKVDDLMNWARRGSIWPMTFGLACCAVEMMHTGAARYDLDRFGIIFRPSPRQSD
CMIVAGTLTNKMAPALRKVYDQMPEPRWVISMGSCANGGGYYHYSYSVVRGCDRIVPVDI
YVPGCPPTAEALLYGLLQLQKKINRRKDFLHWWNK
>P42056 | VDAC2_SOLTU
MVKGPGLYSDIGKKARDLLYRDYVSDHKFTVTTYSTTGVAITASGLKKGELFLADVSTQL
KNKNITTDVKVDTNSNVYTTITVDEPAPGLKTIFSFVVPDQKSGKVELQYLHEYAGINTS
IGLTASPLVNFSGVAGNNTVALGTDLSFDTATGNFTKCNAGLSFSSSDLIASLALNDKGD
TVSASYYHTVKPVTNTAVGAELTHSFSSNENTLTIGTQHLLDPLTTVKARVNSYGKASAL
IQHEWRPKSLFTISGEVDTRAIEKSAKIGLAVALKP
>P42793 | RM05_ARATH
MFPLNFHYEDVLRQDLLLKLNYANVMEVPGLCEIRVVPKAPYNFIIKNGKLAMEIPCGQK
FIQTQRGSTGKSFRSNPFLGSNKDKGYVSDLARQSTLRGHGMSNFLVRILTVMSLLDFPV
EIRKNSIQFSMETEFCEFSPELEDHFEIFEHIRGFNVTIITSANTQDETLLLWSGFLQKD
EGETQ
>P46274 | VDAC1_WHEAT
MGGPGLYSGIGKKAKDLLYRDYQTDHKFTLTTYTANGPAITATSTKKADLTVGEIQSQIK
NKNITVDVKANSASNVITTITADDLAAPGLKTILSFAVPDQKSGKVELQYLHDYAGINAS
IGLTANPVVNLSGAFGTSALAVGADVSLDTATKNFAKYNAALSYTNQDLIASLNLNNKGD
SLTASYYHIVEKSGTAVGAELTHSFSSNENSLTFGTQHTLDPLTLVKARINNSGKASALI
QHEFMPKSLCTISAEVDTKAIEKSSKVGIAIALKP
>P46485 | GCSH_FLATR
MALRIWASSTAKALRLSSASRPHFSPLFRCFSSAAVLDGLKYANSHEWVKHEGSVATIGI
TDHAQDHLGEVVFVELPEVGGSVTKATGFGAVESVKATSDVNSPISGEIVEVNSKLTKTP
GLINKSPYEDGWMIKVKPSNPSELDSLMGPKEYTKFCEEEGAAAH
>P46487 | MDHM_EUCGU
MRASMLRLIRSRSSSAAPRPHLLRRAYGSESVPERKVAVLGAAGGIGQPLALLMKLNPLV
SQLALYDIAGTPGVAADVGHINTRSEVAGYVGEEQLGQALEGSDVVIIPAGVPRKPGMTR
DDLFNINAGIVKSLCTAIAKYCPNAVVNMISNPVNSTVPIAAEIFKKAGTYNEKKLLGVT
TLDVVRAKTFYAGKAKVPVEEVNVPVVGGHAGITILPLFSQAVPKANLADEDIKALTKRT
QDGGTEVVEAKAGKGSATLSMAYAGALFADACLKGLNGVPDVVECSFVQSSIITELPFFA
SKVKLGKNGVEEVLELGPMSDYEKQGLEILIPELKASIEKGIKFANQ
>P46740 | RT03_PROWI
MGQKTNPISLRLQNVNRNFDSCWYSDYFYAKCFSRDLYLNNYINTFFKLYRLPQARVCVN
FGIQNIKVYPFFCIPKASRVSLAKNLGLFQHLSKAWNSSPKYFVSSKSKQLSQLKINDAS

LPVNNLNNNLFFNSSKHNEIRNKQNFFHNLELNKLNLSFKNSAYSDVKNYSFLENISAKL
LLNFKEKSVINEVYYTTTNLGQIGNNISTNSIYSKQDMSDNSSKSLLKMLLREYYKSSDN
KSSILYENKSPKSLENIFGKINEVSNINKKNLYLLNSETDNIPNLYKRKSKPTKFLRNEH
NKVIEDKKTSLYFMPDSNQIKNDIYQGKISKISSISNNSKDSLTDYNLHLYQAYLHNILG
NSKSLVSRNQFKYKNYIENFLSSQYNIDSQLFPFISKQNWQSAGFIADEIVYFIERRVSF
SRIKNRILRQASMQSYVRGIRITCSGRVGGKSKKAQRATQECVKYGETSLHVFECKIDFA
SRIANTSFGLVGIKVWICFK
>P46742 | RT10_PROWI
MQQVQLKLKSFDPVYINQLISLLNDVLDTLEIQNSKEIFLPSKIKKITVIRSPHIHKKSR
DQFQIKRYKRSMIISFTNIDILHAFLEICKDLHVVGVQIHISVKYHS
>P46744 | RT12_PROWI
MPTINQLLRKKSSRQAPKLKSKKPALAGCPQKRGVCFRVYTRTPKKPNSALRKVAKIRLC
NGIVVIASIPGEGHNLQEHSVVCIRGGRVKDLPGVKYKVVRGRLDLQGVVNRKQSRSLYG
TPKSK
>P46745 | RT07_PROWI
MNAPITQKNTAYISSDKLSLLELENSNKFINLLMVDGKKSRAIRLFYDTLVLLKRKNLKE
NSKKESLLGIIGQLSPKNSIEDLVNNSNKLSNNSNEEIKIPLIDNSKSDDKSNLLETISV
LEVLSIALKNVTPSVELRKVRRAGNTFLIPAILSQHKANTLAIRWVIESAKKKQQNSKQN
FAECLADEIYQAYLKQGKARQKRDELHSAAISNRANIRYRWW
>P46747 | RT13_PROWI
MVYIQNTHLNDKKQIYQACAQIYGLGHHHCLQICDVLGVSPETRLGMLSTGQHTLLAQII
TQNYDTGSDVRRFTRQNIQRLVNIHSYRGYRHIQGLPVRGQRSHGNARTVRKLKNVIKI
>P46750 | RT19_PROWI
MARSLSKPPFCEVKLATNNSVTKIWSRRSAILPQFVGKTVSIHNGRIFIPCKISPEMIGH
KFGEFAVTRKKPIHKKKK
>P46751 | RM16_PROWI
MLQPNNTKFRKFQKSRVKGVQSNTDQLRYGKFGIKTVSAARIPARTIEAVRRVITRKFKR
LGVIWIRVFPDIAVSGKPAEVRMGKGKGAPQYWVCKVKRGAILFEFDGISPQLAKQAARL
ADSKLPIKTRFVMYS
>P46752 | RT14_PROWI
MFNSIKRDLKRRKLYKKYESKRLLYKALISDCNLNQDLRFILTQKLNKLPRNSSQVRVKN
RCILTGRGHSVYKFCRISRIKFRDLANQGLIQGCVKSSW
>P46773 | RT03_ORYSJ
MARKGNPISVRLDLNRSSDPSRFSDYYYGKSLYQDVNLRSYFSSIRPPTILTFGFRLGRC
IILHFPKRTFIHFFLPRRPLRLKRRDKSRPGKDKGRWWAFGKVGPIGCLHSSEGTEEERN
EVRGRGAGKRVESIDREKQNEIRIWPKKMQRYGYHDRSPSRKKNFSKSLRVSGAFKHPKY
AGVVNDIAFLIENDGPTSHLLKRTLPAVRPSLNYSVMQYFFNTKNKMHFDPVVVLNHFVA
PGVAEPSTMGGAKGGSLDKRIRSRIAFFVESSTSEKKCLARAKKRLIHFIRQANDLRFAG
TTKTTISLFPFFGATFFFPRDGVGVYNNPFFEYAREQLLGQLRIKCRNLMGKDKVMELIE
KFIYLGRIGKLIKGIEMMIEIILRKRIIPYGYNSYLNEVQKMRSFLSNRTNTNTLIESVK
IKSVYQSASLIAQDISFQLGNNPISFRSIFSQIVKDIPLIMPKGVEGIRICCSGRLGGAE
IARTECGKYGKTSCNVFNQKIDYALAEVSTRNGISGVKVRISYSQNKKGRAISETYEI
>P46801 | RM16_ORYSJ
MEKHLVMYLTRKSIMLLRKYPLVTEFQVSKCGSHIVKIRRDVLYPKRTKYSKYSKCRCSR

GCEPDGTQLGFGRYGTKSCRAGRLSYRAIEAARRATIGQFHRAMSGQFRRNCKIWVRVLA
DLPITGKPAEVRMGRGKGNPTGWIARVSTGQIPFEMDGVSLSNARQAARLAAHKPCSSTK
FVQWS
>P48857 | RT12_BRANA
MPTFNQLIRHGREEKRRTDRTRALDKCPQKLGACLRVSTRTPKKPNSALRKIAKVRLSNR
HDIFAYIPGEGHNLQEHSQVLIRGGRVKDLPGVKFHCIRGVKDLMGIPGRRRGRSKYGAE
KPKSI
>P49357 | GLYM_FLAPR
MAMALALRRLSSSADKPLQRLFNGGHLYSMSSLPSEAVYEKERPGVTWPKQLNAPLEVVD
PEIADIIELEKARQWKGLELIPSENFTSLSVMQAVGSVMTNKYSEGYPGARYYGGNEYID
MAETLCQKRALEAFRLDPAKWGVNVQPLSGSPANFHVYTALLKAHDRIMALDLPHGGHLS
HGYQTDTKKISAVSIFFETMPYRLNESTGYIDYDQLEKSATLFRPKLIVAGASAYARLYD
YARIRKVCDKQKAIMLADMAHISGLVAAGVIPSPFDYADVVTTTTHKSLRGPRGAMIFFR
KGLKEVNKQGKEVFYDYEDKINQAVFPGLQGGPHNHTITGLAVALKQATTAEYKAYQEQV
MSNSAKFAETLVKSGYELVSGGTENHLVLVNLKNKGIDGSKVEKVLEAVHIAANKNTVPG
DVSAMVPGGIRMGTPALTSRGFVEEDFAKVAYFFDLAVKLAVKIKGEAKGTKLKDFVTAM
ESSAIQSEISKLRHDVEEYAKQFPTIGFEKETMKYKN
>P49358 | GLYN_FLAPR
MAMASALRRLSSSSNKPLQRLFNGGHLYSMSSLPSEAVYEKERPGVTWPKQLNAPLEVGD
PEIADIIELEKARQWKGLELILSENFTSLSVMQAVGSVMTNKYSEGYPGARYYGGNEYID
MAETLCQKRALEAFRLDAAKWGVNVQPLSGSPANFHVYTALLKAHDRIMALDLPHGGHLS
HGYQTDTKKISAVSIFFETMPYRLNESTGYIDYDQLEKSATLFRPKLIVAGASAYARLYD
YARIRKVCDKQKAILLADMAHISGLVAAGVIPSPFDYADVVTTTTHKSLRGPRGAMIFFR
KGVKEVNKQGKEVLYDYEDKINQAVFPGLQGGPHNHTITGLAVALKQATTAEYKAYQEQV
MSNCAKFAETLVKSGYELVSGGTENHLVLVNLKNKGIDGSRVEKVLEAVHIAANKNTVPG
DVSAMVPGGIRMGTPALTSRGFVEEDFAKVAYLFDLAVKLAVKIKGEAQGTKLKDFVAAM
QSSAFQSEISKLRHDVEEYAKQFPTIGFEKETMKYKN
>P49359 | GCSH_FLAPR
MALRIWASSTANALRLSSATRPHFSPLSRCFSSVLDGLKYANSHEWVKHEGSVATIGITD
HAQDHLGEVVFVDLPEAGGSVTKATGFGAVESVKATSDVNSPISGEIVEVNSKLSETPGL
INSSPYEDGWMIKVKPSNPSELDSLMGAKEYTKFCEEEDSAH
>P49361 | GCSPA_FLAPR
MERARRLANKAILGRLVSQTKHNPSISSPALCSPSRYVSSLSPYVCSGTNVRSDRNLNGF
GSQVRTISVEALKPSDTFPRRHNSATPEEQTKMAEFVGFPNLDSLIDATVPKSIRLDSMK
YSKFDEGLTESQMIAHMQDLASKNKIFKSFIGMGYYNTSVPTVILRNIMENPGWYTQYTP
YQAEIAQGRLESLLNFQTMVTDLTGLPMSNASLLDEGTAAAEAMAMCNNIQKGKKKTFII
ASNCHPQTIDICKTRADGFDLKVVTSDLKDFDYSSGDVCGVLVQYPGTEGELLDYSEFIK
NAHANGVKVVMASDLLALTILKPPGELGADIVVGSAQRFGVPMGYGGPHAAFLATSQEYK
RMMPGRIIGVSVDSSGKPALRMAMQTREQHIRRDKATSNICTAQALLANMAAMFGVYHGP
EGLKTIAKRVHGLAGTFAAGLKKLGTVQVQDLPFFDTVKVTCVDSKAIAEEAYKHKMNLR
IVDKNTITVAFDETTTIEDVDTLFKVFALGKPVTFTAASIAPEVQDAIPSGLVRETPYLT
HPIFNMYHTEHELLRYISKLQSKDLSLCHSMIPLGSCTMKLNATTEMMPVTWPAFADIHP
FAPTEQAQGYQEMFKNLGDLLCTITGFDSFSLQPNAGAAGEYAGLMVIRAYHMARGDHHR
NVCIIPVSAHGTNPASAAMCGMKIITVGTDSKGNINIEELRKAAEANKENLSALMVTYPS

THGVYEEGIDEICKIIHDNGGQVYMDGANMNAQVGLTSPGWIGADVCHLNLHKTFCIPHG
GGGPGMGPIGVKKHLAPYLPSHPVVATGGIPAPEQSQPLGTIAAAPWGSALILPISYTYI
AMMGSQGITNASKIAILNANYMAKRLENHYPILFRGVNGTVAHEFIVDLRPLKTTAGIEP
EDVAKRLIDYGFHGPTMSWPVPGTLMIEPTESESKAELDRFCDALISIRQEIAEIEKGNV
DLNNNVIKGAPHPPQLLMADKWTKPYSREYAAYPAPWLRAAKFWPTTCRVDNVYGDRNLI
CTLQPPQEYEEKAEATA
>P49362 | GCSPB_FLAPR
MERARRLAILGRLVSQTKHNPSISSPALCSPSRYVSSLSPYVCSGTNVRSDRNLNGFGSQ
VRTISVEALKPSDTFPRRHNSATPEEQTKMAEFVGFPNLDSLIDATVPKSIRLDSMKYSK
FDEGLTESQMIAHMQDLASKNKIFKSFIGMGYYNTSVPTVILRNIMENPGWYTQYTPYQA
EIAQGRLESLLNFQTMITDLTGLPMSNASLLDEGTAAAEAMAMCNNIQKGKKKTFIIASN
CHPQTIDICKTRADGFDLKVVTSDLKDFDYSSGDVCGVLVQYPGTEGELLDYSEFIKNAH
ANGVKVVMASDLLALTILKPPGELGADIVVGSAQRFGVPMGYGGPHAAFLATSQEYKRMM
PGRIIGVSVDSSGKPALRMAMQTREQHIRRDKATSNICTAQALLANMAAMFGVYHGPEGL
KTIAKRVHGLAGTFASGLKKLGTVQVQDLPFFDTVKVTCADSKAIAEEAYKHKMNLRIVD
KNTITVAFDETTTIEDVDTLFKVFALGKPVTFTAASIAPEVQDAIPSGLVRETPYLTHPI
FNMYHTEHELLRYISKLQSKDLSLCHSMIPLGSCTMKLNATTEMMPVTWPAFADIHPFAP
TEQAQGYQEMFKNLGDLLCTITGFDSFSLQPNAGAAGEYAGLMVIRAYHMARGDHHRNVC
IIPVSAHGTNPASAAMCGMKIITVGTDSKGNINIEELRKAAEANKENLSALMVTYPSTHG
VYEEGIDEICKIIHDNGGQVYMDGANMNAQVGLTSPGWIGADVCHLNLHKTFCIPHGGGG
PGMGPIGVKKHLAPYLPSHPVVPTGGIPAPEQSQPLGTIAAAPWGSALILPISYTYIAMM
GSQGITNASKIAILNANYMAKRLENHYPILFRGVNGTVAHEFIVDLRPLKTTAGIEPEDV
AKRLIDYGFHGPTMSWPVPGTLMIEPTESESKAELDRFCDALISIRQEIAEIEKGNVDFN
NNVIKGAPHPPQLLMADKWTKPYSREYAAYPAPWLRAAKFWPTTCRVDNVYGDRNLICTL
QPPQEYEEKAEATA
>P49363 | GCST_FLAPR
MRGGLWQLGQSITRRLGQSDKKTIARRCYASEADLKKTVLYDFHVANGGKMVPFAGWSMP
IQYKDSIMESTINCRENGSLFDVSHMCGLSLKGKDCVPFLEKLVVADVAGLRPGTGSLTV
FTNEKGGAIDDSVITKVTDDHIYLVVNAGCRDKDLAHIEEHMKAFKAKGGDVSWHIYDER
SLLALQGPLAGSTLQHLTKEDLSKMYFGDFRIIDINGSKCFLTRTGYTGEDGFEISVPSE
NAVDLAKAILEKSEGKVRLTGLGARDSLRLEAGLCLYGNDMEQHITPVEAGLTWAIGKRR
RAEGGFLGADVILKQIADGPAIRRVGLFSTGPPARSHSEIQNEKGENIGEVTSGGFSPCL
KKNIGMGYVKSGLHKPGTKLKIVIRGKTYEGSVTKMPFVPTKYYKPA
>P49386 | RT03_BRANA
MARKGNPISVRLGKNRSSDSSRFSEYYYGKFVYQDVNLRSYFGSIRPPTRLTFGFRLGRC
ILLHFPKRTFIHFFLPRRPRRLKRREKTRPGKEKGRWWTTPGKAGPIGCLRDDTEEERNE
VRGRGARKRVESIRLDDRKKQNEIRGWPKKKQRYGYHDRTPSIKKNLSKSLRISGAFKHP
KYGGVVNDIAFLIENDDSFRKTKLFKFFFPKKSRSDGPTSYLRTLPAVGPSLNFLVMQYF
FNTKNQMNFDPVVVLNHFVAPGAAEPSTMGRANGTGDRSLQKRIRSRIAFFVESSTSEKK
CLAEAKNRLTHLIRLANDLGFAGTTKTTISLFPFFGATFFFLRDGVGVTNNLDAREQLLN
QLRVKCWNLLGKDKVMELIEKFKDLGGIEELIKVIDMMIEIILRKRGIPYGYNSYFNEVQ
KMRSFLSNRTNTKTLIESVKIKSVYQSASLIAQDISFQLKNKRRSTHSIFAKIVKEIPKR
VEGIRICFSGRLKDAAEKAQTKCYKHRKTSRNVFNQKIDYAPAEVSTRYGISGVKVWISY
SQKKGGRAISETYEI

>P49387 | RT14_BRANA
MSEKQNSRDHKRRLLAAKFELRRKLYKAFCKDPDLPSDMRDKHRYKLSKLPRNSSFARVR
NRCISTGRPRSVSEFFRIYRIVFRGLASRGSLMGIKKSSW
>P49388 | RM05_BRANA
MFPLNFHYEDVSRQDPLLNRITPTLWKFLGSCEIRVVPNGPYNFIIKNGKLAMEIPRGQK
FIQTQRGSTGKSFRSNPFLGSNKDKGYVSDLARQSTLRGHGMSNFSVRISTVMSLLDFPV
EIRKNSIQFSMETEFCEFAPQLQDHFEIFEHIRGFNVTIVTSANTQDETLPLWSGFLQKD
EGETQ
>P49389 | RM16_BRANA
MYLTRKSIMLLRKYLLVTESQVSKCGFHIVKKKGDVLYPKRTKYSKGRCSRGCKPDGTKL
GFGRYGTKSCRAGRLSYRAIEAARRATIGHSFRRAMSGQFRRNCKIWVRVLADLPITGKP
AEVRMGRGKGNPTGWIARVSTGQIPFEMDGVSLANARQAARLAAHKPCSSTKFVQWS
>P50433 | GLYM_SOLTU
MAMAIALRRLSATVDKPVKSLYNGGSLYYMSSLPNEAVYDKEKSGVAWPKQLNAPLEVVD
PEIADIIEHEKARQWKGLELIPSENFTSVSVMQAVGSVMTNKYSEGYPGARYYGGNEYID
MAETLCQKRALEAFRLDPAKWGVNVQPLSGSPANFQVYTALLKPHERIMALDLPHGGHLS
HGYQTDTKKISAVSIFFETMPYRLDESTGYIDYDQLEKSATLFRPKLIVAGASAYARLYD
YDRIRKVCNKQKAILLADMAHISGLVAAGVIPSPFDYADVVTTTTHKSLRGPRGAMIFYR
KGVKEVNKQGKEVFYDYEDKINQAVFPGLQGGPHNHTITGLAVALKQATTPEYRAYQEQV
LSNSSKFAQALGEKGYELVSGGTDNHLVLVNMKNKGIDGSRVEKVLEAVHIAANKNTVPG
DVSAMVPGGIRMGTPALTSRGFLEEDFVKVADFFDAAVKIAVKVKAETQGTKLKDFVATL
ESSAPIKSEIAKLRHDVEEYAKQFPTIGFEKETMKYKN
>P50892 | RT12_RAPSA
MPTLNQLIRHGREEKRRTDRTRALDKCPQKLGACPRVSTRTPKKPNSAPRKIAKVRLSNR
HDIFAHIPGEGHNSQEHSQVLIRGGRVKDSPGVKSHCIRGVKDLMGIPGRRSGRSKYGAE
KPKSI
>P50893 | RT19_PLASU
MSRAIWKGPFIDPFFFRKNGSSNSNNKIYSRRSVVSPKFIGREVEIYNGHKWITIKIKED
MIGHKFGEFAFTRKATIHKKKTK
>P51132 | UCRI2_TOBAC
MLRIAGRRASSLSRWPVRSVAPSSSAFISANHFSSDDDSSSPRSISPSLASVFLHHTRGF
SSNSVSHAHDMGLVPDLPPTVAAIKNPTSKIVYDEHNHERYPPGDPSKRAFAYFVLTGGR
FVYASLVRLLILKFVLSMSASKDVLALASLEVDLSSIEPGTTVTVKWRGKPVFIRRRTED
DINLANSVDLGSLRDPQQDAERVKSPEWLVVIGVCTHLGCIPLPNAGDFGGWFCPCHGSH
YDISGRIRKGPAPYNLEVPTYSFLEENKLLIG
>P51135 | UCRI5_TOBAC
MLRIAGRKLSSSAAARSSSAFFTRNPFTFTDDSSSPTRSPSPTSLASQFLDQFRGFSSNS
VSPAHQTGLVSDLPATVAAIKNPSSKIVYDDSNHERYPPGDPSKRAFAYFVLTGGRFVYA
SLVRLLILKFVLSMSASKDVLALASLEVDLSSIEPGTTVTVKWRGKPVFIRRRTDEDINL
ANSVDLGSLRDPQQDAERVKNPEWLVVIGVCTHLGCIPLPNAGDFGGWFCPCHGSHYDIS
GRIRKGPAPYNLEVPTYSFMEENKLLIG
>P51409 | RM05_SOLTU
MDQLMFPLYFHYEDVLRQDLLLKLNYANVMEVPGLCKIIVVPKTAPSIKNGKLAMEISCG
QKLKQRASTGKSFRSNPFLGSNKDKKGYVSDLARQSTLRGHGMSHFLVRISTVMSLLDSP

LEIRERSIQFSMETEFCEFSPELEDHFEIFEHIRGFNVTIVTSANTQDETLLLWSGFLQK
DEGETQ
>P52901 | ODPA1_ARATH
MALSRLSSRSNIITRPFSAAFSRLISTDTTPITIETSLPFTAHLCDPPSRSVESSSQELL
DFFRTMALMRRMEIAADSLYKAKLIRGFCHLYDGQEAVAIGMEAAITKKDAIITAYRDHC
IFLGRGGSLHEVFSELMGRQAGCSKGKGGSMHFYKKESSFYGGHGIVGAQVPLGCGIAFA
QKYNKEEAVTFALYGDGAANQGQLFEALNISALWDLPAILVCENNHYGMGTAEWRAAKSP
SYYKRGDYVPGLKVDGMDAFAVKQACKFAKQHALEKGPIILEMDTYRYHGHSMSDPGSTY
RTRDEISGVRQERDPIERIKKLVLSHDLATEKELKDMEKEIRKEVDDAIAKAKDCPMPEP
SELFTNVYVKGFGTESFGPDRKEVKASLP
>P52903 | ODPA_SOLTU
MALSTSRAINHIMKPLSAAVCATRRLSSDSTATITVETSLPFTSHNIDPPSRSVETSPKE
LMTFFKDMTEMRRMEIAADSLYKAKLIRGFCHLYDGQEAVAVGMEAAITKKDCIITAYRD
HCIFLGRGGTLVEAFAELMGRRDGCSRGKGGSMHFYKKESGFYGGHGIVGAQVPLGIGLA
FAQKYKKEDYVTFAMYGDGAANQGQLFEALNMAALWDLPAILVCENNHYGMGTAEWRAAK
SPAYYKRGDYVPGLRVDGMDVFAVKQACTFAKQHALKNGPIILEMDTYRYHGHSMSDPGS
TYRTRDEISGVRQERDPVERIRSLILAHNIATEAELKDIEKENRKVVDEAIAKAKESPMP
DPSELFTNVYVKGFGVEAYGADRKELRATLP
>P52904 | ODPB_PEA
MLGVIRNKTIRPSFSAFRFFSSAKQMTVRDALNSALDVEMSADSKVFLMGEEVGEYQGAY
KVTKGLLEKYGPERVLDTPITEAGFTGIGVGAAYYGLKPVVEFMTFNFSMQAIDHIINSA
AKSNYMSAGQISVPIVFRGLNGDAAGVGAQHSHCYASWYGSCPGLKVLVPHSAEDARGLL
KAAIRDPDPVVFLENELLYGESFPVSAEVLDSSFWLPIGKAKIEREGKDVTITAFSKMVG
FALKAAEILEKEGISAEVINLRSIRPLDRPTINASVRKTNRLVTVEEGFPQHGVGAEICT
SVIEESFGYLDATVERIGGADVPMPYAGNLERLVVPHVEDIVRAAKRACHRSVPLAAAA
>P54260 | GCST_SOLTU
MRGGLWQLGQSITRRLAQADKKTIGRRCFASDADLKKTVLYDFHVVNGGKMVPFAGWSMP
IQYKDSIMDSTVNCRENGSLFDVSHMCGLSLKGKDTIPFLEKLVIADVAGLAPGTGSLTV
FTNEKGGAIDDSVVTKVTNDHIYLVVNAGCRDKDLAHIEEHMKSFKSKGGDVSWHIHDER
SLLALQGPLAAPVLQYLTKDDLSKMYFGEFRVLDINGAPCFLTRTGYTGEDGFEISVPSE
NALDLAKALLEKSEGKIRLTGLGARDSLRLEAGLCLYGNDMEQHTTPVEAGLTWAIGKRR
RAEGGFLGAEVILKQIEEGPKIRRVGFFSSGPPPRSHSEIQDSNGQNIGEITSGGFSPCL
KKNIAMGYVKTGNHKAGTNVKIVIRGKSYDGVVTKMPFVPTKYYKP
>P60096 | RT12_MAGGA
MPTLNQLIRHGREEKRRTDRTRASDQCPQKQGVRPRVPTRTPKKPNSAPRKIAKVRLSNR
HDIFAHIPGEGHNSQEHPMVLIRGGRVKDSPGVKSHRIRGVKDLLGIPNRRRGRSKYGAE
RPKSI
>P60097 | RT12_MAGSO
MPTLNQLIRHGREEKRRTDRTRASDQCPQKQGVRPRVPTRTPKKPNSAPRKIAKVRLSNR
HDIFAHIPGEGHNSQEHPMVLIRGGRVKDSPGVKSHRIRGVKDLLGIPNRRRGRSKYGAE
RPKSI
>P60098 | RT12_WHEAT
MPTKNQLIRHGREEKRRTDRTRALDQCPQKQGVCLRVSTRTPKKPNSALRKIAKVRLSNR
HDIFAYIPGEGHNLQEHSIVLVRGGRVKDLPGVKFHCIRGVKDLLGIPDRRKGRSKYGAE

RPKSK
>P60099 | RT12_MAIZE
MPTKNQLIRHGREEKRRTDRTRALDQCPQKQGVCLRVSTRTPKKPNSALRKIAKVRLSNR
HDIFAYIPGEGHNLQEHSIVLVRGGRVKDLPGVKFHCIRGVKDLLGIPDRRKGRSKYGAE
RPKSK
>P60159 | NU3M_HELAN
MLEFAPICIYLVISLLVSLILLGVPFLFASNSSTYPEKLSAYECGFDPFGDARSRFDIRF
YLVSILFIIFDLEVTFFFPWAVSLNKIDLFGFWSMMAFLLILTIGFLYEWKRGALDWE
>P60160 | NU3M_WHEAT
MLEFAPICIYLVISLLVSLILLGVPFLFASNSSTYPEKLSAYECGFDPFGDARSRFDIRF
YLVSILFIIFDLEVTFFFPWAVSLNKIDLFGFWSMMAFLLILTIGFLYEWKRGALDWE
>P60621 | COX1_RAPSA
MKNLVRWLFSTNHKDIGTLYFIFGAIAGVMGTCFSVLIRMELARPGDQILGGNHQLYNVL
ITAHAFLMIFFMVMPAMIGGFGNWFVPILIGAPDMAFPRLNNISFWLLPPSLLLLLSSAL
VEVGSGTGWTVYPPLSGITSHSGGAVDLAIFSLHLSGVSSILGSINFITTIFNMRGPGMT
MHRLPLFVWSVLVTAFLLLLSLPVLAGAITMLLTDRNFNTTFFDPAGGGDPILYQHLFWF
FGHPEVYILILPGFGIISHIVSTFSGKPVFGYLGMVYAMISIGVLGFLVWAHHMFTVGLD
VDTRAYFTAATMIIAVPTGIKIFSWIATMWGGSIQYKTPMLFAVGFIFLFTIGGLTGIVL
ANSGLDIALHDTYYVVAHFHYVLSMGAVFALFAGFYYWVGKIFGRTYPETLGQIHFWITF
FGVNLTFFPMHFLGLSGMPRRIPDYPDAYAGWNALSSFGSYISVVGICCFFVVVTITLSS
GNNKRCAPSPWALELNSTTLEWMVQSPPAFHTFGELPAIKETKSYVK
>P62772 | CYC_BRANA
ASFDEAPPGNSKAGEKIFKTKCAQCHTVDKGAGHKQGPNLNGLFGRQSGTTAGYSYSAAN
KNKAVEWEEKTLYDYLLNPKKYIPGTKMVFPGLKKPQDRADLIAYLKEATA
>P62773 | CYC_BRAOL
ASFDEAPPGNSKAGEKIFKTKCAQCHTVDKGAGHKQGPNLNGLFGRQSGTTAGYSYSAAN
KNKAVEWEEKTLYDYLLNPKKYIPGTKMVFPGLKKPQDRADLIAYLKEATA
>P68526 | ATP6_TRITI
MRFLSTDMKDRNMLFAAITTNQPIRSKCSRLPDLHDFFPTNISQNFAITPNLDITPTPER
IAGVTIVLQIEEYLGQNESEQGAVNLARTVLGARHRNGETWQGILEDIRAGGGMDNFIQN
LPGAYPETPLDQFAIIPIIDLHVGNFYLSFTNEVLYMLLTVVLVVFLFFVVTKKGGGKSV
PNAWQSLVELIYDFVLNLVNEQIGGLSGNVKQKFFPRISVTFTFSLFRNPQGMIPFSFTV
TSHFLITLALSFSIFIGITIVGFQRHGLHFFSFLLPAGVPLPLAPFLVLLELISYCFRAL
SLGIRLFANMMAGHSLVKILSGFAWTMLFLNNIFYFIGDLGPLFIVLALTGLELGVAISQ
AHVSTISICIYLNDATNLHQNESFHN
>P68527 | ATP6_WHEAT
MRFLSTDMKDRNMLFAAITTNQPIRSKCSRLPDLHDFFPTNISQNFAITPNLDITPTPER
IAGVTIVLQIEEYLGQNESEQGAVNLARTVLGARHRNGETWQGILEDIRAGGGMDNFIQN
LPGAYPETPLDQFAIIPIIDLHVGNFYLSFTNEVLYMLLTVVLVVFLFFVVTKKGGGKSV
PNAWQSLVELIYDFVLNLVNEQIGGLSGNVKQKFFPRISVTFTFSLFRNPQGMIPFSFTV
TSHFLITLALSFSIFIGITIVGFQRHGLHFFSFLLPAGVPLPLAPFLVLLELISYCFRAL
SLGIRLFANMMAGHSLVKILSGFAWTMLFLNNIFYFIGDLGPLFIVLALTGLELGVAISQ
AHVSTISICIYLNDATNLHQNESFHN
>P68535 | RT12_PETHY

MPSLNQLIRHGREEKRRTDRTRALDQCPQKQGVCPRVSTRTPKKPNSAPRKIAKVRLSNR
HDIFAHIPGEGHNLQEHSMVLIRGGRVKDSPGVKSHCIRGVKDLLGIPDRRRGRSKYGAE
KPKSI
>P68536 | RT12_PETPA
MPSLNQLIRHGREEKRRTDRTRALDQCPQKQGVCPRVSTRTPKKPNSAPRKIAKVRLSNR
HDIFAHIPGEGHNLQEHSMVLIRGGRVKDSPGVKSHCIRGVKDLLGIPDRRRGRSKYGAE
KPKSI
>P68539 | COX1_WHEAT
MTNMVRWLFSTNHKDIGTLYFIFGAIAGVMGTCFSVLIRMELARPGDQILGGNHQLYNVL
ITAHAFLMIFFMVMPAMIGGFGNWFVPILIGAPDMAFPRLNNISFWLLPPSLLLLLSSAL
VEVGSGTGWTVYPPLSGITSHSGGAVDLAIFSLHLSGISSILGSINFITTIFNMRGPGMT
MHRLPLFVWSVLVTAFLLLLSLPVLAGAITMLLTDRNFNTTFFDPAGGGDPILYQHLFWF
FGHPEVYILILPGFGIISHIVSTFSRKPVFGYLGMVYAMISIGVLGFLVWAHHMFTVGLD
VDTRAYFTAATMIIAVPTGIKIFSWIATMWGGSIQYKTPMLFAVGFIFLFTIGGLTGIVL
ANSGLDIALHDTYYVVAHFHYVLSMGAVFALFAGFYYWVGKIFGRTYPETLGQIHFWITF
FGVNLTFFPMHFLGLSGMPRRIPDYPDAYAGWNALSSFGSYISVVGIRRFFVVVAITSSS
GKNQKCAESPWAVEQNPTTLEWLVQSPPAFHTFGELPAVKETKS
>P68540 | COX1_AEGCO
MTNMVRWLFSTNHKDIGTLYFIFGAIAGVMGTCFSVLIRMELARPGDQILGGNHQLYNVL
ITAHAFLMIFFMVMPAMIGGFGNWFVPILIGAPDMAFPRLNNISFWLLPPSLLLLLSSAL
VEVGSGTGWTVYPPLSGITSHSGGAVDLAIFSLHLSGISSILGSINFITTIFNMRGPGMT
MHRLPLFVWSVLVTAFLLLLSLPVLAGAITMLLTDRNFNTTFFDPAGGGDPILYQHLFWF
FGHPEVYILILPGFGIISHIVSTFSRKPVFGYLGMVYAMISIGVLGFLVWAHHMFTVGLD
VDTRAYFTAATMIIAVPTGIKIFSWIATMWGGSIQYKTPMLFAVGFIFLFTIGGLTGIVL
ANSGLDIALHDTYYVVAHFHYVLSMGAVFALFAGFYYWVGKIFGRTYPETLGQIHFWITF
FGVNLTFFPMHFLGLSGMPRRIPDYPDAYAGWNALSSFGSYISVVGIRRFFVVVAITSSS
GKNQKCAESPWAVEQNPTTLEWLVQSPPAFHTFGELPAVKETKS
>P68541 | ATPAM_RAPSA
MELSPRAAELTNLFESRIRNFYANFQVDEIGRVVSVGDGIAQVYGLNEIQAGEMVLFANG
VKGMALNLENENVGIVVFGGDTAIKEGDLVKRTGSIVDVPAGKAMLGRVVDAMGVPIDGR
GALSDHEQRRVEVKAPGILERKSVHEPMQTGLKAVDSLVPIGRGQRELLIGDRQTGKTTI
AIDTILNQKQINSRATSESETMYCVYVAIGQKRSTVGQLIQTLEEANALEYSILVAATAS
DPAPLQFLAPYSGCAMGEYFRDNGMHALIIYDDLSKQAVAYRQMSLLLRRPPGREAFPGD
VFYLHSRLLERAAKRSDQTGAGSLTALPVIETQAGDVSAYIPTNVISITDGQICLETELF
YRGIRPAINVGLSVSRVGSAAQLKAMKQVCGSSKLELAQYREVAAFAQFGSDLDAATQAL
LNRGARLTEVPKQPQYAPLPIEKQILVIYAAVNGFCDRMPLDRISQYEKAIPNSVKPELL
QALKGGLTNERKMEPDAFLKERALALI
>P68542 | ATPAM_BRACM
MELSPRAAELTNLFESRIRNFYANFQVDEIGRVVSVGDGIAQVYGLNEIQAGEMVLFANG
VKGMALNLENENVGIVVFGGDTAIKEGDLVKRTGSIVDVPAGKAMLGRVVDAMGVPIDGR
GALSDHEQRRVEVKAPGILERKSVHEPMQTGLKAVDSLVPIGRGQRELLIGDRQTGKTTI
AIDTILNQKQINSRATSESETMYCVYVAIGQKRSTVGQLIQTLEEANALEYSILVAATAS
DPAPLQFLAPYSGCAMGEYFRDNGMHALIIYDDLSKQAVAYRQMSLLLRRPPGREAFPGD
VFYLHSRLLERAAKRSDQTGAGSLTALPVIETQAGDVSAYIPTNVISITDGQICLETELF

YRGIRPAINVGLSVSRVGSAAQLKAMKQVCGSSKLELAQYREVAAFAQFGSDLDAATQAL
LNRGARLTEVPKQPQYAPLPIEKQILVIYAAVNGFCDRMPLDRISQYEKAIPNSVKPELL
QALKGGLTNERKMEPDAFLKERALALI
>P80261 | NDUS3_SOLTU
MDNQFIFKYSWETLPKKWVKKMERSEHGNRFDTNTDYLFQLLCFMKLHTYTRVQVLIDIC
GVDYPSRKQRFEVVYNLLSIRYNSRIRVQTSADEVTRISSVVSLFPSAGWWEREVWDMFG
VFSINHPDLRRILTDYGFEGHPLRKDFPLSGYVEVRYDDPEKRVVSEPIEMTQEFRYFDF
ASPWEQRSDG
>P83372 | CISY_FRAAN
MAFFRTVTKLRSRLGQPPSLRDSVRCLQTQASSDLDLHSQLKELIPEQQERLKKLKKEHG
KVQLGTITVDMVIGGMRGMTGLLWETSLLDPDEGIRFRGLSIPECQKVLPGATPGGEPLP
EGLLWLLLTGKVPSRSKNMHYPVNNGVVPKFQIMCSRPLMLCLLEHIPMTQFTTGVMALQ
VQSEFQKAYDKGIPKSRYWEPTYEDSLSLIAQLPVVASYVYRRIYKGGRMIPVDDSLDYG
GNFSHLLGFDDHKMQELMRLYVTIHSDHEGGNVSAHTGHLVASALSDPFLSFAAALNGLA
GPLHGLANQEVLLWIKSVVDECGENITKDQLKDYVWKTLNSGKVVPGFGHGVLRKTDPRY
TCQREFALKHLPDDPLFRLVSKLYDVVPPILTELGKVKNPWPNVDAHSGVLLNHFGLTEA
RYFTVLFGVSRSIGIGSQLIWDRALGLPLERPKSVTMESLESFCKKAAS
>P83373 | MDHM_FRAAN
MRPSMSLIRSVSRVARRGYSSESVPQRKVAVLGAAGGIGQPLALLMKLNPLVSQLSLYDI
AGTPGVAADVSHINTRSEVKGYAGEEQLGEALEGCDVVIIPAGVPRKPGMTRDDLFNINA
GIVRSLTAAIAKYCPHAIINMISNPVNSTVPIASEVLKKAGVYDEKKLFGVTTLDVVRAK
TFYAGKAGVPVAEVNVPVVGGHAGITILPLFSQATPKANLSDDYIKALTKRTQDGGTEVV
EAKAGKGSATLSMAYAGALFADACLXGLNGVPDVVECSYVQSSITELPFFASKVRLGKNG
VEEVLDLGPLSDFEKEGLKQLKPELKSSIEKGIKFANQS
>P83483 | ATPBM_ARATH
MASRRVLSSLLRSSSGRSAAKLGNRNPRLPSPSPARHAAPCSYLLGRVAEYATSSPASSA
APSSAPAKDEGKKTYDYGGKGAIGRVCQVIGAIVDVRFEDQEGLPPIMTSLEVQDHPTRL
VLEVSHHLGQNVVRTIAMDGTEGLVRGRKVLNTGAPITVPVGRATLGRIMNVLGEPIDER
GEIKTEHYLPIHRDAPALVDLATGQEILATGIKVVDLLAPYQRGGKIGLFGGAGVGKTVL
IMELINNVAKAHGGFSVFAGVGERTREGNDLYREMIESGVIKLGEKQSESKCALVYGQMN
EPPGARARVGLTGLTVAEYFRDAEGQDVLLFIDNIFRFTQANSEVSALLGRIPSAVGYQP
TLASDLGALQERITTTKKGSITSVQAIYVPADDLTDPAPATTFAHLDATTVLSRQISELG
IYPAVDPLDSTSRMLSPHILGEEHYNTARGVQKVLQNYKNLQDIIAILGMDELSEDDKLT
VARARKIQRFLSQPFHVAEIFTGAPGKYVDLKENINSFQGLLDGKYDDLSEQSFYMVGGI
DEVVAKAEKIAKESAA
>P83484 | ATPBN_ARATH
MASRRVLSSLLRSSSGRSAAKLVNRNPRLPSPSPARHAAPCSYLLGRVAEYATSSPASSA
APSSAPAKDEGKKTYDYGGKGAIGRVCQVIGAIVDVRFEDQEGLPPIMTSLEVQDHPTRL
VLEVSHHLGQNVVRTIAMDGTEGLVRGRKVLNTGAPITVPVGRATLGRIMNVLGEPIDER
GEIKTEHYLPIHRDAPALVDLATGQEILATGIKVVDLLAPYQRGGKIGLFGGAGVGKTVL
IMELINNVAKAHGGFSVFAGVGERTREGNDLYREMIESGVIKLGEKQSESKCALVYGQMN
EPPGARARVGLTGLTVAEYFRDAEGQDVLLFIDNIFRFTQANSEVSALLGRIPSAVGYQP
TLASDLGALQERITTTKKGSITSVQAIYVPADDLTDPAPATTFAHLDATTVLSRQISELG
IYPAVDPLDSTSRMLSPHILGEEHYNTARGVQKVLQNYKNLQDIIAILGMDELSEDDKLT

VARARKIQRFLSQPFHVAEIFTGAPGKYVDLKENINSFQGLLDGKYDDLSEQSFYMVGGI
DEVVAKAEKIAKESAA
>P92532 | RT12_ARATH
MPTFNQLIRHGREEKRRTDRTRALDKCPQKTGVCLRVSTRTPKKPNSALRKIAKVRLSNR
HDIFAYIPGEGHNLQEHSTVLIRGGRVKDLPGVKFHCIRGVKDLMGIPGRRRGRSKYGAE
KPKSI
>P92547 | ATP62_ARATH
MERLTRLNHFLVNMRWDFYEGVIQAGYIRNLQRELDHTPAELLGSKLDLIFFRESLNLST
YVNNWYMQNLGVPGPVNFIEKYHDACFSNYMKLMEIPSPLDQFEIVPLIPMHIGNFYFSF
TNSSLFMLLTLSFFLLLIHFVTKKGGGNLVPNAWQSLVELLYDFVLNLVKEQIGGLSGNV
KQMFFPCILVTFLFLLFCNLQGMIPYSFTVTSHFLITLALSFSIFIGITIVGFQRHGLHF
FSFLLPAGVPLPLAPFLVLLELISYCFRALSLGIRLFANMMAGHSLVKILSGFAWTMLCM
NDIFYFIGALGPLFIVLALTGLELGVAILQAYVFTILICIYLNDAINLH
>P92549 | ATPAM_ARATH
MELSPRAAELTNLFESRIRNFYANFQVDEIGRVVSVGDGIAQVYGLNEIQAGEMVLFANG
VKGMALNLENENVGIVVFGGDTAIKEGDLVKRTGSIVDVPAGKAMLGRVVDAMGVPIDGK
GALSDHEQRRVEVKAPGILERKSVHEPMQTGLKAVDSLVPIGRGQRELLIGGRQTGKTTI
AIDTILNQKQINSRATSESETMYCVYVAIGQKRSTVGQLIQTLEEANALEYSILVAATAS
DPAPLQFLAPYSGCAMGEYFRDNGMHALIIYDDLSKQAVAYRQMSLLLRRPPGREAFPGD
VFYLHSRLLERAAKRSDQTGAGSLTALPVIETQAGDVSAYIPTNVISITDGQICLETELF
YRGIRPAINVGLSVSRVGSAAQLKAMKQVCGSLKLELAQYREVAAFAQFGSDLDAATQAL
LNRGARLTEVLKQPQYAPLPIEKQILVIYAAVNGFCDRMPLDRISQYEKAILNSVKPELL
QALKGGLTNERKMELDAFLKERALALI
>P92557 | RT07_ARATH
MGGLDGEQKLLIKKLVNFRMKEGKRTRVRAIVYQTFHRPARTERDVIKLMVDAVENIKPI
CEVAKVGVAGTIYDVPGIVARDRQQTLAIRWILEAAFKRRISYRISLEKCSFAEILDAYQ
KRGSARRKRENLHGLASTNRSFAHFRWW
>P92969 | RPOT1_ARATH
MWRNILGRASLRKVKFLSDSSSSGTHYPVNRVRGILSSVNLSGVRNGLSINPVNEMGGLS
SFRHGQCYVFEGYATAAQAIDSTDPEDESSGSDEVNELITEMEKETERIRKKARLAAIPP
KRVIAGMGAQKFYMLKQRQVKMETEEWERAARECREILADMCEQKLAPNLPYMKSLFLGW
FEPVRNAIQDDLDTFKIKKGKIPYAPFMEQLPADKMAVITMHKMMGLLMTNAEGVGIVKL
VNAATQIGEAVEQEVRINSFLQKKNKKNATDKTINTEAENVSEEIVAKETEKARKQVTVL
MEKNKLRQVKALVRKHDSFKPWGQEAQVKVGARLIQLLMENAYIQPPAEQFDDGPPDIRP
AFKQNFRTVTLENTKTSRRYGCIECDPLVLKGLDKSARHMVIPYLPMLIPPQNWTGYDQG
AHFFLPSYVMRTHGAKQQRTVMKRTPKEQLEPVYEALDTLGNTKWKINKKVLSLVDRIWA
NGGRIGGLVDREDVPIPEEPEREDQEKFKNWRWESKKAIKQNNERHSQRCDIELKLEVAR
KMKDEEGFYYPHNVDFRGRAYPIHPYLNHLGSDLCRGILEFCEGKPLGKSGLRWLKIHIA
NLYAGGVDKLAYEDRIAFTESHLEDIFDSSDRPLEGKRWWLNAEDPFQCLAACINLSEAL
RSPFPEAAISHIPIHQDGSCNGLQHYAALGRDKLGADAVNLVTGEKPADVYTEIAARVLK
IMQQDAEEDPETFPNATYAKLMLDQVDRKLVKQTVMTSVYGVTYSGARDQIKKRLKERGT
FEDDSLTFHASCYAAKITLKALEEMFEAARAIKSWFGDCAKIIASENNAVCWTTPLGLPV
VQPYRKPGRHLVKTTLQVLTLSRETDKVMARRQMTAFAPNFIHSLDGSHMMMTAVACNRA
GLSFAGVHDSFWTHACDVDVMNTILREKFVELYEKPILENLLESFQKSFPDISFPPLPER

GDFDLRKVLESTYFFN
>P93032 | IDH2_ARATH
MSRQSFSLLKNLRSIASGSKIQTRSVTYMPRPGDGKPRPVTLIPGDGVGPLVTNAVQQVM
EAMHAPVYFEPFEVHGDMKSLPEGLLESIKKNKVCLKGGLKTPVGGGVSSLNVNLRKELD
LFASLVNCFNLPGLASRHENVDIVVIRENTEGEYAGLEHEVVPGVVESLKVITKFCSERI
AKYAFEYAYLNNRKKVTAVHKANIMKLADGLFLESCQEVAKKYPSIAYNEIIVDNCCMQL
VARPEQFDVMVTPNLYGNLVANTAAGIAGGTGVMPGGNVGAEYAVFEQGASAGNVGKDTT
EEQKNANPVALLLSSAMMLRHLQFPSFADRLETAVKRVIAEGNCRTEDLGGNSTTQEVVD
AVIANLD
>P93256 | GCST_MESCR
MRGGGLWQLGQSVTRRLAQAEKKVIARRCFASEADLKKTALYDFHVANGGKMVPFAGWSM
PIQYKDSIMDSTINCRENGSLFDVAHMCGLSLKGKDCIPFLEKLVVGDIAGLAPGTGTLS
VLTNEKGGAIDDTVITKVTDDHIYLVVNAGCREKDLAHIEEHMKAFKAKGGDVSWHIHDE
RSLLALQGPLAAPVLQHLTKEDLSKFYFGQFTFLDINGFPCYLTRTGYTGEDGFEISVPN
EYAVDLAKAMLEKSEGKVRLTGRGARDSLRLEAGLCLYGNDLEQHITPIEAGLTWAVGKR
RRAEGGFLGAEVILKQIADGPPQRRVGFISSGPPARGHSEIQNEKGESIGEITSGGFSPC
LKKNIAMGYVKSGNHKAGTKVNILVRGKPYEGVVTKMPFVPTKYYKSP
>P93285 | COX2_ARATH
MIVLKWLFFTISPCDAAEPWQLGFQDAATPIMQGIIDLHHDIFFFLILILVFVLWILVRA
LWHFHYKKNAIPQRIVHGTTIEILWTIFPSIILMFIAIPSFALLYSMDEVVVDPAITIKA
IGHQWYWTYEYSDYNSSDEQSLTFDSYMIPEEDLELGQLRLLEVDNRVVVPAKTHLRIIV
TSADVLHSWAVPSLGVKCDAVPGRLNQISILVQREGVYYGQCSEICGTNHAFMSIVVEAV
SRKDYGSWVSNQLIPQTGEA
>P93298 | ATP61_ARATH
MRRIFLFDENSLNSSSTIDTSSASTIDTSFASQCTNFSSGQASGTQDTHAGIFEDCPGLN
PNDERVVELQCEIREKCEALTQDPEMGLILGEALHAESDNVPFLQSIADDLTQNGVSGEA
FQEALNIVGQAAASPLDQFEIVPLIPMHIGNFYFSFTNSSLFMLLTLSFFLLLIHFVTKK
GGGNLVPNAWQSLVELLYDFVLNLVKEQIGGLSGNVKQMFFPCILVTFLFLLFCNLQGMI
PYSFTVTSHFLITLALSFSIFIGITIVGFQRHGLHFFSFLLPAGVPLPLAPFLVLLELIS
YCFRALSLGIRLFANMMAGHSLVKILSGFAWTMLCMNDIFYFIGALGPLFIVLALTGLEL
GVAILQAYVFTILICIYLNDAINLH
>P93306 | NDUS2_ARATH
MTTRKRQIKNFTLNFGPQHPAAHGVLRLVLEMNGEVVERAEPHIGLLHRGTEKLIEYKTY
LQALPYFDRSDYVSMMAQEHAYSLAVEKLLNCEVPLRAQYIRVLFCEITRILNHLLALTT
HAMDVGALTPFLWAFEEREKLLEFYERVSGARMHASFIRPGGVAQDLPLGLCRDIDSFTQ
QFASRIDELEEMLTGNRIWKQRLVDIGTVTAQQAKDWGFSGVMLRGPGVCWDLRRAAPYD
VYDQLDFDVPVGTRGDCYDRYCIRIEEMRQSLRIIVQCLNQMPSGMIKADDRKLCPPSRC
RMKLSMESSIHHFELYTEGFSVPASSTYTAVEAPKGEFGVFLVSNGSNRPYRCKIRAPGF
AHLQGLDFMSKHHMLADVVTIIGTQDIVFGEVDR
>P93311 | RM02_ARATH
MRPGRARALRQFTLSTGKSAGRNSSGRITVFHRGGGSKRLLRRIDLKRSTSSMGIVESIE
YDPNRSSQIAPVRWIKGGCQKKMNTIEKFAPPRKILEPTTNTISGLFSFSFLPGKVDKRK
VACFSPGLMAAYVVVGLPTGMPPLSSSKSAFASKGAGSTKTLVKDVFFSAFSSPKAKRET
ASLAFASSFGFPRIAVAGAKPAFFAPRMRQKVRGKSTFSLCEVQKGRTHSILWAHRIKGK

AGLSWQSFRRQDTLGLVGAAGHKKSKPKTDQGNLPAKPIGERAKQLKALRGLRAKDGACK
VDRAPVTYIIASHQLEAGKMVMNCDWSKPSTSSFLQSAQNDHPKPLFTV
>P93313 | NU4M_ARATH
MLEHFCECYFNLSGLILCPVLGSIILLFIPNSRIRLIRLIGLCASLITFLYSLVLWIQFD
SSTAKFQFVESLRWLPYENINFYLGIDGISLFFVILTTFLIPICILVGWSGMRSYGKEYI
IAFLICEFLMIAVFCMLDLLLFYVFPESVLIPMFIIIGVWGSRQRKIKAAYQFFLYTLLG
SLFMLLAILLILFQTGTTDLQILLTTEFSERRQIFLWIAFFASFAVKVPMVPVHIWLPEA
HVEAPTAGSVILAGILLKFGTYGFLRFSIPMFPEATLCFTPFIYTLSAIAIIYTSLTTLR
QIDLKKIIAYSSVAHMNLVTIGMFSPNIQGIGGSILLMLSHGLVSSALFLCVGVLYDRHK
TRLVRYYGGLVSTMPNFSTIFFSFTLANMSLPGTSSFIGEFLILVGAFQRNSLVATLAAL
GMILGAAYSLWLYNRVVSGNLKPDFLHKFSDLNGREVFIFIPFLVGLVWMGVYPKVFLDC
MHTSVSNLVQHGKFH
>P93401 | NU2M_OENBE
MFNLFLAVFPEIFIINATFILLIHGVVFSTSKKDDYPPLVSNVGWLGLLSVLITLLLLAA
GAPLLTIAHLFWNNFFRRDNFTYFCQILLLLSTAGTISMCFDFFEQERFDAFEFIVLILL
STCSMLFMISAYDLIAMYLAIELQSLCFYVLAASKRKSEFSTEAGLKYLILGAFSSGILL
FGCSMIYGSTGATHFDQLAKILTGYEITGARSSGIFMGILFIAVGFLFKITAVPFHMWAP
DIYEGSPTPVTAFLSIAPKISIFANILRVFIYGSYGATLQQIFFFCSIASMILGALAAMA
QTKVKRLLAYSSIGHVGYICIGFSCGTIEGIQSLLIGLFIYALTTINAFAIVLALRQTRV
KYIADLGALAKTNPILAITFSITMFSYAGIPPLAGFCSKFYLFFAALGCGAYFLASVGVV
TSVIGCFYYIRLVKRMFFDTPRTWILYEPMDRNKSLLLAMTSSFITLFFLYPSPLFSVTH
QMALSLYL
>P98012 | COX2_BETVU
MIVREWLFFTMAPCDAAEPWQLGFQDAATPMMQGIIDLHHDIFFFLILILVFVSWILVRA
LWHFHYKKNPIPQRIVHGTTIEIIRTIFPSIILMFIAIPSFALLYSMDEVVVDPAITIKA
IGHQWYRSYEYSDYNSSDEQSLTFDSYTIPEDDPELGQSRLLEVDNRVVVPAKTHIRIIV
TSADVLHSWAVPSSGVKCDAVPGRLNQTSILVQREGVYYGQCSEICGTNHAFMPIVVEAV
SRKDYGSRVSNQLIPQTGEA
>Q00583 | HMDH3_HEVBR
MDEVRRRPPKHIVRKDHDGEVLNSFSHGHHLPPLKPSDYSLPLSLYLANALVFSLFFSVA
YFLLHRWREKIRKSTPLHIVTFPEIAALICLVASVIYLLGFFGIGFVHSFSRASTDSWDV
EEYDDDNIIIKEDTRPTGACAAPSLDCSLSLPTKIHAPIVSTTTTSTLSDDDEQIIKSVV
SGSIPSYSLESKLGNCKRAALIRRETLQRMSGRSLEGLPLDGFDYESILGQCCEMAIGYV
QIPVGIAGPLLLDGKEYTVPMATTEGCLVASANRGCKAIYASGGATSVLLRDGMTRAPVV
RFPTAKRAADLKFFMEDPDNFDTIAVVFNKSSRFARLQSVQCAIAGKNLYMRFSCSTGDA
MGMNMVSKAVQNVIDYLQNDFPDMDVIGLTGNFCADKKAAAVNWIEGRGKSVVCEAIIKE
EVVKKVLKTNVAALVELNMIKNLTGSAVAGSLGGFNAHASNMVTAVYIATGQDPAQNVES
SHCITMMEAVNDGKDLHISVSMPSIELGTVGGGTQLASQSACLNLLGVKGASKDSPGSNS
RLLATIVAGSVLAGELSLMSAIAAGQLVNSHMKYNRSAKDVSKITF
>Q01859 | ATPBM_ORYSJ
MATRRALSSLVRAASRLRGASPAPRPRGPLHRPSPSGYLFNRAAAYATAAAAKEAAPPAP
ATGKATGGGKITDEFTGAGAVGQVCQVIGAVVDVRFDEGLPPILTALEVLDHNIRLVLEV
AQHLGENMVRTIAMDGTEGLVRGQRVLNTGSPITVPVGRATLGRIMNVIGEPIDEKGDIT
TNHFLPIHREAPAFVEQATEQQILVTGIKVVDLLAPYQRGGKIGLFGGAGVGKTVLIMEL

INNVAKAHGGFSVFAGVGERTREGNDLYREMIESGVIKLGDKQSESKCALVYGQMNEPPG
ARARVGLTGLTVAEHFRDAEGQDVLLFIDNIFRFTQANSEVSALLGRIPSAVGYQPTLAT
DLGGLQERITTTKKGSITSVQAIYVPADDLTDPAPATTFAHLDATTVLSRQISELGIYPA
VDPLDSTSRMLSPHVLGEDHYNTARGVQKVLQNYKNLQDIIAILGMDELSEDDKLTVARA
RKIQRFLSQPFHVAEVFTGAPGKYVELKESVNSFQGVLDGKYDDLPEQSFYMVGGIEEVI
AKAEKIAKESAS
>Q01902 | RT07_WHEAT
MGDFDGEQKELIKKLVNFRMIDGKRTRVRAIVYKTFHRLARTERDVIKLMVDAVDNIKPI
CEVVKVGVAGTIYDVPGIVARDRQQTLAIRWILGAAFKRRISYRISLEKCSFAEILDAYR
KRGISRKRRENLHGLASTNRSFAHFRWW
>Q01915 | ATPAM_SOYBN
MEFSVRAAELTTLLESRITNFYTNFQVDEIGRVVSVGDGIARVYGLNEIQAGEMVEFASG
VKGIALNLENENVGIVVFGSDTAIKEGDLVKRTGSIVDVPAGKAMLGRVVDALGVPIDGR
GALSDHERRRVEVKAPGIIERKSVHEPMQTGLKAVDSLVPIGRGQRELIIGDRQTGKTAI
AIDTILNQKQMNSRATSESETLYCVYVAIGQKRSTVAQLVQILSEANALEYSILVAATAS
DPAPLQFLAPYSGCAMGEYFRDNGMHALIIYDDLSKQAVAYRQMSLLLRRPPGREAFPGD
VFYLHSRLLERAAKRSDQTGAGSLTALPVIETQAGDVSAYIPTNVISITDGQICLETELF
YRGIRPAINVGLSVSRVGSAAQLKAMKQVCGSLKLELAQYREVAAFAQFGSDLDAATQAL
LNRGARLTEVLKQPQYAPLPIEKQILVIYAAVNGFCDRMPLDKIPQYERDILTTIKPELL
QSLKGGLTSERKIELEKFLKEKGGTYYI
>Q04050 | NU4M_BRACM
MLEHFCECYSNLSGLILCPVLGSITPLFIPNSRIRPIRLIGLCASLITFLYSPVPRIQFD
SSTAKSQFVESLRWLPYENINFYLGIDGISLFFVILTTFLIPICISVGWSGMRSYGKEYI
TAFLIREFLMIAVFRMLDLLLFYVFPESVPIPMFIIIGVWGSRQRKIKAAYQFFLYTLLG
SLFMLLAILLILFQTGTTDLQISLTTEFSERRQIFLWIASFASFAVKVPMVPVHIWLPEA
HVEAPTAGSVILAGIPLKFGTHGFLRFSIPMFPEATLCSTPFIYTLSAIAIIYTSLTTSR
QIDLKKIIAYSSVAHMNLVTIGMFSPNIQGIGGSILPMLSHGLVPSALFLCVGVLYDRHK
TRLVRYYGGLVSTMPNLSTIFFSFTLANMSSPGTSSFIGEFPILVGAFQRNSLVATLAAL
GMILGAAYSLWLYNRVVSGNLKPDFLHKFSDSNGREVSIFIPFLVGLVRMGVHPKVFPDC
MHTSVSNLVQHGKFH
>Q04654 | ATP6_VICFA
MNLYLQLDLYLYNDNLYLYLYLESGVVPIPSPLEQFEIIPFLPMKIGDLYFSFTNPSLFM
LLTLSLVLLLVHFVTKKGGGKSVPNAWQSLVELIYDFVPNLVNEQIGGLSGNVKQQFFPC
IFVTFTFLLFCNLQGMIPYSFTVTSHFLITLGLSFSIFIGITIVGFQRNGLHFLSFLLPA
GVPLPLAPFLVLLELISYCFRALSLGIRLFANMMAGHSLVKILSGFAWTMLCMNDLLYFI
GDLGPLFIVLALTGPELGVAISQAHVSTISICIYLNDATNLHQTCLLFIYN
>Q04715 | RM16_PETHY
MLLRKYLLVTESQVSKCGFLIVKKKRDVLYPKRTKYSKYRKGRCSRGCKPDGTQLGFGRY
GTKSCRAGRLSYRAIEAARRAIIGHFHRAMSGQFRRNGKIWVRVLADIPITGKPTEVRMG
RGKGNPTGWIARVSRGQILFEMDGVSLSNARQALH
>Q05143 | COX1_PROWI
MVTRWLYSTNHKDIGTMYLIFGAFSGVLGTVFSLLIRMELAQPGNQILNGNHQLYNVIIT
AHAFLMIFFMLMPALMGGFGNWFLPILIGAPDMAFPRLNNISFWLLPPSLLLLVSSALVE
VGAGTGWTVYPPLASIASHSGGSVDLAIFSLHLAGVSSILGAINFICTVFNMRAPGMSML

DLLFVWAVFITAWLLLLCLPVLAGGITMLLTDRNFNTSFFDPAGGGDPILYQHLFWFFGH
PEVYILIIPGFGIISHVIATFSKKPIFGYLGMVYAMCSIGILGFIVWAHHMYVVGLDIDT
RAYFTAATMIIAVPTGIKIFSWVATMWGGSIELRTPMLFAVGFLFLFTVGGLTGVVLANS
GLDVAFHDTYYVVAHFHYVLSMGAVFALFSGFYYWIGKITGLQYPETLGQIHFWLMFLGV
NITFFPMHFLGLAGMPRRIPDYPDCYAGWNAVASYGSYLSITAVLFFFYVVYKTLTSNEV
CPRNPWETTPGVSPTLEWMLPSPPAFHTFEEIQV
>Q06735 | ATPAM_BETVU
MEFSPRAAELTNLLESRITNFYTNFQVDEIGRVVSVGDGIARVYGLNEIQAGEMVEFASG
VKGIALNLENENVGIVVFGSDTAIKEGDLVKRTGSIVDVPAGKAMLGRVVDALGVPIDGR
GALSDHERRRVEVKAPGIIERKSVHEPMQTGLKAVDSLVPIGRGQRELIIGDRQTGKTAI
AIDTILNQKQLNSKATSESETLYCVYVAVGQKRSTVAQLVQILSEANALEYSILVAATAS
DPAPLQFLAPYSGCAMGEYFRDNGMHALIIYDDLSKQAVAYRQMSLLLRRPPGREAFPGD
VFYLHSRLLERAAKRSDQTGAGSLTALPVIETQAGDVSAYIPTNVISITDGQICLETELF
YRGIRPAINVGLSVSRVGSAAQLKAMKQVCGSPKLELAQYREVAAFAQFGSDLDAATQAL
LNRGARLTEVPKQPQYAPLPIEKQILVIYAAVNGFCDRMPLDKISQYERTIPNSVKPELL
QSLKGGLTNEKKMELDSFLKECALNY
>Q0DI31 | CYC_ORYSJ
MASFSEAPPGNPKAGEKIFKTKCAQCHTVDKGAGHKQGPNLNGLFGRQSGTTPGYSYSTA
NKNMAVIWEENTLYDYLLNPKKYIPGTKMVFPGLKKPQERADLISYLKEATS
>Q31708 | RT04_ARATH
MWLLKKLIQRDIDLSPLRFQTCRLLSGNVWNRELTIIQRRILRRLRNRKRSIKKRKIYPK
KYLTSYIQLQTTRKLPFFYGDLPITEMHRGTKRTSYIPFLLNLETRFDVIPLRLYFLETI
PQARQLISHRRVCVNKGMVSITHFKLSHGDIISFQENNAIIRGEEIRRSFYKEILVEKII
GKLLHQPLRMWRRSKTEWFHLLKTKRGCRLLLKSRFLQQLRSSMQEEDLERTKKFGSEKV
CLGSSFAEHKRMKRNLLKSLFLSKRRKDKNLNLPTRTISPIVYNSSLSLYSNSTYCFASP
HKLTMKRRIKRIELPTHYLEVNYRTPKAVVFYGPNIGHIPHDIRLKDLNLLLWSRNGRGQ
NI
>Q31720 | ATP6_BRANA
MQIGLVAQSPLDQFEIVPLIPMNIGNFYFSFTNSSLFMLLTLSFFLLLIHFITKKGGGNL
VPNAWQSLVELLYDFVLNLVKEQIGGLSGNVKQMFFPCILVTFLFLLFCNLQGMIPYSFT
VTSHFLITLALSFSIFIGITIVGFQRHGLHFFSFLLPAGVPLPLAPFLVLLELISYCFRA
LSLGIRLFANMMAGHSLVKILSGFAWTMLCMNEIFYFIGALGPLFIVLALTGLELGVAIL
QAYVFTILICIYLNDAINLH
>Q33994 | NDUS3_BETTR
MDNQFIFKYSWETLPKKWVKKIEKSEHGNRFDTNTDYLFQLLCFLKLHTYTRFQVLIDIC
GVDYPSRKRRFEVVYNLLSTRYNSRIRLQTSADEVTRISSVVSLFPSAGWWEREVWDMFG
VSFINHPDLRRILTDYGFEGHPLRKDFPLSGYVEVRYDDPEKRVVSEPIEMTQEFRYFDF
ASPWEQRNGNEG
>Q34011 | NDUS3_BETWE
MDNQFIFKYSWETLPKKWVKKIEKSEHGNRFDTNTDYLFQLLCFLKLHTYTRFQVLIDIC
GVDYPSRKRRFEVVYNLLSTRYNSRIRLQTSADEVTRISSVVRLFPSAGWWEREVWDMFG
VSFINHPDLRRILTDYGFEGHPLRKDFPLSGYVEVRYDDPEKRVVSEPIEMTQEFRYFDF
ASPWEQRNGNEG
>Q35322 | NDUS3_ORYSJ

MDNQFIFQYSWEILPKKWVHKMKRSEHGNRFYTNTDYLFPLLCFLKWHTYTRVQVLIDIC
GVDYPSRKRRFEVVYNLLSTRYNSRIRVQTSADEVTRISSVVSLFPSAGWWEREVWDMFG
VSFINHPDLRRILTDYGFEGHPLRKDFPLSGYVEVRYDDPEKRVVSEPIEMTQEFRYFDF
ASPWEQRSDG
>Q36450 | NDUS2_NICSY
MTTKNRQIKNFTSNFGPQHPAAHGVSRSVLEMNGEVVERAEPHIGLLQRGTEKLIEYKTY
LQALPYSDRSEYVSMMAQEHAHSSAVERLLNCEVPLRAQYIRVLFREITRISNHSLALTT
HAMDVGASTPFLWAFEEREKLLEFYERVSGARMHASFIRPGGVAQDLPLGLCIDIDSFTQ
QFASRIDELEEMSTGNRIWKQRLVDIGTVTAQQAKDWGFSGVMLRGSGVCWDLRKAAPYD
VHDQLDPDIPVGTRGDRYDRYCIRIEEMRQSVRIIVQCLNQMPSGMIKADDRKLCPPSRS
RMKLSMESSIHHFEPYTEGFSVPAPSTYTAVEAPKGEFGVFLVSNGSNRPYRRKIRAPCF
AHSQGLDSMSKHHMPADVVTIIGTQDIVSGEVDR
>Q36518 | NU3M_PLASU
MIEYLAVLIYFLFSLALASLIIFLSFIFAPQKPDPEKISAYECGFDPFDDARGKFDIRFY
LVAILFIIFDLEVTFLFPWAVTLGKIGFFGFWTMMAFLIILTIGFIYEWKKGALEWE
>Q36664 | NU3M_PINSY
MSEFAPICIYLVISLLVCLIPLGVPFLFASNGSTYPEKLSAYECGFDPFGDARSRFDIRF
YLVSILFIIFDLEVTFFFPWAVSLNKIDLFGFWSMMVFLLILTIGFLYEWKKGALDWE
>Q36665 | RT12_PINSY
MPTSNQSIRHGREKKRRTDRTRALEKCPQKRGVCLRVSTRTPKKPNSALRKIAKVRLSNR
HDIFAYIPGEGHNLQEHSMVLIRGGRVKDLPGVKFHRIRGVKDLLGIPGRKRGRSKYGAE
RPKSK
>Q37625 | NU3M_PROWI
MYEFLGILIYFFIALALSLLLLGLPFLVSTRKADPEKISAYECGFDPFDDARGRFDIQFY
LVAILFIIFDLEVAFLFPWALTLNKIGYFGFWSMMLFLFILTVGFIYEWRKGALDWS
>Q37626 | NU6M_PROWI
MDFLFYIFSSLTLISGSLVIQARNPVHSVLFLVLVFFNAAGLLVLLGLDFFALIFLVVYV
GAIAVLFLFVVMMLNIRITEISEKRLRYLPVGGVLGVLFLFEICILIDNDCIPLLSYDIE
NTALLANYNQLSFIDWRMYLSTSHTIDALGSLLYTYYFYFFLVASLILLVAMIGAIVLTM
QKGIRIKRQQVFLQNTRDFAKTIRKVA
>Q37627 | NU4LM_PROWI
MDLSKYLTVSMILFLLGIWGIFLNRKNIIVMLMSIELMLLAVNLNFLLFSVYIDDCIGQL
FALLILTVAAAESAIGLALLVVYYRIRGTIAVEFINLMKG
>Q37787 | NDUS3_BETVU
MDNQFIFKYSWETLPKKWVKKIEKSEHGNRFDTNTDYLFQLLCFLKLHTYTRFQVLIDIC
GVDYPSRKRRFEVVYNLLSTRYNSRIRLQTCADEVTRISLVVSLFPSAGWWEREVWDMFG
VSFINHPDLRRILTDYGFEGHPLRKDFPLSGYVEVRYDDPEKRVVSEPIEMTQEFRYFDF
ASPWEQRNGNEG
>Q41346 | CYC_STELP
MGFKEGDAKKGANLFKTRCAQCHTLGEGEGNKIGPNLHGLFGRHTGSVEGFSYTDANKAK
GIEWNKDTLFEYLENPKKYIPGTKMAFGGLKKDKDRNDLITFLQDSTK
>Q41629 | ADT1_WHEAT
MTQNLGISVPIMSPSPMFANAPPEKKGVKNFAIDFLMGGVSAAVSKTAAAPIERVKLLIQ
NQDEMIKAGRLSEPYKGIGDCFGRTIKDEGFGSLWRGNTANVIRYFPTQALNFAFKDYFK

RMFNFKKDKDGYWKWFGGNLASGGAAGASSLFFVYSLDYARTRLANDAKASKGGGERQFN
GLVDVYRKTLKSDGIAGLYRGFNISCVGIIVYRGLYFGLYDSLKPVLLTGTLQVCFFASF
ALGWLITNGAGLASYPIDTVRRRMMMTSGEAVKYKSSLDAFQQILKKEGAKSLFKGAGAN
ILRAIAGAGVLSGYDQLQILFFGKKYGSGGA
>Q41630 | ADT2_WHEAT
MTQNLGISVPMMSSSPLFANAPPEKKGVKNFAIDFLMGGVSAAVSKTAAAPIERVKLLIQ
NQDEMIKAGRLSEPYKGIGDCFGRTIKDEGFGSLWRGNTANVIRYFPTQALNFAFKDYFK
RMFNFKKDKDGYWKWFGGNLASGGAAGASSLFFVYSLDYGRTRLANDAKASKGGGDRQFN
GLVDVYRKTLKSDGIAGLYRGFNISCVGIIVYRGLYFGLYDSLKPVLLTGTLQVCSFASF
ALGWLITNGAGLASYPIDTVRRRMMMTSGEAVKYKSSLDAFQQIPAKEGAKSLFKGAGAN
ILRAIAGAGVLSGYDQLQILFFGKKYGSGGA
>Q41898 | ATP5E_MAIZE
MSATTAAVPFWRAAGMTYIGYSNICAALVRNCLKEPFKSEAASREKVHFSISKWTDGKQE
KPTVRTESDD
>Q42525 | HXK1_ARATH
MGKVAVGATVVCTAAVCAVAVLVVRRRMQSSGKWGRVLAILKAFEEDCATPISKLRQVAD
AMTVEMHAGLASDGGSKLKMLISYVDNLPSGDEKGLFYALDLGGTNFRVMRVLLGGKQER
VVKQEFEEVSIPPHLMTGGSDELFNFIAEALAKFVATECEDFHLPEGRQRELGFTFSFPV
KQTSLSSGSLIKWTKGFSIEEAVGQDVVGALNKALERVGLDMRIAALVNDTVGTLAGGRY
YNPDVVAAVILGTGTNAAYVERATAIPKWHGLLPKSGEMVINMEWGNFRSSHLPLTEFDH
TLDFESLNPGEQILEKIISGMYLGEILRRVLLKMAEDAAFFGDTVPSKLRIPFIIRTPHM
SAMHNDTSPDLKIVGSKIKDILEVPTTSLKMRKVVISLCNIIATRGARLSAAGIYGILKK
LGRDTTKDEEVQKSVIAMDGGLFEHYTQFSECMESSLKELLGDEASGSVEVTHSNDGSGI
GAALLAASHSLYLEDS
>Q42560 | ACO1_ARATH
MASENPFRSILKALEKPDGGEFGNYYSLPALNDPRIDKLPYSIRILLESAIRNCDEFQVK
SKDVEKILDWENTSPKQVEIPFKPARVLLQDFTGVPAVVDLACMRDAMNNLGGDSNKINP
LVPVDLVIDHSVQVDVARSENAVQANMELEFQRNKERFAFLKWGSNAFHNMLVVPPGSGI
VHQVNLEYLARVVFNTNGLLYPDSVVGTDSHTTMIDGLGVAGWGVGGIEAEATMLGQPMS
MVLPGVVGFKLTGKLRDGMTATDLVLTVTQMLRKHGVVGKFVEFHGEGMRELSLADRATI
ANMSPEYGATMGFFPVDHVTLQYLRLTGRSDDTVSMIEAYLRANKMFVDYSEPESKTVYS
SCLELNLEDVEPCVSGPKRPHDRVPLKEMKADWHSCLDNRVGFKGFAVPKEAQSKAVEFN
FNGTTAQLRHGDVVIAAITSCTNTSNPSVMLGAALVAKKACDLGLEVKPWIKTSLAPGSG
VVTKYLAKSGLQKYLNQLGFSIVGYGCTTCIGNSGDIHEAVASAIVDNDLVASAVLSGNR
NFEGRVHPLTRANYLASPPLVVAYALAGTVDIDFETQPIGTGKDGKQIFFRDIWPSNKEV
AEVVQSSVLPDMFKATYEAITKGNSMWNQLSVASGTLYEWDPKSTYIHEPPYFKGMTMSP
PGPHGVKDAYCLLNFGDSITTDHISPAGSIHKDSPAAKYLMERGVDRRDFNSYGSRRGND
EIMARGTFANIRIVNKHLKGEVGPKTVHIPTGEKLSVFDAAMKYRNEGRDTIILAGAEYG
SGSSRDWAAKGPMLLGVKAVISKSFERIHRSNLVGMGIIPLCFKAGEDAETLGLTGQELY
TIELPNNVSEIKPGQDVTVVTNNGKSFTCTLRFDTEVELAYFDHGGILQYVIRNLIKQ
>Q42777 | MCCA_SOYBN
MASLALLRRTTLSHSHVRARAFSEGKSSNRHRIEKILVANRGEIACRITRTARRLGIQTV
AVYSDADRDSLHVATADEAIRIGPPPARLSYLNGASIVDAAIRSGAQAIHPGYGFLSESA
DFAKLCEESGLTFIGPPASAIRDMGDKSASKRIMGAAGVPLVPGYHGYDQDIEKMKLEAD

RIGYPVLIKPTHGGGGKGMRIVHTPDEFVESFLAAQREAAASFGVNTILLEKYITRPRHI
EVQIFGDKHGNVLHLYERDCSVQRRHQKIIEEAPAPNISADFRAQLGVAAVSAAKAVNYY
NAGTVEFIVDTVSDEFYFMEMNTRLQVEHPVTEMIVGQDLVEWQILVANGEALPLSQSQV
PLSGHAFEARIYAENVQKGFLPATGVLHHYHVPVSSAVRVETGVKEGDKVSMHYDPMIAK
LVVWGENRAAALVKLKDSLSKFQVAGLPTNVNFLQKLANHRAFAIGNVETHFIDNYKEDL
FVDANNSVSVKEAYEAARLNASLVAACLIEKEHFILARNPPGGSSLLPIWYSSPPFRIHH
QAKRRMELEWDNEYGSGSSKIMKLTITYQPDGRYLIETEQNGSPVLEVKSTYVKDNYFRV
EAAGVINDVNVAVYSKDQIRHIHIWQGSCHHYFREKLGLELSEDEESQHKPKVETSANPQ
GTVVAPMAGLVVKVLVENKTRVEEGQPVLVLEAMKMEHVVKAPSSGYVHGLQLMVGEQVS
DGSVLFSVKDQ
>Q43008 | SODM_ORYSJ
MALRTLASRKTLAAAALPLAAAAAARGVTTVALPDLPYDYGALEPAISGEIMRLHHQKHH
ATYVANYNKALEQLDAAVAKGDAPAIVHLQSAIKFNGGGHVNHSIFWNNLKPISEGGGDP
PHAKLGWAIDEDFGSFEALVKKMSAEGAALQGSGWVWLALDKEAKKLSVETTANQDPLVT
KGANLVPLLGIDVWEHAYYLQYKNVRPDYLSNIWKVMNWKYAGEVYENATA
>Q43175 | CISY_SOLTU
MVFYRSVSLLSKLRSRAVQQSNVSNSVRWLQVQTSSGLDLRSELVQELIPEQQDRLKKIK
SDMKGSIGNITVDMVLGGMRGMTGLLWKPHYLDPDEGIRFRGLSIPECQKVLPAAKPGGE
PLPEGLLWLLLTGKVPSKEQVNSIVSGIAESGIISLIIMYTTIDALPVTAHPMTQFATGV
MALQVQSEFQKAYEKGIHKSKYWEPTYEDSMNLIAQVPLVAAYVYRRMYKNGDTIPKDES
LDYGANFAHMLGFSSSEMHELLMRLYVTIHSDHEGGNVSAHTGHLVASALSDPYLSFAAA
LNGLAGPLHGLANQEVLLWIKSVVEECGENISKEQLKDYVWKTLNSGKVVPGFGHGVLRK
TVPRYTCQREFAMKHLPEDPLFQLVSKLYEVFLLFLQNLAKLKPWPNVDAHSGVLLNYYG
LTEARYYTVLFGVSRALGICSQLIWDRALGLPLERPKSVTMEWLENQCKKA
>Q56XE8 | HXK4_ARATH
MGKVLVMLTAAAAVVACSVATVMVRRRMKGRRKWRRVVGLLKDLEEACETPLGRLRQMVD
AIAVEMQAGLVSEGGSKLKMLLTFVDDLPNGSETGTYYALHLGGSYFRIIKVHLGGQRSS
LEVQDVERHSIPTSLMNSTSEVLFDFLASSLQRFIEKEGNDFSLSQPLKRELAFTFSFPV
KQTSISSGVLIKWTKGFAISEMAGEDIAECLQGALNKRGLDIRVAALVNDTVGALSFGHF
HDPDTIAAVVFGTGSNACYLERTDAIIKCQNPRTTSGSMVVNMEWGNFWSSRLPRTSYDL
ELDAESMNSNDMGFEKMIGGMYLGDIVRRVILRMSQESDIFGPISSILSTPFVLRTNSVS
AMHEDDTSELQEVARILKDLGVSEVPMKVRKLVVKICDVVTRRAARLAAAGIAGILKKVG
RDGSGGGRRSDKQIMRRTVVAVEGGLYLNYRMFREYMDEALRDILGEDVAQHVVVKAMED
GSSIGSALLLASSQSVQTIPSV
>Q5M729 | OPD23_ARATH
MAYASRIINHSKKLKDVSTLLRRENAATIRYYSNTNRAPLNREDTFNSRLGYPPLERISI
CSTSTLPVSIIFSTTRSNLSSAMGRPIFGKEFSCLMQSARGFSSGSDLPPHQEIGMPSLS
PTMTEGNIARWLKKEGDKVAPGEVLCEVETDKATVEMECMEEGYLAKIVKAEGSKEIQVG
EVIAITVEDEEDIGKFKDYTPSSTADAAPTKAEPTPAPPKEEKVKQPSSPPEPKASKPST
PPTGDRVFASPLARKLAEDNNVPLSDIEGTGPEGRIVKADIDEYLASSGKGATAKPSKST
DSKAPALDYVDIPHSQIRKVTASRLAFSKQTIPHYYLTVDTCVDKLMALRSQLNSFKEAS
GGKRISVNDLVVKAAALALRKVPQCNSSWTDDYIRQFKNVNINVAVQTENGLYVPVVKDA
DRKGLSTIGEEVRLLAQKAKENSLKPEDYEGGTFTVSNLGGPFGIKQFCAVVNPPQAAIL
AVGSAEKRVVPGNGPDQFNFASYMPVTLSCDHRVVDGAIGAEWLKAFKGYIENPKSMLL

>Q5YLB5 | GYRA_NICBE
MKLHTLNPQTPLTQSKPMAFSTGITPSRFSGLRKTSSELRFLSSVTPPPRKQLRPVSARR
KEEEVGDEGNGSVILRDRGENEDRNGGERVVLTELHKEATEAYMSYAMSVLLGRALPDVR
DGLKPVHRRILYAMHELGLSSKKPYKKCARVVGEVLGKFHPHGDTAVYDSLVRMAQDFSL
RSPLIRGHGNFGSIDADPPAAMRYTECRLEALTESMLLADLEQNTVDFVPNFDNSQKEPS
LLPARVPNLLLNGASGIAVGMATNIPPHNLGELVDALSALIHNPEATLQELLEYMPGPDF
PTGGIIMGNIGILEAFRTGRGRVVIRGKTDIELLDSKTKRAAIIIQEIPYQTNKASLVEK
IADLVENKILEGVSDIRDESDRSGMRIVIELKRGSDPAIVLNNLYRLTALQSSFSCNMVG
ILNGQPKLMGLKELLQAFLDFRCSVVERRARFKLSQAQERNHIVEGIIVGLDNLDEVINT
IRKASSNALAAASLRKEFELSEKQAEAILDISLRRLTALERNKFVEEGKSLRTQISKLEE
LLSSKKQILQLIEEEAIEIKNKFFNPRRSMLEDTDSGDLEDIDVIPNEEMLLAISEKGYV
KRMKPDTFNLQNRGTIGKSVGKLRVNDAMSDFLVCRAHDKVLYFSDKGTVYSSPAYKIPE
CSRTAAGTPLVQILSLSDGERITSIIPVSEFAADQYLVMLTVNGYIKKVSLNYFASIRCT
GIIAIQLVPDDELKWVKCCSNNDFVAMASQNGMVILTPCANIRALGRNTRGSVAMRLKEG
DKVASMDIIPDALQKELDKTLEVQQRQYRSMKGPWLLFVSESGYGKRVPVSRFRTSPLNR
VGLFGYKFSSEDCLAAVFVVGFSLGEDGESDEQVVLVSQSGTVNRIKVRDISIQSRYARG
VILMRLEHAGKIQSASLISAADADPEDEDATAVAA
>Q6K548 | VDAC1_ORYSJ
MVGPGLYPEIGKKARDLLYRDYQTDHKFTLTTYTSNGVAITATSTKKADLIFGEIQSQIK
NKNITVDVKANSDSNVVTTVTVDELTPGLKSILSFAVPDQRSGKFELQYSHDYAGVSASI
GLTASPVVNLSSVFGTKALAVGADVSLDTATGNLTKYNAGLSFSNDDLIASLNLNNKGDS
LTASYYHIVNHSATAVGAELTHSFSSNENSLTFGTQHTLDPLTVVKARFNNSGKASALLQ
HEWRPKSVWTISAEVDTKAIDKSSKVGIAVALKP
>Q8H1Y0 | ODPA2_ARATH
MALSRLSSRSNTFLKPAITALPSSIRRHVSTDSSPITIETAVPFTSHLCESPSRSVETSS
EEILAFFRDMARMRRMEIAADSLYKAKLIRGFCHLYDGQEALAVGMEAAITKKDAIITSY
RDHCTFIGRGGKLVDAFSELMGRKTGCSHGKGGSMHFYKKDASFYGGHGIVGAQIPLGCG
LAFAQKYNKDEAVTFALYGDGAANQGQLFEALNISALWDLPAILVCENNHYGMGTATWRS
AKSPAYFKRGDYVPGLKVDGMDALAVKQACKFAKEHALKNGPIILEMDTYRYHGHSMSDP
GSTYRTRDEISGVRQVRDPIERVRKLLLTHDIATEKELKDMEKEIRKEVDDAVAQAKESP
IPDASELFTNMYVKDCGVESFGADRKELKVTLP
>Q8L6J5 | RPO1B_TOBAC
MWRYISKHAYSRKFRNSHDSALLGFSQYSSSFGKTRPLQCLCEESTTHPNLGLSQNSIFS
RISRKVRHLEGICEESSKNPHLGLSQNSTFSSVKGDFRICGKRGSGSLGRLRSYGSAAEA
IVSTSEEDIDEIQELIEEMDKENEALKANLQPKQPKTIGGMGVGKYNFLRRRQIKVETEA
WEEAAKEYQELLMDMCEQKLAPNLPYMKSLFLGWFEPLRDAIAAEQKLCDEGKNRGAYAP
FFDQLPAEMMAVITMHKLMGLLMTGGGTGSARVVQAASYIGEAIEHEARIHRFLEKTKKS
NALSGDLEETPGDMMKERERLRKKVKILMKKQKLRQVRKIVKQQDDEKPWGQDNLVKVGC
RLIQILMETAYIQPPNDQLDDGPPDIRPAFVHTLKTVETMKGSRRYGVIQCDPLVRKGLD
KTARHMVIPYMPMLVPPQSWLGYDKGGYLFLPSYIMRTHGAKQQREAVKRVPKKQLEPVF
QALDTLGNTKWRVNRKVLGIVDRIWASGGRLADLVDREDVPLPEAPDTEDEAEIRKWKWK
VKGVKKENCERHSQRCDIELKLAVARKMKDEDGFYYPHNLDFRGRAYPMHPYLNHLGSDL
CRGILEFAEGRPLGTSGLRWLKIHLANVYGGGVDKLSYEGRVAFSENHLEDIFDSAERPL
EGKRWWLGAEDPFQCLATCINIAEALRSPSPETAISYMPIHQDGSCNGLQHYAALGRDKL

GAAAVNLVAGDKPADVYSGIAARVLDIMKRDAAKDPANDPNVMRARLLINQVDRKLVKQT
VMTSVYGVTYIGARDQIKKRLKERGVIEDDNELFAAACYAAKTTLTALGEMFEAARSIMS
WLGDCAKIIAMENHPVRWTTPLGLPVVQPYRKLGRHLIKTSLQILTLQRETDKVMVKRQR
TAFPPNFVHSLDGSHMMMTAIACKESGLSFAGVHDSYWTHACDVDQMNKILREKFVELYD
APILENLLESFQQSFPDLQFPPLPERGDFDLREVLESPYFFN
>Q8L7B5 | CH60B_ARATH
MYRLVSNVASKARIARKCTSQIGSRLNSTRNYAAKDIRFGVEARALMLRGVEDLADAVKV
TMGPKGRNVIIEQSWGAPKVTKDGVTVAKSIEFKDRIKNVGASLVKQVANATNDVAGDGT
TCATVLTRAIFTEGCKSVAAGMNAMDLRRGIKLAVDTVVTNLQSRARMISTSEEIAQVGT
ISANGDREIGELIAKAMETVGKEGVITIQDGKTLFNELEVVEGMKIDRGYISPYFITNPK
TQKCELEDPLILIHEKKISNINAMVKVLELALKKQRPLLIVAEDVESDALATLILNKLRA
NIKVCAVKAPGFGENRKANLHDLAALTGAQVITEELGMNLDNIDLSMFGNCKKVTVSKDD
TVVLDGAGDKQAIGERCEQIRSMVEASTSDYDKEKLQERLAKLSGGVAVLKIGGASETEV
SEKKDRVTDALNATKAAVEEGIVPGGGVALLYASKELEKLSTANFDQKIGVQIIQNALKT
PVYTIASNAGVEGAVVVGKLLEQDNPDLGYDAAKGEYVDMIKAGIIDPLKVIRTALVDAA
SVSSLLTTTEAVVTEIPTKEVASPGMGGGGMGGMGGMGGMGGMGF
>Q8LAD2 | SUCA2_ARATH
MSRQVTRLLGSLRHSGGGCSGSSKVCSLTSLVQSRSFGTTPPPPAAVFVDKNTRVICQGI
TGKNGTFHTEQAIEYGTKMVAGVTPKKGGTEHLGLPVFNTVAEAKAETKANASVIYVPAP
FAAAAIMEGLAAELDLIVCITEGIPQHDMVRVKAALNSQSKTRLIGPNCPGIIKPGECKI
GIMPGYIHKPGKIGIVSRSGTLTYEAVFQTTAVGLGQSTCVGIGGDPFNGTNFVDCLEKF
FVDPQTEGIVLIGEIGGTAEEDAAALIKENGTDKPVVAFIAGLTAPPGRRMGHAGAIVSG
GKGTAQDKIKSLRDAGVKVVESPAKIGAAMFELFQERGLLK
>Q8LBZ7 | DHSB1_ARATH
MASGLIGRLVGTKPSKLATAARLIPARWTSTGAEAETKASSGGGRGSNLKTFQIYRWNPD
NPGKPELQNYQIDLKDCGPMVLDALIKIKNEMDPSLTFRRSCREGICGSCAMNIDGCNGL
ACLTKIQDEASETTITPLPHMFVIKDLVVDMTNFYNQYKSIEPWLKRKTPASVPAKEILQ
SKKDRAKLDGMYECILCACCSTSCPSYWWNPESYLGPAALLHANRWISDSRDEYTKERLE
AIDDEFKLYRCHTILNCARACPKGLNPGKQITHIKQLQR
>Q8LFC0 | IDH1_ARATH
MSRRSLTLLKNLARNANGSGIQTRSVTYMPRPGDGAPRAVTLIPGDGIGPLVTNAVEQVM
EAMHAPIFFEKYDVHGEMSRVPPEVMESIRKNKVCLKGGLKTPVGGGVSSLNVQLRKELD
LFASLVNCFNLPGLPTRHENVDIVVIRENTEGEYAGLEHEVVPGVVESLKVITKFCSERI
AKYAFEYAYLNNRKKVTAVHKANIMKLADGLFLESCREVAKKYPSITYNEIIVDNCCMQL
VAKPEQFDVMVTPNLYGNLVANTAAGIAGGTGVMPGGNVGADHAVFEQGASAGNVGKDKI
VLENKANPVALLLSSAMMLRHLQFPSFADRLETAVKKVIAEGKCRTKDLGGTSTTQEVVD
AVIAKLD
>Q8LFT2 | DRP3B_ARATH
MSVDDLPPSSASAVTPLGSSVIPIVNKLQDIFAQLGSQSTIALPQVAVVGSQSSGKSSVL
EALVGRDFLPRGNDICTRRPLRLQLVQTKPSSDGGSDEEWGEFLHHDPVRRIYDFSEIRR
EIEAETNRVSGENKGVSDIPIGLKIFSPNVLDISLVDLPGITKVPVGDQPSDIEARIRTM
ILTYIKEPSCLILAVSPANTDLANSDALQIAGNADPDGHRTIGVITKLDIMDRGTDARNH
LLGKTIPLRLGYVGVVNRSQEDILMNRSIKDALVAEEKFFRSRPVYSGLTDRLGVPQLAK
KLNQVLVQHIKALLPSLKSRINNALFATAKEYESYGDITESRGGQGALLLSFITKYCEAY

SSTLEGKSKEMSTSELSGGARILYIFQSVFVKSLEEVDPCEDLTADDIRTAIQNATGPRS
ALFVPDVPFEVLVRRQISRLLDPSLQCARFIFDELVKISHQCMMKELQRFPVLQKRMDEV
IGNFLREGLEPSQAMIRDLIEMEMDYINTSHPNFIGGTKAVEQAMQTVKSSRIPHPVARP
RDTVEPERTASSGSQIKTRSFLGRQANGIITDQAVPTAADAERPAPAGSTSWSGFSSIFR
GSDGQAAAKNNLLNKPFSETTQEVYQNLSTIYLKEPPTILKSSETHSEQESVEIEITKLL
LKSYYDIVRKNVEDLVPKAIMHFLVNYTKRELHNVFIEKLYRENLIEELLKEPDELAIKR
KRTQETLRILQQANRTLDELPLEAESVERGYKIGSEAKHEELPGTRRSRTETNGNGRLHM

>Q8LPW2 | RT13_SOYBN
MFGSARILSDVTLRLRQNLSVHGVRVQNINIGGGVGGEIPDNKRLVYALQNLHGIGRSKA
QHIVAELGVENKFVKDLSKRELYSIRELLSKYLIGNDLKKCVERDVGRLVGIQCYRGIRH
VDSLPCRGQRTHTNARTRRSRKTFSGSR
>Q8RWN9 | OPD22_ARATH
MASRIINHSKKLKHVSALLRRDHAVAVRCFSNSTHPSLVGREDIFKARLNYSSVERISKC
GTGNVTMLSGISTTSTKLSSPMAGPKLFKEFISSQMRSVRGFSSSSDLPPHQEIGMPSLS
PTMTEGNIARWLKKEGDKVAPGEVLCEVETDKATVEMECMEEGFLAKIVKEEGAKEIQVG
EVIAITVEDEDDIQKFKDYTPSSDTGPAAPEAKPAPSLPKEEKVEKPASAPEAKISKPSS
APSEDRIFASPLARKLAEDNNVPLSSIKGTGPEGRIVKADVEDFLASGSKETTAKPSKQV
DSKVPALDYVDIPHTQIRKVTASRLAFSKQTIPHYYLTVDTCVDKMMGLRSQLNSFQEAS
GGKRISVNDLVIKAAALALRKVPQCNSSWTDEYIRQFKNVNINVAVQTENGLYVPVVKDA
DKKGLSTIGEEVRFLAQKAKENSLKPEDYEGGTFTVSNLGGPFGIKQFCAVINPPQAAIL
AIGSAEKRVVPGTGPDQYNVASYMSVTLSCDHRVIDGAIGAEWLKAFKGYIETPESMLL
>Q8VWF8 | RPOT2_NICSY
MSSTKTPISLTIKLNQFTDKPTGLDINPYHNSPIMWRNIIKQLSSRTPQKLLFSSKNRTY
SFLGFGQDSIFKDNTKFRSLIPISCSNIVMGFQNLGEYLPGDEFLSRPLIKNQVNNNFCC
RKSYASVAEAVAVSSTDAEEDVSVVDEVHELLTELKKEEKKQFAFRRRKQRMLTSGMGHR
KYQTLKRRQVKVETEAWEQAAKEYKELLFDMCEQKLAPNLPYVKSLFLGWFEPLRDKIAE
EQELCSQGKSKAAYAKYFYQLPADMMAVITMHKLMGLLMTGGDHGTARVVQAALVIGDAI
EQEVRIHNFLEKTKKQKAEKDKQKEDGEHVTQEQEKLRKKVTNLMKKQKLRAVGQIVRRQ
DDSKPWGQDARAKVGSRLIDLLLQTAYIQPPANQLAVDPPDIRPAFVHSVRTVAKETKSA
SRRYGIIQCDELVFKGLERTARHMVIPYMPMLVPPVKWTGYDKGGHLYLPSYVMRTHGAR
QQREAVKRASRNQLQPVFEALDTLGNTKWRINKRVLSVVDRIWAGGGRLADLVDRDDAPL
PEEPDTEDEALRTKWRWKVKSVKKENRERHSQRCDIELKLAVARKMKDEESFFYPHNVDF
RGRAYPMHPHLNHLGSDICRGVLEFAEGRPLGESGLRWLKIHLANLFAGGVEKLSLEGRI
GFTENHMDDIFDSSDKPLEGRRWWLNAEDPFQCLAVCINLSEAVRSSSPETSVSHIPVHQ
DGSCNGLQHYAALGRDKLGAAAVNLVAGEKPADVYSGIAARVLDIMKRDAQRDPAEFPDA
VRARVLVNQVDRKLVKQTVMTSVYGVTYIGARDQIKRRLKERGAIADDSELFGAACYAAK
VTLTALGEMFEAARSIMTWLGECAKIIASENEPVRWTTPLGLPVVQPYRKIGRHLIKTSL
QILTLQRETEKVMVKRQRTAFPPNFIHSLDGSHMMMTAVACRRAGLNFAGVHDSYWTHAC
DVDKLNRILREKFVELYEAPILEKLLESFQTSYPTLLFPPLPERGDFDMRDVLESPYFFN

>Q93Y94 | RPOT1_NICSY
MWRYISKQAYSRKFRNSHDSALLGFSQYSSSFGKTRPLQCLCEESTTNPNLGLSQNSIFS
RISRKVRHLEGICEESSKNPHLGLSQNSLFSSVKGDFRVCGKRGSGSLGFLRSYGSAAEA

IASTSEEDIDEIQELIEEMNKENEALKTNLQPKQPKTIGGMGVGKYNLLRRRQIKVETEA
WEEAAKEYQELLMDMCEQKLAPNLPYMKSLFLGWFEPLRDAIAAEQKLCDEGKNRGAYAP
FFDQLPAEMMAVITMHKLMGLLMTGGGTGSARVVQAASHIGEAIEHEARIHRFLEKTKKS
NALSGDLEDTPGDIMKERERVRKKVKILMKKQKLQQVRKIVKQQDDEKPWGQDNLVKVGC
RLIQILMETAYIQPPNDQLDDCPPDIRPAFVHTLKTVETMKGSRRYGVIQCDPLVRKGLD
KTARHMVIPYMPMLVPPQSWLGYDKGAYLFLPSYIMRTHGAKQQREAVKRVPKKQLEPVF
QALDTLGNTKWRLNRKVLGIVDRIWASGGRLADLVDREDVPLPEEPDAEDEAQIRKWKWK
VKGVKKENCERHSQRCDIELKLAVARKMKDEDGFYYPHNLDFRGRAYPMHPYLNHLGSDL
CRGILEFAEGRPLGKSGLRWLKIHLANVYGGGVDKLSYEGRVAFSENHVEDIFDSAERPL
EGKRWWLGAEDPFQCLATCINIAEALRSPSPETAISYMPIHQDGSCNGLQHYAALGRDTL
GAAAVNLVAGDKPADVYSGIAARVLDIMKRDAAKDPANDPNVMRARLLINQVDRKLVKQT
VMTSVYGVTYIGARDQIKRRLKERGVIEDDNELFAACYAAKTTLTALGEMFEAARSIMS
WLGDCAKIIAMENHPVRWTTPLGLPVVQPYRKLGRHLIKTSLQILTLQRETDKVMVKRQR
TAFPPNFVHSLDGSHMMMTAIACKESGLSFAGVHDSYWTHASDVDQMNKILREKFVELYD
APILENLLESFQQSFPDLQFPPLPERGDFDLREVLESPYFFN
>Q93ZM7 | CH60C_ARATH
MYRVLSKLSSSIGSSTSRKLVSGRIISSRNYAAKDISFGIGARAAMLQGVSEVAEAVKVT
MGPKGRNVIIESSYGGPKITKDGVTVAKSISFQAKAKNIGAELVKQVASATNKVAGDGTT
CATVLTQAILIEGCKSVAAGVNVMDLRVGINMAIAAVVSDLKSRAVMISTPEEITQVATI
SANGEREIGELIARAMEKVGKEGVITVADGNTLDNELEVVEGMKLARGYISPYFITDEKT
QKCELENPIILIHEKKISDINSLLKVLEAAVKSSRPLLIVAEDVESDALAMLILNKHHGG
LKVCAIKAPGFGDNRKASLDDLAVLTGAEVISEERGLSLEKIRPELLGTAKKVTVTRDDT
IILHGGGDKKLIEERCEELRSANEKSTSTFDQEKTQERLSKLSGGVAVFKVGGASESEVG
ERKDRVTDALNATRAAVEEGIIPGGGVALLYATKALDNLQTENEDQRRGVQIVQNALKAP
AFTIAANAGYDGSLVVGKLLEQDDCNFGFDAAKGKYVDMVKAGIIDPVKVIRTALTDAAS
VSLLLTTTEASVLVKADENTPNHVPDMASMGM
>Q945K7 | IDH5_ARATH
MTMAANLARRLIGNRSTQILGAVNSSSGAASSVARAFCSSTTPITATLFPGDGIGPEIAE
SVKKVFTTAGVPIEWEEHYVGTEIDPRTQSFLTWESLESVRRNKVGLKGPMATPIGKGHR
SLNLTLRKELNLYANVRPCYSLPGYKTRYDDVDLITIRENTEGEYSGLEHQVVRGVVESL
KIITRQASLRVAEYAFLYAKTHGRERVSAIHKANIMQKTDGLFLKCCREVAEKYPEITYE
EVVIDNCCMMLVKNPALFDVLVMPNLYGDIISDLCAGLVGGLGLTPSCNIGEDGVALAEA
VHGSAPDIAGKNLANPTALLLSGVMMLRHLKFNEQAEQIHSAIINTIAEGKYRTADLGGS
STTTEFTKAICDHL
>Q94B78 | GCSP2_ARATH
MERARRLAYRGIVKRLVNDTKRHRNAETPHLVPHAPARYVSSLSPFISTPRSVNHTAAFG
RHQQTRSISVDAVKPSDTFPRRHNSATPDEQTHMAKFCGFDHIDSLIDATVPKSIRLDSM
KFSKFDAGLTESQMIQHMVDLASKNKVFKSFIGMGYYNTHVPTVILRNIMENPAWYTQYT
PYQAEISQGRLESLLNFQTVITDLTGLPMSNASLLDEGTAAAEAMAMCNNILKGKKKTFV
IASNCHPQTIDVCKTRADGFDLKVVTSDLKDIDYSSGDVCGVLVQYPGTEGEVLDYAEFV
KNAHANGVKVVMATDLLALTVLKPPGEFGADIVVGSAQRFGVPMGYGGPHAAFLATSQEY
KRMMPGRIIGISVDSSGKQALRMAMQTREQHIRRDKATSNICTAQALLANMAAMYAVYHG
PAGLKSIAQRVHGLAGIFSLGLNKLGVAEVQELPFFDTVKIKCSDAHAIADAASKSEINL
RVVDSTTITASFDETTTLDDVDKLFKVFASGKPVPFTAESLAPEVQNSIPSSLTRESPYL

THPIFNMYHTEHELLRYIHKLQSKDLSLCHSMIPLGSCTMKLNATTEMMPVTWPSFTDIH
PFAPVEQAQGYQEMFENLGDLLCTITGFDSFSLQPNAGAAGEYAGLMVIRAYHMSRGDHH
RNVCIIPVSAHGTNPASAAMCGMKIITVGTDAKGNINIEEVRKAAEANKDNLAALMVTYP
STHGVYEEGIDEICNIIHENGGQVYMDGANMNAQVGLTSPGFIGADVCHLNLHKTFCIPH
GGGGPGMGPIGVKNHLAPFLPSHPVIPTGGIPQPEKTAPLGAISAAPWGSALILPISYTY
IAMMGSGGLTDASKIAILNANYMAKRLEKHYPVLFRGVNGTVAHEFIIDLRGFKNTAGIE
PEDVAKRLMDYGFHGPTMSWPVPGTLMIEPTESESKAELDRFCDALISIREEIAQIEKGN
ADVQNNVLKGAPHPPSLLMADTWKKPYSREYAAFPAPWLRSSKFWPTTGRVDNVYGDRKL
VCTLLPEEEQVAAAVSA
>Q95747 | RM16_ARATH
MYLTIKSIMLLWKYLLVTESQVSKCGFHIVKKKGDVLYPKRTKYSKYRKGRCSRGCKPDG
TKLGFGRYGIKSCKAGCLSYRAIEAARRAIIGHFHRAMSGQFRRNGKIWVRVFADLPITG
KPTEVRMGRGKGNPTGWIARVSTGQILFEMDGVSLANARQAATLAAHKLCLSTKFVQWS
>Q95748 | NDUS3_ARATH
MDNQFIFKYSWETLPKKWVKKMERSEHGNRFDTNTDYLFQLLCFLKLHTYTRVQVLIDIC
GVDYPSRKKRFEVVYNLLSTRYNSRIRVQTSADEVTRISSVVSLFPSAGWWEREVWDMFG
VSFINHPDLRRILTDYGFEGHPLRKDFPLSGYVQVRYDDPEKRVVSEPIEMTQEFRYFDF
ASPWEQRSDG
>Q95749 | RT03_ARATH
MARKGNPISVRLGKNRSSDSSWFSEYYYGKFVYQDVNLRSYFGSIRPPTRLTFGFRLGRC
IILHFPKRTFIHFFLPRRPRRLKRREKTRPGKEKGRWWTTFGKAGPIECLHSSDDTEEER
NEVRGRGARKRVESIRLDDRKKQNEIRGWPKKKQRYGYHDRLPSIKKNLSKLLRISGAFK
HPKYAGVVNDIAFLIENDDSFKKTKLFKLFFQNKSRSDGPTSYLRTLPAVRPSLNFLVMQ
YFFNTKNQINFDPVVVLNHFVAPGAAEPSTMGRANAQGRSLQKRIRSRIAFFVESLTSEK
KCLAEAKNRLTHFIRLANDLRFAGTTKTTISLFPFFGATFFFLRDGVGVYNNLDAREQLL
NQLRVKCWNLVGKDKIMELIEKLKNLGGIEELIKVIDMMIEIILRKRGIPYRYNSYFYEV
KKMRSFLSNRTNTKTLIESVKIKSVYQSASLIAQDISFQLKNKRRSFHSIFAKIVKEIPK
GVEGIRICFSGRLKDAAEKAQTKCYKHRKTSCNVFNHKIDYAPVEVFTRYGILGVKVWIS
YSQKKGRRAISETYEI
>Q95869 | RT12_NICSY
MPTKNQLIRHGREEKRRTDRTRALDQCPQKQGVCPRVSTRTPKKPNSAPRKIAKVRLSNR
HDIFAHIPGEGHNSQEHSMVLIRGGRVKDSPGVKFHCIRGVKDLLGIPDRRRGRSKYGAE
KPKSI
>Q96007 | NU3M_ALLCE
MSEFSPIFIYLVMSLLVSLILLGLPFLFASNSSTYPEKLSAYECGFDPFGDARSRFDIRF
YLVSILFIIFDLEVTFFFPWAVSLNKIDLFGFWSMMAFLLILTIGFLYEWKRGALDWE
>Q96008 | RT12_ALLCE
MPTFNQLIRHGREEKRRTDRTRALDQCPQKQGVCLRVLTITPKKPNSALRKIAKVRLTNR
HDIFAYIPGEGHNSQEHSIVLVRGGRVKDLPGVKFHCIRGVKDLLGIPDRRRGRSKYGAE
KPKSK
>Q96033 | RT12_HELAN
MPTLNQLIRHGREEKRRTDRTRALDQCPQKQGVCLRVSTRTPKKPNSALRKIAKVRLSNR
HDIFAYIPGEGHNLQEHSIVLIRGGRVKDLPGVKFHCIRGVKDLLGIPDRRKGRSKYGAE
KPKSR

>Q96253 | ATP5E_ARATH
MASNAAVPFWRAAGMTYISYSNICANIVRNCLKEPHKAEALTREKVHFSLSKWADGKPQK
PVLRSDTPEV
>Q9C641 | EFGM_ARATH
MARFPTSPAPNRLLRLFSSNKRSSSPTAALLTGDFQLIRHFSAGTAARVAKDEKEPWWKE
SMDKLRNIGISAHIDSGKTTLTERVLFYTGRIHEIHEVRGRDGVGAKMDSMDLEREKGIT
IQSAATYCTWKDYKVNIIDTPGHVDFTIEVERALRVLDGAILVLCSVGGVQSQSITVDRQ
MRRYEVPRVAFINKLDRMGADPWKVLNQARAKLRHHSAAVQVPIGLEENFQGLIDLIHVK
AYFFHGSSGENVVAGDIPADMEGLVAEKRRELIETVSEVDDVLAEKFLNDEPVSASELEE
AIRRATIAQTFVPVFMGSAFKNKGVQPLLDGVVSFLPSPNEVNNYALDQNNNEERVTLTG
SPDGPLVALAFKLEEGRFGQLTYLRVYEGVIKKGDFIINVNTGKRIKVPRLVRMHSNDME
DIQEAHAGQIVAVFGIECASGDTFTDGSVKYTMTSMNVPEPVMSLAVQPVSKDSGGQFSK
ALNRFQKEDPTFRVGLDPESGQTIISGMGELHLDIYVERMRREYKVDATVGKPRVNFRET
ITQRAEFDYLHKKQSGGAGQYGRVTGYVEPLPPGSKEKFEFENMIVGQAIPSGFIPAIEK
GFKEAANSGSLIGHPVENLRIVLTDGASHAVDSSELAFKMAAIYAFRLCYTAARPVILEP
VMLVELKVPTEFQGTVAGDINKRKGIIVGNDQEGDDSVITANVPLNNMFGYSTSLRSMTQ
GKGEFTMEYKEHSAVSNEVQAQLVNAYSASKATE
>Q9FMV1 | UMP7_ARATH
MATSIARLSRRGVTSNLIRRCFAAEAALARKTELPKPQFTVSPSTDRVKWDYRGQRQIIP
LGQWLPKVAVDAYVAPNVVLAGQVTVWDGSSVWNGAVLRGDLNKITVGFCSNVQERCVVH
AAWSSPTGLPAATIIDRYVTVGAYSLLRSCTIEPECIIGQHSILMEGSLVETRSILEAGS
VVPPGRRIPSGELWGGNPARFIRTLTNEETLEIPKLAVAINHLSGDYFSEFLPYSTVYLE
VEKFKKSLGIAV
>Q9FV51 | AMP1C_ARATH
MLQKISQSISLCNGDQFKPLIYLAGAPTNFISSPLSGKKKSSSLRIKRIQQLQSTLEDRI
NPPLVCGTVSPRLSVPDHILKPLYVESSKVPEISSELQIPDSIGIVKMKKACELAARVLD
YAGTLVRPFVTTDEIDKAVHQMVIEFGAYPSPLGYGGFPKSVCTSVNECMFHGIPDSRPL
QNGDIINIDVAVYLDGYHGDTSKTFLCGDVNGSLKQLVKVTEECLEKGISVCKDGASFKQ
IGKIISEHAAKYGYNMERFIGHGVGTVLHSEPLIYLHSNYDYELEYMIEGQTFTLEPILT
IGTTEFVTWPDKWTIVTADGGPAAQFEHTILITTTGAEILTISS
>Q9LFV6 | RPOT2_ARATH
MSSAQTPLFLANQTKVFDHLIPLHKPFISSPNPVSQSFPMWRNIAKQAISRSAARLNVSS
QTRGLLVSSPESIFSKNLSFRFPVLGSPCHGKGFRCLSGITRREEFSKSERCLSGTLARG
YTSVAEEEVLSTDVEEEPEVDELLKEMKKEKKRESHRSWRMKKQDQFGMGRTKFQNLWRR
QVKIETEEWERAAAEYMELLTDMCEQKLAPNLPYVKSLFLGWFEPLRDAIAKDQELYRLG
KSKATYAHYLDQLPADKISVITMHKLMGHLMTGGDNGCVKVVHAACTVGDAIEQEIRICT
FLDKKKKGDDNEESGGVENETSMKEQDKLRKKVNELIKKQKLSAVRKILQSHDYTKPWIA
DVRAKVGSRLIELLVRTAYIQSPADQQDNDLPDVRPAFVHTFKVAKGSMNSGRKYGVIEC
DPLVRKGLEKSGRYAVMPYMPMLVPPLKWSGYDKGAYLFLTSYIMKTHGAKQQREALKSA
PKGQLQPVFEALDTLGSTKWRVNKRVLTVVDRIWSSGGCVADMVDRSDVPLPEKPDTEDE
GILKKWKWEVKSAKKVNSERHSQRCDTELKLSVARKMKDEEAFYYPHNMDFRGRAYPMPP
HLNHLGSDLCRGVLEFAEGRPMGISGLRWLKIHLANLYAGGVDKLSLDGRLAFTENHLDD
IFDSADRPLEGSRWWLQAEDPFQCLAVCISLTEALRSPSPETVLSHIPIHQDGSCNGLQH
YAALGRDTLGAEAVNLVAGEKPADVYSGIATRVLDIMRRDADRDPEVFPEALRARKLLNQ

VDRKLVKQTVMTSVYGVTYIGARDQIKRRLKERSDFGDEKEVFGAACYAAKVTLAAIDEM
FQAARAIMRWFGECAKIIASENETVRWTTPLGLPVVQPYHQMGTKLVKTSLQTLSLQHET
DQVIVRRQRTAFPPNFIHSLDGSHMMMTAVACKRAGVCFAGVHDSFWTHACDVDKLNIIL
REKFVELYSQPILENLLESFEQSFPHLDFPPLPERGDLDLKVVLDSPYFFN
>Q9LJL3 | PREP1_ARATH
MLRTVSCLASRSSSSLFFRFFRQFPRSYMSLTSSTAALRVPSRNLRRISSPSVAGRRLLL
RRGLRIPSAAVRSVNGQFSRLSVRAVATQPAPLYPDVGQDEAEKLGFEKVSEEFISECKS
KAILFKHKKTGCEVMSVSNEDENKVFGVVFRTPPKDSTGIPHILEHSVLCGSRKYPVKEP
FVELLKGSLHTFLNAFTYPDRTCYPVASTNTKDFYNLVDVYLDAVFFPKCVDDAHTFQQE
GWHYELNDPSEDISYKGVVFNEMKGVYSQPDNILGRIAQQALSPENTYGVDSGGDPKDIP
NLTFEEFKEFHRQYYHPSNARIWFYGDDDPVHRLRVLSEYLDMFEASPSPNSSKIKFQKL
FSEPVRLVEKYPAGRDGDLKKKHMLCVNWLLSEKPLDLQTQLALGFLDHLMLGTPASPLR
KILLESGLGEALVSSGLSDELLQPQFGIGLKGVSEENVQKVEELIMDTLKKLAEEGFDND
AVEASMNTIEFSLRENNTGSFPRGLSLMLQSISKWIYDMDPFEPLKYTEPLKALKTRIAE
EGSKAVFSPLIEKLILNNSHRVTIEMQPDPEKATQEEVEEKNILEKVKAAMTEEDLAELA
RATEELKLKQETPDPPEALRCVPSLNLGDIPKEPTYVPTEVGDINGVKVLRHDLFTNDII
YTEVVFDIGSLKHELLPLVPLFCQSLLEMGTKDLTFVQLNQLIGRKTGGISVYPLTSSVR
GKDEPCSKIIVRGKSMAGRADDLFNLMNCLLQEVQFTDQQRFKQFVSQSRARMENRLRGS
GHGIAAARMDAMLNIAGWMSEQMGGLSYLEFLHTLEKKVDEDWEGISSSLEEIRRSLLAR
NGCIVNMTADGKSLTNVEKSVAKFLDLLPENPSGGLVTWDGRLPLRNEAIVIPTQVNYVG
KAGNIYSTGYELDGSAYVISKHISNTWLWDRVRVSGGAYGGFCDFDSHSGVFSYLSYRDP
NLLKTLDIYDGTGDFLRGLDVDQETLTKAIIGTIGDVDSYQLPDAKGYSSLLRHLLGVTD
EERQRKREEILTTSLKDFKDFAQAIDVVRDKGVAVAVASAEDIDAANNERSNFFEVKKAL

>Q9LKA3 | MDHM2_ARATH
MFRSMIVRSASPVKQGLLRRGFASESVPDRKVVILGAAGGIGQPLSLLMKLNPLVSSLSL
YDIANTPGVAADVGHINTRSQVSGYMGDDDLGKALEGADLVIIPAGVPRKPGMTRDDLFN
INAGIVKNLSIAIAKYCPQALVNMISNPVNSTVPIAAEIFKKAGTYDEKKLFGVTTLDVV
RARTFYAGKSDVNVAEVNVPVVGGHAGITILPLFSQASPQANLSDDLIRALTKRTQDGGT
EVVEAKAGKGSATLSMAYAGALFADACLKGLNGVPNVVECSFVQSTITELPFFASKVRLG
KNGVEEVLDLGPLSDFEKEGLEALKAELKSSIEKGIKFANQ
>Q9LPS1 | HXK3_ARATH
MGKVAVAFAAVAVVAACSVAAVMVGRRMKSRRKWRTVVEILKELEDDCDTPVGRLRQVVD
AMAVEMHAGLASEGGSKLKMLLTFVDDLPTGREKGTYYALHLGGTYFRILRVLLGDQRSY
LDVQDVERHPIPSHLMNSTSEVLFNFLAFSLERFIEKEENGSDSQGVRRELAFTFSFPVK
HTSISSGVLIKWTKGFEISEMVGQDIAECLQGALNRRGLDMHVAALVNDTVGALSLGYYH
DPDTVVAVVFGTGSNACYLERTDAIIKCQGLLTTSGSMVVNMEWGNFWSSHLPRTSYDID
LDAESSNANDMGFEKMISGMYLGDIVRRVILRMSEDSDIFGPISPVLSEPYVLRTNSVSA
IHEDDTPELQEVARILKDIGVSDVPLKVRKLVVKICDVVTRRAGRLAAAGIAGILKKIGR
DGSGGITSGRSRSEIQMQKRTVVAVEGGLYMNYTMFREYMEEALVEILGEEVSQYVVVKA
MEDGSSIGSALLVASLQS
>Q9M1D3 | CISY5_ARATH
MVMQDLKSQMQEIIPEQQDRLKKLKSEQGKVPVGNITVDMVLGGMRGMTGLLWETSLLDA
DEGIRFRGMSIPECQKILPSAESGEEPLPESLLWLLLTGKVPTKEQANALSTELAHRAAV

PAIDALPSTAHPMTQFASGVMALQVQSEFQKAYEQGDISKSKYWEPTFEDALNLIARVPV
VASYVYRRMYKDGSIIPLDDSLDYGANFSHMLGFDSPQMKELMRLYVTIHSDHEGGNVSA
HAGHLVGSALSDPYLSFAAALNGLAGPLHGLANQEVLLWIKLVVEECGESISKEQLKDYV
WKTLNSGKVVPGYGHGVLRKTDPRYICQREFALKHLPDDPLFQLVSKLYEVVPPILTELG
KVKNPWPNVDAHSGVLLNYYGLTEARYYTVLFGVSRSLGICSQLIWDRALGLPLERPKSV
NMDWLDNFTRLNR
>Q9M5K2 | DLDH2_ARATH
MAMASLARRKAYFLTRNISNSPTDAFRFSFSLTRGFASSGSDDNDVVIIGGGPGGYVAAI
KAAQLGLKTTCIEKRGALGGTCLNVGCIPSKALLHSSHMYHEAKHVFANHGVKVSSVEVD
LPAMLAQKDTAVKNLTRGVEGLFKKNKVNYVKGYGKFLSPSEVSVDTIDGENVVVKGKHI
IVATGSDVKSLPGITIDEKKIVSSTGALSLTEIPKKLIVIGAGYIGLEMGSVWGRLGSEV
TVVEFAADIVPAMDGEIRKQFQRSLEKQKMKFMLKTKVVGVDSSGDGVKLIVEPAEGGEQ
TTLEADVVLVSAGRTPFTSGLDLEKIGVETDKGGRILVNERFSTNVSGVYAIGDVIPGPM
LAHKAEEDGVACVEFIAGKHGHVDYDKVPGVVYTYPEVASVGKTEEQLKKEGVSYNVGKF
PFMANSRAKAIDTAEGMVKILADKETDKILGVHIMSPNAGELIHEAVLAINYDASSEDIA
RVCHAHPTMSEAIKEAAMATYDKPIHM
>Q9M5K3 | DLDH1_ARATH
MAMASLARRKAYFLTRNLSNSPTDALRFSFSLSRGFASSGSDENDVVIIGGGPGGYVAAI
KASQLGLKTTCIEKRGALGGTCLNVGCIPSKALLHSSHMYHEAKHSFANHGIKVSSVEVD
LPAMLAQKDNAVKNLTRGIEGLFKKNKVTYVKGYGKFISPNEVSVETIDGGNTIVKGKHI
IVATGSDVKSLPGITIDEKKIVSSTGALSLSEVPKKLIVIGAGYIGLEMGSVWGRLGSEV
TVVEFAGDIVPSMDGEIRKQFQRSLEKQKMKFMLKTKVVSVDSSSDGVKLTVEPAEGGEQ
SILEADVVLVSAGRTPFTSGLDLEKIGVETDKAGRILVNDRFLSNVPGVYAIGDVIPGPM
LAHKAEEDGVACVEFIAGKHGHVDYDKVPGVVYTHPEVASVGKTEEQLKKEGVSYRVGKF
PFMANSRAKAIDNAEGLVKILADKETDKILGVHIMAPNAGELIHEAVLAINYDASSEDIA
RVCHAHPTMSEALKEAAMATYDKPIHI
>Q9MF82 | RT07_BETVU
MGGLDSEQKQLIKKLVNFHMKEGKRTKVRAIVYQTFHRLARTEGDVIKLMIDAVENIKPI
CKVEKVRVAGTIYDVPGIVARDRQQTLAIRWILEAAFKRRISYRISLEKCLFDEILDAYR
KRGISRKKRENLHGLASANRSFAHFRWW
>Q9SIB9 | ACO2M_ARATH
MYLTASSSASSSIIRAASSRSSSLFSFRSVLSPSVSSTSPSSLLARRSFGTISPAFRRWS
HSFHSKPSPFRFTSQIRAVSPVLDRLQRTFSSMASEHPFKGIFTTLPKPGGGEFGKFYSL
PALNDPRVDKLPYSIRILLESAIRNCDNFQVTKEDVEKIIDWEKTSPKQVEIPFKPARVL
LQDFTGVPAVVDLACMRDAMNKLGSDSNKINPLVPVDLVIDHSVQVDVARSENAVQANME
LEFQRNKERFAFLKWGSTAFQNMLVVPPGSGIVHQVNLEYLGRVVFNTKGLLYPDSVVGT
DSHTTMIDGLGVAGWGVGGIEAEATMLGQPMSMVLPGVVGFKLAGKMRNGVTATDLVLTV
TQMLRKHGVVGKFVEFYGNGMSGLSLADRATIANMSPEYGATMGFFPVDHVTLQYLKLTG
RSDETVAMIEAYLRANNMFVDYNEPQQDRVYSSYLELNLDDVEPCISGPKRPHDRVTLKE
MKADWHSCLDSKVGFKGFAIPKEAQEKVVNFSFDGQPAELKHGSVVIAAITSCTNTSNPS
VMLGAGLVAKKACDLGLQVKPWIKTSLAPGSGVVTKYLLKSGLQEYLNEQGFNIVGYGCT
TCIGNSGEINESVGAAITENDIVAAAVLSGNRNFEGRVHPLTRANYLASPPLVVAYALAG
TVNIDFETEPIGKGKNGKDVFLRDIWPTTEEIAEVVQSSVLPDMFRATYESITKGNPMWN
KLSVPENTLYSWDPNSTYIHEPPYFKDMTMDPPGPHNVKDAYCLLNFGDSITTDHISPAG

NIQKDSPAAKFLMERGVDRKDFNSYGSRRGNDEIMARGTFANIRIVNKLMNGEVGPKTVH
IPSGEKLSVFDAAMRYKSSGEDTIILAGAEYGSGSSRDWAAKGPMLQGVKAVIAKSFERI
HRSNLVGMGIIPLCFKSGEDADTLGLTGHERYTIHLPTDISEIRPGQDVTVTTDNGKSFT
CTVRFDTEVELAYFNHGGILPYVIRNLSKQ
>Q9SMX3 | VDAC2_ARATH
MVKGPGLYTEIGKKARDLLYRDYQGDQKFSVTTYSSTGVAITTTGTNKGSLFLGDVATQV
KNNNFTADVKVSTDSSLLTTLTFDEPAPGLKVIVQAKLPDHKSGKAEVQYFHDYAGISTS
VGFTATPIVNFSGVVGTNGLSLGTDVAYNTESGNFKHFNAGFNFTKDDLTASLILNDKGE
KLNASYYQIVSPSTVVGAEISHNFTTKENAITVGTQHALDPLTTVKARVNNAGVANALIQ
HEWRPKSFFTVSGEVDSKAIDKSAKVGIALALKP
>Q9SRH5 | VDAC1_ARATH
MVKGPGLYTEIGKKARDLLYKDHNSDQKFSITTFSPAGVAITSTGTKKGDLLLGDVAFQS
RRKNITTDLKVCTDSTFLITATVDEAAPGLRSIFSFKVPDQNSGKVELQYLHEYAGISTS
MGLTQNPTVNFSGVIGSNVLAVGTDVSFDTKSGNFTKINAGLSFTKEDLIASLTVNDKGD
LLNASYYHIVNPLFNTAVGAEVSHKLSSKDSTITVGTQHSLDPLTSVKARVNSAGIASAL
IQHEWKPKSFFTISGEVDTKSIDKSAKVGLALALKP
>Q9SVM8 | GRP2_ARATH
MAFCNKLGGLLRQNISSNGNVPVTSMLGSLRLMSTKLFIGGLSWGTDDASLRDAFAHFGD
VVDAKVIVDRETGRSRGFGFVNFNDEGAATAAISEMDGKELNGRHIRVNPANDRPSAPRA
YGGGGGYSGGGGGYGGGGGGYGGGGGGYGGGGDGGGGF
>Q9SZJ5 | GLYM_ARATH
MAMAMALRRLSSSIDKPIRPLIRSTSCYMSSLPSEAVDEKERSRVTWPKQLNAPLEEVDP
EIADIIEHEKARQWKGLELIPSENFTSVSVMQAVGSVMTNKYSEGYPGARYYGGNEYIDM
AETLCQKRALEAFRLDPEKWGVNVQPLSGSPANFHVYTALLKPHERIMALDLPHGGHLSH
GYQTDTKKISAVSIFFETMPYRLDESTGYIDYDQMEKSATLFRPKLIVAGASAYARLYDY
ARIRKVCNKQKAVMLADMAHISGLVAANVIPSPFDYADVVTTTTHKSLRGPRGAMIFFRK
GVKEINKQGKEVLYDFEDKINQAVFPGLQGGPHNHTITGLAVALKQATTSEYKAYQEQVL
SNSAKFAQTLMERGYELVSGGTDNHLVLVNLKPKGIDGSRVEKVLEAVHIASNKNTVPGD
VSAMVPGGIRMGTPALTSRGFVEEDFAKVAEYFDKAVTIALKVKSEAQGTKLKDFVSAME
SSSTIQSEIAKLRHEVEEFAKQFPTIGFEKETMKYKN
>Q9TC96 | NDUS2_NEPOL
MAIEHASSIKKVKNFTLNFGPQHPAAHGVLRLVLELNGEVVARADPHIGLLHRGTEKLIE
YKTYTQALPYFDRLDYVSMMCQEHAYSLAVEKLLHCEVPERAQYIRVLFSEITRILNHLL
ALTTHAMDVGALTPFLWAFEEREKLIEFYERVSGSRMHAAYIRPGGVACDLPANLCEDIY
LFCQQFASRIDEMEEMLTNNRIWKQRLVDIGIVTAENAFAWGFSGVLLRGSGVAWDLRKT
QPYDVYNRMIFDVPVGTQGDCYDRYLCRVEEMRQSIHIIMQCLNQLPKGMIKADDKKITP
PSRSQMKQSMESLIHHFKLFTEGYTVPNSETYTSVEAPKGEFGVYLVSNGTNRPYRCKIR
APGFLHLQGLDMMSKNHMLADVVTIIGTQDIVFGEVDR
>Q9XGY5 | TIM13_ORYSJ
MDSFSSSSGSPPNTEALMDQIKAQLAQAYAQEFLETVGNKCFAKCVTKPGSSLSGSESSC
ISRCVDRYIEATGIVSRALFSSTR
>Q9ZP06 | MDHM1_ARATH
MFRSMLVRSSASAKQAVIRRSFSSGSVPERKVAILGAAGGIGQPLALLMKLNPLVSSLSL
YDIANTPGVAADVGHINTRSEVVGYMGDDNLAKALEGADLVIIPAGVPRKPGMTRDDLFN

INAGIVKNLCTAIAKYCPHALINMISNPVNSTVPIAAEIFKKAGMYDEKKLFGVTTLDVV
RARTFYAGKANVPVAEVNVPVIGGHAGVTILPLFSQATPQANLSSDILTALTKRTQDGGT
EVVEAKAGKGSATLSMAYAGALFADACLKGLNGVPDVIECSYVQSTITELPFFASKVRLG
KNGVEEVLDLGPLSDFEKEGLEALKPELKSSIEKGVKFANQ
>Q9ZPX5 | DHSA2_ARATH
MWRCLRVASSSRRSESNGAFITSQLSRFFSAPPSAGDKSSYTIVDHTYDAVVVGAGGAGL
RAAIGLSEHGFNTACITKLFPTRSHTVAAQGGINAALGNMSVDDWRWHMYDTVKGSDWLG
DQDAIQYMCREAPKAVIELENYGLPFSRTEDGKIYQRAFGGQSLEFGIGGQAYRCACAAD
RTGHALLHTLYGQAMKHNTQFFVEYFALDLIMNSDGTCQGVIALNMEDGTLHRFHAGSTI
LATGGYGRAYFSATSAHTCTGDGNAMVARAGLPLQDLEFVQFHPTGIYGAGCLITEGARG
EGGILRNSEGEKFMDRYAPTARDLASRDVVSRSMTMEIRQGRGAGPMKDYLYLYLNHLPP
EVLKERLPGISETAAIFAGVDVTREPIPVLPTVHYNMGGIPTNYHGEVITLRGDDPDAVV
PGLMAAGEAACASVHGANRLGANSLLDIVVFGRACANRVAEIQKPGEKLKPLEKDAGEKS
IEWLDRIRNSNGSLPTSKIRLNMQRVMQNNAAVFRTQETLEEGCDLIDKTWDSFGDVKVT
DRSMIWNSDLIETMELENLLVNACITMHSAEARKESRGAHAREDFTKRDDANWMKHTLGY
WEEGNVKLEYRPVHMKTLDDEVDTFPPKPRVY
>Q9ZT91 | EFTM_ARATH
MASVVLRNPSSKRLVPFSSQIYSRCGASVTSSYSISHSIGGDDLSSSTFGTSSFWRSMAT
FTRNKPHVNVGTIGHVDHGKTTLTAAITKVLAEEGKAKAIAFDEIDKAPEEKKRGITIAT
AHVEYETAKRHYAHVDCPGHADYVKNMITGAAQMDGGILVVSGPDGPMPQTKEHILLARQ
VGVPSLVCFLNKVDVVDDPELLELVEMELRELLSFYKFPGDDIPIIRGSALSALQGTNDE
IGRQAILKLMDAVDEYIPDPVRVLDKPFLMPIEDVFSIQGRGTVATGRIEQGVIKVGEEV
EILGLREGGVPLKSTVTGVEMFKKILDNGQAGDNVGLLLRGLKREDIQRGMVIAKPGSCK
TYKKFEAEIYVLTKDEGGRHTAFFSNYRPQFYLRTADITGKVELPENVKMVMPGDNVTAV
FELIMPVPLETGQRFALREGGRTVGAGVVSKVMT
