# Supplementary material for: Plant-mPLoc: A Top-Down Strategy to Augment the Power for Predicting Plant Protein Subcellular Localization
Source: PLoS One. 2010 Jun 28;5(6):e11335. doi: 10.1371/journal.pone.0011335 (PMC2893129; doi:10.1371/journal.pone.0011335)
Supplement: Table S3 — List of the results predicted by TargetP (Emanuelsson et al. J. Mol. Biol. 2000, 300: 1005–1016) and Plant-mPLoc on the 1,775 independent proteins in the Table S2, and their experimental subcellular locations as annotated in Swiss-Prot databank (version 55.3 released on 29-Apr-2008). Note for TargetP outputs, “C” means “Chloroplast”, “M” means “Mitochondrion”, “S” means “Secretory pathway”, and “_” means “Any other location”. (0.41 MB PDF) [file pone.0011335.s003.pdf]

Table S3. List of the results predicted by TargetP (Emanuelsson et al. J. Mol. Biol. 2000, 300: 1005-1016) and Plant-mPLoc on the 1,775 independent proteins in the **Table S2**, and their experimental subcellular locations as annotated in Swiss-Prot databank (version 55.3 released on 29-Apr-2008). Note for TargetP outputs, "C" means "Chloroplast", "M" means "Mitochondrion", "S" means "Secretory pathway", and "_" means "Any other location".

Protein accession number	TargetP	Plant-mPLoc	Experimental result annotated in Swiss-Prot database
A0A325	_	Chloroplast.	Chloroplast.
A0A327	_	Chloroplast.	Chloroplast.
A0A333	M	Chloroplast.	Chloroplast.
A0A337	M	Chloroplast.	Chloroplast.
A0A356	_	Chloroplast. Plastid.	Chloroplast.
A0A357	M	Chloroplast.	Chloroplast.
A0A371	_	Chloroplast.	Chloroplast.
A0A374	M	Chloroplast.	Chloroplast.
A0A375	_	Chloroplast.	Chloroplast.
A0A376	_	Chloroplast.	Chloroplast.
A0A381	_	Mitochondrion. Plastid.	Chloroplast.
A0A385	S	Chloroplast.	Chloroplast.
A0A392	_	Chloroplast.	Chloroplast.
A0ZZ25	_	Chloroplast.	Chloroplast.
A0ZZ26	M	Chloroplast. Nucleus.	Chloroplast.
A0ZZ27	_	Chloroplast.	Chloroplast.
A0ZZ33	C	Chloroplast.	Chloroplast.
A0ZZ43	_	Chloroplast.	Chloroplast.
A0ZZ58	M	Chloroplast.	Chloroplast.

A0ZZ70	_	Chloroplast.	Chloroplast.
A0ZZ74	_	Chloroplast.	Chloroplast.
A0ZZ75	_	Chloroplast.	Chloroplast.
A0ZZ77	_	Chloroplast.	Chloroplast.
A0ZZ83	M	Chloroplast.	Chloroplast.
A0ZZ85	S	Chloroplast.	Chloroplast.
A0ZZ92	_	Chloroplast.	Chloroplast.
A1E9M3	C	Chloroplast. Plastid.	Chloroplast.
A1E9N8	M	Chloroplast. Plastid.	Chloroplast.
A1E9Q2	C	Chloroplast.	Chloroplast.
A1E9R5	–	Chloroplast. Nucleus. Plastid.	Chloroplast.
A1E9S6	M	Chloroplast.	Chloroplast.
A1E9T2	_	Chloroplast.	Chloroplast.
A1E9T3	_	Chloroplast.	Chloroplast.
A1E9U5	_	Chloroplast. Plastid.	Chloroplast.
A1E9U6	C	Chloroplast. Plastid.	Chloroplast.
A1E9U7	M	Chloroplast.	Chloroplast.
A1E9W0	_	Chloroplast. Plastid.	Chloroplast.
A1E9W2	M	Mitochondrion. Plastid.	Chloroplast.
A1E9W3	M	Chloroplast.	Chloroplast.
A1E9W6	_	Chloroplast.	Chloroplast.
A1E9X2	M	Chloroplast. Plastid.	Chloroplast.
A1E9X4	S	Chloroplast.	Chloroplast.
A1E9Y7	_	Chloroplast.	Chloroplast.
A1E9Z0	M	Chloroplast. Plastid.	Chloroplast.

A1EA17	_	Chloroplast.	Chloroplast.
A1EA30	C	Chloroplast. Plastid.	Chloroplast.
A1EA31	M	Chloroplast.	Chloroplast.
A1EA41	C	Chloroplast. Plastid.	Chloroplast.
A1EA43	_	Chloroplast. Plastid.	Chloroplast.
A1EA45	_	Chloroplast.	Chloroplast.
A1EA50	C	Chloroplast.	Chloroplast.
A1XFT4	C	Chloroplast.	Chloroplast.
A1XFZ7	C	Chloroplast.	Chloroplast.
A2CI70	_	Chloroplast.	Chloroplast.
A2T365	_	Chloroplast.	Chloroplast.
A2T381	M	Chloroplast. Plastid.	Chloroplast.
A2XDA1	C	Chloroplast.	Chloroplast.
A2Y205	C	Chloroplast.	Chloroplast.
A2YQD9	M	Chloroplast. Cytoplasm.	Chloroplast.
A4GG84	_	Chloroplast.	Chloroplast.
A4GG95	M	Chloroplast.	Chloroplast.
A4GGD7	M	Chloroplast.	Chloroplast.
A4GGE4	M	Chloroplast.	Chloroplast.
A4GYQ0	_	Chloroplast.	Chloroplast.
A4GYR2	M	Chloroplast.	Chloroplast.
A4GYR8	_	Chloroplast.	Chloroplast.
A4GYT5	C	Chloroplast.	Chloroplast.
A4GYT6	_	Chloroplast.	Chloroplast.
A4GYU3	M	Chloroplast. Plastid.	Chloroplast.
A4GYU6	_	Chloroplast.	Chloroplast.

A4GYU9	_	Chloroplast.	Chloroplast.
A4GYV2	C	Chloroplast.	Chloroplast.
A4QJA7	_	Chloroplast.	Chloroplast.
A4QJC3	_	Chloroplast.	Chloroplast.
A4QJE0	_	Chloroplast.	Chloroplast.
A4QJE7	M	Chloroplast.	Chloroplast.
A4QJF0	_	Chloroplast.	Chloroplast.
A4QJF3	M	Chloroplast.	Chloroplast.
A4QJF6	C	Chloroplast.	Chloroplast.
A4QJJ1	_	Chloroplast.	Chloroplast.
A4QJK1	M	Chloroplast.	Chloroplast.
A4QJM3	C	Chloroplast.	Chloroplast.
A4QJM4	_	Chloroplast.	Chloroplast.
A4QJN7	M	Chloroplast.	Chloroplast.
A4QJV5	C	Chloroplast.	Chloroplast.
A4QJV6	_	Chloroplast.	Chloroplast.
A4QJW6	_	Chloroplast.	Chloroplast.
A4QJY0	_	Chloroplast.	Chloroplast.
A4QK51	C	Chloroplast.	Chloroplast.
A4QK53	_	Chloroplast.	Chloroplast.
A4QK68	_	Chloroplast.	Chloroplast.
A4QKD0	_	Chloroplast.	Chloroplast.
A4QKD7	M	Chloroplast.	Chloroplast.
A4QKD8	C	Chloroplast.	Chloroplast.
A4QKF5	_	Chloroplast.	Chloroplast.
A4QKJ4	M	Chloroplast.	Chloroplast.

A4QKK0	_	Chloroplast.	Chloroplast.
A4QKL7	_	Chloroplast.	Chloroplast.
A4QKM5	C	Chloroplast.	Chloroplast.
A4QKN0	M	Chloroplast.	Chloroplast.
A4QKN3	_	Chloroplast.	Chloroplast.
A4QKP3	M	Chloroplast.	Chloroplast.
A4QKT3	M	Chloroplast.	Chloroplast.
A4QKT9	_	Chloroplast.	Chloroplast.
A4QKV5	C	Chloroplast.	Chloroplast.
A4QKW3	M	Chloroplast.	Chloroplast.
A4QL21	M	Chloroplast.	Chloroplast.
A4QL44	_	Chloroplast.	Chloroplast.
A4QL57	M	Chloroplast.	Chloroplast.
A4QLB4	_	Chloroplast.	Chloroplast.
A4QLD0	C	Chloroplast.	Chloroplast.
A4QLD1	_	Chloroplast.	Chloroplast.
A4QLD9	C	Chloroplast.	Chloroplast.
A4QLE1	_	Chloroplast.	Chloroplast.
A4QLE4	M	Chloroplast.	Chloroplast.
A4QLF7	_	Chloroplast.	Chloroplast.
A4QLI6	_	Chloroplast.	Chloroplast.
A4QLJ6	M	Chloroplast.	Chloroplast.
A4QLK2	_	Chloroplast.	Chloroplast.
A4QLL8	C	Chloroplast.	Chloroplast.
A4QLM6	M	Chloroplast.	Chloroplast.
A4QLM9	_	Chloroplast.	Chloroplast.

A4QLN2	M	Chloroplast.	Chloroplast.
A4QLP5	_	Chloroplast.	Chloroplast.
A4QLS5	_	Chloroplast.	Chloroplast.
A4QLU1	_	Chloroplast.	Chloroplast.
A4QLV7	C	Chloroplast.	Chloroplast.
A4QLV8	_	Chloroplast.	Chloroplast.
A4QLW6	C	Chloroplast.	Chloroplast.
A6BM50	_	Chloroplast.	Chloroplast.
A6H5H5	M	Chloroplast.	Chloroplast.
A6H5K6	_	Chloroplast.	Chloroplast.
A6H5L3	_	Chloroplast. Plastid.	Chloroplast.
A6H5L7	_	Chloroplast.	Chloroplast.
A6H5M3	C	Chloroplast.	Chloroplast.
A6H5Q1	_	Chloroplast.	Chloroplast.
A6MM38	M	Chloroplast.	Chloroplast.
A6MM69	C	Chloroplast.	Chloroplast.
A6MM72	_	Chloroplast.	Chloroplast.
A6MMF4	_	Chloroplast. Plastid.	Chloroplast.
A6MMF8	_	Chloroplast.	Chloroplast.
A6MMG1	M	Chloroplast.	Chloroplast.
A6MMI6	_	Chloroplast.	Chloroplast.
A6MMJ9	_	Chloroplast.	Chloroplast.
A6MMK9	M	Chloroplast.	Chloroplast.
A6MMN2	_	Chloroplast.	Chloroplast.
A6MMN9	_	Chloroplast. Plastid.	Chloroplast.
A6MMP0	C	Chloroplast.	Chloroplast.

A6MMS3	C	Chloroplast.	Chloroplast.
A6MMT6	_	Chloroplast.	Chloroplast.
A6MMY0	_	Chloroplast.	Chloroplast.
A6MMY3	M	Chloroplast.	Chloroplast.
A6YGA4	_	Chloroplast.	Chloroplast.
A6YGC4	_	Chloroplast.	Chloroplast.
A6YGD0	_	Mitochondrion. Plastid.	Chloroplast.
A6YGD3	M	Chloroplast.	Chloroplast.
A6YGD9	S	Chloroplast.	Chloroplast.
A7Y3B9	_	Chloroplast.	Chloroplast.
A7Y3I9	M	Chloroplast.	Chloroplast.
A8SEA5	M	Chloroplast.	Chloroplast.
A8SED5	_	Chloroplast. Plastid.	Chloroplast.
A8SED6	C	Chloroplast.	Chloroplast.
A8SEE2	M	Chloroplast.	Chloroplast.
A8Y9B8	_	Chloroplast. Plastid.	Chloroplast.
A8Y9B9	C	Chloroplast. Plastid.	Chloroplast.
A8Y9C2	_	Chloroplast. Plastid.	Chloroplast.
A8Y9C5	M	Chloroplast. Plastid.	Chloroplast.
A8Y9G0	_	Chloroplast. Plastid.	Chloroplast.
A9LYH7	_	Chloroplast.	Chloroplast.
O03060	S	Chloroplast.	Chloroplast.
O04683	C	Chloroplast. Cytoplasm.	Chloroplast.
O04997	C	Chloroplast.	Chloroplast.
O19816	M	Chloroplast.	Chloroplast.

O20163	_	Chloroplast.	Chloroplast.
O22077	C	Chloroplast.	Chloroplast.
O22340	C	Chloroplast.	Chloroplast.
O22573	C	Chloroplast.	Chloroplast.
O24310	C	Chloroplast.	Chloroplast.
O24600	C	Chloroplast. Mitochondrion. Plastid.	Chloroplast.
O24634	C	Chloroplast.	Chloroplast.
O47039	_	Chloroplast.	Chloroplast.
O47041	_	Chloroplast.	Chloroplast.
O47125	_	Chloroplast.	Chloroplast.
O47129	_	Chloroplast.	Chloroplast.
O47170	_	Chloroplast.	Chloroplast.
O50039	C	Chloroplast.	Chloroplast.
O62951	M	Chloroplast.	Chloroplast.
O62953	_	Chloroplast.	Chloroplast.
O62961	_	Chloroplast.	Chloroplast.
O62964	_	Chloroplast.	Chloroplast.
O62992	_	Chloroplast.	Chloroplast.
O63070	_	Chloroplast.	Chloroplast.
O64416	C	Chloroplast.	Chloroplast.
O65037	C	Chloroplast.	Chloroplast.
O65349	C	Chloroplast.	Chloroplast.
O78248	_	Chloroplast.	Chloroplast.
O78253	_	Chloroplast.	Chloroplast.
O78310	C	Chloroplast.	Chloroplast.

O80363	C	Chloroplast.	Chloroplast.
O81192	C	Chloroplast.	Chloroplast.
O81193	C	Chloroplast.	Chloroplast.
O81220	C	Chloroplast.	Chloroplast.
O98451	_	Chloroplast.	Chloroplast.
O98455	M	Chloroplast.	Chloroplast.
O98456	M	Mitochondrion. Plastid.	Chloroplast.
O98458	_	Chloroplast.	Chloroplast.
O98461	_	Chloroplast.	Chloroplast.
O98634	M	Chloroplast.	Chloroplast.
O98636	_	Chloroplast.	Chloroplast.
O98691	_	Chloroplast. Plastid.	Chloroplast.
P00225	_	Chloroplast.	Chloroplast.
P00230	_	Chloroplast.	Chloroplast.
P00231	_	Chloroplast. Cytoplasm.	Chloroplast.
P00232	_	Chloroplast.	Chloroplast.
P00233	_	Chloroplast.	Chloroplast.
P00234	_	Chloroplast. Cytoplasm.	Chloroplast.
P00235	_	Chloroplast. Cytoplasm.	Chloroplast.
P00238	_	Chloroplast.	Chloroplast.
P00240	_	Chloroplast.	Chloroplast.
P00870	_	Chloroplast.	Chloroplast.
P00873	C	Chloroplast.	Chloroplast.
P04045	C	Chloroplast.	Chloroplast.

P04713	C	Chloroplast.	Chloroplast.
P05346	C	Chloroplast.	Chloroplast.
P05698	_	Chloroplast. Plastid.	Chloroplast.
P05727	M	Mitochondrion. Plastid.	Chloroplast.
P06260	S	Chloroplast. Plastid.	Chloroplast.
P06263	S	Chloroplast.	Chloroplast.
P06269	M	Chloroplast. Plastid.	Chloroplast.
P06271	_	Chloroplast.	Chloroplast.
P06272	_	Chloroplast.	Chloroplast.
P06273	_	Chloroplast. Nucleus.	Chloroplast.
P06356	M	Chloroplast.	Chloroplast.
P06357	M	Chloroplast.	Chloroplast.
P06359	M	Chloroplast.	Chloroplast.
P06360	_	Mitochondrion. Plastid.	Chloroplast.
P06362	_	Chloroplast.	Chloroplast.
P06373	_	Chloroplast.	Chloroplast.
P06378	M	Chloroplast.	Chloroplast.
P06382	_	Chloroplast.	Chloroplast.
P06384	M	Chloroplast.	Chloroplast.
P06386	M	Chloroplast.	Chloroplast.
P06388	_	Chloroplast.	Chloroplast.
P06506	C	Chloroplast.	Chloroplast.
P06586	M	Chloroplast.	Chloroplast.
P07088	C	Chloroplast.	Chloroplast.

P07089	C	Chloroplast.	Chloroplast.
P07689	C	Chloroplast.	Chloroplast.
P07838	_	Chloroplast. Cytoplasm.	Chloroplast.
P07839	M	Chloroplast. Cytoplasm.	Chloroplast.
P08211	_	Chloroplast.	Chloroplast.
P08241	_	Chloroplast.	Chloroplast.
P08242	_	Chloroplast.	Chloroplast.
P08527	C	Chloroplast. Plastid.	Chloroplast.
P08528	M	Mitochondrion. Plastid.	Chloroplast.
P08529	_	Chloroplast.	Chloroplast.
P08530	_	Chloroplast. Plastid.	Chloroplast.
P08698	_	Chloroplast.	Chloroplast.
P08705	C	Chloroplast.	Chloroplast.
P08706	C	Chloroplast.	Chloroplast.
P08817	C	Chloroplast.	Chloroplast.
P08974	_	Chloroplast.	Chloroplast.
P09364	_	Chloroplast.	Chloroplast.
P09594	_	Chloroplast.	Chloroplast.
P09597	_	Chloroplast.	Chloroplast.
P09735	_	Chloroplast. Cytoplasm.	Chloroplast.
P09842	C	Chloroplast.	Chloroplast.
P0C2Y3	M	Plastid.	Chloroplast.
P0C305	M	Chloroplast. Plastid.	Chloroplast.
P0C312	_	Chloroplast.	Chloroplast.

P0C313	_	Chloroplast.	Chloroplast.
P0C321	S	Chloroplast. Plastid.	Chloroplast.
P0C322	S	Chloroplast. Plastid.	Chloroplast.
P0C324	S	Chloroplast. Plastid.	Chloroplast.
P0C325	S	Chloroplast. Plastid.	Chloroplast.
P0C329	S	Chloroplast. Plastid.	Chloroplast.
P0C331	S	Chloroplast. Plastid.	Chloroplast.
P0C333	S	Chloroplast. Plastid.	Chloroplast.
P0C334	S	Chloroplast. Plastid.	Chloroplast.
P0C335	_	Chloroplast. Plastid.	Chloroplast.
P0C336	_	Chloroplast. Plastid.	Chloroplast.
P0C338	_	Chloroplast. Plastid.	Chloroplast.
P0C341	_	Chloroplast. Plastid.	Chloroplast.
P0C375	S	Chloroplast. Plastid.	Chloroplast.
P0C379	_	Chloroplast. Plastid.	Chloroplast.
P0C381	S	Chloroplast.	Chloroplast.
P0C408	S	Chloroplast. Plastid.	Chloroplast.
P0C439	_	Chloroplast.	Chloroplast.
P0C440	_	Chloroplast.	Chloroplast.
P0C449	_	Chloroplast.	Chloroplast.
P0C453	M	Chloroplast. Plastid.	Chloroplast.
P0C456	M	Chloroplast. Plastid.	Chloroplast.
P0C457	M	Chloroplast. Plastid.	Chloroplast.
P0C460	C	Chloroplast.	Chloroplast.
P0C463	M	Chloroplast. Plastid.	Chloroplast.
P0C473	M	Chloroplast. Plastid.	Chloroplast.

P0C474	M	Chloroplast. Plastid.	Chloroplast.
P0C475	M	Chloroplast. Plastid.	Chloroplast.
P0C477	M	Chloroplast. Plastid.	Chloroplast.
P0C478	_	Chloroplast.	Chloroplast.
P0C484	M	Chloroplast. Plastid.	Chloroplast.
P0C485	M	Chloroplast. Plastid.	Chloroplast.
P0C487	M	Chloroplast.	Chloroplast.
P0C488	M	Chloroplast.	Chloroplast.
P0C491	_	Mitochondrion. Plastid.	Chloroplast.
P0C492	_	Chloroplast. Plastid.	Chloroplast.
P0C498	_	Chloroplast. Plastid.	Chloroplast.
P0C500	_	Chloroplast. Plastid.	Chloroplast.
P0C501	_	Chloroplast. Plastid.	Chloroplast.
P0C502	_	Chloroplast. Plastid.	Chloroplast.
P0C508	_	Chloroplast. Plastid.	Chloroplast.
P10797	C	Chloroplast.	Chloroplast.
P10798	C	Chloroplast.	Chloroplast.
P11422	_	Chloroplast.	Chloroplast.
P11646	S	Chloroplast. Plastid.	Chloroplast.
P11705	_	Chloroplast. Nucleus.	Chloroplast.
P11893	C	Chloroplast.	Chloroplast.
P11964	C	Chloroplast.	Chloroplast.
P12116	M	Chloroplast. Nucleus.	Chloroplast.
P12136	M	Chloroplast.	Chloroplast.

P12139	M	Chloroplast.	Chloroplast.
P12149	C	Chloroplast.	Chloroplast.
P12163	S	Chloroplast. Plastid.	Chloroplast.
P12174	_	Chloroplast.	Chloroplast.
P12196	C	Chloroplast.	Chloroplast.
P12199	M	Chloroplast. Plastid.	Chloroplast.
P12210	_	Chloroplast.	Chloroplast.
P12216	S	Chloroplast. Plastid.	Chloroplast.
P12217	_	Chloroplast.	Chloroplast.
P12299	C	Chloroplast.	Chloroplast.
P12468	C	Chloroplast.	Chloroplast.
P13788	M	Chloroplast.	Chloroplast.
P14149	C	Mitochondrion. Plastid.	Chloroplast.
P15102	C	Chloroplast. Mitochondrion.	Chloroplast.
P16025	_	Chloroplast. Plastid.	Chloroplast.
P16032	C	Chloroplast.	Chloroplast.
P16037	_	Chloroplast.	Chloroplast.
P16131	_	Chloroplast.	Chloroplast.
P16132	C	Chloroplast.	Chloroplast.
P16134	_	Chloroplast.	Chloroplast.
P16306	_	Chloroplast.	Chloroplast.
P17353	M	Mitochondrion. Plastid.	Chloroplast.
P17537	M	Chloroplast.	Chloroplast.
P17652	M	Chloroplast.	Chloroplast.

P17673	C	Chloroplast.	Chloroplast.
P17688	C	Chloroplast.	Chloroplast.
P17703	_	Chloroplast.	Chloroplast.
P17933	_	Chloroplast.	Chloroplast.
P18566	C	Chloroplast.	Chloroplast.
P18905	M	Cell wall. Plastid.	Chloroplast.
P19044	S	Chloroplast. Plastid.	Chloroplast.
P19683	C	Chloroplast.	Chloroplast.
P19824	C	Chloroplast.	Chloroplast.
P21357	C	Chloroplast.	Chloroplast.
P22243	M	Chloroplast.	Chloroplast.
P22337	M	Chloroplast.	Chloroplast.
P22433	C	Chloroplast.	Chloroplast.
P23981	C	Chloroplast.	Chloroplast.
P24064	_	Chloroplast.	Chloroplast.
P24674	_	Chloroplast.	Chloroplast.
P24676	_	Chloroplast.	Chloroplast.
P24677	_	Chloroplast.	Chloroplast.
P24678	_	Chloroplast.	Chloroplast.
P24679	_	Chloroplast.	Chloroplast.
P24929	C	Chloroplast.	Chloroplast.
P25079	_	Chloroplast.	Chloroplast.
P25459	_	Chloroplast. Plastid.	Chloroplast.
P25827	_	Chloroplast.	Chloroplast.
P25828	_	Chloroplast.	Chloroplast.
P25839	_	Chloroplast.	Chloroplast.

P25875	M	Chloroplast. Plastid.	Chloroplast.
P26180	_	Chloroplast.	Chloroplast.
P26289	S	Chloroplast. Plastid.	Chloroplast.
P26573	C	Chloroplast.	Chloroplast.
P26574	C	Chloroplast.	Chloroplast.
P26577	C	Chloroplast.	Chloroplast.
P26667	C	Chloroplast.	Chloroplast.
P26959	_	Chloroplast.	Chloroplast.
P26960	_	Chloroplast.	Chloroplast.
P26962	_	Chloroplast.	Chloroplast.
P26964	_	Chloroplast.	Chloroplast.
P26985	C	Chloroplast.	Chloroplast.
P27064	_	Chloroplast.	Chloroplast.
P27065	_	Chloroplast.	Chloroplast.
P27107	_	Chloroplast.	Chloroplast.
P27723	M	Chloroplast. Plastid.	Chloroplast.
P27789	M	Chloroplast. Cytoplasm.	Chloroplast.
P27818	C	Chloroplast.	Chloroplast.
P28252	_	Chloroplast.	Chloroplast.
P28258	_	Chloroplast.	Chloroplast.
P28399	_	Chloroplast.	Chloroplast.
P28441	_	Chloroplast.	Chloroplast.
P28459	_	Chloroplast.	Chloroplast.
P28553	C	Chloroplast.	Chloroplast.
P28644	_	Chloroplast.	Chloroplast.
P28805	_	Chloroplast. Plastid.	Chloroplast.

P29036	C	Chloroplast.	Chloroplast.
P29108	C	Chloroplast.	Chloroplast.
P29684	C	Chloroplast.	Chloroplast.
P30523	_	Chloroplast.	Chloroplast.
P30829	_	Chloroplast.	Chloroplast.
P30830	_	Chloroplast.	Chloroplast.
P31162	_	Chloroplast.	Chloroplast.
P31163	C	Chloroplast.	Chloroplast.
P31593	C	Chloroplast. Cytoplasm.	Chloroplast.
P32087	M	Chloroplast.	Chloroplast.
P32088	_	Chloroplast.	Chloroplast.
P32976	_	Chloroplast.	Chloroplast.
P34915	_	Chloroplast.	Chloroplast.
P36476	_	Chloroplast.	Chloroplast.
P36493	M	Chloroplast.	Chloroplast.
P37215	C	Chloroplast.	Chloroplast.
P37216	C	Chloroplast.	Chloroplast.
P38550	_	Chloroplast.	Chloroplast.
P41096	C	Chloroplast.	Chloroplast.
P41606	M	Chloroplast. Plastid.	Chloroplast.
P41609	_	Chloroplast.	Chloroplast.
P41621	_	Chloroplast.	Chloroplast.
P41630	M	Chloroplast.	Chloroplast.
P41641	M	Chloroplast.	Chloroplast.
P41645	_	Chloroplast.	Chloroplast.
P41646	_	Chloroplast.	Chloroplast.

P41648	_	Chloroplast.	Chloroplast.
P41651	M	Chloroplast.	Chloroplast.
P41652	_	Mitochondrion. Plastid.	Chloroplast.
P41758	M	Chloroplast.	Chloroplast.
P42044	C	Chloroplast. Mitochondrion.	Chloroplast.
P42045	C	Chloroplast. Mitochondrion.	Chloroplast.
P48267	M	Mitochondrion. Plastid.	Chloroplast.
P48686	_	Chloroplast.	Chloroplast.
P48690	_	Chloroplast.	Chloroplast.
P48702	_	Chloroplast.	Chloroplast.
P48713	_	Chloroplast.	Chloroplast.
P48714	_	Chloroplast.	Chloroplast.
P48719	_	Chloroplast.	Chloroplast.
P49086	C	Chloroplast.	Chloroplast.
P49131	C	Chloroplast.	Chloroplast.
P49132	C	Chloroplast.	Chloroplast.
P49158	_	Chloroplast.	Chloroplast.
P49169	_	Chloroplast.	Chloroplast.
P49170	C	Chloroplast. Plastid.	Chloroplast.
P49244	C	Chloroplast.	Chloroplast.
P49245	C	Chloroplast.	Chloroplast.
P50362	C	Chloroplast.	Chloroplast.
P50371	_	Chloroplast.	Chloroplast.
P52422	C	Chloroplast.	Chloroplast.

P52733	_	Chloroplast. Nucleus.	Chloroplast.
P52762	C	Chloroplast.	Chloroplast.
P52769	_	Chloroplast.	Chloroplast.
P52771	_	Chloroplast.	Chloroplast.
P52772	_	Chloroplast.	Chloroplast.
P55229	C	Chloroplast.	Chloroplast.
P55231	_	Chloroplast.	Chloroplast.
P55238	C	Chloroplast.	Chloroplast.
P55243	_	Chloroplast.	Chloroplast.
P56293	_	Chloroplast.	Chloroplast.
P56303	_	Chloroplast.	Chloroplast.
P56315	S	Chloroplast.	Chloroplast.
P56317	_	Mitochondrion.	Chloroplast.
P56351	_	Mitochondrion. Plastid.	Chloroplast.
P56353	_	Chloroplast.	Chloroplast.
P56355	M	Mitochondrion. Plastid.	Chloroplast.
P56357	M	Chloroplast.	Chloroplast.
P56358	_	Chloroplast.	Chloroplast.
P56359	_	Chloroplast.	Chloroplast.
P56361	_	Chloroplast.	Chloroplast.
P56363	_	Chloroplast.	Chloroplast.
P56367	_	Chloroplast.	Chloroplast.
P56751	S	Chloroplast. Plastid.	Chloroplast.
P56753	_	Chloroplast. Plastid.	Chloroplast.

P56782	S	Chloroplast. Plastid.	Chloroplast.
P56784	_	Chloroplast.	Chloroplast.
P56791	C	Chloroplast.	Chloroplast.
P56792	_	Chloroplast.	Chloroplast.
P56794	M	Chloroplast.	Chloroplast.
P56797	_	Chloroplast.	Chloroplast.
P56799	M	Chloroplast.	Chloroplast.
P56801	_	Chloroplast.	Chloroplast.
P58271	_	Chloroplast.	Chloroplast.
P58419	S	Chloroplast.	Chloroplast.
P58420	S	Chloroplast.	Chloroplast.
P59032	_	Chloroplast.	Chloroplast.
P59136	M	Chloroplast.	Chloroplast.
P59142	M	Chloroplast.	Chloroplast.
P59145	M	Chloroplast.	Chloroplast.
P59146	M	Chloroplast.	Chloroplast.
P59152	_	Chloroplast.	Chloroplast.
P60282	_	Chloroplast.	Chloroplast.
P60290	_	Chloroplast.	Chloroplast.
P60577	_	Chloroplast.	Chloroplast.
P61841	_	Mitochondrion. Plastid.	Chloroplast.
P61845	_	Chloroplast.	Chloroplast.
P62127	C	Chloroplast.	Chloroplast.
P62128	C	Chloroplast.	Chloroplast.
P62729	M	Mitochondrion. Plastid.	Chloroplast.

P62731	M	Mitochondrion. Plastid.	Chloroplast.
P68158	C	Chloroplast.	Chloroplast.
P68751	_	Chloroplast.	Chloroplast.
P68752	_	Chloroplast.	Chloroplast.
P69041	_	Chloroplast.	Chloroplast.
P69250	C	Chloroplast.	Chloroplast.
P69378	S	Chloroplast. Plastid.	Chloroplast.
P69379	S	Chloroplast. Plastid.	Chloroplast.
P69570	_	Chloroplast.	Chloroplast.
P69663	_	Mitochondrion. Plastid.	Chloroplast.
P69664	_	Mitochondrion. Plastid.	Chloroplast.
P69667	_	Chloroplast.	Chloroplast.
P80042	S	Chloroplast. Plastid.	Chloroplast.
P80093	C	Chloroplast.	Chloroplast.
P81372	_	Chloroplast. Cytoplasm.	Chloroplast.
P82192	_	Chloroplast.	Chloroplast.
P82277	C	Chloroplast.	Chloroplast.
P83524	_	Chloroplast. Cytoplasm.	Chloroplast.
P83584	_	Chloroplast. Cytoplasm.	Chloroplast.
P83585	_	Chloroplast. Cytoplasm.	Chloroplast.
P92209	_	Chloroplast. Plastid.	Chloroplast.
P92225	_	Chloroplast. Plastid.	Chloroplast.

P92309	_	Chloroplast. Plastid.	Chloroplast.
P92429	_	Chloroplast. Plastid.	Chloroplast.
P92892	_	Chloroplast. Plastid.	Chloroplast.
P93960	_	Chloroplast. Plastid.	Chloroplast.
P93964	_	Chloroplast. Plastid.	Chloroplast.
P93974	_	Chloroplast. Plastid.	Chloroplast.
Q00218	C	Chloroplast.	Chloroplast.
Q00775	C	Chloroplast.	Chloroplast.
Q00864	M	Chloroplast.	Chloroplast.
Q00866	_	Chloroplast.	Chloroplast.
Q01923	_	Chloroplast. Plastid.	Chloroplast.
Q04450	C	Chloroplast.	Chloroplast.
Q06021	_	Chloroplast.	Chloroplast.
Q06022	_	Chloroplast.	Chloroplast.
Q06030	C	Chloroplast.	Chloroplast.
Q06FM0	_	Mitochondrion. Plastid.	Chloroplast.
Q06FM3	C	Chloroplast.	Chloroplast.
Q06FM9	_	Chloroplast.	Chloroplast.
Q06FN4	_	Chloroplast.	Chloroplast.
Q06FP4	M	Chloroplast.	Chloroplast.
Q06FP5	M	Chloroplast.	Chloroplast.
Q06FX0	_	Chloroplast. Nucleus.	Chloroplast.
Q06FX1	_	Chloroplast.	Chloroplast.
Q06GJ6	_	Chloroplast.	Chloroplast.
Q06GL9	_	Chloroplast.	Chloroplast.

Q06GM1	M	Chloroplast.	Chloroplast.
Q06GM5	_	Chloroplast.	Chloroplast.
Q06GM8	_	Chloroplast. Plastid.	Chloroplast.
Q06GN5	_	Chloroplast.	Chloroplast.
Q06GN7	M	Chloroplast.	Chloroplast.
Q06GN8	_	Chloroplast.	Chloroplast.
Q06GR2	C	Chloroplast.	Chloroplast.
Q06GR8	_	Chloroplast.	Chloroplast.
Q06GR9	M	Chloroplast. Nucleus.	Chloroplast.
Q06GS0	_	Chloroplast.	Chloroplast.
Q06GT3	_	Chloroplast.	Chloroplast.
Q06GT7	–	Mitochondrion. Plastid.	Chloroplast.
Q06GT9	_	Chloroplast.	Chloroplast.
Q06GU8	M	Chloroplast.	Chloroplast.
Q06GV6	_	Chloroplast.	Chloroplast.
Q06GV8	M	Chloroplast.	Chloroplast.
Q06GW2	_	Chloroplast.	Chloroplast.
Q06GX3	C	Chloroplast.	Chloroplast.
Q06GX4	M	Chloroplast.	Chloroplast.
Q06GX5	_	Chloroplast.	Chloroplast.
Q06GZ5	M	Chloroplast.	Chloroplast.
Q06GZ9	M	Chloroplast.	Chloroplast.
Q06H06	M	Chloroplast. Nucleus.	Chloroplast.
Q06H07	_	Chloroplast.	Chloroplast.

Q06H14	S	Chloroplast. Plastid.	Chloroplast.
Q06R66	_	Chloroplast.	Chloroplast.
Q06R80	S	Chloroplast.	Chloroplast.
Q06R91	_	Chloroplast.	Chloroplast.
Q06RA9	M	Chloroplast.	Chloroplast.
Q06RB1	M	Chloroplast. Plastid.	Chloroplast.
Q06RC1	_	Chloroplast.	Chloroplast.
Q06RD3	M	Chloroplast.	Chloroplast.
Q06RD9	_	Chloroplast.	Chloroplast.
Q06RE8	S	Chloroplast. Plastid.	Chloroplast.
Q06SD8	M	Chloroplast.	Chloroplast.
Q06SE3	_	Chloroplast.	Chloroplast.
Q06SF4	_	Chloroplast.	Chloroplast.
Q06SF9	_	Chloroplast.	Chloroplast.
Q06SG4	C	Chloroplast.	Chloroplast.
Q06SG6	_	Chloroplast.	Chloroplast.
Q06SG9	M	Chloroplast.	Chloroplast.
Q06SI6	M	Chloroplast.	Chloroplast.
Q08183	C	Chloroplast.	Chloroplast.
Q08935	C	Chloroplast.	Chloroplast.
Q09FP6	C	Chloroplast.	Chloroplast.
Q09FR2	M	Chloroplast.	Chloroplast.
Q09FS5	_	Chloroplast.	Chloroplast.
Q09FS9	M	Chloroplast. Plastid.	Chloroplast.
Q09G10	_	Chloroplast.	Chloroplast.
Q09G22	C	Chloroplast.	Chloroplast.

Q09G54	_	Chloroplast.	Chloroplast.
Q09MB1	_	Chloroplast.	Chloroplast.
Q09MB2	_	Chloroplast.	Chloroplast.
Q09MB9	_	Chloroplast.	Chloroplast.
Q09MC6	S	Chloroplast.	Chloroplast.
Q09ME0	M	Chloroplast.	Chloroplast.
Q09ME2	_	Chloroplast.	Chloroplast.
Q09ME5	M	Chloroplast.	Chloroplast.
Q09MF3	C	Chloroplast.	Chloroplast.
Q09MF6	M	Chloroplast.	Chloroplast.
Q09MF7	M	Chloroplast. Plastid.	Chloroplast.
Q09MI7	M	Chloroplast. Nucleus.	Chloroplast.
Q09MI8	_	Chloroplast.	Chloroplast.
Q09MJ5	S	Chloroplast. Plastid.	Chloroplast.
Q09WV5	_	Chloroplast.	Chloroplast.
Q09WV9	_	Mitochondrion. Plastid.	Chloroplast.
Q09WW1	C	Chloroplast.	Chloroplast.
Q09WX0	M	Chloroplast.	Chloroplast.
Q09WX8	M	Chloroplast.	Chloroplast.
Q09WY0	M	Mitochondrion. Plastid.	Chloroplast.
Q09WY1	_	Chloroplast.	Chloroplast.
Q09WY5	M	Chloroplast.	Chloroplast.
Q09WZ3	C	Chloroplast.	Chloroplast.
Q09WZ5	M	Chloroplast.	Chloroplast.

Q09WZ6	C	Chloroplast. Plastid.	Chloroplast.
Q09X09	_	Chloroplast.	Chloroplast.
Q09X19	_	Chloroplast.	Chloroplast.
Q09X25	_	Chloroplast.	Chloroplast.
Q09X26	M	Chloroplast. Nucleus.	Chloroplast.
Q0DDE3	C	Chloroplast.	Chloroplast.
Q0DEC8	C	Chloroplast.	Chloroplast.
Q0DUI8	C	Chloroplast.	Chloroplast.
Q0G9G9	S	Chloroplast.	Chloroplast.
Q0G9H1	M	Chloroplast.	Chloroplast.
Q0G9H9	_	Chloroplast.	Chloroplast.
Q0G9I3	_	Chloroplast.	Chloroplast.
Q0G9I4	_	Chloroplast.	Chloroplast.
Q0G9I7	_	Chloroplast. Plastid.	Chloroplast.
Q0G9M1	M	Chloroplast.	Chloroplast.
Q0G9M9	_	Chloroplast.	Chloroplast.
Q0G9P9	C	Chloroplast.	Chloroplast.
Q0G9Q0	_	Chloroplast.	Chloroplast.
Q0G9R4	M	Chloroplast.	Chloroplast.
Q0G9S2	_	Chloroplast.	Chloroplast.
Q0G9S4	M	Mitochondrion. Plastid.	Chloroplast.
Q0G9S6	_	Chloroplast.	Chloroplast.
Q0G9S9	C	Chloroplast.	Chloroplast.
Q0G9U0	M	Chloroplast.	Chloroplast.
Q0G9U1	_	Chloroplast. Plastid.	Chloroplast.

Q0G9W0	M	Chloroplast.	Chloroplast.
Q0P3J3	_	Chloroplast.	Chloroplast.
Q0P3L1	M	Chloroplast.	Chloroplast.
Q0P3L3	_	Chloroplast.	Chloroplast.
Q0P3L4	M	Chloroplast.	Chloroplast.
Q0P3L6	M	Chloroplast.	Chloroplast.
Q0P3L8	_	Chloroplast.	Chloroplast.
Q0P3L9	M	Chloroplast.	Chloroplast.
Q0P3M5	_	Chloroplast. Nucleus.	Chloroplast.
Q0P3M9	C	Chloroplast.	Chloroplast.
Q0P3N0	M	Chloroplast.	Chloroplast.
Q0P3P4	_	Mitochondrion.	Chloroplast.
Q0PUX0	_	Chloroplast.	Chloroplast.
Q0ZIV5	_	Chloroplast.	Chloroplast.
Q0ZIY6	C	Chloroplast.	Chloroplast.
Q0ZIY7	_	Chloroplast. Plastid.	Chloroplast.
Q0ZIZ4	_	Chloroplast.	Chloroplast.
Q0ZIZ5	C	Chloroplast.	Chloroplast.
Q0ZIZ8	M	Chloroplast.	Chloroplast.
Q0ZJ22	M	Chloroplast.	Chloroplast.
Q0ZJ28	_	Chloroplast.	Chloroplast.
Q14F95	C	Chloroplast.	Chloroplast.
Q14FA1	_	Chloroplast.	Chloroplast.
Q14FA8	S	Chloroplast.	Chloroplast.
Q14FB1	M	Chloroplast.	Chloroplast.
Q14FB6	C	Chloroplast.	Chloroplast.

Q14FC4	C	Chloroplast.	Chloroplast.
Q14FD2	_	Chloroplast.	Chloroplast.
Q14FE9	_	Chloroplast.	Chloroplast.
Q14FF5	M	Chloroplast.	Chloroplast.
Q14FG5	_	Chloroplast.	Chloroplast.
Q14FG6	M	Chloroplast. Nucleus.	Chloroplast.
Q14FH4	S	Chloroplast. Plastid.	Chloroplast.
Q19V92	M	Chloroplast.	Chloroplast.
Q19VA6	_	Chloroplast.	Chloroplast.
Q19VB3	_	Chloroplast.	Chloroplast.
Q19VB6	_	Chloroplast.	Chloroplast.
Q19VC3	_	Chloroplast.	Chloroplast.
Q1ACE0	_	Chloroplast.	Chloroplast.
Q1ACF1	M	Chloroplast. Plastid.	Chloroplast.
Q1ACF2	_	Chloroplast.	Chloroplast.
Q1ACF5	_	Chloroplast.	Chloroplast.
Q1ACF7	S	Chloroplast.	Chloroplast.
Q1ACF8	_	Chloroplast.	Chloroplast.
Q1ACF9	_	Chloroplast.	Chloroplast.
Q1ACG3	_	Chloroplast.	Chloroplast.
Q1ACG7	_	Chloroplast.	Chloroplast.
Q1ACH6	_	Chloroplast.	Chloroplast.
Q1ACH9	M	Chloroplast.	Chloroplast.
Q1ACL0	_	Mitochondrion.	Chloroplast.
Q1ACL1	M	Chloroplast.	Chloroplast.
Q1ACM7	M	Chloroplast.	Chloroplast.

Q1ACN3	_	Chloroplast.	Chloroplast.
Q1ACN6	_	Chloroplast.	Chloroplast.
Q1ACP2	M	Mitochondrion. Plastid.	Chloroplast.
Q1KVR1	_	Chloroplast.	Chloroplast.
Q1KVR2	_	Chloroplast.	Chloroplast.
Q1KVR6	_	Chloroplast.	Chloroplast.
Q1KVR9	_	Chloroplast.	Chloroplast.
Q1KVT1	C	Chloroplast.	Chloroplast.
Q1KVT5	M	Chloroplast.	Chloroplast.
Q1KVT6	_	Chloroplast.	Chloroplast.
Q1KVT9	M	Chloroplast.	Chloroplast.
Q1KVV0	_	Chloroplast.	Chloroplast.
Q1KVX0	C	Chloroplast.	Chloroplast.
Q1KVX4	_	Chloroplast.	Chloroplast.
Q1KVX7	_	Chloroplast.	Chloroplast.
Q1KXP8	_	Mitochondrion. Plastid.	Chloroplast.
Q1KXQ3	S	Chloroplast.	Chloroplast.
Q1KXR8	_	Chloroplast.	Chloroplast.
Q1KXR9	M	Chloroplast.	Chloroplast.
Q1KXS0	_	Chloroplast.	Chloroplast.
Q1KXS3	_	Chloroplast.	Chloroplast.
Q1KXS6	C	Chloroplast.	Chloroplast.
Q1KXT6	M	Chloroplast.	Chloroplast.
Q1KXT7	_	Chloroplast.	Chloroplast.
Q1KXW1	_	Chloroplast.	Chloroplast.

Q1KXX7	M	Chloroplast.	Chloroplast.
Q20EV0	M	Chloroplast.	Chloroplast.
Q20EV1	M	Chloroplast.	Chloroplast.
Q20EY2	M	Chloroplast.	Chloroplast.
Q20F02	M	Chloroplast.	Chloroplast.
Q20F07	_	Chloroplast.	Chloroplast.
Q20F08	_	Chloroplast.	Chloroplast.
Q20F09	M	Mitochondrion. Plastid.	Chloroplast.
Q20F10	M	Chloroplast.	Chloroplast.
Q20F12	M	Chloroplast.	Chloroplast.
Q27RZ0	M	Mitochondrion. Plastid.	Chloroplast.
Q27S25	C	Chloroplast.	Chloroplast.
Q2L8Z0	S	Chloroplast. Plastid.	Chloroplast.
Q2L8Z5	_	Chloroplast.	Chloroplast.
Q2L8Z9	_	Chloroplast.	Chloroplast.
Q2L928	M	Chloroplast.	Chloroplast.
Q2L940	M	Mitochondrion. Plastid.	Chloroplast.
Q2L941	_	Chloroplast.	Chloroplast.
Q2L942	_	Chloroplast.	Chloroplast.
Q2L945	_	Chloroplast.	Chloroplast.
Q2L946	M	Chloroplast.	Chloroplast.
Q2MI43	_	Chloroplast.	Chloroplast.
Q2MI44	_	Chloroplast. Plastid.	Chloroplast.
Q2MI51	S	Chloroplast. Plastid.	Chloroplast.

Q2MI58	_	Chloroplast.	Chloroplast.
Q2MI60	_	Chloroplast.	Chloroplast.
Q2MI61	_	Chloroplast.	Chloroplast.
Q2MI62	M	Chloroplast.	Chloroplast.
Q2MI64	_	Chloroplast.	Chloroplast.
Q2MI68	C	Chloroplast.	Chloroplast.
Q2MI95	S	Chloroplast. Plastid.	Chloroplast.
Q2MI96	_	Chloroplast. Plastid.	Chloroplast.
Q2MIA8	_	Chloroplast.	Chloroplast.
Q2MIB0	_	Chloroplast.	Chloroplast.
Q2MIB1	_	Chloroplast.	Chloroplast.
Q2MIB9	_	Chloroplast.	Chloroplast.
Q2MID0	_	Chloroplast.	Chloroplast.
Q2MID9	M	Chloroplast.	Chloroplast.
Q2MIE3	_	Chloroplast.	Chloroplast.
Q2MIE6	C	Chloroplast.	Chloroplast.
Q2MIE8	_	Chloroplast.	Chloroplast.
Q2MIF1	_	Chloroplast.	Chloroplast.
Q2MIF6	M	Chloroplast. Plastid.	Chloroplast.
Q2MIH9	_	Chloroplast.	Chloroplast.
Q2MIJ7	_	Chloroplast.	Chloroplast.
Q2MIK8	C	Chloroplast.	Chloroplast.
Q2PMN9	_	Chloroplast.	Chloroplast.
Q2PMP7	_	Chloroplast.	Chloroplast.
Q2PMP8	M	Chloroplast.	Chloroplast.
Q2PMQ2	C	Chloroplast.	Chloroplast.

Q2PMR3	_	Chloroplast. Plastid.	Chloroplast.
Q2PMS6	S	Chloroplast. Plastid.	Chloroplast.
Q2PMU5	M	Chloroplast.	Chloroplast.
Q2QD42	C	Chloroplast.	Chloroplast.
Q2TGZ2	_	Chloroplast.	Chloroplast.
Q2VEB7	C	Chloroplast.	Chloroplast.
Q2VED0	S	Chloroplast.	Chloroplast.
Q2VED6	_	Chloroplast.	Chloroplast.
Q2VED8	_	Chloroplast.	Chloroplast.
Q2VEE3	M	Mitochondrion. Plastid.	Chloroplast.
Q2VEE4	_	Chloroplast.	Chloroplast.
Q2VEH3	_	Chloroplast. Plastid.	Chloroplast.
Q2VEH5	M	Chloroplast.	Chloroplast.
Q2VEJ3	S	Chloroplast. Plastid.	Chloroplast.
Q2WGF1	_	Mitochondrion. Plastid.	Chloroplast.
Q31795	_	Chloroplast.	Chloroplast.
Q31796	_	Chloroplast.	Chloroplast.
Q31886	_	Chloroplast.	Chloroplast.
Q32616	_	Chloroplast.	Chloroplast.
Q32701	_	Chloroplast.	Chloroplast.
Q32RF8	_	Chloroplast.	Chloroplast.
Q32RG2	S	Chloroplast.	Chloroplast.
Q32RK7	_	Chloroplast.	Chloroplast.
Q32RM2	_	Mitochondrion.	Chloroplast.
Q32RM8	M	Chloroplast.	Chloroplast.

Q32RN4	_	Chloroplast.	Chloroplast.
Q32RN7	_	Chloroplast.	Chloroplast.
Q32RN8	M	Chloroplast.	Chloroplast.
Q32RP7	M	Mitochondrion. Plastid.	Chloroplast.
Q32RP8	M	Chloroplast.	Chloroplast.
Q32RP9	M	Chloroplast.	Chloroplast.
Q32RQ1	_	Chloroplast.	Chloroplast.
Q32RQ9	C	Chloroplast.	Chloroplast.
Q32RS1	M	Chloroplast.	Chloroplast.
Q32RS5	_	Chloroplast.	Chloroplast.
Q32RT8	_	Chloroplast.	Chloroplast.
Q32RT9	M	Chloroplast.	Chloroplast.
Q32RU8	M	Chloroplast.	Chloroplast.
Q32RV2	_	Chloroplast.	Chloroplast.
Q32RV4	_	Chloroplast.	Chloroplast.
Q32RV8	_	Chloroplast.	Chloroplast.
Q32RW6	S	Chloroplast.	Chloroplast.
Q32RY2	M	Chloroplast.	Chloroplast.
Q32RZ7	_	Chloroplast.	Chloroplast.
Q32S07	_	Chloroplast.	Chloroplast.
Q332R5	_	Chloroplast.	Chloroplast.
Q332R9	_	Mitochondrion. Plastid.	Chloroplast.
Q332S0	_	Chloroplast.	Chloroplast.
Q332S9	M	Chloroplast.	Chloroplast.
Q332T6	_	Chloroplast.	Chloroplast.

Q332U5	M	Chloroplast. Plastid.	Chloroplast.
Q332V2	_	Chloroplast.	Chloroplast.
Q332V5	M	Chloroplast.	Chloroplast.
Q332V7	_	Chloroplast. Plastid.	Chloroplast.
Q332X6	M	Chloroplast.	Chloroplast.
Q332Z6	M	Chloroplast.	Chloroplast.
Q33301	S	Chloroplast. Plastid.	Chloroplast.
Q33BX4	M	Chloroplast.	Chloroplast.
Q33BY9	_	Chloroplast.	Chloroplast.
Q33BZ2	_	Chloroplast.	Chloroplast.
Q33BZ5	M	Mitochondrion. Plastid.	Chloroplast.
Q33BZ6	_	Chloroplast.	Chloroplast.
Q33C07	_	Chloroplast.	Chloroplast.
Q33C11	_	Chloroplast. Plastid.	Chloroplast.
Q33C26	_	Chloroplast.	Chloroplast.
Q33C48	_	Chloroplast.	Chloroplast.
Q36996	_	Chloroplast. Plastid.	Chloroplast.
Q37082	_	Chloroplast. Plastid.	Chloroplast.
Q38933	_	Chloroplast.	Chloroplast.
Q39161	C	Chloroplast. Mitochondrion.	Chloroplast.
Q39473	C	Chloroplast.	Chloroplast.
Q39617	C	Chloroplast.	Chloroplast.
Q39743	C	Chloroplast.	Chloroplast.
Q39748	S	Chloroplast.	Chloroplast.
Q3BAH4	_	Mitochondrion.	Chloroplast.

		Plastid.	
Q3BAJ9	M	Mitochondrion. Plastid.	Chloroplast.
Q3BAK4	C	Chloroplast.	Chloroplast.
Q3BAL9	_	Chloroplast. Plastid.	Chloroplast.
Q3BAN8	M	Chloroplast.	Chloroplast.
Q3BAP8	_	Chloroplast.	Chloroplast.
Q3BAQ1	M	Chloroplast. Nucleus.	Chloroplast.
Q3C1G7	_	Chloroplast.	Chloroplast.
Q3C1J4	_	Chloroplast.	Chloroplast.
Q3C1K6	M	Chloroplast.	Chloroplast.
Q3C1K7	M	Chloroplast.	Chloroplast.
Q3C1L5	_	Chloroplast.	Chloroplast.
Q3C1L8	M	Chloroplast.	Chloroplast.
Q3C1L9	_	Chloroplast.	Chloroplast.
Q3C1M0	_	Chloroplast.	Chloroplast.
Q3C1M2	C	Chloroplast.	Chloroplast.
Q3C1N5	_	Chloroplast.	Chloroplast.
Q3C1N7	_	Chloroplast.	Chloroplast.
Q3C1Q4	S	Chloroplast.	Chloroplast.
Q3MKB3	_	Chloroplast.	Chloroplast.
Q3V4X1	_	Chloroplast.	Chloroplast.
Q3V4Z6	M	Mitochondrion. Plastid.	Chloroplast.
Q3V4Z8	_	Chloroplast.	Chloroplast.
Q3V502	_	Chloroplast. Plastid.	Chloroplast.

Q3V532	M	Chloroplast.	Chloroplast.
Q3V542	_	Chloroplast.	Chloroplast.
Q3V543	_	Chloroplast. Nucleus.	Chloroplast.
Q3ZJ12	_	Mitochondrion.	Chloroplast.
Q3ZJ37	M	Chloroplast.	Chloroplast.
Q3ZJ38	_	Chloroplast.	Chloroplast.
Q3ZJ47	C	Chloroplast.	Chloroplast.
Q3ZJ78	_	Chloroplast.	Chloroplast.
Q3ZJ83	_	Chloroplast.	Chloroplast.
Q3ZJ84	M	Chloroplast.	Chloroplast.
Q3ZJ86	_	Chloroplast.	Chloroplast.
Q3ZJ88	_	Chloroplast.	Chloroplast.
Q40406	_	Chloroplast.	Chloroplast.
Q40677	_	Chloroplast. Mitochondrion.	Chloroplast.
Q41578	C	Chloroplast.	Chloroplast.
Q42796	C	Chloroplast.	Chloroplast.
Q42822	C	Chloroplast.	Chloroplast.
Q42843	C	Chloroplast. Cytoplasm.	Chloroplast.
Q42850	C	Chloroplast.	Chloroplast.
Q42876	C	Chloroplast. Cytoplasm.	Chloroplast.
Q42967	C	Chloroplast.	Chloroplast.
Q43036	_	Chloroplast.	Chloroplast.
Q43058	C	Chloroplast.	Chloroplast.
Q43086	C	Chloroplast.	Chloroplast.

Q43087	C	Chloroplast.	Chloroplast.
Q43092	C	Chloroplast.	Chloroplast.
Q43467	C	Chloroplast.	Chloroplast.
Q43503	_	Chloroplast.	Chloroplast.
Q43727	C	Chloroplast.	Chloroplast.
Q43784	C	Chloroplast.	Chloroplast.
Q49KT5	_	Chloroplast.	Chloroplast.
Q49KU1	_	Chloroplast.	Chloroplast.
Q49KV0	_	Chloroplast.	Chloroplast.
Q49KV9	M	Chloroplast.	Chloroplast.
Q49KW0	_	Chloroplast.	Chloroplast.
Q49KW2	_	Chloroplast.	Chloroplast.
Q49KW5	C	Chloroplast.	Chloroplast.
Q49KX3	_	Chloroplast.	Chloroplast.
Q49KX4	C	Chloroplast.	Chloroplast.
Q49KX7	M	Chloroplast.	Chloroplast.
Q49KZ0	_	Chloroplast.	Chloroplast.
Q49L07	M	Chloroplast. Nucleus.	Chloroplast.
Q49U13	S	Chloroplast.	Chloroplast.
Q4FG71	M	Chloroplast.	Chloroplast.
Q4FG72	M	Chloroplast.	Chloroplast.
Q4FG73	M	Chloroplast.	Chloroplast.
Q4H186	_	Chloroplast.	Chloroplast.
Q4H195	_	Chloroplast.	Chloroplast.
Q4H1A1	_	Chloroplast.	Chloroplast.
Q4LAM6	_	Chloroplast.	Chloroplast.

Q4VZH3	M	Chloroplast.	Chloroplast.
Q4VZK2	C	Chloroplast.	Chloroplast.
Q4VZK3	M	Chloroplast.	Chloroplast.
Q4VZK5	_	Chloroplast.	Chloroplast.
Q4VZL1	_	Chloroplast.	Chloroplast.
Q4VZM8	_	Chloroplast.	Chloroplast.
Q4VZM9	M	Chloroplast.	Chloroplast.
Q4VZP1	_	Chloroplast.	Chloroplast.
Q4VZP2	M	Chloroplast. Nucleus.	Chloroplast.
Q4VZP3	_	Chloroplast.	Chloroplast.
Q507P6	_	Chloroplast.	Chloroplast.
Q507Q9	_	Chloroplast.	Chloroplast.
Q507T3	_	Chloroplast.	Chloroplast.
Q52TG6	M	Chloroplast.	Chloroplast.
Q56B59	_	Chloroplast.	Chloroplast.
Q56P11	_	Chloroplast.	Chloroplast.
Q56P13	_	Chloroplast.	Chloroplast.
Q589A4	_	Mitochondrion. Plastid.	Chloroplast.
Q589B1	S	Chloroplast. Plastid.	Chloroplast.
Q589B8	_	Chloroplast.	Chloroplast.
Q589B9	_	Chloroplast. Nucleus.	Chloroplast.
Q589C0	_	Chloroplast.	Chloroplast.
Q5D1B9	M	Chloroplast.	Chloroplast.
Q5D1C1	_	Chloroplast.	Chloroplast.

Q5F4M7	_	Chloroplast.	Chloroplast.
Q5GA98	_	Chloroplast.	Chloroplast.
Q5GAA8	_	Chloroplast.	Chloroplast.
Q5GAA9	_	Chloroplast.	Chloroplast.
Q5GAB0	_	Chloroplast.	Chloroplast.
Q5GF59	_	Chloroplast.	Chloroplast.
Q5GF70	_	Chloroplast.	Chloroplast.
Q5GGS4	_	Chloroplast.	Chloroplast.
Q5GIQ4	_	Chloroplast.	Chloroplast.
Q5GIR6	_	Chloroplast.	Chloroplast.
Q5J2U8	_	Chloroplast.	Chloroplast.
Q5J2V8	S	Chloroplast.	Chloroplast.
Q5J2W0	_	Chloroplast.	Chloroplast.
Q5J2Z9	_	Chloroplast.	Chloroplast.
Q5J300	_	Chloroplast.	Chloroplast.
Q5QA83	_	Chloroplast.	Chloroplast.
Q5SCW6	M	Chloroplast.	Chloroplast.
Q5SCX1	S	Chloroplast.	Chloroplast.
Q5SCY6	_	Mitochondrion. Plastid.	Chloroplast.
Q5SD08	M	Chloroplast.	Chloroplast.
Q5SD15	_	Chloroplast.	Chloroplast.
Q5SD16	_	Chloroplast.	Chloroplast.
Q5SD18	M	Chloroplast.	Chloroplast.
Q5SD19	_	Chloroplast.	Chloroplast.
Q5SD20	_	Chloroplast.	Chloroplast.
Q5SD26	M	Chloroplast.	Chloroplast.

Q5SD27	C	Chloroplast.	Chloroplast.
Q5SD28	_	Chloroplast.	Chloroplast.
Q5SD37	M	Chloroplast.	Chloroplast.
Q5VH50	S	Chloroplast.	Chloroplast.
Q5YJV9	_	Chloroplast.	Chloroplast.
Q5YJX3	_	Chloroplast.	Chloroplast.
Q5YJY5	_	Chloroplast.	Chloroplast.
Q5YK03	M	Chloroplast.	Chloroplast.
Q5YK05	M	Chloroplast.	Chloroplast.
Q5YK53	_	Chloroplast.	Chloroplast.
Q5YLB5	C	Chloroplast. Mitochondrion.	Chloroplast. Mitochondrion.
Q646L1	_	Chloroplast.	Chloroplast.
Q67BD8	_	Chloroplast.	Chloroplast.
Q67IA4	_	Mitochondrion. Plastid.	Chloroplast.
Q67IB0	_	Mitochondrion. Plastid.	Chloroplast.
Q67IB6	_	Mitochondrion. Plastid.	Chloroplast.
Q67ID1	_	Mitochondrion. Plastid.	Chloroplast.
Q67IE3	_	Mitochondrion. Plastid.	Chloroplast.
Q67II8	_	Mitochondrion. Plastid.	Chloroplast.
Q68RU3	_	Chloroplast.	Chloroplast.
Q68RU7	_	Mitochondrion. Plastid.	Chloroplast.
Q68RV6	S	Chloroplast.	Chloroplast.

Q68RW9	M	Chloroplast.	Chloroplast.
Q68RX1	_	Chloroplast.	Chloroplast.
Q68RX2	_	Chloroplast.	Chloroplast.
Q68RX4	C	Chloroplast.	Chloroplast.
Q68RY4	M	Chloroplast.	Chloroplast.
Q68S04	M	Chloroplast.	Chloroplast.
Q6BDG1	_	Chloroplast.	Chloroplast.
Q6BDG8	_	Chloroplast.	Chloroplast.
Q6BDH3	_	Chloroplast.	Chloroplast.
Q6BDJ4	_	Chloroplast.	Chloroplast.
Q6EIH3	M	Chloroplast.	Chloroplast.
Q6EIJ4	M	Chloroplast.	Chloroplast.
Q6EIJ6	M	Chloroplast.	Chloroplast.
Q6EM84	_	Mitochondrion. Plastid.	Chloroplast.
Q6EMB0	_	Mitochondrion. Plastid.	Chloroplast.
Q6ENA1	_	Chloroplast. Plastid.	Chloroplast.
Q6ENA4	S	Chloroplast. Plastid.	Chloroplast.
Q6ENA5	S	Chloroplast. Plastid.	Chloroplast.
Q6ENA7	S	Chloroplast. Plastid.	Chloroplast.
Q6ENA9	M	Chloroplast. Plastid.	Chloroplast.
Q6ENB2	_	Chloroplast.	Chloroplast.
Q6ENC9	_	Chloroplast. Plastid.	Chloroplast.
Q6END5	M	Chloroplast. Plastid.	Chloroplast.
Q6END9	_	Chloroplast. Plastid.	Chloroplast.
Q6ENE2	_	Chloroplast. Plastid.	Chloroplast.

Q6ENG9	S	Chloroplast. Plastid.	Chloroplast.
Q6ENH0	_	Chloroplast. Plastid.	Chloroplast.
Q6ENH1	_	Chloroplast. Plastid.	Chloroplast.
Q6ENH2	M	Chloroplast.	Chloroplast.
Q6ENH6	M	Chloroplast. Plastid.	Chloroplast.
Q6ENI1	_	Chloroplast.	Chloroplast.
Q6ENJ5	M	Chloroplast. Plastid.	Chloroplast.
Q6ENJ6	S	Chloroplast.	Chloroplast.
Q6ENP4	S	Chloroplast. Plastid.	Chloroplast.
Q6ENP5	S	Chloroplast. Plastid.	Chloroplast.
Q6ENQ1	_	Chloroplast.	Chloroplast.
Q6ENQ5	M	Chloroplast. Plastid.	Chloroplast.
Q6ENR2	S	Chloroplast. Plastid.	Chloroplast.
Q6ENR3	M	Chloroplast.	Chloroplast.
Q6ENS0	_	Chloroplast. Plastid.	Chloroplast.
Q6ENS1	_	Chloroplast.	Chloroplast.
Q6ENS3	_	Chloroplast.	Chloroplast.
Q6ENS5	M	Chloroplast.	Chloroplast.
Q6ENS7	_	Chloroplast.	Chloroplast.
Q6ENU0	C	Chloroplast.	Chloroplast.
Q6ENU3	_	Chloroplast. Plastid.	Chloroplast.
Q6ENW5	C	Chloroplast. Plastid.	Chloroplast.
Q6ENX2	_	Chloroplast. Nucleus. Plastid.	Chloroplast.
Q6ENX3	_	Chloroplast. Plastid.	Chloroplast.
Q6ENX8	M	Chloroplast.	Chloroplast.
Q6ENY4	M	Chloroplast. Plastid.	Chloroplast.

Q6ENY5	S	Chloroplast. Plastid.	Chloroplast.
Q6EVY6	_	Chloroplast.	Chloroplast.
Q6EVZ0	_	Mitochondrion. Plastid.	Chloroplast.
Q6EW11	_	Chloroplast.	Chloroplast.
Q6EW12	_	Chloroplast.	Chloroplast.
Q6EW13	M	Chloroplast.	Chloroplast.
Q6EW15	_	Chloroplast.	Chloroplast.
Q6EW19	C	Chloroplast.	Chloroplast.
Q6EW28	C	Chloroplast.	Chloroplast.
Q6EW30	M	Plastid.	Chloroplast.
Q6EW50	_	Chloroplast.	Chloroplast.
Q6EW56	_	Chloroplast.	Chloroplast.
Q6EW66	M	Chloroplast.	Chloroplast.
Q6EW67	_	Chloroplast.	Chloroplast.
Q6EW71	_	Chloroplast.	Chloroplast.
Q6H9K4	M	Chloroplast.	Chloroplast.
Q6H9K7	M	Chloroplast.	Chloroplast.
Q6H9L0	M	Chloroplast.	Chloroplast.
Q6H9L1	M	Chloroplast.	Chloroplast.
Q6H9L3	M	Chloroplast.	Chloroplast.
Q6KGV8	M	Mitochondrion. Plastid.	Chloroplast.
Q6KGW1	_	Mitochondrion. Plastid.	Chloroplast.
Q6KGW4	_	Mitochondrion. Plastid.	Chloroplast.
Q6KGW7	M	Mitochondrion.	Chloroplast.

		Plastid.	
Q6KGX6	_	Mitochondrion. Plastid.	Chloroplast.
Q6KGX9	_	Mitochondrion. Plastid.	Chloroplast.
Q6L380	_	Chloroplast. Plastid.	Chloroplast.
Q6L394	S	Chloroplast. Plastid.	Chloroplast.
Q6L397	M	Chloroplast.	Chloroplast.
Q6L3A4	_	Chloroplast.	Chloroplast.
Q6L3A7	_	Chloroplast. Plastid.	Chloroplast.
Q6L3B3	S	Chloroplast. Plastid.	Chloroplast.
Q6L3B4	M	Chloroplast. Plastid.	Chloroplast.
Q6L3B6	_	Chloroplast.	Chloroplast.
Q6L3B9	_	Chloroplast.	Chloroplast.
Q6L3D3	_	Chloroplast.	Chloroplast.
Q6L3D7	S	Chloroplast. Plastid.	Chloroplast.
Q6L3E0	S	Chloroplast.	Chloroplast.
Q6L3F1	S	Chloroplast. Plastid.	Chloroplast.
Q6LA06	M	Chloroplast.	Chloroplast.
Q6LA20	_	Chloroplast.	Chloroplast.
Q6PP78	M	Chloroplast.	Chloroplast.
Q6PSB9	M	Chloroplast.	Chloroplast.
Q6PSE2	M	Chloroplast.	Chloroplast.
Q6QHD9	_	Chloroplast.	Chloroplast.
Q6QUL7	_	Chloroplast.	Chloroplast.
Q6TND0	_	Chloroplast.	Chloroplast.
Q6VQA9	_	Chloroplast.	Chloroplast.

Q6YLW2	_	Chloroplast. Plastid.	Chloroplast.
Q6YP18	_	Chloroplast.	Chloroplast.
Q6YXJ8	M	Chloroplast.	Chloroplast.
Q6YXK5	_	Chloroplast.	Chloroplast.
Q6YXK6	_	Chloroplast.	Chloroplast.
Q6YXL2	_	Chloroplast.	Chloroplast.
Q6YXM4	M	Chloroplast.	Chloroplast.
Q6YXM5	M	Chloroplast.	Chloroplast.
Q6YXP3	M	Chloroplast.	Chloroplast.
Q6YXP6	_	Chloroplast.	Chloroplast.
Q6YXQ3	S	Chloroplast.	Chloroplast.
Q6YXQ4	M	Chloroplast.	Chloroplast.
Q6YXQ5	_	Chloroplast.	Chloroplast.
Q70CZ6	_	Chloroplast.	Chloroplast.
Q70D04	_	Chloroplast.	Chloroplast.
Q70D38	_	Chloroplast.	Chloroplast.
Q70D40	_	Chloroplast.	Chloroplast.
Q70XV7	_	Chloroplast.	Chloroplast.
Q70XX2	M	Chloroplast.	Chloroplast.
Q70XX3	M	Mitochondrion. Plastid.	Chloroplast.
Q70XX6	C	Chloroplast.	Chloroplast.
Q70XY2	_	Chloroplast.	Chloroplast.
Q70XY3	M	Plastid.	Chloroplast.
Q70XY4	_	Chloroplast.	Chloroplast.
Q717X5	_	Chloroplast.	Chloroplast.
Q71L19	_	Mitochondrion.	Chloroplast.

		Plastid.	
Q71L39	_	Mitochondrion. Plastid.	Chloroplast.
Q71T50	_	Mitochondrion. Plastid.	Chloroplast.
Q75VA5	_	Chloroplast.	Chloroplast.
Q75VB1	_	Chloroplast.	Chloroplast.
Q75VB2	_	Chloroplast.	Chloroplast.
Q75VB5	_	Chloroplast.	Chloroplast.
Q76IH4	_	Chloroplast.	Chloroplast.
Q76IH5	_	Chloroplast.	Chloroplast.
Q76LM0	_	Chloroplast.	Chloroplast.
Q7FNS1	_	Chloroplast.	Chloroplast.
Q7FNS6	_	Chloroplast. Plastid.	Chloroplast.
Q7GED0	_	Chloroplast. Plastid.	Chloroplast.
Q7GED9	_	Chloroplast. Plastid.	Chloroplast.
Q7GUC7	_	Chloroplast.	Chloroplast.
Q7H6F6	_	Chloroplast. Plastid.	Chloroplast.
Q7H6J1	_	Chloroplast. Plastid.	Chloroplast.
Q7H6J2	_	Chloroplast. Plastid.	Chloroplast.
Q7HEK3	_	Chloroplast.	Chloroplast.
Q7HKI2	_	Chloroplast.	Chloroplast.
Q7HKI3	_	Chloroplast.	Chloroplast.
Q7HUK6	_	Chloroplast.	Chloroplast.
Q7ICQ5	_	Chloroplast.	Chloroplast.
Q7IS11	M	Chloroplast.	Chloroplast.
Q7IZ70	_	Chloroplast.	Chloroplast.

Q7J1C8	_	Chloroplast.	Chloroplast.
Q7J5Z8	_	Chloroplast.	Chloroplast.
Q7J5Z9	_	Chloroplast.	Chloroplast.
Q7JEV7	_	Chloroplast.	Chloroplast.
Q7JEV9	_	Chloroplast.	Chloroplast.
Q7JEW0	_	Chloroplast.	Chloroplast.
Q7JEW7	_	Chloroplast.	Chloroplast.
Q7XE48	C	Chloroplast.	Chloroplast.
Q7YJG1	_	Chloroplast.	Chloroplast.
Q7YJG2	M	Chloroplast.	Chloroplast.
Q7YJT3	S	Chloroplast.	Chloroplast.
Q7YJT7	_	Chloroplast.	Chloroplast.
Q7YJU1	M	Mitochondrion. Plastid.	Chloroplast.
Q7YJU2	_	Chloroplast.	Chloroplast.
Q7YJU6	_	Chloroplast. Plastid.	Chloroplast.
Q7YJV4	_	Chloroplast.	Chloroplast.
Q7YJX8	_	Chloroplast.	Chloroplast.
Q7YJY5	S	Chloroplast. Plastid.	Chloroplast.
Q7YKM5	_	Chloroplast.	Chloroplast.
Q7YKQ2	_	Chloroplast.	Chloroplast.
Q7YKQ9	M	Chloroplast.	Chloroplast.
Q7YKX3	M	Chloroplast.	Chloroplast.
Q7YM14	_	Chloroplast.	Chloroplast.
Q7YM28	_	Chloroplast.	Chloroplast.
Q7YM30	_	Chloroplast.	Chloroplast.
Q7YMG2	_	Chloroplast.	Chloroplast.

Q7YMV6	_	Chloroplast.	Chloroplast.
Q7YMV7	_	Chloroplast.	Chloroplast.
Q859W3	_	Chloroplast.	Chloroplast.
Q85AI6	_	Mitochondrion. Plastid.	Chloroplast.
Q85AM2	_	Chloroplast.	Chloroplast.
Q85AW7	_	Chloroplast.	Chloroplast.
Q85BB6	S	Chloroplast. Plastid.	Chloroplast.
Q85BJ7	_	Chloroplast.	Chloroplast.
Q85BR7	_	Chloroplast.	Chloroplast.
Q85C28	C	Chloroplast.	Chloroplast.
Q85C71	_	Chloroplast.	Chloroplast.
Q85CS9	M	Chloroplast.	Chloroplast.
Q85CT4	_	Chloroplast.	Chloroplast.
Q85FG6	_	Chloroplast.	Chloroplast.
Q85FH3	S	Chloroplast. Plastid.	Chloroplast.
Q85FH6	S	Chloroplast.	Chloroplast.
Q85FI2	M	Chloroplast.	Chloroplast.
Q85FI4	M	Chloroplast.	Chloroplast.
Q85FI6	_	Chloroplast.	Chloroplast.
Q85FJ0	M	Chloroplast.	Chloroplast.
Q85FL5	S	Chloroplast. Plastid.	Chloroplast.
Q85FM7	_	Chloroplast.	Chloroplast.
Q85FN0	_	Chloroplast.	Chloroplast.
Q85FN7	S	Chloroplast. Plastid.	Chloroplast.
Q85FN9	M	Chloroplast.	Chloroplast.
Q85UZ8	M	Chloroplast.	Chloroplast.

Q85V01	M	Chloroplast.	Chloroplast.
Q85WB7	C	Chloroplast. Cytoplasm.	Chloroplast.
Q85WL0	S	Chloroplast.	Chloroplast.
Q85WS8	_	Chloroplast. Nucleus.	Chloroplast.
Q85WT6	_	Chloroplast.	Chloroplast.
Q85WU8	C	Chloroplast.	Chloroplast.
Q85WV0	C	Chloroplast.	Chloroplast.
Q85WW7	M	Chloroplast.	Chloroplast.
Q85WX8	M	Chloroplast.	Chloroplast.
Q85WY6	_	Chloroplast.	Chloroplast.
Q85WY8	_	Chloroplast.	Chloroplast.
Q85WY9	_	Chloroplast.	Chloroplast.
Q85WZ0	M	Chloroplast.	Chloroplast.
Q85WZ6	_	Chloroplast.	Chloroplast.
Q85WZ7	_	Chloroplast.	Chloroplast.
Q85X25	_	Chloroplast.	Chloroplast.
Q85X50	_	Chloroplast.	Chloroplast.
Q85X62	M	Chloroplast. Plastid.	Chloroplast.
Q85X72	_	Chloroplast. Plastid.	Chloroplast.
Q85XY8	M	Chloroplast.	Chloroplast.
Q85ZU5	_	Chloroplast.	Chloroplast.
Q8GVA0	_	Chloroplast.	Chloroplast.
Q8HQR0	_	Chloroplast.	Chloroplast.
Q8HQS2	_	Chloroplast.	Chloroplast.
Q8HQT1	_	Chloroplast.	Chloroplast.

Q8HQT2	_	Chloroplast.	Chloroplast.
Q8HQT5	_	Chloroplast.	Chloroplast.
Q8HQT6	_	Chloroplast.	Chloroplast.
Q8HTL2	M	Chloroplast.	Chloroplast.
Q8HTL3	_	Chloroplast.	Chloroplast.
Q8HTL6	_	Chloroplast.	Chloroplast.
Q8HUG7	_	Chloroplast.	Chloroplast.
Q8HUG9	_	Chloroplast.	Chloroplast.
Q8HUH2	_	Chloroplast.	Chloroplast.
Q8HVA1	_	Chloroplast.	Chloroplast.
Q8L803	C	Chloroplast.	Chloroplast.
Q8LB10	C	Mitochondrion.	Chloroplast.
Q8LU58	_	Chloroplast.	Chloroplast.
Q8LW11	_	Chloroplast.	Chloroplast.
Q8M930	_	Chloroplast.	Chloroplast.
Q8M986	_	Chloroplast.	Chloroplast.
Q8M9L8	_	Chloroplast.	Chloroplast.
Q8M9U4	_	Chloroplast.	Chloroplast.
Q8M9U6	_	Chloroplast.	Chloroplast.
Q8M9U7	_	Chloroplast.	Chloroplast.
Q8M9U9	_	Chloroplast.	Chloroplast.
Q8M9V1	M	Mitochondrion. Plastid.	Chloroplast.
Q8M9V2	_	Chloroplast.	Chloroplast.
Q8M9V4	_	Chloroplast.	Chloroplast.
Q8M9V6	_	Chloroplast.	Chloroplast.
Q8M9V7	_	Chloroplast.	Chloroplast.

Q8M9Y6	C	Chloroplast.	Chloroplast.
Q8M9Y7	M	Chloroplast.	Chloroplast.
Q8MA11	_	Chloroplast. Nucleus.	Chloroplast.
Q8MA20	_	Chloroplast.	Chloroplast.
Q8MA31	_	Chloroplast.	Chloroplast.
Q8MA72	_	Chloroplast.	Chloroplast.
Q8MAI8	_	Chloroplast. Plastid.	Chloroplast.
Q8MAJ0	_	Chloroplast. Plastid.	Chloroplast.
Q8MC55	_	Chloroplast.	Chloroplast.
Q8MC99	M	Chloroplast.	Chloroplast.
Q8MCA4	M	Chloroplast.	Chloroplast.
Q8MCA5	_	Chloroplast.	Chloroplast.
Q8MCK8	M	Chloroplast.	Chloroplast.
Q8MCM0	M	Chloroplast.	Chloroplast.
Q8MCM2	M	Chloroplast.	Chloroplast.
Q8MCM7	M	Chloroplast.	Chloroplast.
Q8MCN2	M	Chloroplast.	Chloroplast.
Q8MCN8	M	Chloroplast.	Chloroplast.
Q8MCR5	M	Chloroplast.	Chloroplast.
Q8MCR7	M	Chloroplast.	Chloroplast.
Q8MCR9	M	Chloroplast.	Chloroplast.
Q8MD24	M	Chloroplast.	Chloroplast.
Q8MEC7	_	Chloroplast.	Chloroplast.
Q8MEC8	_	Chloroplast.	Chloroplast.
Q8MEC9	_	Chloroplast.	Chloroplast.
Q8MED5	_	Chloroplast.	Chloroplast.

Q8MEE6	_	Chloroplast.	Chloroplast.
Q8MEH7	_	Chloroplast.	Chloroplast.
Q8MEW7	_	Chloroplast.	Chloroplast.
Q8MEW9	M	Chloroplast.	Chloroplast.
Q8MEX3	_	Chloroplast.	Chloroplast.
Q8MEX4	M	Chloroplast.	Chloroplast.
Q8MEX7	_	Chloroplast.	Chloroplast.
Q8MEX8	_	Chloroplast.	Chloroplast.
Q8MEY0	_	Chloroplast.	Chloroplast.
Q8MEY1	_	Chloroplast.	Chloroplast.
Q8RU60	_	Chloroplast.	Chloroplast.
Q8RXX5	C	Chloroplast.	Chloroplast.
Q8S8U0	C	Chloroplast.	Chloroplast.
Q8S8U3	_	Chloroplast.	Chloroplast.
Q8S8U7	S	Chloroplast.	Chloroplast.
Q8S8U9	M	Chloroplast.	Chloroplast.
Q8S8V4	_	Chloroplast.	Chloroplast.
Q8S8V6	M	Mitochondrion. Plastid.	Chloroplast.
Q8S8V7	_	Chloroplast.	Chloroplast.
Q8S8W2	_	Chloroplast.	Chloroplast.
Q8S8Y1	_	Chloroplast.	Chloroplast.
Q8S8Y4	S	Chloroplast. Plastid.	Chloroplast.
Q8S8Y5	M	Chloroplast.	Chloroplast.
Q8S8Y6	_	Chloroplast.	Chloroplast.
Q8SE90	_	Chloroplast.	Chloroplast.
Q8SKV2	_	Chloroplast.	Chloroplast.

Q8SM04	M	Chloroplast.	Chloroplast.
Q8SM20	_	Chloroplast.	Chloroplast.
Q8SM90	_	Chloroplast.	Chloroplast.
Q8W463	C	Chloroplast.	Chloroplast.
Q8W7A1	_	Chloroplast.	Chloroplast.
Q8W8E6	_	Chloroplast.	Chloroplast.
Q8W8V0	_	Mitochondrion. Plastid.	Chloroplast.
Q8WHL6	_	Chloroplast.	Chloroplast.
Q8WHM4	_	Chloroplast.	Chloroplast.
Q8WHM8	_	Chloroplast.	Chloroplast.
Q8WHX3	_	Chloroplast. Plastid.	Chloroplast.
Q8WHY2	_	Chloroplast.	Chloroplast.
Q8WHY3	_	Chloroplast.	Chloroplast.
Q8WHZ0	M	Chloroplast.	Chloroplast.
Q8WHZ7	_	Chloroplast.	Chloroplast.
Q8WHZ9	_	Chloroplast.	Chloroplast.
Q8WI25	M	Chloroplast. Nucleus.	Chloroplast.
Q8WIJ9	_	Chloroplast.	Chloroplast.
Q8WIT2	_	Chloroplast.	Chloroplast.
Q8WIV4	_	Chloroplast.	Chloroplast.
Q8WIV8	_	Chloroplast.	Chloroplast.
Q8WJ31	_	Chloroplast.	Chloroplast.
Q8WJ37	_	Chloroplast.	Chloroplast.
Q8WJP1	_	Chloroplast.	Chloroplast.
Q8WJP3	_	Chloroplast.	Chloroplast.

Q8WJR4	_	Chloroplast.	Chloroplast.
Q8WJR5	_	Chloroplast.	Chloroplast.
Q8WJV2	M	Chloroplast.	Chloroplast.
Q8WKE7	_	Chloroplast.	Chloroplast.
Q8WKL8	_	Chloroplast.	Chloroplast.
Q8WKN0	_	Chloroplast.	Chloroplast.
Q8WKP4	_	Chloroplast.	Chloroplast.
Q94KR7	C	Chloroplast.	Chloroplast.
Q94PL3	_	Chloroplast.	Chloroplast.
Q95BX6	_	Chloroplast.	Chloroplast.
Q95DP2	_	Chloroplast.	Chloroplast.
Q95DR2	_	Chloroplast.	Chloroplast.
Q95E84	_	Chloroplast.	Chloroplast.
Q95EA5	_	Chloroplast.	Chloroplast.
Q95ED6	_	Chloroplast.	Chloroplast.
Q95ED9	_	Chloroplast.	Chloroplast.
Q95EE5	_	Chloroplast.	Chloroplast.
Q95EF3	M	Chloroplast.	Chloroplast.
Q95EF8	_	Chloroplast.	Chloroplast.
Q95GM3	_	Chloroplast.	Chloroplast.
Q95GM7	_	Chloroplast.	Chloroplast.
Q95GN5	_	Chloroplast.	Chloroplast.
Q95GN7	_	Chloroplast.	Chloroplast.
Q95GT2	_	Chloroplast.	Chloroplast.
Q95GT7	_	Chloroplast.	Chloroplast.
Q95H44	S	Chloroplast. Plastid.	Chloroplast.

Q95H50	M	Mitochondrion. Plastid.	Chloroplast.
Q95H51	_	Chloroplast.	Chloroplast.
Q95H56	_	Chloroplast. Plastid.	Chloroplast.
Q95H60	_	Chloroplast. Plastid.	Chloroplast.
Q95H61	M	Chloroplast.	Chloroplast.
Q95H62	_	Chloroplast. Plastid.	Chloroplast.
Q9AWA5	C	Chloroplast.	Chloroplast.
Q9BA08	_	Chloroplast.	Chloroplast.
Q9BBN0	_	Chloroplast.	Chloroplast.
Q9BBN7	_	Chloroplast.	Chloroplast.
Q9BBN8	_	Chloroplast. Plastid.	Chloroplast.
Q9BBP3	S	Chloroplast.	Chloroplast.
Q9BBP9	M	Chloroplast.	Chloroplast.
Q9BBR0	M	Chloroplast.	Chloroplast.
Q9BBR1	M	Chloroplast.	Chloroplast.
Q9BBR2	_	Chloroplast. Plastid.	Chloroplast.
Q9BBS6	_	Chloroplast.	Chloroplast.
Q9BBS7	_	Chloroplast.	Chloroplast.
Q9BBS8	_	Chloroplast. Nucleus.	Chloroplast.
Q9BBS9	_	Chloroplast.	Chloroplast.
Q9BBT3	C	Chloroplast.	Chloroplast.
Q9BBT5	M	Chloroplast.	Chloroplast.
Q9BCJ9	_	Chloroplast.	Chloroplast.
Q9C5U8	C	Chloroplast.	Chloroplast.
Q9FKG3	C	Chloroplast.	Chloroplast.

Q9FS90	M	Chloroplast.	Chloroplast.
Q9FSD9	M	Chloroplast.	Chloroplast.
Q9FV46	C	Chloroplast.	Chloroplast.
Q9FVC8	C	Chloroplast.	Chloroplast.
Q9G1H4	_	Chloroplast.	Chloroplast.
Q9GE26	_	Mitochondrion. Plastid.	Chloroplast.
Q9GF29	_	Chloroplast.	Chloroplast.
Q9GF34	_	Chloroplast.	Chloroplast.
Q9GF37	_	Chloroplast.	Chloroplast.
Q9GF40	_	Chloroplast.	Chloroplast.
Q9GF47	_	Chloroplast.	Chloroplast.
Q9GF55	_	Chloroplast.	Chloroplast.
Q9GF61	_	Chloroplast.	Chloroplast.
Q9GFK0	_	Mitochondrion. Plastid.	Chloroplast.
Q9GFK8	_	Mitochondrion. Plastid.	Chloroplast.
Q9GFL3	_	Mitochondrion. Plastid.	Chloroplast.
Q9GFL8	_	Mitochondrion. Plastid.	Chloroplast.
Q9GFM9	_	Mitochondrion. Plastid.	Chloroplast.
Q9GFN8	_	Chloroplast.	Chloroplast.
Q9GGE2	M	Chloroplast.	Chloroplast.
Q9GHB1	_	Chloroplast.	Chloroplast.
Q9GHE1	_	Chloroplast.	Chloroplast.
Q9GHE5	_	Chloroplast.	Chloroplast.

Q9GI85	_	Chloroplast.	Chloroplast.
Q9LFV6	C	Chloroplast. Mitochondrion. Plastid.	Chloroplast. Mitochondrion.
Q9LLC1	C	Chloroplast.	Chloroplast.
Q9LS02	C	Chloroplast.	Chloroplast.
Q9M3I5	_	Chloroplast. Plastid.	Chloroplast.
Q9M3K7	_	Chloroplast.	Chloroplast.
Q9M3M1	_	Chloroplast. Plastid.	Chloroplast.
Q9M3N0	_	Chloroplast.	Chloroplast.
Q9M401	C	Chloroplast.	Chloroplast.
Q9M4B8	M	Chloroplast.	Chloroplast.
Q9MDA1	_	Chloroplast.	Chloroplast.
Q9MDK3	C	Chloroplast.	Chloroplast.
Q9MSA7	_	Chloroplast.	Chloroplast.
Q9MSR8	_	Chloroplast.	Chloroplast.
Q9MST4	M	Chloroplast.	Chloroplast.
Q9MSV4	M	Chloroplast.	Chloroplast.
Q9MTH6	_	Chloroplast. Plastid.	Chloroplast.
Q9MTH9	S	Chloroplast. Plastid.	Chloroplast.
Q9MTI0	S	Chloroplast. Plastid.	Chloroplast.
Q9MTI3	M	Chloroplast.	Chloroplast.
Q9MTI6	_	Chloroplast.	Chloroplast.
Q9MTJ2	C	Chloroplast.	Chloroplast.
Q9MTK1	M	Chloroplast.	Chloroplast.
Q9MTM2	_	Chloroplast.	Chloroplast.
Q9MTM4	M	Chloroplast.	Chloroplast.

		Nucleus.	
Q9MTP4	C	Chloroplast. Plastid.	Chloroplast.
Q9MTP5	S	Chloroplast. Plastid.	Chloroplast.
Q9MTQ1	_	Chloroplast.	Chloroplast.
Q9MUL3	S	Chloroplast.	Chloroplast.
Q9MUL4	S	Chloroplast.	Chloroplast.
Q9MUL8	M	Chloroplast.	Chloroplast.
Q9MUM3	S	Chloroplast.	Chloroplast.
Q9MUM5	_	Chloroplast.	Chloroplast.
Q9MUM8	S	Chloroplast.	Chloroplast.
Q9MUN0	M	Chloroplast.	Chloroplast.
Q9MUN2	M	Chloroplast. Plastid.	Chloroplast.
Q9MUP2	_	Chloroplast.	Chloroplast.
Q9MUP5	C	Chloroplast.	Chloroplast.
Q9MUQ9	S	Chloroplast. Plastid.	Chloroplast.
Q9MUR6	_	Chloroplast.	Chloroplast.
Q9MUR9	M	Chloroplast.	Chloroplast.
Q9MUS7	M	Chloroplast.	Chloroplast.
Q9MUT8	_	Chloroplast.	Chloroplast.
Q9MUT9	_	Chloroplast.	Chloroplast.
Q9MUU4	_	Chloroplast.	Chloroplast.
Q9MUU7	_	Chloroplast.	Chloroplast.
Q9MUV0	_	Chloroplast.	Chloroplast.
Q9MUV1	M	Chloroplast.	Chloroplast.
Q9MUZ3	_	Chloroplast.	Chloroplast.
Q9MUZ4	_	Chloroplast.	Chloroplast.

Q9MUZ5	_	Chloroplast.	Chloroplast.
Q9MV46	_	Chloroplast.	Chloroplast.
Q9MV48	_	Chloroplast.	Chloroplast.
Q9MV49	_	Chloroplast.	Chloroplast.
Q9MV51	_	Chloroplast.	Chloroplast.
Q9MVD8	_	Chloroplast.	Chloroplast.
Q9MVW4	M	Chloroplast.	Chloroplast.
Q9S795	_	Chloroplast. Mitochondrion. Peroxisome.	Chloroplast.
Q9SA18	C	Chloroplast.	Chloroplast.
Q9SAK2	C	Chloroplast.	Chloroplast.
Q9SCY0	C	Chloroplast.	Chloroplast.
Q9SIK1	_	Chloroplast.	Chloroplast.
Q9SM59	C	Chloroplast.	Chloroplast.
Q9SMG1	M	Chloroplast.	Chloroplast.
Q9SS38	_	Chloroplast. Cytoplasm. Mitochondrion.	Chloroplast.
Q9SYL9	C	Chloroplast.	Chloroplast.
Q9T390	_	Chloroplast.	Chloroplast.
Q9T399	_	Chloroplast.	Chloroplast.
Q9T3P6	_	Chloroplast.	Chloroplast.
Q9T467	M	Chloroplast.	Chloroplast.
Q9T4F2	_	Chloroplast.	Chloroplast.
Q9T4F6	_	Chloroplast.	Chloroplast.
Q9TIB6	M	Chloroplast.	Chloroplast.
Q9TK08	M	Chloroplast.	Chloroplast.

Q9TKB6	_	Chloroplast.	Chloroplast.
Q9TKD1	M	Chloroplast.	Chloroplast.
Q9TKD3	_	Chloroplast.	Chloroplast.
Q9TKI5	M	Chloroplast.	Chloroplast.
Q9TKP0	M	Chloroplast.	Chloroplast.
Q9TKP8	_	Chloroplast.	Chloroplast.
Q9TKS4	M	Chloroplast.	Chloroplast.
Q9TKV3	S	Chloroplast.	Chloroplast.
Q9TKV8	S	Chloroplast.	Chloroplast.
Q9TKX3	_	Chloroplast. Mitochondrion.	Chloroplast.
Q9TKX4	M	Chloroplast.	Chloroplast.
Q9TKX9	S	Chloroplast. Plastid.	Chloroplast.
Q9TKZ4	_	Chloroplast.	Chloroplast.
Q9TKZ6	_	Chloroplast.	Chloroplast.
Q9TL04	_	Chloroplast.	Chloroplast.
Q9TL17	_	Chloroplast.	Chloroplast.
Q9TL22	_	Chloroplast.	Chloroplast.
Q9TL23	M	Chloroplast.	Chloroplast.
Q9TL25	S	Chloroplast.	Chloroplast.
Q9TL29	_	Chloroplast.	Chloroplast.
Q9TLD8	_	Chloroplast.	Chloroplast.
Q9TM55	_	Mitochondrion. Plastid.	Chloroplast.
Q9TMB2	_	Chloroplast.	Chloroplast.
Q9TMC8	_	Chloroplast.	Chloroplast.
Q9TN87	_	Chloroplast.	Chloroplast.

Q9TN89	_	Chloroplast.	Chloroplast.
Q9TNA1	_	Chloroplast.	Chloroplast.
Q9TNB2	_	Chloroplast.	Chloroplast.
Q9XGS0	C	Chloroplast.	Chloroplast.
Q9XGX5	C	Chloroplast.	Chloroplast.
Q9XPP8	_	Chloroplast.	Chloroplast.
Q9XPS8	_	Chloroplast. Nucleus. Plastid.	Chloroplast.
Q9XPS9	_	Chloroplast. Plastid.	Chloroplast.
Q9XR19	_	Chloroplast.	Chloroplast.
Q9ZP40	C	Chloroplast.	Chloroplast.
A2Y4S9	_	Mitochondrion.	Mitochondrion.
O04866	M	Mitochondrion.	Mitochondrion.
O05000	S	Mitochondrion.	Mitochondrion.
O22642	_	Mitochondrion.	Mitochondrion.
O23936	M	Cytoplasm. Mitochondrion.	Mitochondrion.
O49066	M	Mitochondrion.	Mitochondrion.
O49850	M	Mitochondrion.	Mitochondrion.
O49954	M	Mitochondrion.	Mitochondrion.
O64966	_	Endoplasmic reticulum.	Endoplasmic reticulum.
O80433	M	Cytoplasm. Mitochondrion.	Mitochondrion.
O80988	M	Mitochondrion.	Mitochondrion.
O81235	M	Mitochondrion.	Mitochondrion.
O81796	M	Mitochondrion.	Mitochondrion.
P00051	_	Mitochondrion.	Mitochondrion.

P00052	_	Mitochondrion.	Mitochondrion.
P00053	_	Mitochondrion.	Mitochondrion.
P00054	_	Mitochondrion.	Mitochondrion.
P00056	_	Mitochondrion.	Mitochondrion.
P00057	_	Mitochondrion.	Mitochondrion.
P00058	_	Mitochondrion.	Mitochondrion.
P00059	_	Mitochondrion.	Mitochondrion.
P00060	_	Mitochondrion.	Mitochondrion.
P00061	_	Mitochondrion.	Mitochondrion.
P00062	_	Mitochondrion.	Mitochondrion.
P00063	_	Mitochondrion.	Mitochondrion.
P00064	_	Mitochondrion.	Mitochondrion.
P00065	_	Mitochondrion.	Mitochondrion.
P00066	_	Mitochondrion.	Mitochondrion.
P00067	_	Mitochondrion.	Mitochondrion.
P00068	_	Mitochondrion.	Mitochondrion.
P00069	_	Mitochondrion.	Mitochondrion.
P00070	_	Mitochondrion.	Mitochondrion.
P00071	_	Mitochondrion.	Mitochondrion.
P00072	_	Mitochondrion.	Mitochondrion.
P00073	_	Mitochondrion.	Mitochondrion.
P00074	_	Mitochondrion.	Mitochondrion.
P00075	C	Mitochondrion.	Mitochondrion.
P00412	S	Mitochondrion.	Mitochondrion.
P00413	S	Mitochondrion.	Mitochondrion.
P04373	S	Mitochondrion.	Mitochondrion.

P05488	_	Mitochondrion.	Mitochondrion.
P05490	S	Mitochondrion.	Mitochondrion.
P05491	_	Mitochondrion.	Mitochondrion.
P05492	_	Mitochondrion.	Mitochondrion.
P05493	_	Mitochondrion.	Mitochondrion.
P05494	_	Mitochondrion.	Mitochondrion.
P05495	_	Mitochondrion.	Mitochondrion.
P05500	_	Mitochondrion.	Mitochondrion.
P07506	_	Mitochondrion.	Mitochondrion.
P07924	_	Mitochondrion.	Mitochondrion.
P07925	_	Mitochondrion.	Mitochondrion.
P08681	S	Mitochondrion.	Mitochondrion.
P08742	_	Mitochondrion.	Mitochondrion.
P08743	_	Mitochondrion.	Mitochondrion.
P08744	_	Mitochondrion.	Mitochondrion.
P08977	_	Mitochondrion.	Mitochondrion.
P0C520	_	Mitochondrion.	Mitochondrion.
P0C521	_	Mitochondrion.	Mitochondrion.
P0C522	_	Mitochondrion.	Mitochondrion.
P12786	_	Mitochondrion.	Mitochondrion.
P12857	C	Mitochondrion.	Mitochondrion.
P12862	_	Mitochondrion.	Mitochondrion.
P14578	_	Mitochondrion.	Mitochondrion.
P14875	M	Mitochondrion.	Mitochondrion.
P15451	_	Mitochondrion.	Mitochondrion.
P15758	_	Mitochondrion.	Mitochondrion.

P16048	M	Mitochondrion.	Mitochondrion.
P16265	S	Mitochondrion.	Mitochondrion.
P17614	C	Chloroplast. Mitochondrion.	Mitochondrion.
P18260	_	Mitochondrion.	Mitochondrion.
P18630	S	Mitochondrion.	Mitochondrion.
P20113	S	Mitochondrion.	Mitochondrion.
P22201	_	Mitochondrion.	Mitochondrion.
P23209	M	Mitochondrion.	Mitochondrion.
P24459	_	Mitochondrion.	Mitochondrion.
P24794	_	Mitochondrion.	Mitochondrion.
P25083	_	Mitochondrion.	Mitochondrion.
P25855	M	Mitochondrion.	Mitochondrion.
P26846	S	Mitochondrion.	Mitochondrion.
P26847	S	Mitochondrion.	Mitochondrion.
P26848	S	Mitochondrion.	Mitochondrion.
P26850	S	Mitochondrion.	Mitochondrion.
P26853	S	Mitochondrion.	Mitochondrion.
P26856	M	Mitochondrion.	Mitochondrion.
P26857	S	Mitochondrion.	Mitochondrion.
P26859	M	Mitochondrion.	Mitochondrion.
P26860	_	Mitochondrion.	Mitochondrion.
P26861	S	Mitochondrion.	Mitochondrion.
P26862	_	Mitochondrion. Plastid.	Mitochondrion.
P26864	M	Mitochondrion.	Mitochondrion.
P26865	_	Mitochondrion.	Mitochondrion.

P26866	M	Mitochondrion. Plastid.	Mitochondrion.
P26869	_	Mitochondrion.	Mitochondrion.
P26870	_	Mitochondrion.	Mitochondrion.
P26871	C	Mitochondrion. Plastid.	Mitochondrion.
P26872	_	Mitochondrion.	Mitochondrion.
P26873	M	Mitochondrion.	Mitochondrion.
P26874	M	Cytoplasm. Plastid.	Mitochondrion.
P27062	S	Mitochondrion.	Mitochondrion.
P27070	_	Mitochondrion. Plastid.	Mitochondrion.
P27080	_	Mitochondrion.	Mitochondrion.
P27084	M	Mitochondrion.	Mitochondrion.
P27527	M	Cytoplasm. Plastid.	Mitochondrion.
P27572	S	Mitochondrion.	Mitochondrion.
P27754	_	Mitochondrion.	Mitochondrion.
P27928	_	Mitochondrion.	Mitochondrion.
P28520	_	Chloroplast.	Mitochondrion.
P29057	_	Endoplasmic reticulum.	Endoplasmic reticulum.
P29185	M	Mitochondrion.	Mitochondrion.
P29380	_	Mitochondrion.	Mitochondrion.
P29677	M	Mitochondrion.	Mitochondrion.
P29685	C	Chloroplast. Mitochondrion.	Mitochondrion.
P31023	M	Cytoplasm. Mitochondrion.	Mitochondrion.
P31167	_	Mitochondrion.	Mitochondrion.

P31691	C	Mitochondrion.	Mitochondrion.
P31692	M	Mitochondrion.	Mitochondrion.
P35017	M	Mitochondrion.	Mitochondrion.
P37399	M	Chloroplast. Mitochondrion.	Mitochondrion.
P37841	C	Mitochondrion.	Mitochondrion.
P37900	M	Mitochondrion.	Mitochondrion.
P41978	M	Mitochondrion.	Mitochondrion.
P41979	M	Mitochondrion.	Mitochondrion.
P41980	M	Mitochondrion.	Mitochondrion.
P42027	M	Extracell. Mitochondrion.	Mitochondrion.
P42056	_	Mitochondrion.	Mitochondrion.
P42793	_	Mitochondrion.	Mitochondrion.
P46274	_	Mitochondrion.	Mitochondrion.
P46485	M	Mitochondrion.	Mitochondrion.
P46487	M	Mitochondrion.	Mitochondrion.
P46740	_	Mitochondrion.	Mitochondrion.
P46742	S	Mitochondrion.	Mitochondrion.
P46744	C	Mitochondrion. Plastid.	Mitochondrion.
P46745	_	Mitochondrion.	Mitochondrion.
P46747	_	Mitochondrion.	Mitochondrion.
P46750	C	Cytoplasm.	Mitochondrion.
P46751	_	Mitochondrion. Plastid.	Mitochondrion.
P46752	M	Mitochondrion.	Mitochondrion.
P46773	M	Mitochondrion.	Mitochondrion.

P46801	_	Mitochondrion.	Mitochondrion.
P48857	_	Mitochondrion. Plastid.	Mitochondrion.
P49357	M	Mitochondrion.	Mitochondrion.
P49358	M	Mitochondrion.	Mitochondrion.
P49359	M	Mitochondrion.	Mitochondrion.
P49361	M	Mitochondrion.	Mitochondrion.
P49362	M	Mitochondrion.	Mitochondrion.
P49363	M	Cytoplasm. Mitochondrion.	Mitochondrion.
P49386	M	Mitochondrion.	Mitochondrion.
P49387	_	Mitochondrion.	Mitochondrion.
P49388	_	Mitochondrion.	Mitochondrion.
P49389	_	Mitochondrion.	Mitochondrion.
P50433	M	Mitochondrion.	Mitochondrion.
P50892	_	Mitochondrion. Plastid.	Mitochondrion.
P50893	C	Cytoplasm. Plastid.	Mitochondrion.
P51132	C	Mitochondrion.	Mitochondrion.
P51135	C	Mitochondrion.	Mitochondrion.
P51409	_	Mitochondrion.	Mitochondrion.
P52901	C	Mitochondrion.	Mitochondrion.
P52903	C	Mitochondrion.	Mitochondrion.
P52904	M	Mitochondrion.	Mitochondrion.
P54260	M	Cytoplasm. Mitochondrion.	Mitochondrion.
P60096	_	Mitochondrion. Plastid.	Mitochondrion.

P60097	_	Mitochondrion. Plastid.	Mitochondrion.
P60098	_	Mitochondrion. Plastid.	Mitochondrion.
P60099	_	Mitochondrion. Plastid.	Mitochondrion.
P60159	S	Mitochondrion.	Mitochondrion.
P60160	S	Mitochondrion.	Mitochondrion.
P60621	M	Mitochondrion.	Mitochondrion.
P62772	_	Mitochondrion.	Mitochondrion.
P62773	_	Mitochondrion.	Mitochondrion.
P68526	_	Mitochondrion.	Mitochondrion.
P68527	_	Mitochondrion.	Mitochondrion.
P68535	_	Chloroplast.	Mitochondrion.
P68536	_	Chloroplast.	Mitochondrion.
P68539	_	Mitochondrion.	Mitochondrion.
P68540	_	Mitochondrion.	Mitochondrion.
P68541	_	Mitochondrion.	Mitochondrion.
P68542	_	Mitochondrion.	Mitochondrion.
P80261	_	Mitochondrion.	Mitochondrion.
P83372	M	Cytoplasm. Mitochondrion.	Mitochondrion.
P83373	M	Mitochondrion.	Mitochondrion.
P83483	M	Chloroplast. Mitochondrion.	Mitochondrion.
P83484	M	Chloroplast. Mitochondrion.	Mitochondrion.
P92532	_	Mitochondrion. Plastid.	Mitochondrion.

P92547	M	Mitochondrion.	Mitochondrion.
P92549	_	Mitochondrion.	Mitochondrion.
P92557	_	Mitochondrion. Plastid.	Mitochondrion.
P92969	M	Chloroplast. Mitochondrion. Plastid.	Mitochondrion.
P93032	M	Mitochondrion.	Mitochondrion.
P93256	M	Cytoplasm. Mitochondrion.	Mitochondrion.
P93285	S	Mitochondrion.	Mitochondrion.
P93298	_	Mitochondrion.	Mitochondrion.
P93306	M	Mitochondrion.	Mitochondrion.
P93311	M	Mitochondrion.	Mitochondrion.
P93313	S	Mitochondrion.	Mitochondrion.
P93401	S	Mitochondrion.	Mitochondrion.
P98012	_	Mitochondrion.	Mitochondrion.
Q00583	_	Endoplasmic reticulum.	Endoplasmic reticulum.
Q01859	M	Chloroplast. Mitochondrion.	Mitochondrion.
Q01902	_	Mitochondrion. Plastid.	Mitochondrion.
Q01915	_	Mitochondrion.	Mitochondrion.
Q04050	_	Mitochondrion.	Mitochondrion.
Q04654	S	Mitochondrion.	Mitochondrion.
Q04715	_	Mitochondrion.	Mitochondrion.
Q05143	M	Mitochondrion.	Mitochondrion.
Q06735	_	Mitochondrion.	Mitochondrion.

Q0DI31	_	Mitochondrion.	Mitochondrion.
Q31708	M	Mitochondrion.	Mitochondrion.
Q31720	_	Mitochondrion.	Mitochondrion.
Q33994	_	Mitochondrion.	Mitochondrion.
Q34011	_	Mitochondrion.	Mitochondrion.
Q35322	_	Mitochondrion.	Mitochondrion.
Q36450	M	Mitochondrion.	Mitochondrion.
Q36518	S	Mitochondrion.	Mitochondrion.
Q36664	S	Mitochondrion.	Mitochondrion.
Q36665	C	Mitochondrion. Plastid.	Mitochondrion.
Q37625	S	Mitochondrion.	Mitochondrion.
Q37626	S	Mitochondrion.	Mitochondrion.
Q37627	S	Mitochondrion.	Mitochondrion.
Q37787	_	Mitochondrion.	Mitochondrion.
Q41346	_	Mitochondrion. Nucleus.	Mitochondrion.
Q41629	_	Mitochondrion.	Mitochondrion.
Q41630	_	Mitochondrion.	Mitochondrion.
Q41898	M	Mitochondrion.	Mitochondrion. Mitochondrion.
Q42525	S	Chloroplast. Mitochondrion.	Mitochondrion.
Q42560	_	Cytoplasm. Mitochondrion.	Cytoplasm. Mitochondrion.
Q42777	M	Mitochondrion.	Mitochondrion.
Q43008	S	Mitochondrion.	Mitochondrion.
Q43175	M	Cytoplasm. Mitochondrion.	Mitochondrion.

Q56XE8	C	Mitochondrion.	Mitochondrion.
Q5M729	M	Mitochondrion.	Mitochondrion.
Q5YLB5	C	Chloroplast. Mitochondrion.	Chloroplast. Mitochondrion.
Q6K548	_	Mitochondrion.	Mitochondrion.
Q8H1Y0	M	Mitochondrion.	Mitochondrion.
Q8L6J5	M	Chloroplast. Mitochondrion. Plastid.	Mitochondrion.
Q8L7B5	M	Mitochondrion.	Mitochondrion.
Q8LAD2	M	Cytoplasm. Mitochondrion.	Mitochondrion.
Q8LBZ7	M	Cytoplasm. Mitochondrion.	Mitochondrion.
Q8LFC0	M	Mitochondrion.	Mitochondrion.
Q8LFT2	C	Mitochondrion.	Mitochondrion.
Q8LPW2	M	Mitochondrion.	Mitochondrion.
Q8RWN9	M	Mitochondrion.	Mitochondrion.
Q8VWF8	_	Chloroplast. Mitochondrion. Plastid.	Chloroplast. Mitochondrion.
Q93Y94	M	Chloroplast. Mitochondrion. Plastid.	Mitochondrion.
Q93ZM7	M	Chloroplast.	Mitochondrion.
Q945K7	M	Chloroplast. Mitochondrion.	Mitochondrion.
Q94B78	M	Mitochondrion.	Mitochondrion.
Q95747	S	Mitochondrion.	Mitochondrion.
Q95748	_	Mitochondrion.	Mitochondrion.

Q95749	M	Mitochondrion.	Mitochondrion.
Q95869	_	Chloroplast.	Mitochondrion.
Q96007	S	Mitochondrion.	Mitochondrion.
Q96008	_	Mitochondrion. Plastid.	Mitochondrion.
Q96033	_	Mitochondrion. Plastid.	Mitochondrion.
Q96253	S	Mitochondrion.	Mitochondrion.
Q9C641	M	Mitochondrion.	Mitochondrion.
Q9FMV1	M	Mitochondrion.	Mitochondrion.
Q9FV51	C	Chloroplast. Mitochondrion.	Chloroplast. Mitochondrion.
Q9LFV6	C	Chloroplast. Mitochondrion. Plastid.	Chloroplast. Mitochondrion.
Q9LJL3	C	Chloroplast. Mitochondrion.	Chloroplast. Mitochondrion.
Q9LKA3	M	Mitochondrion.	Mitochondrion.
Q9LPS1	S	Mitochondrion.	Mitochondrion.
Q9M1D3	_	Cytoplasm. Mitochondrion.	Mitochondrion.
Q9M5K2	M	Cytoplasm. Mitochondrion.	Mitochondrion.
Q9M5K3	M	Cytoplasm. Mitochondrion.	Mitochondrion.
Q9MF82	_	Mitochondrion. Plastid.	Mitochondrion.
Q9SIB9	C	Cytoplasm. Mitochondrion.	Mitochondrion.
Q9SMX3	_	Mitochondrion.	Mitochondrion.
Q9SRH5	_	Mitochondrion.	Mitochondrion.

Q9SVM8	M	Mitochondrion.	Mitochondrion.
Q9SZJ5	M	Mitochondrion.	Mitochondrion.
Q9TC96	_	Mitochondrion.	Mitochondrion.
Q9XGY5	_	Mitochondrion.	Mitochondrion.
Q9ZP06	M	Mitochondrion.	Mitochondrion.
Q9ZPX5	M	Mitochondrion.	Mitochondrion.
Q9ZT91	C	Mitochondrion.	Mitochondrion.
