# Supplementary material for: Plant-mPLoc: A Top-Down Strategy to Augment the Power for Predicting Plant Protein Subcellular Localization
Source: PLoS One. 2010 Jun 28;5(6):e11335. doi: 10.1371/journal.pone.0011335 (PMC2893129; doi:10.1371/journal.pone.0011335)
Supplement: Table S4 — The degenerate testing dataset used for comparing the performance between Predotar (Small et al., Proteomics 2004, 4: 1581–1590) and Plant-mPLoc of this paper. The dataset contains 381 plant proteins classified into 3 subcellular locations: (1) endoplasmic reticulum, (2) mitochondrion, and (3) plastid. To avoid bias, none of the proteins included here occurs in the training dataset of TargetP, nor in the training dataset of Plant-mPLoc. See the text of the paper for further explanation. (0.25 MB PDF) [file pone.0011335.s004.pdf]

Table S4. The degenerate testing dataset used for comparing the performance between Predotar (Small et al., Proteomics 2004, 4: 1581-1590) and Plant-mPLoc of this paper. The dataset contains 381 plant proteins classified into 3 subcellular locations: (1) endoplasmic reticulum, (2) mitochondrion, and (3) plastid. To avoid bias, none of the proteins included here occurs in the training dataset of TargetP, nor in the training dataset of Plant-mPLoc. See the text of the paper for further explanation.

---

(1) 48 endoplasmic reticulum proteins

```
>A2X8W3 | HMDH1_ORYSI
MDVRRGGGGGRIVGAARRALTWGALPLPMRITNGLAMVSLVLSSCDLLRLCSDRERPLGG
REFATVVCQLASVVYLLSLFAHPDAPATTTGDDDDGQGGSRRARPAAAEPAPMHGHGGGM
MEADDEEIVAAVASGALPSHRLESRLGDCRRAARLRREALRRVTGRGVEGLPFDGMDYQA
ILGQCCEMPVGYVQLPVGVAGPLLLDGREYHVPMATTEGCLVASVNRGCRAISASGGAFS
VLLRDAMSRAPAVKLPSAMRAAELKAFAEAPANFELLAAVFNRSSRFGRLQDIRCALAGR
NLYMRFSCITGDAMGMNMVSKGVENVLGYLQNVFPDMDVISVSGNYCSDKKPTAVNWIEG
RGKSVVCEAIIKGDVVQKVLKTTVEKLVELNIIKNLAGSAVAGALGGFNAHASNIVTALF
IATGQDPAQNVESSQCITMLEEVDDGDDLHISVTMPSIEVGTIGGGTCLASQAACLNLLG
VKGSNHGSPGANAKRLATIVAGSVLAGELSLLAALASGHLVKSHMMYNRSSKDVAKAAS
>O24581 | BIP3_MAIZE
MDRVRGSAFLLGVLLLAGSLFAFSVAKEETKKLGTVIGIDLGTTYSCVGVYKNGHVEIIAN
DQGNRITPSWVAFTDSERLIGEAAKNQAAVNPERTIFDVKRLIGRKFQDKEVQRDMKLVP
YKIINKDGKPYIQVKIKDGENKVFSPEEISAMILGKMKDTAEAYLGKKINDAVVTVPAYF
NDAQRQATKDAGVIAGLNVARIINEPTAAAIAYGLDKKGGEKNILVFDLGGGTFDVSILT
IDNGVFEVLATNGDTHLGGEDFDQRIMEYFIKLIKKKYSKDISKDNRALGKLRREAERAK
RALSNQHQVRVEIESLFDGTDFSEPLTRARFEELNNDLFRKTMGPVKKAMEDAGLEKSQI
HEIVLVGGSTRIPKVQQLLKDYFNGKEPNKGVNPDEAVAFGAAVQGSILSGEGGDETKDI
LLLDVAPLTLGIETVGGVMTKLIPRNTVIPTKKSQVFTTYQDQQTTVSIQVFEGERSMTK
DCRLLGKFDLNGIPSAPRGTPQIEVTFEVDANGILNVKAEDKGTGKSEKITITNEKGRLS
QEEIDRMVREAEEFAEEDKKVKERIDARNQLETYVYNMKNTVGDKDKLADKLEAEEKEKV
EEALKEALEWLDDNQSAEKEDYEEKLKEVEAVCNPIVSAVYQRSGGAPGGDADGGVDDDH
DEL
>O24594 | HMDH_MAIZE
MEVRGGVGQGSAARHPPAPEPSRAAARVQAGDALPLPIRHTNLIFSALFAASLAYLMRRW
REKIRSSTPLHAVGLAEMLAIFGLVASLIYLLSFFGIAFVQSIVSSGDDDEDFLVGSGSS
GSAAAPSRQHAQAPAPCELLGSPAAAPEKMPEDDEEIVASVVAGKVPSYALEARLGDCRR
AAGIRREALRRITGRDIEGLPLDGFDYASILGQCCELPVGYVQLPVGVAGPLLLDGRRFY
LPMATTEGCLVASTNRGCKAIAESGGATSVVLRDAMTRAPVARFPTARRAAELKAFLEDP
ANFDTLSVVFNRSSRFARLQGVQCAMAGRNLYMRFSCSTGDAMGMNMVSKGVQNVLDFLQ
DDFHDMDVISISGNFCSDKKPSAVNWIEGRGKSVVCEAVIGEEVVKKVLKTDVQSLVELN
TIKNLAGSAVAGALGGFNAHASNIVTAIFIATGQDPAQNVESSHCITMLEPVNAGRDLHI
SVTMPSIEVGTVGGGTQLASQSACLDLLGVRGASRDRPGSNARLLATVVAGGVLAGELSL
LSALAAGQLVKSHMKYNRSSKDVSSTTATEKTRQREVDV
```

>O64966 | HMDH1_GOSHI
METHRRSSTNSIRSHKPARPIALEDDSTKASDALPLPLYLTNAVFFTLFFSAVYFLLCRW
REKIRSSTPLHVVTFSEIVAILASVASFIYLLGFFGIDFVQSLVLRPSADVWATEDDEVE
SEVLLRNEDARHVPCGQALDRSIRSLQPPEPIVTAEKVFDEMPVTVMTEEDEEIIRSVVC
GMTPSYSLESKLDDCKRAAAIRREALQRITGKSLSGLPLDGFDYESILGQCCEMPVGYEQ
IPVGIAGPLLLNGREYSVPMATTEGCLVASTNRGCKAIHLSGGATSVLLRDGMTRAPVVR
FGTAKRAADLKLYLEDPENFETLACVFNRSSRFARLQSIKCAIAGKNLYLRFSCFTGDAM
GMNMVSKGVQNVLDFLQTDFPDMDVIGISGNFCSDKKPAAVNWIEGRGKSVVCEAIINGD
VVTKVLKTSVESLVELNMLKNLTGSAMAGALGGFNAHASNIVTAVYIATGQDPAQNVESS
HCITMMEAVNGGKDLHVSVTMPSIEVGTVGGGTQLASQSACLNLLGVKGASKESPGANSI
LLATIVAGAVLAGELSLMSALAAGQLVKSHMKYNRSSKDVSKVSS
>O81108 | ACA2_ARATH
MESYLNENFDVKAKHSSEEVLEKWRNLCGVVKNPKRRFRFTANLSKRYEAAAMRRTNQEK
LRIAVLVSKAAFQFISGVSPSDYTVPEDVKAAGFEICADELGSIVESHDVKKLKFHGGVD
GLAGKLKASPTDGLSTEAAQLSQRQELFGINKFAESEMRGFWVFVWEALQDMTLMILGVC
AFVSLIVGIATEGWPKGSHDGLGIAASILLVVFVTATSDYRQSLQFRDLDKEKKKITVQV
TRNGFRQKLSIYDLLPGDIVHLAIGDQVPADGLFLSGFSVVIDESSLTGESEPVMVNAQN
PFLMSGTKVQDGSCKMMITTVGMRTQWGKLMATLTEGGDDETPLQVKLNGVATIIGKIGL
FFAVVTFAVLVQGMFMRKLSTGTHWVWSGDEALELLEYFAIAVTIVVVAVPEGLPLAVTL
SLAFAMKKMMNDKALVRHLAACETMGSATTICSDKTGTLTTNHMTVVKSCICMNVQDVAN
KGSSLQSEIPESAVKLLIQSIFNNTGGEVVVNKHGKTELLGTPTETAILELGLSLGGKFQ
EERKSYKVIKVEPFNSTKKRMGVVIELPEGGRMRAHTKGASEIVLAACDKVVNSSGEVVP
LDEESIKYLNVTINEFANEALRTLCLAYMDIEGGFSPDDAIPASGFTCVGIVGIKDPVRP
GVKESVELCRRAGITVRMVTGDNINTAKAIARECGILTDDGIAIEGPVFREKNQEELLEL
IPKIQVMARSSPMDKHTLVKQLRTTFDEVVAVTGDGTNDAPALHEADIGLAMGIAGTEVA
KESADVIILDDNFSTIVTVAKWGRSVYINIQKFVQFQLTVNVVALVVNFSSACLTGSAPL
TAVQLLWVNMIMDTLGALALATEPPNDELMKRLPVGRRGNFITNAMWRNILGQAVYQFIV
IWILQAKGKAMFGLDGPDSTLMLNTLIFNCFVFCQVFNEISSREMEEIDVFKGILDNYVF
VVVIGATVFFQIIIIEFLGTFASTTPLTITQWIFSIFIGFLGMPIAAGLKTIPV
>P0C5E5 | PRO7_ORYSI
MKIIFVFALLAIAACSASAQFDVLGQSYRQYQLQSPVLLQQQVLSPYNEFVRQQYGIAAS
PFLQSAAFQLRNNQVWQQLALVAQQSHYQDINIVQAIAQQLQLQQFGDLYFDRNLAQAQA
LLAFNVPSRYGIYPRYYGAPSTITTLGGVL
>P14891 | HMDH1_ARATH
MDLRRRPPKPPVTNNNNSNGSFRSYQPRTSDDDHRRRATTIAPPPKASDALPLPLYLTNA
VFFTLFFSVAYYLLHRWRDKIRYNTPLHVVTITELGAIIALIASFIYLLGFFGIDFVQSF
ISRASGDAWDLADTIDDDDHRLVTCSPPTPIVSVAKLPNPEPIVTESLPEEDEEIVKSVI
DGVIPSYSLESRLGDCKRAASIRREALQRVTGRSIEGLPLDGFDYESILGQCCEMPVGYI
QIPVGIAGPLLLDGYEYSVPMATTEGCLVASTNRGCKAMFISGGATSTVLKDGMTRAPVV
RFASARRASELKFFLENPENFDTLAVVFNRSSRFARLQSVKCTIAGKNAYVRFCCSTGDA
MGMNMVSKGVQNVLEYLTDDFPDMDVIGISGNFCSDKKPAAVNWIEGRGKSVVCEAVIRG
EIVNKVLKTSVAALVELNMLKNLAGSAVAGSLGGFNAHASNIVSAVFIATGQDPAQNVES
SQCITMMEAINDGKDIHISVTMPSIEVGTVGGGTQLASQSACLNLLGVKGASTESPGMNA
RRLATIVAGAVLAGELSLMSAIAAGQLVRSHMKYNRSSRDISGATTTTTTTT

>P20698 | PRO28_ORYSJ
MKIIFFFALLAIAACSASAQFDAVTQVYRQYQLQPHLMLQQQMLSPCGEFVRQQCSTVAT
PFFQSPVFQLRNCQVMQQQCCQQLRMIAQQSHCQAISSVQAIVQQLRLQQFASVYFDQSQ
AQAQAMLALNMPSICGIYPSYNTAPCSIPTVGGIWY
>P24067 | BIP2_MAIZE
MDRARGSAFLLGVLLAGSLFAFSVAKEETKKLGTVIGIDLGTTYSCVGVYKNGHVEIIAN
DQGNRITPSWVAFTDSERLIGEAAKNQAAVNPERTIFDVKRLIGRKFADKEVQRDMKLVP
YKIINKDGKPYIQVKIKDGENKVFSPEEISAMILGKMKDTAEAYLGKKINDAVVTVPAYF
NDAQRQATKDAGVIAGLNVARIINEPTAAAIAYGLDKKGGEKNILVFDLGGGTFDVSILT
IDNGVFEVLATNGDTHLGGEDFDQRIMEYFIKLIKKKYSKDISKDNRALGKLRREAERAK
RALSNQHQVRVEIESLFDGTDFSEPLTRARFEELNNDLFRKTMGPVKKAMEDAGLEKSQI
HEIVLVGGSTRIPKVQQLLRDYFDGKEPNKGVNPDEAVAFGAAVQGSILSGEGGDETKDI
LLLDVAPLTLGIETVGGVMTKLIPRNTVIPTKKSQVFTTYQDQQTTVSIQVFEGERSMTK
DCRLLGKFDLNGIAPAPRGTPQIEVTFEVDANGILNVKAEDKGTGKSEKITITNEKGRLS
QEEIDRMVREAEEFAEEDKKVKERIDARNQLETYVYNMKNTVGDKDKLADKLEAEEKEKV
EEALKEALEWLDDNQSAEKEDYEEKLKEVEAVCNPIVSAVYQRSGGAPGGDADGGVDDDH
DEL
>P25803 | CYSEP_PHAVU
MATKKLLWVVLSFSLVLGVANSFDFHDKDLASEESLWDLYERWRSHHTVSRSLGEKHKRF
NVFKANLMHVHNTNKMDKPYKLKLNKFADMTNHEFRSTYAGSKVNHPRMFRGTPHENGAF
MYEKVVSVPPSVDWRKKGAVTDVKDQGQCGSCWAFSTVVAVEGINQIKTNKLVALSEQEL
VDCDKEENQGCNGGLMESAFEFIKQKGGITTESNYPYKAQEGTCDASKVNDLAVSIDGHE
NVPANDEDALLKAVANQPVSVAIDAGGSDFQFYSEGVFTGDCSTDLNHGVAIVGYGTTVD
GTNYWIVRNSWGPEWGEHGYIRMQRNISKKEGLCGIAMLPSYPIKNSSDNPTGSFSSPKD
EL
>P29057 | HMDH1_HEVBR
MDTTGRLHHRKHATPVEDRSPTTPKASDALPLPLYLTNAVFFTLFFSVAYYLLHRWRDKI
RNSTPLHIVTLSEIVAIVSLIASFIYLLGFFGIDFVQSFIARASHDVWDLEDTDPNYLID
EDHRLVTCPPANISTKTTIIAAPTKLPTSEPLIAPLVSEEDEMIVNSVVDGKIPSYSLES
KLGDCKRAAAIRREALQRMTRRSLEGLPVEGFDYESILGQCCEMPVGYVQIPVGIAGPLL
LNGREYSVPMATTEGCLVASTNRGCKAIYLSGGATSVLLKDGMTRAPVVRFASATRAAEL
KFFLEDPDNFDTLAVVFNKSSRFARLQGIKCSIAGKNLYIRFSCSTGDAMGMNMVSKGVQ
NVLEFLQSDFSDMDVIGISGNFCSDKKPAAVNWIEGRGKSVVCEAIIKEEVVKKVLKTNV
ASLVELNMLKNLAGSAVAGALGGFNAHAGNIVSAIFIATGQDPAQNVESSHCITMMEAVN
DGKDLHISVTMPSIEVGTVGGGTQLASQSACLNLLGVKGANKESPGSNSRLLAAIVAGSV
LAGELSLMSAIAAGQLVKSHMKYNRSSKDMSKAAS
>P32291 | FAD3E_PHAAU
MIQAQTLQHFGNGAREGDQSYFDPGAPPPFKIADIRAAIPKHCWEKSTLRSLSYVLRDVL
VVTALAASAISFNSWFFWPLYWPAQGTMFWALFVLGHDCGHGSFSNSSKLNSFVGHILHS
LILVPYNGWRISHRTHHQNHGHVEKDESWVPLTEKVYKNLDDMTRMLRYSFPFPIFAYPF
YLWNRSPGKEGSHFNPYSNLFSPGERKGVVTSTLCWGIVLSVLLYLSLTIGPIFMLKLYG
VPYLIFVMWLDFVTYLHHHGYTHKLPWYRGQEWSYLRGGLTTVDRDYGWINNVHHDIGTH
VIHHLFPQIPHYHLVEATKSAKSVLGKYYREPQKSGPLPFHLLKYLLQSISQDHFVSDTG
DIVYYQTDPKLHQDSWTKSK

>P33490 | ABP1_TOBAC
MARHVLVVVAVLLFATAEASQCSINGLPLVRNISELPQENYGRSGLSHTTIAGSVLHGMK
EIEVWLQTFAPGSRTPIHRHSCEEIFVVLKGQGILYLTPSSHSKYPGNPQEFHIFPNSTF
HIPVNDVHQVWNTGEHEDLQVLVVISRPPVKVFMYDDWSMPHTAAKLKFPYYWDEECYQT
TSWKDEL
>P33522 | CRU4_BRANA
MGPTSLLSFFFTFLTLFHGFTAQQWPNECQLDQLNALEPSQIIKSEGGRIEVWDHHAPQL
RCSGFAFERFVIEPQGLYLPTFLNAGKLTFVVHGHALMGKVTPGCAETFNDSPVFGQGQG
QEQGQGQGQGQGQGFRDMHQKVEHLRSGDTIATPPGVAQWFYNNGNEPLILVAAADIANN
LNQLDRNLRPFLLAGNNPQGQQWLQGRQQQKQNNIFNGFAPQILAQAFKISVETAQKLQN
QQVNRGNIVKVQGQFGVIRPPLRQGQGGQQPQEEGNGLEETLCTMRCTENLDDPSSADVY
KPSLGYISTLNSYNLPILRFLRLSALRGSIHNNAMVLPQWNVNANAALYVTKGKAHIQNV
NDNGQRVFDQEISKGQLLVVPQGFAVVKRATSQQFQWIEFKSNDNAQINTLAGRTSVMRG
LPLEVISNGYQISPQEARSVKFSTLETTLTQSSGPMGYGMPRVEA
>P37116 | NCPR_PHAAU
MASNSDLVRAVESFLGVSLGDSVSDSLLLIATTSAAVVVGLLVFLWKKSSDRSKEVKPVV
VPRDLMMEEEEEVDVAAGKTKVTIFFGTQTGTAEGFAKALAEEIKARYEKAAVKVVDLDD
YAADDDLYEEKLKKESLVFFMLATYGDGEPIDNAARFYKWFTEGKDERGIWLQKLTYGVF
GLGNRQYEHFNKIGKVVDEELAEQGAKRLVAVGLGDDDQSIEDDFSAWKESLWSELDQLL
RDEDDANTVSTPYTAAILEYRVVIHDPTAASTYDNHSTVANGNTEFDIHHPCRVNVAVQK
ELHKPESDRSCIHLEFDISGTSITYDTGDHVGVYAENCNETVEETGKLLGQNLDLFFSLH
TDKDDGTSLGGSLLPPFPGPCSLRTALARYADLLNPPRKAALLALATHASEPSDERLKFL
SSPQGKDEYSKWVVGSQRSLVEVMAEFPSAKPPLGVFFAAIAPRLQPRYYSISSSPRFAP
QRVHVTCALVYGPTPTGRIHKGVCSTWMKNAIPSEKSQDCSSAPIFIRPSNFKLPVDHSI
PIIMVGPGTGLAPFRGFLQERYALKEDGVQLGPALLFFGCRNRQMDFIYEDELKSFVEQG
SLSELIVAFSREGAEKEYVQHKMMDKAAHLWSLISQGGYLYVCGDAKGMARDVHRTLHSI
VQEQENVDSTKAEAIVKKLQMDGRYLRDVW
>P43256 | HMDH2_ARATH
MEDLRRRFPTKKNGEEISNVAVDPPLRKASDALPLPLYLTNTFFLSLFFATVYFLLSRWR
EKIRNSTPLHVVDLSEICALIGFVASFIYLLGFCGIDLIFRSSSDDDVWVNDGMIPCNQS
LDCREVLPIKPNSVDPPRESELDSVEDEEIVKLVIDGTIPSYSLETKLGDCKRAAAIRRE
AVQRITGKSLTGLPLEGFDYNSILGQCCEMPVGYVQIPVGIAGPLLLDGVEYSVPMATTE
GCLVASTNRGFKAIHLSGGAFSVLVKDAMTRAPVVRFPSARRAALVMFYLQDPSNFERLS
LIFNKSSRFARLQSITCTIAGRNLYPRFACSTGDAMGMNMVSKGVQNVLDFVKSEFPDMD
VIGISGNYCSDKKASAVNWIEGRGKHVVCEAFIKAEIVEKVLKTSVEALVELNTLKNLVG
SAMAGSLGGFNAHSSNIVSAVFIATGQDPAQNVESSHCMTMILPDGDDLHISVSMPCIEV
GTVGGGTQLASQAACLNLLGVKGSNNEKPGSNAQQLARIVAGSVLAGELSLMSAIAAGQL
VKSHMKYNRSSRDIGPSSQVNR
>P46313 | FAD6E_ARATH
MGAGGRMPVPTSSKKSETDTTKRVPCEKPPFSVGDLKKAIPPHCFKRSIPRSFSYLISDI
IIASCFYYVATNYFSLLPQPLSYLAWPLYWACQGCVLTGIWVIAHECGHHAFSDYQWLDD
TVGLIFHSFLLVPYFSWKYSHRRHHSNTGSLERDEVFVPKQKSAIKWYGKYLNNPLGRIM
MLTVQFVLGWPLYLAFNVSGRPYDGFACHFFPNAPIYNDRERLQIYLSDAGILAVCFGLY
RYAAAQGMASMICLYGVPLLIVNAFLVLITYLQHTHPSLPHYDSSEWDWLRGALATVDRD

YGILNKVFHNITDTHVAHHLFSTMPHYNAMEATKAIKPILGDYYQFDGTPWYVAMYREAK
ECIYVEPDREGDKKGVYWYNNKL
>P47195 | C80A1_BERST
MDYIVGFVSISLVALLYFLLFKPKHTNLPPSPPAWPIVGHLPDLISKNSPPFLDYMSNIA
QKYGPLIHLKFGLHSSIFASTKEAAMEVLQTNDKVLSGRQPLPCFRIKPHIDYSILWSDS
NSYWKKGRKILHTEIFSQKMLQAQEKNRERVAGNLVNFIMTKVGDVVELRSWLFGCALNV
LGHVVFSKDVFEYSDQSDEVGMDKLIHGMLMTGGDFDVASYFPVLARFDLHGLKRKMDEQ
FKLLIKIWEGEVLARRANRNPEPKDMLDVLIANDFNEHQINAMFMETFGPGSDTNSNIIE
WALAQLIKNPDKLAKLREELDRVVGRSSTVKESHFSELPYLQACVKETMRLYPPISIMIP
HRCMETCQVMGYTIPKGMDVHVNAHAIGRDPKDWKDPLKFQPERFLDSDIEYNGKQFQFI
PFGSGRRICPGRPLAVRIIPLVLASLVHAFGWELPDGVPNEKLDMEELFTLSLCMAKPLR
VIPKVRI
>P48020 | HMDH1_SOLTU
MDVRRRPVKPLYTSKDASAGEPLKQQEVSSPKASDALPLPLYLTNGLFFTMFFSVMYFLL
VRWREKIRNSIPLHVVTLSELLAMVSLIASVIYLLGFFGIGFVQSFVSRSNSDSWDIEDE
NAEQLIIEEDSRRGPCAAATTLGCVVPPPPVRKIAPMVPQQPAKVALSQTEKPSPIIMPA
LSEDDEEIIQSVVQGKTPSYSLESKLGDCMRAASIRKEALQRITGKSLEGLPLEGFDYSS
ILGQCCEMPVGYVQIPVGIAGPLLLDGREYSVPMATTEGCLVASTNRGCKAIFVSGGADS
VLLRDGMTRAPVVRFTTAKRAAELKFFVEDPLNFETLSLMFNKSSRFARLQGIQCAIAGK
NLYITFSCSTGDAMGMNMVSKGVQNVLDYLQSEYPDMDVIGISGNFCSDKKPAAVNWIEG
RGKSVVCEAIIKEEVVKKVLKTEVAALVELNMLKNLTGSAMAGALGGFNAHASNIVSAVY
LATGQDPAQNVESSHCITMMEAVNDGKDLHVSVTMPSIEVGTVGGGTQLASQSACLNLLG
VKGANRDAPGSNARLLATIVAGSVLAGELSLMSAISAGQLVKSHMKYNRSIKDISK
>P48021 | HMDH_CAMAC
MDVRRRSINSIHQIPSVGGTAPPMLKPKQPTKVDAVDLPDSPKASDALPLPLYITNGVFF
TLFFTVVYYLLVRWREKIRNSTPLHVVTLSEIAAIFTFVASFIYLLGFFGIGLVQPFTSR
SSHDDVWGVDDDEDVDEIVLKEDTRTVPCAAAPVDCPLPPIKPKVVDPVPISPPSSEEDE
EIIKSVVEGTTPSYALESKLGDSHRAAAIRREALQRMTKKSLAGLPLDGFDYDSILGQCC
EMPVGYVQIPVGIAGPLLLDGREYSVPMATTEGCLVASTNRGCKAIFACGGATSVLLRDA
MTRAPVVRFGSAKRAADLKFFLENPLNFETLAAVFNSSSRFGKLQNIKCAIAGKNLYMRY
SCSTGDAMGMNMISKGVQNVLDFLQDDFPDMDVIGISGNYCSDKKPAAVNWIEGRGKSVV
CEAVIKEEVVKKVLKTNVASLVELNMLKNLTGSAMAGALGGFNAHASNIVSAVYLATGQD
PAQNVESSHCITMMEAINDGKDLHVSVTMPSIEVGTVGGGTQLASQSACLNLLGVKGASK
EAPGSNARLLATIVAGSVLAGELSLMSAIAAGQLVNSHMKYNRSNKDVTKASS
>P48022 | HMDH2_SOLLC
MDVRRRSEEPVYPSKVFAADEKPLKPHKKQQQQEDKNTLLIDASDALPLPLYLTTNGLF
FTMFFSVMYFLLSRWREKIRNSTPLHVVTLSELGAIVSLIASVIYLLGFFGIGFVQTFVS
RGNNDSWDENDEEFLLKEDSRCGPATTLGCAVPAPPARQIAPMAPPQPSMSMVEKPAPLI
TSASSGEDEEIIKSVVQGKIPSYSLESKLGDCKRAASIRKEVMQRITGKSLEGLPLEGFN
YESILGQCCEMPIGYVQIPVGIAGPLLLNGKEFSVPMATTEGCLVASTNRGCKAIYASGG
ATCILLRDGMTRAPCVRFGTAKRAAELKFFVEDPIKFESLANVFNQSSRFARLQRIQCAI
AGKNLYMRLCCSTGDAMGMNMVSKGVQNVLDYLQNEYPDMDVIGISGNFCSDKKPAAVNW
IEGRGKSVVCEAIITEEVVKKVLKTEVAALVELNMLKNLTGSAMAGALGGFNAHASNIVS
AVFIATGQDPAQNIESSHCITMMEAVNDGKDLHISVTMPSIEVGTVGGGTQLASQSACLN

LLGVKGANREAPGSNARLLATVVAGSVLAGELSLMSAISSGQLVNSHMKYNRSTKDVTKA
SS
>P48623 | FAD3E_ARATH
MVVAMDQRTNVNGDPGAGDRKKEERFDPSAQPPFKIGDIRAAIPKHCWVKSPLRSMSYVV
RDIIAVAALAIAAVYVDSWFLWPLYWAAQGTLFWAIFVLGHDCGHGSFSDIPLLNSVVGH
ILHSFILVPYHGWRISHRTHHQNHGHVENDESWVPLPERVYKKLPHSTRMLRYTVPLPML
AYPLYLCYRSPGKEGSHFNPYSSLFAPSERKLIATSTTCWSIMFVSLIALSFVFGPLAVL
KVYGVPYIIFVMWLDAVTYLHHHGHDEKLPWYRGKEWSYLRGGLTTIDRDYGIFNNIHHD
IGTHVIHHLFPQIPHYHLVDATKAAKHVLGRYYREPKTSGAIPIHLVESLVASIKKDHYV
SDTGDIVFYETDPDLYVYASDKSKIN
>P48624 | FAD3E_BRANA
MVVAMDQRSNVNGDSGARKEEGFDPSAQPPFKIGDIRAAIPKHCWVKSPLRSMSYVTRDI
FAVAALAMAAVYFDSWFLWPLYWVAQGTLFWAIFVLGHDCGHGSFSDIPLLNSVVGHILH
SFILVPYHGWRISHRTHHQNHGHVENDESWVPLPEKLYKNLPHSTRMLRYTVPLPMLAYP
IYLWYRSPGKEGSHFNPYSSLFAPSERKLIATSTTCWSIMLATLVYLSFLVDPVTVLKVY
GVPYIIFVMWLDAVTYLHHHGHDEKLPWYRGKEWSYLRGGLTTIDRDYGIFNNIHHDIGT
HVIHHLFPQIPHYHLVDATRAAKHVLGRYYREPKTSGAIPIHLVESLVASIKKDHYVSDT
GDIVFYETDPDLYVYASDKSKIN
>P48625 | FAD3E_SOYBN
MVKDTKPLAYAANNGYQQKGSSFDFDPSAPPPFKIAEIRASIPKHCWVKNPWRSLSYVLR
DVLVIAALVAAAIHFDNWLLWLIYCPIQGTMFWALFVLGHDCGHGSFSDSPLLNSLVGHI
LHSSILVPYHGWRISHRTHHQNHGHIEKDESWVPLTEKIYKNLDSMTRLIRFTVPFPLFV
YPIYLFSRSPGKEGSHFNPYSNLFPPSERKGIAISTLCWATMFSLLIYLSFITSPLLVLK
LYGIPYWIFVMWLDFVTYLHHHGHHQKLPWYRGKEWSYLRGGLTTVDRDYGWIYNIHHDI
GTHVIHHLFPQIPHYHLVEATQAAKPVLGDYYREPERSAPLPFHLIKYLIQSMRQDHFVS
DTGDVVYYQTDSLLLHSQRD
>P48626 | FAD3E_TOBAC
MGSLGISEIYDKNSFNEMEFEFDPSAPPPFRLAEIRNVIPKHCWVKDPLRSLSYVVRDVI
FVATLIGIAIHLDSWLFYPLYWAIQGTMFWAIFVLGHDCGHGSFSDSQLLNNVVGHILHS
AILVPYHGWRISHKTHHQNHGNVETDESWVPMPEKLYNKVGYSTKFLRYKIPFPLLAYPM
YLMKRSPGKSGSHFNPYSDLFQPHERKYVVTSTLCWTVMAALLLYLCTAFGSLQMFKIYG
APYLIFVMWLDFVTYLHHHGYEKKLPWYRGKEWSYLRGGLTTVDRDYGLFNNIHHDIGTH
VIHHLFPQIPHYHLREATKAAKPVLGKYYREPKKSGPIPFHLVKDLTRSMKQDHYVSDSG
EIVFYQTDPHIFRSAPKDE
>P48631 | FD6E2_SOYBN
MGAGGRTDVPPANRKSEVDPLKRVPFEKPQFSLSQIKKAIPPHCFQRSVLRSFSYVVYDL
TIAFCLYYVATHYFHLLPGPLSFRGMAIYWAVQGCILTGVWVIAHECGHHAFSDYQLLDD
IVGLILHSALLVPYFSWKYSHRRHHSNTGSLERDEVFVPKQKSCIKWYSKYLNNPPGRVL
TLAVTLTLGWPLYLALNVSGRPYDRFACHYDPYGPIYSDRERLQIYISDAGVLAVVYGLF
RLAMAKGLAWVVCVYGVPLLVVNGFLVLITFLQHTHPALPHYTSSEWDWLRGALATVDRD
YGILNKVFHNITDTHVAHHLFSTMPHYHAMEATKAIKPILGEYYRFDETPFVKAMWREAR
ECIYVEPDQSTESKGVFWYNNKL
>P49118 | BIP_SOLLC
MAACSRRGNSLVVLAIVLLGCLSALSNAKEEATKLGTVIGIDLGTTYSCVGVYKNGHVEI

IANDQGNRITPSWVAFTDNERLIGEAAKNLAAVNPERTIFDVKRLIGRKFEDKEVQRDMK
LVPYKIVSKDGKPYIQVKIKDGEVKVFSPEEISAMILTKMKETAEAFLGKTIKDAVVTVP
AYFNDAQRQATKDAGVIAGLNVARIINEPTAAAIAYGLDKKGGEKNILVFDLGGGTFDVS
ILTIDNGVFEVLATNGDTHLGGEDFDQRIMEYFIKLIKKKHGKDISKDNRALGKLRREAE
RAKRSLSSQHQVRVEIESLFDGTDFSEPLTRARFEELNNDLFRKTMGPVKKAMDDAGLQK
NQIDEIVLVGGSTRIPKVQQLLKDYFDGKEPSKGVNPDEAVAYGAAVQGGILSGEGGDET
KDILLLDVAPLTLGIETVGGVMTKFIPRNTVIPTKKSQVFTTYQDQQTTVSIQVFEGERS
LTKDCRNLGKFDLTGIPPAPRGTPQIEVTFEVDANGILNVKAEDKGTGKAEKITITNDKG
RLSQEEIERMVREAEEFAEEDKKVKEKIDARNALETYVYNMKNQINDKDKLADKLESDEK
EKIETATKEALEWLDDNQSAEKEDYDEKLKEVEAVCNPIITAVYQRSGGAPGGGASEEED
DSHDEL
>Q00583 | HMDH3_HEVBR
MDEVRRRPPKHIVRKDHDGEVLNSFSHGHHLPPLKPSDYSLPLSLYLANALVFSLFFSVA
YFLLHRWREKIRKSTPLHIVTFPEIAALICLVASVIYLLGFFGIGFVHSFSRASTDSWDV
EEYDDDNIIIKEDTRPTGACAAPSLDCSLSLPTKIHAPIVSTTTTSTLSDDDEQIIKSVV
SGSIPSYSLESKLGNCKRAALIRRETLQRMSGRSLEGLPLDGFDYESILGQCCEMAIGYV
QIPVGIAGPLLLDGKEYTVPMATTEGCLVASANRGCKAIYASGGATSVLLRDGMTRAPVV
RFPTAKRAADLKFFMEDPDNFDTIAVVFNKSSRFARLQSVQCAIAGKNLYMRFSCSTGDA
MGMNMVSKAVQNVIDYLQNDFPDMDVIGLTGNFCADKKAAAVNWIEGRGKSVVCEAIIKE
EVVKKVLKTNVAALVELNMIKNLTGSAVAGSLGGFNAHASNMVTAVYIATGQDPAQNVES
SHCITMMEAVNDGKDLHISVSMPSIELGTVGGGTQLASQSACLNLLGVKGASKDSPGSNS
RLLATIVAGSVLAGELSLMSAIAAGQLVNSHMKYNRSAKDVSKITF
>Q01559 | HMDH_NICSY
MDVRRRSEKPAYPTKEFAAGEKPLKPHKQQQEQDNSLLIASDALPLPLYLTNGLFFTMFF
SVMYYLLSRWREKIRNSTPLHVVTFSELVAIASLIASVIYLLGFFGIGFVQSFVSRDNND
ECWDEEDENDEQFLLEEDSRRGPATTLGCTAVPPPPALQIVPMVPPQPSKVAAMSEKPAP
LVTPAASEEDEEIIKSVVQGKMPSYSLESKLGDCKRAASIRKEALQRITGKSLEGLPLEG
FDYESILGQCCEMPIGYVQIPVGIAGPLLLDGREYSVPMATTEGCLVASTNRGCKAIYAS
GGATSVLLRDGMTRAPCVRFGTAKRAAELKFFVEDPVKFETLAAVFNQSSRFARLQRIQC
AIAGKNLYMRFVCSTGDAMGMNMVSKGVQNVLDYLQNEYPDMDVIGISGNFCSDKKPAAV
NWIEGRGKSVVCEAIITEEVVKKVLKTEVAALVELNMLKNLTGSAMAGALGGFNAHASNI
VSAVYIATGQDPAQNIESSHCITMMEAVNDGKDLHVSVTMPSIEVGTVGGGTQLASQSAC
LNLLGVKGANREVPGSNARLLATIVAGSVLAGELSLMSAISAGQLVKSHMKYNRSTKDVT
KASS
>Q03163 | HMDH_CATRO
MDSRRRSPTVTAKAAAGELPLAPHEGQNQQPSIPRSSDVLPLPLYLANGVFFTLFFSVMY
FLLTRWREKIRNATPLHVVTLSELAALASLIASVIYLVSFFGLDFVQSLIYKPNNEGWEI
EEEILMVEDSRNGTNCTTLGCAVPPPSVPKIAPVVPQQPSKMVIIEKPAPLITPQNSEED
EDIIKAVVAGKIPSYSLESKLGDCKRAAGIRREALQRITGKSLEGLPLEGFDYASILGQC
CEMPVGYVQLPVGIAGPLLLDGREYMLPMATTEGCLVASTNRGCKAILASGGANSVLLRD
GMTRAPVVRFGTAKRAAELKFYMEDTQNFETISVVFNKSSRFAKLQSVQCAIAGKNLYIR
FSCSTGDAMGMNMVSKGVQNVLEFLQTDYPDMDVLGISGNFCADKKPAAVNWIEGRGKSV
VCEAIIKEEIVKTVLKTEVAALIELNMVKNLAGSAIAGALGGFNAHASNIVSAIFIATGQ
DPAQNVESSQCITMMEAVNDGKDLHISVTMPSIEVGTVGGGTQLASQSACLNLLGVKGAS

KDSPGANSRLLATIVAGSVLAGELSLMSAISAGQLVRSHMKYNRSSKDITNIASSQLESD
S
>Q05JG2 | ABAH1_ORYSJ
MGAFLLFVCVLAPFLLVCAVRGRRRQAGSSEAAACGLPLPPGSMGWPYVGETFQLYSSKN
PNVFFNKKRNKYGPIFKTHILGCPCVMVSSPEAARFVLVTQAHLFKPTFPASKERMLGPQ
AIFFQQGDYHAHLRRIVSRAFSPESIRASVPAIEAIALRSLHSWDGQFVNTFQEMKTYAL
NVALLSIFGEEEMRYIEELKQCYLTLEKGYNSMPVNLPGTLFHKAMKARKRLGAIVAHII
SARRERQRGNDLLGSFVDGREALTDAQIADNVIGVIFAARDTTASVLTWMVKFLGDHPAV
LKAVTEEQLQIAKEKEASGEPLSWADTRRMKMTSRVIQETMRVASILSFTFREAVEDVEY
QGYLIPKGWKVLPLFRNIHHNPDHFPCPEKFDPSRFEVAPKPNTFMPFGNGTHSCPGNEL
AKLEMLVLFHHLATKYRWSTSKSESGVQFGPFALPLNGLPMSFTRKNTEQE
>Q0DJ45 | PRO7_ORYSJ
MKIIFVFALLAIAACSASAQFDVLGQSYRQYQLQSPVLLQQQVLSPYNEFVRQQYGIAAS
PFLQSAAFQLRNNQVWQQLALVAQQSHYQDINIVQAIAQQLQLQQFGDLYFDRNLAQAQA
LLAFNVPSRYGIYPRYYGAPSTITTLGGVL
>Q0DN94 | PROA_ORYSJ
MAAYTSKIFALFALIALSASATTAITTMQYFPPTLAMGTMDPCRQYMMQTLGMGSSTAMF
MSQPMALLQQQCCMQLQGMMPQCHCGTSCQMMQSMQQVICAGLGQQQMMKMAMQMPYMCN
MAPVNFQLSSCGCC
>Q0DY59 | HMDH1_ORYSJ
MDVRRGGGGGRIVGAARRALTWGALPLPMRITNGLAMVSLVLSSCDLLRLCSDRERPLGG
REFATVVYLVSLFAHPDAPATTTGDDDDGQGGSRRARPAAAEPAPMHGHGGGMMEADDEE
IVAAVASGALPSHRLESRLGDCRRAARLRREALRRVTGRGVEGLPFDGMDYQAILGQCCE
MPVGYVQLPVGVAGPLLLDGREYHVPMATTEGCLVASVNRGCRAISASGGAFSVLLRDAM
SRAPAVKLPSAMRAAELKAFAEAPANFELLAAVFNRSSRFGRLQDIRCALAGRNLYMRFS
CITGDAMGMNMVSKGVENVLGYLQNVFPDMDVISVSGNYCSDKKPTAVNWIEGRGKSVVC
EAIIKGDVVQKVLKTTVEKLVELNIIKNLAGSAVAGALGGFNAHASNIVTALFIATGQDP
AQNVESSQCITMLEEVNDGDDLHISVTMPSIEVGTIGGGTCLASQAACLNLLGVKGSNHG
SPGANAKRLATIVAGSVLAGELSLLAALASGHLVKSHMMYNRSSKDVAKAAS
>Q39287 | FAD6E_BRAJU
MGAGGRMQVSPSPKKSETDTLKRVPCETPPFTVGELKKAIPPHCFKRSIPRSFSYLIWDI
IVASCFYYVATTYFPLLPHPLSYVAWPLYWACQGVVLTGVWVIAHECGHHAFSDYQWLDD
TVGLIFHSFLLVPYFSWKYSHRRHHSNTGSLERDEVFVPKKKSDIKWYGKYLNNPLGRTV
MLTVQFTLGWPLYWAFNVSGRPYPEGFACHFHPNAPIYNDRERLQIYVSDAGILAVCYGL
YRYAAAQGVASMVCLYGVPLLIVNAFLVLITYLQHTHPSLPHYDSSEWDWLRGALATVDR
DYGILNKVFHNITDTHVAHHLFSTMPHYHAMEVTKAIKPILGDYYQFDGTPWVKAMWREA
KECIYVEPDRQGEKKGVFWYNNKL
>Q41437 | HMDH2_SOLTU
MDVRRRSEKPVYPSKVFGADEKPLKPHNNQQQEDNNTLLIDASDALPLPLYLTNGLFFTM
FFSVMYFLLSRWREKIRNSTPLHVVTLSELGAIVSLIASVIYLLGFFGIGFVQTFVSRGN
NDSWDENDEEFLLKEDSRCGPATTLGCAIPAPPARQISPMAPPQPAMSMVEKPSPLITPA
SSEEDEEIINSVVQGKFPSYSLVIQLGDVSAAASLRKEVMQRITGKSLEGLPLEGFTYES
ILGQCCEMPIGYVQIPVGIAGPLLLNGKEFSVPMATTEGCLVASTNRGCKAIYASGGATC
IVLRDGMTRAPCVRFGTAKRAAELKFFVEDPIKFETLANVFNQSSRFGRLQRIQCAIAGK

NLYMRFVCSTGDAMGMNMVSKGVQNVLDYLQNEYPDMDVIGISGNFCSDKKPAAVNWIEG
RGKSVVCEAIITEEVVKKVLKTEVAALVELNMLKNLTGSAMAGALGGFNAHASNIVSAVF
IATGQDPAQNIESSHCITMMEAVNDGKDLHISVTMPSIEVGTVGGGTQLASQSACLNLLG
VKGANREAPGSNARLLATVVAGSVLAGELSLMSAISAGQLVNSHMKYNRSTKASS
>Q41438 | HMDH3_SOLTU
MDVRRRPVKPLYPSEHISSGEPLKPHNQDSSVKASDALPLPLYLTNGLFFTMFFSVMYFL
LHRWREKIRNGIPLHVLNFSELVAMVSLIASVIYLLGFFGIGFVQSFVSKGNNDSWDVED
ESPEQFIDRTVTPPPVRRNIPMKSVPVAEKTAQIITPFSSEDDEVVIKSVVEGRIPSYSL
ESKLGDCKRAAFIRKEALQRSSGKSLEGLPLDGFDYESILGQCCEMPIGYIQIPVGIAGP
LLLNGKEFSVPMATTEGCLVASTNRGCKAIYVSGGATSVLFRDAMTRAPVVRFGSAKRAA
ELKFFVEDPMNFETLSVVFNKSSRFARLQNIQCAIAGKNLYMRFSCSTGDAMGMNMVSKG
VQNVLDYLQNEYPDMDIIGISGNYCSDKKPAAVNWIEGRGKSVVCEAIIKEDVVKKVLKT
EVATLVELNMLKNLTGSAMAGALGGFNAHASNIVSAVYLATGQDPAQNIESSHCITMMEA
VNDGKDLHISVTMPSIEVGTVGGGTQLASQSACLNLLGVKGANREAPGSNARLLATIVAG
SVLAGELSLMSAISAGQLVKSHMKYNRSCKDVTK
>Q42434 | BIP_SPIOL
MAVAWKSRASSIAFGIVLLGSLFAFVSAKDEAPKLGTVIGIDLGTTYSCVGVYKDGKVEI
IANDQGNRITPSWVAFTNDERLIGEAAKNQAAANPERTIFDVKRLIGRKFEDKEVQKDMK
LVPYKIVNRDGKPYIQVKVQEGETKVFSPEEISAMILTKMKETAETFLGKKIKDAVVTVP
AYFNDAQRQATKDAGVIAGLNVARIINEPTAAAIAYGLDKRGGEKNILVFDLGGGTFDVS
VLTIDNGVFEVLATNGDTHLGGEDFDQRLMEYFIKLIKKKHTKDISKDNRALGKLRRECE
RAKRALSSQHQVRVEIESLFDGVDFSEPLTRARFEELNNDLFRKTMGPVKKAMDDAGLEK
NQIDEIVLVGGSTRIPKVQQLLKEFFNGKEPSKGVNPDEAVAFGAAVQGSILSGEGGEET
KEILLLDVAPLTLGIETVGGVMTKLIPRNTVIPTKKSQVFTTYQDQQTTVTIQVFEGERS
LTKDCRLLGKFDLTGIAPAPRGTPQIEVTFEVDANGILNVKAEDKASGKSEKITITNDKG
RLSQEEIERMVREAEEFAEEDKKVKEKIDARNSLETYIYNMKNQISDADKLADKLESDEK
EKIEGAVKEALEWLDDNQSAEKEDYDEKLKEVEAVCNPIITAVYQRSGGPSGESGADSED
SEEGHDEL
>Q4G2J4 | DER21_MAIZE
MAQAVEEWYRQMPIITRSYLTAAVVTTVGCTLEIISPYHLYLNPKLVVQHYEIWRLVTNF
LYFRKMDLDFLFHMFFLARYCKLLEENSFRGRTADFFYMLLFGATVLTSIVLIGGMIPYI
SETFARILFLSNSLTFMMVYVWSKHNPFIHMSFLGLFTFTAAYLPWVLLGFSILVGSSTW
VDLLGMIAGHVYYFLEDVYPRMTGRRPLKTPSFIKALFADDNVVVAQPPNAGIGAGARFG
AMGLDPQAQ
>Q4G2J5 | DER12_MAIZE
MSSPAEYYKSLPPISKAYGTLCFFTTVLVRLHILNPLFLYLYYPRVFKKFEVWRIFTSFF
FLGPFSINFGIRLLMIARYGVMLEKGAFDKRTADFLWMMIFGAISLLVLSVIPQLNTYVL
GLPMVSMLVYVWSRENPNAQINIYGILQLKAFYLPWVMLLLDVIFGSPLMPGLLGIMVGH
LYYYFAVLHPLATGKNYLKTPKWVHKIVARFRIGMQANAPVRAPANGNAGTGAFRGRSYR
LNQ
>Q4G2J6 | DER11_MAIZE
MSSPAEYYKSLPPISKAYGTLCFFTTVLVQLQILHPLFLYLDYPLVFKKFEIWRLLTSFF
FLAPFSMKFGIRLLMIARYGVMLEKGAFDKRTADFLWMMIFGAISLLVLSIIPLFNSFFL
GIPMVSMLLYVWSRENPNAQINIYGLVQLRSFYLPWAMLLLDVIFGSSLMPGLLGIMVGH

LYYFFAVLHPLATGKSYLKTPKWVHKIVARFRIGMQANSPVRPPANGNSGSGVFRGRSYR
LNQ
>Q9C5Y2 | KAO2_ARATH
MTETGLILMWFPLIILGLFVLKWVLKRVNVWIYVSKLGEKKHYLPPGDLGWPVIGNMWSF
LRAFKTSDPESFIQSYITRYGRTGIYKAHMFGYPCVLVTTPETCRRVLTDDDAFHIGWPK
STMKLIGRKSFVGISFEEHKRLRRLTSAPVNGPEALSVYIQFIEETVNTDLEKWSKMGEI
EFLSHLRKLTFKVIMYIFLSSESEHVMDSLEREYTNLNYGVRAMGINLPGFAYHRALKAR
KKLVAAFQSIVTNRRNQRKQNISSNRKDMLDNLIDVKDENGRVLDDEEIIDLLLMYLNAG
HESSGHLTMWATILMQEHPMILQKAKEEQERIVKKRAPGQKLTLKETREMVYLSQVIDET
LRVITFSLTAFREAKSDVQMDGYIIPKGWKVLTWFRNVHLDPEIYPDPKKFDPSRWEGYT
PKAGTFLPFGLGSHLCPGNDLAKLEISIFLHHFLLKYRVERSNPGCPVMFLPHNRPKDNC
LARITRTMP
>Q9C7S7 | ERO1_ARATH
MGKGAIKEEESEKKRKTWRWPLATLVVVFLAVAVSSRTNSNVGFFFSDRNSCSCSLQKTG
KYKGMIEDCCCDYETVDNLNTEVLNPLLQDLVTTPFFRYYKVKLWCDCPFWPDDGMCRLR
DCSVCECPENEFPEPFKKPFVPGLPSDDLKCQEGKPQGAVDRTIDNRAFRGWVETKNPWT
HDDDTDSGEMSYVNLQLNPERYTGYTGPSARRIWDSIYSENCPKYSSGETCPEKKVLYKL
ISGLHSSISMHIAADYLLDESRNQWGQNIELMYDRILRHPDRVRNMYFTYLFVLRAVTKA
TAYLEQAEYDTGNHAEDLKTQSLIKQLLYSPKLQTACPVPFDEAKLWQGQSGPELKQQIQ
KQFRNISALMDCVGCEKCRLWGKLQVQGLGTALKILFSVGNQDIGDQTLQLQRNEVIALV
NLLNRLSESVKMVHDMSPDVERLMEDQIAKVSAKPARLRRIWDLAVSFW
>Q9FUJ3 | CKX2_ARATH
MANLRLMITLITVLMITKSSNGIKIDLPKSLNLTLSTDPSIISAASHDFGNITTVTPGGV
ICPSSTADISRLLQYAANGKSTFQVAARGQGHSLNGQASVSGGVIVNMTCITDVVVSKDK
KYADVAAGTLWVDVLKKTAEKGVSPVSWTDYLHITVGGTLSNGGIGGQVFRNGPLVSNVL
ELDVITGKGEMLTCSRQLNPELFYGVLGGLGQFGIITRARIVLDHAPKRAKWFRMLYSDF
TTFTKDQERLISMANDIGVDYLEGQIFLSNGVVDTSFFPPSDQSKVADLVKQHGIIYVLE
VAKYYDDPNLPIISKVIDTLTKTLSYLPGFISMHDVAYFDFLNRVHVEENKLRSLGLWEL
PHPWLNLYVPKSRILDFHNGVVKDILLKQKSASGLALLYPTNRNKWDNRMSAMIPEIDED
VIYIIGLLQSATPKDLPEVESVNEKIIRFCKDSGIKIKQYLMHYTSKEDWIEHFGSKWDD
FSKRKDLFDPKKLLSPGQDIF
>Q9FUY7 | C79F2_ARATH
MMMKISFNTCFQILLGFIVFIASITLLGRIFSRPSKTKDRCRQLPPGRPGWPILGNLPEL
IMTRPRSKYFHLAMKELKTDIACFNFAGTHTITINSDEIAREAFRERDADLADRPQLSIV
ESIGDNYKTMGTSSYGEHFMKMKKVITTEIMSVKTLNMLEAARTIEADNLIAYIHSMYQR
SETVDVRELSRVYGYAVTMRMLFGRRHVTKENMFSDDGRLGKAEKHHLEVIFNTLNCLPG
FSPVDYVDRWLGGWNIDGEEERAKVNVNLVRSYNNPIIDERVEIWREKGGKAAVEDWLDT
FITLKDQNGNYLVTPDEIKAQCVEFCIAAIDNPANNMEWTLGEMLKNPEILRKALKELDE
VVGKDRLVQESDIRNLNYLKACCRETFRIHPSAHYVPPHVARQDTTLGGYFIPKGSHIHV
CRPGLGRNPKIWKDPLAYEPERHLQGDGITKEVTLVETEMRFVSFSTGRRGCVGVKVGTI
MMAMMLARFLQGFNWKLHRDFGPLSLEEDDASLLMAKPLLLSVEPRLASNLYPKFRP
>Q9XEL8 | HMDH2_CAPAN
MDVRRRSEEAVYSSKVFAADEKPLKPHKQQQEEDNTLLIDASDALPLPLYFTNGLFFTMF
FSVMYFLLSRWREKIRNSTPLHVVTLSELGAIVSLIASVIYLLGFFGIGFVQTFVARGNN

DSWDEEDENDEQFILEEDSRRGPCAAATTLGCAVPTPPAKHIAPIVPQQPAVSIAEKPAP
LVTPAASEEDEEIIKSVVQGKIPSYSLESKLGDCKRAASIRKEVLQRITGKSLEGLPLDG
FNYESILGQCCEMTIGYVQIPVGIAGPLLLNGREYSVPMATTEGCLVASTNRGCKAIYAS
GGATSILLRDGMTRAPCVRFGTAKRAAELKFFVEDPINFETLANVFNQSSRFARLQRIQC
AIAGKNLHMRFVCSTGDAMGMNMVSKGVQNVLDYLQNEYADMDVIGISANFCSDKKPAAV
NWIEGRGKSVVCEAIITEEVVKKVLKTEVAALVELNMLKNLTGSALAGALGGFNAHASNI
VSAVYIATGQDPAQNIESSHCITMMEAVNDGKDLHISVTMPSIEVGTVGGGTQLASQSAC
LNLLGVKGANREAPGSNARLLATIVAGSVLAGELSLMSAISAGQLVNSHMKYNRSTKDVT
KASS
>Q9XHL5 | HMDH3_ORYSJ
MEVRRRAPLPPPPGRVQAGDALPLPIRHTNLIFSALFAASLAYLMRRWREKIRSSTPLHV
VGLAEMLAIFGLVASLIYLLSFFGIAFVQSIVSSSDDEEEDFLVGPARGSSAAAAVAPPP
PPSSPAQCSLLGSPHDDAARERMPEEDEEIVSSVVAGKVPSYVLETKLGDCRRAAGIRRE
AVRRITGRQIEGLPLDGFDYASILGQCCELPVGYVQLPVGIAGPLLLDGQRFYVPMATTE
GCLVASTNRGCKAIAESGGAVSVVLRDGMTRAPVARLPTARRAAELKAFLEDSVNFNTLS
MVFNRSSRFARLQGVQCAMAGRNLYMRFSCCTGDAMGMNMVSKGVQNVLDYLQDDFPDMD
VISISGNFCSDKKPAAVNWIEGRGKSVVCEAVIKEDVVKKVLKTNVQSLVELNVIKNLAG
SAVAGALGGFNAHASNIVTAIFIATGQDPAQNVESSHCITMLEAVNDGRDLHISVTMPSI
EVGTVGGGTQLASQAACLDLLGVKGANRESPGSNARLLATVVAGGVLAGELSLLSALAAG
QLVKSHMKYNRSSKDMSKVIS
>Q9ZTN2 | ERD2_PETHY
MNIFRLAGDMTHLASVLVLLLKIHTIKSCAGVSLKTQELYALVFVTRYLDIFTDFISLYN
TTMKLVFLGSSLSIVWYMRHHKIVRRSYDKDQDTFRHLFLVLPCLLLALVINEKFTFKEV
MWTFSIYLEAVAILPQLVLLQRTRNIDNLTGQYIFLLGAYRSFYILNWVYRYFTEPHFVH
WITWIAGLIQTLLYADFFYYYFQSWKNNTKLELPA

(2) 263 mitochondrion proteins

>A2Y4S9 | CYC_ORYSI
MASFSEAPPGNPKAGEKIFKTKCAQCHTVDKGAGHKQGPNLNGLFGRQSGTTPGYSYSTA
NKNMAVIWEENTLYDYLLNPKKYIPGTKMVFPGLKKPQERADLISYLKEATS
>O05000 | NU2M_ARATH
MKAEFVRILPHMFNLFLAVFPEIFIINATFILLIHGVVFSTSKKYDYPPLASNVGWLGLL
SVLITLLLLAAGAPLLTIAHLFWNNLFRRDNFTYFCQIFLLLSTAGTISMCFDFFDQERF
DAFEFIVLILLSTCGMLFMISAYDLIAMYLAIELQSLCFYVIAASKRKSEFSTEAGLKYL
ILGAFSSGILLFGCSMIYGSTGATHFDQLAKILTGYEITGARSSGIFMGILFIAVGFLFK
ITAVPFHMWAPDIYEGSPTPVTAFLSIAPKISIFANILRVFIYGSYGATLQQIFFFCSIA
SMILGALAAMAQTKVKRLLAYSSIGHVGYICIGFSCGTIEGIQSLLIGIFIYALMTMDAF
AIVLALRQTRVKYIADLGALAKTNPILAITFSITMFSYAGIPPLAGFCSKFYLFFAALGC
GAYFLALVGVVTSVIGCFYYIRLVKRMFFDTPRTWILYEPMDRNKSLLLAMTSFFITLFL
LYPSPLFSVTHQMALSLYL
>O22642 | CYC_FRIAG
MASFSEAPPGDFKSGEKIFKTKCAQCHTVDKGAGHKQGPNLNGLFGRQSGTTAGYSYSAA
NKNKAVNWDENTLYDYLLNPKKYIPGTKMVFPGLKKPQDRADLIAYLKEATSS
>O23936 | GCST_FLATR
MRGGLWQVGQSITRRLGQSDKKTIVRRWYASEADLKKTVLYDFHVANGGKMVPFAGWSMP

IQYKDSIMESTINCRENGSLFDVSHMCGLSLKGKDCVAFLEKLVVADVAGLAPGTGSLTV
FTNEKGGAIDDSVITKVTDDHIYLVVNAGCRDKDLAHIEQHMKAFKAKGGDVSWHIHDER
SLLALQGPLAGSTLQHLTKDDLSKMYFGDFRIIDISGSKCFLTRTGYTGEDGFEISVPSE
NAVDLAKAILEKSEGKVRLTGLGARDSLRLEAGLCLYGNDMEQHITPVEAGLTWAIGKRR
RAEGGFLGAEVILKQIADGPAIRRVGLFSTGPPARSHSEIQNEQGENIGEVTSGGFSPCL
KKNIGMGYVKSGLHKPGTKLKIVIRGKTYEGSVTKMPFVPTKYYKPA
>O49850 | GCSP_FLAAN
MERARRLAMLGRLVSQTKHNPSISSPALCSPSRYVSSLSPYVCGGTNVRSDRNLNGFGSQ
VRTISVEALKPSDTFPRRHNSATPEEQTKMAEFVGFSNLDSLIDATVPKSIRLDSMKYSK
FDEGLTESQMIAHMQDLASKNKIFKSFIGMGYYNTSVPTVILRNIMENPGWYTQYTPYQA
EIAQGRLESLLNFQTMITDLTGLPMSNASLLDEGTAAAEAMAMCNNIQKGKKKTFIIASN
CHPQTIDICKTRADGFDLKVVTSDLKDFDYSSGDVCGVLVQYPGTEGELLDYSEFIKNAH
ANGVKVVMASDLLALTILKPPGELGADIVVGSAQRFGVPMGYGGPHAAFLATSQEYKRMM
PGRIIGVSVDSSGKPALRMAMQTREQHIRRDKATSNICTAQALLANMAAMYGVYHGPEGL
KTIAKRVHGLAGTFAAGLKKLGTVQVQDLPFFDTVKVTCADSKAIAEEACKHKMNLRIVD
KNTITVAFDETTTIEDVDTLFKVFALGKPVPFTAASIAPEVQDAIPSGLVRETPYLTHPI
FNMYHTEHELLRYISKLQSKDLSLCHSMIPLGSCTMKLNATTEMMPVTWPAFADIHPFAP
TEQAQGYQEMFKNLGDLLCTITGFDSFSLQPNAGAAGEYAGLMVIRAYHMARGDHHRNVC
IIPVSAHGTNPASAAMCGMKIITVGTDSKGNINIEELRKAAEANKENLSALMVTYPSTHG
VYEEGIDEICKIIHDNGGQVYMDGANMNAQVGLTSPGWIGADVCHLNLHKTFCIPHGGGG
PGMGPIGVKKHLAPYLPSHPVVPTGGIPAPEESQPLGTIAAAPWGSALILPISYTYIAMM
GSQGITNASKIAILNANYMAKRLENHYPILFRGVNGTVAHEFIVDLRPLKTTAGIEPEDV
AKRLIDYGFHGPTMSWPVPGTLMIEPTESESKAELDRFCDALISIRQEIAEIEKGNVDFN
NNVIKGAPHPPQLLMADKWTKPYSREYAAYPAPWLRAAKFWPTTCRVDNVYGDRNLICTL
QPPQEYEEKAEATA
>O64966 | HMDH1_GOSHI
METHRRSSTNSIRSHKPARPIALEDDSTKASDALPLPLYLTNAVFFTLFFSAVYFLLCRW
REKIRSSTPLHVVTFSEIVAILASVASFIYLLGFFGIDFVQSLVLRPSADVWATEDDEVE
SEVLLRNEDARHVPCGQALDRSIRSLQPPEPIVTAEKVFDEMPVTVMTEEDEEIIRSVVC
GMTPSYSLESKLDDCKRAAAIRREALQRITGKSLSGLPLDGFDYESILGQCCEMPVGYEQ
IPVGIAGPLLLNGREYSVPMATTEGCLVASTNRGCKAIHLSGGATSVLLRDGMTRAPVVR
FGTAKRAADLKLYLEDPENFETLACVFNRSSRFARLQSIKCAIAGKNLYLRFSCFTGDAM
GMNMVSKGVQNVLDFLQTDFPDMDVIGISGNFCSDKKPAAVNWIEGRGKSVVCEAIINGD
VVTKVLKTSVESLVELNMLKNLTGSAMAGALGGFNAHASNIVTAVYIATGQDPAQNVESS
HCITMMEAVNGGKDLHVSVTMPSIEVGTVGGGTQLASQSACLNLLGVKGASKESPGANSI
LLATIVAGAVLAGELSLMSALAAGQLVKSHMKYNRSSKDVSKVSS
>O81235 | SODM_ARATH
MAIRCVASRKTLAGLKETSSRLLRIRGIQTFTLPDLPYDYGALEPAISGEIMQIHHQKHH
QAYVTNYNNALEQLDQAVNKGDASTVVKLQSAIKFNGGGHVNHSIFWKNLAPSSEGGGEP
PKGSLGSAIDAHFGSLEGLVKKMSAEGAAVQGSGWVWLGLDKELKKLVVDTTANQDPLVT
KGGSLVPLVGIDVWEHAYYLQYKNVRPEYLKNVWKVINWKYASEVYEKENN
>O81796 | IDH3_ARATH
MARRSVSIFNRLLANPPSPFTSLSRSITYMPRPGDGAPRTVTLIPGDGIGPLVTGAVEQV
MEAMHAPVHFERYEVLGNMRKVPEEVIESVKRNKVCLKGGLATPVGGGVSSLNMQLRKEL

DIFASLVNCINVPGLVTRHENVDIVVIRENTEGEYSGLEHEVVPGVVESLKVITKFCSER
IARYAFEYAYLNNRKKVTAVHKANIMKLADGLFLESCREVAKHYSGITYNEIIVDNCCMQ
LVAKPEQFDVMVTPNLYGNLIANTAAGIAGGTGVMPGGNVGAEHAIFEQGASAGNVGNDK
MVEQKKANPVALLLSSAMMLRHLRFPTFADRLETAVKQVIKEGKYRTKDLGGDCTTQEVV
DAVIAALE
>P00051 | CYC_CUCMA
ASFDEAPPGNSKAGEKIFKTKCAQCHTVDKGAGHKQGPNLNGLFGRQSGTTPGYSYSAAN
KNRAVIWEEKTLYDYLLNPKKYIPGTKMVFPGLKKPQDRADLIAYLKEATA
>P00052 | CYC_PHAAU
ASFDEAPPGNSKSGEKIFKTKCAQCHTVDKGAGHKQGPNLNGLFGRQSGTTAGYSYSTAN
KNMAVIWEEKTLYDYLLNPKKYIPGTKMVFPGLKKPQDRADLIAYLKESTA
>P00053 | CYC_CANSA
ASFBZAPPGBSKAGEKIFKTKCAECHTVGRGAGHKQGPNLNGLFGRQSGTTAGYSYSAAN
KNMAVTWZZKTLYDYLLNPKKYIPGTKMVFPGLKKPZBRADLIAYLKESTA
>P00054 | CYC_SESIN
ASFBZAPPGBVKSGEKIFKTKCAQCHTVDKGAGHKQGPNLNGLFGRQSGTTPGYSYSAAN
KNMAVIWGENTLYDYLLNPKKYIPGTKMVFPGLKKPQERADLIAYLKEATA
>P00056 | CYC_MAIZE
ASFSEAPPGNPKAGEKIFKTKCAQCHTVEKGAGHKQGPNLNGLFGRQSGTTAGYSYSAAN
KNKAVVWEENTLYDYLLNPXKYIPGTKMVFPGLXKPQERADLIAYLKEATA
>P00057 | CYC_RICCO
ASFBZAPPGBVKAGEKIFKTKCAQCHTVEKGAGHKQGPNLNGLFGRQSGTTAGYSYSAAN
KNMAVQWGENTLYDYLLNPKKYIPGTKMVFPGLKKPQDRADLIAYLKZATA
>P00058 | CYC_GOSBA
ASFQZAPPGBAKAGEKIFKTKCAQCHTVDKGAGHKQGPNLNGLFGRQSGTTAGYSYSAAN
KNMAVQWGENTLYDYLLNPKKYIPGTKMVFPGLKKPQDRADLIAYLKZSTA
>P00059 | CYC_ABUTH
ASFQZAPPGBAKAGEKIFKTKCAQCHTVEKGAGHKQGPNLNGLFGRQSGTTPGYSYSAAN
KNMAVNWGENTLYDYLLNPKKYIPGTKMVFPGLKKPQDRADLIAYLKZSTA
>P00060 | CYC_SOLLC
ASFNEAPPGNPKAGEKIFKTKCAQCHTVEKGAGHKEGPNLNGLFGRQSGTTAGYSYSAAN
KNMAVNWGENTLYDYLLNPKKYIPGTKMVFPGLKKPQERADLIAYLKEATA
>P00061 | CYC_SOLTU
ASFGEAPPGNPKAGEKIFKTKCAQCHTVDKGAGHKEGPNLNGLFGRQSGTTAGYSYSNAN
KNMAVTWGENTLYDYLLNPKKYIPGTKMVFPGLKKPQERADLIAYLKEATA
>P00062 | CYC_SAMNI
ASFAEAPPGNPKAGEKIFKTKCNQCHTVDKGAGHKQGPNLNGLFGRQSGTTAGYSYSAAN
KNMAVNWEEKTLYDYLLNPKKYIPGTKMVFPGLKKPQDRADLIAYLKQSTA
>P00063 | CYC_ACENE
ASFAEAPPGNPAAGEKIFKTKCAQCHTVDKGAGHKQGPNLNGLFGRQSGTTAGYSYSAAN
KNMAVNWGYNTLYDYLLNPKKYIPGTKMVFPGLKKPQDRADLIAYLKQSTAA
>P00064 | CYC_ALLPO
ATFSZAPPGBZKAGQKIFKLKCAQCHTVEKGAGHKQGPNLNGLFGRQSGTAAGYSYSAAN
KNMAVVWZZBTLYDYLLNPKKYIPGTKMVFPGLKKPQDRADLIAYLKESTA

>P00065 | CYC_ARUMA
ASFAEAPPGNPKAGEKIFKTKCAQCHTVEKGAGHKQGPNLNGLFGRQSGTTAGYSYSAAN
KNMAVIWEESTLYDYLLNPXKYIPGTKMVFPGLXKPQERADLIAYLKESTA
>P00066 | CYC_NIGDA
ASFBZAPAGBSASGEKIFKTKCAZCHTVBZGAGHKZGPNLHGLFGRQSGTVAGYSYSAAN
KNKAVNWEEKTLYDYLLNPKKYIPGTKMVFPGLKKPZZRABLLAYLKESTA
>P00067 | CYC_TROMA
ASFAEAPAGDNKAGDKIFKNKCAQCHTVDKGAGHKQGPNLNGLFGRQSGTTAGYSYSAAN
KNKAVLWZZATLYDYLLNPKKYIPGTKMVFPGLKKPQDRADLIAYLKESTA
>P00068 | CYC_WHEAT
ASFSEAPPGNPDAGAKIFKTKCAQCHTVDAGAGHKQGPNLHGLFGRQSGTTAGYSYSAAN
KNKAVEWEENTLYDYLLNPKKYIPGTKMVFPGLKKPQDRADLIAYLKKATSS
>P00069 | CYC_GUIAB
ASFAEAPAGDAKAGEKIFKTKCAZCHTVZKGAGHKQGPNLNGLFGRQSGTTAGYSYSAAN
KNKAVAWZZBSLYDYLLNPKKYIPGTKMVFPGLKKPZZRADLIAYLKASTA
>P00070 | CYC_HELAN
MASFAEAPAGNPTTGEKIFKTKCAQCHTVEKGAGHKQGPNLNGLFGRQSGTTAGYSYSAG
NKNKAVIWEENTLYDYLLNPKKYIPGTKMVFPGLKKPQERADLIAYLKTSTA
>P00071 | CYC_PASSA
ASFAEAPPGDKDVGGKIFKTKCAZCHTVZLGAGHKQGPNLNGLFGRQSGTTAGYSYSAAN
KNKAVLWABBTLYDYLLNPKKYIPGTKMVFPGLKKPQDRADLIAYLKHATA
>P00072 | CYC_FAGES
ATFSEAPPGNIKSGEKIFKTKCAQCHTVEKGAGHKQGPNLNGLFGRQSGTTAGYSYSAAN
KNKAVTWGEDTLYEYLLNPKKYIPGTKMVFPGLKKPQERADLIAYLKBSTZ
>P00073 | CYC_SPIOL
ATFSEAPPGNKDVGAKIFKTKCAQCHTVDLGAGHKQGPNLNGLFGRQSGTAASYSYSAAN
KNKAVIWSEDTLYEYLLNPKKYIPGTKMVFPGLKKPQDRADLIAYLKDSTQ
>P00074 | CYC_GINBI
ATFSEAPPGDPKAGEKIFKTKCAZCHTVZKGAGHKQGPNLHGLFGRQSGTTAGYSYSTGN
KNKAVNWGZZTLYEYLLNPKKYIPGTKMVFPGLKKPZZRADLISYLKQATSQE
>P00075 | CYC_ENTIN
STFABAPPGBPAKGAKIFKAKCAZCHTVBAGAGHKQGPNLNGAFGRTSGTAAGFSYSAAB
KBKTADWBZBTLYDYLLNPKKYIPGTKMVFAGLKKPZBRADLIAFLKDATA
>P00412 | COX2_MAIZE
MILRLLECRFFTIALCDAAEPWQLGFQDAATPMMQGIIDLHHDIFFFLILILVFVLWMLV
RALWHFNEQTNPIPQRIVHGTTIEIIWTIFPSVILLFIAIPSFALLYSMDGVLVDPAITI
KAIGHQWYWTYEYSDYNSSDEQSLTFDSYMIPEDDLELGQLRLLEVDNRVVVPAKTHLRM
IVTSADVLHSWAVPSLGVKCDAVPGRLNLTSILVQREGVYYGQCSEICGTNHAFMPIVVE
AVTLKDYADWVSNQLILQTN
>P00413 | COX2_WHEAT
MILRSLSCRFLTIALCDAAEPWQLGFQDAATPMMQGIIDLHHDIFFFLILILVFVLWMLV
RALWHFNEQTNPIPQRIVHGTTIEIIWTIFPSVILLFIAIPSFALLYSMDGVLVDPAITI
KAIGHQWYWTYEYSDYNSSDEQSLTFDSYTIPEDDPELGQSRLLEVDNRVVVPAKTHLRM
IVTPADVLHSWAVPSLGVKCDAVPGRLNLTSILVQREGVYYGQCSEICGTNHAFMPIVVE

AVTLKDYADWVSNQLILQTN
>P04373 | COX2_ORYSJ
MILRSLECRFLTIALCDAAEPWQLGSQDAATPMMQGIIDLHHDIFFFLILILVFVSRMLV
RALWHFNEQTNPIPQRIVHGTTIEIIRTIFPSVIPLFIAIPSFALLYSMDGVLVDPAITI
KAIGHQWYRSYEYSDYNSSDEQSLTFDSYTIPEDDPELGQSRLLEVDNRVVVPAKTHLRM
IVTPADVLHSWAVPSSGVKCDAVPGRSNLTSISVQREGVYYGQCSEICGTNHAFTPIVVE
AVTLKDYADWVSNQLILQTN
>P05488 | RT13_TOBAC
MLYISGARLVGDEQVRIASTKIDGIGPKKAIQVRYRLGISGNIKIKELTKYQIDQIEQMI
GQDHVVHWELKRGERADIERLISISCYRGIRHQDGSPLRGQRTHTNARTCRKLIRK
>P05490 | COX2_OENBE
MIVNECLFFTIALCDAAEPWQLGFQDAATPMMQGIIDLHHDILFFLILILVFVLWILVRA
LWHFYYKKNPIPQRIVHGTTIEILWTIFPSIILMFIAIPSFALLYSMDEVVVDPAMTLKA
IGHQWYWTYEYSDYNSSDEQSLTFDSYMIPEDDLELGQLRLLEVDNRVVVPVKTNLRLIV
TSADVLHSWAVPSLGVKCDAVPGRLNQISMLVQREGVYYGQCSEICGTNHAFMPIVIEAV
SATDYTNWVSNLFIPPTS
>P05491 | COX2_SOYBN
MKFEWLFLTIAPCDAAEPWQLGFQDAATPMMQGIIDLHHDIFFFLILILVFVSRILVRAL
WHFHYKKNPIPQRIVHGTTIEILRTIFPSIIPMFIAIPSFALLYSMDEVVVDPAITIKAI
GHQWYRTYEYSDYNSSDEQSLTFDSYTIPEDDLELGQSRLLEVDNRVVVPAKTHLRIIVT
PADVPHSWAVPSLGVKCDAVPGRLNQISISVQREGVYYGQCSEICGTNHAFTPIVVEAVP
SKDYGSRVFNQLIPQTTGEA
>P05492 | ATPAM_OENBI
MEFSPRAAELTTLLESRITNFYTNFQVDEIGRVISVGDGIARVYGLNEIQAGEMVEFASG
VKGIALNLENENVGIVVFGSDTAIKEGDLVKRTGSIVDVPAGKSLLGRVVDALGVPIDGR
GALGDHERRRVEVKVPGIIERKSVHEPMQTGLKAVDSLVPIGRGQRELIIGDRQTGKTAI
AIDTILNQKQMNSRATSESETLYCVYVAIGQKRSTVAQLVQILSEGNALEYSILVAATAS
DPAPLQFLAPYSGCAMGEYFRDNGMHALIIYDDLSKQAVAYRQMSLLLRRPPGREAFPGD
VFYLHSRLLERAAKRSDQTGAGSLTALPVIETQAGDVSAYIPTNVISITDGQICLETELF
YRGIRPAINVGLSVSRVGSAAQLKAMKQVCGSLKLELAQYREVAAFAQFGSDLDAATQAL
LNRGARLTEILKQPQYAPLPIEKQIIVIYAAVNGFCDRMPLDRISQYERAIPQSVKQELL
QSLVEKGGLNNERKIEPDAFLKENAKPYIKG
>P05493 | ATPAM_PEA
MEFSVRAAELTTLLESRITNFYTNFQVDEIGRVVSVGDGIARVYGLNEIQAGELVEFASG
VKGIALNLENENVGIVVFGSDTSIKEGDLVKRTGSIVDVPAGKAMLGRVVDALGVPIDGR
GALSDHERRRVEVKAPGIIERKSVHEPMQTGLKAVDSLVPIGRGQRELIIGDRQTGKTAI
AIDTILNQKQMNSRATSESETLYCVYVAIGQKRSTVAQLVQILSEANALEYSILVAATAS
DPAPLQFLAPYSGCAMGEYFRDNGMHALIIYDDLSKQAVAYRQMSLLLRRPPGREAFPGD
VFYLHSRLLERAAKRSDQTGAGSLTALPVIETQAGDVSAYIPTNVISITDGQICLETELF
YRGIRPAINVGLSVSRVGSAAQLKAMKQVCGSLKLELAQYREVAAFAQFGSDLDAATQAL
LNRGARLTEVLKQPQYAPLPIEKQILVIYAAVNGFCDRMPLDKIAQYERDILSTIKQELL
QSLKGGLTGERKIEPDAFLKEKALSLI
>P05494 | ATPAM_MAIZE
MEFSPRAAELTTLLESRMINFYTNLKVDEIGRVVSVGDGIARVYGLNEIQAGEMVEFASG

VKGIALNLENENVGIVVFGSDTAIKEGDLVKRTGSIVDVPAGKAMLGRVVDALGVPIDGK
GALSDHERRRVEVKAPGIIERKSVHEPMQTGLKAVDSLVPIGRGQRELIIGDRQTGKTAI
AIDTILNQKQMNSRGTNESETLYCVYVAIGQKRSTVAQLVQILSEANALEYSMLVAATAS
DPAPLQFLAPYSGCAMGEYFRDNGMHALIIYDDLSKQAVAYRQMSLLLRRPPGREAFPGD
VFYLHSRLLERAAKRSDQTGAGSLTALPVIETQAGDVSAYIPTNVISITDGQICLETELF
YRGIRPAINVGLSVSRVGSAAQLKAMKQVCGSSKLELAQYREVAAFAQFGSDLDAATQAL
LNRGARLTEVPKQPQYEPLPIEKQIVVIYAAVNGFCDRMPLDRISQYEKNILSTINPELL
KSFLEKGGLTNERKMEPDASLKESALNL
>P05495 | ATPAM_NICPL
MELSPRAAELTSLLESRISNFYTNFQVDEIGRVVSVGDGIARVYGLNEIQAGEMVEFASG
VKGIALNLENENVGIVVFGSDTAIKEGDLVKRTGSIVDVPAGKAMLGRVVDGLGVPIDGR
GALSDHERRRVEVKAPGIIERKSVHEPMQTGLKAVDSLVPIGRGQRELIIGDRQTGKTAI
AIDTILNQKQLNSRATSESETLYCVYVAIGQKRSTVAQLVQILSEANALEYSILVAATAS
DPAPLQFLAPYSGCAMGEYFRDNGMHALIIYDDLSKQAVAYRQMSLLLRRPPGREAFPGD
VFYLHSRLLERAAKRSDQTGAGSLTALPVIETQAGDVSAYIPTNVIPITDGQICLETELF
YRGIRPAINVGLSVSRVGSAAQLKTMKQVCGSSKLELAQYREVAALAQFGSDLDAATQAL
LNRGARLTEVPKQPQYAPLPIEKQILVIYAAVNGFCDRMPLDRISQYERAIPNSVKPELL
QSFLEKGGLTNERKMEPDTFLKESALAFI
>P05500 | ATP6_OENBE
MKRFYKTAFFSEIGSEEVSHFWADTMSSHSPLEQFSILPLIPMNIGNLYFSFTNSSLFML
LTLSLVLLLVNFVTKKGGGNLVPNAWQSLVELIYDFVLNLVNEQIGGLSGNVKQKFFPCI
LVTFTFLLFCNLQGMIPYSFTVTSHFLITLGLSFSIFIGITIVGFQRNGLHFLSFLLPAG
VPLPLAPFLVLLELISYCFRALSLGIRLFANMMAGHSLVKILSGFAWTMLCMNDLFYFIG
DLGPLFIVLALTGLELGVAILQAYVFTILICIYLNDAINLH
>P07506 | COX1_SOYBN
MTNPVRWLFSTNHKDIGTLYFIFGAIAGVMGTCFSVLIRMELARPGDQILGGNHQLYNVL
ITGHAFLMIFFMVMPAMIGGSGNWSVPILIGAPDMAFPRLNNISFWLLPPSLLLLLSSAL
VEVGSGTGWTVYPPLSGITSHSGGAVDSAISSLHLSGVSSILGSINFITTISNMRGPGMT
MHRSPLFVWSVPVTAFPLLLSLPVLAGAITMLLTDRNFNTTFSDPAGGGDPILYQHLFRF
FGHPEVYIPILPGSGIISHIVSTFSGKPVFGYLGMVYAMISIGVLGFLVWAHHMFTVGLD
VDTRAYFTAATMIIAVPTGIKIFSWIATMWGGSIQYKTPMLFAVGFIFLFTIGGLTGIVL
ANSGLDIALHDTYYVVAHFHYVLSMGAVFALFAGFHYWVGKIFGRTYPETLGQIHFWITF
FGVNLTLFPMHFLGLSGMPRRIPDYPDAYAGWNALSSFGSYISVVGIRRFFVVVTITSSS
GNNITRANIPWAVEQNSTTLEWLVQSPPAFHTFGELPAIKETKSYVK
>P07924 | RT13_WHEAT
MSYISGARSLPDEQVRIASTKMDGIGPKKAIQLRYRLGISGNIKMNELTKYQIDQIEQMI
AQDHVVHWELKRGERADIERLISISRYRGIRHQDGSPLRGQRTHTNARTARKQIRK
>P07925 | ATP6_MAIZE
MERNGEIVNNGSIIIPGGGGPVTESPLDQFGIHPILDLNIGKYYVSFTNLSLSMLLTLGL
VLLLVFVVTKKGGGKSVPNAFQSLVELIYDFVPNLVNEQIGGLSGNVKHKFFPCISVTFT
FSLFRNPQGMIPFSFTVTSHFLITLALSFSIFIGITIVGFQRHGLHFFSFLLPAGVPLPL
APFLVLLELISHCFRALSSGIRLFANMMAGHSSVKILSGFAWTMLFLNNIFYFLGDLGPL
FIVLALTGLELGVAISQAHVSTISICIYLNDATNLHQNESFHNCIKTRSQS
>P08681 | COX1_CHLRE

MRWLYSTSHKDIGLLYLVFAFFGGLLGTSLSMLIRYELALPGRGLLDGNGQLYNVIITGH
GIIMLLFMVMPALFGGFGNWLLPIMIGAPDMAFPRLNNISFWLNPPALALLLLSTLVEQG
PGTGWTAYPPLSVQHSGTSVDLAILSLHLNGLSSILGAVNMLVTVAGLRAPGMKLLHMPL
FVWAIALTAVLVILAVPVLAAALVMLLTDRNINTAYFCESGDLILYQHLFWFFGHPEVYI
LILPAFGIVSQVVSFFSQKPVFGLTGMICAMGAISLLGFIVWAHHMFTVGLDLDTVAYFT
SATMIIAVPTGMKIFSWMATIYSGRVWFTTPMWFAVGFICLFTLGGVTGVVLANAGVDML
VHDTYYVVAHFHYVLSMGAVFGIFAGVYFWGNLITGLGYHEGRAMVHFWLLFIGVNLTFF
PQHFLGLAGMPRRMFDYADCFAGWNAVSSFGASISFISVIVFATTFQEAVRTVPRTATTL
EWVLLATPAHHALSQVPVLRTASSH
>P08742 | COX1_MAIZE
MTNLVRWLFSTNHKDIGTLYFIFGAIAGVMGTCFSVLIRMELARPGDQILGGNHQLYNVL
ITAHAFLMIFFMVMPAMIGGFGNWFVPILIGAPDMAFPRLNNISFWLLPPSLLLLLSSAL
VEVGSGTGWTVYPPLSGITSHSGGAVDLAIFSLHLSGVSSILGSINFITTIFNMRGPGMT
MHRLPLFVWSVLVTAFLLLLSLPVLAGAITMLLTDRNFNTTFFDPAGGGDPILYQHLFWF
FGHPEVYILILPGFGIISHIVSTFSRKPVFGYLGMVYAMISIGVLGFLVWAHHMFTVGLD
VDTRAYFTAATMIIAVPTGIKIFSWIATMWGGSIQYKTPMLFAVGFIFLFTIGGLTGIVL
ANSGLDIALHDTYYVVAHFHYVLSMGAVFALFAGFYYWVGKIFGRTYPETLGQIHFWITF
FGVNLTFFPMHFLGLSGMPRRIPDYPDAYAGWNALSSFGSYISVVGIRRFFVVVAITSSS
GKNKRCAESPWAVEQNPTTLEWLVQSPPAFHTFGELPTIKETRNQSSC
>P08743 | COX1_OENBE
MTNPVRWLFSTNHKDIGTLYFIFGAIAGVMGTCFSVLIRMELARPGDQILGGNHQLYNVL
ITAHAFLMIFFMVMPAMIGGSGNWSVPILIGAPDMAFPRLNNISFWLLPPSLLLLLSSAL
VEVGSGTGWTVYPPLSGITSHSGGAVDSAISSLHLSGVSSILGSINFITTISNMRGLGMT
MHRSPLFVWSVLATAFPILLSLPVLAGAITMLLTDRNFNTTFSDPAGGGDPILYQHLFRF
FGHPEVYILILPGSGIISHIVSTFSGKPVFGYLGMVYAMISIGVLGFLVWAHHMFTVGLD
VDTRAYFTAATMIIAVPTGVKIFSWIATMWGGSIQYKTPMLFAVGSIFLFTVGGLAGIVP
ANSGLDIALHDTYYAGAHFHYVLSMGAVFALFAGFRYWVGKIFGRTYPETLGQIHFWITF
FGVNPTFFPMHFLGLSGMPRPIPDYPESYAGWNALSSFGSYISVVGIRCFFVVVTITSSS
GNNKRCAPSPWAVEKNSTTLEWMVQSPPAFHTFGELPATKETKSYVK
>P08744 | COX2_PEA
MKLEWLFLTIAPCDAAEPWQLGFQDAATPMMQGIIDLHHDIFFFLILILVFVSRILVRAL
WHFHYKKNPIPQRIVHGTTIEILRTIFPSIIPMFIAIPSFALLYSMDGVLVDPAMTIKAI
GHQWYRTYEYSDYNSSDEQSLTFDSYTIPEDDLELGQSGLLEVDNRVVVPAKTHLRIIVT
PADVPHSWAVPSLGVKCDAVPGRLNQISISVQREGVYYGQCSEICGTNHAFPIVVEAVPS
KDYGSRVSNQLIPQTGEA
>P08977 | RT13_MAIZE
MSYISGARSLPDEQVRIASTKMDGIGPKKAIQLRYRLGISGNIKIHELTKYQIDQIEQMI
AQDHVVHWELKRGERADIERLISISRYRGIRHQDGSPLRGQRTHTNARTARKQIRKGNER
RLPKEQATD
>P0C520 | ATPAM_ORYSA
MEFSPRAAELTTLLESRMTNFYTNFQVDEIGRVVSVGDGIARVYGLNEIQAGEMVEFASG
VKGIALNLENENVGIVVFGSDTAIKEGDLVKRTGSIVDVPAGKAMLGRVVDALGVPIDGK
GALSDHERRRVEVKAPGIIERKSVHEPMQTGLKAVDSLVPIGRGQRELIIGDRQTGKTAI
AIDTILNQKQMNSRGTNESETLYCVYVAIGQKRSTVAQLVQILSEANALEYSILVAATAS

DPAPLQFLAPYSGCAMGEYFRDNGMHALIIYDDLSKQAVAYRQMSLLLRRPPGREAFPGD
VFYLHSRLLERAAKRSDQTGAGSLTALPVIETQAGDVSAYIPTNVISITDGQICLETELF
YRGIRPAINVGLSVSRVGSAAQLKAMKQVCGSLKLELAQYREVAAFAQFGSDLDAATQAL
LNRGARLTEVSKQPQYEPLPIEKQIVVIYAAVNGFCDRMPLDRISQYEKAILSTINPELL
KSFNEKGGLTNERKIELDAFLKQTAKEIN
>P0C521 | ATPAM_ORYSI
MEFSPRAAELTTLLESRMTNFYTNFQVDEIGRVVSVGDGIARVYGLNEIQAGEMVEFASG
VKGIALNLENENVGIVVFGSDTAIKEGDLVKRTGSIVDVPAGKAMLGRVVDALGVPIDGK
GALSDHERRRVEVKAPGIIERKSVHEPMQTGLKAVDSLVPIGRGQRELIIGDRQTGKTAI
AIDTILNQKQMNSRGTNESETLYCVYVAIGQKRSTVAQLVQILSEANALEYSILVAATAS
DPAPLQFLAPYSGCAMGEYFRDNGMHALIIYDDLSKQAVAYRQMSLLLRRPPGREAFPGD
VFYLHSRLLERAAKRSDQTGAGSLTALPVIETQAGDVSAYIPTNVISITDGQICLETELF
YRGIRPAINVGLSVSRVGSAAQLKAMKQVCGSLKLELAQYREVAAFAQFGSDLDAATQAL
LNRGARLTEVSKQPQYEPLPIEKQIVVIYAAVNGFCDRMPLDRISQYEKAILSTINPELL
KSFNEKGGLTNERKIELDAFLKQTAKEIN
>P0C522 | ATPAM_ORYSJ
MEFSPRAAELTTLLESRMTNFYTNFQVDEIGRVVSVGDGIARVYGLNEIQAGEMVEFASG
VKGIALNLENENVGIVVFGSDTAIKEGDLVKRTGSIVDVPAGKAMLGRVVDALGVPIDGK
GALSDHERRRVEVKAPGIIERKSVHEPMQTGLKAVDSLVPIGRGQRELIIGDRQTGKTAI
AIDTILNQKQMNSRGTNESETLYCVYVAIGQKRSTVAQLVQILSEANALEYSILVAATAS
DPAPLQFLAPYSGCAMGEYFRDNGMHALIIYDDLSKQAVAYRQMSLLLRRPPGREAFPGD
VFYLHSRLLERAAKRSDQTGAGSLTALPVIETQAGDVSAYIPTNVISITDGQICLETELF
YRGIRPAINVGLSVSRVGSAAQLKAMKQVCGSLKLELAQYREVAAFAQFGSDLDAATQAL
LNRGARLTEVSKQPQYEPLPIEKQIVVIYAAVNGFCDRMPLDRISQYEKAILSTINPELL
KSFNEKGGLTNERKIELDAFLKQTAKEIN
>P12786 | COX1_PEA
MTNPVRWLFSTNHKDIGTLYFIFGAIAGVMGTCFSVLIRMELARPGDQILGGNHQLYNVL
ITAHAFFMIFFMVMPAMIGGSGNWSVPILIGAPDMAFPRLNNISFWLLPPSLLLLLSSAL
VEVGSGTGWTVYPPLSGITSHSGGAVDSAISSLHLSGVSSILGSINFLTTISNMRGPGMT
MHRSPLFVWSVPVTAFPLLLSLPVLAGAITMLLTDRNFNTTFSDPAGGGDPILYQHLFRF
FGHPEVYIPILPGSGIISHIVSTFSGKPVFGYLGMVYAMISIGVLGFLVWAHHMFTVGLD
VDTRAYFTAATMIIAVPTGIKIFSWIATMWGGSIQYKTPMLFAVGFIFLFTIGGLTGIVP
ANSGLDIALHDTYYVVAHFHYVLSMGAVFALFAGFHYWVGKIFGRTYPETLGKIHFWITF
FGVNLTLFPMHFLGLSGMPRRIPDYPDAYAGWNALSSFGSYISVVGIRRFFVVVTITSSS
GNNITRANIPWAVEQNSTTLEWLVQSPPAFHTFGELPAIKETKSYVK
>P12857 | ADT2_MAIZE
MADQANQPTVLHKLGGQFHLSSSFSEGVRARNICPSFSPYERRFATRNYMTQSLWGPSMS
VSGGINVPVMPTPLFANAPAEKGGKNFMIDFMMGGVSAAVSKTAAAPIERVKLLIQNQDE
MIKSGRLSEPYKGIADCFKRTIKDEGFSSLWRGNTANVIRYFPTQALNFAFKDYFKRLFN
FKKDRDGYWKWFAGNLASGGAAGASSLFFVYSLDYARTRLANDAKAAKGGGDRQFNGLVD
VYRKTLKSDGIAGLYRGFNISCVGIIVYRGLYFGLYDSIKPVVLTGSLQDNFFASFALGW
LITNGAGLASYPIDTVRRRMMMTSGEAVKYKSSLDAFQQILKKEGPKSLFKGAGANILRA
IAGAGVLSGYDQLQILFFGKKYGSGGA
>P12862 | ATPAM_WHEAT

MEFSPRAAELTTLLESRMTNFYTNFQVDEIGRVVSVGDGIARVYGLNEIQAGEMVEFASG
VKGIALNLENENVGIVVFGSDTAIKEGDLVKRTGSIVDVPAGKAMLGRVVDALGVPIDGK
GALSDHERRRVEVKAPGIIERKSVHEPMQTGLKAVDSLVPIGRGQRELIIGDRQTGKTAI
AIDTILNQKQMNSRGTNESETLYCVYVAIGQKRSTVAQLVQILSEANALEYSILVAATAS
DPAPLQFLAPYSGCAMGEYFRDNGMHALIIYDDLSKQAVAYRQMSLLLRRPPGREAFPGD
VFYLHSRLLERAAKRSDQTGAGSSTALPVIETQAGDVSAYIPTNVISITDGQICLETDVF
YRGIRPAINVGLSVSRVGSAAQLKAMKQVCGSSKLELAQYREVAAFAQFGSDLDAASQAL
LNRGARLTEVPKQPQYEPLPIEKQIVVIYAAVNGFCDRMPLDRISQYEKAILSTINPELQ
KSFLEKGGLTNERKMEPDASLKESTLPYL
>P14578 | COX1_ORYSJ
MTNLVRWLFSTNHKDIGTLYFIFGAIAGVMGTCFSVLIRMELARPGDQILGGNHQLYNVL
ITAHAFLMIFFMVMPAMIGGFGNWFVPILIGAPDMAFPRLNNISFWLLPPSLLLLLSSAL
VEVGSGTGWTVYPPLSGITSHSGGAVDLAIFSLHLSGVSSILGSINFITTIFNMRGPGMT
MHRLPLFVWSVLVTAFLLLLSLPVLAGAITMLLTDRNFNTTFFDPAGGGDPILYQHLFWF
FGHPEVYILILPGFGIISHIVSTFSRKPVFGYLGMVYAMISIGVLGFLVWAHHMFTVGLD
VDTRAYFTAATMIIAVPTGIKIFSWIATMWGGSIQYKTPMLFAVGFIFLFTIGGLTGIVL
ANSGLDIALHDTYYVVAHFHYVLSMGAVFALFAGFYYWVGKIFGRTYPETLGQIHFWITF
FGVNLTFFPMHFLGLSGMPRRIPDYPDAYAGWNALSSFGSYISVVGIRRFFVVVAITSSS
GKNKRCAESPWAVEQNPTTLEWLVQSPPAFHTFGELPAIKETKS
>P14875 | RT14_OENBE
MEKRNIRDHKRRLLATKYELRRKLYKAFCNDPALPSDMRDKHRYKLSKLPRNSSFARVRN
RCISTGRPRSVYEFFRISRIVFRGLASRGPLMGIKKSSW
>P15451 | CYC_CHLRE
MSTFAEAPAGDLARGEKIFKTKCAQCHVAEKGGGHKQGPNLGGLFGRVSGTAAGFAYSKA
NKEAAVTWGESTLYEYLLNPKKYMPGNKMVFAGLKKPEERADLIAYLKQATA
>P15758 | RT13_OENBE
MSYISGARLVADEQVRIASTKMDGIGPKKAIQVRSRLGGNIKRKELTKYQIDQIEQMRGQ
DHVVHWELKRGERADIERFISISCYRGIRHQDGLPLRGQRSHTNARTSRKRIRK
>P16048 | GCSH_PEA
MALRMWASSTANALKLSSSSRLHLSPTFSISRCFSNVLDGLKYAPSHEWVKHEGSVATIG
ITDHAQDHLGEVVFVELPEPGVSVTKGKGFGAVESVKATSDVNSPISGEVIEVNTGLTGK
PGLINSSPYEDGWMIKIKPTSPDELESLLGAKEYTKFCEEEDAAH
>P16265 | NU3M_MAIZE
MLEFAPICIYLVISLLVSLILLGVPFLFASNSSTYPEKLSAYECGFDPFGDARSRFDIRF
YLVSILFIIFDLEVTFFFPWAVSLNKIDLFGFWSMMAFLLILFIGSLYEWKRGALDWE
>P17614 | ATPBM_NICPL
MASRRLLASLLRQSAQRGGGLISRSLGNSIPKSASRASSRASPKGFLLNRAVQYATSAAA
PASQPSTPPKSGSEPSGKITDEFTGAGSIGKVCQVIGAVVDVRFDEGLPPILTALEVLDN
QIRLVLEVAQHLGENMVRTIAMDGTEGLVRGQRVLNTGSPITVPVGRATLGRIINVIGEA
IDERGPITTDHFLPIHREAPAFVEQATEQQILVTGIKVVDLLAPYQRGGKIGLFGGAGVG
KTVLIMELINNVAKAHGGFSVFAGVGERTREGNDLYREMIESGVIKLGEKQSESKCALVY
GQMNEPPGARARVGLTGLTVAEHFRDAEGQDVLLFIDNIFRFTQANSEVSALLGRIPSAV
GYQPTLATDLGGLQERITTTKKGSITSVQAIYVPADDLTDPAPATTFAHLDATTVLSRQI
SELGIYPAVDPLDSTSRMLSPHILGEDHYNTARGVQKVLQNYKNLQDIIAILGMDELSED

DKMTVARARKIQRFLSQPFHVAEVFTGAPGKYVDLKESINSFQGVLDGKYDDLSEQSFYM
VGGIDEVIAKAEKIAKESAA
>P18260 | ATPAM_HELAN
MEFSPRAAELTTLLESRISNFYTNFQVDEIGRVVSVGDGIARVYGLNEIQAGEMVEFASG
VKGIALNLENENVGIVVFGSDTAIKEGDLVKRTGSIVDVPAGKAMLGRVVDALGVPIDGR
GALSDHERRRVEVKAPGIIERKSVHEPMQTGLKAVDSLVPIGRGQRELIIGDRQTGKTAI
AIDTILNQKQMNSRSTSESETLYCVYVAIGQKRSTVAQLVQILSEANAMEYSILVAATAS
DPAPLQFLAPYSGCAMGEYFRDNGMHALIIYDDLSKQAVAYRQMSLLLRRPPGREAFPGD
VFYLHSRLLERAAKRSDQTGAGSLTALPVIETQAGDVSAYIPTNVIPITDGQICSETELF
YRGIRPAINVGLSVSRVGSAAQLKTMKQVCGSSKLELAQYREVAALAQFGSDLDAATQAL
LNRGARLTEVPKQPQYAPLPIEKQILVIYAAVNGFCDRMPLDRISQYERAILKSIKTELL
QSLLEKGGLTNERKMEPDTFLKECALPYTI
>P18630 | NU3M_OENBE
MLEFAPICISLVISLLLSLILLVVPFLFSSNSSTYPEKLSAYECGFDPFGDARSRFDIRF
YLVSILFIIFDLEVTFFFPWAVSFNKIDLFGFWSMMAFLLILTIGFLYEWKRGALDWE
>P20113 | NU4M_CHLRE
MFISVLLILFALCVTLIPEAHYHMVRVWSFVATIIPMWVVTWMWWNFDASGHGLQMLVIL
GRSHLAFGIDGVALSLMLLTTVLFPICMMLLRTVAGFMTFILLEVLVLSALCVLDLLGFY
ILFEASLILLFLLIGRAPYGSLEAAYKIVLYTMAGSLVLLPTLFMIYSECGTTNVLYMTC
AYNHQTVLGWGLLAVLAVKIPLMPVHLWLPEAHVAAPTAGSVLLAGVLLKLGGIGFLRFM
LPVVPEFCVSVFPLVSTLCLVSFLFSTLSTLRQIDLKKIVAYSSIAHMSMVTLAIFSQSE
FSAYSSSFLMIAHGLISPALFLIVGILYDRAHTKFILYFSGLGASMPIGSTLFFLFTLGN
LAFPLFPNFIAEVLCMVSIFAVHELLAYVFCVCQVLGAAYGFWAFNRVVHGLPRGPADVT
RTEFHTVLPLLIGAVWLGIKPMA
>P22201 | ATPAM_BRANA
MELSPRAAELTNLFESRIRNFYANFQVDEIGRVVSVGDGIAQVYGLNEIQAGEMVLFANG
VKGMALNLENENVGIVVFGGDTAIKEGDLVKRTGSIVDVPAGKAMLGRVVDAMGVPIDGR
GALSDHEQRRVEVKAPGILERKSVHEPMQTGLKAVDSLVPIGRGQRELLIGDRQTGKTTI
AIDTILNQKQINSRATSESETMYCVYVAIGQKRSTVGQLIQTLEEANALEYSILVAATAS
DPAPLQFLAPYSGCAMGEYFRDNGMHALIIYDDLSKQAVAYRQMSLLLRRPPGREAFPGD
VFYLHSRLLERAAKRSDQTGAGSLTALPVIETQAGDVSAYIPTNVISITDGQICLETELF
YRGIRPAINVGLSVSRVGSAAQLKAMKQVCGSSKLELAQYREVAAFAQFGSDLDAATQAL
LNRGARLTEVPKQPQYAPLPIEKQILVIYAAVNGFCDRMPLDRISQYEKAIPNSVKPELL
QALKGGLTNERKMEPDAFLKERALRLI
>P23209 | RT13_DAUCA
MLYISGARLVADKQVRIALTKMYGIGPKKAIQVCYRLGISGNIKIKELTKYQIDQMEQMI
GQDHVVHWELKRGERADIERFISISCYRGIRHQDGLPLRGQRTHTNARTCRKQIRK
>P24459 | ATPAM_PHAVU
MEFSSRAAELTTLLESRMTNFYTNFQVDEIGRVVSVGDGIARVYGLNEIQAGEMVEFASG
VKGIALNLENENVGIVVFGSDTAIKEGDLVKRTGSIVDVPAGKAMLGRVVDALGVPIDGR
GALSDHERRRVEVKAPGIIERKSVHEPMQTGLKAVDSLVPIGRGQRELIIGDRQTGKTAI
AIDTILNQKQMNSRATSESETLYCVYVAIGQKRSTVAQLVQILSEANALEYSILVAATAS
DPAPLQFLAPYSGCAMGEYFRDNGMHALIIYDDLSKQAVAYRQMSLLLRRPPGREAFPGD
VFYLHSRLLERAAKRSDQTGAGSLTALPVIETQAGDVSAYIPTNVISITDGQICLETELF

YRGIRPAINVGLSVSRVGSAAQLKAMKQACGSLKLELAQYREVAAFAQFGSDLDAATQAL
LNRGARLTEVLKQPQYAPLPIEKQILVIYAAVNGFCDRMPLDKIPQYERDILTTIKPELL
QSLKGGLTSERKIELEKFLKEKAKNYTL
>P24794 | COX1_BETVU
MTNLVRWLFSTNHKDIGTLYFIFGAIAGVMGTCFSVLIRMELARPGDQILGGNHQLYNVL
ITAHAFLMIFFMVMPAMIGGFGNWFVPILIGAPDMAFPRLNNISFWLLPPSLLLLLSSAL
VEVGSGTGWTVYPPLSGITSHSGGAVDLAIFSLHLSGVSSILGSINFITTIFNMRGPGMT
MHRLPLFVWSVLVTAFLLLLSLPVLAGAITMLLTDRNFNTTFFDPAGGGDPILYQHLFWF
FGHPEVYILILPGFGIISHIVSTFSGKPVFGYLGMVYAMISIGVLGFLVWAHHMFTVGLD
VDTRAYFTAATMIIAVPTGIKIFSWIATMWGGSIQYKTPMLFAVGFIFLFTVGGLTGIVL
ANSGLDIALHDTYYVVAHFHYVLSMGAVFALFAGFYYWVGKIFGRTYPETLGQIHFWITF
FGVNLTFFPMHFLGLSGMPRRIPDYPDAYAGWNALSSFGSYISVVGICCFFVVVTITLSS
GKNKRCAPSPWAVEENSTTLEWMVQSPPAFHTFGELPAIKETKS
>P25083 | ADT1_SOLTU
MADMNQHPTVFQKAANQLDLRSSLSQDVHARYGGVQPAIYQRHFAYGNYSNAGLQRGQAT
QDLSLITSNASPVFVQAPQEKGFAAFATDFLMGGVSAAVSKTAAAPIERVKLLIQNQDEM
LKAGRLSEPYKGIGECFGRTIKEEGFGSLWRGNTANVIRYFPTQALNFAFKDYFKRLFNF
KKDRDGYWKWFAGNLASGGAAGASSLFFVYSLDYARTRLANDRKASKKGGERQFNGLVDV
YKKTLKSDGIAGLYRGFNISCVGIIVYRGLYFGMYDSLKPVLLTGNLQDSFFASFGLGWL
ITNGAGLASYPIDTVRRRMMMTSGEAVKYKSSLDAFSQIVKNEGPKSLFKGAGANILRAV
AGAGVLAGYDKLQVLVLGKKFGSGGA
>P25855 | GCSH1_ARATH
MALRMWASSTANALKLSSSVSKSHLSPFSFSRCFSTVLEGLKYANSHEWVKHEGSVATIG
ITAHAQDHLGEVVFVELPEDNTSVSKEKSFGAVESVKATSEILSPISGEIIEVNKKLTES
PGLINSSPYEDGWMIKVKPSSPAELESLMGPKEYTKFCEEEDAAH
>P26846 | NU2M_MARPO
MFEHDFLALFPEIFLINATIILLIYGVVFSTSKKYDYPPLVRNVGWLGLLSVLITILLVA
VGSPLAVANLVYNNLIIDNFTYFCQIFLLLSTASTMVMCLDYFKQESLNAFESIVLILLS
TCSMLFMISAYDLIAMYLAIELQSLCFYVIAASKRDSEFSTEAGLKYFILGAFSSGILLF
GCSMIYGFTGVTNFEELAKIFTGYEITLFGAQSSGIFMGILFIAVGFLFKITAVPFHMWA
PDVYEGSPTIVTAFFSIAPKISILANMLRVFIYSFYDPTWQQLFFFCSIASMILGALAAM
AQNKVKRLLAYSSIGHVGYLLIGFSCGTIEGIQSLLIGIFIYVLMTVNVFAIVLALRQNR
FKYIADLGALAKTNPILAITLSITMFSYAGIPPLAGFCSKFYLFFAALGCGAYLLLALIGV
VTSVISCFYYIRFVKIMYFDTPKTWVLYKPMDREKSLLLAITVFFITFFFLYPSPLFLVT
HQMALCLCL
>P26847 | NU3M_MARPO
MEFAPIFVYLVISLLLSLILIGVSFLFASSSSLAYPEKLSAYECGFDPFDDARSRFDIRF
YLVSILFIIFDLEVTFLFPWAVSLNKIGLFGFWSMMVFLFILTIGFVYEWKKGALDWE
>P26848 | NU4M_MARPO
MLQVLAPFYSNLSGLILLPLLGSLIILVIPNSRVRLIRGITIWTSLITFLYSLFFWIRFE
NDTAKFQFVETIRWLPYSNINFYIGIDGISLFFVILTTFLTPICILVGFYSVKSYKKEYM
IAFFICESFLIAVFCSLDLLIFYVFFESVLIPMFIIIGVWGSRQRKIKAAYQFFLYTLMG
SLFMLLAILFIFFQTGTTDLQILLTTEFSERRQILLWIAFFASFSVKVPMVPVHIWLPEA
HVEAPTAGSVILAGILLKLGTYGFLRFSIPMFPEATLYFTPFIYTLSVIAIIYTSLTTIR

QIDLKKIIAYSSVAHMNFVTIGMFSLNIQGIEGSILLMLSHGLVSSALFLCVGALYDRHK
TRIVKYYGGLVSTMPIFSTIFLFFTLANMSLPGTSSFIGEFLILVGAFQRNSLVATLAAL
GMILGAAYSLWLYNRVVFGNFKPNFILKFSDLNRREVLIFLPFIVGVIWMGVYPEVFLEC
MHTSVSNLVQHGKFD
>P26850 | NU6M_MARPO
MILFYVFVVLALVSGAMVIRAKNPVHSVLFLILVFCNTSGLLVLLGLDFFAMIFLVVYVG
AIAVLFLFVVMMLHIRIEEIHENVLRYLPVGGIIGLIFLLEIFLMVDNDYIPILPTKLSA
TYLTYTVYAGKIHSWTNLETLGNLLYTTYFFLFLVSSLILLVALIGAIVLTMHKTTKVKR
QDVFIQNAIDFQNTIKKVR
>P26853 | ATP6_MARPO
MACSPLEQFAIIQLIPIHIGNLYFSFTNSSLFMLLTISLVLLLVHFVTLNGGNLVPNAWQ
SFVEMIYDFVLNLVNEQISGASSVKQRFFPLIYVTFTFLLFCNLIGMIPYSFTVTSHFII
TLGLSFSLFIGITIVGFQTHGLHFFSILLPQGVPLPLAPFLVLLELISYCFRALSLGIRL
FANMMAGHSLVKILSGFAWTMLSMGGILYLGQLAPFFIVFALTGLELGVAILQAYVFTIL
LCIYLNDAINLH
>P26856 | COX1_MARPO
MNNFAQRWLFSTNHKDIGTLYLIFGAIAGVMGTCFSVLIRMELAQPGNQILGGNHQLYNV
LITAHAFLMIFFMVMPAMIGGFGNWFVPILIGSPDMAFPRLNNISFWLLPPSLLLLLSSA
LVEVGCGSGWTVYPPLSGITSHSGGSVDLAIFSLHLSGVSSILGSINFITTIFNMRAPGL
TMHRLPLFVWSVLVTAFLLLLSLPVLAGAITMLLTDRNFNTTFFDPAGGGDPILYQHLFW
FFGHPEVYILILPGFGIISHIVSTFSRKPVFGYLGMVYAMISIGVLGFIVWAHHMFTVGL
DVDTRAYFTAATMIIAVPTGIKIFSWIATMWGGSIQYKTPMLFAVGFIFLFTVGGLTGIV
LANSGVDIALHDTYYVVAHFHYVLSMGAVFALFAGFYYWIGKITGLQYPETLGQIHFWIT
FFGVNLTFFPMHFLGLAGMPRRIPDYPDAYAGWNAFSSFGSYVSVVGIFCFFVVVFLTLT
SENKCAPSPWAVEQNSTTLEWMVPSPPAFHTFEELPAIKESI
>P26857 | COX2_MARPO
MNLIWIFPIAFCDAAEPWQLGFQDPATPMMQGIIDLHNDIFFFLIVILIFVLWMLVRALW
HFHYKRNPIPERIVHGTTIEIIWTIFPSIILMFIAIPSFALLYSMDEVVDPAITIKAIGH
QWYWTYEYSDYNSSDEQSLTFDSYMIPEDDLELGQLRLLEVDNRVVVPAKTHLRMIITSA
DVLHSWAVPSLGVKCDAVPGRLNQTSIFIKREGVYYGQCSELCGTNHGFMPIVVEAVSLD
DYVSWVSNKLD
>P26859 | RM02_MARPO
MRNSCWKGKALKQLTFHLKRNSAGRNSSGRITVFHRGGGSKRLQRKIDFKRSTSSMGIVE
RIEYDPNRSSWIALVRWIEGVLRPGKRLAFSKANSRREKNMFFFGLLFSFSSLPRQAQRI
KYEKTRALRPCEQILESSWVLGTRDLRAKEVSLGPLGSFLGLPSIAVAGAKPAFFAFRMK
GPSSLTGRERLSPLRGENTFSQSEGQRWKTQSGAPRRKSLVLSWSQGPKARNGLMISAHD
IGKKDRRPEMAGPHTIPEHAPRALHAVGPSGSGRVLRTSEPFTYILASENLEVGNTVMNF
HGSKPSTLLNYHQPSQKANDPSGLRVEETAWDSQAWLHPRGDYASSENKYILDSYYQMVG
NCIPLAKIPIGTWVHNIERNPGQGAKLTRAAGTFAQIIQKVENTPQCIVRLPSGVDKLID
SRCRATIGIVSNLNHGKRKFNKAGQSRWLGRRPIVRGVAMNPVDHPHGGGEGRTKGGRPS
VSPWGKPTKGGFKTVVRKRRN
>P26860 | RM05_MARPO
MFSPNRNRLEFHYNQVIRPDLLLKINYENIMEVPRLCKIIVVPKAPSNFIKNVKLAMEIV
CGQKFIQTRSRGSTGKSFRFNKFVLNQESKRDTGYVTYLARSTLRGHIMYNFLEKLVTII

SFYDYPVKIQKNSIQLSMATSLLRLFPEIQDHFEIFEHIRGFDVTIVTSANTQDETVILW
SGFLQKEV
>P26861 | RM06_MARPO
MEAKFFCFLEIIGVGYKASTNAQGSILYLKLGFSHEIRLQVTPSVRVFCLKPNLICCTGM
DHQKVTQFAAIVKSCKPPEVYKGKGIQYRNEIIHKKQGKKK
>P26862 | RM16_MARPO
MLYPKRTKFRKYQKGRCKGCKADGTQLCFGKYGIKSCEAGRISYQAIEAARRAISRKFRR
NSKIWVRVFADIPITSKPAEVRMGKGKGNTKGWIARVLKGQILFEMDCVSLSNAQQAATL
AAHKLGLSIKFFKWS
>P26864 | RT02_MARPO
MYNSNLLVIQKLLSTNAYLGHRIPTSDFQGYLYGFRNEMAIIDLEKTLICLRRTCNLIGS
IISAKGHLLLVNTNPEYNKIIQQMAKKTNQSYINHKWIGGFLTNWKHMKKVKKHFQDFSA
HPNLKDAFTSSPFDYFPRFKKMQKCFEGIMTHNIPDCLVIINANQNSMAILEANQLQIPI
VALVDSNIPNRLHKLITYPVPVNDDSIKFVYLFCNLITKTVILSKRSQRPKVKVKRL
>P26865 | RT03_MARPO
MAQKVNPISVRLNLNRSSDSSWFSDYYYGKLLYQDLNFRDYFGSIRPPTGNTFGFRLGRC
IIHHFPKRTFIHVFFLDRLSQSRHQGLGAIPSVKLIRRINDNTVKQRNEVGIWPKKRYEY
HDRLPSIQKIDQLLRVSDWMADIHSTFQSIWPKDENDDRRASEERYAFSRFAPSILVAVR
AEKKKAIFGSEGDFFGFTGRAFLDYFVMQYFFNLKNQIQFDPMVNRSPVAQGVAKTSMIG
KAIPAKTEQGTQSGESICQPRSTLYFDAIIFLRYARFRKATSLSSRYYYLKKMQSLFSNQ
TKTNTLIQPVKIASVYQSASLIAQEISWKLEQKKSFRQICRSIFKQIKKCPYVKGIRIGC
SGRLNGAEIAKTECKKYGETSLHVFSDQIDYAKTQASTPYGILGVKVWVSYFLTQKKGTS
CAISKTYKIS
>P26866 | RT04_MARPO
MFASRFKVCRQILENVWQTKKLTLKQKFLISELQKQKKNKKQSDFSIQLQTIKKLSLFYG
NLPIKKMQRAKTHTYIDKKNSLLFNIEKRLDVILVRLNFCSTMFQARQLISHKNICVNYK
KVNIPGFQVSNGDLISIQENSLDFFKSNIRKNFQTNRIRRMKPNHLEVNYKTLKAVVLYE
PQQIQFPYKIDLDLLD
>P26869 | RT10_MARPO
MTAKICIVIKSFENQRSGLLLNTRKIGLPKKQTLYTVLRSPHIDKKSREQFEMRIHKQLL
VIETETHKLREKLNWLKLHDLLGVQVKIIFYYQTRLDKVCKS
>P26870 | RT11_MARPO
MQKKHGITNMQKKHCITYIQSTFGNTIITLTDYNGNTKTWSSSGSVGFKGSRRSTNYAAQ
ATAENAARVAIQLGFKFVEVRIKGLGYGKESSLRGLKLGGLIITKIRDVTPTPHNGCRPP
KKRRV
>P26871 | RT12_MARPO
MPTMNQLVRKGRESKRRTKRTRALNKCPQKQGVCLRVSTRSPKKPNSALRKIAKVRLTNR
NEIIAYIPGEGHNLQEHSVVMVRGGRVQDLPGVKYHCIRGVKDLQGIPGRRRGRSKYGTK
KPKDYI
>P26872 | RT13_MARPO
MSYILGTNLNSNKQVKIALTRIFGIGPKKAIQVCDQLGLSDTIKVNKLTKYQFDQILKII
SQNYLVDSELKRVIQRDIKRLISIGCYRGFRHNAGLPLRGQRTHTNAKTCRKLRYVSIRS

>P26873 | RT14_MARPO

MSNQIIRDHKRRLLVAKYELKRMHYKAICQDRNLPNKIRYEYFFKLSKLPRNSSKTRVRN
RCIFTGRPRSVYKLFRISRIVFRELASKGSLIGINKSCW
>P26874 | RT19_MARPO
MTRSIWKGPFVDTCLFKQKKIRWRIWSRRSCILPQFVGCYAQIYNGKGFVGLKITEEMVG
HKFGEFASTRKTSSLGKRALPSKTKIKPIKKVR
>P27062 | NU3M_PANGI
MSEFAPICIYLVISLLVSLIPLGVPFPFASNSSTYPDKLSAYECGFDPSGDARSRFDIRF
YLVSILFIIPDPEVTFSFPWAVPPNKIDPFGSWSMMAFLLILTIGSLYEWKRGASDRE
>P27070 | RT12_PANGI
MPTLNQLIRHGREEKRRTDRTRASDQCPQKQGVRPRVPTRTPKKPNSAPRKIAKVRLANR
HDIFPHIPGEGHNSQEHSMVLIRGGRVKDSPGVKSHCIRGVKDLLGIPDRRRGRSKYGAE
KPKSI
>P27080 | ADT_CHLRE
MAKEEKNFMVDFLAGGLSAAVSKTAAAPIERVKLLIQNQDEMIKQGRLASPYKGIGECFV
RTVREEGFGSLWRGNTANVIRYFPTQALNFAFKDKFKRMFGFNKDKEYWKWFAGNMASGG
AAGAVSLSFVYSLDYARTRLANDAKSAKKGGGDRQFNGLVDVYRKTIASDGIAGLYRGFN
ISCVGIVVYRGLYFGMYDSLKPVVLVGPLANNFLAAFLLGWGITIGAGLASYPIDTIRRR
MMMTSGSAVKYNSSFHCFQEIVKNEGMKSLFKGAGANILRAVAGAGVLAGYDQLQVILLG
KKYGSGEA
>P27084 | SODM_PEA
MAARTLLCRKTLSSVLRNDAKPIGAAIAAASTQSRGLHVFTLPDLAYDYGALEPVISGEI
MQIHHQKHHQTYITNYNKALEQLHDAVAKADTSTTVKLQNAIKFNGGGHINHSIFWKNLA
PVSEGGGEPPKESLGWAIDTNFGSLEALIQKINAEGAALQWLGLDKDLKRLVVETTQDPL
VTKGASLVPLLGIDVWEHAYYLQYKNVRPDYLKNIWKVINWKHASEVYEKESS
>P27527 | RT19_PETHY
MPRRSIWKGSFVDAFLLRMKKKRDLLFNRKIWSRRSSILPEFVDCFVRIYNGKTFVRCKI
TEGKVGHKFGEFAFTRKRRPSRTNIGPGRKRGKK
>P27572 | NU4M_WHEAT
MLEHFCECYFDLSGLILCPVLGSIILLFIPNSSIRLIRLIGLCVSLITFLYSLVLWIQFD
PSTAKFQFVESLRWLPYENIHLYMGIDGLSLFFVILTTFLIPICILVGWSGMRSFGKEYI
IAFLICEFLMIAVFCMLDLLLFYVFFESVLIPMFIIIGVWGSRQRKIKAAYQFFLYTLLG
SVFMLLAILLILLQTGTTDLQILLTTEFSERRQILLWIAFFASFAVKVPMVPVHIWLPEA
HVEAPTAGSVILAGILLKLGTYGFLRFSIPMFPEATLCFTPFIYTLSAIAIIYTSLTTLR
QIDLKKIIAYSSVAHMNLVTIGMFSLNIQGIGGSILLMLSHGLVSSALFLCVGVLYDRHK
TRLVRYYGGLVSTMPNFSTIFFFFTLANMSLPGTSSFIGEFLILVGAFQRNSLVATLRAL
GMILGAAYSLWLYNRVVSGNLKPDFLYKFSDLNGREVFIFLPFLVGVVWMGVYPKVFLDC
MHTSVSNLVQHGKFH
>P27754 | RT03_OENBE
MARKGNPISVRLDLNRSSDSSWFSDYYYGKSVYQDVNLRSYFGSIRPPTRLTFGFRLGRC
IILHFPKRTFIHFFLPRRPRRLKRGKKSRPGKEKARWWEFGKVGPIGCLHSNDDTEEERN
EVGGRGAGKRVESIRLDDREKQNEIRIWPKKKQGYGYHDRSPSIKKNLSKSLRVSGAFKH
PKYAGVLNDIAFLIENDDSFRKTKLFKFFFPKKSRSDGPTSHLLKRTPPAVRPSLNYSVM
QYYLNTKNQMHFDPLVVLNHFVAPGVAEPSTMGGANRQGRSNELRIRSRIAFFVESSTSE
KKSLAEDKKGLPHFIRQENDLRFAGRTKTTISLFPFFGATFFFPRDGVGVYKHLFFEDAR

EQLLGQLRIKCWKLMGKDKVMELIEKFIDLGGIGELIKGIEIMIEIILRNRRIPYGYNSY
LNEVKKMRSLLSNRTKTNTIIESVKIKSVYQSASPIAQDISFQPRNKRRSFRSIFSQIVK
DIPLVMKKGVEGIRICCSGRSKGAEIARTECGKYGKTSRNVFNQKIDYAPAEVSTRYGIL
GVKVWISYSK
>P27928 | RT03_MAIZE
MARKGNPISVRLDLNRSSDPSRFSDYYYGKSLYQDVNLRSYFSSIRPPTRLTFGFRLGRC
IILHFPKRTFIHFFLPRRPLRLKRRDKSRPGKDKGRWWAFGKVGPIGCLHSSEGTEEERN
EVRGRGAGKRVESIDREKQNEIRIWPKKMQRYGYHDRTPSRKKNFSKSLRVSGAFKHPKY
AGVVNDIAFLIENDDSFIKTKLFKFFFLPKKSRSDGPTSHLLKRTLPAVRPSLNYSVMQY
FFNTKNKMHFDPVVVLNHFVAPGVAEPSTMGGAKGGSLDKRIRSRIAFFVESSTSDKKCL
ARAKKRLIHFIRQANDLRFAGTTKTTISLFPFFGATFFFSRDGVGVYNNPFYEYAREQLL
GQLRIKCRNLMGKDKVMELIEKFIDLGRIGKLIKGIEMMIEIILRKRRIPYGYNSYLNEV
QKMRSFLSNRTNTNTLIESVKIKSVYQSASLIAQDISFQLRNNPISFRSIFSKIVKDIPL
IMPKGVEGIRICCSGRLGGAEIARTECGKYGKTSCNVFNQKIDYAPAEVSTRDGISGVKV
RISYSQNKKGRAISETYEI
>P28520 | RT12_ORYSJ
MPTKNQLIRHGREEKQRTDRTRASDQCPQKQGVCLRVSTRTPKKPNSALRKIAKVRLSNR
HDIFAHIPGEGHNSQEHSIVLVRGGRVKDSPGVKSHRIRGVKDLLGIPDRRKGRSKYGAE
RPKSK
>P29057 | HMDH1_HEVBR
MDTTGRLHHRKHATPVEDRSPTTPKASDALPLPLYLTNAVFFTLFFSVAYYLLHRWRDKI
RNSTPLHIVTLSEIVAIVSLIASFIYLLGFFGIDFVQSFIARASHDVWDLEDTDPNYLID
EDHRLVTCPPANISTKTTIIAAPTKLPTSEPLIAPLVSEEDEMIVNSVVDGKIPSYSLES
KLGDCKRAAAIRREALQRMTRRSLEGLPVEGFDYESILGQCCEMPVGYVQIPVGIAGPLL
LNGREYSVPMATTEGCLVASTNRGCKAIYLSGGATSVLLKDGMTRAPVVRFASATRAAEL
KFFLEDPDNFDTLAVVFNKSSRFARLQGIKCSIAGKNLYIRFSCSTGDAMGMNMVSKGVQ
NVLEFLQSDFSDMDVIGISGNFCSDKKPAAVNWIEGRGKSVVCEAIIKEEVVKKVLKTNV
ASLVELNMLKNLAGSAVAGALGGFNAHAGNIVSAIFIATGQDPAQNVESSHCITMMEAVN
DGKDLHISVTMPSIEVGTVGGGTQLASQSACLNLLGVKGANKESPGSNSRLLAAIVAGSV
LAGELSLMSAIAAGQLVKSHMKYNRSSKDMSKAAS
>P29185 | CH61_MAIZE
MYRAAASLASKARQAGNSLATRQVGSRLAWSRNYAAKDIKFGVEARALMLRGVEELADAV
KVTMGPKGRNVVIEQSFGAPKVTKDGVTVAKSIEFKDRVKNVGASLVKQVANATNDTAGD
GTTCATVLTKAIFTEGCKSVAAGMNAMDLRRGISMAVDAVVTNLKGMARMISTSEEIAQV
GTISANGEREIGELIAKAMEKVGKEGVITIADGNTLYNELEVVEGMKLDRGYISPYFITN
SKTQKCELEDPLILIHDKKVTNMHAVVKVLEMALKKQKPLLIVAEDVESEALGTLIINKL
RAGIKVCAVKAPGFGENRKANLQDLAILTGGEVITEELGMNLENFEPHMLGTCKKVTVSK
DDTVILDGAGDKKSIEERAEQIRSAIENSTSDYDKEKLQERLAKLSGGVAVLKIGGASEA
EVGEKKDRVTDALNATKAAVEEGIVPGGGVALLYASKELDKLQTANFDQKIGVQIIQNAL
KTPVHTIASNAGVEGAVVVGKLLEQENTDLGYDAAKGEYVDMVKTGIIDPLKVIRTALVD
AASVSSLMTTTESIIVEIPKEEAPAPAMGGGMGGMDY
>P29380 | CYC1_ARATH
MQVADISLQGDAKKGANLFKTRCAQCHTLKAGEGNKIGPELHGLFGRKTGSVAGYSYTDA
NKQKGIEWKDDTLFEYLENPKKYIPGTKMAFGGLKKPKDRNDLITFLEEETK

>P29677 | MPPA_SOLTU
MYRCASSRLSSLKARQGNRVLTRFSSSAAVATKPSGGLFSWITGDTSSSVTPLDFPLNDV
KLSPPLPDYVEPAKTQITTLANGLKVASEASVNPAASIGLYVDCGSIYETPASYGATHLL
ERMAFKSTLNRSHLRIVREIEAIGGNVTASASREHMIYTYDALKTYVPQMVEMLADCVRN
PAFLDWEVKEQLEKVKAEISEYSKNPQHLLLEAVHSAGYAGPYGNSLMATEATINRLNST
VLEEFVAENYTAPRMVLAASGVEHEEFLKVAEPLLSDLPKVATIEEPKPVYVGGDYRCQA
DAEMTHFALAFEVPGGWMSEKESMTLTVLQMLMGGGGSFSAGGPGKGMYSRLYLRVLNQY
PQIHAFSAFSSIYNNTGLFGIQGTTSSDFGPQAVDVAVKELIAVANPSEVDQVQLNRAKQ
ATKSAILMNLESRMVASEDIGRQLLTYGERNPVEHFLKAIDAVSAKDIASVVQKLISSPL
TMASYGDVLSLPSYDAVSSRFRSK
>P29685 | ATPBM_HEVBR
MASRRLLSSLLRSSSRRSVSKSPISNINPKLSSSSPSSKSRASPYGYLLTRAAEYATSAA
AAAPPQPPPAKPEGGKGGGKITDEFTGKGAIGQVCQVIGAVVDVRFDEGLPPILTSLEVL
DHSIRLVLEVAQHMGEGMVRTIAMDGTEGLVRGQRVLNTGSPITVPVGRANPWTYHEVIG
EPIDERGDIKTSHFLPIHREAPAFVDQATEQQILVTGIKVVDLLAPYQRGGKIGLFGGAG
VGKTVLIMELINNVAKAHGGFSVFAGVGERTREGNDLYREMIESGVIKLGDKQADSKCAL
VYGQMNEPPGARARVGLTGLTVAEHFRDAEGQDVLLFIDNIFRFTQANSEVSALLGRIPS
AVGYQPTLATDLGGLQERITTTKKGSITSVQAIYVPADDLTDPAPATTFAHLDATTVLSR
QISELGIYPAVDPLDSTSRMLSPHILGEEHYNTARGVQKVLQNYKNLQDIIAILGMDELS
EDDKLTVARARKIQRFLSQPFHVAEVFTGAPGKYVELKESITSFQGVLDGKYDDLPEQSF
YMVGGIDEVIAKADKIAKESAS
>P31167 | ADT1_ARATH
MVDQVQHPTIAQKAAGQFMRSSVSKDVQVGYQRPSMYQRHATYGNYSNAAFQFPPTSRML
ATTASPVFVQTPGEKGFTNFALDFLMGGVSAAVSKTAAAPIERVKLLIQNQDEMIKAGRL
SEPYKGIGDCFGRTIKDEGFGSLWRGNTANVIRYFPTQALNFAFKDYFKRLFNFKKDRDG
YWKWFAGNLASGGAAGASSLLFVYSLDYARTRLANDAKAAKKGGGGRQFDGLVDVYRKTL
KTDGIAGLYRGFNISCVGIIVYRGLYFGLYDSVKPVLLTGDLQDSFFASFALGWVITNGA
GLASYPIDTVRRRMMMTSGEAVKYKSSLDAFKQILKNEGAKSLFKGAGANILRAVAGAGV
LSGYDKLQLIVFGKKYGSGGA
>P31691 | ADT_ORYSJ
MAEQANQPTVLQKFGGQFHLGSSFSEGVRARNICPSVSSYDRRFTTRSYMTQGLVNGGIN
VPMMSSSPIFANAPAEKGGKNFMIDFLMGGVSAAVSKTAAAPIERVKLLIQNQDEMIKAG
RLSEPYKGIGDCFGRTIKDEGFASLWRGNTANVIRYFPTQALNFAFKDYFKRLFNFKKDK
DGYWKWFGGNLASGGAAGASSLFFVYSLDYARTRLANDAKAAKGGGERQFNGLVDVYRKT
LKSDGIAGLYRGFNISCVGIIVYRGLYFGMYDSLKPVVLTGSLQDNFFASFALGWLITNG
AGLASYPIDTVRRRMMMTSGEAVKYKSSMDAFSQILKNEGAKSLFKGAGANILRAIAGAG
VLSGYDQLQILFFGKKYGSGGA
>P31692 | ADT_CHLKE
MLSSALYQQAGLSGLLRASAMGPQTPFIASPKETQADPMAFVKDLLAGGTAGAISKTAVA
PIERVKLLLQTQDSNPMIKSGQVPRYTGIVNCFVRVSSEQGVASFWRGNLANVVRYFPTQ
AFNFAFKDTIKGLFPKYSPKTDFWRFFVVNLASGGLAGAGSLLIVYPLDFARTRLAADVG
SGKSREFTGLVDCLSKVVKRGGPMALYQGFGVSVQGIIVYRGAYFGLYDTAKGVLFKDER
TANFFAKWAVAQAVTAGAGVLSYPFDTVRRRLMMQSGGERQYNGTIDCWRKVAQQEGMKA
FFKGAWSNVLRGAGGAFVLVLYDEIKKFINPNAVSSASE

>P35017 | SODM_HEVBR
MALRSLVTRKNLPSAFKAATGLGQLRGLQTFSLPDLPYDYGALEPAISGEIMQLHHQKHH
QTYITNYNKALEQLNDAIEKGDSAAVVKLQSAIKFNGGGHVNHSIFWKNLAPVREGGGEL
PHGSLGWAIDADFGSLEKLIQLMNAEGAALQGSGWVWLALDKELKKLVVETTANQDPLVT
KGPTLVPLLGIDVWEHAYYLQYKNVRPDYLKNIWKVMNWKYASEVYAKECPSS
>P37399 | ATPBM_DAUCA
MASRRLLSSFLRSSTRRSLRPSFSNPRPSFLTSYCSSPASILRRYATAAPAKEPAASKPA
GTAGTGKGTITDEKTGAGAIGQVCQIIGAVVDVKFEEGLPPIMTALEVIDFEIRLVLEVA
PSLGENTVRTIAMDGTEGLVRGQKVLNTGAPITIPVGRATLGRIINVIGEPIDHRGEIKT
DQYLPIHREAPTFVDQATEQQILVTGIKVVDLLAPYQKGGKIGLFGGDWVGKTVLIMELI
NNVAKAHAVFAGVGERTREGNDLYKEMMESGVIKLGDQQAESKCALVYGQMNEPPGSRAR
VGLTGLTVAEHFRDAEGEDVLLFVDKRFRFTQANSEVSALLGRIPSAVGYQPTLATDLGG
LQERITTTKKGSITSVQAIYVPADDLTDPAPATTFAHLDATTVLSRQISELGIYPAVDPL
DSTSRMLTPESGEEHYNTARGVQKVLQNYKNLQDIIAILGMDELSEDDKLTVARARKIQR
FLSQPFHVAEIFTGAPGKYVELKECVTSFQGVLDGKYDDLPEQSFYMLGGIEEVIAKAEK
MAKENPQ
>P37900 | HSP7M_PEA
MAATLLRSLQRRNLSSSSVSAFRSLTGSTKTSYATHKLASLTRPFSSRPAGNDVIGIDLG
TTNSCVSVMEGKNPKVIENSEGARTTPSVVAFNQKSELLVGTPAKRQAVTNPTNTLFGTK
RLIGRRFDDAQTQKEMKMVPYKIVRAPNGDAWVEANGQQYSPSQIGAFVLTKIKETAEAY
LGKTISKAVVTVPAYFNDAQRQATKDAGRIAGLDVQRIINEPTAAALSYGMNNKEGLIAV
FDLGGGTFDVSILEISNGVFEVKATNGDTFLGGEDFDNALLDFLVSEFKRTESIDLAKDK
LALQRLREAAEKAKIELSSTSQTEINLPFISADASGAKHLNITLTRSKFEALVNNLIERT
KAPCKSCLKDANISIKDVDEVLLVGGMTRVPKVQQVVSEIFGKSPSKGVNPDEAVAMGAA
LQGGILRGDVKELLLLDVTPLSLGIETLGGIFTRLISRNTTIPTKKSQVFSTAADNQTQV
GIKVLQGEREMAADNKSLGEFDLVGIPPAPRGLPQIEVTFDIDANGIVTVSAKDKSTGKE
QQITIRSSGGLSDDEIDKMVKEAELHAQRDQERKALIDIRNSADTSIYSIEKSLAEYREK
IPAEVAKEIEDAVSDLRTAMAGENADDIKAKLDAANKAVSKIGQHMSGGSSGGPSEGGSQ
GGEQAPEAEYEEVKK
>P41978 | SODM2_MAIZE
MALRTLASKNALSFALGGAARPSAASARGVTTVALPDLSYDFGALEPVISGEIMRLHHQK
HHATYVVNYNKALEQLDAVVVKGDASAVVQLQGAIKFNGGGHFNHSIFWENLKPISEGGE
PPHGKLGWAIDEDFGSFEALVKRMNAEGAALQGSGWVWLALDKEPKKLSVETTANQDPLV
TKGASLVPLLGIDVWEHAYYLQYKNVRPDYLNNIWKVMNWKYAGEVYENVLA
>P41979 | SODM3_MAIZE
MALRTLASKNALSFALGGAARPSAESARGVTTVALPDLSYDFGALEPVISGEIMRLHHQK
NHATYVVNYNKALEQIDDVVVKGDDSAVVQLQGAIKFNGGGHVNHSIFWKNLKPISEGGG
EPPHGKLGWAIDEDFGSFEALVKRMNAEGAALQGSGWVWLALDKEAKKVSVETTANQDPL
VTKGASLVPLLGIDVWEHAYYLQYKNVRPDYLNNIWKVMNWKYAGEVYENVLA
>P41980 | SODM4_MAIZE
MALRTLASKNALSFALGGAARPSAASARGVTTVALPDLSYDFGALEPAISGEIMRLHHQK
HHATYVGNYNKALEQLDAAVAKGDASAVVQLQGAIKFNGGGHVNHSIFWKNLKPISEGGG
EPPHGKLGWAIDEDFGSFEALVKRMNAEGAALQGSGWVWLALDKEPKKLSVETTANQDPL
VTKGASLVPLLGIDVWEHAYYLQYKNVRPDYLNNIWKVMNWKYAGEVYENVLA

>P42027 | NDUS7_BRAOL
MAMITRNTATRLPLVLQSHRAAAVSHLHTSLPALSPATTPTSYTRPGPPSTSAPPPGLSK
TAEFVISKVDDLMNWARRGSIWPMTFGLACCAVEMMHTGAARYDLDRFGIIFRPSPRQSD
CMIVAGTLTNKMAPALRKVYDQMPEPRWVISMGSCANGGGYYHYSYSVVRGCDRIVPVDI
YVPGCPPTAEALLYGLLQLQKKINRRKDFLHWWNK
>P42056 | VDAC2_SOLTU
MVKGPGLYSDIGKKARDLLYRDYVSDHKFTVTTYSTTGVAITASGLKKGELFLADVSTQL
KNKNITTDVKVDTNSNVYTTITVDEPAPGLKTIFSFVVPDQKSGKVELQYLHEYAGINTS
IGLTASPLVNFSGVAGNNTVALGTDLSFDTATGNFTKCNAGLSFSSSDLIASLALNDKGD
TVSASYYHTVKPVTNTAVGAELTHSFSSNENTLTIGTQHLLDPLTTVKARVNSYGKASAL
IQHEWRPKSLFTISGEVDTRAIEKSAKIGLAVALKP
>P42793 | RM05_ARATH
MFPLNFHYEDVLRQDLLLKLNYANVMEVPGLCEIRVVPKAPYNFIIKNGKLAMEIPCGQK
FIQTQRGSTGKSFRSNPFLGSNKDKGYVSDLARQSTLRGHGMSNFLVRILTVMSLLDFPV
EIRKNSIQFSMETEFCEFSPELEDHFEIFEHIRGFNVTIITSANTQDETLLLWSGFLQKD
EGETQ
>P46274 | VDAC1_WHEAT
MGGPGLYSGIGKKAKDLLYRDYQTDHKFTLTTYTANGPAITATSTKKADLTVGEIQSQIK
NKNITVDVKANSASNVITTITADDLAAPGLKTILSFAVPDQKSGKVELQYLHDYAGINAS
IGLTANPVVNLSGAFGTSALAVGADVSLDTATKNFAKYNAALSYTNQDLIASLNLNNKGD
SLTASYYHIVEKSGTAVGAELTHSFSSNENSLTFGTQHTLDPLTLVKARINNSGKASALI
QHEFMPKSLCTISAEVDTKAIEKSSKVGIAIALKP
>P46485 | GCSH_FLATR
MALRIWASSTAKALRLSSASRPHFSPLFRCFSSAAVLDGLKYANSHEWVKHEGSVATIGI
TDHAQDHLGEVVFVELPEVGGSVTKATGFGAVESVKATSDVNSPISGEIVEVNSKLTKTP
GLINKSPYEDGWMIKVKPSNPSELDSLMGPKEYTKFCEEEGAAAH
>P46740 | RT03_PROWI
MGQKTNPISLRLQNVNRNFDSCWYSDYFYAKCFSRDLYLNNYINTFFKLYRLPQARVCVN
FGIQNIKVYPFFCIPKASRVSLAKNLGLFQHLSKAWNSSPKYFVSSKSKQLSQLKINDAS
LPVNNLNNNLFFNSSKHNEIRNKQNFFHNLELNKLNLSFKNSAYSDVKNYSFLENISAKL
LLNFKEKSVINEVYYTTTNLGQIGNNISTNSIYSKQDMSDNSSKSLLKMLLREYYKSSDN
KSSILYENKSPKSLENIFGKINEVSNINKKNLYLLNSETDNIPNLYKRKSKPTKFLRNEH
NKVIEDKKTSLYFMPDSNQIKNDIYQGKISKISSISNNSKDSLTDYNLHLYQAYLHNILG
NSKSLVSRNQFKYKNYIENFLSSQYNIDSQLFPFISKQNWQSAGFIADEIVYFIERRVSF
SRIKNRILRQASMQSYVRGIRITCSGRVGGKSKKAQRATQECVKYGETSLHVFECKIDFA
SRIANTSFGLVGIKVWICFK
>P46742 | RT10_PROWI
MQQVQLKLKSFDPVYINQLISLLNDVLDTLEIQNSKEIFLPSKIKKITVIRSPHIHKKSR
DQFQIKRYKRSMIISFTNIDILHAFLEICKDLHVVGVQIHISVKYHS
>P46744 | RT12_PROWI
MPTINQLLRKKSSRQAPKLKSKKPALAGCPQKRGVCFRVYTRTPKKPNSALRKVAKIRLC
NGIVVIASIPGEGHNLQEHSVVCIRGGRVKDLPGVKYKVVRGRLDLQGVVNRKQSRSLYG
TPKSK
>P46745 | RT07_PROWI

MNAPITQKNTAYISSDKLSLLELENSNKFINLLMVDGKKSRAIRLFYDTLVLLKRKNLKE
NSKKESLLGIIGQLSPKNSIEDLVNNSNKLSNNSNEEIKIPLIDNSKSDDKSNLLETISV
LEVLSIALKNVTPSVELRKVRRAGNTFLIPAILSQHKANTLAIRWVIESAKKKQQNSKQN
FAECLADEIYQAYLKQGKARQKRDELHSAAISNRANIRYRWW
>P46747 | RT13_PROWI
MVYIQNTHLNDKKQIYQACAQIYGLGHHHCLQICDVLGVSPETRLGMLSTGQHTLLAQII
TQNYDTGSDVRRFTRQNIQRLVNIHSYRGYRHIQGLPVRGQRSHGNARTVRKLKNVIKI
>P46750 | RT19_PROWI
MARSLSKPPFCEVKLATNNSVTKIWSRRSAILPQFVGKTVSIHNGRIFIPCKISPEMIGH
KFGEFAVTRKKPIHKKKK
>P46751 | RM16_PROWI
MLQPNNTKFRKFQKSRVKGVQSNTDQLRYGKFGIKTVSAARIPARTIEAVRRVITRKFKR
LGVIWIRVFPDIAVSGKPAEVRMGKGKGAPQYWVCKVKRGAILFEFDGISPQLAKQAARL
ADSKLPIKTRFVMYS
>P46752 | RT14_PROWI
MFNSIKRDLKRRKLYKKYESKRLLYKALISDCNLNQDLRFILTQKLNKLPRNSSQVRVKN
RCILTGRGHSVYKFCRISRIKFRDLANQGLIQGCVKSSW
>P46773 | RT03_ORYSJ
MARKGNPISVRLDLNRSSDPSRFSDYYYGKSLYQDVNLRSYFSSIRPPTILTFGFRLGRC
IILHFPKRTFIHFFLPRRPLRLKRRDKSRPGKDKGRWWAFGKVGPIGCLHSSEGTEEERN
EVRGRGAGKRVESIDREKQNEIRIWPKKMQRYGYHDRSPSRKKNFSKSLRVSGAFKHPKY
AGVVNDIAFLIENDGPTSHLLKRTLPAVRPSLNYSVMQYFFNTKNKMHFDPVVVLNHFVA
PGVAEPSTMGGAKGGSLDKRIRSRIAFFVESSTSEKKCLARAKKRLIHFIRQANDLRFAG
TTKTTISLFPFFGATFFFPRDGVGVYNNPFFEYAREQLLGQLRIKCRNLMGKDKVMELIE
KFIYLGRIGKLIKGIEMMIEIILRKRIIPYGYNSYLNEVQKMRSFLSNRTNTNTLIESVK
IKSVYQSASLIAQDISFQLGNNPISFRSIFSQIVKDIPLIMPKGVEGIRICCSGRLGGAE
IARTECGKYGKTSCNVFNQKIDYALAEVSTRNGISGVKVRISYSQNKKGRAISETYEI
>P46801 | RM16_ORYSJ
MEKHLVMYLTRKSIMLLRKYPLVTEFQVSKCGSHIVKIRRDVLYPKRTKYSKYSKCRCSR
GCEPDGTQLGFGRYGTKSCRAGRLSYRAIEAARRATIGQFHRAMSGQFRRNCKIWVRVLA
DLPITGKPAEVRMGRGKGNPTGWIARVSTGQIPFEMDGVSLSNARQAARLAAHKPCSSTK
FVQWS
>P48857 | RT12_BRANA
MPTFNQLIRHGREEKRRTDRTRALDKCPQKLGACLRVSTRTPKKPNSALRKIAKVRLSNR
HDIFAYIPGEGHNLQEHSQVLIRGGRVKDLPGVKFHCIRGVKDLMGIPGRRRGRSKYGAE
KPKSI
>P49357 | GLYM_FLAPR
MAMALALRRLSSSADKPLQRLFNGGHLYSMSSLPSEAVYEKERPGVTWPKQLNAPLEVVD
PEIADIIELEKARQWKGLELIPSENFTSLSVMQAVGSVMTNKYSEGYPGARYYGGNEYID
MAETLCQKRALEAFRLDPAKWGVNVQPLSGSPANFHVYTALLKAHDRIMALDLPHGGHLS
HGYQTDTKKISAVSIFFETMPYRLNESTGYIDYDQLEKSATLFRPKLIVAGASAYARLYD
YARIRKVCDKQKAIMLADMAHISGLVAAGVIPSPFDYADVVTTTTHKSLRGPRGAMIFFR
KGLKEVNKQGKEVFYDYEDKINQAVFPGLQGGPHNHTITGLAVALKQATTAEYKAYQEQV
MSNSAKFAETLVKSGYELVSGGTENHLVLVNLKNKGIDGSKVEKVLEAVHIAANKNTVPG

DVSAMVPGGIRMGTPALTSRGFVEEDFAKVAYFFDLAVKLAVKIKGEAKGTKLKDFVTAM
ESSAIQSEISKLRHDVEEYAKQFPTIGFEKETMKYKN
>P49358 | GLYN_FLAPR
MAMASALRRLSSSSNKPLQRLFNGGHLYSMSSLPSEAVYEKERPGVTWPKQLNAPLEVGD
PEIADIIELEKARQWKGLELILSENFTSLSVMQAVGSVMTNKYSEGYPGARYYGGNEYID
MAETLCQKRALEAFRLDAAKWGVNVQPLSGSPANFHVYTALLKAHDRIMALDLPHGGHLS
HGYQTDTKKISAVSIFFETMPYRLNESTGYIDYDQLEKSATLFRPKLIVAGASAYARLYD
YARIRKVCDKQKAILLADMAHISGLVAAGVIPSPFDYADVVTTTTHKSLRGPRGAMIFFR
KGVKEVNKQGKEVLYDYEDKINQAVFPGLQGGPHNHTITGLAVALKQATTAEYKAYQEQV
MSNCAKFAETLVKSGYELVSGGTENHLVLVNLKNKGIDGSRVEKVLEAVHIAANKNTVPG
DVSAMVPGGIRMGTPALTSRGFVEEDFAKVAYLFDLAVKLAVKIKGEAQGTKLKDFVAAM
QSSAFQSEISKLRHDVEEYAKQFPTIGFEKETMKYKN
>P49359 | GCSH_FLAPR
MALRIWASSTANALRLSSATRPHFSPLSRCFSSVLDGLKYANSHEWVKHEGSVATIGITD
HAQDHLGEVVFVDLPEAGGSVTKATGFGAVESVKATSDVNSPISGEIVEVNSKLSETPGL
INSSPYEDGWMIKVKPSNPSELDSLMGAKEYTKFCEEEDSAH
>P49361 | GCSPA_FLAPR
MERARRLANKAILGRLVSQTKHNPSISSPALCSPSRYVSSLSPYVCSGTNVRSDRNLNGF
GSQVRTISVEALKPSDTFPRRHNSATPEEQTKMAEFVGFPNLDSLIDATVPKSIRLDSMK
YSKFDEGLTESQMIAHMQDLASKNKIFKSFIGMGYYNTSVPTVILRNIMENPGWYTQYTP
YQAEIAQGRLESLLNFQTMVTDLTGLPMSNASLLDEGTAAAEAMAMCNNIQKGKKKTFII
ASNCHPQTIDICKTRADGFDLKVVTSDLKDFDYSSGDVCGVLVQYPGTEGELLDYSEFIK
NAHANGVKVVMASDLLALTILKPPGELGADIVVGSAQRFGVPMGYGGPHAAFLATSQEYK
RMMPGRIIGVSVDSSGKPALRMAMQTREQHIRRDKATSNICTAQALLANMAAMFGVYHGP
EGLKTIAKRVHGLAGTFAAGLKKLGTVQVQDLPFFDTVKVTCVDSKAIAEEAYKHKMNLR
IVDKNTITVAFDETTTIEDVDTLFKVFALGKPVTFTAASIAPEVQDAIPSGLVRETPYLT
HPIFNMYHTEHELLRYISKLQSKDLSLCHSMIPLGSCTMKLNATTEMMPVTWPAFADIHP
FAPTEQAQGYQEMFKNLGDLLCTITGFDSFSLQPNAGAAGEYAGLMVIRAYHMARGDHHR
NVCIIPVSAHGTNPASAAMCGMKIITVGTDSKGNINIEELRKAAEANKENLSALMVTYPS
THGVYEEGIDEICKIIHDNGGQVYMDGANMNAQVGLTSPGWIGADVCHLNLHKTFCIPHG
GGGPGMGPIGVKKHLAPYLPSHPVVATGGIPAPEQSQPLGTIAAAPWGSALILPISYTYI
AMMGSQGITNASKIAILNANYMAKRLENHYPILFRGVNGTVAHEFIVDLRPLKTTAGIEP
EDVAKRLIDYGFHGPTMSWPVPGTLMIEPTESESKAELDRFCDALISIRQEIAEIEKGNV
DLNNNVIKGAPHPPQLLMADKWTKPYSREYAAYPAPWLRAAKFWPTTCRVDNVYGDRNLI
CTLQPPQEYEEKAEATA
>P49362 | GCSPB_FLAPR
MERARRLAILGRLVSQTKHNPSISSPALCSPSRYVSSLSPYVCSGTNVRSDRNLNGFGSQ
VRTISVEALKPSDTFPRRHNSATPEEQTKMAEFVGFPNLDSLIDATVPKSIRLDSMKYSK
FDEGLTESQMIAHMQDLASKNKIFKSFIGMGYYNTSVPTVILRNIMENPGWYTQYTPYQA
EIAQGRLESLLNFQTMITDLTGLPMSNASLLDEGTAAAEAMAMCNNIQKGKKKTFIIASN
CHPQTIDICKTRADGFDLKVVTSDLKDFDYSSGDVCGVLVQYPGTEGELLDYSEFIKNAH
ANGVKVVMASDLLALTILKPPGELGADIVVGSAQRFGVPMGYGGPHAAFLATSQEYKRMM
PGRIIGVSVDSSGKPALRMAMQTREQHIRRDKATSNICTAQALLANMAAMFGVYHGPEGL
KTIAKRVHGLAGTFASGLKKLGTVQVQDLPFFDTVKVTCADSKAIAEEAYKHKMNLRIVD

KNTITVAFDETTTIEDVDTLFKVFALGKPVTFTAASIAPEVQDAIPSGLVRETPYLTHPI
FNMYHTEHELLRYISKLQSKDLSLCHSMIPLGSCTMKLNATTEMMPVTWPAFADIHPFAP
TEQAQGYQEMFKNLGDLLCTITGFDSFSLQPNAGAAGEYAGLMVIRAYHMARGDHHRNVC
IIPVSAHGTNPASAAMCGMKIITVGTDSKGNINIEELRKAAEANKENLSALMVTYPSTHG
VYEEGIDEICKIIHDNGGQVYMDGANMNAQVGLTSPGWIGADVCHLNLHKTFCIPHGGGG
PGMGPIGVKKHLAPYLPSHPVVPTGGIPAPEQSQPLGTIAAAPWGSALILPISYTYIAMM
GSQGITNASKIAILNANYMAKRLENHYPILFRGVNGTVAHEFIVDLRPLKTTAGIEPEDV
AKRLIDYGFHGPTMSWPVPGTLMIEPTESESKAELDRFCDALISIRQEIAEIEKGNVDFN
NNVIKGAPHPPQLLMADKWTKPYSREYAAYPAPWLRAAKFWPTTCRVDNVYGDRNLICTL
QPPQEYEEKAEATA
>P49363 | GCST_FLAPR
MRGGLWQLGQSITRRLGQSDKKTIARRCYASEADLKKTVLYDFHVANGGKMVPFAGWSMP
IQYKDSIMESTINCRENGSLFDVSHMCGLSLKGKDCVPFLEKLVVADVAGLRPGTGSLTV
FTNEKGGAIDDSVITKVTDDHIYLVVNAGCRDKDLAHIEEHMKAFKAKGGDVSWHIYDER
SLLALQGPLAGSTLQHLTKEDLSKMYFGDFRIIDINGSKCFLTRTGYTGEDGFEISVPSE
NAVDLAKAILEKSEGKVRLTGLGARDSLRLEAGLCLYGNDMEQHITPVEAGLTWAIGKRR
RAEGGFLGADVILKQIADGPAIRRVGLFSTGPPARSHSEIQNEKGENIGEVTSGGFSPCL
KKNIGMGYVKSGLHKPGTKLKIVIRGKTYEGSVTKMPFVPTKYYKPA
>P49386 | RT03_BRANA
MARKGNPISVRLGKNRSSDSSRFSEYYYGKFVYQDVNLRSYFGSIRPPTRLTFGFRLGRC
ILLHFPKRTFIHFFLPRRPRRLKRREKTRPGKEKGRWWTTPGKAGPIGCLRDDTEEERNE
VRGRGARKRVESIRLDDRKKQNEIRGWPKKKQRYGYHDRTPSIKKNLSKSLRISGAFKHP
KYGGVVNDIAFLIENDDSFRKTKLFKFFFPKKSRSDGPTSYLRTLPAVGPSLNFLVMQYF
FNTKNQMNFDPVVVLNHFVAPGAAEPSTMGRANGTGDRSLQKRIRSRIAFFVESSTSEKK
CLAEAKNRLTHLIRLANDLGFAGTTKTTISLFPFFGATFFFLRDGVGVTNNLDAREQLLN
QLRVKCWNLLGKDKVMELIEKFKDLGGIEELIKVIDMMIEIILRKRGIPYGYNSYFNEVQ
KMRSFLSNRTNTKTLIESVKIKSVYQSASLIAQDISFQLKNKRRSTHSIFAKIVKEIPKR
VEGIRICFSGRLKDAAEKAQTKCYKHRKTSRNVFNQKIDYAPAEVSTRYGISGVKVWISY
SQKKGGRAISETYEI
>P49387 | RT14_BRANA
MSEKQNSRDHKRRLLAAKFELRRKLYKAFCKDPDLPSDMRDKHRYKLSKLPRNSSFARVR
NRCISTGRPRSVSEFFRIYRIVFRGLASRGSLMGIKKSSW
>P49388 | RM05_BRANA
MFPLNFHYEDVSRQDPLLNRITPTLWKFLGSCEIRVVPNGPYNFIIKNGKLAMEIPRGQK
FIQTQRGSTGKSFRSNPFLGSNKDKGYVSDLARQSTLRGHGMSNFSVRISTVMSLLDFPV
EIRKNSIQFSMETEFCEFAPQLQDHFEIFEHIRGFNVTIVTSANTQDETLPLWSGFLQKD
EGETQ
>P49389 | RM16_BRANA
MYLTRKSIMLLRKYLLVTESQVSKCGFHIVKKKGDVLYPKRTKYSKGRCSRGCKPDGTKL
GFGRYGTKSCRAGRLSYRAIEAARRATIGHSFRRAMSGQFRRNCKIWVRVLADLPITGKP
AEVRMGRGKGNPTGWIARVSTGQIPFEMDGVSLANARQAARLAAHKPCSSTKFVQWS
>P50433 | GLYM_SOLTU
MAMAIALRRLSATVDKPVKSLYNGGSLYYMSSLPNEAVYDKEKSGVAWPKQLNAPLEVVD
PEIADIIEHEKARQWKGLELIPSENFTSVSVMQAVGSVMTNKYSEGYPGARYYGGNEYID

MAETLCQKRALEAFRLDPAKWGVNVQPLSGSPANFQVYTALLKPHERIMALDLPHGGHLS
HGYQTDTKKISAVSIFFETMPYRLDESTGYIDYDQLEKSATLFRPKLIVAGASAYARLYD
YDRIRKVCNKQKAILLADMAHISGLVAAGVIPSPFDYADVVTTTTHKSLRGPRGAMIFYR
KGVKEVNKQGKEVFYDYEDKINQAVFPGLQGGPHNHTITGLAVALKQATTPEYRAYQEQV
LSNSSKFAQALGEKGYELVSGGTDNHLVLVNMKNKGIDGSRVEKVLEAVHIAANKNTVPG
DVSAMVPGGIRMGTPALTSRGFLEEDFVKVADFFDAAVKIAVKVKAETQGTKLKDFVATL
ESSAPIKSEIAKLRHDVEEYAKQFPTIGFEKETMKYKN
>P50892 | RT12_RAPSA
MPTLNQLIRHGREEKRRTDRTRALDKCPQKLGACPRVSTRTPKKPNSAPRKIAKVRLSNR
HDIFAHIPGEGHNSQEHSQVLIRGGRVKDSPGVKSHCIRGVKDLMGIPGRRSGRSKYGAE
KPKSI
>P50893 | RT19_PLASU
MSRAIWKGPFIDPFFFRKNGSSNSNNKIYSRRSVVSPKFIGREVEIYNGHKWITIKIKED
MIGHKFGEFAFTRKATIHKKKTK
>P51132 | UCRI2_TOBAC
MLRIAGRRASSLSRWPVRSVAPSSSAFISANHFSSDDDSSSPRSISPSLASVFLHHTRGF
SSNSVSHAHDMGLVPDLPPTVAAIKNPTSKIVYDEHNHERYPPGDPSKRAFAYFVLTGGR
FVYASLVRLLILKFVLSMSASKDVLALASLEVDLSSIEPGTTVTVKWRGKPVFIRRRTED
DINLANSVDLGSLRDPQQDAERVKSPEWLVVIGVCTHLGCIPLPNAGDFGGWFCPCHGSH
YDISGRIRKGPAPYNLEVPTYSFLEENKLLIG
>P51135 | UCRI5_TOBAC
MLRIAGRKLSSSAAARSSSAFFTRNPFTFTDDSSSPTRSPSPTSLASQFLDQFRGFSSNS
VSPAHQTGLVSDLPATVAAIKNPSSKIVYDDSNHERYPPGDPSKRAFAYFVLTGGRFVYA
SLVRLLILKFVLSMSASKDVLALASLEVDLSSIEPGTTVTVKWRGKPVFIRRRTDEDINL
ANSVDLGSLRDPQQDAERVKNPEWLVVIGVCTHLGCIPLPNAGDFGGWFCPCHGSHYDIS
GRIRKGPAPYNLEVPTYSFMEENKLLIG
>P51409 | RM05_SOLTU
MDQLMFPLYFHYEDVLRQDLLLKLNYANVMEVPGLCKIIVVPKTAPSIKNGKLAMEISCG
QKLKQRASTGKSFRSNPFLGSNKDKKGYVSDLARQSTLRGHGMSHFLVRISTVMSLLDSP
LEIRERSIQFSMETEFCEFSPELEDHFEIFEHIRGFNVTIVTSANTQDETLLLWSGFLQK
DEGETQ
>P52901 | ODPA1_ARATH
MALSRLSSRSNIITRPFSAAFSRLISTDTTPITIETSLPFTAHLCDPPSRSVESSSQELL
DFFRTMALMRRMEIAADSLYKAKLIRGFCHLYDGQEAVAIGMEAAITKKDAIITAYRDHC
IFLGRGGSLHEVFSELMGRQAGCSKGKGGSMHFYKKESSFYGGHGIVGAQVPLGCGIAFA
QKYNKEEAVTFALYGDGAANQGQLFEALNISALWDLPAILVCENNHYGMGTAEWRAAKSP
SYYKRGDYVPGLKVDGMDAFAVKQACKFAKQHALEKGPIILEMDTYRYHGHSMSDPGSTY
RTRDEISGVRQERDPIERIKKLVLSHDLATEKELKDMEKEIRKEVDDAIAKAKDCPMPEP
SELFTNVYVKGFGTESFGPDRKEVKASLP
>P54260 | GCST_SOLTU
MRGGLWQLGQSITRRLAQADKKTIGRRCFASDADLKKTVLYDFHVVNGGKMVPFAGWSMP
IQYKDSIMDSTVNCRENGSLFDVSHMCGLSLKGKDTIPFLEKLVIADVAGLAPGTGSLTV
FTNEKGGAIDDSVVTKVTNDHIYLVVNAGCRDKDLAHIEEHMKSFKSKGGDVSWHIHDER
SLLALQGPLAAPVLQYLTKDDLSKMYFGEFRVLDINGAPCFLTRTGYTGEDGFEISVPSE

NALDLAKALLEKSEGKIRLTGLGARDSLRLEAGLCLYGNDMEQHTTPVEAGLTWAIGKRR
RAEGGFLGAEVILKQIEEGPKIRRVGFFSSGPPPRSHSEIQDSNGQNIGEITSGGFSPCL
KKNIAMGYVKTGNHKAGTNVKIVIRGKSYDGVVTKMPFVPTKYYKP
>P60096 | RT12_MAGGA
MPTLNQLIRHGREEKRRTDRTRASDQCPQKQGVRPRVPTRTPKKPNSAPRKIAKVRLSNR
HDIFAHIPGEGHNSQEHPMVLIRGGRVKDSPGVKSHRIRGVKDLLGIPNRRRGRSKYGAE
RPKSI
>P60097 | RT12_MAGSO
MPTLNQLIRHGREEKRRTDRTRASDQCPQKQGVRPRVPTRTPKKPNSAPRKIAKVRLSNR
HDIFAHIPGEGHNSQEHPMVLIRGGRVKDSPGVKSHRIRGVKDLLGIPNRRRGRSKYGAE
RPKSI
>P60098 | RT12_WHEAT
MPTKNQLIRHGREEKRRTDRTRALDQCPQKQGVCLRVSTRTPKKPNSALRKIAKVRLSNR
HDIFAYIPGEGHNLQEHSIVLVRGGRVKDLPGVKFHCIRGVKDLLGIPDRRKGRSKYGAE
RPKSK
>P60099 | RT12_MAIZE
MPTKNQLIRHGREEKRRTDRTRALDQCPQKQGVCLRVSTRTPKKPNSALRKIAKVRLSNR
HDIFAYIPGEGHNLQEHSIVLVRGGRVKDLPGVKFHCIRGVKDLLGIPDRRKGRSKYGAE
RPKSK
>P60159 | NU3M_HELAN
MLEFAPICIYLVISLLVSLILLGVPFLFASNSSTYPEKLSAYECGFDPFGDARSRFDIRF
YLVSILFIIFDLEVTFFFPWAVSLNKIDLFGFWSMMAFLLILTIGFLYEWKRGALDWE
>P60160 | NU3M_WHEAT
MLEFAPICIYLVISLLVSLILLGVPFLFASNSSTYPEKLSAYECGFDPFGDARSRFDIRF
YLVSILFIIFDLEVTFFFPWAVSLNKIDLFGFWSMMAFLLILTIGFLYEWKRGALDWE
>P60621 | COX1_RAPSA
MKNLVRWLFSTNHKDIGTLYFIFGAIAGVMGTCFSVLIRMELARPGDQILGGNHQLYNVL
ITAHAFLMIFFMVMPAMIGGFGNWFVPILIGAPDMAFPRLNNISFWLLPPSLLLLLSSAL
VEVGSGTGWTVYPPLSGITSHSGGAVDLAIFSLHLSGVSSILGSINFITTIFNMRGPGMT
MHRLPLFVWSVLVTAFLLLLSLPVLAGAITMLLTDRNFNTTFFDPAGGGDPILYQHLFWF
FGHPEVYILILPGFGIISHIVSTFSGKPVFGYLGMVYAMISIGVLGFLVWAHHMFTVGLD
VDTRAYFTAATMIIAVPTGIKIFSWIATMWGGSIQYKTPMLFAVGFIFLFTIGGLTGIVL
ANSGLDIALHDTYYVVAHFHYVLSMGAVFALFAGFYYWVGKIFGRTYPETLGQIHFWITF
FGVNLTFFPMHFLGLSGMPRRIPDYPDAYAGWNALSSFGSYISVVGICCFFVVVTITLSS
GNNKRCAPSPWALELNSTTLEWMVQSPPAFHTFGELPAIKETKSYVK
>P62772 | CYC_BRANA
ASFDEAPPGNSKAGEKIFKTKCAQCHTVDKGAGHKQGPNLNGLFGRQSGTTAGYSYSAAN
KNKAVEWEEKTLYDYLLNPKKYIPGTKMVFPGLKKPQDRADLIAYLKEATA
>P62773 | CYC_BRAOL
ASFDEAPPGNSKAGEKIFKTKCAQCHTVDKGAGHKQGPNLNGLFGRQSGTTAGYSYSAAN
KNKAVEWEEKTLYDYLLNPKKYIPGTKMVFPGLKKPQDRADLIAYLKEATA
>P68526 | ATP6_TRITI
MRFLSTDMKDRNMLFAAITTNQPIRSKCSRLPDLHDFFPTNISQNFAITPNLDITPTPER
IAGVTIVLQIEEYLGQNESEQGAVNLARTVLGARHRNGETWQGILEDIRAGGGMDNFIQN

LPGAYPETPLDQFAIIPIIDLHVGNFYLSFTNEVLYMLLTVVLVVFLFFVVTKKGGGKSV
PNAWQSLVELIYDFVLNLVNEQIGGLSGNVKQKFFPRISVTFTFSLFRNPQGMIPFSFTV
TSHFLITLALSFSIFIGITIVGFQRHGLHFFSFLLPAGVPLPLAPFLVLLELISYCFRAL
SLGIRLFANMMAGHSLVKILSGFAWTMLFLNNIFYFIGDLGPLFIVLALTGLELGVAISQ
AHVSTISICIYLNDATNLHQNESFHN
>P68527 | ATP6_WHEAT
MRFLSTDMKDRNMLFAAITTNQPIRSKCSRLPDLHDFFPTNISQNFAITPNLDITPTPER
IAGVTIVLQIEEYLGQNESEQGAVNLARTVLGARHRNGETWQGILEDIRAGGGMDNFIQN
LPGAYPETPLDQFAIIPIIDLHVGNFYLSFTNEVLYMLLTVVLVVFLFFVVTKKGGGKSV
PNAWQSLVELIYDFVLNLVNEQIGGLSGNVKQKFFPRISVTFTFSLFRNPQGMIPFSFTV
TSHFLITLALSFSIFIGITIVGFQRHGLHFFSFLLPAGVPLPLAPFLVLLELISYCFRAL
SLGIRLFANMMAGHSLVKILSGFAWTMLFLNNIFYFIGDLGPLFIVLALTGLELGVAISQ
AHVSTISICIYLNDATNLHQNESFHN
>P68535 | RT12_PETHY
MPSLNQLIRHGREEKRRTDRTRALDQCPQKQGVCPRVSTRTPKKPNSAPRKIAKVRLSNR
HDIFAHIPGEGHNLQEHSMVLIRGGRVKDSPGVKSHCIRGVKDLLGIPDRRRGRSKYGAE
KPKSI
>P68536 | RT12_PETPA
MPSLNQLIRHGREEKRRTDRTRALDQCPQKQGVCPRVSTRTPKKPNSAPRKIAKVRLSNR
HDIFAHIPGEGHNLQEHSMVLIRGGRVKDSPGVKSHCIRGVKDLLGIPDRRRGRSKYGAE
KPKSI
>P68539 | COX1_WHEAT
MTNMVRWLFSTNHKDIGTLYFIFGAIAGVMGTCFSVLIRMELARPGDQILGGNHQLYNVL
ITAHAFLMIFFMVMPAMIGGFGNWFVPILIGAPDMAFPRLNNISFWLLPPSLLLLLSSAL
VEVGSGTGWTVYPPLSGITSHSGGAVDLAIFSLHLSGISSILGSINFITTIFNMRGPGMT
MHRLPLFVWSVLVTAFLLLLSLPVLAGAITMLLTDRNFNTTFFDPAGGGDPILYQHLFWF
FGHPEVYILILPGFGIISHIVSTFSRKPVFGYLGMVYAMISIGVLGFLVWAHHMFTVGLD
VDTRAYFTAATMIIAVPTGIKIFSWIATMWGGSIQYKTPMLFAVGFIFLFTIGGLTGIVL
ANSGLDIALHDTYYVVAHFHYVLSMGAVFALFAGFYYWVGKIFGRTYPETLGQIHFWITF
FGVNLTFFPMHFLGLSGMPRRIPDYPDAYAGWNALSSFGSYISVVGIRRFFVVVAITSSS
GKNQKCAESPWAVEQNPTTLEWLVQSPPAFHTFGELPAVKETKS
>P68540 | COX1_AEGCO
MTNMVRWLFSTNHKDIGTLYFIFGAIAGVMGTCFSVLIRMELARPGDQILGGNHQLYNVL
ITAHAFLMIFFMVMPAMIGGFGNWFVPILIGAPDMAFPRLNNISFWLLPPSLLLLLSSAL
VEVGSGTGWTVYPPLSGITSHSGGAVDLAIFSLHLSGISSILGSINFITTIFNMRGPGMT
MHRLPLFVWSVLVTAFLLLLSLPVLAGAITMLLTDRNFNTTFFDPAGGGDPILYQHLFWF
FGHPEVYILILPGFGIISHIVSTFSRKPVFGYLGMVYAMISIGVLGFLVWAHHMFTVGLD
VDTRAYFTAATMIIAVPTGIKIFSWIATMWGGSIQYKTPMLFAVGFIFLFTIGGLTGIVL
ANSGLDIALHDTYYVVAHFHYVLSMGAVFALFAGFYYWVGKIFGRTYPETLGQIHFWITF
FGVNLTFFPMHFLGLSGMPRRIPDYPDAYAGWNALSSFGSYISVVGIRRFFVVVAITSSS
GKNQKCAESPWAVEQNPTTLEWLVQSPPAFHTFGELPAVKETKS
>P68541 | ATPAM_RAPSA
MELSPRAAELTNLFESRIRNFYANFQVDEIGRVVSVGDGIAQVYGLNEIQAGEMVLFANG
VKGMALNLENENVGIVVFGGDTAIKEGDLVKRTGSIVDVPAGKAMLGRVVDAMGVPIDGR

GALSDHEQRRVEVKAPGILERKSVHEPMQTGLKAVDSLVPIGRGQRELLIGDRQTGKTTI
AIDTILNQKQINSRATSESETMYCVYVAIGQKRSTVGQLIQTLEEANALEYSILVAATAS
DPAPLQFLAPYSGCAMGEYFRDNGMHALIIYDDLSKQAVAYRQMSLLLRRPPGREAFPGD
VFYLHSRLLERAAKRSDQTGAGSLTALPVIETQAGDVSAYIPTNVISITDGQICLETELF
YRGIRPAINVGLSVSRVGSAAQLKAMKQVCGSSKLELAQYREVAAFAQFGSDLDAATQAL
LNRGARLTEVPKQPQYAPLPIEKQILVIYAAVNGFCDRMPLDRISQYEKAIPNSVKPELL
QALKGGLTNERKMEPDAFLKERALALI
>P68542 | ATPAM_BRACM
MELSPRAAELTNLFESRIRNFYANFQVDEIGRVVSVGDGIAQVYGLNEIQAGEMVLFANG
VKGMALNLENENVGIVVFGGDTAIKEGDLVKRTGSIVDVPAGKAMLGRVVDAMGVPIDGR
GALSDHEQRRVEVKAPGILERKSVHEPMQTGLKAVDSLVPIGRGQRELLIGDRQTGKTTI
AIDTILNQKQINSRATSESETMYCVYVAIGQKRSTVGQLIQTLEEANALEYSILVAATAS
DPAPLQFLAPYSGCAMGEYFRDNGMHALIIYDDLSKQAVAYRQMSLLLRRPPGREAFPGD
VFYLHSRLLERAAKRSDQTGAGSLTALPVIETQAGDVSAYIPTNVISITDGQICLETELF
YRGIRPAINVGLSVSRVGSAAQLKAMKQVCGSSKLELAQYREVAAFAQFGSDLDAATQAL
LNRGARLTEVPKQPQYAPLPIEKQILVIYAAVNGFCDRMPLDRISQYEKAIPNSVKPELL
QALKGGLTNERKMEPDAFLKERALALI
>P80261 | NDUS3_SOLTU
MDNQFIFKYSWETLPKKWVKKMERSEHGNRFDTNTDYLFQLLCFMKLHTYTRVQVLIDIC
GVDYPSRKQRFEVVYNLLSIRYNSRIRVQTSADEVTRISSVVSLFPSAGWWEREVWDMFG
VFSINHPDLRRILTDYGFEGHPLRKDFPLSGYVEVRYDDPEKRVVSEPIEMTQEFRYFDF
ASPWEQRSDG
>P83372 | CISY_FRAAN
MAFFRTVTKLRSRLGQPPSLRDSVRCLQTQASSDLDLHSQLKELIPEQQERLKKLKKEHG
KVQLGTITVDMVIGGMRGMTGLLWETSLLDPDEGIRFRGLSIPECQKVLPGATPGGEPLP
EGLLWLLLTGKVPSRSKNMHYPVNNGVVPKFQIMCSRPLMLCLLEHIPMTQFTTGVMALQ
VQSEFQKAYDKGIPKSRYWEPTYEDSLSLIAQLPVVASYVYRRIYKGGRMIPVDDSLDYG
GNFSHLLGFDDHKMQELMRLYVTIHSDHEGGNVSAHTGHLVASALSDPFLSFAAALNGLA
GPLHGLANQEVLLWIKSVVDECGENITKDQLKDYVWKTLNSGKVVPGFGHGVLRKTDPRY
TCQREFALKHLPDDPLFRLVSKLYDVVPPILTELGKVKNPWPNVDAHSGVLLNHFGLTEA
RYFTVLFGVSRSIGIGSQLIWDRALGLPLERPKSVTMESLESFCKKAAS
>P83373 | MDHM_FRAAN
MRPSMSLIRSVSRVARRGYSSESVPQRKVAVLGAAGGIGQPLALLMKLNPLVSQLSLYDI
AGTPGVAADVSHINTRSEVKGYAGEEQLGEALEGCDVVIIPAGVPRKPGMTRDDLFNINA
GIVRSLTAAIAKYCPHAIINMISNPVNSTVPIASEVLKKAGVYDEKKLFGVTTLDVVRAK
TFYAGKAGVPVAEVNVPVVGGHAGITILPLFSQATPKANLSDDYIKALTKRTQDGGTEVV
EAKAGKGSATLSMAYAGALFADACLXGLNGVPDVVECSYVQSSITELPFFASKVRLGKNG
VEEVLDLGPLSDFEKEGLKQLKPELKSSIEKGIKFANQS
>P83483 | ATPBM_ARATH
MASRRVLSSLLRSSSGRSAAKLGNRNPRLPSPSPARHAAPCSYLLGRVAEYATSSPASSA
APSSAPAKDEGKKTYDYGGKGAIGRVCQVIGAIVDVRFEDQEGLPPIMTSLEVQDHPTRL
VLEVSHHLGQNVVRTIAMDGTEGLVRGRKVLNTGAPITVPVGRATLGRIMNVLGEPIDER
GEIKTEHYLPIHRDAPALVDLATGQEILATGIKVVDLLAPYQRGGKIGLFGGAGVGKTVL
IMELINNVAKAHGGFSVFAGVGERTREGNDLYREMIESGVIKLGEKQSESKCALVYGQMN

EPPGARARVGLTGLTVAEYFRDAEGQDVLLFIDNIFRFTQANSEVSALLGRIPSAVGYQP
TLASDLGALQERITTTKKGSITSVQAIYVPADDLTDPAPATTFAHLDATTVLSRQISELG
IYPAVDPLDSTSRMLSPHILGEEHYNTARGVQKVLQNYKNLQDIIAILGMDELSEDDKLT
VARARKIQRFLSQPFHVAEIFTGAPGKYVDLKENINSFQGLLDGKYDDLSEQSFYMVGGI
DEVVAKAEKIAKESAA
>P83484 | ATPBN_ARATH
MASRRVLSSLLRSSSGRSAAKLVNRNPRLPSPSPARHAAPCSYLLGRVAEYATSSPASSA
APSSAPAKDEGKKTYDYGGKGAIGRVCQVIGAIVDVRFEDQEGLPPIMTSLEVQDHPTRL
VLEVSHHLGQNVVRTIAMDGTEGLVRGRKVLNTGAPITVPVGRATLGRIMNVLGEPIDER
GEIKTEHYLPIHRDAPALVDLATGQEILATGIKVVDLLAPYQRGGKIGLFGGAGVGKTVL
IMELINNVAKAHGGFSVFAGVGERTREGNDLYREMIESGVIKLGEKQSESKCALVYGQMN
EPPGARARVGLTGLTVAEYFRDAEGQDVLLFIDNIFRFTQANSEVSALLGRIPSAVGYQP
TLASDLGALQERITTTKKGSITSVQAIYVPADDLTDPAPATTFAHLDATTVLSRQISELG
IYPAVDPLDSTSRMLSPHILGEEHYNTARGVQKVLQNYKNLQDIIAILGMDELSEDDKLT
VARARKIQRFLSQPFHVAEIFTGAPGKYVDLKENINSFQGLLDGKYDDLSEQSFYMVGGI
DEVVAKAEKIAKESAA
>P92532 | RT12_ARATH
MPTFNQLIRHGREEKRRTDRTRALDKCPQKTGVCLRVSTRTPKKPNSALRKIAKVRLSNR
HDIFAYIPGEGHNLQEHSTVLIRGGRVKDLPGVKFHCIRGVKDLMGIPGRRRGRSKYGAE
KPKSI
>P92547 | ATP62_ARATH
MERLTRLNHFLVNMRWDFYEGVIQAGYIRNLQRELDHTPAELLGSKLDLIFFRESLNLST
YVNNWYMQNLGVPGPVNFIEKYHDACFSNYMKLMEIPSPLDQFEIVPLIPMHIGNFYFSF
TNSSLFMLLTLSFFLLLIHFVTKKGGGNLVPNAWQSLVELLYDFVLNLVKEQIGGLSGNV
KQMFFPCILVTFLFLLFCNLQGMIPYSFTVTSHFLITLALSFSIFIGITIVGFQRHGLHF
FSFLLPAGVPLPLAPFLVLLELISYCFRALSLGIRLFANMMAGHSLVKILSGFAWTMLCM
NDIFYFIGALGPLFIVLALTGLELGVAILQAYVFTILICIYLNDAINLH
>P92549 | ATPAM_ARATH
MELSPRAAELTNLFESRIRNFYANFQVDEIGRVVSVGDGIAQVYGLNEIQAGEMVLFANG
VKGMALNLENENVGIVVFGGDTAIKEGDLVKRTGSIVDVPAGKAMLGRVVDAMGVPIDGK
GALSDHEQRRVEVKAPGILERKSVHEPMQTGLKAVDSLVPIGRGQRELLIGGRQTGKTTI
AIDTILNQKQINSRATSESETMYCVYVAIGQKRSTVGQLIQTLEEANALEYSILVAATAS
DPAPLQFLAPYSGCAMGEYFRDNGMHALIIYDDLSKQAVAYRQMSLLLRRPPGREAFPGD
VFYLHSRLLERAAKRSDQTGAGSLTALPVIETQAGDVSAYIPTNVISITDGQICLETELF
YRGIRPAINVGLSVSRVGSAAQLKAMKQVCGSLKLELAQYREVAAFAQFGSDLDAATQAL
LNRGARLTEVLKQPQYAPLPIEKQILVIYAAVNGFCDRMPLDRISQYEKAILNSVKPELL
QALKGGLTNERKMELDAFLKERALALI
>P92557 | RT07_ARATH
MGGLDGEQKLLIKKLVNFRMKEGKRTRVRAIVYQTFHRPARTERDVIKLMVDAVENIKPI
CEVAKVGVAGTIYDVPGIVARDRQQTLAIRWILEAAFKRRISYRISLEKCSFAEILDAYQ
KRGSARRKRENLHGLASTNRSFAHFRWW
>P92969 | RPOT1_ARATH
MWRNILGRASLRKVKFLSDSSSSGTHYPVNRVRGILSSVNLSGVRNGLSINPVNEMGGLS
SFRHGQCYVFEGYATAAQAIDSTDPEDESSGSDEVNELITEMEKETERIRKKARLAAIPP

KRVIAGMGAQKFYMLKQRQVKMETEEWERAARECREILADMCEQKLAPNLPYMKSLFLGW
FEPVRNAIQDDLDTFKIKKGKIPYAPFMEQLPADKMAVITMHKMMGLLMTNAEGVGIVKL
VNAATQIGEAVEQEVRINSFLQKKNKKNATDKTINTEAENVSEEIVAKETEKARKQVTVL
MEKNKLRQVKALVRKHDSFKPWGQEAQVKVGARLIQLLMENAYIQPPAEQFDDGPPDIRP
AFKQNFRTVTLENTKTSRRYGCIECDPLVLKGLDKSARHMVIPYLPMLIPPQNWTGYDQG
AHFFLPSYVMRTHGAKQQRTVMKRTPKEQLEPVYEALDTLGNTKWKINKKVLSLVDRIWA
NGGRIGGLVDREDVPIPEEPEREDQEKFKNWRWESKKAIKQNNERHSQRCDIELKLEVAR
KMKDEEGFYYPHNVDFRGRAYPIHPYLNHLGSDLCRGILEFCEGKPLGKSGLRWLKIHIA
NLYAGGVDKLAYEDRIAFTESHLEDIFDSSDRPLEGKRWWLNAEDPFQCLAACINLSEAL
RSPFPEAAISHIPIHQDGSCNGLQHYAALGRDKLGADAVNLVTGEKPADVYTEIAARVLK
IMQQDAEEDPETFPNATYAKLMLDQVDRKLVKQTVMTSVYGVTYSGARDQIKKRLKERGT
FEDDSLTFHASCYAAKITLKALEEMFEAARAIKSWFGDCAKIIASENNAVCWTTPLGLPV
VQPYRKPGRHLVKTTLQVLTLSRETDKVMARRQMTAFAPNFIHSLDGSHMMMTAVACNRA
GLSFAGVHDSFWTHACDVDVMNTILREKFVELYEKPILENLLESFQKSFPDISFPPLPER
GDFDLRKVLESTYFFN
>P93032 | IDH2_ARATH
MSRQSFSLLKNLRSIASGSKIQTRSVTYMPRPGDGKPRPVTLIPGDGVGPLVTNAVQQVM
EAMHAPVYFEPFEVHGDMKSLPEGLLESIKKNKVCLKGGLKTPVGGGVSSLNVNLRKELD
LFASLVNCFNLPGLASRHENVDIVVIRENTEGEYAGLEHEVVPGVVESLKVITKFCSERI
AKYAFEYAYLNNRKKVTAVHKANIMKLADGLFLESCQEVAKKYPSIAYNEIIVDNCCMQL
VARPEQFDVMVTPNLYGNLVANTAAGIAGGTGVMPGGNVGAEYAVFEQGASAGNVGKDTT
EEQKNANPVALLLSSAMMLRHLQFPSFADRLETAVKRVIAEGNCRTEDLGGNSTTQEVVD
AVIANLD
>P93285 | COX2_ARATH
MIVLKWLFFTISPCDAAEPWQLGFQDAATPIMQGIIDLHHDIFFFLILILVFVLWILVRA
LWHFHYKKNAIPQRIVHGTTIEILWTIFPSIILMFIAIPSFALLYSMDEVVVDPAITIKA
IGHQWYWTYEYSDYNSSDEQSLTFDSYMIPEEDLELGQLRLLEVDNRVVVPAKTHLRIIV
TSADVLHSWAVPSLGVKCDAVPGRLNQISILVQREGVYYGQCSEICGTNHAFMSIVVEAV
SRKDYGSWVSNQLIPQTGEA
>P93298 | ATP61_ARATH
MRRIFLFDENSLNSSSTIDTSSASTIDTSFASQCTNFSSGQASGTQDTHAGIFEDCPGLN
PNDERVVELQCEIREKCEALTQDPEMGLILGEALHAESDNVPFLQSIADDLTQNGVSGEA
FQEALNIVGQAAASPLDQFEIVPLIPMHIGNFYFSFTNSSLFMLLTLSFFLLLIHFVTKK
GGGNLVPNAWQSLVELLYDFVLNLVKEQIGGLSGNVKQMFFPCILVTFLFLLFCNLQGMI
PYSFTVTSHFLITLALSFSIFIGITIVGFQRHGLHFFSFLLPAGVPLPLAPFLVLLELIS
YCFRALSLGIRLFANMMAGHSLVKILSGFAWTMLCMNDIFYFIGALGPLFIVLALTGLEL
GVAILQAYVFTILICIYLNDAINLH
>P93306 | NDUS2_ARATH
MTTRKRQIKNFTLNFGPQHPAAHGVLRLVLEMNGEVVERAEPHIGLLHRGTEKLIEYKTY
LQALPYFDRSDYVSMMAQEHAYSLAVEKLLNCEVPLRAQYIRVLFCEITRILNHLLALTT
HAMDVGALTPFLWAFEEREKLLEFYERVSGARMHASFIRPGGVAQDLPLGLCRDIDSFTQ
QFASRIDELEEMLTGNRIWKQRLVDIGTVTAQQAKDWGFSGVMLRGPGVCWDLRRAAPYD
VYDQLDFDVPVGTRGDCYDRYCIRIEEMRQSLRIIVQCLNQMPSGMIKADDRKLCPPSRC
RMKLSMESSIHHFELYTEGFSVPASSTYTAVEAPKGEFGVFLVSNGSNRPYRCKIRAPGF

AHLQGLDFMSKHHMLADVVTIIGTQDIVFGEVDR
>P93311 | RM02_ARATH
MRPGRARALRQFTLSTGKSAGRNSSGRITVFHRGGGSKRLLRRIDLKRSTSSMGIVESIE
YDPNRSSQIAPVRWIKGGCQKKMNTIEKFAPPRKILEPTTNTISGLFSFSFLPGKVDKRK
VACFSPGLMAAYVVVGLPTGMPPLSSSKSAFASKGAGSTKTLVKDVFFSAFSSPKAKRET
ASLAFASSFGFPRIAVAGAKPAFFAPRMRQKVRGKSTFSLCEVQKGRTHSILWAHRIKGK
AGLSWQSFRRQDTLGLVGAAGHKKSKPKTDQGNLPAKPIGERAKQLKALRGLRAKDGACK
VDRAPVTYIIASHQLEAGKMVMNCDWSKPSTSSFLQSAQNDHPKPLFTV
>P93313 | NU4M_ARATH
MLEHFCECYFNLSGLILCPVLGSIILLFIPNSRIRLIRLIGLCASLITFLYSLVLWIQFD
SSTAKFQFVESLRWLPYENINFYLGIDGISLFFVILTTFLIPICILVGWSGMRSYGKEYI
IAFLICEFLMIAVFCMLDLLLFYVFPESVLIPMFIIIGVWGSRQRKIKAAYQFFLYTLLG
SLFMLLAILLILFQTGTTDLQILLTTEFSERRQIFLWIAFFASFAVKVPMVPVHIWLPEA
HVEAPTAGSVILAGILLKFGTYGFLRFSIPMFPEATLCFTPFIYTLSAIAIIYTSLTTLR
QIDLKKIIAYSSVAHMNLVTIGMFSPNIQGIGGSILLMLSHGLVSSALFLCVGVLYDRHK
TRLVRYYGGLVSTMPNFSTIFFSFTLANMSLPGTSSFIGEFLILVGAFQRNSLVATLAAL
GMILGAAYSLWLYNRVVSGNLKPDFLHKFSDLNGREVFIFIPFLVGLVWMGVYPKVFLDC
MHTSVSNLVQHGKFH
>P93401 | NU2M_OENBE
MFNLFLAVFPEIFIINATFILLIHGVVFSTSKKDDYPPLVSNVGWLGLLSVLITLLLLAA
GAPLLTIAHLFWNNFFRRDNFTYFCQILLLLSTAGTISMCFDFFEQERFDAFEFIVLILL
STCSMLFMISAYDLIAMYLAIELQSLCFYVLAASKRKSEFSTEAGLKYLILGAFSSGILL
FGCSMIYGSTGATHFDQLAKILTGYEITGARSSGIFMGILFIAVGFLFKITAVPFHMWAP
DIYEGSPTPVTAFLSIAPKISIFANILRVFIYGSYGATLQQIFFFCSIASMILGALAAMA
QTKVKRLLAYSSIGHVGYICIGFSCGTIEGIQSLLIGLFIYALTTINAFAIVLALRQTRV
KYIADLGALAKTNPILAITFSITMFSYAGIPPLAGFCSKFYLFFAALGCGAYFLASVGVV
TSVIGCFYYIRLVKRMFFDTPRTWILYEPMDRNKSLLLAMTSSFITLFFLYPSPLFSVTH
QMALSLYL
>P98012 | COX2_BETVU
MIVREWLFFTMAPCDAAEPWQLGFQDAATPMMQGIIDLHHDIFFFLILILVFVSWILVRA
LWHFHYKKNPIPQRIVHGTTIEIIRTIFPSIILMFIAIPSFALLYSMDEVVVDPAITIKA
IGHQWYRSYEYSDYNSSDEQSLTFDSYTIPEDDPELGQSRLLEVDNRVVVPAKTHIRIIV
TSADVLHSWAVPSSGVKCDAVPGRLNQTSILVQREGVYYGQCSEICGTNHAFMPIVVEAV
SRKDYGSRVSNQLIPQTGEA
>Q00583 | HMDH3_HEVBR
MDEVRRRPPKHIVRKDHDGEVLNSFSHGHHLPPLKPSDYSLPLSLYLANALVFSLFFSVA
YFLLHRWREKIRKSTPLHIVTFPEIAALICLVASVIYLLGFFGIGFVHSFSRASTDSWDV
EEYDDDNIIIKEDTRPTGACAAPSLDCSLSLPTKIHAPIVSTTTTSTLSDDDEQIIKSVV
SGSIPSYSLESKLGNCKRAALIRRETLQRMSGRSLEGLPLDGFDYESILGQCCEMAIGYV
QIPVGIAGPLLLDGKEYTVPMATTEGCLVASANRGCKAIYASGGATSVLLRDGMTRAPVV
RFPTAKRAADLKFFMEDPDNFDTIAVVFNKSSRFARLQSVQCAIAGKNLYMRFSCSTGDA
MGMNMVSKAVQNVIDYLQNDFPDMDVIGLTGNFCADKKAAAVNWIEGRGKSVVCEAIIKE
EVVKKVLKTNVAALVELNMIKNLTGSAVAGSLGGFNAHASNMVTAVYIATGQDPAQNVES
SHCITMMEAVNDGKDLHISVSMPSIELGTVGGGTQLASQSACLNLLGVKGASKDSPGSNS

RLLATIVAGSVLAGELSLMSAIAAGQLVNSHMKYNRSAKDVSKITF
>Q01859 | ATPBM_ORYSJ
MATRRALSSLVRAASRLRGASPAPRPRGPLHRPSPSGYLFNRAAAYATAAAAKEAAPPAP
ATGKATGGGKITDEFTGAGAVGQVCQVIGAVVDVRFDEGLPPILTALEVLDHNIRLVLEV
AQHLGENMVRTIAMDGTEGLVRGQRVLNTGSPITVPVGRATLGRIMNVIGEPIDEKGDIT
TNHFLPIHREAPAFVEQATEQQILVTGIKVVDLLAPYQRGGKIGLFGGAGVGKTVLIMEL
INNVAKAHGGFSVFAGVGERTREGNDLYREMIESGVIKLGDKQSESKCALVYGQMNEPPG
ARARVGLTGLTVAEHFRDAEGQDVLLFIDNIFRFTQANSEVSALLGRIPSAVGYQPTLAT
DLGGLQERITTTKKGSITSVQAIYVPADDLTDPAPATTFAHLDATTVLSRQISELGIYPA
VDPLDSTSRMLSPHVLGEDHYNTARGVQKVLQNYKNLQDIIAILGMDELSEDDKLTVARA
RKIQRFLSQPFHVAEVFTGAPGKYVELKESVNSFQGVLDGKYDDLPEQSFYMVGGIEEVI
AKAEKIAKESAS
>Q01902 | RT07_WHEAT
MGDFDGEQKELIKKLVNFRMIDGKRTRVRAIVYKTFHRLARTERDVIKLMVDAVDNIKPI
CEVVKVGVAGTIYDVPGIVARDRQQTLAIRWILGAAFKRRISYRISLEKCSFAEILDAYR
KRGISRKRRENLHGLASTNRSFAHFRWW
>Q01915 | ATPAM_SOYBN
MEFSVRAAELTTLLESRITNFYTNFQVDEIGRVVSVGDGIARVYGLNEIQAGEMVEFASG
VKGIALNLENENVGIVVFGSDTAIKEGDLVKRTGSIVDVPAGKAMLGRVVDALGVPIDGR
GALSDHERRRVEVKAPGIIERKSVHEPMQTGLKAVDSLVPIGRGQRELIIGDRQTGKTAI
AIDTILNQKQMNSRATSESETLYCVYVAIGQKRSTVAQLVQILSEANALEYSILVAATAS
DPAPLQFLAPYSGCAMGEYFRDNGMHALIIYDDLSKQAVAYRQMSLLLRRPPGREAFPGD
VFYLHSRLLERAAKRSDQTGAGSLTALPVIETQAGDVSAYIPTNVISITDGQICLETELF
YRGIRPAINVGLSVSRVGSAAQLKAMKQVCGSLKLELAQYREVAAFAQFGSDLDAATQAL
LNRGARLTEVLKQPQYAPLPIEKQILVIYAAVNGFCDRMPLDKIPQYERDILTTIKPELL
QSLKGGLTSERKIELEKFLKEKGGTYYI
>Q04050 | NU4M_BRACM
MLEHFCECYSNLSGLILCPVLGSITPLFIPNSRIRPIRLIGLCASLITFLYSPVPRIQFD
SSTAKSQFVESLRWLPYENINFYLGIDGISLFFVILTTFLIPICISVGWSGMRSYGKEYI
TAFLIREFLMIAVFRMLDLLLFYVFPESVPIPMFIIIGVWGSRQRKIKAAYQFFLYTLLG
SLFMLLAILLILFQTGTTDLQISLTTEFSERRQIFLWIASFASFAVKVPMVPVHIWLPEA
HVEAPTAGSVILAGIPLKFGTHGFLRFSIPMFPEATLCSTPFIYTLSAIAIIYTSLTTSR
QIDLKKIIAYSSVAHMNLVTIGMFSPNIQGIGGSILPMLSHGLVPSALFLCVGVLYDRHK
TRLVRYYGGLVSTMPNLSTIFFSFTLANMSSPGTSSFIGEFPILVGAFQRNSLVATLAAL
GMILGAAYSLWLYNRVVSGNLKPDFLHKFSDSNGREVSIFIPFLVGLVRMGVHPKVFPDC
MHTSVSNLVQHGKFH
>Q04654 | ATP6_VICFA
MNLYLQLDLYLYNDNLYLYLYLESGVVPIPSPLEQFEIIPFLPMKIGDLYFSFTNPSLFM
LLTLSLVLLLVHFVTKKGGGKSVPNAWQSLVELIYDFVPNLVNEQIGGLSGNVKQQFFPC
IFVTFTFLLFCNLQGMIPYSFTVTSHFLITLGLSFSIFIGITIVGFQRNGLHFLSFLLPA
GVPLPLAPFLVLLELISYCFRALSLGIRLFANMMAGHSLVKILSGFAWTMLCMNDLLYFI
GDLGPLFIVLALTGPELGVAISQAHVSTISICIYLNDATNLHQTCLLFIYN
>Q04715 | RM16_PETHY
MLLRKYLLVTESQVSKCGFLIVKKKRDVLYPKRTKYSKYRKGRCSRGCKPDGTQLGFGRY

GTKSCRAGRLSYRAIEAARRAIIGHFHRAMSGQFRRNGKIWVRVLADIPITGKPTEVRMG
RGKGNPTGWIARVSRGQILFEMDGVSLSNARQALH

>Q05143 | COX1_PROWI
MVTRWLYSTNHKDIGTMYLIFGAFSGVLGTVFSLLIRMELAQPGNQILNGNHQLYNVIIT
AHAFLMIFFMLMPALMGGFGNWFLPILIGAPDMAFPRLNNISFWLLPPSLLLLVSSALVE
VGAGTGWTVYPPLASIASHSGGSVDLAIFSLHLAGVSSILGAINFICTVFNMRAPGMSML
DLLFVWAVFITAWLLLLCLPVLAGGITMLLTDRNFNTSFFDPAGGGDPILYQHLFWFFGH
PEVYILIIPGFGIISHVIATFSKKPIFGYLGMVYAMCSIGILGFIVWAHHMYVVGLDIDT
RAYFTAATMIIAVPTGIKIFSWVATMWGGSIELRTPMLFAVGFLFLFTVGGLTGVVLANS
GLDVAFHDTYYVVAHFHYVLSMGAVFALFSGFYYWIGKITGLQYPETLGQIHFWLMFLGV
NITFFPMHFLGLAGMPRRIPDYPDCYAGWNAVASYGSYLSITAVLFFFYVVYKTLTSNEV
CPRNPWETTPGVSPTLEWMLPSPPAFHTFEEIQV
>Q06735 | ATPAM_BETVU
MEFSPRAAELTNLLESRITNFYTNFQVDEIGRVVSVGDGIARVYGLNEIQAGEMVEFASG
VKGIALNLENENVGIVVFGSDTAIKEGDLVKRTGSIVDVPAGKAMLGRVVDALGVPIDGR
GALSDHERRRVEVKAPGIIERKSVHEPMQTGLKAVDSLVPIGRGQRELIIGDRQTGKTAI
AIDTILNQKQLNSKATSESETLYCVYVAVGQKRSTVAQLVQILSEANALEYSILVAATAS
DPAPLQFLAPYSGCAMGEYFRDNGMHALIIYDDLSKQAVAYRQMSLLLRRPPGREAFPGD
VFYLHSRLLERAAKRSDQTGAGSLTALPVIETQAGDVSAYIPTNVISITDGQICLETELF
YRGIRPAINVGLSVSRVGSAAQLKAMKQVCGSPKLELAQYREVAAFAQFGSDLDAATQAL
LNRGARLTEVPKQPQYAPLPIEKQILVIYAAVNGFCDRMPLDKISQYERTIPNSVKPELL
QSLKGGLTNEKKMELDSFLKECALNY
>Q0DI31 | CYC_ORYSJ
MASFSEAPPGNPKAGEKIFKTKCAQCHTVDKGAGHKQGPNLNGLFGRQSGTTPGYSYSTA
NKNMAVIWEENTLYDYLLNPKKYIPGTKMVFPGLKKPQERADLISYLKEATS
>Q31708 | RT04_ARATH
MWLLKKLIQRDIDLSPLRFQTCRLLSGNVWNRELTIIQRRILRRLRNRKRSIKKRKIYPK
KYLTSYIQLQTTRKLPFFYGDLPITEMHRGTKRTSYIPFLLNLETRFDVIPLRLYFLETI
PQARQLISHRRVCVNKGMVSITHFKLSHGDIISFQENNAIIRGEEIRRSFYKEILVEKII
GKLLHQPLRMWRRSKTEWFHLLKTKRGCRLLLKSRFLQQLRSSMQEEDLERTKKFGSEKV
CLGSSFAEHKRMKRNLLKSLFLSKRRKDKNLNLPTRTISPIVYNSSLSLYSNSTYCFASP
HKLTMKRRIKRIELPTHYLEVNYRTPKAVVFYGPNIGHIPHDIRLKDLNLLLWSRNGRGQ
NI
>Q31720 | ATP6_BRANA
MQIGLVAQSPLDQFEIVPLIPMNIGNFYFSFTNSSLFMLLTLSFFLLLIHFITKKGGGNL
VPNAWQSLVELLYDFVLNLVKEQIGGLSGNVKQMFFPCILVTFLFLLFCNLQGMIPYSFT
VTSHFLITLALSFSIFIGITIVGFQRHGLHFFSFLLPAGVPLPLAPFLVLLELISYCFRA
LSLGIRLFANMMAGHSLVKILSGFAWTMLCMNEIFYFIGALGPLFIVLALTGLELGVAIL
QAYVFTILICIYLNDAINLH
>Q33994 | NDUS3_BETTR
MDNQFIFKYSWETLPKKWVKKIEKSEHGNRFDTNTDYLFQLLCFLKLHTYTRFQVLIDIC
GVDYPSRKRRFEVVYNLLSTRYNSRIRLQTSADEVTRISSVVSLFPSAGWWEREVWDMFG
VSFINHPDLRRILTDYGFEGHPLRKDFPLSGYVEVRYDDPEKRVVSEPIEMTQEFRYFDF

ASPWEQRNGNEG
>Q34011 | NDUS3_BETWE
MDNQFIFKYSWETLPKKWVKKIEKSEHGNRFDTNTDYLFQLLCFLKLHTYTRFQVLIDIC
GVDYPSRKRRFEVVYNLLSTRYNSRIRLQTSADEVTRISSVVRLFPSAGWWEREVWDMFG
VSFINHPDLRRILTDYGFEGHPLRKDFPLSGYVEVRYDDPEKRVVSEPIEMTQEFRYFDF
ASPWEQRNGNEG
>Q35322 | NDUS3_ORYSJ
MDNQFIFQYSWEILPKKWVHKMKRSEHGNRFYTNTDYLFPLLCFLKWHTYTRVQVLIDIC
GVDYPSRKRRFEVVYNLLSTRYNSRIRVQTSADEVTRISSVVSLFPSAGWWEREVWDMFG
VSFINHPDLRRILTDYGFEGHPLRKDFPLSGYVEVRYDDPEKRVVSEPIEMTQEFRYFDF
ASPWEQRSDG
>Q36450 | NDUS2_NICSY
MTTKNRQIKNFTSNFGPQHPAAHGVSRSVLEMNGEVVERAEPHIGLLQRGTEKLIEYKTY
LQALPYSDRSEYVSMMAQEHAHSSAVERLLNCEVPLRAQYIRVLFREITRISNHSLALTT
HAMDVGASTPFLWAFEEREKLLEFYERVSGARMHASFIRPGGVAQDLPLGLCIDIDSFTQ
QFASRIDELEEMSTGNRIWKQRLVDIGTVTAQQAKDWGFSGVMLRGSGVCWDLRKAAPYD
VHDQLDPDIPVGTRGDRYDRYCIRIEEMRQSVRIIVQCLNQMPSGMIKADDRKLCPPSRS
RMKLSMESSIHHFEPYTEGFSVPAPSTYTAVEAPKGEFGVFLVSNGSNRPYRRKIRAPCF
AHSQGLDSMSKHHMPADVVTIIGTQDIVSGEVDR
>Q36518 | NU3M_PLASU
MIEYLAVLIYFLFSLALASLIIFLSFIFAPQKPDPEKISAYECGFDPFDDARGKFDIRFY
LVAILFIIFDLEVTFLFPWAVTLGKIGFFGFWTMMAFLIIILTIGFIYEWKKGALEWE
>Q36664 | NU3M_PINSY
MSEFAPICIYLVISLLVCLIPLGVPFLFASNGSTYPEKLSAYECGFDPFGDARSRFDIRF
YLVSILFIIFDLEVTFFFPWAVSLNKIDLFGFWSMMVFLLILTIGFLYEWKKGALDWE
>Q36665 | RT12_PINSY
MPTSNQSIRHGREKKRRTDRTRALEKCPQKRGVCLRVSTRTPKKPNSALRKIAKVRLSNR
HDIFAYIPGEGHNLQEHSMVLIRGGRVKDLPGVKFHRIRGVKDLLGIPGRKRGRSKYGAE
RPKSK
>Q37625 | NU3M_PROWI
MYEFLGILIYFFIALALSLLLLGLPFLVSTRKADPEKISAYECGFDPFDDARGRFDIQFY
LVAILFIIFDLEVAFLFPWALTLNKIGYFGFWSMMLFLFILTVGFIYEWRKGALDWS
>Q37626 | NU6M_PROWI
MDFLFYIFSSLTLISGSLVIQARNPVHSVLFLVLVFFNAAGLLVLLGLDFFALIFLVVYV
GAIAVLFLFVVMMLNIRITEISEKRLRYLPVGGVLGVLFLFEICILIDNDCIPLLSYDIE
NTALLANYNQLSFIDWRMYLSTSHTIDALGSLLYTYYFYFFLVASLILLVAMIGAIVLTM
QKGIRIKRQQVFLQNTRDFAKTIRKVA
>Q37627 | NU4LM_PROWI
MDLSKYLTVSMILFLLGIWGIFLNRKNIIVMLMSIELMLLAVNLNFLLFSVYIDDCIGQL
FALLILTVAAAESAIGLALLVVYYRIRGTIAVEFINLMKG
>Q37787 | NDUS3_BETVU
MDNQFIFKYSWETLPKKWVKKIEKSEHGNRFDTNTDYLFQLLCFLKLHTYTRFQVLIDIC
GVDYPSRKRRFEVVYNLLSTRYNSRIRLQTCADEVTRISLVVSLFPSAGWWEREVWDMFG
VSFINHPDLRRILTDYGFEGHPLRKDFPLSGYVEVRYDDPEKRVVSEPIEMTQEFRYFDF

ASPWEQRNGNEG
>Q41346 | CYC_STELP
MGFKEGDAKKGANLFKTRCAQCHTLGEGEGNKIGPNLHGLFGRHTGSVEGFSYTDANKAK
GIEWNKDTLFEYLENPKKYIPGTKMAFGGLKKDKDRNDLITFLQDSTK
>Q41629 | ADT1_WHEAT
MTQNLGISVPIMSPSPMFANAPPEKKGVKNFAIDFLMGGVSAAVSKTAAAPIERVKLLIQ
NQDEMIKAGRLSEPYKGIGDCFGRTIKDEGFGSLWRGNTANVIRYFPTQALNFAFKDYFK
RMFNFKKDKDGYWKWFGGNLASGGAAGASSLFFVYSLDYARTRLANDAKASKGGGERQFN
GLVDVYRKTLKSDGIAGLYRGFNISCVGIIVYRGLYFGLYDSLKPVLLTGTLQVCFFASF
ALGWLITNGAGLASYPIDTVRRRMMMTSGEAVKYKSSLDAFQQILKKEGAKSLFKGAGAN
ILRAIAGAGVLSGYDQLQILFFGKKYGSGGA
>Q41630 | ADT2_WHEAT
MTQNLGISVPMMSSSPLFANAPPEKKGVKNFAIDFLMGGVSAAVSKTAAAPIERVKLLIQ
NQDEMIKAGRLSEPYKGIGDCFGRTIKDEGFGSLWRGNTANVIRYFPTQALNFAFKDYFK
RMFNFKKDKDGYWKWFGGNLASGGAAGASSLFFVYSLDYGRTRLANDAKASKGGGDRQFN
GLVDVYRKTLKSDGIAGLYRGFNISCVGIIVYRGLYFGLYDSLKPVLLTGTLQVCSFASF
ALGWLITNGAGLASYPIDTVRRRMMMTSGEAVKYKSSLDAFQQIPAKEGAKSLFKGAGAN
ILRAIAGAGVLSGYDQLQILFFGKKYGSGGA
>Q41898 | ATP5E_MAIZE
MSATTAAVPFWRAAGMTYIGYSNICAALVRNCLKEPFKSEAASREKVHFSISKWTDGKQE
KPTVRTESDD
>Q42525 | HXK1_ARATH
MGKVAVGATVVCTAAVCAVAVLVVRRRMQSSGKWGRVLAILKAFEEDCATPISKLRQVAD
AMTVEMHAGLASDGGSKLKMLISYVDNLPSGDEKGLFYALDLGGTNFRVMRVLLGGKQER
VVKQEFEEVSIPPHLMTGGSDELFNFIAEALAKFVATECEDFHLPEGRQRELGFTFSFPV
KQTSLSSGSLIKWTKGFSIEEAVGQDVVGALNKALERVGLDMRIAALVNDTVGTLAGGRY
YNPDVVAAVILGTGTNAAYVERATAIPKWHGLLPKSGEMVINMEWGNFRSSHLPLTEFDH
TLDFESLNPGEQILEKIISGMYLGEILRRVLLKMAEDAAFFGDTVPSKLRIPFIIRTPHM
SAMHNDTSPDLKIVGSKIKDILEVPTTSLKMRKVVISLCNIIATRGARLSAAGIYGILKK
LGRDTTKDEEVQKSVIAMDGGLFEHYTQFSECMESSLKELLGDEASGSVEVTHSNDGSGI
GAALLAASHSLYLEDS
>Q42560 | ACO1_ARATH
MASENPFRSILKALEKPDGGEFGNYYSLPALNDPRIDKLPYSIRILLESAIRNCDEFQVK
SKDVEKILDWENTSPKQVEIPFKPARVLLQDFTGVPAVVDLACMRDAMNNLGGDSNKINP
LVPVDLVIDHSVQVDVARSENAVQANMELEFQRNKERFAFLKWGSNAFHNMLVVPPGSGI
VHQVNLEYLARVVFNTNGLLYPDSVVGTDSHTTMIDGLGVAGWGVGGIEAEATMLGQPMS
MVLPGVVGFKLTGKLRDGMTATDLVLTVTQMLRKHGVVGKFVEFHGEGMRELSLADRATI
ANMSPEYGATMGFFPVDHVTLQYLRLTGRSDDTVSMIEAYLRANKMFVDYSEPESKTVYS
SCLELNLEDVEPCVSGPKRPHDRVPLKEMKADWHSCLDNRVGFKGFAVPKEAQSKAVEFN
FNGTTAQLRHGDVVIAAITSCTNTSNPSVMLGAALVAKKACDLGLEVKPWIKTSLAPGSG
VVTKYLAKSGLQKYLNQLGFSIVGYGCTTCIGNSGDIHEAVASAIVDNDLVASAVLSGNR
NFEGRVHPLTRANYLASPPLVVAYALAGTVDIDFETQPIGTGKDGKQIFFRDIWPSNKEV
AEVVQSSVLPDMFKATYEAITKGNSMWNQLSVASGTLYEWDPKSTYIHEPPYFKGMTMSP
PGPHGVKDAYCLLNFGDSITTDHISPAGSIHKDSPAAKYLMERGVDRRDFNSYGSRRGND

EIMARGTFANIRIVNKHLKGEVGPKTVHIPTGEKLSVFDAAMKYRNEGRDTIILAGAEYG
SGSSRDWAAKGPMLLGVKAVISKSFERIHRSNLVGMGIIPLCFKAGEDAETLGLTGQELY
TIELPNNVSEIKPGQDVTVVTNNGKSFTCTLRFDTEVELAYFDHGGILQYVIRNLIKQ
>Q42777 | MCCA_SOYBN
MASLALLRRTTLSHSHVRARAFSEGKSSNRHRIEKILVANRGEIACRITRTARRLGIQTV
AVYSDADRDSLHVATADEAIRIGPPPARLSYLNGASIVDAAIRSGAQAIHPGYGFLSESA
DFAKLCEESGLTFIGPPASAIRDMGDKSASKRIMGAAGVPLVPGYHGYDQDIEKMKLEAD
RIGYPVLIKPTHGGGGKGMRIVHTPDEFVESFLAAQREAAASFGVNTILLEKYITRPRHI
EVQIFGDKHGNVLHLYERDCSVQRRHQKIIEEAPAPNISADFRAQLGVAAVSAAKAVNYY
NAGTVEFIVDTVSDEFYFMEMNTRLQVEHPVTEMIVGQDLVEWQILVANGEALPLSQSQV
PLSGHAFEARIYAENVQKGFLPATGVLHHYHVPVSSAVRVETGVKEGDKVSMHYDPMIAK
LVVWGENRAAALVKLKDSLSKFQVAGLPTNVNFLQKLANHRAFAIGNVETHFIDNYKEDL
FVDANNSVSVKEAYEAARLNASLVAACLIEKEHFILARNPPGGSSLLPIWYSSPPFRIHH
QAKRRMELEWDNEYGSGSSKIMKLTITYQPDGRYLIETEQNGSPVLEVKSTYVKDNYFRV
EAAGVINDVNVAVYSKDQIRHIHIWQGSCHHYFREKLGLELSEDEESQHKPKVETSANPQ
GTVVAPMAGLVVKVLVENKTRVEEGQPVLVLEAMKMEHVVKAPSSGYVHGLQLMVGEQVS
DGSVLFSVKDQ
>Q43008 | SODM_ORYSJ
MALRTLASRKTLAAAALPLAAAAAARGVTTVALPDLPYDYGALEPAISGEIMRLHHQKHH
ATYVANYNKALEQLDAAVAKGDAPAIVHLQSAIKFNGGGHVNHSIFWNNLKPISEGGGDP
PHAKLGWAIDEDFGSFEALVKKMSAEGAALQGSGWVWLALDKEAKKLSVETTANQDPLVT
KGANLVPLLGIDVWEHAYYLQYKNVRPDYLSNIWKVMNWKYAGEVYENATA
>Q56XE8 | HXK4_ARATH
MGKVLVMLTAAAAVVACSVATVMVRRRMKGRRKWRRVVGLLKDLEEACETPLGRLRQMVD
AIAVEMQAGLVSEGGSKLKMLLTFVDDLPNGSETGTYYALHLGGSYFRIIKVHLGGQRSS
LEVQDVERHSIPTSLMNSTSEVLFDFLASSLQRFIEKEGNDFSLSQPLKRELAFTFSFPV
KQTSISSGVLIKWTKGFAISEMAGEDIAECLQGALNKRGLDIRVAALVNDTVGALSFGHF
HDPDTIAAVVFGTGSNACYLERTDAIIKCQNPRTTSGSMVVNMEWGNFWSSRLPRTSYDL
ELDAESMNSNDMGFEKMIGGMYLGDIVRRVILRMSQESDIFGPISSILSTPFVLRTNSVS
AMHEDDTSELQEVARILKDLGVSEVPMKVRKLVVKICDVVTRRAARLAAAGIAGILKKVG
RDGSGGGRRSDKQIMRRTVVAVEGGLYLNYRMFREYMDEALRDILGEDVAQHVVVKAMED
GSSIGSALLLASSQSVQTIPSV
>Q5M729 | OPD23_ARATH
MAYASRIINHSKKLKDVSTLLRRENAATIRYYSNTNRAPLNREDTFNSRLGYPPLERISI
CSTSTLPVSIIFSTTRSNLSSAMGRPIFGKEFSCLMQSARGFSSGSDLPPHQEIGMPSLS
PTMTEGNIARWLKKEGDKVAPGEVLCEVETDKATVEMECMEEGYLAKIVKAEGSKEIQVG
EVIAITVEDEEDIGKFKDYTPSSTADAAPTKAEPTPAPPKEEKVKQPSSPPEPKASKPST
PPTGDRVFASPLARKLAEDNNVPLSDIEGTGPEGRIVKADIDEYLASSGKGATAKPSKST
DSKAPALDYVDIPHSQIRKVTASRLAFSKQTIPHYYLTVDTCVDKLMALRSQLNSFKEAS
GGKRISVNDLVVKAAALALRKVPQCNSSWTDDYIRQFKNVNINVAVQTENGLYVPVVKDA
DRKGLSTIGEEVRLLAQKAKENSLKPEDYEGGTFTVSNLGGPFGIKQFCAVVNPPQAAIL
AVGSAEKRVVPGNGPDQFNFASYMPVTLSCDHRVVDGAIGAEWLKAFKGYIENPKSMLL
>Q5YLB5 | GYRA_NICBE
MKLHTLNPQTPLTQSKPMAFSTGITPSRFSGLRKTSSELRFLSSVTPPPRKQLRPVSARR

KEEEVGDEGNGSVILRDRGENEDRNGGERVVLTELHKEATEAYMSYAMSVLLGRALPDVR
DGLKPVHRRILYAMHELGLSSKKPYKKCARVVGEVLGKFHPHGDTAVYDSLVRMAQDFSL
RSPLIRGHGNFGSIDADPPAAMRYTECRLEALTESMLLADLEQNTVDFVPNFDNSQKEPS
LLPARVPNLLLNGASGIAVGMATNIPPHNLGELVDALSALIHNPEATLQELLEYMPGPDF
PTGGIIMGNIGILEAFRTGRGRVVIRGKTDIELLDSKTKRAAIIIQEIPYQTNKASLVEK
IADLVENKILEGVSDIRDESDRSGMRIVIELKRGSDPAIVLNNLYRLTALQSSFSCNMVG
ILNGQPKLMGLKELLQAFLDFRCSVVERRARFKLSQAQERNHIVEGIIVGLDNLDEVINT
IRKASSNALAAASLRKEFELSEKQAEAILDISLRRLTALERNKFVEEGKSLRTQISKLEE
LLSSKKQILQLIEEEAIEIKNKFFNPRRSMLEDTDSGDLEDIDVIPNEEMLLAISEKGYV
KRMKPDTFNLQNRGTIGKSVGKLRVNDAMSDFLVCRAHDKVLYFSDKGTVYSSPAYKIPE
CSRTAAGTPLVQILSLSDGERITSIIPVSEFAADQYLVMLTVNGYIKKVSLNYFASIRCT
GIIAIQLVPDDELKWVKCCSNNDFVAMASQNGMVILTPCANIRALGRNTRGSVAMRLKEG
DKVASMDIIPDALQKELDKTLEVQQRQYRSMKGPWLLFVSESGYGKRVPVSRFRTSPLNR
VGLFGYKFSSEDCLAAVFVVGFSLGEDGESDEQVVLVSQSGTVNRIKVRDISIQSRYARG
VILMRLEHAGKIQSASLISAADADPEDEDATAVAA

>Q6K548 | VDAC1_ORYSJ

MVGPGLYPEIGKKARDLLYRDYQTDHKFTLTTYTSNGVAITATSTKKADLIFGEIQSQIK
NKNITVDVKANSDSNVVTTVTVDELTPGLKSILSFAVPDQRSGKFELQYSHDYAGVSASI
GLTASPVVNLSSVFGTKALAVGADVSLDTATGNLTKYNAGLSFSNDDLIASLNLNNKGDS
LTASYYHIVNHSATAVGAELTHSFSSNENSLTFGTQHTLDPLTVVKARFNNSGKASALLQ
HEWRPKSVWTISAEVDTKAIDKSSKVGIAVALKP

>Q8H1Y0 | ODPA2_ARATH

MALSRLSSRSNTFLKPAITALPSSIRRHVSTDSSPITIETAVPFTSHLCESPSRSVETSS
EEILAFFRDMARMRRMEIAADSLYKAKLIRGFCHLYDGQEALAVGMEAAITKKDAIITSY
RDHCTFIGRGGKLVDAFSELMGRKTGCSHGKGGSMHFYKKDASFYGGHGIVGAQIPLGCG
LAFAQKYNKDEAVTFALYGDGAANQGQLFEALNISALWDLPAILVCENNHYGMGTATWRS
AKSPAYFKRGDYVPGLKVDGMDALAVKQACKFAKEHALKNGPIILEMDTYRYHGHSMSDP
GSTYRTRDEISGVRQVRDPIERVRKLLLTHDIATEKELKDMEKEIRKEVDDAVAQAKESP
IPDASELFTNMYVKDCGVESFGADRKELKVTLP

>Q8L6J5 | RPO1B_TOBAC

MWRYISKHAYSRKFRNSHDSALLGFSQYSSSFGKTRPLQCLCEESTTHPNLGLSQNSIFS
RISRKVRHLEGICEESSKNPHLGLSQNSTFSSVKGDFRICGKRGSGSLGRLRSYGSAAEA
IVSTSEEDIDEIQELIEEMDKENEALKANLQPKQPKTIGGMGVGKYNFLRRRQIKVETEA
WEEAAKEYQELLMDMCEQKLAPNLPYMKSLFLGWFEPLRDAIAAEQKLCDEGKNRGAYAP
FFDQLPAEMMAVITMHKLMGLLMTGGGTGSARVVQAASYIGEAIEHEARIHRFLEKTKKS
NALSGDLEETPGDMMKERERLRKKVKILMKKQKLRQVRKIVKQQDDEKPWGQDNLVKVGC
RLIQILMETAYIQPPNDQLDDGPPDIRPAFVHTLKTVETMKGSRRYGVIQCDPLVRKGLD
KTARHMVIPYMPMLVPPQSWLGYDKGGYLFLPSYIMRTHGAKQQREAVKRVPKKQLEPVF
QALDTLGNTKWRVNRKVLGIVDRIWASGGRLADLVDREDVPLPEAPDTEDEAEIRKWKWK
VKGVKKENCERHSQRCDIELKLAVARKMKDEDGFYYPHNLDFRGRAYPMHPYLNHLGSDL
CRGILEFAEGRPLGTSGLRWLKIHLANVYGGGVDKLSYEGRVAFSENHLEDIFDSAERPL
EGKRWWLGAEDPFQCLATCINIAEALRSPSPETAISYMPIHQDGSCNGLQHYAALGRDKL
GAAAVNLVAGDKPADVYSGIAARVLDIMKRDAAKDPANDPNVMRARLLINQVDRKLVKQT
VMTSVYGVTYIGARDQIKKRLKERGVIEDDNELFAAACYAAKTTLTALGEMFEAARSIMS

WLGDCAKIIAMENHPVRWTTPLGLPVVQPYRKLGRHLIKTSLQILTLQRETDKVMVKRQR
TAFPPNFVHSLDGSHMMMTAIACKESGLSFAGVHDSYWTHACDVDQMNKILREKFVELYD
APILENLLESFQQSFPDLQFPPLPERGDFDLREVLESPYFFN
>Q8L7B5 | CH60B_ARATH
MYRLVSNVASKARIARKCTSQIGSRLNSTRNYAAKDIRFGVEARALMLRGVEDLADAVKV
TMGPKGRNVIIEQSWGAPKVTKDGVTVAKSIEFKDRIKNVGASLVKQVANATNDVAGDGT
TCATVLTRAIFTEGCKSVAAGMNAMDLRRGIKLAVDTVVTNLQSRARMISTSEEIAQVGT
ISANGDREIGELIAKAMETVGKEGVITIQDGKTLFNELEVVEGMKIDRGYISPYFITNPK
TQKCELEDPLILIHEKKISNINAMVKVLELALKKQRPLLIVAEDVESDALATLILNKLRA
NIKVCAVKAPGFGENRKANLHDLAALTGAQVITEELGMNLDNIDLSMFGNCKKVTVSKDD
TVVLDGAGDKQAIGERCEQIRSMVEASTSDYDKEKLQERLAKLSGGVAVLKIGGASETEV
SEKKDRVTDALNATKAAVEEGIVPGGGVALLYASKELEKLSTANFDQKIGVQIIQNALKT
PVYTIASNAGVEGAVVVGKLLEQDNPDLGYDAAKGEYVDMIKAGIIDPLKVIRTALVDAA
SVSSLLTTTEAVVTEIPTKEVASPGMGGGGMGGMGGMGGMGGMGF
>Q8LAD2 | SUCA2_ARATH
MSRQVTRLLGSLRHSGGGCSGSSKVCSLTSLVQSRSFGTTPPPPAAVFVDKNTRVICQGI
TGKNGTFHTEQAIEYGTKMVAGVTPKKGGTEHLGLPVFNTVAEAKAETKANASVIYVPAP
FAAAAIMEGLAAELDLIVCITEGIPQHDMVRVKAALNSQSKTRLIGPNCPGIIKPGECKI
GIMPGYIHKPGKIGIVSRSGTLTYEAVFQTTAVGLGQSTCVGIGGDPFNGTNFVDCLEKF
FVDPQTEGIVLIGEIGGTAEEDAAALIKENGTDKPVVAFIAGLTAPPGRRMGHAGAIVSG
GKGTAQDKIKSLRDAGVKVVESPAKIGAAMFELFQERGLLK
>Q8LBZ7 | DHSB1_ARATH
MASGLIGRLVGTKPSKLATAARLIPARWTSTGAEAETKASSGGGRGSNLKTFQIYRWNPD
NPGKPELQNYQIDLKDCGPMVLDALIKIKNEMDPSLTFRRSCREGICGSCAMNIDGCNGL
ACLTKIQDEASETTITPLPHMFVIKDLVVDMTNFYNQYKSIEPWLKRKTPASVPAKEILQ
SKKDRAKLDGMYECILCACCSTSCPSYWWNPESYLGPAALLHANRWISDSRDEYTKERLE
AIDDEFKLYRCHTILNCARACPKGLNPGKQITHIKQLQR
>Q8LFC0 | IDH1_ARATH
MSRRSLTLLKNLARNANGSGIQTRSVTYMPRPGDGAPRAVTLIPGDGIGPLVTNAVEQVM
EAMHAPIFFEKYDVHGEMSRVPPEVMESIRKNKVCLKGGLKTPVGGGVSSLNVQLRKELD
LFASLVNCFNLPGLPTRHENVDIVVIRENTEGEYAGLEHEVVPGVVESLKVITKFCSERI
AKYAFEYAYLNNRKKVTAVHKANIMKLADGLFLESCREVAKKYPSITYNEIIVDNCCMQL
VAKPEQFDVMVTPNLYGNLVANTAAGIAGGTGVMPGGNVGADHAVFEQGASAGNVGKDKI
VLENKANPVALLLSSAMMLRHLQFPSFADRLETAVKKVIAEGKCRTKDLGGTSTTQEVVD
AVIAKLD
>Q8LFT2 | DRP3B_ARATH
MSVDDLPPSSASAVTPLGSSVIPIVNKLQDIFAQLGSQSTIALPQVAVVGSQSSGKSSVL
EALVGRDFLPRGNDICTRRPLRLQLVQTKPSSDGGSDEEWGEFLHHDPVRRIYDFSEIRR
EIEAETNRVSGENKGVSDIPIGLKIFSPNVLDISLVDLPGITKVPVGDQPSDIEARIRTM
ILTYIKEPSCLILAVSPANTDLANSDALQIAGNADPDGHRTIGVITKLDIMDRGTDARNH
LLGKTIPLRLGYVGVVNRSQEDILMNRSIKDALVAEEKFFRSRPVYSGLTDRLGVPQLAK
KLNQVLVQHIKALLPSLKSRINNALFATAKEYESYGDITESRGGQGALLLSFITKYCEAY
SSTLEGKSKEMSTSELSGGARILYIFQSVFVKSLEEVDPCEDLTADDIRTAIQNATGPRS
ALFVPDVPFEVLVRRQISRLLDPSLQCARFIFDELVKISHQCMMKELQRFPVLQKRMDEV

IGNFLREGLEPSQAMIRDLIEMEMDYINTSHPNFIGGTKAVEQAMQTVKSSRIPHPVARP
RDTVEPERTASSGSQIKTRSFLGRQANGIITDQAVPTAADAERPAPAGSTSWSGFSSIFR
GSDGQAAAKNNLLNKPFSETTQEVYQNLSTIYLKEPPTILKSSETHSEQESVEIEITKLL
LKSYYDIVRKNVEDLVPKAIMHFLVNYTKRELHNVFIEKLYRENLIEELLKEPDELAIKR
KRTQETLRILQQANRTLDELPLEAESVERGYKIGSEAKHEELPGTRRSRTETNGNGRLHM

>Q8LPW2 | RT13_SOYBN
MFGSARILSDVTLRLRQNLSVHGVRVQNINIGGGVGGEIPDNKRLVYALQNLHGIGRSKA
QHIVAELGVENKFVKDLSKRELYSIRELLSKYLIGNDLKKCVERDVGRLVGIQCYRGIRH
VDSLPCRGQRTHTNARTRRSRKTFSGSR
>Q8RWN9 | OPD22_ARATH
MASRIINHSKKLKHVSALLRRDHAVAVRCFSNSTHPSLVGREDIFKARLNYSSVERISKC
GTGNVTMLSGISTTSTKLSSPMAGPKLFKEFISSQMRSVRGFSSSSDLPPHQEIGMPSLS
PTMTEGNIARWLKKEGDKVAPGEVLCEVETDKATVEMECMEEGFLAKIVKEEGAKEIQVG
EVIAITVEDEDDIQKFKDYTPSSDTGPAAPEAKPAPSLPKEEKVEKPASAPEAKISKPSS
APSEDRIFASPLARKLAEDNNVPLSSIKGTGPEGRIVKADVEDFLASGSKETTAKPSKQV
DSKVPALDYVDIPHTQIRKVTASRLAFSKQTIPHYYLTVDTCVDKMMGLRSQLNSFQEAS
GGKRISVNDLVIKAAALALRKVPQCNSSWTDEYIRQFKNVNINVAVQTENGLYVPVVKDA
DKKGLSTIGEEVRFLAQKAKENSLKPEDYEGGTFTVSNLGGPFGIKQFCAVINPPQAAIL
AIGSAEKRVVPGTGPDQYNVASYMSVTLSCDHRVIDGAIGAEWLKAFKGYIETPESMLL
>Q8VWF8 | RPOT2_NICSY
MSSTKTPISLTIKLNQFTDKPTGLDINPYHNSPIMWRNIIKQLSSRTPQKLLFSSKNRTY
SFLGFGQDSIFKDNTKFRSLIPISCSNIVMGFQNLGEYLPGDEFLSRPLIKNQVNNNFCC
RKSYASVAEAVAVSSTDAEEDVSVVDEVHELLTELKKEEKKQFAFRRRKQRMLTSGMGHR
KYQTLKRRQVKVETEAWEQAAKEYKELLFDMCEQKLAPNLPYVKSLFLGWFEPLRDKIAE
EQELCSQGKSKAAYAKYFYQLPADMMAVITMHKLMGLLMTGGDHGTARVVQAALVIGDAI
EQEVRIHNFLEKTKKQKAEKDKQKEDGEHVTQEQEKLRKKVTNLMKKQKLRAVGQIVRRQ
DDSKPWGQDARAKVGSRLIDLLLQTAYIQPPANQLAVDPPDIRPAFVHSVRTVAKETKSA
SRRYGIIQCDELVFKGLERTARHMVIPYMPMLVPPVKWTGYDKGGHLYLPSYVMRTHGAR
QQREAVKRASRNQLQPVFEALDTLGNTKWRINKRVLSVVDRIWAGGGRLADLVDRDDAPL
PEEPDTEDEALRTKWRWKVKSVKKENRERHSQRCDIELKLAVARKMKDEESFFYPHNVDF
RGRAYPMHPHLNHLGSDICRGVLEFAEGRPLGESGLRWLKIHLANLFAGGVEKLSLEGRI
GFTENHMDDIFDSSDKPLEGRRWWLNAEDPFQCLAVCINLSEAVRSSSPETSVSHIPVHQ
DGSCNGLQHYAALGRDKLGAAAVNLVAGEKPADVYSGIAARVLDIMKRDAQRDPAEFPDA
VRARVLVNQVDRKLVKQTVMTSVYGVTYIGARDQIKRRLKERGAIADDSELFGAACYAAK
VTLTALGEMFEAARSIMTWLGECAKIIASENEPVRWTTPLGLPVVQPYRKIGRHLIKTSL
QILTLQRETEKVMVKRQRTAFPPNFIHSLDGSHMMMTAVACRRAGLNFAGVHDSYWTHAC
DVDKLNRILREKFVELYEAPILEKLLESFQTSYPTLLFPPLPERGDFDMRDVLESPYFFN

>Q93Y94 | RPOT1_NICSY
MWRYISKQAYSRKFRNSHDSALLGFSQYSSSFGKTRPLQCLCEESTTNPNLGLSQNSIFS
RISRKVRHLEGICEESSKNPHLGLSQNSLFSSVKGDFRVCGKRGSGSLGFLRSYGSAAEA
IASTSEEDIDEIQELIEEMNKENEALKTNLQPKQPKTIGGMGVGKYNLLRRRQIKVETEA
WEEAAKEYQELLMDMCEQKLAPNLPYMKSLFLGWFEPLRDAIAAEQKLCDEGKNRGAYAP

FFDQLPAEMMAVITMHKLMGLLMTGGGTGSARVVQAASHIGEAIEHEARIHRFLEKTKKS
NALSGDLEDTPGDIMKERERVRKKVKILMKKQKLQQVRKIVKQQDDEKPWGQDNLVKVGC
RLIQILMETAYIQPPNDQLDDCPPDIRPAFVHTLKTVETMKGSRRYGVIQCDPLVRKGLD
KTARHMVIPYMPMLVPPQSWLGYDKGAYLFLPSYIMRTHGAKQQREAVKRVPKKQLEPVF
QALDTLGNTKWRLNRKVLGIVDRIWASGGRLADLVDREDVPLPEEPDAEDEAQIRKWKWK
VKGVKKENCERHSQRCDIELKLAVARKMKDEDGFYYPHNLDFRGRAYPMHPYLNHLGSDL
CRGILEFAEGRPLGKSGLRWLKIHLANVYGGGVDKLSYEGRVAFSENHVEDIFDSAERPL
EGKRWWLGAEDPFQCLATCINIAEALRSPSPETAISYMPIHQDGSCNGLQHYAALGRDTL
GAAAVNLVAGDKPADVYSGIAARVLDIMKRDAAKDPANDPNVMRARLLINQVDRKLVKQT
VMTSVYGVTYIGARDQIKRRLKERGVIEDDNELFAACYAAKTTLTALGEMFEAARSIMS
WLGDCAKIIAMENHPVRWTTPLGLPVVQPYRKLGRHLIKTSLQILTLQRETDKVMVKRQR
TAFPPNFVHSLDGSHMMMTAIACKESGLSFAGVHDSYWTHASDVDQMNKILREKFVELYD
APILENLLESFQQSFPDLQFPPLPERGDFDLREVLESPYFFN
>Q93ZM7 | CH60C_ARATH
MYRVLSKLSSSIGSSTSRKLVSGRIISSRNYAAKDISFGIGARAAMLQGVSEVAEAVKVT
MGPKGRNVIIESSYGGPKITKDGVTVAKSISFQAKAKNIGAELVKQVASATNKVAGDGTT
CATVLTQAILIEGCKSVAAGVNVMDLRVGINMAIAAVVSDLKSRAVMISTPEEITQVATI
SANGEREIGELIARAMEKVGKEGVITVADGNTLDNELEVVEGMKLARGYISPYFITDEKT
QKCELENPIILIHEKKISDINSLLKVLEAAVKSSRPLLIVAEDVESDALAMLILNKHHGG
LKVCAIKAPGFGDNRKASLDDLAVLTGAEVISEERGLSLEKIRPELLGTAKKVTVTRDDT
IILHGGGDKKLIEERCEELRSANEKSTSTFDQEKTQERLSKLSGGVAVFKVGGASESEVG
ERKDRVTDALNATRAAVEEGIIPGGGVALLYATKALDNLQTENEDQRRGVQIVQNALKAP
AFTIAANAGYDGSLVVGKLLEQDDCNFGFDAAKGKYVDMVKAGIIDPVKVIRTALTDAAS
VSLLLTTTEASVLVKADENTPNHVPDMASMGM
>Q945K7 | IDH5_ARATH
MTMAANLARRLIGNRSTQILGAVNSSSGAASSVARAFCSSTTPITATLFPGDGIGPEIAE
SVKKVFTTAGVPIEWEEHYVGTEIDPRTQSFLTWESLESVRRNKVGLKGPMATPIGKGHR
SLNLTLRKELNLYANVRPCYSLPGYKTRYDDVDLITIRENTEGEYSGLEHQVVRGVVESL
KIITRQASLRVAEYAFLYAKTHGRERVSAIHKANIMQKTDGLFLKCCREVAEKYPEITYE
EVVIDNCCMMLVKNPALFDVLVMPNLYGDIISDLCAGLVGGLGLTPSCNIGEDGVALAEA
VHGSAPDIAGKNLANPTALLLSGVMMLRHLKFNEQAEQIHSAIINTIAEGKYRTADLGGS
STTTEFTKAICDHL
>Q94B78 | GCSP2_ARATH
MERARRLAYRGIVKRLVNDTKRHRNAETPHLVPHAPARYVSSLSPFISTPRSVNHTAAFG
RHQQTRSISVDAVKPSDTFPRRHNSATPDEQTHMAKFCGFDHIDSLIDATVPKSIRLDSM
KFSKFDAGLTESQMIQHMVDLASKNKVFKSFIGMGYYNTHVPTVILRNIMENPAWYTQYT
PYQAEISQGRLESLLNFQTVITDLTGLPMSNASLLDEGTAAAEAMAMCNNILKGKKKTFV
IASNCHPQTIDVCKTRADGFDLKVVTSDLKDIDYSSGDVCGVLVQYPGTEGEVLDYAEFV
KNAHANGVKVVMATDLLALTVLKPPGEFGADIVVGSAQRFGVPMGYGGPHAAFLATSQEY
KRMMPGRIIGISVDSSGKQALRMAMQTREQHIRRDKATSNICTAQALLANMAAMYAVYHG
PAGLKSIAQRVHGLAGIFSLGLNKLGVAEVQELPFFDTVKIKCSDAHAIADAASKSEINL
RVVDSTTITASFDETTTLDDVDKLFKVFASGKPVPFTAESLAPEVQNSIPSSLTRESPYL
THPIFNMYHTEHELLRYIHKLQSKDLSLCHSMIPLGSCTMKLNATTEMMPVTWPSFTDIH
PFAPVEQAQGYQEMFENLGDLLCTITGFDSFSLQPNAGAAGEYAGLMVIRAYHMSRGDHH

RNVCIIPVSAHGTNPASAAMCGMKIITVGTDAKGNINIEEVRKAAEANKDNLAALMVTYP
STHGVYEEGIDEICNIIHENGGQVYMDGANMNAQVGLTSPGFIGADVCHLNLHKTFCIPH
GGGGPGMGPIGVKNHLAPFLPSHPVIPTGGIPQPEKTAPLGAISAAPWGSALILPISYTY
IAMMGSGGLTDASKIAILNANYMAKRLEKHYPVLFRGVNGTVAHEFIIDLRGFKNTAGIE
PEDVAKRLMDYGFHGPTMSWPVPGTLMIEPTESESKAELDRFCDALISIREEIAQIEKGN
ADVQNNVLKGAPHPPSLLMADTWKKPYSREYAAFPAPWLRSSKFWPTTGRVDNVYGDRKL
VCTLLPEEEQVAAAVSA
>Q95747 | RM16_ARATH
MYLTIKSIMLLWKYLLVTESQVSKCGFHIVKKKGDVLYPKRTKYSKYRKGRCSRGCKPDG
TKLGFGRYGIKSCKAGCLSYRAIEAARRAIIGHFHRAMSGQFRRNGKIWVRVFADLPITG
KPTEVRMGRGKGNPTGWIARVSTGQILFEMDGVSLANARQAATLAAHKLCLSTKFVQWS
>Q95748 | NDUS3_ARATH
MDNQFIFKYSWETLPKKWVKKMERSEHGNRFDTNTDYLFQLLCFLKLHTYTRVQVLIDIC
GVDYPSRKRRFEVVYNLLSTRYNSRIRVQTSADEVTRISSVVSLFPSAGWWEREVWDMFG
VSFINHPDLRRILTDYGFEGHPLRKDFPLSGYVQVRYDDPEKRVVSEPIEMTQEFRYFDF
ASPWEQRSDG
>Q95749 | RT03_ARATH
MARKGNPISVRLGKNRSSDSSWFSEYYYGKFVYQDVNLRSYFGSIRPPTRLTFGFRLGRC
IILHFPKRTFIHFFLPRRPRRLKRREKTRPGKEKGRWWTTFGKAGPIECLHSSDDTEEER
NEVRGRGARKRVESIRLDDRKKQNEIRGWPKKKQRYGYHDRLPSIKKNLSKLLRISGAFK
HPKYAGVVNDIAFLIENDDSFKKTKLFKLFFQNKSRSDGPTSYLRTLPAVRPSLNFLVMQ
YFFNTKNQINFDPVVVLNHFVAPGAAEPSTMGRANAQGRSLQKRIRSRIAFFVESLTSEK
KCLAEAKNRLTHFIRLANDLRFAGTTKTTISLFPFFGATFFFLRDGVGVYNNLDAREQLL
NQLRVKCWNLVGKDKIMELIEKLKNLGGIEELIKVIDMMIEIILRKRGIPYRYNSYFYEV
KKMRSFLSNRTNTKTLIESVKIKSVYQSASLIAQDISFQLKNKRRSFHSIFAKIVKEIPK
GVEGIRICFSGRLKDAAEKAQTKCYKHRKTSCNVFNHKIDYAPVEVFTRYGILGVKVWIS
YSQKKGRRAISETYEI
>Q95869 | RT12_NICSY
MPTKNQLIRHGREEKRRTDRTRALDQCPQKQGVCPRVSTRTPKKPNSAPRKIAKVRLSNR
HDIFAHIPGEGHNSQEHSMVLIRGGRVKDSPGVKFHCIRGVKDLLGIPDRRRGRSKYGAE
KPKSI
>Q96007 | NU3M_ALLCE
MSEFSPIFIYLVMSLLVSLILLGLPFLFASNSSTYPEKLSAYECGFDPFGDARSRFDIRF
YLVSILFIIFDLEVTFFFPWAVSLNKIDLFGFWSMMAFLLILTIGFLYEWKRGALDWE
>Q96008 | RT12_ALLCE
MPTFNQLIRHGREEKRRTDRTRALDQCPQKQGVCLRVLTITPKKPNSALRKIAKVRLTNR
HDIFAYIPGEGHNSQEHSIVLVRGGRVKDLPGVKFHCIRGVKDLLGIPDRRRGRSKYGAE
KPKSK
>Q96033 | RT12_HELAN
MPTLNQLIRHGREEKRRTDRTRALDQCPQKQGVCLRVSTRTPKKPNSALRKIAKVRLSNR
HDIFAYIPGEGHNLQEHSIVLIRGGRVKDLPGVKFHCIRGVKDLLGIPDRRKGRSKYGAE
KPKSR
>Q96253 | ATP5E_ARATH
MASNAAVPFWRAAGMTYISYSNICANIVRNCLKEPHKAEALTREKVHFSLSKWADGKPQK

PVLRSDTPEV
>Q9C641 | EFGM_ARATH
MARFPTSPAPNRLLRLFSSNKRSSSPTAALLTGDFQLIRHFSAGTAARVAKDEKEPWWKE
SMDKLRNIGISAHIDSGKTTLTERVLFYTGRIHEIHEVRGRDGVGAKMDSMDLEREKGIT
IQSAATYCTWKDYKVNIIDTPGHVDFTIEVERALRVLDGAILVLCSVGGVQSQSITVDRQ
MRRYEVPRVAFINKLDRMGADPWKVLNQARAKLRHHSAAVQVPIGLEENFQGLIDLIHVK
AYFFHGSSGENVVAGDIPADMEGLVAEKRRELIETVSEVDDVLAEKFLNDEPVSASELEE
AIRRATIAQTFVPVFMGSAFKNKGVQPLLDGVVSFLPSPNEVNNYALDQNNNEERVTLTG
SPDGPLVALAFKLEEGRFGQLTYLRVYEGVIKKGDFIINVNTGKRIKVPRLVRMHSNDME
DIQEAHAGQIVAVFGIECASGDTFTDGSVKYTMTSMNVPEPVMSLAVQPVSKDSGGQFSK
ALNRFQKEDPTFRVGLDPESGQTIISGMGELHLDIYVERMRREYKVDATVGKPRVNFRET
ITQRAEFDYLHKKQSGGAGQYGRVTGYVEPLPPGSKEKFEFENMIVGQAIPSGFIPAIEK
GFKEAANSGSLIGHPVENLRIVLTDGASHAVDSSELAFKMAAIYAFRLCYTAARPVILEP
VMLVELKVPTEFQGTVAGDINKRKGIIVGNDQEGDDSVITANVPLNNMFGYSTSLRSMTQ
GKGEFTMEYKEHSAVSNEVQAQLVNAYSASKATE
>Q9FMV1 | UMP7_ARATH
MATSIARLSRRGVTSNLIRRCFAAEAALARKTELPKPQFTVSPSTDRVKWDYRGQRQIIP
LGQWLPKVAVDAYVAPNVVLAGQVTVWDGSSVWNGAVLRGDLNKITVGFCSNVQERCVVH
AAWSSPTGLPAATIIDRYVTVGAYSLLRSCTIEPECIIGQHSILMEGSLVETRSILEAGS
VVPPGRRIPSGELWGGNPARFIRTLTNEETLEIPKLAVAINHLSGDYFSEFLPYSTVYLE
VEKFKKSLGIAV
>Q9FV51 | AMP1C_ARATH
MLQKISQSISLCNGDQFKPLIYLAGAPTNFISSPLSGKKKSSSLRIKRIQQLQSTLEDRI
NPPLVCGTVSPRLSVPDHILKPLYVESSKVPEISSELQIPDSIGIVKMKKACELAARVLD
YAGTLVRPFVTTDEIDKAVHQMVIEFGAYPSPLGYGGFPKSVCTSVNECMFHGIPDSRPL
QNGDIINIDVAVYLDGYHGDTSKTFLCGDVNGSLKQLVKVTEECLEKGISVCKDGASFKQ
IGKIISEHAAKYGYNMERFIGHGVGTVLHSEPLIYLHSNYDYELEYMIEGQTFTLEPILT
IGTTEFVTWPDKWTIVTADGGPAAQFEHTILITTTGAEILTISS
>Q9LFV6 | RPOT2_ARATH
MSSAQTPLFLANQTKVFDHLIPLHKPFISSPNPVSQSFPMWRNIAKQAISRSAARLNVSS
QTRGLLVSSPESIFSKNLSFRFPVLGSPCHGKGFRCLSGITRREEFSKSERCLSGTLARG
YTSVAEEEVLSTDVEEEPEVDELLKEMKKEKKRESHRSWRMKKQDQFGMGRTKFQNLWRR
QVKIETEEWERAAAEYMELLTDMCEQKLAPNLPYVKSLFLGWFEPLRDAIAKDQELYRLG
KSKATYAHYLDQLPADKISVITMHKLMGHLMTGGDNGCVKVVHAACTVGDAIEQEIRICT
FLDKKKKGDDNEESGGVENETSMKEQDKLRKKVNELIKKQKLSAVRKILQSHDYTKPWIA
DVRAKVGSRLIELLVRTAYIQSPADQQDNDLPDVRPAFVHTFKVAKGSMNSGRKYGVIEC
DPLVRKGLEKSGRYAVMPYMPMLVPPLKWSGYDKGAYLFLTSYIMKTHGAKQQREALKSA
PKGQLQPVFEALDTLGSTKWRVNKRVLTVVDRIWSSGGCVADMVDRSDVPLPEKPDTEDE
GILKKWKWEVKSAKKVNSERHSQRCDTELKLSVARKMKDEEAFYYPHNMDFRGRAYPMPP
HLNHLGSDLCRGVLEFAEGRPMGISGLRWLKIHLANLYAGGVDKLSLDGRLAFTENHLDD
IFDSADRPLEGSRWWLQAEDPFQCLAVCISLTEALRSPSPETVLSHIPIHQDGSCNGLQH
YAALGRDTLGAEAVNLVAGEKPADVYSGIATRVLDIMRRDADRDPEVFPEALRARKLLNQ
VDRKLVKQTVMTSVYGVTYIGARDQIKRRLKERSDFGDEKEVFGAACYAAKVTLAAIDEM
FQAARAIMRWFGECAKIIASENETVRWTTPLGLPVVQPYHQMGTKLVKTSLQTLSLQHET

DQVIVRRQRTAFPPNFIHSLDGSHMMMTAVACKRAGVCFAGVHDSFWTHACDVDKLNIIL
REKFVELYSQPILENLLESFEQSFPHLDFPPLPERGDLDLKVVLDSPYFFN
>Q9LJL3 | PREP1_ARATH
MLRTVSCLASRSSSSLFFRFFRQFPRSYMSLTSSTAALRVPSRNLRRISSPSVAGRRLLL
RRGLRIPSAAVRSVNGQFSRLSVRAVATQPAPLYPDVGQDEAEKLGFEKVSEEFISECKS
KAILFKHKKTGCEVMSVSNEDENKVFGVVFRTPPKDSTGIPHILEHSVLCGSRKYPVKEP
FVELLKGSLHTFLNAFTYPDRTCYPVASTNTKDFYNLVDVYLDAVFFPKCVDDAHTFQQE
GWHYELNDPSEDISYKGVVFNEMKGVYSQPDNILGRIAQQALSPENTYGVDSGGDPKDIP
NLTFEEFKEFHRQYYHPSNARIWFYGDDDPVHRLRVLSEYLDMFEASPSPNSSKIKFQKL
FSEPVRLVEKYPAGRDGDLKKKHMLCVNWLLSEKPLDLQTQLALGFLDHLMLGTPASPLR
KILLESGLGEALVSSGLSDELLQPQFGIGLKGVSEENVQKVEELIMDTLKKLAEEGFDND
AVEASMNTIEFSLRENNTGSFPRGLSLMLQSISKWIYDMDPFEPLKYTEPLKALKTRIAE
EGSKAVFSPLIEKLILNNSHRVTIEMQPDPEKATQEEVEEKNILEKVKAAMTEEDLAELA
RATEELKLKQETPDPPEALRCVPSLNLGDIPKEPTYVPTEVGDINGVKVLRHDLFTNDII
YTEVVFDIGSLKHELLPLVPLFCQSLLEMGTKDLTFVQLNQLIGRKTGGISVYPLTSSVR
GKDEPCSKIIVRGKSMAGRADDLFNLMNCLLQEVQFTDQQRFKQFVSQSRARMENRLRGS
GHGIAAARMDAMLNIAGWMSEQMGGLSYLEFLHTLEKKVDEDWEGISSSLEEIRRSLLAR
NGCIVNMTADGKSLTNVEKSVAKFLDLLPENPSGGLVTWDGRLPLRNEAIVIPTQVNYVG
KAGNIYSTGYELDGSAYVISKHISNTWLWDRVRVSGGAYGGFCDFDSHSGVFSYLSYRDP
NLLKTLDIYDGTGDFLRGLDVDQETLTKAIIGTIGDVDSYQLPDAKGYSSLLRHLLGVTD
EERQRKREEILTTSLKDFKDFAQAIDVVRDKGVAVAVASAEDIDAANNERSNFFEVKKAL

>Q9LKA3 | MDHM2_ARATH
MFRSMIVRSASPVKQGLLRRGFASESVPDRKVVILGAAGGIGQPLSLLMKLNPLVSSLSL
YDIANTPGVAADVGHINTRSQVSGYMGDDDLGKALEGADLVIIPAGVPRKPGMTRDDLFN
INAGIVKNLSIAIAKYCPQALVNMISNPVNSTVPIAAEIFKKAGTYDEKKLFGVTTLDVV
RARTFYAGKSDVNVAEVNVPVVGGHAGITILPLFSQASPQANLSDDLIRALTKRTQDGGT
EVVEAKAGKGSATLSMAYAGALFADACLKGLNGVPNVVECSFVQSTITELPFFASKVRLG
KNGVEEVLDLGPLSDFEKEGLEALKAELKSSIEKGIKFANQ
>Q9LPS1 | HXK3_ARATH
MGKVAVAFAAVAVVAACSVAAVMVGRRMKSRRKWRTVVEILKELEDDCDTPVGRLRQVVD
AMAVEMHAGLASEGGSKLKMLLTFVDDLPTGREKGTYYALHLGGTYFRILRVLLGDQRSY
LDVQDVERHPIPSHLMNSTSEVLFNFLAFSLERFIEKEENGSDSQGVRRELAFTFSFPVK
HTSISSGVLIKWTKGFEISEMVGQDIAECLQGALNRRGLDMHVAALVNDTVGALSLGYYH
DPDTVVAVVFGTGSNACYLERTDAIIKCQGLLTTSGSMVVNMEWGNFWSSHLPRTSYDID
LDAESSNANDMGFEKMISGMYLGDIVRRVILRMSEDSDIFGPISPVLSEPYVLRTNSVSA
IHEDDTPELQEVARILKDIGVSDVPLKVRKLVVKICDVVTRRAGRLAAAGIAGILKKIGR
DGSGGITSGRSRSEIQMQKRTVVAVEGGLYMNYTMFREYMEEALVEILGEEVSQYVVVKA
MEDGSSIGSALLVASLQS
>Q9M1D3 | CISY5_ARATH
MVMQDLKSQMQEIIPEQQDRLKKLKSEQGKVPVGNITVDMVLGGMRGMTGLLWETSLLDA
DEGIRFRGMSIPECQKILPSAESGEEPLPESLLWLLLTGKVPTKEQANALSTELAHRAAV
PAIDALPSTAHPMTQFASGVMALQVQSEFQKAYEQGDISKSKYWEPTFEDALNLIARVPV
VASYVYRRMYKDGSIIPLDDSLDYGANFSHMLGFDSPQMKELMRLYVTIHSDHEGGNVSA

HAGHLVGSALSDPYLSFAAALNGLAGPLHGLANQEVLLWIKLVVEECGESISKEQLKDYV
WKTLNSGKVVPGYGHGVLRKTDPRYICQREFALKHLPDDPLFQLVSKLYEVVPPILTELG
KVKNPWPNVDAHSGVLLNYYGLTEARYYTVLFGVSRSLGICSQLIWDRALGLPLERPKSV
NMDWLDNFTRLNR
>Q9M5K2 | DLDH2_ARATH
MAMASLARRKAYFLTRNISNSPTDAFRFSFSLTRGFASSGSDDNDVVIIGGGPGGYVAAI
KAAQLGLKTTCIEKRGALGGTCLNVGCIPSKALLHSSHMYHEAKHVFANHGVKVSSVEVD
LPAMLAQKDTAVKNLTRGVEGLFKKNKVNYVKGYGKFLSPSEVSVDTIDGENVVVKGKHI
IVATGSDVKSLPGITIDEKKIVSSTGALSLTEIPKKLIVIGAGYIGLEMGSVWGRLGSEV
TVVEFAADIVPAMDGEIRKQFQRSLEKQKMKFMLKTKVVGVDSSGDGVKLIVEPAEGGEQ
TTLEADVVLVSAGRTPFTSGLDLEKIGVETDKGGRILVNERFSTNVSGVYAIGDVIPGPM
LAHKAEEDGVACVEFIAGKHGHVDYDKVPGVVYTYPEVASVGKTEEQLKKEGVSYNVGKF
PFMANSRAKAIDTAEGMVKILADKETDKILGVHIMSPNAGELIHEAVLAINYDASSEDIA
RVCHAHPTMSEAIKEAAMATYDKPIHM
>Q9M5K3 | DLDH1_ARATH
MAMASLARRKAYFLTRNLSNSPTDALRFSFSLSRGFASSGSDENDVVIIGGGPGGYVAAI
KASQLGLKTTCIEKRGALGGTCLNVGCIPSKALLHSSHMYHEAKHSFANHGIKVSSVEVD
LPAMLAQKDNAVKNLTRGIEGLFKKNKVTYVKGYGKFISPNEVSVETIDGGNTIVKGKHI
IVATGSDVKSLPGITIDEKKIVSSTGALSLSEVPKKLIVIGAGYIGLEMGSVWGRLGSEV
TVVEFAGDIVPSMDGEIRKQFQRSLEKQKMKFMLKTKVVSVDSSSDGVKLTVEPAEGGEQ
SILEADVVLVSAGRTPFTSGLDLEKIGVETDKAGRILVNDRFLSNVPGVYAIGDVIPGPM
LAHKAEEDGVACVEFIAGKHGHVDYDKVPGVVYTHPEVASVGKTEEQLKKEGVSYRVGKF
PFMANSRAKAIDNAEGLVKILADKETDKILGVHIMAPNAGELIHEAVLAINYDASSEDIA
RVCHAHPTMSEALKEAAMATYDKPIHI
>Q9MF82 | RT07_BETVU
MGGLDSEQKQLIKKLVNFHMKEGKRTKVRAIVYQTFHRLARTEGDVIKLMIDAVENIKPI
CKVEKVRVAGTIYDVPGIVARDRQQTLAIRWILEAAFKRRISYRISLEKCLFDEILDAYR
KRGISRKKRENLHGLASANRSFAHFRWW
>Q9SIB9 | ACO2M_ARATH
MYLTASSSASSSIIRAASSRSSSLFSFRSVLSPSVSSTSPSSLLARRSFGTISPAFRRWS
HSFHSKPSPFRFTSQIRAVSPVLDRLQRTFSSMASEHPFKGIFTTLPKPGGGEFGKFYSL
PALNDPRVDKLPYSIRILLESAIRNCDNFQVTKEDVEKIIDWEKTSPKQVEIPFKPARVL
LQDFTGVPAVVDLACMRDAMNKLGSDSNKINPLVPVDLVIDHSVQVDVARSENAVQANME
LEFQRNKERFAFLKWGSTAFQNMLVVPPGSGIVHQVNLEYLGRVVFNTKGLLYPDSVVGT
DSHTTMIDGLGVAGWGVGGIEAEATMLGQPMSMVLPGVVGFKLAGKMRNGVTATDLVLTV
TQMLRKHGVVGKFVEFYGNGMSGLSLADRATIANMSPEYGATMGFFPVDHVTLQYLKLTG
RSDETVAMIEAYLRANNMFVDYNEPQQDRVYSSYLELNLDDVEPCISGPKRPHDRVTLKE
MKADWHSCLDSKVGFKGFAIPKEAQEKVVNFSFDGQPAELKHGSVVIAAITSCTNTSNPS
VMLGAGLVAKKACDLGLQVKPWIKTSLAPGSGVVTKYLLKSGLQEYLNEQGFNIVGYGCT
TCIGNSGEINESVGAAITENDIVAAAVLSGNRNFEGRVHPLTRANYLASPPLVVAYALAG
TVNIDFETEPIGKGKNGKDVFLRDIWPTTEEIAEVVQSSVLPDMFRATYESITKGNPMWN
KLSVPENTLYSWDPNSTYIHEPPYFKDMTMDPPGPHNVKDAYCLLNFGDSITTDHISPAG
NIQKDSPAAKFLMERGVDRKDFNSYGSRRGNDEIMARGTFANIRIVNKLMNGEVGPKTVH
IPSGEKLSVFDAAMRYKSSGEDTIILAGAEYGSGSSRDWAAKGPMLQGVKAVIAKSFERI

HRSNLVGMGIIPLCFKSGEDADTLGLTGHERYTIHLPTDISEIRPGQDVTVTTDNGKSFT
CTVRFDTEVELAYFNHGGILPYVIRNLSKQ
>Q9SMX3 | VDAC2_ARATH
MVKGPGLYTEIGKKARDLLYRDYQGDQKFSVTTYSSTGVAITTTGTNKGSLFLGDVATQV
KNNNFTADVKVSTDSSLLTTLTFDEPAPGLKVIVQAKLPDHKSGKAEVQYFHDYAGISTS
VGFTATPIVNFSGVVGTNGLSLGTDVAYNTESGNFKHFNAGFNFTKDDLTASLILNDKGE
KLNASYYQIVSPSTVVGAEISHNFTTKENAITVGTQHALDPLTTVKARVNNAGVANALIQ
HEWRPKSFFTVSGEVDSKAIDKSAKVGIALALKP
>Q9SRH5 | VDAC1_ARATH
MVKGPGLYTEIGKKARDLLYKDHNSDQKFSITTFSPAGVAITSTGTKKGDLLLGDVAFQS
RRKNITTDLKVCTDSTFLITATVDEAAPGLRSIFSFKVPDQNSGKVELQYLHEYAGISTS
MGLTQNPTVNFSGVIGSNVLAVGTDVSFDTKSGNFTKINAGLSFTKEDLIASLTVNDKGD
LLNASYYHIVNPLFNTAVGAEVSHKLSSKDSTITVGTQHSLDPLTSVKARVNSAGIASAL
IQHEWKPKSFFTISGEVDTKSIDKSAKVGLALALKP
>Q9SVM8 | GRP2_ARATH
MAFCNKLGGLLRQNISSNGNVPVTSMLGSLRLMSTKLFIGGLSWGTDDASLRDAFAHFGD
VVDAKVIVDRETGRSRGFGFVNFNDEGAATAAISEMDGKELNGRHIRVNPANDRPSAPRA
YGGGGGYSGGGGGYGGGGGGYGGGGGGYGGGGDGGGGF
>Q9SZJ5 | GLYM_ARATH
MAMAMALRRLSSSIDKPIRPLIRSTSCYMSSLPSEAVDEKERSRVTWPKQLNAPLEEVDP
EIADIIEHEKARQWKGLELIPSENFTSVSVMQAVGSVMTNKYSEGYPGARYYGGNEYIDM
AETLCQKRALEAFRLDPEKWGVNVQPLSGSPANFHVYTALLKPHERIMALDLPHGGHLSH
GYQTDTKKISAVSIFFETMPYRLDESTGYIDYDQMEKSATLFRPKLIVAGASAYARLYDY
ARIRKVCNKQKAVMLADMAHISGLVAANVIPSPFDYADVVTTTTHKSLRGPRGAMIFFRK
GVKEINKQGKEVLYDFEDKINQAVFPGLQGGPHNHTITGLAVALKQATTSEYKAYQEQVL
SNSAKFAQTLMERGYELVSGGTDNHLVLVNLKPKGIDGSRVEKVLEAVHIASNKNTVPGD
VSAMVPGGIRMGTPALTSRGFVEEDFAKVAEYFDKAVTIALKVKSEAQGTKLKDFVSAME
SSSTIQSEIAKLRHEVEEFAKQFPTIGFEKETMKYKN
>Q9TC96 | NDUS2_NEPOL
MAIEHASSIKKVKNFTLNFGPQHPAAHGVLRLVLELNGEVVARADPHIGLLHRGTEKLIE
YKTYTQALPYFDRLDYVSMMCQEHAYSLAVEKLLHCEVPERAQYIRVLFSEITRILNHLL
ALTTHAMDVGALTPFLWAFEEREKLIEFYERVSGSRMHAAYIRPGGVACDLPANLCEDIY
LFCQQFASRIDEMEEMLTNNRIWKQRLVDIGIVTAENAFAWGFSGVLLRGSGVAWDLRKT
QPYDVYNRMIFDVPVGTQGDCYDRYLCRVEEMRQSIHIIMQCLNQLPKGMIKADDKKITP
PSRSQMKQSMESLIHHFKLFTEGYTVPNSETYTSVEAPKGEFGVYLVSNGTNRPYRCKIR
APGFLHLQGLDMMSKNHMLADVVTIIGTQDIVFGEVDR
>Q9XGY5 | TIM13_ORYSJ
MDSFSSSSGSPPNTEALMDQIKAQLAQAYAQEFLETVGNKCFAKCVTKPGSSLSGSESSC
ISRCVDRYIEATGIVSRALFSSTR
>Q9ZP06 | MDHM1_ARATH
MFRSMLVRSSASAKQAVIRRSFSSGSVPERKVAILGAAGGIGQPLALLMKLNPLVSSLSL
YDIANTPGVAADVGHINTRSEVVGYMGDDNLAKALEGADLVIIPAGVPRKPGMTRDDLFN
INAGIVKNLCTAIAKYCPHALINMISNPVNSTVPIAAEIFKKAGMYDEKKLFGVTTLDVV
RARTFYAGKANVPVAEVNVPVIGGHAGVTILPLFSQATPQANLSSDILTALTKRTQDGGT

EVVEAKAGKGSATLSMAYAGALFADACLKGLNGVPDVIECSYVQSTITELPFFASKVRLG
KNGVEEVLDLGPLSDFEKEGLEALKPELKSSIEKGVKFANQ
>Q9ZPX5 | DHSA2_ARATH
MWRCLRVASSSRRSESNGAFITSQLSRFFSAPPSAGDKSSYTIVDHTYDAVVVGAGGAGL
RAAIGLSEHGFNTACITKLFPTRSHTVAAQGGINAALGNMSVDDWRWHMYDTVKGSDWLG
DQDAIQYMCREAPKAVIELENYGLPFSRTEDGKIYQRAFGGQSLEFGIGGQAYRCACAAD
RTGHALLHTLYGQAMKHNTQFFVEYFALDLIMNSDGTCQGVIALNMEDGTLHRFHAGSTI
LATGGYGRAYFSATSAHTCTGDGNAMVARAGLPLQDLEFVQFHPTGIYGAGCLITEGARG
EGGILRNSEGEKFMDRYAPTARDLASRDVVSRSMTMEIRQGRGAGPMKDYLYLYLNHLPP
EVLKERLPGISETAAIFAGVDVTREPIPVLPTVHYNMGGIPTNYHGEVITLRGDDPDAVV
PGLMAAGEAACASVHGANRLGANSLLDIVVFGRACANRVAEIQKPGEKLKPLEKDAGEKS
IEWLDRIRNSNGSLPTSKIRLNMQRVMQNNAAVFRTQETLEEGCDLIDKTWDSFGDVKVT
DRSMIWNSDLIETMELENLLVNACITMHSAEARKESRGAHAREDFTKRDDANWMKHTLGY
WEEGNVKLEYRPVHMKTLDDEVDTFPPKPRVY
>Q9ZT91 | EFTM_ARATH
MASVVLRNPSSKRLVPFSSQIYSRCGASVTSSYSISHSIGGDDLSSSTFGTSSFWRSMAT
FTRNKPHVNVGTIGHVDHGKTTLTAAITKVLAEEGKAKAIAFDEIDKAPEEKKRGITIAT
AHVEYETAKRHYAHVDCPGHADYVKNMITGAAQMDGGILVVSGPDGPMPQTKEHILLARQ
VGVPSLVCFLNKVDVVDDPELLELVEMELRELLSFYKFPGDDIPIIRGSALSALQGTNDE
IGRQAILKLMDAVDEYIPDPVRVLDKPFLMPIEDVFSIQGRGTVATGRIEQGVIKVGEEV
EILGLREGGVPLKSTVTGVEMFKKILDNGQAGDNVGLLLRGLKREDIQRGMVIAKPGSCK
TYKKFEAEIYVLTKDEGGRHTAFFSNYRPQFYLRTADITGKVELPENVKMVMPGDNVTAV
FELIMPVPLETGQRFALREGGRTVGAGVVSKVMT

(3) 70 plastid proteins

>A2T833 | RR4_ANEMR
MSRYRGPRMKMIRRPGTLPGLTSKTPGTKVGSSDRSTSSKKISQYRIRLEEKQKLRLHYG
LTERQLLKYVFTARGAKGSTGQLLLQLLEMRLDNTIFRLGIVPTIPAARQLVNHRHVSIN
EHIIDIPSYNCKPGDVITINNREKCRLVDRRDMNSLQKPEIPNHLTFDSKEFLGSVQQII
DRDWIDLKINELLVVEYYSRRV
>A7M8Z3 | RR2_CUSGR
MTKKYWNINLEDMLEARVHLGHSTQNWNPKMAPYISAKRKGIHIINLTRTARFLSEACDL
VFYAASKGKKFLIVGTNNAADEAVARASKRARCHYVNKKWLGGMLTNWSTTETRLQKFRD
LRMEQKKGGLNNLPKKEATMLKRKLARLQKYLGGIQYMTGLPDIVIIIDQHKEYTALQEC
RILGIPTISLIDTNCDPNLSDIAIPANDDAMASIRFILNKLVFAICQGYFSDLRKP
>A7M8Z9 | RR14_CUSGR
MARKSLIQREKKRKKLEQKYHLIRRSLKQEIHKVSLLSEKREIYVKLQSLPRNSAPTRLR
RRCFMTGRPRANYRDFELSGHILREMVETCLLPGAMRSSW
>A7M903 | RR4_CUSGR
MSRYRGPRFKKIRRLGALPGLTNKSPRAIRDLRNQSRSEYRIRLEEKQKLRFHYGLTEKQ
LINYVRIARKAKGSTGKVLLQLLEMRLDNILFRLGMASTIPAARQLVNHRHVLVNGRLVD
RPSYRCKPRDIIMPKNTTKSGVLVQNSLQLFTGKELATHLNLFSTPYKGLVNKIVDTNWI
GLKINELLVVEYYSRQT
>A7M907 | ACCD_CUSGR
MQNWIDNSFQAEFEQESYFGSLGENSTNPSSGGDRYPEALIIRDITGKTSAIYFDITDDI

LENDPHQTILLSPIENDIWTEKDVIIDTYRYINELIFCDEKSQQKQKDRTEFIKKEQLQL
ISNRNPDHYRNLWNQCENCFIPNYKKVLKSNMQICEECGSYFKMTSSDRIDLLIDEGTWN
PLDQDMVSLDSSEFDSEAELECYEDNIKEWNEEMCQAFMRKLSKDLKEGQALERTANLIE
EPWLPEYIQPEEKVEEWTKPDLDEGEESQDEERWIWELDKGEESQEIEDSEANDEDDDDA
PYVERLAFYKKETGLLDAVQTGVGQLNGRPVALGVMDFRFLAGSMGCVVGEKITRLIEYA
TNNLLPLIILSASGGARVHEGSLSLMQMAKISAALYDYQSNKRLFYISILTSPTTGGVTA
SFAMLGDIIITEPGTFVAFAGPRVVQQILNETIPEEEQEAEALFEKGFFDLIVPRHLLKN
VISELLNLHAL
>A7M920 | RK20_CUSGR
MTRIKRGSIARARRTKIRFFASKFRGSHSRLTRTIIQQGLRAFVSSQRDRHKKKRDFRRL
WITRLNAAIRAIGVGYSYSASIHNLYKSQLILNRKILTQIAISNRNCLYMISNEILKSGV

>A7M922 | CLPP_CUSGR
MPIGVPRVRFLYDEDTGQVWIDIYNRLYRERCLFLTHTINTKIGNQLAGLFIYLGIQDDP
KDIFFFLNSPGGGIISGLAIYDSMQVVRPDTQTICVGLAASMACFLLVGGTITKRLAFPH
ARVMMHQPLSTFFETQTGDAVMEVDELLKMRENLIEVYAQRTGKPHWVISEDIERDVFLS
PTEAKTYGLVDVVGVTLI
>A7M929 | RR11_CUSGR
MVKLIPRLSSRRNGRIRLRKNTRKISQGVIHIQASLQNTIVTVTDVRGRVVSWASAGSAG
FKGTTRRTPFAAQTAATNAIRTAINHGMREADVLIKGPGLGRDAALRAIRRSGIRLELIL
DVTPMPHNGCRPPKKRRV
>A7M931 | RR8_CUSGR
MGRDTIAEVLTVIRNANMDGKKMVKIPSSNITENIIKLLLREGFLENVRKHYENGNYFLV
LTLRHQKTKKGPVTNILNLKQISRPGRRIYSMSKKIPRILGGIGIVILSTSHGILTDREA
RLEGIGGEILCYIW
>A7M937 | RK2_CUSGR
MIIKLYKIEPTSTLTRNGTSQAKPKNLISGKRNCGKGRNANGIITIRHRGGGHKRLCRKI
NFKRNEKDISGKIKTIEYDPNRNAYICLIHYVNGEKWYILHPRGALIGDTIISGPEVSIK
IGNSLPLNEIPLGTSLHNLEITRGKGGQLARAAGAAAKLIAKEGQSATLKLPSGELRFIS
KQCSATVGQVGNIGVNQKSLGKAGVKRWLGKRPIVRGLVMNPIDHPHGGGEGRAPIGRNQ
PKTPWGYPALGKKTRRKNKYSNKFILRHRM
>A7M939 | RR7_CUSGR
MSRRGTAEKKKAKSDPIYRNRLVNMLVNRILKHGKKSLAYQIMYRAVKTIQQNTEKNPLS
VLRQAIRGVTPDLTVKARRVSGSTHQVPIEIRSTQGKALAVRWLLAASRKRPGRDMAFKL
SSELVDAAKGRGDAIRKKEETHRMAEANRAFAHFR
>A7M955 | RR2_CUSRE
MTKRYWNINLEEMMGAGVHFGHGTRKWNPQMAPFIAAKRKGIHIINITRTARFLSEACDL
VFDAARRGKKFLIVGTNNKAADSVARASRKARCHYVNKKWLAGMLTNWSITETRLNKFRD
LRMEQKKGGLNHLLKRDATMFKRQLARLQTYLGGIQYMTGLPDIVIIVDQHKEYTALREC
ITLGIPTICLIDTNCDPNLSDISIPANDDAISSIRFILNKLVFAICEGRFSYLINP
>A7M957 | RPOC1_CUSRE
MNKNFSSMIDRYKHQQLRIGLVSPQQISAWATKILPNGEIVGEVKKPYTFLYKTNKPEKD
GLFCERIFGPIKSGICACGNYRVIGDEKEEPKFCEQCGVEFVDSRIRRYRMGCIKLACPV
THVWYLKRLPSYIANILDKPIKELEGLVYCDFYFARPITKKPTFLRLRGLLKYEIQSWKS

SIPLFFTTQGFDTFRNREISTGAGAIREQLADLDLKISLENSLLEWKYLGEEERIGNEWE
DRKAGRRKGFLVRRMELVKHFIRTNIEPEWMVLSLLPVLPPELRPIIQIDGGKLMSSDIN
ELYRRVIYRNNTLTDLLTTSRSTPGELVMCQEKLVQEAVDTLLDNGIRGQPMRDGHNKVY
KSFSDIIEGKEGRFRETLLGKRVDYSGRSVIVVGPSLSLHRCGLPRKIAIELFQPFVIRD
LIRQHLASNIGVAKSKIREKEPIIWEILQEVMRGHPVLLNRAPTLHRLGIQAFQPVLVEG
HVLCLHPLVCKGFNADFDGDQMAVHVPLSLEAQAEARLLMFSHMNLLSPAIGDPISVPTQ
DMLIGLYVLTSDNRRDICTNRYTKCNIQTLQTKSSDSSNSKSKNWYKNRPFFCNSYDAIG
AYRQKQINLESPLWLRWRLDRRVITSSETPIEVHYESRGTFSEIYGHFLIVRSLKKKILF
IYLRTTVGHISLYREIEEAIQGFSRAWSSDT
>A7M958 | RPOB_CUSRE
MLVDGKGGITTIPGLNQIQLEGFCRFIDQGLMEELSKFQKIEDIDQEIEFQLFVETYQLV
EPLIKERDAVYDSLTYSSELYVSAGLIRKASKDMQEQTIFIGSLPIMNSLGTFIVNGIYR
IVINQILQSPGIYYRSELDQNGISVYTGTIISDWGGRLELEIDRKARVWARVSRKQKISI
LVLLSAMGLNLREILENVCYPEILLSFLRDKEKKKIGSKENAILEFYKKFACVGGDPLFS
ESLCKELQNKFFQQRCELGRIGRRNMNRRLHLDIPHNNTFLLPRDILEATDHLIGLKFGM
GTLDDMNHLQNKRIRSVADLLQDQFGLALVRLENAVQGTLCGAIRHKRIPTPQNLVTSTL
LTTTYESFFGLHPLSQVLDGTNPLTQIVHARKVSSLGPGGLTGRTASFRIRDIHPSHYGR
ICPIDTSEGINVGLIGSLAIHVRIGNWGSLESPFYEISDRLTGVRVLHLSPGRDEYYMVA
AGNSLALNQDIQEDQVVPARYRQEFLTIAWEQVNLRSIFPFQYFSIGASLIPFIEHNDAN
RALMSSNMQRQAVPLTWSEKCIVGTGMERQAALDSGSLAIAEREGRVIYTDTEKILVSGD
GKTINIPLVMYQRSNKNTCMYQQPQVRRGQFIKKGQILAGGAATVEGELALGKSVLVAYM
PWEGYNFEDAVLISECLVYEDIFTSFHIKKYEIQIHMTTQGPEKVTNEIPHLEAHLIRNL
DKNGIVLQGSWVEPGDVLVGKLTPQVVKESAYAPEDRLLRAILGIPVSASKETCLKVPIG
ARGRVIDVRWIQKKGGYGYNPEKIRVYILQKREIKVGDKVAGRHGNKGIISKILPRQDMP
YLQDGRSVDLVFNPLGVPSRMNVGQIFECSLGLAGSLLDRHYRIAPFDERYEQEASRKIV
FSELYEASKQTANPWAFEPEYPGKSRIFDGRTGKTFEHPVLIGKPYILKLIHQVDDKIHG
RSIGHYALVTQQPLRGRAKQGGQRVGEMEVWALEGFGVAHILQEMLTYKSDHIRARQEVL
GTTIVGGTIPSPKNAPESFRLLVRELRSLALELTHFLVSEKNFQVNRKEA
>A7M964 | RR14_CUSRE
MARKSLIQRDKKRKKLELKYHWIRGSLKKEIHKVPLLSDKWEIYVKLQSLPRNSAPTRLH
RRCFLTGRPRANYRDFGLSGHRLREMVQACLLPGATRSSW
>A7M968 | RR4_CUSRE
MSRYRGPRFKKIRRLGALPGLTNKRPRTVRDLRNQSRSGKKSHYRIRLEEKQKLRFHYGL
TERQLISYVRIARKAKGSTGEVLIQLLEMRLDNILFRLGMAYTIPAARQLVNHRHILVNG
HIVDIPSYRCKPRDIITSKDKPKSGALIKNSIKAFPREELPNHLTLHPAPYKGLINQIID
TKWVGLKINELLVVEYYSRQT
>A7M986 | RK20_CUSRE
MTRIKRGSIARRRRKKMCFFASSFRGAHSRLTRTITQQGIRALVSADRDRDRQKRDFRRL
WITRLNAVIREMGIYYNYSKLIRDLYNNQLLLNRKILSQIAISNSKCLYMISNGILQI
>A7M996 | RR11_CUSRE
MAKSIPRISSRRNGRIGSGNNVRRIPKGVIHVQASFHNTIVTVTDVRGRVVSWSSAGTSG
FKGTRRGTPFAAQTAATNAIRTVVDQGMLRAEVLIKGPGLGRDAALRAIRRSGILLTFVR
DLTPMPHNGCRPPKQRRV
>A7M998 | RR8_CUSRE

MGRDTIAEIITSIRNADMDRKRVVRIASTHITENIVKLLLREGFFENVRKHRENNKNFFV
LTLRHRRNKKRPLRNILKLKRISRPGLRIYYKSQKIPRILGGMGVIIISTSRGIMTDREA
RLEGIGGEILFYIW
>A7M9A1 | RR3_CUSRE
MGQKINPLGFRLGTTQDHHSLWFSQPKNYSESLQEDKKIRDFINNYVKKNMRKASGAEGI
ARISIQKRIDLIQVVIFMGFPKFLIETRPQGIEELQRTLQKEFNCGNQKLNITITRIEKP
YGNPNILAEFIAVQLKNRVSFRKAIKKAIELAEQADTKGIQVQIAGRIDGKEIARVEWIR
EGRVPRQTIRANLDYSSYPVRTIYGVLGIKIWIFID
>A7M9A4 | RK2_CUSRE
MVIKLYKNYAPSTHNGTLKGQVKSARGKNLISGKHRCGKGRNARGIITARHRGGGHKRLY
RKIDFWRNKKNIYGRIVTIEYDPNRNAHICLIHYRNGEKRYILHPRGAIIGDTIVSGTEV
SIKIGNALPLTEMPLGTAIHNLEITRGKGGQLARAAGAVAKLIAKEGKSATLKLPSGEVR
LISKSCSATVGQVGNVGVNQKSLGRAGAQRWLGKRPVVRGVVMNPVDHPHGGGEGRAPIG
RKKPTTPWGYPALGRKTRKVNKYSEKFIIRHRRKQ
>A8W3B6 | RPOB_CUSEX
MLEDGKGGITTIPGLNQIQFEGFCRFIDQGLTEELYKFQKIEDIDQEIEFQLFAETYQLV
EPLIKERDAVYDSLTYSSELYVSAGLIRKASKDMQEQKIFIGSIPIMNSLGTSIVNGIYR
IVINQILQSPGIYYRSELDQNGISVYTGTIISDWGGRSELEIDRKARIWARVSRKQKISI
LVLSSAMGSNLREIIENVCYPEILLSFLRDKEKKKIGSKENAILEFYKKFACVGGDPLFS
ESLCKELQNKFFQQRCELGRIGRRNMNRRLHLDIPQNNTFLLPRDILEATDLLIGLKFGM
GTLDDMNHLQNKRIRSVADLLQDKFGLALVRLENAVQGTICGAIHHKKIPTPQNLVTSTL
LTTTYESFFGLHPLSQVLDRTNPLTQIVHARKVSSLGPGGLTGRTASFRIRDIHPSHYGR
ICPIDTSEGINVGLIGSLAIHVRIGNWGSLESPFFKISDRLTGVRVLHLSPGRDEYYMVA
AGNSLALNQDIQEDLVVPARFRQEFLTIAWEQVNLRSIFPFQYFSIGTSLIPFIEHNDAN
RALMSSNMQRQAVPLAWSEKCIVGTGVERQAALDSGSLAIAEREGRVIYTDTEKILVSGD
GKTISIPLVMYQRSNKNTCMYQQPQVRRGQFIKKGQILADGAATVEGELALGKSVLVAYM
PWEGYNYEDAVLISECLVYEDIFTSFHIRKYEIQTHVTTQGPEKVTNEIPHLEAHLIRNL
DKNGIVLQGSWVEPGDVLVGKLTPQVVKESSYAPEDRLLRAILGIQVSASKETCLKVPIG
GRGRVIDVRWIQKKGGYGYNPEKIRVYILQKREIKVGDKVAGRHGNKGIISKILPRQDMP
YLQDGRSVDLVFNPLGVPSRMNVGQIFECSLGLAGSLLDRHYRIAPFDERYEQEASRKIV
FSELYEASKQTANPWAFEPEYPGKSRIFDGRTGNTFEHPVLIGKPYILKLIHQVDDKIHG
RSSGHYALVTQQPLRGRAKQGGQRVGEMEVWALEGFGVAHILQEMLTYKSDHIRARQEVL
GTTIVGGTIPNPKNAPESFRLLVRELRSLALELTHFLVSEKNFQVNKREA
>A8W3C6 | RR4_CUSEX
MSRYRGPRFKKIRRLGALPGLTNKRPRTVRDLRNQSRSGKKSQYRIRLEEKQKLRFHYGL
TERQLINYVRIARKAKGSTGEILIQLLEMRLDNILFRLGMASTIPAARQLVNHRHIFVNG
HIVDIPSYRCKPRDIITSKDKPKSGALIKNSIEAFPREELPNHLTLHPAPYKGLINQIID
TKWVGLKINELLVVEYYSRQT
>A8W3F4 | RR11_CUSEX
MAKSIPRISSRRNGPIGSGKTVRRIPKGVIHVQASFHNTIVAVTDVRGRVVSWSSAGTSG
FKGTRRGTPFAAQTAATKAIRTVVDQGMLRAEVLIKGPGLGRDAALRAIRRSGILLTFVR
DVTPMPHNGCRPPKKRRV
>A8W3F6 | RR8_CUSEX
MGRDTIAEIITSIRNADMDRKRVVRIASTHITENIVKLLLREGFFENVRKHRENNKNFFV

LTLRHRRNKKRPLRNILKLKRISRPGLRIYYKSQRIPRILGGRGVVIISTSRGIMTDREA
RLEGIGGEILFYIW
>A8W3F9 | RR3_CUSEX
MGQKINPLGFRLGTTQDHHSLWFSQPKNYSESLQEDKKIRDFIKNYVQNNMIKASGAEGI
ARIYIQKRIDLIQVVIFMGFPKLLIETRPQGIKELQRTLQKEFNFGNQKLNITITRIEKP
YGNPNILAEFIAVQLKNRVSFRKAMKKAIELAEQADTKGIQVQIAGRVDGKEIARVEWIR
EGRVPRQTIRANLDYCSYPVRTIYGVLGVKIWIFLDQAK
>A8W3I9 | RR4_CUSOB
MSRYRGPRFKKIRRLGALPGLTNKSPRAIRDLRNQSRSEYRIRLEEKQKLRFHYGLTEKQ
LINYVQIARKAKGSTGKVLLQLLEMRLDNILFRLGMASTIPAARQLVNHRHVLVNGRLVD
RPSYRCKPRDIIMPKNTTKSGVLVQNSLELFTGKELANHLNLFSTPYKGLVNKIVDTNWI
GLKINELLVVEYYSRQA
>A8W3L5 | RR11_CUSOB
MVKLIPRISSRRNGRIRLRKNKHKIPQGVIHIQASLQNTIVTVTDVRGRVVSWASAGSAG
FKGTTRRTPFAAQTAATNAIHTAINQGMREADVLIKGPGLGRDAALRAIRRSGIRLELIL
DVTPMPHNGCRPPKKRRV
>A8W3L7 | RR8_CUSOB
MSRDTIAEVLTVIRNANMDGKKTVKIPSSNITENIIKLLLREGFLENVRKHYENGNYFLV
LTLRHQKTKKGTFTNILNLKKISRPGRRIYSTSKKIPRILGGIGIVILSTSHGILTDREA
RLEGIGGEILCYIW
>A8W3M0 | RR3_CUSOB
MGQKINPLGLRLGTNQDHYSIWFSQPKAYSKSLQEDQKIRSFIRKYIQNMKISPSGVEGL
ARISIYKRIDLIELRIFLGFPKLLLENRPQGLEKLQITLQKELNCGNRRLNIVITKVEKP
YSNPNILAEFIAGQLKNRVSVRQAMKKAIELAEEADTKGIQVQVAGRLNGQDIARVQWIR
EGRVPRQTIRATFDYCSYPVRTIYGILGIKIWIFVGEAK
>O63057 | RR7_LATCL
MSRRGTAEEKTAKSDPIYRNRLVNMLVNRILKHGKKALAYQIIYRAMKKIQQKTETNPLS
VLRQAIRGVTPDIAVKARRVGGSTHQVPIEIGSTQGKALAIRWLLAASRKRPGRDMAFKL
SSELVDAAKGSGDAIRKKEETHKMAEANRAFAHFR
>O78678 | RR4_OROMI
MSRYRGPRFKKIRRLGALPGLTNKRPKAEKDLINQSRFVKKSQYRIRLEEKQKLRFHYGL
TERQLLKYIRIAGKAKGSTGQVLLQLLEMRLDNILFRLGMALTIPAARQLVNHRHVLVNG
RIVDIPSYRCKPLDIITAKDKQQSRTLIQNSLNSSPHAKIPNHLTLDLFQYKGIVNQIIN
SKWVGLKINEFLIVEYYSRQIKT
>O99010 | RK12_PROWI
MSATTLEILEKLKAITLLEATELVSQIEKTFGVDASAPAVSGLRPILVEPIKQEDIIEEK
TTFDVILEEVPSDKRVPILKVIRALTSLDLKQAKESITDLPKTILQGVSKEESEAAKQQL
EAVGGKIKVS
>P19975 | FRI1_PEA
MALSSSKFSSFSGFSLSPVSGNGVQKPCFCDLRVGEKWGSRKFRVSATTAPLTGVIFEPF
EEVKKDYLAVPSVPLVSLARQNFADECESVINEQINVEYNASYVVHSLFAYFDRDNVALK
GFAKFFKESSEEHREHAEKLMKYQNTRGGRVVLHPIKDVPSEFEHVEKGDALYAMELALS
LEKLTNEKLLNVHSVAERNNDLEMTHFIEGEYLAEQVEAIKKISEYVAQLRRVGKGHGVW
HFDQRLLHGVHGA

>P19976 | FRI1_SOYBN
MALAPSKVSTFSGFSPKPSVGGAQKNPTCSVSLSFLNEKLGSRNLRVCASTVPLTGVIFE
PFEEVKKSELAVPTAPQVSLARQNYADECESAINEQINVEYNASYVYHSLFAYFDRDNVA
LKGFAKFFKESSEEEREHAEKLMKYQNTRGGRVVLHPIKNAPSEFEHVEKGDALYAMELA
LSLEKLVNEKLLNVHSVADRNNDPQMADFIESEFLSEQVESIKKISEYVAQLRRVGKGHG
VWHFDQRLLD
>P27071 | RR14_EPIVI
MARKSLIQREKGRLKLENKYHFIRRSSKNEISKVPSLSDKWEIYGKLESPPRNSAPTRLR
RRCFYTGRPRANYRDFGLCGHILREMVNACLLPGATRSSW
>P29036 | FRI1_MAIZE
MMLRVSPSPAAAVPTQLSGAPATPAPVVRVAAPRGVASPSAGAACRAAGKGKEVLSGVVF
QPFEEIKGELALVPQSPDKSLARHKFVDDCEAALNEQINVEYNASYAYHSLFAYFDRDNV
ALKGFAKFFKESSDEEREHAEKLMEYQNKRGGRVRLQSIVTPLTEFDHPEKGDALYAMEL
ALALEKLVNEKLHNLHGVATRCNDPQLTDFIESEFLEEQGEAINKISKYVAQLRRVGKGH
GVWHFDQMLLEEEA
>P29390 | FRI2_MAIZE
MMLRVSSSPAAAVANHLSGGAAATTAPARVTAQRSGVSLSAAAAAGKGKEVLSGVVFQPF
EEIKGELALVPQSPDRSLARHKFVDDCEAAINEQINVEYNASYAYHSLFAYFDRDNVALK
GFAKFFKESSDEEREHAEKLMEYQNKRGGRVRLQSIVAPLTEFDHPEKGDALYAMELTLA
LEKLVNEKLHSLHGVATRCNDPQLIDFIESEFLEEQGEAINKVSKYVAQLRRVGNKGHGV
WHFDQMLLQEAA
>P30055 | RR3_EPIVI
MGQKINPLGFRLGTTQSHHSFWFAQPKNYYKGIQEDQKIRDFIKNYVKNNIIISPDTEGI
AYIEIQKRIDFLKIMIFIGFKKFLIENRQLGIIKEALHIDLKKNFHYVNRKLIIDIIRIT
KPYRNPNILAEFIADQLKNRVSFRKTMKKAIELTESEDTKGIQVQISGRIDGKEIARVEW
IREGRVPLQTIQAKINYCSYMVRTTHGVLGIKIWIFIEKE
>P30056 | RR4_EPIVI
MSRYRGPSLKKIRRLGALPGLTNKRSKAENDFIKKLRSDKKSQYRIRLEEKQKLRFNYGL
RERQLRKYFSIAIKTRGSTGKVLMQLLEMRLDNIIFRLGMASTIPAARQLVNHRHVLVNG
RIVDIPSYRCKSRDIIMARDEQQSNTFINNCINYSTHNRMEAPNHLTLLHPFKGLVNQII
DSKWVGFKINELLVVEYYFRKT
>P30057 | RR7_EPIVI
MSRRGTAEEKTAKPDPIYWNRLVNMLVNRILKHGKKSLAYQIIYRALKKIQQKTEKNPLY
VLRQAIRGVTPDIAVKARRVGGSTHQVPIEIGSTQGKALAVRWLLVASKKRPGQNMAFKL
SSELVDAAKGSGDAIRKKEETHKMAEASRAFAHLR
>P30058 | RR8_EPIVI
MGRDTILEIINSIRNADRGRKRVVRITSTNITENFVKILFIEGFIENARKHREKNKYYFT
LTLRHRRNSKRPYINILNLKRISRPGLRIYSNSQQIPLILGGIGIVILYTSRGIMTDREA
RLKGIGGELLCYIW
>P30059 | RR11_EPIVI
MAKAIPITGSRRNVHVGSRKSSFRIQKGVIHVQTSFNNTIVAVTDIKGRVVSWSSAGTCG
FKGTRRGTSFAAQIAATNAIRIVQGMQRAEVMIKGPGIGRDAVLRAIRGSGVLLTFVRDV
TPMPHNGCRPPKKRRV
>P30062 | RR19_EPIVI

MIHSPTLKKNLFVANHLRAKINKLNNKKKKEIIVTWSRASTIIPIMIGHMISIHNGKEHL
PIYITDHMVGHKLGEFVPTLNFRGHAKSDNRSRR
>P30063 | CLPP_EPIVI
MPIGVPKVPFQIPGEEDASWVDVYNRLYRERLLFLGQEVDSDISNQLIGLMVYLSIEDDT
KEIYLFINSPGGWVIPGVAIYDTMQFVRPEVHTICMGLAASMGSFLLVGGEITKRLAFPH
ARVMIHQPASYFFGAQTGEFILEAEELLKLRETLTRVYVQRTGKPLWVISEDMERDVFMS
AKDAQAYGIVDLVAVE
>P30064 | ACCD_EPIVI
MERWRFSLMVFNNKLKHEYGIKKSLDSVSSIENTIENKDSNSKPNIKGRAQNIRSCGGRD
NYSYNNIIYLFGVEYNRNLIPNDTFLFRDNKGDSFYISFNIENHIFEIDINFSCLSELEI
ERYFYSYHGFDYFNFNNGSAIEDYLYNHYMYDTPSNNHITSCIESYLQSQICVNPSIISD
SSDSYIFGCFDKRRTHNESRRSGIRNRAQSSYLTIRESFNNLDSTKKYKHLWVQCENCYG
LNYKKFLKSKINLCEQCGYHFKMSSSERIEVLVDPDTWYPMDEDMSSLDPIEFHSEEEPY
KDRIYSYQKRTGLTEAVQTGLGQLNGIPIAIGVMDFQFMGGSMGSVVGEKITRLIEYATN
KILPLIIVCASGGARMQEGSLSLMQMAKISSALFDYQSNKKLLYVSILTSPTTGGVTASF
GMLGDIIIAEPNSYIAFAGKRVIEQTLHKIVPEGSQAAEYLFQKGLFDLIIPRNLLKSVL
GELFKLHAFFPLN
>P30065 | RK2_EPIVI
MAIHLYKTSTPSTRNGTVYSQVKSNPRKNLIYGQHHCGKGRNVRGIITTRHRGGGHKRLY
RKISFIRNEKYIYGRIITIEYDPNRNAYICLIHYGDGDKRYILHPRGAIIGDTLVSGTEV
PIIIGNALPLTDMPLGTAIHNIEITLGKGGQLVRAAGAVAKLIAKEGKLATLKLPSGEVR
LISKNCSATVGQVGNVGVNKKSLGRAGSKRWLGKRPVVRGVVMNPIDHPHGGGEGRAPIG
RKKPTTPWGYPALGRRSRKINKYSDNFIVRRRSK
>P30066 | RK16_EPIVI
MLSPQKTRFRKQHRGRMKGISYRGNNICFGKYGLKALEPAWITPRQIEAGRRAITRKFRR
GGKIWVRVFPDKPVTVRSSETRMGSGKGSHKYFVAVVKPGLILYEIGGVTENIAKRAILI
AASKMPMQTQFIISG
>P30071 | MATK_EPIVI
MYKQNLFIISSNDSKYNFLLRNKKLYYEIILEGLTFILEFFFFIRLISYLEGKKNKIEKL
NNLRSILSIFPFLEDNFSNLKFILDILIPRKVHAEILVQTLRYWIKDATYLHLLRFWLNN
NWNSIINQNKDGYFYPQKNKILLLLYNSYVCKYESIFVFLRNQSYHFRSTPFIMLLERIY
FYFKIERLVNPFLKVKDFKANLWLIKEPCLHYFRYRRQYILASKGTSLFLNKWKCYVITF
WQWYFSLWFFSRSIYIKKLLNNSFEILTYHSSLNINPSFVLSQILETPFISNNTIKKVNI
LVPIILSISDKEKFFNVLGHPTSNLFRVDLSDYNIIGRLECIYRNLYHYLSGSSKKNSLY
LIKYILLLSCVRTLARKHKSTVRTFAFQKKRLCLELLEGFFISEEDILFLKFQKVYSSLG
GVYISQFWYLNIYSINYLA
>P33207 | FABG_ARATH
MAAAVAAPRLISLKAVAKLGFREISQIRQLAPLHSAIPHFGMLRCRSRQPFSTSVVKAQA
TATEQSPGEVVQKVESPVVVITGASRGIGKAIALALGKAGCKVLVNYARSAKEAEEVAKQ
IEEYGGQAITFGGDVSKATDVDAMMKTALDKWGTIDVVVNNAGITRDTLLIRMKQSQWDE
VIALNLTGVFLCTQAAVKIMMKKKRGRIINISSVVGLIGNIGQANYAAAKGGVISFSKTA
AREGASRNINVNVVCPGFIASDMTAELGEDMEKKILGTIPLGRYGKAEEVAGLVEFLALS
PAASYITGQAFTIDGGIAI
>P34832 | RR7_CUSRE

MSRRGTAEKKTAKSDPIYRNRLVNMLVNRILKHGKKSLAYQIIYRAVKKIQQKTETNPLS
VLRQAIRGVTPDITVKARRVGGSTHQVPVEIGSTQGKALAVRWLLAASRKRPGRDMAFKL
SSELVDAAKGSGDAIRKKEETHRMAEANRAFAHFR
>P46292 | RR7_CUSEU
MSRRGTAEKKKAKSDPIYRNRLVNMLVNRILKHGKKSLAYQIMYRAVKTIQQNTEKNPLS
VLRQAIRGVTPDLTVKARRVSGSTHQVPIEIRSTQGKALAVRWLLAASRKRPGRDMAFKL
SSELVDAAKGRGDAIRKKEETHRMAEANRAFAHFR
>P46296 | RR12_CUSEU
MPTIKQLIRNTRQPIRKVTKSPALRSCPQRRGTCTRVYTITPKKPNSALRKVARVRLTSG
FEITAYIPGIGHNLQEHSVVLVRGGRVKDLPGVRYHIVRGTLDAVGVKDRQQGRSKYGVK
KPK
>P49162 | RK20_LATCL
MTRIKRGYIARRRRTKMRLFASSFRGAHSRLTRTITQQKIRALFSAHRDRDKQKINFRRL
WIARINAVIRKRGVSYSYSKLIHDLYKRQLLLNRKILAQIAISNRNCLYMISNEIIKETE
RKEYTGII
>Q2EEV4 | RK20_HELSJ
MTRVKRSIQRIIKKEFILKKAKGYKGGNSSLFKATKQTLLKAEKNAYRDRRYKQRLWNKI
WITRINGLLRSYNINWSVIKFFLKNNKIKLNRHILSQVSIYDPVLLQIYILFYINTLCRI

>Q2EEX1 | RR4_HELSJ
MGRYRGPRLRIIRRLGDLPLFTKKKPHIASKRLPLGHPVRKIKRRPSIYGLRLLAKQRCC
YSYGLRDYQLKNYIKKARNAQGDPIKNLIFLLESRLDSKIYRSGIVSTMAAARQLITHGH
VLVDNIKITIPSYSCNESQILDYKNTKLTSELIEKIKLSFNPIYVLEYYAIKL
>Q2EEX4 | ACCD_HELSJ
MTILAWIKDKKNKAILNTPEYSSQSSLSWCFTHKEAASNKAVSFINLSKRRALWTRCEKC
GMIQFMRFFKENANLCLSCSYHHIMTSDERIALLVEKGTWYPLNETISPKDPIKFTDTQS
YAQRIQSTQEKLGMQDAVQTGTGLINGIPFAIGIMDFRFMGGSMGSVVGEKLTRLIEYAT
KQGLFLLIVSASGGARMQEGIYSLMQMAKISAALNVYQNEANLLYISLCTSPTTGGVTAS
FAMLGDIIFSEPEAIIGFAGRRVIQQTLQQELPEDFQTSESLLHHGLIDAIVPRCFLVNA
ISEVASIFAYAPSKYKKLGNISHYHENTLSWATEEILRRNCINNKKVEYRTIEKIYQTTL
YKESFFRLNKLLSKLKSEINFTNKMKKQNNAFNTSSVYANYYDVMLCNYNIGTHSLNLLF
NEESEFCKYFPFNMDHMKKENRIKYNFITENSNDFIRKKTINDFSIMLIGD
>Q33584 | RBL_LATCL
MSPQTETKASVGFKAGVKEYKLAYYTPEYETKDTDILAAFRVTPQPGVPPEEAGAAVAAE
SSTGTWTTVWTDGLTSLDRYKGRCYHIEPVPGETDQYICYVAYPLDLFEEGSVTNMFTSI
VGNAFGFKALRALRLEDLRISPAYIKTFQGPPHGIQVERDKLNKYGRPLLGCTIKPKLGL
SAKNYGRACYECLRGGLDFTKDDENVNSQPFMRWRDRFLFCAEAIYKSQAETGEIKGHYL
NATAGTCEEMMKRAVFARELGVPIIMHDYLTGGFTANTSLAHYCRDNGLLLHIHRAMHAV
IDRQKNHGIHFRVLAKALRMSGGDHIHSGTVVGKLEGERDITLGFVDLLRDDFIEKDRSR
GIYFTQDWVSLPGVIPVASGGIHVWHMPALTEIFGDDSVLQFGGGTLGHPWGNAPGAVAN
RVALEACVKARNEGRDLAQEGNEIIHEACKWSPELAAACEVWREIVFNFAAVDVLDK
>Q49CA2 | RPOA_CUSJA
MVREKVTVSTRTLQWKCVESRTDSKRLYYGRFILSPLMKGQADTIGIALRRALLAEIEGT
RITRVKFANASHEYSTIAGIQESVHEILMNLKEIVLRSNLYGTCDASISFKGPGYVTAED

IILPPHVEIVDSTQHIAWLTEPIDLCIGLKIERNRGYFIKTHANFEDGSYPIDAVFMPVQ
NANHSIHSYGNEKQEILFLEIWTNGSLTPKEALHEASRNLIDLFIPFLHMEKENLPLEDA
DHTIPLSPFTVYYKVAKNKKKLSLESLFIDQLEFPPKIYNCLKKSNIFTLLDLLNNSQED
LIKIEHFRLEDVKQILAILGKHFALDLPKNLN
>Q49CB8 | RR4_CUSSA
MSRYRGPRLKKIRRLGALPGLTNKSPRAIRDLRNNSRSEYRIRLEEKQKLRFHYGLTEKQ
LINYVQIARKAKGSTGKVLLQLLEMRLDNILFRLGMASTIXAARQLVNHRHVLVNGRLVN
RPSYRCKPHDIIMPKNTTKSGVLVQNSLELFTGKELANHLNLFSTPYKGLVNKIVDTNWI
GLKINELLVVEYYSRQA
>Q49CC1 | RBL_CUSSA
MSPQTETKTSVGFKAGVKDYKLTYYTPYYETKATDILAAFRVTPQPGVPPEEAGAAVAAE
SSTGTWTTVWTDGLTSLDRYKGRCYHIERVFGEKDQYIAYVAYPLDLFEEGSVTNMFTSI
VGNVFGFKALRALRLEDLRIPPAYTKTFKGPPHGIQVERDKLNKYGRPLLGCTIKPKLGL
SAKNYGRAVYECLRGGLDFTKDDENVNSQPFMRWRDRFLFCAEAIYKSQAETGEIKGHYL
NATAGTCEEMLRRAYFAKELGVPIIMHDYLTGGFTANTSLAHFCRENGLLLHIHRAMHAV
IDRQKNHGIHFRVLAKALRLSGGDHIHAGTVVGKLEGEREITLGFVDLLRDNFVEKDRSR
GIYFTQDWVSLPGVLPVASGGIHVWHMPALTDIFGDDSVLQFGGGTLGHPWGNAPGAVAN
RVALEACVQARNEGLDLAQDGNSIIRQASNWSPELAAACEVWKEIQFNFKSVDTLDLNEI
K
>Q5I6K6 | MATK_BARAL
MEEIRRYLQLERSQQHDFLYPLIFQEYIYAFAHDRGFSRSILLENENPGYDNKSSLLVMK
RLITRMYQQNHFLISPNDFNQKNPFFAHNKNLYSQIIAEGFAFIVEIPFSLRLISEGKKK
KIVKSQNLRSIHSIFPFLEDNFSYLNFVLDILIPHPVHVEILVQTLRYWVKDASSLHLLR
FFLNKYWNSLITPKKASSSFSTRNQRLFVFLYNSHVSEYESSFVFLRNQSSHLGSTPFGV
LLERIYFYGKIERLVNVFVKVKDFRANLWLVKEPCIHYIRYQRKFILASKGTSLFMNKWK
CYLITFWQWHFSLWFYPRRIYINQLSNHSFAFLGYLSSLRMNPSVVRSQSLENAFLINNA
IKKVDTLVPIIPMIASLAKAKFCNVFGHPISKPVRADLSDSNIIDRFGCICRNFSHYYSG
SSKKKSLYRIKYILRLSCARTLARKHKSTVRTFLKKLGSELLEEFLLSEEDVLFLTFPKA
SPSLQGVYRSRIWYLDIISINDLVDHKSKF
>Q6RH25 | RR7_HELSJ
MKNLIKNKIKLSTYNITLIEHFIRFLIKGWKKKKAINLLSLSLNYIALTINKDPISTLEM
AVRYTIPVYLEVTPSIITNENKTLWHKRFICKSSKVRTFQAIKWIIKAAKEKKDKSLYIH
LAKEIVDASRNSGKAVKYKEQMENKLKLAQLHM
>Q6RH26 | RR12_HELSJ
MPTLQQLVRKGRIQKHTKTKTPALNNSPQKRGICIRSYTITPKKPNSALRKVARVRLTSK
LEITAYIPGIGHNIQEHSVLLIRGGRVKDLPGVRYQIIRGTRDVSGVTARRQSHSKYGTK
KVKN
>Q8SKY1 | RPOA_CUSRE
MVREKVTVSTRTLQWKCVESRTDSKRLYYGRFILSPLTKGQADTIGIAMRRALLAEIEGT
RITRVKFANTSHEYSTIAGIQESVHEILMNLKEIVLRSNLYGTCDASISIKGPGYVTAED
IILPPHVEIVDSTQHIAWLTEPINFFIGLKIERNHGYFIKTHANFEDGSYPIDALFMPVR
NANHSINSYGNEKQEILFLEIWTNGSLTPKEALHEASRNLIDLFIPFLHMEEENLHLEDA
DHTIPLSPFTVYDKVAKLRKNKKKLSLESIFIDQLEFPPKIYNCLKKSNIFTLLDLLNNS
QEDLIKIEHFHLEDVKQILGILGKHFALDLPKNLN

>Q9TIS6 | MATK_CASLN
MEEIQRYLQLERSQQHDFLYPLIFQEYIYTFAHDRGFGRSILSENPGYDNKYSLLIVKRL
ITRMYQQNHLIISPNDSNQNQFLGRNKNLYSQIISEGFAFIVEIPFSLRLISCLEGKNKK
IIKSQNLRSILSIFPFLEDNFSHLNLVLDILIPHPVHGEILVQTLRYWVKDASSLHLLRF
FLNKNWNSLITPKKASSSFLKRNQRLFLFLYNSHVCEYESVFVFLRNQSSHLRSTPFGVF
LERIYFYGKIERLVNVFVKVKDFQANLWLVKEPCIHYIRYQRKAILASKGTSLFMNKWKC
YLITFWQWHFSLWFYPRRIYINQLSNHSFEFLGYQSSLRMNPSVVRSQILENSFLINNAI
KKVDTFIPIIPLIVSLAKAKFCNVXGHPISKPVRADLSDSNIIDRFGCICRNFSHYHSGS
SKKKSLYRIKYILRLSCARTLARKHKTTVRTFLKRLGSELLEEFLLSEEDVLFLTFPKAS
SSLQGVYRNRIWYLDIISINDLADHKSKL
>Q9TJQ5 | RK2_PROWI
MAIRFFKPTTPGRRHGSVLNFDDLSSKKPEKKLTRGWSRAQGRNNKGRITTRHRGGGHKR
LYREIDFIRNKIGTSAIVKSIEYDPNRTARIALVCYKDGEKRYILSPTGLKVGEMVIASP
NAPITVGNTLPLYNIPLGTSVHNVELQPGAGGQLVRSAGSVAQIVAKEGQWATLHLPSGE
YRLIPQKCWATVGRVGNLDNSNITLGKAGRSRWLSQRPHVRGSAMNPVDHPHGGGEGKAP
IGRARPVSLWGKPALGVKTRKRKKFSNNLIINL
>Q9TJQ8 | EFTU_PROWI
MARAKFERKKPHVNIGTIGHVDHGKTTLTAAITMALAARGGGKGKKYAEIDSAPEEKARG
ITINTAHVEYETESRHYAHVDCPGHADYVKNMITGAAQMDGAILVVSGADGPMPQTKEHI
LLAKQVGVPNIVVFINKEDQVDDIELIELVELEVRETLQRYDFPGDEVPMIPGSALMALT
ALTDNPKIKPGENKWVDKIYNLMDIVDSYIPTPKRNIEKPFLMAIEDVFSITGRGTVATG
RVERGVVKIGDSVEIVGLGATKITTVTGLEMFQKTLDESIAGDNVGILLRGIQKTEIQRG
MVLAKPKSITPHTNFEAQVYVLNKEEGGRDTPFFSGYRPQFYVRTTDVTGKIESFCTDAG
EPIKMVLPGDRIKMKAELIQPIAIERNMRFAIREGGKTVGAGVVGKILK
>Q9TJR0 | RR12_PROWI
MPTFQQLVRSARKPHAKKTKSPALQGCPQRRGVCTRVYTTTPKKPNSALRKVARVRLSSK
FEITAYIPGIGHNLQEHSVVLVRGGRVKDLPGVRYHIVRGSLDSIGVKNRNQSRSKYGVK
RPKS
>Q9TJR1 | RK20_PROWI
MTRVKRGNIARNRRNEILDLAKGFRGSSSKLYRTAQQRTIKALTNSYKDRKNKKREFVKI
WVSRINAAVRLSGLNYSSFQNQLKFHKIRLNRKICSQIALQDQESFTKLLDLII
