# Supplementary material for: Plant-mPLoc: A Top-Down Strategy to Augment the Power for Predicting Plant Protein Subcellular Localization
Source: PLoS One. 2010 Jun 28;5(6):e11335. doi: 10.1371/journal.pone.0011335 (PMC2893129; doi:10.1371/journal.pone.0011335)
Supplement: Table S5 — List of the results predicted by Predotar (Small et al., Proteomics 2004, 4:1581–90) and Plant-mPLoc on the 381 independent proteins in the Table S4, and their experimental subcellular locations as annotated in Swiss-Prot databank (version 55.3 released on 29-Apr-2008). Note for the Predotar output, “ER” means “Endoplasmic reticulum”. (0.16 MB PDF) [file pone.0011335.s005.pdf]

Table S5. List of the results predicted by Predotar (Small et al., Proteomics 2004, 4:1581-90) and Plant-mPLoc on the 381 independent proteins in the **Table S4**, and their experimental subcellular locations as annotated in Swiss-Prot databank (version 55.3 released on 29-Apr-2008). Note for the Predotar output, "ER" means "Endoplasmic reticulum".

Protein accession number	Predotar	Plant-mPLoc	Experimental result annotated in Swiss-Prot database
A2X8W3	Unable to identify	Endoplasmic reticulum.	Endoplasmic reticulum.
O24581	ER	Endoplasmic reticulum. Nucleus.	Endoplasmic reticulum.
O24594	Unable to identify	Endoplasmic reticulum.	Endoplasmic reticulum.
O64966	Unable to identify	Endoplasmic reticulum.	Endoplasmic reticulum.
O81108	Unable to identify	Chloroplast. Endoplasmic reticulum. Vacuole.	Endoplasmic reticulum.
P0C5E5	ER	Vacuole.	Vacuole.
P14891	Unable to identify	Endoplasmic reticulum.	Vacuole (directly from the Endoplasmic reticulum).
P20698	ER	Endoplasmic reticulum. Vacuole.	Endoplasmic reticulum.
P24067	ER	Endoplasmic reticulum. Nucleus.	Endoplasmic reticulum.
P25803	ER	Endoplasmic reticulum.	Endoplasmic reticulum.
P29057	Unable to identify	Endoplasmic reticulum.	Vacuole (directly from Endoplasmic reticulum).
P32291	Unable to identify	Chloroplast. Endoplasmic	Vacuole (directly from the Endoplasmic

		reticulum.	reticulum).
P33490	ER	Endoplasmic reticulum.	Endoplasmic reticulum.
P33522	ER	Endoplasmic reticulum.	Endoplasmic reticulum.
P37116	Unable to identify	Endoplasmic reticulum.	Endoplasmic reticulum.
P43256	Unable to identify	Endoplasmic reticulum.	Endoplasmic reticulum.
P46313	Unable to identify	Endoplasmic reticulum.	Endoplasmic reticulum.
P47195	ER	Endoplasmic reticulum.	Endoplasmic reticulum.
P48020	Unable to identify	Endoplasmic reticulum.	Endoplasmic reticulum.
P48021	Unable to identify	Endoplasmic reticulum.	Endoplasmic reticulum.
P48022	Unable to identify	Endoplasmic reticulum.	Endoplasmic reticulum.
P48623	Unable to identify	Chloroplast. Endoplasmic reticulum.	Endoplasmic reticulum.
P48624	Unable to identify	Chloroplast. Endoplasmic reticulum.	Endoplasmic reticulum.
P48625	Unable to identify	Chloroplast. Endoplasmic reticulum.	Endoplasmic reticulum.
P48626	Unable to identify	Chloroplast. Endoplasmic reticulum.	Endoplasmic reticulum.
P48631	Unable to identify	Endoplasmic reticulum.	Endoplasmic reticulum.
P49118	ER	Endoplasmic	Endoplasmic

		reticulum. Nucleus.	reticulum.
Q00583	Unable to identify	Endoplasmic reticulum.	Endoplasmic reticulum.
Q01559	Unable to identify	Endoplasmic reticulum.	Endoplasmic reticulum.
Q03163	Unable to identify	Endoplasmic reticulum.	Endoplasmic reticulum.
Q05JG2	ER	Endoplasmic reticulum.	Endoplasmic reticulum.
Q0DJ45	ER	Vacuole.	Endoplasmic reticulum.
Q0DN94	ER	Endoplasmic reticulum. Vacuole.	Endoplasmic reticulum.
Q0DY59	Unable to identify	Endoplasmic reticulum.	Endoplasmic reticulum.
Q39287	Unable to identify	Endoplasmic reticulum.	Endoplasmic reticulum.
Q41437	Unable to identify	Endoplasmic reticulum.	Endoplasmic reticulum.
Q41438	Unable to identify	Endoplasmic reticulum.	Endoplasmic reticulum.
Q42434	ER	Endoplasmic reticulum. Nucleus.	Endoplasmic reticulum.
Q4G2J4	Unable to identify	Endoplasmic reticulum.	Endoplasmic reticulum.
Q4G2J5	Unable to identify	Endoplasmic reticulum.	Endoplasmic reticulum.
Q4G2J6	Unable to identify	Endoplasmic reticulum.	Endoplasmic reticulum.
Q9C5Y2	ER	Endoplasmic reticulum.	Endoplasmic reticulum.
Q9C7S7	Unable to identify	Endoplasmic reticulum.	Endoplasmic reticulum.

Q9FUJ3	ER	Endoplasmic reticulum.	Endoplasmic reticulum.
Q9FUY7	ER	Endoplasmic reticulum.	Endoplasmic reticulum. Extracellular.
Q9XEL8	Unable to identify	Endoplasmic reticulum.	Endoplasmic reticulum.
Q9XHL5	Unable to identify	Endoplasmic reticulum.	Endoplasmic reticulum.
Q9ZTN2	Unable to identify	Endoplasmic reticulum.	Endoplasmic reticulum.
A2Y4S9	Unable to identify	Mitochondrion.	Mitochondrion.
O05000	ER	Mitochondrion.	Mitochondrion.
O22642	Unable to identify	Mitochondrion.	Mitochondrion.
O23936	Possibly Mitochondrion	Cytoplasm. Mitochondrion.	Mitochondrion.
O49850	Mitochondrion	Mitochondrion.	Mitochondrion.
O64966	Unable to identify	Endoplasmic reticulum.	Endoplasmic reticulum.
O81235	Mitochondrion	Mitochondrion.	Mitochondrion.
O81796	Mitochondrion	Mitochondrion.	Mitochondrion.
P00051	Unable to identify	Mitochondrion.	Mitochondrion.
P00052	Unable to identify	Mitochondrion.	Mitochondrion.
P00053	Unable to identify	Mitochondrion.	Mitochondrion.
P00054	Unable to identify	Mitochondrion.	Mitochondrion.
P00056	Unable to identify	Mitochondrion.	Mitochondrion.
P00057	Unable to identify	Mitochondrion.	Mitochondrion.
P00058	Unable to identify	Mitochondrion.	Mitochondrion.
P00059	Unable to identify	Mitochondrion.	Mitochondrion.
P00060	Unable to identify	Mitochondrion.	Mitochondrion.

P00061	Unable to identify	Mitochondrion.	Mitochondrion.
P00062	Unable to identify	Mitochondrion.	Mitochondrion.
P00063	Unable to identify	Mitochondrion.	Mitochondrion.
P00064	Unable to identify	Mitochondrion.	Mitochondrion.
P00065	Unable to identify	Mitochondrion.	Mitochondrion.
P00066	Unable to identify	Mitochondrion.	Mitochondrion.
P00067	Unable to identify	Mitochondrion.	Mitochondrion.
P00068	Unable to identify	Mitochondrion.	Mitochondrion.
P00069	Unable to identify	Mitochondrion.	Mitochondrion.
P00070	Unable to identify	Mitochondrion.	Mitochondrion.
P00071	Unable to identify	Mitochondrion.	Mitochondrion.
P00072	Unable to identify	Mitochondrion.	Mitochondrion.
P00073	Unable to identify	Mitochondrion.	Mitochondrion.
P00074	Unable to identify	Mitochondrion.	Mitochondrion.
P00075	Unable to identify	Mitochondrion.	Mitochondrion.
P00412	Possibly ER	Mitochondrion.	Mitochondrion.
P00413	Possibly ER	Mitochondrion.	Mitochondrion.
P04373	Unable to identify	Mitochondrion.	Mitochondrion.
P05488	Unable to identify	Mitochondrion.	Mitochondrion.
P05490	Unable to identify	Mitochondrion.	Mitochondrion.
P05491	Possibly ER	Mitochondrion.	Mitochondrion.
P05492	Unable to identify	Mitochondrion.	Mitochondrion.
P05493	Unable to identify	Mitochondrion.	Mitochondrion.
P05494	Unable to identify	Mitochondrion.	Mitochondrion.
P05495	Unable to identify	Mitochondrion.	Mitochondrion.
P05500	Unable to identify	Mitochondrion.	Mitochondrion.

P07506	Unable to identify	Mitochondrion.	Mitochondrion.
P07924	Unable to identify	Mitochondrion.	Mitochondrion.
P07925	Unable to identify	Mitochondrion.	Mitochondrion.
P08681	ER	Mitochondrion.	Mitochondrion.
P08742	Possibly mitochondrion	Mitochondrion.	Mitochondrion.
P08743	Unable to identify	Mitochondrion.	Mitochondrion.
P08744	Possibly ER	Mitochondrion.	Mitochondrion.
P08977	Unable to identify	Mitochondrion.	Mitochondrion.
P0C520	Unable to identify	Mitochondrion.	Mitochondrion.
P0C521	Unable to identify	Mitochondrion.	Mitochondrion.
P0C522	Unable to identify	Mitochondrion.	Mitochondrion.
P12786	Unable to identify	Mitochondrion.	Mitochondrion.
P12857	Unable to identify	Mitochondrion.	Mitochondrion.
P12862	Unable to identify	Mitochondrion.	Mitochondrion.
P14578	Possibly mitochondrion	Mitochondrion.	Mitochondrion.
P14875	Unable to identify	Mitochondrion.	Mitochondrion.
P15451	Unable to identify	Mitochondrion.	Mitochondrion.
P15758	Unable to identify	Mitochondrion.	Mitochondrion.
P16048	Mitochondrion	Mitochondrion.	Mitochondrion.
P16265	ER	Mitochondrion.	Mitochondrion.
P17614	Mitochondrion	Chloroplast. Mitochondrion.	Mitochondrion.
P18260	Unable to identify	Mitochondrion.	Mitochondrion.
P18630	ER	Mitochondrion.	Mitochondrion.
P20113	ER	Mitochondrion.	Mitochondrion.

P22201	Unable to identify	Mitochondrion.	Mitochondrion.
P23209	Mitochondrion	Mitochondrion.	Mitochondrion.
P24459	Unable to identify	Mitochondrion.	Mitochondrion.
P24794	Possibly mitochondrion	Mitochondrion.	Mitochondrion.
P25083	Unable to identify	Mitochondrion.	Mitochondrion.
P25855	Mitochondrion	Mitochondrion.	Mitochondrion.
P26846	ER	Mitochondrion.	Mitochondrion.
P26847	ER	Mitochondrion.	Mitochondrion.
P26848	ER	Mitochondrion.	Mitochondrion.
P26850	ER	Mitochondrion.	Mitochondrion.
P26853	Unable to identify	Mitochondrion.	Mitochondrion.
P26856	Unable to identify	Mitochondrion.	Mitochondrion.
P26857	ER	Mitochondrion.	Mitochondrion.
P26859	Possibly mitochondrion	Mitochondrion.	Mitochondrion.
P26860	Unable to identify	Mitochondrion.	Mitochondrion.
P26861	Possibly ER	Mitochondrion.	Mitochondrion.
P26862	Possibly mitochondrion	Chloroplast.	Mitochondrion.
P26864	Possibly mitochondrion	Mitochondrion.	Mitochondrion.
P26865	Unable to identify	Mitochondrion.	Mitochondrion.
P26866	Possibly mitochondrion	Chloroplast. Mitochondrion.	Mitochondrion.
P26869	Possibly mitochondrion	Mitochondrion.	Mitochondrion.
P26870	Unable to identify	Mitochondrion.	Mitochondrion.
P26871	Unable to identify	Chloroplast.	Mitochondrion.

P26872	Unable to identify	Mitochondrion.	Mitochondrion.
P26873	Unable to identify	Mitochondrion.	Mitochondrion.
P26874	Unable to identify	Chloroplast.	Mitochondrion.
P27062	ER	Mitochondrion.	Mitochondrion.
P27070	Unable to identify	Mitochondrion.	Mitochondrion.
P27080	Unable to identify	Mitochondrion.	Mitochondrion.
P27084	Mitochondrion	Mitochondrion.	Mitochondrion.
P27527	Mitochondrion	Chloroplast.	Mitochondrion.
P27572	Unable to identify	Mitochondrion.	Mitochondrion.
P27754	Unable to identify	Mitochondrion.	Mitochondrion.
P27928	Unable to identify	Mitochondrion.	Mitochondrion.
P28520	Unable to identify	Chloroplast.	Mitochondrion.
P29057	Unable to identify	Endoplasmic reticulum.	Endoplasmic reticulum.
P29185	Mitochondrion	Mitochondrion.	Mitochondrion.
P29380	Unable to identify	Mitochondrion.	Mitochondrion.
P29677	Mitochondrion	Mitochondrion.	Mitochondrion.
P29685	Mitochondrion	Chloroplast. Mitochondrion.	Mitochondrion.
P31167	Unable to identify	Mitochondrion.	Mitochondrion.
P31691	Unable to identify	Mitochondrion.	Mitochondrion.
P31692	Unable to identify	Mitochondrion.	Mitochondrion.
P35017	Mitochondrion	Mitochondrion.	Mitochondrion.
P37399	Mitochondrion	Chloroplast. Mitochondrion.	Mitochondrion.
P37900	Mitochondrion	Mitochondrion.	Mitochondrion.
P41978	Mitochondrion	Mitochondrion.	Mitochondrion.

P41979	Mitochondrion	Mitochondrion.	Mitochondrion.
P41980	Mitochondrion	Mitochondrion.	Mitochondrion.
P42027	Mitochondrion	Mitochondrion.	Mitochondrion.
P42056	Possibly mitochondrion	Mitochondrion.	Mitochondrion.
P42793	Unable to identify	Mitochondrion.	Mitochondrion.
P46274	Unable to identify	Mitochondrion.	Mitochondrion.
P46485	Mitochondrion	Mitochondrion.	Mitochondrion.
P46740	Unable to identify	Mitochondrion.	Mitochondrion.
P46742	Unable to identify	Mitochondrion.	Mitochondrion.
P46744	Possibly mitochondrion	Chloroplast.	Mitochondrion.
P46745	Unable to identify	Mitochondrion.	Mitochondrion.
P46747	Unable to identify	Mitochondrion.	Mitochondrion.
P46750	Unable to identify	Chloroplast. Mitochondrion.	Mitochondrion.
P46751	Possibly mitochondrion	Chloroplast.	Mitochondrion.
P46752	Unable to identify	Mitochondrion.	Mitochondrion.
P46773	Unable to identify	Mitochondrion.	Mitochondrion.
P46801	Possibly mitochondrion	Mitochondrion.	Mitochondrion.
P48857	Unable to identify	Chloroplast.	Mitochondrion.
P49357	Mitochondrion	Mitochondrion.	Mitochondrion.
P49358	Mitochondrion	Mitochondrion.	Mitochondrion.
P49359	Mitochondrion	Mitochondrion.	Mitochondrion.
P49361	Possibly mitochondrion	Mitochondrion.	Mitochondrion.
P49362	Mitochondrion	Mitochondrion.	Mitochondrion.

P49363	Possibly mitochondrion	Cytoplasm. Mitochondrion.	Mitochondrion.
P49386	Unable to identify	Mitochondrion.	Mitochondrion.
P49387	Unable to identify	Mitochondrion.	Mitochondrion.
P49388	Unable to identify	Mitochondrion.	Mitochondrion.
P49389	Possibly mitochondrion	Mitochondrion.	Mitochondrion.
P50433	Possibly mitochondrion	Mitochondrion.	Mitochondrion.
P50892	Unable to identify	Chloroplast.	Mitochondrion.
P50893	Possibly mitochondrion	Chloroplast.	Mitochondrion.
P51132	Mitochondrion	Mitochondrion.	Mitochondrion.
P51135	Mitochondrion	Mitochondrion.	Mitochondrion.
P51409	Unable to identify	Mitochondrion.	Mitochondrion.
P52901	Mitochondrion	Mitochondrion.	Mitochondrion.
P54260	Possibly mitochondrion	Cytoplasm. Mitochondrion.	Mitochondrion.
P60096	Unable to identify	Mitochondrion.	Mitochondrion.
P60097	Unable to identify	Mitochondrion.	Mitochondrion.
P60098	Unable to identify	Chloroplast.	Mitochondrion.
P60099	Unable to identify	Chloroplast.	Mitochondrion.
P60159	ER	Mitochondrion.	Mitochondrion.
P60160	ER	Mitochondrion.	Mitochondrion.
P60621	Possibly mitochondrion	Mitochondrion.	Mitochondrion.
P62772	Unable to identify	Mitochondrion.	Mitochondrion.
P62773	Unable to identify	Mitochondrion.	Mitochondrion.
P68526	Unable to identify	Mitochondrion.	Mitochondrion.

P68527	Unable to identify	Mitochondrion.	Mitochondrion.
P68535	Unable to identify	Chloroplast.	Mitochondrion.
P68536	Unable to identify	Chloroplast.	Mitochondrion.
P68539	Possibly mitochondrion	Mitochondrion.	Mitochondrion.
P68540	Possibly mitochondrion	Mitochondrion.	Mitochondrion.
P68541	Unable to identify	Mitochondrion.	Mitochondrion.
P68542	Unable to identify	Mitochondrion.	Mitochondrion.
P80261	Possibly plastid	Mitochondrion.	Mitochondrion.
P83372	Mitochondrion	Cytoplasm. Mitochondrion.	Mitochondrion.
P83373	Mitochondrion	Chloroplast. Mitochondrion.	Mitochondrion.
P83483	Mitochondrion	Chloroplast. Mitochondrion.	Mitochondrion.
P83484	Mitochondrion	Chloroplast. Mitochondrion.	Mitochondrion.
P92532	Unable to identify	Chloroplast.	Mitochondrion.
P92547	Unable to identify	Mitochondrion.	Mitochondrion.
P92549	Unable to identify	Mitochondrion.	Mitochondrion.
P92557	Unable to identify	Chloroplast. Mitochondrion.	Mitochondrion.
P92969	Mitochondrion	Chloroplast. Mitochondrion. Plastid.	Mitochondrion.
P93032	Possibly mitochondrion	Mitochondrion.	Mitochondrion.
P93285	Unable to identify	Mitochondrion.	Mitochondrion.
P93298	Unable to identify	Mitochondrion.	Mitochondrion.

P93306	Unable to identify	Mitochondrion.	Mitochondrion.
P93311	Mitochondrion	Mitochondrion.	Mitochondrion.
P93313	Unable to identify	Mitochondrion.	Mitochondrion.
P93401	ER	Mitochondrion.	Mitochondrion.
P98012	Unable to identify	Mitochondrion.	Mitochondrion.
Q00583	Unable to identify	Endoplasmic reticulum.	Endoplasmic reticulum.
Q01859	Mitochondrion	Mitochondrion.	Mitochondrion.
Q01902	Unable to identify	Chloroplast. Mitochondrion.	Mitochondrion.
Q01915	Unable to identify	Mitochondrion.	Mitochondrion.
Q04050	Unable to identify	Mitochondrion.	Mitochondrion.
Q04654	Possibly ER	Mitochondrion.	Mitochondrion.
Q04715	Mitochondrion	Mitochondrion.	Mitochondrion.
Q05143	Possibly mitochondrion	Mitochondrion.	Mitochondrion.
Q06735	Unable to identify	Mitochondrion.	Mitochondrion.
Q0DI31	Unable to identify	Mitochondrion.	Mitochondrion.
Q31708	Mitochondrion	Mitochondrion.	Mitochondrion.
Q31720	Unable to identify	Mitochondrion.	Mitochondrion.
Q33994	Unable to identify	Mitochondrion.	Mitochondrion.
Q34011	Unable to identify	Mitochondrion.	Mitochondrion.
Q35322	Possibly plastid	Extracell. Mitochondrion.	Mitochondrion.
Q36450	Unable to identify	Mitochondrion.	Mitochondrion.
Q36518	ER	Mitochondrion.	Mitochondrion.
Q36664	ER	Mitochondrion.	Mitochondrion.
Q36665	Unable to identify	Chloroplast.	Mitochondrion.

Q37625	ER	Mitochondrion.	Mitochondrion.
Q37626	ER	Mitochondrion.	Mitochondrion.
Q37627	ER	Mitochondrion.	Mitochondrion.
Q37787	Unable to identify	Mitochondrion.	Mitochondrion.
Q41346	Unable to identify	Mitochondrion. Nucleus.	Mitochondrion.
Q41629	Unable to identify	Mitochondrion.	Mitochondrion.
Q41630	Possibly plastid	Mitochondrion.	Mitochondrion.
Q41898	Unable to identify	Mitochondrion.	Mitochondrion.
Q42525	Unable to identify	Chloroplast. Mitochondrion.	Mitochondrion.
Q42560	Unable to identify	Cytoplasm. Mitochondrion.	Cytoplasm. Mitochondrion.
Q42777	Mitochondrion	Mitochondrion.	Mitochondrion.
Q43008	Mitochondrion	Mitochondrion.	Mitochondrion.
Q56XE8	ER	Mitochondrion.	Mitochondrion.
Q5M729	Possibly mitochondrion	Mitochondrion.	Mitochondrion.
Q5YLB5	Possibly plastid	Chloroplast. Mitochondrion.	Chloroplast. Mitochondrion.
Q6K548	Unable to identify	Mitochondrion.	Mitochondrion.
Q8H1Y0	Mitochondrion	Mitochondrion.	Mitochondrion.
Q8L6J5	Possibly mitochondrion	Chloroplast. Mitochondrion. Plastid.	Mitochondrion.
Q8L7B5	Mitochondrion	Mitochondrion.	Mitochondrion.
Q8LAD2	Possibly mitochondrion	Mitochondrion.	Mitochondrion.
Q8LBZ7	Mitochondrion	Cytoplasm. Mitochondrion.	Mitochondrion.

Q8LFC0	Mitochondrion	Mitochondrion.	Mitochondrion.
Q8LFT2	Unable to identify	Mitochondrion.	Mitochondrion.
Q8LPW2	Possibly mitochondrion	Mitochondrion.	Mitochondrion.
Q8RWN9	Possibly mitochondrion	Mitochondrion.	Mitochondrion.
Q8VWF8	Unable to identify	Chloroplast. Mitochondrion. Plastid.	Chloroplast. Mitochondrion.
Q93Y94	Possibly mitochondrion	Chloroplast. Mitochondrion. Plastid.	Mitochondrion.
Q93ZM7	Mitochondrion	Mitochondrion.	Mitochondrion.
Q945K7	Possibly mitochondrion	Chloroplast. Mitochondrion.	Mitochondrion.
Q94B78	Mitochondrion	Mitochondrion.	Mitochondrion.
Q95747	ER	Mitochondrion.	Mitochondrion.
Q95748	Possibly plastid	Mitochondrion.	Mitochondrion.
Q95749	Unable to identify	Mitochondrion.	Mitochondrion.
Q95869	Unable to identify	Chloroplast.	Mitochondrion.
Q96007	ER	Mitochondrion.	Mitochondrion.
Q96008	Unable to identify	Chloroplast. Mitochondrion.	Mitochondrion.
Q96033	Unable to identify	Chloroplast.	Mitochondrion.
Q96253	Unable to identify	Mitochondrion.	Mitochondrion.
Q9C641	Mitochondrion	Mitochondrion.	Mitochondrion.
Q9FMV1	Mitochondrion	Mitochondrion.	Mitochondrion.
Q9FV51	Unable to identify	Chloroplast. Mitochondrion.	Chloroplast. Mitochondrion.
Q9LFV6	Possibly plastid	Chloroplast. Mitochondrion.	Chloroplast.

		Plastid.	Mitochondrion.
Q9LJL3	Mitochondrion	Chloroplast. Mitochondrion.	Chloroplast. Mitochondrion.
Q9LKA3	Mitochondrion	Chloroplast. Mitochondrion.	Mitochondrion.
Q9LPS1	ER	Mitochondrion.	Mitochondrion.
Q9M1D3	Unable to identify	Cytoplasm. Mitochondrion.	Mitochondrion.
Q9M5K2	Mitochondrion	Cytoplasm. Mitochondrion.	Mitochondrion.
Q9M5K3	Mitochondrion	Cytoplasm. Mitochondrion.	Mitochondrion.
Q9MF82	Unable to identify	Chloroplast. Mitochondrion.	Mitochondrion.
Q9SIB9	plastid	Cytoplasm. Mitochondrion.	Mitochondrion.
Q9SMX3	Possibly mitochondrion	Mitochondrion.	Mitochondrion.
Q9SRH5	Possibly mitochondrion	Mitochondrion.	Mitochondrion.
Q9SVM8	Possibly mitochondrion	Mitochondrion.	Mitochondrion.
Q9SZJ5	Mitochondrion	Mitochondrion.	Mitochondrion.
Q9TC96	Unable to identify	Mitochondrion.	Mitochondrion.
Q9XGY5	Unable to identify	Mitochondrion.	Mitochondrion.
Q9ZP06	Mitochondrion	Mitochondrion.	Mitochondrion.
Q9ZPX5	Possibly mitochondrion	Mitochondrion.	Mitochondrion.
Q9ZT91	Possibly mitochondrion	Mitochondrion.	Mitochondrion.
A2T833	Mitochondrion	Chloroplast.	Plastid.

A7M8Z3	Unable to identify	Chloroplast.	Plastid.
A7M8Z9	Unable to identify	Chloroplast. Plastid.	Plastid.
A7M903	Mitochondrion	Chloroplast.	Plastid.
A7M907	Unable to identify	Chloroplast.	Plastid.
A7M920	Mitochondrion	Chloroplast.	Plastid.
A7M922	Unable to identify	Chloroplast.	Plastid.
A7M929	Mitochondrion	Chloroplast.	Plastid.
A7M931	Unable to identify	Chloroplast.	Plastid.
A7M937	Possibly plastid	Chloroplast.	Plastid.
A7M939	Unable to identify	Mitochondrion. Plastid.	Plastid.
A7M955	Unable to identify	Chloroplast.	Plastid.
A7M957	Unable to identify	Chloroplast. Nucleus. Plastid.	Plastid.
A7M958	Unable to identify	Chloroplast. Plastid.	Plastid.
A7M964	Unable to identify	Chloroplast. Plastid.	Plastid.
A7M968	Mitochondrion	Chloroplast.	Plastid.
A7M986	Mitochondrion	Chloroplast.	Plastid.
A7M996	Unable to identify	Chloroplast.	Plastid.
A7M998	Unable to identify	Chloroplast.	Plastid.
A7M9A1	Unable to identify	Chloroplast.	Plastid.
A7M9A4	Possibly mitochondrion	Chloroplast. Plastid.	Plastid.
A8W3B6	Unable to identify	Chloroplast. Plastid.	Plastid.
A8W3C6	Mitochondrion	Chloroplast.	Plastid.
A8W3F4	Unable to identify	Chloroplast.	Plastid.
A8W3F6	Unable to identify	Chloroplast.	Plastid.

A8W3F9	Unable to identify	Chloroplast.	Plastid.
A8W3I9	Mitochondrion	Chloroplast.	Plastid.
A8W3L5	Mitochondrion	Chloroplast.	Plastid.
A8W3L7	Unable to identify	Chloroplast.	Plastid.
A8W3M0	Unable to identify	Chloroplast.	Plastid.
O63057	Unable to identify	Mitochondrion. Plastid.	Plastid.
O78678	Mitochondrion	Chloroplast.	Plastid.
O99010	Unable to identify	Chloroplast.	Plastid.
P19975	plastid	Chloroplast.	Chloroplast. Plastid.
P19976	plastid	Chloroplast.	Chloroplast. Plastid.
P27071	Unable to identify	Chloroplast. Plastid.	Plastid.
P29036	Mitochondrion	Chloroplast.	Chloroplast. Plastid.
P29390	Mitochondrion	Chloroplast.	Chloroplast. Plastid.
P30055	Unable to identify	Chloroplast.	Plastid.
P30056	Possibly mitochondrion	Chloroplast. Plastid.	Plastid.
P30057	Unable to identify	Mitochondrion. Plastid.	Plastid.
P30058	Unable to identify	Chloroplast.	Plastid.
P30059	Unable to identify	Chloroplast.	Plastid.
P30062	Mitochondrion	Chloroplast.	Plastid.
P30063	Unable to identify	Chloroplast.	Plastid.
P30064	Unable to identify	Chloroplast.	Plastid.
P30065	Possibly plastid	Chloroplast. Plastid.	Plastid.
P30066	Possibly mitochondrion	Chloroplast. Plastid.	Plastid.
P30071	Unable to identify	Chloroplast.	Plastid.

P33207	Mitochondrion	Chloroplast.	Chloroplast. Plastid.
P34832	Unable to identify	Mitochondrion.	Plastid.
P46292	Unable to identify	Mitochondrion. Plastid.	Plastid.
P46296	Unable to identify	Chloroplast.	Plastid.
P49162	Mitochondrion	Chloroplast.	Plastid.
Q2EEV4	Possibly mitochondrion	Plastid.	Plastid.
Q2EEX1	Possibly mitochondrion	Mitochondrion. Plastid.	Plastid.
Q2EEX4	Unable to identify	Chloroplast.	Plastid.
Q33584	Unable to identify	Chloroplast. Plastid.	Plastid.
Q49CA2	Unable to identify	Chloroplast. Plastid.	Plastid.
Q49CB8	Mitochondrion	Chloroplast.	Plastid.
Q49CC1	Unable to identify	Chloroplast. Plastid.	Plastid.
Q5I6K6	Unable to identify	Chloroplast.	Plastid.
Q6RH25	Unable to identify	Mitochondrion. Plastid.	Plastid.
Q6RH26	Unable to identify	Chloroplast.	Plastid.
Q8SKY1	Unable to identify	Chloroplast. Plastid.	Plastid.
Q9TIS6	Unable to identify	Chloroplast.	Plastid.
Q9TJQ5	Mitochondrion	Chloroplast.	Plastid.
Q9TJQ8	Unable to identify	Chloroplast. Mitochondrion.	Plastid.
Q9TJR0	Unable to identify	Chloroplast.	Plastid.
Q9TJR1	Unable to identify	Chloroplast.	Plastid.
